# Proline-, Glutamic Acid-, Leucine-Rich Protein 1 (PELP1): Diversity, Structural Conservation, and Evolutionary Origins Across the Species

**DOI:** 10.3390/ijms262411989

**Published:** 2025-12-12

**Authors:** Nomfundo Mngomezulu, Siphesihle M. Msweli, Suresh B. Pakala, Khajamohiddin Syed

**Affiliations:** 1 Department of Biochemistry and Microbiology, Faculty of Science, Agriculture and Engineering, University of Zululand, Empangeni 3886, South Africa; mngomezulunomfundot@gmail.com (N.M.); mooisphesihle@gmail.com (S.M.M.); 2 Department of Biochemistry, School of Life Sciences, University of Hyderabad, Hyderabad 500046, India

**Keywords:** scaffolding protein, RIX1 domain, NUC domain, Nuclear Localization Signal, phylogenetic analysis

## Abstract

Proline-, Glutamic acid-, Leucine-rich Protein 1 (PELP1) is a multifunctional nuclear protein essential for ribosome biogenesis and steroid receptor signaling. It contains two hallmark domains: the RIX1 (Ribosome Export 1) domain, which mediates rRNA processing, and the NUC (nucleolar) domain, associated with nucleolar function. While PELP1’s biological roles are well-characterized in mammals, particularly *Homo sapiens*, its distribution, structural diversity, and evolutionary origin across the domain of life remain largely unexplored. This study addresses this gap by conducting a comprehensive data mining of PELP1 proteins across the NCBI, UniProt, and EukProt databases. A total of 646 PELP1 proteins were identified exclusively in eukaryotes, specifically within the Opisthokonta clade, comprising Fungi, Filasterea, and Metazoa, while no homologs were detected in Bacteria, Viruses, Plants, or Oomycota. Domain analysis revealed that PELP1 proteins contain one RIX1 domain and one or two NUC202 domains. Motif analysis identified LXXLL and PXXP motifs, indicative of receptor-mediated signaling capability, although leucine and proline residues were not universally conserved within these motifs. Amino acid composition analysis showed enrichment of proline, glutamic acid, and cysteine across most PELP1 proteins. Despite low overall sequence identity, structural modeling demonstrated strong conservation of the α-helical fold, with an average root-mean-square deviation (RMSD) of 1.9 Å across species. Evolutionary analysis suggests that ancestral PELP1 emerged before the divergence of opisthokonts, originating from an RIX1-domain-containing protein that subsequently acquired a NUC202 domain. Phylogenetic clustering and sequence identity patterns resolved three major evolutionary lineages corresponding to fungi, filastereans, and metazoans. Overall, these findings reveal that PELP1 proteins exhibit extensive sequence divergence while maintaining a conserved structural architecture, reflecting evolutionary adaptation that preserves functional integrity across opisthokonts.

## 1. Introduction

**Figure 1:**
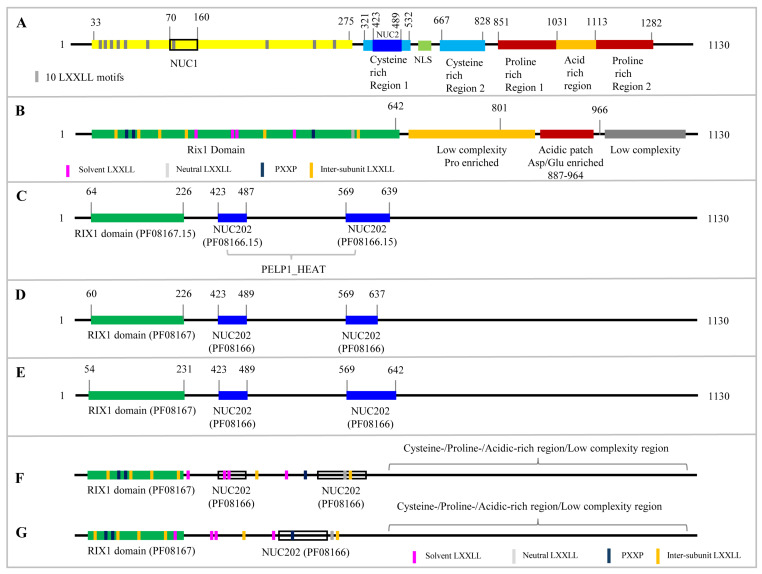
Schematic representation of the canonical structure of the Proline-, Glutamic acid-, and Leucine-rich Protein 1(PELP1). The schematic diagram is not drawn to scale unless the motifs/regions are indicated with a scale, such as the beginning and end of these areas/motifs. (**A**) The characteristic human PELP1 motifs and different regions reported by Vadlamudi and co-workers [13,14,15]. (**B**) The characteristic human PELP1 domain, motifs, and amino acid regions analysis by Gordon and co-workers [6]. The characteristic human PELP1 protein domains identified by the (**C**) National Center for Biotechnology Information (NCBI) Batch Web CD-Search Tool [16], (**D**) Hidden Markov Model Scan (HMMSCAN) [17,18] at the HMMER website [19], and (**E**) the MOTIF search tool (https://www.genome.jp/tools/motif/, accessed on 19 September 2025). (**F**,**G**) The characteristic canonical domain representations of PELP1 proteins across the species are based on the results of this study. According to the study results, PELP1 proteins contain one RIX1 domain (RIbosome eXport 1) and either one or two NUC202 (nucleolar) domains. Most PELP1 proteins contain eleven LXXLL (LM) and three PXXP (PM) motifs. The majority of PELP1 proteins have a C-terminal region enriched in cysteine, proline, or acidic amino acids, particularly glutamic acid, and this region has been reported to be low-complexity. Based on the percentage identity among PELP1 proteins observed in this study, we maintained the same criterion for the low-complexity region at the C-terminal end.

Scaffolding proteins are essential master regulators in cells that integrate interactions among multiple binding partners to facilitate protein–protein interactions, enzymatic cascades, and intricate signaling pathways. These proteins accomplish this by utilizing their structural architecture, which typically includes several signaling motifs and protein-binding domains [1]. Proline-, Glutamic acid-, and Leucine-rich Protein 1 (PELP1) is a well-known scaffolding protein with a unique amino acid composition and is highly conserved across species [2]. The PELP1 protein acts as a versatile hub within the cell, mainly because of its specialized structural parts, where it consists of two primary domains: a highly conserved N-terminal “RIX1” domain (RIbosome eXport 1), a NUC domain (nucleolar domain), and a less conserved, proline/glutamic acid-rich C-terminal domain (Figure 1). Most importantly, the RIX1 domain contains key signaling motifs, including eleven LXXLL and three PXXP motifs (X indicates any amino acid), which are known to mediate protein–protein interactions during steroid receptor signaling [3,4,5,6]. PELP1 was initially identified as a transcriptional coactivator of the estrogen receptor alpha (ERα) and subsequently found to coregulate several central steroid receptors (SRs), including androgen and glucocorticoid receptors [7,8,9]. PELP1 interacts with multiple transcription factors and coregulators to modulate the target gene expression and activity. These interactions ultimately facilitate tumorigenesis and metastasis by promoting cancer cell proliferation and enhancing their invasive potential [10,11,12] (Table 1).

PELP1 was expressed in many tissues, most prominently in the brain, testes, ovaries, and uterus [13,14]. PELP1 showed both cytoplasmic and nuclear localization, with enrichment in chromatin and the nuclear matrix. PELP1 subcellular distribution, along with its post-translational modification status, represents a potential regulatory mechanism for its coactivation of steroid receptors, both in normal and cancer tissues [2,6]. PELP1 functions as an epigenetic scaffold protein, where its N-terminal histone-binding domain directly recognizes histone modifications and facilitates recruitment to target genomic loci. At these sites, PELP1 coordinates multiple chromatin-modifying complexes, including histone deacetylase 2 (HDAC2), the histone demethylase KDM1, PRMT6, etc. [36,37,40]. By serving as a physical bridge between transcription factors and these enzymatic effectors, PELP1 orchestrates chromatin remodeling to alter gene expression patterns that are essential for hormone signaling and cell cycle progression (Table 1 and Table 2).

PELP1 function is dynamically regulated by multiple kinases, which phosphorylate specific residues to act as a molecular switch, modulating its protein–protein interactions and subcellular localization [28,30,31,32]. For example, Src kinase-mediated phosphorylation enables PELP1 to scaffold non-genomic signaling complexes that activate ERK1/2 or the MAPK pathway [3,32]. This intricate phospho-regulation enables PELP1 to integrate diverse signals from hormones, growth factors, and cell cycle checkpoints, thereby dictating its role in distinct complexes that govern gene expression, cell survival, and proliferation (Table 1 and Table 2). Consequently, dysregulation of this regulatory mechanism is a key driver of oncogenesis and therapy resistance [28,30,31,32]. Beyond its nuclear roles, PELP1 localizes to the nucleolus, where it interacts with RNA Polymerase I regulators, facilitating ribosomal biogenesis to meet the anabolic demands of hyper-proliferative cancer cells [42,43]. Furthermore, PELP1 contributes to the DNA damage response (DDR) by modulating the coactivator functions of p53 [41]. This activity promotes cell survival and maintains genomic integrity, underscoring a multifaceted role in tumor cell survival [41] (Table 1).

PELP1 is a known oncoprotein whose dysregulation is a hallmark of numerous cancers, particularly those driven by hormone signaling [7,8,9,12,20,21,24,32,43]. In breast cancer, PELP1 overexpression drives tumorigenesis and therapy resistance by hyperactivating estrogen receptor-α (ER-α) signaling through both genomic and non-genomic mechanisms [7,8,31,33]. Furthermore, its scaffolding function facilitates crosstalk with growth factor pathways, such as the Src/PI3K pathway, enabling tumors to bypass endocrine therapies like tamoxifen [3]. This pro-oncogenic role extends to other malignancies: PELP1 acts as a co-activator for the androgen receptor (AR) in prostate cancer and promotes proliferation and metastasis in ovarian and pancreatic cancers (Table 1). Beyond oncology, emerging evidence suggests that PELP1 plays a role in the brain [14,44]. This functional versatility underscores its role as a central signaling node, integrating diverse cellular processes critical in both normal physiology and disease pathogenesis (Table 2).

The complex pleiotropy of PELP1, encompassing roles in nuclear receptor coactivation, epigenetic scaffolding, cytoplasmic kinase signaling, and DNA damage repair, presents a fundamental biological question: how did such functional diversity evolve within a single protein? Investigating PELP1’s evolution is not merely a phylogenetic exercise but a strategic approach to understandthe assembly of complex signaling networks. Hence, we attempted to understand the distribution patterns, diversification, structural diversity, and conservation, as well as the evolutionary origins of PELP1 across the domains of life.

## 2. Results and Discussion

### 2.1. PELP1 Is Present Only in Eukaryotes

To identify PELP1 proteins across domains of life, an extensive search was conducted at the National Center for Biotechnology Information (NCBI) database [45], UniProt database [46] at the HMMER web server [19], and EukProt V3 [47] using the human PELP1 (UniProt ID: Q8IZL8) as a reference protein. Screening of all hit proteins revealed the presence of PELP1 proteins only in the eukaryotes (Table 3). PELP1 proteins were not found in Archaea, Bacteria, Viruses, Plants, or Oomycota. A total of 646 PELP1 proteins were identified in the clade Opisthokonta. Within this clade, species belonging to the kingdoms such as Fungi and Metazoa, the class Filasterea had the PELP1 proteins (Table 3). Among these groups, Metazoa had the highest number of 638 PELP1, followed by 6 in Fungi and 2 in Filasterea, indicating PELP1 plays a key role in metazoans. In Fungi, species belonging to the phylum Mucoromycota and the class Glomeromycetes have 4 PELP1 proteins, and 2 PELP1 proteins are found in species from the class Kickxellomycetes and the phylum Zoopagomycota. Among metazoan species of Chordata, the highest number of 600 PELP1 proteins, followed by 15 PELP1 proteins in Arthropoda, 9 in Mollusca, 5 in Echinodermata, 4 in Cnidaria, 3 in Annelida, and 2 in Brachiopoda (Table 3). This indicates that PELP1 plays a significant role in chordates, as very few PELP1 homologs were found in invertebrates. PELP1 proteins in Chordata are represented from classes such as Mammalia, Amphibia, Aves, and Reptilia (Sarcopterygii and Lepidosauria), and representative fish classes including Actinopteri, Hyperoartia, Chondrichthyes, Cladistia, and Leptocardii (Table 3). The PELP1 proteins identified in this study are presented, along with their species of origin, protein IDs, and other relevant information, including proteins with PELP motifs, in Tables S1–S4.

### 2.2. Domain and Motif Analysis of PELP1 Proteins

Analysis of PELP1 characteristic domains, such as RIX1 and NUC202, in 646 PELP1 proteins revealed that all proteins have one RIX1 and two NUC202 domains, except for 57 PELP1 proteins, which had only one NUC202 domain (Figure 1 and Table S1). The PELP1 with a single NUC202 domain is represented by species of the kingdoms Metazoa and Fungi, as well as the class Filasterea and classes/superclasses such as Chordata, Cnidaria, Echinodermata, Mollusca, Arthropoda, Mucoromycota, and Zoopagomycota (Table S1). This indicates that PELP1 proteins with single NUC202 domains are common across organisms.

**Figure 2:**
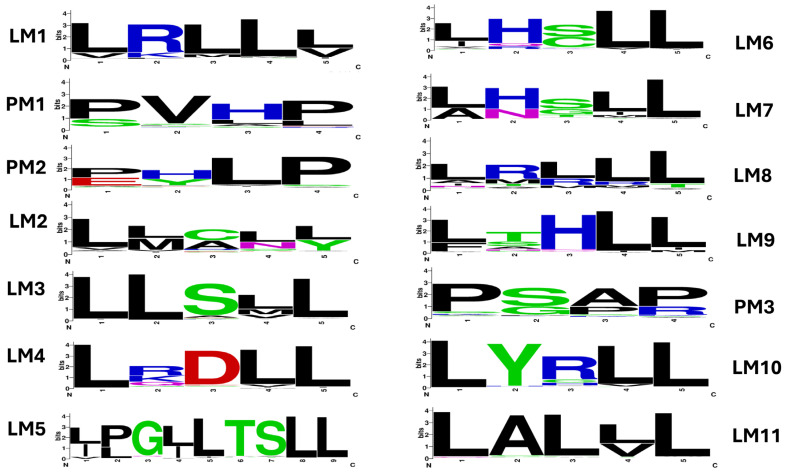
Analysis of amino acid patterns at LXXLL (LM) and PXXP (PM) motifs in Proline-, Glutamic acid-, Leucine-rich Protein 1 (PELP1) proteins. A sequence logo for these motifs was constructed using the motif sequences presented in Table S5. A detailed analysis of amino acid occurrence at each position at different motifs was also presented in Table S5, and the percentage of predominantly conserved amino acids is presented in Table 4. The numbers assigned to LM and PM motifs were the same as described in the literature [6].

To determine whether the identified PELP1 proteins have functional motifs, we further analyzed the LXXLL (LM) and PXXP (PM) motifs, as these motifs are known for mediating protein–protein interactions during steroid receptor signaling [2,3,4,5,49,50]. A comprehensive analysis of LM and PM motifs in 646 proteins revealed that these motifs (LM1-LM11 and PM1-PM3) are present in all PELP1 proteins identified in this study, with a few exceptions, where some proteins have fewer of these motifs (Figure 2 and Table S5). This suggests that the PELP1 proteins identified in this study might be functional.

Analysis of amino acid patterns in these motifs revealed an interesting pattern where non-conservation of leucine and proline amino acids was observed (Figure 2 and Table S5). Furthermore, *Salvator merianae* PELP1 has five amino acids in the PM1 motif, whereas *Ovis aries* PELP1 has six amino acids at the LM2 motif (Table S5).

The canonical structures of LXXLL and PXXP were derived based on the human PELP1. In this study, analysis of many PELP1 sequences necessitated proposing the actual patterns for these motifs (Table 4). As shown in Table 4, leucine and proline are predominantly present at these motifs but not invariantly conserved, indicating a divergence in the amino acid patterns of the motifs. A detailed analysis of amino acid patterns in these motifs is presented in Table S5.

### 2.3. PELP1 Proteins Are Indeed Enriched with Amino Acids Proline, Glutamic Acid, and Cysteine

Based on the human PELP1 protein, these proteins have been reported to contain regions enriched in amino acids such as cysteine, proline, and acidic residues, including aspartic acid/glutamic acid (Figure 1). In the current study, where many PELP1 proteins have been analyzed, it is essential to determine the enrichment status of these amino acids in these proteins to determine whether this is a characteristic of PELP1 proteins.

**Figure 3:**
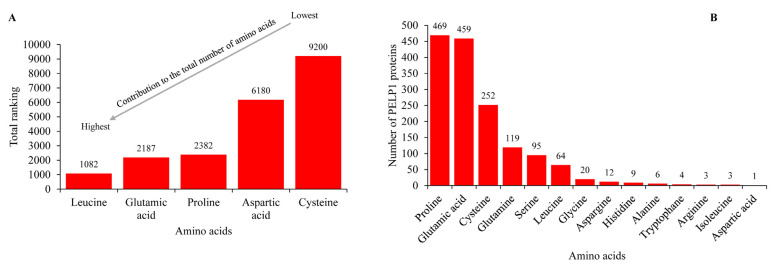
Analysis of amino acid enrichment in Proline-, Glutamic acid-, Leucine-rich Protein 1 (PELP1) proteins. (**A**) Contribution of selected amino acids to the total number of amino acids in PELP1 proteins. Each amino acid was given a rank based on its contribution, where one is the highest and so on. For each amino acid, all the ranks for 646 PELP1 proteins were combined and presented in the figure, where the highest ranking means this amino acid contributes the least, and the lowest ranking means it contributes the most among the amino acids. The number next to the bars indicates an amino acid’s overall ranking. (**B**) Amino acid enrichment analysis in PELP1 proteins. The number of PELP1 protein molecules that are particularly enriched in a particular amino acid is presented in the figure. The number next to the bars indicates the number of PELP1 proteins. For more information on the analysis, please see Table S6.

To unravel the amino acid enrichment phenomenon in the PELP1 proteins, we have assigned the ranks for amino acids such as leucine, glutamic acid, proline, aspartic acid, and cysteine based on their percentage contribution to the total number of amino acids in a particular PELP1. For example, the highest contributing amino acid gets rank 1, then the second highest contributing amino acid gets rank 2, and so on. After assigning the ranks to all amino acids for all PELP1 proteins, we then combine these ranks to see an overall picture of amino acid contributions (Figure 3A and Table S6). Analysis revealed that leucine is indeed the highest contributing amino acid, followed by glutamic acid, proline, aspartic acid, and cysteine (Figure 3A and Table S6). This indicates the amino acids such as leucine, glutamic acid, and proline are indeed highly populated in these PELP1 proteins. However, to determine whether these amino acids are indeed enriched in these proteins, we then performed amino acid enrichment analysis (Figure 3B and Table S6).

Amino acid enrichment analysis revealed that proline, glutamic acid, and cysteine are indeed enriched in 469, 459, and 252 PELP1 proteins, respectively (Figure 3B and Table S6). This strongly suggests that the phenomenon observed for Human PELP1 is universally conserved across PELP1 proteins, with these three amino acids enriched in the mostly C-terminal regions of the proteins (Figure 1). Interestingly, leucine, although highly populated in the PELP1, is only enriched in 64 PELP1 (Figure 3B and Table S6). Apart from these amino acids, glutamine, serine, and glycine are enriched in 119, 95, and 20 PELP1 proteins, respectively (Figure 3B and Table S6). A notable point is that not all PELP1 proteins are enriched in signature amino acids, such as proline or glutamic acid, especially in lower eukaryotes, including fungi, or in filastereans or invertebrates (Table S6).

### 2.4. Most PELP1 Proteins Have Classical Nuclear Localization Signaling

The presence of the NUC202 domain in all PELP1 proteins clearly indicates that these proteins have nucleolar function. Generally, nucleolar proteins contain a classical Nuclear Localization Signal (cNLS) in their sequence. In this study, we further investigated the presence of NLS in PELP1 proteins. The cNLS mapper [51] predicted that 84% of the PELP1 proteins (540 out of 646) have cNLS in their sequence (Table S7). This indicates that these 540 proteins most probably perform a function in the nucleus. These 540 PELP1 proteins have a score of 5 or higher, indicating a high likelihood that they have a nucleolar function. A thorough breakdown of the cNLS score revealed that 128 PELP1 proteins have a score of ≥8 indicating they exclusively localized to the nucleus, 30 PELP1 proteins have a score of 7–7.5 indicating their partial localization to the nucleus, and 393 PELP1 proteins have a score between 5 and 6.5, indicating these PELP1s have localization both in the nucleus and cytoplasm [51]. Among different patterns of cNLSs, some of the cNLSs are particularly dominant, such as the NLS sequence “PSAPKKIKLD”, which is present in 297 PELP1 proteins, followed by “RGTKRKMEDR” in 29 PELP1 proteins, “PSAPKKPKLS” in 20 PELP1 proteins, “KGVKRKREEG” in 16 PELP1 proteins, and “RGMKRKREGE” in 13 PELP1 proteins (Table S7). The cNLS mapper did not predict the cNLS for 106 PELP1 proteins. This does not mean that these proteins did not function in the nucleolus, as the cNLS mapper was explicitly developed to identify NLSs recognized by the classical importin-alpha/beta pathway and cannot predict those that are directly recognized by other members of the importin-beta family [51]. These 106 PELP1 proteins are from across all taxonomic ranks and are not limited to a specific taxonomic group (Table S7).

### 2.5. Structural Fold Conservation and Diversification of PELP1 Proteins Across the Species

**Figure 4:**
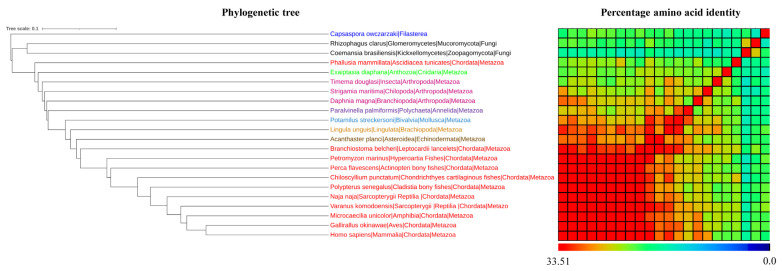
Evolutionary analysis and amino acid sequence similarity analysis of representative Proline-, Glutamic acid-, Leucine-rich Protein 1 (PELP1) proteins from different classes/superclasses. In the phylogenetic tree, PELP1 proteins from different classes/superclasses were indicated in various colors. The percentage of amino acid identity is represented as a heatmap. The scale below the heatmap indicates the increase in percentage identity as the color changes from blue to red.

Representative PELP1 proteins from organisms across different classes/subclasses were selected to examine structural conservation, if any (Figure 4). Phylogenetic analysis revealed that PELP1 proteins were grouped as per their classes/superclasses, with the exception that *Phallusia mammillata* was not grouped along with other chordates (Figure 4), indicating its divergence from PELP1 from other chordates. The phylogenetic tree has three distinct branches, with PELP1 from Filasterea as the outgroup, and the remaining two branches contain PELP1s from Fungi and Metazoa (Figure 4). This clearly indicates that there was likely a single common ancestral PELP1-like protein in a very early eukaryotic ancestor that split (diverged) into three separate evolutionary branches (lineages), each branch evolving independently: one leading to fungi, one to filastereans, and one to animals.

**Figure 5:**
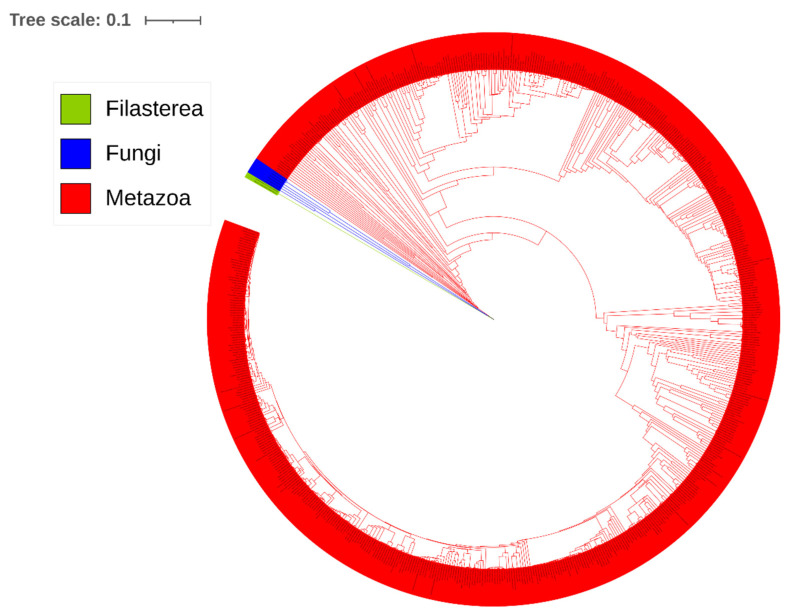
Evolutionary analysis of Proline-, Glutamic acid-, Leucine-rich Protein 1 (PELP1) proteins. The phylogenetic tree was constructed using 646 PELP1 protein sequences (Supplementary Dataset S1). A high-resolution phylogenetic tree with individual node information is provided in Supplementary Figure S1.

Analysis of amino acid percentage identity among selected PELP1 revealed that the average percentage identity among PELP1 proteins was 26.9% with 14.88% and 64.20% being the lowest and highest percentage amino acid identity among PELP1 proteins (Figure 4 and Table S8). This suggests that, overall, PELP1 proteins have a very low amino acid sequence identity, indicating significant divergence in their primary sequences. An interesting pattern was observed, where PELP1 proteins from Filasteria and Fungi have a very low percentage of amino acid sequence identity with their metazoan counterparts (Figure 4), further indicating that the ancestral PELP1 has diverged into three lineages, as described earlier. However, this phylogenetic tree was constructed using only 22 PELP1 representatives from each of the classes/subclasses. To support this hypothesis of different-lineage evolution, we built a tree that includes all 646 PELP1 proteins (Figure 5). As anticipated, PELP1 proteins were aligned according to their kingdom, such as Fungi, Metazoa, or in the case of Filasterea (Figure 5). From the phylogenetic tree, it is clearly visible that there are three branches, each representing filastereans, fungi, and metazoans, indicating that the ancestral PELP1-like protein diverged into three lineages in opisthokonts. This means that PELP1 proteins from each of these groups of organisms evolved independently of one another.

**Figure 6:**
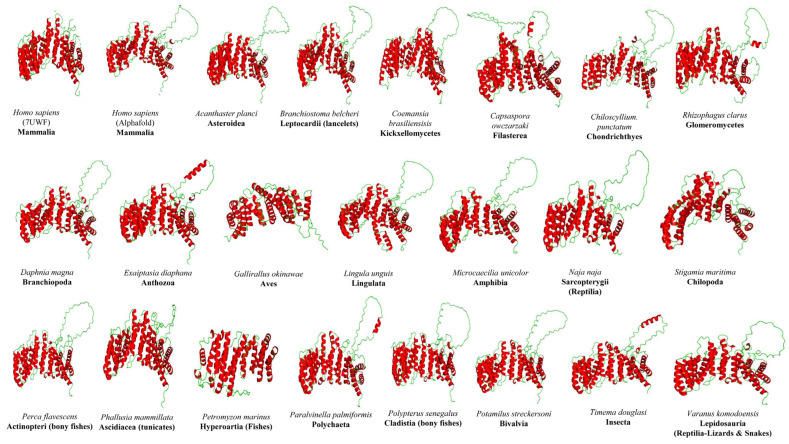
Structural analysis of Proline-, Glutamic acid-, Leucine-rich Protein 1 (PELP1) proteins from different organisms. Under each three-dimensional PELP1 structure, the species name and the species class/superclass to which it belongs are indicated. Helices were shown in red, and loops in green.

The observed average amino acid identity among PELP1 proteins is low, raising the question of whether their three-dimensional structures are diverse or whether the structural fold is conserved despite their low percentage identity across different organisms. To address this question, we performed a structural analysis of 22 PELP1 proteins, including those from Homo sapiens (Figure 6). The two available human PELP1 structures have some amino acids missing, so we included this PELP1 to maintain consistency for comparison with other PELP1 structures (Figure 6).

Structural analysis of PELP1 proteins revealed the presence of only α-helices in their structure, with loops connecting each of the helices (Figure 6). PELP1 from different organisms differed in the number of helices in their structure (Table 5). The highest number of 27 α-helices was observed for the PELP1s from *H. sapiens* and *Exaiptasia diaphana* (Table 5). PELP1 proteins from *Petromyzon marinus*, *Gallirallus okinawae*, and *Perca flavescens* all have ≤20 α-helices in their structure, and this is due to their protein amino acid length being short compared to other PELP1s, except *P. flavescens* (Figure S2). *P. marinus* PELP1 has only 385 amino acids, which is the shortest among all PELP1s used for structural analysis (Figure S2). The majority of the LXXLL (LM) and PXXP (PM) motifs were found to be located in the helix regions (Figure S2).

**Figure 7:**
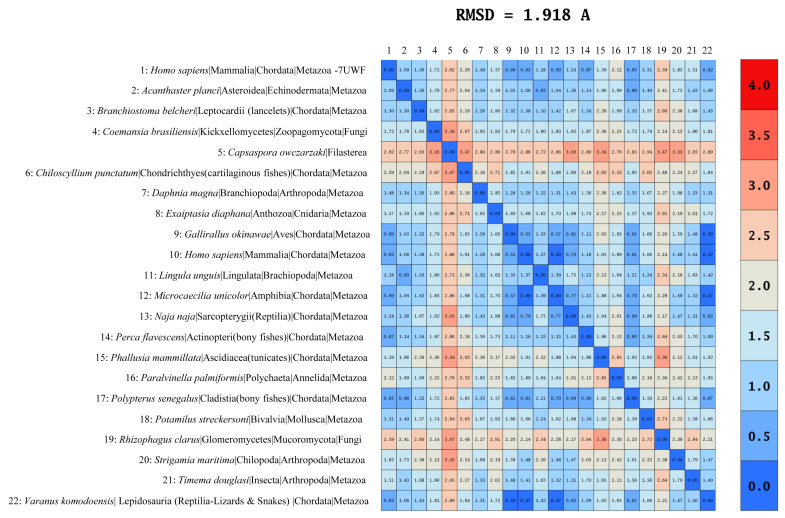
Comparative structural analysis of Proline-, Glutamic acid-, Leucine-rich Protein 1 (PELP1) proteins across the domains of life. The Root Mean Square Deviation (RMSD) values among PELP1 proteins are shown in the figure, along with the average RMSD value at the top of the figure. The species from which the PELP1 proteins were obtained for analysis is presented, along with its taxonomy.

The average Root Mean Square Deviation (RMSD) among PELP1 proteins was found to be 1.9 Å, indicating a structural fold conservation across the domains of life (Figure 7). The RMSD values are considered very important in comparing protein structural similarities and differences [52,53,54]. Proteins with RMSD values between 0 and 1 Å are considered nearly identical, those with 1.0–2.0 Å values are considered highly similar or a fold conservation, those with 2.0–3.0 Å values are considered moderately similar proteins, and those with values between 3.0 and 5.0 Å are considered structurally different proteins, as overall fold may differ [52,53,54]. Considering this fact and the observed average RMSD value among PELP1 protein models from different organisms, we can safely say that the overall fold among PELP1 proteins is conserved across the domains of life.

The RMSD between PELP1 of *H. sapiens* of AlphaFold and the crystal structure (7UWF) and the PELP1s from Aves, amphibia, Sarcopterygii (Reptilia), and Cladistia (bony fishes) and Lepidosauria (Reptilia—Lizards & Snakes) was ≤1 Å, indicating these proteins are nearly identical in nature (Figure 7). PELP1s from Asteroidea, Leptocardii (lancelets), Kickxellomycetes, Chondrichthyes (cartilaginous fishes), Branchiopoda, Anthozoa, Brachiopoda, Actinopteri (bony fishes), Ascidiacea (tunicates), Polychaeta, Bivalvia, Chilopoda, and Insecta have RMSD values between 1.0 and 2.0 Å, with the H. sapiens AlphaFold PELP1 model indicating the structural conservation among these proteins. PELP1 from Filasterea had a 2.9 Å resolution, and PELP1 from Glomeromycetes had a 2.1 Å resolution, suggesting that these proteins are moderately similar (Figure 7). A notable point is that all the Alphafold-generated structures contain many loops, which is why we observe these RMSD differences. However, despite this drawback, the observed RMSD differences are not high and thus strongly suggest that the overall structural fold of PELP1 proteins is conserved across the domains of life.

### 2.6. PELP1 Originated in Opisthokonta

**Figure 8:**
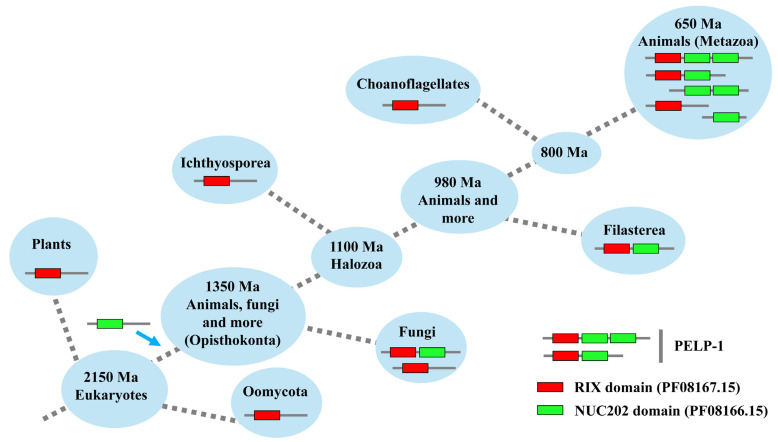
Evolutionary origin of Proline-, Glutamic acid-, Leucine-rich Protein 1 (PELP1) in eukaryotes. The timelines on the divergence between different animal groups and the evolutionary trend were generated using OneZoom [55]. The blue arrow indicates the acquisition of the NUC202 domain by opisthokonts.

To determine the origin of PELP1, we have screened a large number of hit proteins across the domains of life, focusing on the two characteristic domains, RIX and NUC202 (see Tables S1–S4). Proteins with only RIX domains were found in plants and oomycota, indicating that proteins with the RIX domain originated before the early eukaryotic divergence (Figure 8). A protein with both RIX and NUC202 was found in Fungi, suggesting that ancestral PELP1 evolved before the divergence of eukaryotes. This might have occurred because the proteins already had RIX domains, which acquired NUC202 domains in the Opisthokonta, as we did not find proteins with NUC202 domains in plants or oomycota (Figure 8). Ichthyosporea and choanoflagellates have proteins with the RIX domain, and we did not find PELP1 in these species. However, PELP1, which contains one RIX and one NUC202 domain, was found in Filasterea (Figure 8).

In metazoans, we identified PELP1 proteins with one RIX and one NUC202 domain, as well as those with one RIX and two NUC202 domains (Tables S1–S4). Furthermore, we identified proteins containing either the RIX or NUC202 domain, suggesting that a combinatorial event between these domains led to the evolution of PELP1 in metazoans, as described above. The presence of different combinations of RIX, NUC202 domain PELP1s, and the existence of proteins with either RIX or NUC202 domains further strongly suggests that the ancestral PELP1 proteins split into three lineages and were passed on to fungi, filastereans, and metazoans. This hypothesis is further strongly supported by the phylogenetic analysis and the percentage of amino acid identity among PELP1 proteins, as described in Section 2.5. The ancestral PELP1 might have a single RIX and a single NUC202 domain, as Fungi and Filasterea have only this type of PELP1.

In this study, we did not find any proteins with the RIX domain or NUC202 domain from Archaea, Viruses, or Bacteria. Furthermore, proteins with the RIX domain have been identified in protist species (Hapista) (Table S2), indicating that the RIX domain evolved in ancestral eukaryotes before the divergence into different groups. Proteins with RIX domains are abundantly present in fungi or plants (Table S2). Proteins with a single or double NUC202 protein were found only in metazoa (Tables S3 and S4). The presence of proteins with either only a RIX or NUC202 domain (single copy or multiple copies) clearly suggests that these may be the ancestral proteins that led to the development of PELP1.

## 3. Materials and Methods

### 3.1. Selection of a Suitable Method for Analyzing PELP1 Proteins

PELP1 protein domains are well defined (Figure 1), enabling their identification among numerous proteins across different domains of life. However, to facilitate their easy identification among a large number of proteins, a suitable program is required. In this study, human PELP1 domains were analyzed using three tools: the National Center for Biotechnology Information (NCBI) Batch Web CD-Search Tool [16], Hidden Markov Model Scan (HMMSCAN) [17,18] at the HMMER website [19], and the MOTIF search tool (https://www.genome.jp/tools/motif/, accessed on 19 September 2025) (Figure 1). All three programs gave similar results, demonstrating their reliability for PELP1 screening among other proteins. Among these three programs, we selected the MOTIF search tool for further analysis due to its user-friendly features, including clear domain position and the ability to easily extract domain sequences, as the results will highlight domain amino acids in different colors within the complete protein sequences, and one can copy and paste them into a file.

### 3.2. Data Mining of PELP1 Across the Domains of Life

Data mining of PELP1 proteins across the domains of life was carried out using the human PELP1 protein as a reference protein (NCBI protein ID: AAC17708.2), following the method described elsewhere [56]. The human PELP1 protein sequence was blasted against the three different databases: (i) UniProt database [46] at the HMMER web server [19]; (ii) NCBI database [45] and the EukProt V3 database [47]. The EukProt V3 database contains highly curated genomes of eukaryotic taxa, particularly species of taxa that are otherwise considered orphans. All the hit proteins were downloaded and subjected to motif analysis using the MOTIF search tool (https://www.genome.jp/tools/motif/, accessed 12 July 2025). The proteins with characteristic PELP1 motifs, such as RIX1 (PF08167.15) and NUC202 (PF08166.15), were selected as PELP1 proteins and subjected to duplicate analysis. Sequences that shared 100% identity and were from the same species were considered duplicates, and only one of each duplicate sequence was included in the study for further analysis. The hit proteins with PELP1 characteristic motifs from these three databases were further compared to each other, and one representative protein was retained by deleting the other proteins with 100% sequence identity from the same species. In our analysis, the same set of PELP1 homologs was identified in all three databases (UniProt, NCBI, and EukProt); therefore, the UniProt sequences were used for further study. The final list of PELP1 proteins, as well as proteins with either RIX or NUC202 domains from different taxa, along with their taxonomic ranks and other characteristics, including domain information, is presented in Tables S1–S4.

### 3.3. Phylogenetic Analysis

Phylogenetic analysis of PELP1 proteins was conducted following the same method used for evolutionary analysis of NF-κB (Nuclear Factor kappa-light-chain-enhancer of activated B cells) family members in a previous study [56]. First, the PELP1 protein sequences were aligned with the MAFFT v6.864 program with default auto settings [57], which is available on the T-REX web server [58]. The alignments were then automatically subjected to interpret the best tree using the maximum likelihood method available on the T-rex web server [58]. Finally, the best-inferred tree was visualized, colored, and generated by the Interactive Tree of Life (iTOL) [59].

### 3.4. Identification of Classical Nuclear Localization Signal

The cNLS mapper [51] was used to identify the cNLS in PELP1 proteins. For the cNLS mapper analysis, each sequence was submitted individually. cNLS mapper generated three outputs: predicted position within the protein, predicted monopartite NLS motifs, and score reflecting the strength of nuclear import. Interpretation was done according to the cNLS mapper criteria: ≥8 (strongly localized in the nucleus), 7–8 partially localized to the nucleus, 3–5 (both located in the nucleus and cytoplasm), 1–2 (localized to the cytoplasm) [51].

### 3.5. Identification of LXXLL (LM) and PXXP (PM) Motifs in PELP1 Proteins

LM and PM motifs in PELP1 proteins were identified by performing individual PELP1 protein alignment with the Human PELP1 protein using the Clustal Omega program [60]. The amino acid sequence alignment was checked for the LM and PM motifs in PELP1 proteins, where the relevant motifs align with those of human PELP1. The LM and PM motifs identified in all PELP1 proteins are presented in Table S5 along with the information for dominant amino acids at each position in these motifs. The percentage predominance of amino acids at each position is calculated assuming the total number of amino acids is 100%. Amino acids or patterns of amino acids contributing more than 10% at the specific position are shown in Table 4. Amino acids conserved (100%) as the particular position(s) are represented by their symbol and in bold (Table 4).

### 3.6. Generation Motifs Sequence Logos

PELP1 motif sequence logos were generated following the method described elsewhere [61]. Briefly, LM1-LM11 and PM1-PM3 motif sequences (Table S5) were aligned using the ClustalW multiple alignment program in MEGA12 [62]. After sequence alignment, the aligned set of amino acids was selected and entered into the WebLogo program (http://weblogo.berkeley.edu/logo.cgi, accessed 12 July 2025). As a selection parameter, the image format was chosen as PNG (bitmap) at a resolution of 300 dpi. The percentage dominance of amino acids at a particular position is calculated based on the total number of amino acids, which is set at 100%.

### 3.7. Protein Sequence Identity Analysis and Heatmap Representations

The percentage of amino acid sequence identity among PELP1 proteins was determined using the Clustal Omega program [60]. PELP1 protein sequences were submitted to Clustal Omega, and the results for percentage identity were downloaded into an Excel file. This Excel file was saved as a tab-delimited file for creating the heat maps, following the method described previously by our laboratory [63]. The tab-delimited file was imported into the multi-experiment viewer (MeV) version 4.9.0 [64]. The data were clustered using a Euclidean distance metric and hierarchical clustering. The vertical and horizontal axes represent the PELP1 proteins from different classes/superclasses. The percentage identity among different PELP1 proteins was represented using a rainbow scheme, where blue means 0% identity and red represents 100% identity.

### 3.8. Amino Acid Composition and Enrichment Analysis

To identify which amino acids are enriched and highly populated in PELP1 proteins, we asked ChatGPT to generate a program in an Excel file where one can paste the protein sequences, and it automatically generates the results showing which amino acids are enriched and the composition of each amino acid in the protein. We provided two Excel program files in the Supplementary Datasets (S2 and S3): a simplified version and a detailed version, respectively.

Here, the methodology information generated by ChatGPT is presented: “The amino acid composition of the protein sequence was analyzed by calculating the frequency of each residue relative to the total sequence length. Counts of individual amino acids were obtained by parsing the sequence and calculating the percentage composition by dividing the number of occurrences of each residue by the total number of residues.

To assess enrichment, the observed amino acid frequencies were compared against average background frequencies reported in UniProt reference proteomes, which provide established baseline distributions of residues across diverse proteins [65]. Enrichment was determined using fold-change calculations, where the observed frequency of a residue was divided by its expected background frequency. Residues with values ≥ 1.5 times higher than the expected frequency were considered enriched. In addition, log_2_ (fold enrichment) values were calculated to normalize the fold differences, allowing for a more straightforward interpretation of over- and under-represented residues.

This approach follows widely accepted principles in sequence analysis and compositional bias studies, where deviations in amino acid usage are interpreted in the context of protein structure and function [66,67]. The Excel-based computational framework developed for this study implements these calculations automatically, providing residue counts, percentages, and enrichment calls for any protein sequence input.

The program was validated using proteins known to be enriched in specific amino acids (Table 6). The Excel program gave the results that match their reported nature with respect to the type of amino acids that are enriched (Table 6).

### 3.9. Protein Modeling

A total of 22 representative PELP1 proteins belonging to each of the classes/superclasses were selected for protein modeling (Table 5). The proteins were randomly selected from each class/superclass, with no criteria applied. In this study, we also included human PELP1 (UniProt ID: Q8IZL8). The reason to include is that, for the two human PELP1 crystal structures, 7UWF [6] and 9 DUM [73], many amino acids are not crystallized, and gaps exist in the structures. For this reason, we also constructed a three-dimensional (3D) protein model of human PELP1 for accurate comparative analysis with PELP1 proteins from different species. All PELP1 sequences were first aligned against the crystallized human PELP1 sequence 7UWF [6]. The amino acid sequences that were not aligned with the 7UWF sequence were removed, and the remaining sequence, which spans across the RIX1 and NUC202 domains, was selected for protein modeling. A multiple sequence alignment of PELP1 proteins used for protein modeling is presented as Figure S2. Three-dimensional protein models were generated using the AlphFold database [74]. The 3D protein structures were visualized, and high-quality 3D protein model images were generated using the PyMol software, version 2.2.5 [75]. All 22 PELP1 3D models were superimposed individually with 7UWF, and the extra-long loops, especially those that extended beyond the space defined by 7UWF, were removed. The 3D models with loops removed were superimposed alongside 7UWF, and the RMSD value was calculated. A graphical image showing the RMSD values among all PELP1 proteins, including 7UWF, is presented (Figure 7). *P. marinus* PELP1 has only 385 amino acids, which is the shortest among all PELP1s used for structural analysis. The 3D model of this PELP1 is not used for RMSD calculation, as this protein is not complete.

## 4. Conclusions

Analysis of PELP1 proteins across the domains of life revealed that these proteins are exclusive to eukaryotes and are particularly abundant in metazoans, indicating a strong evolutionary association with the development of multicellular complexity. Despite significant sequence divergence, the α-helical structural fold of PELP1 remains conserved across species, underscoring a remarkable structural stability throughout evolution. Amino acid analyses further suggested that enrichment in proline, glutamic acid, and cysteine seems to be a conserved characteristic of these proteins. The evolutionary analysis, combined with sequence identity, demonstrates that ancestral PELP1 proteins diverged into three parallel lineages, which evolved into filastereans, fungi, and metazoans, originating from early eukaryotic proteins containing RIX1 and NUC202 domains.

The conclusions drawn in this study on PELP1 proteins are based on currently available data from various databases. The future availability of more PELP1 proteins, especially from other opisthokonts, may alter their evolutionary narrative, particularly regarding the number of lineages. Furthermore, domain analysis, especially in the context of the NUC202 comparison between opisthokonts, will provide a clearer picture of the lineages. However, in the current study, this is not possible due to the identification of only a handful of PELP1 proteins in Fungi (six) and Filasterea (two). Whether this is possible or not remains to be seen, as we analyzed a substantial number of proteins from fungi in this study and have already identified many proteins with NUC202 or RIX domains. Furthermore, the positional location of PELP1 signature amino acids, such as proline, glutamic acid, and cysteine, needs to be determined to map the positional enrichment of these amino acid regions.

In conclusion, this study’s findings reveal that PELP1 exemplifies evolutionary adaptation through sequence diversification while maintaining structural and functional conservation, which is essential for its regulatory role in eukaryotic cells. The identification of PELP1 only in opisthokonts suggests that the assembly of complex signaling networks has developed in these organisms. Future work involves unraveling the biological roles of newly identified PELP1 proteins in species of Fungi and Filasterea.

## Figures and Tables

**Table 1:** Proline-, Glutamic acid-, Leucine-rich Protein 1 (PELP1) interacting partners and their functional significance.

S. No	Interacting Partner	Mechanism of Action	Function	Model System	Reference
Nuclear receptor partner					
1	ERα and ERβ (Estrogen Receptor alpha and beta)	Increased ERα and ERβ transcriptional responses	Modulates the overall estrogenic response and proliferation of endometrial cancer cells	Endometrial cancer	[7]
2	ER-related receptor-alpha and Proline-rich nuclear receptor coregulatory protein2	Activation of aromatase	Tumor proliferation	Breast cancer	[20]
3	AR (Androgen Receptor)	Coactivator of AR	E2-stimulated proliferation of Prostate cancer cells	Prostate cancer	[8]
4	GR (Glucocorticoid receptor)	GR corepressor and coactivator in a context-dependent manner, depending on cell type	Not known	A549 cells (GR corepressor) HEK293 (biphasic effect)	[21]
5	GR	Activates Brk expression	Cancer progression and metastasis	Triple Negative Breast Cancer (TNBC)	[9]
6	RXRα	PELP1 acts as a coactivator of the RXRα-peroxisome proliferator-activated receptor (PPARγ) heterodimer	Not known	MCF-7 cells	[22]
**Transcription factors and coregulators**					
7	STAT3	Transcriptional regulation of STAT3 and its target genes	Oncogenesis	MCF-7 and HeLa cells	[10]
8	β-catenin	Coactivator of β-catenin	Glioblastoma (GBM) progression	Glioblastoma	[12]
9	FHL2 (Four-and-a-half LIM only protein 2)	Enhances the transcriptional activity of FHL2	Prostate cancer progression	Prostate cancer cells	[23]
10	FHL2 (Four-and-a-half LIM only protein 2)	Transactivation of CCND1, CCND2, CDK6, ANG, CCL2, and MMP3	EMS progression	Endometriosis (EMS)	[24]
11	AIB1 (Amplified In Breast Cancer 1) or SRC-3 or NCOA3	Cytoplasmic PELP1 overexpression increases Thr24 phosphorylation of AIB1 and enhances aldehyde dehydrogenase activity, and thereby promotes advanced cancer phenotypes	Estrogen-independent breast cancer progression	Estrogen receptor-positive (ER^+^) and ER-negative (ER^−^) cell lines	[11]
12	CARM1 (Arginine methyltransferase)	Enhance ERα transactivation	Tumor proliferation	Breast cancer	[25]
**Cell cycle regulators**					
13	pRb	Regulates cyclin D1 expression at the transcriptional level	E2-mediated cell cycle progression	Breast cancer	[26]
14	CDK2/4	Activates the pRb/E2F pathway, thereby contributing to tumorigenesis by accelerating cell cycle progression	Promotes E2-driven tumor growth	Breast cancer	[27]
**Kinases**					
15	PI3K (p85 subunit)	Increased PI3K activity and activation of AKT	Tamoxifen resistance	Breast cancer	[28]
16	EGFR	ER transactivation	Increased tumorigenic function	Breast cancer	[28]
17	HRS (Hepatocyte growth factor-regulated tyrosine kinase substrate)	Activates MAPK in an EGFR-dependent manner and also activates the Elk-1 pathway	Upregulation of MAPK in breast tumors	Breast cancer	[29]
18	PFKFB3 and PFKFB4	Not known	Breast cancer progression	ER^+^ Breast cancer	[30]
19	GSK3β	Activation of the Wnt/β-catenin signaling pathway	E2-mediated neuroprotection and cognitive function	Brain/PELP1 FBKO mice	[31]
20	IGF1R ERα Src	Activation of the ERK1/2 pathway	ACC cell proliferation	Adrenocortical carcinoma (ACC)	[32]
21	Src	Activation of the MAPK signaling pathway and increased ER’s transcriptional activity	Cell growth and survival	Breast cancer	[3]
**Chromatin modifiers**					
22	Histone H1	Chromatin remodeling	Estrogen receptor genomic functions	Breast cancer	[33]
23	Histone H3	Chromatin remodeling	Pancreatic carcinogenesis	Pancreatic cancer cells	[34]
24	SETDB1	PELP1 is a mediator of SETDB1-mediated AKT methylation and phosphorylation	Breast cancer progression	Breast cancer	[35]
25	KDM1	Demethylation of dimethyl H3K9	Switches chromatin from an active to a repressive state.	Breast cancer cells	[36]
26	HDAC2	Transcriptional repressive function of PELP1	Not known	NIH3T3 and C3H10T1/2 cells COS-7 cells	[37]
27	ERR alpha, and histone demethylases	Activation of aromatase	Cancer progression	Breast cancer	[38]
28	MacroH2A1	Transcriptional regulation	Not known	Breast cancer cells	[39]
29	PRMT6	Regulates the alternative splicing of genes involved in cancer	Tumor progression	Breast cancer	[40]
**DNA damage response proteins**					
30	p53	p53 coactivator functions	DNA damage response	Breast cancer	[41]
**Ribosome Biogenesis**					
31	LAS1L	Involved in 60S ribosomal subunit synthesis	Ribosome biogenesis	HCT116 cells U2OS cells	[42]
32	SENP3-associated complex (PELP1, TEX10 and WDR18)	Maturation and nucleolar release of the large ribosomal subunit	Ribosome biogenesis	HeLa cells	[43]
**Others**					
33	mERalpha-LBD	Not known	Role in estrogen signaling and brain functions during aging	Mouse brain	[44]

**Table 2:** Proline-, Glutamic acid-, Leucine-rich Protein 1 (PELP1) physiological functions.

S. No.	Physiological Role	Function	References
1	Nuclear receptor co-regulation	PELP1 acts as a transcriptional co-activator/co-repressor for ER, AR, and GR	[7,8,21]
2	Non-genomic signaling	PELP1, through its scaffolding activity, connects ER or PR to kinases like Src, PI3K, and activates MAPK/ERK and AKT signaling pathways	[3,28,32]
3	Cell cycle regulation	PELP1 interacts with CDK2, CDK4 and pRb and thereby modulates G1/S transition	[26,27]
4	Chromatin remodeling/epigenetic functions	PELP1 interacts or recruits KDM1, HDAC2, PRMT6, CARM1 and thereby modules chromatin remodeling and transcription	[25,36,37,40]
5	DNA damage response (DDR)	ATM/ATR phosphorylates PELP1, and its phosphorylation is key for modulating the activity of the tumor suppressor protein p53-mediated DNA damage signaling	[41]
6	Ribosome biogenesis	PELP1 is part of the Rix1/TEX10-WDR18 complex, essential for maturation of the large 60S ribosomal subunit and rRNA processing	[42]
7	Regulation of RNA Splicing	PELP1 interacts with splicing factors and PRMT6 to influence alternative splicing	[40]
8	Neuronal and brain functions	PELP1 mediates estrogen-driven neuroprotection, and cognitive processes	[14]
9	Regulation of apoptosis	Through p53 and stress-activated pathways, PELP1 modulates cell survival and apoptosis	[41]

**Table 3:** Identification of Proline-, Glutamic acid-, Leucine-rich Protein 1 (PELP1) proteins and their representative domains in eukaryotes. Eukaryotes were classified according to the National Center for Biotechnology Information (NCBI) taxonomy [48]. The PELP1 domains include RIX1 (Ribosome Export 1) (PF08167.15) and NUC202 (nucleolar) (PF08166.15). Detailed information on PELP1 proteins identified in this study is presented in Tables S1–S4.

Domain	Clade	Kingdom	Phylum	Class	Number of PELP1 Proteins	Number of Proteins with RIX Domain	Number of Proteins with 2 NUC Domains	Number of Proteins with 1 NUC Domain
Eukaryota	Amoebozoa		Evosea	Eumycetozoa	0	1	0	0
	Haptista		Haptophyta		0	4	0	0
			Haptophyta	Pavlovophyceae	0	1	0	0
	Core chlorophytes	Viridiplantae	Chlorophyta	Chlorophyceae	0	8	0	0
				Mamiellophyceae	0	1	0	0
				Pyramimonadophyceae	0	1	0	0
				Trebouxiophyceae	0	9	0	0
	Sar		Oomycota		0	78	0	0
	Embryophyta	Viridiplantae	Streptophyta	Magnoliopsida	0	372	0	0
				Bryopsida	0	8	0	0
				Zygnemophyceae	0	4	0	0
				Charophyceae	0	2	0	0
				Lycopodiopsida	0	2	0	0
				Klebsormidiophyceae	0	1	0	0
				Polypodiopsida	0	1	0	0
	Opisthokonta			Ichthyosporea	0	3	0	0
				Choanoflagellata	0	4	0	0
				Filasterea	2	0	0	0
				Fonticula alba (species)	0	1	0	0
		Fungi	Ascomycota	Dipodascomycetes	0	8	0	0
				Dothideomycetes	0	189	0	0
				Eurotiomycetes	0	217	0	0
				Geoglossomycetes	0	2	0	0
				Lecanoromycetes	0	10	0	0
				Leotiomycetes	0	77	0	0
				Lipomycetes	0	2	0	0
				Orbiliomycetes	0	12	0	0
				Pezizomycetes	0	11	0	0
				Pichiomycetes	0	54	0	0
				Pneumocystomycetes	0	5	0	0
				Saccharomycetes	0	77	0	0
				Schizosaccharomycetes	0	3	0	0
				Sordariomycetes	0	336	0	0
				Taphrinomycetes	0	1	0	0
				Tremellomycetes	0	1	0	0
				Xylonomycetes	0	1	0	0
			Basidiomycota	Agaricomycetes	0	143	0	0
				Dacrymycetes	0	3	0	0
				Exobasidiomycetes	0	5	0	0
				Malasseziomycetes	0	1	0	0
				Microbotryomycetes	0	17	0	0
				Mixiomycetes	0	1	0	0
				Pucciniomycetes	0	15	0	0
				Tremellomycetes	0	33	0	0
				Wallemiomycetes	0	3	0	0
			Blastocladiomycota	Blastocladiomycetes	0	1	0	0
			Chytridiomycota	Chytridiomycetes	0	6	0	0
				Monoblepharidomycetes	0	1	0	0
				Chytridiomycetes	0	1	0	0
				Neocallimastigomycete	0	4	0	0
			Cryptomycota		0	1	0	0
			Mucoromycota	Glomeromycetes	4	25	0	0
				Mortierellomycetes	0	19	0	0
				Mucoromycetes	0	19	0	0
				Umbelopsidomycetes	0	3	0	0
			Zoopagomycota	Basidiobolomycetes	0	1	0	0
				Dimargaritomycetes	0	4	0	0
				Harpellomycetes	0	1	0	0
				Kickxellomycetes	2	19	0	0
				Zoopagomycetes	0	1	0	0
		Metazoa	Arthropoda	Branchiopoda	0	1	0	0
				Hexanauplia	5	1	0	0
				Insecta	9	39	0	1
				Chilopoda	1		0	0
				Malacostraca	0	2	0	0
			Chordata	Actinopteri (bony fishes)	217	5	5	2
				Mammalia	315	12	15	0
				Amphibia	13	1	0	0
				Sarcopterygii (Reptilia)	15	0	0	0
				Aves	7	1	0	17
				Hyperoartia (Fishes)	2	0	0	1
				Ascidiacea (tunicates)	2	0	1	0
				Leptocardii (lancelets)	5	0	0	0
				Lepidosauria (Reptilia—Lizards & Snakes)	20	0	3	1
				Chondrichthyes (cartilaginous fishes)	3	0	0	1
				Cladistia (bony fishes)	1	0	0	0
			Mollusca	Cephalopoda	0	5	0	0
				Bivalvia	9	1	0	0
			Brachiopoda	Lingulata	2	0	2	0
			Echinodermata	Asteroidea	4	0	0	0
				Echinoidea	1	0	1	0
			Cnidaria	Anthozoa	4	0	0	1
			Annelida	Polychaeta	3	0	0	0
Total number of proteins					646	1902	27	24

**Table 4:** Comparative quantitative analysis of amino acid patterns at LXXLL (LM) and PXXP (PM) motifs in Proline-, Glutamic acid-, Leucine-rich Protein 1 (PELP1) proteins. A detailed analysis of amino acid patterns within these motifs for each PELP1 protein is presented in Table S5.

Motif	Human PELP1 Patterns	PELP1 Patterns
LM1	LXXLL	L(60)/A(13)-R(56)/M(18)-L(57)/R(24)-L(72)/R(11)-L(82)/T(10)
PM1	PXXP	P(63)/S(26)-V(80)-H(60)/L(16)-P(66)/L(18)
PM2	PXXP	P(43)/E(37)-H(46)/Y(31)-L(84)-P(84)
LM2	LXXLL	L(79)-L(43)/M(40)-C(44)/A(34)-L(46)/N(28)-L(48)/Y(38)
LM3	LXXLL	L(95)-L(95)-S(85)-L(54)/M(21)/V(16)-L(92)
LM4	LXXLL	L(98)-R(43)/K(27)/Q(18)-D(89)-L(88)-L(95)
LM5	LXXLLXXLL	L(52)/I(40)-P(65)/L(33)-G(91)-L(62)/I(36)-L(94)-T(93)-S(92)-L(97)-L(96)
LM6	LXXLL	L(65)/I(19)-H(77)-S(47)/C(40)-L(92)-L(94)
LM7	LXXLL	L(63)/A(34)-H(65)/N(30)-S(48)/G(26)/T(12)-L(71)-L(94)
LM8	LXXLL	L(80)/V(14)-R(78)(14)-L(82)-L(90)-L(60)/V(29)
LM9	LXXLL	L(68)/F(27)-T(25)/S(23)/A(12)-H(90)-L(94)-L(84)
PM3	PXXP	P(80)-S(60)/G(28)-A(59)/P(27)-P(61)R(27)
LM10	LXXLL	L(98)-Y(95)-R(72)/C(11)-L(91)-L(97)
LM11	LXXLL	L(95)-A(92)-L(93)-L(55)/V(39)-L(95)

Note: The percentage predominance of amino acids at each position is calculated assuming the total number of amino acids is 100%. Amino acids or patterns of amino acids contributing more than 10% at the specific position are shown in the table. Amino acids conserved (100%) as the specific position(s) are represented by their symbol and in bold. The numerical values in the table are percentage values.

**Table 5:** Comparative analysis of ɑ-helices in Proline-, Glutamic acid-, Leucine-rich Protein 1 (PELP1) protein across selected species belonging to different classes/superclasses.

Species Name and Its Taxonomy	Number of Helices in PELP1
*Capsaspora owczarzaki*|Filasterea	24
*Coemansia brasiliensis*|Kickxellomycetes|Zoopagomycota|Fungi	26
*Rhizophagus clarus*|Glomeromycetes|Mucoromycota|Fungi	24
*Phallusia mammillata*|Ascidiacea(tunicates)|Chordata|Metazoa	23
*Exaiptasia diaphana*|Anthozoa|Cnidaria|Metazoa	27
*Timema douglasi*|Insecta|Arthropoda|Metazoa	22
*Strigamia maritima*|Chilopoda|Arthropoda|Metazoa	21
*Daphnia magna*|Branchiopoda|Arthropoda|Metazoa	26
*Paralvinella palmiformis*|Polychaeta|Annelida|Metazoa	23
*Potamilus streckersoni*|Bivalvia|Mollusca|Metazoa	22
*Lingula unguis*|Lingulata|Brachiopoda|Metazoa	23
*Acanthaster planci*|Asteroidea|Echinodermata|Metazoa	26
*Branchiostoma belcheri*|Leptocardii (lancelets)|Chordata|Metazoa	25
*Petromyzon marinus*|Hyperoartia (fishes)|Chordata|Metazoa	17
*Perca flavescens*|Actinopteri(bony fishes)|Chordata|Metazoa	19
*Chiloscyllium punctatum*|Chondrichthyes(cartilaginous fishes)|Chordata|Metazoa	20
*Polypterus senegalus*|Cladistia(bony fishes)|Chordata|Metazoa	23
*Naja naja*|Sarcopterygii(Reptilia)|Chordata|Metazoa	21
*Varanus komodoensis*|Lepidosauria (Reptilia—Lizards & Snakes)|Chordata|Metazoa	25
*Microcaecilia unicolor*|Amphibia|Chordata|Metazoa	22
*Gallirallus okinawae*|Aves|Chordata|Metazoa	16
*Homo sapiens*|Mammalia|Chordata|Metazoa	27

**Table 6:** Proteins enriched with specific amino acids, their species name, protein ID, and the reference are shown in the table.

Species Name	Protein ID	Enriched Amino Acid	References
*Phaseolus vulgaris*	P10496	Glycine	[68]
*Homo sapiens*	Q8N4B5	Proline	[69]
*Homo sapiens*	Q86XN7	Proline and serine	[70]
*Homo sapiens*	P21291	Glycine and cysteine	[71]
*Gossypium klotzschianum*	A0A7J8VRY5	Glycine	[72]

## Data Availability

The original contributions presented in this study are included in the article/supplementary material. Further inquiries can be directed to the corresponding authors.

## Supplementary Materials

The following supporting information can be downloaded at: https://www.mdpi.com/article/10.3390/ijms262411989/s1.
