# Supplementary material for: Proline-, Glutamic Acid-, Leucine-Rich Protein 1 (PELP1): Diversity, Structural Conservation, and Evolutionary Origins Across the Species
Source: Int J Mol Sci. 2025 Dec 12;26(24):11989. doi: 10.3390/ijms262411989 (PMC12733202; doi:10.3390/ijms262411989)
Supplement: Supplementary file 1 [file ijms-26-11989-s001.zip › Figure S2.pdf]

Figure S2. Multiple sequence alignment of Proline-, Glutamic acid-, Leucine-rich Protein1 (PELP1) proteins belonging to different classes/superclasses. In the alignment, helices are indicated with red font. The LXXLL (LM) motif is highlighted using bold and underlining, and the PXXP (PM) motif is also emphasized with bold and underlining.

```
C.brasiliensis      ------------SEAVKANIDLVLDAVLSQNLFAFVGASDISAEITKRYSSAIHKWLARI    48
R.clarus            ---------------IEAHVPFILETITQHQLL------SSKNEPTREQQVALHKWCTRI    39
C.owczarzaki        ------------------------------------------------------------    0
E.diaphana          -----------------LSISTLLEAVSETQSL--------------QGSGCTKEWIGKL    29
P.mammillata        ------------------------------------------------------------    0
S.maritima          ------------------------------------------------------------    0
D.magna             ------------------------------------------------------------    0
T.douglasi          ------------------------------------------------------------    0
P.marinus           ------------------------------------------------------------    0
P.flavescens        ------------------------------------------------------------    0
P.senegalus         ------------------------------------------------------------    0
C.punctatum         ---------------------------VLNSDW--------------DNSSDLSGLISIS    19
M.unicolor          -------------------------------------------------STSIGGLTGIS    11
H.sapiens           -----------PHLPGL---MCLLRLHGSVGGA--------------QNLSALGALVSLS    32
N.naja              ------------------------------------------------------------    0
G.okinawae          ------------------------------------------------------------    0
V.komodoensis       ------------------------------------------------------------    0
P.palmiformis       ------------------------------------------------------------    0
A.planci            LEVLRQKSNTSSPDTSINLLSSFIEDSTELQIF--------------SQERNCASWIGHI    46
B.belcheri          -------LTSLSEKEGKTLLSWTIEAANEHQLL--------------QSEQSAQDWVSHI    39
L.unguis            -----------------------------------------------------------I    1
P.streckersoni      -------------------------------------------------------ILSYI    5

C.brasiliensis      NSLATGRTSDARMAGVLLIKHTAQQ-SPQLFSENVAKWNTVLLS-VLSKAEITPVIEVTL    106
R.clarus            NSLLQSKVTNARWAGVCFIKISLKQ-SEELFVQNLQSWTTSLMV-LLTKSEPTFILKEII    97
C.owczarzaki        -----------------------------SSHDWCLKLLQAISH-VMKSKSAAASLDAAL    30
E.diaphana          NSLLNS--NNDRWLGIALLGPTLQQCNTDVFTANCISWMKVLQHILELPTSSLSLIQTSV    87
P.mammillata        ---------AERWKGFYTMELVLSRCTEDVILKNGISWIEIAATTVVKNHIEESVRTIAA    51
S.maritima          --------------PLMLLNYLVEDCSDSVFVENCMSWLDKLSK-IIQVNCNVKIVSLAC    45
D.magna             ----NN--PNTRAEGALLLEVVITQCGTDMFTSNCVLWTQQVMR-LLHGPTKRTVGQNVF    53
T.douglasi          -----------RLQGLLILEAFLPQCSDQVFVEHAVSWMQQCLK-GVETRNDARTTALSY    48
```

```
P.marinus           -----------RWDGLSLLAALVRDCSTQVFVQHCCAWLRAVTQ-LLQPYEPEGGLALAV    48
P.flavescens        -------------------------SSSDLFQQHCVSWLRSLQQ-VIQSQAPVQTIQLAV    34
P.senegalus         -----S--VKTRFEGLCIMAVMVKDSSTELFQQHCLTWLRSIQQ-IIQSQDPLQTMELAV    52
C.punctatum         NSSLRS--VKSRFEGVCLLAVLVADSPTDTFQDQCISWLRAVQH-IIQSQDPQTTTELAI    76
M.unicolor          NARLSS--IKTRFEGLCLLSLLAAESPTDHFQQHCVSWLRSIQH-IIQSQDLPPTMELAV    68
H.sapiens           NARLSS--IKTRFEGLCLLSLLVGESPTELFQQHCVSWLRSIQQ-VLQTQDPPATMELAV    89
N.naja              --------PARVFEGLCLLSLLVTESGSETFAQNCLAWLRSLQH-LLQSQDPPATMELAV    51
G.okinawae          -----S--MKTRFEGLCLLSLLVSESPTEPFQQHCLGWLRCLQH-LLQSQDPAPTMALGV    52
V.komodoensis       -----------RFEGLCLLSLLVTESTTETFSQNCLSWLRSLQH-LIQSQDPAPTMELAV    48
P.palmiformis       --------SKSRLKGLLLLHQILDQCDSPIFQEHALSWSQMLLS-VLQSYNSDAVHQMAA    51
A.planci            NGCLNS--SKTRLEGLIVLRALVNQCPAEVFQQYCASWVRLLTQ-ILQSYDPPPVIALAS    103
B.belcheri          NTSLGA--AKTRLEGLCLLGTVVRQCSADTFIQHGTTWIRLLAQ-VLQAYDSPLTLQMAS    96
L.unguis            NSGLNA--AKSRVESLVLLNDLLSQCSTETLSQYTITWTRLLIQ-IIKGYAPASIHRLAC    58
P.streckersoni      HDCLNS--YKNSGPGLLLLSHLIKECSTEVFMENAMTWLRLCTQ-IIQSYNKAAVHSLAC    62


C.brasiliensis      QTLLLFIDAVRDIPMLYREIVSAHVPRMNQAILAMV-DKNTDLTVPVLEMLEHSATWFPT    165
R.clarus            TALTEIFNKTQNKPELQREITTQQLPRFNTFLIKLS-GLNKELLPTILNALSHSVKNFPT    156
C.owczarzaki        TCLARVLE-SHLPAELRRDILSTHLARLVQQTTALASSAGLTTQVAAFGALSAMIRSLSS    89
E.diaphana          TILDDLMRYTTQFPELMRNTSNSTIPAILSALLSL----QGKDTVCSLVGICACLKYFPG    143
P.mammillata        SNFAKLLEISTQIPSVSKELSSNHVINILQLLLSS----DNCNPVPCLACMEAIMKHFPA    107
S.maritima          DVVINIISFVVQNSDLNRQFCNSVLSNLVTSLLDI--------NEHKLCCLRKVMKTFPG    97
D.magna             KVLGKLLAFSSQFPELSRQLSTTVISQLVTLLCEPEFSQKASIAVHVLECLKSCMKYYGN    113
T.douglasi          KLLKHVLEMSSNFSELTKQVASSVIPKFLERFSK-----DMEGMLAGLRCLEVVLTNYGG    103
P.marinus           RVLADLLRFSAQLPELAREVSQSHLPALMSALLAL----RDQRECMAMEGLLACMRFYPK    104
P.flavescens        NILKDLLQYSSQLAELAREVGLNSILGILTSLLGL----KTECELAAMEGMTACMTYYPR    90
P.senegalus         HVLHDLLQYSSQLPELARNIGLNSIPGILTSLLSV----KPECHIAAMEGMHACMTFYPR    108
C.punctatum         WVLHDLLKYSSQLLELAREVAMNHVPAIVTALLAL----KPECQGAALEGMKACMTFYPR    132
M.unicolor          LVLHDLLKYSSLLPALSREISMNLIPGLLTSLLDL----KPECQAPALEGMKACMTFYPX    124
H.sapiens           AVLRDLLRYAAQLPALFRDISMNHLPGLLTSLLGL----RPECEQSALEGMKACMTYFPR    145
N.naja              LVLRDLLQYSAQLPEVARDIGTNHIPGLLTSLLAL----KPECQLPVLEGCKACMTFYPR    107
G.okinawae          AVLHQLLLFSAQLPELARDIGTNHIPGLLTSLLGL----KPECEVSTLEGIKSCMTFYPR    108
V.komodoensis       LVLRDLLECSCQLPELARDISTNHVPGLLTSLLAL----RPECQLSALEGSKACMMFYPR    104
P.palmiformis       EVLSELLHHAVTFPAISRDLATSLVPTIIPTLLDV----GTECELSAFRCLKTCLHFFTG    107
A.planci            VALRNILKEAAQVSELSREISNTFITPIITAALEL----KTEWQDSAIQILAACIQSFPG    159
B.belcheri          HVLAAVVQQAAQYPEVAREVASTHVPALVQSLLGA---QDHQWFPAALEALHSCMKNFPG    153
L.unguis            YVLGSLTEKSSTCPELARQVALDSLPQLIPALLVM----KEESLEAALYCIGKCMQFYSG    114
P.streckersoni      DVCANILSIATSFTDLSREITSSVIPQLLPPLLIA----NKEWRQFAFICINSCIRNFSG    118
                         .         : ::     :  :                   :
```

```
C.brasiliensis      LFRPSIDKAEALCLRLLDGSNSRMLLEQCQQAARCLAAFSLAGGKN------TPEERWFQ    219
R.clarus            IFRPVNDQTQKLCLNILLDDTCYYESELVKMAIECFPRIINFGGKL-------NMDYWKL    209
C.owczarzaki        AMRPHVDKVEAVCSEALDSVHI----DVVRAAASCLALCPTCGGSSSTYTQANPSDAWLV    145
E.diaphana          PSSQFKTQIEDYLISCLDRNSP----NIRKYCAQCFALVAGLSNNQND----KQKKNWDL    195
P.mammillata        PCNPHKGKVSKYIFPHTLSLKT----NAQDAAIRCWSMVALLGGGGQGRK--NHALQWEQ    161
S.maritima          PCRQFQIKIENLVMEIFLESTN---QQLQREAAQCFLILPNLNLESD--------VGWNE    146
D.magna             PCGPVKGAIERHLLSLIDWDEMATDSSLRAVWAACMAYLPSVGSSGSQGA--QHRSNWIE    171
T.douglasi          ACGPGRVSLERFLMKLVDVPTA---PRLLRAVGRCVALMPLVGGGGTQRT--NHRQQWIK    158
P.marinus           TSGTTKHWIAIAVLPQWLDNKP----RVTKARRA--------------SHYRCRRTCWQR    146
P.flavescens        ACGSLRDKLGAYFLSKMDSTNK----KTQEMACLCYGHLPCLGGLLDRGVGAGRAEGWTN    146
P.senegalus         ACGSLRGKLGAYFLSKMDSENT----RMQELACSCYSILPTLGSGFSQGI--KTSEAWAQ    162
C.punctatum         ACGSLRGKLAAYFLSKLDTGSP----HMQELACQCYVLLPGLGAGFAQGM--KYVESWAQ    186
M.unicolor          ACGSLRGKLASFFLSQLEAESP----QIQQLACECYTLLPSLGSGFAQGV--KHTECWEH    178
H.sapiens           ACGSLKGKLASFFLSRVDALSP----QLQQLACECYSRLPSLGAGFSQGL--KHTESWEQ    199
N.naja              ACGSLRGKLSTYFLSCMDAETP----HLQQLACECYALLPSLGAGFAQGL--KYRESWEQ    161
G.okinawae          ACGSMRGKLAAYFLSRIDSESP----QLQQLACECYALLPALGRGFSQGL--RHTECWHQ    162
V.komodoensis       ACGSLRGKLAAYFLSRVDAETP----QVQQLACECYALLPSLGAGFTQGL--KYTECWEQ    158
P.palmiformis       ACSTLKNKIEAKLREGLLKAKA--SSEALSIITECYTLLALCGGGGDKK-----------    154
A.planci            PCGTFRTRTENFLIQVMGSSGS---VDIAVSASQCLALLPRVGGGGQGGT--KHAEAWSY    214
B.belcheri          PCGPSKGKVESVLCGLMDTNQP----RLSLLVQQTCPLLAGCGGGGAGGV--KYAEAWAH    207
L.unguis            PCGTFKGKIENWVIDQLTKENP----RVVEAAVSVFPWLSQCGGGGNQGI--KHAESWSS    168
P.streckersoni      SCGPFKNKIEACVLEDIRTCKP------CKAAVVCFSLLARCGGGGNQGV--KYTEGWKQ    170


C.brasiliensis      C-----------MQKAAGTMRQCVDHI--MC-----VDAN--------------------    241
R.clarus            T-----------LLKLVGSLNNILDRL--FD-----TIDE--------------------    231
C.owczarzaki        L-----------ANRALLTATKLLDVVRIDETAYLAPAVAGTPSSSASSSAKATKTNGGS    194
E.diaphana          Q-----------WYRVVGTLNVTLDILHAYE-----STTSE-------------------    220
P.mammillata        Q-----------FDETLKAWKFISTNL--WD-----PKLDE-------------------    184
S.maritima          C-----------FKKILATIHNILTEL--FE-----TVERG-------------------    169
D.magna             F-----------CLQLIDSIHFTINGM--FR-----NIEEL-------------------    194
T.douglasi          A-----------HLTLCHTLHHLLNQL--YQ-----PAEDM-------------------    181
P.marinus           PTVYNAILKYKARHCL-LSLHSGLNAL--YR-----PSVRC-------------------    179
P.flavescens        Q-----------IHCLLASANGLLAQI--YQ-----GSESD-------------------    169
P.senegalus         Q-----------LQCILATAHSLLGQL--YE-----GAETD-------------------    185
C.punctatum         Q-----------LHYLLATLHGLVEQL--YE-----GAETD-------------------    209
M.unicolor          Q-----------LQCLLATLHATVGQL--YE-----EAETE-------------------    201
H.sapiens           E-----------LHSLLASLHTLLGAL--YE-----GAETA-------------------    222
```

```
N.naja              Q-----------AHSLVATLHCLLGRL--YE-----GAETE-------------------   184
G.okinawae          E-----------LRGLLGTLHRVLGGL--LG-----GSQNE-------------------   185
V.komodoensis       Q-----------AHCLLATLHSLMGTL--YE-----GAETD-------------------   181
P.palmiformis       ----------------------------------------D-------------------   155
A.planci            Y-----------CHRVLATIKDLTDKI--YH-----DSQPE-------------------   237
B.belcheri          L-----------CDQVLGSLHQVLDHA--YQ-----DMETG-------------------   230
L.unguis            N-----------CHKLLGSLHDTLDQL--YE-----GVESG-------------------   191
P.streckersoni      Q-----------CDQLTDSMSHTLSLL--YD-----GMETD-------------------   193


C.brasiliensis      -----SNDQPQQQFALAGL-----------------------------------------   255
R.clarus            ---EKGISKKLTGFEMPQV-----------------------------------------   247
C.owczarzaki        KGNKSAKNGQSGALPMDESTTEPVVANNVAAGVLLPLFPYLDQQQQLAQPAAGTNTSISS   254
E.diaphana          --I-----KIIETLPVKTI-----------------------------------------   232
P.mammillata        --GEVDPSIGSLSFHFDPL-----------------------------------------   201
S.maritima          --DPIWKSDYTESLSFVAI-----------------------------------------   186
D.magna             ---K-IQEVSSNPLKLPEL-----------------------------------------   209
T.douglasi          ---RT-LLPDAETLSLRKV-----------------------------------------   196
P.marinus           ---RRQTDGDEADMALPEP-----------------------------------------   195
P.flavescens        --VAVQYEGSGVELAFPHL-----------------------------------------   186
P.senegalus         ---PVIYMGPGVELNFSPL-----------------------------------------   201
C.punctatum         ---PVRYEGPGVMLPLPEL-----------------------------------------   225
M.unicolor          ---PLHYEGPGVELLLPVA-----------------------------------------   217
H.sapiens           ---PVQNEGPGVEMLLS-S-----------------------------------------   237
N.naja              ---PLHYDGPGEEVLLPPR-----------------------------------------   200
G.okinawae          ---PLPYEGPGVEMLLPPP-----------------------------------------   201
V.komodoensis       ---PLHYEGPGMEIPLPAP-----------------------------------------   197
P.palmiformis       ---IDSYRRPASRLFTVDC-----------------------------------------   171
A.planci            ---KQSDS-SHPPFTMPDA-----------------------------------------   252
B.belcheri          ---LQTYNTPQASLHLPTV-----------------------------------------   246
L.unguis            ---VEENKKKSEKLSLSPV-----------------------------------------   207
P.streckersoni      ---KNLREQCAKLIFTSTV-----------------------------------------   209
                                 .

C.brasiliensis      ----------SDDFT-KSIPQAADRIAAMAEVIVALLTQPTPMEISVPADRIVDAASRVA   304
R.clarus            ----------SEDYV-VAFPILFSRFKCLSECLISLISLPTSSPVQIPVNQILDLLYRVY   296
C.owczarzaki        LSQAALARDANVNSP-TLSALIARRIQATFIVLAAMLQQPVSFVVSMPTHRILSLRHV-L   312
E.diaphana          ----------ETDEP-KKTNTCTSRLRSLLKCLDAMIRQGSRRVSMVPLDITIALLERVL   281
P.mammillata        ----------PSSEP-DLSITLVQRFLMVCKCLCGLLNLNFPHLLNVSVDKIFNLLIANF   250
```

```
S.maritima          ----------PNAQPMLKFQSLYQRFNLLCYCISEMLSGVYSGIKQIPVRDVIGFVTRVL    236
D.magna             ----------QHKNPMVLLHDQQRRFVNLCSALVVLLNEPFPTTKNVPIDRFLAMISRLL    259
T.douglasi          ----------RDKDPVTRVQRLTTQLGNVAKFLQAMLNGVFPVPKNVSAQAVLDVVCRGL    246
P.marinus           ----------QADSP-VYLLLVKQEFSCLARALFAMLREEFAAPVKVPAQPILTLVCRAL    244
P.flavescens        ----------DQSDP-LLLLQLQHRYTAVCLALKHTLRVDPASAVRLPVRPILNLVCRVL    235
P.senegalus         ----------DERDP-LFILRLSQRYAGLCQSLSKLLSIDVTVPVKLPVQDIINLVCRAL    250
C.punctatum         ----------NDGDP-FHVLQLRQRFSALSKCLSLLLSSDFPVPVKLPVQDVLNLVCRIL    274
M.unicolor          ----------DDGES-LHVLRLKHRFSGISKCLCLLLSSSFSVPVTVPVQDVLDAICRVL    266
H.sapiens           ----------EDGDA-HVLLQLRQRFSGLARCLGLMLSSEFGAPVSVPVQEILDFICRTL    286
N.naja              ----------EEE-A-NSLLLAKCQFAGLAKCLCQMLRNDFGAPVAVPAQAILDLVCRAL    248
G.okinawae          ----------QDGDT-GLLLTLHTRFSGLARVLRLLLSKDLVAPVTVPVQDILDLVCRAL    250
V.komodoensis       ----------EEGET-NFVLHLKQRFSGLAKCLCRMLSNEFVAPVTVPVQDILDFICRAL    246
P.palmiformis       ----------PDAEP-TRTQMLVHHYRVLAQTMATMLRPSVSVTLHVPVEDILTIICRAT    220
A.planci            ----------PLKEP-TRMYTLVRQFKVMSACLQHMLRAEFPEMVKIPVKSILSLCVRIL    301
B.belcheri          ----------PESDP-ARTFVLSTRFHNLCGCLQQLVSQEFPSVVRIPTPDILSFLCRAL    295
L.unguis            ----------QGTGM-ERIVGLRNRLDMLCGCLGGMLSTTFPAVVRIPVPEVIGFIQRAL    256
P.streckersoni      ----------PDSLS-ERLTNLVGRWKVLCDCLSSLLSESFPAVVNLPIERILGLICRTL    258
                                            .       :   :         :     .

C.brasiliensis      MISMRA-ANSKSKRAEYDLIPLLTPQLQRASIR---------------------------    336
R.clarus            NIYDVSFVTDSKDKNEYYTLMLGIPSLLINCNK---------------------------    329
C.owczarzaki        YLSEQQLLQEASKRPNKLAWIAWLPFLQHCTLD---------------------------    345
E.diaphana          SLNGKMLRGSI--SVDNVLLQGCLPLIHLDAVH---------------------------    312
P.mammillata        DVTPKSLKHII----ESHLSVDCLQTVHIHCLY---------------------------    279
S.maritima          IITPFSIKS---ATTENLMLAGVLPLLHEYAFR---------------------------    266
D.magna             ALNSKSLSKTSRANFEQLTLASIIPDLQSSMLD---------------------------    292
T.douglasi          SVQCASLLSRNS-SSEAVILACHLPDIHLQLLD---------------------------    278
P.marinus           AISPKRLAAVG----EATMQLLVLPAVHTDALS---------------------------    273
P.flavescens        AVSSKSINLTG----DGSVRLLVLPVIHTHTLE---------------------------    264
P.senegalus         AVTRKKLNWLG----DGPLKMLVLPTIHFRTLD---------------------------    279
C.punctatum         NISTKNM-----------------------------------------------------    281
M.unicolor          SINSKNISWLG----DGPLKTLLLPSIHAEVLD---------------------------    295
H.sapiens           SVSSKNISLHG----DGPLRLLLLPSIHLEALD---------------------------    315
N.naja              DVSVKSMSWFG----DGPLRMLLLPSIHLEALD---------------------------    277
G.okinawae          NITSKNLSWFG----DGPLKMLLLPSVHMDMLD---------------------------    279
V.komodoensis       DISMKNISWFG----DGPLRMLFLHSVHLESLD---------------------------    275
P.palmiformis       SISPKFLMTRP--THQRVLLCSVLPTIYKMSLH---------------------------    251
A.planci            VPS--MLFKGL----KSALQTGNFSS----------------------------------    321
B.belcheri          GVNAKMLFGKA--SMEHVLLMSALPKMHCSALS---------------------------    326
```

```
L.unguis            AVTGRALLLRP--STDNLQLLSVLPFLHTSVLRVLSCLISRYVLPFLHTSVLRVLSCLIS    314
P.streckersoni      SVHGKMLLSRP--TTERVILASLIPAVHESALE---------------------------    289

C.brasiliensis      -------------IMAALAI----SLGSHMQPFLSAVARTATAIHTQQ------------    367
R.clarus            -------------VLSAVTL----SVGEHLTRHLRMVSSILLKLLNNS------------    360
C.owczarzaki        -------------LLNVAIT----RCGAGILGDADDMLQTLVHVLEQHRR----LQPV-H    383
E.diaphana          -------------LLCSLVQ----RCGSLLLPNANRIAKLMVNELS-WMSDKHLNASQNG    354
P.mammillata        -------------VLRACIE----SGHTALCRRATQVNRMFLNLLTMWRD----TNPTFG    318
S.maritima          -------------VLHSLII----VCRKYLIPESSTISKLIIKGLK-WTS---RVQSETT    305
D.magna             -------------VLKSLVA----VCRTQLLTRATTVMNFFVQVLQ-WTFTP-VDRRRVG    333
T.douglasi          -------------VLKSLIL----CAGRNLLPHASIICKLVLQELK-WTS---TDSPQYG    317
P.marinus           -------------LLEALIRTRAARCGKRLLRFGDVINRAFPQVLATWSPPA-ETGGDPG    319
P.flavescens        -------------VLSALIT----AVRSGMVQYAAVLQRLFSQTLSAWAPPL-E--ASLG    304
P.senegalus         -------------VLIALIE----TGGSRLIRYSTVLCHLFAQTLNAWSFTN-DT-ILPG    320
C.punctatum         -------------------------------------------VLTAWSTPR-ES-AVPG    296
M.unicolor          -------------LLAALIM----ACGSRLVRFADLFCRLFAQVLTLWSGKR-DT-VIPG    336
H.sapiens           -------------LLSALIL----ACGSRLLRFGILIGRLLPQVLNSWSIGR-DS-LSPG    356
N.naja              -------------LLAALIL----ACGPRLVRFGGALCRLFPQVLNMWRAGQ-DL-PSPG    318
G.okinawae          -------------VLAALIL----ACGARLVRWGSVLGRLFPQVLSAWSSAR-DP-PPPG    320
V.komodoensis       -------------LLFALIL----ACGPRLVRFGGTLCRLFPQVLTAWSAGR-DL-VPAG    316
P.palmiformis       -------------MLKHLIT----CVETGLILHCSVINKLLIQILD-SVAGC-TDGGDGN    292
A.planci            ------VHSEAFNILAALLI----SCRGNLSLHKDIINELFVKSHA-FRP---SVQSDDA    367
B.belcheri          -------------ILEALII----SCRSYLVPHASIISQLLIQTLG-WTT---SEEGVPG    365
L.unguis            RYVLPFLHTSVLRVLSCLIL----SCQKNLISYTSTVNQLLIQTLS-WTI---TEDTADG    366
P.streckersoni      -------------MLKVLFT----CCKGLLIPFTRVISDMISQELS-WTK---SVDQ-YG    327

C.brasiliensis      --IVSPTISVALHSLLRLFIERYGYG--FVTH---LPYDIIVSVVN-DICVHKKSHAPST    419
R.clarus            --KTRWLLRVSTYNLISLCMQKYGIG--LTNF---ISSSILLSFVIDDIEIIQKSQYDVS    413
C.owczarzaki        RQRKA-ETLVAALTTLETLLSTLG---GMLDATQLGSVGELL----CEILCGGALFQAVS    435
E.diaphana          SIKPYSNLSCAVYDCILHWGQLNA---GFHE----DVIKDITRYLLKEIKVGKNIETKLR    407
P.mammillata        MSKNCIKIRLNIYGVMLYWVESCGSKSRFLMG-QKHFTQPLLEYMVENFSLIPDYE-RGN    376
S.maritima          SKKPFSDLRCQIYSTLTLEN------SSVE------------------------------    329
D.magna             IERPYGKLRSQVYRNLCLWVVASRSACGWGK-----CVDVLFSQLLSDILVHRDTV-QLL    387
T.douglasi          VERPYRVLRTKAYETLVTWLRAAKVGSSVEK-----VSDELVIALIQDIHTNKADV-TLT    371
P.marinus           RESPYSEVRVRAYGALAAWVDACGATCRVLQD-ELQHAHDLLRHLVRDASPASDLI-RVR    377
P.flavescens        QQRAYSSVRVSVYRTLELWIQVAGASASILQG-SPSHSELLFSHLLGDITPGAESV-KLR    362
P.senegalus         QQRAYSTVRVQVYKTVQIWVSVAGSSSGVLQG-SANSSEVLLGHILSDITPGTDTI-KLR    378
```

```
C.punctatum         QERAYSSVRVKAYEVLGTWLKVCGASSGVLQG-PFHHCDILLANLIADVTPAVDTT-KLR    354
M.unicolor          QEKPYSAVRTKVYQVLELWIKVCGASSGVLQG-AGHQCDVLLAHLTSDISPPTDTI-KLR    394
H.sapiens           QERPYSTVRTKVYAILELWVQVCGASAGMLQG-G-ASGEALLTHLLSDISPPADAL-KLR    413
N.naja              LQRPYSAVRARLYQVLDLWVQVAGAASGVLLG-HSSQSDALLGHLINDISPPSDAL-KVR    376
G.okinawae          QERPFGAVRTRLYEVLELWVQVAGAASGVLQG-AGTPPEVLLTHLLNDITPPADNV-KLK    378
V.komodoensis       QERPYSAVRTRLYQVLDLWAQVAGAASGLLQG-HGTQSEALLGHLLSDISPPTDTL-KLR    374
P.palmiformis       HDNPFSELKQSVYEVASTWLIVAGTTSAMVI-----VAGDFIDHILTDARPRVETI-KSI    346
A.planci            KYRVLSGLKLSVYGALEIWLETSGSCSGIET-----GADKLLEAILNDARPQAQMT-KLR    421
B.belcheri          RQRPHSTLRSRAYTVLTVWLNVCGAAGGVDS-----HADIILQHVLTDTTPQADTT-KLK    419
L.unguis            RKRPYGNLRKEVYSTLVVWVQTLGASSNVHN-----IIDQLLTQLKRDITPDVDVI-KLE    420
P.streckersoni      QEKPYGQLRRLVYECVITWCQNLDSNTGITG----DDEAQLIHQILHDLTPQMDIL-KID    382


C.brasiliensis      NSTKA-DHTASKKRSGNGKSRNPSA-LNTDE----SVHTTRIHWNDTG----LAALSTVL    469
R.clarus            INT---TVSTNNKKSG--KKKESTQITNSDA----LVSSSGIIYLQTNSDVQCSALEVLK    464
C.owczarzaki        PAT----VPQSHLSGKKQKQQQQQKNIEESLGAASGALAVSRPLQFANTDICKLSLKALT    491
E.diaphana          AASAE--GNKQKGLSKKGKK---RKMQAEIE---QLSTNPEKSDVYCNEKLCHAALTTLF    459
P.mammillata        TDIQ------AKKPIVSGRKKS-KRLINS------DVSFSRIVDSSANSQVCIASLKVLR    423
S.maritima          ---------------NNRKKRK-RGNTFSGV----GSVGPIKEDPLVNNSICLNCLKALK    369
D.magna             TVNTP----SNAKMTRKGKK----KM---DT---TG-VILQKDDLTANADVCQMALQCLS    432
T.douglasi          VLGA-----KNKNLSKRQK----RKLAQDDRSQTTSSQRVKLLNHCANSSLCSAALSLLQ    422
P.marinus           ------------------------------------------------------------    377
P.flavescens        VGLSA----DAVPGGKPGPRRT-KQLVIVDT---VGPSLQRKGDLLANQDTCLSALRALR    414
P.senegalus         TEKAMP--ELSNNIGKVGGKRT-KGLDVHEA--TSHMQGYRKQESNANSDCCKAALKSLN    433
C.punctatum         PSKPAGDGSQPFNYGKPSAKRQ-KLMDLEDA---AAMPGHRKRDPTANGDVCLAALKVCS    410
M.unicolor          VGR---LGAEILSNGKPGS-KK-QKMDVGDG---PQLQNHRKQDVNANSDTCTMALRVLS    446
H.sapiens           SPRGS-PDG-SLQTGKPSAPKK-LKLDVGEA---MAPPSHRKGDSNANSDVCAAALRGLS    467
N.naja              PPPSP-LGVSPTSEGKPSAAKK-PKLSPVGS---LG-CPFRKHDPQANSDVCLAALQEAL    430
G.okinawae          A-----------------------------------------------------------    379
V.komodoensis       ESA-------PGPDRRPSAPKK-PKLAGL------G-PLHHKHDPRANSDVCLAALGLVR    419
P.palmiformis       SGSKI-LEAAEQILGKRKKKKG-KGSKEYLQ-----SSWQHKSKPFANWELCSKALEALR    399
A.planci            VTTE---KPTSMNAKKSGKSKQ-KGETAGGETTTTSLASQQKTSPPSSSRVCLAALKVLR    477
B.belcheri          ASRP-----GSQEPPRRQKKKQ-RGVDVSDQ----GLSGHRKVDSQANSDVCSAALGVLK    469
L.unguis            TINK---KPDQYTE-PPNKRK--KGNKAGLE----GVSTRKVLDHSANADVCLSALAALR    470
P.streckersoni      SSKSS-QKPGADTV-LSGKKKK-GGYSEISK----GISTQRKVDLTANAALVSSGLAALN    435


C.brasiliensis      VLLQRTPTALATALRTRIDSQILTLLMLFSIGGIE---------IPFASRQTLTSFKVLLY    521
R.clarus            ILL---------------------------KTCGSS---------ISSNKRLSLD--NILLS    488
```

```
C.owczarzaki        RLLYTAGSRLPDAVRARIDATASRLVLETQLGAEAISLL-SMQRPACSPMVEPTCRRALY    550
E.diaphana          SVMMNFGDNMKKGTFQEIQLTLLEVVSLFQPSS---------QGVFPQPYNSPDCRKEIY    510
P.mammillata        ICIYSNGSMLPSFVHKSLQKHVILLLMDIMRAK-----------NIIPYTHCAGCRKELY    472
S.maritima          AVHLAIGSTISSAVYLEITKFTIHLMCDVQKTI----------SNRPTPYDDFKCRHALY    419
D.magna             TILLCCGPRIKPAIHKEMQEIVLSILVDIMNGA---------ELNLLVPYNDPRCRAAMY    483
T.douglasi          WILRTAGCLVKPSLHKVLQETTLGLILDIQRSS--------GPAQFPAPYTVANCRRELY    474
P.marinus           ------------------------------------------------------------    377
P.flavescens        QIILASGTLLKDDIHKRLHDVVLPLCVRLQQQQSSSSISCESAGGVSGQYSSALARQELY    474
P.senegalus         LIIINSGTLLKEETHKKLQELVVLLVGFQQHGSLAA------NGTQTSPYFSPENRKELY    487
C.punctatum         DVILSCGSLLKEETHKKLHELVVPLLLRLQQNS-----------SMTSPYLRADCRRELY    459
M.unicolor          RAILLCGTLIKEDTHRRLHELVVPLLIHLQQST----------LPMTSPYASSECRKELY    496
H.sapiens           RTILMCGPLIKEETHRRLHDLVLPLVMGVQQGE----------VLGSSPYTSSRCRRELY    517
N.naja              -----------SFCPQRLQELAIPLLIRLGQAD----------LPLGSPYASASCRRELY    469
G.okinawae          ------------------------------------------------------------    379
V.komodoensis       RSPRA----PCLNPTQRLQELVIPLLIRLGQAE----------PLPGSPYASAACRRELF    465
P.palmiformis       QLMIHCGTELKPFDHKKIQDFIIPPLLSISQC----------SETVAPPYGHPDCRKRLY    449
A.planci            CLLTSIGTRVKPNFHKDVHEFVIPLLLHLQQLP---------PILIPAPYSNADCRRALY    528
B.belcheri          TVLEVVGPIIKPSFHKEVQEFVIPLLLKIQ-QN---------QSDPPIPYSCAKCRKGLY    519
L.unguis            HLLVAVGSLLKVQRIRDIQEIALPLALRVQAFT---------DQGSPGPYSDPECRRELY    521
P.streckersoni      WLLTTSGSSLGRKALQGIQEFVITTLLTIH-QS---------IMSPPIPYTDSECRQSLY    485

C.brasiliensis      RCL--EASLLSPD-PWQRAIIPHAISAFNCGL-EDPSPQVQKVCSEALLTIDPIVHSRLP    577
R.clarus            RILLRNISSTYNS-KYQPTILPHALRIFSAGQ-NSQSIQLRSFCSYALSICDLIFHNRLP    546
C.owczarzaki        RLLL--ACVVASASPVHSPVLMQVLRLLTTGT-RDPALKVSTFCREAVAVCDNIIRPKFP    607
E.diaphana          HCLL--GCVLSRP-SGAPNAISCVIKYLNDGL-QDMSFQVSNYCREALTITTALIHPVVP    566
P.mammillata        HLLL--ALTVVPS-PEYPAPLQLALTIIRHGSARDSDMDVRSFCMEASATLSNIVRPRIP    529
S.maritima          DVIL--AFVLVAP-QETGSCIQLCMKIFSCGR-QDAYQKISSLCLQAQSTCLEIIHPRLP    475
D.magna             RVLE--KLVLCPS-PQWPAPLNYASAIFSSGM-NDPNIEVSSVCIEALASIQSILRPRGP    539
T.douglasi          ELLL--VLVLEPH-PKWPPPTHLAMRAFSLGQ-IDSHQEVSAVCISALSSIEKLIHPP--    528
P.marinus           ------------------------------------------------------------    377
P.flavescens        RLLL--ALVLVPS-PCWPPPLTCAVSILSNGR-NDHNLKVSTFCTEALTICNSLLHPRLP    530
P.senegalus         HVLL--ALLLVPS-PRWPPPLQCAVRAFSLGL-GDINIMVASFCREALAICNVLIHPRTP    543
C.punctatum         RLLL--FLLLVPN-SKWPPPLHCAVRMFSQGQ-NDSNTEVSSFCLEAMVICNCLIHPRVP    515
M.unicolor          RLLL--CLLLTPH-PRWPPPLHCAVCIFSQGQ-KENNMKVSSFCAEALVICNCILHPRVP    552
H.sapiens           CLLL--ALLLAPS-PRCPPPLACALQAFSLGQ-REDSLEVSSFCSEALVTCAALTHPRVP    573
N.naja              RLLL--ALSLAPA-PTC-------------------------------------------    483
G.okinawae          ------------------------------------------------------------    379
V.komodoensis       RLLL--ALVLAPA-PACPPPLHCALWLLKQGC-ADPSLQVSSFCSEALVVCNALLHPRVP    521
```

```
P.palmiformis       SVLE--ASVLVPS-ALWPPPIHVALSVFSKAAVCESDLETRSYCHQALVICQNLVHPRIP    506
A.planci            HVLL--SSVTAPH-PRWPAPLQCTAGLFSRGR-QDASINVSSFCAEASIICQSIIHPRVP    584
B.belcheri          QLLL--AAVLVLH-PRWPPPTQCAVKIFSVGQ-QDYDLQVSSYCREALLTCMSIIHPRAP    575
L.unguis            HVIQ--CLFLIPA-SKCPPPTHCVIRLFTGGQ-NDPDRKVSSFCQEALTICEAVIHPRVP    577
P.streckersoni      RVLL--ALSLLTH-HTVPPPLQCALGLFRTGL-LDTSLKVSSFCIEASRVCEALIHPRVP    541

C.brasiliensis      A  578
R.clarus            P  547
C.owczarzaki        T  608
E.diaphana          S  567
P.mammillata        A  530
S.maritima          P  476
D.magna             -  539
T.douglasi          -  528
P.marinus           -  377
P.flavescens        S  531
P.senegalus         S  544
C.punctatum         S  516
M.unicolor          S  553
H.sapiens           P  574
N.naja              -  483
G.okinawae          -  379
V.komodoensis       A  522
P.palmiformis       P  507
A.planci            C  585
B.belcheri          T  576
L.unguis            S  578
P.streckersoni      C  542
```
