# Supplementary material for: Proline-, Glutamic Acid-, Leucine-Rich Protein 1 (PELP1): Diversity, Structural Conservation, and Evolutionary Origins Across the Species
Source: Int J Mol Sci. 2025 Dec 12;26(24):11989. doi: 10.3390/ijms262411989 (PMC12733202; doi:10.3390/ijms262411989)
Supplement: Supplementary file 1 [file ijms-26-11989-s001.zip › Supplementary Dataset S1.pdf]

Supplementary Dataset S1: Proline-, Glutamic acid-, Leucine-rich Protein1 (PELP1) protein sequences used for the phylogenetic tree construction.

```
> Catagonus wagneri [A0A8C3VJ76]
MELAVAVLKDLLRYAAQLPTLFRDISMNHLPGLLTSLLGLRPECELSALEGMKACMTYFP
RACGSLKGKLASFFLSRVDALSPQLQQLACECYSRLPSLGAGFSQGLKHTESWEQELHSL
LASLHGLLGALYEGAETAPVQYEGPGVEALLSPSEDGDAHVLLRLRQRFSGLARCLGLLL
SSEFGAPVSVPVQEVLDVICRTLSVSARNISLLGDGPLRLLLLPSLHLEALDLLSALILA
CGGRLLRFGALISRLLPQVLNAWSIGRDTLSPGQERPYSTVRTKVYAVLELWVQVCGASA
GVLQGGASGEALLTHLLSDISPPADALKLRSPRGSPDGGLQTGKPSAPKKLKLDMGETVA
PPSHRKGDSNANSDVCAAALRGLSRTILMCGPLIKEETHRRLHELVLPLVMGVQQGEVLG
SSPYTSSRCRCELYRLLLALLLSPSPHCPPPLACALQAFSLGQREDSLEVSSFCSEALVT
CAALTHPRVPPLQSMVPTCPTPAPVPPPEALSPFRAPAFHPPGPMPSVGPMPPARPGPPA
TANHLGLSGPSLVSVPPRLLPGPENHRAGSNEEPVLAPSGTPPPTLPSDETFGGRVPRPA
FVHYDKEEASDVEISLESDSDDSVVIVPEGLPPLPPPPTSGTTPPPVAPAGPPTASPPVP
AKEEPEELPAAPGPLPPPPPTPVPGPVTLPPPQLVPEGTPGGGAPALEEDLTVININSS
DEEEEEEEEEEEEEEEEEEEEDFEEEEEDEEEYFEEEEEEEEEEFEEEFEEEEGELEEEEED
EEEEEEEELEEAEREAGSPPSVPPPQELVEEEPPVPPTLLEEGAEGGGDKVPPLAETSAA
EEMEMETESAALQEKEQDDTAAMLADFIDCPPDDEKPQPATEPDS
> Peromyscus maniculatus bairdii [A0A8C8T9F8]
MELAVAILRDLLRYASQLPTLFRDISTNHLPGLLTSLLGLRPECEQSALEGMKACVTYFP
RACGSLKGKLASFFLSRLDALNPQLQQLACECYSRLPSLGAGFSQGLKHTENWEQELHSL
LASLHSLLGTLYEGAETAPVQSEGPGVEMLLSQSEDSNTHVLLQLRQRFSGLARCLGLML
SSEFGAPVSVPVQEILDLICRILSISSKNINLLGDGPLRLLLLPSIHLEALDLLSALILA
CGGRLLRFGALISRLLPQVLSTWSTGRDTLAPGQERPYSTIRTKVYAILELWVQVCGASA
GMLQGGASGEALLTHLLSDISPPADALKLCGPRGSSDGGLQSGKPSAPKKLKLDVGEALA
PSSHRKGDRNANSDVCAAALRGLSRTILMCGPLIKEETHRRLHDLVLPLVMSVQQGEVLS
GSPYNSACCRRELYRLLLALLLAPSPRCPPPLACALKAFSLGQEEDSLEVSSFCSEALVT
CAALTHPRVPPLQSSGPACPAPAPVPPPEAPSPFRAPPFHPPGPIPSIGAMPSPGPAPPA
GPLPSAGPVPSRPGPPATANHLGLSVPGLVSVPPRLLPGPENHRAGSSEDPVLAPSGTPP
PSIPPDETFGGRVPRPAFVHYDKEEASDVEISLESDSDDSVVIVPEGLPSLPPPPPSGSP
PPVAPAGPPTASPPVPAKEDSEELPATPGPLPPPPPPPPPPVSGPVTLPPPQLVPEGTPGG
GGGGGGGGGGGPTAMEEDLTVININSSDEEEEEEEEEEEEEDEDEEEEEDFEEEEEDEEE
YFEEEEEEEFEEEFEEEEGAEEVPEPETAPTLAPEVLPSQEEVEQEGGSPTAGPPQELVE
EEASAPPALLEEGTEGGGDKVPPPPETPAEEEMEAEAEAAAPQEKEQDDTAAMLADFIDC
PPDDEKPPPAEEPDS
> Notothenia coriiceps [A0A6I9MQI2]
SQAPVQTIQLAVNILKDLLQYSSQLPEVAREVGLNSILGILTSLLGLKTECELAAMEGMT
ACMTYYTRACGSLRDKLGAYFLSKMDSTNKKTQEMACQCYGRLPSLGGLLDRGVSAGRAE
GWTNQIHCLLASANGLLAQIYHGSETDGTVQYEGPGVELAFPHLDQSDPVLLLQLQHRFT
AVCLAVKHTLRVDPSSAARLPVRPILNLVCRVLAVSSKSINLTGDGSVRLLVLPIIHTNT
LEVLSALITAVRSGMVQYAAVLQRLFSQTLSAWTPLPEASLGQQRAYSSVRVSVYRTLEL
WVQVAGASASILQGSPGHSELLFSHLLGDITPGAESIKLRAGLSSDAVPGGKPGPRRTKT
LVMSDSVGPSLQRKGDVLANQDTCLSALRALRHIILTSGTLLKDDIHKRLHDVVLPLCVR
LQQQQSSSSTSCESAGSVSGQYSSAPSRKELHRLLLALVLVPSPCWPPPLTCAVSILSKG
RNDRNLKVSTFCIEALTICNSLLHPRIPSIALPLPPLTLKPTPNAPVLPTSQAPGLTLPT
LLGGPSPGTPFPTRHSLGLGSASLLGSLENHLSLVPGLPGQTDMILSPHAHHQQDAAGLG
LPEGQRPVFVRYDREEAEDVEISLASDSDDSVVIVPPGMLHLETQQDEAATAANSLNIVN
APGGATLPLPESLSMVPTTAATTIDRVSLPNDLATSSPLLTTSTTPINSFTPSNSSVVSL
VPPLNSSSLSAAPLGDSLPGRPQLQQMLMQPPAAGQPGPIGLPLQMHQLQNQLSQSGRHL
HQHQAPPASNEDSAVININSTDEEDEEEEDDEELDEEEEGLDEEDEDEEASDFVEEEFYD
GEEYDDYDEEEGEELEEDEEEDGEIPPLEGTEDKAGEAGMEGGKVLRAVVDEGGMAGFSV
EAEAEGGIEEIQTNRAMFGEDRVKVQKVESIGVLEEAREGEEDENERMDDPTMPQILCVT
GGALEDREEAEEEGGAARGGEGGEQEEARTWEQGANEMELKAASEKCTANQNQESADEPA
QEASVSDSLPSNQQEAQPEGERDPAAAEPATSTGPKTKQEEEAEQMEAGGGESDGEEGKG
VKRKREETNREEEPRSEKTKMDDDSMASMLADFVACPPDDEDEPPDPTAPR
> Poeciliopsis prolifica [A0A0S7LEI3]
GLCAGVLSRFQGLCLLSMLVKDSSSDLFQQHCLSWLRSVQQVIQSKAPCQSIQLAVSILQ
DLLQYSCQLPELAREVGLNSILGILTSLLGLKTECELAAMEGMRACMVYYPRACGSLKHK
LGAYFLSKMDSTSRKTQEMACQGYSRLPCLGGLVDRSVGTGRAEGWTNQIQCLLASANGI
LSQFYKGSETDEAVQYRGPGVELPFPHLDQSDPLLLLQLQHRYTAVCLALKHTLRADPAS
AVYLPVRPILNLVCRALAVNPRNTSLTSDGSLRLLVLPSVHINTLEVLAELITVVRSGMV
QYAAVIQKLFSQMLCAWTAVPEANVGQQRAYSSLRVSVYRTLELWLQLVGVSANVLQGAS
SHAELLFTHLLADITPGAESIKLRAGLSADVVPGGKPGPRRTKQLVMADPVGASVQRKGD
PVSNQDTCLSALKVLRRIIQTSGTLLKNDIHKVRICQNCT
> Nothobranchius pienaari [A0A1A8LH09]
MAASAWLHGSAAMRLTEGLVLLMKEQRPEFLPEVLANYREHGVLSLQSASDVAGLVGLCN
GKLSSSKTRLEGLCLLSMLVKDSSSDLFQQHCLSWLRSLQQVVQSQAPVQTIQLAVNVLK
DLLQYSCQLPELAREVGLNSILGILTSLLGLKTECELSAMEGMKACMVYYPRACGSLKDK
LGAYFLSKMDSTSRKTQEAACQGYSHLPCLGGLLDRAVGASRAEGWTNQIHCLLASANSL
LAQIYQGSETDEAVRYQGPGVELAFPHIDHAEPLLLLQHRYAAVCMTIKHTLRTDPASAV
SVPVRPILNLVCRALAVNSKNMILTADGSLRFLVLPSVHINALEVLSELITVVRSGMVQY
AAVIQRLFSQTLSAWTPLPETSVGQQRAYSSVRVSVFRSLELWVKVAGASACILHGTSSH
VDLLLNHLLGDITPGAESVKLRAGLSADVVPVGKPGPRRTKQLVVADAVGPSLQRKGDLL
SNQDTCLAALKALRQVLQSTGMLLKDDIHKRLHEVVLPLCVRLQQQQSSSISAWEPAGGV
SGQYSSALTRRELYRVLLALVLVPSPSWPPPLTCAVSILSNGRIDRHLKVSSFCAEALTI
CNSILHPRRPSLALPLPPLTLKPTATPSVLSCQGPGPRLTLPTLLGGPSSAPPFPARHTL
```

NLGPSSLLGSLDNHFSLVPGLPGQAPGPGDLMASSQTHHQPDPSGLAPPEGQRPVFVRYD
KEETEDVEISLASDSDDSVVIVPPGMLNVEKQQNDLAANSQSLLSSAPGGAELVTMVPTS
STPTTLDVTSLPNDLATSSALLTSSVTPVNSFPPSSTSVVSLVPSVNSSSASAPPGGLGD
PLAGKPQLQQMLMQPSTTGQPSSMALPLQMHQLTQQGRHLHQHPPTPAANEESAVININS
TDDEEDDEEDMEEDEELEDEEDGIDEEDEEEEGSDEFYEGEDYDDFDEEEGEELGEEE
> Homo sapiens [C9JFV4]
MLLPSLSPRVQPAFTQCARPQPPPHAPSPVGGRRGVFASSRHATTGTRGKMAAAVLSGPSAGSAAGVPGGTGGLSAVSSG
PRLRLLLLESVSGLLQPRTGSAVAPVHPPNRSAPHLPGLMCLLRLHGSVGGAQNLSALGALVSLSNARLSSIKTRFEGLC
LLSLLVGESPTELFQQHCVSWLRSIQQVLQTQDPPATMELAVAVLRDLLRYAAQLPALFRDISMNHLPGLLTSLLGLRPE
CEQSALEGMKACMTYFPRACGSLKGKLASFFLSRVDALSPQLQQLACECYSRLPSLGAGFSQGLKHTESWEQELHSLLAS
LHTLLGALYEGAETAPVQNEGPGVEMLLSSEDGDAHVLLQLRQRFSGLARCLGLMLSSEFGAPVSVPVQEILDFICRTLS
VSSKNISLHGDGPLRLLLLPSIHLEALDLLSALILACGSRLLRFGILIGRLLPQVLNSWSIGRDSLSPGQERPYSTVRTK
VYAILELWVQVCGASAGMLQGGASGEALLTHLLSDISPPADALKLRSPRGSPDGSLQTGKPSAPKKLKLDVGEAMAPPSH
RKGDSNANSDVCAAALRGLSRTILMCGPLIKEETHRRLHDLVLPLVMGVQQGEVLGSSPYTSSRCRRELYCLLLALLLAP
SPRCPPPLACALQAFSLGQREDSLEVSSFCSEALVTCAALTHPRVPPLQPMGPTCPTPAPVPPPEAPSPFRAPPFHPPGP
MPSVGSMPSAGPMPSAGPMPSAGPVPSARPGPPTTANHLGLSVPGLVSVPPRLLPGPENHRAGSNEDPILAPSGTPPPTI
PPDETFGGRVPRPAFVHYDKEEASDVEISLESDSDDSVVIVPEGLPPLPPPPPSGATPPPIAPTGPPTASPPVPAKEEPE
ELPAAPGPLPPPPPPPPPVPGPVTLPPPQLVPEGTPGGGGPPALEEDLTVININSSDEEEEEEEEEEEEEEEEEEEEEDF
EEEEEDEEEYFEEEEEEEEEEFEEEFEEEEGELEEEEEEEDEEEEELEEVEDLEFGTAGGEVEEGAPPPPTLPPALPPPE
SPPKVQPEPEPEPGLLLEVEEPGTEEERGADTAPTLAPEALPSQGEVEREGESPAAGPPPQELVEEEPSAPPTLLEEETE
DGSD
> Homo sapiens [A8K548]
MAAAVLSGPSAGSAAGVPGGTGGLSAVSSGPRLRLLLLESVSGLLQPRTGSAVAPVHPPNRSAPHLPGLMCLLRLHGSVG
GAQNLSALGALVSLSNARLSSIKTRFEGLCLLSLLVGESPTELFQQHCVSWLRSIQQVLQTQDPPATMELAVAVLRDLLR
YAAQLPALFRDISMNHLPGLLTSLLGLRPECEQSALEGMKACMTYFPRACGSLKGKLASFFLSRVDALSPQLQQLACECY
SRLPSLGAGFSQGLKHTESWEQELHSLLASLHTLLGALYEGAETAPVQNEGPGVEMLLSSEDGDAHVLLQLRQRFSGLAR
CLGLMLSSEFGAPVSVPVQEILDFICRTLSVSSKNISLHGDGPLRLLLLPSIHLEALDLLSALILACGSRLLRFGILIGR
LLPQVLNSWSIGRDSLSPGQERPYSTVRTKVYAILELWVQVCGASAGMLQGGASGEALLTHLLSDISPPADALKLRSPRG
SPDGSLQTGKPSAPKKLKLDVGEAMAPPSHRKGDSNANSDVCAAALRGLSRTILMCGPLIKEETHRRLHDLVLPLVMGVQ
QGEVLGSSPYTSSRCRRELYCLLLALLLAPSPRCPPPLACALQAFSLGQREDSLEVSSFCSEALVTCAALTHPRVPPLQP
MGPTCPTPAPVPPPEAPSPFRAPPFHPPGPMPSVGSMPSAGPMPSAGPMPSAGPVPSARPGPPTTANHLGLSVPGLVSVP
PRLLPGPENHRAGSNEDPILAPSGTPPPTIPPDETFGGRVPRPAFVHYDKEEASDVEISLESDSDDSVVIVPEGLPPLPP
PPPSGATPPPIAPTGPPTASPPVPAKEEPEELPAAPGPLPPPPPPPPPVPGPVTLPPPQLVPEGTPGGGGPPALEEDLTV
ININSSDEEEEEEEEEEEEEEEEEEEEEDFEEEEEDEEEYFEEEEEEEEEEFEEEFEEEEGELEEEEEEEDEEEEELEEV
EGLEFGTAGGEVEEGAPPPPTLPPALPPPESPPKVQPEPEPEPGLLLEVEEPGTEEERGADTAPTLAPEALPSQGEVERE
GESPAAGPPPQELVEEEPSAPPTLLEEETEDGSDKVQPPPETPAEEEMETETEAEALQEKEQDDTAAMLADFIDCPPDDE
KPPPPTEPDSKVQPPPETPAEEEMETETEAEALQEKEQDDTAAMLADFIDCPPDDEKPPPPTEPDS SLDESDDDSIPSL
KADDYLSSDSDIES
> Pan troglodytes [A0A2J8KQW1]
MAAAVLSGPSAGSAAGVPGGTGGLSAVSSGPRLRLLLLESVSGLLQPRTGSAVAPVHPPNRSAPHLPGLMCLLRLHGSVG
GAQNLSALGALVSLSNARLSSIKTRFEGLCLLSLLVGESPTELFQQHCVSWLRSIQQVLQTQDPPATMELAVAVLRDLLR
YAAQLPALFRDISMNHLPGLLTSLLGLRPECEQSALEGMKACMTYFPRACGSLKGKLASFFLSRVDALSPQLQQLACECY
SRLPSLGAGFSQGLKHTESWEQELHSLLASLHTLLGALYEGAETAPVQNEGPGVEMLLSSEDGDAHVLLQLRQRFSGLAR
CLGLMLSSEFGAPVSVPVQEILDFICRTLSVSSKNISLHGDGPLRLLLLPSIHLEALDLLSALILACGSRLLRFGILIGR
LLPQVLNSWSIGRDSLSPGQERPYSTVRTKVYAILELWVQVCGASAGMLQGGASGEALLTHLLSDISPPADALKLRSPRG
SPDGSLQTGKPSAPKKLKLDVGEAMAPPSHRKGDSNANSDVCAAALRGLSRTILMCGPLIKEETHRRLHDLVLPLVMGVQ
QGEVLGSSPYTSSRCRRELYCLLLALLLAPSPRCPPPLACALQAFSLGQREDSLEVSSFCSEALVTCAALTHPRVPPLQP
MGPTCPTPAPVPPPEAPSPFRAPPFHPPGPMPSVGSMPSAGPMPSAGPMPSAGPVSSARPGPPTTANHLGLSVPGLVSVP
PRLLPGPENHRAGSNEDPILAPSGTPPPTIPPDETFGGRVPRPAFVHYDKEEASDVEISLESDSDDSVVIVPEGLPPLPP
PPPSGATPPPIAPTGPPTASPPVPAKEEPEELPAAPGPLPPPPPPPPPVPGPVTLPPPQLVPEGTPGGGGPPALEEDLTV
ININSSDEEEEEEEEEEEEEEEEEEEEDFEEEEEDEEEYFEEEEEEEEEFEEDFEEEEGELEEEEEEEDDEEEELEDVED
LEFGTAGGEVEEGAPPPPTLPPALPPPESPPKVQPEPEPEPGLLLEVEEPGTEEERGADTAPTLAPEALPSQGEVEREGE
SPAAGPPPQELVEEEPSAPPTLLEEETEDGSDKVQPPPETPAEEEMETETEAEALQEKEQDDTAAMLADFIDCPPDDEKP
PPPTEPDS
> Pan troglodytes H2QBY0
MLLPSLSPRVQPAFTQCARPQPPPQAPSPVGGRRGVFASSRHATTGTRGKMAAAVLSGPSAGSAAGVPGGTGGLSAVSSG
PRLRLLLLESVSGLLQPRTGSAVAPVHPPNRSAPHLPGLMCLLRLHGSVGGAQNLSALGALVSLSNARLSSIKTRFEGLC
LLSLLVGESPTELFQQHCVSWLRSIQQVLQTQDPPATMELAVAVLRDLLRYAAQLPALFRDISMNHLPGLLTSLLGLRPE
CEQSALEGMKACMTYFPRACGSLKGKLASFFLSRVDALSPQLQQLACECYSRLPSLGAGFSQGLKHTESWEQELHSLLAS
LHTLLGALYEGAETAPVQNEGPGVEMLLSSEDGDAHVLLQLRQRFSGLARCLGLMLSSEFGAPVSVPVQEILDFICRTLS
VSSKNISLHGDGPLRLLLLPSIHLEALDLLSALILACGSRLLRFGILIGRLLPQVLNSWSIGRDSLSPGQERPYSTVRTK
VYAILELWVQVCGASAGMLQGGASGEALLTHLLSDISPPADALKLRSPRGSPDGSLQTGKPSAPKKLKLDVGEAMAPPSH
RKGDSNANSDVCAAALRGLSRTILMCGPLIKEETHRRLHDLVLPLVMGVQQGEVLGSSPYTSSRCRRELYCLLLALLLAP
SPRCPPPLACALQAFSLGQREDSLEVSSFCSEALVTCAALTHPRVPPLQPMGPTCPTPAPVPPPEAPSPFRAPPFHPPGP
MPSVGSMPSAGPMPSAGPMPSAGPVSSARPGPPTTANHLGLSVPGLVSVPPRLLPGPENHRAGSNEDPILAPSGTPPPTI
PPDETFGGRVPRPAFVHYDKEEASDVEISLESDSDDSVVIVPEGLPPLPPPPPSGATPPPIAPTGPPTASPPVPAKEEPE
ELPAAPGPLPPPPPPPPPVPGPVTLPPPQLVPEGTPGGGGPPALEEDLTVININSSDEEEEEEEEEEEEEEEEEEEEDF
EEEEEDEEEYFEEEEEEEEEFEEDFEEEEGELEEEEEEEDDEEEELEDVEDLEFGTAGGEVEEGAPPPPTLPPALPPES
PPKVQPEPEPEPGLLLEVEEPGTEEERGADTAPTLAPEALPSQGEVEREGESPAAGPPPQELVEEEPSAPPTLLEEETED
GSDKVQPPPETPAEEEMETETEAEALQEKEQDDTAAMLADFIDCPPDDEKPPPPTEPDS
> Pan troglodytes [A0A2J8KQT6]
MLLPSLSPRVQPAFTQCARPQPPPQAPSPVGGRRGVFASSRHATTGTRGKMAAAVLSGPSAGSAAGVPGGTGGLSAVSSG
PRLRLLLLESVSGLLQPRTGSAVAPVHPPNRSAPHLPGLMCLLRLHGSVGGAQNLSALGALVSLSNARLSSIKTRFEGLC
LLSLLVGESPTELFQQHCVSWLRSIQQVLQTQDPPATMELAVAVLRDLLRYAAQLPALFRDISMNHLPGLLTSLLGLRPE
CEQSALEGMKACMTYFPRACGSLKGKLASFFLSRVDALSPQLQQLACECYSRLPSLGAGFSQGLKHTESWEQELHSLLAS
LHTLLGALYEGAETAPVQNEGPGVEMLLSSEDGDAHVLLQLRQRFSGLARCLGLMLSSEFGAPVSVPVQEILDFICRTLS
VSSKNISLHGDGPLRLLLLPSIHLEALDLLSALILACGSRLLRFGILIGRLLPQVLNSWSIGRDSLSPGQERPYSTVRTK

VYAILELWVQVCGASAGMLQGGASGEALLTHLLSDISPPADALKLRSPRGSPDGSLQTGKPSAPKKLKLDVGEAMAPPSH
RKGDSNANSDVCAAALRGLSRTILMCGPLIKEETHRRLHDLVLPLVMGVQQGEVLGSSPYTSSRCRRELYCLLLALLLAP
SPRCPPPLACALQAFSLGQREDSLEVSSFCSEALVTCAALTHPRVPPLQPMGPTCPTPAPVPPPEAPSPFRAPPFHPPGP
MPSVGSMPSAGPMPSAGPMPSAGPVSSARPGPPTTANHLGLSVPGLVSVPPRLLPGPENHRAGSNEDPILAPSGTPPPTI
PPDETFGGRVPRPAFVHYDKEEASDVEISLESDSDDSVVIVPEGLPPLPPPPPSGATPPPIAPTGPPTASPPVPAKEEPE
ELPAAPGPLPPPPPPPPPVPGPVTLPPPQLVPEGTPGGGGPPALEEDLTVININSSDEEEEEEEEEEEEEEEEEEEEDFE
EEEEDEEEYFEEEEEEEEEFEEDFEEEEGELEEEEEEEDDEEEELEDVEDLEFGTAGGEVEEGAPPPPTLPPALPPPESP
PKVQPEPEPEPGLLLEVEEPGTEEERGADTAPTLAPEALPSQGEVEREGESPAAGPPPQELVEEEPSAPPTLLEEETEDG
SDKVQPPPETPAEEEMETETEAEALQEKEQDDTAAMLADFIDCPPDDEKPPPPTEPDS
> Pan troglodytes [A0A2R9BTR3]
MLLPSLSPRVQPAFTQCARPQPPPQAPSPVGGRRGVFASSRHATTGTRGKMAAAVLSGPSAGSAAGVPGGTGGLSAVSSG
PRLRLLLLESVSGLLQPRTGSAVAPVHPPNRSAPHLPALMCLLRLHGSVGGAQNLSALGALVSLSNARLSSIKTRFEGLC
LLSLLVGESPTELFQQHCVSWLRSIQQVLQTQDPPATMELAVAVLRDLLRYAAQLPALFRDISMNHLPGLLTSLLGLRPE
CEQSALEGMKACMTYFPRACGSLKGKLASFFLSRVDALSPQLQQLACECYSRLPSLGAGFSQGLKHTESWEQELHSLLAS
LHTLLGALYEGAETAPVQNEGPGVEMLLSSEDGDAHVLLQLRQRFSGLARCLGLMLSSEFGAPVSVPVQEILDFICRTLS
VSSKNISLHGDGPLRLLLLPSIHLEALDLLSALILACGSRLLRFGILIGRLLPQVLNSWSIGRDSLSPGQERPYSTVRTK
VYAILELWVQVCGASAGMLQGGASGEALLTHLLSDISPPADALKLRSPRGSPDGSLQTGKPSAPKKLKLDVGEAMAPPSH
RKGDSNANSDVCAAALRGLSRTILMCGPLIKEETHRRLHDLVLPLVMGVQQGEVLGSSPYTSSRCRRELYCLLLALLLAP
SPRCPPPLACALQAFSLGQREDSLEVSSFCSEALVTCAALTHPRVPPLQPMGPTCPTPAPVPPPEAPSPFRAPPFHPPGP
MPSVGSMPSAGPMPSAGPMPSAGPVSSARPGPPTTANHLGLSVPGLVSVPPRLLPGPENHRAGSNEDPILAPSGTPPPTI
PPDETFGGRVPRPAFVHYDKEEASDVEISLESDSDDSVVIVPEGLPPLPPPPPSGATPPPIAPTGPPTASPPVPAKEEPE
ELPAAPGPLPPPPPPPPPVPGPVTLPPPQLVPEGTPGGGGPPALEEDLTVININSSDEEEEEEEEEEEEEEEEEEEEEDF
EEEEEDEEEYFEEEEEEEEFEEDFEEEEGELEEEEEEEDDEEEELEDVEDLEFGTAGGEVEEGAPPPPTLPPALPPPESP
PKVQPEPEPEPGLLLEVEEPGTEEERGADTAPTLAPEALPSQGEVEREGESPAAGPPPQELVEEEPSAPPTLLEEETEDG
SDKVQPPPETPAEEEMETETEAEALQEKEQDDTAAMLADFIDCPPDDEKPPPPTEPDS
> Pan troglodytes [A0A2J8SPL0]
MAAAVLSGPSAGSAAGVPGGTGGLSAVSSGPRLRLLLLESVSGLLQPRTGSAVAPVHPPNRSAPHLPGLMCLLRLHGSVG
GAQNLSALGALVSLSNARLSSIKTRFEGLCLLSLLVGESPTELFQQHCVSWLRSIQQVLQTQDPPATMELAVAVLRDLLR
YAAQLPALFRDISMNHLPGLLTSLLGLRPECEQSALEGMKACMTYFPRACGSLKGKLASFFLSRVDALSPQLQQLACECY
SRLPSLGAGFSQGLKHTESWEQELHSLLASLHTLLGALYEGAETAPVQNEGPGVEMLLSSEDGDAHVLLQLRQRFSGLAR
CLGLMLSSEFGAPVSVPVQEILDFICRTLSVSSKNISLHGDGPLRLLLLPSIHLEALDLLSALILACGSRLLRFGILISR
LLPQVLNSWSIGRDSLSPGQERPYSTVRTKVYAILELWVQVCGASAGMLQGGASGEALLTHLLSDISPPADALKLRSPRG
SPDGSLQTGKPSAPKKLKLDVGEAMAPPSHRKGDSNANSDVCAAALRGLSRTILMCGPLIKEETHRRLHDLVLPLVMGVQ
QGEVLGSSPYTSSRCRRELYCLLLALLLAPSPRCPPPLACALQAFSLGQREDSLEVSSFCSEALVTCAALTHPRVPPLQP
MGPTGPTPAPAPPPEAPSPFRAPPFHPPGPMPSAGPMPSAGPMPSAGPVPSARPGPPTTANHLGLSVPGLVSVPPRLLPG
PENHRAGSNEDPILAPSGTPPPTIPPDETFGGRVPRPAFVHYDKEEASDVEISLESDSDDSVVIVPEGLPPLPPPPPSGA
TPPPIAPTGPPTASPPVPAKEEPEELPAAPGPLPPPPPPPPPVPGPVTLPPPQLVPEGTPGGGGPPALEEDLTVININSS
DEEEEEEEEEEEEEEEEEEEEEEDFEEEEEDEEEYFEEEEEEEFEEEFEEEEGELEEEEEEEDEEEEEEELEEVEDLEFG
TAGGEVEEGGPPPPTLPPTLPPPESPPKVQSEPEPEPGLLLEVEEPGTEEEHGADTAPTLAPEVLPSQGEVEREGESPAA
GPPPQELVEEEPSAPPTLLEEETEDGSDKVQPPPETPAEEEMETETEAEAHQEKEQDDTAAMLADFIDCPPDDEKPLPPT
EPDS
> Myotis myotis [A0A7J7T7B6]
MAAAVLSGPSAGSAAGVPGGAGGLSAVGSGPRLRMLLLESVSGLLQPRAGSTIPPVHPPV
RSAPHLPGLMCLLRLHGTVGGAQNLSAVGALVGLSNARLSSIKTRFEGLCLLSLLVGESS
TEMFQQHCVSWLRSIQQVLQSQDPPATMDLAVVVLRDLLRYAAQLPTVFRDISMNHLPGL
LTSLLGLRPECELSALEGMKACMTYFPRACGSLKGKLASFFLSRVDSLSPQLQQLACECY
SRLPSLGAGFSQGLKHTESWEQELHSLLASLHSLLGALYEGAEPAPVQYEGPGVEMLFSL
EDADAHNLFRLRQRFSGLACCLGLMLSSEFVAPVSIPVQEILDFICRTLSISAKNISLLG
DGPLRLLLLPSIHLEALDLLSALILACGGRLLRFGALISRLLPQVLNAWSIGRDTVSLGQ
EKPYSAMRTKVYAVLELWVQVCGASAGVLQAGASGEALLTHLLSDISPPAEALKLRSPRG
SPDGSLQTGKPSAPKKLKLDMGEAATPPSHRKGDSNANSDVCAAALRGLSRTILMCGPLI
KEETHRRLHDLVLPLVMGVQQGEVLGSSPYTSSRCRRELYRLLLALLLAPSPRCPPPLAC
ALQAFSLGQREDNLEVPPPPETSAAEEMETETAPPQEKEQDDTAAMLADFIDCPPDDEKP
PPVTEPDS
> Nyctereutes procyonoides [A0A811ZLL4]
MAAAVLSGSSAGSAAGVPGGAGGLSAVGSGPRLRLLLLESVSGLLQPRAGSAAAPLHPPV
RSAPHLPGLMCLLRLHGTVGGAQNLSAVGALVGLSNARLGSIKTRFEGLCLLSLLVGESP
TEMFQQHCVSWLRSIQQVLQSQDPPPTMELAVAVLRDLLRYAAQLPTLFRDISMNHLPGL
LTSLLGLRPECELSALEGMKACMTYFPRACGSLKGKLASFFLSRVDALSPQLQQLACECY
SRLPSLGAGFSQGLKHTESWEQELHSLLASLHSLLGALYEGAETAPVQYEGPGVEVLLTP
SEDGDTHVLLRLRQRFSGLARCLGLMLSSEFGAPVSVPVQEILDVICRTLSISAKNISLL
GDGPLRLLLLPSIHLDALDLLSALILACRGRLLRFGALISRLLPQVLNAWNLGRDTLAPG
QERPYSTVRTKVYAVLELWVQVCGASAGMLQGGSSGEALLSHLLSDISPPADTLKLRSPR
GSPDGGLQTGKPSAPKKLKLDVGEAMAPPSHRKGDSNANSDVCTAALRGLSRTILMCGPL
IKEETHRRLHDLVLPLVMGVQQGEVLGSSPYTSSRCRQELYRLLLALLLAPSPRCPPPLA
CALQAFSLGQREDSLEVSSFCSEALVTCAALTHPRVPSLRSMGPACPTPAPAPPPEAPSS
FRAPPFHPPGPMPSVGPMPSVGPMPSVGPMPPAGPMPPAGPMAPTRPGPPATANHLGLSV
PGLVSVPPRLIPGPENHRAGSNEDPVLAPSGTPPPAIPPDETFGGRVPRPAFVHYDKEEA
SDVEISLESDSDDSVVIVPEGLPPLPPPLPSGTTPPPVAPAGPPAASPPVPAKEEPEELP
VAPGPLPPPPPPPPVPGPVALPPPQLIPEATPGGGGPPALEEDLTVININSSDEEEEEEE
EEEEEEEEEEEEEDFEEEEEEEEYFEEEEEEEEEFEEEFEEEEGELEEEDEEEEEEEEEE
LDELEEVAFGPAAGAAEEGGPPPPSPPPALPPAQSPKMQPEPQGETGLLLEVEEQAVEEE
PGAEAAPTLAPEVLPPQGEGPREVGSPPAVPPPQELIEEEPPAPPTLLEEGTESGGDKVL
VPQETPAAEDVEAEVEAETAALQEKEQDDTAAMLADFIDCPPDDDKPPPATEPDS
> Pongo abelii [A0A2J8SPM6]
MLLPSLSPRVQPAFIQCAGPQPPPHAPSPVGGRRGVFASSRHATTEAQGKMAAAVLSGPSAGSAAGVPGGTGGLSAVSSG
PRLRLLLLESVSGLLQPRTGSAVAPVHPPNRSAPHLPGLMCLLRLHGSVGGAQNLSALGALVSLSNARLSSIKTRFEGLC

LLSLLVGESPTELFQQHCVSWLRSIQQVLQTQDPPATMELAVAVLRDLLRYAAQLPALFRDISMNHLPGLLTSLLGLRPE
CEQSALEGMKACMTYFPRACGSLKGKLASFFLSRVDALSPQLQQLACECYSRLPSLGAGFSQGLKHTESWEQELHSLLAS
LHTLLGALYEGAETAPVQNEGPGVEMLLSSEDGDAHVLLQLRQRFSGLARCLGLMLSSEFGAPVSVPVQEILDFICRTLS
VSSKNISLHGDGPLRLLLLPSIHLEALDLLSALILACGSRLLRFGILISRLLPQVLNSWSIGRDSLSPGQERPYSTVRTK
VYAILELWVQVCGASAGMLQGGASGEALLTHLLSDISPPADALKLRSPRGSPDGSLQTGKPSAPKKLKLDVGEAMAPPSH
RKGDSNANSDVCAAALRGLSRTILMCGPLIKEETHRRLHDLVLPLVMGVQQGEVLGSSPYTSSRCRRELYCLLLALLLAP
SPRCPPPLACALQAFSLGQREDSLEVSSFCSEALVTCAALTHPRVPPLQPMGPTGPTPAPAPPPEAPSPFRAPPFHPPGP
MPSAGPMPSAGPMPSAGPVPSARPGPPTTANHLGLSVPGLVSVPPRLLPGPENHRAGSNEDPILAPSGTPPPTIPPDETF
GGRVPRPAFVHYDKEEASDVEISLESDSDDSVVIVPEGLPPLPPPPPSGATPPPIAPTGPPTASPPVPAKEEPEELPAAP
GPLPPPPPPPPPVPGPVTLPPPQLVPEGTPGGGGPPALEEDLTVININSSDEEEEEEEEEEEEEEEEEEEEEDFEEEEED
EEEYFEEEEEEEEEFEEEFEEEEGELEEEEEEEDEEEEEELEEVEDLEFGTAGGEVEEGGPPPPTLPPTLPPPESPPKVQ
SEPEPEPGLLLEVEEPGTEEEHGADTAPTLAPEVLPSQGEVEREGESPAAGPPPQELVEEEPSAPPTLLEEETEDGSDKV
QPPPETPAEEEMETETEAEAHQEKEQDDTAAMLADFIDCPPDDEKPLPPTEPDS
> Mandrillus leucophaeus [A0A2K5ZZ41]
SDPPALAPQNPGITEPPPHAHSPVGGRRGVFASSRHATTGARGKMAAAVLSGPSAGSAAGVPGGTGGLSAVNSGPRLRLL
LLESVSGLLQPRTGSAVAPVHPPNRSAPHLPGLMCLLRLHGSVGGAQNLSALGALVSLSNARLSSIKTRFEGLCLLSLLV
GESPTELFQQHCVSWLRSIQQVLQTQDPPATMELAVAVLRDLLRYAAQLPALFRDISMNHLPGLLTSLLGLRPECEQSAL
EGMKACMTYFPRACGSLKGKLASFFLSRVDALSPQLQQLACECYSRLPSLGAGFSQGLKHTESWEQELHSLLASLHTLLG
ALYEGAETAPVQNEGPGVEMLLSSEDGDAHVLLRLRQRFSGLARCLGLMLSSEFGAPVSVPVQEILDFICRTLSVSSKNI
SLHGDGPLRLLLLPSIHLEALDLLSALILACGSRLLRFGILISRLLPQVLNSWSIGRDSLSPGQERPYSTVRTKVYAVLE
LWVQVCGASAGMLQGGASGEALLTHLLSDISPPADALKLRSPRGSPDGSLQTGKPSAPKKLKLDVGEAMAPPSHRKGDSN
ANSDVCAAALKGLSRTILMCGPLIKEETHRRLHDLVLPLVMGVQQGEVLGSSPYTSSRCRRELYCLLLALLLAPSPRCPP
PLACALQAFSLGQREDSLEVSSFCSEALVTCAALTHPRVPPLQPMGPTCPTPAPVPPPEAPSPFRAPPFHPPGPMPSVGP
MPSAGPMPSAGPMPSAGPVPSARPGPPTTANHLGLSVSGLVSVPPRLLPGPENHRAGSNEDPILAPSGTPPPAIPPDETF
GGRVPRPAFVHYDKEEASDVEISLESDSDDSVVIVPEGLPPLPPPPPSGATPPPIAPTGPPTASPPVPAKEEPEELPAAP
GPLPPPPPPPPPVPGPVTLPPPQLVPEGTPGGGGPPALEEDLTVININSSDEEEEEEEEEEEEEEEEEEEEEDFEEEEED
EEEYFEEEEEEEEEFEEEFEEEEGELEEEEEEEDEEEEEELEEVEELEFGTAGGEVEEGGPPPPTLPPALPPPESPPKVP
PEPEPEPGLLLEVEEPGAEEEHGADTAPTLAPEVLPSQGEVEREEGSPSAGPPPQELVEEEPSAPPTLLEEGTEDGGDRV
QPPPETSAEEEMETETEAEALQEKEQDDTAAMLADFIDCPPDDEKPPPPTEPDS
> Mandrillus leucophaeus [A0A2K5ZZ66]
QPPPHAHSPVGGRRGVFASSRHATTGARGKMAAAVLSGPSAGSAAGVPGGTGGLSAVNSGPRLRLLLLESVSGLLQPRTG
SAVAPVHPPNRSAPHLPGLMCLLRLHGSVGGAQNLSALGALVSLSNARLSSIKTRFEGLCLLSLLVGESPTELFQQHCVS
WLRSIQQVLQTQDPPATMELAVAVLRDLLRYAAQLPALFRDISMNHLPGLLTSLLGLRPECEQSALEGMKACMTYFPRAC
GSLKGKLASFFLSRVDALSPQLQQLACECYSRLPSLGAGFSQGLKHTESWEQELHSLLASLHTLLGALYEGAETAPVQNE
GPGVEMLLSSEDGDAHVLLRLRQRFSGLARCLGLMLSSEFGAPVSVPVQEILDFICRTLSVSSKNISLHGDGPLRLLLLP
SIHLEALDLLSALILACGSRLLRFGILISRLLPQVLNSWSIGRDSLSPGQERPYSTVRTKVYAVLELWVQVCGASAGMLQ
GGASGEALLTHLLSDISPPADALKLRSPRGSPDGSLQTGKPSAPKKLKLDVGEAMAPPSHRKGDSNANSDVCAAALKGLS
RTILMCGPLIKEETHRRLHDLVLPLVMGVQQGEVLGSSPYTSSRCRRELYCLLLALLLAPSPRCPPPLACALQAFSLGQR
EDSLEVSSFCSEALVTCAALTHPRVPPLQPMGPTCPTPAPVPPPEAPSPFRAPPFHPPGPMPSVGPMPSAGPMPSAGPMP
SAGPVPSARPGPPTTANHLGLSVSGLVSVPPRLLPGPENHRAGSNEDPILAPSGTPPPAIPPDETFGGRVPRPAFVHYDK
EEASDVEISLESDSDDSVVIVPEGLPPLPPPPPSGATPPPIAPTGPPTASPPVPAKEEPEELPAAPGPLPPPPPPPPPVP
GPVTLPPPQLVPEGTPGGGGPPALEEDLTVININSSDEEEEEEEEEEEEEEEEEEEEEDFEEEEEDEEEYFEEEEEEEEE
FEEEFEEEEGELEEEEEEEDEEEEEELEEVEELEFGTAGGEVEEGGPPPPTLPPALPPPESPPKVPPEPEPEPGLLLEVE
EPGAEEEHGADTAPTLAPEVLPSQGEVEREEGSPSAGPPPQELVEEEPSAPPTLLEEGTEDGGDRVQPPPETSAEEEMET
ETEAEALQEKEQDDTAAMLADFIDCPPDDEKPPPPTEPDS
> Macaca nemestrina [A0A2K6BMN5]
MLLPSLSPRVQPAFIQCAGPQPPPHAHSPVGGRRGVFTSSRHATTGARGKMAAAVLSGSSAGSAAGVPGGTGGLSAVNSG
PRLRLLLLESVSGLLQPRTGSAVAPVHPPNRSAPHLPGLMCLLRLHGSVGGAQNLSALGALVSLSNARLSSIKTRFEGLC
LLSLLVGESPTELFQQHCVSWLRSIQQVLQTQDPPATMELAVAVLRDLLRYAAQLPALFRDISMNHLPGLLTSLLGLRPE
CEQSALEGMKACMTYFPRACGSLKGKLASFFLSRVDALSPQLQQLACECYSRLPSLGAGFSQGLKHTESWEQELHSLLAS
LHTLLGALYEGAETAPVQNEGPGVEMLLSSEDGDAHVLLRLRQRFSGLARCLGLMLSSEFGAPVSVPVQEILDFICRTLS
VSSKNISLHGDGPLRLLLLPSIHLEALDLLSALILACGSRLLRFGILISRLLPQVLNSWSIGRDSLSPGQERPYSTVRTK
VYAVLELWVQVCGASAGMLQGGASGEALLTHLLSDISPPADALKLRSPRGSPDGSLQTGKPSAPKKLKLDVGEAMAPPSH
RKGDSNANSDVCAAALKGLSRTILMCGPLIKEETHRRLHDLVLPLVMGVQQGEVLGSSPYTSSRCRRELYCLLLALLLAP
SPRCPPPLACALQAFSLGQREDSLEVSSFCSEALVTCAALTHPRVPPLQPMGPTCPTPAPVPPPEAPSPFRAPPFHPPGP
MPSVGPMPSAGPMPSAGPMPSAGPVPSARPGPPTTANHLGLSVSGLVSVPPRLLPGPENHRAGSNEDPILAPSGTPPPTI
PPDETFGGRVPRPAFVHYDKEEASDVEISLESDSDDSVVIVPEGLPPLPPPPPSGATPPPIAPTGPPTASPPVPAKEEPE
ELPAAPGPLPPPPPPPPPVPGPVTLPPPQLVPEGTPGGGGPPALEEDLTVININSSDEEEEEEEEEEEEEEEEEEEEEDF
EEEEEDEEEYFEEEEEEEEEFEEEFEEEEGELEEEEEEEDEEEEEELEEVEELEFGTAGGEVEEGGPPPPTLPPALPPPE
SPPKVQPEPEPEPGLLLEVEEPGAEEEHGADTAPTLAPEVLPSQGEVEREEGSPSAGPPPQELVEEEPSAPPTLLEEGTE
DGGDRVQPPPETPAEEEMETETEAEALQEKEQDDTAAMLADFIDCPPDDEKPPPPTEPDS
> Macaca fascicularis [A0A2K5U1C9]
MLLPSLSPRVQPAFIQCAGPQPPPHAHSPVGGRRGVFTSSRHATTGARGKMAAAVLSGSSAGSAAGVPGGTGGLSAVNSG
PRLRLLLLESVSGLLQPRTGSAVAPVHPPNRSAPHLPGLMCLLRLHGSVGGAQNLSALGALVSLSNARLSSIKTRFEGLC
LLSLLVGESPTELFQQHCVSWLRSIQQVLQTQDPPATMELAVAVLRDLLRYAAQLPALFRDISMNHLPGLLTSLLGLRPE
CEQSALEGMKACMTYFPRACGSLKGKLASFFLSRVDALSPQLQQLACECYSRLPSLGAGFSQGLKHTESWEQELHSLLAS
LHTLLGALYEGAETAPVQNEGPGVEMLLSSEDGDAHVLLRLRQRFSGLARCLGLMLSSEFGAPVSVPVQEILDFICRTLS
VSSKNISLHGDGPLRLLLLPSIHLEALDLLSALILACGSRLLRFGILISRLLPQVLNSWSIGRDSLSPGQERPYSTVRTK
VYAVLELWVQVCGASAGMLQGGASGEALLTHLLSDISPPADALKLRSPRGSPDGSLQTGKPSAPKKLKLDVGEAMAPPSH
RKGDSNANSDVCAAALKGLSRTILMCGPLIKEETHRRLHDLVLPLVMGVQQGEVLGSSPYTSSRCRRELYCLLLALLLAP
SPRCPPPLACALQAFSLGQREDSLEVSSFCSEALVTCAALTHPRVPPLQPMGPTCPTPAPVPPPEAPSPFRAPPFHPPGP
MPSVGPMPSAGPMPSAGPMPSAGPVPSARPGPPTTANHLGLSVSGLVSVPPRLLPGPENHRAGSNEDPILAPSGTPPPTI
PPDETFGGRVPRPAFVHYDKEEASDVEISLESDSDDSVVIVPEGLPPLPPPPPSGATPPPIAPTGPPTASPPVPAKEEPE
ELPAAPGPLPPPPPPPPPVPGPVTLPPPQLVPEGTPGGGGPPALEEDLTVININSSDEEEEEEEEEEEEEEEEEEEEEDF
EEEEEDEEEYFEEEEEEEEEFEEEFEEEEGELEEEEEEEDEEEEEELEEVEELEFGTAGGEVEEGGPPPPTLPPALPPPE
SPPKVQPEPEPEPGLLLEVEEPGAEEEHGADTAPTLAPEVLPSQGEVEREEGSPSAGPPPQELVEEEPSAPPTLLEEGTE
DGGDRVQPPPETPAEEEMETETEAEALQEKEQDDTAAMLADFIDCPPDDEKPPPPTEPDS

> Colobus angolensis palliates [A0A2K5ICW2]
MLLPSLSPRVQPAFTQCAGPRPPPHAHSPVGGRRSVFASSRHATTGARGKMAAAVLSGPSAGSAAGVPGGTGGLSAVNSG
PRLRLLLLESVSGLLQPRTGSAVAPVHPPNRSAPHLPGLMCLLRLHGSVGGAQNLSALGALVSLSNARLSSIKTRFEGLC
LLSLLVGESPTELFQQHCVSWLRSIQQVLQTQDPPATMELAVAVLRDLLRYAAQLPALFRDISMNHLPGLLTSLLGLRPE
CEQSALEGMKACMTYFPRACGSLKGKLASFFLSRVDALSPQLQQLACECYSRLPSLGAGFSQGLKHTESWEQELHSLLAS
LHTLLGALYEGAETAPVQNEGPGVEMLLSSEDGDAHVLLRLLQRFSGLACCLGLMLSSEFGAPVSVPVQEILDFICRTLS
VSSKNISLHGDGPLRLLLLPSIHLEALDLLSALILACGSRLLRFGILISRLLPQVLNSWSIGRDSLSPGQERPYSTVRTK
VYAVLELWVQVCGASAGMLQGGASGEALLTHLLSDISPPADALKLRSPRGSPDGSLQTGKPSAPKKLKLDVGEAMAPPSH
RKGDSNANSDVCAAALRGLSRTILMCGPLIKEETHRRLHDLVLPLVMGVQQGEVLGSSPYTSSRCRRELYCLLLALLLAP
SPRCPPPLACALQAFSLGQREDSLEVSSFCSEALVTCAALTHPRVPPLQPMGPTCPTPAPVPPPEAPSPFRAPPFHPPGP
MPSVGPMPSAGPMPSAGPMPSAGPVPSARPGPPTTANHLGLSVSGLVSVPPRLLPGPENHRAGSNEDPILAPSGTPPPAI
PPDETFGGRVPRPAFVHYDKEEASDVEISLESDSDDSVVIVPEGLPPLPPPPPSGATPPPIAPTGPPTASPPVPAKEEPE
ELPAAPGPLPPPPPPPPPPVPGPVTLPPPQLVPEGTPGGGGPPALEEDLTVININSSDEEEEEEEEEEEEEEEEEEEEDF
EEEEEDEEEYFEEEEEEEEEFEEEFEEEEGELEEEEEEEDEEEEEELEEVEELEFGTAGGEVEEGGPPPPTLPPALPPPE
SPPKVQPEPEPEPGLLLEVEEPGAEEEHGADTAPTLAPEVLPSQGEVEREEGSPAAGPPPQELVEEEPSAPPTLLEEGTE
DGGDRVQPPPETPAEEEMETETEAEALQEKEQDDTAAMLADFIDCPPDDEKPPPPTEPDS
> Papio Anubis [A0A096P2G2]
MLLPSLSPRVQPAFIQCAGPQPPPHAHSPVGGRRGVFASSRHATTGARGKMAAAVLSGPSAGSAAGVPGGTGGLSAVNSG
PRLRLLLLESVSGLLQPRTGSAVAPVHPPNRSAPHLPGLMCLLRLHGSVGGAQNLSALGALVSLSNARLSSIKTRFEGLC
LLSLLVGESPTELFQQHCVSWLRSIQQVLQTQDPPATMELAVAVLRDLLRYAAQLPALFRDISMNHLPGLLTSLLGLRPE
CEQSALEGMKACMTYFPRACGSLKGKLASFFLSRVDALSPQLQQLACECYSRLPSLGAGFSQGLKHTESWEQELHSLLAS
LHTLLGALYEGAETAPVQNEGPGVEMLLSSEDGDAHILLRLRQRFSGLARCLGLMLSSEFGAPVSVPVQEILDFICRTLS
VSSKNISLHGDGPLRLLLLPSIHLEALDLLSALILACGSRLLRFGILISRLLPQVLNSWSIGRDSLSPGQERPYSTVRTK
VYAVLELWVQVCGASAGMLQGGASGEALLTHLLSDISPPADALKLRSPRGSPDGSLQTGKPSAPKKLKLDVGEAMAPPSH
RKGDSNANSDVCAAALKGLSRTILMCGPLIKEETHRRLHDLVLPLVMGVQQGEVLGSSPYTSSRCRRELYCLLLALLLAP
SPRCPPPLACALQAFSLGQREDSLEVSSFCSEALVTCAALTHPRVPPLQPMGPTCPTPAPVPPPEAPSPFRAPPFHPPGP
MPSVGPMPSAGPMPSAGPMPSAGPVPSARPGPPTTANHLGLSVSGLVSVPPRLLPGPENHRAGSNEDPILAPSGTPPPAI
PPDETFGGRVPRPAFVHYDKEEASDVEISLESDSDDSVVIVPEGLPPPPPPPPGATPPPIAPTGPPTASPPVPAKEEPEE
LPAAPGPLPPPPPPPPPPVPGPVTLPPPQLVPEGTPGGGGPPALEEDLTVININSSDEEEEEEEEEEEEEEEEEEEEDFE
EEEEDEEEYFEEEEEEEEEFEEEFEEEEGELEEEEEEEDEEEEEELEEVEELEFGTAGGEVEEGGPPPPTLPPALPPPES
PPKVPPEPEPEPGLLLEVEEPGAEEEHGADTAPTLAPEVLPSQGEVEREEGSPSAGPPPQELVEEEPSAPPTLLEEGTED
GGDRVQPPPETPAEEEMETETEAEALQEKEQDDTAAMLADFIDCPPDDEKPPPPTEPDS
> Cercocebus atys [A0A2K5LPK3]
MLLPSLSPRVQPAFIQCAGPQPPPHAHSPVGGRRGVFASSRHATTGAPGKMAAAVLSGPSAGSAAGVPGGTGGLSAVNSG
PRLRLLLLESVSGLLQPRTGSAVAPVHPPNRSAPHLPGLMCLLRLHGSVGGAQNLSALGALVSLSNARLSSIKTRFEGLC
LLSLLVGESPTELFQQHCVSWLRSIQQVLQTQDPPATMELAVAVLRDLLRYAAQLPALFRDISMNHLPGLLTSLLGLRPE
CEQSALEGMKACMTYFPRACGSLKGKLASFFLSRVDALSPQLQQLACECYSRLPSLGAGFSQGLKHTESWEQELHSLLAS
LHTLLGALYEGAETAPVQNEGPGVEMLLSSEDGDAHVLLRLRQRFSGLARCLGLMLSSEFGAPVSVPVQEILDFICRTLS
VSSKNISLHGDGPLRLLLLPSIHLEALDLLSALILACGSRLLRFGILISRLLPQVLNSWSIGRDSLSPGQERPYSTVRTK
VYAVLELWVQVCGASAGMLQGGASGEALLTHLLSDISPPADALKLRSPRGSPDGSLQTGKPSAPKKLKLDVGEAMAPPSH
RKGDSNANSDVCAAALKGLSRTILMCGPLIKEETHRRLHDLVLPLVMGVQQGEVLGSSPYTSSRCRRELYCLLLALLLAP
SPRCPPPLACALQAFSLGQREDSLEVSSFCSEALVTCAALTHPRVPPLQPMGPTCPTPAPVPPPEAPSPFRAPPFHPPGP
MPSVGPMPSAGPMPSAGPMPSAGPVPSARPGPPTTANHLGLSVSGLVSVPPRLLPGPENHRAGSNEDPILAPSGSPPPAI
PPDETFGGRVPRPAFVHYDKEEASDVEISLESDSDDSVVIVPEGLPPLPPPPPSGATPPPIAPTGPPTASPPVPAKEEPE
ELPAAPGPLPPPPPPPPPPVPGPVTLPPPQLVPEGTPGGGGPPALEEDLTVININSSDEEEEEEEEEEEEEEEEEEEEDF
EEEEEDEEEYFEEEEEEEEEFEEEFEEEEGELEEEEEEEDEEEEEELEEVEELEFGTAGGEVEEGGPPPPTLPPALPPPE
SPPKVPPEPEPEPGLLLEVEEPGAEEEHGADTAPTLAPEVLPSQGEVEREEGSPSAGPPPQELVEEEPSAPPTLLEEGTE
DGGDRVQPPPETPAEEEMETETEAEALQEKEQDDTAAMLADFIDCPPDDEKPPPPTEPDS
> Chlorocebus sabaeus [A0A0D9RKY8]
MKIVRTLAPFVRTTCTLNITGPPRTRSPAITPGTQHPGASACRLLSPRMLLPSLSPRVQPAFTQCAGPQPPPHAHSSVGR
RRGVFASSRHATTGARGKMAAAVLSGPSAGSAAGVPGGTGGLSAVNSGPRLRLLLLESVSGLLQPRTGSAVAPVHPPNRS
APHLPGLMCLLRLHGSVGGAQNLSALGALVSLSNARLSSIKTRFEGLCLLSLLVGESPTELFQQHCVSWLRSIQQVLQTQ
DPPATMELAVAVLRDLLRYAAQLPALFRDISMNHLPGLLTSLLGLRPECEQSALEGMKACMTYFPRACGSLKGKLASFFL
SRVDALSPQLQQLACECYSRLPSLGAGFSQGLKHTESWEQELHSLLASLHTLLGALYEGAETAPVQNEGPGVEMLLSSED
GDAHVLLRLRQRFSGLARCLGLMLSSEFGAPVSVPVQEILDFICRTLSVSSKNISLHGDGPLRLLLLPSIHLEALDLLSA
LILACGSRLLRFGILISRLLPQVLNSWSIGRDSLSPGQERPYSTVRTKVYAVLELWVQVCGASAGMLQGGASGEALLTHL
LSDISPPADALKLRSPRGSPDGSLQTGKPSAPKKLKLDVGEAMAPPSHRKGDSNANSDVCAAALRGLSRTILMCGPLIKE
ETHRRLHDLVLPLVMGVQQGEVLGSSPYTSSRCRRELYCLLLALLLAPSPRCPPPLACALQAFSLGQREDSLEVSSFCAE
ALVTCAALTHPRVPPLQPMGPTCPTPAPVPPPEAPSPFRAPPFHPPGPMPSAGPMPSAGPMPSAGPVPSARPGPPTTANH
LGLSVSGLVSVPPRLLPGPENHRAGSNEDPILAPSGTPPPAIPPDETFGGRVPRPAFVHYDKEEASDVEISLESDSDDSV
VIVPEGLPPLPPPPPSGATPPPIAPTGPPTASPPVPAKEEPEELPAAPGPLPPPPPPPPPVPGPVTLPPPQLVPEGTPGG
GGPPALEEDLTVININSSDEEEEEEEEEEEEEEEEEEEEEDFEEEEEDEEEYFEEEEEEEEEFEEEFEEEEGELEEEEEE
EDEEEEEELEEVEELEFGAAGGEVEEGGPPPPTLPPALPPPESPPKVQPEPEPEPGLLLEVEEPGAEEEHGADTAPTLAP
EVLPSQGEVEREEGSPSAGPPPQELVEEEPSAPPTLLEEGTEDGGDRVQPPPETPAEEEMETETEAETLQEKEQDDTAAM
LADFIDCPPDDEKPPPPTEPDS
> Cebus imitator [A0A2K5SHN5]
PPPSPLPLPTRRPSQCVCVIVPRHAITGARGKMAAAVLSGPTAGSAAGVPGGTGGLSAVSSGPRLRLLLLESVSGLLQPR
TGSAVAPVHPPNRSAPHLPGLMCLLRLHGSVGGAQNLSAVGALVNLSNARLSSIKTRFEGLCLLSLLVGESPTELFQQHC
VSWLRSIQQVLQSQDPPPTMELAVAVLRDLLRYAAQLPALFRDISMNHLPGLLTSLLGLRPECEQSALEGMKACMTYFPR
ACGSLKGKLASFFLSRVDALSPQLQQLACECYSRLPSLGAGFSQGLKHTESWEQELHSLLASLHTLLGALYEGAETAPVQ
NEGPGVEMLLSSEDGDAHVLLRLRQRFSGLARCLGLMLSSEFGAPVSVPVQEILDFICRTLSVSSKNISLHGDGPLRLLL
LPSIHLEALDLLSALILACGSRLLRFGILISRLLPQILNSWSIGRDSLSPGQERPYSTVRTKVYAVLELWVQVCGASAGV
LQGGASGEALLTHLLSDISPPADALKLRSPRGSPDGGLQTGKPSAPKKLKLDVGEAMAPPSHRKGDSNANSDVCAAALRG
LSRTILMCGPLIKEETHRRLHDLVLPLVMGVQQGEVLGSSPYTSSRCRRELYCLLLALLLAPSPRCPPPLACALQAFSLG
QREDSLEVSSFCSEALVTCAALTHPRVPPLQSMGPTCPTPAPVPPPETPSPFRAPPFHPPGPMPSVGPMPSAGPMPSAGP
MPSAGPVSSARPGPPTTANHLGLSVPGLVSVPPRLLPGPENHRASSNEDPILAPSGTPPPAIPPDETFGGRVPRPAFVHY
DKEEASDVEISLESDSDDSVVIVPEGLPPLPPPPPSGATPPPVAPSGPPTASPPVPAKEEPEELPAAPGPLPPPPPPPPP

VPGPVTLPPPQLVPEGTPGGGGPPALEEDLTVININSSDEEEEEEEEEEEEEEEEEEEEEEDFEEEEEDEEEYFEEEEEEE
EEFEEEFEEEEGELEEEEEEEDEDEEEEELEEVEELEFGTAGGEVEEGGPPPPTLPPALPPPESPPKVQPEPEPEPGLLL
EVEEPGAEEEHEADTAPTLAPEVLPSQGEVERDGGSPAAGLPPQELVEEEPSAPPTLLEEGTEDGGDKVQLPPETSAEEE
METETEAEALQEKEQDDTAAMLADFIDCPPDDEKPPPATEPDS
> Aotus nancymaae [A0A2K5EHP0]
MAAAVLSGPTAGSAAGVPSGTGGLSAVSSGPRLRLLLLESVSGLLQPRTGSAVAPVHPPNRSAPHLPGLMCLLRLHGSVG
GAQNLSAVGALVSLSNARLSSIKTRFEGLCLLSLLVGESPTELFQQHCVSWLRSIQQVLQSQDPPPTMELAVAVLRDLLR
YAAQLPALFRDISMNHLPGLLTSLLGLRPECEQSALEGMKACMTYFPRACGSLKGKLASFFLSRVDALSPQLQQLACECY
SRLPSLGAGFSQGLKHTESWEQELHSLLASLHTLLGALYEGAETAPVQNEGPGVEMLLSSEDGDAHVLLRLRQRFSGLAR
CLGLMLSSEFGAPVSVPVQEILDFICRTLSVNSKNISLHGDGPLRLLLLPSIHLEALDLLSALILACGSRLLRFGILISR
LLPQVLNSWSIGRDSLSPGQERPYSTVRTKVYAVLELWVQVCGASAGVLQGGASGEALLTHLLSDISPPADALKLRSPRG
SPDGGLQTGKPSAPKKLKLDVGEAMAPPSHRKGDSNANSDVCAAALRGLSRTILMCGPLIKEETHRRLHDLVLPLVMGVQ
QGEVLGSSPYTSSRCRRELYCLLLALLLAPSPRCPPPLACALQAFSLGQREDSLEVSSFCSEALVTCAALTHPRVPPLQS
MGPTCPTPAPVPPPEAPSPFRAPPFHPPGPMPSVGPMPSAGPMPSAGPMPSAGPMPSAGPVPSARPGPPTTANHLGLSVP
GLVSVPPRLLPGPENHRASSNEDPVLAPSGTPPPTIPPDETFGGRVPRPAFVHYDKEEASDVEISLESDSDDSVVIVPEG
LPTLPPPPPSGATPPPVAPSGPPTASPPVPAKEDPEELPAAPGPLPPPPPPPPPVPGPVTLPPPQLVPEGTPGGGGPPAL
EEDLTVININSSDEEEEEEEEEEEEEEEEEEEEEEDFEEEEEDEEEYFEEEEEEEEEFEEEFEEEEGELEEEEEEEDEDEE
EEELEEVEELEFGTAGGDVEEGGPPPPTLPPALPPSESPPKVQPEPEPEPGLLLEVEEPGAEEEHEADTAPTLAPEVLPS
QGEVERDGGSPAAGLPPQELVEEEPSAPPTLLEEGTEDGGDKVQLPPETSAEEEMETETEAEALQEKEQDDTAAMLADFI
DCPPDDEKPPPATEPDS
> Pan troglodytes [A0A2I3T6G1]
MLLPSLSPRVQPAFTQCARPQPPPQAPSPVGGRRGVFASSRHATTGTRGKMAAAVLSGPSAGSAAGVPGGTGGLSAVSSG
PRLRLLLLESVSGLLQPRTGSAVAPVHPPNRSAPHLPGLMCLLRLHGSVGGAQNLSALGALVSLSNARLSSIKTRFEGLC
LLSLLVGESPTELFQQHCVSWLRSIQQVLQTQDPPATMELAVAVLRDLLRYAAQLPALFRDISMNHLPGLLTSLLGLRPE
CEQSALEGMKACMTYFPRACGSLKGKLASFFLSRVDALSPQLQQLACECYSRLPSLGAGFSQGLKHTESWEQELHSLLAS
LHTLLGALYEGAETAPVQNEGPGVEMLLSSEDGDAHVLLQLRQRFSGLARCLGLMLSSEFGAPVSVPVQEILDFICRTLS
VSSKNISLHGDGPLRLLLLPSIHLEALDLLSALILACGSRLLRFGILIGRLLPQVLNSWSIGRDSLSPGQERPYSTVRTK
VYAILELWVQVCGASAGMLQGGASGEALLTHLLSDISPPADALKLRSPRGSPDGSLQTGKPSAPKKLKLDVGEAMAPPSH
RKGDSNANSDVCAAALRGLSRTILMCGPLIKEETHRGPLLCTHWGRLHDLVLPLVMGVQQGEVLGSSPYTSSRCRRELYC
LLLALLLAPSPRCPPPLACALQAFSLGQREDSLEVSSFCSEALVTCAALTHPRVPPLQPMGPTCPTPAPVPPPEAPSPFR
APPFHPPGPMPSVGSMPSAGPMPSAGPMPSAGPVSSARPGPPTTANHLGLSVPGLVSVPPRLLPGPENHRAGSNEDPILA
PSGTPPPTIPPDETFGGRVPRPAFVHYDKEEASDVEISLESDSDDSVVIVPEGLPPLPPPPPSGATPPPIAPTGPPTASP
PVPAKEEPEELPAAPGPLPPPPPPPPPVPGPVTLPPPQLVPEGTPGGGGPPALEEDLTVININSSDEEEEEEEEEEEEEE
EEEEEEEDFEEEEEDEEEYFEEEEEEEEEFEEDFEEEEGELEEEEEEEEDDEEEELEDVEDLEFGTAGGEVEEGAPPPPTL
PPALPPPESPPKVQPEPEPEPGLLLEVEEPGTEEERGADTAPTLAPEALPSQGEVEREGESPAAGPPPQELVEEEPSAPP
TLLEEETEDGSDKVQPPPETPAEEEMETETEAEALQEKVRGQAGKVPWARRWGWREKG
> Callithrix jacchus [F7I3S5]
MAAAVLSGPTAGSAAGVPGGTGGLSAVSSGPRLRLLLLESVSGLLQPRTGSAVAPVHPPNRSAPHLPGLMCLLRLHGSVG
GAQNLSAVGALVSLSNARLSSIKTRFEGLCLLSLLVGESPTELFQQHCVSWLRSIQQVLQSQDPPPTMELAVAVLRDLLR
YAAQLPALFRDISMNHLPGLLTSLLGLRPECEQSALEGMKACMTYFPRACGSLKGKLASFFLSRVDALSPQLQQLACECY
SKLPSLGAGFSQGLKHTESWEQELHSLLASLHTLLGALYEGAETAPVQHEGPGVEMLLSSEDGDAHVLLRLWQRFSGLAH
CLGLMLSSEFGAPVSVPVQEILDFICRTLSVSSKNISLHGDGPLRLLLLPSIHLEALDLLSALIVACGSRLLRFGIPISR
LLPQVLNSWSIGRDSLSPGQERPYSTVRTKVYAVLELWVQVCGASAGVLQGGASGEALLTHLLSDISPPADALKLRSPRG
SPDGGLQTGKPSAPKKLKLDVGEAMTPPSHRKGDSNANSDVCAAALRGLSRTILMCGPLIKEETHRRLHDLVLPLVMGVQ
QGEVLGSSPYTSSRCRRELYCLLLALLLSPSPRCPPPLACALQAFSLGQREDSLEVSSFCSEALVICAALTHPRVPPLQS
MGPTCPTPAPVPPPEAPSPFRAPSFHPPGPMPSVGPMPSAGPMPSAGPMPSAGPVPSAGPVPSARPGPPTTANHLGLSVP
GLVSVPPRLLPGPENHRASSNEDPILAPSGTPPPTIPPDETFGGRVPRPAFVHYDKEEASDVEISLESDSDDSVVIVPEG
LPPLPPPPPSGATPPPVAPSGPPTASPPLPAKEEPEELPAAPGPLPPPPPPPPPVPGPVTLPPPQLVPEGTPGGGGPPAL
EEDLTVININSSDEEEEEEEEEEEEEEEEEEEEEEDFEEEEEDEEEYFEEEEEEEEEFEEEFEEEEGELEEEEEEEDEDEE
EEELEEVEELEFGTAGGEVEEGGPPPPTLPPALPPPESPPKVQPEPEPEPGLLLEVEEPGAEEEHEADTAPTLAPEVLPS
QGEVERDEGSPEAGLPPQELVEEEPSAPPTLLEEGTEDGGDKVQLPPETSAEEEMETETEAEALQEKEQDDTAAMLADFI
DCPPDDEKPPPATEPDS
> Pan paniscus [A0A2R9BZC1]
MLLPSLSPRVQPAFTQCARPQPPPQAPSPVGGRRGVFASSRHATTGTRGKMAAAVLSGPSAGSAAGVPGGTGGLSAVSSG
PRLRLLLLESVSGLLQPRTGSAVAPVHPPNRSAPHLPALMCLLRLHGSVGGAQNLSALGALVSLSNARLSSIKTRFEGLC
LLSLLVGESPTELFQQHCVSWLRSIQQVLQTQDPPATMELAVAVLRDLLRYAAQLPALFRDISMNHLPGLLTSLLGLRPE
CEQSALEGMKACMTYFPRACGSLKGKLASFFLSRVDALSPQLQQLACECYSRLPSLGAGFSQGLKHTESWEQELHSLLAS
LHTLLGALYEGAETAPVQNEGPGVEMLLSSEDGDAHVLLQLRQRFSGLARCLGLMLSSEFGAPVSVPVQEILDFICRTLS
VSSKNISLHGDGPLRLLLLPSIHLEALDLLSALILACGSRLLRFGILIGRLLPQVLNSWSIGRDSLSPGQERPYSTVRTK
VYAILELWVQVCGASAGMLQGGASGEALLTHLLSDISPPADALKLRSPRGSPDGSLQTGKPSAPKKLKLDVGEAMAPPSH
RKGDSNANSDVCAAALRGLSRTILMCGPLIKEETHRGPLLCTHWGRLHDLVLPLVMGVQQGEVLGSSPYTSSRCRRELYC
LLLALLLAPSPRCPPPLACALQAFSLGQREDSLEVSSFCSEALVTCAALTHPRVPPLQPMGPTCPTPAPVPPPEAPSPFR
APPFHPPGPMPSVGSMPSAGPMPSAGPVSSARPGPPTTANHLGLSVPGLVSVPPRLLPGPENHRAGSNEDPILA
PSGTPPPTIPPDETFGGRVPRPAFVHYDKEEASDVEISLESDSDDSVVIVPEGLPPLPPPPPSGATPPPIAPTGPPTASP
PVPAKEEPEELPAAPGPLPPPPPPPPPVPGPVTLPPPQLVPEGTPGGGGPPALEEDLTVININSSDEEEEEEEEEEEEEE
EEEEEEEDFEEEEEDEEEYFEEEEEEEEEFEEDFEEEEGELEEEEEEEEDDEEEELEDVEDLEFGTAGGEVEEGAPPPPTLP
PALPPPESPPKVQPEPEPEPGLLLEVEEPGTEEERGADTAPTLAPEALPSQGEVEREGESPAAGPPPQELVEEEPSAPPT
LLEEETEDGSDKVQPPPETPAEEEMETETEAEALQEKVRGQAGKVPWARRWGWREKG
> Cercocebus atys [A0A2K5LPK4]
MLLPSLSPRVQPAFIQCAGPQPPPHAHSPVGGRRGVFASSRHATTGAPGKMAAAVLSGPSAGSAAGVPGGTGGLSAVNSG
PRLRLLLLESVSGLLQPRTGSAVAPVHPPNRSAPHLPGLMCLLRLHGSVGGAQNLSALGALVSLSNARLSSIKTRFEGLC
LLSLLVGESPTELFQQHCVSWLRSIQQVLQTQDPPATMELAVAVLRDLLRYAAQLPALFRDISMNHLPGLLTSLLGLRPE
CEQSALEGMKACMTYFPRACGSLKGKLASFFLSRVDALSPQLQQLACECYSRLPSLGAGFSQGLKHTESWEQELHSLLAS
LHTLLGALYEGAETAPVQNEGPGVEMLLSSEDGDAHVLLRLRQRFSGLARCLGLMLSSEFGAPVSVPVQEILDFICRTLS
VSSKNISLHGDGPLRLLLLPSIHLEALDLLSALILACGSRLLRFGILISRLLPQVLNSWSIGRDSLSPGQERPYSTVRTK
VYAVLELWVQVCGASAGMLQGGASGEALLTHLLSDISPPADALKLRSPRGSPDGSLQTGKPSAPKKLKLDVGEAMAPPSH
RKGDSNANSDVCAAALKGLSRTILMCGPLIKEETHRRLHDLVLPLVMGVQQGEVLGSSPYTSSRCRRELYCLLLALLLAP

SPRCPPPLACALQAFSLGQREDSLEVSSFCSEALVTCAALTHPRVPPLQPMGPTCPTPAPVPPPEAPSPFRAPPFHPPGP
MPSVGPMPSAGPMPSAGPMPSAGPVPSARPGPPTTANHLGLSVSGLVSVPPRLLPGPENHRAGSNEDPILAPSGSPPPAI
PPDETFGGRVPRPAFVHYDKEEASDVEISLESDSDDSVVIVPEGLPPLPPPPPSGATPPPIAPTGPPTASPPVPAKEEPE
ELPAAPGPLPPPPPPPPPPVPGPVTLPPPQLVPEGTPGGGGPPALEEDLTVININSSDEEEEEEEEEEEEEEEEEEEEEDF
EEEEEDEEEYFEEEEEEEEEEFEEEFEEEEGELEEEEEEEDEEEEEEELEEVEELEFGTAGGEVEEGGPPPPTLPPALPPPE
SPPKVPPEPEPEPEPGLLLEVEEPGAEEEHGADTAPTLAPEVLPSQGEVEREEGSPSAGPPPQELVEEEPSAPPTLLEEGTE
DGGDRVQPPPETPAEEEMETETEAEALQEKVRGLLISSWDYRRPPPPG
> Cercocebus atys [A0A2K5LPL2]
MLLPSLSPRVQPAFIQCAGPQPPPHAHSPVGGRRGVFASSRHATTGAPGKMAAAVLSGPSAGSAAGVPGGTGGLSAVNSG
PRLRLLLLESVSGLLQPRTGSAVAPVHPPNRSAPHLPGLMCLLRLHGSVGGAQNLSALGALVSLSNARLSSIKTRFEGLC
LLSLLVGESPTELFQQHCVSWLRSIQQVLQTQDPPATMELAVAVLRDLLRYAAQLPALFRDISMNHLPGLLTSLLGLRPE
CEQSALEGMKACMTYFPRACGSLKGKLASFFLSRVDALSPQLQQLACECYSRLPSLGAGFSQGLKHTESWEQELHSLLAS
LHTLLGALYEGAETAPVQNEGPGVEMLLSSEDGDAHVLLRLRQRFSGLARCLGLMLSSEFGAPVSVPVQEILDFICRTLS
VSSKNISLHGDGPLRLLLLPSIHLEALDLLSALILACGSRLLRFGILISRLLPQVLNSWSIGRDSLSPGQERPYSTVRTK
VYAVLELWVQVCGASAGMLQGGASGEALLTHLLSDISPPADALKLRSPRGSPDGSLQTGKPSAPKKLKLDVGEAMAPPSH
RKGDSNANSDVCAAALKGLSRTILMCGPLIKEETHRRLHDLVLPLVMGVQQGEVLGSSPYTSSRCRRELYCLLLALLLAP
SPRCPPPLACALQAFSLGQREDSLEVSSFCSEALVTCAALTHPRVPPLQPMGPTCPTPAPVPPPEAPSPFRAPPFHPPGP
MPSVGPMPSAGPMPSAGPMPSAGPVPSARPGPPTTANHLGLSVSGLVSVPPRLLPGPENHRAGSNEDPILAPSGSPPPAI
PPDETFGGRVPRPAFVHYDKEEASDVEISLESDSDDSVVIVPEGLPPLPPPPPSGATPPPIAPTGPPTASPPVPAKEEPE
ELPAAPGPLPPPPPPPPPPVPGPVTLPPPQLVPEGTPGGGGPPALEEDLTVININSSDEEEEEEEEEEEEEEEEEEEEEEDF
EEEEEDEEEYFEEEEEEEEEEFEEEFEEEEGELEEEEEEEDEEEEEEELEEVEELEFGTAGGEVEEGGPPPPTLPPALPPPE
SPPKVPPEPEPEPEPGLLLEVEEPGAEEEHGADTAPTLAPEVLPSQGEVEREEGSPSAGPPPQELVEEEPSAPPTLLEEGTE
DGGDRVQPPPETPAEEEMETETEAEALQEKVRGPGREGTLGEKVGLSGEWVAAAYLRRRLPSLNHSVCVCDYSESCIFVP
KRSRMTQLPCWPTSSIVPLMMRSHRLPQSLTPSHLLHPTLCFQ
> Sapajus apella [A0A6J3H4F1]
MAAAVLSGPTAGSAAGVPGGTGGLSAVSSGPRLRLLLLESVSGLLQPRTGSAVAPVHPPNRSAPHLPGLMCLLRLHGSVG
GAQNLSAVGALVNLSNARLSSIKTRFEGLCLLSLLVGESPTELFQQHCVSWLRSIQQVLQSQDPPPTMELAVAVLRDLLR
YAAQLPALFRDISMNHLPGLLTSLLGLRPECEQSALEGMKACMTYFPRACGSLKGKLASFFLSRVDALSPQLQQLACECY
SRLPSLGAGFSQGLKHTESWEQELHSLLASLHTLLGALYEGAETAPVQNEGPGVEMLLSSEDGDAHVLLRLRQRFSGLAR
CLGLMLSSEFGAPVSVPVQEILDFICRTLSVSSKNISLHGDGPLRLLLLPSIHLEALDLLSALILACGSRLLRFGILISR
LLPQILNSWSIGRDSLSPGQERPYSTVRTKVYAVLELWVQVCGASAGVLQGGASGEALLTHLLSDISPPADALKLRSPRG
SPDGGLQTGKPSAPKKLKLDVGEAMAPPSHRKGDSNANSDVCAAALRGLSRTILMCGPLIKEETHRRLHDLVLPLVMGVQ
QGEVLGSSPYTSSRCRRELYCLLLALLLAPSPRCPPPLACALQAFSLGQREDSLEVSSFCSEALVTCAALTHPRVPPLQS
MGPTCPTPAPVPPPETPSPFRAPPFHPPGPMPSVGPMPSAGPMPSAGPMPSAGPMPSAGPMPSAGPVSSARPGPPTTANH
LGLSVPGLVSVPPRLLPGPENHRASSNEDPILAPSGTPPPAIPPDETFGGRVPRPAFVHYDKEEASDVEISLESDSDDSV
VIVPEGLPPLPPPPPSGATPPPVAPSGPPTASPPVPAKEEPEELPAAPGPLPPPPPPPPPPVPGPVTLPPPQLVPEGTPGG
GGPPALEEDLTVININSSDEEEEEEEEEEEEEEEEEEEEEEEDFEEEEEDEEEYFEEEEEEEEEEFEEEFEEEEGELEEEEEE
EDEDEEEEEELEEVEELEFGTAGGEVEEGGPPPPTLPPALPPPESPPKVQPEPEPEPEPGLLLEVEEPGAEEEHEADTAPTLA
PEVLPSQGEVERDGGSPAAGLPPQELVEEEPSAPPTLLEEGTEDGGDKVQLPPETSAEEEMETETEAEALQEKEQDDTAA
MLADFIDCPPDDEKPPPATEPDS
> Macaca mulatta [A0A5F7ZGA1]
MEIVRTLAPFVRTTCTLNITGPPRTRSPAITPGTQHPGASAPRLLSPRMLLPSLSPRVQPAFIQCAGPQPPPHAHSPVGG
RRGVFTSSRHATTGARGKMAAAVLSGSSAGSAAGVPGGTGGLSAVNSGPRLRLLLLESVSGLLQPRTGSAVAPVHPPNRS
APHLPGLMCLLRLHGSVGGAQNLSALGALVSLSNARLSSIKTRFEGLCLLSLLVGESPTELFQQHCVSWLRSIQQVLQTQ
DPPATMELAVAVLRDLLRYAAQLPALFRDISMNHLPGLLTSLLGLRPECEQSALEGMKACMTYFPRACGSLKGKLASFFL
SRVDALSPQLQQLACECYSRLPSLGAGFSQGLKHTESWEQELHSLLASLHTLLGALYEGAETAPVQNEGPGVEMLLSSED
GDAHVLLRLRQRFSGLARCLGLMLSSEFGAPVSVPVQEILDFICRTLSVSSKNISLHGDGPLRLLLLPSIHLEALDLLSA
LILACGSRLLRFGILISRLLPQVLNSWSIGRDSLSPGQERPYSTVRTKVYAVLELWVQVCGASAGMLQGGASGEALLTHL
LSDISPPADALKLRSPRGSPDGSLQTGKPSAPKKLKLDVGEAMAPPSHRKGDSNANSDVCAAALKGLSRTILMCGPLIKE
ETHRRLHDLVLPLVMGVQQGEVLGSSPYTSSRCRRELYCLLLALLLAPSPRCPPPLACALQAFSLGQREDSLEVTVMGQT
LFGSWSLLFPGAHTCPFFFPQVSSFCSEALVTCAALTHPRVPPLQPMGPTCPTPAPVPPPEAPSPFRAPPFHPPGPMPSV
GPMPSAGPMPSAGPMPSAGPVPSARPGPPTTANHLGLSVSGLVSVPPRLLPGPENHRSGSNEDPILAPSGTPPPTIPPDE
TFGGRVPRPAFVHYDKEEASDVEISLESDSDDSVVIVPEGLPPLPPPPPSGATPPPIAPTGPPTASPPVPAKEEPEELPA
APGPLPPPPPPPPPPVPGPVTLPPPQLVPEGTPGGGGPPALEEDLTVININSSDEEEEEEEEEEEEEEEEEEEEEEDFEEEE
EDEEEYFEEEEEEEEEEFEEEFEEEEGELEEEEEEEDEEEEEEELEEVEELEFGTAGGEVEEGGPPPPTLPPALPPPESPPK
VQPEPEPEPEPGLLLEVEEPGAEEEHGADTAPTLAPEVLPSQGEVEREEGSPSAGPPPQELVEEEPSAPPTLLEEGTEDGGD
RVQPPPETPAEEEMETETEAEALQEKEQDDTAAMLADFIDCPPDDEKPPPPTEPDS
> Saimiri boliviensis boliviensis [A0A2K6SZB7]
MLLPSLSPRVQLAFTQCAGPQPPPHTPSPLGGRRSVFASSRHATTGAPGKMAAAVLSGPTAGSAAGVPGGTGGLSAVSSG
PRLRLLLLESVSGLLQPRTGSAVAPVHPPNRSAPHLPGLMCLLRLHGSVGGAQNLSAVGALVSLSNARLSSIKTRFEGLC
LLSLLVGESPTELFQQHCVSWLRSIQQVLQSQDPPPTMELAVAVLRDLLRYAAQLPALFRDISMNHLPGLLTSLLGLRPE
CEQSALEGMKACMTYFPRACGSLKGKLASFFLSRVDALSPQLQQLACECYSRLPSLGAGFSQGLKHTESWEQELHSLLAS
LHTLLGALYEGAETAPVQNEGPGVEMLLSSEDGDAHVLLQLRQRFSGLARCLGLMLSSEFGAPVSVPVQEILDFICRTLS
VSSKNISLHGDGPLRLLLLPSIHLEALDLLSALILACGSRLLRFGILISRLLPQVLNSWSIGRDSLSPGQERPYSTVRTK
VYAVLELWVQVCGASAGVLQGGASGEALLTHLLSDISPPADALKLRSPRGSPDGGLQTGKPSAPKKLKLDVGEAMAPPSH
RKGDSNANSDVCAAALRGLSRTILMCGPLIKEETHRRLHDLVLPLVMGVQQGEVLGSSPYTSSRCRRELYCLLLALLLAP
SPRCPPPLACALQAFSLGQREDSLEVSSFCSEALVTCAALTHPRVPPLQSMGPTCPTPAPVPPPEAPSPFRAPPFHPPGP
MPSVGPMPSAGPMPSAGPMPSAGPMPSAGPMPSAGPVPSVRPGPPTTANHLGLSVPGLVSVPPRLLPGPENHRASSNEDP
ILAPSGTPPPTIPPDETFGGRVPRPAFVHYDKEEASDVEISLESDSDDSVVIVPEGLPPLPPPPPSGATPPPVAPSGPPT
ASPPVPAKEEPEELPAAPGPLPPPPPPPPPPVPGPVTLPPPQLVPEGTPGGGGPPALEEDLTVININSSDEEEEEEEEEEE
EEEEEEEEEEDFEEEEEDEEEYFEEEEEEEEEEFEEEFEEEGELEEEEEEEEDEDEEEEEELEEVEELEFGTAGGEAEEGGPP
PPTLPPALPPPESPPKVQPEPEPEPEPGLLLEVEEPGAEEEHEADTAPTLAPEVLPSQGEVERAGGSPEAGLPPQELVEEEP
SAPPTLLEEGTEDGGDKVQLPPETSAEEEMETETEAEALQEKEQDDTAAMLADFIDCPPDDEKPPPATEPDS
> Otolemur garnettii [H0WLZ2]
MAAAVLSGPSGGSAAGVPGGTGGLSAVNSGPRLRLLLLESVSGLLQPRTGSAVAPVHPPVRSAPHLPGLMCLLRLHGTVG
GAQNLSAVGALVSLSNARLGSIKTRFEGLCLLSLLVGESPTEIFQQHCVSWLRSIQQVLQSQDPPPTMELAVAVLKDLLR
YAAQLPTLFRDISMNHLPGLLTSLLGLRLECEQSALEGMKACMTYFPRACGSLKGKLASFFLSRVDSLSPQLQQLACECY

SRLPSLGAGFSQGLKHTESWEQELHSLLASLHSLLGALYEGAETAPVQNEGPGVETLLSSSEDGDAHVLLRLWQRFSGLA
RCLGLMLSSEFGAPVSVPVQEILDIICRTLSVSGKNISLLGDGPLRLLLLPSIHLEALDLLSALILACGGRLLRFGGLIS
RLLPQVLSAWSIGRDSLSPGQERPYSTIRTKVYAILELWVQVCGASAGVLQGGASGEALLTHLLSDISPPADALKLRSPR
GSPDGGLQTGKPSAPKKLKLDVGEAMAPPSHRKGDSNANSDVCAAALRGLSRTILMCGPLIKEETHRRLHDLILPLVMGV
QQGEVLGSSPYTSSCCRRELYRLLLALLLAPSPRCPPPLACALQAFSLGQQQDNLEVSSFCSEALVTCAALTHPRVPPLQ
SMSPTCPTPAPVPPPEAPSPFRAPPFHPPGPMPPVGPIPSAGPMPSAGPMPSAGPVPSARPGPPATANHLGLSVPGLVSV
PPRLLPGPENHRAGSNEDPVLAPSGTPPPTIPPDETFGGRVPRPAFVHYDKEEASDVEISLESDSDDSVVIVPEGLPPLP
PPPPSGTTPPPVASTGPLTASPPMPAKEEPEELPATPGPLPPPPPPPPPVPGPVTLPPPQLVPEGTPGGGGPPALEEDLT
VININSSDEEEEEEEEDEEEEEEEEEEEEDFEEEEEDEEEYFEEEEEEEEEFEEEFEEEGELEEEEEEEEDDEEEELEEVE
DLEFGAAGGEVEEGGPPPPTLPPALPPPESPQGQPEPEPEAGLLLEVEEPGAEEDPGAEAAPTLAPEVLPAQEEVEREGE
SPIAGPPPQELVEEEPSAPPTLLEEGTGDGGDKVPPPPETPLEEEMETEAEATALQEKEQDDTAAMLADFIDCPPDDEKP
PPTTEPDS
> Cercocebus atys [A0A2K5LPL1]
MLLPSLSPRVQPAFIQCAGPQPPPHAHSPVGGRRGVFASSRHATTGAPGKMAAAVLSGPSAGSAAGVPGGTGGLSAVNSG
PRLRLLLLESVSGLLQPRTGSAVAPVHPPNRSAPHLPGLMCLLRLHGSVGGAQNLSALGALVSLSNARLSSIKTRFEGLC
LLSLLVGESPTELFQQHCVSWLRSIQQVLQTQDPPATMELAVAVLRDLLRYAAQLPALFRDISMNHLPGLLTSLLGLRPE
CEQSALEGMKACMTYFPRACGSLKGKLASFFLSRVDALSPQLQQLACECYSRLPSLGAGFSQGLKHTESWEQELHSLLAS
LHTLLGALYEGAETAPVQNEGPGVEMLLSSEDGDAHVLLRLRQRFSGLARCLGLMLSSEFGAPVSVPVQEILDFICRTLS
VSSKNISLHGDGPLRLLLLPSIHLEALDLLSALILACGSRLLRFGILISRLLPQVLNSWSIGRDSLSPGQERPYSTVRTK
VYAVLELWVQVCGASAGMLQGGASGEALLTHLLSDISPPADALKLRSPRGSPDGSLQTGKPSAPKKLKLDVGEAMAPPSH
RKGDSNANSDVCAAALKGLSRTILMCGPLIKEETHRRLHDLVLPLVMGVQQGEVLGSSPYTSSRCRRELYCLLLALLLAP
SPRCPPPLACALQAFSLGQREDSLEVAVMGQTLFGPWTLLFPGAHTCPFFFPQVSSFCSEALVTCAALTHPRVPPLQPMG
PTCPTPAPVPPPEAPSPFRAPPFHPPGPMPSVGPMPSAGPMPSAGPMPSAGPVPSARPGPPTTANHLGLSVSGLVSVPPR
LLPGPENHRAGSNEDPILAPSGSPPPAIPPDETFGGRVPRPAFVHYDKEEASDVEISLESDSDDSVVIVPEGLPPLPPPP
PSGATPPPIAPTGPPTASPPVPAKEEPEELPAAPGPLPPPPPPPPPVPGPVTLPPPQLVPEGTPGGGGPPALEEDLTVIN
INSSDEEEEEEEEEEEEEEEEEEEEEDFEEEEEDEEEYFEEEEEEEEEFEEEFEEEEGELEEEEEEEEDEEEEEEELEEVEE
LEFGTAGGEVEEGGPPPPTLPPALPPPESPPKVPPEPEPEPGLLLEVEEPGAEEEHGADTAPTLAPEVLPSQGEVEREEG
SPSAGPPPQELVEEEPSAPPTLLEEGTEDGGDRVQPPPETPAEEEMETETEAEALQEKVRGPGREGTLGEKVGLSGEWVA
AAYLRRRLPSLNHSVCVCDYSESCIFVPKRSRMTQLPCWPTSSIVPLMMRSHRLPQSLTPSHLLHPTLCFQ
> Propithecus coquereli [A0A2K6G9E4]
MLLPGLSPRVQPPFPQCAGPQPPPQTPSPPSVRRGVFASSRHATTRARGKMAAAVLSGPSTGSAAGVPGGTGGLSAVNSG
PRLRLLLLESVSGLLQPRAGPAVAPVHPPVRSAPHLPGLMCLLRLHGTVGGAQNLSAVAALVSLSNARLGSIKTRFEGLC
LLSLLVGESPTEIFQQHCVSWLRSIQQVLQSQDPPPTMELAVAVLRDLLRYAAQLPALFRDISMNHLPGLLTSLLGLRPE
CEQSALEGMKACMTYFPRACGSLKGKLASFFLSRVDSLIPQLQQLACECYSRLPSLGAGFSQGLKHTESWEQELHSLLTS
LHSLLGTLYEGAETAPVQNEGPGVETLLSSSEDGDAHVLLRLRQRFSGLARCLVLMLSSEFGAPVSVPVQEILDLICRTL
SISGKNINLLGDGPLRLLLLPSIHLEALDLLCALILACGGRLLRFGVLISRLLPQVLNAWSIGRDSLSPGQERPYSTIRT
KVYAILELWVKVCGASAGVLQGGASGEALLTHLLSDISPPADALKLRSPRGSPDGGLQTGKPSAPKKLKLDVGEAMAPPS
HRKGDSNANSDVCAAALRGLSRTILMCGWHSNLGVLHDLVLPLVMGVQQGEVLGSSPYTSSCCRRELYRLLLALLLAPSP
RCPPPLACALQAFSLGQQEDSLEVSSFCSEALVTCAALTHPRVPPLQSMGPTCPAPAQVPPPEAPSPFRAPPFHPPGPMP
SAGSMASAGPMPSAGPMPSTGSMPSAGPVPPAPTANHLGLPVPGLVSVPPRLLPGPENHRAGSNEDPVLAPSGTPPPTIP
PDETFGGRVPRPAFVHYDKEEASDVEISLESDSDDSVVIVPEGLPPLPPPPPSGTTPPPIAPAGPPTASPPVPAKEEPEE
LPAAPGPLPPPPPPPPPVPGPVTLPPPQLVPEGTPGWGGPPALEEDLTVININSSDEEEEEEEEEEEEEEEEEEEEEEDFE
EEEEDEEEYFEEEEEEEEEFEEEFEEEEGELEEEEEEEEDEEEEEEELEEVEELEFGSAGGEVEEGGPPPPTLPPALPPPES
PKVQPEPEPEPEPGLLLEVEEPGAEEEHGAETAPTLAPEVLPSQGEVEREGGSPAAGPPPQELVEEEPSAPPTLLEEGTEGG
GDKVPPPPETPAEEEMETEAEATALQEKEQDDTAAMLADFIDCPPDDEKPPPTTEPDS
> Sapajus apella [A0A6J3H434]
MHTWNLSAVGALVNLSNARLSSIKTRFEGLCLLSLLVGESPTELFQQHCVSWLRSIQQVLQSQDPPPTMELAVAVLRDLL
RYAAQLPALFRDISMNHLPGLLTSLLGLRPECEQSALEGMKACMTYFPRACGSLKGKLASFFLSRVDALSPQLQQLACEC
YSRLPSLGAGFSQGLKHTESWEQELHSLLASLHTLLGALYEGAETAPVQNEGPGVEMLLSSEDGDAHVLLRLRQRFSGLA
RCLGLMLSSEFGAPVSVPVQEILDFICRTLSVSSKNISLHGDGPLRLLLLPSIHLEALDLLSALILACGSRLLRFGILIS
RLLPQILNSWSIGRDSLSPGQERPYSTVRTKVYAVLELWVQVCGASAGVLQGGASGEALLTHLLSDISPPADALKLRSPR
GSPDGGLQTGKPSAPKKLKLDVGEAMAPPSHRKGDSNANSDVCAAALRGLSRTILMCGPLIKEETHRRLHDLVLPLVMGV
QQGEVLGSSPYTSSRCRRELYCLLLALLLAPSPRCPPPLACALQAFSLGQREDSLEVSSFCSEALVTCAALTHPRVPPLQ
SMGPTCPTPAPVPPPETPSPFRAPPFHPPGPMPSVGPMPSAGPMPSAGPMPSAGPMPSAGPVSSARPGPPTTAN
HLGLSVPGLVSVPPRLLPGPENHRASSNEDPILAPSGTPPPAIPPDETFGGRVPRPAFVHYDKEEASDVEISLESDSDDS
VVIVPEGLPPLPPPPPSGATPPPVAPSGPPTASPPVPAKEEPEELPAAPGPLPPPPPPPPPVPGPVTLPPPQLVPEGTPG
GGGPPALEEDLTVININSSDEEEEEEEEEEEEEEEEEEEEEEDFEEEEEDEEEYFEEEEEEEEEFEEEFEEEEGELEEEEE
EEDEDEEEEELEEVEELEFGTAGGEVEEGGPPPPTLPPALPPPESPPKVQPEPEPEPGLLLEVEEPGAEEEHEADTAPTL
APEVLPSQGEVERDGGSPAAGLPPQELVEEEPSAPPTLLEEGTEDGGDKVQLPPETSAEEEMETETEAEALQEKEQDDTA
AMLADFIDCPPDDEKPPPATEPDS
> Cavia porcellus [H0UTE9]
MAAAVLSGPSAGSPAGAPGGAGGLSAMSSGPRLRLLLLESVSGLLQPRTGSSVAPVHPPVCSAPHLPGLMCLLRLHGTVG
GAQNLSAVGALVNLSNACLSSMKTRFEGLCLLSLLVGESPTDMFQQHCVSWLRSIQQVLQSQDPPPTMELAVAVLKELLR
YAAQLPALSRDISVNHLPGLLTSLLGLRPECAQSALEGMKACMTYFPRACGSLKGKLASFFLSRVHSLSPQLQQLACECY
SRVPSLGAGFSQGLKHTESWEKELHNLLASLHSLLGALYEGAEAAPVQSEGPGVEMLLSHSEEGDAHVLLRLRQKFSGLA
RCLGLMLSSEFEAPVSVPVQEVLDLICRILSVSSKNISLLGDGPLRLLLLPSIHLEALDLLSALILACGGRLLRFGALIS
RLLPQVLNAWSIGRDTLSPGQERPYSTIRTKVYAVLELWVQVCGASAGVLQGGASGEALFTHLLSDISPPADTLKLRSPR
GSPDGGLQTGKPSAPKKLKLDMGEAVAPPSQRKGDSNANSDVCAAALRGLSRTILMCGPLIKEETHRRLHDLVLPLAMGV
QQGEVLGSSPYSSSCCRRELYRLLLALLLAPSPRCPPPLSCALQAFSLGQREHSLEVSSFCSEALVTCAALTHPRVPPLQ
TVGPPCPTSSAVPPPEAPSPFRAPPFHPPGPMPSVGPMPSAGPLPAAGPMSSVGPVPSVGSIGPVPPARPGPPATANHLG
LSVPGLVSVPPRLLPGPENHRAGSSEDPVLAPSGTPPPTIPPDETFGGRVPRPAFVHYDKEEASDVEISLESDSDDSVVI
VPEGLPPLPPPPPSGTSPPPVAPAGPPTASPPVPAKEEPEELPATPGPLPPPPPPPPVPGPVTLPPPQLVPEGTPGGAGPP
ALEEDLTVININSSDEEEEEEEDEEEEEEEEEEEEEEEDFEEEEEEEEEEYLEEEEEEEEEFEEEFEEEEGELEEEEEEEEEEE
EEEELEEVEDLEFGPAGAEVEEESGPPPPTLPPALPPPESPRVPPEPEPEPEPELLLEVEEPGTEEGPGPAETAPTLAPEGL
PAQGEVERGGESPSAGPPPPEPAQEEPSAPPALQEEETEGGRDKVPLPPETPMEELETVAEATAVQEKEEDATAAMLADF
IDCPPDDEKPLPAAEPDS
> Ryukyu mouse [A0A6P5QR15]

MAAAVLSGASAGSPAGAPGGPGGLSAVGSGPRLRLLLLESISGLLQPRTGSPVAPVHPPIQWAPHLPGLMCLLRLHGTAG
GAQNLSALGALVNLSNAHLGSIKTRFEGLCLLSLLVGESPTELFQQHCVSWLRSIQQVLQSQDSPSTMELAVAVLRDLLR
YASQLPTLFRDISTNHLPGLLTSLLGLRPECEQSALEGMKACVTYFPRACGSLKGKLASFFLSRLDSLNPQLQQLACECY
SRLPSLGAGFSQGLKHTENWEQELHSLLTSLHSLLGSLFEETEPAPVQSEGPGIEILLSHSEDGKTHVLLQLRQRFSGLA
RCLGLMLSSEFGAPVSVPVQEILDLICRILGISSKNINLLGDGPLRLLLLPSLHLEALDLLSALILACGSRLLRFGALIS
RLLPQVLNAWSTGRDTLAPGQERPYSTIRTKVYAILELWVQVCGASAGVLQGGASGEALLTHLLSDISPPADALKLCSTR
GSSDGGLQSGKPSAPKKLKLDMGEALAPPSQRKGDRNANSDVCAAALRGLSRTILMCGPLIKEETHRRLHDLVLPLVMSV
QQGEVLGSSPYNSSCCRLGLYRLLLALLLAPSPRCPPPLACALKAFSLGQWEDSLEVSSFCSEALVTCAALTHPRVPPLQ
SSGPACPTPAPVPPPEAPSSFRAPAFHPPGPMPSIGAVPSTGPLPSAGPIPTVGSMASTGQVPSRPGPPATANHLGLSVP
GLVSVPPRLLPGPENHRAGSSEDPVLAPSGTPPPSIPPDETFGGRVPRPAFVHYDKEEASDVEISLESDSDDSVVIVPEG
LPSLPPAPPSGTPPPAAPAGPPTASPPVPAKEDSEELPATPGPPPPPPPPPPPPPASGPVTLPPPQLVPEGTPGGGGPTAME
EDLTVININSSDEEEEEEEEEEEEDEDEEEEDFEEEEEDEEEYFEEEEEEEEFEEEFEEEEGELEEEEEEEEEELEEVED
VEFGSAGEVEEGGPPPPTLPPALPPTDSPKVQPEAEPEPGLLLEVEEPGPEEVPGPETAPTLAPEVLPSQEEGEQEVGSP
AAGPPQELVEESSAPPALLEKGNEGGGDKVPPPPETPAEEEMETEAEVSAPQEKEQDDTAAMLADFIDCPPDDEKAPPAT
EPDS
> Peromyscus maniculatus bairdii [A0A6J0CK24]
MAAAVLSGPSAGSPAGAPGGAGGLSTVGSGPRLRLLLLESVSGLLQPRTGSPVAPVHPPIHWAPHLPGLMCLLRLHGTVG
GAQNLSALGALVNLSNAHLGSIKTRFEGLCLLSLLVGESPTELFQQHCVSWLRSIQQVLQSQDSPPTMELAVAILRDLLR
YASQLPTLFRDISTNHLPGLLTSLLGLRPECEQSALEGMKACVTYFPRACGSLKGKLASFFLSRLDALNPQLQQLACECY
SRLPSLGAGFSQGLKHTENWEQELHSLLASLHSLLGTLYEGAETAPVQSEGPGVEMLLSQSEDSNTHVLLQLRQRFSGLA
RCLGLMLSSEFGAPVSVPVQEILDLICRILSISSKNINLLGDGPLRLLLLPSIHLEALDLLSALILACGGRLLRFGALIS
RLLPQVLSTWSTGRDTLAPGQERPYSTIRTKVYAILELWVQVCGASAGMLQGGASGEALLTHLLSDISPPADALKLCGPR
GSSDGGLQSGKPSAPKKLKLDVGEALAPSSHRKGDRNANSDVCAAALRGLSRTILMCGPLIKEETHRRLHDLVLPLVMSV
QQGEVLSGSPYNSACCRRELYRLLLALLLAPSPRCPPPLACALKAFSLGQEEDSLEVSSFCSEALVTCAALTHPRVPPLQ
SSGPACPAPAPVPPPEAPSPFRAPPFHPPGPIPSIGAMPSPGPAPPAGPLPSAGPIPTLGSMSSAGSVQSTGPVPSRPGP
PATANHLGLSVPGLVSVPPRLLPGPENHRAGSSEDPVLAPSGTPPPSIPPDETFGGRVPRPAFVHYDKEEASDVEISLES
DSDDSVVIVPEGLPSLPPPPSGSPPPVAPAGPPTASPPVPAKEDSEELPATPGPLPPPPPPPPPPVSGPVTLPPPQLVPE
GTPGGGGPTAMEEDLTVININSSDEEEEEEEEEEEEDEDEEEEDFEEEEEDEEEYFEEEEEEEEFEEEFEEEEGELEEEE
EEEEEEEEELEEVEEFGSIGGEVEEGGPPPPTLPPALPPTDSPKAQPEAEPEPGLLLEVEEPGAEEVPEPETAPTLAPEV
LPSQEEVEQEGGSPTAGPPQELVEEEASAPPALLEEGTEGGGDKVPPPPETPAEEEMEAEAEAAAPQEKEQDDTAAMLAD
FIDCPPDDEKPPPAEEPDS
> Rhinopithecus roxellana [A0A2K6P9H7]
MLLPSLSPRVQPAFTQCAGPQPPPHAHSPVGGRRGVFASSRHATTGARGKMAAAVLSGPSAGSAAGVPGGTGGLSAVNSG
PRLRLLLLESVSGLLQPRTGSAVAPVHPPNRSAPHLPGLMCLLRLHGSVGGAQNLSALGALVSLSNARLSSIKTRFEGLC
LLSLLVGESPTELFQQHCVSWLRSIQQVLQTQDPPATMELAVAVLRDLLRYAAQLPALFRDISMNHLPGLLTSLLGLRPE
CEQSALEGMKACMTYFPRACGSLKGKLASFFLSRVDALSPQLQQLACECYSRLPSLGAGFSQGLKHTESWEQELHSLLAS
LHTLLGALYEGAETAPVQNEGPGVEMLLSSEDGDAHVLLRLRQRFSGLARCLGLMLSSEFGAPVSVPVQEILDFICRTLS
VSSKNISLHGDGPLRLLLLPSIHLEALDLLSALILACGSRLLRFGILISRLLPQVLNSWSIGRDSLSPGQERPYSTVRTK
VYALLELWVQVCGASAGVLQGGASGEALLTHLLSDISPPADALKLRSPRGSPDGSLQTGKPSAPKKLKLDVGEAMAPPSH
RKGDSNANSDVCAAALRGLSRTILMCGPLIKEETHRRLHDLVLPLVMGVQQGEVLGSSPYTSSRCRRELYCLLLALLLAP
SPRCPPPLACALQAFSLGQREDSLEVSSFCSEALVTCAALTHPRVPPLQPMGPTCPTPAPVPPPEAPSPFRAPPFHPPGP
MPSVGPMPSAGSMPSAGPMPSAGPVPSARPGPPTTANHLGLSVSGLVSVPPRLLPGPENHRAGSNEDPILAPSGTPPPAI
PPDETFGGRVPRPAFVHYDKEEASDVEISLESDSDDSVVIVPEGLPPLKKSHPAGVSGGVPDTVSTKIGAEEVRPRSGRA
IEEEDEEEEEEELEEVEELEFGTAGGEVEEGGPPPPTLPPALPPESPPKVQPEPEPEPGLLLEVEEPGAEEEHGADTAPT
LAPEVLPSQGEVEREEGSPLAGPPPQELVEEEPSAPPTLLEEGTEDGGDRVQPPPETPAEEEMETETEEAEALQEKEQDD
TAAMLADFIDCPPDDEKPPPPTEPDS
> Rattus norvegicus [A0A0G2JSQ9]
MLSLATRASVLIHSESGRSPLLKTPPRAACVALMLRHHATPLLEPVKMAAAVLSGPTTGSPAGAPGGPGGLSAAGSGPRL
RLLLLESVSGLLQPRTGSHVAPVHPPIQWAPYLPGLMCLLRLHGTAGGAQNLSALGALVNLSNAHLSSIKTRFEGLCLLS
LLVGESPTELFQQHCVSWLRSIQQVLQSQDSPPTMELAVAILRDLLRYASQLPTLFRDISTNHLPGLLTSLLGLRPECEQ
SALEGMKACVTYFPRACGFLKGKLASFFLSRLDSLNPQLQQLACECYSRLPSLGAGFSQGLKHTENWEQELHSLLTSLHS
LLGSLFEETETAPVQSEGPGVEMLLSPSEDDNTHVLLQLWQRFSGLARCLGLMLSSEFGAPVSVPVQEILDLICRILGIS
SKNINLLGDGPLRLLLLPSLHLEALDLLSALILACGGRLLRFGALISRLLPQVLNTWSTGRDALAPGQERPYSTIRTKVY
AILELWVQVCGASAGMLQGGASGEALLTHLLSDISPPADALKLCSTRGSSDGGLQSGKPSAPKKLKLDMGEALAPPSQRK
GDRNANSDVCAAALRGLSRTILMCGPLVKEETHRRLHDLVLPLVMSVQQGEVLGSSPYNSSCCRLELYRLLLALLLAPSP
RCPPPLSCALKAFSLGQWEDSLEVSSFCSEALVTCSALTHPRVPPLQSSGPACPTPAPVPPPEAPSSFRAPAFHTPGPMP
SIGALPSPGPVPSAGPIPTVGSMSSAGSVPSTGPVPSRPGPPATANHLGLAVPGLVSVPPRLLPGSENHRAGSGEDPVLA
PSGTPPPSIPPDETFGGRVPRPAFVHYDKEEASDVEISLESDSDDSVVIVPEGLPSLPPPPSGTPPPVAPTGPPTASPPV
PAKEDSEELPATPGPLPPPPPPPPPPVSGPVTLPPPQLVPEGTPGGGGPTAMEEDLTVININSSDEEEEEEEEEEEDEDE
EEEDFEEEEEDEEEYFEEEEEEEEFEEEFEEEEGELEEEEEEEEEELEEVEDVEFGSAGEVEEGGPPPPTLPPALPPTDS
PKVQPEAEPEPGLLLEVEEPGAEDGPGPEIAPTLAPEVLPSQEEVEREGESPTAGPPQELVEEESSAPPTLLEEGTEGGG
DKVPPPPETPAQEEMETETEASAPQGKEQDDTAAMLADFIDCPPDDEKPPPATEPDS
> Mesocricetus auratus [A0A1U7QA24]
MAAAVLSGPSAGSPAGAPGGPGGLSTVGSGPRLRLLLLESVSGLLQPRTGSPVAPVHPSMHWAPHLPGLMCLLRLHGTVG
GAQNLSALGALVNISNAHLGSIKTRFEGLCLLSLLVGESPTELFQQHCVSWLRSIQQVLQSQDSPPTMELAVAILRDLLR
YASQLPTLFRDISTNHLPGLLTSLLGLRPECEQSALEGMKACVTYFPRACGSLKGKLASFFLSRLDALNPQLQQLACECY
SRLPSLGAGFSQGLKHTENWEQELHSLLASLHSLLGTLYEGAETDPVQSEGPGVETLPSHSEDGTTHVLLQLRQRFSGLA
RCLGLMLSSEFGAPVSVPVQEILDLICRILSVSNKSINLLGDGPLRLLLLPSIHLEALDLLSALILACGGRLLRFGALII
RLLPQVLNTWSTGRDTLAPGQERPYSTIRTKVYAILELWVQVCGSSAGVLQGGASGEALLTHLLSDISPPADTLKLCATR
GSSDGGLQGGKPSAPKKLKLDMGEALAPASHRKADRNANSDVCAAALRGLSRTILMCGPLIKEETHRRLHDLVLPLAMSV
QQGEVLGGSPYNSSCCRRELYRLLLALLLAPSPRCPPPLACALKAFSLGQCEDSLEVSSFCSEALVTCSALTHPRVPPLQ
SSGPACPTPAPVPPPEAPSPFRAPPFHPPGPMPSIGAMPSPGPIPSAGPLPSAGPLPTVGSISSAGPMPSTGPVPSRPGP
PATANHLGLSVPGLVSVPPRLLPGPENHRAGSSEDPVLAPSGTPPPSIPPDETFGGRVPRPAFVHYDKEEASDVEISLES
DSDDSVVIVPEGLPSLPPPPSGSPPPVTATGPATASPPAPAKEDSEELPATPGPLPPPPPPPPPPVSGPVALPPPQLVPE
GTAGGGGPSAMEEDLTVININSSDEEEEEEEEEEEEDEDEEEEDFEEEEEDEEEYLEEEEEEEEFEEEFEEEEGELEEEE
EEEEEEEELEEVEDVEFGSAGGEVEEGGPPPPTLPPALPPTDSPKAQPEAEPEPGLLLDVEEPGAEEAPGPETAPTLTPE
VLPSQEEVVQGAGSPAVGPPQELVEEESSVPPTLLEEGTEGECDKGSPPPETPAEAEMETEAEAATPQEKEQDDTAAMLA

DFIDCPPDDEKPPPDPEPDS
> Peromyscus maniculatus bairdii [A0A6I9MHE3]
MAAAVLSGPSAGSPAGAPGGAGGLSTVGSGPRLRLLLLESVSGLLQPRTGSPVAPVHPPIHWAPHLPGLMCLLRLHGTVG
GAQNLSALGALVNLSNAHLGSIKTRFEGLCLLSLLVGESPTELFQQHCVSWLRSIQQVLQSQDSPPTMELAVAILRDLLR
YASQLPTLFRDISTNHLPGLLTSLLGLRPECEQSALEGMKACVTYFPRACGSLKGKLASFFLSRLDALNPQLQQLACECY
SRLPSLGAGFSQGLKHTENWEQELHSLLASLHSLLGTLYEGAETAPVQSEGPGVEMLLSQSEDSNTHVLLQLRQRFSGLA
RCLGLMLSSEFGAPVSVPVQEILDLICRILSISSKNINLLGDGPLRLLLLPSIHLEALDLLSALILACGGRLLRFGALIS
RLLPQVLSTWSTGRDTLAPGQERPYSTIRTKVYAILELWVQVCGASAGMLQGGASGEALLTHLLSDISPPADALKLCGPR
GSSDGGLQSGKPSAPKKLKLDVGEALAPSSHRKGDRNANSDVCAAALRGLSRTILMCGPLIKEETHRRLHDLVLPLVMSV
QQGEVLSGSPYNSACCRRELYRLLLALLLAPSPRCPPPLACALKAFSLGQEEDSLEVSSFCSEALVTCAALTHPRVPPLQ
SSGPACPAPAPVPPPEAPSPFRAPPFHPPGPIPSIGAMPSPGPAPPAGPLPSAGPIPTLGSMSSAGSVQSTGPVPSRPGP
PATANHLGLSVPGLVSVPPRLLPGPENHRAGSSEDPVLAPSGTPPPSIPPDETFGGRVPRPAFVHYDKEEASDVEISLES
DSDDSVVIVPEGLPSLPPPPPSGSPPPVAPAGPPTASPPVPAKEDSEELPATPGPLPPPPPPPPPPVSGPVTLPPPQLVPE
GTPGGGGGGGGGGGGGGPTAMEEDLTVININSSDEEEEEEEEEEEEEDEDEEEEEDFEEEEEDEEEYFEEEEEEEEFEEEFE
EEEGELEEEEEEEEEEEEEELEEVEEFGSIGGEVEEGGPPPPTLPPALPPTDSPKAQPEAEPEPGLLLEVEEPGAEEVPEP
ETAPTLAPEVLPSQEEVEQEGGSPTAGPPQELVEEEASAPPALLEEGTEGGGDKVPPPPETPAEEEMEAEAEAAAPQEKE
QDDTAAMLADFIDCPPDDEKPPPAEEPDS
> Rhinopithecus bieti [A0A2K6MRA3]
MLLPSLSPRVQPAFTQCAGPQPPPHAHSPVGGRRGVFASSRHATTGARGKMAAAVLSGPSAGSAAGVPGGTGGLSAVNSG
PRLRLLLLESVSGLLQPRTGSAVAPVHPPNRSAPHLPGLMCLLRLHGSVGGAQNLSALGALVSLSNARLSSIKTRFEGLC
LLSLLVGESPTELFQQHCVSWLRSIQQVLQTQDPPATMELAVAVLRDLLRYAAQLPALFRDISMNHLPGLLTSLLGLRPE
CEQSALEGMKACMTYFPRACGSLKGKLASFFLSRVDALSPQLQQLACECYSRLPSLGAGFSQGLKHTESWEQELHSLLAS
LHTLLGALYEGAETAPVQNEGPGVEMLLSSEDGDAHVLLRLRQRFSGLARCLGLMLSSEFGAPVSVPVQEILDFICRTLS
VSSKNISLHGDGPLRLLLLPSIHLEALDLLSALILACGSRLLRFGILISRLLPQVLNSWSIGRDSLSPGQERPYSTVRTK
VYALLELWVQVCGASAGVLQGGASGEALLTHLLSDISPPADALKLRSPRGSPDGSLQTGKPSAPKKLKLDVGEAMAPPSH
RKGDSNANSDVCAAALRGLSRTILMCGPLIKEETHRRLHDLVLPLVMGVQQGEVLGSSPYTSSRCRRELYCLLLALLLAP
SPRCPPPLACALQAFSLGQREDSLEVSSFCSEALVTCAALTHPRVPPLQPMGPTCPTPAPVPPPEAPSPFRAPPFHPPGP
MPSVGPMPSAGPMPSAGPMPSAGPVPSARPGPPTTANHLGLSVSGLVSVPPRLLPGPENHRAGSNEDPILAPSGTPPPAI
PPDETFGGRVPRPAFVHYDKEEASDVEISLESDSDDSVVIVPEGLPPLPPPPPSGWGTPVPAKEEPEELPAAPGPVPPPP
PPPPPVPGPVTLPPPQLVPEGTPGGGGPPALEEDLTVININSSDEEEEEEEEEEEEEEEEEEEEEEEDFEEEEEDEEEYFEE
EEEEEEEFEEEFEEEEAAMLADFIDCPPDDEKPPPPTEPDS
> Ursus americanus [A0A452R2C2]
MSTVRQVQWPREERGLSGGGGAPAGGLPHGEGGEFPSLHGLPWGEPRLLRPRGVPPGIPSVPCFLAAHTSPRPHSILGVT
ELRGSGGGVCSGPASTPARWPVSWGARLPLRVATGCFLVRFEGLCLLSLLVGESPTEMFQQHCVSWLRSIQQVLQSQDPP
PTMELAVAVLRDLLRYAAQLPTLFRDISMNHLPGLLTSLLGLRPECELSALEGMKACMTYFPRACGSLKGKLASFFLSRV
DALSPQLQQLACECYSRLPSLGAGFSQGLKHTESWEQELHSLLASLHSLLGALYEGAETAPVQYEGPGVEALLTPSEDGD
AHVLLRLRQRFCGLARCLGLMLSSEFGAPVSVPVQEILDVICRTLSISAKNISLLGDGPLRLLLLPSIHLDALDLLSALI
LACGGRLLRFGALISRLLPQVLNAWNFGRDTLSPGQERPYSTVRTKVYAVLELWVQVCGASAGVLQGGASGEALLSHLLS
DISPPADALKLRSPRGSPDGGLQAGKPSAPKKLKLDMGEAMAPPSHRKGDSNANSDVCAAALRGLSRTVLMCGPLIKEET
HRRLHDLVLPLVMGVQQGEVLGSSPYTSSRCRRELYRLLLALLLAPSPRCPPPLACALQAFSLGQREDSLEVSSFCSEAL
VTCAALTHPRVPPLQSMGPACPTPAPVPPPEAPSPFRAPPFHTAGPMPSVGPMPSVGPMSSAGPMPSAGPMPPTRPGPPA
TANHLGLSVPGLVSVPPRLLPGPENHRAGSNEDPVLAPSGTPPPAIPPDETFGGRVPRPAFVHYDKEEASDVEISLESDS
DDSVVIVPEGLPPLPPPPPSGTTPPPVAPAGPPTASPPVPAKEEPEELPAAPGPLPPPPPPPPVPGPVALPPPQLVPEGTP
GSGGPPALEEDLTVININSSDEEEEEEEEEEEEEEEEEEEEEEEDFEEEEEDEEEYFEEEEEEEEEEFEEEFEEEEGELEEEE
DEDEEEEEEELEELEEVEFGPAGGPAEEGGPPPASPAPALPPAQSPKVQPEPEGEPGLLLEVEEPGAEEEAGAEAAPTLAP
EVLPSQGEGPRDTGSPPAGPSPQELMEEEPLAPPTLLEEGTESGGDKVPPPAETAVAEDVETELETATAALQEKEQDDTA
AMLADFIDCPPDDEKPPPAPEPES
> Sapajus apella [A0A6J3H3H8]
MAAAVLSGPTAGSAAGVPGGTGGLSAVSSGPRLRLLLLESVSGLLQPRTGSAVAPVHPPNRSAPHLPGLMCLLRLHGSVG
GAQNLSAVGALVNLSNARLSSIKTRFEGLCLLSLLVGESPTELFQQHCVSWLRSIQQVLQSQDPPPTMELAVAVLRDLLR
YAAQLPALFRDISMNHLPGLLTSLLGLRPECEQSALEGMKACMTYFPRACGSLKGKLASFFLSRVDALSPQLQQLACECY
SRLPSLGAGFSQGLKHTESWEQELHSLLASLHTLLGALYEGAETAPVQNEGPGVEMLLSSEDGDAHVLLRLRQRFSGLAR
CLGLMLSSEFGAPVSVPVQEILDFICRTLSVSSKNISLHGDGPLRLLLLPSIHLEALDLLSALILACGSRLLRFGILISR
LLPQILNSWSIGRDSLSPGQERPYSTVRTKVYAVLELWVQVCGASAGVLQGGASGEALLTHLLSDISPPADALKLRSPRG
SPDGGLQTGKPSAPKKLKLDVGEAMAPPSHRKGDSNANSDVCAAALRGLSRTILMCGPLIKEETHRRLHDLVLPLVMGVQ
QGEVLGSSPYTSSRCRRELYCLLLALLLAPSPRCPPPLACALQAFSLGQREDSLEVSSFCSEALVTCAALTHPRVPPLQS
MGPTCPTPAPVPPPETPSPFRAPPFHPPGPMPSVGPMPSAGPMPSAGPMPSAGPMPSAGPMPSAGPVSSARPGPPTTANH
LGLSVPGLVSVPPRLLPGPENHRASSNEDPILAPSGTPPPAIPPDETFGGRVPRPAFVHYDKEEASDVEISLESDSDDSV
VIVPEGLPPLPPPPPSGATPPPVAPSGPPTASPPVPAKEEPEELPAAPGPLPPPPPPPPPPVPGPVTLPPPQLVPEGTPGG
GGPPALEEDLTVININSSDEEEEEEEEEEEEEEEEEEEEEEEDFEEEEEDEEEYFEEEEEEEEEEFEEEFEEEEGAC
> Odobenus rosmarus divergens [A0A2U3VTQ1]
MAAAVLSGPSAGSAAGVPGGTGGLSAVGSGPRLRLLLLESVSGLLQPRAGSAVAPVHPPVRSAPHLPGLMCLLRLHGTVG
GAQNLSAVGALVGLSNARLGSIKTRFEGLCLLSLLVGESPTEMFQQHCVSWLRSIQQVLQSQDPPPTMELAVAVLRDLLR
YAAQLPTLFRDISMNHLPGLLTSLLGLRPECELSALEGMKACMTYFPRACGSLRGKLASFFLSRVDALSPQLQQLACECY
SRLPSLGAGFSQGLKHTESWQQELRSLLASLHSLLGALYEGAETAPVQYEGPGVEVLLTPSEDGDAHALLRLRQRFCGLA
RCLGLMLSSEFGAPVSVPVQEILDVICRTLSISAKNISLLGDGPLRLLLLPSIHLDALDLLSALILACGGRLLRFGGLIS
RLLPQVLNAWNLGRDTLSPGQERPYSTVRTKVYAVLELWVQVCGASAGVLQGGASGEALLSHLLSDISPPADALKLRSPR
GSPDGGLQTGKPSAPKKLKLDLGEAMAPPSHRKGDSNANSDVCAAALRGLSRTVLMCGPLIKEETHRRLHDLVVPLVMGL
QRGETLGSSPYTSSRCRQELYRLLLALLLAPSPRCPPPLACALQAFSLGQREDSLEVSSFCSEALVVCAALTHPRVPPLQ
PTGPPCPTPAPVPPPEAPSPFRAPPFHPPAGPPPPARPGPPAAANHLGLSVPGLVSVPPRLLPGPENHRASSNEDPVLAP
GGSPPPALPPDETFGGRVPRPAFVHYDKEEASDVEISLESDSDDSVVIVPEGLPPLPPAPPSGATPPPAAPAGPPTASPP
VPAKEEPEELPAAPGPLPPPPPPPPAPGPVALPPPQLVAEGTPGGGGPPSLEEDLTVININSSDEEEEEEEEEEEEEEEEDFE
EEEEEEEEYFEEEEEEEFEEEFEEDEGELEEDDDDDEDDDELEELDEAGFGPAGAPAGDGGPAPPSPAPAPPPAQSPATRP
APEAEPGLLLEVEEPAEEEEEEGGEGPGARAAPTLAPEVLPSQGEGPQATGSPPAGPPPQELVEEEPSAPPTLLEEGTDSG
GDRVPPPPPPETPAAEEAAAEAEAETAALQEKEQDDTAAMLADFIDCPPDDEKPPPAPEPDS
> Rhinopithecus bieti [A0A2K6MR90]
MEIVRTLAPFVRTTCTLNITGPPRTRSPAITPGTEHPGASAPSLLSPRMLLPSLSPRVQPAFTQCAGPQPPPHAHSPVGG

RRGVFASSRHATTGARGKMAAAVLSGPSAGSAAGVPGGTGGLSAVNSGPRLRLLLLESVSGLLQPRTGSAVAPVHPPNRS
APHLPGLMCLLRLHGSVGGAQNLSALGALVSLSNARLSSIKTRFEGLCLLSLLVGESPTELFQQHCVSWLRSIQQVLQTQ
DPPATMELAVAVLRDLLRYAAQLPALFRDISMNHLPGLLTSLLGLRPECEQSALEGMKACMTYFPRACGSLKGKLASFFL
SRVDALSPQLQQLACECYSRLPSLGAGFSQGLKHTESWEQELHSLLASLHTLLGALYEGAETAPVQNEGPGVEMLLSSED
GDAHVLLRLRQRFSGLARCLGLMLSSEFGAPVSVPVQEILDFICRTLSVSSKNISLHGDGPLRLLLLPSIHLEALDLLSA
LILACGSRLLRFGILISRLLPQVLNSWSIGRDSLSPGQERPYSTVRTKVYALLELWVQVCGASAGVLQGGASGEALLTHL
LSDISPPADALKLRSPRGSPDGSLQTGKPSAPKKLKLDVGEAMAPPSHRKGDSNANSDVCAAALRGLSRTILMCGPLIKE
ETHRRLHDLVLPLVMGVQQGEVLGSSPYTSSRCRRELYCLLLALLLAPSPRCPPPLACALQAFSLGQREDSLEVSSFCSE
ALVTCAALTHPRVPPLQPMGPTCPTPAPVPPPEAPSPFRAPPFHPPGPMPSVGPMPSAGPMPSAGPMPSAGPVPSARPGP
PTTANHLGLSVSGLVSVPPRLLPGPENHRAGSNEDPILAPSGTPPPAIPPDETFGGRVPRPAFVHYDKEEASDVEISLES
DSDDSVVIVPEGLPPLPPPPPSAVMKRRRKRKKRKKKKRKKRKRKKTLRKRKRMKRNILKRKKRRKKSLKKNLRKKKVQP
EPEPEPGLLLEVEEPGAEEEHGADTAPTLAPEVLPSQGEVEREEGSPSAGPPPQELVEEEPSAPPTLLEEGTEDGGDRVQ
PPPETPAEEEMETETEEAEALQEKEQDDTAAMLADFIDCPPDDEKPPPPTEPDS
> Dipodomys ordii [A0A1S3EWX8]
MSSGPRLRLLLLESVSGLLQPRPGPPAPPVHPPVHWAPHFPGLMCLLRLHGSVGGAQNLSALGTLVSLSNAHLGSMRTRF
EGLCLLSLLVGESPTEFFQQHCVSWLRCIQHVLQSQDSPPTMELAVAVLRDLLRYAAQLPTLFRDISTNHLPGLLTSLLG
LRPECEHAALEGMKACMTYFPRACGSLKGKLASFFLSRVDALSPQLQQLACECYSRLPSLGAGFSQGLKHTENWEQELHS
LLTSLHSLLGALYEGSETDDVDTHALFRLQQRFSGLAHCMALMLSSEFGAPVSVPVQEVLDLICRILSVNGKNISLLGDG
PLRLLLLPSIHLEALDLLSSLILACGGRLLRFGTLINRLLPQVLNVWSIGRDTLSPGQERPYSTIRIKVYAVLELWVKVC
GASAGVLQGGAPGEALLTHLLSDISPPTDTLKLRSPRGNSDGGLQSGKPSAPKKLKLDMGDAMAPPSHRKGDSNANSDVC
AAALRGLSRTILMCGPLIKEETHRRLHDLVLPLLMSLQQGEVLGSSPYNSSCCRRELYRLLLALLLAPSPRCPPPLACAL
QAFSFGQQEDSLEVSSFCSEALVTCAALTHPRVPPLQTMGPTCATSAPVPPPEAPSSFRAPPFHPPGPMPSVGPMPTPGP
ISSAGPLPSAGPMPSAGPMSSTGPIPSAGPLPSAGPIPSTRPGPPATTNHLGLSVPGLVSVPPRLLPGPENHRAGPSEDP
VLASSGTPPPTIPPDETFGGRVPRPAFVHYDKEEASDVEISLESDSDDSVVIVPEGLPPLPPPPPTSTPPPSVAPTGPPA
ASPPVPAKEEPEELSATPGSLPPPPPPPPPPIAGSATLPPPQLVPEGAPGGGGPPALEEDLTVININSSDEEEEEEEEEE
EEEDDEEEEDFEEEDDDEEEYFEEEEEEEEFEEEFEEEEGELEEEEEEEEEEEEEEELEEVEDLEFGPAGGEVEEGGPPPP
TLPPALPPPESPKVQPEPEPEPGLLLEVEEPGTEEEPGAETAPTLAPEVLPSQGEVEGEGGSPTVGPPPQELVEEPSAHP
PLLEEETEGGSDKVPPPPETSEVEEEMETEAETAALQEKEQDDTAAMLADFIDCPPDDEKPPPTMESDS
> Zalophus californianus [A0A6P9F556]
MAAAVLSGPSAGSAAGVPGGTAGLSAVGSGPRLRLLLLESVSGLLQPRAGSAVAPVHPPVRSAPHLPGLMCLLRLHGTVG
GAQNLSAVAALVGLSNARLGSIKTRFEGLCLLSLLVGESPTEMFQQHCVSWLRSIQQVLQSQDPPPTMELAVAVLRDLLR
YAAQLPTLFRDISMNHLPGLLTSLLGLRPECELSALEGMKACMTYFPRACGSLRGKLASFFLSRVDALSPQLQQLACECY
SRLPSLGAGFSQGLKHTESWQQELRSLLASLHSLLGALYEGAETAPVQYEGPGVEVLLTPSEDGDAHALLRLRQRFCGLA
RCLGLMLSSEFGAPVSVPVQEILDVICRTLSISAKNISLLGDGPLRLLLLPSIHLDALDLLSALILACGGRLLRFGGLIS
RLLPQVLNAWNLGRDTLSPGQERPYSTVRTKVYAVLELWVQVCGASAGVLQGGASGEALLSHLLSDISPPADALKLRSPR
GSPDGGLQTGKPSAPKKLKLDLGEAMAPPSHRKGDSNANSDVCAAALRGLSRTVLMCGPLIKEETHRRLHDLVVPLVMGL
QRGETLGSSPYTSSRCRQELYRLLLALLLAPSPRCPPPLACALQAFSLGQREDSLEVSSFCSEALVVCAALTHPRVPPLQ
PTGPTCPTPAPVPPPEAPSPFRAPPFHPPAGPPPPARPGPPAAANHLGLSVPGLVSVPPRLLPGPENHRAGSSEDPVLAP
GGSPPPALPPDETFGGRVPRPAFVHYDKEEASDVEISLESDSDDSVVIVPEGLPPLPPAPASGATPPPAAPAGPPTASPP
VPAKEEPEELPAAPGPLPPPPPPPAPGPVALPPPQLVPEGTPGGGGPPSLEEDLTVININSSDEEEEEEEEEEEEEDFEEE
EEEEEYFEEEEEEEFEEEFEEEEEEGELEDDDDDDEDDDDELEELDEADFGPAGGPAEAGGPAPPSPAPAPPPASSPGVR
AAPEAEPGLLLEVEEPAADDDDDEDEDDDDDDEEEEEEEEDGGEGPGARAAPTLAPEVLPSQGEGPQAAGSPPAGPPSQEL
VEEEPSAPPTLLEEGADSGGDGVRRPPPETPAAEEAAAEAEAETAALQEKEQDDTAAMLADFIDCPPDDEKPPPAPEPDS
> Callorhinus ursinus [A0A3Q7NH21]
MAAAVLSGPSAGSAAGVPGGTGGLSAVGSGPRLRLLLLESVSGLLQPRAGSAVAPVHPPVRSAPHLPGLMCLLRLHGTVG
GAQNLSAVGALVGLSNARLGSIKTRFEGLCLLSLLVGESPTEMFQQHCVSWLRSIQQVLQSQDPPPTMELAVAVLRDLLR
YAAQLPTLFRDISMNHLPGLLTSLLGLRPECELSALEGMKACMTYFPRACGSLRGKLASFFLSRVDALSPQLQQLACECY
SRLPSLGAGFSQGLKHTESWQQELRSLLASLHSLLGALYEGAETAPVQYEGPGVEVLLTPSEDGDAHALLRLRQRFCGLA
RCLGLMLSSEFGAPVSVPVQEILDVICRTLSISAKNISLLGDGPLRLLLLPSIHLDALDLLSALILACGGRLLRFGGLIS
RLLPQVLNAWNLGRDTLSPGQERPYSTVRTKVYAVLELWVQVCGASAGVLQGGASGEALLSHLLSDISPPADALKLRSPR
GSPDGGLQTGKPSAPKKLKLDLGEAMAPPSHRKGDSNANSDVCAAALRGLSRTVLMCGPLIKEETHRRLHDLVVPLVMGL
QRGETLGSSPYTSSRCRQELYRLLLALLLAPSPRCPPPLACALQAFSLGQREDSLEVSSFCSEALVVCAALTHPRVPPLQ
PTGPTCPTPAPVPPPEAPSPFRAPPFHPPAGPPPPARPGPPAAANHLGLSVPGLVSVPPRLLPGPENHRAGSNEDPVLAP
GGSPPPALPPDETFGGRVPRPAFVHYDKEEASDVEISLESDSDDSVVIVPEGLPPLPPAPPSGATPPPAAPAGPPAASPP
VPAKEEPEELPAAPGPLPPPPPPPAPGPVALPPPQLVPEGTPGGGGPPSLEEDLTVININSSDEEEEEEEEEEEEEDFEEEE
EEEEYFEEEEEEEFEEEFEEEEEEGELEDDDEDDEDDDDELEELDEADFGPAGGPAEAGGPAPPSPAPAPPPASSPGVRP
APEAEPGLLLEVEEPAADEDDDDDEDDEDDDEEEEEEEEEDGGEGPGARAAPTLAPEVLPSQGEGPQAAGSPPAGPPPPEL
VEEPPSAPPTLLEEGTDSGGDGVPPPPPETPAAEEAAAEAEAETAALQEKEQDDTAAMLADFIDCPPDDEKPPPAPEPDS
>Neotoma lepida [A0A1A6G328]
MAAAVLSGPSAGSPAGAPGGTGGLSTVGSGPRLRLLLLESVSGLLQPRTGSPVAPVHPPIHWAPHLPGLMCLLRLHGTVG
GAQNLSALGALVNLSNAHLGSIKTRFEGLCLLSLLVGESPTELFQQHCVSWLRSIQQVLQDPPPTMELAVAILRDLLRYA
SXLPTLFRDISTNHLPGLLTSLLGLRPECEQSALEGMKACVTYFPRACGSLKGKLASFFLSRLDALNPQLQQLACECYSR
LPSLGAGFSQGLKHTENWEQELHSLLASLHSLLGTLYEGAETEDGNTHVLLQLRQRFSGLARCLGLMLSSEFGAPVSVPV
QEILDLICRILSISSKNINLLGDGPLRLLLLPSIHLEALDLLSAXILACGGKLLRFGALISRLLPQVLSTWSTGRDTLAP
GQERPYSTIRTKVYAILELWVQVCGASAGMLQGGASGEALLTHLLSDISPPADALKLCGTRGSSDGGLQSGKPSAPKKLK
LDVGEALAPSSHRKGDRNANSDRLHDLVLPLVMSVQQGEVLGGSPYNNSCCRRELYRLLLALLLAPSPRCPPPLACALKA
FSLGQGEDSLEVSSFCSEALVTCAALTHPRVPPLQSSGPACPTPAPVPPPEAPSPFRAPPFHPPGPMPSIGAMPSPGPIP
SAGPIPTVGPVSSAGPIPSTGPVPSRPGPPATANHLGLSVPGLVSVPPRLLPGPENHRAGSSEDPVLAPSGTPPPSIPPD
ETFGGRVPRPAFVHYDKEEASDVEISLESDSDDSVVIVPEGLPSLPPPPPSGSPTPVAPTGPPTASPPVPAKEDSEELPA
TPGPLPPPPPVSYTHLDVYKRQPTAMEEDLTVININSSDEEEEEEEEEEEEEDEDEEEEEDFEEEEEDEEEYFEEEEEEEF
EEEFEEEEGELEEEEEEEEEEEELEEVEDVEFGSVGGEVEEGGPPPPTLPPALPPTDSPKVQPEAEPEPGLLLEVEEPGAE
EVPGPETAPTLAPEVLPSQEELEQEGGSPTAGPPQELVEEESSAPPPLLEEGTEGGGDKVPPPPETPAEEEMEAEAEAST
PQEKEQDDTAAMLADFIDCPPDDEKPPPATEPDS
> Ailuropoda melanoleuca [A0A7N5KN65]
VHFVQRRVPFSDSTRGVFVGTRVMSAVRQVQGPRGSVAFQGVEGLLREAPPAPAPGCPTWDPLRSLLPCCPHLTPSPHSI
LGVTELRGSGGGVCSGPASALARWPASSGARLPLRVVTGYFLVRFEGLCLLSLLVGESPTEMFQQHCVSWLRSIQQVLQS
QDPPPTMELAVAVLRDLLRYAAQLPTLFRDISMNHLPGLLTSLLGLRPECELSALEGMKACMTYFPRACGSLKGKLASFF

LSRVDALSPQLQQLACECYSRLPSLGAGFSQGLKHTESWEQELHCLLASLHSLLGTLYEGAETAPVQYEGPGVEVLLTPS
EDGDAHVLLRLRQRFCGLARCLALMLSSEFGAPVSVPVQEILDVICRTLSISAKNISLLGDGPLRLLLLPSIHLDALDLL
SALILACGGRLLRFGALISRLLPQVLNAWNLGRDTLSPGQERPYSTVRTKVYAVLELWVQVCGASAGVLQGGASGEALLS
HLLSDISPPADALKLRSPRGSPDGGLQAGKPSAPKKLKLDMGEAVAPPSHRKGDSNANSDVCAAALRGLSRTVLMCGPLI
KEETHRRLHDLVLPLVMGVQQGEVLGSSPYTSSHCRRELYRLLLALLLAPSPRCPPPLACALQAFSLGQREDSLEVSSFC
SEALVTCAALTHPRVPPLQSMGPACPTPAPVPPPEAPSPFRAPPFHTPGPMPSVGPMPSVGPMPSAGPMPSAGSMASAGP
MPPTRPGPPATANHLGLSVPGLVSVPPRLLPGPENHRAGSNEDPVLAPSGTPPPPIPPDETFGGRAPRPAFVHYDKEEAS
DVEISLESDSDDSVVIVPEGLPSLPPPPPSGTTPPPVAPAGPPTASPPVAAKEEPEELPAALAGPGVRARGARCARGARC
ARAGGARCAHTRRRVRPSLQRPQRGCVTTEDKSLASSHGICSFSRH
> Ailuropoda melanoleuca [A0A7N5P5V1]
VHFVQRRVPFSDSTRGVFVGTRVMSAVRQVQGPRGSVAFQGVEGLLREGGGEPVSGPHSILGVTDALARWPASSGARLPL
RVVTGYFLVRFEGLCLLSLLVGESPTEMFQQHCVSWLRSIQQVLQSQDPPPTMELAVAVLRDLLRYAAQLPTLFRDISMN
HLPGLLTSLLGLRPECELSALEGMKACMTYFPRACGSLKGKLASFFLSRVDALSPQLQQLACECYSRLPSLGAGFSQGLK
HTESWEQELHCLLASLHSLLGTLYEGAETAPVQYEGPGVEVLLTPSEDGDAHVLLRLRQRFCGLARCLALMLSSEFGAPV
SVPVQEILDVICRTLSISAKNISLLGDGPLRLLLLPSIHLDALDLLSALILACGGRLLRFGALISRLLPQVLNAWNLGRD
TLSPGQERPYSTVRTKVYAVLELWVQVCGASAGVLQGGASGEALLSHLLSDISPPADALKLRSPRGSPDGGLQAGKPSAP
KKLKLDMGEAVAPPSHRKGDSNANSDVCAAALRGLSRTVLMCGPLIKEETHRRLHDLVLPLVMGVQQGEVLGSSPYTSSH
CRRELYRLLLALLLAPSPRCPPPLACALQAFSLGQREDSLEVSSFCSEALVTCAALTHPRVPPLQSMGPACPTPAPVPPP
EAPSPFRAPPFHTPGPMPSVGPMPSVGPMPSAATANHLGLSVPGLVSVPPRLLPGPENHRAGSNEDPVLAPSGTPPPPIP
PDETFGGRAPRPAFVHYDKEEASDVEISLESDSDDSVVIVPEGLPSLPPPPPSGTTPPPVAPAGPPTASPPVAAKEEPEE
LPAALAGPGVRARGARCARGARCARAGGARCAHTRRRVRPSLQRPQRGCVTTEDKSLASSHGICSFSRH
> Cricetulus griseus [G3GYB0]
MYGDGNQGSATGSCSCGKTIAPGTLVPPATLTHIRKHLKAYDRCPFLIRFHLKSTSVCGRKQDQWVREVISCFENKECGH
GHGKSLPHQEPLPHASTQIPVTTEGTPPDTSTPAQIQSTHQSTFPSGALSLNKELTPHSETTAVTSGYETTAVTSGYETT
AVTSGCDPEARPKAKANKKQQENKQQKEKPESSAGTPGLVPVLSLLVIVFFLTAAVVYMLCTRRRVRQQRSAGESPTELF
QQHCVSWLRSIQQVLQSQDSPSTMELAVAILRDLLRYASQLPTLFRDISTNHLPGLLTSLLGLRPECEQSALEGMKACVT
YFPRACGSLKGKLASFFLSRLDALNPQLQQLRQRFSGLARCLGLMLSSEFGAPVSVPVQEILDLICRIRSISSKNINLLG
DGPLRLLLLPSIHLEALDLLSALILACGGRLLRFGALIIRLLPQVLNTWSTGRDTLAPGQERPYSTIRTKVYAILELWVQ
VCGSSAGMLQGGASGEALLTHLLSDISPPTDALKLCATRGSSDGGLPSGKPSAPKKLKLDMGEALAPASHRKGDRNANSD
VCAAALREEEEEEEEEEEEDEDEEEEDFEEEEEDEEEYFEEEEEEEFEEEFEEEEGELEEEEEEEEEEEEELEEVEDVEF
GSAGGEVEEGGPPPPTLPPALPPTDSPKIQPEAEPEPGLLLEVEEPGAEEVPGPETAPPLAPEVLPPQEEVVQEGGSPPA
GPPQELVEEESSAPPNLLEEGTEGGGDKVPPPPETPAETEMETEAEAASPQEKEQDDTAAMLADFIDCPPDDEKPPPDPE
PDS
> Lepisosteus oculatus [W5N9Q9]
MAASAWVDGPTSARLAEGLLSALQEEQPGHLPGLLASYREHGGVSAQSAGVVGNLIGFSNARLSSTKTRFQGLCLLSVLV
RDSSSDVFQQHCLSWLRAVQQVIQSQAPLPSVQLAVTILQDLLQYSSQLPELSREIGLNSIPGILTSLLGLKQECHLGAM
EGMTACMTYYPRACGSLREKLGAYFLSKMDSDNPKIQEMACACYGRLSSLGGVFERGSRQAEGWAQHLHCLLATAHGVLG
QLYEGVEAEGGVQYEGPGIELLLPPLDDTDPLLVLQLRHRYRAVTLALTHTLSVDLSSPVRLPVQSVLNLVCRALAVTAK
GISVSGDGCLKLLVLPSLHRDTLELLSTLITVAGSRLVQYSSVLTHITPHQATSKYLCQRCNPSPCFNTHSAVRVALYRS
LDLWVRVGGAAASVLQQSPTHSELLLAHLLGDITPGADSIKLRPGRTGMADLAGHPGKAGGKRGKGLDLGEAVGAGITLQ
RKSDVLANQDTCLSALRALRQIVLTSGTLLKEDTHKRLQELALPLCVRLQQCGGAAEAGGLYASPLARRALYQLVLALVL
VPSPRWPPPLHCAVRIFSQGLNDPSVQVSSFCSEALTVCNSLLHPRTPSIALPLPSLALKSSPAPPAVPTTATASAPQAA
LSLPSLLGTPAQGPASFTARHPIGLAQGLLGGPLDNHLPLQPPALPQSATHQQHPPAPQPHLPPPAGDLLSTPQLGDPGA
LGGPEGHRPVFVRYDKEEPEDVEISLESDSDDSVVIVPEGMLQNKQELTQTVPGSAMGPAAVGSGTRRLGEEGGAEAGVH
SPLANELPAHQILPPNSAVMNAFPSQNQTQVSGLVTPPALQAPAGSLGESLPPAREEDLTVININSSDEEEEEDELEDEE
EEEEEGLEDLEEEDEEGSEFAEEEEEEEYYGEEEEFEEEGEFEEEEEEGVMEGEEIEEEEEEEEGVFELERERDGEEGDE
RERVDVEAYKQPLDLEVMKEGEGLEGEGRVLEEVNGDGGGREEEREAFPPQEKAESSPSQGTVGQEMQGKEAQPKVEGVE
VPGQELTQELGTAQEILPSQAEEKATSSQDQDLEPVQEEDVSERQERAGPEEEALGQEPAEDSMASMLADFVDCPPDDEE
EPPKLDT
> Lepisosteus oculatus [W5N9Q7]
MAASAWVDGPTSARLAEGLLSALQEEQPGHLPGLLASYREHGGVSAQSAGVVGNLIGFSNARLSSTKTRFQGLCLLSVLV
RDSSSDVFQQHCLSWLRAVQQVIQSQAPLPSVQLAVTILQDLLQYSSQLPELSREIGLNSIPGILTSLLGLKQECHLGAM
EGMTACMTYYPRACGSLREKLGAYFLSKMDSDNPKIQEMACACYGRLSSLGGVFERGSRQAEGWAQHLHCLLATAHGVLG
QLYEGVEAEGGVQYEGPGIELLLPPLDDTDPLLVLQLRHRYRAVTLALTHTLSVDLSSPVRLPVQSVLNLVCRALAVTAK
GISVSGDGCLKLLVLPSLHRDTLELLSTLITVAGSRLVQYSSVLTRLFSQTLSAWTPLPEASPGQQRAYSAVRVALYRSL
DLWVRVGGAAASVLQQSPTHSELLLAHLLGDITPGADSIKLRPGRTGMADLAGHPGKAGGKRGKGLDLGEAVGAGITLQR
KSDVLANQDTCLSALRALRQIVLTSGTLLKEDTHKRLQELALPLCVRLQQCGGAAEAGGLYASPLARRALYQLVLALVLV
PSPRWPPPLHCAVRIFSQGLNDPSVQVSSFCSEALTVCNSLLHPRTPSIALPLPSLALKSSPAPPAVPTTATASAPQAAL
SLPSLLGTPAQGPASFTARHPIGLAQGLLGGPLDNHLPLQPPALPQSATHQQHPPAPQPHLPPPAGDLLSTPQLGDPGAL
GGPEGHRPVFVRYDKEEPEDVEISLESDSDDSVVIVPEGMLQNKQELTQTVPGSAMGPAAVGSGTRRLGEEGGAEAGVHS
PLANELPAHQILPPNSAVMNAFPSQNQTQVSGLVTPPALQAPAGSLGESLPPAQLQQMLMQPPQPAALSLSMQMQLVQTP
RLQQQQQQQQQGEEDLTVININSSDEEEEEDELEDEEEEEEEEEGLEDLEEEDEEGSEFAEEEEEEEYYGEEEEFEEEGE
FEEEEEEGVMEGEEIEEEEEEEEGEVLPSEEGAVMMDPRGEREGLTGVFELERERDGEEGDERERVDVEAYKQPLDLEVM
KEGEGLEGEGRVLEEVNGDGGGREEEREAFPPQEKAESSPSQGTVGQEMQGKEAQPKVEGVEVPGQELTQELGTAQEILP
SQAEEKATSSQDQDLEPVQEVKSSLELKGEELSQGELAAKLQEDVSERQERAGPEEEALGQEVRSSAPEEEIGCARERDI
EEHAEERQGKRKREEEEEEDLTEQSAEKKKPAEDSMASMLADFVDCPPDDEEEPPKLDT
> Takifugu rubripes [A0A674N5N0]
MATSAWLRGPSAMRLTEGLVSVLKEQRPDYIPVLLTGYREHGVFHTQGASAVAGLVGFSNAKLGSSKTRFEGLCLLSMLV
KDSSSDLFQQHCLSWLRSLQQIVQSQAPVQTIQLAVNILKDVLQYSSQIPELAREVGLNSILGILTSLLGLKTECELAAM
EGMTACMTHYPRACGSLKDKLGAYFLSKMDSTNKKTQEMACQCYAHLPCLGGVLDRGAGAGRAEGWTNQIHCLLASANSH
LALIYQGAEMEGTMQYEGPGVELAFPLLDQSDPLFLLQLQHRFTAVCLALRHTLRVDPASAVRIPVRPILNLVCRALAVS
CKSFNLTGDGNVRLLILPIIHLNILEVLAALIIAVRSSMVQYAAVLQRLFSQTLSAWTPAAEASVGQQRAFSSVRVSVYR
TLELWLQVAGASTSILHGSPNHSEILFNHLLSDITPGAESVKLRVGLSAEIVPGGKPGPRRTKSLVISDTVGPSLQRKGD
IMANQDTCLTALRALRQIILVSGTLLKDDIHKRLHDVLLPLCVRLQQQLSSNMSCDSTAGISGQYSSALTRRELYRLLL
ALVLVPSPCWPPPLTCVVSILSSGRTDRNLKVSTFCCEALTVCNSLLHPRSPSIALPMPPLSIKPAHAVSVLPTPQASTP
GLTLPTLLGEPTPPPPFPSPHTLGMGPSSLLGSLENHLSLVPGLRGQTSGPSEMILSPHAHHQDLAGLGPPEGQRPVFVR
YDREEAEDVEISLASDSDDSVVIVPPGMLNMENQQDDVAAANSQSMTGEPVTMVPNTATAAPIDGVSLPNDLTTSAPLLT

TSAPPINSFPPSSASVVSLVPALNSNPLTAPPGGLVEPIPSRPQLQQMLMQPSAAVQQGPLSLPLQIHQLQSQLGQQGRP
LQQQPPVASNEDSGVININSTDDEEDDEDMEDDEEEEEEEGVEDEEEEEDEVSSFADEEFFEDYEEFEYDAEELEEEDEE
EGDIPPLEGAEEQGEQEEVEQGGVLQAAVEAAEIVDFSVEGEAGGGIEEIQTKRALFPEDRMKVQEVESIGVMEEARGEA
EEDETERVCDPTMPQILCVTGGALEEKEEEGGRVQEDMSSWERDAKKVEPQGPSGEAAASTNSMPQDESNTQKKLLLVSF
PSQADEDTMASMLADFVACPPDDDDGPSASNQS
> Greater amberjack [A0A3B4TMI1]
MATSAWLHGPSAMRLTEGLVSVLKEHRPEYLPALLANYREHGVFPTQSASAVGGLVGFSNAKLGSSKTRFEGLCLLSMLV
KDSSSDLFEQHCLSWLRSLQQVIQSQAPVQTIQLAVNILKDLLQYSSQLAELAREVGLNSILGILTSLLGLKTECELSAM
EGMTACMTYYPRACGSLRDKLGAYFLSKMDSSNKKTQEMACQCYGRLPCLGGLLDRGVGAGRAEGWTNQIHCLLASANGL
LTQIYQGSETDGAVQYEGPGVELAFPHLDQSDPLLLLQLQHRYTAVCLALKHTLRVDPASAVRLPVRPILNLVCRALAVS
SKSINLTGDGSVRLLVLPIIHTNTLDILSALITAVRSSMVQYTVVLQRLFSQTLSAWTPVHEASLGQQRAYSSVRVSVYR
TLELWVQVAGASASILQGSPGHSELLFNHLLGDITPGAESIKLRAGLSADAVPGGKPGPRRTKPLVMADAVGPSLQRKGD
FLANQDTCLSALRALRQIILTGGTLLKDDIHKRLHDVVLPLCVRLQQQQSSSNAACESAGGVSGQYSSALTRRELYRLLL
ALVLVPSPCWPPPLTCAVSILSSGRADRNLKVSTFCAEALTICNSLLHPRTPSIALPLPPLTLKPTPTAPVLPSSQGPTP
GLTLPTLLGGTAPGPPFPSRHSIGLGPASLLGSLENHLSLVPGLPGQAHTPGDMILSPHTHHLPDQAGLGPPEGQRPVFI
RYDKEEAEDVEISLASDSDDSVVIVPPGMLNMENQQDDAAAANSQNLVSAAPGGTTVSLPGGECVTMVPTTAVPTTIDSV
SLSNNLATSSPLLTTSTTPINSFPPSSTSVVSLVPPLNSSTLTAPPGGLGDSMPGRPQLQQMLMQPSTAGQPGPMGLPLQ
MHQLQNQLSQQGRHLHQHQPPPASNEDSAVININSTDEEEEEEEEDMEDDEELEEEEEGMDEEDEEEEVSDFAEEEFYDG
EEYEDYDEEEGEELEEEEEEEEDGDIPPLEGAEDKAGEVGVEEGKVVQAAVDEEGLAGFSVEGDEEGGIEEIQPNRVLFPE
DRMKVQEVESIGVLEEARGGEGEEDESERMDDPTMPQILCVTGGAMEEREETEEGGAAGGGAGAGEGGGAVQEEASLWEQ
GASEAELRTSSEERTTNQSQQESRPEPTQEASVGENQPCSGQEEQQQQQPSAAPEGDSAAADPDSSATLKPKEPVETSSE
NRDRGEAEQQETEGGGGGESDGEEGKGVKRKREEVHREEEAGQSTEKKKMDDEAMASMLADFVACPPDDEDGASGSTRS
> Salmo salar [A0A1S3N8I3]
MAATAAWMHGPANMRLTEGLVSVLKEQRPEYLPALLANYREHGVVSTQSSAAVGGLVGVSNAKLGNSKTRFEGLCLLSVL
VKDSSSDVFQQHCLSWLRSLQQVIQSQAPLPSIQLAVGVLQDLLQYSSQLPELAREVGLNSILGILTSLLGLKSEFHLAA
MEGMTACMTFYPRACGSLRDKLGACFLSKMDSVNPEVQEAACECYGRLPCLGGVLERGGGGRPAEGWTNQLHCLLASANG
MLAQLYQGTESGMVPYEGPGVELPYPPLDDTDPLVLLQLQHRYRGVCLALKHTLSVDPATAVRLPVQQVLNLVCRALAVS
SKSINLTGDGSVRLLVLPSIHNHTVKVLQALITAVGSGLVQYSNMLQRLFSQTLSAWTPLPETSLGQQRAFSAVRVSLYQ
TLELWVKVGGASAGVLQGSPTHSEILLAHLLGDITPGADSVRLRAGQSTVADLVSSKPCPKSRKPGLGMGNGGGASLQRK
GDSLANQDTCVSALRALRQIILTSGTLLKEDVHKRLHDVVLPLCVRLQQQHGGDCGTGAVSGQYGSALPRRELYHLLLAL
VLVPPPRWPPPLTCTVSILSHGRRDRSLKVSSFCTEALAICNSLLHPRTPSLCLPLPPLTLKPSPAASLLTPSQASSLTL
PTLLGGPFPGRHSLGLGHTLLGSLDNHLSLVPPGLSGQGSTPGDLLLSPHQGELAGLGLSEGQRPVFIRYDKEEAEDVEI
SLESDSDDSVVIFPRGMLMLENQDGTSTVANLPVSSLVPGGVTLPVPGPGDDPGGDISLPLANDLPSTSLPHPLLPSSSA
PNSINSFPPAPLASLVPPLNSTGVTQLGAPSVGLGVGADSLPGAQLQQMLLQGQPPAPGQPTPLGLPIQMLQNQLAQPSR
ALQQQQASEEDHSVININSSDEEEEEDEEMEDEDELGEEEDEEGLEDEEEEEEEGSDFPDEEEEFYGEEFDDYEEEEGEEM
EEEEEEEEEEEEEGEIRPLDREGRRGGMGREEGEVLREVPEDGGMGGFCVEGEMEGGIEELGTNRRVYGEEGVKAQEVES
IGVLEEEREGEEDDADGMNDPTMPQILCVTGGALEEREELGEGHGQEVGSYEQQGADRPEATPSSEGPAPQHKQEAEPAQ
EVRVGGSDQPSNQGEEPAKQGGDSAKEEVKPSAAPSETEGEEPEREKGGEEGEEDGEEDGRGMKRKREGEEEGTGQGTEK
KKLDEEVMASMLADFVACPPDDEEKGPSASNLPS
> Takifugu rubripes [H2SWC2]
MATSAWLRGPSAMRLTEGLVSVLKEQRPDYIPVLLTGYREHGVFHTQGASAVAGLVGFSNAKLGSSKTRFEGLCLLSMLV
KDSSSDLFQQHCLSWLRSLQQIVQSQAPVQTIQLAVNILKDVLQYSSQIPELAREVGLNSILGILTSLLGLKTECELAAM
EGMTACMTHYPRACGSLKDKLGAYFLSKMDSTNKKTQEMACQCYAHLPCLGGVLDRGAGAGRAEGWTNQIHCLLASANSH
LALIYQGAEMEGTMQYEGPGVELAFPLLDQSDPLFLLQLQHRFTAVCLALRHTLRVDPASAVRIPVRPILNLVCRALAVS
CKSFNLTGDGNVRLLILPIIHLNILEVLAALIIAVRSSMVQYAAVLQRLFSQTLSAWTPAAEASVGQQRAFSSVRVSVYR
TLELWLQVAGASTSILHGSPNHSEILFNHLLSDITPGAESVKLRVGLSAEIVPGGKPGPRRTKSLVISDTVGPSLQRKGD
IMANQDTCLTALRALRQIILVSGTLLKDDIHKRLHDVLLPLCVRLQQQQLSSNMSCDSTAGISGQYSSALTRRELYRLLL
ALVLVPSPCWPPPLTCVVSILSSGRTDRNLKVSTFCCEALTVCNSLLHPRSPSIALPMPPLSIKPAHAVSVLPTPQASTP
GLTLPTLLGEPTPPPPFPSPHTLGMGPSSLLGSLENHLSLVPGLRGQTSGPSEMILSPHAHHQDLAGLGPPEGQRPVFVR
YDREEAEDVEISLASDSDDSVVIVPPGMLNMENQQDDVAAANSQSMTAAAGGAAVTLAEGEPVTMVPNTATAAPIDGVSL
PNDLTTSAPLLTTSAPPINSFPPSSASVVSLVPALNSNPLTAPPGGLVEPIPSRPQLQQMLMQPSAAVQQGPLSLPLQIH
QLQSQLGQQGRPLQQQPPVASNEDSGVININSTDDEEDDEDMEDDEEEEEEEGVEDEEEEEDEVSSFADEEFFEDYEEFE
YDAEELEEEDEEEGDIPPLEGAEEQGEQEEVEQGGVLQAAVEAAEIVDFSVEGEAGGGIEEIQTKRALFPEDRMKVQEVE
SIGVMEEARGEAEEDETERVCDPTMPQILCVTGGALEEKEEEGGRVQEDMSSWERDAKKVEPQGPSGEAAASTAEQEESG
GEQQAGSSEERPSSLGEEQEVTVSEEDAALTHPGGSDESTPEPQETVTEKREAAAAAEQQELHAGGGEEGKGVKRKREEL
QSENELEGSSKQADEDTMASMLADFVACPPDDDDGPSASNQS
> Hucho hucho [A0A4W5MDN7]
MAATAAWMHGPANMRLTEGLVSVLKEQRPEYLPALLANYREHGVVSTQSSAAVGSLVGLSNAKLGNSKTRFEGLCLLSVL
VKDSSSDVFQQHCLSWLRSLQQVIQSQAPLQSVQLAVGVLQDLLQYSSQLPELAREVGLNSILGILTSLLGLKSECHLAA
MEGMTACMTFYPRACGSLRDKLGACFLSKMDSVNPEVQEVACECYGRLPCLGGVLERGGGGRRAEGWTNQLHCLLASANG
MLAQLYQGTELEGMVPYEGPGVELPYPPLDDTDPLVLLQLQHRYRGVCLALKHTLGVDPASAVRLPVQQVLNLVCRALAV
SSKSINLTGDGSVRLLVLPSIHNHTVQVLHALITAVGSGLVQYSSMLQRLFSQTLSAWTPLPETNLGQQRAFSAVRVSLY
RTLELWVKVGGASAGVLQGSPTHSEILLAHLLGDITPGADSVRLRAGQSTVADLVSSKPCPKSRKPGLGMGNGGGASLQR
KGDSLANQDTCVSALRALRQIILTSGTLLKEDVHKRLHDVVLPLCVRLQQQHGGDCGAVGVSGQYGSALPRRELYRLLLA
LVLVPPSRWPPPLTCTVSILSHGRRDRSLKVSSFCTEALTICNSLLHPRTPSLCLPLPPLTLKPSPATSLLTPSQASSLT
LPTLLGGPFPGRHSLGLGHTLLGSLDNHLSLVPPGLSGQGSTPGDLLLSPHQGELAGLGLSEGQRPVFIRYDKEEAEDVE
ISLESDSDDSVVIFPRGMLMLENQDGTSTVATLPVSSLVPGGVTLPVPGPGDDPGGDISLPLANDLPSTSLPHPLLPSSS
APNSINSFPPAPLASLVPPLNSTVTQLGAPSVGLGVGADSLPGAQLQQMLLQGQPPAPGQPTPLGLPIQMLQNQLGQPSR
ALQQQQASEEDHSVININSSDEEEEEDEEMEDEDELGEEEDEEGLEDEEEEEEEGSDFPDEEEEFYGEEFDDYEEEEGEEM
EEEEEEEEEGEIRPLDREGRRGGMGREEGEVLREVPEDGGMGGFCVEGEMEGGIEELETNRRVYGEEGVKAQEVESIGVL
EEEREGEDDDADGMNDPTMPQILCVTGGALEEREELGEGHGHEVGSCEQQGADRPEATPSSEGPAPQHKQEAEPAPEVRV
GGNDQPSNQGEEPAKQGGDSAKEEVKPSAAPSETEGEEPEREKGGEEGEEDGEVDGRGMKRKREGEGEEEGSGQGTEKKK
LDEEVMASMLADFVACPPDDEEKGPSASNLPS
>Salmo salar [A0A1S3N8Q5]
MAATAAWMHGPANMRLTEGLVSVLKEQRPEYLPALLANYREHGVVSTQSSAAVGGLVGVSNAKLGNSKTRFEGLCLLSVL
VKDSSSDVFQQHCLSWLRSLQQVIQSQAPLPSIQLAVGVLQDLLQYSSQLPELAREVGLNSILGILTSLLGLKSEFHLAA
MEGMTACMTFYPRACGSLRDKLGACFLSKMDSVNPEVQEAACECYGRLPCLGGVLERGGGGRPAEGWTNQLHCLLASANG

MLAQLYQGTESEGMVPYEGPGVELPYPPLDDTDPLVLLQLQHRYRGVCLALKHTLSVDPATAVRLPVQQVLNLVCRALAV
SSKSINLTGDGSVRLLVLPSIHNHTVKVLQALITAVGSGLVQYSNMLQRLFSQTLSAWTPLPETSLGQQRAFSAVRVSLY
QTLELWVKVGGASAGVLQGSPTHSEILLAHLLGDITPGADSVRLRAGQSTVADLVSSKPCPKSRKPGLGMGNGGGASLQR
KGDSLANQDTCVSALRALRQIILTSGTLLKEDVHKRLHDVVLPLCVRLQQQHGGDCGTGAVSGQYGSALPRRELYHLLLA
LVLVPPPRWPPPLTCTVSILSHGRRDRSLKVSSFCTEALAICNSLLHPRTPSLCLPLPPLTLKPSPAASLLTPSQASSLT
LPTLLGGPFPGRHSLGLGHTLLGSLDNHLSLVPPGLSGQGSTPGDLLLSPHQGELAGLGLSEGQRPVFIRYDKEEAEDVE
ISLESDSDDSVVIFPRGMLMLENQDGTSTVANLPVSSLVPGGVTLPVPGPGDDPGGDISLPLANDLPSTSLPHPLLPSSS
APNSINSFPPAPLASLVPPLNSTGVTQLGAPSVGLGVGADSLPGAQLQQMLLQGQPPAPGQPTPLGLPIQMLQNQLAQPS
RALQQQQASEEDHSVININSSDEEEEEDEEMEDEDELGEEEDEEGLEDEEEEEEGSDFPDEEEEFYGEEFDDYEEEEGEE
MEEEEEEEEEEEEEGEIRPLDREGRRGGMGREEGEVLREVPEDGGMGGFCVEGEMEGGIEELGTNRRVYGEEGVKAQEVE
SIGVLEEEREGEEDDADGMNDPTMPQILCVTGGALEEREELGEGHGQEVGSYEQQGADRPEATPSSEGPAPQHKQEAEPA
QEVRVGGSDQPSNQGEEPAKQGGDSAKEEVKPSAAPSETEGEEPEREKGGEEGEEDGEEDGRGMKRKREGEEEGTGQGTE
KKKLDEEVMASMLADFVACPPDDEEKGPSASNLPS
> Chanos chanos [A0A6J2VXJ3]
MATAAWLHGPNNMRLTEGLVSALKEDRPEYLPALLANYREHGVVGIQSTGTVGGLVGLSNARLGSSKTRFEGLCLLSVLV
KDSSSEVFQQHCLSWLRSLQQVIQSQAPLPSVQLAVSVLQDVLQYSSQLPELAREVGLNSILGILTSLLGLKTECHLAAM
EGMMACMTYYPKACGSMREKLGAYFLSKMDSDNPKVQEIASECYGRLPCLGGVLERGGGGRRAEGWTNQLHCLLASANSL
LGQLYQGAESEGTVQYEGPGVELPFSPLDDMDPLLVLQLQHRYRAVCLALKHTLSVDPACPVRLPVQNVLNLVCRALAVS
GKTINVTGDGCLKLLVLPSTHIDTLDLLSALIKAVGRGLVQYCSVLTRLFSQSLSVWTPLPEASLGQQRAYSAVRVALYR
TLELWVRVGGASTSVLQGSTTHSEILFAHLMGDITPGAEAVKLRAAQSAMSDLVGPAGKAGPRRTKGLGIGDTGGVSLQR
KGDALANQDTCLSALRALRQIVLTSGTLLKEDLHKRLQDLVVPLCVRLQQQQAHGGCDAGAVSGQYGSAAPRRELYRLLL
AMVLVPSPRWPPPLSCAVSVFSNGRRDRAITVSSFCAEALTICNTLLHPRSPSISLPLPPLALKPTPSAPIHAPSQNPSL
SLPPLLGAPTPGHPFPARHPLSIGPAALLGSLENHLPLAPSVLPTPAGPTASPGDLLLSSAQPSELAGVGAPEGQRQVFV
RYDKEEAEDVEISLESDSDDSVVIVPPGMLMMEAQDGAANAQTLPPPPTGGTVPVTTAGPGGDAGVGETSLPNDLPTSIT
HQILPSNDNNINSFPGPSQAAQLVSLVPPLNSTVTPLTAPSGGLGESLSGSAQLQQMLMQPPPGGQPTQLGLPIQIQLQS
QMAPSTRQIQPQQPQGPNDEDQNVININSSDDEDEEDEEMEDEDELGEEEEEEEGLEDEEEEEDGSDYPEEEEYYDAEEY
DDYEEEEGEELEEEEEEESEEIPPLEGENRRGLIGGEEGEVMIRGEGEVERGMGMFCVERERQPEGGTEEMEAGGAVYTE
ETVKEQDEGNAEERENSGMGERDGGVASEQLMEAQLIGGPTEQREQETSEQVAEQEVSSWEQEGAVSEGMPVSTESSVLC
QGQDVPTEVIGHEQGPETEPEDQPKQSAEPNREEAASQHVEAAKEQEEVRDENGEGEDGRGTKRKMEDRGEEETEQQSSE
KKKLDDEAMASMLADFVDCPPDEEEHGTSQSHS
> Makobe Island cichlid [A0A3B4GL05]
MATSAWLHGPSAMRLTEGLVSVLKEQRPEHLPALLAGYREHGVFQTQSACAVGGLVGFSNAKLGSSKTRFEGLCLLSMLV
KDSSSDLFQQHCLSWLRSLQQVIQSQAPVETVQLAVNVLKDLLQYSSQLAELAREVGLNSILGILTSLLGLKSECELAAM
EGMTACMTYYPRACGSLRDKLAAYFLSKMDSTDRKTQEMACQCYGRLPCLGGVLDRGVGAGRAEGWTNQIHCLLASANGL
LAQLYQGSETDGAMQYQGPGMELAFPHLDPTDPLLLLQLQHRYTAVCLALKHTLRVDPASAVRLPVRPMLNLVCRALAVS
SKSINLTGDGSVRLLVLPIIHSNTLEVLSSVITVVRVGMVQYAAVIQRLFSQTLSAWTPLPETSVGQQRAYSAVRVSVYR
TLELWVQVAGASSSILQGSPGHSELLFNHLLGDITPGAESVKLRAGLSADVVPGGKPGPRRTKHLVMADTVGPSLQRKGD
HLANQDTCLAAVRALRQIIQTSGTLLKDDIHKRLHEVVLPLCVRLQQQQSCSSNACESAGSASGQYSSALTRRELYRLLL
ALVLVPSPCWPPPLTCAVSILSNGRTDRNVKVSSFCAEALTICNLILHPRFPSIALPLPPLTMKPSSTTPVLPSSQGPAP
GLTLPNLLGGPAPGPPFPARHSLNLGPASLLSSFENHLSLVPGLPGQGPTAGDMILSPHSHHQPDPAGLGPPDGQRPVFV
RFDKEEAEDVEISLASDSDDSVVIVPPGMLNMETQQDDAAANSQSMSSSAPGGTGVTLAGAESVTMVPTTAAAATIDGVS
LPNDLAASSPLLTASTTSINSFPPSSASVVSLVPPLNSSTFTAPPVGLGESLPGRPQLQQMLMQPSTASQAGPMGLPLQM
HLQNQLSQQGRHLHQQPAAAASSEDSAVININSTDDEEEDDEDIDEDEELEEDEEEEGIDEEDEEEDVSELADEFFDGEE
YEELDEEDGEDLEEEEEEEEEEEDGDIPPLEGVEDKDGGSGIQEGKLDEERISAFNVEERTEG
> Red-bellied piranha [A0A3B4CPL2]
MATAAWLHGPNNTRLTEGLLSVLKEERPEYLPALLANYREHGVVGSQNSGAVGGLVGISNLRLGSSKTRFEGLCLLSVLV
KDSSSEVFHQHCLSWLRSLQQIIQSQAPLPSVQLAVSVLQDLLQYSSQLPELAREVGLNSILGILTSLLGLKSECHLAAM
EGMMACMRYYPRACGSLKEKLGVYFLSKMDSDNPKVQEVACECYGRLPCLGGVLERGGGGRRAEGWTNQVHCLLASANSI
LGQLYQGIETEETIQYDGPGVELPFPPLDDIDPLLILQLRQRYKAVCLALKHTLSVDPATPVRISIQHVLNLVCRALAVT
NKSINVTGEGCLKLLVLPSIHSDSLELLSALIKAVGGGLVQYCSVLTRLFSQSLSAWTPLPEASLGQQRAYSTVRVAIYC
TLELWVHVGGASSSVLQGSTSHSEMLFAHLMGDITPGTEAVKLRAGQTALSDFVGAAGKAGPRRTKGLGIGDQAGVSLQR
KGDALANQDTCFAALRALRQIILTSGTVLKEDLHKRLQDLVVPLCVRLQQQAQCGAEVAGVSGQYGSASPRRELYRLLLA
LILVPSPRWPPPLSCAVSVFSQGRRDRSIMVSSFCAEALTICNTLLHPRSPSISLPLPPLTLKPTPAAPVLGSTQNPSLS
LPTLLGGPAPGPSFPARHPLGLGPVALLGTLENHLPLAPPVLPTPPGATGAQSDLLLSPAQPAELAGLGAPEGQRQVFVR
YDKEEPEDVEISLESDSDDSVVIMPQGMMLEMQEGASNAQTLPAPSGGTMPTPAEVGSVETSLPNELPTSISHQILPTDS
NNINSFSGPGQADQLVSLVPPLNSTAVSLAASPGGLGNSLPAGPQLQQMLMQPSPGGQSAQLGLSLHMQLQSQLAQTSRQ
LQQQPSSAPEEDQNVININSSDDEEEEDEEMEDEDELGEEEEEEEEGLEEDEEEEEGSDFPEEEEFYPGEEYEDFEEEEG
EEMEEEEEEEEEEEVQPLEGEDQRAVMGEEEGEVMIEEQEERRIDAFHVEREGQVEAGIEEMEGVRSIYGEEGMKDKVSV
EEIENIGAVERNESEASEPQIEARVIVDEAEEVREGTSAAATAAAAPPEQDVRPWEQEGTGQEPVPEAPEDSAIPKESQE
VAAEAGVSEQSAEGEQEETANQSTASTSEEVPSQQAEEAAVKEKDEGQQEEEEEARGTKRKIEDCEEGERSEQGSEKKKL
DDEAMASMLADFVDCPPDDEDHGASQSHS
> Larimichthys crocea [A0A6G0HHX5]
MATSAWLRGPSALRLTEALVSVLKEQRPEYLPALLANYREHGVFPTQGASSVGGLVGFTNAKLGSSKTRFEGLCLLSMLV
KDSSSDLFQQHCLSWLRSLQQVIQSQAPVQTIQLAVNILKDVLQYSSQLAELAREVGLNSILGILTSLLGLKTECELAAM
EGMTACMTYYPRACGSLKDKLGAYFLSKMDSTNKKTQEMACQCYGCLPCLGGLLDRGVGAGRAEGWTSQIHCLLASANGQ
LAQIYQGSETDGTVQYEGPGVELAFPHLDQSDPLLLLQLQHRYTAVCMALKHTLRVDPASAVRLPVRPILNLVCRALAVS
SKSINLTGDGSVRLLVLPIIHTNTLEVLSALITAVRSGMVQYAAVLQRLFSQTLSAWTPVPESSLGQQRAYSSVRVSVHR
TLELWVQVAGASASIFQGSPGHSELLFSHLLGDITPGAESIKLRAGLSADAVPGGKPGPRRTKPLVMADTVGPSLQRKGD
LLANQDTCLSALRALRQIILTSGTLLKDDIHKRLHDVVLPLCVRLQQQQSSSNTVCETAGSVSGQYSSALTRRELYRLLL
ALVLVPSPCWPPPLTCAVSILSNGCIDRNLKVSTFCTESLTICNSLIHPRTPSITLPLPPLAMKPTPTAPVLPTSQGPTS
GLTLPALLGGPAPGPSFPTRHSLGLGPASLLGSLENHLSLVPGLPGQAPTPGDMILSPHAHHQQDPAGLGLPESQRPVFI
RYDREEADDVEISLASDSDDSVVIVPPGMLNMENQQDETAAANSQNMASAAPGGATVTLPGGESVTMVPTTSTTTTMDEV
SLPNDLATSSPLLTTSTTPINSFPPSNASVVSLVPPLSSSTLTAPPGGLGDSLPGRPQLQQMLMQPSTPGPIGLPLQIHQ
LQNQLSQQGRHLHQHQPPPPANNEDSGVININSTDDEEEEDMEDDEELEEEEEGMDEEDEEEEVSDFAEEEFYDGEEYED
YDEEEGEELEEEEEEEDGDIPPLEGAEDKAGEVGIEEGKMLRAAVDEGGMAGFSVEGETEGGIEEIQTNRALFREDRMKV
QEVESIGVLEEAREGEGEGEEDENERMDDPTMPQILCVTGGALEEREEAEEEGGGGEGGPQEEVSWEEGANETQPKVTSE
ECKTDEHQQESGAEPAQEAAASDNQPSCHQEGQLAAVQGDVTAADPETLTDQKTKEEEETDAEQRDKIKAEQQETEEGQG

RVSDEEEGKGVKRKREEVHREEEAGQSTEKKKLDDDTMASMLADFVACPPDDEDGASGSNHS
> Salmo salar [A0A1S3N940]
MAATAAWMHGPANMRLTEGLVSVLKEQRPEYLPALLANYREHGVVSTQSSAAVGGLVGVSNAKLGNSKTRFEGLCLLSVL
VKDSSSDVFQQHCLSWLRSLQQVIQSQAPLPSIQLAVGVLQDLLQYSSQLPELAREVGLNSILGILTSLLGLKSEFHLAA
MEGMTACMTFYPRACGSLRDKLGACFLSKMDSVNPEVQEAACECYGRLPCLGGVLERGGGGRPAEGWTNQLHCLLASANG
MLAQLYQGTESEGMVPYEGPGVELPYPPLDDTDPLVLLQLQHRYRGVCLALKHTLSVDPATAVRLPVQQVLNLVCRALAV
SSKSINLTGDGSVRLLVLPSIHNHTVKVLQALITAVGSGLVQYSNMLQRLFSQTLSAWTPLPETSLGQQRAFSAVRVSLY
QTLELWVKVGGASAGVLQGSPTHSEILLAHLLGDITPGADSLRAGQSTVADLVSSKPCPKSRKPGLGMGNGGGASLQRKG
DSLANQDTCVSALRALRQIILTSGTLLKEDVHKRLHDVVLPLCVRLQQQHGGDCGTGAVSGQYGSALPRRELYHLLLALV
LVPPPRWPPPLTCTVSILSHGRRDRSLKVSSFCTEALAICNSLLHPRTPSLCLPLPPLTLKPSPAASLLTPSQASSLTLP
TLLGGPFPGRHSLGLGHTLLGSLDNHLSLVPPGLSGQGSTPGDLLLSPHQGELAGLGLSEGQRPVFIRYDKEEAEDVEIS
LESDSDDSVVIFPRGMLMLENQDGTSTVANLPVSSLVPGGVTLPVPGPGDDPGGDISLPLANDLPSTSLPHPLLPSSSAP
NSINSFPPAPLASLVPPLNSTGVTQLGAPSVGLGVGADSLPGAQLQQMLLQGQPPAPGQPTPLGLPIQMLQNQLAQPSRA
LQQQQASEEDHSVININSSDEEEEEDEEMEDEDELGEEEDEEGLEDEEEEEEGSDFPDEEEEFYGEEFDDYEEEEGEEME
EEEEEEEEEEEEGEIRPLDREGRRGGMGREEGEVLREVPEDGGMGGFCVEGEMEGGIEELGTNRRVYGEEGVKAQEVESI
GVLEEEREGEEDDADGMNDPTMPQILCVTGGALEEREELGEGHGQEVGSYEQQGADRPEATPSSEGPAPQHKQEAEPAQE
VRVGGSDQPSNQGEEPAKQGGDSAKEEVKPSAAPSETEGEEPEREKGGEEGEEDGEEDGRGMKRKREGEEEGTGQGTEKK
KLDEEVMASMLADFVACPPDDEEKGPSASNLPS
> Oreochromis aureus [A0A668U741]
MATSAWLHGPSAMRLTEGLVSVLKEQRPEHLPALLAGYREHGVFQTQSACAVGGLVGFSNAKLGSSKTSRFEGLCLLSML
VKDSSSDLFQQHCLSWLRSLQQVIQSQAPVESVQLAVNILKDLLQYSSQLAELAREVGLNSILGILTSLLGLKSECELAA
MEGMTACMTYYPRACGSLRDKLAAYFLSKMDSTNRKTQEMACQCYSRLPCLGGVLDRGVGAGRAEGWTNQIHCLLASANS
LLAQLYQGSETDGAMQYQGAGVELAFPHLDQTDPLLLLQLQHRYTAVCLALKHTLRVDPASAVRLPVRPILNLVCRALAV
SSKSINLTGDGSVRLLVLPIIHSNTLEVLSSLITAVRVGMVQYAAVIQRLFSQTLSAWTPLPETSVGQQRAYSAVRVSVY
RTLELWVQVAGASSNILQGSPGHSELLFNHLLGDITPGAESIKVGESCIWTRLSADVVPGGKPGPRRTKHLVMADTVGPS
LQRKGDHLANQDTCLAAVRALRQIIQTSGTLLKDDIHKRLHEVVLPLCVRLQQQSCSSNACESAGSASGQYSSALTRRE
LYRLLLALVLVPSPCWPPPLTCAVSILSNGRTDRNVKVSSFCAEALTICNLILHPRFPSIALPLPPLTMKPSSTTPVLPS
SQGPAPGLTLPTLLGGPAPGPPFPTRHSLNLGPASLLSSFENHLSLVPGLPGQGPTSGDMILSPHTHHQPDPAGLGPPEG
QRPVFVRFDKEEAEDVEISLASDSDDSVVIVPPGMLNMETQQDDTAANSQSMPSSAPGGTGVTLPGAESVTMVPTTAATA
TIDGVSLPNDLAASSPLLTASTTSINSFPPSSASVVSLVPPLNSSTFTAPPVGLGESLPGRPQLQQMLMQPSTPSQAGPM
GLPLQMHLQNQLSQQGRHLHQQPAAPASSEDSAVININSTDDEEEDDEDIDEDEELEEDEEEEGIDEEDEEEDVSELADD
FYDGEEYEELDEEDGEELEEEEDEEEEEEDGDIPPLEGVEDKDGGSGIQEGKLDEGRISGFNVEERTEGGIEEIQTNRAL
FGEDKMKVQEVESIGVLEAAREGEVEEEDNERMDDPTMPQILCVTGGALEEREEAEEEREQAGMDDEAMASMLADFVACP
PDDEDGASGSKAS
> Collichthys lucidus [A0A4U5VV59]
MATSAWLRGPSALRLTEALVSVLKEQRPEYLPALLANYREHGVFPTQGASSVGGLVGFTNAKLGSSKTRFEGLCLLSMLV
KDSSSDLFQQHCLSWLRSLQQVIQSQAPVQTIQLAVNILKDVLQYSSQLAELAREVGLNSVLGILTSLLGLKTECELAAM
EGMTACMTYYPRACGSLKDKLGAYFLSKMDSTNKKTQEMACQCYGCLPCLGGLLDRGVGAGRAEGWTSQIHCLLASANGQ
LAQIYQGSETDGTVQYEGPGVELAFPHLDQSDPLLLLQLQYRYTAVCMALKHTLRVDPASAVRLPVRPILNLVCRALAVS
SKSINLTGDGSVRLLILPIIHTNTLEVLSALITAVRSGMVQYAAVLQRLFSQTLSAWTPVPESSLGQQRAYSSVRVSVHR
TLELWVQVAGASTSIFQGSPGHSELLFSHLLGDITPGAESIKLRAGLSADAVPGGKPGPRRTKPLVMADTVGPSLQRKGD
LLANQDTCLSALRALRQIILTSGTLLKDDIHKRLHDVVLPLCVRLQQQSSSNTVCETAGSVSGQYSSALTRRELYRLLL
ALVLVPSPCWPPPLTCAVSILSNGRIDRNLKVSTFCTESLTICNSLLHPRTPSITLPLPPLAMKPTPTAPVLPTSQGPTS
GLTLPALLGGPAPGPSFPTRHSLGLGPASLLGSLENHLSLVPGLPGQAPTPGDMILSPHAHHQQDPAGLGLPESQRPVFV
RYDREEAEDVEISLASDSDDSVVIVPPGMLNMENQQDETAAANSQNMASAAPGGATVTLPGGESVTMVPTTSTTTTMDEV
SLPNDLATSSPLLTTSTTPINSFPPSNASVVSLVPPLSSSALTAPPGGLGDSLPGRPQLQQMLMQPSTPGQPGPIGLPLQ
IHQLQNQLSQQGRHLHQHQPPPPPANNEDSGVININSTDDEEEEDMEDDEELEEEEEGMDEEDEEEEVSDFAEEEFYDGE
EYEDYDEEEGEELEEEEEEEEDGDIPPLEGAEDKAGEVGIEEGKMLRAAVDEGGMAGFSVEGEAEGGIEEIQTNRALFRED
RVKVQEVESIGVLEEAREGGGEGEGEEDENERMDDPTMPQILCVTGGALEEREEAEEEGEGGGGGEGGPQEEVSWEEGAN
ETEPKVTSEECKTDEHQQESGAEPAQEAAASDNQPSCHQEGQLAAVQGDVTAADPETLTDQKTKEEEKTDAEQRDKIKAE
QQETEEGQVRVSDEEEGKGVKRKREEVHREEEAGQSTEKKKLDDDTMASMLADFVACPPDDEDGASGSNHS
> Electrophorus electricus [A0A4W4FJQ6]
MAAAAWLHGPNGVRLTEGLASALREDRAEHLPALLADYREHGVVATHHSGAVGGLVGISNARLGSSKTRFEGLCLLSMLV
KDSSSEVFQQHCLSWLRTLQQIIQSQAPLPTVQLAVSVLSDLLQYSSQLPELAREVGLNSVLGILTSLLGLKSECHLAAM
EGMMACMTYYPRACGSLKEKLGVYFLSKMDSDNPKVQEVACECYGRLPCLGGVLERGGGGRRAEGWTNQVHCLLASANNI
LGQLYQGIETEETVQYEGPGVELPFPPIDDIDPLLILQLRHRYRAVCLALRHTLGVDPATSVRLSVQHVLNLVCRALAVS
IKSINATGEGCLKLLVLPSIHSDSLELLSALIKAVRSGLVRYCSVLNRLFSQSLSTWASLPEASLGQQRSYSAVRVTLYH
TLELWVRVGGASASVLQGSTSHSELLFTHLLGDITPGTEAVKLRAGQPAMPDLVGAAAKAGPRRTKGLGIGEPGCISLQR
KGDALANQDTCFAALQALRQIVLTSGTLLKEDLHKRLQDLVVPLCVRLQQQAQCGAEKGSVSGQYGGALPRRELYRLLLA
LVLVPSPRWPPPLSCAVSVFSQGRRDRNIVVSSFCAEALTICNSLLHPRTPSISLPLPPLTLKPTPSAPVHAPTQNPSLS
VPTLLGAPAPGPSFPARHPLGLGPAALLGSLENHLPMVPPVLPTPAGASGPPGDLLLSPAQPAELAGLGAPEGQRQVFVR
YDKEEPEDVEISLESDSDDSVVIMPEGMMLEMQEGAVNPQTLAPPPGGAMPAPAAGVVGEAGTVETALPNELPTSISHQI
LPSDANNINSFPGPSQGEQLASLVPSLNSNAVPLTAPPGGLGNSLPAAPQLQQMLLQPSPGGQAAQLTLSLHMQLQNQLA
QTSRQLQQQQQPAGAEEDQNVININSSDDDEEEDDEGIEDEEELGEEEEEEEEEEEEEEEDEEEEIQPLDGDNRRGVMAEEQ
GVVMIEEQEEGGMGAFHVERERQVEGGIEEMEAVRSVCGEEGMKDKGAVEEIENIGAVEGNESLAPEQQIETHVIGEDAE
QPEESAPAEAVEQEERPWAQEESAPEPSAAPEDTGMPREDQEVTVEGGVNEESAGGEQDVAPDQSAVSTSGEAAPQQGDE
PGEKAKGEEEQAGALEEEEEEEEARGTKRKIEDVEEAEASEQGSENKRDEMMASMLADFVDCPPDDEDHGASQSHS
> Electrophorus electricus [A0A4W4FJ25]
MAAAAWLHGPNGVRLTEGLASALREDRAEHLPALLADYREHGVVATHHSGAVGGLVGISNARLGSSKTRFEGLCLLSMLV
KDSSSEVFQQHCLSWLRTLQQIIQSQAPLPTVQLAVSVLSDLLQYSSQLPELAREVGLNSVLGILTSLLGLKSECHLAAM
EGMMACMTYYPRACGSLKEKLGVYFLSKMDSDNPKVQEVACECYGRLPCLGGVLERGGGGRRAEGWTNQVHCLLASANNI
LGQLYQGIETEETVQYEGPGVELPFPPIDDIDPLLILQLRHRYRAVCLALRHTLGVDPATSVRLSVQHVLNLVCRALAVS
IKSINATGEGCLKLLVLPSIHSDSLELLSALIKAVRSGLVRYCSVLNRLFSQSLSTWASLPEASLGQQRSYSAVRVTLYH
TLELWVRVGGASASVLQGSTSHSELLFTHLLGDITPGTEAVKLRAGQPAMPDLVGAAAKAGPRRTKGLGIGEPGCISLQR
KGDALANQDTCFAALQALRQIVLTSGTLLKEDLHKRLQDLVVPLCVRLQQQAQCGAEKGSVSGQYGGALPRRELYRLLLA
LVLVPSPRWPPPLSCAVSVFSQGRRDRNIVVSSFCAEALTICNSLLHPRTPSISLPLPPLTLKPTPSAPVHAPTQNPSLS
VPTLLGAPAPGPSFPARHPLGLGPAALLGSLENHLPMVPPVLPTPAGASGPPGDLLLSPAQPAELAGLGAPEGQRQVFVR

YDKEEPEDVEISLESDSDDSVVIMPEGMMLEMQEGAVNPQTLAPPPGGAMPAPAAGVVGEAGTVETALPNELPTSISHQI
LPSDANNINSFPGPSQGEQLASLVPSLNSNAVPLTAPPGGLGNSLPAAPQLQQMLLQPSPGGQAAQLTLSLHMQLQNQLA
QTSRQLQQQQPAGAEEDQNVININSSDDDEEEDDEGIEDEEELGEEEEEEEALEEDEEEEGSDFHDDDIYTEYHDFDMQ
EEEGEEIEDEEEEEEEEEEEEEDEEEEIQPLDGDNRRGVMAEEQGVVMIEEQEEGGMGAFHVERERQVEGGIEEMEAVRS
VCGEEGMKDKGAVEEIENIGAVEGNESLAPEQQIETHVIGEDAEQPEESAPAEAVEQEERPWAQEESAPEPSAAPEDTGM
PREDQEVTVEGGVNEESAGGEQDVAPDQSAVSTSGEAAPQQGDEPGEKAKGEEEQAGALEEEEEEEARGTKRKIEDVEEA
EASEQGSENKRDEMMASMLADFVDCPPDDEDHGASQSHS
> Channel catfish [A0A2D0RAA6]
MATAAWLHGPKNVRLTEGLVSALKEERPEYLPALLANYREHGVVGTQNSGTVGGLVGISNSRLGSSKTRFEGLCLLSMLV
KDSSSEVFQQHCLSWLRSLQQIIQSQAPLPSVQLAVSVLQDVLQYSSQLPELAREVGLNSILGILTSLLGLKSECHLAAM
EGMMACMTYYPRACGSLKEKLGVYFLSKMDSDNPKVQDVACECYGWLPCLGGVLERGGGGRRAEGWTSQVHCLLASANSI
LGQLYQGIETEETIQYEGPGVELPFPPLDEVDPLLILQLRHRYRAVCLALKHTLSVDPATSVRLPIQHVLNVVCRALAVS
IKNINVTSEGCLKLLVLPSVHSDSLEVLSALIKAVGAGLVQYCNVLSRLFSQALCAWTSLPEASLGQQRAYSAVRVALYR
TLELWVRVGRASSSVLQGSSSHSELLFAHLIGDITPGTEAVKLRAGQTAMSDLVAAAGKTGPRRTKGMGIGDPGGVSLQR
KGDALANQDTCFAALRVLKQIVLTSGTLLKEDLHKKLQELVVPLCVKLQQQAQYSNWDVGSIGGQYGSASPRCELYALLL
ALVLVPSPRWPAPLSCAVCVFSQGRRDRNITVSSFCAEALTICNALLHPRTPSISLPLPPLTLKPTPAASVLAPTQNPSL
SLPTLMGAPAPGPSFAPRHPLSLGPASLLGSLENHLPLGPPVLPTSVGATGAQGDLLLSSPAQAAELAGLAAPPETQRQV
FLRYDKEEPEDVEISLESDSDDSVVIMPQGMMLEMQEGAANTQSLPPPSGSAITVPNAGLGSEAGPVESSLSSDLPTSIG
HQMLPADANNINSFPGSSQTEQLVSLVPPLNSNSVPLAVSSAALGNSLPAGAQLQQMLMQPSPGGQPSQLGLSLHMQLQN
QLVQSSRQPAASEQDQNVININSTDDEEEEEDEEMEDEDELGEEEEEEEGLDEDEEEEEGSDFHDVYYQGADFEAYDDDD
EGEEMEEEEDEEDDEEDIQQLDADNRRDVIGAEEREVMIEGQEERGVGTFPMEGERPVEGGIEEMKAVQSIYEEEDIKDK
GSIEEIENIGAVERNESVVGEQQIETLVIGAEGEQSEENASVDAADQEVQPQEQEGSRSESVVTPEDSGISPGGQEQEVA
VEVRDNEPSAKAEQEEIPNPSTATTSEDTAPQVVVETAEKEVVEGTKVEEEEARGTKRKLEDQEEGEVSEQNSEKKKLDD
EAMASMLADFVDCPPDDEDRSASHSHS
> Carassius auratus [A0A6P6PRC2]
MASAVWLHGPNITRLTEGLVSVLKEDRPEYLPALLANYREHGVVGAQSTGAVGGLVGISNARLGSSKTRFEGLCLLSVLV
KDSSSEVFQQHCLSWLRTLQQVIQSQAPLPTVQLAVSVLQDLLQYSSQLPELAREVGLNSILGILTSLLSLKSECHLVAM
KGMMACMIYYPRACGSLREKLGAYFLSKMDSDNPKVQEVACECYGRLPCLGGVLERGGGGRRAEGWTNQLHCLLASANGM
LGQLYQGAETEGTVQYEGPGIELPFPPLDDVDPLLILQLHHRYKAICLAIKHTLSADPASSVRLPVQHVLNFVCRALAVN
TKSISPTGEGCLRLMVLPSVHNDTLELLSALIKAVGGGLIQYSSVLTRLFSQSLSAWTPLPEASLGQQRAYSSVRVTVYR
TIELWVRVGGASLLQASPSHTELLFTHLMGDITPASEAVKLRSGQQSQSMNDLIGSAGKSGPRRSKGLGMGDGISLQRKG
DVLANQDTCVAALRALRQIILTSGTLLKEDLHKRIQDLVVPLCVRLQQQSHCLLEVGAVSGQYGSPSPRRELYRLLLALV
LVPSPRWPPPLSCAVSAFSHGRRDRNIMVSSFCAEALTICNTLIHPRTPSISLPLTPLTLKSTPTAPVLASGQNSSLSIP
TLLGGPATGSFPARHPMGLGPATLLGSLENHLPLVPPVLPTPAGSTAIPGDLLLSPAQPGELAGLGAPEGQRQVFVRYDK
EEPEDVEISLESDSDDSVVIMPAGMMMEMQDGVVNAQSLSQSAVPAIGGLQPSAPIVGEVGSVDTSLPNELPTSIPLQIL
PANANNINSFPGPSQTAQLVSLVPPLNSTTASLSASPAGLADSMTGGPQLQQMLMQTSPGGQPPTLGMSLQMQLQNQIAQ
TSRQLQPQPPANEVDQNVININSSDDEEEEEEELEEEDELGEEEEEEEEGLEDDEEEEEGSDLIDEEYCEDEFDYDDEEDE
EEEGSEEIQPLEGDNDRAMMGEGEAEVMIEAEQQGMEMFCMEREREVEPGIEEMEGVRSVYADERIKDKGTLEEIENIGA
VERNEPVVDKDQIESLVISGDAEGPEENTRVEVVEPEVKTCEQEVARPEDPAEDAGPSQQGQELTVEDEVQKQEPELKPE
DTTNVQSAPSTSEQEVLQSAEETEEEEGEKESSEQGDDTEARGTKRKMEDREEGESSEQGTEKKKIDEAMASMLADFVDC
PPDDDDRGASQSQS
> Esox Lucius [A0A3P8ZVG1]
MAATAAWMHGPSNMRLTEGLVSVLKEHRPEYLPALLANYREHGVVSTQSSGAVGGLVGLSNAKLGNSKTRFEGLCLLSVL
VKDSSSDVFQQHCLSWLRSLQQVIQSQAPLPSIQLAVGVLQDLLQYSSQLPELAREVGLNSILGILTSLLGLKSECHLAA
MEGMTACMIFYPRACGSLRDKLGAYFLSKMDSANPKVQELACECYGRLPCLGGVLERGGGGRRAEGWTNQLHCLLASANG
LLAQLYQGSESEGTVQYEGPGVELPYPPLDDTDPLLLLQLRHRYRAVCLALKHTLCIDPASAVRLPLQQVLNLVCRALAV
SSKSINVTGDGCVKLLVLPAVHKDTLEVLHALITAVGSGLVQYSSVLQRLFSQTLSAWTPQPETSLGQQRAFSAVRVSVY
RTLELWVRVAGASASILQGSPTHSEILFAHLLGDIMPGADSVRLRTGQSAVADLVSSKPCPKSRKPGLAMSDGGGASLQR
KGDSLANQDTCLSALRALRQIILTSGSLLKEDIHKRLHDVVLPLCVRLQQQHGASGCDGVTGAGAGGVSGQYGSPQPRRE
LYSLLLALVLAPPPRWPPPLTCTVSVLSHGRRDRSLKVSSFCAEALTICNSLLHPRTPSLSLPLPPLTLKPTPAAPLLNQ
SQTSGPTLPTLLGGPTPGSSFPGRHHLGMGAASLLGSLDNHLPLVPPGLSGHCSTPGDLLPSPHHGELPGLGLQEGKRPV
FIRYDKEESEDVEISLDSDSDDSVVIVPRGMLALDSQDGTTAVVTPPGSSLPQGGATLPMPIPGGDPGGDISLPLPNDLP
STSLPHPLLPSSTTNSTINSFPPAPLVSLVPPINSTGVTQLGAPSTGLGVGTDSLPGAQLQQMLLQGQPPAQGQPTPLGL
PIQMQQLQNQLAQSSRALQQASEEDPAVININSSDEEEEEDEEMEDEDELGEEEEDEEGMEDEEEEEGSDFPDDEDEEFY
DGEDFDYEEGEEMEDEEEEEEEGEIRPSDRDGRRRMAAAGREEGEVLRGSAEEVGMGGFRVEREGDGGIEELDTDRRAFGE
EGIKEEEEEEDDGEGMNDPTMPQILCVTGGALEEAEELCDEAVGGDGPEVRGQEQAGAIRLEGPPHSEEGAAPLHSLEAEP
VLEEGETSKQGGENEIRQETGSVVALGETEGGASEGEKGGEEGGKGDGDEEEDGKGIKRKREGEEEGMGHGTEKKKLDEE
AMASMLADFVACPPDDEENGPSASSGPS
> Esox Lucius [A0A3P8Y054]
MAATAAWMHGPSNMRLTEGLVSVLKEHRPEYLPALLANYREHGVVSTQSSGAVGGLVGLSNAKLGNSKTRFEGLCLLSVL
VKDSSSDVFQQHCLSWLRSLQQVIQSQAPLPSIQLAVGVLQDLLQYSSQLPELAREVGLNSILGILTSLLGLKSECHLAA
MEGMTACMIFYPRACGSLRDKLGAYFLSKMDSANPKVQELACECYGRLPCLGGVLERGGGGRRAEGWTNQLHCLLASANG
LLAQLYQGSESEGTVQYEGPGVELPYPPLDDTDPLLLLQLRHRYRAVCLALKHTLCIDPASAVRLPLQQVLNLVCRALAV
SSKSINVTGDGCVKLLVLPAVHKDTLEVLHALITAVGSGLVQYSSVLQRLFSQTLSAWTPQPETSLGQQRAFSAVRVSVY
RTLELWVRVAGASASILQGSPTHSEILFAHLLGDIMPGADSVRLRTGQSAVADLVSSKPCPKSRKPGLAMSDGGGASLQR
KGDSLANQDTCLSALRALRQIILTSGSLLKEDIHKRLHDVVLPLCVRLQQQHGASGCDGVTGAGAGGVSGQYGSPQPRRE
LYSLLLALVLAPPPRWPPPLTCTVSVLSHGRRDRSLKVSSFCAEALTICNSLLHPRTPSLSLPLPPLTLKPTPAAPLLNQ
SQTSGPTLPTLLGGPTPGSSFPGRHHLGMGAASLLGSLDNHLPLVPPGLSGHCSTPGDLLPSPHHGELPGLGLQEGKRPV
FIRYDKEESEDVEISLDSDSDDSVVIVPRGMLALDSQDGTTAVVTPPGSSLPQGGATLPMPIPGGDPGGDISLPLPNDLP
STSLPHPLLPSSTTNSTINSFPPAPLVSLVPPINSTGVTQLGAPSTGLGVGTDSLPGAQLQQMLLQGQPPAQGQPTPLGL
PIQMQQLQNQLAQSSRALQQASEEDPAVININSSDEEEEEDEEMEDEDELGEEEEDEEGMEDEEEEEGSDFPDDEDEEFY
DGEDFDYEEGEEMEDEEEEEEEGEIRPSDRDGRRRMAAAGREEGEVLRGSAEEVGMGGFRVEREGDGGIEELDTDRRAFGE
EGIKAQEVESIGVLEEDREEEEEEEDDGEGMNDPTMPQILCVTGGALEEAEELCDEAVGGDGPEVRGQEQAGAIRLEGPPH
SEEGAAPLHSLEAEPVLEVGSGAQPPNQEGETSKQGGENEIRQETGSVVALGETEGGASEGEKGGEEGGKGDGDEEEDGK
GIKRKREGEEEGMGHGTEKKKVRKSLEMLKSMSIQPFYCSLSFSTIQPLCF
> Onychostoma macrolepis [A0A7J6CXH6]
MASAAWLHGPNITRLTEGLVSVLKEDRPEYLPALLANYREHGVVGAQSTGAVGGLVGISNARLGSSKTRFEGLCLLSVLV

KDSSSEVFQQHCLSWLRTLQQVIQSQAPLPTVQLAVSVLLDLLQYSSQLPELAREVGLNSILGILTSLLSLKSECHLAAM
KGMTACMIYYPRACGSLREKLGAYFLSKMDSDNPKVQEMACECYGRLPCLGGVLERGGGGRRAEGWTNQLHCLLASANGM
LGQLYQGAETEGTVQYEGPGVELPFPPLDDVDPLLILQLHHRYKAVCLAIKHTLSADPASSVRLPVQHVLNFVCRALAVN
TKSISPTGEGCLRLMVLPSIHIDTLELLSVLIKAVGGGLVQYSSVLTRLFSQSLSAWTPLPEASLGQQRAYSAVRVTVYR
TIELWVRVGGASLLQASPSHTELLFTHLMGDITPASEAVKLRSGQQSQSINDLIGSAGKSGPRRTKGLGMGDGISLQRKG
DVLANQDTCVAALRALRQIILTSGTLLKEDLHKRIQDLVVPLCMRLQQQSHCVQEVGAVSGQYGSPSPRRELYRLLLALV
LVPSPRWPPPLSCAVSAFSHGRRDHNIMVSSFCAEALTICNTLIHPRTPSISLPLTPLTLKSTPVAPVLASGQNPSLSIP
TLLGGPATGPPFPARHPMGLGPATLLGSLENHLPLAPPVLPTPAGTTATPGDLLLSPAQPCELAGLGAPEGQRQVFVRYD
KEEPEDVEISLESDSDDSVVIMPAGMMMEMQDGAANAQSLSQSAVPVVGGLQPSAPIVGEVGAVDTSLPNELPTSIPHQI
LPANANNINSFPGPSQTAQLVSLVPPLNSTTASLSASPAGLADSLTGGPQLQQMLMQTSPGGQPPTLGLSLQMQLQNQIA
QTSRQLQQQPPANEVDQNVININSSDDEEEEEEELEEEDELGEEEEEEEGLEDEEEEEEGSDFIDDEYCEDEFEEYEDEE
DEDEEESEEIQPLEGDNGRGMMGEEEAEVMIEAEEQQGMEMFCMEREQEVEPGIEEMEGVRSVYADERIKEKGTVEEIEN
IGAVERNEPVVGKDQIESLVISGDAEGHEEDTRVEAVEPEVKTCEQEVAKPEDPEEDAGPSQQGQELSVEDEIQKQEPEL
QPEDTTSQSAPSTSEQEVLQSVAETAEEEVEKESGEQGEDSEVRGTKRKMEDREEGESSEQGTEKKKMDEAMASMLADFV
DCPPDDDDRGASHS
> Sinocyclocheilus rhinocerous [A0A673FT26]
MASAAWLHGPNITRLTEGLVSVLKEDRPEYLPALLANYREHGVVGAQSTGAVGGLVGISNARLGSSKTRFEGLCLLSVLV
KDSSGEVFQQHCLSWLRTLQQVIQSQAPLPTVQLAVSVLQDLLQYSSQLPELAREVGLNSILGILTSLLSLKSECHLIAM
KGMMACMIYYPRACGSLREKLGAYFLSKMDSDNPKVQEVACECYGRVPCLGGVLERGGGGRRAEGWTNQLHCLLASATGM
LGQLYQGAETEGTVQYEGPGVELPLSPLDDVDPLLILQLHHRYKAICLAFKHTLSADPASSVRLPVQHVLNFVCRALAVN
TKSISPTGEGCLRLMVLPSIHNDTLELLAALIKAVGGGLVQYSSVLTRLFSQSLSAWTPLPEASLGQQRAYSAVRVTVYR
TIELWVRVGGASLLQASPSHTELLFTHLMGDITPASEAVKLRSGQQSQSMNDLIGSAGKSGPRRTKGLGMGDGISLQRKG
DVLANQDTCVAALRALRQIILTSGTLLKEDLHKRIQDLVVPLCVRLQQQSHCVLEVGAVSGQYGSPSPRRELYRLLLALV
LVPSPRWPPPLSCAVSAFSHGRRDHNIMVSSFCAEALTICNTLIHPRTPSISLPLTPLTLKLTPTAPVLASGQNPSLSIP
TLLGGPATGPPFPARHPMGLGPATLLGSLENHLPLAPPVLPTPAGTTATPGDLLLSPAQPGELAGLGAPEGQRQVFVRYD
KEEPEDVEISLESDSDDSVVIMPAGMMMEMQDGAANAQSLSQSAVPAVGGLQSSAPIVGEVGSVDTSLSNELPTSIPHQI
LPANANNINSFPGPSQTAQLVSLVPPLNSTTVSLSASPAGLADSLTGGPQLQQMLMQTSPGGQPPTLGLSLQMQLQNQIA
QTSRQLQPQPPANEVDQNVININSSDDEEEEEEELEDEDELGEEEEEEEGLEDEEEEEEGSDFIDEEYSADELEDYEDEE
EEDEEESEEIQPLEGDNGREMMGEEEAEVMIEAEEQQGMEMFCMEREREVEPGIEEMEGVRSVYADERIKDKGTMEEIEN
IGAVERNEPVVGKDQIESLVISEDAEGHEEDTRVEAVEPEVKTCEQEVARPEDPAEDAGPSQQGQELTVEDKVQKQEPEL
QPEDTTNQSTPSTSEQEVLQSVAETAEEEVEKESGEQGEDSEARGTKRKMEEREEGESSEHGTEKKKMDDEAMASMLADF
VDCPPDDDDRGASQSQS
> Betta splendens [A0A6P7LPE1]
MAASAWLHGPSAMRLTEGLVSVLKEPRPEYLPSLLGGYREHGVFQTQSASVVGGLVGFSNAKLGSSKTRFEGLCLLSMLV
KDSSSDLFQQHCISWLRSLQQVIQSQAPVQTIQLAVNILKDLLQYSSQIAELSREVGLNSILGFLTSLLGLKAECELAAM
EGMIACMTYYPRACGSLRDKLGAYFLSKMDSTNKKTQEMACQCYSRLPCLGGLMERGVSAGRADGWTNQIHSLVASANGL
LAQMYNSSETDGTVQYEGPGVELAFSHLDDSDPLILLQLQHRYTAVCLALKYTLRADPASAVHLPVRPILNLVCRALAVS
LKSISLIGDGSVKSLLLPIIHINTLEVLSAVITVVNSGMIPYAAILQRLFSQTLSVWTPPFECSLGQQRAYSSVRVSVYR
ALELWVQLAGPSANILQGSSTHSDLLFTHLLGDITPGAESVKLRAGLSADAVPGGKPGPRRTKPLVIADTAGPSLQRKGD
LLANQDTCLSALRALRQIILTSGTLLKDDIHKRLHDVLLPLCVRLQQQQTSSSSACETAGGISGQYSSALSRRELYRLLL
ALVLVPSPSWPPPLTCAVSIISRGRTDRNLKVSAFCTEALTICNSLLHPRTPSIALPLPSLTLKPNPTAPVLPSSSGPTP
GLTLTTLLGGPAPAPPFPTRHPLGLGTPSLLGSLENHLSLVPGLPGQGPTSGDMILSPRTHHPPETAGLGPPDGQRPVFV
RYDKEEAEDVEISLASDSDDSVVIVPPGMLNMDNQQDETGAAPNSQNMASAAPGTTVALQGSESVTMVPTTAATTTVDGI
SLSNDLSTSSSLITTSSTPINSFPPSSTSVVSLVPSLNSSTLTTPPVLGDSLPGRPQLQQMLMQPSTAGQSSSIGLPLQM
HHLQNQLSQQGRHLHQHQPPPASNEDSAVININSTDEEDEDEDMEDDEELEEEEEGMDEEDEVSDFADEELYDGEEYEDY
DEEEGEELEEEEEEEEEDEDGDIPPLEEAGEKAEELGIEEGKVHQAAVDEPEVAAFSIDADTEGGIEEVQTNRAMFGEDR
MKVQKVESIGVLEEAREGGGDEDEIERIDDPTMPQILCVTGGALEERDEPEEEGAGAGEEVVQEASSWEQGAGETEPTAA
SEDADQDKQESGAETAHDLSVSDKQPPVLEREQQEAVQGDSATAQPEAEAATGLNTEGVEADREKTGDVEEQQQQPHTEG
GGGGERDEEEAKGVKRKREEACREEAGPSSEKRKIDDDAMASMLADFVALPPDDDDAEEERPSGSNT
> Bagarius yarrelli [A0A556TYY2]
MATAAWLHGPKNMRLTEGLVSALKEERPEYLPAILADYREHGVVGTQNCGTVGGLVGISNSRLGSSKTRFEGLCLLSMLV
KDSSSEVFQQHCLSWLRSLQQIIQSQAPLPSVQLAVSVLQDVLQYSSQLPELAREVGLNSILGILTSLLGLKPECQLAAM
EGMMACMTYYPRACGSLKEKLGVYFLSKMDSDNPNVQDVACKCYGRLPCLGGVLERGGGGGRRAEGWTSQVHCLLASANS
ILGHLYQDIETEETIQYEGPGVDLPFPPLDDVDPLLILQLRHRYRAVCLALTHTLSVDPATSVRLPIQHVLNLVCRALAV
SIKNVNVTSEGCLRLLVLPTIHSDSLEVLSALIKVVGAGLVQYCNVLSRLFSQALCAWTPLPEASLGQQRAYSAVRVALY
HTLELWVRVGRASSAVLQGNSSHSELLFAHLIGDITPGTEAVKLRSGQIAMPDLVAAAGKSGPRRTKGMGIGDPGGVSLQ
RKGDSLANQDTCFAALRVLRQIILTSGTLLKDDLHKKLQELVVPLCIRLQQQAQCSNWDVGSISGQYGSAAPRCELYALL
LALVLVPSPRWPAPLSCAVCVFSQGSKDRSITVSSFCAEALTICNALLHPRTPSISVPLSPLTVKATPTASVLAPTQNPS
LSLPTLLGAPAPGPSFAARHPLTLDSASLLSSLENHLPLGPPVLSTPAGATGAQGELLLSSSAQAAELAGLAAPPETQRQ
VFVRYDKEEPEDVEISLESDSDDSVVIMPQGMMLEMQEGASNTQSLTLPPGSVIPVPNATLGSEAGPVETSLSSDLPTTI
GHQMLSADANIMNSFPGSSQTEQLVSLVPPLNSNAVPLAASSGALGNSLPAGAQLQQMLMQPSPGGQPGQLGLSLHMQLQ
NQLVQSSRQPAANEQDQNVININSTDDEEEEEDEGIEDEDELGEEEEEEEGLEEDEEEEGLEEDEEEEGSDFPEDGYQGV
EFGFDEEEDEGEEIEEEEDDEDDEEEIQALDADSRRDVVGEEEGEIMIEGQEERGVGTFPMEAERLVEGGIEEMKAVQSI
YEEEGELKDKGGIEEIENIGAVERNESGVGDQQIETRVIGAEGEQMEENASVEAADQEVQPQEQEGFRPESEVTTEESGI
PPEAQEQEGAVEVRADTPCANAEQEEIPNPSTATTAEKTAPLHIVETAEKEVVEEVQVAEEEEARGTKRKMEDMEEGEVS
EKSSEKKKLDDEAMASMLADFVDCPPDDEDHGASQSQS
> Hucho hucho [A0A4W5RM09]
MAATAAWMHGPSNMRLTEGLLSVLKEQRPEYLPALLANYREHGVFSTQSTGAVGGLVGLSNAKLGNSKTKFEGLCLLSVL
VKDSSSDVFQQHCLSWLRSLQQVIQSQDPLPSVQLAVGVLQDLIQYSSQLPELAREVGLNSVLGILTSLLGLKSECHLAA
MEGMTACMTFYPRACGSLRDKLGAYFLSKMDSANPKVQEVACECYGRLTCLGGVVERGGGGRRAEGWTNQLHCLLASANG
ILAQLYQGSESEGTVQYEGPGVELPYPPLDDADPLLLLQLQHRYRAACLALKHTLGVDPASAVRLPVQQVLNLVCRALAV
SSKSINVTGDGCVRLLVLPAIHKDTLQVLHALITAVGSGLVQYSSVLQRLFSQTLSAWTPLPETSLGQQRAFSAVRVSLY
RTLELWVRVGGASASVLQGSPTHSELLLAHLLGDITPGADSVRPSSSPQLRDVVSSKPCPKSRKPGLAMSNGGRASFQRK
GDSLANQDTCLSALRALRQVIVTSGSLLKEDIHKRLHDAVLPLCVRLQQQHGANWNDSGAGGLSGQYGSAPPRRELYRLL
LALVLVPPPRWPPPLTCTVSILSHGRRDRSLKVSSFCTEALTICNSLLHPRTPSLSFPLKPTPSQASNLTLLGGPTPGPP
FPSSLDNHLPLVHPGLSGSQRPVFIRYDKEEAEDVEISLESDSEDSVVIVPQGMLMLESQDGTSTAATLPGSSLAPGGVT
LPVPGSGGELGGDISLSNDLPSTSLPHPLLPSSTPNSTNSFPPALLVSLVPPLNSTSVAQLGEPSVGLGVGADSLPGAQL

QQMLLQSQPPAPGQPTALGLPIQVHQNQLAQSSRALQQQQQSIDEDHTVININSSDEEEEDELEEEEDEEEEESDFPDEE
EYYDEELDDYEAEEGEEMEDEEEREIRLLDGEGRGGMGGREEGEVLRGLAEEGGIGGFRVEREGEGGIKAQEVESIGVLE
EEREGEEDDGEGINDPTMPQILCVTSGALREREELDEEGAGHGQKVRSWEQKGGNRPEGVSSSEEGPAAQHKQEAEPAPE
VRVDGDDQPSNQEGEISKRGIDNTGQETAPRETKGEGPEREKERKEGEDGRGMKRKREGEEEGTGQGTEKIKLDEEAMAS
MLADFVACPPDNEENVPSAANRPS
> Sinocyclocheilus graham [A0A672QIR9]
MASAAWLHGPDITRLTEGLVSVLKEDRPEYLPALLANYREHGVVGAQSTGAVGGLVGISNARLGSSKTRFEGLCLLSVLV
KDSSSEVFQQHCLSWLRTLQQVIQSQAPLPTVQLAVSVLQDLLQYSSQLPELAREVGLNSILGILTSLLSLKSECHLVAM
KGMMACMIYYPRACGSLREKLGAYFLSKMDSDNPKVQEVACECYGRVPCLGGVLERGGGGRRAEGWTNQLHCLLASATGM
LGQLYQGAETEGTVQYEGPGVELPFPPLDDVDPLLILQLHHRYKAICLAFKHTLSADPASSVRLPVQHVLNFVCRALAVN
TKSISPTGEGCLRLMVLPSIHNDTLELLAALIKAVGGGLVQYSSVLTRLFSQSLSAWTPLPEASLGQQRAYSAVRVTVYR
TIELWVRVGGASLLQASPSHTELLFTHLMGDITPASEAVKLRSVQQSQSMNDLIGSADKSGPRRTKGLGMGDGISLQRKG
DVLANQDTCVAALRALRQIILTSGTLLKEDLHKRIQDLVVPLCVRLQQQSHCVLEVGAVSGQYGSPSPRRELYRLLLALV
LVPSPRWPPPLSCAVSAFSHGRRDHNIIVSSFCAEALTICNTLIHPRTPSISLPLTPLTLKLTPTAPVLASGQNPSLSIP
TVLGGPARGSPFPARHPMGLGPATLLDSLENHLPLAPPVLPTPAGTTATPGDLLLSSAQPGELAGLGAPEGQRQVFVRYD
KEEPEDVEISLESDSDDSVVIMPAGMMMEMQDGAANAQSLSQSAVPTVGGLQSSAPIVSEVGSVDTLLSNELPTSIPHQI
LPANANNINSFPGPSQTAQLVSLVPPLNSTTASLSASPAGLADSLTGGPQLQQMLMQTSPGGQPPTLGLSLQMQLQNQIA
QTSRQLQPQPPANEVDQNVININSSDDEEEEEEELEDEDELGEEEEEEEEGLEDEEEEEEGSDFIDEEYSAEEFEDFEDEE
GEDEDEEESEEIQPLEGDNGRGMMGEEEAEVMIEAEEQQGMEMFCMEREREVEPGIEEMEGVRSVYADERIKDKGTMEEI
ENIGAVERNEPVVGKDQIESLVISEDAEGHEEDTRVEAVEPEVKTCEQEVARPEDPAEDAGPSQQGQELTVEDEVQKQEP
ELQPEDTTNQSTPSTSEQEVLQSVAETAEEEVEKESGEQGEDSEARGTKRKMEDREEGESSEHGTEKKKMDDEAMASMLA
DFVDCPPDDDDRGASQSQS
> Oreochromis aureus [A0A668U1Q7]
MATSAWLHGPSAMRLTEGLVSVLKEQRPEHLPALLAGYREHGVFQTQSACAVGGLVGFSNAKLGSSKTRFEGLCLLSMLV
KDSSSDLFQQHCLSWLRSLQQVIQSQAPVESVQLAVNILKDLLQYSSQLAELAREVGLNSILGILTSLLGLKSECELAAM
EGMTACMTYYPRACGSLRDKLAAYFLSKMDSTNRKTQEMACQCYSRLPCLGGVLDRGVGAGRAEGWTNQIHCLLASANSL
LAQLYQGSETDGAMQYQGAGVELAFPHLDQTDPLLLLQLQHRYTAVCLALKHTLRVDPASAVRLPVRPILNLVCRALAVS
SKSINLTGDGSVRLLVLPIIHSNTLEVLSSLITAVRVGMVQYAAVIQRLFSQTLSAWTPLPETSVGQQRAYSAVRVSVYR
TLELWVQVAGASSNILQGSPGHSELLFNHLLGDITPGAESIKLRAGLSADVVPGGKPGPRRTKHLVMADTVGPSLQRKGD
HLANQDTCLAAVRALRQIIQTSGTLLKDDIHKRLHEVVLPLCVRLQQQQSCSSNACESAGSASGQYSSALTRRELYRLLL
ALVLVPSPCWPPPLTCAVSILSNGRTDRNVKVSSFCAEALTICNLILHPRFPSIALPLPPLTMKPSSTTPVLPSSQGPAP
GLTLPTLLGGPAPGPPFPTRHSLNLGPASLLSSFENHLSLVPGLPGQGPTSGDMILSPHTHHQPDPAGLGPPEGQRPVFV
RFDKEEAEDVEISLASDSDDSVVIVPPGMLNMETQQDDTAANSQSMPSSAPGGTGVTLPGAESVTMVPTTAATATIDGVS
LPNDLAASSPLLTASTTSINSFPPSSASVVSLVPPLNSSTFTAPPVGLGESLPGRPQLQQMLMQPSTPSQAGPMGLPLQM
HLQNQLSQQGRHLHQQPAAPASSEDSAVININSTDDEEEDDEDIDEDEELEEDEEEEGIDEEDEEEDVSELADDFYDGEE
YEELDEEDGEELEEEEDEEEEEEDGDIPPLEGVEDKDGGSGIQEGKLDEGRISGFNVEERTEGGIEEIQTNRALFGEDKM
KVQEVESIGVLEAAREGEVEEEDNERMDDPTMPQILCVTGGALEEREEAEEEREQAGAELQKGEECTWEQGAKEDEPQAA
ESEQPASHSQQQESAAEPAEEASVSDNQLPSCEDEQPEAVQAGDSTAVADSENSTEQNVTEQQEADTEKSSEKEAEQPET
GGGGAQESNGEEGKGVKRKREEGHGKEEVGQSPEKKKMDDEAMASMLADFVACPPDDEDGASGSKAS
> Salarias fasciatus [A0A672I4G0]
MAISFSTSSNNMAASWLHGPSVLRLTDGLVSVLKEQRPEHVPALLACYREHGAFSSQGSGALGGLVGFSNAKLGSNKTRF
EGLCLLSMLVKDSSSDLFQQHCLSWLRSLQQIIQSQAPVQTVQLAVNVLKDVLQYSSQLAELAREVGLNSILGILTSLMG
LKTECELAAMEGMTACMTFYPRACGSLRDKLGAYVLSKMDSTNRKTQEMACQCFGRLPCLGGLLDRGLSAGRAEAWTNQI
HCLLASANGLLSQMYQGAETDGALQYQGPGVELPFPHLDQTDPLLLLQLLHRYTAVCLALKHTLRVDPATAVRLPARRIL
NTVCRALAVSSKSINLTGDGSVRLLVLPAIHTNTLEVLAGLITSVRGGLVQYAAVLQRLFSQTLSSWTPLPEASVGQQRA
FSSVRVSVYKTLALWVQVGGASTGVLQGSQTHSELLFNHLLGDITPSADSVKVSPDRTVVAGGKPGPRRTKQLVIADGMG
PALQRKGDLVANQDSCLSALKALRHIILTSGTLLKDDVHKRLHDVVLPLCVRLQQQQSSSAALETAGGVSGPYGSALSRR
ELYSDLLKLLLALVLVPSPSWPPPVSCATSILSKGRIDRNLKVSSFCAEALTVCNCILHPRTPSISLPLPPLALKPTPAA
PVMPPSQGPGPGLTLPTTLLGSSAPGPPFPSRHSLGLGPGPGPASLLGSLENHLSLVPGLPGQGPGPGDLLLSPHAHHQP
ELGPPEGQRPVFVRYDKEEAEDVEISLDSDSDDSVVIVPPGMLGADGQQDEAAPPNSHSVLSAAPGDAGAPPPGGDGASM
APPAAAPSTVDGSSLHNDLASSSPLLPTSTAPINSFPPSVASVVSLVPPLNSGALAAPPGGLGDPLPGRPQLQQMLMQPP
PPGQPPSVGLPLQMHQLQNQLGPPGRHLHPHPPPPPANNEDSAVININSTDEEDEDEEDMEDEEELEEEEDEGMDEEDEE
EEVSDIAEDEFYDGEEYEDYDEEDGEELEEEEEEDEEGGIPPLEGGDDKESETEDGKVLRAPVDEGGPSGFGVEGEPEAG
IEEVQTSRTLFGEDRMKVQEVESIGVLEAAREAEGEAEEDEPAVQLVTDADMAATPPGGRQEQEEPPAAPPPPSSSSSSC
AAEQLTQAEKEEEGEEEEEEPAGEGGDGGESDGQEGKGLKRKREEAPIEEEEEEGQSTEKKQVDDEAMASMLADFVACPP
DDEEGASGSGRS
> Danio rerio [F1RE30]
MASTSWLHGPNITRLTEGLVSVLKEERPEFLPALLANYREHAAVGTQSSGAVGGLVGLSNARLGSSKTRFEGLCLLSVLV
KDSSSEVFQQHCLSWLRTLQQIIQSQAPLPTVQLAVNVLQDMLQYSSQLPELAREVGLNSILGVLTSLLSLKSECHLAAM
KGMMACMTYYPRACGSLREKLGAYFLSKMDSDNPKVQEVACECYGRLPCLGGVLERGGGGRRAEGWTNQLHCLLASANSI
LGQLYHGAETEGTVQYEGPGVELPFPPLDDVDPLLILQLQHRYKAVTLAMKHTLSVDPASSVRLPVQHVLNLLCRALAVN
TKSISPTGDGCQKLLVLPSIHNDTLELLSALIKAVGGGLVQYSSVLTRLFSQSLSAWTPLPEASLGQQRAYSAVRVAVYR
LIELWVRVGGAALLQGSPSHTELLFTHLMGDVTPGSEAVKLRSVQQSSMTDLIGSAGKSGPRRAKGMGEGLTIQRKGDVL
ANQDTCVAALRALRQIVLTSGTLLKEELHKRIQDLVVPLCVRLQQQAHCVQEVGAISGQYGSPSPRRELYRVLLALVLVP
SPRWPPPLSCAVSIFSHGRRDRNIKVSSFCAEALIICNTLIHPRTPSISLPLTPLTLKSTPSAPVLASGQNSSLSIPTLL
GGPTTGPPFPARHPMGLGPANLLGSLENHLPLAPQVLSSSAGTPGDLLLSPAQPGELTGLGVPEGQRQVFVRYDKEEPDD
VEISLESDSDDSVVIMPAGMMMEMQDGAANTQSLSQPAVSTVGGLQTSTPVVGEVGSADTSSANELPGSIPHQILPANNI
NSFPGPNQTAQHVAIVPPLNSATASLSATVVGLADSLTGGPQLQQMLMQTSPGGQPPTLGLSLQMQLQNQIAQTSRQLQQ
QPPGNDVDQNVININSSDDEEEDEEELEEEDELGEEEEEEEEGLDDEEEEEEGSDFIDDEEYLEGEEFEYEDDEGEDEDEEE
SEEIQPLEGDDGRAIIGEEEAEVMIEPDEQQRLEMFCVEGEREVEPGIEEMEGVRSIYAEDRIKEKSTLEEIENIGAVER
NEPVIDKQQIETLVIGGDVEEPEEDTTVEAVEPEVKECEQEEVKPDDPAEEEEEEAGPSQQAQEVIVEDEVQKQEPELQPE
DKADQSAPSTSEQETIESVAETVEEEKATTEEKDDEETRGTKRKMEDREEGESSEQGSEKKKIDDEAMASMLADFVDCPP
DDDDHGASQTQS
> Oreochromis niloticus [I3J7N9]
MATSAWLHGPSAMRLTEGLVSVLKEQRPEHLPALLAGYREHGVFQTQSACAVGGLVGFSNAKLGSSKTRFEGLCLLSMLV
KDSSSDLFQQHCLSWLRSLQQVIQSQAPVETVQLAVNILKDLLQYSSQLAELAREVGLNSILGILTSLLGLKSECELAAM
EGMTACMTYYPRACGSLRDKLAAYFLSKMDSTNRKTQEMACQCYGRLPCLGGVLDRGVGAGRAEGWTNQIHCLLASANSL

LAQLYQGSETDGAMQYQGAGVELAFPHLDQTDPLLLLQLQHRYTAVCLALKHTLRVDPASAVRLPVRPILNLVCRALAVS
SKSINLTGDGSVRLLVLPIIHSNTLEVLSSLITAVRVGMVQYAAVIQRLFSQTLSAWTPLPETSVGQQRAYSAVRVSVYR
TLELWVQVAGASSNILQGSPGHSELLFNHLLGDITPGAESIKLRAGLSADVVPGGKPGPRRTKHLVMADTVGPSLQRKGD
HLANQDTCLAAVRALRQIIQTSGTLLKDDIHKRLHEVVLPLCVRLQQQQSCSSNACESAGSASGQYSSALTRRELYRLLL
ALVLVPSPCWPPPLTCAVSILSNGRTDRNVKVSSFCAEALTICNLILHPRFPSIALPLPPLTMKPSSTTPVLPSSQGPAP
GLTLPTLLGGPAPGPPFPTRHSLNLGPASLLSSFENHLSLVPGLPGQGPTSGDMILSPHTHHQPDPAGLGPPEGQRPVFV
RFDKEEAEDVEISLASDSDDSVVIVPPGMLNMETQQDDTAANSQSMPSSAPGGTGVTLPGAESVTMVPTTAATATIDGVS
LPNDLAASSPLLTASTTSINSFPPSSASVVSLVPPLNSSTFTAPPVGLGESLPGRPQLQQMLMQPSTPSQAGPMGLPLQM
HLQNQLSQQGRHLHQQPAAPASSEDSAVININSTDDEEEDDEDIDEDEELEEDEEEEGIDEEDEEEDVSELADDFYDGEE
YEELDEEDGEELEEEEDEEEEEEDGDIPPLEGVEDKDGGSGIQEGKLDEGRISGFNVEERTEGGIEEIQTNRALFGEDKM
KVQEVESIGVLEAAREGEVEEEDNERMDDPTMPQILCVTGGALEEREEAEEEREQAGAELQKGEECTWEQGAKEDEPQAA
ESEQPASHSQQEESAAEPAEEASVSDNQLPSCEDEQPEAVQAGDSTAVADSENSTEQNVTEQQEADTEKSSEKEAEQPET
GGGGQESNGEEGKGVKRKREEGHGKEEVGQSPEKKKMDDEAMASMLADFVACPPDDEDGASGSKAS
> Oreochromis niloticus [A0A669DJR7]
MATSAWLHGPSAMRLTEGLVSVLKEQRPEHLPALLAGYREHGVFQTQSACAVGGLVGFSNAKLGSSKTRFEGLCLLSMLV
KDSSSDLFQQHCLSWLRSLQQVIQSQAPVETVQLAVNILKDLLQYSSQLAELAREVGLNSILGILTSLLGLKSECELAAM
EGMTACMTYYPRACGSLRDKLAAYFLSKMDSTNRKTQEMACQCYGRLPCLGGVLDRGVGAGRAEGWTNQIHCLLASANSL
LAQLYQGSETDGAMQYQGAGVELAFPHLDQTDPLLLLQLQHRYTAVCLALKHTLRVDPASAVRLPVRPILNLVCRALAVS
SKSINLTGDGSVRLLVLPIIHSNTLEVLSSLITAVRVGMVQYAAVIQRLFSQTLSAWTPLPETSVGQQRAYSAVRVSVYR
TLELWVQVAGASSNILQGSPGHSELLFNHLLGDITPGAESIKLRAGLSADVVPGGKPGPRRTKHLVMADTVGPSLQRKGD
HLANQDTCLAAVRALRQIIQTSGTLLKDDIHKRLHEVVLPLCVRLQQQQSCSSNACESAGSASGQYSSALTRRELYRLLL
ALVLVPSPCWPPPLTCAVSILSNGRTDRNVKVSSFCAEALTICNLILHPRFPSIALPLPPLTMKPSSTTPVLPSSQGPAP
GLTLPTLLGGPAPGPPFPTRHSLNLGPASLLSSFENHLSLVPGLPGQGPTSGDMILSPHTHHQPDPAGLGPPEGQRPVFV
RFDKEEAEDVEISLASDSDDSVVIVPPGMLNMETQQDDTAANSQSMPSSAPGGTGVTLPGAESVTMVPTTAATATIDGVS
LPNDLAASSPLLTASTTSINSFPPSSASVVSLVPPLNSSTFTAPPVGLGESLPGRPQLQQMLMQPSTPSQAGPMGLPLQM
HLQNQLSQQGRHLHQQPAAPASSEDSAVININSTDDEEEDDEDIDEDEELEEDEEEEGIDEEDEEEDVSELADDFYDGEE
YEELDEEDGEELEEEEDEEEEEEDGDIPPLEGVEDKDGGSGIQEGKLDEGRISGFNVEERTEGGIEEIQTNRALFGEDKM
KVQEVESIGVLEAAREGEVEEEDNERMDDPTMPQILCVTGGALEEREEAEEEREQAGAELQKGEECTWEQGAKEDEPQAA
ESEQPASHSQQQNTAIQRLLNCHMWPQSDKLTPILLFSILHAAGQEESAAEPAEEASVSDNQLPSCEDEQPEAVQAGDST
AVADSENSTEQNVTEQQEADTEKSSEKEAEQPETGGGGQESNGEEGKGVKRKREEGHGKEEVGQSPEKKKMDDEAMASML
ADFVACPPDDEDGASGSKAS
> Makobe Island cichlid [A0A3B4GKZ7]
MATSAWLHGPSAMRLTEGLVSVLKEQRPEHLPALLAGYREHGVFQTQSACAVGGLVGFSNAKLGSSKTRFEGLCLLSMLV
KDSSSDLFQQHCLSWLRSLQQVIQSQAPVETVQLAVNVLKDLLQYSSQLAELAREVGLNSILGILTSLLGLKSECELAAM
EGMTACMTYYPRACGSLRDKLAAYFLSKMDSTDRKTQEMACQCYGRLPCLGGVLDRGVGAGRAEGWTNQIHCLLASANGL
LAQLYQGSETDGAMQYQGPGMELAFPHLDPTDPLLLLQLQHRYTAVCLALKHTLRVDPASAVRLPVRPMLNLVCRALAVS
SKSINLTGDGSVRLLVLPIIHSNTLEVLSSVITVVRVGMVQYAAVIQRLFSQTLSAWTPLPETSVGQQRAYSAVRVSVYR
TLELWVQVAGASSSILQGSPGHSELLFNHLLGDITPGAESVKKRHKSDHLGKPGPRRTKHLVMADTVGPSLQRKGDHLAN
QDTCLAAVRALRQIIQTSGTLLKDDIHKRLHEVVLPLCVRLQQQQSCSSNACESAGSASGQYSSALTRRELYRLLLALVL
VPSPCWPPPLTCAVSILSNGRTDRNVKVSSFCAEALTICNLILHPRFPSIALPLPPLTMKPSSTTPVLPSSQGPAPGLTL
PNLLGGPAPGPPFPARHSLNLGPASLLSSFENHLSLVPGLPGQGPTAGDMILSPHSHHQPDPAGLGPPDGQRPVFVRFDK
EEAEDVEISLASDSDDSVVIVPPGMLNMETQQDDAAANSQSMSSSAPGGTGVTLAGAESVTMVPTTAAAATIDGVSLPND
LAASSPLLTASTTSINSFPPSSASVVSLVPPLNSSTFTAPPVGLGESLPGRPQLQQMLMQPSTASQAGPMGLPLQMHLQN
QLSQQGRHLHQQPAAAASSEDSAVININSTDDEEEDDEDIDEDEELEEDEEEEGIDEEDEEEDDGDIPPLEGVEDKDGGS
GIQEGKLDEERISAFNSNGEEGKGVKRKREEGHGKEEAGQSPEKKKMDDEAMASMLADFVACPPDDEDGASGSKAS
> Parambassis ranga [A0A6P7K636]
MATSAWLHGPSAMRLTEGLVSILKEQRPEYLPALLASYREHGVFQTQNATAAGGLVGFSNAKLGSSKTRFEGLCLLSMLV
KDSSSDVFQQHCLSWLRSLQQVIQSQAPVQTIHLAVNILKDLLQYSSQLPELAREIGLNSILGILTSLLGLKSECELAAM
EGMIACMTYYPRACGSLKDKLGAYFLSKMDSTNRKLQEMACQCYGRLPCLGGLLDRGVGTGRAESWTNQIHCLLASANGL
LAQIYQGSETDGAVQSQNHGVELAFPHLNQTDPLLLLQLQHRYNAVCLALKHTLRVDPAAAVRLPVRPVLNLVCRALAVS
AKSINLTGDGSAKLLILPSIHTNTLEVLSGLITAVRSGMVQYAAVLQRLFSQTLSAWMPLPEASLGQQRAYSSVRVSVYR
TLELWVQVAGASASVLQGSPSHSELLFSHLLSDITPGAESVKLRAGLTADVVPGGKPGPRRTKQLVMADTAGPSLQRKGD
LLANQDTCISALRALRQIIQSSGTLLKDDIHKRLHDVVLPLCVRLQQQQSSSNTTCESTGGVSGQYSSALTRKELYRLLL
ALVLVPSPCWPPPLTCAVSILSNGRNDRSLKVSSFCAEALTICNSILHPRTPSVALPLPPLALKPTTSASVLPSSQGPAP
GLTLPTLLGGPPPVPPFPSQHSLGLGPASLLGSLENHLPLVPGLPSHGPTPGDMILSPHTQHQSDPAGLGHPEGQRPVFV
RYDKEEAEDVEISLASDSDDSVVIVPPGMLNMENQQDDAVVNSQNVLSTAPGGGGTLPGGESVTMVPTTAVTSTIDGASL
TNDLATSSPLLTTSTPPINSFPPTASVASLVPPLNSSTLTVPPGVLGDSLPGRPQLQQILMQPPTQGQPGPIGLPHHLLH
NQLSQQGRHLHQHQPPSANNEDSAVININSTDDEEEDEEDMEDDEELDEEEEEGMDEEDEEEEVSELGDEEFYDGEEYEE
FDEDGEELDEEEEEEEEDGDIPPLEGAEDKAADAGIEEGKVLRAAVDEAGISGFSVEGDTEGGIEEIQTNRALFAEDRIKV
PEVESIGVLEEGREGEEDEDETERMDDPTMPQILCVTGGTLEEREETEEGAEAGELQEEVSSWEQEAKESELTSTSEEQS
TCQSQQEPAAEPAQEAVVSESQPFSHQEEQLAADQGEDSLVATTTDAKTPTGPNGKEKEESDAEKRDTREAEQQEEDRRG
AEEETDGEEGKGMKRKREEEQQEEEGQITEKKKLDDEAMASMLADFVACPPDDEDDVSGSNHS
> Labrus bergylta [A0A3Q3E5A5]
MASSAWLRGSSAPRLTEGLVSMLKEQRPEYLPALLSSYREHGVFTGASAAGGLVGFSNAKLGSSKTRFEGLCLLSMLVKD
SSSDVFQQHCLSWLRSLQQVIQSQAPVQTIQLAVNILKDLLQYSSQLAELAREVGLNSILGILTSLLGLKTECELAAMEG
MTACMTFYPRACGSLRDKLGAYFLSKMDNSNNKTREMACQCYGRLPCLGGLLDRGVGAGRAEGWTNQIHCLLASANGLLG
QIYQGAETDGTLQYEGPGVELAFPPLDQSDPLFLLQLQQRYTAVCTALKHTLGVDPSSAVRLPVRPILNLLCRALAVSSK
SINLTGDGSVRLLVLPVVHTHTLEVLSALITVVRSGMVQYSAVLQRLFSQTLSAWTPLPESSLGQQRASVRVSVYRTLEL
WLQVGGASAGVLQGSPGHSEMLFSQLLSDITPGAESVKVRNRKMSSLVFRAWKPGPRRTKTLVMADSVGPSLQRKGDLLA
NQDTCLSALRALRQILLTSGTLLKDDIHKRLHEVVLPLCVRLQQQQSSCSTSCESAGGVSGQYSSALSRRELYRLLLALV
LVPSPCWPPPLTCAVSILSSGRTDRNIKVSSFCTEALTVCNALLHPRVSPISLPLPPLTLKPTPSAPVLSSAQGTGLTLP
TLLGGPAPGTPFPSRHSLGLAPASLLGSLENHLSLVPGLTPLGWAPPEGQRPVFVRYDREEAEDVEISLASDSDDSVVIV
PPGMLNMEAQQEDPGMGNPPSLVSAAPGGTTVSLPGADTVTTATTSTIDGVSLPNNHATSSPLLTTSTTPINSFPPSSTS
VVSLVPPHNSSSLTAPPGVLEESLPGRQLQQMLMQPSGQPGPLPLPLQMHQLQNQLSAQGRLLHPHQAPPSSNEDSGVIN
INSTDEEEEEEEEDMEDDEELDEEEEEGMDEEDEEFYEEDEYEDYDEEEGEELEEEEEEEEDGEIPPLEGAEEKAEEGKVL
RAAVDEGGMAGFSVEGEEEGGIEEIQTNRSLFGEDRMKVQEVESIGVLEEAREAEAEEDESERMDDPTMPQILCVTGGAM
EEREEPEEEEEEEGAGGRAEEGGGVGRLQEEAGLWQGAANEIEPSASSEESSANKNQEVRRIKSIIINMFHLPRSLSLAP

SLSQMDEESMASMLADFVACPPDDEDGASGSNRS
> Carassius auratus [A0A6P6PMN7]
MYSLESSVQNHKNPAAQSTGAVGGLVGISNARLGSSKTRFEGLCLLSVLVKDSSSEVFQQHCLSWLRTLQQVIQSQAPLP
TVQLAVSVLQDLLQYSSQLPELAREVGLNSILGILTSLLSLKSECHLVAMKGMMACMIYYPRACGSLREKLGAYFLSKMD
SDNPKVQEVACECYGRLPCLGGVLERGGGGRRAEGWTNQLHCLLASANGMLGQLYQGAETEGTVQYEGPGIELPFPPLDD
VDPLLILQLHHRYKAICLAIKHTLSADPASSVRLPVQHVLNFVCRALAVNTKSISPTGEGCLRLMVLPSVHNDTLELLSA
LIKAVGGGLIQYSSVLTRLFSQSLSAWTPLPEASLGQQRAYSSVRVTVYRTIELWVRVGGASLLQASPSHTELLFTHLMG
DITPASEAVKLRSGQQSQSMNDLIGSAGKSGPRRSKGLGMGDGISLQRKGDVLANQDTCVAALRALRQIILTSGTLLKED
LHKRIQDLVVPLCVRLQQQSHCLLEVGAVSGQYGSPSPRRELYRLLLALVLVPSPRWPPPLSCAVSAFSHGRRDRNIMVS
SFCAEALTICNTLIHPRTPSISLPLTPLTLKSTPTAPVLASGQNSSLSIPTLLGGPATGSFPARHPMGLGPATLLGSLEN
HLPLVPPVLPTPAGSTAIPGDLLLSPAQPGELAGLGAPEGQRQVFVRYDKEEPEDVEISLESDSDDSVVIMPAGMMMEMQ
DGVVNAQSLSQSAVPAIGGLQPSAPIVGEVGSVDTSLPNELPTSIPLQILPANANNINSFPGPSQTAQLVSLVPPLNSTT
ASLSASPAGLADSMTGGPQLQQMLMQTSPGGQPPTLGMSLQMQLQNQIAQTSRQLQPQPPANEVDQNVININSSDDEEEE
EEELEEEDELGEEEEEEEGLEDDEEEEEGSDLIDEEYCEDEFDYDDEEDEEEEGSEEIQPLEGDNDRAMMGEGEAEVMIE
AEQQGMEMFCMEREREVEPGIEEMEGVRSVYADERIKDKGTLEEIENIGAVERNEPVVDKDQIESLVISGDAEGPEENTR
VEVVEPEVKTCEQEVARPEDPAEDAGPSQQGQELTVEDEVQKQEPELKPEDTTNVQSAPSTSEQEVLQSAEETEEEEGEK
ESSEQGDDTEARGTKRKMEDREEGESSEQGTEKKKIDEAMASMLADFVDCPPDDDDRGASQSQS
> Clupea harengus [A0A6P8GZD5]
MATAGWLHGSASMRLTEGLVSVLKEERPEYLPSLLANYREHGVVSAQSCGAVGGLVGLSNARLGSSKTRFEGLCLLSVLV
KDGSNELFQQHCLSWLRSLQQVIQSQAPLPSVQMAVIALQDLLQYSSQIPELAREIGLNSILGILTSLLSLKAESHLVAM
EGMMACMTYYPRACGSLREKLGAYFLSKMDSDNPKVQEVACECYGRLPCLGGVLERGGGSRRAESWTNQTHCLLASANSI
LAKLYQGAETDAVVQYEGPGLELPFSPLDDIDPLLVLQFRHRYRAVCLALKNTLSVDPGSPVRLPVQHVLNFVCRALAVS
SKNINLTGDGCLKLLVLPSIHSETVEVLSTLIKVAGGSLVQYSSVLTRLFSQSLSAWTPPPEASVGQQRAYCAVRVCLYR
TLELWVRVGGASASVLQGSPTHTEILFTHLMSDLTPGSEAIKLKVGPSSGSDMVGPKAGPRRTKGLALGDTGTMTMQRKG
DALANQDTCLSALRVLRHIILNCGTLLKDELHKRLQDIVVPLCVRLQQQQQLCDVVLSGQYGSPAPRRELYRLLLALLLA
PCPRWPPPLPCAVSLFSHGRHDRSLKVSSFCAEALTICNCLLHPRTPSISLPLPPLALKPTSSAPVIPPSQNPVLTLPTL
LAGPSPGPPFFPSRHPLSLGSLENHLSLAAPVLPPSAIPGTPGDLLLSPSQPADLAGLGAPEAQRHIFIRYDKEEPEDVEI
SLESDSDDSVVIVPPGVLTQEGQDGASNSQPMPSSSGTPVQGPVAGPCPSAVPTVSGTEAVSSGDAPLTSDLPTPTSLPH
QVLPATPSTSSSFPGSQATPLVPLVPPLSSATPLAPPPVVLGEPLPSAQLQQMLMQPSPSPSGLGMSMQVHMHNHMSQLV
VRQQQQNIAGEEEQTVININSTDDEDEEDEEIEDEDEMGEEEEEGLDEEEEEEEGSDFPEEDEYYDGFEEYDEDEEEGED
IEEEEEEGAEDIPPLEGESERGIMGREDGEVLRPEEERGMELFSMDRERLQGEGGDLAEEKRGVCEEEEEEEEGEGVVDA
KERVSLEEKDATGAAEREEGISTAAQREREREREREREREREREREREREREREREREREREALGPHVEGQDEGTSRLEMGTADVGSWDQKEGLLEG
VSSLGAGGAEQIQGVGPEGRDLGGLPQARPEPEELASCSDVNAPGKEMRLPGMEEAPKAGAEGEEKEGEGDSRGIKRKLD
DLEEEVGEEGTEKKKMDDEAMASMLADFVDCPPDDEENIPSSPSHS
> Oreochromis niloticus [A0A669DES8]
MATSAWLHGPSAMRLTEGLVSVLKEQRPEHLPALLAGYREHGVFQTQSACAVGGLVGFSNAKLGSSKTSRFEGLCLLSML
VKDSSSDLFQQHCLSWLRSLQQVIQSQAPVETVQLAVNILKDLLQYSSQLAELAREVGLNSILGILTSLLGLKSECELAA
MEGMTACMTYYPRACGSLRDKLAAYFLSKMDSTNRKTQEMACQCYGRLPCLGGVLDRGVGAGRAEGWTNQIHCLLASANS
LLAQLYQGSETDGAMQYQGAGVELAFPHLDQTDPLLLLQLQHRYTAVCLALKHTLRVDPASAVRLPVRPILNLVCRALAV
SSKSINLTGDGSVRLLVLPIIHSNTLEVLSSLITAVRVGMVQYAAVIQRLFSQTLSAWTPLPETSVGQQRAYSAVRVSVY
RTLELWVQVAGASSNILQGSPGHSELLFNHLLGDITPGAESIKVGESCIWTRLSADVVPGGKPGPRRTKHLVMADTVGPS
LQRKGDHLANQDTCLAAVRALRQIIQTSGTLLKDDIHKRLHEVVLPLCVRLQQQQSCSSNACESAGSASGQYSSALTRRE
LYRLLLALVLVPSPCWPPPLTCAVSILSNGRTDRNVKVSSFCAEALTICNLILHPRFPSIALPLPPLTMKPSSTTPVLPS
SQGPAPGLTLPTLLGGPAPGPPFFPTRHSLNLGPASLLSSFENHLSLVPGLPGQGPTSGDMILSPHTHHQPDPAGLGPPEG
QRPVFVRFDKEEAEDVEISLASDSDDSVVIVPPGMLNMETQQDDTAANSQSMPSSAPGGTGVTLPGAESVTMVPTTAATA
TIDGVSLPNDLAASSPLLTASTTSINSFPPSSASVVSLVPPLNSSTFTAPPVGLGESLPGRPQLQQMLMQPSTPSQAGPM
GLPLQMHLQNQLSQQGRHLHQQPAAPASSEDSAVININSTDDEEEDDEDIDEDEELEEDEEEEGIDEEDEEEDVSELADD
FYDGEEYEELDEEDGEELEEEEDEEEEEEDGDIPPLEGVEDKDGGSGIQEGKLDEGRISGFNVEERTEGGIEEIQTNRAL
FGEDKMKVQEVESIGVLEAAREGEVEEEDNERMDDPTMPQILCVTGGALEEREEAEEEREQAGAELVSDNQLPSCEDEQP
EAVQAGDSTAVADSENSTEQNVTEQQEADTEKSSEKEADNGEEGKGVKRKREEGHGKEEVGQSPEKKKMDDEAMASMLAD
FVACPPDDEDGASGSKAS
> Salarias fasciatus [A0A672I5J6]
MELWNWARCLSRELALCLSLESSRTMFPVNRERDGTGSAGPQRPGPGPGPGSSPAYCSTMAISFSTSSSARSRSLAIPDP
EAAPRRAATHFTRSPPPPQHKELTSRRRAVMTRREDNMAASWLHGPSVLRLTDGLVSVLKEQRPEHVPALLACYREHGAF
SSQGSGALGGLVGFSNAKLGSNKTRFEGLCLLSMLVKDSSSDLFQQHCLSWLRSLQQIIQSQAPVQTVQLAVNVLKDVLQ
YSSQLAELAREVGLNSILGILTSLMGLKTECELAAMEGMTACMTFYPRACGSLRDKLGAYVLSKMDSTNRKTQEMACQCF
GRLPCLGGLLDRGLSAGRAEAWTNQIHCLLASANGLLSQMYQGAETDGALQYQGPGVELPFPHLDQTDPLLLLQLLHRYT
AVCLALKHTLRVDPATAVRLPARRILNTVCRALAVSSKSINLTGDGSVRLLVLPAIHTNTLEVLAGLITSVRGGLVQYAA
VLQRLFSQTLSSWTPLPEASVGQQRAFSSVRVSVYKTLALWVQVGGASTGVLQGSQTHSELLFNHLLGDITPSADSVKLR
GALSGEVVAGGKPGPRRTKQLVIADGMGPALQRKGDLVANQDSCLSALKALRHIILTSGTLLKDDVHKRLHDVVLPLCVR
LQQQQSSSAALETAGGVSGPYGSALSRRELYRLLLALVLVPSPSWPPPVSCATSILSKGRIDRNLKVSSFCAEALTVCNC
ILHPRTPSISLPLPPLALKPTPAAPVMPPSQGPGPGLTLPTTLLGSSAPGPPFFPSRHSLGLGPGPGPASLLGSLENHLSL
VPGLPGQGPGPGDLLLSPHAHHQPELGPPEGQRPVFVRYDKEEAEDVEISLDSDSDDSVVIVPPGMLGADGQQDEAAPPN
SHSVLSAAPGDAGAPPPGGDGASMAPPAAAPSTVDGSSLHNDLASSSPLLPTSTAPINSFPPSVASVVSLVPPLNSGALA
APPGGLGDPLPGRPQLQQMLMQPPPPGQPPSVGLPLQMHQLQNQLGPPGRHLHPHPPPPPANNEDSAVININSTDEEDED
EEDMEDEEELEEEEDEGMDEEDEEEEVSDIAEDEFYDGEEYEDYDEEDGEELEEEEEEDEEGGIPPLEGGDDKESETEDG
KVLRAPVDEGGPSGFGVEGEPEAGIEEVQTSRTLFGEDRMKVQEVESIGVLEAAREAEGEAEEDADRMDDPTMPQILCVT
GGTMEEQEEEPGDAAAPEEEEEEEAAAGGSWEGGAKPGEPSPAAEEERTAGQQEPAVQLVTDADMAATPPGGRQEQEEPP
AAPPPPSSSSSSCAAEQLTQAEKEEEGEEEEEEPAGEGGDGGESDGQEGKGLKRKREEAPIEEEEEEGQSTEKKQVRFPS
> Sinocyclocheilus graham [A0A672PS34]
MASAAWLHGHNITRLTEGLVSVLKEERPEYLPALLANYREHGVVGTQSTGAVGGLVGISNARLGSSKTRFEGLCLLSVLV
KDSSNEVFQQHCLSWLRTLQQVIQSQAPLPTVQLAVSVLQDMLQYSSQLPELGREVGLNSILGILTSLLSLKSECHLAAM
KGMMACMIYYPRACGSLREKLGAYFLSKMDSDNPKVQEVACESFGRLPCLGGVLERGGGGRRAEGWTNQLHCLLASANSM
LGQLYQGAETEGTVQYEGPGIELPFPPLDDVDPLLILQLHHRYKAVCLAIKHTLSADPASSVRLPVQHVLKLLCQALAVN
TKNISPTGEGCLKLLVLPSIHNDTLELLSALIKAVGGGLVQYSSVLTRLLSQSLSAWTPLPEASLGQQRAYSAVRVTVYR
TIELWVRVGGAALLQGSPTHTELLFTHLIGDITPASEAVKLRSGQQSQSMNDLIGSADKSGPRRTKGLGMGDGISLQRKG
DVLANQDTCVAALRALRQIILTSGTLLKEDLHKRIQDLVVPLCVRLQQQSHCVLDVGAVSGQYGSPTPRRELYRLLLALV

LVPSPRWPPPLSCAVSAFSHGRRDHNIMVSSFCVEALTICNTLIHPRTPSISLPLTPLTLKSTPSASVLASGQNPSLSIP
TLLGGPATGPPFPSRHSMGLGPATLFGSLENHLPLAPPVLPTPAATTATPGDLLLSPAQPGELAGLGAPEGQRQVFVRYD
KEEPEDVEISLESDSDDSVVIMPAGMMMEMQDGAANAQSLSQPAVPAVGGLQSSAPIVGEVGSVDTLLPNELPTSIPHQI
LQANANNINSFPGPSQTAQLVSLVPPLNSTTASLSASPAGLADSLTGGPQLQQMLMQTSPGGQPPTLGLSLQMQLQNQIA
QTSRQLQQQHPANEVDQNVININSSDDEEEEEEELEDEFGEEEEEEEEEGLEEEEEEEEGSDFMDEECCEGEEFDEDEDDE
DEEESEEILPLDGDNSRGMMGEEEAEVMIEEEDQQGMEMFCMEREQEVEPGIEEMEGGVRSVYADDRIKDKGTVEEIENI
GAVERNEPVVGKEQIESLVISGETEGHEEDTRVEAVEPEVKTCEQEVAKPEDPTENAGPSQQGQELTVEDEVQKQEPELQ
TEDTTNQSTPSTSEQEALQSVAVTAEVEKESGEQGEDSDARGTKRKMEDHEEGESSEQGTEKKKMDEAMASMLADFVDCP
PDDDDHGASQSQS
> Red-bellied piranha [A0A3B4CMZ4]
MATAAWLHGPNNTRLTEGLLSVLKEERPEYLPALLANYREHGVVGSQVRRSSSLLHGLANRLALAAQFEGLCLLSVLVKD
SSSEVFHQHCLSWLRSLQQIIQSQAPLPSVQLAVSVLQDLLQYSSQLPELAREVGLNSILGILTSLLGLKSECHLAAMEG
MMACMRYYPRACGSLKEKLGVYFLSKMDSDNPKVQEVACECYGRLPCLGGVLERGGGGRRAEGWTNQVHCLLASANSILG
QLYQGIETEETIQYDGPGVELPFPPLDDIDPLLILQLRQRYKAVCLALKHTLSVDPATPVRISIQHVLNLVCRALAVTNK
SINVTGEGCLKLLVLPSIHSDSLELLSALIKAVGGGLVQYCSVLTRLFSQSLSAWTPLPEASLGQQRAYSTVRVAIYCTL
ELWVHVGGASSSVLQGSTSHSEMLFAHLMGDITPGTEAVKLRAGQTALSDFVGAAGKAGPRRTKGLGIGDQAGVSLQRKG
DALANQDTCFAALRALRQIILTSGTVLKEDLHKRLQDLVVPLCVRLQQQAQCGAEVAGVSGQYGSASPRRELYRLLLALI
LVPSPRWPPPLSCAVSVFSQGRRDRSIMVSSFCAEALTICNTLLHPRSPSISLPLPPLTLKPTPAAPVLGSTQNPSLSLP
TLLGGPAPGPSFPARHPLGLGPVALLGTLENHLPLAPPVLPTPPGATGAQSDLLLSPAQPAELAGLGAPEGQRQVFVRYD
KEEPEDVEISLESDSDDSVVIMPQGMMLEMQEGASNAQTLPAPSGGTMPTPAEVGSVETSLPNELPTSISHQILPTDSNN
INSFSGPGQADQLVSLVPPLNSTAVSLAASPGGLGNSLPAGPQLQQMLMQPSPGGQSAQLGLSLHMQLQSQLAQTSRQLQ
QQQPSSAPEEDQNVININSSDDEEEEDEEMEDEDELGEEEEEEEGLEEDEEEEEGSDFPEEEEFYPGEEYEDFEEEEGEE
MEEEEEEEEEEEVQPLEGEDQRAVMGEEEGEVMIEEQEERRIDAFHVEREGQVEAGIEEMEGVRSIYGEEGMKDKVSVEE
IENIGAVERNESEASEPQIEARVIVDEAEEVREGTSAAATAAAAPPEQDVRPWEQEGTGQEPVPEAPEDSAIPKESQEVA
AEAGVSEQSAEGEQEETANQSTASTSEEVPSQQAEEAAVKEKDEGQQEEEEEARGTKRKIEDCEEGERSEQGSEKKKLDD
EAMASMLADFVDCPPDDEDHGASQSHS
> Carassius auratus [A0A6P6KIU3]
MASVAWLHGPNITRLTEGLVTVLKEDRPEYLPALLANYREHGVVGTQSTGAVGGLVGISNARLGSSKTRFEGLCLLSMLV
KDSSSEVFQQHCLSWLRTLQQVIQSQAPLPTVQLAVSVLQDLLQYSSQMPELAREVGLNSILGILTSLLSLKSECHLAAM
KGMMACMLYYPRACGSLREKLGAYFLSKMDSDNPKVQEVACESFGRLPCLGGVLERGGGGRRAEGWTNQLHCLLASASSM
LGQLYQGAETEGPVQYEGPGVELPFPPLDDVDPLLILQLHHRYKAICLAIKHTLSADPASSVRLPVQHVLKLVCQALAVN
TKSISPTGEGCLKLLVLPSIHNDTLELLSALIKAVGGGLVQYSSVLTRLLSQSLSAWTPLPEASLGQQRAYSAVRVTVYR
TIELWVRVGGASLLQGSPTHTELLFTHLMGDITPASEAVRLRSGQQSQSMNDLIGSAGKSGPRRTKGLVMGDGISLQRKG
DVLANQDTCVAALRALRQIILTSGTLLKEDLHKRIQDLVVPLCVRLQQQSRCVLDVGAVSGQYGSPTPRRELYRLLLALV
LVPSSRWPPPLSCAVSVFSHGRRDHNIMVSSFCAEALTICNTLIHPRTPSISLPLTPLTLKSAPVLASGQNHSLSIPNLL
GGPPFPSRHPMGLGPATLLGSLENHLPMAPPVLPTPAGTTATPGDLLLSTAQPSELAGLGAPEGQRQVFVRYEKEEPEDV
EISLESDSDDSVVIMPAGMMMETQDGAANALSLSQPAVPTPIVGEVGSVDTLLPNDLQTTIPHQILPANANNINSFPGPS
LTSQLVSLVPPLNSTTASLSASPAGLADSLTGGPQLQQMLMQTSPGGQPPTLGLSLQIQLQNQIAQTSRQLQQQPPANEV
DQTVININSSDDDEEEELEEDELGEEEEEEEGLEEEEEEEEEGSDLMDEEYYEGEEFEEYEEEDDEEESEEIQPLEGDNSS
GMMGEEDAEVMIEAEDQQGMEMFCMERERQVEPGIEEMEGVRSVYAEDRIKDKGTVEEIENIGAVERNEPVVGKEQIESL
VISGETEGHEEDTTVEAVEPEVKTCEQEVAKPEDPPDNDRPSQQDQELTVEVQKQEPELQPEDATNQSAPSTSEQEAQQS
VAVTEEEEVKKETGEQVEDSDAKGTKRKMEDREEGESSEQGTEKKKMDEAMACMLADFVDCPPDDDDHGSSQSQS
> Hucho hucho [A0A4W5RB61]
MAATAAWMHGPSNMRLTEGLLSVLKEQRPEYLPALLANYREHGVFSTQSTGAVGGLVGLSNAKLGNSKTKFEGLCLLSVL
VKDSSSDVFQQHCLSWLRSLQQVIQSQDPLPSVQLAVGVLQDLIQYSSQLPELAREVGLNSVLGILTSLLGLKSECHLAA
MEGMTACMTFYPRACGSLRDKLGAYFLSKMDSANPKVQEVACECYGRLTCLGGVVERGGGGRRAEGWTNQLHCLLASANG
ILAQLYQGSESEGTVQYEGPGVELPYPPLDDADPLLLLQLQHRYRAACLALKHTLGVDPASAVRLPVQQVLNLVCRALAV
SSKSINVTGDGCVRLLVLPAIHKDTLQVLHALITAVGSGLVQYSSVLQRLFSQTLSAWTPLPETSLGQQRAFSAVRVSLY
RTLELWVRVGGASASVLQGSPTHSELLLAHLLGDITPGADSVRPSSSPQLRDVVSSKPCPKSRKPGLAMSNGGRASFQRK
GDSLANQDTCLSALRALRQVIVTSGSLLKEDIHKRLHDAVLPLCVRLQQQHGANWNDSGAGGLSGQYGSAPPRRELYRLL
LALVLVPPPRWPPPLTCTVSILSHGRRDRSLKVSSFCTEALTICNSLLHPRTPSLSFPLKPTPSQASNLTLLGGPTPGPP
FPSSLDNHLPLVHPGLSGSQRPVFIRYDKEEAEDVEISLESDSEDSVVIVPQGMLMLESQDGTSTAATLPGSSLAPGGVT
LPVPGSGGELGGDISLSNDLPSTSLPHPLLPSSTPNSTNSFPPALLVSLVPPLNSTSVAQLGEPSVGLGVGADSLPGAQL
QQMLLQSQPPAPGQPTALGLPIQVHQNQLAQSSRALQQQQQSIDEDHTVININSSDEEEEDELEEEEDEEEEESDFPDEE
EYYDEELDDYEAEEGEEMEDEEEREIRLLDGEGRGGMGGREEGEVLRGLAEEGGIGGFRVEREGEGGIKAQEVESIGVLE
EEREGEEDDGEGINDPTMPQILCVTSGALREREELDEEGAGHGQKVRSWEQKGGNRPEGVSSSEEGPAAQHKQEAEPAPE
VRVDGDDQPSNQEGEISKRGIDNTGQETAPRETKGEGPEREKERKEGEDGRGMKRKREGEEEGTGQGTEKIKVRKGLSNT
LFKKCPYNHCTTQASLYCTTHIFPMCYMDMA
> Sinocyclocheilus rhinocerous [A0A673IVK1]
MASAAWLHGPNITRLTEGVVSVLKEDRPEYLPALLANYREHGVVGTQSTGAVGGLVGISNARLGSSKTRFEGLCLLSVLV
KDSSNEVFQQHCLSWLRTLQQVIQSQAPLPTAQLAVSVLQDMLQYSSQLPELAREVGLNSILGILTSLLSLKSECHLAAM
KGMMACMIYYPRACGSLREKLGAYFLSKMDSDNPKVQEVACESFGRLPCLGGVLERGGGGRRAEGWTNQLHCLLASANSM
LGQLYQGAETEGTVQYEGPGVELPFPPLDDVDPLLILQLHHRYKAVCLAIKHTLSADPASSVRLPVQHVLKLVCQALAVN
TKSISPTGEGCLKLLVLPSIHNDTLELLSALIKAVGGGLVQYSGVLTRLLSQSLSAWTPLPEASLGQQRAYSAVRVTVYR
TIELWVRVGGASLLQASPTHTEFLFTHLMGDITPASEAVKLRSGQLSQSMNDLIGSAGKSGPHRTKGLGMGDGISLQRKG
DVLANQDTCVAALRALRQIILTSGTLLKEDLHKRIQDLVVPLCVRLQQQSHCVLDVGAVSGQYGSPTPRRELYRLLLALV
LVPSPRWPPPLSCAVSAFSHGRRDHNIMVSSFCAEALTICNTLIHPRTPSISLPLTPLTLKSTPSASVLASGQNPSLSIP
TLLGGPATGPPFPSRNPMGLGPATLFGSLENHLPLAPPVLQTPAATTATPGDLLLSPAQPGELAGLGAPEGQRQVFVRYD
KEEPEDVEISLESDSDDSVVIMPAGMMMEMQDGAANAQSLSQPAVPAVGGLQSSAPIVGEVGSVDTLLPNELPTSIPHQI
LPANASNINSFPGPSQTAQLVSLVPPLNSNTASLSASPAGLADSLTGGPQLQQMLMQTSPGRQPPTLGLSLQMQLQNQIA
QTSRQLQQQQPANEVDQNVININSSDDEEEELEEEDELGEEEEEEEGLEEEEEEEEGSDFMDEEYCEGEEFDEYEDDEDE
EESEEILPLDGDNSRGMMGEEEAEVMIEEEDQQGMEMFCMEREQEVEPGIEEMEGGVRSVYADDRIKDKGTVEEIENIGA
VERNEPVVGKEQIESLVISGETEGHEEDTRVEAVEPEVKTCEQEVAKPEDPTENAGPSQQGQELTVEDEVQKQEPELQTE
DTPNQSTPSTSEQEALQSVAVTVEEEVEKESGEHGEDSDARGTKRKMEDCEEGESSEQGIEKKKMDEAMASMLADFVDCP
PDDDDHGASQSQS
> Takifugu bimaculatus [A0A4Z2BNV1]
MATSAWLRGPSAMRLTEGLVSVLKEQRPDYIPVLLTGYREHGVFHTQGASAVAGLVGFSNAKLGSSKTRFEGLCLLSMLV

KDSSSDLFQQHCLSWLRSLQQIVQSQAPVQTIQLAVNILKDVLQYSSQIPELAREVGLNSILGILTSLLGLKTECELAAM
EGMTACMTHYPRACGSLKDKLGAYFLSKMDSTNKKTQEMACQCYAHLPCLGGVLDRGAGAGRAEGWTNQIHCLLASANSH
LALIYQGAEMEGTMQYEGPGVELAFPLLDQSDPLFLLQLQHRFTAVCLALRHTLRVDPASAVRIPVRPILNLVCRALAVS
CKSFNLTGDGNVRLLILPIIHLNILEVLAALIIAVRSSMVQYAAVLQRLFSQTLSAWTPAAEASVGQQRAFSSVRVSVYR
TLELWLQVAGASTSILHGSPNHSEILFNHLLSDITPGAESVKLRVGLSAEIVPGGKPGPRRTKSLVISDTVGPSLQRKGD
IMANQDTCLTALRALRQIILVSGTLLKDDIHKRLHDVLLPLCVRLQQQQLSSNMSCDSTAGISGQYSSALTRRELYRLLL
ALVLVPSPCWPPPLTCVVSILSSGRTDRNLKVSTFCCEALTICNSLLHPRSPSIALPMPPLSIKPAHAVSVLPTPQASTP
GLTLPTLLGEPTPPPPFPSPHTLGMGPSSLLGSLENHLSLVPGLRGQTSGPSEMILSPHAHHQDLAGLGPPEGQRPVFVR
YDREEAEDVEISLASDSDDSVVIVPPGMLNMENQQDDVAAANSQSMTAAAGGAAVTLAEGEPVTMVSNTATAAPIDGVSL
PNDLTTSAPLLTTSAPPINSFPPSSASVVSLVPALNSNPLTAPPGGLG
> Pangasianodon hypophthalmus [A0A5N5PI34]
MATAAWLHGPKNMRLTEGLVSALKEERPEYLPALLANYREHGVVGTQSCGTVGGLVGISNSCLGSSKTRFEGLCLLSMLV
KDSSSEVFQQHCLSWLRSLQQIIQSQAPLPSVQLAVSVLQDVLHYSSQLPELAREVGLNSILGILTSLLGLKSECHLAAM
EGLMACMTYYPRACGSLKEKLGVYFLSKMDSDNPKVQDVACECYGRLPCLGGVLERGGSGRRAEGWTSQVHCLLATANSI
LGKLYQGIETEETIQYEGPGVELPFPPLDDVDPLLILQLRHRYRAVCLALKHTLSVDPATSVRLSIQHVLNVVCRALAVS
IKNINVTSEGCLKLLVLPSIHSDSLEVLSALIKAVGAGLVQYCNVLSRLFSQALCAWMPLPEASLGQQRAYSAVRVALYH
TLELWVRVGRASSSLLQGSSSHSELLFAQLIGDITPGTEAVKLRAGQTAMSDLVGTAGKTGPRRTKGLGIGDPGGVSLQR
KGDVLANQDTCFAALRVLRQIILTSGTLLKEDLHKKLQELVVPLCVRLQQQAQCSNWDVGSVSGQYGSAAPRCELYALLL
ALVLVPSPRWPAPLSCAVCVFSQGRRDRNITVSSFCAEALTICNALLHPRTPSISLPLLTLKPTPAASVLAPTQNPSLSL
PTVLGAPAPGPSFAPRHPLSLGPASILGSLENHLPLGPPVLPTPAGATGAQGELLLSSPAQAADLAGLAAPPETQRQVFV
RYDKEEPEDVEISLESDSDDSVVIMPQGMMLEMQEGAANTQSLPPPTGSAMPVPNAGLGSEAGPVETSLSSDLPTSIGHQ
MLPADANNINSFPGSSQTEQLVSLVPPLNSNAVPLAASSGALGNSLPAGAQLQQMLMQPSPGGQTSQLGLSLHMQLQNQL
VQSSRQPAANEQDQNVININSTDDEEEEEDEEMEDEDELGEEEEEEEEGLEEDEEEEEGSDYHEDGYYPGEEFDGFDDDDE
GEEMEEEEEEEDDEEETQPLEADNRRDVIDAEEGEVLIEGQEERGVDTFPMEGERPVEGGIEEMKAVQSIYEEEGLKDKG
GIEEIENIGAVERNESVVGEQQIETHVIGAEGEQSEENVSVEASDQELQPQEQEGSRSESVVTPEDSGIPPEGQEQEVAI
EVRNNEPSAEAEKEEIPNPSTATTSEDTAPQQVVETAEKGVVEGTQVEEDEARGTKRKIEDREEGEVSEQSSEKKKLDDE
AMASMLADFVDCPPDDEDHGASHSHS
> Sinocyclocheilus anshuiensis [A0A671N4S6]
MASAAWLHGPNITRLTEGLVSVLKEDRPEYLPALLANYREHGVVGTQSTGAVGGLVGISNARLGSSKTRFEGLCLLSVLV
KDSSNEVFQQHCLSWLRTLQQVIQSQAPIPTVHLAVSVLQDMLQYSSQLPELAREVGLNSILGILTSLLSLKSECHLAAM
KGMMACMIYYPRACGSLREKLGAYFLSKMDSDNPKVQEVACESFGRLPCLGGVLERGGGGRRAEGWTNQLHCLLASANSM
LGQLHQGAETEGTVQYEGPGVELPFPPLDDVDPLLILQLHHRYKAVCLAIKHTLSADPASSVRLPVQHVLKLVCQALAVN
TKSISPTGEGCLKLLVLPSIHNDTLELLSALIKAVGVGLVQYSSVLTRLLSQSLSAWTPLPEASLGQQRAYSAVRVTVYR
TIELWVRVGGASLLQGSPTHTELLFSHLMGDITPASEAVKLRSGQQSQSMNDLIGSAGKSGPRRTKGLGMGDGISLQRKG
DVLANQDTCVAALRALRQIILTSGTLLKEDLHKRIQDLVVPLCVRLQQQSLCVLDVGAVSGQYGSPTPRRELYRLLLSLV
LVPSPLWPPPLSCAVSAFSHGRRDHNIMVSSFCAEALTICNTLIHPRTPSISLPLTPLTLKSTPSASGQNPSLSIPTLLG
GPATGPPFPSRHPMGLGPATLFGSLENHLPLAPPVLPTPAATTATPGDLLLSPAQPGELAGLGAPEGQRQVFVRYDKEEP
EDVEISLESDSDDSVVIMPAGMMMEMQDGAANAQSLSQPAVPAVGGLQSSAPIVGEVGSVDTLLPSELPTSIPHQILPAN
ANNINSFPGPSQTAQLVSLVPPLNSTTASLSASPAGLADSLTGGPQLQQMLMQTSPGGQPPTLGLSLQMQLQNQIAQTSR
QLQQQHPANEVDQNVININSSDDEEEEGEELEEEEDELGEEEEEEEEGLEEEEEEEEEGSDFMDEEYCEGEEFDEYEDEDEE
ESEEILPLDGDNSRGMMGEEEAEVMIEEEDQQGMEMFCMEREQVEPGIEEMEGGVRSVYADDRIKDKGTVEEIENIGAVE
RNEPVVGKEQIVSLVISGETEGHEEDTRVEAVEPEVKTCEQEVAKPEDPTEDAGPSQQGQELTVEDQVQKQEPELQTEDT
TNQSAPSTSEQEALQSVAVTAEVEEEVEKESGEQGEDSDARGTKRKMEDREEGESSEQGTEKKKMDEAMASMLADFVDCP
PDDDDHSASQSQS
> Swamp eel [A0A3Q3IK80]
MTSRSRTVVTFAWSRGKMATSPWLHGPSAMRLTEGLVSVLKEHRPEYLPILLASYREYGVLPTQGSSALGGIVGFSNAKL
GSSKTRFEGLCLLSMLVKDSSSDLFQQHCLSWLRSLQQVIQSQAPVQTIQLTMNILKDLLQYSSQQAELAREVGLNYILG
FLTSLLSLKTECELAAMEGMTACMTYYPRACGSLRDKLGAYFLSKMESTNKKTQEMACQCYGRLPCLGGVLDRGVSAGRA
EGWTKQIHCLLASANNLLAQIYQGSETDEMVKYEGLGVELAFPLLDQSDPLLLLQLQHRYTAVCLALKHTLRLDPSSAVR
LPVRPILNLVCRALAVSSKSINLTGDGSVRMLVLPIIHINTLAVLAALVTAVHSGMIQYATVLQRLFSQTLSAWTPLPEA
TLGQQRAYSSVRASVYRTLELWVQVAGASASILQGGPGHLELLFNHLLSDITPGAESVKLRAGLSADVVPGGKPGPRRTK
PLVIADAGGPSLQRKGDPLANQDTCLSALRVLRQIIMTSGTLLKDDIHKRLHDVVLPLCVRLQQQQSSSSTACESAGAIS
GQYSSALSRRELYRLLLALVLVPSPCWPPPLTCAVSILSSGLTDGNLKVSTFCTEALTICNSLLHPRTPSIALPLPPLTL
KPTPTAPALPSSQGPTPGLTLPTLLGGPASGPPFPTRHSLGLGSTSLLGSLENHLSLVPGLPGQAPTPGDMILSPHTHHQ
PDPTGLGPPEGQRPVFVRYEKEEAEDVEISLASDSDDSVVIVPPGMLNIENQQDDTAASANSQTMASVVPGGTTVTLQGG
ESVSTVPTTAATTTIDGVSLSNNLVTSAPLLTTSATPINSFPPSSTSVVSLVPPLNSSTLTVTPGSLGDSLPGRPQLQQM
LMQSSTTGQPSSMGLPLQMHQLQNQQGRHLHQHQPPPASNEDSAIININSTDEEEEEEDDMEDDEELEEEEEGMDEEDEE
EEVSDFADEEFYDGEEYEEYDEEEGEELEEEEEEEEEEEDGDIPPLEGAEDKAGEAGTEDGKVLQAAVDERGMVGFNVEGE
TEGGIEEIQTNRELFREDRMTVQEVESIGVLEEAQEGQGEEDESERMDDPTMPQILCVTGGALEQREETEEEVVEARAGV
VQEEVSSWEQGTNEMELTASSDESIFTRNQQDPEAEPQKEANMSDNQPPSHHEEQQAVVREEAATDSETFNVLNTTEQEE
TDAEKNNKVEEEQQETEREGGRESDGDEGKGVKRKREEAPIEEKAGQSTEKKKLDDEAMATMLADFVACPPDDEDGASGL
NHS
> Carassius auratus [A0A6P6KKR5]
MASVAWLHGPNITRLTEGLVTVLKEDRPEYLPALLANYREHGVVGTQSTGAVGGLVGISNARLGSSKTRFEGLCLLSMLV
KDSSSEVFQQHCLSWLRTLQQVIQSQAPLPTVQLAVSVLQDLLQYSSQMPELAREVGLNSILGILTSLLSLKSECHLAAM
KGMMACMLYYPRACGSLREKLGAYFLSKMDSDNPKVQEVACESFGRLPCLGGVLERGGGGRRAEGWTNQLHCLLASASSM
LGQLYQEGPVQYEGPGVELPFPPLDDVDPLLILQLHHRYKAICLAIKHTLSADPASSVRLPVQHVLKLVCQALAVNTKSI
SPTGEGCLKLLVLPSIHNDTLELLSALIKAVGGGLVQYSSVLTRLLSQSLSAWTPLPEASLGQQRAYSAVRVTVYRTIEL
WVRVGGASLLQGSPTHTELLFTHLMGDITPASEAVRLRSGQQSQSMNDLIGSAGKSGPRRTKGLVMGDGISLQRKGDVLA
NQDTCVAALRALRQIILTSGTLLKEDLHKRIQDLVVPLCVRLQQQSRCVLDVGAVSGQYGSPTPRRELYRLLLALVLVPS
SRWPPPLSCAVSVFSHGRRDHNIMVSSFCAEALTICNTLIHPRTPSISLPLTPLTLKSAPVLASGQNHSLSIPNLLGGPP
FPSRHPMGLGPATLLGSLENHLPMAPPVLPTPAGTTATPGDLLLSTAQPSELAGLGAPEGQRQVFVRYEKEEPEDVEISL
ESDSDDSVVIMPAGMMMETQDGAANALSLSQPAVPTPIVGEVGSVDTLLPNDLQTTIPHQILPANANNINSFPGPSLTSQ
LVSLVPPLNSTTASLSASPAGLADSLTGGPQLQQMLMQTSPGGQPPTLGLSLQIQLQNQIAQTSRQLQQQPPANEVDQTV
INWINSSDDDEEEELEEDELGEEEEEEEEGLEEEEEEEEEGSDLMDEEYYEGEEFEEYEEEDDEEESEEIQPLEGDNSSGMMG
EEDAEVMIEAEDQQGMEMFCMERERQVEPGIEEMEGVRSVYAEDRIKDKGTVEEIENIGAVERNEPVVGKEQIESLVISG
ETEGHEEDTTVEAVEPEVKTCEQEVAKPEDPPDNDRPSQQDQELTVEVQKQEPELQPEDATNQSAPSTSEQEAQQSVAVT

EEEEVKKETGEQVEDSDAKGTKRKMEDREEGESSEQGTEKKKMDEAMACMLADFVDCPPDDDDHGSSQSQS
> Xiphophorus maculatus [M4A137]
MATSAWMHGAAAMRLTEGLVSVLKEQRPEYLSEVLTNYREHGVFPTQGASDVAGLVGFCNAKLSSSKTRLEGLCLLSMLV
KDSSSDLFQQHCLSWLRSLQQVIQSQAPCQSIQLAVSILQDLLQYSCQLAELAREVGLNSILGILMSLLGLKTECERAAM
EGMRACMIYYPRACGSLKDKLGAYLLSKMDSTSRKTQEMACQGYSLLPCLGGLADRSAGTGRAEGWTNQIQCLLASANGI
LAQIYQGSETDEAVQYRGPGVELPFPHLDQTDPLLLLQLQHRYTAVCLVLKHTLRADPASAVHLPVRPILNLVCRALAVN
SRNTSLTADGSLRLLVLPSVHISTLEVLAELITVVRSGMVQYAAVLQKLFSQMLCAWTAVPEANVGQQRAYSSVRVSVYR
TLELWLQLVGASANVLQGASSHAELLFTHLLADITPGAESIKLRAGLSADVVPGGKPGPRRTKQLVMADPVGASLQRKGD
PVSNQDTCLSALKVLRRIIQTSGTLLKNDIHKRLHEVVLPLCVRLQQQQTSSITACESVGGVSGQYSSALTRRQLYRLLL
ALVLVPSPCWPPPLTCTVSILSNGRLDRNLKVSSFCSEALTICNSLLHPRVPSIALPLPPLALKPPPTASVLSGPGPAPR
LTLPTLLGGPTPGPPFPGRHPLSLGPNSLLGSLENHLSLVPGMPGPASAPGDLLLSPHGQPQPDPAGLGLPDGQRPVFVR
YDKEEAEDVEISLASDSDDSVVIVPPGMLGSVSQLDDPAPGSQNLLSAGAGAGVPLPGGDTVAMVPSTAAPASLDVTSLP
NDLVPTSSTPANSFPSSSGSVVSLVTSLTPGSAPPPAGGLVDPVAVKPQLQQMLLQPPAPGPQGPPGSVALPLQVQLQNQ
LAQQGRHLHQHPPPAPPSNQDSAVININSTDDEDEDEEDMEDDEELEEEEEEDGLDEEEEGSEDFYEGEEYEDFDDEEAE
ELEEEEEDEDGDMPPLEGSEDKSEEVTMEEEEEEEEEEKALGAEEEEGEGAGFNPEGGIEELQSSRALFGEERMKVQEVE
SIGVLEEARGGDRGDGEDDSERMDDPSMPQILCVTGGALEERADSSQEAAQAAGELQDQLTLWDQNHQQNPPTGASEEEP
TAHSQEESVAEREQEAGVREEVALEAELQEEEEPVVVQEEAAAGASGGEETPAAPHDPQQDQTDTQQGYETQAEWTKPVG
EQGEKEEESNTEEEEKGMKRKREEEVIEDEARPSPEKKKQDDEAMASMLADFIACPPDDEDVASGSNQS
> Sinocyclocheilus graham [A0A672PQ99]
MASAAWLHGHNITRLTEGLVSVLKEERPEYLPALLANYREHGVVGTQVGLKFFLVVRFEGLCLLSVLVKDSSNEVFQQHC
LSWLRTLQQVIQSQAPLPTVQLAVSVLQDMLQYSSQLPELGREVGLNSILGILTSLLSLKSECHLAAMKGMMACMIYYPR
ACGSLREKLGAYFLSKMDSDNPKVQEVACESFGRLPCLGGVLERGGGGRRAEGWTNQLHCLLASANSMLGQLYQGAETEG
TVQYEGPGIELPFPPLDDVDPLLILQLHHRYKAVCLAIKHTLSADPASSVRLPVQHVLKLLCQALAVNTKNISPTGEGCL
KLLVLPSIHNDTLELLSALIKAVGGGLVQYSSVLTRLLSQSLSAWTPLPEASLGQQRAYSAVRVTVYRTIELWVRVGGAA
LLQGSPTHTELLFTHLIGDITPASEAVKLRSGQQSQSMNDLIGSADKSGPRRTKGLGMGDGISLQRKGDVLANQDTCVAA
LRALRQIILTSGTLLKEDLHKRIQDLVVPLCVRLQQQSHCVLDVGAVSGQYGSPTPRRELYRLLLALVLVPSPRWPPPLS
CAVSAFSHGRRDHNIMVSSFCVEALTICNTLIHPRTPSISLPLTPLTLKSTPSASVLASGQNPSLSIPTLLGGPATGPPF
PSRHSMGLGPATLFGSLENHLPLAPPVLPTPAATTATPGDLLLSPAQPGELAGLGAPEGQRQVFVRYDKEEPEDVEISLE
SDSDDSVVIMPAGMMMEMQDGAANAQSLSQPAVPAVGGLQSSAPIVGEVGSVDTLLPNELPTSIPHQILQANANNINSFP
GPSQTAQLVSLVPPLNSTTASLSASPAGLADSLTGGPQLQQMLMQTSPGGQPPTLGLSLQMQLQNQIAQTSRQLQQQHPA
NEVDQNVININSSDDEEEEEEELEDEFGEEEEEEEEEGLEEEEEEEGSDFMDEECCEGEEFDEDEDDEDEEESEEILPLD
GDNSRGMMGEEEAEVMIEEEDQQGMEMFCMEREQEVEPGIEEMEGGVRSVYADDRIKDKGTVEEIENIGAVERNEPVVGK
EQIESLVISGETEGHEEDTRVEAVEPEVKTCEQEVAKPEDPTENAGPSQQGQELTVEDEVQKQEPELQTEDTTNQSTPST
SEQEALQSVAVTAEVEKESGEQGEDSDARGTKRKMEDHEEGESSEQGTEKKKMDEAMASMLADFVDCPPDDDDHGASQSQ
S
> Takifugu flavidus [A0A5C6NA05]
MATSAWLRGPSAMRLTEGLVSVLKEQRPDYIPVLLTGYREHGVFHTQGASAVAGLVGFSNAKLGSSKTRFEGLCLLSMLV
KDSSSDLFQQHCLSWLRSLQQIVQSQAPVQTIQLAVNILKDVLQYSSQIPELAREVGLNSILGILTSLLGLKTECELAAM
EGMTACMTHYPRACGSLKDKLGAYFLSKMDSTNKKTQEMACQCYAHLPCLGGVLDRGAGAGRAEGWTNQIHCLLASANSH
LALIYQGAEMGWIRLQQSEYPSDQSSTLCAEPSPVRSSMVQYAAVLQRLFSQTLSAWTPAAEASVGQQRAFSSVRVSVYR
TLELWLQVAGASTSILHGSPNHSEILFNHLLSDITPGAESVKLRVGLSAEIVPGGKPGPRRTKSLVISDTVGPSLQRKGD
IMANQDTCLTALRALRQIILVSGTLLKDDIHKRLHDVLLPLCVRLQQQQLSSNMSCDSTAGISGQYSSALTRRELYRLLL
ALVLVPSPCWPPPLTCVVSILSSGRTDRNLKVSTFCCEALTICNSLLHPRSPSIALPMPPLSIKPAHAVSVLPTPQASTP
GLTLPTLLGEPTPPPPFPSPHTLGMGPSSLLGSLENHLSLVPGLRGQTSGPSEMILSPHAHHQDLAGLGPPEGQRPVFVR
YDREEAEDVEISLASDSDDSVVIVPPGMLNMENQQDDVAAANSQSMTAAAGGAAVTLAEGEPVTMVPNTATAAPIDGVSL
PNDLTTSAPLLTTSAPPINSFPPSSASVVSLVPALNSNPLTAPPGGLVEPMPSRPQLQQMLMQPSAAVQQGPLSLPLQIH
QLQSQLGQQGRPLQHQPPVASNEDSGVININSTDDEEDDEDMEDDEEEEEEEEGVEDEEEEEDEVSDFADEEFYDGEDYEE
FDEDAEELEEEDEEEGDIPPLEGAEEQGEQEEVEQGGVLQAAVEAAEIVDFSVEGEAGGGIEEIQTKRALFPEDRMKVQE
VESIGVMEEARGEAEEDETERVCDPTMPQILCVTGGALEEKEEEGGRVQEDMSSWERDAKKVEPQGPSGEAAASTAEQEE
SGGEQQAGGSEERPSSLGEEQEVTVSEEDAALTHPGGSDESTPEPQETVTEKREAAAAAEQQELHAGGGEEGKGVKRKRE
ELQSENELEGSSKQADEDTMASMLADFVACPPDDDDGPSASNQS
> Sinocyclocheilus rhinocerous [A0A673ISI2]
MASAAWLHGPNITRLTEGVVSVLKEDRPEYLPALLANYREHGVVGTQVGLKFFLVVRFEGLCLLSVLVKDSSNEVFQQHC
LSWLRTLQQVIQSQAPLPTAQLAVSVLQDMLQYSSQLPELAREVGLNSILGILTSLLSLKSECHLAAMKGMMACMIYYPR
ACGSLREKLGAYFLSKMDSDNPKVQEVACESFGRLPCLGGVLERGGGGRRAEGWTNQLHCLLASANSMLGQLYQGAETEG
TVQYEGPGVELPFPPLDDVDPLLILQLHHRYKAVCLAIKHTLSADPASSVRLPVQHVLKLVCQALAVNTKSISPTGEGCL
KLLVLPSIHNDTLELLSALIKAVGGGLVQYSGVLTRLLSQSLSAWTPLPEASLGQQRAYSAVRVTVYRTIELWVRVGGAS
LLQASPTHTEFLFTHLMGDITPASEAVKLRSGQLSQSMNDLIGSAGKSGPHRTKGLGMGDGISLQRKGDVLANQDTCVAA
LRALRQIILTSGTLLKEDLHKRIQDLVVPLCVRLQQQSHCVLDVGAVSGQYGSPTPRRELYRLLLALVLVPSPRWPPPLS
CAVSAFSHGRRDHNIMVSSFCAEALTICNTLIHPRTPSISLPLTPLTLKSTPSASVLASGQNPSLSIPTLLGGPATGPPF
PSRNPMGLGPATLFGSLENHLPLAPPVLQTPAATTATPGDLLLSPAQPGELAGLGAPEGQRQVFVRYDKEEPEDVEISLE
SDSDDSVVIMPAGMMMEMQDGAANAQSLSQPAVPAVGGLQSSAPIVGEVGSVDTLLPNELPTSIPHQILPANASNINSFP
GPSQTAQLVSLVPPLNSNTASLSASPAGLADSLTGGPQLQQMLMQTSPGRQPPTLGLSLQMQLQNQIAQTSRQLQQQQPA
NEVDQNVININSSDDEEEELEEEDELGEEEEEEEGLEEEEEEEEGSDFMDEEYCEGEEFDEYEDDEDEEESEEILPLDGD
NSRGMMGEEEAEVMIEEEDQQGMEMFCMEREQEVEPGIEEMEGGVRSVYADDRIKDKGTVEEIENIGAVERNEPVVGKEQ
IESLVISGETEGHEEDTRVEAVEPEVKTCEQEVAKPEDPTENAGPSQQGQELTVEDEVQKQEPELQTEDTPNQSTPSTSE
QEALQSVAVTVEEEVEKESGEHGEDSDARGTKRKMEDCEEGESSEQGIEKKKMDEAMASMLADFVDCPPDDDDHGASQSQ
S
> Sinocyclocheilus anshuiensis [A0A671N3E1]
MASAAWLHGPNITRLTEGLVSVLKEDRPEYLPALLANYREHGVVGTQSTGAVGGLVGISNARLGSSKTRFEGLCLLSVLV
KDSSNEVFQQHCLSWLRTLQQVIQSQAPIPTVHLAVSVLQDMLQYSSQLPELAREVGLNSILGILTSLLSLKSECHLAAM
KGMMACMIYYPRACGSLREKLGAYFLSKMDSDNPKVQEVACESFGRLPCLGGVLERGGGGRRAEGWTNQLHCLLASANSM
LGQLHQGAETEGTVQYEGPGVELPFPPLDDVDPLLILQLHHRYKAVCLAIKHTLSADPASSVRLPVQHVLKLVCQALAVN
TKIAGVMCFVFFPSYSVGVGLVQYSSVLTRLLSQSLSAWTPLPEASLGQQRAYSAVRVTVYRTIELWVRVGGASLLQGSP
THTELLFSHLMGDITPASEAVKLRSGQQSQSMNDLIGSAGKSGPRRTKGLGMGDGISLQRKGDVLANQDTCVAALRALRQ
IILTSGTLLKEDLHKRIQDLVVPLCVRLQQQSLCVLDVGAVSGQYGSPTPRRELYRLLLSLVLVPSPLWPPPLSCAVSAF
SHGRRDHNIMVSSFCAEALTICNTLIHPRTPSISLPLTPLTLKSTPSASGQNPSLSIPTLLGGPATGPPFPSRHPMGLGP

ATLFGSLENHLPLAPPVLPTPAATTATPGDLLLSPAQPGELAGLGAPEGQRQVFVRYDKEEPEDVEISLESDSDDSVVIM
PAGMMMEMQDGAANAQSLSQPAVPAVGGLQSSAPIVGEVGSVDTLLPSELPTSIPHQILPANANNINSFPGPSQTAQLVS
LVPPLNSTTASLSASPAGLADSLTGGPQLQQMLMQTSPGGQPPTLGLSLQMQLQNQIAQTSRQLQQQHPANEVDQNVINI
NSSDDEEEEGEELEEEEDELGEEEEEEEGLEEEEEEEEGSDFMDEEYCEGEEFDEYEDEDEEESEEILPLDGDNSRGMMG
EEEAEVMIEEEDQQGMEMFCMEREQVEPGIEEMEGGVRSVYADDRIKDKGTVEEIENIGAVERNEPVVGKEQIVSLVISG
ETEGHEEDTRVEAVEPEVKTCEQEVAKPEDPTEDAGPSQQGQELTVEDQVQKQEPELQTEDTTNQSAPSTSEQEALQSVA
VTAEVEEEVEKESGEQGEDSDARGTKRKMEDREEGESSEQGTEKKKMDEAMASMLADFVDCPPDDDDHSASQSQS
> Labeo rohita [A0A498MPE7]
MASAAWLHGPNITRLTEGLVSVLKEDRPEYLPALLANYREHGVVGAQSTGAVGGLVGISNARLGSSKTRFEGLCLLSVLV
KDSSSEVFQQHCLSWLRTLQQVIQSQAPLPTVQLAVSVLQDLLQYSSQLPELAREVGLNSILGILTSLLSLKSECHLAAM
KGMIACMIYYPRACGSLREKLAAYFLSKMDSDNPKVQEVACECYGRLPCLGGVLERGGGGRRAEGWTNHLHCLLASANGM
LGQLYQGAEAVLVVDWSSIAVCLQGFSHSLCLHGHLCLRLVWASKERTGITVTPRMTKNECAVRVTVYRAIELWVRIGGA
SLLQGSPSHTELLFTHLMGDITPASEAVKLRSGQQSQSMNDLISSAGKSGPRRTKGLGMGDGISLQRKGDVLANQDTCVA
ALRALRQIILTSGTLLKEDLHKQQSHCVPEVGAVSGQYGSPAPRRELYRLLLALVLVPSPRWPPPLSCAVSAFSHGRRDR
NIMVSSFCAEALTICNTLIHPRTPSISLPLSPLTLKSTPSAPVLASGQNPSLSIPTLLGGPTAGPPFPARHPMGLGPASL
LGSLENHLPLAPPVVPTPAGTTATPGDLLLSPAQPGELPGLGAPEGQRQVFVRYDKEEPEDVEISLESDSDDSVVIMPVG
MMEMQDGAANAQSLSQPAVPAVGGLQPSAPVVGEVGSVDTSLPNELPTSIPHQILPANANSINSFPGPSQTAQLVSLVPP
LNSTTASLSASPAGLADSLTGGQQLQQMLMQTSPGGQPPTLGLSLQMQIQSQMAQNSRQLQQQPPANEVDQNVININSSD
DEEEEEELEEEDELGEEEEEEEGLEDEEEEEECSDFMEDEEYCEGEDFEDYDDEEDEDEEESEEIQPLEGDNGRGMIGEE
EAEVMIEAEEPQGIEMFCMEREREVEREVEPGIEEMEGVRSVYADDRIKDKATVEEIENIGAVERNEPVVDKEEIESLVI
GGDAEDAEGHEEDIRVEAVEPEAKTCEQEEVKPEDPAEDAGPSQQGQELTVEDEVQKQEPEIQPEDTTNQSAPSTSEQET
LQSVAETAEEEVEKESGEQGDDSDARGTKRKMEDREEGESSEQGTEKKKKDDEAMASMLADFVDCPPDDDDHGASQSQS
> Ameiurus melas [A0A7J6B2A8]
MATAAWLHGPKNVRLTEGLVSALKEERPEYLPALLADYREHGVVGTQNSGTVGGLVGISNSRLGSSKTRFEGLCLLSMLV
KDSSSEVFQQHCLSWLRSLQQIIQSQAPLPSVQLAVSVLQDVLQYSSQLPELAREVGLNSILGILTSLLGLKSDCHLAAM
EGMMACMTYYPRACGSLKEKLGVYFLSKMDSDNPKVQDVACECYGWLPCLGGVLERGGGGRRAEGWTSQVHCLLASANSI
LGQLYQGIETEETIQYEGPGVELPFPPLDEVDPLLILQLRHRYRAVCLALKHTLSVDPATSVRLPIQHVLNVVCRALAVS
IKNIALCAWTSLPEASFGQQRAYSAVRVALYRTLELWVRVGRASSSVLQGSSSHSELLFAHLIGDITPGTEAVKKLQELV
VPLCVKLQQQAQYSNWDVGSVGGQYGSAAPRCELYALLLALMLVPSPRWPAPLSCAVCVFSQGHRDHNITVSSFCAEALT
ICNALLHPRTPSISLPLPPLTLKPTPAASVLSSTQNPSLSLPTLMVAPRHPLSLSPASLLGSLENNLPLGPPVLPTSVGA
TGAQGDLLLSSLAQAAELAGLAAPPETQRQVFLRYDKEEPEDVEISLRK
> Branchiostoma floridae [C3ZET0]
MANVLLTSLSEKDGKTSLPWAIEVANEHQLLQSEQNAQDWVSHINTSLGTAKTRLEGLCLLGTVVQQCSAGTFIQHGTTW
IRMLTQVLQAYDSPLTLQMASHVLGSVVQQAAQYPEVAREVATTHIPTLVQCLLGAQDHQWFPSALEALQSCMKNFPGPC
GSSKGKVESLICGLMDTSQPRLSQLAQQTCPLLAGCGGGGAGGVKYTEAWAHLCDQVLGSLHQVLDHAYQDMETGLQTYS
VPQASLRLKTVPESDPARTFVLSTRFHNLCGCLEQLVSQEFPAVVRIPVPDILAFLCRALGVNPKMLFGKASMEHVLLMS
ALPKMHCSALSILEALIISCRSHLVPHASVISQLLVQTLGWTTSEEGVPGRQRPYSTLRSRAYTVLTVWLNVCSAASGVD
SHADVILQHVLKDATPQADTTKLKASRPGAQEQSKRQKKKQRGVDMTDQGLSGHMKVDSQANSDVCCAALGALKTVLEVV
GSIIKPSFHKETQEFVIPLLLKIHQNQSDPPIPYSCGRCRKGLYQLLLAAVLVLHPRWPPPTQCAVKIFSVGQQDYDLQV
SSFCREALLTCMSIIHPRASTLQSPVIIADTPIISKPSTNHLASNFQGLRRNATEEGTRKNDSHNGNLFTAPSFKSVSQS
EGMVEDKPSQSESTTHESIRIKSSEVPVSFPNKFRREEDIAGPSVIQLDGSSSSSEGQSSSEDSVEFLEDVRNNEADSST
LESGGPGGGVSSTERQQATDMDKMSGNEDVVLIQGRTKEGGTEVSADTTDLTIAGGMKRKYESDVEDEDGESQDTDLQAM
LAAFVPDSTPDGN
> Homo sapiens [O15451]
RLRLLLLESVSGLLQPRTGSAVAPVHPPNRSAPHLPGLMCLLRLHGSVGGAQNLSALGALVSLSNARLSSIKTRFEGLCL
LSLLVGESPTELFQQHCVSWLRSIQQVLQTQDPPATMELAVAVLRDLLRYAAQLPALFRDISMNHLPGLLTSLLGLRPEC
EQSALEGMKACMTYFPRACGSLKGKLASFFLSRVDALSPQLQQLACECYSRLPSLGAGFSQGLKHTESWEQELHSLLASL
HTLLGALYEGAETAPVQNEGPGVEMLLSSEDGDAHVLLQLRQRFSGLARCLGLMLSSEFGAPVSVPVQEILDFICRTLSV
SSKNISLHGDGPCGCCCCPLSTLKALDLLSALILACGSRLLRFGILIGRLLPQVLNSWSIGRDSLSPGQERPYSTVRTKV
YAILELWVQVCGASAGMLQGGASGEALLTHLLSDISPPADALKLRSPRGSPDGSLQTGKPSAPKKLKLDVGEAMAPPSHL
LLPVPCKPSPSASEKIALRSPLSCSEALVTCAALTHPRVPPLQPMGPTCPTPAPVPLLRPHRPSGPHRSILRAPCPQWAP
CPQQAPCPSAGPMPSAGPVPSEPWTSTTANLLGLLSRPSVCPPRLLPGPENHRAGSNEDPILAPSGTPPPTIPPDETFGG
RVPRPAFVHYDKEEASDVEISLESDSDDSVVIVPEGLPPLPPPPPSGATPPPIAPTGPPTASPPVPAKEEPEELPAAPGP
LPPPPPPPPPVPGPVTLPPPQLVPEGTPGGGGPPALEEDLTVININSSDEEEEEEGEEEEEEEEEEEEEEEEEEEEEEED
FEEEEEDEEEYFEEEEEEEEEFEEEFEEEEGELEEEEEEEDEEEEEEELEEVEDLEFGTAGGEVEEGAPPPPTLPPALPPP
ESPPKVQPEPEPEPEPGLLLEVEEPGTEEERGADTAPTLAPEALPSQGEVEREGESPAAGPPPQELVEEEPSXPPTLLEEET
EDGSDKVQPPPETPAEEEMETETEAEALQEKEQDDTAAMLADFIDCPPDDEKPPPPTEPDS
> Macaca mulatta [H9F0I7]
MAAAVLSGSSAGSAAGVPGGTGGLSAVNSGPRLRLLLLESVSGLLQPRTGSAVAPVHPPNRSAPHLPGLMCLLRLHGSVG
GAQNLSALGALVSLSNARLSSIKTRFEGLCLLSLLVGESPTELFQQHCVSWLRSIQQVLQTQDPPATMELAVAVLRDLLR
YAAQLPALFRDISMNHLPGLLTSLLGLRPECEQSALEGMKACMTYFPRACGSLKGKLASFFLSRVDALSPQLQQLACECY
SRLPSLGAGFSQGLKHTESWEQELHSLLASLHTLLGALYEGAETAPVQNEGPGVEMLLSSEDGDAHVLLRLRQRFSGLAR
CLGLMLSSEFGAPVSVPVQEILDFICRTLSVSSKNISLHGDGPLRLLLLPSIHLEALDLLSALILACGSRLLRFGILISR
LLPQVLNSWSIGRDSLSPGQERPYSTVRTKVYAVLELWVQVCGASAGMLQGGASGEALLTHLLSDISPPADALKLRSPRG
SPDGSLQTGKPSAPKKLKLDVGEAMAPPSHRKGDSNANSDVCAAALKGLSRTILMCGPLIKEETHRRLHDLVLPLVMGVQ
QGEVLGSSPYTSSRCRRELYCLLLALLLAPSPRCPPPLACALQAFSLGQREDSLEVSSFCSEALVTCAALTHPRVPPLQP
MGPTCPTPAPVPPPEAPSPFRAPPFHPPGPMPSVGPMPSAGPMPSAGPMPSAGPVPSARPGPPTTANHLGLSVSGLVSVP
PRLLPGPENHRSGSNEDPILAPSGTPPPTIPPDETFGGRVPRPAFVHYDKEEASDVEISLESDSDDSVVIVPEGLPPLPP
PPPSGATPP
> Poeciliopsis prolifica [A0A0S7LEH7]
GLVSVLREQHPEYLSDVLANYREHGVFLNQGASDVAGLVGFSNAKLSSSKTRFQGLCLLS
MLVKDSSSDLFQQHCLSWLRSVQQVIQSKAPCQSIQLAVSILQDLLQYSCQLPELAREVG
LNSILGILTSLLGLKTECELAAMEGMRACMVYYPRACGSLKHKLGAYFLSKMDSTSRKTQ
EMACQGYSRLPCLGGLVDRSVGTGRAEGWTNQIQCLLASANGILSQFYKGSETDEAVQYR
GPGVELPFPHLDQSDPLLLLQLQHRYTAVCLALKHTLRADPASAVYLPVRPILNLVCRAL
AVNPRNTSLTSDGSLRLLVLPSVHINTLEVLAELITVVRSGMVQYAAVIQKLFSQMLCAW
TAVPEANVGQQRAYSSLRVSVYRTLELWLQLVGVSANVLQGASSHAELLFTHLLADITPG

AESIKLRAGLSADVVPGGKPGPRRTKQLVMADPVGASVQRKGDPVSNQDTCLSALKVLRR
IIQTSGTLLKNDIHKRLHEVVLPLCVRLQQQQTSSITACESAGGISGQYSSALTRRQLYR
LLLALVLVPSPCWPPPLTCAVSILSNGRLDRNLKVSSFCSEALTICNSLLHPRTPSIALP
LPPLALKPPPTASVLSGPGPTPRLSLPTLLGGPAPGPPFPGRHPLSLGSNSLLGSLENHL
SLVPGMPGPASAPGDLLLSPHGQPQPDPAGLGPLDGQRPVFVRYDKEEAEDVEISLASDS
DDSVVIVPPGMLGTDSQLDDP
> Poeciliopsis prolifica [A0A0S7LEI8]
GLCAGVLSRFQGLCLLSMLVKDSSSDLFQQHCLSWLRSVQQVIQSKAPCQSIQLAVSILQDLLQYSCQLPELAREVGLNS
ILGILTSLLGLKTECELAAMEGMRACMVYYPRACGSLKHKLGAYFLSKMDSTSRKTQEMACQGYSRLPCLGGLVDRSVGT
GRAEGWTNQIQCLLASANGILSQFYKGSETDEAVQYRGPGVELPFPHLDQSDPLLLLQLQHRYTAVCLALKHTLRADPAS
AVYLPVRPILNLVCRALAVNPRNTSLTSDGSLRLLVLPSVHINTLEVLAELITVVRSGMVQYAAVIQKLFSQMLCAWTAV
PEANVGQQRAYSSLRVSVYRTLELWLQLVGVSANVLQGASSHAELLFTHLLADITPGAESIKLRAGLSADVVPGGKPGPR
RTKQLVMADPVGASVQRKGDPVSNQDTCLSALKVLRRIIQTSGTLLKNDIHKRLHEVVLPLCVRLQQQQTSSITACESAG
GISGQYSSALTRRQLYRLLLALVLVPSPCWPPPLTCAVSILSNGRLDRNLKVSSFCSEALTICNSLLHPRTPSIALPLPP
LALKPPPTASVLSGPGPTPRLSLPTLLGGPAPGPPFPGRHPLSLGSNSLLGSLENHLSLVPGMPGPASAPGDLLLSPHGQ
PQPDPAGLGPLDGQRPVFVRYDKEEAEDVEISLASDSDDSVVIVPPGMLGTDSQLDDP
> Poeciliopsis prolifica [A0A0S7LEQ1]
GLVSVLREQHPEYLSDVLANYREHGVFLNQGASDVAGLVGFSNAKLSSSKTRFQGLCLLSMLVKDSSSDLFQQHCLSWLR
SVQQVIQSKAPCQSIQLAVSILQDLLQYSCQLPELAREVGLNSILGILTSLLGLKTECELAAMEGMRACMVYYPRACGSL
KHKLGAYFLSKMDSTSRKTQEMACQGYSRLPCLGGLVDRSVGTGRAEGWTNQIQCLLASANGILSQFYKGSETDEAVQYR
GPGVELPFPHLDQSDPLLLLQLQHRYTAVCLALKHTLRADPASAVYLPVRPILNLVCRALAVNPRNTSLTSDGSLRLLVL
PSVHINTLEVLAELITVVRSGMVQYAAVIQKLFSQMLCAWTAVPEANVGQQRAYSSLRVSVYRTLELWLQLVGVSANVLQ
GASSHAELLFTHLLADITPGAESIKLRAGLSADVVPGGKPGPRRTKQLVMADPVGASVQRKGDPVSNQDTCLSALKVLRR
IIQTSGTLLKNDIHKRLHEVVLPLCVRLQQQQTSSITACESAGGISGQYSSALTRRQLYRLLLALVLVPSPCWPPPLTCA
VSILSNGRLDRNLKVSQSAGHFRYHTGTKLMRERNVALVS
> Poeciliopsis prolifica [A0A0S7LET2]
GLCAGVLSRFQGLCLLSMLVKDSSSDLFQQHCLSWLRSVQQVIQSKAPCQSIQLAVSILQDLLQYSCQLPELAREVGLNS
ILGILTSLLGLKTECELAAMEGMRACMVYYPRACGSLKHKLGAYFLSKMDSTSRKTQEMACQGYSRLPCLGGLVDRSVGT
GRAEGWTNQIQCLLASANGILSQFYKGSETDEAVQYRGPGVELPFPHLDQSDPLLLLQLQHRYTAVCLALKHTLRADPAS
AVYLPVRPILNLVCRALAVNPRNTSLTSDGSLRLLVLPSVHINTLEVLAELITVVRSGMVQYAAVIQKLFSQMLCAWTAV
PEANVGQQRAYSSLRVSVYRTLELWLQLVGVSANVLQGASSHAELLFTHLLADITPGAESIKLRAGLSADVVPGGKPGPR
RTKQLVMADPVGASVQRKGDPVSNQDTCLSALKVLRRIIQTSGTLLKNDIHKRLHEVVLPLCVRLQQQQTSSITACESAG
GISGQYSSALTRRQLYRLLLALVLVPSPCWPPPLTCAVSILSNGRLDRNLKVSQSAGHFRYHTGTKLMRERNVALVS
> Poeciliopsis prolifica [A0A0S7LEK1]
GLVSVLREQHPEYLSDVLANYREHGVFLNQGASDVAGLVGFSNAKLSSSKTRFQGLCLLSMLVKDSSSDLFQQHCLSWLR
SVQQVIQSKAPCQSIQLAVSILQDLLQYSCQLPELAREVGLNSILGILTSLLGLKTECELAAMEGMRACMVYYPRACGSL
KHKLGAYFLSKMDSTSRKTQEMACQGYSRLPCLGGLVDRSVGTGRAEGWTNQIQCLLASANGILSQFYKGSETDEAVQYR
GPGVELPFPHLDQSDPLLLLQLQHRYTAVCLALKHTLRADPASAVYLPVRPILNLVCRALAVNPRNTSLTSDGSLRLLVL
PSVHINTLEVLAELITVVRSGMVQYAAVIQKLFSQMLCAWTAVPEANVGQQRAYSSLRVSVYRTLELWLQLVGVSANVLQ
GASSHAELLFTHLLADITPGAESIKLRAGLSADVVPGGKPGPRRTKQLVMADPVGASVQRKGDPVSNQDTCLSALKVLRR
IIQTSGTLLKNDIHKRLHEVVLPLCVRLQQQQTSSITACESAGGISGQYSSALTRRQLYR
> Poeciliopsis prolifica [A0A0S7LEK6]
GLVSVLREQHPEYLSDVLANYREHGVFLNQGASDVAGLVGFSNAKLSSSKTRFQGLCLLSMLVKDSSSDLFQQHCLSWLR
SVQQVIQSKAPCQSIQLAVSILQDLLQYSCQLPELAREVGLNSILGILTSLLGLKTECELAAMEGMRACMVYYPRACGSL
KHKLGAYFLSKMDSTSRKTQEMACQGYSRLPCLGGLVDRSVGTGRAEGWTNQIQCLLASANGILSQFYKGSETDEAVQYR
GPGVELPFPHLDQSDPLLLLQLQHRYTAVCLALKHTLRADPASAVYLPVRPILNLVCRALAVNPRNTSLTSDGSLRLLVL
PSVHINTLEVLAELITVVRSGMVQYAAVIQKLFSQMLCAWTAVPEANVGQQRAYSSLRVSVYRTLELWLQLVGVSANVLQ
GASSHAELLFTHLLADITPGAESIKLRAGLSADVVPGGKPGPRRTKQLVMADPVGASVQRKGDPVSNQDTCLSALKVLRR
IIQTSGTLLKNDIHKVRICQNCT
> Poeciliopsis prolifica [A0A0S7LEK5]
GLCAGVLSRFQGLCLLSMLVKDSSSDLFQQHCLSWLRSVQQVIQSKAPCQSIQLAVSILQDLLQYSCQLPELAREVGLNS
ILGILTSLLGLKTECELAAMEGMRACMVYYPRACGSLKHKLGAYFLSKMDSTSRKTQEMACQGYSRLPCLGGLVDRSVGT
GRAEGWTNQIQCLLASANGILSQFYKGSETDEAVQYRGPGVELPFPHLDQSDPLLLLQLQHRYTAVCLALKHTLRADPAS
AVYLPVRPILNLVCRALAVNPRNTSLTSDGSLRLLVLPSVHINTLEVLAELITVVRSGMVQYAAVIQKLFSQMLCAWTAV
PEANVGQQRAYSSLRVSVYRTLELWLQLVGVSANVLQGASSHAELLFTHLLADITPGAESIKLRAGLSADVVPGGKPGPR
RTKQLVMADPVGASVQRKGDPVSNQDTCLSALKVLRRIIQTSGTLLKNDIHKRLHEVVLPLCVRLQQQQTSSITACESAG
GISGQYSSALTRRQLYR
> Marmota monax [A0A5E4D029]
MAAAVLSGPSAGSPAGVPGGAGGLSAMNSGPRLRLLLLESVSGLLQPRTGSTVAPVHPPVCSVPHLPGLMCLLRLHGTVG
GAQNLSALGALVSLSNARLGSIKTRFEGLCLLSLLVGESPTEMFQQHCVSWLRSIQQVLQSQDPPPTMELAVAVLRDLLR
YAAQLPTLFRDISINHLPGLLTSLLGLRPECEQSALEGMKACMTYFPRACGSLKGKLASFFLSRVDALSPQLQQLACECY
SRLPSLGAGFSQGLKHTESWEQELHSLLASLHSLLGALYEGAETAPVQNEGPGVETLLSPSEDGDAHVLLRLRQRFSGLA
RCLGLMLSSEFGAPVSVPVQEVLDLICRTLSVSGKNISLLGDGPLRLLLLPSIHLEALDLLSALILACGGRLLRFGALIS
RLLPQVLNAWSIGRDALSPGQERPYSTIRTKVYAILELWVQVCGASAGVLQGGASGEALLTHLLSDISPPSDALKLRSPR
GSPDGGLQTGKPSAPKKIKLDVGEAMAPPSHRKGENNANSDVCAAALRGLSRTILMCGPLIKEETHRRLHDLILPLVMGV
QQGEVLGSSPYTSSCCRRELYRLLLALLLAPSPRCPPPLACALQAFSLGQREDSLEVSSFCSEALVTCAALTHPRVPPLQ
AMGPTCPTPAPVPPPEAPSPFRAPSFQPPGPMPSVGPMPSAGPIPSAGPVPSARPGPPATANHLGLSVPGLVSVPPRLLP
GPENHRAGSNEDPVLAPSGTPPPTIPPDETFGGRVPRPAFVHYDKEEASDVEISLESDSDDSVVIVPEGLPPLPPPPPSG
TTPPPVAPTGPPTASPPVPAKEEPEELPAAPGPLPPPPPPPPPPVPGPVTLPPPQLVPEGTPGGGGPPALEEDLTVININS
SDEEEEEEEEEEEEEEEEEEEEEEEFEEEEEEEEEYFEEEEEEEEEFEEEFEEEEGELEEEEEEEEEEEEEELEEVEDLE
FGSAGGEVEEGGPPPPTLPPALPPPESPKEQPEPEPEPGLLLEVEEPGSEEQHGTETAPTLVPEVLPSQGEEEREGGSPE
AGPPPQELVEEEPSAPPTLLEEGTEGGGDNVPPPPETAAEEEMETESEAAVLQEKEQDDTAAMLADFIDCPPDDEKPPPT
TEPDS
> Pipistrellus kuhlii[A0A7J7TXW4]

MDLAVVILRDLLRYAAQLPTLFRDISMNHLPGLLTSLLGLRPECELSALEGMKACMTYFPRACGSLKGKLASFFLSRVDA

LSPQLQQLACECYSRLPSLGAGFSQGLKHTESWEQELHSLLASLHSLLGALYEGAEPAPVQYDGPGVEMLFSPAEDADAH
NLFRLRQRFSGLASCLGLLLSSEFGAPVSIPVQEILDFICRTLSISAKNISLLGDGPLRLLLLPSIHLEALDLLSALILA
CGGRLLRFGALISRLLPQVLNAWSIGRDTVSLGQEKPYSAMRTKVYAVLELWVQVCGASAGVLQASASGEALLTHLLSDI
SPPADALKLRSPRGSPDGGLQTGKPSAPKKLKLDMGEAAAPPSHRKGDGNANSDVCAAALRGLSRTILMCGPLIKEETHR
RLHDLVLPLVMGVQQGSVLASSPYTSARCRRELYRLLLALLLAPSPRCPPPLACALQAFSLGQREDNLEVSSFCSEALVT
CAALTHPRVPPLQSVGPTCPTPAPVPPPEAPSPFRAPPFHPPGPLPSAGPMPSAGSVPSVGPLPSAGPMPSAGPVPPSGP
VPPARPGPPATANHLGLSVPGLVSVPPRLLPGPENHRAGSNEEPILASSGTPPPVIPPEETFGGRVPRPAFVHYDKEEAS
DVEISLESDSDDSVVIVPEGLPPPPPPPPSDATPPPVAPAGPPAASPPLPAKEEPEELPTVLGPLPPPPPPPVPGPVTLQ
PPQLVPEGTPSGGGPSALEEDLTVININSSDEEEEEEEEEEEEEEEEEEEEEEEDFEEEEEDEYFEEEEEEEEFEEEFEEEEG
ELEEEEEEEEEEEEEEELEEEELEKEGGSPTAGPPPQELVEEEPCAPPPAMLDEGTEGGGDEVRPPPETSAVEEMETETPP
PQEKEQDDTAAMLADFIDCPPDDEKPPPVTEPDS
> Sarcophilus harrisii (Tasmanian devil) (Sarcophilus laniarius) [G3VCE0]
MAAAVLSGSPTGSGAGGPGGAGGFPAAGPGSRLRMLLLESVSGFLQPRAGPAAPPSPPTAPLVPGLVRLLRLHGMMGGAQ
NLSAVGALVGLSNSRLGSIKTRFEGLCLLSLLVAESPTETFQQHSVSWLRSIQHVLQSQDPAPTMELAVAILRDLLRYSA
QLPELSRDISTNHLPGLLTSLLGLKPECELSALEGMKACMTYFPRACGSLRGKLASFFLSRVEALSPQLQQLACECYSRL
PALGAGFSQGLKHTESWEQELHCLLASLHGLLGALYEGAETAPIQYEGPGVELLLPPPADNDVHGLLQLRQRFSGLARCL
GLMLSSEFGAPVSVPVQDILDIICRTLSVSGKNISWLGDGPLRLLLLPSIHLEALELLSALILSCGSRLVRFGGLICRLL
PQVLNTWSAGRDPLPPGQERPYSAVRAKVYSVLELWVQTCGAGAGILQGGAQSEALLTHLLSDIFPPADALKLRGPRVTQ
DGGLQSGKPSAPKKLKLDSGEAVVPPMHRKGDSNANSDVCTTALRGLSRTILMCGPLIKEETHRRLHELTLPMVMGTQQG
EIPGGSPYTSARCRRELYRLLLALLLAPAPQCPPPLTCALQAFSLGQREENLEVSSFCSEALVTCAALAHPRVPPLQSPG
PSSPPTVPPTQPEPPSPFRAPPFHPPGPRPPTSATANHLGLPVPGLVSVPPRLLAGPENHRAAFNEDSTPAPAGTSPPSQ
PPEEAFGGRPPRPAFVHYEKEETSDVEISLESDSDDSVVIVPEGLLPLPPAPPQGGTPPPALPVVATAASPPLPPKEEPE
ELPPAPGPLPPPPGPGPLPPPQLVAEGTAGGGPPPLEEDLTVININSSDEEEEEEEEEEEEEEEEEDFPEEEEEDEEEEYF
EEEEEEEEEFEEEFEEEEEGELEEEEEEEEEEEEEEEELEEVEFEPAEGEVEDRGAPLPDSSPLPPPSEPPKEEPEPGLLM
EVEEPSAEDEKEGGGAPALALEASPSQGEREQEGRTDVPEAPPAEELAGNEPPVLSEEGTDGRGNQEPPLAKGAETTVTE
SETEPLGEKEQDDTAAMLADFIDCPPDEEKPTPLPEPDS
> Vombatus ursinus (Common wombat) [A0A4X2JSX2]
MAAAVLSGSPTGSGAGGPGGAGGLPAAGPGSRLRMLLLESVSGLLQPRAGPAAPPSPPTAPLVPGLVRLLRLHGMMGGAQ
NLSAVGALVGLSNARLGSIKTRFEGLCLLSLLVAESPTETFQQHSVSWLRSIQHVLQSQDPAPTMELAVAILRDLLRYSA
QLPELSRDISTNHLPGLLTSLLGLKPECELSALEGMKACMTYFPRACGSLRGKLASFFLSRVEALSPQLQQLACECYARL
PALGAGFSQGLKHTESWEQELHCLLASLHGLLGALYEGAETAPIQYEGPGVELLLPPPADNDVHGLLQLRQRFSGLARCL
GLMLSSEFGAPVSVPVQDILDIICRTLSVSGKNISWLGDGPLRLLLLPSIHLEALDLLSALILSCGSRLVRFGGLICRLL
PQVLNTWSAGRDPLPPGQERPYSAVRAKVYSVLELWVQTCGAGAGVLQGGAQSEALLTHLLSDIFPPADALKLRGPRVTQ
DGGLQAGKPSAPKKLKLDSGEAVVPPMHRKGDSNANSDVCTTALRGLSRTILMCGPLIKEETHRRLHELTLPMVMGTQQG
EIPGGSPYTSARCRRELYRLLLALLLAPAPQCPPPLTCALQAFSLGQREENLEVSSFCSEALVTCAALAHPRVPPLQSPG
LSSPPTAPPTQPEPPSPFRAPPFHPPGPRPPVSATANHLGLPVPGLVSVPPRLLPGPENHQAALSEDSAPAPAGTSPPSQ
PPDEAFGGRPPRPAFVHYEKEETSDVEISLESDSDDSVVIVPEGLLPLPPAPPQGGTPPPAPPVVATAASPPQPPKEEPE
ELPPTPGPLPPPPGPGALPAPQLVAEGTTGGGPPPLEEDLTVININSSDEEEEEEEEEEEEEEEEEEDFPEEEEEEEEDEEE
EYFEEEEEEEEEFEEEFEEEEEGELEEEEEEEEEEEEEEELEEVEFEPAEGEVEDRGAPPPDSPPPPPPSEPPKEEPEPGL
LMEVEEPSAEDEKEGGGAPPLALEASPPQGEREEEGRTVVPEAPPAEELAGNEPPVFSGEGTDGRGSQEPPPTRSAEAPA
TETEMAPIGEKEQDDTAAMLADFIDCPPDEEKPTPPPEPDS
>Phyllostomus discolor [A0A6J2NIV8]
MAAAVLSGPSAGSAAGASGGTGGLSALGSGPRLRMLLLESVSGLLQPRTGSTVPPVHPPVRSAPHLPGLMRLLRLHGTVG
GAQNLSAVAALVGLSNARLGSIKTRFEGLCLLSLLVGESSTEMFQQHCVSWLRSIQQVLQSQDPPPTMELAVAVLRDLLR
YAAQLPTLFRDISMNHLPGLLTSLLGLRPECELSALEGMKACMTYFPRACGSLKGKLASFFLSRVDALSPQLQQLACECY
SRLPSLGAGFSQGLKHTESWEQELHSLLASLHSLLGALYEGAEPAPMQYEGPGVEMLFSPSEDGDAHNLLRLRQRFSGLA
CCLGLLLRSEFGAPVSIPVQEILDVICRTLSISAKNISLLGDGPLRLLLLPSIHLEALDLLSALILACGGRLLRFGSLIS
RLLPQVLNAWSIGRDTLSLGQEKPYSAMRTKVYAVLELWVQVCGASAGVLQGGASGEALLTHLLSDISPPADALKLRSPR
GSPDGGLQTGKPSAPKKLKLDMGEAVAPPSHRKGDSNANSDVCAAALRGLSRTILMCGPLIKEETHRRLHDLVLPLVMSV
QQGEVLGSSPYTSSYCRRELYRLLLALLLAPSPRYPPPLACALQAFSLGQREDNLEVSSFCSEALVTCAALTHPRVPPLQ
SMGPACPAPAPVPPPEAPSPFRAPPFHPPGPMPSAGTMPSAGPVPSVGPMPPVGPMPPARPGPPATANHLGLSVPGLASV
PPRLLPGPENHRAGSNEDTILAPSGTPPPTIPPDETFGGRVPRPAFVHYDKEEASDVEISLESDSDDSVVIVPEGLPPLP
LPPPAGTTPPPVAPAGPPTASPPLPAKEEPEELPAAPGPLPPPPPPLPGPVTLQPPQLVPEGTPGGGAPPALEEDLTVIN
INSSDEEEEEEEEEEEEEEEEEEEEDFEEEEEDEEEYFEEEEEEEEEFEEEFEEEEGELEEEEEEEEDEEEEEEELEEVEELEF
GPTGGEVEGGGPPPTSLPPALPPTESPKVQPEPEPEPEPEPEPGLLLEVEEPGAIGEPGAETAPTLAPEVLPSQEEMEREGG
SPPAGPPPQELVEEEPCAPPTLLEEGTEVGGDEVPPPPETVAAEEMEPEPEPETPALQEKEQDDTAAMLADFIDCPPDDE
KPPPVTEPDS
> Sus scrofa [A0A5G2R420]
MLLSSPNAVQPPCTQRARPRPPPHAPSPRGVRRGVFASSRHATTTVRVKMAAAVLSGPSAGSAAGVPGGTGGLSAVVSVP
RLRLLLLDSVSGLLQPRAGSTVAPVHPPAPSAPHLPGLMCLLRLHGTVGGAPNLSAVGALVGLSNARLGSIKTRFEGLCL
LSLLVGESPTEMFQQHCVSWLRSIQQVLQSQDPPPTMELAVAVLKDLLRYAAQLPTLFRDISMNHLPGLLTSLLGLRPEC
ELSALEGMKACMTYFPRACGSLKGKLASFFLSRVDALSPQLQQLACECYSRLPSLGAGFSQGLKHTESWEQELHSLLASL
HGLLGALYEGAETAPMQYEGPAVEALLSPSEDGDAHVLLRLRQRFSGLARCLGLLLSSEFGAPVSVPVQEVLDVICRTLS
VSARNISLLGDGPLRLLLLPSLHLEALDLLSALILACGGRLLRFGALISRLLPQVLSAWSIGRDTLSPGQERPYSTMRTK
VYAVLELWVQVCGASAGVLQGGASGEALLTHLLSDISPPADALKLRSPRGSPDGGLQTGKPSAPKKLKLDMGEPIAPPSH
RKGDSNANSDVCAAALRGLSRTILMCGPLIKEETHRRLHELVLPLVMGVQQGEVLGSSPYTSSRCRRELYRLLLALLLAP
SPRCPPPLACALQAFSLGQREDSLEVSSFCSEALVTCAALTHPRVPTLQSMVPTCPTPAAVPPPEAPSPFRAPAFHPPGP
MPSAGPMPSGPMPSAGPMPSVGPMPPARPGPPATANHLGLSGSSLVSVPPRLLPGPENHRAGSNEEPVLAPSGTPPPTLP
SDETFGGRVPRPAFVHYDKEEASDVEISLESDSDDSVVIVPEGLPPLPPPPPSGTTPPPVAPAGPPAASPPVPAKEEPEE
LPAAPGPLPPPPPTPVPGPATLPPPQLVPEGTPGGGGAPALEEDLTVININSSDEEEEEEEEEEEEEEEEEEEEEEEDFEEE
EEDEEEYFEEEEEEEEEEFEEEFEEEEGELEEEEEDEEEEEEEEELEEVEELEFGSAGAEVEEGGPPPPSLPPALPPAESPK
VQPEPEPEPEPGLLLEVEEPGAEEAPGPETAPTLVPEALPAQGEAEREAGSPPTAPPPQELVEEEPSVPPTLLEEGAEGGGD
KVPPPPETSAAEEMETETESTALQEKEQDDTAAMLADFIDCPPDDEKPPPAAEPDS
>Canada lynx [A0A667HQV9]
MLPRSPRRAQPPPAQCAGTRPPPHAPSPPGARRGAFASSRHATGARVKMAAAVLSGPSAGSAAGVPGGTGGLSAVGSGPR
LRLLLLESVSGLLQPRAGSAVAPVHPPVRSAAHLPGLMCLLRLHGTVGGAQNLSAVGALVGLSNARLGSVKTRFEGLCLL
SLLVGESPTELFQQHCVSWLRSIQQVLQSQDPPPTMELAVAVLRDLLRYAAQLPTLFRDISTNHLPGLLTSLLGLRPECE

LSAMEGMKACMTYFPRACGSLKGKLASFFLSRVDALSPQLQQLACECYARLPSLGAGFSQGLKHTESWEQELHSLLASLH
GLLGALYEGADTAPVQCEGPGLDVLLAPSEDGDAHTLLRLRHRFSGLARCLGLLLSSEFGAPVSVPVQEILDIICRTLSI
SAKNISLLGDGPLRLLLLPSIHLDALDLLSALILACGSRLLRFGALISRLLPQVLNAWNLGRDALPPGQERPYSAVRTKV
YAVLDLWVQVCGASAGVLQGGASGEALLSHLLSDISPPADALKLRSPRGSPDGGLQSGKPSAPKKLKLDMGEATAPPGHR
KGDSNANSDVCAAALRGLSRTVLMCGPLIKEETHRRLHDLVLPLVMGVQQGEVLGSSPYTSSRCRRELYRLLLALLLAPS
PRCPPPLACALQAFSLGQREDSLEVSSFCSEALVTCAALTHPRVPPLQSMGPACPAPAPAPPPEAPSPFRAPPFHPPGPM
PSVGPMPSVGPMPSVGPMPPAGPMPPTRPGPPATANHLGLSVPGLVSVPPRLLPGPENHRAGSNDDPVLAPSGTPPPAVP
PDETFGGRVPRPAFVHYDKEEASDVEISLESDSDDSVVIVPEGLPPLPPPPPTGTTPPPAAPAGPPTASPPMPAKEEPEE
LPAAPGPLPPPPPPPVPGPVALPPPQLVPEGPPGGGGPPALEEDLTVININSSDEEEEEEEEEEEEEEEEEEEEEDFEEE
EEEEEEYFEEEEEEEEEFEEEFEEEEGELEEEEEDEDEEEEDELEELEEVEFGPAGGPAEEGGPPPPSPAPALPPAQPPE
APPEPGVEPGLLLEVEEPGPEDEPGAEAAPTLAPEVLPSQGEGQREAGSPPAGPPPRELVEEEPSAPPPLLEEGTENGGD
KVPPPPETPAAEEMEAAAEAEEQDDTAAMLADFIDCPPDDEKPPAAPEPES
>Felis catus [A0A337SEG8]
MPRAACIRVCRARTAVPDSRREAGSQDAGRAESGGAASPGHSPRRPGTLAPFVPTTWSAPRARRPAVRACSLAARAASPT
SARPARGDAAPSSRPLPTRRPSRCVCVIAPRHHGARVKMAAAVLSGPSAGSAAGVPGGTGGLSAVGSGPRLRLLLLESVS
GLLQPRAGSAVAPVHPPVRSAAHLPGLMCLLRLHGTVGGAQNLSAVGALVGLSNARLGSVKTRFEGLCLLSLLVGESPTE
LFQQHCVSWLRSIQQVLQSQDPPPTMELAVAVLRDLLRYAAQLPTLFRDISTNHLPGLLTSLLGLRPECELSAMEGMKAC
MTYFPRACGSLKGKLASFFLSRVDALSPQLQQLACECYARLPSLGAGFSQGLKHTESWEQELHSLLASLHGLLGALYEGA
DTAPVQCEGPGLDVLLAPSEDGDAHTLLRLRHRFSGLARCLGLLLSSEFGAPVSVPVQEILDIICRTLSISAKNISLLGD
GPLRLLLLPSIHLDALDLLSALILACGSRLLRFGALISRLLPQVLNAWNLGRDALPPGQERPYSAVRTKVYAVLDLWVQV
CGASAGVLQGGASGEALLSHLLSDISPPADALKLRSPRGSPDGGLQSGKPSAPKKLKLDMGEATAPPGHRKGDSNANSDV
CAAALRGLSRTVLMCGPLIKEETHRRLHDLVLPLVMGVQQGEVLGSSPYTSSRCRRELYRLLLALLLAPSPRCPPPLACA
LQAFSLGQREDSLEVSSFCSEALVTCAALTHPRVPPLQSMGPACPAPAPAPPPEAPSPFRAPPFHPPGPMPSVGPMPSVG
PMPSVGPMPPAGPMPPTRPGPPATANHLGLSVPGLVSVPPRLLPGPENHRAGSNDDPVLAPSGTPPPAVPPDETFGGRVP
RPAFVHYDKEEASDVEISLESDSDDSVVIVPEGLPPLPPPPPTGSTPPPAAPAGPPTASPPVPAKEEPEELPAAPGPLPP
PPPPPVPGPVALPPPQLVPEGPPGGGGTPALEEDLTVININSSDEEEEEEEEEEEEEEEEEEEEEDFEEEEEEEEEYFEE
EEEEEEEFEEEFEEEEGELEEEEEDEDEEEEDELEELEEVEFGPAGGPAEEGGPPPPSPAPALPPAQPPEVPPEPGVEPG
LLLEVEEPGPEDEPGAEAAPTLAPEVLPSQGEGQREAGSPPAGPPPQELVEEEPSAPPPLLEEGTENGGDKVPPPPETPA
AEEMEAAAEAETAALQEKVRRAGGGCGCPGDLGRPALPRVAASNGVSLPRKEQDDTAAMLADFIDCPPDDEKPPAAPEPE
S
> Rousettus aegyptiacus [A0A7J8GDR5]
MAAAVLSGPSAGSAAGVPGGTGGLSAVGSVPRLRLLLLESVSGLLQPRAGSAIAPVHPPVRSAPHLPRLMCLLRLHGTVG
GAQNLSAVGALVGLSNARLGSIKTRFEGLCLLSLLVGESSTEMFQQHCVSWLRSIQQVLQSQDPPPTMELAVAVLRDLLR
YAAQLPTLFRDISMNHLPGLLTSLLGLRPECELSALEGMKACMTYFPRACGSLKGKLASFFLSRVDALSPQLQQLACECY
SQVPSLGAGFSQGLKHTESWEQELHSLLASLHSLLGALYEGAEPAPMQYEGPGVEMLLSPSEDGDAHILLRLWQRFSGLA
RCLGLMLSSEFGAPVSVPVQEILDVICRTLSISAKNISLLGDGPLRLLLLPSIHLEALDLLSALILACRGRLLRFGALIS
RLLPQVLNAWSIGRDSLSPGQEKPYSAMRTKVYAILELWVQVCGASAGVLQGGASGEALLTHLLSDISPSADTLKLRSPR
GSPDGGLQMGKPSAPKKLKLDVGEAMAPPSHRKGDSNANSDVCAAALRGLSQTILMCGPLIKEETHRRLHDLVLPLVMGV
QQGEILGSSPYTSSCCRRELYHLLLALLLAPSPRCPPPLACALQAFSLGQREDSLEVSSFCSEALVTCAALTHPRVPPLQ
SMGPTCPTPAPVPPPEAPSPFRAPPFHPPGPVPSAGPMPSAGPVPSVGSMPSVGSMPSAGPMPSAGPMPPTRPGPPATAN
HLGLSVPGLVSLPPRLLPGPENHRSGSNEDPILAPSGTPPPTIPPDETFGGRVPRPAFVHYDKEEASDVEISLESDSDDS
VVIVPEGLPPLPPPPASGTTPPPVTPAGPPTASPPLPTQEEPEELPVALGPLPPPPPPSVPAPVTLAPPQLVPEGTPGGG
GPPALEEDLTVININSSDEEEEEEEEEDEEEEEEEDFEEEEDEEEYFEEEEEEEEEEFEEEYEEEEGELEEDEEEDEEEE
EELEEVEELEFGSAGGEIEEGGPPPPNLPPALPPVESPKLQPEPEPEPEPEPEPGLLLEVEEPGAEEEHGAETAPTLAPE
VLSSQGELEREAGSPPAGPPPQELVEEEPCAPPTLLEEGTEVGGDKVPPPPETSAAEEMQTEKEATALQEKEQDDTAAML
ADFIDCPPDDEKPPPAAEPDS
> Bos taurus [E1BDV5]
MSGWKVSRSSVALKVSFWILAPLVLTTCSLSPTARPEPVLYPAHLPGLESWHFHAAHAHSQQARGVQCTGPGPPPHDPSP
RGIRRGVFASSRHATTRAQLKMAAAVLSGPSAGSAAAVPGGPGGLSAVGSGPRLRLMLLESVSGLLQPRTGSAVAPVHPP
ARSAPHLPGLMCLLRLHGTVGGAQNLSAVGALVGLSNARLGSIKTRFEGLCLLSLLVGESPTEMFQQHCVSWLRSIQQIL
QSQDPPPTMELAVTVLKDLLRYAAQLPAVFRDISMNHLPGLLTSLLGLRPECELSALEGMKACMTHFPRACGSLKGKLAS
FFLSRVDALSPQLQQLACECYSRLPSLGAGFSQGLKHTDSWEQELRSLLASLHSLLGGLYEGAEAAPMQYESPGAETLLS
SSEDADAHTLLRLRQRFSGLARCLGLMLSSEFGAPVSVPVQDILDLICRTLSVSAKNVSLLGDGPLRLLLLPSLHLEALD
LLSALILACGARLLRFGALISRLLPQVLNAWSIGRENLGPGQERPYSTVRTKVYAVLELWVQVCGASAGVLQGGASGEAL
LSHLLSDISPPADALRLRSPRGSPDAGLQTGKPSAPKKLKLDVGEAIAPPSHRKGDSNANSDVCAAALRGLSRTILMCGP
LIKEETHRRLHELVLPLVMGVQQGEALGSSPYTSSHCRRELYHLLLALLLAPSPRCPPPLACALRAFSLGQREDSLEVSS
FCSEALVTCAALTHPRVPPLQSVGPTCPAPAPVPPPEAPAPFRAPAFHAPSPLPSAGPMPSAGPMPPVGPLPPTRPGPPA
TANHLGLSVPGLVSVPPRLLPGPENHRAGSSEDPVLAPSGSPPPTIPPDETFGGRVPRPAFVHYDKEEPSDVEISLESDS
DDSVVIVPEGLPPPPPPSSGTTPPPVAPAGPPAASPPVPAKDEPEELPAAPGPLPPPPPPPVPGPVTLPPPQLVPEGTPG
GGGPPALEEDMTVININSSDEEEEEEEEEEEDEEEDEEEDFEEDEEEEEEYFEEEEEEEEEEFEEEFEEEEGELEDEEDE
EEDEELEELEEVEFGPAGGEVEGGGPAPPSLPPALPPAESPKGPPEPGLEPGLLLEVEEPGTEEAPGPETAPMLAPEVLP
SQGEVEREGGSPPAGPPPQELVEEEPSGPPALLEEGAEGGGDKVSPPPEASAVEETEVEAAALPPEKEQGDTAAMLADFI
DCPPDDEKPPPASEPDS
>Hybrid - Bos Indicus [A0A4W2H5C3]
MSGWKVSRSSVALKVSFWILAPLVLTTCSLSPTARPEPVLYPAHLPGLESWLFHAAHAHSQQARGIQCTGPGPPPHDPSP
RGIRRGVFASSRHATTRAQLKMAAAVLSGPSAGSAAAVPGGPGGLSAVGSGPRLRLMLLESVSGLLQPRTGSAVAPVHPP
ARSAPHLPGLMCLLRLHGTVGGAQNLSAVGALVGLSNARLGSIKTRFEGLCLLSLLVGESPTEMFQQHCVSWLRSIQQIL
QSQDPPPTMELAVTVLKDLLRYAAQLPAVFRDISMNHLPGLLTSLLGLRPECELSALEGMKACMTHFPRACGSLKGKLAS
FFLSRVDALSPQLQQLACECYSRLPSLGAGFSQGLKHTDSWEQELRSLLASLHSLLGGLYEGAEAAPMQYESPGAETLLS
SSEDADAHTLLRLRQRFSGLARCLGLMLSSEFGAPVSVPVQDILDLICRTLSVSAKNVSLLGDGPLRLLLLPSLHLEALD
LLSALILACGARLLRFGALISRLLPQVLNAWSIGRENLGPGQERPYSTVRTKVYAVLELWVQVCGASAGVLQGGASGEAL
LSHLLSDISPPADALRLRSPRGSPDAGLQTGKPSAPKKLKLDVGEAIAPPSHRKGDSNANSDVCAAALRGLSRTILMCGP
LIKEETHRRLHELVLPLVMGVQQGEALGSSPYTSSHCRRELYHLLLALLLAPSPRCPPPLACALRAFSLGQREDSLEVSS
FCSEALVTCAALTHPRVPPLQSVGPTCPAPAPVPPPEAPAPFRAPAFHAPSPLPSAGPMPSAGPMPPVGPLPPTRPGPPA
TANHLGLSVPGLVSVPPRLLPGPENHRAGSSEDPVLAPSGSPPPTIPPDETFGGRVPRPAFVHYDKEEPSDVEISLESDS
DDSVVIVPEGLPPPPPPSSGTTPPPVAPAGPPAASPPVPAKDEPEELPAAPGPLPPPPPPPVPGPVTLPPPQLVPEGTPG
GGGPPALEEDMTVININSSDEEEEEEEEEEEDEEEDEEEDFEEDEEEEEEYFEEEEEEEEEEFEEEFEEEEGELEDEEDE

EEDEELEELEEVEFGPAGGEVEGGGPAPPSLPPALPPAESPKGPPEPGLEPGLLLEVEEPGTEEAPGPETAPMLAPEVLP
SQGEVEREGGSPPAGPPPQELVEEEPSGPPALLEEGAEGGGDKVSPPPEASAVEETEVEAAALPPEKEQGDTAAMLADFI
DCPPDDEKPPPASEPDS
>Capra hircus [A0A452G925]
MAAAVLSGPSAGSAAAVPGGAGGLSAVGSGPRLRLMLLESVSGLLQPRTGSAVAPVHPPARSAPHLPGLMCLLRLHGTVG
GAQNLSAVGALVGLSNARLSSIKTRFEGLCLLSLLVGESPTEMFQQHCVSWLRSIQQILQSQDPPPTMELAVTVLKDLLR
YAAQLPAVFRDISMNHLPGLLTSLLALRPECELSALEGMKACMTHFPRACGSLKGKLASFFLSRVDALSPQLQQLACECY
SRLPSLGAGFSQGLKHTDSWEQELRSLLASLHSLLGGLYEGAEAAPMQYESPGAETLLSPSEDADAHTLLRLRQRFSGLA
RCLGLMLSSEFGAPVSVPVQDILDLICRTLSVSAKNVSLLGDGPLRLLLLPSLHLEALDLLSALILACGARLLRFGALIS
RLLPQVLSAWSIGRENLGPGQERPYSTVRTKVYAVLELWVQVCGASAGVLQGGASGEALLSHLLSDISPPADALRLRSPR
GSPDAGLQTGKPSAPKKLKLDVGEAIAPPSHRKGDSNANSDVCAAALRGLSRTILMCGPLIKEETHRRLHELVLPLVMGV
QQGEALCSSPYTSSHCRRELYRLLLALLLAPSPRCPPPLACALRAFSMGQREDSLEVSSFCSEALVTCAALTHPRVPPLQ
SVGPTCPAPAPVPPPEAPAPFRAPAFHAPGPLPSAGPMPSAGPMPPAGPLPTTRPGPPATANHLGLSVPGLVSVPPRLLP
GPENHRAGSSEDPVLAPSGSPPPTVPPDETFGGRVPRPAFVHYDKEEASDVEISLESDSDDSVVIVPEGLPPPPPPPSGT
TPPPVAPAGPPAASPPVPAKDEPEELPAAPGPLPPPPPPPVPGPVTLPPPQLVPEGTPGGGGPPALEEDLTVININSSDE
EEEEEEEEEEDEEEDEEEDFEEDEEEEEYFEEEEEEEEEFEEEFEEEEGELEDEEDEEEDEELEELEEVEFGPAGGEVE
GGGPAPPSLPPVLPPTESPKGPPEPGLEPGLLLEVEEPGTEEAPGPEMAPMLAPEVLPSQGEVEREGGSPPAGPPPQELV
EEEPSGPPTLLEEGAEGGGDKLSPPPEASAVEEMEVEAAALPPEKEQGDTAAMLADFIDCPPDDEKPPPVSEPDS
>Rhinolophus ferrumequinum [A0A671FCW1]
MAAAVLSGPSAGSAAGVPGGTGGLSAVGSVPRLRLLLLESVSGLLQPRAGSAIAPVHHHVRSASHLPGLMCLLRLHGTVG
GAQNLSAVGALVGLSNARLGSIKTRFDGLCLLSLLVGESSTEMFQQHCVSWLRSIQQVLQSQDPPPTMELAVAVLRDLLR
YAAQLPTLFRDISMNHLPGLLTSLLGLRPECELSALEGMKACMTYFPRACGSLKGKLASFFLSRVDALSPQLQQLACECY
SRVPSLGAGFSQGLKHTESWEQELHSLLASLHSLLGALYEGAEPAPMQYEGPGVEMLLSPSEDGDAHVLLRLWQRFSGLA
RCLGLMLRSEFGAPVSVPVQGILDIVCRTLSISAKNISLLGDGPLRLLLLPSIHLEALDLLSALILACRGRLLRFGALIS
RLLPQVLNAWSIGRDTLSPGQEKPYSAMRTKVYAILELWVQVCGTSAGVLQGGASGEALLTHLLSDISPSADALKLRSPR
GSPDGSLQIGKPSAPKKLKLDVGEAVAPPSHRKGDSNANSDVCAAALRGLSQTILMCGPLIKEETHRRLHDLVLPLVMSV
QQGEVLGSSPYTSSRCRRELYRLLLALLLAPSPRCPPPLTCALQAFSLGQREDSIEVSSFCSEALVTCAALIHPRVPPLQ
SMGPTCPTPAPVPPPEAPSPFRAPPFHPSGPMSSAGTMPSASPVPSVGPMPSVGPMPSAGPMPSAGSMPSAGPMPPARPG
PPATTNHLGLSVPGLVSVPPRLLPGPENHQAGSNEDPILAPSGTPPPTIPPDETFGGRVPRPAFVHYDKEEASDVEISLE
SDSDDSVVIVPEGLPPLPPPPPSGTSPPPVAPAGPPIASPPLPAKEEPEELPATPGPLPPPPPPPPVPGPVTLPPPQLVPE
GTPGGGGPPALEEDMTVININSSDEEEEEEEEEEEEEEEEEEDFEEEEDEEEYFEEEEEEEEEFEEEFEEEEGELEEEEE
EEDEEEEEEELEEVEELEFGSAVGEVEEGGPAPPSLPPALPPAESPKVQPELEPEPEPEPELEPEPEPEPEPGLLLEVEEP
GVEEEEHGAETAPTLAPEVLPSQGELEKEAGSPPAGPPPQELMEEDPCAPPTLLEEETEVGGDKLLPPPEASAAEEMETE
GDATALQEKEQDDTAAMLADFVDCPPDDEKPPPATEPDS
> Oryctolagus cuniculus [G1T8B3]
MPGTSGERVSSSDAGYRLPRQCPAPKFNGNISGTGAFRPGHVPLLLRGTAPRHPTPPKPDTRRPASPPLRPAHALSQLAP
GIRLLAPVRRTTAPSSRPLPTRRASRCVCVIAPRHHSSPGKMAAAVLSGPSAGSPAGVPGGAGGLSAMGSGPRLRLLLLE
SVSGLLQPRTGSTVAPVHLPVRSAPHLPGLVCLLRLHGTVGGAQNLSAVGALVSLSNARLGSIKTRFEGLCLLSLLVGES
STEMFQQHCVSWLRSIQQVLQSQDPPPTMELAVAVLRDLLRYAAQLPVLFRDISINHLPGLLTSLLGLRPECEQSALEGM
KACMTYFPRACGSLKGKLASFFLSRVDALSPQLQQLACECYSRLPSLGAGFSQGLKHTESWEQELHSLLASLHTLLGALY
EGAETVSVPSEDPGVEALLSQSEDGDAHVLLRLRQRFSGLARCLGLMLSSEFGAPVSVPVQEVLDVICRTLSVSSKNISL
LGDGPLRLLLLPSVHLEALDLLSALILACGSRLLRFGALISRLLPQVLNAWSVGRDSLSPGQERPYSTIRTKVYAVLELW
VQVCGASAGVLQGGASGEALLTHLLSDIAPPTDALKLRSPRGSPDGSSQTGKPSAPKKLKLDVGEAVAPPSHRKGDSNAN
SDVCAAALRGLSRTILMCGPLIKEETHRRLHDLVLPLVMSVQQSEVLGSSPYTSSRCRRELYRLLLALLLAPSPRCPPPL
ACALQAFSLGQREDSLEVSSFCSEALVTCAALTHPRVPPLQSVGPTCLTPAPVPPPEAPSPFRAPPFHPPGPMPSAGPMP
SAGPVPSAGPVPSAGPLTSAGPVPSAGPVPSARPGPPATANHLGLPVPGLVSVPPRLLPGPENHRGGSHEDPVLAPSGTP
PPAVPPDETFGGRVPRPAFVHYDKEEASDVEISLESDSDDSVVIVPEGLLPLPPPPPSGTTSPPAAPAGPPTASPPVPAK
EEPEELPAAPGPLPPPPPPPVPGPVALPPPQLVPEGTPGGGGGGGGSGGSGGPPALEEDLTVININSSDEEEEEEEEEE
EEEEEEEEEEDFEEEEEDEEEYFEEEEEEEEEFEEEFEEEEGELEEEDEEEEEEEEEEELEEVEELEFGPAGGEVEEGGPP
PPALPPALPPPESPKVPPEPEPEPEPEPEPGLLLQVEEPGTEEEREVEAAPTLAPEGLPSQGEAEREASSPGVEPPPQALVE
EEPSAPPALLEEETEGEGDKVPQPPETSAEEEVEAGAAELPEKEQDDTAAMLADFIDCPPDDEKPPPATEPDS
>Pipistrellus kuhlii [A0A7J7TXV9]
MAAAVLSGPSAGSAAGVPGGAGGLSAVGSGPRLRMLLLESVSGLLQPRAGSTIPPVHPPARSAPHLPGLMCLLRLHGTVG
GAQNLSAVGALVGLSNARLSSIKTRFEGLCLLSLLVGESSTEIFQQHCVSWLRSIQQVLQSQDPPATMDLAVVILRDLLR
YAAQLPTLFRDISMNHLPGLLTSLLGLRPECELSALEGMKACMTYFPRACGSLKGKLASFFLSRVDALSPQLQQLACECY
SRLPSLGAGFSQGLKHTESWEQELHSLLASLHSLLGALYEGAEPAPVQYDGPGVEMLFSPAEDADAHNLFRLRQRFSGLA
SCLGLLLSSEFGAPVSIPVQEILDFICRTLSISAKNISLLGDGPLRLLLLPSIHLEALDLLSALILACGGRLLRFGALIS
RLLPQVLNAWSIGRDTVSLGQEKPYSAMRTKVYAVLELWVQVCGASAGVLQASASGEALLTHLLSDISPPADALKLRSPR
GSPDGGLQTGKPSAPKKLKLDMGEAAAPPSHRKGDGNANSDVCAAALRGLSRTILMCGPLIKEETHRRLHDLVLPLVMGV
QQGSVLASSPYTSARCRRELYRLLLALLLAPSPRCPPPLACALQAFSLGQREDNLEVSSFCSEALVTCAALTHPRVPPLQ
SVGPTCPTPAPVPPPEAPSPFRAPPFHPPGPLPSAGPMPSAGSVPSVGPLPSAGPMPSAGPVPPSGPVPPARPGPPATAN
HLGLSVPGLVSVPPRLLPGPENHRAGSNEEPILASSGTPPPVIPPEETFGGRVPRPAFVHYDKEEASDVEISLESDSDDS
VVIVPEGLPPPPPPPPPSDATPPPVAPAGPPAASPPLPAKEEPEELPTVLGPLPPPPPPPPVPGPVTLQPPQLVPEGTPSGG
GPSALEEDLTVININSSDEEEEEEEEEEEEEEEEEEEEEEEDFEEEEEDEYFEEEEEEEEFEEEFEEEEGELEEEEEEEEEEE
EEELEEVEELEFGSAGGVVEEGGPPPTSVPPDLPSAESPKVQPEPEPEPEPGLLLEVEEQGPEEERGAETAPTLAPEVLPSQ
EELEKEGGSPTAGPPPQELVEEEPCAPPPAMLDEGTEGGGDEVRPPPETSAVEEMETETPPPQEKEQDDTAAMLADFIDC
PPDDEKPPPVTEPDS
>Sus scrofa [F1RFT3]
MAAAVLSGPSAGSAAGVPGGTGGLSAVVSVPRLRLLLLDSVSGLLQPRAGSTVAPVHPPAPSAPHLPGLMCLLRLHGTVG
GAPLSLQSSHPPLIITHLFLVRFEGLCLLSLLVGESPTEMFQQHCVSWLRSIQQVLQSQDPPPTMELAVAVLKDLLRYAA
QLPTLFRDISMNHLPGLLTSLLGLRPECELSALEGMKACMTYFPRACGSLKGKLASFFLSRVDALSPQLQQLACECYSRL
PSLGAGFSQGLKHTESWEQELHSLLASLHGLLGALYEGAETAPMQYEGPAVEALLSPSEDGDAHVLLRLRQRFSGLARCL
GLLLSSEFGAPVSVPVQEVLDVICRTLSVSARNISLLGDGPLRLLLLPSLHLEALDLLSALILACGGRLLRFGALISRLL
PQVLSAWSIGRDTLSPGQERPYSTMRTKVYAVLELWVQVCGASAGVLQGGASGEALLTHLLSDISPPADALKLRSPRGSP
DGGLQTGKPSAPKKLKLDMGEPIAPPSHRKGDSNANSDVCAAALRGLSRTILMCGPLIKEETHRRLHELVLPLVMGVQQG
EVLGSSPYTSSRCRRELYRLLLALLLAPSPRCPPPLACALQAFSLGQREDSLEVSSFCSEALVTCAALTHPRVPTLQSMV
PTCPTPAAVPPPEAPSPFRAPAFHPPGPMPSAGPMPSAAPCPRRAPSTANHLGLSGSSLVSVPPRLLPGPENHRAGSNEE

PVLAPSGTPPPTLPSDETFGGRVPRPAFVHYDKEEASDVEISLESDSDDSVVIVPEGLPPLPPPPPSGTTPPPVAPAGPP
AASPPVPAKEEPEELPAAPGPLPPPPPTPVPGPATLPPPQLVPEGTPGGGGAPALEEDLTVININSSDEEEEEEEEEEEE
EEEEEEEEEDFEEEEEDEEEYFEEEEEEEEEFEEEFEEEEGELEEEEEDEEEEEEEELEEVELEFGSAGAEVEEGGPPP
PSLPPALPPAESPKVQPEPEPEPGLLLEVEEPGAEEAPGPETAPTLVPEALPAQGEAEREAGSPPTAPPPQELVEEEPSV
PPTLLEEGAEGGGDKVPPPPETSAAEEMETETESTALQEKVSGPGGKVGRLREGAVAACAA
>Ursus americanus [A0A452R2Q0]
MSTVRQVQWPREERGLSGGGGAPAGGRQGTCLRAPLNEGRRLWLTGSPGTGRGDLENLSAVGALVGLSNARLGSIKTRFE
GLCLLSLLVGESPTEMFQQHCVSWLRSIQQVLQSQDPPPTMELAVAVLRDLLRYAAQLPTLFRDISMNHLPGLLTSLLGL
RPECELSALEGMKACMTYFPRACGSLKGKLASFFLSRVDALSPQLQQLACECYSRLPSLGAGFSQGLKHTESWEQELHSL
LASLHSLLGALYEGAETAPVQYEGPGVEALLTPSEDGDAHVLLRLRQRFCGLARCLGLMLSSEFGAPVSVPVQEILDVIC
RTLSISAKNISLLGDGPLRLLLLPSIHLDALDLLSALILACGGRLLRFGALISRLLPQVLNAWNFGRDTLSPGQERPYST
VRTKVYAVLELWVQVCGASAGVLQGGASGEALLSHLLSDISPPADALKLRSPRGSPDGGLQAGKPSAPKKLKLDMGEAMA
PPSHRKGDSNANSDVCAAALRGLSRTVLMCGPLIKEETHRRLHDLVLPLVMGVQQGEVLGSSPYTSSRCRRELYRLLLAL
LLAPSPRCPPPLACALQAFSLGQREDSLEVSSFCSEALVTCAALTHPRVPPLQSMGPACPTPAPVPPPEAPSPFRAPPFH
TAGPMPSVGPMPSVGPMSSAGPMPSAGPMPPTRPGPPATANHLGLSVPGLVSVPPRLLPGPENHRAGSNEDPVLAPSGTP
PPAIPPDETFGGRVPRPAFVHYDKEEASDVEISLESDSDDSVVIVPEGLPPLPPPPPSGTTPPPVAPAGPPTASPPVPAK
EEPEELPAAPGPLPPPPPPPVPGPVALPPPQLVPEGTPGSGGPPALEEDLTVININSSDEEEEEEEEEEEEEEEEEEEEE
DFEEEEEDEEEYFEEEEEEEEEFEEEFEEEEGELEEEEDEDEEEEEEELEELEEVEFGPAGGPAEEGGPPPASPAPALPPA
QSPKVQPEPEGEPGLLLEVEEPGAEEEAGAEAAPTLAPEVLPSQGEGPRDTGSPPAGPSPQELMEEEPLAPPTLLEEGTE
SGGDKVPPPAETAVAEDVETELETATAALQEKEQDDTAAMLADFIDCPPDDEKPPPAPEPES
>Phyllostomus discolor [A0A833ZNV8]
MMMGGEFWGIPPDCTALEPLSLLTRLRRLLGSGESSDLNLSAVAALVGLSNARLGSIKTRFEGLCLLSLLVGESSTEMFQ
QHCVSWLRSIQQVLQSQDPPPTMELAVAVLRDLLRYAAQLPTLFRDISMNHLPGLLTSLLGLRPECELSALEGMKACMTY
FPRACGSLKGKLASFFLSRVDALSPQLQQLACECYSRLPSLGAGFSQGLKHTESWEQELHSLLASLHSLLGALYEGAEPA
PMQYEGPGVEMLFSPSEDGDAHNLLRLRQRFSGLACCLGLLLRSEFGAPVSIPVQEILDVICRTLSISAKNISLLGDGPL
RLLLLPSIHLEALDLLSALILACGGRLLRFGSLISRLLPQVLNAWSIGRDTLSLGQEKPYSAMRTKVYAVLELWVQVCGA
SAGVLQGGASGEALLTHLLSDISPPADALKLRSPRGSPDGGLQTGKPSAPKKLKLDMGEAVAPPSHRKGDSNANSDVCAA
ALRGLSRTILMCGPLIKEETHRRLHDLVLPLVMSVQQGEVLGSSPYTSSYCRRELYRLLLALLLAPSPRYPPPLACALQA
FSLGQREDNLEVSSFCSEALVTCAALTHPRVPPLQSMGPACPAPAPVPPPEAPSPFRAPPFHPPGPMPSAGTMPSAGPVP
SVGPMPPVGPMPPARPGPPATANHLGLSVPGLASVPPRLLPGPENHRAGSNEDTILAPSGTPPPTIPPDETFGGRVPRPA
FVHYDKEEASDVEISLESDSDDSVVIVPEGLPPLPLPPPAGTTPPPVAPAGPPTASPPLPAKEEPEELPAAPGPLPPPPP
PLPGPVTLQPPQLVPEGTPGGGAPPALEEDLTVININSSDEEEEEEEEEEEEEEEEEEEDFEEEEEDEEEYFEEEEEEEE
FEEEFEEEEGELEEEEEEEEDEEEEEELEEVEELEFGPTGGEVEGGGPPPTSLPPALPPTESPKVQPEPEPEPEPEPGLLL
EVEEPGAIGEPGAETAPTLAPEVLPSQEEMEREGGSPPAGPPPQELVEEEPCAPPTLLEEGTEVGGDEVPPPPETVAAEE
MEPEPEPETPALQEKEQDDTAAMLADFIDCPPDDEKPPPVTEPDS
> Mustela putorius furo [M3YH90]
MFTPSAETPEREEGPRCSAAGLLNLSAVGALVGLSNARLGSIKTRFEGLCLLSLLVGESPTEMFQQHCVSWLRSIQQVLQ
SQDPPPTMELAVAVLRDLLRYAAQLPTLFRDISMNHLPGLLTSLLGLRPECELSALEGMKACMTYFPRACGSLKGKLASF
FLSRVDALSPQLQQLACECYSKLPSLGAGFSQGLKHTESWEQELRSLLASLHSLLGALYEGAETAPMQYEGPGVEMLLTP
SEDGDAHVLLRLRQRFCGLARCLGLMLSSEFGAPVSVPVQEILDVICRALSISAKSISLLGDGPLRLLLLPSIHLDALDL
LSALILACGGRLLRFGALISRLLPQVLNAWNLGRDTLSPGQERPYSTVRAKVYAVLELWVQVCGASAGVLQGGASGEALL
SHLLSDISPPADTLKLRSPRGSPDAGLQSGKPSAPKKLKLDMGDAAAPPSHRKGDANANSDVCAAALRGLSRTVLMCGPL
IKEETHRRLHDLVLPLVMGVQQGEVLGSSPYTSSRCRRELYRLLLALLLAPAPRCPPPLSCAVQAFSLGQREDSLEVASF
CSEALVTCAALTHPRVPPLQSMGPACPTPAPVPPPEAPSPFRAPPFHPPGPMPSVGPMPSVGPMPSASAMPPTGPLPPAG
PLPPTRPGPPATANHLGLSVPGLVSVPPRLHPGPENHRAGSNEDPVLAPSGTPPPPIPPDETFGGRVPRPAFVHYDKEEA
SDVEISLESDSDDSVVIVPEGLPPLPPLPPSGSTPPPVAPAGPPTASPPGPAKEEPEELPTAPGPLPPPPPPPVPGPVAL
PPPQLVPEGTPGGGGPPGLEEDLTVININSSDEEEEEEEEEEEEEEEEEEEEEEEEDFEEEEEDEEEYFEEEEEEEEEFEEE
FEEEEGELEEEEEEEEDEEEEEELEELEEVEFGPTGAPAEESGPPPPSPPPALPPAQSPKAQPEPEGEPGLLLEVEEPGAE
EGPGAEAAPTLVPEVLPSEGEGPPDPESVPAGPPPQELEEEEPSAPPTLLEEGTESGGDEVPVPPETPAVADVEAEAEAE
TAALPEKEQDDTAAMLADFIDCPPDDEKPPPAPEPDS
> Hybrid - Bos Indicus [A0A4W2H3I5]
MSGWKVSRSSVALKVSFWILAPLVLTTCSLSPTARPEPVLYPAHLPGLESWLFHAAHAHSQQARGIQCTGPGPPPHDPSP
RGIRRGVFASSRHATTRAQLKMAAAVLSGPSAGSAAAVPGGPGGLSAVGSGPRLRLMLLESVSGLLQPRTGSAVAPVHPP
ARSAPHLPGLMCLLRLHGTVGGAQSQDPPPTMELAVTVLKDLLRYAAQLPAVFRDISMNHLPGLLTSLLGLRPECELSAL
EGMKACMTHFPRACGSLKGKLASFFLSRVDALSPQLQQLACECYSRLPSLGAGFSQGLKHTDSWEQELRSLLASLHSLLG
GLYEGAEAAPMQYESPGAETLLSSSEDADAHTLLRLRQRFSGLARCLGLMLSSEFGAPVSVPVQDILDLICRTLSVSAKN
VSLLGDGPLRLLLLPSLHLEALDLLSALILACGARLLRFGALISRLLPQVLNAWSIGRENLGPGQERPYSTVRTKVYAVL
ELWVQVCGASAGVLQGGASGEALLSHLLSDISPPADALRLRSPRGSPDAGLQTGKPSAPKKLKLDVGEAIAPPSHRKGDS
NANSDVCAAALRGLSRTILMCGPLIKEETHRRLHELVLPLVMGVQQGEALGSSPYTSSHCRRELYHLLLALLLAPSPRCP
PPLACALRAFSLGQREDSLEVSSFCSEALVTCAALTHPRVPPLQSVGPTCPAPAPVPPPEAPAPFRAPAFHAPSPLPSAG
PMPSAGPMPPVGPLPPTRPGPPATANHLGLSVPGLVSVPPRLLPGPENHRAGSSEDPVLAPSGSPPPTIPPDETFGGRVP
RPAFVHYDKEEPSDVEISLESDSDDSVVIVPEGLPPPPPPSSGTTPPPVAPAGPPAASPPVPAKDEPEELPAAPGPLPPP
PPPPVPGPVTLPPPQLVPEGTPGGGGPPALEEDMTVININSSDEEEEEEEEEEEEDEEEDEEEDFEEDEEEEEEYFEEEE
EEEEEFEEEFEEEEGELEDEEDEEEDEELEELEEVEFGPAGGEVEGGGPAPPSLPPALPPAESPKGPPEPGLEPGLLLEV
EEPGTEEAPGPETAPMLAPEVLPSQGEVEREGGSPPAGPPPQELVEEEPSGPPALLEEGAEGGGDKVSPPPEASAVEETE
VEAAALPPEKEQGDTAAMLADFIDCPPDDEKPPPASEPDS
> Bos indicus x Bos taurus [A0A4W2CB06]
MMAVPPCEDDQGVQIDCYCGENLSAVGALVGLSNARLGSIKTRFEGLCLLSLLVGESPTEMFQQHCVSWLRSIQQILQSQ
DPPPTMELAVTVLKDLLRYAAQLPAVFRDISMNHLPGLLTSLLGLRPECELSALEGMKACMTHFPRACGSLKGKLASFFL
SRVDALSPQLQQLACECYSRLPSLGAGFSQGLKHTDSWEQELRSLLASLHSLLGGLYEGAEAAPMQYESPGAETLLSSSE
DADAHTLLRLRQRFSGLARCLGLMLSSEFGAPVSVPVQDILDLICRTLSVSAKNVSLLGDGPLRLLLLPSLHLEALDLLS
ALILACGARLLRFGALISRLLPQVLNAWSIGRENLGPGQERPYSTVRTKVYAVLELWVQVCGASAGVLQGGASGEALLSH
LLSDISPPADALRLRSPRGSPDAGLQTGKPSAPKKLKLDVGEAIAPPSHRKGDSNANSDVCAAALRGLSRTILMCGPLIK
EETHRRLHELVLPLVMGVQQGEALGSSPYTSSHCRRELYHLLLALLLAPSPRCPPPLACALRAFSLGQREDSLEVSSFCS
EALVTCAALTHPRVPPLQSVGPTCPAPAPVPPPEAPAPFRAPAFHAPSPLPSAGPMPSAGPMPPVGPLPPTRPGPPATAN
HLGLSVPGLVSVPPRLLPGPENHRAGSSEDPVLAPSGSPPPTIPPDETFGGRVPRPAFVHYDKEEPSDVEISLESDSDDS
VVIVPEGLPPPPPPSSGTTPPPVAPAGPPAASPPVPAKDEPEELPAAPGPLPPPPPPPVPGPVTLPPPQLVPEGTPGGGG

PPALEEDMTVININSSDEEEEEEEEEEEEDEEEDEEEDFEEDEEEEEEEYFEEEEEEEEFEEEFEEEEGELEDEEDEEED
EELEELEEVEFGPAGGEVEGGGPAPPSLPPALPPAESPKGPPEPGLEPGLLLEVEEPGTEEAPGPETAPMLAPEVLPSQG
EVEREGGSPPAGPPPQELVEEEPSGPPALLEEGAEGGGDKVSPPPEASAVEETEVEAAALPPEKEQGDTAAMLADFIDCP
PDDEKPPPASEPDS
>Bos taurus [A0A3Q1MDL1]
MLSVAFPFSFHLVNYNSQGLLRMLAARPHRVTTCGKTEYRKFPQSKAIQTCPPSLHCCGLFNFFYKNFLSVCGVLGDDGR
TSGVLQTGQPWTLCLPIPLRHLSEPGENDLNLSAVGALVGLSNARLGSIKTRFEGLCLLSLLVGESPTEMFQQHCVSWLR
SIQQILQSQDPPPTMELAVTVLKDLLRYAAQLPAVFRDISMNHLPGLLTSLLGLRPECELSALEGMKACMTHFPRACGSL
KGKLASFFLSRVDALSPQLQQLACECYSRLPSLGAGFSQGLKHTDSWEQELRSLLASLHSLLGGLYEGAEAAPMQYESPG
AETLLSSSEDADAHTLLRLRQRFSGLARCLGLMLSSEFGAPVSVPVQDILDLICRTLSVSAKNVSLLGDGPLRLLLLPSL
HLEALDLLSALILACGARLLRFGALISRLLPQVLNAWSIGRENLGPGQERPYSTVRTKVYAVLELWVQVCGASAGVLQGG
ASGEALLSHLLSDISPPADALRLRSPRGSPDAGLQTGKPSAPKKLKLDVGEAIAPPSHRKGDSNANSDVCAAALRGLSRT
ILMCGPLIKEETHRRLHELVLPLVMGVQQGEALGSSPYTSSHCRRELYHLLLALLLAPSPRCPPPLACALRAFSLGQRED
SLEVSSFCSEALVTCAALTHPRVPPLQSVGPTCPAPAPVPPPEAPAPFRAPAFHAPSPLPSAGPMPSAGPMPPVGPLPPT
RPGPPATANHLGLSVPGLVSVPPRLLPGPENHRAGSSEDPVLAPSGSPPPTIPPDETFGGRVPRPAFVHYDKEEPSDVEI
SLESDSDDSVVIVPEGLPPPPPPSSGTTPPPVAPAGPPAASPPVPAKDEPEELPAAPGPLPPPPPPPVPGPVTLPPPQLV
PEGTPGGGGPPALEEDMTVININSSDEEEEEEEEEEEEDEEEDEEEDFEEDEEEEEEEYFEEEEEEEEFEEEFEEEEGEL
EDEEDEEEDEELEELEEVEFGPAGGEVEGGGPAPPSLPPALPPAESPKGPPEPGLEPGLLLEVEEPGTEEAPGPETAPML
APEVLPSQGEVEREGGSPPAGPPPQELVEEEPSGPPALLEEGAEGGGDKVSPPPEASAVEETEVEAAALPPEKEQGDTAA
MLADFIDCPPDDEKPPPASEPDS
>Ursus americanus [A0A452R2Q5]
VSLPTPGSPLPPESANLSAVGALVGLSNARLGSIKTRFEGLCLLSLLVGESPTEMFQQHCVSWLRSIQQVLQSQDPPPTM
ELAVAVLRDLLRYAAQLPTLFRDISMNHLPGLLTSLLGLRPECELSALEGMKACMTYFPRACGSLKGKLASFFLSRVDAL
SPQLQQLACECYSRLPSLGAGFSQGLKHTESWEQELHSLLASLHSLLGALYEGAETAPVQYEGPGVEALLTPSEDGDAHV
LLRLRQRFCGLARCLGLMLSSEFGAPVSVPVQEILDVICRTLSISAKNISLLGDGPLRLLLLPSIHLDALDLLSALILAC
GGRLLRFGALISRLLPQVLNAWNFGRDTLSPGQERPYSTVRTKVYAVLELWVQVCGASAGVLQGGASGEALLSHLLSDIS
PPADALKLRSPRGSPDGGLQAGKPSAPKKLKLDMGEAMAPPSHRKGDSNANSDVCAAALRGLSRTVLMCGPLIKEETHRR
LHDLVLPLVMGVQQGEVLGSSPYTSSRCRRELYRLLLALLLAPSPRCPPPLACALQAFSLGQREDSLEVSSFCSEALVTC
AALTHPRVPPLQSMGPACPTPAPVPPPEAPSPFRAPPFHTAGPMPSVGPMPSVGPMSSAATANHLGLSVPGLVSVPPRLL
PGPENHRAGSNEDPVLAPSGTPPPAIPPDETFGGRVPRPAFVHYDKEEASDVEISLESDSDDSVVIVPEGLPPLPPPPPS
GTTPPPVAPAGPPTASPPVPAKEEPEELPAAPGPLPPPPPPPVPGPVALPPPQLVPEGTPGSGGPPALEEDLTVININSS
DEEEEEEEEEEEEEEEEEEEEEDFEEEEEDEEEYFEEEEEEEEEEFEEEFEEEEGELEEEEDEDEEEEELEELEEVEFGP
AGGPAEEGGPPPASPAPALPPAQSPKVQPEPEGEPGLLLEVEEPGAEEEAGAEAAPTLAPEVLPSQGEGPRDTGSPPAGP
SPQELMEEEPLAPPTLLEEGTESGGDKVPPPAETAVAEDVETELETATAALQEKVGGKAGWGAAAVCVCVCVCVCVRLFP
ILRLCP
>Gorilla gorilla gorilla [A0A2I2YWX5]
MELAVAVLRDLLRYAAQLPALFRDISMNHLPGLLTSLLGLRPECEQSALEGMKACMTYFPRACGSLKGKLASFFLSRVDA
LSPQLQQLACECYSRLPSLGAGFSQGLKHTESWEQELHSLLASLHTLLGALYEGAETAPVQNEGPGVEMLLSSEDGDAHV
LLQLRQRFSGLACCLGLMLSSEFGAPVSVPVQEILDFICRTLSVSSKNISLHGDGPLRLLLLPSIHLEALDLLSALILAC
GSRLLRFGILISRLLPQVLNSWSIGRDSLSPGQERPYSTVRTKVYAILELWVQVCGASAGMLQGGASGEALLTHLLSDIS
PPADALKLRSPRGSPDGSLQTGKPSAPKKLKLDVGEAMAPPSHRKGDSNANSDVCAAALRGLSRTILMCGPLIKEETHRR
LHDLVLPLVMGVQQGEVLGSSPYTSSRCRRELYCLLLALLLAPSPRCPPPLACALQAFSLGQREDSLEVSSFCSEALVTC
AALTHPRVPPLQPMGPTCPTPAPVPPPEAPSPFRAPPFHPPGPMPSVGSMPSAGPMPSAGPMPSAGPVPSARPGPPTTAN
HLGLSVPGLVSVPPRLLPGPENHRAGSNEDPILAPSGTPPPTIPPDETFGGRVPRPAFVHYDKEEASDVEISLESDSDDS
VVIVPEGLPPLPPPPPSGATPPPIAPTGPPTASPPVPAKEEPEELPAAPGPLPPPPPPPPPVPGPVTLPPPQLVPEGTPG
GGGPPALEEDLTVININSSDEEEEEEEEEEEEEEEEEEEEDFEEEEEDEEEYFEEEEEEEEEEFEEEFEEEEGELEEEEE
EEDEEEEEELEEVEREGESPAAGPPPQELVEEEPSAPPTLLEEETEDGSDKVQPPPETPAEEEMETETEAEALQEKEQDD
TAAMLADFIDCPPDDEKPPPPTEPDS
> Phyllostomus discolor [A0A833ZFJ8]
MFQQHCVSWLRSIQQVLQSQDPPPTMELAVAVLRDLLRYAAQLPTLFRDISMNHLPGLLTSLLGLRPECELSALEGMKAC
MTYFPRACGSLKGKLASFFLSRVDALSPQLQQLACECYSRLPSLGAGFSQGLKHTESWEQELHSLLASLHSLLGALYEGA
EPAPMQYEGPGVEMLFSPSEDGDAHNLLRLRQRFSGLACCLGLLLRSEFGAPVSIPVQEILDVICRTLSISAKNISLLGD
GPLRLLLLPSIHLEALDLLSALILACGGRLLRFGSLISRLLPQVLNAWSIGRDTLSLGQEKPYSAMRTKVYAVLELWVQV
CGASAGVLQGGASGEALLTHLLSDISPPADALKLRSPRGSPDGGLQTGKPSAPKKLKLDMGEAVAPPSHRKGDSNANSDV
CAAALRGLSRTILMCGPLIKEETHRRLHDLVLPLVMSVQQGEVLGSSPYTSSYCRRELYRLLLALLLAPSPRYPPPLACA
LQAFSLGQREDNLEVSSFCSEALVTCAALTHPRVPPLQSMGPACPAPAPVPPPEAPSPFRAPPFHPPGPMPSAGTMPSAG
PVPSVGPMPPVGPMPPARPGPPATANHLGLSVPGLASVPPRLLPGPENHRAGSNEDTILAPSGTPPPTIPPDETFGGRVP
RPAFVHYDKEEASDVEISLESDSDDSVVIVPEGLPPLPLPPPAGTTPPPVAPAGPPTASPPLPAKEEPEELPAAPGPLPP
PPPPLPGPVTLQPPQLVPEGTPGGGAPPALEEDLTVININSSDEEEEEEEEEEEEEEEEEEEDFEEEEEDEEEYFEEEEE
EEEFEEEFEEEEGELEEEEEEEDEEEEEELEEVEELEFGPTGGEVEGGGPPPTSLPPALPPTESPKVQPEPEPEPEPEPG
LLLEVEEPGAIGEPGAETAPTLAPEVLPSQEEMEREGGSPPAGPPPQELVEEEPCAPPTLLEEGTEVGGDEVPPPPETVA
AEEMEPEPEPETPALQEKEQDDTAAMLADFIDCPPDDEKPPPVTEPDS
> Macaca nemestrina [A0A2K6BMK1]
MELAVAVLRDLLRYAAQLPALFRDISMNHLPGLLTSLLGLRPECEQSALEGMKACMTYFPRACGSLKGKLASFFLSRVDA
LSPQLQQLACECYSRLPSLGAGFSQGLKHTESWEQELHSLLASLHTLLGALYEGAETAPVQNEGPGVEMLLSSEDGDAHV
LLRLRQRFSGLARCLGLMLSSEFGAPVSVPVQEILDFICRTLSVSSKNISLHGDGPLRLLLLPSIHLEALDLLSALILAC
GSRLLRFGILISRLLPQVLNSWSIGRDSLSPGQERPYSTVRTKVYAVLELWVQVCGASAGMLQGGASGEALLTHLLSDIS
PPADALKLRSPRGSPDGSLQTGKPSAPKKLKLDVGEAMAPPSHRKGDSNANSDVCAAALKGLSRTILMCGPLIKEETHRR
LHDLVLPLVMGVQQGEVLGSSPYTSSRCRRELYCLLLALLLAPSPRCPPPLACALQAFSLGQREDSLEVSSFCSEALVTC
AALTHPRVPPLQPMGPTCPTPAPVPPPEAPSPFRAPPFHPPGPMPSVGPMPSAGPMPSAGPMPSAGPVPSARPGPPTTAN
HLGLSVSGLVSVPPRLLPGPENHRAGSNEDPILAPSGTPPPTIPPDETFGGRVPRPAFVHYDKEEASDVEISLESDSDDS
VVIVPEGLPPLPPPPPSGATPPPIAPTGPPTASPPVPAKEEPEELPAAPGPLPPPPPPPPPVPGPVTLPPPQLVPEGTPG
GGGPPALEEDLTVININSSDEEEEEEEEEEEEEEEEEEEEDFEEEEEDEEEYFEEEEEEEEEEFEEEFEEEEGELEEEEE
EEDEEEEEELEEVEREEGSPSAGPPPQELVEEEPSAPPTLLEEGTEDGGDRVQPPPETPAEEEMETETEAEALQEKEQDD
TAAMLADFIDCPPDDEKPPPPTEPDS
>Macaca fascicularis [A0A2K5U1C5]
MELAVAVLRDLLRYAAQLPALFRDISMNHLPGLLTSLLGLRPECEQSALEGMKACMTYFPRACGSLKGKLASFFLSRVDA

LSPQLQQLACECYSRLPSLGAGFSQGLKHTESWEQELHSLLASLHTLLGALYEGAETAPVQNEGPGVEMLLSSEDGDAHV
LLRLRQRFSGLARCLGLMLSSEFGAPVSVPVQEILDFICRTLSVSSKNISLHGDGPLRLLLLPSIHLEALDLLSALILAC
GSRLLRFGILISRLLPQVLNSWSIGRDSLSPGQERPYSTVRTKVYAVLELWVQVCGASAGMLQGGASGEALLTHLLSDIS
PPADALKLRSPRGSPDGSLQTGKPSAPKKLKLDVGEAMAPPSHRKGDSNANSDVCAAALKGLSRTILMCGPLIKEETHRR
LHDLVLPLVMGVQQGEVLGSSPYTSSRCRRELYCLLLALLLAPSPRCPPPLACALQAFSLGQREDSLEVSSFCSEALVTC
AALTHPRVPPLQPMGPTCPTPAPVPPPEAPSPFRAPPFHPPGPMPSVGPMPSAGPMPSAGPMPSAGPVPSARPGPPTTAN
HLGLSVSGLVSVPPRLLPGPENHRAGSNEDPILAPSGTPPPTIPPDETFGGRVPRPAFVHYDKEEASDVEISLESDSDDS
VVIVPEGLPPLPPPPPSGATPPPIAPTGPPTASPPVPAKEEPEELPAAPGPLPPPPPPPPPVPGPVTLPPPQLVPEGTPG
GGGPPALEEDLTVININSSDEEEEEEEEEEEEEEEEEEEEEEEDFEEEEDEEEYFEEEEEEEEEFEEEFEEEEGELEEEEE
EEDEEEEEEELEEVEREEGSPSAGPPPQELVEEPSAPPTLLEEGTEDGGDRVQPPPETPAEEEMETETEAEALQEKEQDD
TAAMLADFIDCPPDDEKPPPPTEPDS
>Pan troglodytes [A0A2I3RFU6]
MELAVAVLRDLLRYAAQLPALFRDISMNHLPGLLTSLLGLRPECEQSALEGMKACMTYFPRACGSLKGKLASFFLSRVDA
LSPQLQQLACECYSRLPSLGAGFSQGLKHTESWEQELHSLLASLHTLLGALYEGAETAPVQNEGPGVEMLLSSEDGDAHV
LLQLRQRFSGLARCLGLMLSSEFGAPVSVPVQEILDFICRTLSVSSKNISLHGDGPLRLLLLPSIHLEALDLLSALILAC
GSRLLRFGILIGRLLPQVLNSWSIGRDSLSPGQERPYSTVRTKVYAILELWVQVCGASAGMLQGGASGEALLTHLLSDIS
PPADALKLRSPRGSPDGSLQTGKPSAPKKLKLDVGEAMAPPSHRKGDSNANSDVCAAALRGLSRTILMCGPLIKEETHRG
PLLCTHWGRLHDLVLPLVMGVQQGEVLGSSPYTSSRCRRELYCLLLALLLAPSPRCPPPLACALQAFSLGQREDSLEVSS
FCSEALVTCAALTHPRVPPLQPMGPTCPTPAPVPPPEAPSPFRAPPFHPPGPMPSVGSMPSAGPMPSAGPMPSAGPVSSA
RPGPPTTANHLGLSVPGLVSVPPRLLPGPENHRAGSNEDPILAPSGTPPPTIPPDETFGGRVPRPAFVHYDKEEASDVEI
SLESDSDDSVVIVPEGLPPLPPPPPSGATPPPIAPTGPPTASPPVPAKEEPEELPAAPGPLPPPPPPPPPVPGPVTLPPP
QLVPEGTPGGGGPPALEEDLTVININSSDEEEEEEEEEEEEEEEEEEEEEEEDFEEEEDEEEYFEEEEEEEEEFEEDFEEE
EGELEEEEEEEDDEEEELEDVEREGESPAAGPPPQELVEEEPSAPPTLLEEETEDGSDKVQPPPETPAEEEMETETEAEA
LQEKEQDDTAAMLADFIDCPPDDEKPPPPTEPDS
>Papio anubis [A0A2I3LUZ2]
MELAVAVLRDLLRYAAQLPALFRDISMNHLPGLLTSLLGLRPECEQSALEGMKACMTYFPRACGSLKGKLASFFLSRVDA
LSPQLQQLACECYSRLPSLGAGFSQGLKHTESWEQELHSLLASLHTLLGALYEGAETAPVQNEGPGVEMLLSSEDGDAHI
LLRLRQRFSGLARCLGLMLSSEFGAPVSVPVQEILDFICRTLSVSSKNISLHGDGPLRLLLLPSIHLEALDLLSALILAC
GSRLLRFGILISRLLPQVLNSWSIGRDSLSPGQERPYSTVRTKVYAVLELWVQVCGASAGMLQGGASGEALLTHLLSDIS
PPADALKLRSPRGSPDGSLQTGKPSAPKKLKLDVGEAMAPPSHRKGDSNANSDVCAAALKGLSRTILMCGPLIKEETHRR
LHDLVLPLVMGVQQGEVLGSSPYTSSRCRRELYCLLLALLLAPSPRCPPPLACALQAFSLGQREDSLEVSSFCSEALVTC
AALTHPRVPPLQPMGPTCPTPAPVPPPEAPSPFRAPPFHPPGPMPSVGPMPSAGPMPSAGPMPSAGPVPSARPGPPTTAN
HLGLSVSGLVSVPPRLLPGPENHRAGSNEDPILAPSGTPPPAIPPDETFGGRVPRPAFVHYDKEEASDVEISLESDSDDS
VVIVPEGLPPPPPPPPPGATPPPIAPTGPPTASPPVPAKEEPEELPAAPGPLPPPPPPPPPVPGPVTLPPPQLVPEGTPGG
GGPPALEEDLTVININSSDEEEEEEEEEEEEEEEEEEEEEEEDFEEEEDEEEYFEEEEEEEEEFEEEFEEEEGELEEEEEE
EDEEEEEEELEEVEREEGSPSAGPPPQELVEEEPSAPPTLLEEGTEDGGDRVQPPPETPAEEEMETETEAEALQEKEQDDT
AAMLADFIDCPPDDEKPPPPTEPDS
>Cercocebus atys [A0A2K5LPS0]
MELAVAVLRDLLRYAAQLPALFRDISMNHLPGLLTSLLGLRPECEQSALEGMKACMTYFPRACGSLKGKLASFFLSRVDA
LSPQLQQLACECYSRLPSLGAGFSQGLKHTESWEQELHSLLASLHTLLGALYEGAETAPVQNEGPGVEMLLSSEDGDAHV
LLRLRQRFSGLARCLGLMLSSEFGAPVSVPVQEILDFICRTLSVSSKNISLHGDGPLRLLLLPSIHLEALDLLSALILAC
GSRLLRFGILISRLLPQVLNSWSIGRDSLSPGQERPYSTVRTKVYAVLELWVQVCGASAGMLQGGASGEALLTHLLSDIS
PPADALKLRSPRGSPDGSLQTGKPSAPKKLKLDVGEAMAPPSHRKGDSNANSDVCAAALKGLSRTILMCGPLIKEETHRR
LHDLVLPLVMGVQQGEVLGSSPYTSSRCRRELYCLLLALLLAPSPRCPPPLACALQAFSLGQREDSLEVSSFCSEALVTC
AALTHPRVPPLQPMGPTCPTPAPVPPPEAPSPFRAPPFHPPGPMPSVGPMPSAGPMPSAGPMPSAGPVPSARPGPPTTAN
HLGLSVSGLVSVPPRLLPGPENHRAGSNEDPILAPSGSPPPAIPPDETFGGRVPRPAFVHYDKEEASDVEISLESDSDDS
VVIVPEGLPPLPPPPPSGATPPPIAPTGPPTASPPVPAKEEPEELPAAPGPLPPPPPPPPPVPGPVTLPPPQLVPEGTPG
GGGPPALEEDLTVININSSDEEEEEEEEEEEEEEEEEEEEEEEDFEEEEDEEEYFEEEEEEEEEFEEEFEEEEGELEEEEE
EEDEEEEEEELEEVEREEGSPSAGPPPQELVEEEPSAPPTLLEEGTEDGGDRVQPPPETPAEEEMETETEAEALQEKEQDD
TAAMLADFIDCPPDDEKPPPPTEPDS
>Pan paniscus [A0A2R9BQ99]
MELAVAVLRDLLRYAAQLPALFRDISMNHLPGLLTSLLGLRPECEQSALEGMKACMTYFPRACGSLKGKLASFFLSRVDA
LSPQLQQLACECYSRLPSLGAGFSQGLKHTESWEQELHSLLASLHTLLGALYEGAETAPVQNEGPGVEMLLSSEDGDAHV
LLQLRQRFSGLARCLGLMLSSEFGAPVSVPVQEILDFICRTLSVSSKNISLHGDGPLRLLLLPSIHLEALDLLSALILAC
GSRLLRFGILIGRLLPQVLNSWSIGRDSLSPGQERPYSTVRTKVYAILELWVQVCGASAGMLQGGASGEALLTHLLSDIS
PPADALKLRSPRGSPDGSLQTGKPSAPKKLKLDVGEAMAPPSHRKGDSNANSDVCAAALRGLSRTILMCGPLIKEETHRG
PLLCTHWGRLHDLVLPLVMGVQQGEVLGSSPYTSSRCRRELYCLLLALLLAPSPRCPPPLACALQAFSLGQREDSLEVSS
FCSEALVTCAALTHPRVPPLQPMGPTCPTPAPVPPPEAPSPFRAPPFHPPGPMPSVGSMPSAGPMPSAGPMPSAGPVSSA
RPGPPTTANHLGLSVPGLVSVPPRLLPGPENHRAGSNEDPILAPSGTPPPTIPPDETFGGRVPRPAFVHYDKEEASDVEI
SLESDSDDSVVIVPEGLPPLPPPPPSGATPPPIAPTGPPTASPPVPAKEEPEELPAAPGPLPPPPPPPPPVPGPVTLPPP
QLVPEGTPGGGGPPALEEDLTVININSSDEEEEEEEEEEEEEEEEEEEEEEEDFEEEEDEEEYFEEEEEEEEEFEEDFEEEE
GELEEEEEEEDDEEEELEDVEREGESPAAGPPPQELVEEEPSAPPTLLEEETEDGSDKVQPPPETPAEEEMETETEAEAL
QEKEQDDTAAMLADFIDCPPDDEKPPPPTEPDS
>Colobus angolensis palliatus [A0A2K5ICZ2]
MELAVAVLRDLLRYAAQLPALFRDISMNHLPGLLTSLLGLRPECEQSALEGMKACMTYFPRACGSLKGKLASFFLSRVDA
LSPQLQQLACECYSRLPSLGAGFSQGLKHTESWEQELHSLLASLHTLLGALYEGAETAPVQNEGPGVEMLLSSEDGDAHV
LLRLLQRFSGLACCLGLMLSSEFGAPVSVPVQEILDFICRTLSVSSKNISLHGDGPLRLLLLPSIHLEALDLLSALILAC
GSRLLRFGILISRLLPQVLNSWSIGRDSLSPGQERPYSTVRTKVYAVLELWVQVCGASAGMLQGGASGEALLTHLLSDIS
PPADALKLRSPRGSPDGSLQTGKPSAPKKLKLDVGEAMAPPSHRKGDSNANSDVCAAALRGLSRTILMCGPLIKEETHRR
LHDLVLPLVMGVQQGEVLGSSPYTSSRCRRELYCLLLALLLAPSPRCPPPLACALQAFSLGQREDSLEVSSFCSEALVTC
AALTHPRVPPLQPMGPTCPTPAPVPPPEAPSPFRAPPFHPPGPMPSVGPMPSAGPMPSAGPVPSARPGPPTTAN
HLGLSVSGLVSVPPRLLPGPENHRAGSNEDPILAPSGTPPPAIPPDETFGGRVPRPAFVHYDKEEASDVEISLESDSDDS
VVIVPEGLPPLPPPPPSGATPPPIAPTGPPTASPPVPAKEEPEELPAAPGPLPPPPPPPPPVPGPVTLPPPQLVPEGTPG
GGGPPALEEDLTVININSSDEEEEEEEEEEEEEEEEEEEEEEEDFEEEEDEEEYFEEEEEEEEEFEEEFEEEEGELEEEEE
EEDEEEEEEELEEVEREEGSPAAGPPPQELVEEEPSAPPTLLEEGTEDGGDRVQPPPETPAEEEMETETEAEALQEKEQDD
TAAMLADFIDCPPDDEKPPPPTEPDS
>Rousettus aegyptiacus [A0A7J8GDS8]

MFQQHCVSWLRSIQQVLQSQDPPPTMELAVAVLRDLLRYAAQLPTLFRDISMNHLPGLLTSLLGLRPECELSALEGMKAC
MTYFPRACGSLKGKLASFFLSRVDALSPQLQQLACECYSQVPSLGAGFSQGLKHTESWEQELHSLLASLHSLLGALYEGA
EPAPMQYEGPGVEMLLSPSEDGDAHILLRLWQRFSGLARCLGLMLSSEFGAPVSVPVQEILDVICRTLSISAKNISLLGD
GPLRLLLLPSIHLEALDLLSALILACRGRLLRFGALISRLLPQVLNAWSIGRDSLSPGQEKPYSAMRTKVYAILELWVQV
CGASAGVLQGGASGEALLTHLLSDISPSADTLKLRSPRGSPDGGLQMGKPSAPKKLKLDVGEAMAPPSHRKGDSNANSDV
CAAALRGLSQTILMCGPLIKEETHRRLHDLVLPLVMGVQQGEILGSSPYTSSCCRRELYHLLLALLLAPSPRCPPPLACA
LQAFSLGQREDSLEVSSFCSEALVTCAALTHPRVPPLQSMGPTCPTPAPVPPPEAPSPFRAPPFHPPGPVPSAGPMPSAG
PVPSVGSMPSVGSMPSAGPMPSAGPMPPTRPGPPATANHLGLSVPGLVSLPPRLLPGPENHRSGSNEDPILAPSGTPPPT
IPPDETFGGRVPRPAFVHYDKEEASDVEISLESDSDDSVVIVPEGLPPLPPPPASGTTPPPVTPAGPPTASPPLPTQEEP
EELPVALGPLPPPPPPSVPAPVTLAPPQLVPEGTPGGGGPPALEEDLTVININSSDEEEEEEEEEEDEEEEEEEEDFEEEEE
DEEEYFEEEEEEEEEFEEEYEEEEGELEEDEEEDEEEEEEELEEVEELEFGSAGGEIEEGGPPPPNLPPALPPVESPKLQP
EPEPEPEPEPEPGLLLEVEEPGAEEEHGAETAPTLAPEVLSSQGELEREAGSPPAGPPPQELVEEEPCAPPTLLEEGTEV
GGDKVPPPPPETSAAEEMQTEKEATALQEKEQDDTAAMLADFIDCPPDDEKPPPAAEPDS
> Aotus nancymaae [A0A2K5EHP3]
MELAVAVLRDLLRYAAQLPALFRDISMNHLPGLLTSLLGLRPECEQSALEGMKACMTYFPRACGSLKGKLASFFLSRVDA
LSPQLQQLACECYSRLPSLGAGFSQGLKHTESWEQELHSLLASLHTLLGALYEGAETAPVQNEGPGVEMLLSSEDGDAHV
LLRLRQRFSGLARCLGLMLSSEFGAPVSVPVQEILDFICRTLSVNSKNISLHGDGPLRLLLLPSIHLEALDLLSALILAC
GSRLLRFGILISRLLPQVLNSWSIGRDSLSPGQERPYSTVRTKVYAVLELWVQVCGASAGVLQGGASGEALLTHLLSDIS
PPADALKLRSPRGSPDGGLQTGKPSAPKKLKLDVGEAMAPPSHRKGDSNANSDVCAAALRGLSRTILMCGPLIKEETHRR
LHDLVLPLVMGVQQGEVLGSSPYTSSRCRRELYCLLLALLLAPSPRCPPPLACALQAFSLGQREDSLEVSSFCSEALVTC
AALTHPRVPPLQSMGPTCPTPAPVPPPEAPSPFRAPPFHPPVGPMPSAGPMPSAGPMPSAGPMPSAGPVPSARPGPPTTA
NHLGLSVPGLVSVPPRLLPGPENHRASSNEDPVLAPSGTPPPTIPPDETFGGRVPRPAFVHYDKEEASDVEISLESDSDD
SVVIVPEGLPTLPPPPPSGATPPPVAPSGPPTASPPVPAKEDPEELPAAPGPLPPPPPPPPPVPGPVTLPPPQLVPEGTP
GGGGPPALEEDLTVININSSDEEEEEEEEEEEEEEEEEEEEEEDFEEEEEDEEEYFEEEEEEEEEEFEEEFEEEEAPTLAPE
VLPSQGEVERDGGSPAAGLPPQELVEEEPSAPPTLLEEGTEDGGDKVQLPPETSAEEEMETETEAEALQEKEQDDTAAML
ADFIDCPPDDEKPPPATEPDS
>Phyllostomus discolor [A0A833ZLH4]
MELAVAVLRDLLRYAAQLPTLFRDISMNHLPGLLTSLLGLRPECELSALEGMKACMTYFPRACGSLKGKLASFFLSRVDA
LSPQLQQLACECYSRLPSLGAGFSQGLKHTESWEQELHSLLASLHSLLGALYEGAEPAPMQYEGPGVEMLFSPSEDGDAH
NLLRLRQRFSGLACCLGLLLRSEFGAPVSIPVQEILDVICRTLSISAKNISLLGDGPLRLLLLPSIHLEALDLLSALILA
CGGRLLRFGSLISRLLPQVLNAWSIGRDTLSLGQEKPYSAMRTKVYAVLELWVQVCGASAGVLQGGASGEALLTHLLSDI
SPPADALKLRSPRGSPDGGLQTGKPSAPKKLKLDMGEAVAPPSHRKGDSNANSDVCAAALRGLSRTILMCGPLIKEETHR
RLHDLVLPLVMSVQQGEVLGSSPYTSSYCRRELYRLLLALLLAPSPRYPPPLACALQAFSLGQREDNLEVSSFCSEALVT
CAALTHPRVPPLQSMGPACPAPAPVPPPEAPSPFRAPPFHPPGPMPSAGTMPSAGPVPSVGPMPPVGPMPPARPGPPATA
NHLGLSVPGLASVPPRLLPGPENHRAGSNEDTILAPSGTPPPTIPPDETFGGRVPRPAFVHYDKEEASDVEISLESDSDD
SVVIVPEGLPPLPLPPPAGTTPPPVAPAGPPTASPPLPAKEEPEELPAAPGPLPPPPPPLPGPVTLQPPQLVPEGTPGGG
APPALEEDLTVININSSDEEEEEEEEEEEEEEEEEEEEDFEEEEEDEEEYFEEEEEEEEEFEEEFEEEEGELEEEEEEEEDEE
EEEELEEVEELEFGPTGGEVEGGGPPPTSLPPALPPTESPKVQPEPEPEPEPEPEPGLLLEVEEPGAIGEPGAETAPTLAPE
VLPSQEEMEREGGSPPAGPPPQELVEEEPCAPPTLLEEGTEVGGDEVPPPPETVAAEEMEPEPEPETPALQEKEQDDTAA
MLADFIDCPPDDEKPPPVTEPDS
>Ovis aries [W5PE20]
PIRPALSPGTQARGIQCAGPGPPPHDPSPRDIRRGVFASSRHATTRAQLKMAAAVLSGPSAGSAAAVPGGAGGLSAVGSG
PRLRLMLLESVSGLLQPRTGSAVAPVHPPARSAPHLPGLMCLLRLHGTVGGAQNLSAVGALVGLSNARLGSIKTRFEGLC
LLSLLVGESPTEMFQQHCVSWLRSIQQILQSQDPPPTMELAVTVLKDLLRYAAQLPAVFRDISMNHLPGLLTSLLGLRPE
CELSALEGMKACMTHFPRACGSLKGKLASFFLSRVDALSPQLQQLACECYSRLPSLGAGFSQGLKHTDSWEQELRSLLAS
LHSLLGGLYEGAEAAPMQYESPGAETLLSPSEDVDAHTLLRLRQRFSGLARCLGLMLSSEFGAPVSVPVQDILDLICRTL
SVSAKNVSLLGDGPLRLLLLPSLHLEALDLLSALILACGARLLRFGALISRLLPQVLSAWSIGRENLGPGQERPYSTVRT
KVYAVLELWVQVCGASAGVLQGGASGEALLSHLLSDISPPADALRLRSPRGSPDAGLQTGKPSAPKKLKLDVGEAIAPPS
HRKGDSNANSDVCAAALRGLSRTILMCGPLIKEETHRRLHELVLPLVMGVQQGEALCSSPYTSSHCRRELYRLLLAPSPR
CPPPLACALRAFSMGQQEDSLEVSSFCSEALVTCAALTHPRVPPLQSVGPTCPAPAPVPPPEAPAPFRAPAFHAPGPLPS
AGPMPSAGPMPPAGPPSGSPPPTVPPDETFGGRVPRPAFVHYDKEEASDVEISLESDSDDSVVIVPEGLPPPPPPPSGTT
PPPVAPAGPPAASPPVPAKDEPEELPAAPGPLPPPPPPPVPGPVTLPPPQLVPEGTPGGGGPPALEEDLTVININSSDEE
EEEEEEEEEDEEEDEEEDFEEDEEEEEYFEEEEEEEEEEFEEEFEEEEGELEDEEDEEEDEELEELEEVEFGPAGGEVEG
GPAPPSLPPVLPPAESPKGPPEPGLEPGLLLEVEEPGTEEAPGPEVAPMLAPEVPPPPHPQRLLRWKRWRRRRQLSPRKR
RGGSDMRPDLVCVCNCSELFVCPWKEQGDTAAMLADFIDCPPDDEKPPPASEPDS
>Equus caballus [A0A5F5PZG7]
MAAAVLSGPSAGSAAGAAGGTGGLSAVASGPRLRLLLLESVSGLLQPRAGSTVAPVHPHVRSAAHLPGLMCLLRLHGTVG
GAQNLSAVGALVGLSNARLGSIKTRFEGLCLLSLLVGESPTEMFQQHCVSWLRSIQQVLQSQDSPPTMELAVAVLRDLLR
YAAQLPTLFRDISMNHLPGLLTSLLGLRPECELSALEGMKACMTYFPRACGSLKGKLASFFLSRVDALSPQLQQLACECY
SRLPSLGAGFSQGLKHTESWEQELHSLLASLHSLLGALYEGAETAPMQYEGPGVEVLLSPSEDGDAHALLRLRQRFSGLA
RCLGLMLSSEFGAPVSVPVQEILDVICRTLSISAKNISLLGDGPLRLLLLPSIHLEALDLLSALILACGGRLLRFGALIS
RLLPQVLNAWSIGRDTLSPGQERPYSTMRTKVYAVLELWVQVCGASAGVLQGGASGEALLTHLLSDISPPADALKLRSPR
GSPDGGLQSGKPSAPKKLKLDVGEAMAPPSHRKGESNANSDVCAAALRGLSRTILMCGPLIKEETHRRLHDLVLPLVMGV
QQGEVLGSSPYTSSRCRRELYRLLLALLLAPSPRCPPPLACALQAFSLGQREDSLEVSSFCSEALVTCAALTHPRVPPLQ
SMGPTCPTSAPVPPPEAPSPFRAAPFHPPGPMPSAGPMPSAGPVPSAGPMPSVGPLPSVGPMPSVGPMPPARPGPPATAN
HLGLSVPGLVSVPPRLLPGPENHRAASNEDPVLAPSGTPPPAIPPDETFGGRMPRPAFVHYDKEEASDVEISLESDSDDS
VVIVPEGLPSLPPPPPSGTTPPPVAPAGPPTASPPVPAKEEPEELPAAPGPLPPPPPPPVPGPVTLPPPQLVPEGTPGGG
GPPALEEDLTVININSSDEEEEEEEEEEEEEEEEEEEEEEDFEEEEEDEEEYFEEEEEEEEEEFEEEFEEEEGELEEEEEEE
DEEEEEELEEVEELEFGSAGGEVDEAGPPPPSLPPALPPAESPKVQPEPEPEPGLLLEVEEPGVEEERGAETAPTLAPEV
LPSQGEVEREEGSPPAGPPPQELVEEEPSAPPTLLEEGTEGGDDKVPPLPEPPAAEEMETETEVAALQEKVSGPGREGGS
GVWRKRDCSCLCLSPA
>Propithecus coquereli [A0A2K6G9H7]
MELAVAVLRDLLRYAAQLPALFRDISMNHLPGLLTSLLGLRPECEQSALEGMKACMTYFPRACGSLKGKLASFFLSRVDS
LIPQLQQLACECYSRLPSLGAGFSQGLKHTESWEQELHSLLTSLHSLLGTLYEGAETAPVQNEGPGVETLLSSSEDGDAH
VLLRLRQRFSGLARCLVLMLSSEFGAPVSVPVQEILDLICRTLSISGKNINLLGDGPLRLLLLPSIHLEALDLLCALILA
CGGRLLRFGVLISRLLPQVLNAWSIGRDSLSPGQERPYSTIRTKVYAILELWVKVCGASAGVLQGGASGEALLTHLLSDI
SPPADALKLRSPRGSPDGGLQTGKPSAPKKLKLDVGEAMAPPSHRKGDSNANSDVCAAALRGLSRTILMCGWHSNLGVLH

DLVLPLVMGVQQGEVLGSSPYTSSCCRRELYRLLLALLLAPSPRCPPPLACALQAFSLGQQEDSLEVSSFCSEALVTCAA
LTHPRVPPLQSMGPTCPAPAQVPPPEAPSPFRAPPFHPPGPMPSAGSMASAGPMPSAGPMPSTGSMPSAGPVPPAPTANH
LGLPVPGLVSVPPRLLPGPENHRAGSNEDPVLAPSGTPPPTIPPDETFGGRVPRPAFVHYDKEEASDVEISLESDSDDSV
VIVPEGLPPLPPPPPSGTTPPPIAPAGPPTASPPVPAKEEPEELPAAPGPLPPPPPPPPPVPGPVTLPPPQLVPEGTPGW
GGPPALEEDLTVININSSDEEEEEEEEEEEEEEEEEEEEEDFEEEEEDEEEYFEEEEEEEEEFEEEFEEEEEHGAETAPT
LAPEVLPSQGEVEREGGSPAAGPPPQELVEEEPSAPPTLLEEGTEGGGDKVPPPPETPAEEEMETEAEATALQEKEQDDT
AAMLADFIDCPPDDEKPPPTTEPDS
> Ovis aries [W5PE21]
MAAAVLSGPSAGSAAAVPGGAGGLSAVGSGPRLRLMLLESVSGLLQPRTGSAVAPVHPPARSAPHLPGLMCLLRLHGTVG
GAQNLSAVGALVGLSNARLGSIKTRFEGLCLLSLLVGESPTEMFQQHCVSWLRSIQQILQSQDPPPTMELAVTVLKDLLR
YAAQLPAVFRDISMNHLPGLLTSLLGLRPECELSALEGMKACMTHFPRACGSLKGKLASFFLSRVDALSPQLQQLACECY
SRLPSLGAGFSQGLKHTDSWEQELRSLLASLHSLLGGLYEGAEAAPMQYESPGAETLLSPSEDVDAHTLLRLRQRFSGLA
RCLGLMLSSEFGAPVSVPVQDILDLICRTLSVSAKNVSLLGDGPLRLLLLPSLHLEALDLLSALILACGARLLRFGALIS
RLLPQVLSAWSIGRENLGPGQERPYSTVRTKVYAVLELWVQVCGASAGVLQGGASGEALLSHLLSDISPPADALRLRSPR
GSPDAGLQTGKPSAPKKLKLDVGEAIAPPSHRKGDSNANSDVCAAALRGLSRTILMCGPLIKEETHRRLHELVLPLVMGV
QQGEALCSSPYTSSHCRRELYRLLLAPSPRCPPPLACALRAFSMGQQEDSLEVSSFCSEALVTCAALTHPRVPPLQSVGP
TCPAPAPVPPPEAPAPFRAPAFHAPGPLPSAGPMPSAGPMPPAGPXXXXXXXXXXXXXXXXXXXXXXXXXXXXXXXXXXP
SGSPPPTVPPDETFGGRVPRPAFVHYDKEEASDVEISLESDSDDSVVIVPEGLPPPPPPPSGTTPPPVAPAGPPAASPPV
PAKDEPEELPAAPGPLPPPPPPPPVPGPVTLPPPQLVPEGTPGGGGPPALEEDLTVININSSDEEEEEEEEEEEEEDEEEDE
EEDFEEDEEEEEYFEEEEEEEEEEFEEEFEEEEGELEDEEDEEEDEELEELEEVEFGPAGGEVEGGPAPPSLPPVLPPAES
PKGPPEPGLEPGLLLEVEEPGTEEAPGPEVAPMLAPEVPPPPHPQRLLRWKRWRRRRQLSPRKRSRVTLLPCWPTSLTAP
RTTRSRPLPQSLTPSPPHFPLFFSNKFTSFDSNGCCCLLPPGCSQAEAQVSAGLGLYPQCFPGSL
>Phyllostomus discolor [A0A833ZLB1]
MELAVAVLRDLLRYAAQLPTLFRDISMNHLPGLLTSLLGLRPECELSALEGMKACMTYFPRACGSLKGKLASFFLSRVDA
LSPQLQQLACECYSRLPSLGAGFSQGLKHTESWEQELHSLLASLHSLLGALYEGAEPAPMQYEGPGVEMLFSPSEDGDAH
NLLRLRQRFSGLACCLGLLLRSEFGAPVSIPVQEILDVICRTLSISAKNISLLGDGPLRLLLLPSIHLEALDLLSALILA
CGGRLLRFGSLISRLLPQVLNAWSIGRDTLSLGQEKPYSAMRTKVYAVLELWVQVCGASAGVLQGGASGEALLTHLLSDI
SPPADALKLRSPRGSPDGGLQTGKPSAPKKLKLDMGEAVAPPSHRKGDSNANSDVCAAALRGLSRTILMCGPLIKEETHR
RLHDLVLPLVMSVQQGEVLGSSPYTSSYCRRELYRLLLALLLAPSPRYPPPLACALQAFSLGQREDNLEVSSFCSEALVT
CAALTHPRVPPLQSMGPACPAPAPVPPPEAPSPFRAPPFHPPGPMPSAGTMPSAGPVPSVGPMPPVGPMPPARPGPPATA
NHLGLSVPGLASVPPRLLPGPENHRAGSNEDTILAPSGTPPPTIPPDETFGGRVPRPAFVHYDKEEASDVEISLESDSDD
SVVIVPEGLPPLPLPPPAGTTPPPVAPAGPPTASPPLPAKEEPEELPAAPGPLPPPPPPLPGPVTLQPPQLVPEGTPGGG
APPALEEDLTVININSSDEEEEEEEEEEEEEEEEEEEEEDFEEEEEDEEEYFEEEEEEEEEFEEEFEEEEGELEEEEEEEDEE
EEEELEEEEMEREGGSPPAGPPPQELVEEEPCAPPTLLEEGTEVGGDEVPPPPETVAAEEMEPEPEPETPALQEKEQDDT
AAMLADFIDCPPDDEKPPPVTEPDS
>Rhinopithecus roxellana [A0A2K6P9Q7]
MELAVAVLRDLLRYAAQLPALFRDISMNHLPGLLTSLLGLRPECEQSALEGMKACMTYFPRACGSLKGKLASFFLSRVDA
LSPQLQQLACECYSRLPSLGAGFSQGLKHTESWEQELHSLLASLHTLLGALYEGAETAPVQNEGPGVEMLLSSEDGDAHV
LLRLRQRFSGLARCLGLMLSSEFGAPVSVPVQEILDFICRTLSVSSKNISLHGDGPLRLLLLPSIHLEALDLLSALILAC
GSRLLRFGILISRLLPQVLNSWSIGRDSLSPGQERPYSTVRTKVYALLELWVQVCGASAGVLQGGASGEALLTHLLSDIS
PPADALKLRSPRGSPDGSLQTGKPSAPKKLKLDVGEAMAPPSHRKGDSNANSDVCAAALRGLSRTILMCGPLIKEETHRR
LHDLVLPLVMGVQQGEVLGSSPYTSSRCRRELYCLLLALLLAPSPRCPPPLACALQAFSLGQREDSLEVSSFCSEALVTC
AALTHPRVPPLQPMGPTCPTPAPVPPPEAPSPFRAPPFHPPGPMPSVGPMPSAGSMPSAGPMPSAGPVPSARPGPPTTAN
HLGLSVSGLVSVPPRLLPGPENHRAGSNEDPILAPSGTPPPAIPPDETFGGRVPRPAFVHYDKEEASDVEISLESDSDDS
VVIVPEGLPPLKKSHPAGVSGWGTPVPAKEEPEELPAAPGPLPPPQLVPEGTPGGGGPPALEEDLTVININSNDEEEEEE
EEEEEEEEEEEEEEEEEEHGADTAPTLAPEVLPSQGEVEREEGSPLAGPPPQELVEEEPSAPPTLLEEGTEDGGDRVQPP
PETPAEEEMETETEEAEALQEKEQDDTAAMLADFIDCPPDDEKPPPPTEPDS
>Rhinolophus ferrumequinum [A0A7J7TG01]
MELAVAVLRDLLRYAAQLPTLFRDISMNHLPGLLTSLLGLRPECELSALEGMKACMTYFPRACGSLKGKLASFFLSRVDA
LSPQLQQLACECYSRVPSLGAGFSQGLKHTESWEQELHSLLASLHSLLGALYEGAEPAPMQYEGPGVEMLLSPSEDGDAH
VLLRLWQRFSGLARCLGLMLRSEFGAPVSVPVQGILDIVCRTLSISAKNISLLGDGPLRLLLLPSIHLEALDLLSALILA
CRGRLLRFGALISRLLPQVLNAWSIGRDTLSPGQEKPYSAMRTKVYAILELWVQVCGTSAGVLQGGASGEALLTHLLSDI
SPSADALKLRSPRGSPDGSLQIGKPSAPKKLKLDVGEAVAPPSHRKGDSNANSDVCAAALRGLSQTILMCGPLIKEETHR
RLHDLVLPLVMSVQQGEVLGSSPYTSSRCRRELYRLLLALLLAPSPRCPPPLTCALQAFSLGQREDSIEVSSFCSEALVT
CAALIHPRVPPLQSMGPTCPTPAPVPPPEAPSPFRAPPFHPSGPMSSAGTMPSASPVPSVGPMPSVGPMPSAGPMPSAGS
MPSAGPMPPARPGPPATTNHLGLSVPGLVSVPPRLLPGPENHQAGSNEDPILAPSGTPPPTIPPDETFGGRVPRPAFVHY
DKEEASDVEISLESDSDDSVVIVPEGLPPLPPPPPSGTSPPPVAPAGPPIASPPLPAKEEPEELPATPGPLPPPPPPPVP
GPVTLPPPQLVPEGTPGGGGPPALEEDMTVININSSDEEEEEEEEEEEEEEEEEEEDFEEEEDEEEYFEEEEEEEEEEFEEE
FEEEEGELEEEEEEEEDEEEEEEELEEGELEKEAGSPPAGPPPQELMEEDPCAPPTLLEEETEVGGDKLLPPPEASAAEEME
TEGDATALQEKEQDDTAAMLADFVDCPPDDEKPPPATEPDS
>Rousettus aegyptiacus [A0A7J8GDZ9]
MELAVAVLRDLLRYAAQLPTLFRDISMNHLPGLLTSLLGLRPECELSALEGMKACMTYFPRACGSLKGKLASFFLSRVDA
LSPQLQQLACECYSQVPSLGAGFSQGLKHTESWEQELHSLLASLHSLLGALYEGAEPAPMQYEGPGVEMLLSPSEDGDAH
ILLRLWQRFSGLARCLGLMLSSEFGAPVSVPVQEILDVICRTLSISAKNISLLGDGPLRLLLLPSIHLEALDLLSALILA
CRGRLLRFGALISRLLPQVLNAWSIGRDSLSPGQEKPYSAMRTKVYAILELWVQVCGASAGVLQGGASGEALLTHLLSDI
SPSADTLKLRSPRGSPDGGLQMGKPSAPKKLKLDVGEAMAPPSHRKGDSNANSDVCAAALRGLSQTILMCGPLIKEETHR
RLHDLVLPLVMGVQQGEILGSSPYTSSCCRRELYHLLLALLLAPSPRCPPPLACALQAFSLGQREDSLEVSSFCSEALVT
CAALTHPRVPPLQSMGPTCPTPAPVPPPEAPSPFRAPPFHPPGPVPSAGPMPSAGPVPSVGSMPSVGSMPSAGPMPSAGP
MPPTRPGPPATANHLGLSVPGLVSLPPRLLPGPENHRSGSNEDPILAPSGTPPPTIPPDETFGGRVPRPAFVHYDKEEAS
DVEISLESDSDDSVVIVPEGLPPLPPPPASGTTPPPVTPAGPPTASPPLPTQEEPEELPVALGPLPPPPPPSVPAPVTLA
PPQLVPEGTPGGGGPPALEEDLTVININSSDEEEEEEEEEEDEEEEEEEEDFEEEEEDEEEYFEEEEEEEEEFEEEYEEEEG
ELEEDEEEDEEEEEEELEEGELEREAGSPPAGPPPQELVEEEPCAPPTLLEEGTEVGGDKVPPPPETSAAEEMQTEKEATA
LQEKEQDDTAAMLADFIDCPPDDEKPPPAAEPDS
> Vombatus ursinus [A0A4X2JSV9]
MAAAVLSGSPTGSGAGGPGGAGGLPAAGPGSRLRMLLLESVSGLLQPRAGPAAPPSPPTAPLVPGLVRLLRLHGMMGGAQ
NLSAVGALVGLSNARLGSIKTRFEGLCLLSLLVAESPTETFQQHSVSWLRSIQHVLQSQDPAPTMELAVAILRDLLRYSA
QLPELSRDISTNHLPGLLTSLLGLKPECELSALEGMKACMTYFPRACGSLRGKLASFFLSRVEALSPQLQQLACECYARL

PALGAGFSQGLKHTESWEQELHCLLASLHGLLGALYEGAETAPIQYEGPGVELLLPPPADNDVHGLLQLRQRFSGLARCL
GLMLSSEFGAPVSVPVQDILDIICRTLSVSGKNISWLGDGPLRLLLLPSIHLEALDLLSALILSCGSRLVRFGGLICRLL
PQVLNTWSAGRDPLPPGQERPYSAVRAKVYSVLELWVQTCGAGAGVLQGGAQSEALLTHLLSDIFPPADALKLRGPRVTQ
DGGLQAGKPSAPKKLKLDSGEAVVPPMHRKGDSNANSDVCTTALRGLSRTILMCGPLIKEETHRRLHELTLPMVMGTQQG
EIPGGSPYTSARCRRELYRLLLALLLAPAPQCPPPLTCALQAFSLGQREENLEVSSFCSEALVTCAALAHPRVPPLQSPG
LSSPPTAPPTQPEPPSPFRAPPFHPPGPRPPVSATANHLGLPVPGLVSVPPRLLPGPENHQAALSEDSAPAPAGTSPPSQ
PPDEAFGGRPPRPAFVHYEKEETSDVEISLESDSDDSVVIVPEGLLPLPPAPPQGGTPPPAPPVVATAASPPQPPKEEPE
ELPPTPGPLPPPPGPGALPAPQLVAEGTTGGGPPPLEEDLTVININSSDEEEEEEEEEEEEEEEEEEDFPEEEEEEEDEEE
EYFEEEEEEEEEFEEEFEEEEEGELEEEEEEEEEEEEEEELEEVEFEPAEGEVEDRGAPPPDSPPPPPPSEPPKEEPEPGL
LMEVEEPSAEDEKEGGGAPPLALEASPPQGEREEEGRTVVPEAPPAEELAGNEPPVFSGEGTDGRGSQEPPPTRSAEAPA
TETEMAPIGEVRGIWKGGGCWWVVEEEEAAS
> Ailuropoda melanoleuca [A0A7N5JYW5]
GLSNARLGSIKTRFEGLCLLSLLVGESPTEMFQQHCVSWLRSIQQVLQSQDPPPTMELAVAVLRDLLRYAAQLPTLFRDI
SMNHLPGLLTSLLGLRPECELSALEGMKACMTYFPRACGSLKGKLASFFLSRVDALSPQLQQLACECYSRLPSLGAGFSQ
GLKHTESWEQELHCLLASLHSLLGTLYEGAETAPVQYEGPGVEVLLTPSEDGDAHVLLRLRQRFCGLARCLALMLSSEFG
APVSVPVQEILDVICRTLSISAKNISLLGDGPLRLLLLPSIHLDALDLLSALILACGGRLLRFGALISRLLPQVLNAWNL
GRDTLSPGQERPYSTVRTKVYAVLELWVQVCGASAGVLQGGASGEALLSHLLSDISPPADALKLRSPRGSPDGGLQAGKP
SAPKKLKLDMGEAVAPPSHRKGDSNANSDVCAAALRGLSRTVLMCGPLIKEETHRRLHDLVLPLVMGVQQGEVLGSSPYT
SSHCRRELYRLLLALLLAPSPRCPPPLACALQAFSLGQREDSLEVSSFCSEALVTCAALTHPRVPPLQSMGPACPTPAPV
PPPEAPSPFRAPPFHTPGPMPSVGPMPSVGPMPSAGPMPSAGSMASAGPMPPTRPGPPATANHLGLSVPGLVSVPPRLLP
GPENHRAGSNEDPVLAPSGTPPPPIPPDETFGGRAPRPAFVHYDKEEASDVEISLESDSDDSVVIVPEGLPSLPPPPPSG
TTPPPVAPAGPPTASPPVAAKEEPEELPAALAGPGVRARGARCARGARCARNTQGCSIPCPPSCPSPSYALPSSRPQRVS
YDEESLMVSFPLCFRLTLFFPPFLCDPLFSFLNSTRVKSYYIFL
> Ailuropoda melanoleuca [G1MCP5]
GLSNARLGSIKTRFEGLCLLSLLVGESPTEMFQQHCVSWLRSIQQVLQSQDPPPTMELAVAVLRDLLRYAAQLPTLFRDI
SMNHLPGLLTSLLGLRPECELSALEGMKACMTYFPRACGSLKGKLASFFLSRVDALSPQLQQLACECYSRLPSLGAGFSQ
GLKHTESWEQELHCLLASLHSLLGTLYEGAETAPVQYEGPGVEVLLTPSEDGDAHVLLRLRQRFCGLARCLALMLSSEFG
APVSVPVQEILDVICRTLSISAKNISLLGDGPLRLLLLPSIHLDALDLLSALILACGGRLLRFGALISRLLPQVLNAWNL
GRDTLSPGQERPYSTVRTKVYAVLELWVQVCGASAGVLQGGASGEALLSHLLSDISPPADALKLRSPRGSPDGGLQAGKP
SAPKKLKLDMGEAVAPPSHRKGDSNANSDVCAAALRGLSRTVLMCGPLIKEETHRRLHDLVLPLVMGVQQGEVLGSSPYT
SSHCRRELYRLLLALLLAPSPRCPPPLACALQAFSLGQREDSLEVSSFCSEALVTCAALTHPRVPPLQSMGPACPTPAPV
PPPEAPSPFRAPPFHTPGPMPSVGPMPSVGPMPSAGPMPSAGSMASAGPMPPTRPGPPATANHLGLSVPGLVSVPPRLLP
GPENHRAGSNEDPVLAPSGTPPPPIPPDETFGGRAPRPAFVHYDKEEASDVEISLESDSDDSVVIVPEGLPSLPPPPPSG
TTPPPVAPAGPPTASPPVAAKEEPEELPAALAGPGVRARGARCARGARCASAWVRQRGQGSAQNSEIHPPTPVGLRFEGL
RGRQTADSTAHKVPSARRGARVGSGGAGACFQQVMRIVRH
> Ailuropoda melanoleuca [A0A7N5K3N3]
VHFVQRRVPFSDSTRGVFVGTRVMSAVRQVQGPRGSVAFQGVEGLLREGGGEPVSGPHSILGVTELRGSGGGVCSGPASA
LARWPASSGARLPLRVVTGYFLVRFEGLCLLSLLVGESPTEMFQQHCVSWLRSIQQVLQSQDPPPTMELAVAVLRDLLRY
AAQLPTLFRDISMNHLPGLLTSLLGLRPECELSALEGMKACMTYFPRACGSLKGKLASFFLSRVDALSPQLQQLACECYS
RLPSLGAGFSQGLKHTESWEQELHCLLASLHSLLGTLYEGAETAPVQYEGPGVEVLLTPSEDGDAHVLLRLRQRFCGLAR
CLALMLSSEFGAPVSVPVQEILDVICRTLSISAKNISLLGDGPLRLLLLPSIHLDALDLLSALILACGGRLLRFGALISR
LLPQVLNAWNLGRDTLSPGQERPYSTVRTKVYAVLELWVQVCGASAGVLQGGASGEALLSHLLSDISPPADALKLRSPRG
SPDGGLQAGKPSAPKKLKLDMGEAVAPPSHRKGDSNANSDVCAAALRGLSRTVLMCGPLIKEETHRRLHDLVLPLVMGVQ
QGEVLGSSPYTSSHCRRELYRLLLALLLAPSPRCPPPLACALQAFSLGQREDSLEVSSFCSEALVTCAALTHPRVPPLQS
MGPACPTPAPVPPPEAPSPFRAPPFHTPGPMPSVGPMPSVGPMPSAATANHLGLSVPGLVSVPPRLLPGPENHRAGSNED
PVLAPSGTPPPPIPPDETFGGRAPRPAFVHYDKEEASDVEISLESDSDDSVVIVPEGLPSLPPPPPSGTTPPPVAPAGPP
TASPPVAAKEEPEELPAALAGPGVRARGARCARGARCARAGGARCAHTRRRVRPSLQRPQRGCVTTEDKSLASSHGICSF
SRH
> Ailuropoda melanoleuca [A0A7N5K9X4]
VHFVQRRVPFSDSTRGVFVGTRVMSAVRQVQGPRGSVAFQGVEGLLREAPPAPAPGCPTWDPLRSLLPCCPHLTPSPHSI
LGVTELRGSGGGVCSGPASALARWPASSGARLPLRVVTGYFLVRFEGLCLLSLLVGESPTEMFQQHCVSWLRSIQQVLQS
QDPPPTMELAVAVLRDLLRYAAQLPTLFRDISMNHLPGLLTSLLGLRPECELSALEGMKACMTYFPRACGSLKGKLASFF
LSRVDALSPQLQQLACECYSRLPSLGAGFSQGLKHTESWEQELHCLLASLHSLLGTLYEGAETAPVQYEGPGVEVLLTPS
EDGDAHVLLRLRQRFCGLARCLALMLRCERLWVPVSPCFPFLPRPSLLPCFLQSLLGDGPLRLLLLPSIHLDALDLLSAL
ILACGGRLLRFGALISRLLPQVLNAWNLGRDTLSPGQERPYSTVRTKVYAVLELWVQVCGASAGVLQGGASGEALLSHLL
SDISPPADALKLRSPRGSPDGGLQAGKPSAPKKLKLDMGEAVAPPSHRKGDSNANSDVCAAALRGLSRTVLMCGPLIKEE
THRRLHDLVLPLVMGVQQGEVLGSSPYTSSHCRRELYRLLLALLLAPSPRCPPPLACALQAFSLGQREDSLEVSSFCSEA
LVTCAALTHPRVPPLQSMGPACPTPAPVPPPEAPSPFRAPPFHTPGPMPSVGPMPSVGPMPSAGPMPSAGSMASAGPMPP
TRPGPPATANHLGLSVPGLVSVPPRLLPGPENHRAGSNEDPVLAPSGTPPPPIPPDETFGGRAPRPAFVHYDKEEASDVE
ISLESDSDDSVVIVPEGLPSLPPPPPSGTTPPPVAPAGPPTASPPVAAKEEPEELPAALAGPGVRARGARCARGARCARA
GGARCAHTRRRVRPSLQRPQRGCVTTEDKSLASSHGICSFSRH
> Ailuropoda melanoleuca [A0A7N5JIV2]
MELAVAVLRDLLRYAAQLPTLFRDISMNHLPGLLTSLLGLRPECELSALEGMKACMTYFPRACGSLKGKLASFFLSRVDA
LSPQLQQLACECYSRLPSLGAGFSQGLKHTESWEQELHCLLASLHSLLGTLYEGAETAPVQYEGPGVEVLLTPSEDGDAH
VLLRLRQRFCGLARCLALMLSSEFGAPVSVPVQEILDVICRTLSISAKNISLLGDGPLRLLLLPSIHLDALDLLSALILA
CGGRLLRFGALISRLLPQVLNAWNLGRDTLSPGQERPYSTVRTKVYAVLELWVQVCGASAGVLQGGASGEALLSHLLSDI
SPPADALKLRSPRGSPDGGLQAGKPSAPKKLKLDMGEAVAPPSHRKGDSNANSDVCAAALRGLSRTVLMCGPLIKEETHR
RLHDLVLPLVMGVQQGEVLGSSPYTSSHCRRELYRLLLALLLAPSPRCPPPLACALQAFSLGQREDSLEVSSFCSEALVT
CAALTHPRVPPLQSMGPACPTPAPVPPPEAPSPFRAPPFHTPGPMPSVGPMPSVGPMPSAGPMPSAGSMASAGPMPPTRP
GPPATANHLGLSVPGLVSVPPRLLPGPENHRAGSNEDPVLAPSGTPPPPIPPDETFGGRAPRPAFVHYDKEEASDVEISL
ESDSDDSVVIVPEGLPSLPPPPPSGTTPPPVAPAGPPTASPPVAAKEEPEELPAALAGPGVRARGARCARGGSAGLGGGS
PPLAQRVGRPDSGAFSGCGKKAGRPVRRRKGTGSDKWMAGSRTGRGGGAPWEALGRPHPRGSVLCFAFKMFLI
> Ornithorhynchus anatinus [A0A6I8N2Q5]
MAAAVLSGAPAAAGNGTPAGGSPGSRLCQVLLDSVSGLLQPRPSDTPASPSGAGPPKAPLVPGLVRLLRLHGTVGGAQNQ
TAVGTLIGLSSSRLSSIKTRFEGLCLLSLLVGESPTEVFQQHCVSWLRSIQHILQSQDPVLTVELAVAVLKDLLCYSSQL
PELARDISMNHLPGLLTSLLGLKPECELSALEGMKACMTHYPRACGSLRGKLASFFLARVDTLNPPLQQLACECYARLPA
LGAGFSQGLKHTESWEQELHCLLASLHCLLGALFDGAEPAPLQYEGPGMALLPPPAETAEAHGLLRLWQQFSGLARCLGL

LLSSEFGAPVSIPVQDVLDLICRTLNIHSKNITFMGDGPLKLLLLPWLHLEALELLSTLILACGARLVRFGGLLCRLLPQ
VLNAWSPGRDCLIPGQERPYCAVRAKTYAVLELWVQVCGAGAGVLQGGAQADTLLSHLLSDIALPAEALKLRASRGVPDG
GLQVGKPSAPKKPKLDVGEVGAGAAPIHRKGDGNANSDVCTAALRALSCTILMCGPLVKEETHRRLHELVLPLLMGASEA
GGGGGNPYTSALCRRELFRLLLALLLAPAPRCPPPLHCALRAFSLGQRDPNIQVSSFCSEAIVTCAALVHPRAPPLPPPM
PPGCPAPTSTPPEPPSPFRAPPFHPAPPPGPRAPPPPSNHLGLALPGLVSAPPRLLPGPENHRDEQPPAAPPPPPASAPA
SPPPPAPEEAFGGRAPRPAFVHYEKEEASDVEISLESDSDDSVVIVPEGLLLLPPTHPASPVAPQPATAAPSPPPPPPPP
PPPPPPPPQDEPEELAPATPGPPPPPAPASLPGPPQLVAEGAPGGLEENLTIININSSDEEDEDEEEEEEEEDEEEEEDE
EEFPEEEEEEYLEEEEEEEEEEEEEEGEEFEEEEGELEEEEEEEEEEEEEEEELEEVDFGSDAGPPVPSASPPAKPSAPPS
PPKIQPEPGLLMEVEEQGAAEEDEEDEEDEDEEEGRGQPVAEGRPPPPSPPIPEGPEAPPEANGEKESGVPTSPPLLPPE
GPDPEPPAPTLPREDPEGVAATDEALPEDPAAREPAAPSAPKLEEPDPTAAMLADFIDCPPDEEKLPEPSS
> Phyllostomus discolor [A0A834DU20]
MAAAVLSGPSAGSAAGASGGTGGLSALGSGPRLRMLLLESVSGLLQPRTGSTVPPVHPPVRSAPHLPGLMRLLRLHGTVG
GAQNLSAVAALVGLSNARLGSIKTRFEGLCLLSLLVGESSTEMFQQHCVSWLRSIQQVLQSQDPPPTMELAVAVLRDLLR
YAAQLPTLFRDISMNHLPGLLTSLLGLRPECELSALEGMKACMTYFPRACGSLKGKLASFFLSRVDALSPQLQQLACECY
SRLPSLGAGFSQGLKHTESWEQELHSLLASLHSLLGALYEGAEPAPMQYEGPGVEMLFSPSEDGDAHNLLRLRQRFSGLA
CCLGLLLRSEFGAPVSIPVQEILDVICRTLSISAKNISLLGDGPLRLLLLPSIHLEALDLLSALILACGGRLLRFGSLIS
RLLPQVLNAWSIGRDTLSLGQEKPYSAMRTKVYAVLELWVQVCGASAGVLQGGASGEALLTHLLSDISPPADALKLRSPR
GSPDGGLQTGKPSAPKKLKLDMGEAVAPPSHRKGDSNANSDVCAAALRGLSRTILMCGPLIKEETHRRLHDLVLPLVMSV
QQGEVLGSSPYTSSYCRRELYRLLLALLLAPSPRYPPPLACALQAFSLGQRQLQQKKWSPNQSRRPLLSRRRSRMTQLPC
WLTSLIVLLMMRSHHLSQSLTPSPLLRPPPSIPNKVVYLDIDCCFCLCRNCATSQKPHCLRAFQGIQNIPQVSP
> Polar bear [A0A452UGB4]
GLSNARLGSIKTRFEGLCLLSLLVGESPTEMFQQHCVSWLRSIQQVLQSQDPPPTMEPEVRCELSALEGMKACMTYFPRA
CGSLKGKLASFFLSRVDALSPQLQQLACECYSRLPSLGAGFSQGLKHTESWEQELHSLLASLHPLLGALYEGAETAPVQY
EGPGVEVLLTPSEDGDAHVLLRLRQRFCGLARCLGLMLSSEFGAPVSVPVQEILDVICRTLSISAKNISLLGDGPLRLLL
LPSIHLDALDLLSALILACGGRLLRFGALISRLLPQVLNAWNFGRDTLSPGQERRGWVRGPEEDPACGLRSPRGSPDGGL
QAGKPSAPKKLKLDMGEAMAPPSHRKGDSNANSDVCAAALRGLSRTVLPLIKEETHRRLHDLVLPLVMGVQQGEVLGSSP
YTSSRCRRELYRLLLALLLAPSPRCPPPLACALQAFSHGQREDSLEVSSFCSEALVTCAALTHPRVPPLQCRGPACXXXX
XXXXXXXXXXXXXXXXXXXXXXXXXXXXXXXXXXXXXXXXXXXXXXXXXXXXXXXXXXXXXXXXXXXXXXXXXXXXXXXX
XXXXXXXXXXXXXXXXXXXXXXXXXXXXXXXXXXXXXXXXXXXXXXXXXXXXXXXXXXXXXXXXXXXXXXXXXXXXXXXX
XXXXXXXXXXXXXXXXXXXXXXXXXXLTVININSSDEEEEEEEEEEEEEEEEEEEEEDFEEEEEDEEEYFEEEEEEEEEF
EEEFEEEEGELEEEEDEDEEEEEELEELEEVEFGPAGGPVEEGGPPPASPAPALPPAQSPKVQPEPEGEPGLLLEVEEPGA
EEEAGAEAAPTLAPEVLPSQGEGPRDTGSPPAGPPPQELMEEEPLAPPTLLEEGTESGGDKVPPPAETAVAEDVETELET
ATAALQEKEQDDTAAMLADFIDCPPDDEKPPPAPEPES
> Terrapene carolina triunguis [A0A674JPZ1]
NLQALGGLLGVPNARRGAAKTFEGLCLLSLLVSESPTEPFQQHCLGWLRALQHLLQSQDPAPTMALGAAVLRDLLLYSCQ
LPELARDIATNHVPGLLTSLLALKPECQLSALEGIKACMTCYPRACGSLRGKLAAYFLSRVDADSPQLQQLACECYALLP
SLGAGFTQGLKHTECWEQELHGLLATLHGLLGNLYEGAETDPLPYEGPGVELLLPPAPPNGETSFVLNLCNRFSGLAKCL
QLMLSSEFVAPVTVPVQDVLDLVCRALNISIKNISWFGDGPLKMLLLPSVHLEILDVLAALILACGARLARWGSVLGRLF
PQVLGAWSSSSDSVPPGQEKPYSAVRTRLYQVLELWVQVGGAGAGVLQGPPHHSEALLAHLLSDIAPPTDSLKVGRPGSE
GKPSAPKKPKLSEGGEAPSLHRKQDPMANSDVCKAALQALSRAILLGGSLVKEETHRRLQELVVPLLLRLAQGDVPPGGP
YASPACRHQLYRLLLALLLAPAPACPPPLHCALRAFALGQRDPSLQVSSFCAEALVTCSALARPRVPSLQLPLPGPAPSA
GPAPSDLAASPFRQAPPFPAPPPARPPPANHLGLAPPRLPPSAGPGLALAEEPGEAGDPRLLAPPSPGGVEEGGAGLGGK
PRRPVYVHYEKEEESDVEISLESDSDDSVVIVPKGLLPKPPPPPPPPPPAPPSPPPTQPPEEPPAAPLPPAAGEVLPGLGE
ENPAVININSSEEEEEEEFPEEEEDEEEEEEEEEEEEEYFDEEDEDFDEEEEEEFEEEEERGEEEEEEEYEEEEGLSEEEEE
EEEGALSEEERAPPGPQEPPAAPGEPDDLIMEVEESQAPEPPPPEEVEEEEVVVMKASPPPAPQPSREEEEEEEEEELPSA
PPTPPQQASPLQAPPPGQGEMGAEPPLLEAPAPVLEEEQPPKEEGAPPAGEQGDGAGEGAQPSPKREQPARKAEVRPGG
> Pelodiscus sinensis [K7F5J9]
QNLPALGGLIGVTNARLGTIKTRFEGLCLLSLLVSETPTEAFQLHCLSWLRSLQHLLQSQDPASTMALGVAVLRDLLHYS
CQLPELARDIGTNHIPGLLTSLLALKPECQVSALEGIKACMTFYPRACGSLRGKLAAYFLSHVDSDSPQLQQLACECYAL
LPSLGAGFTQGLKHRECWEQELHALLATLHSLLGNLYEAAETEPLPYEGPGLELLLPPAPPDGETSFVLNLCNRFSGLAK
CLQLMLSCEFVAPVTVPVQDVLDLVCRALNISVKNMSWFGDGPLKMLLLPSVHLEVLDVLAALILACGARLARWGSVLGR
LFPQVLGTWSGCREAVPLGQEKPYSAVRTRLYQVLELWVQVGGAGAGVLQGPSHRSEALLAHLLGDIAPPMDSIKLKVGR
PGSEGKPSAPKKPKLSEGGEAPSLHRKQDSMANSDVCRAALQVWSLGGTPTIMTAEPLVKEETHKKLQELVVPLLLRLAQ
GDVSPVGPYTSPACRHQLYRLLLALLLAPAPACPPPLQCALRAFALGQRDPSLQVRPRWNPTCCSCLLSPPPVPTLLSRC
STSSSTLPAVPLTWLQFLPAITPSPSSSAPAPWLQHSPLFCLPSLTPTPPSFSPLQVSSFCAEALVTCSALARPRVPSLQ
LPLQGPAPSAGPAPSEVAASPFHQVPPFPAPIPARPPPATNPPLPLPLPSTEAAPSLGEENPAVININSSEEEEEEEEEFP
EEEEEEEEEEEEEYFDEEDEDFEEEEEEEFEEEEELEEEEEYEEEEEEEGLSEDEEEEEGALSEEEQPPPAAQELPEAPR
EPDDLIMEVEESQAAELPPPEEEEEEEEEEEVMVMKASSPPASQLQQEEGKELPLAPPTPPPRASPLPAPPPCQEEMGEEP
PLLDDPPPVLEAEQLSREEGMSPGMEQAEVDETATMLADFIDCPPDDEKGPPEPSP
> Terrapene carolina triunguis [A0A674JKX5]
NLQALGGLLGVPNARRGAAKTFEGLCLLSLLVSESPTEPFQQHCLGWLRALQHLLQSQDPAPTMALGAAVLRDLLLYSCQ
LPELARDIATNHVPGLLTSLLALKPECQLSALEGIKACMTCYPRACGSLRGKLAAYFLSRVDADSPQLQQLACECYALLP
SLGAGFTQGLKHTECWEQELHGLLATLHGLLGNLYEGAETDPLPYEGPGVELLLPPAPPNGETSFVLNLCNRFSGLAKCL
QLMLSSEFVAPVTVPVQDVLDLVCRALNISIKNISWFGDGPLKMLLLPSVHLEILDVLAALILACGARLARWGSVLGRLF
PQVLGAWSSSSDSVPPGQEKPYSAVRTRLYQVLELWVQVGGAGAGVLQGPPHHSEALLAHLLSDIAPPTDSVKLKVGRPG
SEGKPSAPKKPKLSEGGEAPSLHRKQDPMANSDVCKAALQGNGAPGKGEPGRLQELVVPLLLRLAQGDVPPGGPYASPAC
RHQLYRLLLALLLAPAPACPPPLHCALRAFALGQRDPSLQVSSFCAEALVTCSALARPRVPSLQLPLPGPAPSAGPAPSD
LAASPFRQAPPFPAPPPARPPPANHLGLAPPRLPPSAGPGLALAEEPGEAGDPRLLAPPSPGGVEEGGAGLGGKPRRPVY
VHYEKEEESDVEISLESDSDDSVVIVPKGLLPKPPPPPPPPPPAPPSPPPTQPPEEPPAAPLPPAAGEVLPGLGEENPAVI
NINSSEEEEEEEFPEEEEDEEEEEEEEEEEEEYFDEEDEDFDEEEEEEFEEEEERGEEEEEEEYEEEEGLSEEEEEEEEGAL
SEEERAPPGPQEPPAAPGEPDDLIMEVEESQAPEPPPPEEVEEEEVVVMKASPPPAPQPSREEEEEEEEEELPSAPPTPPQ
QASPLQAPPPGQGEMGAEPPLLEAPAPVLEEEQPPKEEGAPPAGEQGDGAGEGAQPSPKREQPARKAEVRPGG
> Podarcis muralis [A0A670KFD4]
TLGGLVSLSNTRLGSIKTRFEGLCLLSLLVTESSTEAFSQNCLGWLRSLQHLIQSQDPPPTMELAVLILRDLLEYSCQIP
ELARDIGTNHIPGLLTSLLALKPECQISTLEGSKACMMFYPRACGSLRGKLAAYFLARVDAETPHLQQLACDCYALLPSL
GAGFTQGLKYTECWGQQAHCLLATLHSLLGTLYEGAETDPLHYEGPGVEILLPTPEDGEVNFILHLKHRFSGLAKCLCQM
LSNDFVAPVSVPVQDILDLVCRALNISTKNISWFGDGPLRMLLLPSVHLEALDLLSALILACGPRLVRFGATFCRLFPQV

LTTWSSGRDLSPPGQERPYSAVRTRLYEVLDLWVQTAGAACGILQGPRTQSEALLGHLISDISPPADTLKMRDSRPASDL
KPSAPKKPKLSDLGPLGSLHPKQDSQANSSTCLAALLQELVVPLLIRLGQAETPPGSPYASAPCRRQLHRLLLALVLAPP
PSWPPPFHCALRLFSQGRADPNLQVSSFCTEALAVCNALLHPRVPSLQLPLAAPPGLSPALASPFRPAPPAPFSAPRLLL
PPPASSAANPLGLPPPGLASPAQLPPRLAPEEPSLPTSPGAAEAALGAKLRRSVFVHYDKEEEEDVEISLESDSDDSVVI
VPKGQLGKGPGSAMAVAAAVAQVPTPPAPPPAPPAPPSPPPASEETPPEPPAPPLSLGVPLPPAPTAVLALPASPSPAAM
DPPFPALVEEDPTVININSSEDEEDDEDDYPEDEEYFEEDEEEVHWGGRGWADGQHVNSYFLL
> Pelodiscus sinensis [K7F5K0]
QNLPALGGLIGVTNARLGTIKTRFEGLCLLSLLVSETPTEAFQLHCLSWLRSLQHLLQSQDPASTMALGVAVLRDLLHYS
CQLPELARDIGTNHIPGLLTSLLALKPECQVSALEGIKACMTFYPRACGSLRGKLAAYFLSHVDSDSPQLQQLACECYAL
LPSLGAGFTQGLKHRECWEQELHALLATLHSLLGNLYEAAETEPLPYEGPGLELLLPPAPPDGETSFVLNLCNRFSGLAK
CLQLMLSCEFVAPVTVPVQDVLDLVCRALNISVKNMSWFGDGPLKMLLLPSVHLEVLDVLAALILACGARLARWGSVLGR
LFPQVLGTWSGCREAVPLGQEKPYSAVRTRLYQVLELWVQVGGAGAGVLQGPSHRSEALLAHLLGDIAPPMDSIKLKVGR
PGSEGKPSAPKKPKLSEGGEAPSLHRKQDSMANSDVCRAALQGTPPAEPLSCTIPTPPVLPREVRPHPSTASRTPWPIAM
CAELPCKKLQELVVPLLLRLAQGDVSPVGPYTSPACRHQLYRLLLALLLAPAPACPPPLQCALRAFALGQRDPSLQVASV
PPCHYPFSQLQCPCSLAPAFPIILSPFPDSNASLFLTPAGVVLLRRSSGDLQCPGAPPCTLSATTPARPCPLCWPVPPFP
APIPARPPPATNPPLPLPLPSTEAAPSLGEENPAVININSSEEEEEEEEFPEEEEEEEEEEEEEYFDEEDEDFEEEEEEE
FEEEEELEEEEEYEEEEEEEGLSEDEEEEEGALSEEEQPPPAAQELPEAPREPDDLIMEVEESQAAELPPPEEEEEEEEE
EVMVMKASSPPASQLQQEEGKELPLAPPTPPPRASPLPAPPPCQEEMGEEPPLLDDPPPVLEAEQLSREEGMSPGMEQAE
VDETATMLADFIDCPPDDEKGPPEPSP
> Aquila chrysaetos chrysaetos [A0A663F224]
MKTRFEGLCLLSLLVSESPTEPFQQHCLGWLRCLQHLLQSQDPAPTMALGVAVLHDLLLFSSQLPELARDIGTNHIPGLL
TSLLALKPECEVSTLEGIKSCMTFYPRACGSMRGKLAAYFLSRIDSESPQLQQLACECYALLPALGRGFSQGLRHTECWN
QEVQGLLATLHGLLGALFEGSETDPLPYEGPGVEMLLPAPQDGDTGFVLTLHNRFSGLARVLQLLLSKEFVAPVTVPVQD
VLDLICRALNITSKNINWFGDGPLKMLLLPSVHLDMLDVLSSLILACGARLVRWGSVLGRLFPQVLSAWSGAREAALPGQ
EKPFSAVRTRLYQVLELWVEVAGAASGVLQGPGAPGEVLLAHLLSDITPPAEGIKLKADPKPSAPKRPKLGDGGDAPALH
RKLEPAANSDVCRAALRALRRAILTGGPLIKEETHRRLQELVVPLLLRLPQTEVPPGPPAAAAGPPGTPYASPPCRAALY
QLLLALVLAPAPAGAPPPALCPACLRPGPARPGPPGLLRLHRSSGGGECPGPALGAPPCTPPPSQGPPQSPLPPPSAPCR
PPPSQGRPGLPPPIRWDPPGCRRWRRSPPGRPPPGQPPAAPGGDEEGGPAGGRPRRPVFVHYDKEEPSDVEISLESDSDD
SVVIVPKRGAPPSPPAPKSGGPPPPPPSPPLAPPEPPEEPGPPPPPPPPPPPPLPPVVPPAAPPEPPGIPGEPDPAVININ
SSEEEEEEEEEEEEEEEEFGEEEFFEEEEEEEEEEEEEEFDEEEELGEEEYEEDEEEYEEDEGLTEEEEEEEEEEEEGGAEE
GQPLPVPEEPPPPPGDTGALVMEVDEGHPPPPAGGGGEEEEEEEEEDEEEEEEEGTKAPQEGGEEEGEPPPGIPEPPPPL
LEPPEVPPPTLDAAHTPPHPEPPGGAGSPPPAPPPAGGGAPAPCGQGGGAGRDGHHVGGFH
> Podarcis muralis [A0A670KIL1]
TLGGLVSLSNTRLGSIKTRFEGLCLLSLLVTESSTEAFSQNCLGWLRSLQHLIQSQDPPPTMELAVLILRDLLEYSCQIP
ELARDIGTNHIPGLLTSLLALKPECQISTLEGSKACMMFYPRACGSLRGKLAAYFLARVDAETPHLQQLACDCYALLPSL
GAGFTQGLKYTECWGQQAHCLLATLHSLLGTLYEGAETDPLHYEGPGVEILLPTPEDGEVNFILHLKHRFSGLAKCLCQM
LSNDFVAPVSVPVQDILDLVCRALNISTKNISWFGDGPLRMLLLPSVHLEALDLLSALILACGPRLVRFGATFCRLFPQV
LTTWSSGRDLSPPGQERPYSAVRTRLYEVLDLWVQTAGAACGILQGPRTQSEALLGHLISDISPPADTLKMRDSRPASDL
KPSAPKKPKLSDLGPLGSLHPKQDSQANSSTCLAALLQELVVPLLIRLGQAETPPGSPYASAPCRRQLHRLLLALVLAPP
PSWPPPFHCALRLFSQGRADPNLQVSLSFPQLPRLFLLHRGPGCLQRPPAPPGALPAAPSGRPPGGPQAPPPGPSELSPA
LASPFRPAPPAPFSAPRLLLPPPASSAANPLGLPPPGLASPAQLPPRLAPEEPSLPTSPGAAEAALGAKLRRSVFVHYDK
EEEEDVEISLESDSDDSVVIVPKGQLGKGPGSAMAVAAAVAQVPTPPAPPPAPPAPPSPPPASEETPPEPPAPPLSLGVP
LPPAPTAVLALPASPSPAAMDPPFPALVEEDPTVININSSEDEEDDEDDYPEDEEYFEEDEEEVHWGGRGWADGQHVNSY
FLL
> Podarcis muralis [A0A670KMP7]
TLGGLVSLSNTRLGSIKTRFEGLCLLSLLVTESSTEAFSQNCLGWLRSLQHLIQSQDPPPTMELAVLILRDLLEYSCQIP
ELARDIGTNHIPGLLTSLLALKPECQISTLEGSKACMMFYPRACGSLRGKLAAYFLARVDAETPHLQQLACDCYALLPSL
GAGFTQGLKYTECWGQQAHCLLATLHSLLGTLYEGAETDPLHYEGPGVEILLPTPEDGEVNFILHLKHRFSGLAKCLCQM
LSNDFVAPVSVPVQDILDLVCRALNISTKNISWFGDGPLRMLLLPSVHLEALDLLSALILACGPRLVRFGATFCRLFPQV
LTTWSSGRDLSPPGQERPYSAVRTRLYEVLDLWVQTAGAACGILQGPRTQSEALLGHLISDISPPADTLKMRDSRPASDL
KPSAPKKPKLSDLGPLGSLHPKQDSQANSSTCLAALLQELVVPLLIRLGQAETPPGSPYASAPCRRQLHRLLLALVLAPP
PSWPPPFHCALRLFSQGRADPNLQVSLSFPQLPRGVCPLPTSGISPPPRGPSLCTAGRLPLSLEAPPPGPSELSPALASP
FRPAPPAPFSAPRLLLPPPASSAANPLGLPPPGLASPAQLPPRLAPEEPSLPTSPGAAEAALGAKLRRSVFVHYDKEEEE
DVEISLESDSDDSVVIVPKGQLGKGPGSAMAVAAAVAQVPTPPAPPPAPPAPPSPPPASEETPPEPPAPPLSLGVPLPPA
PTAVLALPASPSPAAMDPPFPALVEEDPTVININSSEDEEDDEDDYPEDEEYFEEDEEEVHWGGRGWADGQHVNSYFLL
> Taeniopygia guttata [A0A674H6W4]
MALQVRGAVPAAANQGFGRGWGRVPADQWRCRFEGLCLLSLLVSESPGDAFQQHCLGWLRLLQHLLQSQDPPPTVALGVS
VLRELLRFSAQLPELARDIGTNHIPGILTSLLALRPECELSTLEGIKSCLSFYPGACGSMRGKLAAHFLSRIDSDSPRLQ
QLACECYALLPALGRGFSQGLRHTECWHQELQGVLATLHGLLGALFEGCETDPLPYEGPGVELLLPPPPDGDAGGVLTLH
SRFSGLCRVLRLLLSKDFVAPVTVPVQDILDLVCRALNVTTKNLVSGPLRGLLLPQVHLDVLDVLGALLLACGARLVRWG
SLLGRLFPQVLSSWSGPRDPPPGQERPFGAVRSRLYQVLELWVQVAGAASGVLQGPGTPGEVLLSHLISDISPPSEGAKL
RAEPKPSAPKRPKLGEGLEGPPLHRKGEPAANSDTCAAALAALRRIVLTGGPLIKEETHRRLQELVVPLALRLPQTVPLE
ISGGPVPVSGSPYASSRCRGALYGLLQALLLGAPPGAAPPLHCALRAFGQGQRDPDVSSQCRESLLLLSSALCRPWLPPA
PSPPPSGPSPHSPPPPFRPLPPPNPSDLPSPLPPPPPPPPFPGGGKAEPPPLPPPAAVSGGEGEGPGGGGAPGGGGGARPR
RPVFVHYEREEPSDVEISLESDSDDSVVIVPKKSPNFPPGLGGGVAAPPPPPPPPPPPPPPPPPPTPPAAPEPPQEGEGKG
EGEEKEGEGQSGGGEGGEVGEGQEGGVGEGPAVININSSEEEEEEEEEEEEFGEEEEEEEEEFFEEEEEEEFEEEEEEEEEE
FEEEEEEEFEEEEEGEGLSEEEEEEEEEDEEEAAGEGPAEGGGGGALLLMEVDEAPPAPPPPPKPPPSPSPTPRGMRRKRR
RGRKQRRPSPNPSPPLPHPARPQSPSPTPRPPLPPPPSRPPPTPPLPPPAGGLSPRPSPPAPPPPLQEEPPEPPPPGTEQE
ERDETTTMLADFIDCPPDDEKTPETPL
> Paramormyrops kingsleyae [A0A3B3R369]
MATAVWLHVSTNMRLTEGLVSVLKEERPEYLPALLANYREHGVVSSQNSTAVGGLIGLSNGRLGSSKTRFEGLCLLAVLV
KDSSSDVFQQHCLSWLRSLQQVIQSQAPLPSVQLAVAILQDLLQYSSQLPELAREVGLNSVLGILTSLLSLKLECHLVAM
EGMKACMTFYPRACGSLRDKLGAYFLSKMDSDNPRMQEVACECYGRLPCLGGVLERGGGSRRAEAWANQMHCLLASAHGL
LGQLYLGAESEGALQYEGPGVELPLPSLDETDPLLQIQLRHRYRAVSLAIRHTLSADPVSPVCVPVQSLINLVCRALAVS
CKNINASGDGSLKLLVLPSIHSDTLELLSALITVAGGRLVQYCGVLTRLLSQTLSAWSPQPESSPGQQRAFSAVRVCLYR
MLELWVQVAGASAGVFQGSPTQTELLLMHLLGDITPGAESVKLRTGLSAATELAGHAGKTPPRRGKGMGDGSGPLLQRKG
DALANQDTCVSALRALRQIILTNGTLLKEDIHKRLQDLVLPLCVRLQQQCGSDVGGASGQYGSAPPRRELYRLLLALVL

APSPRWPPPLSCAVSIFSHGQRDHSLSVSSFCAEALTICNSLLHPRVPSIALPLPPLAVKPTPAAPTLASSQNLSLPTLL
GGPAPAPPFPPRHPLGMGPTGLLTPLENHLPPPPPVLPAQTGPTPAQAELLMSPPGELPPLGPPEGRRPVFVRYDKEEPE
DVEISLESDSDDSVVIVPPGMLILEGQDPASTQPHQPPPGAPVSGLAVGTGSDTGAVNSPLPNELPTTMALPSNSSAVTT
FPGQSQTPLVSLVPPLNSAAQLTTPSVGLGDALPGNQLQQMLLQSSPAGQASQLGLMQLQTQMAQSSRQLQQTQQQAQQP
IASEEDLTVININSSDEDEDEEEEMDEDDIGEEEDEEEEEPLDDDDEEEEEEEEVSDFPDYYDQEEFADYEEEEEDMIEEE
EGIIEGEEEDVDELPQLEGESRSVLLGEEEGEALMGPVSESPMGMFSGEDGDERDEPSGELEGQTAVYRKEGVQDEVKQT
EEGDVGIIEEKLHKEKLEEEAKEGSASLEFQTGRQPPMTARETSQEAELTQEAEAESRQEVSLQEQGSTEEGEAPAVSET
AVLLEEQATESAQEARETEDASGEEAVVELPLVEHGESKEEQTCPEEEAIAARQSKMSGLVESEEVGQEESDNNSDDSRG
VKRKREKGEIGEGEQSVEKKKVSVYAL
> Paramormyrops kingsleyae [A0A3B3R2G0]
MATAVWLHVSTNMRLTEGLVSVLKEERPEYLPALLANYREHGVVSSQNSTAVGGLIGLSNGRLGSSKTRFEGLCLLAVLV
KDSSSDVFQQHCLSWLRSLQQVIQSQAPLPSVQLAVAILQDLLQYSSQLPELAREVGLNSVLGILTSLLSLKLECHLVAM
EGMKACMTFYPRACGSLRDKLGAYFLSKMDSDNPRMQEVACECYGRLPCLGGVLERGGGSRRAEAWANQMHCLLASAHGL
LGQLYLGAESEGALQYEGPGVELPLPSLDETDPLLQIQLRHRYRAVSLAIRHTLSADPVSPVCVPVQSLINLVCRALAVS
CKNINASGDGSLKLLVLPSIHSDTLELLSALITVAGGRLVQYCGVLTRLLSQTLSAWSPQPESSPGQQRAFSAVRVCLYR
MLELWVQVAGASAGVFQGSPTQTELLLMHLLGDITPGAESVKLRTGLSAATELAGHAGKTPPRRGKGMGDGSGPLLQRKG
DALANQDTCVSALRALRQIILTNGTLLKEDIHKRLQDLVLPLCVRLQQQQCGSDVGGASGQYGSAPPRRELYRLLLALVL
APSPRWPPPLSCAVSIFSHGQRDHSLSVSSFCAEALTICNSLLHPRVPSIALPLPPLAVKPTPAAPTLASSQNLSLPTLL
GGPAPAPPFPPRHPLGMGPTGLLTPLENHLPPPPPVLPAQTGPTPAQAELLMSPPGELPPLGPPEGRRPVFVRYDKEEPE
DVEISLESDSDDSVVIVPPGMLILEGQDPASTQPHQPPPGAPVSGLAVGTGSDTGAVNSPLPNELPTTMALPSNSSAVTT
FPGQSQTPLVSLVPPLNSAAQLTTPSVGLGDALPGNQLQQMLLQSSPAGQASQLGLMQLQTQMAQSSRQLQQTQQQAQQP
IASEEDLTVININSSDEDEDEEEEMDEDDIGEEEDEEEEEPLDDDDEEEEEEEEVSDFPDYYDQEEFADYEEEEEDMIEEE
EGIIEGEEEDVDELPQLEGESRSVLLGEEEGEALMGPVSESPMGMFSGEDGDERDEPSGELEGQTAVYRKEGVQDEVKQT
EEGDVGIIEEKLHKEKLEEEAKEGSASLEFQTGRQPPMTARETSQEAELTQEAEAESRQEVSLQEQGSTEEGEAPAVSET
AVLLEEQATESAQEARETEDASGEEAVVELPLVEHGESKEEQTCPEEEAIAARQSKMSGLVESEEVGQEESDNNSDDSRG
VKRKREKGEIGEGEQSVEKKKLDEEAMASMLADFVDCPPDEEENVPSPVQS
> Yellowtail amberjack [A0A3B4XS63]
MATSAWLHGPSAMRLTEGLVSVLKEHRPEYLPALLANYREHGVFPTQSASAVGGLVGFSNAKLGSSKTRFEGLCLLSMLV
KDSSSDLFEQHCLSWLRSLQQVIQSQAPVQTIQLAVNILKDLLQYSSQLAELAREVGLNSILGILTSLLGLKTECELSAM
EGMMACMTYYPRACGSLRDKLGAYFLSKMDSTNKKTQEMACQCYGRLPCLGGLLDRGVGAGRAEGWTNQIHCLLASANGL
LTQIYQGSETDGAVQYEGPGVELAFPHLDQSDPLLLLQLQHRYTAVCLALKHTLRVDPASAVRLPVRPILNLVCRALAVS
SKSINLTGDGSVRLLVLPIIHTNTLDILSALITAVRSSMVQYTVVLQRLFSQTLSAWTPVHEASLGQQRAYSSVRVSVYR
TLELWVQVAGASASILQGSPGHSELLFNHLLGDITPGAESIKLRAGLSADAVPGGKPGPRRTKPLVMADAVGPSLQRKGD
FLANQDTCLSALRALRQIILTGGTLLKDDIHKRLHDVVLPLCVRLQQQQSSSNAACESAGGVGGQYSSALTRRELYRLLL
ALVLVPSPCWPPPLTCAVSILSSGRADRNLKVSTFCAEALTICNSLLHPRTPSIALPLPPLTLKPTPTAPVLPSSQGPTP
GLTLPTLLGGTAPGPPFPSRHSMGLGPASLLGSLENHLSLVPGLPGQAHTPGDMILSPHTHHLPDQAGLGPPEGQRPVFI
RYDKEEAEDVEISLASDSDDSVVIVPPGMLNMENQQDDAAAANSQNLVSAAPGGTTVSLPGGECVTMVPTTAVPTTIDSV
SLSNNLATSSPLLTTSTTPINSFPPSSTSVVSLVPPLNSSTLTAPPGGLGDSMPGRPQLQQMLMQPSTAGQPGPMGLPLQ
MHQLQNQLSQQGRHLHQHQPPPASNEDSAVININSTDEEEEEEEEDMEDDEELEEEDEGMDEEDEEEEVSDFAEEEFYDG
EEYEDYDEEEGEELEEEEEEEEDGDIPPLEGAEDKAGEVGVEEGKVVQAAVDEEGLAGFSVEGDEEGGIEEIQPNRVLFPE
DRMKVQEVESIGVLEEARGGEGEEDESERMDDPTMPQILCVTGGAMEEREETDEGGAAGGGAGAGGGGGAVQEEASLWEQ
GASEAELRTSSEERTTNQSQQESRPEPTQEASVGENQPCSSQEEQQPSAAPEGDSAAADPDSSAALKPKEPAETSSENRD
RGEAEQQETEGGGGGESDGEEGKGVKRKREEVHREEEAGQSTEKKKMDDEAMASMLADFVACPPDDEDGASGSTRS
> Sparus aurata [A0A671XEJ6]
MATSAWLRGPSAMRLTEGLVSVLKEQRPEYIPALLTSYREHGVFPTQSASAVGGLVGFSNAKLSSSKTRFEGLCLLSMLV
KDSSSDLFQQHCLSWLRSLQQVIQSQAPVQTIQLAANVLKDVLQYSSQLPELAREVGLNSILGILTSLLGLKTECELAAM
EGMMACMTYYPRACGSLKDKLGAYFLSKMDSTNKKTQEMACQCYGRLPCLGGLLDRGVGTGRAEGWTNQIHCLLASANGQ
LAQIYQGSETDGTVQYEGPGVELAFPYLDQSDPLLLLQLQHRYTAVCLALKHTLRVDPASAVRLPVRPILNLVCRALAVS
SKSINLTGDGSVRLLVLPIIHTNTLEVLSSLITAVRSGMVQYAAVLQRLFSQTLSAWTPLPEASLGQQRAYSSVRVSVYR
TLELWLQVAGTSASILQGSPGHSELLFSHLLGDITPGAESVKVISVAQADVVPGGKPGPRRTKTLVMADAVGPSLQRKGD
LLANQDTCLSALRVLRQIILTSGTLMKDDIHKRLHDVVLPLCVRLQQQSSSNTTCESAGGVSGQYSSALSRRELYRFLLA
LVLVPSPCWPPPLTCAVSILSNGRNDRNLKVSTFCIEALTICNSLLHPRTPSITLPLPPLALKPTPTAPVLPSSQGPTSG
LTLPTLLGGPAPGPPFPTRHTLGLGPSSLLGSLENHLSLVPGLPGQTPTPGDMILSPHAHHQQDPAGLAPPEGQRPVFVR
YDREEADDVEISLASDSDDSVVIVPPGMLNMENQQDETTGANSQNMASTAQGGTTAALPGGESVTMVPTTATAATIDGVA
LPNDLATSSPLLTTSTTPINSFPSSGASVVSLVPPLNSSTLAAPPGGLGDSLPSRPQLQQMLLQPSAPGQPGPIGLPLQI
HQLQNQLSQQGRHLHQHQPPPPSNEDSGVININSTDDEEEEEEDMEDDEELEEEEEEGMEEEEEEEVSDFGDEEFYDGEE
YEDYDEEEGDELDEEEEEEDGDIPPLEGAEDKAGEDGTEEGKMLRAAAVEEGELPGFSVEGEAEGGIEEIQTNRALFAED
RMKVQEVESIGVLEEAREGEGEDDESERMDDPTMPQILCVTGGAMEERDEAEEGAVEAGRAEGGGALQEEANLWEQGANE
TEPKAASEESTSNKSLQVLSQL
> Myripristis murdjan [A0A667Y036]
MATSAWLHGPSSMRLTEGLVSVLKEQRPEYLPALLANYREHGVFPTQGAAAAGGLVGFSNAKLGSSKTKFEGLCLLSMLV
KDSSSELFQQHCLSWLRSVQQVIQSQAPVQTIHLAVSVLQDLLQYSAQLAELAREVGLNSVLGILTSLLGLKTECELAAM
EGMTACMIYYPKACGSLRDKLGAYFLSKMDSSNKKTQEMACYCYGRLPCLGGVLERGVGAGRAEGWANQIHCLLASANGL
LAQMYQGSESDGTVQYEGPGVELPFPPFDQSDPLLLLQLQHRYSAVCLALKHTLRVDPASAVRVPVRPVLNLVCRALAIS
SRSINVTGDGSLRLLVLPSVHTSTLDILSALITVVRSSMVQYSAVLQRLFSQTLSAWAPQPEASPGQQRAFSSVRVAVYR
TLELWVQVGGASSSILQGNHTHSELLFGHLLGDITPGAESVKLRAGPSSEMLVSQKPGPRRTKSLVIVDGGPSLQKKGDL
LANQDTCLSALKALRQIVMTSGTLLKDDIHKRLHDVVLPLCVRLQQQQSSSSAALDSVGGISGQYSSAPPRRALYRLLLA
LVLVPSPRCPPPVTCAVSIFSRGRTDRSLKVSSLCTEALTVCNSLLHPRNPSISLPLPPLTFKPTPTAPVLPPSQGPSSG
LTLPTLLGGPSPGPPFPTRHSLSLGPAALLGSLENHLSLVPTGLPGQAPTPGDMLLSPHGHPQPDLAGLGPPEGQRPVFV
RYDKEEAEDVEISLASDSDDSVVIVPPGMLSMENQQDEASITANSAAQNLASITPGATSVALPGGEGGATGVTTTTAPTT
IDGVSLANDLPSSSSSLLTTSTTTPINSFPPSSASLVSLVPPLNSTPLTAPPSALGESLPGGPQLQQMLMQPSTQGQPSP
MGLPLQMHQLQNQLNQQGRHLHQHQPQPASNEDSAVININSSDDEEEEEEEEMEDDDELEEEEGMEEEEEEEDGSDFAEDEF
YEGEEYEEYDEEEAEELEEEEEEEEEEDGDIPPLEGAEDRRGGLGIEEGKVLRGAVEEEEIGGFSVEGEAEGGIEEIQANR
ALFGEERMKVQEVESIGVLEEAREGEGEEDESERMDDPTMPQILCVTGGALDERQEPEEEAEGGGEGAPQEEGRSWEQGA
SEAQLTASSEEGSATQNQQEQRGEAAQEAGASDGQPPDQQEEQPAAKPDGDSAPAHPETSAAASEKESEEEEKDGEKIEQ
EGAEEQGTPGEGEGDAEDGKGVKRKREEDQKEEEAGQSTEKKKMDDEAMASMLADFIACPPDDEDGASGSNRS
> Latimeria chalumnae [H3AFK1]

METLLGILQGEERGGGVSALLGSLKEHRNLAAQSSSVMGGLVAISNSRLGSVKTRFEGLCLLALLVAESSTEHFQQHCLL
WLRNVQHVLQSQDPQPTVDLAVFVLRDLLKYSSQLPELSREISLNHIPGILTSLLGLKPECQVAALEGMRACMTFYPRPC
GSLRSKLAAFFLCRQEAENPELQETAACASCCKPPRPGGIAQWPNHPHQDALLHALLAASHRGWEALHSGRIAPQHAFPA
ANRPDQDALLHALLAARPEGATLGQDAPLHAFLVADHPDQDALLHAARCLCCNMSHKDANINCPAKNSLWFPLLGISHVA
QMDVSLHEIIAGCGGRLRNVSVTFPLCPKITPERLSSGNIFKKKQHVGSGGVYGGRRRKAVLLPEVMFLRSALRPLGEGG
LITHSVTFHLLRRINPALSKTPKKIRGFVNLVLMTAKRLLLKFWRRDISPSARERQILITDTAAHGQVVMKSRALSHVVL
QCGSLLPEETHRKLHELVLPLLMRLQQSAGPASTPYLRAECRRELYRLLLFLLLTPSAKRPPPLHCAIRTFSLGQKDNDI
EVAAFCAEALVICNSLIHPRVPSLQIPTAAAGSAMFKSPVLTPEPSSSFRQPQAQSFSLPLQPSRPVTNQLGLPLSGLVA
GQQLVTRLPPPLTTSMLLLPMVPCSVKGENHLSDQQMDIDPGLQGDLHSPCQDAFDDKGHKPVFVHYDKEEDSDVEISLE
SDSDDSVVIVPEGLPPKPPASPESKKAEEPEEAAKPAAPATTATAAAVAATAPVSMTAPTVAQPLSQAQTLLQNPVPESQ
GGLPPVAEEDLTVININSSDEEDEEEEDDFPEDDDYFDEDDEDDFDEEDYEAEMDEVEEELGEEEEEEELMEGEDDELEL
EEIDDVEEEEDLEEDYEAEEEMMSEEEEEELQQPQLKQDVEPQSEEPPQPQAKEEATSQQDKPIEEPDGEKQEVTSQAEE
PQEELVLKDQESPPPLLPAPPATVDASPPREEVRVCEEGKPGGDEEVKETEGTEKVEVEEEVKEEGRDLERIEEDETAAM
LADFVDCPPDEEEPPPASASS
> Sparus aurata [A0A671XEJ1]
MATSAWLRGPSAMRLTEGLVSVLKEQRPEYIPALLTSYREHGVFPTQSASAVGGLVGFSNAKLSSSKTRFEGLCLLSMLV
KDSSSDLFQQHCLSWLRSLQQVIQSQAPVQTIQLAANVLKDVLQYSSQLPELAREVGLNSILGILTSLLGLKTECELAAM
EGMMACMTYYPRACGSLKDKLGAYFLSKMDSTNKKTQEMACQCYGRLPCLGGLLDRGVGTGRAEGWTNQIHCLLASANGQ
LAQIYQGSETDGTVQYEGPGVELAFPYLDQSDPLLLLQLQHRYTAVCLALKHTLRVDPASAVRLPVRPILNLVCRALAVS
SKSINLTGDGSVRLLVLPIIHTNTLEVLSSLITAVRSGMVQYAAVLQRLFSQTLSAWTPLPEASLGQQRAYSSVRVSVYR
TLELWLQVAGTSASILQGSPGHSELLFSHLLGDITPGAESVKLRAGLSADVVPGGKPGPRRTKTLVMADAVGPSLQRKGD
LLANQDTCLSALRVLRQIILTSGTLMKDDIHKRLHDVVLPLCVRLQQQSSSNTTCESAGGVSGQYSSALSRRELYRFLLA
LVLVPSPCWPPPLTCAVSILSNGRNDRNLKVSTFCIEALTICNSLLHPRTPSITLPLPPLALKPTPTAPVLPSSQGPTSG
LTLPTLLGGPAPGPPFPTRHTLGLGPSSLLGSLENHLSLVPGLPGQTPTPGDMILSPHAHHQQDPAGLAPPEGQRPVFVR
YDREEADDVEISLASDSDDSVVIVPPGMLNMENQQDETTGANSQNMASTAQGGTTAALPGGESVTMVPTTATAATIDGVA
LPNDLATSSPLLTTSTTPINSFPSSGASVVSLVPPLNSSTLAAPPGGLGDSLPSRPQLQQMLLQPSAPGQPGPIGLPLQI
HQLQNQLSQQGRHLHQHQPPPPSNEDSGVININSTDDEEEEEEDMEDDEELEEEEEEGMEEEEEEEVSDFGDEEFYDGEE
YEDYDEEEGDELDEEEEEEDGDIPPLEGAEDKAGEDGTEEGKMLRAAAVEEGELPGFSVEGEAEGGIEEIQTNRALFAED
RMKVQEVESIGVLEEAREGEGEDDESERMDDPTMPQILCVTGGAMEERDEAEEGAVEAGRAEGGGALQEEANLWEQGANE
TEPKAASEESTSNKSLQESGGEPVQEAVTSDDQPSSQQEEQPAAVQEGDMAPADSETSRDGNTKEQEETDPETREKIKAE
QETEGGGGGGGRESDGEEGKGVKRKREEAYREEEAGQSTEKKKMDEDAMASMLADFVACPPDDEDGATGSNRS
> Lates calcarifer [A0A4W6DPF8]
MATSAWLHGPSAMRLTEGLVSVLKEHRPEYLPALLANYREHGVFPTQGASAAGGLVGFSNAKLGSSKTRFEGLCLLSILV
KDSSSDLFEQHCLSWLRSLQQVIQSQAPVQTIQLAVNILKDLLQYSSQLPELAREVGLNSILGILTSLLGLKTECELSAM
EGMIACMTYYPRACGSLRDKLGAYFLSKMDSTNKKTQEMACQCYGRLPCLGGLLDRGVGAGRAEGWTNQIHCLLASANCL
LAQIYQGSETDGTVQYEGPGVELAFPHLDQSDPLLLLQLQNRYTAVCLALKHTLRVDPASAVRLPVRPTLNMVCRALAVS
SKSINLTGDGSVRMLVLPIIHTNTLEVLSALITAVRCGMVQYTVVLQRLFSQTLSAWTPLHEDSVGQQRAYSSVRVAVYR
TLELWVKVAGASSGILQGSPGHSELLFSHLLGDITPGAESVKLRAGLSIDVVPGGKPGPRRTKPLVIADAGGPSHQRKGD
ILANQDTCLSALRAVSQSVGFCSMLIFSQRLHDVVLPLSVRLQQQQSSSNTACEPAGGGSGQYSSAVTRRELYRLLLALV
LVPSPCWPPPLTCAVSILSNGRTDRNLKVSTFCVEALTICNSLLHPRTPSITLPLPPLTLKPTPAVPVLPSSQGPTSGLT
LPTLLGGTAPAPPFPTRHSLSLAPTSLLGSLENHLSLVPGLPGQAPTTGDMILSPHTHHLPDQAGLGPPEGQRPVFVRYE
KEEAEDVEISLASDSDDSVVIVPPGMLNMENQQDDTAVAANSQNMVSSVPGGTTVTLPGGECVTMVPTTTAPTTIDNVSL
SNNLTTSSPLLTTSTTPINSFPPSSTSVVSLVPPLNSSTLTAPPGVLGDSMSGRPQLQQMLMQPSAPGQPGPMGLPLQMH
QLQNQLSQQGRHLHQHQPTPASNEDSAVININSTDEEEEEEEEDMEDEEELEEEEEGMDEEDEEEEVSDFAEEEFYDGEE
YEDYDEEEGEELEEDEEEEDGDIPPLERAEDKAGEVGIEEGKVLQAAVDEGGIAGFSVEGDEEGGIEEIQTNRVLFGEDR
LKVQEVESIGVLEEARAGEGQEDESERMDDPTMPQILCVTGGALEEREETEAEPVQEASVSDNQPSSHQEGQLAAAQEGD
SAAADPETSAGLNTKEQEETNAEKRDKVVAEQQETKGEGGGESDGEEGKGVKRKREEEEAGQNTEKKKLDDETMASMLAD
FVACPPDDEDGASGSNHS
> Cynoglossus semilaevis [A0A3P8V874]
MASTAWLHGSSVLRLTEGLVSVLKDQRPEHLPAVLASYREHGVLSTQSASTVGGLVGYSNAKLGSSKTRFEGLCLLSVLV
KDGSSDLFEQHCLSWLRSLQQVIQSHASGQTIKLAVSILKDLLQYSSQLPELSREVGLNSILGILTSLLGLKAECELWAM
DGMMACMTYYPRACGSLRDKLGAYFLTKMDCTNKNTQEMASQCYGRLPCLGGLLDRGVSTGRTEGWTNQVHCLVASANAL
LAEIYQGSETDGTVQYDGPGIELAFPHLDQMDPLTLLQLQHRYTSVCLALKHTLRSDPASPVRVPIRPVLNLVCRALAVS
CKSINLTGDGSVRLLILPNIHTSTLEVLAALITVVRSGMVQYTLVLQRLFSQTLASWTPSHEAGVGQQRAYSSVRVSVYR
TLELWVQVAGASASILQGSPVHSELLFSQLLRDIAPGVDTGKLRSRLSADEPLVEKLATRRNKPSVMGDSGGTSLPRKGD
HLANQDTCLTALRALRQVILTSGALLKDDMHKRLHEVVLPLCVHLQQQQQSGSSIVYDAAGHLGGQYRSSFARRELYRLL
MALVLVPSSCWPSPLTCAVSIFSKGRNDSNLKISTFCTEALTICNSLLHPRTPSTALPLPPLTLKSPPTAPVLTSTQGPG
PTSGLTLPTLLGATAPGPIFPVRHSLNLGPSSLLGSLENQLSLVPGLSCPAPTPGEVILSPPAHHLPDQAGLGPAECQRP
VFVRYEKEEAEDVEISLASDSDDSVVIVPPGMLNMENSQDAVSTAANSHSMLSVASGAPAVTLSAGECTPLTANSATPST
IDSATLQNNLSTSSSPLITSNNPVNSCPSSSTSLVSMVPPLNASTLTPTSGLGNSMSSRAQLQQMLMQPSAGAQPPSIAP
PLQMHQLQNQLTQPGRAFHQRPSPLPTSNEDTAIININSTDDEEEEDMEDEEDVEEDEEDEDEEVSDFAEEEEEEFYDGD
EYEDYDEEEAEELEEEGEDEDGDMPPKVEEEGERHVHPQEVDGDIPPLEGAEDNAEDAEGLQPPALLDDCVSRYSGEGYE
EGGIEEIQSNRVLFGDDRIKVQEVESIGVLEEARADEEDESERADDPSMPQILCVXXXXXXXGVDGRQHKRGRRQRRRRR
RGVAGSDGAVGARSQ
> Lates calcarifer [A0A4W6DPF0]
MATSAWLHGPSAMRLTEGLVSVLKEHRPEYLPALLANYREHGVFPTQGASAAGGLVGFSNAKLGSSKTRFEGLCLLSILV
KDSSSDLFEQHCLSWLRSLQQVIQSQAPVQTIQLAVNILKDLLQYSSQLPELAREVGLNSILGILTSLLGLKTECELSAM
EGMIACMTYYPRACGSLRDKLGAYFLSKMDSTNKKTQEMACQCYGRLPCLGGLLDRGVGAGRAEGWTNQIHCLLASANCL
LAQIYQGSETDGTVQYEGPGVELAFPHLDQSDPLLLLQLQNRYTAVCLALKHTLRVDPASAVRLPVRPTLNMVCRALAVS
SKSINLTGDGSVRMLVLPIIHTNTLEVLSALITAVRCGMVQYTVVLQRLFSQTLSAWTPLHEDSVGQQRAYSSVRVAVYR
TLELWVKVAGASSGILQGSPGHSELLFSHLLGDITPGAESVKLRAGLSIDVVPGGKPGPRRTKPLVIADAGGPSHQRKGD
ILANQDTCLSALRALRQIILTSGTLLKDDIHKRLHDVVLPLSVRLQQQQSSSNTACEPAGGGSGQYSSAVTRRELYRLLL
ALVLVPSPCWPPPLTCAVSILSNGRTDRNLKVSTFCVEALTICNSLLHPRTPSITLPLPPLTLKPTPAVPVLPSSQGPTS
GLTLPTLLGGTAPAPPFPTRHSLSLAPTSLLGSLENHLSLVPGLPGQAPTTGDMILSPHTHHLPDQAGLGPPEGQRPVFV
RYEKEEAEDVEISLASDSDDSVVIVPPGMLNMENQQDDTAVAANSQNMVSSVPGGTTVTLPGGECVTMVPTTTAPTTIDN
VSLSNNLTTSSPLLTTSTTPINSFPPSSTSVVSLVPPLNSSTLTAPPGVLGDSMSGRPQLQQMLMQPSAPGQPGPMGLPL
QMHQLQNQLSQQGRHLHQHQPTPASNEDSAVININSTDEEEEEEEEDMEDEEELEEEEEGMDEEDEEEEVSDFAEEEFYD

GEEYEDYDEEEGEELEEDEEEEDGDIPPLERAEDKAGEVGIEEGKVLQAAVDEGGIAGFSVEGDEEGGIEEIQTNRVLFG
EDRLKVQEVESIGVLEEARAGEGQEDESERMDDPTMPQILCVTGGALEEREETEQEGEGAGGGVVQEEANLWEQGSSELE
LTAPSEDGTTNQNQQDLRAEPVQEASVSDNQPSSHQEGQLAAAQEGDSAAADPETSAGLNTKEQEETNAEKRDKVVAEQQ
ETKGEGGGESDGEEGKGVKRKREEEEAGQNTEKKKVNICG
> Amphiprion percula [A0A3P8SUL3]
MATSTWLHGPSAMRLTEGLVSVLKEQRPEYLPALLANYREHGVFPTQGAGAVGGLVGFSNAKLGSSKTRFEGLCLLAMLV
KDSSSDLFQQHCLSWLRSLQQVIQSQAPVQTIQLAVNILKDLLHYSSQLPELTREVGLNSVLGILTTLLGLKTECELAAM
EGMTACMTYYPRACGSLRDKLGAYFLSKMDSSNRKTQEMACQCYGRLPCLGGLLDRGVGAGRAEGWTNQIHCLLASANAL
LAQIYQGAETDGAVQYQGPGVELSFPHLDQTDPLLLLQLQHRYTAVSLALKHTLRVDPASAVRLPVRPILNLVCRALAVS
SKSINLTGDGSVRLLVLPIIHTDTLEILSSLITVVRRGMVQYAAVLQKLFSQTLSAWTPLPEASVGQQRAYSSVRVSVYK
TLELWVQVAGASANILQGSPSHSELLFGHLLGDITPGAESIKLRAGLSADVVPGGKPGPRRTKQLVMADAVGPSLQRKGD
LLANQDTCLSALRGLTQIIQTSGTLLKDDIHKRLHDVVLPLCVRLQQQQCSTSTACESAWGISGQYSSATTRQELYRLLL
ALVLVPSPCWPPPLTCAVSILSNGRTDRNLKVSSFCAEALTICNSILHPRTPSVALPLPPLTLKPTHTAPVLPSSQGPAP
GLTLPTLLGGPTPGPPFPTRHSLGLGPTSLLGSLENHLSLVPGLPGQAPAPGDMILSPHTHHQPDSAGLGPPEGQRPVFV
RYDKEETEDVEISLESDSDDSVVIVPPGMLNMENQQEDTAANSQNILSAVPGSASVTLPGGESVTMVPTTAATTTVEGTS
LPNDLATSSPLLTTSTTPINSFPPSSASVVSLVPPLTSSTLTGPPGGLGDSMPGRPHLQQMLMQPSTPGQPSQMGLPLQM
HQLQNQLSQQGRHLHQHPPAPTSNEDSAVININSTDDEEEDEEDMEDDEELEEEEEGMDEEDEEEEVSDLAEDEFYDGEE
YEDYDEEEGEDLEEEEEEEEEGDIPPLEGGEDKGGEAGIAEGKVLRAAVDEGGMSGFNVEGDAEGGIEEIQTSRALFADD
RMKVQEVESIGVLEEAREGEGGEEDDSEKMNDPTMPQILCVTGGALDEREETEETEEEGGGTGAGLQEGASSWEQGAKEN
EQAAASEEQTANQSPQESAAEPAQEASVSDNQPSSSPQEEQVVAVEEEDTPADPEPSAVLNTKEQEETVDLKKDKMDTEQ
QEAGGREEGSDGEEGKGVKRKREEGQQEEEAEQSTEKKKLDDEAMASMLADFVACPPDDEDGATGSNCS
> Amphiprion ocellaris [A0A3Q1B505]
MATSTWLHGPSAMRLTEGLVSVLKEQRPEYLPALLANYREHGVFPTQGAGAVGGLVGFSNAKLGSSKTRFEGLCLLAMLV
KDSSSDLFQQHCLSWLRSLQQVIQSQAPVQTIQLAVNILKDLLHYSSQLPELTREVGLNSVLGILTTLLGLKTECELAAM
EGMTACMTYYPRACGSLRDKLGAYFLSKMDSSNRKTQEMACQCYGRLPCLGGLLDRGVGAGRAEGWTNQIHCLLASANAL
LAQIYQGAETDGAVQYQGPGVELSFPHLDQTDPLLLLQLQHRYTAVSLALKHTLRVDPASAVRVPVRPILNLVCRALAVS
SKSINLTGDGSVRLLVLPIIHTDTLEVLSSLITVVRRGMVQYAAVLQKLFSQTLSAWTPLPEASVGQQRAYSSVRVSVYK
TLELWVQVAGASANILQGSPSHSELLFGHLLGDITPGAESVKLRAGLSADVVPGGKPGPRRTKQLVMADAVGPSLQRKGD
LLANQDTCLSALRGLTQIIQTSGTLLKDDIHKRLHDVVLPLCVRLQQQQCSTSTACESAWGISGQYSSATTRQELYRLLL
ALVLVPSPCWPPPLTCAVSILSNGRTDRNLKVSSFCAEALTICNSILHPRTPSVALPLPPLTLKPTHTAPVLPSSQGPAP
GLTLPTLLGGPTPGPPFPTRHSLGLGPTSLLGSLENHLSLVPGLPGQTPAPGDMILSPHTHHQPDSAGLGPPEGQRPVFV
RYDKEETEDVEISLESDSDDSVVIVPPGMLNMENQQEDTAANSQNILSAVPGSASVTLPGGESVSMVPTTAATTTVEGTS
LPNDLATSSPLLTTSTTPINSFPPSSASVVSLVPPLTSSTLTGPPGGLGDSMPGRPQLQQMLMQPSTPGQPSQMGLPLQM
HQLQNQLSQQGRHLHQHPPAPTSNEDSAVININSTDDEEEDEEDMEDDEELEEEEEGMDEEDEEEEVSDLAEDEFYDGEE
YEDYDEEEGEDLEEEEEEEEEGDIPPLEGGEDKGGEAGIAEGKVLRAAVDEGGMSGFNVEGDAEGGIEEIQTSRALFADD
RMKVQEVESIGVLEEAREGEGGEEDDSEKMNDPTMPQILCVTGGALDEREETEEEGGGTGAGLQEGASSWEQGAKENEQA
AASEEQTANQSPQESAAEPAQEASVSDNQPSSSPQEEQVVAVEEDTPADPEPSAVLNTKEQEETVDLKKDKMDTEQQEAG
GREEGSDGEEGKGVKRKREEGQQEEEAEQSTEKKKLHDEAMASMLADFVACPPDDEDGATGSNCS
> Amphiprion ocellaris [A0A3Q1B3Z8]
MATSTWLHGPSAMRLTEGLVSVLKEQRPEYLPALLANYREHGVFPTQGAGAVGGLVGFSNAKLGSSKTRFEGLCLLAMLV
KDSSSDLFQQHCLSWLRSLQQVIQSQAPVQTIQLAVNILKDLLHYSSQLPELTREVGLNSVLGILTTLLGLKTECELAAM
EGMTACMTYYPRACGSLRDKLGAYFLSKMDSSNRKTQEMACQCYGRLPCLGGLLDRGVGAGRAEGWTNQIHCLLASANAL
LAQIYQGAETDGAVQYQGPGVELSFPHLDQTDPLLLLQLQHRYTAVSLALKHTLRVDPASAVRVPVRPILNLVCRALAVS
SKSINLTGDGSVRLLVLPIIHTDTLEVLSSLITVVRRGMVQYAAVLQKLFSQTLSAWTPLPEASVGQQRAYSSVRVSVYK
TLELWVQVAGASANILQGSPSHSELLFGHLLGDITPGAESVKVGSTCSASDVVPGGKPGPRRTKQLVMADAVGPSLQRKG
DLLANQDTCLSALRGLTQIIQTSGTLLKDDIHKRLHDVVLPLCVRLQQQQCSTSTACESAWGISGQYSSATTRQELYRLL
LALVLVPSPCWPPPLTCAVSILSNGRTDRNLKVSSFCAEALTICNSILHPRTPSVALPLPPLTLKPTHTAPVLPSSQGPA
PGLTLPTLLGGPTPGPPFPTRHSLGLGPTSLLGSLENHLSLVPGLPGQTPAPGDMILSPHTHHQPDSAGLGPPEGQRPVF
VRYDKEETEDVEISLESDSDDSVVIVPPGMLNMENQQEDTAANSQNILSAVPGSATATTTVEGTSLPNDLATSSPLLTTS
TTPINSFPPSSASVVSLVPPLTSSTLTGPPGGLGDSMPGRPQLQQMLMQPSTPGQPSQMGLPLQMHQLQNQLSQQGRHLH
QHPPAPTSNEDSAVININSTDDEEEDEEDMEDDEELEEEEEGMDEEDEEEEVSDLAEDEFYDGEEYEDYDEEEGEDLEEE
EEEEEEGDIPPLEGGEDKGGEAGIAEGKVLRAAVDEGGMSGFNVEGDAEGGIEEIQTSRALFADDRMKVQEVESIGVLEE
AREGEGGEEDDSEKMNDPTMPQILCVTGGALDEREETEEEGGGTGPSSSPQEEQVVAVEEDTPADPEPSAVLNTKEQEET
VDLKKDKMDTEQQEAGGREEGSDGEEGKGVKRKREEGQQEEEAEQSTEKKKVKI
> Amphiprion percula [A0A3P8SUN3]
MATSTWLHGPSAMRLTEGLVSVLKEQRPEYLPALLANYREHGVFPTQGAGAVGGLVGFSNAKLGSSKTRFEGLCLLAMLV
KDSSSDLFQQHCLSWLRSLQQVIQSQAPVQTIQLAVNILKDLLHYSSQLPELTREVGLNSVLGILTTLLGLKTECELAAM
EGMTACMTYYPRACGSLRDKLGAYFLSKMDSSNRKTQEMACQCYGRLPCLGGLLDRGVGAGRAEGWTNQIHCLLASANAL
LAQIYQGAETDGAVQYQGPGVELSFPHLDQTDPLLLLQLQHRYTAVSLALKHTLRVDPASAVRLPVRPILNLVCRALAVS
SKSINLTGDGSVRLLVLPIIHTDTLEILSSLITVVRRGMVQYAAVLQKLFSQTLSAWTPLPEASVGQQRAYSSVRVSVYK
TLELWVQVAGASANILQGSPSHSELLFGHLLGDITPGAESIKVGSTCSASDVVPGGKPGPRRTKQLVMADAVGPSLQRKG
DLLANQDTCLSALRGLTQIIQTSGTLLKDDIHKRLHDVVLPLCVRLQQQQCSTSTACESAWGISGQYSSATTRQELYRLL
LALVLVPSPCWPPPLTCAVSILSNGRTDRNLKVSSFCAEALTICNSILHPRTPSVALPLPPLTLKPTHTAPVLPSSQGPA
PGLTLPTLLGGPTPGPPFPTRHSLGLGPTSLLGSLENHLSLVPGLPGQAPAPGDMILSPHTHHQPDSAGLGPPEGQRPVF
VRYDKEETEDVEISLESDSDDSVVIVPPGMLNMENQQEDTAANSQNILSAVPGSATATTTVEGTSLPNDLATSSPLLTTS
TTPINSFPPSSASVVSLVPPLTSSTLTGPPGGLGDSMPGRPHLQQMLMQPSTPGQPSQMGLPLQMHQLQNQLSQQGRHLH
QHPPAPTSNEDSAVININSTDDEEEDEEDMEDDEELEEEEEGMDEEDEEEEVSDLAEDEFYDGEEYEDYDEEEGEDLEEE
EEEEEEGDIPPLEGGEDKGGEAGIAEGKVLRAAVDEGGMSGFNVEGDAEGGIEEIQTSRALFADDRMKVQEVESIGVLEE
AREGEGGEEDDSEKMNDPTMPQILCVTGGALDEREETEETEEEAAEPAQEASVSDNQPSSSPQEEQVVAVEEEDTPADPE
PSAVLNTKEQEETVDLKKDKMDTEQQEAGGREEGSDGEEGKGVKRKREEGQQEEEAEQSTEKKKLDDEAMASMLADFVAC
PPDDEDGATGSNCS
> Anabas testudineus [A0A3Q1H932]
MATSAWLHGPSAMRLTEGLVSVLKEHRTEYLPALLGSYREHGVFQTQGPSAIGGLVGFSNAKLGSSKTRFEGLCLLSILV
KDSSSDLFQQHCISWLRSLQQVIQSQAPVQTIQLAVNILKDLLQYSSQLAELAREVGLNFILGFLTSLLGLKAECELAAM
EGMMACMTYYPRACGSLRDKLGAYFLSKMDSTNKKIQEMGCQCYGYLPCLGGLLDRGVSAGRAEGWTNQIHCLLASANGL
LTHIYHGLETDGTVQYEGPGVELAFPHLDLSDPWLLLQLQHRYTAVCLSLKHTLRVDPTSAVRLPVRPILNLVCRALAVS
LKSINLTGDGSVRVLVLPIIHINTLEVLSALITVVHSGMVQYAAVLQRLFSQTLSAWTPPYEAGLGQQRAYSSVRVSVYR

ALELWVQVAGASASILQGSPGHTELLFSHLLGDITPGAESVKLRAGLSADVIPGGKPGPRRTKPLVIADSVGPSLQRKGD
LLANQDTCLSALRALRQIILTSGTLLKDDIHKRLHEVVLPLCVRLQQQTSSGTACESAGGISGQYSSALSRRELYRLLLA
LVLVPSPSWPPPLTCAVSILSNGRIDCNLKVSTFCTEALTICNSLLHPRTPSISLPLPSLTLKPNPTAAVLPSSQGPTPG
LTLPTLLEGPPPGPPFPTRHPLGLGPSSLLGSLENHLSLVPGQAPIPGDMILSPRTHHQPEPAGLGPPDGQRPVFVRYDK
EEAEDVEISLASDSDDSVVIVPPGMLNVENQQDDTVAAANPQNMASAAPGTTVTLPGAETATMVPTTATTTTIDGISISN
DLTTSSPLITTSTTLINSFPPSSTSVVSLVPPLNSSQLTAPPGSLGDSLPGRPQLQQMLMQPSTSGQPGPMGLPLHHLQN
QLSQPGRHLHQHQPPPLPSSNEDSAVININSTDEEEEDEEDMDDDEELEEEEEGMDEEDEEEEVSDYADEEFYDGEEYED
FDDEEGEEEEDEEEDEDGDIPPLEGVEEKAGEMGMEEGKVLQAAVEEGEMAGLSMEGDSEGGIEEVQTNRAQFGEDRMNV
QEVESIGVLEEAREGEGDEDECERMDDPTMPQILCVTGGALEERDETEEEGARSGGGEVQEEASSWQQGASETEATASSE
DHTKSQSKEESGVETAHKASVNDNQPPSHQVEQVEADQQRDSASPDLETRSEMGLNTDNEEPHCERRAKVEEELQETEAG
GGAVSDDEVKGVKRKREEEVGQSTEKKKLDEEAMDAMLADFVACPPDDEDGASGSNCS
> Echeneis naucrates [A0A665V2D9]
MATSAWLHGPSAMRLTEGLVSVLKEHRPEFLPPLLANYREHGVFSVQGANAVGGLVGFSNAKLGSSKTRFEGLCLLAMLV
KDSSSDLFEQHCLTWLRSLQQVIQSQAPVQTIQLAVNILKDLLQYSSQLAELAREVGLNSILGILTSLLGLKNECELSAM
EGMKACMTYYPRACGSLRDKLGAYFLSKMDSTNKKTQEMACQCYGCLPCLGGLLDRGVGAGRAEGWTNQIHCLLASANGL
LAQIYQGSETDGTVQYEGPGMELAFPHLDQSDALLLLQLQHRYTAICLALKHTLRVDPASAVRLPIRAILNLVCRALAVS
SKSISLTGDGSLRLLVLPIIHTNTLDVLSALITAVCSNMVQYTVVLQRLFSQTLCAWTPPHEASLGQQRAYSSVRVSVYR
TLELWVQVAGASASILQGSPGNSELLLNHLLSDITPGSESIKLWAGLSADVVPGGKPGPRRTKPLALADTLGLSLQRKGD
LLANQDTCLSALRALRQIILTSGTLLKDDIHKRLHDVVLPMCAHLQQQQSSSNGACDSARVLSGQYSSALTRRELYRLLL
ALVLVPSPCWPPPLTCAVSILSSGRSDLSLKVSTFCAEALSICNSLLHPRTPSIALPLPPLTLKATPTTPVLPSSQGAAT
GLTLPTLLAGTNPGPPFPTRHSLGLGPTSLLGSLENHLSMVPGLPGQGPTSGDMILSPHTHHLSDQAGLGPPEGQRPVFV
RYDKEEAEDVEISLASDSDDSVVIVPPGMLNVENREEDVTTVANPQNLATATLGGITALLPGDRVATAPTTPAPTTIDGV
SLSNNLATSSTLLTTSTTPINSFPSSSTSVVSLVPPLNSSTLTAPSSGLGDSMPGRPHLQQMLMQPSSAGQSAPMGLPLQ
MHQLQNQLGQQGRHLHQHQPPPASNEDSAIININSTDEEEEEEDMEDDEELEDEEEGVDEEEEEEEEVSDFAEDEFYDGEE
YEDYDEEEGEEVEEEEEEEDGDIPPLEGAEDKAVDLGVEDRRVVQAAEEEEAMATFRLEGEEEGGGIEELQPNRVLFPEDR
MKVQEVESIGVLEEARGVEGEEDESERMNDPTMPQILCVTGGTMEEREETEEEKGAAGVAGAAVQREAHLWNQGTGETEL
TSPSEERPTNQGQQHSRADLEQEVSVSENQQCIHQEQLQPSTTQKEDKAAADAETFACPNPKDQVVTSSLKRHEEEVKLQ
DTEGSEGEGEGEGEEGKGVKRKRDDVHREEEAGQGIEKKKMDDEAMASMLADFVACPPDEEDGASGSSYS
> Cyprinodon variegatus [A0A3Q2DCP0]
MAASAWMHGAAAMRLTEGLVSVLKEQRPEFLSEALENYREHGVFSSQGSGDAAGLVGFSNAKLSSSKTRFEGLCLLSMLV
KDSSSELFQQHCLSWLRSLQQVIQSQAPTQTIQLAVNILKDLLQYSSQIAELAREVGLNSILGILTSLLGLKSECELAAM
EGMKACMIYYPRACGSLKDKLGAYFLSKMDSANRKTQETACQGYGRLPCLGGLADRSVGTGRAEGWTNQIQCLLASGNGL
LAQLYQGSETDDAVQYGGPGVEIRFPHLDQTDPLLLLQLRHRYAAVCLAIKHTLRVDPASAVHLPVRPILNLVCRGLAVN
AKSISLTADGSLRLLVLPGVHISTLEVLAELITVVRSGMMQYAAVIQRLFSQTLSAWTSPPESSVGQQRAFSSVRVSVYR
TLELWVQVVGASAHVLQGATSHAELLFNQLLGDIMPGAESIKLRAGLSADVVPGGKPGPRRTKQLVMADAAGPSLQRKGD
PLSNQDTCLSALKVLRRIIQTSGTLLKDDIHKRLHEVVLPLCVRLQQQQSGSMAASESVGGISGQYSSALTRRELYRLLL
ALVLVPSPSWPPPLTCAVSILSNGRVDRNLKVSSFCSEALTVCNSLLHPRAPSIALPLPPLALKPAPTPSVLSCQGPAPR
LALPTLLGGPAPGPPFSARHPLSMAPNSLLGSLENHLGLVPGLPGQGSGPGDLLLSPHSHSQTDPAGLGPPEGQRPVFVR
YDKEEAEDVEISLESDSDDSVVIVPPGMLAADNPPDDPAANPQSVLSSAPGGAIPAGETVTLATAASAQTALDVTSLPDD
LVTSSALLATSSAPINSFPPSSGSVVSLVSSLSAAAPAGGQADSMAAKPQLQQMLMQPSSAAAPGPMALPLQMHQLQNQL
AQQGRHLHQHPPAPAANEESAVININSTDDEEEDEEDMEDDEDLEEEEEDGIDEEDEEEEGSEEFYEGEEFEEFDEEEAE
ELEEDEEDEDGDMPPLEGSEDKSEEVVMEEEEEKVLPAEEEEGEGGVEELQSSRALMTKERLKVQEVESIGVLEEARDDD
DSERMDDPTMPQILCVTGGAPEEGGDGGREAAEAVGELQEKMELWDRSIEDNEPAGGQSQQEPVTESSEKEEPPPPLLLE
EQPPMAEGEGVASAGGTGDQEDAAAAPSVPEPEKTEAEQGDGKEEELTKPEEGGGGEEGGKEQEEESNEEEKGTKRKRED
ENMEEETGQSPEKKKQEDEAMASMLADFVACPPDDDDGGAAASGSKNP
> Myripristis murdjan [A0A667XQU4]
MATSAWLHGPSSMRLTEGLVSVLKEQRPEYLPALLANYREHGVFPTQVRAAAGGLVGFSNAKLGSSKTKFEGLCLLSMLV
KDSSSELFQQHCLSWLRSVQQVIQSQAPVQTIHLAVSVLQDLLQYSAQLAELAREVGLNSVLGILTSLLGLKTECELAAM
EGMTACMIYYPKACGSLRDKLGAYFLSKMDSSNKKTQEMACYCYGRLPCLGGVLERGVGAGRAEGWANQIHCLLASANGL
LAQMYQGSESDGTVQYEGPGVELPFPPFDQSDPLLLLQLQHRYSAVCLALKHTLSLQSITILSLCVNCANFYYFGILLLN
IMVHSMVQYSAVLQRLFSQTLSAWAPQPEASPGQQRAFSSVRVAVYRTLELWVQVGGASSSILQGNHTHSELLFGHLLGD
ITPGAESVKLRAGPSSEMLVSQKPGPRRTKSLVIVDGGPSLQKKGDLLANQDTCLSALKALRQIVMTSGTLLKDDIHKRL
HDVVLPLCVRLQQQQSSSSAALDSVGGISGQYSSAPPRRALYRLLLALVLVPSPRCPPPVTCAVSIFSRGRTDRSLKVSS
LCTEALTVCNSLLHPRNPSISLPLPPLTFKPTPTAPVLPPSQGPSSGLTLPTLLGGPSPGPPFPTRHSLSLGPAALLGSL
ENHLSLVPTGLPGQAPTPGDMLLSPHGHPQPDLAGLGPPEGQRPVFVRYDKEEAEDVEISLASDSDDSVVIVPPGMLSME
NQQDEASITANSAAQNLASITPGATSVALPGGEGGATGVTTTTAPTTIDGVSLANDLPSSSSSLLTTSTTTPINSFPPSS
ASLVSLVPPLNSTPLTAPPSALGESLPGGPQLQQMLMQPSTQGQPSPMGLPLQMHQLQNQLNQQGRHLHQHQPQPASNED
SAVININSSDDEEEEEEEMEDDDELEEEEGMEEEEEEDGSDFAEDEFYEGEEYEEYDEEEAEELEEEEEEEEEDGDIPPL
EGAEDRRGGLGIEEGKVLRGAVEEEEIGGFSVEGEAEGGIEEIQANRALFGEERMKVQEVESIGVLEEAREGEGEEDESE
RMDDPTMPQILCVTGGALDERQEPEEEAEGGGEGAPQEEGRSWEQGASEAQLTASSEEGSATQNQQVGPTVLPSLLTQMD
DEAMASMLADFIACPPDDEDGASGSNRS
> Cynoglossus semilaevis [A0A3P8V864]
MASTAWLHGSSVLRLTEGLVSVLKDQRPEHLPAVLASYREHGVLSTQSASTVGGLVGYSNAKLGSSKTRFEGLCLLSVLV
KDGSSDLFEQHCLSWLRSLQQVIQSHASGQTIKLAVSILKDLLQYSSQLPELSREVGLNSILGILTSLLGLKAECELWAM
DGMMACMTYYPRACGSLRDKLGAYFLTKMDCTNKNTQEVCHCYGRLPCLGGLLDRGVSTGRTEGWTNQVHCLVASANALL
AEIYQGSETDGTVQYDGPGIELAFPHLDQMDPLTLLQLQHRYTSVCLALKHTLRSDPASPVRVPIRPVLNLVCRALAVSC
KSINLTGDGSVRLLILPNIHTSTLEVLAALITVVRSGMVQYTLVLQRLFSQTLASWTPSHEAGVGQQRAYSSVRVSVYRT
LELWVQVAGASASILQGSPVHSELLFSQLLRDIAPGVDTGKLRSRLSADEPLVEKLATRRNKPSVMGDSGGTSLPRKGDH
LANQDTCLTALRALRQVILTSGALLKDDMHKRLHEVVLPLCVHLQQQQQSGSSIVYDAAGHLGGQYRSSFARRELYRLLM
ALVLVPSSCWPSPLTCAVSIFSKGRNDSNLKISTFCTEALTICNSLLHPRTPSTALPLPPLTLKSPPTAPVLTSTQGPGP
TSGLTLPTLLGATAPGPIFPVRHSLNLGPSSLLGSLENQLSLVPGLSCPAPTPGEVILSPPAHHLPDQAGLGPAECQRPV
FVRYEKEEAEDVEISLASDSDDSVVIVPPGMLNMENSQDAVSTAANSHSMLSVASGAPAVTLSAGECTPLTANSNNLSTS
SSPLITSNNPVNSCPSSSTSLVSMVPPLNASTLTPTSGLGNSMSSRAQLQQMLMQPSAGAQPPSIAPPLQMHQLQNQLTQ
PGRAFHQRPSPLPTSNEDTAIININSTDDEEEDMEDEEDELEEEGEDEDGDMPPKVEEEGERHVHPQEVDGDIPPLEGA
EDNAEDAEGLQPPALLDDCVSRYSGEGYEEGGIEEIQSNRVLFGDDRIKVQEVESIGVLEEARADEEDESERADDPSMPQ
ILCVXXXXXXXGVDGRQHKRGRRQRRRRRRGVAGSDGAVGARKLNQVLWSAPQLDDEAMASMLADFVACPPDDEDHASGS
K

> Mastacembelus armatus [A0A7N8XTX5]
MATSAWLHGPSAMKLTEGLVSVLKEHRPEYLPDLLANYREFGVFLNQGASTVGALVGFSNAKLGSSKTRFEGLCLLSMLV
KDSSSDLFQQHCLSWLRSLQLVIQSQAPVQTIQLAVNILKDLLQYSSQVAELAREVGINSILGFLTSLLSLKTECELAAM
EGMTACMTYYPRACGSLRDKLGAYFLSKMDSTNKKTQEMACQCYGRLPCLGGLLDRGVSAGRAEGWTNQIHCLLASANGL
LAQIYQGSETDGAVPYDGPGVELAFPYLDQSDPLLLLLLQHRYTAVCMALKHTLRLDPASAVRLPVRPILNLVCRTLAVT
SKNINLTGDGSLRILVLPIIHTNTLEVLSALIKIVQSGMVQYAAILQRLFSQTLSAWTPLPEANLGQQRAYSSVRVSVYR
TLELWAQVAGTSATILQGSPSHSELLFSHLLSDITPGTESIKLRAGLLADVVPGGKPGPRRTKPLVITDTVVHSLQRKGD
PLANHDTCLSALRALRQIILTSGVLLKDDIHKRLHDVVLPLCVRLQQQLSSSSAACESAGVISGQYSSALTRRELYRLLL
ALVLVPPPCWPPPLTCAVSILSNGRTDCHLKVSTFCTEALSICNSLLHPRTPSIALPLPSLALKPTPTAPVLPSTQGPTP
GLTLPTILGGPTPGHPFSARHSLGLGPASLLDSLENHLSLVSALPGQASTAGDMILSPHTHHQPDPAGLGPPEGQRPVFI
RYDKEEAEDVEISLASDSDDSVVIVPPGMLNIENQQDDTAEAANSQIMTSVAEGVTTVTLPTTTVPVTTVPTTAGTTTID
GITLPNNLASSPPLLTTSTTPINSFPSSSTSVVSLVPALSSSSLAPPLGDSLPARPQLQQMLMQPSTPNQPNSMGLPVQM
HKLQNQLGQHGRHLHQHQPPPTSKEDSAVININSTDDEEEEEDMEDDEELEEEEEGMDEEDEEEEDFVDEEFYGEDDYEE
YDEEEGEDLDEEEEEEEEGDIPPLEGAEDKVGELGMEEGKVLRTVDEGGMAGFSMEGDREGGIEEIQSNRALVEEDRMKVQ
EVESIGVLEAAREGEEDENERMDDPTMPTILCVTGGALEERQETEVEGGTIGGEVEQEEVGPWEQRASEIEPTGSSEECT
TNRNQQKSGAEPANGASMSSFQPPSHQEEQLVAAQGDSEAGDDPEKSSLNTKEREDVDVEKRDEMKEEPKETEGGGRGES
DGEEGRRMKRKREEVHRDEDVRESTEKKKVKKKKKKKKTWLTLLHLFSHLHPIDHARYSC
> Sailfin molly [A0A3B3VVJ6]
MATSAWMHGTAAMRLTEGLVSVLKEQRPEYLSEVLANYREHGVFPTQGASDVAGLVGFSNAKLSSSKTRFEGLCLLSMLV
KDSSSDLFQQHCLSWLRSLQQVIQSQAPCQSIQLAVSILQDLLQYSCQLAELAREVGLNSILGILTSLLGLKTECELAAM
EGMRACMVYYPRACGSLKDKLGAYFLSKMDSTSRKTQEMACQGYSHLPCLGGLADRSVGTSRAEGWTNQIQCLLASANGI
LAQIYQGSETDEAVQYRGPGVELPFPHLDQTDPLLLLQLQHRYTAVCLALKHTLRADPASAVQLPIRPILNLVCRALAVN
SRSTSLTADGSLRLLVLPSVHIITLEVLAELITVVRSGMVQYAAVIQRLFSQTLCAWTALPEANVGQQRAYSSVRVSVYR
TLELWLQLVGASANVLQGASSHAELLFTHLLADIMPGAESVKLRAGLSADVVPGGKPGPRRTKQLVMADPVGASLQRKGD
PGSNQDTCLSALKVLRRIIQTSGTLLKNDIHKRLHEVVLPLCVRLQQQQTSSIAACESVGGVSGQYSSALTRRQLYRLLL
ALVLVPSPCWPPPLTCAVSILSNGRLDRNLKVSSFCSEALIVCNSLLHPRTPSIALPLPPLALKPPPTPSVLSGPGPAPR
LTLPTPQGGPAPGPPFPGRHPLSLGPNSLLVSLENHLSLVPGLPGQGPASAPGDLLLSPHGQPQQDPAGLGLPDGQRPVF
VRYDKEEAEDVEISLASDSDDSVVIVPPGMLGADSQLDDPAPGSQNLLSAAVGAGANLPGGDTVAMVPSTAAPASLDVTS
LPNDLVPTSSTPANSFPPSSGSVVSLVTSLTPGSAPPPAGGLADPVAVKPQLQQMLLQPPAPGPQGPPGSMAMPLQVQLQ
NQLAQQGRHLHQHPPPAPPSNQDSAVININSTDDEDEDEEDMEDDEELEDEEDGLDEEEEEEEEGSEDFYEGEDYEDFDED
AEELEEEEEEDEDGDMPPLEGSEDKSEEVTMEEEEKALRAEEEEGEGAGFNPEGGIEELQSSRALFGEERMKVQKVESIGV
LEEARDGEEGGDRGDREDDSERMDDPSMPQILCVTGGALEERADSSQEAAQAAGELQDQLTLWDQNRQQNPPTGALEEEP
TAHSQQESVAEREQEAGEGEEVAVEAELQEEEEPVVVQEEEAAAGASGGEETPAAPHDPQQDQTDTQQGYETQAEWTKPE
GEKEEESNSEEEKRMKRKREEDDIEDEGGPSPEKKKQDDEAMASMLADFIACPPDDEDVASGSSQS
> Tiger tail seahorse [A0A3Q2YL26]
MSASSWLHGPSAGRLTEGFLSVLKEQRPEYLPSLLDSYREHGVFRTQGASAVGGLLGFSNAKLASSKTKFEGLCLLAMLV
QDSSSELFQQHCLSWLRSLQQVIQSQAPVHTIQLAVTILKDLLQYSSQLAELAREVGLNCVLGILTSLLGLKTECELAAM
EGMTACMTYYPRACGSLRDKLGAYFLSKMDSSNKKSQEMACQCYARLPCLGGLTDRASGASRADRWSDQVHCLLASANGA
LAHLYRGAESDRTAQYEGPGVELGFPPLDHSDALLPLQLQRRYTAVCLALKHTLRVDPASAVRLPVRPILNLVCRALAVS
AKSINLTGDGSVRLLLLPAIHTCTLEVLSALFTVVRTSMVQYAAVLQRLFSQTLSAWTPPPEANPGQQRAYSSVRVSVYR
SLEAWVQVAGASAGILQGSSSHTELLFAHLLGDITPGAESIKLRAGLSADVVPGGKPGPRRTKPLVMADAVVPSLQRKGD
SLANQDTCLSALRALRQIILTSGTLLKDDLHKRLHEVLLPLCVRLQQQQSSCNAASESAVSVSGQYGGALTRRELYRLLL
ALVLVPSPTWPPPLTCAVSILSKGRVDRNLKVSSFCSEALTMCNALVHPRAPSIAIPLPPLTLKATPSAPVLPSSQGPTP
GLPLPTLLGGPGSSGPFPARHSLALLSSLENHLSLVPGHASGDAILSPHPQHQPETAGLGPPDGQRPVFVRYDKEEAEDV
EISLASDSDDSVVIVPPGMLAAEVREPVETSAAPPQNPASAAAPPAGGAPEPMALAQAATDGVSLPNDLATSPLPTAPVT
SFAPPASSVVSLVPPGGSGEGETPAVRPQLQQMLMQPSGPGALGLPLQMQQLQNQMGPPQGRLPASSNNEDSAVININST
DDEDEEEDMEDDEELEEDEDEEEEVSDFPEGEFYEGEEFDEFDEEGEELEEEEEDEEEDEEDEEEDDGGEDGPMPPLEGA
EDKANVGGIEEEEVRRAEVAPAASSADGEAVGGIQEIQLSRADIADERTRVQEVESIGVLEGAREDEEEEEESERMDDPT
MPQILCVTGGAPEEGEEPAKAETNEVAAFRHEPPVAELHPPLQEVAPGPVQEASAGDDQPPVLQNELLAAPPEDSGPPPS
EPAALLGPEERPQEEEKDKDTQGAGMESDAEESEGDEGKGVKRKRDDAHMDEEAGQSTDKKKMHDDAIATMLADFVPCPP
DDDEVTASGSNP
> Poecilia Formosa [A0A087Y3D1]
MATSAWMHGTAAMRLTEGLVSVLKEQRPEYLSEVLANYREHGVFPTQGASDVAGLVGFSNAKLSSSKTRFEGLCLLSMLV
KDSSSDLFQQHCLSWLRSLQQVIQSQAPCQSIQLAVSILQDLLQYSCQLAELAREVGLNSILGILTSLLGLKTECELAAM
EGMRACMVYYPRACGSLKDKLGAYFLSKMDSTSRKTQEMACQGYSHLPCLGGLADRSVGTSRAEGWTNQIQCLLASANGI
LAQIYQGSETDEAVQYRGPGVELPFPHLDQTDPLLLLQLQHRYTAVCLALKHTLRADPASAVQLPIRPILNLVCRALAVN
SRSTSLTADGSLRLLVLPSVHISSLEVLAELITVVRSGMVQYAAVIQRLFSQTLCAWTALPEANVGQQRAYSSVRVSVYR
TLELWLQLVGASANVLQGASSHAELLFTHLLADIMPGAESVKLRAGLSADVVPGGKPGPRRTKQLVMADPVGASLQRKGD
PGSNQDTCLSALKVLRRIIQTSGTLLKNDIHKRLHEVVLPLCVRLQQQQTSSIAACESVGGVSGQYSSALTRRQLYRLLL
ALVLVPSPCWPPPLTCAVSILSNGRLDRNLKVSSFCSEALIVCNSLLHPRTPSIALPLPPLALKPPPTPSVLSGPGPAPR
LTLPTLLGGPAPGPPFPGRHPLSLGSNSLLGSLENHLSLVPGLPGQGPASAPGDLLLSPHGQPQQDPAGLGLPDGQRPVF
VRYDKEEAEDVEISLASDSDDSVVIVPPGMLGADSQLDDPAPGSQNLLSAAVGAGANLPGGDTVAMVPSTAAPASLDVTS
LPNDLVPTSSTPANSFPP
> Poecilia reticulata [A0A3P9N6D6]
MATSAWMHGTSAMRLTEGLVSVLKEQRPEYLSEVLANYREHGVFPTQGASDVAGLVGFSNAKLSSSKTRFEGLCLLSMLV
KDSSSDLFQQHCLSWLRSLQQVIQSQAPCQSIQLAVSILQDLLQYSCQLAELAREVGLNSILGILTSLLGLKTECELAAM
EGMRACMVYYPRACGSLKDKLGAYFLSKMDSTSRKTQEMACQGYSRLPCLGGLADRSVGTGRAEGWTNQIQCLLASANGI
LAQIYKGSETDEAVQYRGPGVELPFPHLDQTDPLLLLQLQHRYTAVCLALKHTLRADPASAVHLPVRPILNLVCRALAVN
SRSTSLTADGSLRLLVLPSVHISTLEVLAELITVVRSGMVQYAAVIQRLFSQTLCAWTALPEATVGQQRAYSSVRVSVYR
TLELWLQLVGASANVLQGASSHAELLFTHLLADITPGAESVKLRAGLSADVVPGGKPGPRRTKQLVMADPVGASLQRKGD
PGSNQDTCLSALKVLRRIIQTSGTLLKNDIHKRLHEVVLPLCVRLQQQQTSSIAACESVGGVSGQYSSALTRRQLYRLLL
ALVLVPSPCWPPPLTCAVSILSNGRLDRNLKVSSFCSEALIVCNSLLHPRTPSIALPLPPLALKPPPTPSVLSGPGPAPR
LTLPTLLGGPAPGPPFPGRHPLSLGPNSLLGSLENHLSLVPGLPGQGPASAPGDLLLSPHGQPQPDTAGLGPPDGQRPVF
VRYDKEEAEDVEISLASDSDDSVVIVPPGMLGADSQLDDPAPGSQNLLSAAVGAGANLPGGDIVAMVPSTAAPASLDVTS
LPNDLVPTSSTPANSFPPSSGSVVSLVTSLTPGSAPPPAGGLADPVAVKPQLQQMLLQPPAPGPQGPPGSMAMPLQVQLQ
NQLAQQGRHLHQHPPPAPPSNQDSAVININSTDDEDEDEEDMEDDEELEDEEDGLDEEEEEEEEGSEDFYEGEEYEDFDDE
EAEELEEEDEDEDGDMPPLEGSEDKSEEVTMEMEEEEEEEKALRVEEGEGEGVGFNPEGGIEELQSSRALFGEERMKVQE

VESIGVLEEARDGEEGGGRGDREDDSERMDDPSMPQILCVTGGALEERADSSQEAAQAAGELQDQLTQWDQNHQQNPPTG
ASEEEPTTHSQQESVAEREQEAAEGEEVAVEAELQEEDEPVVVQEEEAAAGASGGEETPAAPHDPQQDQTDTQQGYETQA
EWTKPEGEKEEESNSEEEKRMKRKREEEDLEDEAGPSPEKKKQDDEAMASMLADFIACPPDDEDIASGSNQS
> Shortfin molly [A0A3B3WEA9]
MATSAWMHGTAAMRLTEGLVSVLKEQRPEYLSEVLANYREHGVFPTQGASDVAGLVGFSNAKLSSSKTRFEGLCLLSMLV
KDSSSDLFQQHCLSWLRSLQQVIQSQAPCQSIQLAVSILQDLLQYSCQLAELAREVGLNSILGILTSLLGLKTECELAAM
EGMRACMVYYPRACGSLKDKLGAYFLSKMDSTSRKTQEMACQGYSHLPCLGGLADRSVGTGRAEGWTNQIQCLLASANGI
LAQIYQGSETDEAVQYRGPGVELPFPHLDQTDPLLLLQLQHRYTAVCLALKHTLRADPASAVQLPIRPILNLVCRALAVN
SRSTSLTADGSLRLLVLPSVHISSLEVLAELITVVRSGMVQYAAVIQRLFSQTLCAWTALPEANVGQQRAYSSVRVSVYR
TLELWLQLVGASANVLQGASSHAELLFTHLLADIMPGAESVKLRAGLSADVVPGGKPGPRRTKQLVMADPVGASLQRKGD
PGSNQDTCLSALKVLRRIIQTSGTLLKNDIHKRLHEVVLPLCVRLQQQTSSIAACESVGGVSGQYSSALTRRQLYRLLL
ALVLVPSPCWPPPLTCAVSILSNGRLDRNLKVSSFCSEALIVCNSLLHPRTPSIALPLPPLALKPPPTPSVLSGPGPAPR
LTLPTLLGGPAPGPPFPGRHPLSLGSNSLLGSLENHLQVPGLPGQGPASAPGDLLLSPHGQPQPDPAGLGPPDGQRPVFV
RYDKEEAEDVEISLASDSDDSVVIVPPGMLGADSQLDDPAPGSQNLLSAAVGAGANLPGGDAVAMVPSTAAPASLDVTSL
PNDLVPTSSTRANSFPPSSGSVVSLVTSLTPGSAPTPAGGLADPVAVKPQLQQMLLQPPAPGPQGPPGSMAMPLQVQLQN
QLAQQGRHLHQHPPPAPPSNQDSAVININSTDDEDEDEEDMEDDEELEDEEDGLDEEEEEEEGSEDFYEGEDYEDFDDED
AEELEEEDEDGDMPPLEGSEDKSEEVTMEEEEEEKALRAEEEEGEGAGFTPEGGIEELQSSRALFGEERMKVQKVESIGV
LEEARDGEEGGDRGDREDDSERMDDPSMPQILCVTGGALEERADSSQEAAQAAGELQDQLTLWDQNRQQNPPTGALEEEP
TAHSQQESVAEREQEAGEGEEVAVEAELQEEEEPVVVQEEEAAAGASGGEETPDAPHDPQQDQTDTQQGYETQAEWTKPE
GEKEEESNSEEEKKMKRKREEDDIEDEGGQSPEKKKQDDEAMASMLADFIACPPDDEDVASGSNQS
> Mola mola [A0A3Q3XQ36]
MATSALLRGPSAMQLTEGLVSVLKEQRPEYLPALLASYREHGVFQAQVRSGRRGLCLLSMLVKDSSSDLFQQHCLSWLRS
LQQVIQSQAPVQTIQLAVNILKDMLQYSSQLPELAREIGLNSVLGILTSLLGLKTECELAAMEGMTACMTYYPRACGSLK
DKLGAYFLSRMDCTNKKTREMACQCYGHLPCLGGLLDRGVGARRAEGWTNQIHCLLASANSQLSQIYQGAETDGTVQYEG
PGLELAFPHLDQSDPLLLLQLQHRFTAACLALKHSLRMDPASAVRLPVRPILNLVCRALAVSCKSILVLYGCMHLWLLTF
GMVQYAALLQRLFSQTLSAWTALPEASLGQQRAYSSVRVSVYRTLELWLQVAGSSACILQGSPCQSELLFSHLLNDITPG
AESIKLRVGLSADVVPGGKPGPRRTKSFVIADTVGVSLQRKGDLMANQETCLSALRALRQIILTSGTLLKGDIHKRLHDV
LLPLCVRLQQQSSSSPLCESAGGISGQYSSALTRRELYRLLLALVLVPSPCWPPPMACALSILSNGRSDRNLKVSSFCS
EALTICNSLLHPRTPSITLPLPPLTLKPTPAPSAPPVSQGSTPGLTLPTLLGGPPLGPPFPPRQTLNLGPTSLLGSLENH
LSLVPGLPGQTPAPGEMILSPHSHHQHDPTVVGPPEGQRPVFVRYDREEAEDVEISLASDSDDSVVIVPPGMLSMENQQD
ETAAANSQNMASATSGGALVTLTEGESTATTTTVGVSLPNDLTTSSPLLTTSTTPINSFPPSSASVVSLVPPLNTSTSTI
TVQPDGMGESMPGRPQLQQILMQPSTPGQPGPISLPLQMHQLQNQLSQQGRHLHQHQPPAASNEDSGVININSTDDEEEE
EEDMEDDEELEEEEEEEEGMDEEVEEEEEVSDFADEEFYDGEEYDEFDEEEGEELEEEEEEEEDGDIPPLEGAEERAENGG
REEGKMQQAAVEEAGIVGFSVEGETEAGIEEIQTNRPFSVEDRMKVQEVESIGVLEEAREGERGDDESERMDDPTMPQIL
CVTGGPIEEREEEGRKAGKGERGGGIQKEASSWEEEASGSELKITSEEATPNKNQQVLRVLKFHLSLLNCSKMTEG
> Poecilia Formosa [A0A096LPN8]
MRLTEGLVSVLKEQRPEYLSEVLANYREHGVFPTQGASDVAGLVGFSNAKLSSSKTRFEGLCLLSMLVKDSSSDLFQQHF
VTALSSCCVLLQSQAPCQSIQLAVSILQDLLQYSCQLAELAREVGLNSILGILTSLLGLKTECELAAMEGMRACMVYYPR
ACGSLKDKLGAYFLSKMDSTSRKTQEMACQGYSHLPCLGGLADRSVGTSRAEGWTNQIQCLLASANGILAQIYQGSETDE
AVQYRGPGVELPFPHLDQTDPLLLLQLQHRYTAVCLALKHTLRADPASAVQLPIRPILNLVCRAGFALTSIHFSLTADGS
LRLLVLPSVHISSLELLSVKVRSGMVQYAAVIQRLFSQTLCAWTALPEANVGQQRAYSSVRVSVYRTLELWLQLVGASAN
VLQGASSHAELLFTHLLADIMPGAESVKLRAGLSADVVPGGKPGPRRTKQLVMADPVGASLQRKGDPGSNQDTCLSALKV
LRRIIQTSGTLLKNDIHKRLHEVVLPLCVRLQQQTSSIAACESVGGVSGQYSSALTRRQLYRLLLALVLVPSPCWPPPL
TCAVSILSNGRLDRNLKVSSFCSEALIVCNSLLHPRTPSIALPLPPLALTSVLSGPGPAPRLTLPTLLGGPAPGPPFPGR
HPLSLGSNSLLGSLENHLSLVPGLPGQGPASAPGDLLLSPHGQPQQDPAGLGLPDGQRPVFVRYDKEEAEDVEISLASDS
DDSVVIVPPGMLGADSQLDDPAPGSQNLLSAAVGAGANLPGGDTVAMVPSTAAPASLDVTSLPNDLVPTSSTPANSFPPS
SGSVVSLVTSLTPGSAPPPAGGNQDSAVININSTDDEDEDEEDIEDFYEGEDYEDFDEDAEELEEEEEDEDGDMPPRALF
GEERMKVQKVESIGVLEEDSERMDDPSMPQILCVTGGALEERADSSQEAAQAAGELQDQESVAEREQEAGEGEEVAVEAE
LQEEEEPVVVQEEEAAAGADQTDTQQGYETQAEWTKPEGEKEEESNSEEEKRMKRKREEDDIEDEGGPSPEKKKQDDEAM
ASMLADFIACPPDDE
> Oreochromis aureus [A0A668U477]
MATSAWLHGPSAMRLTEGLVSVLKEQRPEHLPALLAGYREHGVFQTQSACAVGGLVGFSNAKLGSSKTRFEGLCLLSMLV
KDSSSDLFQQHCLSWLRSLQQVIQSQAPVESVQLAVNILKDLLQYSSQLAELAREVGLNSILGILTSLLGLKSECELAAM
EGMTACMTYYPRACGSLRMACQCYSRLPCLGGVLDRGVGAGRAEGWTNQIHCLLASANSLLAQLYQGSETDGAMQYQGAG
VELAFPHLDQTDPLLLLQLQHRYTAVCLALKHTLRVDPASAVRLPVRPILNLVCRALAVSSKSINLTGDGSVRLLVLPII
HSNTLEVLSSLITAVRVGMVQYAAVIQRLFSQTLSAWTPLPETSVGQQRAYSAVRVSVYRTLELWVQVAGASSNILQGSP
GHSELLFNHLLGDITPGAESIKLRAGLSADVVPGGKPGPRRTKHLVMADTVGPSLQRKGDHLANQDTCLAAVRALRQIIQ
TSGTLLKDDIHKRLHEVVLPLCVRLQQQSCSSNACESAGSASGQYSSALTRRELYRLLLALVLVPSPCWPPPLTCAVSI
LSNGRTDRNVKVSSFCAEALTICNLILHPRFPSIALPLPPLTMKPSSTTPVLPSSQGPAPGLTLPTLLGGPAPGPPFPTR
HSLNLGPASLLSSFENHLSLVPGLPGQGPTSGDMILSPHTHHQPDPAGLGPPEGQRPVFVRFDKEEAEDVEISLASDSDD
SVVIVPPGMLNMETQQDDTAANSQSMPSSAPGGTGVTLPGAESVTMVPTTAATATIDGVSLPNDLAASSPLLTASTTSIN
SFPPSSASVVSLVPPLNSSTFTAPPVGLGESLPGRPQLQQMLMQPSTPSQAGPMGLPLQMHLQNQLSQQGRHLHQQPAAP
ASSEDSAVININSTDDEEEDDEDIDEDEELEEDEEEEGIDEEDEEEDVSELADDFYDGEEYEELDEEDGEELEEEEDEEE
EEEDGDIPPLEGVEDKDGGSGIQEGKLDEGRISGFNVEERTEGGIEEIQTNRALFGEDKMKVQEVESIGVLEAAREGEVE
EEDNERMDDPTMPQILCVTGGALEEREEAEEEREQAGAELQKGEECTWEQGAKEDEPQAAESEQPASHSQQQESAAEPAE
EASVSDNQLPSCEDEQPEAVQAGDSTAVADSENSTEQNVTEQQEADTEKSSEKEAEQPETGGGGAQESNGEEGKGVKRKR
EEGHGKEEVGQSPEKKKMDDEAMASMLADFVACPPDDEDGASGSKAS
> Japanese medaka HNI [A0A3P9M5Q7]
MATSVGLHGSPALRLTEGLVSFLKEQRPEYLPEVLVNYREHGLQIQGANDVAGLVGFSNAKLSSSKTRFEGLCLLSVLVK
DSSSDLFQQHCLFWLRSLQQIIQSQAPVQTVQLAVNILKDLLQYSSHLAELAREVGLNSILGILTSLLGLKTECELAAME
GMVACMLFYPRACGSLKDKLGAYFLSKMDSNNMKTQEMACLCYSHLPCLGGLLDRAISAGKADGWTNQIHCLLATADALL
TQIYKGSEPEMVVQYTGPGVELAFPYLDPTEPLLLLRLQQRYTAVCRAMKHTLRVDPASAVRLPVKPILNLVCRALAVNS
KSTSLSADGSLMLLILPSIHCNTLEMLSDLITVVRSSMVPYAAIIQRLFSQTLSAWTPLVEAGVGRQRGYSSVRVSVYKT
LELWVKVVGASVSILQENPMHADLLFSNLLGDITPGPESIKFRAGLGVDVVPGGKPGPRRTKHLVIADPMGPSLQRKGDP
LSNQDTCLAALRALRQIIQTCGTLIKEDIHKRLHEVVLPLCVCLQQQKGSSSVFESTGAISGQYSSARARRELFRLLLAL
VLVPSPSWPPPLTCAVSIFSNGRTDRNLKVSSFCAEALTICNTMVHPRTPSIALPLPPLTLKPPTSAPVLSPQGSAPRLT
LPTLLEGHGSGPPFPSRQSLNLGPSSMLSSLENHLSTVPGLPAQGPTPGDLFSPHRQHDSGLGLPESQRPVFVRFDKEEP

DDVEISLASDSDDSVVIVPPGMISLDQQQDEAAANVQTMLSAAPAGGGVALPAAESNSIVPITAATSTIDVSTLPNDLPA
SSSLLATSASSVNSFPPSGGSAAPLPPALNSGIVSDSHGDLADALPCKPQLQQMLMQAPAAGQPSAMGLPLQMLQTPLSQ
QGRLHPPLPAPPSNEDSAIININSTDDEDEDEDLEDDEELEEEEEDGLDEEDEEEGSEDFYDGEEYEDYDEEEGEELEEE
EEEEEEEIGDMPPLEGSEEKLVDVAIEEGQVLHPAAAAADDKEPSQHHAEVDSERGIQELQPQRTVFGEDRVKLQEVESI
GVLEDVQVGVEEDESERMDDPTMPQIICVSGGAQESVESGDEGGGTTSVLQEDRSHQAAKEEELEAAAEEQAGFRAEEPE
GETAPEDVSSSDALDPPAGRPAGAPAEQAEAGRQSPAEEEEKQAENPASEGGEQPKGGEEEEKSEPPREESDKEEAQGMK
RKREDEVEEEAGPSTEKKKMDEDAMASMLADFVACPPDDEAAAAAAGSTCS

> Mastacembelus armatus [A0A3Q3RF58]

MATSAWLHGPSAMKLTEGLVSVLKEHRPEYLPDLLANYREFGVTVGALVGFSNAKLGSSKTRFEGLCLLSMLVKDSSSDL
FQQHCLSWLRSLQLVIQSQAPVQTIQLAVNILKDLLQYSSQVAELAREVGINSILGFLTSLLSLKTECELAAMEGMTACM
TYYPRACGSLRDKLGAYFLSKMDSTNKKTQEMACQCYGRLPCLGGLLDRGVSAGRAEGWTNQIHCLLASANGLLAQIYQG
SETDGAVPYDGPGVELAFPYLDQSDPLLLLLLQHRYTAVCMALKHTLRLDPASAVRLPVRPILNLVCRTLAVTSKNINLT
GDGSLRILVLPIIHTNTLEVLSALIKIVQSGMVQYAAILQRLFSQTLSAWTPLPEANLGQQRAYSSVRVSVYRTLELWAQ
VAGTSATILQGSPSHSELLFSHLLSDITPGTESIKLRAGLLADVVPGGKPGPRRTKPLVITDTVVHSLQRKGDPLANHDT
CLSALRALRQIILTSGVLLKDDIHKRLHDVVLPLCVRLQQQLSSSSAACESAGVISGQYSSALTRRELYRYYFYSYTIKN
RFTPAFFVFGYYFESKNILFVSTFCTEALSICNSLLHPRTPSIALPLPSLALKPTPTAPVLPSTQGPTPGLTLPTILGGP
TPGHPFSARHSLGLGPASLLDSLENHLSLVSALPGQASTAGDMILSPHTHHQPDPAGLGPPEGQRPVFIRYDKEEAEDVE
ISLASDSDDSVVIVPPGMLNIENQQDDTAEAANSQIMTSVAEGVTTVTLPTTTVPVTTVPTTAGTTTIDGITLPNNLASS
PPLLTTSTTPINSFPSSSTSVVSLVPALSSSSLAPPLGDSLPARPQLQQMLMQPSTPNQPNSMGLPVQMHKLQNQLGQHG
RHLHQHQPPPTSKEDSAVININSTDDEEEEEDMEDDEELEEEEEGMDEEDEEEDFVDEEFYGEDDYEEYDEEEGEDLDE
EEEEEEGDIPPLEGAEDKVGELGMEEGKVLRTVDEGGMAGFSMEGDREGGIEEIQSNRALVEEDRMKVQEVESIGVLEAA
REGEEDENERMDDPTMPTILCVTGGALEERQETEVEGGTIGGEVEQEEVGPWEQRASEIEPTGSSEECTTNRNQQVIVQF
FLMCHL

> Indian medaka [A0A3B3BH77]

MATSVGLHGSSALKLTEGLVSFLKEQRPEYLPEVLVNYREHGLQIQGQNGAAGLVGFSNTKLSSSKTRFEGLCLLSMLVK
DSTSDLFQQHCLFWLRSLQQIIQSQAPVQTIQLAVNILKDLLQYSCQLAELAREVGLNSILGILTSLLGLKTECELAAME
GMVACMVFYPRACGSLKDKLRAYFISKMDSNNKKTQEMACLCYSHLPCVGGLLDRAVSAGKADGWTNQIHCLLATADGLL
SQIYKGSEPETVVEYQGPGVELAFPYLDPTETLQLLRLQQRYTAVCRAVKHTLRVDPASAVRLPVKPVLNLVCRALAVNA
KNASFSADGSLMLLILPSIHCNTLEMLSDLITVVRSSMVPYAAIIQRLFSQTLSAWTPVVEAGVGRQRGYSSVRVSVYKT
LELWVKVVGASVSILQENPMHADLLFNNLLGDITPGPESIKLRAGLGVDVVPGGKPGPRRTKHLVIADPMGPSLQRKGDP
LSNQDTCLAALRALRQIIQTCGTLIKEDIHKRLHEVVLPLCVCLQQQKGSSSVFESTGAISGQYSSALARRELYRLLLAL
VLVPSPSWPPPLTCAVSIFSNGRTDRNLKVSSFCAEALTICNTIVHPRTHSIALPLPPLTLRPSASAPVLSPQGSAPRLT
LPTLLEGHGSGPPFPSRQSLNLGPSSLLSSLENHLSTVPGLPAQAPTPGDLFSPHPHQQHDSAGLGLPESQRPVFVRFDK
EEPDDVEISLASDSDDSVVIVPPGMITLDNQQDEAAANPQTMLSAAPAGSGVTLPVVESNSMVPITAATTTIDVSTLPND
LPTSSSLLTTSASSINSFPPPSASAATLPPALNSSIVSDPHSDLGDSLPCKPQLQQMLMQTPTSSQGSSMGLPLQMHQLQ
NQLTQQGRLHQQLPAPPSNEDSTIININSTDDEDEEEDLEDDEELEEEEEGLDEEDEEEGSDDFYDGEEYEDYDEEEGEE
LEEEEEEEENGDMPPLEGSEEKLVDVAIEEGQVLHPAAAADEKVLTEHNVEADSERGIQELQPQRTMLGEERVKLQEVES
IGVLEDALVGVEEEDESERMDDPTMPQIICVSGGAQESIETGDEGEGTAHVLQEDTSNQPAKEDELKAAPEEQTSFSAEQ
EPEVETAQEDESSSSKTSDQPAELPAAAPEEPTEAGQQSPADLNTGEQQEENQASEDGEQPKDGEEEEKNRSIEPQREES
DKEEAHGTKRKREEELTEEAGPSTEKKKVDDEAMASMLADFVACPPDDEAAPGSTCS

> Poecilia Formosa [A0A096MG46]

MATSAWMHGTAAMRLTEGLVSVLKEQRPEYLSEVLANYREHGVFPTQGASDVAGLVGFSNAKLSSSKTRFEGLCLLSMLV
KDSSSDLFQQHFVTALSSCCVLLQSQAPCQSIQLAVSILQDLLQYSCQLAELAREVGLNSILGILTSLLGLKTECELAAM
EGMRACMVYYPRACGSLKDKLGAYFLSKMDSTSRKTQEMACQGYSHLPCLGGLADRSVGTSRAEGWTNQIQCLLASANGI
LAQIYQGSETDEAVQYRGPGVELPFPHLDQTDPLLLLQLQHRYTAVCLALKHTLRADPASAVQLPIRPILNLVCRAGFAL
TSIHFSLTADGSLRLLVLPSVHISSLELLSVKVRSGMVQYAAVIQRLFSQTLCAWTALPEANVGQQRAYSSVRVSVYRTL
ELWLQLVGASANVLQGASSHAELLFTHLLADIMPGAESVKLRAGLSADVVPGGKPGPRRTKQLVMADPVGASLQRKGDPG
SNQDTCLSALKVLRRIIQTSGTLLKNDIHKRLHEVVLPLCVRLQQQTSSIAACESVGGVSGQYSSALTRRQLYRLLLAL
VLVPSPCWPPPLTCAVSILSNGRLDRNLKVSSFCSEALIVCNSLLHPRTPSIALPLPPLALKPSVLSGPGPAPRLTLPTL
LGGPAPGPPFPGRHPLSLGSNSLLGSLENHLSLVPGLPGQGPASAPGDLLLSPHGQPQQDPAGLGLPDGQRPVFVRYDKE
EAEDVEISLASDSDDSVVIVPPGMLGADSQLDDPAPGSQNLLSAAVGAGANLPGGDTVAMVPSTAAPASLDVTSLPNDLV
PTSSTPANSFPPSSGSVVSLVTSLTPGSAPPPAGGLADPVAVKPQLQQMLLQPPAPGPQGPPGSMAMPLQVQLQNQLAQQ
GRHLHQHPPPAPPSNQDSAVININSTDDEDEDEEDMEDDGEDFYEGEDYEDFDEDAEELEEEEEDEDGDMPRFNPEGGIE
ELQSSRALFGEERMKVQKVESIGVLEEDDSERMDDPSMPQILCVTGGALEERADSSQEAAQAAGELQDQLTLWDQNRQQN
PPTGALEEEPTAHSQQVGSRPGPPQGHEILQKLTKWLLQHKTESQAFRLKFRPIPQESVAEREQEAGEGEEVAVEAELQE
EEEPVVVQEEEAAAGASGGEETPDAPHDPQQDQTDTQQGYETQAEWTKPEGEKEEESNSEEEKRMKRKREEDDIEDEGGP
SPEKKKQDDEAMASMLADFIACPPDDEDVASGSNQ

> Poecilia Formosa [A0A096M2U1]

MRLTEGLVSVLKEQRPEYLSEVLANYREHGVFPTQGASDVAGLVGFSNAKLSSSKTRFEGLCLLSMLVKDSSSDLFQQHF
VTALSSCCVLLQSQAPCQSIQLAVSILQDLLQYSCQLAELAREVGLNSILGILTSLLGLKTECELAAMEGMRACMVYYPR
ACGSLKDKLGAYFLSKMDSTSRKTQEMACQGYSHLPCLGGLADRSVGTSRAEGWTNQIQCLLASANGILAQIYQGSETDE
AVQYRGPGVELPFPHLDQTDPLLLLQLQHRYTAVCLALKHTLRADPASAVQLPIRPILNLVCRAGFALTSIHFSLTADGS
LRLLVLPSVHISSLEAVCQGRSRVRSGMVQYAAVIQRLFSQTLCAWTALPEANVGQQRAYSSVRVSVYRTLELWLQLVGA
SANVLQGASSHAELLFTHLLADIMPGAESVKVKRVCTLNEEMTKQLVMADPVGASLQRKGDPGSNQDTCLSALKVLRRII
QTSGTLLKNDIHKRLHEVVLPLCVRLQQQTSSIAACESVGGVSGQYSSALTRRQLYRLLLALVLVPSPCWPPPLTCAVS
ILSNGRLDRNLKVSSFCSEALIVCNSLLHPRTPSIALPLPPLALNTLCPVRPRPRPQVDPAHPAGRSRNHLSLVPGLPGQ
GPASAPGDLLLSPHGQPQQDPAGLGLPDGQRPVFVRYDKEEAEDVEISLASDSDDSVVIVPPGMLGADSQLDDPAPGSQN
LLSAAVGAGANLPGGDTVAMVPSTAAPASLDVTSLPNDLVPTSSTPANSFPPSSGSVVSLHPPPAPPSNQDSAVININST
DDEDEDEEDMEDDGSEDFYEGEDYEDFDEDAEELEEEEEDEDGDMPRFNPEGGIEELQSSRALFGEERMKVQKVESIGVL
EEESQAFRLKFRPIPQESVAEREQEAGEGEEVAVEAELQEEEEPVVVQEEEAAAGASGGEETPDAPHDPQQDQTDTQQGY
ETQAEWTKPEGEKEEESNSEEEKRMKRKREEDDIEDEGGPSPEKKKQDDEAMASMLADFIACPPDDEDVASGSN

> Haplochromis burtoni [A0A3Q3CSY8]

MATSAWLHGPSAMRLTEGLVSVLKEQRPEHLPALLAGYREHGVFQTQSACAVGGLVGFSNAKLGSTLSWTRGGSQAPVET
VQLAVNVLKDLLQYSSQLAELAREVGLNSILGILTSLLGLKSECELAAMEGMTACMTYYPRACGSLRDKLAAYFLSKMDS
TDRKTQEMACQCYGRLPCLGGVLDRGVGAGRAEGWTNQIHCLLASANGLLAQLYQGSETDGAMQYQGPGMELAFPHLDPT
DPLLLLQLQHRYTAVCLALKHTLRVDPASAVRLPVRPMLNLVCRALAVSSKSINLTGDGSVRLLVLPIIHSNTLEVLSSV
ITVVRVGMVQYAAVIQRLFSQTLSAWTPLPETSVGQQRAYSAVRVSVYRTLELWVQVAGASSSILQGSPGHSELLFNHLL

GDITPGAESVKVGERCIWTSRRPAPGPPFPARHSLNLGPASLLSSFENHLSLVPGLPGQGPTAGDMILSPHSHHQPDPAG
LGPPDGQRPVFVRFDKEEAEDVEISLASDSDDSVVIVPPGMLNMETQQDDAAANSQSMSSSAPGGTGVTLAGAESVTMVP
TTAAAATIDGVSLPNDLAASSPLLTASTTSINSFPPSSASVVSLVPPLNSSTFTAPPVGLGESLPGRPQLQQMLMQPSTA
SQAGPMGLPLQMHLQNQLSQQGRHLHQQPAAAASSEDSAVININSTDDEEEDDEDIDEDEELEEDEEEEGIDEEDEEEDV
SELADEFFDGEEYEELDEEDGEDLEEEEEEEEEEEEDGDIPPLEGVEDKDGGSGIQEGKLDEERISAFNVEERTEGGIEEI
QTNRALFGEDTMKVQEVESIGVLEAAREGEVEEEDNERMDDPTMPQILCVTGGALEEREEAEEEREQAGAELQKEECSWE
QGAKEDEPQAAEPEQPASQQVDPAAL
> Haplochromis burtoni [A0A3Q2VWM9]
MAGPCAAGAELVPRFLNVLISLFSKTVLSLQSACAVGGLVGFSNAKLGSIETVQLAVNVLKDLLQYSSQLAELAREVGLN
SILGILTSLLGLKSECELAAMEGMTACMTYYPRACGSLRDKLAAYFLSKMDSTDRKTQEMACQCYGRLPCLGGVLDRGVG
AGRAEGWTNQIHCLLASANGLLAQLYQGSETEPVRAVKAFIVSGTRCIPLSFPSIRVDPASAVRLPVRPMLNLVCRALAV
SSKSINLTGDGSVRLLVLPIIHSNTLEVLSSLILVTLWETGDSLSCCTGEDLIMCHQVYSSLVSPPSHSAVRVSVYRTLE
LWVQVAGASSSILQGSPGHSELLFNHLLGDITPGAESVKVGERCIWTSRRPAPGPPFPARHSLNLGPASLLSSFENHLSL
VPGLPGQGPTAGDMILSPHSHHQPDPAGLGPPDGQRPVFVRFDKEEAEDVEISLASDSDDSVVIVPPGAESVTMVPTTAA
AATIDGVSLPNDLAASSPLLTASTTSINSFPPSSASVVSLVPPLNSSTFTAPPVGLGESLPGRPQLHEDSAVININSTDD
EEEDDEDIDEDEELEEDEEEEGIDEEDEEEDVSELADEFFDGEEYEELDEEDGEDLEEEEEEEEEEEEDGDIPPLEGVEDK
DGGSGIQEGKLDEERISAFNVEERTEGGIEEIQTNRALFGEDTMKVQEVERALEEREEAEEEREQAGAELQKEECSWEQG
AKEDEPQAAEPEQPASQQVDPAAFVLNALDNVMRLCSTSNSECK
> Xenopus tropicalis [F7DYK4]
MAAVCVGTRGMEVTITGLLERDLSEGELAETIRGLREHGALRGEGLPAAMSGLLSSCNSRLISAGTRVEGLSLLALAVEE
SPTGVFEQHCVSWLRSILQIIQSQDPPRVVSLAVFVLRSLLAHSSALPELSREISTNHIPGLLTSLLGLRRQCLVPALEG
IRSCFISYPRACGSLRGKLTAFLLSLIDAENQQIQEVACQCYSLLPSLGSGFSQGIKHTENWERQIQSVICSLHTVFLQL
YQGSETDTARYEGSGTELELPPVEDDGTHSVLQLARRFTALAQCMRLLLREQFPAPVRVPVSDVLSLVCRVVNVSPKNLS
WHGEESLKLLLLPRMHSSVLEILEATMIACGPRLLPFSAVICRLFPQLLLSWAAVKGMAGIPSGQERPYSSLRCSVYRVL
ETWVTMCGISSGVLQGPTHHSDILLANLLSDITPPTDAIKMSSFVQLGAKKQKVSEVGGDDFQSHRKRDTTANVELCTAA
LKGLCCVVLHCGSVIKEDVHRRLQELSIPLLLRLQQGPDQWLGPYTSSECRKELYRLLLCLTLTPNPRLPAPLHCAIKIF
RVGISEESLQVSRFSTEALAICRILIHPRVPSLQRPLPHHGPRPPLQSDAPTLRPPAPPPTFPAMPPANHLPPRPTVQVM
ATEPPVSAAVPSPPPEESFGEKPRRAVFIHFDKEEPSDVEISLESDSDDSVVIVPEGLFAKSDSKPEPSPPTVKPTEEAT
EPVAQPAVPSSSTAPPPPPPPPPPAPPVCAGPSSVPVPIAEAPPPPPPQQEVDTVININSSDDEEDGEEDEDEGLYDDEDE
EDYYDEEEDEDLEGLEEDDYEEDEEGITEEEEEDLEEGEEDEEEGEDEECLMPDELPVGCVAEEIPDGTETSSLHEVDPD
EGPPRLSPVQEDEVADTGLLMLVESEDRERSRDSHEESAPETDLARSPPQPPVLTPPPPLEEPPPMEEGDAPQLEVPELE
APPIAPEPEKEIEERKPDEVHVEKPEPEEEEPMADADTMLADFVDCPPDDDKLPEPCT
>Xenopus tropicalis [A0A803KBS8]
MAAVCVGTRGMEVTITGLLERDLSEGELAETIRGLREHGALRGEGLPAAMSGLLSSCNSRLISAGTRVEGLSLLALAVEE
SPTGVFEQHCVSWLRSILQIIQCLVPALEGIRSCFISYPRACGSLRGKLTAFLLSLIDAENQQIQEVACQCYSLLPSLGS
GFSQGIKHTENWERQIQSVICSLHTVFLQLYQGSETDTARYEGSGTELELPPVEDDGTHSVLQLARRFTALAQCMRLLLR
EQFPAPVRVPVSDVLSLVCRVVNVSPKNLSWHGEESLKLLLLPRMHSSVLEILEATMIACGPRLLPFSAVICRLFPQLLL
SWAAVKGMAGIPSGQERPYSSLRCSVYRVLETWVTMCGISSGVLQGPTHHSDILLANLLSDITPPTDAIKMSSFVQLGAK
KQKVSEVGGDDFQSHRKRDTTANVELCTAALKGLCCVVLHCGSVIKEDVHRRLQELSIPLLLRLQQGPDQWLGPYTSSEC
RKELYRLLLCLTLTPNPRLPAPLHCAIKIFRVGISEESLQVSRFSTEALAICRILIHPRVPSLQRPLPHHGPRPPLQSDA
PTLRPPAPPPTFPAMPPANHLPPRPTVQVMATEPPVSAAVPSPPPEESFGEKPRRAVFIHFDKEEPSDVEISLESDSDDS
VVIVPEGLFAKSDSKPEPSPPTVKPTEEATEPVAQPAVPSSSTAPPPPPPPPPPAPPVCAGPSSVPVPIAEAPPPPPPQQE
VDTVININSSDDEEDGEEDEDEGLYDDEDEEDYYDEEEDEDLEGLEEDDYEEDEEGITEEEEEDLEEGEEDEEEGEDEEC
LMPDELPVGCVAEEIPDGTETSSLHEVDPDEGPPRLSPVQEDEVADTGLLMLVESEDRERSRDSHEESAPETDLARSPPQ
PPVLTPPPPLEEPPPMEEGDAPQLEVPELEAPPIAPEPEKEIEERKPDEVHVEKPEPEEEEPMADADTMLADFVDCPPDD
DKLPEPCT
> Dissostichus mawsoni [A0A7J5XKJ0]
MAIQVHIKLLLLLLLLLLLLLQHDFTGPSQFSQFHLGVLGSVQRHLTVADLAAQFALHPVDGALELDQLSVQSSEPLVRH
EQTGNNQQLSENTFYSHTVKLQPVDRRRERCWWSRGFQQCQTGLEQNQGLCLLSMLVKDSSSDLFQQHCVSWLRSLQQVI
QAPVQTIQLAVNILKDLLQYSSQLPEVAREVGLNSILGILTSLLGLKTECELSAMEGMTACMTYYTRACGSLRMACQCYG
RLPSLGGLLDRGVSAGRAEGWTNQIHCLLASANGLLAQIYHGSETDGTVQYEGPGVELAFPHLDQSDPVLLLQLQHRFTA
VCLALKHTLSVRCGMVQYAAVLQRLFSQTLSAWTPLPEASLGQQRAYSSVRVSVYRTLELWVQGSPGHSELLFSHLLGDI
TPGAESIKRLHDVVLPLCVRLQQQQSSSSTSCESAGSVSGQYSSAPSRKELHRLLLALVLVPSPCWPPPLTCAVSILSKG
RNDRNLKVSTFCIEALTICNSLLHPRIPSIALPLPPLTLKPTTNAPVLPTSQAPGLTLPTLLGGPPPAPLSPPATPSAWA
PPPCWNHLSLVPGQTDMILSPHGHHQQDAAGLGLPEGQRPVFVRYDREEAEDVEISLASDSDDSVVIVPPGMLHLETQQD
EAAAAANPEHGQRSRGGHAPSPRVPLHGPHHSSHHHRPGLLPNDLATSSPLLTTSTTPINSFPPSNSSVVSLLSLGCTSR
GLVAGPTAAPADADAAPRRGAAGPHGLPLQMHQLQNQLSQSGRHLHQHQAPRPATKTPPSSTSTAPMRRTKRRRTWRTRR
SWTRRRRALTRRTKMRSERFCRGGYDDYDEEEGEELEEDEEEDGEIPPLEGAEDKAGEAGIEGGKVLRAVVDEGGMAGFS
VEAEAEGGIEEIQTNRAMFGEDRVKVQKVESIGVLEEAREGEGEEDENERMDDPTMPQILCVTGGALEDREEAEEEGGAA
RGGEQEEARTWEQGANEIELKAASEECTANQNQESADEPAQEASVSDSLPSNQQEAQPEGERDPEAAEPATSTGPNTKQQ
EEAEQTEAGGGESDGEEGKGVKRKREEEPGSEKKKMDDDSMASMLADFVACPPDDEDGASGSNRS
> Petromyzon marinus [S4RY41]
RWDGLSLLAALVRDCSTQVFVQHCCAWLRAVTQLLQPYEPEGGLALAVRVLADLLRFSAQLPELAREVSQSHLPALMSAL
LALRDQRECMAMEGLLACMRFYPKTSGTTKHWIAIAVLPQWLDNKPRVTKARRASHYRCRRTCWQRPTVYNAILKYKARH
CLLSLHSGLNALYRPSVRCRRQTDGDEADMALPEPQADSPVYLLLVKQEFSCLARALFAMLREEFAAPVKVPAQPILTLV
CRALAISPKRLAAVGEATMQLLVLPAVHTDALSLLEALIRTRAARCGKRLLRFGDVINRAFPQVLATWSPPAETGGDPGR
ESPYSEVRVRAYGALAAWVDACGATCRVLQDELQHAHDLLRHLVRDASPASDLIRVRPLSPAESP
> Cryptotermes secundus [A0A2J7RT13]
MDGMLSLFETVAHSNCDEVLMQTFLKTCTEHQAFVNKAPVILQSVVANINGKLNSTATRYDGLLLLKTFLPQCPVEIFGK
NVISWMQQCIKSVDGKQNKHALASTSYQVLKTLLQMSQQMPELKRTVSGFVVPKIIDTFQRIAPVEVSLSMLECLGVLMY
NYGPSCGQHKNMLEKCVLQYVDSSDVNVIKRVAHCVAFLPLLGGGGSLGANHVSQWKEEHQKVCATLHYILDELFDGVRE
IQNSHSSVTSDRMLSLPPTSATVPLPRVYQLMNRFMNVSKFLQAMLLSEFPVEKTVLPGEILGIVCRGLAVTSHTMGKKV
SADLLMVGAMLPQIHIALLKVLDSLTTCCGNNLLPYAPIICKLVLQTLKWTSAEKWPYGIEKPYGQLRVAACNTLMLWLQ
TSNCGSCVELISEQLVSGLLQDIWFEKEAVALSVQYISSKKEQKHKRNTSEQQQGVETSRKYIPDQKSNSRTCRAALQVL
QWMLHSAAVFIKPTTHRLLQEKTVGLLFDIQRASGASDRPFPYADTACRLELYRLLHTLVLEPHTTWPPPTQFALHMLSA
GRSDPNLEVSSFCTSALIAAEKLVHPASGTLHFPVSLEEMLESTKGYKREYLTKAKESSSFLEASEEEEANKGVHSRGGS
NESEQGTSNINNAPSSSEEVVIVYEDRMDVEDDGGETSESEDQDISENDVSLDSEESDSSVKNNQSYGLSKILSKTHDQE

ITLVNISESDEEFQHKEERSASSLVIRDKSCSCSSVEVCERDVSETYKSNTDSKIPCTSPVSCNREQSVISSADSREKVA
TSRDVGNKNKSISYQQQKKSNISSASANNRYENIIQASTGKGSKDIATNIKTSAVNKTHSVTENKEERQGDGQVLHVCWK
DDNEPSPKKIKLDTPESVKKYYKLDTETLETSSQSVVKDSTAVPITGNGRQEQPVNGVSQDPKDITEEEMLQAFVDVLSE
> Coregonus sp. 'balchen' [A0A6F9CSM8]
MAATAAWMHGPSNMRLTEGFVSVLKEQRPEYLPALLANYREHGVFSTQSTGAVGGLVGLSNAKLGNSKTKFEGLCLLSVL
VKDSSSDSQAPLPSIQLAVGVLQDLLQYSSQLPELAREVGLNSVLGILTSLLGLKSECHLAAMEGMTACMTFYPRACGSL
RVACECYGRLPCLGGVVERGGGGRRAEGWTRPGVELPYPPLDDTDPLLLLQLQHRYRAACLGLKHTLSVDPASAVRLPVQ
QVLNLVCRALAVSSKSINLTGDGCVRLLVLPAIHKDTLQVLHALITALGSGLVLYSSVLQRLFSQTLAVRVSVYRTLELW
VRVGGASAGVLQGSPTHSELLLAHLLGDITPGADSVRPSTSPQLRDLVSSKPCPKSRKPGLAMGDGGGASLQRKGDSLAN
QDTCLSALRALRQRLHDVVLPLCARLQQQHGATGSDSGAGRVSGQYGSAPPRRELYRLLLALVLVSSFCTEALTICNSLL
HPRTPSLSFPLPPLTLKPTPSQASSLTLLGGPTPGTPFPSRHHLGLGHTALLGSLDNHLPLVVPGLSGQGSTPGDLLLSP
HQGELAGLGLPEGQRPVFIRYDKEEAEDVEISLESDSDDSVVIVPQGMLMLESQDGTSAAATLPGSSLAPGGVTLPVPGP
GGDPGRDISLPLSFSNNLPSTSLPHPLLPSSTINSFPPAPLVSLVPPLNSTSVAQLGEPSVGLGVGADSLPGAQLQQMLL
QGQPPAPGQPTALGLPIQMHQNQLAQSSRALQQQQSIDEDHTVININSSDEEEEDEEMEDEDELGEEEEEEGLEDEEEE
GSDFPDEEEYYDEEEFDDYEAEEGEEMEDEEEEGEIRPLDREGRRGEMGGQEEGEVLRGLAEEGGIGGFRVEREGEGGIE
ELETDRSAYGEEGIKAQEVESIGVLEEEREGEEDDDEGMNDPTMPQILCVTGGALEEREELDREGHGQKVRSWEQKGANR
PEGMSSSEEGPAPQHQQEAGPAPEVRVDGDNQPSNQEGETSKQGGDNSGQEMRSSTAPSETKGEGPEREKGGKEGEEGEA
EEEDGRGMKRKREGEEEEEEGTGQGTEKNKLDEEAMASMLADFVACPPDNVENVPSASNRPS
> Ictidomys tridecemlineatus [A0A287DE52]
MAAAVLSGPSAGSPAGVPGGTGGLSAMNSGPRLRLLLLESVSGLLQPRTGSTIAPVHPPVCSVPHLPGLMCLLRLHGTVG
GAQNLSALGALVSLSNARLGSIKTRFEGLCLLSLLVGESPTEMFQQHCVSWLRSIQQVLQSQDPPPTMELAVAVLRDLLR
YAAQLPTLFRDISINHLPGLLTSLLGLRPECEQSALEGMKACMTYFPRACGSLKGKLASFFLSRVDALSPQLQQLACECY
SRLPSLGAGFSQGLKHTESWEQELHSLLASLHSLLGALYEGAETAPVQNEGPGVETLLSPSEDGDAHVLLRLRQRFSGLA
RCLGLMLSSEFGAPVSVPVQEVLDLICRTLSVSGKNISLLGDGPLRLLLLPSIHLEALDLLSALILACGGRLLRFGALIS
RLLPQVLNAWSIGRDTLSPGQERPYSTIRTKVYAILELWVQVCGASAGVLQGGASGEALLTHLLSDISPPSDALKLRSPR
GSPDGGLQTGKPSAPKKIKLDVGEAMAPPSHRKGENNANSDVCAAALRGLSRTILMCGPLIKEETHRRLHDLILPLVMGV
QQGEVLGSSPYTSSCCRRELYRLLLALLLAPSPRCPPPLACALQAFSLGQREDSLEVSSFCSEALVTCAALTHPRVPPLQ
AMGPTCPTPAPVPPPEAPSPFRAPPFQPPGPMPSVGPMPSAGPMPSAGPLTSSGSMPSVGPMPSAGPMPSAGPIPSAGPV
PSARPGPPATANHLGLSVPGLVSVPPRLLPGPENHRAGSSEDPVLAPSGTPPPTIPPDETFGGRVPRPAFVHYDKEEASD
VEISLESDSDDSVVIVPEGLPPLPPPPPSGTTPPPVAPTGPPTASPPVPAKEEPEELPAAPGPLPPPPPPPVPGPVTLPP
PQLVPEGTPGGGGPPALEEDLTVININSSDEEEEEEEEEEEEEEEEEEEEEEFEEEEEEEEEYFEEEEEEEEEFEEEFEE
EEGELEEEEEEEEEEEEEELEEVEELEFGSAGGEVEEGGPPPPTLPPALPPPESPKEHPEPEPEPGLLLEVEEPGSEEQH
GTETAPTLAPEVLPSQGEEEREGRSPEAGPPPQELVEEEPSAPPTLLEEGTEGGGDNVPPPPETAAEEEMETETEAAVLQ
EKEQDDTAAMLADFIDCPPDDEKPPPTTEPDT
> Molossus molossus [A0A7J8D0Y0]
MAAAVLSGPSAGSAAGVPGGAGGLSAVGSGPRLRMLLLESVSALLQPRAGSTVPPVHPPVRSAPHLPGLMCLLRLHGTVG
GAQNLSAVGALVGLSNARLGSIKTRFEGLCLLSLLVGESSTEMFQQHCVSWLRSIQQVLQSQDPPPTMDLAVAVLRDLLR
YAAQLPTLFRDISMNHLPGLLTSLLGLRPECELSALEGMKACMTYFPRACGSLKGKLASFFLSRVDALSPQLQQLACECY
SRLPSLGAGFSQGLKHTESWEQELHSLLASLHSLLGALYEGAEPAPVQYEGPGVEMLFSPSEDGDAHNLLRLWQRFSGLA
CCLGLMLSSEFGAPVSIPVQEILDVICRTLSISAKNISLLGDGPLRLLLLPSIHLEALDLLSALILACGGRLLRFGALIS
RLLPQVLNAWSIGRDTLSLGQEKPYSAMRTKVYAVLELWVQVCGASAGVLQGGASGEALLTHLLSDISPPADALKLRSPR
GSPDGSLQTGKPSAPKKLKLDMGEAMAPPSHRKGDSNANSDVCAAALRGLSRTILMCGPLIKEETHRRLHDLVLPLVMGV
QQGEILGSSPYTSSRCRRELYRLLLALLLAPSPRCPPPLACALQAFSLGQREDSLEVSSFCSEALVTCAALTHPRVPPLQ
SMGPTCATPAPVPPPEAPSPFRAPPFHPPGPMPSAGTMPSAGTMPSAGPVPSAGTMPSAGPLPPARPGPPATANHLGLSV
PGLVSVPPRLLPGPENHRAGSNEDPVLAPSGTPPPTIPPDETFGGRVPRPAFVHYDKEEASDVEISLESDSDDSVVIVPE
GLPPLPLPPPSGTTPPPVTPAGPPAVSPPLPAKEEPEELPAVPGPLPPPPPPPVPGPVTLQPPQLVPEGAPGGGGPPALE
EDLTVININSSDEEEEEEEEEEEEEEEEEEDFEEEEEDEEEYFEEEEEEEEEFEEEFEEEEGELEEEEEEEEDEEEEEEL
EELEFGPAGGEVEEGGPPPTSLPPALPPTESPKVQPEPEPEPGLLLEVEEPGAEEEHGAETAPTLAPEVLPSQEELEKEG
GSPPAGPPPQELVEEEPCAPPALLEEGTEGGGDEVPPPPEAPAAEEMDMETETETPALQEKEQDDTAAMLADFIDCPPDD
EKPPPDTEPDS
>Myotis myotis [A0A7J7T786]
MAAAVLSGPSAGSAAGVPGGAGGLSAVGSGPRLRMLLLESVSGLLQPRAGSTIPPVHPPVRSAPHLPGLMCLLRLHGTVG
GAQNLSAVGALVGLSNARLSSIKTRFEGLCLLSLLVGESSTEMFQQHCVSWLRSIQQVLQSQDPPATMDLAVVVLRDLLR
YAAQLPTVFRDISMNHLPGLLTSLLGLRPECELSALEGMKACMTYFPRACGSLKGKLASFFLSRVDSLSPQLQQLACECY
SRLPSLGAGFSQGLKHTESWEQELHSLLASLHSLLGALYEGAEPAPVQYEGPGVEMLFSLEDADAHNLFRLRQRFSGLAC
CLGLMLSSEFVAPVSIPVQEILDFICRTLSISAKNISLLGDGPLRLLLLPSIHLEALDLLSALILACGGRLLRFGALISR
LLPQVLNAWSIGRDTVSLGQEKPYSAMRTKVYAVLELWVQVCGASAGVLQAGASGEALLTHLLSDISPPAEALKLRSPRG
SPDGSLQTGKPSAPKKLKLDMGEAATPPSHRKGDSNANSDVCAAALRGLSRTILMCGPLIKEETHRRLHDLVLPLVMGVQ
QGEVLGSSPYTSSRCRRELYRLLLALLLAPSPRCPPPLACALQAFSLGQREDNLEVSSFCSEALVTCAALTHPRVPPLQS
MGPTCPTPAPVPPPEAPSPFRAPPFHPPGPMPSAGTMPSAGPVPSVGPLPSAGPVPPAGPVPPARPGPPATANHLGLSVP
GLVSVPPRLLPGPENHRAGSNEDPILASSGTPPPVIPPDETFGGRVPRPAFVHYDKEEASDVEISLESDSDDSVVIVPEG
LPPLPLPPPSGSTPPPVAPAGPPAASPPLPAKEEPEELPTVPGPLPPPPPPPVPGPVTLQPPQLVPEGTPSGGGPPALEE
DLTVININSSDEEEEEEEEEEEEEEEEEEEDFEEEEEDEEEYFEEEEEEEEEFEEEFEEEEGELEEEEEEEEEEEEEELE
EVEELEFGSAGGEVEEGGPPPTSLPPALPPPESPKVQPEPEPEPGLLLEVEEPGAEEERGAETAPTLAPEVLPSQEELEK
EGGSPPAGPPPQELVEEEPSAPPPALLDEGTEGGGDEVPPPPETSAAEEMETETAPPQEKEQDDTAAMLADFIDCPPDDE
KPPPVTEPDS
>Myotis lucifugus [G1NY24]
CSLPPEPGAQSPGIPGAGPQPPSAPLHRRASRCVCVIAPRHATTRARGKMAAAVLSGPSAGSAAAVPGGAGGLSAVGSGP
RLRMLLLESVSGLLQPRAGSTIPPVHPPVRSAPHLPGLMCLLRLHGTVGGAQNLSAVGALVGLSNARLSSIKTRFEGLCL
LSLLVGESSTEMFQQHCVSWLRSIQQVLQSQDPPATMDLAVVVLRDLLRYAAQLPTVFRDISMNHLPGLLTSLLGLRPEC
ELSALEGMKACMTYFPRACGSLKGKLASFFLSRVDSLSPQLQQLACECYSRLPSLGAGFSQGLKHTESWEQELHTLLASL
HSLLGALYEGAEPAPVQYEGPGVEMLFSLEDADAHNLFRLRQRFSGLACCLGLMLSSEFVAPVSIPVQEILDFICRTLSI
SAKNISLLGDGPLRLLLLPSIHLEALDLLSALILACGGRLLRFGALISRLLPQVLNAWSIGRDTVSLGQEKPYSAMRTKV
YAVLELWVQVCGASAGVLQAGASGEALLTHLLSDISPPAEALKLRSPRGSPDGGLQTGKPSAPKKLKLDMGEAATPPSHR
KGDSNANSDVCAAALRGLSRTILMCGPLIKEETHRRLHDLVLPLVMGVQQGEVLGSSPYTSSRCRRELYRLLLALLLAPS
PRCPPPLACALQAFSLGQREDNLEVSSFCSEALVTCAALTHPRVPPLQSMGPTCPTPAPVPPPEAPSPFRAPPFHPPGPM
PSAGTMPSAGPVPSVGPLPSAGPVPPAGPVPPARPGPPATANHLGLSVPGLVSVPPRLLPGPENHRAGSNEDPILASSGT

PPPVIPPDETFGGRVPRPAFVHYDKEEASDVEISLESDSDDSVVIVPEGLPPLPLPPPSGSTPPPVAPAGPPAASPPLPA
KEEPEELPTVPGPLPPPPPPPVPGPVTLQPPQLVPEGTPSGGGPPALEEDLTVININSSDEEEEEEEEEEEEEEEEEEEE
DFEEEEEDEEEYFEEEEEEEFEEEFEEEEGELEEEEEEEEEEEEEEELEEVEELEFGSAGGEVEEGGPPPTSLPPALPPP
ESPKVQPEPEPEPGLLLEVEEPGAEEERGAETAPTLAPEVLPSQEELEKEGGSPPAGPPPQELVEEEPCAPPPALLDEGT
EGGGDEVPPPPETSAAEEMETETAPPQEKEQDDTAAMLADFIDCPPDDEKPPPVTEPDS
>Felis catus [M3XBH8]
MPRAACIRVCRARTAVPDSRREAGSQDAGRAESGGAASPGHSPRRPGTLAPFVPTTWSAPRARRPAVRACSLAARAASPT
SARPARGDAAPSSRPLPTRRPSRCVCVIAPRHHGARVKMAAAVLSGPSAGSAAGVPGGTGGLSAVGSGPRLRLLLLESVS
GLLQPRAGSAVAPVHPPVRSAAHLPGLMCLLRLHGTVGGAQNLSAVGALVGLSNARLGSVKTRFEGLCLLSLLVGESPTE
LFQQHCVSWLRSIQQVLQSQDPPPTMELAVAVLRDLLRYAAQLPTLFRDISTNHLPGLLTSLLGLRPECELSAMEGMKAC
MTYFPRACGSLKGKLASFFLSRVDALSPQLQQLACECYARLPSLGAGFSQGLKHTESWEQELHSLLASLHGLLGALYEGA
DTAPVQCEGPGLDVLLAPSEDGDAHTLLRLRHRFSGLARCLGLLLSSEFGAPVSVPVQEILDIICRTLSISAKNISLLGD
GPLRLLLLPSIHLDALDLLSALILACGSRLLRFGALISRLLPQVLNAWNLGRDALPPGQERPYSAVRTKVYAVLDLWVQV
CGASAGVLQGGASGEALLSHLLSDISPPADALKLRSPRGSPDGGLQSGKPSAPKKLKLDMGEATAPPGHRKGDSNANSDV
CAAALRGLSRTVLMCGPLIKEETHRRLHDLVLPLVMGVQQGEVLGSSPYTSSRCRRELYRLLLALLLAPSPRCPPPLACA
LQAFSLGQREDSLEVSSFCSEALVTCAALTHPRVPPLQSMGPACPAPAPAPPPEAPSPFRAPPFHPPGPMPSVGPMPSVG
PMPSVGPMPPAGPMPPTRPGPPATANHLGLSVPGLVSVPPRLLPGPENHRAGSNDDPVLAPSGTPPPAVPPDETFGGRVP
RPAFVHYDKEEASDVEISLESDSDDSVVIVPEGLPPLPPPPPTGSTPPPAAPAGPPTASPPVPAKEEPEELPAAPGPLPP
PPPPPVPGPVALPPPQLVPEGPPGGGTPALEEDLTVININSSDEEEEEEEEEEEEEEEEEEEEEEDFEEEEEEEEEYFEE
EEEEEEEFEEEFEEEEGELEEEEEDEDEEEEDELEELEEVEFGPAGGPAEEGGPPPPSPAPALPPAQPPEVPPEPGVEPG
LLLEVEEPGPEDEPGAEAAPTLAPEVLPSQGEGQREAGSPPAGPPPQELVEEEPSAPPPLLEEGTENGGDKVPPPPETPA
AEEMEAAAEAETAALQEKEQDDTAAMLADFIDCPPDDEKPPAAPEPES
>Equus caballus [F6TIQ3]
MAAAVLSGPSAGSAAGAAGGTGGLSAVASGPRLRLLLLESVSGLLQPRAGSTVAPVHPHVRSAAHLPGLMCLLRLHGTVG
GAQNLSAVGALVGLSNARLGSIKTRFEGLCLLSLLVGESPTEMFQQHCVSWLRSIQQVLQSQDSPPTMELAVAVLRDLLR
YAAQLPTLFRDISMNHLPGLLTSLLGLRPECELSALEGMKACMTYFPRACGSLKLACECYSRLPSLGAGFSQGLKHTESW
EQELHSLLASLHSLLGALYEGAETAPMQYEGPGVEVLLSPSEDGDAHALLRLRQRFSGLARCLGLMLSSEFGAPVSVPVQ
EILDVICRTLSISAKNISLLGDGPLRLLLLPSIHLEALDLLSALILACGGRLLRFGALISRLLPQVLNAWSIGRDTLSPG
QERPYSTMRTKVYAVLELWVQVCGASAGVLQGGASGEALLTHLLSDISPPADALKLRSPRGSPDGGLQSGKPSAPKKLKL
DVGEAMAPPSHRKGESNANSDVCAAALRGLSRTILMCGPLIKEETHRRLHDLVLPLVMGVQQGEVLGSSPYTSSRCRREL
YRLLLALLLAPSPRCPPPLACALQAFSLGQREDSLEVSSFCSEALVTCAALTHPRVPPLQSMGPTCPTSAPVPPPEAPSP
FRAAPFHPPGPMPSAGPMPSAGPVPSAGPMPSVGPLPSVGPMPSVGPMPPARPGPPATANHLGLSVPGLVSVPPRLLPGP
ENHRAASNEDPVLAPSGTPPPAIPPDETFGGRMPRPAFVHYDKEEASDVEISLESDSDDSVVIVPEGLPSLPPPPPSGTT
PPPVAPAGPPTASPPVPAKEEPEELPAAPGPLPPPPPPPVPGPVTLPPPQLVPEGTPGGGGPPALEEDLTVININSSDEE
EEEEEEEEEEEEEEEEEEEDFEEEEEDEEEYFEEEEEEEEEFEEEFEEEEGELEEEEEEEDEEEEEELEEVEELEFGSAG
GEVDEAGPPPPSLPPALPPAESPKVQPEPEPEPGLLLEVEEPGVEEERGAETAPTLAPEVLPSQGEVEREEGSPPAGPPP
QELVEEEPSAPPTLLEEGTEGGDDKVPPLPEPPAAEEMETETEVAALQEKEQDDTAAMLADFIDCPPDEEKPPSATEPDS
> Strongylocentrotus purpuratus [A0A7M7NL82]
MLAGAVEMEHRLIENASKLLQQNVHQATSKQASKLLARQSTENSVDNLIFSGKRSGTLHDWISQVNVNLSTSASRLEGLV
VLRAIVQQCSQETFSECCVTWVRLLINIFQPHQTPSIVEITSGILVDILRIASRSNEISRDLAINHIPGLLGTLLNAESE
EWLVPSLRLLSVCMECFPGPCGAISYKLEAMVTPHLDSTYPDIQKLSQRCYPLLARLGGGGSGGIKYTEAFKHYTMTILY
TLQTQLAVLYADCSTEPEPTPEEGIEPISLSLAPTEEPMRTLHLTNQLKSLSNCLQIMLRNEFPAKASIPVKSTLEYISS
ALEVTGKTLGDHVTSESSMLAIVLPRIHQTTLQLLKALIESCCMNHYNHQINQIFLRMLGGTHSQDQVKPFRNLRAEIYR
TIVMWLKTAGVSSGFATLVGEKLIHQCIQDATPPTSATKLASGVQAKSDKKGGKVKVKGQSAHITDLSSGGQQTDQYFDR
VLCLTALQALRQLMLTSGVKLKANLHQDVYKFVIPLLTKIQSCDTSNMPSPYTCCDCRRELYGILLAGVLVPHPQAPSPL
NLAVAIFKEGKNDKSIEVSSACCEAAVICDVIIHPRRPPIPCAKPQVPLTSMSLQAPLTSALSLSQTPEGQVVLVMETED
SGNATLSSAKKDSVLTQCEVHIVTLSPNANVQAASDELSHGSSQDQDTSHTVHDSGSTDEAGVFKSSAARKDDDEEEEEE
EEEEKDDGKESDEGNEMEGMKAETMEESPGSSSRKRKHNTRANQQAKKPATEEVKSMLSEFIDAPPDSE
>Ictidomys tridecemlineatus [I3MPV5]
MAAAVLSGPSAGSPAGVPGGTGGLSAMNSGPRLRLLLLESVSGLLQPRTGSTIAPVHPPVCSVPHLPGLMCLLRLHGTVG
GAQNLSALGALVSLSNARLGSIKTRFEGLCLLSLLVGESPTEMFQQHCVSWLRSIQQVLQSQDPPPTMELAVAVLRDLLR
YAAQLPTLFRDISINHLPGLLTSLLGLRPECEQSALEGMKACMTYFPRACGSLKGKLASFFLSRVDALSPQLQQLACECY
SRLPSLGAGFSQGLKHTESWEQELHSLLASLHSLLGALYEGAETAPVQNEGPGVETLLSPSEDGDAHVLLRLRQRFSGLA
RCLGLMLSSEFGAPVSVPVQEVLDLICRTLSVSGKNISLLGDGPLRLLLLPSIHLEALDLLSALILACGGRLLRFGALIS
RLLPQVLNAWSIGRDTLSPGQERPYSTIRTKVYAILELWVQVCGASAGVLQGGASGEALLTHLLSDISPPSDALKLRSPR
GSPDGGLQTGKPSAPKKIKLDVGEAMAPPSHRKGENNANSDVCAAALRGLSRTILMCGPLIKEETHRRLHDLILPLVMGV
QQGEVLGSSPYTSSCCRRELYRLLLALLLAPSPRCPPPLACALQAFSLGQREDSLEVSSFCSEALVTCAALTHPRVPPLQ
AMGPTCPTPAPVPPPEAPSPFRAPPFQPPGPMPSVGSMPSVGPMPSAGPMPSAGPIPSAGPVPSARPGPPATANHLGLSV
PGLVSVPPRLLPGPENHRAGSSEDPVLAPSGTPPPTIPPDETFGGRVPRPAFVHYDKEEASDVEISLESDSDDSVVIVPE
GLPPLPPPPPSGTTPPPVAPTGPPTASPPVPAKEEPEELPAAPGPLPPPPPPPVPGPVTLPPPQLVPEGTPGGGGPPALE
EDLTVININSSDEEEEEEEEEEEEEEEEEEEEEEFEEEEEEEEYFEEEEEEEEEEFEEEFEEEEGELEEEEEEEEEEEEE
ELEEVEELEFGSAGGEVEEGGPPPPTLPPALPPPESPKEHPEPEPEPGLLLEVEEPGSEEQHGTETAPTLAPEVLPSQGE
EEREGRSPEAGPPPQELVEEEPSAPPTLLEEGTEGGGDNVPPPPETAAEEEMETETEAAVLQEKEQDDTAAMLADFIDCP
PDDEKPPPTTEPDT
>Gorilla gorilla gorilla [G3QQ30]
MLLPSLSPRVQPAFTQCARPQPPPHARSPVGGRRGVFASSRHATTGTRGKMAAAVLSGPSAGSAAGVPGGTGGLSAVSSG
PRLRLLLLESVSGLLQPRTGSAVAPVHPPNRSAPHLPGLMCLLRLHGSVGGAQNLSALGALVSLSNARLSSIKTRFEGLC
LLSLLVGESPTELFQQHCVSWLRSIQQVLQTQDPPATMELAVAVLRDLLRYAAQLPALFRDISMNHLPGLLTSLLGLRPE
CEQSALEGMKACMTYFPRACGSLKGKLASFFLSRVDALSPQLQQLACECYSRLPSLGAGFSQGLKHTESWEQELHSLLAS
LHTLLGALYEGAETAPVQNEGPGVEMLLSSEDGDAHVLLQLRQRFSGLACCLGLMLSSEFGAPVSVPVQEILDFICRTLS
VSSKNISLHGDGPLRLLLLPSIHLEALDLLSALILACGSRLLRFGILISRLLPQVLNSWSIGRDSLSPGQERPYSTVRTK
VYAILELWVQVCGASAGMLQGGASGEALLTHLLSDISPPADALKLRSPRGSPDGSLQTGKPSAPKKLKLDVGEAMAPPSH
RKGDSNANSDVCAAALRGLSRTILMCGPLIKEETHRRLHDLVLPLVMGVQQGEVLGSSPYTSSRCRRELYCLLLALLLAP
SPRCPPPLACALQAFSLGQREDSLEVSSFCSEALVTCAALTHPRVPPLQPMGPTCPTPAPVPPPEAPSPFRAPPFHPPGP
MPSVGSMPSAGPMPSAGPMPSAGPVPSARPGPPTTANHLGLSVPGLVSVPPRLLPGPENHRAGSNEDPILAPSGTPPPTI
PPDETFGGRVPRPAFVHYDKEEASDVEISLESDSDDSVVIVPEGLPPLPPPPPSGATPPPIAPTGPPTASPPVPAKEEPE
ELPAAPGPLPPPPPPPPPPVPGPVTLPPPQLVPEGTPGGGGPPALEEDLTVININSSDEEEEEEEEEEEEEEEEEEEEDF

EEEEEDEEEYFEEEEEEEEEFEEEFEEEEGELEEEEEEEDEEEEEELEEVEDLEFGTAGGEVEEGAPPPPTLPPALPPPE
SPPKVQPEPEPEPGLLLEVEEPGTEEERGADTAPTLAPEALPSQGEVEREGESPAAGPPPQELVEEEPSAPPTLLEEETE
DGSDKVQPPPETPAEEEMETETEAEALQEKEQDDTAAMLADFIDCPPDDEKPPPPTEPDS
> Homo sapiens [Q8IZL8]
MAAAVLSGPSAGSAAGVPGGTGGLSAVSSGPRLRLLLLESVSGLLQPRTGSAVAPVHPPNRSAPHLPGLMCLLRLHGSVG
GAQNLSALGALVSLSNARLSSIKTRFEGLCLLSLLVGESPTELFQQHCVSWLRSIQQVLQTQDPPATMELAVAVLRDLLR
YAAQLPALFRDISMNHLPGLLTSLLGLRPECEQSALEGMKACMTYFPRACGSLKGKLASFFLSRVDALSPQLQQLACECY
SRLPSLGAGFSQGLKHTESWEQELHSLLASLHTLLGALYEGAETAPVQNEGPGVEMLLSSEDGDAHVLLQLRQRFSGLAR
CLGLMLSSEFGAPVSVPVQEILDFICRTLSVSSKNISLHGDGPLRLLLLPSIHLEALDLLSALILACGSRLLRFGILIGR
LLPQVLNSWSIGRDSLSPGQERPYSTVRTKVYAILELWVQVCGASAGMLQGGASGEALLTHLLSDISPPADALKLRSPRG
SPDGSLQTGKPSAPKKLKLDVGEAMAPPSHRKGDSNANSDVCAAALRGLSRTILMCGPLIKEETHRRLHDLVLPLVMGVQ
QGEVLGSSPYTSSRCRRELYCLLLALLLAPSPRCPPPLACALQAFSLGQREDSLEVSSFCSEALVTCAALTHPRVPPLQP
MGPTCPTPAPVPPPEAPSPFRAPPFHPPGPMPSVGSMPSAGPMPSAGPMPSAGPVPSARPGPPTTANHLGLSVPGLVSVP
PRLLPGPENHRAGSNEDPILAPSGTPPPTIPPDETFGGRVPRPAFVHYDKEEASDVEISLESDSDDSVVIVPEGLPPLPP
PPPSGATPPPIAPTGPPTASPPVPAKEEPEELPAAPGPLPPPPPPPPPVPGPVTLPPPQLVPEGTPGGGGPPALEEDLTV
ININSSDEEEEEEEEEEEEEEEEEEEEEDFEEEEEDEEEYFEEEEEEEEEFEEEFEEEEGELEEEEEEEDEEEEEELEEV
EDLEFGTAGGEVEEGAPPPPTLPPALPPPESPPKVQPEPEPEPGLLLEVEEPGTEEERGADTAPTLAPEALPSQGEVERE
GESPAAGPPPQELVEEEPSAPPTLLEEETEDGSDKVQPPPETPAEEEMETETEAEALQEKEQDDTAAMLADFIDCPPDDE
KPPPPTEPDS
> Macaca mulatta [Q1W1Y5]
MAAAVLSGSSAGSAAGVPGGTGGLSAVNSGPRLRLLLLESVSGLLQPRTGSAVAPVHPPNRSAPHLPGLMCLLRLHGSVG
GAQNLSALGALVSLSNARLSSIKTRFEGLCLLSLLVGESPTELFQQHCVSWLRSIQQVLQTQDPPATMELAVAVLRDLLR
YAAQLPALFRDISMNHLPGLLTSLLGLRPECEQSALEGMKACMTYFPRACGSLKGKLASFFLSRVDALSPQLQQLACECY
SRLPSLGAGFSQGLKHTESWEQELHSLLASLHTLLGALYEGAETAPVQNEGPGVEMLLSSEDGDAHVLLRLRQRFSGLAR
CLGLMLSSEFGAPVSVPVQEILDFICRTLSVSSKNISLHGDGPLRLLLLPSIHLEALDLLSALILACGSRLLRFGILISR
LLPQVLNSWSIGRDSLSPGQERPYSTVRTKVYAGLELWVQVCGASAGMLQGGASGEALLTHLLSDISPPADALKLRSPRG
SPDGSLQTGKPSAPKKLKLDVAEAMAPPSHRKGDSNANSDVCAAALKGLSRTILMCGPLIKEETHRRLHDLVLPLVMGVQ
QGEVLGSSPYTSSRCRRELYCLLLALLLAPSPRCPPPLACALQAFSLGQREDSLEVSSFCSEALVTCAALTHPRVPPLQP
MGPTCPTPAPVPPPEAPSPFRAPPFHPPGPMPSVGSMPSAGPMPSAGPMPSAGPVPSTRLGPPTTANHLGLSVSGLVSVP
PRLLPGPENHRSGSNEDPILAPSGTPPPTIPPDETFGGRVPRPAFVHYDKEEASDVEISLESDSDDSVVIVPEGLPPLPP
PPPSGATPPPIAPTGPPTASPPVPAKEEPEELPAAPGPLPPPPPPPPPVPGPVTLPPPQLVPEGTPGGVGPPALEEDLTV
ININSSDEEEEEEEEEGEEEEEEEEEEEDFEEEEEDEEEYFEEEEEEEEEFEEEFEEEEGELEEEEEEEDEEEEEELEEV
EELEFGTAGGEVEEGGPPPPTLPPALPPPESPPKVQPEPEPEPGLLLEVEEPGAEEEHGADTAPTLAPEALPSQGEVERE
EGSPAAGPPPQELVEEEPSAPPTLLEEETEDGGDRVQPPPETPAEEEMETETEAEALQEKEQDDTAAMLADFIDCPPDDE
KPPPPTEPDS
> Macaca mulatta [F7EPR1]
MEIVRTLAPFVRTTCTLNITGPPRTRSPAITPGTQHPGASAPRLLSPRMLLPSLSPRVQPAFIQCAGPQPPPHAHSPVGG
RRGVFTSSRHATTGARGKMAAAVLSGSSAGSAAGVPGGTGGLSAVNSGPRLRLLLLESVSGLLQPRTGSAVAPVHPPNRS
APHLPGLMCLLRLHGSVGGAQNLSALGALVSLSNARLSSIKTRFEGLCLLSLLVGESPTELFQQHCVSWLRSIQQVLQTQ
DPPATMELAVAVLRDLLRYAAQLPALFRDISMNHLPGLLTSLLGLRPECEQSALEGMKACMTYFPRACGSLKLACECYSR
LPSLGAGFSQGLKHTESWEQELHSLLASLHTLLGALYEGAETAPVQNEGPGVEMLLSSEDGDAHVLLRLRQRFSGLARCL
GLMLSSEFGAPVSVPVQEILDFICRTLSVSSKNISLHGDGPLRLLLLPSIHLEALDLLSALILACGSRLLRFGILISRLL
PQVLNSWSIGRDSLSPGQERPYSTVRTKVYAVLELWVQVCGASAGMLQGGASGEALLTHLLSDISPPADALKLRSPRGSP
DGSLQTGKPSAPKKLKLDVGEAMAPPSHRKGDSNANSDVCAAALKGLSRTILMCGPLIKEETHRRLHDLVLPLVMGVQQG
EVLGSSPYTSSRCRRELYCLLLALLLAPSPRCPPPLACALQAFSLGQREDSLEVSSFCSEALVTCAALTHPRVPPLQPMG
PTCPTPAPVPPPEAPSPFRAPPFHPPGPMPSVGPMPSAGPMPSAGPMPSAGPVPSARPGPPTTANHLGLSVSGLVSVPPR
LLPGPENHRSGSNEDPILAPSGTPPPTIPPDETFGGRVPRPAFVHYDKEEASDVEISLESDSDDSVVIVPEGLPPLPPPP
PSGATPPPIAPTGPPTASPPVPAKEEPEELPAAPGPLPPPPPPPPPVPGPVTLPPPQLVPEGTPGGGGPPALEEDLTVIN
INSSDEEEEEEEEEEEEEEEEEEEEEDFEEEEEDEEEYFEEEEEEEEEFEEEFEEEEGELEEEEEEEDEEEEEELEEVEE
LEFGTAGGEVEEGGPPPPTLPPALPPPESPPKVQPEPEPEPGLLLEVEEPGAEEEHGADTAPTLAPEVLPSQGEVEREEG
SPSAGPPPQELVEEEPSAPPTLLEEGTEDGGDRVQPPPETPAEEEMETETEAEALQEKEQDDTAAMLADFIDCPPDDEKP
PPPTEPDS
> Macaca mulatta [F6QQ11]
MTLLFLRRFEGLCLLSLLVGESPTELFQQHCVSWLRSIQQVLQTQDPPATMELAVAVLRDLLRYAAQLPALFRDISMNHL
PGLLTSLLGLRPECEQSALEGMKACMTYFPRACGSLKGKLASFFLSRVDALSPQLQQLACECYSRLPSLGAGFSQGLKHT
ESWEQELHSLLASLHTLLGALYEGAETAPVQNEGPGVEMLLSSEDGDAHVLLRLRQRFSGLARCLGLMLSSEFGAPVSVP
VQEILDFICRTLSVSSKNISLHGDGPLRLLLLPSIHLEALDLLSALILACGSRLLRFGILISRLLPQVLNSWSIGRDSLS
PGQERPYSTVRTKVYAVLELWVQVCGASAGMLQGGASGEALLTHLLSDISPPADALKLRSPRGSPDGSLQTGKPSAPKKL
KLDVGEAMAPPSHRKGDSNANSDVCAAALKGLSRTILMCGPLIKEETHRRLHDLVLPLVMGVQQGEVLGSSPYTSSRCRR
ELYCLLLALLLAPSPRCPPPLACALQAFSLGQREDSLEVSSFCSEALVTCAALTHPRVPPLQPMGPTCPTPAPVPPPEAP
SPFRAPPFHPPGPMPSVGPMPSAGPMPSAGPMPSAGPVPSARPGPPTTANHLGLSVSGLVSVPPRLLPGPENHRSGSNED
PILAPSGTPPPTIPPDETFGGRVPRPAFVHYDKEEASDVEISLESDSDDSVVIVPEGLPPLPPPPPSGATPPPIAPTGPP
TASPPVPAKEEPEELPAAPGPLPPPPPPPPPVPGPVTLPPPQLVPEGTPGGGGPPALEEDLTVININSSDEEEEEEEEEE
EEEEEEEEEEEDFEEEEEDEEEYFEEEEEEEEEFEEEFEEEEGELEEEEEEEDEEEEEELEEVEELEFGTAGGEVEEGGP
PPPTLPPALPPPESPPKVQPEPEPEPGLLLEVEEPGAEEEHGADTAPTLAPEVLPSQGEVEREEGSPSAGPPPQELVEEE
PSAPPTLLEEGTEDGGDRVQPPPETPAEEEMETETEAEALQEKEQDDTAAMLADFIDCPPDDEKPPPPTEPDS
>Macaca mulatta [F6QPZ9]
MEIVRTLAPFVRTTCTLNITGPPRTRSPAITPGTQHPGASAPRLLSPRMLLPSLSPRVQPAFIQCAGPQPPPHAHSPVGG
RRGVFTSSRHATTGARGKMAAAVLSGSSAGSAAGVPGGTGGLSAVNSGPRLRLLLLESVSGLLQPRTGSAVAPVHPPNRS
APHLPGLMCLLRLHGSVGGAQNLSALGALVSLSNARLSSIKTRFEGLCLLSLLVGESPTELFQQHCVSWLRSIQQVLQGK
LASFFLSRVDALSPQLQQLACECYSRLPSLGAGFSQGLKHTESWEQELHSLLASLHTLLGALYEGAETAPVQNEGPGVEM
LLSSEDGDAHVLLRLRQRFSGLARCLGLMLSSEFGAPVSVPVQEILDFICRTLSVSSKNISLHGDGPLRLLLLPSIHLEA
LDLLSALILACGSRLLRFGILISRLLPQVLNSWSIGRDSLSPGQERPYSTVRTKVYAVLELWVQVCGASAGMLQGGASGE
ALLTHLLSDISPPADALKLRSPRGSPDGSLQTGKPSAPKKLKLDVGEAMAPPSHRKGDSNANSDVCAAALKGLSRTILMC
GPLIKEETHRRLHDLVLPLVMGVQQGEVLGSSPYTSSRCRRELYCLLLALLLAPSPRCPPPLACALQAFSLGQREDSLEV
SSFCSEALVTCAALTHPRVPPLQPMGPTCPTPAPVPPPEAPSPFRAPPFHPPGPMPSVGPMPSAGPMPSAGPMPSAGPVP
SARPGPPTTANHLGLSVSGLVSVPPRLLPGPENHRSGSNEDPILAPSGTPPPTIPPDETFGGRVPRPAFVHYDKEEASDV

EISLESDSDDSVVIVPEGLPPLPPPPPSGATPPPIAPTGPPTASPPVPAKEEPEELPAAPGPLPPPPPPPPPVPGPVTLP
PPQLVPEGTPGGGGPPALEEDLTVININSSDEEEEEEEEEEEEEEEEEEEEDFEEEEEDEEEYFEEEEEEEEEEFEEEFE
EEEGELEEEEEEDEEEEEELEEVEELEFGTAGGEVEEGGPPPPTLPPALPPPESPPKVQPEPEPEPGLLLEVEEPGAEE
EHGADTAPTLAPEVLPSQGEVEREEGSPSAGPPPQELVEEEPSAPPTLLEEGTEDGGDRVQPPPETPAEEEMETETEAEA
LQEKEQDDTAAMLADFIDCPPDDEKPPPPTEPDS
>Octodon degus [A0A6P3FIP0]
MAAAVLSGPSAGSPAGVPGGGGGLSAMSSGPRLRLLLLESVSGLLQPRTGSSVAPVHPPVRSAPHLPGLMCLLRLHGTVG
GAQNLSAVGALVNLSNACLGSMKTRFEGLCLLSLLVGESPTDMFQQHCVSWLRSIQQVLQSQDPPPTMELAVAVLKELLR
YAAQLPTLSRDISVNHLPGLLTSLLGLRPECEQSALEGMKACMTYFPRACGSLKGKLASFFLSRVHSLSPQLQQLACECY
SRLPSLGAGFSQGLKHTESWEQELHSLLASLHSLLGALYEGTETAPVQSEGPGVEMLLSHSEEGDAHVLLRLQQRFSGLA
RCLGLMLSSEFEAPVSVPVQEVLDLICRILSVSGKNISLLGDGPLRLLLLPSIHLEALDLLSALILACGGRLLRFGALIS
RLLPQVLNAWSVGRDPFSSGQERPYSTIRTKVYAVLELWVQVCGASAGVLQGGVSGEALLTHLLSDISPPADALKLRSPR
GSPDGGLQTGKPSAPKKLKLDVGEAVAPPSQRKGDSNANSDVCAAALRGLSRSILMCGPLIKEETHRRLHDLVLPLAMGV
QQGEVLGSSPYSSSCCRRELYRLLLALLLAPSPRCPPPLSCALQAFSLGQREDSLEVSSFCSEALVTCAALTHPRVPPLQ
TMGPTCPTPASVPPPEAPSPFRAPSFHPPGPMPSLGPMPSVGPMPSTGPLSAAGPLSSVGPMPSAGPMPSAGPMPSGGPM
PPARPGPPATANHLGLSVPGLVSVPPRLLPGPENHRGGSTEDPVLASSGTPPPTIAPDETFGGRVPRPAFVHYDKEEASD
VEISLESDSDDSVVIVPEGLPPLPPPPPSGTSPPPVAPAGPPTASPPMPAKEEPEELPATPGPLPPPPPPPVPGPVTLPP
PQLVPEGTTGGAGPPALEEDLTVININSSDEEEEEEEEEEEEEEEEEDFEEEEEEEEYFEEEEEEEEFEEEFEEEEGELE
EEEEEEEEEEEEELEEVEDLEFGSTGGEVEEESGPPPPTLPPALPPPESPKVPPEPEPEPGLLLEVEEPGTEEEPGAETA
PTLAPEVLPSQGEEEREGESPTGGPPLQEPPEEEPSAPPTLLEEETEGGSDKVLPLPEAATQEEMETETEATALQEKEED
DTAAMLADFIDCPPDDEKPPSVTEPDS
> Erinaceus europaeus [A0A1S2ZMJ1]
MAAAVLSGPSAGSAAGVPGGTGGLSAVNSGPRLRLLLLESVSGLLQPRAGSAVASVHPPARSAPHLPGLMCLLRLHGTVG
GAQNLSAVGALVGLSNARLGSIKTRFEGLCLLSLLVGESPTEMFQQHCVSWLRSIQQVLQSQDPPPTMELAVAVLKDLLR
YAAQLPTLFREISMNHLPGLLTSLLGLRLECELSALEGMKACMTYFPRACGSLKGKLASFFLSRIDALSPQLQQLACECY
SKLPSLGAGFSQGLKHTESWEQELHNLLTSLHSLLGALYEGAEAVPMQHEGPGVEMLLSPSEDGDAHVLLRLRQRFSGLA
HCLGLMLSSEFVAPVSVPVQEILDVICRTLSISAKNISLLGDGPLRLLLLPSIHLEALDLLSALILACGSRLLRFGSLII
RLLPQVLNAWSIGRDNLSPGQEKPYSTLRTKVYTVLELWVQVCGASAGMLQGGAPGEALLTHLLSDISPPADTLKLRSPR
GSPDGGLQTGKPSAPKKLKLDMGEAMAPPSHRKGDSNANSDVCAAALRGLSRTILMCGPLIKEETHRKLHDLVLPLLMGF
QQGDVLGTSPYTSSRCRQELYRLLLALLLAPSPRCPPPLTCALQAFSLGQREDSLEVSSFCSEALVTCAALTHPRVPPLQ
PMGSTCPTPAPVPPPEAQSPFRAPPFHPPGPIPSAGPMPSAGPVASTAPMPSAAPMPSAGPMPSVGPMPPTRPGPPATAN
HLGLSVPGLVSVPPRLLPGAENHRAGSNEDPVLAPSGTPPPIPSDETFGGRVPRPAFVHYDKEEASDVEISLESDSDDSV
VIVPEGLPPLPPQPPSGATPPPVAPAGPPAASPPVPAKEEPEELPAAPGPLPLPPLPPVPGPVTLPPPQLVPEGTPGGGG
ATAMEEDLTVININSSDEEEEEEEEEEEEEEEEEEEEEDFEEEEEDEEEYFEEEEEEEFEEEFEEEEGELEEEEEEEEED
EEEEELEEVEELEFGSAVGEAEEGGPPAPSLPAALPPAESPKVQPEPEPEPGLLLEVEEPGAEEEQGAEMAPTLAPEVLP
SQGQAEREAGSPPASSPPPQELVEEEPSAPPALLEEAAESGDDKKPPLPEPSAAEEEMEMETEAADLPEKEQDDTAAMLA
DFIDCPPDDDKPPPDTEPDS
> Mus musculus [Q9DBD5]
MAAAVLSGASAGSPAGAPGGPGGLSAVGSGPRLRLLLLESISGLLQPRTASPVAPVHPPIQWAPHLPGLMCLLRLHGTAG
GAQNLSALGALVNLSNAHLGSIKTRFEGLCLLSLLIGESPTELFQQHCVSWLRSIQQVLQSQDSPSTMELAVAVLRDLLR
HASQLPTLFRDISTNHLPGLLTSLLGLRPECEQSALEGMKACVTYFPRACGSLKGKLASFFLSRLDSLNPQLQQLACECY
SRLPSLGAGFSQGLKHTENWEQELHSLLTSLHSLLGSLFEETEPAPVQSEGPGIEMLLSHSEDGNTHVLLQLRQRFSGLA
RCLGLMLSSEFGAPVSVPVQEILDLICRILGISSKNINLLGDGPLRLLLLPSLHLEALDLLSALILACGSRLLRFGALIS
RLLPQVLNAWSTGRDTLAPGQERPYSTIRTKVYAILELWVQVCGASAGMLQGGASGEALLTHLLSDISPPADALKLCSTR
GSSDGGLQSGKPSAPKKLKLDMGEALAPPSQRKGDRNANSDVCAAALRGLSRTILMCGPLIKEETHRRLHDLVLPLVMSV
QQGEVLGSSPYNSSCCRLGLYRLLLALLLAPSPRCPPPLACALKAFSLGQWEDSLEVSSFCSEALVTCAALTHPRVPPLQ
SSGPACPTPAPVPPPEAPSSFRAPAFHPPGPMPSIGAVPSTGPLPSAGPIPTVGSMASTGQVPSRPGPPATANHLGLSVP
GLVSVPPRLLPGPENHRAGSGEDPVLAPSGTPPPSIPPDETFGGRVPRPAFVHYDKEEASDVEISLESDSDDSVVIVPEG
LPSLPPAPPSGTPPPAAPAGPPTASPPVPAKEDSEELPATPGPPPPPPPPPPPASGPVTLPPPQLVPEGTPGGGGPTAME
EDLTVININSSDEEEEEEEEEEEEDEDEEEEDFEEEEEDEEEYFEEEEEEEEFEEEFEEEEGELEEEEEEEEEELDEVED
VEFGSAGEVEEGGPPPPTLPPALPPSDSPKVQPEAEPEPGLLLEVEEPGPEEVPGPETAPTLAPEVLPSQEEGEQEVGSP
AAGPPQELVEESSAPPALLEEGTEGGGDKVPPPPETPAEEMETEAEVPAPQEKEQDDTAAMLADFIDCPPDDEKPPPATE
PDS
> Rattus norvegicus [Q56B11]
MAAAVLSGPTTGSPAGAPGGPGGLSAAGSGPRLRLLLLESVSGLLQPRTGSHVAPVHPPIQWAPYLPGLMCLLRLHGTAG
GAQNLSALGALVNLSNAHLSSIKTRFEGLCLLSLLVGESPTELFQQHCVSWLRSIQQVLQSQDSPPTMELAVAILRDLLR
YASQLPTLFRDISTNHLPGLLTSLLGLRPQCEQSALEGMKACVTYFPRACGFLKGKLASFFLSRLDSLNPQLQQLACECY
SRLPSLGAGFSQGLKHTENWEQELHSLLTSLHSLLGSLFEETETAPVQSEGPGVEMLLSPSEDDNTHVLLQLWQRFSGLA
RCLGLMLSSEFGAPVSVPVQEILDLICRILGISSKNINLLGDGPLRLLLLPSLHLEALDLLSALILACGGRLLRFGALIS
RLLPQVLNTWSTGRDALAPGQERPYSTIRTKVYAILELWVQVCGASAGMLQGGASGEALLTHLLSDISPPADALKLCSTR
GSSDGGLQSGKPSAPKKLKLDMGEALAPPSQRKGDRNADSDVCAAALRGLSRTILMCGPLVKEETHRRLHDLVLPLVMSV
QQGEVLGSSPYNSSCCRLELYRLLLALLLAPSPRCPPPLSCALKAFSLGQWEDSLEVSSFCSEALVTCSALTHPRVPPLQ
SSGPACPTPAPVPPPEAPSSFRAPAFHTPGPMPSIGALPSPGPVPSAGPIPTVGSMSSAGSVPSTGPVPSRPGPPATANH
LGLAVPGLVSVPPRLLPGSENHRAGSGEDPVLAPSGTPPPSIPPDETFGGRVPRPAFVHYDKEEASDVEISLESDSDDSV
VIVPEGLPSLPPPPSGTPPPVAPIGPPTASPPVPAKEDSEELPATPGPLPPPPPPPPPVSGPVTLPPPQLVPEGTPGGGG
PTAMEEDLTVININSSDEEEEEEEEEEEEDEDVEEEDFEEEEEDEEEYFEEEEEEEEFEEEFEEEEGELEEEEEEEEEEL
EEVEDVEFGSAGEVEEGGPPPPTLPPALPPTDSPKVQPEAEPEPGLLLEVEEPGAEDGPGPEIAPTLAPEVLPSQEEVER
EGESPTAGPPQELVEEESSAPPTLLEEGTEGGGDKVPPPPETPAQEEMETETEASAPQGKEQDDTAAMLADFIDCPPDDE
KPPPATEPDS
> Neomonachus schauinslandi [A0A2Y9HLD1]
MAAAVLSGPSAGSAAGAPGGAGGLSAVGSGPRLRLLLLESVSGLLQPRAGSAVAPVHPPVRSAPHLPGLMCLLRLHGTVG
GAQNLSAVGALVGLSNARLGSIKTRFEGLCLLSLLVGESPTEMFQQHCVSWLRSIQQVLQSQDPPPTMELAVAVLRDLLR
YAAQLPTLFRDISMNHLPGLLTSLLGLRPECELSALEGMKACMTYFPRACGSLKGKLASFFLSRVDALSPQLQQLACECY
SRLPSLGAGFSQGLKHTESWQQELRSLLASLHSLLGALYEGAETAPVQYEGPGVEVLLTPSEDGDAHALLRLRQRFCGLA
RCLGLMLSSEFGAPVSVPVQEILDVICRTLSISAKNISLLGDGPLRLLLLPSIHLDALDLLSALILACGGRLLRFGGLVS
RLLPQVLNAWNMGRDTLSPGQERPYSTVRTKVYAVLELWVQVCGASAGVLQGGASGEALLSHLLSDIAPPADALKLRSPR
GSPDGGLQSGKPSAPKKLKLDLGEAMAPPSHRKGDSNANSDVCAAALRGLSRTVLMCGPLIKEETHRRLHDLVVPLVMGL

QRGETLGSSPYTSSRCRRELYRLLLALLLAPSPRCPPPLACALHAFSLGQREDSLEVSSFCSEALVVCAALTHPRVPPLQ
PAGPPCPTPAPVPPPEAPSPFRAPPFHPPAGPLPPARPGPPATANHLGLSVPGLVSVPPRLLPGPENHRAGSNEDPVLAP
SGTPPPALPPDETFGGRVPRPAFVHYDKEEASDVEISLESDSDDSVVIVPEGLPPLPPPPPSGTTPPPVAPAGPPTASPP
VPAKEEPEELPAAPGPLPPPPPPPPVPGPVALPPPQLVPEGTPGGGGPPTLEEDLTVININSSDEEEEEEEEEEEEEEEEE
EEEEDFEEEEEDEEEYFEEEEEEEEEFEEEFEEEEGELEEEEDEEDEEDEEELEELEEAEFGPAGGPAEEGGPAPPSPAPA
LPPAQSPTVQPEPEGEPGLLLEVEEPGAEEEPGAQAAPTLAPEVLPSQGEGPRATGSPPAGPPPQELVEEEPLAPPTLLE
EGTESGGDRVPPLPETPAAEDGEAEAEAETAALQEKEQDDTAAMLADFIDCPPDDEKPPPAPEPDS
> Pantherophis guttatus [A0A6P9CU85]
MGSMKTRFEGLCLLSLLVTESGSETFAQNCLVWLRSLQHLLQSQDPPATMELAVLVLRDLLRYSAQLPELARDIGTNHIP
GLLTSLLALKPECQLPVLEGCQACMMFYPRACGSLRGKLATYFLSCMDAETPHLQQLACECYALLPSLGAGFAQGLKYRE
SWEQQAHSLVATLHRLLGTLYEGAETEPLHYDGPGEEVLLPPRDEETNSLLLLLAKRRFAGLAKCLCRMLRNDFGAPVAV
PAQAILDLVCRALDVSVKNMSWFGDGPLRMLLLPSIHLEALDLLAALILACGPRLVRFGGALCRLFPQVLNMWRAGQDLS
SPGLQRPYSAVRARLYQVLDLWVQVAGAASGVLLGHSSQSDALLGHFINDISPPSDTLKIQLSPASEGKPSAAKKPKLSA
VGSLGCPFRKHDPQANSDVCLAALQGLSRIILLSGSLMKEQLHTRLQELAIPLLIQLGQAELPLGSPFAGASCRRELYRL
LLALSLSPAPACPPPLHCALRLLSQGRTDPNLLVSSFCTEAAVICSALLHPRVPSLQLPLAAPSAHLPGSLEVSPAAAVA
AATLSPFCPAIPVPFPAPRPLPPAPGPLPPNSLGLPLPGFAAPQPPPRLVPEEPLPPPSPGTAEAAALATGAKLRRSVFV
HYDKEEEDDVEISLESDSDDSVVIVPKGQLGKTTGALNSATVPAAAPAPPPLPAPPPPPLLSRHPLWWPVRRPPWSPQRL
FPWESHCLPRPRRCCRLPRLQEDPTVININSSEEEEEDDDDFPEDEEYLDEEEEEEEFDEEEGDFEEMDEEEDDFDEDDE
EGLTEEEEEEEEGLTDEEEEEEEEAGLPPLPRRNEDEPPALLPAVKDGPLKLPEEEEDDGGAGLLMEVEEEAFHPPEEEEE
EEQEEEQEGERTVGALLLLKAEEDTALPLLPGAQPLPLPPLQEGPGLLGAPMDQEGHPEQKEGAVEAASAVSLQPEEAIH
AGSSVDRARASPDSAAGQGEPEKLLQPDEEVVEVKEEEARALDETEAMLADFVDCPPDEEKDPAQPCS
>Alligator sinensis [A0A3Q0FKA0]
MGALNPHVLPLRPGLHEGRCDPAAQAPHRATTGASPAARPPPPALREPGRPVRGLRDSGAAAQQNAPALGGLVGVTSARL
GCGKTRFEGLCLLSLLVQESPSDLFQQHCLAWLRSLQHLLQSQDPAATVALGVTVLRDLLLYSAQLPELARDISTNHIPG
LLTSLLALKPECQLSTLEGTRACMTHYPRACGSLRDKLAAYFLARVDSKSPELQQLACKCYALLPRLGGGFPQGLPRREC
WEQELHCILATLHGLLGTLYEGAETDPVPYEGPGVELLLPALQDGEPGCIPSLRARFAGLARCLRLMLSSEFGSPVTVPV
QDILNLVCRALNISSKNISWFGDGPLKMLLLPSIHLESLDVLAALIVACGARLARWGSVLARLFSQVLDVWSGARDAPPG
QEKPYSAVRSRLYQVLELWLQVAGAGGGVLQGSGPLSEALLGHMLSDIMPPTDSIKMRTGHHSLSEGGKPSAPKKPKLME
GAGDAPALHRKVDPAANSDVCQAALQALYRAVLLGGPLIKEEMHRRLQELVVPLLLRLSQDGGPTGSPYAHPACRLALHR
LLLALLLAPAPAAAPPPLHCALRVFTQGQHDPSLEVSSFCAEALVICSAVARPRVPSLQLSLSRPSPTPPAQAPAPDIAS
PPPPAPPAPEPVEEPVVAVPPPPATAPSVPELPPTSLAEDDPAVININSSEEEEEEEEGFDEEEEEYFEDEEDDEEEEEE
EEGFEEEEDEEEEDFEAELEEEELEEEDEEEEYEEDEGLSEEEEEDEEEGHSGPPPSVPTPVPPAADINKAPEPEDLIME
VEETPAPPEEEEEEEDEEVVVMKTPALPQQEEEAGGGQEGAAGVPPPPLAVPVPEEAEPPALLPQPPQIEEVPPTSPEEGA
GEGSAEARPPPGAPEEEVVVAIKEEVEEVDEAATMLADFIDCPPDDDKTPPETGS
> Pogona vitticeps [A0A6J0U8G0]
MQLAVLILRDLLGYSCQLPELAREISTNHIPGLLTSLLALKPECQLSALEGSKACMMFYPRACGSLRGRLAAYFLACLEA
ETPQLQQLACECYALLPSLGAGFTQGLKYKECWEHQAHCLLATLHSLLGTLYEGAETDPLHYEGPGMEILLPAPEDGESS
FLLQLKHRFSALMKCLCKMLSDEFVAPVTVPVQDVLDLVCRSLDISAKSISWFGDGPLRMLLLPAIHLESLDLLAALLLA
CGPRLVRFGGTLCRLFPQVLTMWSSSRDLFPPGQERPYSAIRTRLYQVLDLWIQVAGAASGILQGPGTQTEALLGHLISD
ISPPSDTLKIREGCSGSEGKPSAPKKPRLSDVGGSGSLSHKHDPRANSSVCLAALQGLSRAVLLTGSLIKEQTHKRLQEL
AVLLLVRLSQAEPHPGSPYASADCRRELYRLLLALVLAPSPSCPPPLHCALRLLGQGRLDLNLQVSSFCAEALVVCSALL
HPVALAAGAKLRRSVFIHYDKEEEEDVEISLESDSDDSVVIVPKGLLGKGGALGTTAAPSSPPPPPPPPPPPPSEEAPPEA
VVPLPLGPPPLAPPPAAVALPSSPAPEASDAALPALMEEDPTVININSSEEEEEEEEDEEDEEEEDFPEDEEYFDEEEVSG
WVPRRPAGGAPWALCTLHSSSHEEDGCRPLLRPGWALSLAPIKDSLWLSLAPRLTCMLLRQTFGGGRGEEATGWLCVCMC
VCVCVRKRETGWGLVCFVPREGGQVRSLPVLLSFADQEEEFDEEEGEFEEELDEEEMEELDEEEEDDFDEEEEGITEEEE
EEEEEEEEGLTEEEEGLPPTLPHRNEEEPPELLPVKEEPPKLLEEEELDEEEEEEEEGAGLHMEVEEEAFHPVQEEEEEV
EEEENCVEGGRTAVEVVLLKAEGPPLPPVVQELQLPSALSLLPPPRQEAPSPPPAPLEEEERLPMEQLGPADTEASTKTA
TATTTAAAAAADPQERREEEEEEETGEILVCAGGSRDTGLEAGLVRGESEKQPPPPLADEVVEVKEEEEEEESLAVDETEAM
LADFVDCPPDDEKAPPGPSS
> Xenopus laevis [Q58HI1]
MEVTIAGILERDLSEGELAEAIRGLREHGAFRGEGLPAAMSGLLSSCNSRLTSASSRIEGLSLLALAVEESPTDVFVQHC
VSWLRSLLQIIQSQDPPRVVSLAVFVLRSLLAHSSALPELSREISTNHIPGLLTSLLGLRRQCLVPALEGIRSCFLSYPR
ACGSLRGKLTAFLLSLLDAENQQIQEVACQCYSLLPSLGSGFSQGIKHTENWERQIQSVICSLHSVFLQLYQGSETDTAR
YEGSGTELEFPSVEDDGTHGVLQLARRFTALGQCMRLLLREQFPAPVRVPVSDILSLVCRVVNVSPKNLSWHGEESLKLL
LLPRVHSSILEILEATIIACGPRLLPFSAVICRLFPQLLLSWAAVKGITGIPSGQERPYSSLRCSVYRVLETWVTTCGIS
SGVLQGPMHHSDILLANLLSDITPPTDAIKMSTFVQLGAKKQKVSEVGDDDFQSHRKRDNTANVELCAAALKGLCCVILH
CGSVIKEDVHRRLQELSIPLLLRLQQGSDQWLGPYISSDCRKELYRLLLCLTLTPNPKLPAPLHCAIRIFRGGTTEESLQ
VSRFSTEALAICRILIHPRVPSLQRPLPHLAPRPSVQSDAPTLRPPAALSTFPAMPPANHLPPRPTVPAMSTEPPIPAAV
PSPPPEESFGEKPRRAVFIHFDKEEPSDVEISLESDSDDSVVIVPEGLFAKSDSKPEPSPPAVKPPTEEVTEQVAPSAVP
SSSTAAPPPPPPPPAPPVCAGPSSAPVPIAEAPPPPQQEVDTVININSSDDEEDGEEDEEEGLYDDEDEEDYYDEEEDED
LEGLEEDDYDYEEDEEGITEEEEEDLEEEGEDDEEEVEDEECLMPDEMQIGSEAEEIPDGIETSSLHEGELEEGPPRLSP
VQEDEAVDTGLLMLVESEDREPSGEAPGEGLPESDLTRSPPQPPVLTPPSPPDEPPPMEESDVPPLEEPDLEVAPVAEEP
VEETEEKKPEEVTVEKPEPEPEEEEQIADADAMLADFVDCPPDDDKLPEPCT
> Puma concolor [A0A6P6HJV5]
MELAVAVLRDLLRYAAQLPTLFRDISTNHLPGLLTSLLGLRPECELSAMEGMKACMTYFPRACGSLKGKLASFFLSRVDA
LSPQLQQDLLSALILACGSRLLRFGALISRLLPQVLNAWNLGRDALPPGQERPYSAVRTKVYAVLDLWVQVCGASAGVLQ
GGASGEALLSHLLSDISPPADALKLRSPRGSPDGGLQSGKPSAPKKLKLDMGEATAPPGHRKGDSNANSDVCAAALRGLS
RTVLMCGPLIKEETHRRLHDLVLPLVMGVRQGEVLGSSPYTSSRCRRELYRLLLALLLAPSPRCPPPLACALQAFSLGQR
EDSLEVSSFCSEALSGRILLGRLPEPFKRPPLIAARSYPPPSTPHPALSAPPAPLGSFNTPRTLLPAVPSALGLEHLPTP
DRALPPARSFGSVMTIAFSARPSRSVLLPTAHARMQSQ
> Austrofundulus limnaeus [A0A2I4CY47]
MAAPSGLHGTGAMRLTEGLVSVLKEQRPQFLAEALSNYREHGVFSTQNTSDAAGLVGFSNAKLSSSKTRSEGLCLLSMLV
KDSSSDLFQQHCLSWLRSLQQIIQSQAPVQTIQLAVSILKDLLQYSCQLAELAREVSLNFILGILTSLLGLKTECELAAM
EGMKACMIYYPRACGSLKDKLGVYFLSKMDSTNRKTQEVACQGYSHLPCLGGLLDKAGNVGRAEGWTKQIHCLLASANSL
LAQIYQGSETDEAMQYQGPGMELAFDYLDQTDPLLLLQLQHRYTAVCMTIKHTLRADPASAVHLPVRLILNLVCRALAVN
SKSINLTADGSLRLLILPSVHISSLEVLSELITEVRSGMIQYAAVIQRLFSQTLTAWTPLPDTSVGQQRAYSSVRVCVYR
TLELWVKVVGSCAGILQGASSHAGLLFNHLLLDITPEAESVKLRTGLSVDVVPGGKPGPRRTKQLVLAETVGPSLQRKGD

LLSNQDTCLSALRALRQIIQTSGTLLKDEIHKRLHEVVLPLCVRLQQQQSSSISACDPAGGISGQYSSALTRRELYRLLL
ALVLVPSPCWPPPLSCAVSILSNGRTERNLKVSTFCAEALTVCNSIVHSRSPSIALPLPPLALKPTTTPSVLSCQGPGPR
LTLPTLLGGPAPSPSFPTRHTLSMGTTSLLGSLENHLSLIPGMPSQASAPGDLMLSPQTQHQSDPVAPPEGQRSVFVRYD
KEEAEDVEISLASDSDDSVVIVPPGMLNTENQQNDLAANSQNVLSSAAGSTGVSLPGADSVSMVPTTAASTTVDVASLPN
DLATSSSTPINSFPPSSASVVSLVPSLNSSSFSALPGGGQGDSGAGKPGLQQMLMQPSAAGQPGSVTLPLQMHQLQNQLT
QQGRHLHQHPPAPTSNEESAVININSTDEEEDEDEEEDMDDEEELEDEEDGIDEDDEEEEGSEEFYDGEEYEEFEEEEGE
ELEEEEEEEDEDGDIPPLDGSEDKSEEISMEDDKVLQAAMEDGEVESYSVEGEAGGGIEELQTSRTLFRGDRMKIQEVES
IGVLEEAREAEREEDESERMDDPTMPQILCVTGGAPEDRSESAEESGGGIGGLQEEVSLWEQGAKGEEVAPPCEEHTASQ
SQQESAAENEEGIERDEQLSSHQEEEPAAAEGEDTAAGAEDTSAGEKVEEQEQTDVDQGNITEAEQVTEEETVAEERKED
GEESDGKEEKGLKRKREDDNTVDEDGQNTEKKMMEEEAMASMLADFNPCPPDDEDDGSGLNRSSTSPPEGM
>Mummichog [A0A3Q2PJM5]
MATSAWLHGAAAMRLTEGLVSVLKEQRPEYLSDVLANYREHGVFSAQGTSDIAGLVGFSNAKLGSSKTRFEGLCLLSMLV
KDSSSDLFQQHCLSWLRSLQQVIQSQAPTQTIHLAVNILKDLLQYSCQLAELAREVGLNCILGILTSLLGLKTECEFAAM
EGMKACMIYYPRACGSLKDKLGAYFLSKMDSTNRKTQEMACQGYSRLPCLGGLADRSVGTGRAEGWTNQIQCLLASANGI
LAEIYQDFETDEAVQYRGPGVELPFAHLDQSDPLLFLQLQHRYTALCLAIKHTLRADPASAVQLPVRPILNLVCRALAVN
SKSINLTADGSLRLLVLPSIHISTLEVLAELITVVRNGMIQYAAVIQRLFSQTLSAWAASPETNVGQQRAYSSVRVSVYR
TLELWVKLVGASANILQGTSSHAELLFNHLLGDITPGAESVKLRAGLSVDVVPGGKPGPRRTKPLVIADTVGPSLQRKGD
PLSNQDTCLSALKVLRSIIQTSGTLLKDDIHKRLHEVVLPLCVRLQQQQSSSIAACESVGGISGQYSSALTRRELYRLLL
ALVLVPSPCWPPPLTCTVSILSNGRIDRSLKVSCFCAEALTICNSILHPRTPSIALPLPPLALKPTPTPSVLSCPGPTPR
LAMSTLLGGPAPGPPFSARHPLSLGPNSLLGSLENHLSLVPGLPGQASAPGDLMMSPHTHSQPDPTGLCPTEGQRPVFVR
YDKEEAEDVEISLASDSDDSVVIVPPGMLSTENQLDDPATNSQNILSSTPCGAGIALPGGDAVTMVPTTAAPTTLDVSSL
PNDLVTSSALLTTSSTPVNSFPPSSGSVVSLVSSLSSSSASAPAGGLADSMAVKPQLQQMLMQPSASGQSNPMALPLQMH
QLQNQLAQQGRHLHQHPPAPASNEESAVININSTDDEDEDEEDMEDEEELDEEDGMDEEDEEEEGSEEFYEGEEYEDFDE
EDAEELEEEEEDEDGDMPPLEGSEDKSEEVTMEEEEEKALRAEVEEGEVAGLNVEGDTGGGIEELQTSRAVFGEERMKVQ
EVESIGVLEEAREGDGEEDESERMDDPTMPQILCVTGGALEERCESSEEAAEAAGELQEKMNLWDQSVRENEPITAAEEG
STNHSQQESVAESAQEAAVREEQPSTEQPAVGEDVASAGGTEEEEEVLAAQSITEQEQTEADQGDLKEAERATPEGRGEG
EEEGGKEQEKESNIEEDKGMKRKREDENIEDEEGPSPEKKKEDEMMVSMLADFVAVPPDDEEVASGSNRS
> Thamnophis sirtalis [A0A6I9Z579]
MGSMKTRFEGLCLLSLLVTESGSETFAENCLLWLRSLQHLLQSQDPPGTMELAVLVLRDLLSYSAQLPELARDIATNHIP
GLLTSLLALKPECQLPVLEGCQACMTFYPRACGSLRGKLATYFLSCMDAETPHLQQLACECYALLPSLGAGFAQGLKYRE
SWEQQAHSLVATLHRLLGMLYEGAETEPLHYDGPGEEVLLPPLRDEETDSLLLLLAKRRFAGLAKCLCRMLRNDFGAPVA
VPAQAILDLVCRALDVSVKNMSWFGDGPLRMLLLPSIHLEALDLLAALIVACGPRLVRFGGILCRLFPQVLNVWRAGQDL
PSPGLQRPYSAVRARLYQVLDLWVQVAGAASGVLLGHSSQSDALLGHLINDISPPSDTLKIQPSPPSEGKPSAAKKPKLS
AVGGLGCPFRKHDPQA
>Maylandia zebra [A0A3P9D719]
MNVNIISHSRNRFTLILFEGLCLLSMLVKDSSSDLFQQHCLSWLRSLQQVIQSQAPVETVQLAVNILKDLLQYSSQLAEL
AREVGLNSILGILTSLLGLKSECELAAMEGMTACMTYYPRACGSLRDKLAAYFLSKMDSTDRKTQEMACQCYGRLPCLGG
VLDRGVGAGRAEGWTNQIHCLLASANGLLAQLYQGSETDGAMQYQGPGMELAFPHLDPTDPLLLLQLQHRYTAVCLALKH
TLRVDPASAVRLPVRPMLNLVCRALAVSSKSINLTGDGSVRLLVLPIIHSITLEVLSSVITVVRVGMVQYAAVIQRLFSQ
TLSAWTPLPETSVGQQRAYSAVRVSVYRTLELWVQVAGASSSILQGSPGHSELLFNHLLGDITPGAESVKKRHKSDHLGL
SADVVPGGKPGPRRTKHLVMADTVGPSLQRKGDHLANQDTCLAAVRALRQIIQTSGTLLKDNIHKRLHEVVLPLCVRLQQ
QQSCSSNACESAGSASGQYSSALTRRELYRLLLALVLVPSPCWPPPLTCAVSILSNGRTDRNVKVSSFCAEALTICNLIL
HPRFPSIALPLPPLTMKPSSTTPVLPSSQGPAPGLTLPTLLGGPAPGPPFPARHSVNLGPASLLSSFENHLSLVPGLPGQ
GPTAGDMILSPHSHHQPDPAGLGPPEGQRPVFVRFDKEEAEDVEISLASDSDDSVVIVPPGMLNMETQQDDAAANSQSMS
SSAPGGTGVTLAGAESVTMVPTTAAAATIDGVSLPNALAASSPLLTASTTSINSFPPSSASVVSLVPPLNSSTFTAPPVG
LGESLPGRPQLQQMLMQPSTASQAGPMGLPLQMHLQNQLSQQGRHLHQQPAAAASSEDSAVININSTDDEEEDDEDIDED
EELEEDEEEEGIDEEDEEEDVSELADEFFDGEEYEELDEEDGEDLEEEEEEEEEEEEDGDIPPLEGVEDKDGGSGIQEGKL
DEGRISAFNVEERTEGGIEEIQTNRALFGEDTMKVQEVESIGVLEAAREGEVEEEDNERMDDPTMEQAGAELQKEECSWE
QGAKEDEPQAAESEQPASQQVDPEEDTEKSSEKEAEQPETGAGGGQESNGEEGKGVKRKREEGHGKEEVGQSPEKKKMDD
EAMASMLADFVACPPDDEDGASGSKAS
> Mummichog [A0A3Q2UHN9]
MATSAWLHGAAAMRLTEGLVSVLKEQRPEYLSDVLANYREHGVFSAQGTSDIAGLVGFSNAKLGSSKTRFEGLCLLSMLV
KDSSSDLFQQHCLSWLRSLQQVIQSQAPTQTIHLAVNILKDLLQYSCQLAELAREVGLNCILGILTSLLGLKTECEFAAM
EGMKACMIYYPRACGSLKDKLGAYFLSKMDSTNRKTQEMACQGYSRLPCLGGLADRSVGTGRAEGWTNQIQCLLASANGI
LAEIYQDFETDEAVQYRGPGVELPFAHLDQSDPLLFLQLQHRYTALCLAIKHTLRADPASAVQLPVRPILNLVCRALAVN
SKSINLTADGSLRLLVLPSIHISTLEVLAELITVVRNGMIQYAAVIQRLFSQTLSAWAASPETNVGQQRAYSSVRVSVYR
TLELWVKLVGASANILQGTSSHAELLFNHLLGDITPGAESVKLRAGLSVDVVPGGKPGPRRTKPLVIADTVGPSLQRKGD
PLSNQDTCLSALKVLRSIIQTSGTLLKDDIHKRLHEVVLPLCVRLQQQQSSSIAACESVGGISGQYSSALTRRELYRLLL
ALVLVPSPCWPPPLTCTVSILSNGRIDRSLKVSCFCAEALTICNSILHPRTPSIALPLPPLALKPTPTPSVLSCPGPTPR
LAMSTLLGGPAPGPPFSARHPLSLGPNSLLGSLENHLSLVPGLPGQASAPGDLMMSPHTHSQPDPTGLCPTEGQRPVFVR
YDKEEAEDVEISLASDSDDSVVIVPPGMLSTENQLDDPATNSQNILSSTPCGAGIALPGGDAVTMVPTTAAPTTLDVSSL
PNDLVTSSALLTTSSTPVNSFPPSSGSVVSLVSSLSSSSASAPAGGLADSMAVKPQLQQMLMQPSASGQSNPMALPLQMH
QLQNQLAQQGRHLHQHPPAPASNEESAVININSTDDEDEDEEDMEDEEELDEEDGMDEEDEEEEGSEEFYEGEEYEDFDE
EDAEELEEEEEDEDGDMPPLEGSEDKSEEVTMEEEEEKALRAEVEEGEVAGLNVEGDTGGGIEELQTSRAVFGEERMKVQ
EVESIGVLEEAREGDGEEDESERMDDPTMPQILCVTGGALEERCESSEEAAEAAGELQEKMNLWDQSVRENEPITAAEEG
STNHSQQESVAESAQEAAVREEQPSTEQPAVGEDVASAGGTEEEEEVLAAQSITEQEQTEADQGDLKEAERATPEGRGEG
EEEGGKEQEKESNIEEDKGMKRKREDENIEDEEGPSPEKKKEDVNL
>Maylandia zebra [A0A3P9D6C2]
MLVKDSSSDLFQQHCLSWLRSLQQVIQSQAPVETVQLAVNILKDLLQYSSQLAELAREVGLNSILGILTSLLGLKSECEL
AAMEGMTACMTYYPRACGSLRDKLAAYFLSKMDSTDRKTQEMACQCYGRLPCLGGVLDRGVGAGRAEGWTNQIHCLLASA
NGLLAQLYQGSETDGAMQYQGPGMELAFPHLDPTDPLLLLQLQHRYTAVCLALKHTLRVDPASAVRLPVRPMLNLVCRAL
AVSSKSINLTGDGSVRLLVLPIIHSITLEVLSSVITVVRVGMVQYAAVIQRLFSQTLSAWTPLPETSVGQQRAYSAVRVS
VYRTLELWVQVAGASSSILQGSPGHSELLFNHLLGDITPGAESVKLRAGLSADVVPGGKPGPRRTKHLVMADTVGPSLQR
KGDHLANQDTCLAAVRALRQIIQTSGTLLKDNIHKRLHEVVLPLCVRLQQQQSCSSNACESAGSASGQYSSALTRRELYR
LLLALVLVPSPCWPPPLTCAVSILSNGRTDRNVKVSSFCAEALTICNLILHPRFPSIALPLPPLTMKPSSTTPVLPSSQG
PAPGLTLPTLLGGPAPGPPFPARHSVNLGPASLLSSFENHLSLVPGLPGQGPTAGDMILSPHSHHQPDPAGLGPPEGQRP
VFVRFDKEEAEDVEISLASDSDDSVVIVPPGMLNMETQQDDAAANSQSMSSSAPGGTGVTLAGAESVTMVPTTAAAATID

GVSLPNALAASSPLLTASTTSINSFPPSSASVVSLVPPLNSSTFTAPPVGLGESLPGRPQLQQMLMQPSTASQAGPMGLP
LQMHLQNQLSQQGRHLHQQPAAAASSEDSAVININSTDDEEEDDEDIDEDEELEEDEEEEGIDEEDEEEDVSELADEFFD
GEEYEELDEEDGEDLEEEEEEEEEEEEDGDIPPLEGVEDKDGGSGIQEGKLDEGRISAFNVEERTEGGIEEIQTNRALFGE
DTMKVQEVESIGVLEAAREGEVEEEDNERMDDPTMPQILCVTGGALEEREEAEEEREQAGAELQKEECSWEQGAKEDEPQ
AAESEQPASQQESAAEPAEDASVSDNQLPSCEDEPLEAVQAGDSTAVADSENATEPNVTEKQEEDTEKSSEKEAEQPETG
AGGGQESNGEEGKGVKRKREEGHGKEEVGQSPEKKKMDDEAMASMLADFVACPPDDEDGASGSKAS
>Oryzias melastigma [A0A834CQ56]
MSREEGSGRPMREMSGEEGSGRPMREMSREGGRSCLLMLQKLSGEEAYLLRALKPKEIFQLVHGCAGQQTETKLAGLKEE
LSPEVVEGALEGLFQALTTESERSESSSSAEKSRSNKKERGKMATSVGLHGSSALKLTEGLVSFLKEQRPEYLPEVLVNY
REHGLQIQGQNGAAGLVGFSNTKLSSSKTRFEGLCLLSMLVKDSTSDLFQQHCLFWLRSLQQIIQSQAPVQTIQLAVNIL
KDLLQYSCQLAELAREVGLNSILGILTSLLGLKTECELAAMEGMVACMVFYPRACGSLKDKLRAYFISKMDSNNKKTQEM
ACLCYSHLPCVGGLLDRAVSAGKADGWTNQIHCLLATADGLLSQIYKGSEPETVVEYQGPGVELAFPYLDPTETLQLLRL
QQRYTAVCRAVKHTLRVDPASAVRLPVKPVLNLVCRALAVNAKNASFSADGSLMLLILPSIHCNTLEMLSDLITVVRSSM
VPYAAIIQRLFSQTLSAWTPVVEAGVGRQRGYSSVRVSVYKTLELWVKVVGASVSILQENPMHADLLFNNLLGDITPGPE
SIKLRAGLGVDVVPGGKPGPRRTKHLVIADPMGPSLQRKGDPLSNQDTCLAALRALRQIIQTCGTLIKEDIHKRLHEVVL
PLCVCLQQQKGSSSVFESTGAISGQYSSALARRELYRLLLALVLVPSPSWPPPLTCAVSIFSNGRTDRNLKVSSFCAEAL
TICNTIVHPRTHSIALPLPPLTLRPSASAPVLSPQGSAPRLTLPTLLEGHGSGPPFPSRQSLNLGPSSLLSSLENHLSTV
PGLPAQAPTPGDLFSPHPHQQHDSAGLGLPESQRPVFVRFDKEEPDDVEISLASDSDDSVVIVPPGMITLDNQQDEAAAN
PQTMLSAAPAGSGVTLPVVESNSMVPITAATTTIDVSTLPNDLPTSSSLLTTSASSINSFPPPSASAATLPPALNSSIVS
DPHSDLGDSLPCKPQLQQMLMQTPTSSQGSSMGLPLQMHQLQNQLTQQGRLHQQLPAPPSNEDSTIININSTDDEDEED
LEDDEELEEEEEGLDEEDEEEGSDDFYDGEEYEDYDEEEGEELEEEEEEEENGDMPPLEGSEEKLVDVAIEEGQVLHPAA
AADEKVLTEHNVEADSERGIQELQPQRTMLGEERVKLQEVESIGVLEDALVGVEEEDESERMDDPTMPQIICVSGGAQES
IETGDEGEGTAHVLQEDTSNQPAKEDELKAAPEEQTSFSAEQEPEVETAQEDESSSSKTSDQPAELPAAAPEEPTEAGQQ
SPADLNTGEQQEENQASEDGEQPKDGEEEEKNRSIEPQREESDKEEAHGTKRKREEELTEEAGPSTEKKKVDDEAMASML
ADFVACPPDDEAAPGSTCS
> Trichechus manatus latirostris [A0A2Y9DKX7]
MAAAVLSGPSTGSAAGVPGGTGGLSAMGSGPRLRLLLLESVSGLLQPRTGSTVPSVHPPVRPVPHLPGLMCLLQLHGTVG
GAQNLSALGALVGLSNARLGSIKTRFEGLCLLSLLVGESPTEMFQQHCVSWLRSIQQVLQSQDPAPTMELAVAILKDLLR
YAAQLPTLFRDISMNHLPGLLTSLLGLRPECELSALEGMKACMIYFPRACGSLKGKLASFFLSRVEALSPHLQQLACECY
SRLPSLGAGFSQGLKHTESWEQELHCLLASLHSLLGTLYEGVETAPVQYEGPGVEMLLSSSEDGDAHALLRLRQRFSGLA
RCLGLMLSSEFGAPVSVPVQEILDLICRTLSVSGKNISLLGDGPLRLLLLPSIHLEALDLLSALILACGSRLLRFGGLIS
RLLPQVLNAWSMGRDILSPGQERPYSTVRAKVYAVLELWVKVCGASAGVLQGGASAEALLTHLLSDISPPADALKLRSPR
GSPDGGLQTGKPSAPKKLKLDVGEAAAPPSHRKGDSNANSDVCAAALRGLSRSILMCGPLIKEETHKRLHDLVLPLVMGI
QQGEVLGSSPYTSSRCRRELYHLLLALLLAPSPRCPPPLACALQAFSLGQREDSLEVSSFCSEALVTCAALTHPRVPPLQ
SVGTPCPTPAPVPPLESPSPFRAPPFHPPGPMPSVGPMPSAGPMPSAAPVPSARPGPPATANHLGLSAPGLVSVPPRLLS
GPENHRAGSNEDPVLAPSGSPPPSIPPDETFGGRVPRPAFVHYDKEEASDVEISLESDSDDSVVIVPEGLPPLPPPPPSG
STPPPVAPAGPPAASPPVPAKEEPEELPAAPGPLPPPPPLPVPGPVTLPPPQLVPEGTPGGGGPPALEEDLTVININSSD
EEEEDEEEEEEEEEEEEDEDFEEEEEDEEEYFEEEEEEEEEFEEEFEEEEGELEEEEDEEDEEEEEEELEEVEELELGSAGGE
VEEGGPAPPTLPPALPPSESPKAPPEPEPEPGLLLEVEEPGAEEEHGDEMAPTLVPEVLPSQGEVERKGGSPPAGPPPQE
LVEEEPPAPPTLLEEETEDGGDKVPPPPEASAEEEMDTETEAAALQEKEQDDTAAMLADFIDCPPDDEKPPAATEPDS
> Acinonyx jubatus [A0A6J1YDE5]
MAAAVLSGPSAGSAAGVPGGTGGLSAVGSGPRLRLLLLESVSGLLQPRAGSAVAPVHPPVRSAAHLPGLMCLLRLHGTVG
GAQNLSAVGALVGLSNARLGSVKTRFEGLCLLSLLVGESPTELFQQHCVSWLRSIQQVLQSQDPPPTMELAVAVLRDLLR
YAAQLPTLFRDISTNHLPGLLTSLLGLRPECELSAMEGMKACMTYFPRACGSLKGKLASFFLSRVDALSPQLQQLACECY
ARLPSLGAGFSQGLKHTESWEQELHSLLASLHGLLGALYEGADAAPVQCEGPGLDVLLAPSEDGDAHTLLRLRHRFSGLA
RCLGLLLSSEFGAPVSVPVQEILDIICRTLSISAKNISLLGDGPLRLLLLPSIHLDALDLLSALILACGSRLLRFGALIS
RLLPQVLNAWNLGRDALPPGQERPYSAVRTKVYAVLDLWVQVCGASAGVLQGGASGEALLSHLLSDISPPADALKLRSPR
GSPDGGLQSGKPSAPKKLKLDMGEATAPPGHRKGDSNANSDVCAAALRGLSRTVLMCGPLIKEETHRRLHDLVLPLVMGV
QQGEVLGSSPYTSSRCRRELYRLLLALLLAPSPRCPPPLACALQAFSLGQREDSLEVSSFCSEALVTCAALTHPRVPPLQ
SMGPACPAPAPAPPPEAPSPFRAPPFHPPGPMPSVGPMPSVGPMPSVGPMPPAGPMPPTRPGPPATANHLGLSVPGLVSV
PPRLLPGPENHRAGSNDDPVLAPSGTPPPAVPPDETFGGRVPRPAFVHYDKEEASDVEISLESDSDDSVVIVPEGLPPLP
PPPPTGTTPPPAAPAGPPTASPPVPAKEEPEELPAAPGPLPPPPPPPVPGPVALPPPQLVPEGPPGGGGPPALEEDLTVI
NINSSDEEEEEEEEEEEEEEEEEEEEEEDFEEEEEEEEEYFEEEEEEEEEFEEEFEEEEGELEEEEEDEDEEEEEDELEEL
EEVEFGPAGGPAEEGGPPPPSPAPALPPAQPPEAPPEPGVEPGLLLEVEEPGPEDEPGAEAAPTLAPEVLPSQGEGQREA
GSPPAGPPPQELVEEEPSAPPPLLEEGTENGGDKVPPPPETPAAEEMEAAAEAETAALQEKEQDDTAAMLADFIDCPPDD
EKPPAAPEPES
> Vicugna pacos [A0A6J3AAU9]
MAAAVLSGPSAGSAAGVPGGTGGLSAVASGPRLRLLLLESVSGLLQPRAGSTVAPVHPPVRSAPHLPGLMCLLRLHGTVG
GAQNLSAVGALVGLSNARLGSIKTRFEGLCLLSLLVAESPTEMFQQHCVSWLRSIQQVLQSQDPPPTMELAVAILRDLLR
YAAQLPTLFRDISMNHLPGLLTSLLGLRPECELSALEGMKACMTYFPRACGSLKGKLASFFLSRVDALSPQLQQLACECY
SRLPSLGAGFSQGLKHTESWEQELHSLLASLHSLLGALYEGAETAPVQHEGPAVETLLSPSEDGDAHVLLQLRQRFSGLA
RCLGLMLSSEFGAPVSVPVQEVLDVICRTLSISAKNIFSPQSLLGDGPLRLLLLPSLHLEALDLLSALILACGGRLLRFG
ALISRLLPQVLNAWSIGRDALSPGQERPYSTVRTKVYAVLELWVQVCGASAGVLQGGASGEALLTHLLSDIAPAADALKL
RSPRGSPDGGLQTGKPSAPKKLKLDVGEAIAPPSHRKGDSNANSDVCAAALRGLSRTILMCGPLIKEETHRRLHDLVLPL
VMGVQQGEVLGSSPYTSARCRLELYRLLLALLLAPSPRCPPPLACALQAFSLGQREDSLEVSSFCSEALVTCAALTHPRV
PPLQSMGPACPTPAPVPPPEAPSPFRAPAFHPPGPMPSVGPMPSASPMPSAGPMPSAGPVSSVGPMPSVGSMPPARPGPP
ATANHLGLSVPGLVSVPPRLLPGSENHRASSNEDPILAPSGTPPPTVPPDETFGGRVPRPAFVHYDKEEASDVEISLESD
SDDSVVIVPEGLPPLPPPPPSGTTPPPVAPAGPPTASPPVPAKEEPEELPATPGPLPPPPPSPVPGPVTLPPPQLVPEGT
PGGGGAPALEEDLTVININSSDEEEEEEEEEEEEEEEEEEEEEDFEEEEEDEEEYFEEEEEEEEEFEEEFEEEEGELEEEEE
EEDDEEEEELEEVEELEFGPAGGEVEEGGPPPPSLPPALPPAESPKVPPEPEPEPGLLLEVEEPGAEEGPGAETAPTLAP
EVLPPQGEVQREEGSPPAVPSPQELGEEEPSAPPTLLEEGAEGAGDQVPPPPEASAAEEMETETEAAGLQEKEQDDTAAM
LADFIDCPPDDENPPPAPEPDS
> Pteropus vampyrus (Large flying fox) [A0A6P3Q6V1]
MAAAVLSGPSAGSAAGVPGGTGGLSAVGSVPRLRLLLLESVSGLLQPRAGSAIAPVHPPVRSAPHLPGLMCLLRLHGTVG
GAQNLSAVGALVGLSNARLGSIKTRFEGLCLLSLLVGESSTEMFQQHCVSWLRSIQQVLQSQDPPPTMELAVAVLRDLLR
YAAQLPTLFRDISMNHLPGLLTSLLGLRPECELSALEGMKACMTYFPRACGSLKGKLASFFLSRVDALSPQLQQLACECY
SQVPSLGAGFSQGLKHTESWEQELHSLLASLHSLLGALYEGAEPAPMQYEGPGVEMLLSPSEDGDAHILLRLWQRFSGLA

RCLGLMLSSEFGAPVSVPVQEILDVICRTLSISAKNISLLGDGPLRLLLLPSIHLEALDLLSALILACRGRLLRFGALIS
RLLPQVLNAWSIGRDSLSPGQEKPYSAMRTKVYAILELWVQVCGASAGVLQGGASGEALLTHLLSDISPSADTLKLRSPR
GSPDGGLQMGKPSAPKKLKLDVGEAMAPPSHRKGDSNANSDVCAAALRGLSQTILMCGPLIKEETHRRLHDLVLPLVMGV
QQGEVLGSSPYTSSCCRRELYHLLLALLLAPSPRCPPPLACALQAFSLGQREDSLEVSSFCSEALVTCAALTHPRVPPLQ
SMGPTCPTPAPVPPPEAPSPFRAPPFHPPGPMPSAGPMPSAGPVPSVGSVPSVGSVPSVGSMPSAGPMPSAGPMPPTRPG
PPATANHLGLSVPGLVSLPPRLLPGPENHRAGSNEDPILAPSGTPPPTIPPDETFGGRVPRPAFVHYDKEEASDVEISLE
SDSDDSVVIVPEGLLPLPPPPPSGTTPPPVTPAGPPTASPPLPTQEEPEELPVAPGPLPPPPPPPVPAPVTLAPPQLVPE
GTPGGGGPPALEEDLTVININSSDEEEEEEEEEDEEEEEEEDFEEEEEDEEEYFEEEEEEEEEFEEEYEEEEGELEEDEE
EDEEEEEELEEVEELEFGSAGGEIEEGGPPPPNLPPALPPVESPKLHPEPEPEPEPEPEPGLLLEVEEPGAEEEHGAETA
PTLAPEVLPSQEELEREAESPPAGPPQELIEEEPCAPPALLEEGTEVGSDKVPPPPETSAAEEMQTETEATALQEKEQDD
TAAMLADFIDCPPDDEKLPPATEPDS
> Bos indicus [A0A6P5DHI5]
MAAAVLSGPSAGSAAAVPGGPGGLSAVGSGPRLRLMLLESVSGLLQPRTGSAVAPVHPPA
RSAPHLPGLMCLLRLHGTVGGAQNLSAVGALVGLSNARLGSIKTRFEGLCLLSLLVGESP
TEMFQQHCVSWLRSIQQILQSQDPPPTMELAVTVLKDLLRYAAQLPAVFRDISMNHLPGL
LTSLLGLRPECELSALEGMKACMTHFPRACGSLKGKLASFFLSRVDALSPQLQQLACECY
SRLPSLGAGFSQGLKHTDSWEQELRSLLASLHSLLGGLYEGAEAAPMQYESPGAETLLSS
SEDADAHTLLRLRQRFSGLARCLGLMLSSEFGAPVSVPVQDILDLICRTLSVSAKNVSLL
GDGPLRLLLLPSLHLEALDLLSALILACGARLLRFGALISRLLPQVLNAWSIGRENLGPG
QERPYSTVRTKVYAVLELWVQVCGASAGVLQGGASGEALLSHLLSDISPPADALRLRSPR
GSPDAGLQTGKPSAPKKLKLDVGEAIAPPSHRKGDSNANSDVCAAALRGLSRTILMCGPL
IKEETHRRLHELVLPLVMGVQQGEALGSSPYTSSHCRRELYHLLLALLLAPSPRCPPPLA
CALRAFSLGQREDSLEVSSFCSEALVTCAALTHPRVPPLQSVGPTCPAPAPVPPPEAPAP
FRAPAFHAPSPLPSAGPMPSAGPMPPVGPLPPTRPGPPATANHLGLSVPGLVSVPPRLLP
GPENHRAGSSEDPVLAPSGSPPPTIPPDETFGGRVPRPAFVHYDKEEPSDVEISLESDSD
DSVVIVPEGLPPPPPPPSSGTTPPPVAPAGPPAASPPVPAKDEPEELPAAPGPLPPPPPPP
VPGPVTLPPPQLVPEGTPGGGGPPALEEDMTVININSSDEEEEEEEEEEEEEDEEEDEEED
FEEDEEEEEEYFEEEEEEEEEEFEEEFEEEEGELEDEEDEEEDEELEELEEVEFGPAGGEV
EGGGPAPPSLPPALPPAESPKGPPEPGLEPGLLLEVEEPGTEEAPGPETAPMLAPEVLPS
QGEVEREGGSPPAGPPPQELVEEEPSGPPALLEEGAEGGGDKVSPPPEASAVEETEVEAA
ALPPEKEQGDTAAMLADFIDCPPDDEKPPPASEPDS
> Tursiops truncates [A0A6J3QLT7]
MAAAVLSGPSAGSAAGVPGGTGGLSAVNSGPRLRLLLLESVSGLLQPRAGSTVSPVHPPTRSVPHLPGLMCLLRLHGTVG
GAQNLSAVGALVGLSNARLGSIKTRFEGLCLLSLLVGESPTEMFQQHCVSWLRSIQQVLQSQDPPPTMELAVAVLGDLLR
YAAQLPTLFRDISMNHLPGLLTSLLGLRPESELSALEGMKACMTYFPRACGSLKGKLASFFLSRVDALSPQLQQLACECY
SRLPSLGAGFSQGLKHTDSWEQELHSLLTSLHSLLGALYAGADTAPMQYEGPGVETLLSPSEDGDAHVLLRLWQRFSGLA
RCLGLMLSSEFGAPVSVPVQEILDLICRTLSVSAKNISLLGDGPLRLLLLPSLHLEALDLLSALILACRGRLLRFGALIS
RLLPQVLNAWSIGRDSLSPGQERPYSTMRTKVYTVLELWVQVCGASAGVLQGGASGEALLTHLLSDISPPADALKLRSPR
GSPDGGLQTGKPSAPKKLKLDMGEALAPPSHRKGDSNANSDVCAAALRGLSRTVLMCGPLIKEETHRRLHDLVLPLVMGV
QQGEVLGSSPYTSSRCRRELYRLLLALLLAPSPRCPPPLACALQAFSLGQREDSLEVSAFCSEALVTCAALTHPRVPPLQ
TMGPACPTPAPVPPPEAPSPFRAPAFHPPGPMPSVGPMPSVGPMPSAGPMPSPGPVPPAGPMPSVGPMPPARPGPPATAN
HLGLSVPGLVSVSPRLLPGPENHRAGSNEDAVLAPSGTPPPTIPPDETFGGRVPRPAFVHYDKEEASDVEISLESDSDDS
VVIVPEGLPPLPPPPPSGTTPPPAAPVGPPTASPPVPAKEEPEELPAAPGPLPPPPPPPVPGPVALPPPQLVPEGTPSGG
GPPALEEDLTVININSSEEEEDDDEEDEDEEDFEEEEEEEEEEEYFEEEEEEEEEFEEEFEEEEGELEEEEEDEDEEEEV
EEVEFGPAGGEVEEEGPAPPSLPPALPPAASPKVQPQPEPEPGLLLEVEEPGAEEGPGAETAPTLAPEVLPSQGEVEREG
GSPPAVPSPQELVEEEPSVPPTLLEEGAEGGGDKVPTPPEASAAEEMETEAEAAALQEKEQDDTAAMLADFIDCPPDDEK
PPPATEPDS
> Lipotes vexillifer [A0A340X8S8]
MAAAVLSGPSAGSAAGVPGGTGGLSAVNSGPRLRLLLLESVSGLLQPRAGSTVSPVHPPVRSVPHLPGLMCLLRLHGTVG
GAQNLSAVGALVGLSNARLGSIKTRFEGLCLLSLLVGESPTEMFQQHCVSWLRSIQQVLQSQDPPPTMELAVAVLGDLLR
YAAQLPTLFRDISMNHLPGLLTSLLGLRPESELSALEGMKACMTYFPRACGSLKGKLASFFLSRVDALSPQLQQLACECY
SRLPSLGAGFSQGLKHTDSWEQELHSLLASLHSLLGALYAGADTAPMQYEGPGLETLLSPSEDGDAHVLLRLWQRFSGLA
RCLGLMLSSEFGAPVSVPVQEILDLICRTLSVSAKNISLLGDGPLRLLLLPSLHLEALDLLSALILACRGRLLRFGALIS
RLLPQVLNAWSIGRDSLSPGQERPYSTMRTKVYAVLELWVQVCGASAGVLQGGASGEALLTHLLSDISPPADALKLRSPR
GSPDGGLQTGKPSAPKKLKLDMGEAMAPPSHRKGDSNANSDVCAAALRGLSRTVLMCGPLIKEETHRRLHDLVLPLVMGV
QQGEVLGSSPYTSSRCRRELYRLLLALLLAPSPRCPPPLACALQAFSLGQREDSLEVSAFCSEALVTCAALTHPRVPPLQ
TMGPACPTPAPVPPPEAPSPFRAPAFHPPGPMPSVGPMPSVGPMPSAGPMPSPGPVPPAGPMPSVGPMPPARPGPPATAN
HLGLSVPGLVSVSPRLLPGPENHRAGSNEDAVLAPSGTPPPTIPPDETFGGRVPRPAFVHYDKEEASDVEISLESDSDDS
VVIVPEGLPPLPPPPPSGTTPPPAAPVGPPTASPPVPAKEEPEELPAAPGPLPPPPPPPVPGPVALPPPQLVPEGTPSGG
GPPALEEDLTVININSSEEDEDEDEDEEDEEDFEEEEEEEEEEEEEYFEEEEEEEEEFEEEFEEEEGELEEEEEDEDEEEE
VEEVEFGPAGGEVEGGGPAPPSLPPALPPTASPKVQPQPEPEPGLMLEVEEPGAEEGPGAETAPTLAPEALPSQGEVERE
RGSPPAVPPPQELVEEEPSVPPTLLEEGAEGGGDKVPPPPEASAAEEMETEAEALPEKEQDDTAAMLADFIDCPPDDEKP
PPATEPDS
> Neophocaena asiaeorientalis asiaeorientalis [A0A341AJN1]
MAAAVLSGPSAGSAAGVPGGTGGLSAVNSGPRLRLLLLESVSGLLQPRAGSTVSPVHHPTRSVPHLPGLMCLLRLHGTVG
GAQNLSAVGALVGLSNARLGSIKTRFEGLCLLSLLVGESPTEMFQQHCVSWLRSIQQVLQSQDPPPTMELAVAVLGDLLR
YAAQLPTLFRDISMNHLPGLLTSLLGLRPESELSALEGMKACMTYFPRACGSLKGKLASFFLSRVDALSPQLQQLACECY
SRLPSLGAGFSQGLKHTDSWEQELHSLLASLHSLLGALYAGADTAPMQYEGPGVETLLSPSEDGDAHVLLRLWQRFSGLA
RCLGLMLSSEFGAPVSVPVQEILDLICRTLSVSAKNISLLGDGPLRLLLLPSLHLEALDLLSALILACRGRLLRFGALIS
RLLPQVLNAWSIGRDSLSPGQERPYSTMRTKVYAVLELWVQVCGASAGVLQGGASGEALLTHLLSDISPPADALKLRSPR
GSPDGGLQTGKPSAPKKLKLDMGEALAPPSHRKGDSNANSDVCAAALRGLSRTVLMCGPLIKEETHRRLHDLVLPLVMGV
QQGEVLGSSPYTSSRCRRELYRLLLALLLAPSPRCPPPLACALQAFSLGQREDSLEVSAFCSEALVTCAALTHPRVPPLQ
TMGPACPTPAPVPPPEAPSPFRAPAFHPPGPMPSVGPMPSVGPMPSAGPMSSPGPVPPAGPMPSVGPIPPARPGPPATAN
HLGLSVPGLVSVSPRLLPGPENHRAGSNEDAVLAPSGTPPPTIPQDETFGGRVPRPAFVHYDKEEASDVEISLESDSDDS
VVIVPEGLPPLPPPPPSGTTPPPAAPVGPPTASPPVPAKEEPEELPAAPGPLPPPPPPPVPGPVALPPPQLVPEGTPSGG
GPPALEEDLTVININSSEEEEEDDEEDEDEEDFEEEEEEEEEEEYFEEEEEEEEEFEEEFEEEEGELEEEEEDEDEEEEV
EEVEFGPAGGEVEEGGPAPPSLPPALPPAASPKVQPQPEPEPGLLLEVEEPGAEEGPGAETAPTLAPEVLPSQGEVEREG

GSPPAVPPPQELVEEEPSVPPTLLEEGAEGGGDKVPPPPEASAAEEMEAEAAALQEKEQDDTAAMLADFIDCPPDDEKPP
PATEPDS
> Odocoileus virginianus texanus [A0A6J0Z4P6]
MAAAVLSGPSAGSAAGVPGGTGGLSAVGSGPRLRLMLLESVSGLLQPRTGSAVAPVHPPARSAPHLPGLMCLLRLHGTVG
GAQNLSAVGALVGLSNARLGSIKTRFEGLCLLSLLVGESPTEMFQQHCVSWLRSIQQILQSQDPPPTMELAVTVLKDLLR
YAAQLPAVSRDISMNHLPGLLTSLLGLRPECELSALEGMKACMTHFPRACGSLKGKLASFFLSRVDALSPQLQQLACECY
SRLPSLGAGFSQGLKHTDSWEQELRSLLASLHSLLGGLYEGAEAAPMQYESPGVETLLSPSEDADAHTLLQLRQRFSGLA
RCLGLMLSSEFGAPVTVPVQDILDLICRTLSVSAKNVSLLGDGPLRLLLLPSLHLEALDLLSALVLACGARLLRFGALIS
RLLPQVLNAWSIGRENLGPGQERPYSTVRTKVYAVLELWVQVCGASAGMLQGGASGEALLSHLLSDISPPADALRLRSPR
GSPDGGLQTGKPSAPKKLKLDVGEAMAPPSHRKGDSNANSDVCAAALRGLSRTILMCGPLIKEETHRRLHELVLPLVMGV
QQGEALGSSPYTSSHCRRELYRLLLALLLAPSPRCPPPLACALRAFSLGQREDSLEVSSFCSEALVTCAALTHPRVPPLQ
SVGPTCPAPAPVPPPEAPAPFRAPAFHTPGPLPSAGTMPSAGPMPPAGPLPPTRPGPPATANHLGLSVPGLVSVPPRLLP
GPENHRAGSSEDPVLAPSGSPPPTVPPDETFGGRVPRPAFVHYDKEEASDVEISLESDSDDSVVIVPEGLPPPPPPPSGT
TPPPVAPAGPPAASPPVPAKDEPEELPAAPGPLPPPPPPPVPGPVTLPPPQLVPEGTPGGGGPPALEEDLTVININSSDE
EEEEDEEEEEEDEEDEEEDFEEDEEEEEEYFEEEEEEEEFEEEFEEEEGELEDEEDEEEDEELEELEEVEFGPAAGDGEG
GGPVPPSLPPPALPPAESPKGPPEPGLEPGLLLEVEEPGTEEAPGPETAPTLAPEVLPPQGETEREAGSPPAGPPPQELV
EAEPSGPPALLEEGAEGGGEKVSPPEEAAAAAEAAALPPEKGDTAAMLADFIDCPPDDEKPPPASEPDS
> Bison bison bison [A0A6P3I1Z8]
MLSVAFPFSFHLVNYNSQGLLRMLAARPHRVTTCGKTEYRKFPQSKAIQTCPPSLHCCGLFNFFYKNFLSVCGVLGDDGR
TSGVLQTGQPWTLYLPIPLRHLSGSGENDLNLSAVGALVGLSNARLGSIKTRFEGLCLLSLLVGESPTEMFQQHCVSWLR
SIQQILQSQDPPPTMELAVTVLKDLLRYAAQLPAVFRDISMNHLPGLLTSLLGLRPECELSALEGMKACMTHFPRACGSL
KGKLASFFLSRVDALSPQLQQLACECYSRLPSLGAGFSQGLKHTDSWEQELRSLLASLHSLLGGLYEGAEAAPMQYESPG
AETLLSSSEDADAHTLLRLRQRFSGLARCLGLMLSSEFGAPVSVPVQDILDLICRTLSVSAKNVSLLGDGPLRLLLLPSL
HLEALDLLSALILACGARLLRFGALISRLLPQVLNAWSIGRENLGPGQERPYSTVRTKVYAVLELWVQVCGASAGVLQGG
ASGEALLSHLLSDISPPADALRLRSPRGSPDAGLQTGKPSAPKKLKLDVGEAIAPPSHRKGDSNANSDVCAAALRGLSRT
ILMCGPLIKEETHRRLHELVLPLVMGVQQGEALGSSPYTSSHCRRELYHLLLALLLAPSPRCPPPLACALRAFSLGQRED
SLEVSSFCSEALVTCAALTHPRVPPLQSVGPTCPAPAPVPPPEAPAPFRAPAFHAPSPLPSAGPMPSAGPMPPVGPLPPT
RPGPPATANHLGLSVPGLVSVPPRLLPGPENHRAGSSEDPVLAPSGSPPPTIPPDETFGGRVPRPAFVHYDKEEPSDVEI
SLESDSDDSVVIVPEGLPPPPPPSSGTTPPPVAPAGPPAASPPVPAKDEPEELPAAPGPLPPPPPPPVPGPVTLPPPQLV
PEGTPGGGGPPALEEDMTVININSSDEEEEEEEEEEEEEDEEEDEEEDFEEDEEEEEEYFEEEEEEEEEFEEEFEEEEGEL
EDEEDEEEDEELEELEEVEFGPAGGEVEGGGPAPPSLPPALPPAESPKGPPEPGLEPGLLLEVEEPGTEEAPGPETAPML
APEVLPSQGEVEREGGSPPAGPPPQELVEEEPSGPPALLEEGAEGGGDKVSPPPEASAVEETEVEAAALPPEKEQGDTAA
MLADFIDCPPDDEKPPPASEPDS
> Balaenoptera acutorostrata scammony [A0A452CLR0]
MDPVFAYPTPPPIGAWGKLDLNLSAVGALVGLSNARLGSIKTRFEGLCLLSLLVGESPTEMFQQHCVSWLRSIQQVLQSQ
DPPPTMELAVAVLRDLLRYAAQLPTLFRDISMNHLPGLLTSLLGLRPESELSALEGMKACMTYFPRACGSLKGKLASFFL
SRVDALSPQLQQLACECYSRLPSLGAGFSQGLKHTDSWEHELHSLLASLHSLLGALYEGAETAPVQYEGPGVETLLSPSE
DGDAHVLLRLWQRFSGLARCLGLMLSSEFGAPVSVPVQDILDLICRTLSVSAKNISLLGDGPLRLLLLPSLHLEALDLLS
ALVLACGGRLLRFGALISRLLPQVLNAWSIGRDSLSPGQERPYSTMRTKVYAVLELWVQVCGASAGVLQGGASGEALLTH
LLSDISPPADALKLRSPRGSPDGGLQTGKPSAPKKLKLDMGEAMAPPSHRKGDSNANSDVCAAALRGLSRTILMCGPLIK
EETHRRLHDLVLPLVMGVQQGEVLGNSPYTSSRCRRELYRLLLALLLAPSPRCPPPLACALQAFSLGQREDSLEVSTFCS
EALVTCAALTHPRVPPLQTMGPACPTPAPVPPPEAPSPLRAPAFHPPGPVPSVGPVPSVGPMPSAGPMPSPGPVPPAGPM
PSVGPMPPTRPGPPATANHLGLSVPGLVSVSPRLLPGPENHRAGSNEDAVLAPSGTPPPTIPPDETFGGRVPRPAFVHYD
KEEASDVEISLESDSDDSVVIVPEGLPPLPPPPPXXXXXXXXXXXXXXXXXXXXXXXXXXXXXAGPPTASPPVPAKEEPE
ELPAAPGPLPPPPPPPVPGPVALPPPQLVPEGTPSGGGPPALEEDLTVININSSDEEEEEEEEEEEEEEEEEEEEDFEEEEE
DEEEYFEEEEEEEEEFEEEFEEEEGELEEEEEDEDEEEEVEEVEFGPAGGEGEEGGPAPPSLPPALPPAASPKVQPQPEP
EPGLLLEVEEPGAEEGPGAETAPTLAPEVLPSQGEVEREGGSPPAVPPPQELVEEEPSVPPTLLEEGAEGGGDKVPPPPE
ASAAEEMETEVEAAALQEKEQDDTAAMLADFIDCPPDDEKPPPATEPDA
>Delphinapterus leucas [A0A2Y9Q2R7]
MNCFLCSLATTHLSLTFLTRFEGLCLLSLLVGESSTEMFQQHCVSWLRSIQQVLQSQDPPPTMELAVAVLGDLLRYAAQL
PTLFRDISMNHLPGLLTSLLGLRPESELSALEGMKACMTYFPRACGSLKGKLASFFLSRVDALSPQLQQLACECYSRLPS
LGAGFSQGLKHTDSWEQELHSLLASLHSLLGALYAGADTAPMQYEGPGVETLLSPSEDGDAHVLLRLWQRFSGLARCLGL
MLSSEFGAPVSVPVQEILDLICRTLSVSAKNISLLGDGPLRLLLLPSLHLEALDLLSALILACRGRLLRFGALISRLLPQ
VLNAWSIGRDSLSPGQERPYSTMRTKVYAVLELWVQVCGASAGVLQGGASGEALLTHLLSDISPPADALKLRSPRGSPDG
GLQTGKPSAPKKLKLDMGEALAPPSHRKGDSNANSDVCAAALRGLSRTVLMCGPLIKEETHRRLHDLVLPLVMGVQQGEV
LGSSPYTSSRCRRELYRLLLALLLAPSPRCPPPLACALQAFSLGQREDSLEVSAFCSEALVTCAALTHPRVPPLQTMGPA
CPTPAPVPPPEAPSPFRAPAFHPPGPMPSVGPMPSVGPMPSAGPMPSPGPVPPAGPMPSVGPMPPARPGPPATANHLGLS
VPGLVSVSPRLLPGPENHRAGSNEDAVLAPSGTPPPTLPPDETFGGRVPRPAFVHYDKEEASDVEISLESDSDDSVVIVP
EGLPPLPPPPPSGTTPPPAALVGPPTASPPVPAKEEPEELPAAPGPLPPPPPPPVPGPVALPPPQLVPEGTPSGGGPPAL
EEDLTVININSSEEEEEDDEEDEDEEDFEEEEEEEEEEEEYFEEEEEEEEEFEEEFEEEEGELEEEEEDEDEEEEVEEVEF
GPAGGEVEEGGPAPPSLPPALPPAASPKVQPQPEPEPGLLLEVEEPGAEEGPGAETAPTLAPEVLPSQGEVEREGGSPPA
VPPPQELVEEEPSVPPTLLEEGAEGGGDKVPPPPEASAAEEMETEAEAAALQEKEQDDTAAMLADFIDCPPDDEKPPPAT
EPDS
> Leptonychotes weddellii [A0A7F8RPS3]
MELAVAVLRDLLRYAAQLPTLFRDISMNHLPGLLTSLLGLRPECELSALEGMKACMTYFPRACGSLKGKLASFFLSRVDA
LSPQLQQLACECYSRLPSLGAGFSQGLKHTESWQQELRSLLASLHSLLGALYEGAETAPVQYEGPGVEVLLTPAEDGDAH
ALLRLRQRFCGLARCLGLMLSSEFGAPVSVPVQEILDVICRTLSISAKNISLLGDGPLRLLLLPSIHLDALDLLSALILA
CGGRLLRFGGLVSRLLPQVLNAWNMGRDTLSPGQERPYSTVRTKVYAVLELWVQVCGASAGVLQGGASGEALLSHLLSDI
APPADALKLRSPRGSPDGGLQSGKPSAPKKLKLDLGEAMAPPSHRKGDSNANSDVCAAALRGLSRTVLMCGPLIKEETHR
RLHDLVVPLVMGLQRGETLGSSPYTSSRCRRELYRLLLALLLAPSPRCPPPLACALHAFSLGQREDSLEVSSFCSEALVV
CAALTHPRVPPLQPAGPPCPTPAPVPPPEAPSPFRAPPFHPPAGPLPPARPGPPATANHLGLSVPGLVSVPPRLLPGPEN
HRAGSNEDPVLAPSGTPPPALPPDETFGGRVPRPAFVHYDKEEASDVEISLESDSDDSVVIVPEGLPPLPPPPPSGTTPP
PVAPAGPPTASPPVPAKEEPEELPAAPGPLPPPPPPPVPGPVALPPPQLVPEGTPGGGGPPTLEEDLTVININSSDEEEE
EEEEEEEEEEEEEEEEDFEEEEEDEEEYFEEEEEEEEEFEEEFEEEEGELEEEEEEEEDEEDEEELEELEEAEFGPAGGPA
EEGGPAPPSPAPALPPAQSPTVQPEPEGEPGLLLEVEEPGAEEEPGAQAAPTLAPEVLPSQGEGPRATGSPPAGPPPQEL
VEEEPLAPPTLLEEGTESGGDRVPPLPETPAAEDGEAEAEAESAALQEKEQDDTAAMLADFIDCPPDDEKPPPAPEPDS
> Phascolarctos cinereus [A0A6P5ILR5]

MMGGAQNLSAVGALVGLSNARLGSIKTRFEGLCLLSLLVAESPTETFQQHSVSWLRSIQHVLQSQDPAPTMELAVVILRD
LLRYSAQLPELSRDISTNHLPGLLTSLLGLKPECELSALEGMKACMTYFPRACGSLRGKLASFFLSRVEALSPQLQQLAC
ECYARLPALGAGFSQGLKHTESWEQELHCLLASLHGLLGALYEGAETAPIQYEGPGVELLLPPPADNDVHGLLQLRQRFS
GLARCLGLMLSSDFGAPVSVPVQDILDIICRTLSVSGKNISWLGDGPLRLLLLPSIHLEALDLLSALILSCGSRLVRFGG
LICRLLPQVLNTWSAGRDPLPPGQERPYSAVRAKVYSVLELWVQTCGAGAGVLQGGAQSEALLTHLLSDIFPPADALKLR
GPRVTQDGGLQSGKPSAPKKLKLDSGETVVPPLHRKGDSNANSDVCTTALRGLSRTILMCGPLIKEETHRRLHELTLPMV
MGTQQGEIPGGSPYTSARCRRELYRLLLALLLAPAPQCPPPLTCALQAFSLGQHEENLEVSSFCSEALVTCAALAHPRVP
PLQSPGPSSPPTAPPTQPEPPSPFRAPPFHPPGPRPPVSATANHVGLPVPGLVSVPPRLLPGPENHRAALSEDSAPAPAG
TSPPSQPPDEAFGGRPPRPAFVHYEKEETSDVEISLESDSDDSVVIVPEGLLPLPPAPPQGGTPPPAPPVVATPTSPPLP
PKEEPEELPPPPGPLPPPPGPGALPPPQLVAEGTPGGAPAPLEEDLTVININSSDEEEEEEEEEEEEEEEDFPEEEEEEE
EEEEYFEEEEEEEEEFEEEFEEEEEGELEEEEEEEEEEEEEEEELEEVEFEPAEGEVEDRGAPPPDSSPPPPPSEPPKEEP
EPGLLMEVEEPSAEDEKEGGGAPPLALEASPPQGEREEEGRTVVPEAPPAEELAGNEPPALSGEGTDGRGSQEPPPAKAA
EAPATETEMAPMGEKEQDDTAAMLADFIDCPPDEEKPTPPPEPDS
> Geotrypetes seraphini [A0A6P8PX50]
MAAVATHEVWEQLVESVSGLLRRGDRPGATTPAPLLLLPGLVRGLREHGGLRAQSSTAIGGLTGISSARLNSIKTRFEGL
CLLSLLAAESPTDHFQQHCVSWLRSVQHIIQSQDLPPTMELAVLVLHDLLKYSSLLPALSREISMNLIPGLLTSLLDLKP
ECQAPALEGMKACMTFYPRACGSLRGKLASFFMNRLETESPQIQQLACECYTLLPSLGSGFAQGVKHTECWEHQLQCLLA
TLHATVGQLYEGAETELLHYEGAGVELLPPVADDGEALHVLRLKHRFSGICKCLCLLLSSSFSVPVTVPVQDVLDAICRV
LSISSKNISWLGDGPLKTLLLPSVHAEVLDLLAALILACGSRLVRFAGLFCRLFAQVLTLWSGKRDDIIPGQEKPYSAVR
TKVYQVLEMWIKVCGASSGVLQGPGHHCDVLLAHLTSDISPPSDTIKLRVGRLGAEISLNNGKPGSKKQKMDLGDGSQLQ
SHRKQDVNANSDTCIMALRVLSRAILLCGTLIKEDTHRRLHELVMPLLIRLQQSALPTTSPYTSSECRKELYRLLLCLLL
TPHPRWPPPLHCAVCIFSQGQKENNMKVSSFCAEALVICNCIVHPRVPSLQLPVTSSATNTAATDLLKSSVLGADMPSTF
RHSPAPTSLRSQTNHLTIPLQGLASGQPLASRLQSSMVSNLTLPSVPPCTVGVENHQLDEHMEVESRVHSMPSPSEETFG
GKTHRPVFIHYEKEEASDVEISLDSDSDDSVVIVPEGLLPKQTLSPPTKVVEEAEESSKEHAITVTVATTVNATISVPAS
PASSNQILAPTAALPPPPPPLPPPPPPPLQLVAETPASAVEEDLTVINLNSSEEEEEEEEEEEEDFAEDEYFDDEEDFEEE
EYDGEMMDEEEEEDEEGEGQLSLDAIDMDEEEDLDDLEEDLECEDEVMTEEEEEEEEEDEEDIMPCIEEEPEEELRVGFVE
KEALETEGEPAAQADSEAREEKSGAGEQIDESPPRLSPVREGTQEAKEEEEVATEADTDLLMTVESDSAEREEEEEEEVK
ETPLLVQETQTPQLPEETSPPPPPPPPPPPPPPSSPPPPPPPPPPPPLPPPLPPPLPTESEECEKIQMEERSDQEVETSPAQ
TSTEERIGELVTEKDIKPDKEEETPQSTEKKAEAEIDDTAAMLADFIDCPPDEDKTSPDISS
> Delphinapterus leucas [A0A2Y9Q244]
MAAAVLSGPSAGSAAGVPGGTGGLSAVNSGPRLRLLLLESVSGLLQPRAGSTVSPVHHPTRSVPHLPGLMCLLRLHGTVG
GAQNLSAVGALVGLSNARLGSIKTRFEGLCLLSLLVGESSTEMFQQHCVSWLRSIQQVLQSQDPPPTMELAVAVLGDLLR
YAAQLPTLFRDISMNHLPGLLTSLLGLRPESELSALEGMKACMTYFPRACGSLKGKLASFFLSRVDALSPQLQQLACECY
SRLPSLGAGFSQGLKHTDSWEQELHSLLASLHSLLGALYAGADTAPMQYEGPGVETLLSPSEDGDAHVLLRLWQRFSGLA
RCLGLMLSSEFGAPVSVPVQEILDLICRTLSVSAKNISLLGDGPLRLLLLPSLHLEALDLLSALILACRGRLLRFGALIS
RLLPQVLNAWSIGRDSLSPGQERPYSTMRTKVYAVLELWVQVCGASAGVLQGGASGEALLTHLLSDISPPADALKLRSPR
GSPDGGLQTGKPSAPKKLKLDMGEALAPPSHRKGDSNANSDVCAAALRGLSRTVLMCGPLIKEETHRRLHDLVLPLVMGV
QQGEVLGSSPYTSSRCRRELYRLLLALLLAPSPRCPPPLACALQAFSLGQREDSLEVSAFCSEALVTCAALTHPRVPPLQ
TMGPACPTPAPVPPPEAPSPFRAPAFHPPGPMPSVGPMPSVGPMPSAGPMPSPGPVPPAGPMPSVGPMPPARPGPPATAN
HLGLSVPGLVSVSPRLLPGPENHRAGSNEDAVLAPSGTPPPTLPPDETFGGRVPRPAFVHYDKEEASDVEISLESDSDDS
VVIVPEGLPPLPPPPPSGTTPPPAALVGPPTASPPVPAKEEPEELPAAPGPLPPPPPPPVPGPVALPPPQLVPEGTPSGG
GPPALEEDLTVININSSEEEEEDDEEDEDEEDFEEEEEEEEEEEYFEEEEEEEEEFEEEFEEEEGELEEEEEDEDEEEEV
EEVEFGPAGGEVEEGGPAPPSLPPALPPAASPKVQPQPEPEPGLLLEVEEPGAEEGPGAETAPTLAPEVLPSQGEVEREG
GSPPAVPPPQELVEEEPSVPPTLLEEGAEGGGDKVPPPPEASAAEEMETEAEAAALQEKEQDDTAAMLADFIDCPPDDEK
PPPATEPDS
> Balaenoptera acutorostrata scammoni [A0A452CLT6]
MAAAVLSGPSAGSAAGVPGGTGGLSAVNSGPRLRLLLLESVSGLLQPRAGSTVSPVHPPVRSVPHLPGLMCLLRLHGTVG
GAQNLSAVGALVGLSNARLGSIKTRFEGLCLLSLLVGESPTEMFQQHCVSWLRSIQQVLQSQDPPPTMELAVAVLRDLLR
YAAQLPTLFRDISMNHLPGLLTSLLGLRPESELSALEGMKACMTYFPRACGSLKGKLASFFLSRVDALSPQLQQLACECY
SRLPSLGAGFSQGLKHTDSWEHELHSLLASLHSLLGALYEGAETAPVQYEGPGVETLLSPSEDGDAHVLLRLWQRFSGLA
RCLGLMLSSEFGAPVSVPVQDILDLICRTLSVSAKNISLLGDGPLRLLLLPSLHLEALDLLSALVLACGGRLLRFGALIS
RLLPQVLNAWSIGRDSLSPGQERPYSTMRTKVYAVLELWVQVCGASAGVLQGGASGEALLTHLLSDISPPADALKLRSPR
GSPDGGLQTGKPSAPKKLKLDMGEAMAPPSHRKGDSNANSDVCAAALRGLSRTILMCGPLIKEETHRRLHDLVLPLVMGV
QQGEVLGNSPYTSSRCRRELYRLLLALLLAPSPRCPPPLACALQAFSLGQREDSLEVSTFCSEALVTCAALTHPRVPPLQ
TMGPACPTPAPVPPPEAPSPLRAPAFHPPGPVPSVGPVPSVGPMPSAGPMPSPGPVPPAGPMPSVGPMPPTRPGPPATAN
HLGLSVPGLVSVSPRLLPGPENHRAGSNEDAVLAPSGTPPPTIPPDETFGGRVPRPAFVHYDKEEASDVEISLESDSDDS
VVIVPEGLPPLPPPPPXXXXXXXXXXXXXXXXXXXXXXXXXXXXXXAGPPTASPPVPAKEEPEELPAAPGPLPPPPPPPVP
GPVALPPPQLVPEGTPSGGGPPALEEDLTVININSSDEEEEEEEEEEEEEEEEEEDFEEEEEDEEEYFEEEEEEEEEEFEE
EFEEEEGELEEEEEDEDEEEEVEEVEFGPAGGEGEEGGPAPPSLPPALPPAASPKVQPQPEPEPGLLLEVEEPGAEEGPG
AETAPTLAPEVLPSQGEVEREGGSPPAVPPPQELVEEEPSVPPTLLEEGAEGGGDKVPPPPEASAAEEMETEVEAAALQE
KEQDDTAAMLADFIDCPPDDEKPPPATEPDA
> Dipodomys ordii [A0A1S3EZN2]
MAAAVLSGPTAGSSVGVPGGTGGLSAMSSGPRLRLLLLESVSGLLQPRPGPPAPPVHPPVHWAPHFPGLMCLLRLHGSVG
GAQNLSALGTLVSLSNAHLGSMRTRFEGLCLLSLLVGESPTEFFQQHCVSWLRCIQHVLQSQDSPPTMELAVAVLRDLLR
YAAQLPTLFRDISTNHLPGLLTSLLGLRPECEHAALEGMKACMTYFPRACGSLKGKLASFFLSRVDALSPQLQQLACECY
SRLPSLGAGFSQGLKHTENWEQELHSLLTSLHSLLGALYEGSETAPVQSEDSGMETLLSHLDDVDTHALFRLQQRFSGLA
HCMALMLSSEFGAPVSVPVQEVLDLICRILSVNGKNISLLGDGPLRLLLLPSIHLEALDLLSSLILACGGRLLRFGTLIN
RLLPQVLNVWSIGRDTLSPGQERPYSTIRIKVYAVLELWVKVCGASAGVLQGGAPGEALLTHLLSDISPPTDTLKLRSPR
GNSDGGLQSGKPSAPKKLKLDMGDAMAPPSHRKGDSNANSDVCAAALRGLSRTILMCGPLIKEETHRRLHDLVLPLLMSL
QQGEVLGSSPYNSSCCRRELYRLLLALLLAPSPRCPPPLACALQAFSFGQQEDSLEVSSFCSEALVTCAALTHPRVPPLQ
TMGPTCATSAPVPPPEAPSSFRAPPFHPPGPMPSVGPMPTPGPISSAGPLPSAGPMPSAGPMSSTGPIPSAGPLPSAGPI
PSTRPGPPATTNHLGLSVPGLVSVPPRLLPGPENHRAGPSEDPVLASSGTPPPTIPPDETFGGRVPRPAFVHYDKEEASD
VEISLESDSDDSVVIVPEGLPPLPPPPPTSTPPPSVAPTGPPAASPPVPAKEEPEELSATPGSLPPPPPPPPPPPIAGSAT
LPPPQLVPEGAPGGGGPPALEEDLTVININSSDEEEEEEEEEEEEEEEDDEEEEDFEEEDDDEEEYFEEEEEEEEEFEEEFEE
EEGELEEEEEEEEEEEEEEELEEVEDLEFGPAGGEVEEGGPPPPTLPPALPPPESPKVQPEPEPEPEPGLLLEVEEPGTEEEP
GAETAPTLAPEVLPSQGEVEGEGGSPTVGPPPQELVEEPSAHPPLLEEETEGGSDKVPPPPETSEVEEEMETEAETAALQ
EKEQDDTAAMLADFIDCPPDDEKPPPTMESDS

> Tursiops truncates [A0A6J3QMB5]
MAAAVLSGPSAGSAAGVPGGTGGLSAVNSGPRLRLLLLESVSGLLQPRAGSTVSPVHPPTRSVPHLPGLMCLLRLHGTVG
GAQNLSAVGALVGLSNARLGSIKTRFEGLCLLSLLVGESPTEMFQQHCVSWLRSIQQVLQSQDPPPTMELAVAVLGDLLR
YAAQLPTLFRDISMNHLPGLLTSLLGLRPESELSALEGMKACMTYFPRACGSLKGKLASFFLSRVDALSPQLQQLASPMQ
YEGPGVETLLSPSEDGDAHVLLRLWQRFSGLARCLGLMLSSEFGAPVSVPVQEILDLICRTLSVSAKNISLLGDGPLRLL
LLPSLHLEALDLLSALILACRGRLLRFGALISRLLPQVLNAWSIGRDSLSPGQERPYSTMRTKVYTVLELWVQVCGASAG
VLQGGASGEALLTHLLSDISPPADALKLRSPRGSPDGGLQTGKPSAPKKLKLDMGEALAPPSHRKGDSNANSDVCAAALR
GLSRTVLMCGPLIKEETHRRLHDLVLPLVMGVQQGEVLGSSPYTSSRCRRELYRLLLALLLAPSPRCPPPLACALQAFSL
GQREDSLEVSAFCSEALVTCAALTHPRVPPLQTMGPACPTPAPVPPPEAPSPFRAPAFHPPGPMPSVGPMPSVGPMPSAG
PMPSPGPVPPAGPMPSVGPMPPARPGPPATANHLGLSVPGLVSVSPRLLPGPENHRAGSNEDAVLAPSGTPPPTIPPDET
FGGRVPRPAFVHYDKEEASDVEISLESDSDDSVVIVPEGLPPLPPPPPSGTTPPPAAPVGPPTASPPVPAKEEPEELPAA
PGPLPPPPPPPVPGPVALPPPQLVPEGTPSGGGPPALEEDLTVININSSEEEEDDDEEDEDEEDFEEEEEEEEEEEYFEE
EEEEEEEFEEEFEEEEGELEEEEEDEDEEEEVEEVEFGPAGGEVEEEGPAPPSLPPALPPAASPKVQPQPEPEPEPGLLLEV
EEPGAEEGPGAETAPTLAPEVLPSQGEVEREGGSPPAVPSPQELVEEEPSVPPTLLEEGAEGGGDKVPTPPEASAAEEME
TEAEAAALQEKEQDDTAAMLADFIDCPPDDEKPPPATEPDS
> Vicugna pacos [A0A6J3AAJ0]
MAAAVLSGPSAGSAAGVPGGTGGLSAVASGPRLRLLLLESVSGLLQPRAGSTVAPVHPPVRSAPHLPGLMCLLRLHGTVG
GAQNLSAVGALVGLSNARLGSIKTRFEGLCLLSLLVAESPTEMFQQHCVSWLRSIQQVLQSQDPPPTMELAVAILRDLLR
YAAQLPTLFRDISMNHLPGLLTSLLGLRPECELSALEGMKACMTYFPRACGSLKGKLASFFLSRVDALSPQLQQLACECY
SRLPSLGAGFSQGLKHTESWEQELHSLLASLHSLLGALYEGAETAPVQHEGPAVETLLSPSEDGDAHVLLQLRQRFSGLA
RCLGLMLSSEFGAPVSVPVQEVLDVICRTLSISAKNIFSPQSLLGDGPLRLLLLPSLHLEALDLLSALILACGGRLLRFG
ALISRLLPQVLNAWSIGRDALSPGQERPYSTVRTKVYAVLELWVQVCGASAGVLQGGASGEALLTHLLSDIAPAADALKL
RSPRGSPDGGLQTGKPSAPKKLKLDVGEAIAPPSHRKGDSNANSDVCAAALRGLSRTILMCGPLIKEETHRVSSFCSEAL
VTCAALTHPRVPPLQSMGPACPTPAPVPPPEAPSPFRAPAFHPPGPMPSVGPMPSASPMPSAGPMPSAGPVSSVGPMPSV
GSMPPARPGPPATANHLGLSVPGLVSVPPRLLPGSENHRASSNEDPILAPSGTPPPTVPPDETFGGRVPRPAFVHYDKEE
ASDVEISLESDSDDSVVIVPEGLPPLPPPPPSGTTPPPVAPAGPPTASPPVPAKEEPEELPATPGPLPPPPPSPVPGPVT
LPPPQLVPEGTPGGGGAPALEEDLTVININSSDEEEEEEEEEEEEEEEEEEEEEDFEEEEEDEEEYFEEEEEEEEEEFEEEFE
EEEGELEEEEEEEEDDEEEEELEEVEELEFGPAGGEVEEGGPPPPSLPPALPPAESPKVPPEPEPEPEPGLLLEVEEPGAEEG
PGAETAPTLAPEVLPPQGEVQREEGSPPAVPSPQELGEEEPSAPPTLLEEGAEGAGDQVPPPPEASAAEEMETETEAAGL
QEKEQDDTAAMLADFIDCPPDDENPPPAPEPDS
> Bison bison bison [A0A6P3HSW0]
MLSVAFPFSFHLVNYNSQGLLRMLAARPHRVTTCGKTEYRKFPQSKAIQTCPPSLHCCGLFNFFYKNFLSVCGVLGDDGR
TSGVLQTGQPWTLYLPIPLRHLSGSGENDLNLSAVGALVGLSNARLGSIKTRFEGLCLLSLLVGESPTEMFQQHCVSWLR
SIQQILQSQDPPPTMELAVTVLKDLLRYAAQLPAVFRDISMNHLPGLLTSLLGLRPECELSALEGMKACMTHFPRACGSL
KGKLASFFLSRVDALSPQLQQLACECYSRLPSLGAGFSQGLKHTDSWEQELRSLLASLHSLLGGLYEGAEAAPMQYESPG
AETLLSSSEDADAHTLLRLRQRFSGLARCLGLMLSSEFGAPVSVPVQDILDLICRTLSVSAKNVSLLGDGPLRLLLLPSL
HLEALDLLSALILACGARLLRFGALISRLLPQVLNAWSIGRENLGPGQERPYSTVRTKVYAVLELWVQVCGASAGVLQGG
ASGEALLSHLLSDISPPADALRLRSPRGSPDAGLQTGKPSAPKKLKLDVGEAIAPPSHRKGDSNANSDVCAAALRGLSRT
ILMCGPLIKEETHRRLHELVLPLVMGVQQGEALGSSPYTSSHCRRELYHLLLALLLAPSPRCPPPLACALRAFSLGQRED
SLEVSSFCSEALVTCAALTHPRVPPLQSVGPTCPAPAPVPPPEAPAPFRAPAFHAPSPLPSAGPMPSAGPMPPVGPLPPT
RPGPPATANHLGLSVPGLVSVPPRLLPGPENHRAGSSEDPVLAPSGSPPPTIPPDETFGGRVPRPAFVHYDKEEPSDVEI
SLESDSDDSVVIVPEGLPPPPPPSSGTTPPPVAPAGPPAASPPVPAKDEPEELPAAPGPLPPPPPPPVPGPVTLPPPQLV
PEGTPGGGGPPALEEDMTVININSSDEEEEEEEEEEEEEDEEEDEEEDFEEDEEEEEEYFEEEEEEEEEFEEEFEEEEGEL
EDEEDEEEDEELEELEEVEFGPAGGEVEGGGPAPPSLPPALPPAESPKGPPEPGLEPGLLLEVEEPGTEEAPGPETAPML
APEVLPSQGEVEREGGSPPAGPPPQELVEEEPSGPPALLEEGAEGGGDKVSPPPEASAVEETEVEAAALPPEKGDTAAML
ADFIDCPPDDEKPPPASEPDS
> Bos indicus [A0A6P5DF39]
MLSVAFPFSFHLVNYNSQGLLRMLAARPHRVTTCGKTEYRKFPQSKAIQTCPPSLHCCGLFNFFYKNFLSVCGVLGDDGR
TSGVLQTGQPWTLCLPIPLRHLSXPGENDLAKXSEVKEQVSGVRGMMAVPPCEDDQGVQIDCYCGENLSAVGALVGLSNA
RLGSIKTRFEGLCLLSLLVGESPTEMFQQHCVSWLRSIQQILQSQDPPPTMELAVTVLKDLLRYAAQLPAVFRDISMNHL
PGLLTSLLGLRPECELSALEGMKACMTHFPRACGSLKGKLASFFLSRVDALSPQLQQLACECYSRLPSLGAGFSQGLKHT
DSWEQELRSLLASLHSLLGGLYEGAEAAPMQYESPGAETLLSSSEDADAHTLLRLRQRFSGLARCLGLMLSSEFGAPVSV
PVQDILDLICRTLSVSAKNVSLLGDGPLRLLLLPSLHLEALDLLSALILACGARLLRFGALISRLLPQVLNAWSIGRENL
GPGQERPYSTVRTKVYAVLELWVQVCGASAGVLQGGASGEALLSHLLSDISPPADALRLRSPRGSPDAGLQTGKPSAPKK
LKLDVGEAIAPPSHRKGDSNANSDVCAAALRGLSRTILMCGPLIKEETHRRLHELVLPLVMGVQQGEALGSSPYTSSHCR
RELYHLLLALLLAPSPRCPPPLACALRAFSLGQREDSLEVSSFCSEALVTCAALTHPRVPPLQSVGPTCPAPAPVPPPEA
PAPFRAPAFHAPSPLPSAGPMPSAXPMPPVGPLPPTRPGPPATANHLGLSVPGLVSVPPRLLPGPENHRAGSSEDPVLAP
SGSPPPTIPPDETFGGRVPRPAFVHYDKEEPSDVEISLESDSDDSVVIVPEGLPPPPPPSSGTTPPPVAPAGPPAASPPV
PAKDEPEELPAAPGPLPPPPPPPVPGPVTLPPPQLVPEGTPGGGGPPALEEDMTVININSSDEEEEEEEEEEEEEDEEEDE
EEDFEEDEEEEEEYFEEEEEEEEEFEEEFEEEEGELEDEEDEEEDXELEELEEVEFGPAGGEVEGGGPAPPSLPPALPPA
ESPKGPPEPGLEPGLLLEVEEPGTEEAPGPETAPMLAPEVLPSQGEVEREGGSPPAGPPPQELVEEEPSGPPALLEEGAE
GGGDKVSPPPEASAVEETEVEAAALPPEKEQGDTAAMLADFIDCPPDDEKPPPASEPDS
> Phyllostomus discolour [A0A7E6ECF7]
MAAAVLSGPSAGSAAGASGGTGGLSALGSGPRLRMLLLESVSGLLQPRTGSTVPPVHPPVRSAPHLPGLMRLLRLHGTVG
GAQNLSAVAALVGLSNARLGSIKTRFEGLCLLSLLVGESSTEMFQQHCVSWLRSIQQVLQGKLASFFLSRVDALSPQLQQ
LACECYSRLPSLGAGFSQGLKHTESWEQELHSLLASLHSLLGALYEGAEPAPMQYEGPGVEMLFSPSEDGDAHNLLRLRQ
RFSGLACCLGLLLRSEFGAPVSIPVQEILDVICRTLSISAKNISLLGDGPLRLLLLPSIHLEALDLLSALILACGGRLLR
FGSLISRLLPQVLNAWSIGRDTLSLGQEKPYSAMRTKVYAVLELWVQVCGASAGVLQGGASGEALLTHLLSDISPPADAL
KLRSPRGSPDGGLQTGKPSAPKKLKLDMGEAVAPPSHRKGDSNANSDVCAAALRGLSRTILMCGPLIKEETHRRLHDLVL
PLVMSVQQGEVLGSSPYTSSYCRRELYRLLLALLLAPSPRYPPPLACALQAFSLGQREDNLEVSSFCSEALVTCAALTHP
RVPPLQSMGPACPAPAPVPPPEAPSPFRAPPFHPPGPMPSAGTMPSAGPVPSVGPMPPVGPMPPARPGPPATANHLGLSV
PGLASVPPRLLPGPENHRAGSNEDTILAPSGTPPPTIPPDETFGGRVPRPAFVHYDKEEASDVEISLESDSDDSVVIVPE
GLPPLPLPPPAGTTPPPVAPAGPPTASPPLPAKEEPEELPAAPGPLPPPPPPLPGPVTLQPPQLVPEGTPGGGAPPALEE
DLTVININSSDEEEEEEEEEEEEEEEEEEEEEDFEEEEEDEEEYFEEEEEEEEEFEEEFEEEEGELEEEEEEEDEEEEEELEE
VEELEFGPTGGEVEGGGPPPTSLPPALPPTESPKVQPEPEPEPEPEPEPGLLLEVEEPGAIGEPGAETAPTLAPEVLPSQEE
MEREGGSPPAGPPPQELVEEEPCAPPTLLEEGTEVGGDEVPPPPETVAAEEMEPEPEPETPALQEKEQDDTAAMLADFID
CPPDDEKPPPVTEPDS

> Odocoileus virginianus texanus [A0A6J0Z390]
MQSGVEGRRAGVRRRIRVSGRDSRTSLSANLSAVGALVGLSNARLGSIKTRFEGLCLLSLLVGESPTEMFQQHCVSWLRS
IQQILQSQDPPPTMELAVTVLKDLLRYAAQLPAVSRDISMNHLPGLLTSLLGLRPECELSALEGMKACMTHFPRACGSLK
GKLASFFLSRVDALSPQLQQLACECYSRLPSLGAGFSQGLKHTDSWEQELRSLLASLHSLLGGLYEGAEAAPMQYESPGV
ETLLSPSEDADAHTLLQLRQRFSGLARCLGLMLSSEFGAPVTVPVQDILDLICRTLSVSAKNVSLLGDGPLRLLLLPSLH
LEALDLLSALVLACGARLLRFGALISRLLPQVLNAWSIGRENLGPGQERPYSTVRTKVYAVLELWVQVCGASAGMLQGGA
SGEALLSHLLSDISPPADALRLRSPRGSPDGGLQTGKPSAPKKLKLDVGEAMAPPSHRKGDSNANSDVCAAALRGLSRTI
LMCGPLIKEETHRRLHELVLPLVMGVQQGEALGSSPYTSSHCRRELYRLLLALLLAPSPRCPPPLACALRAFSLGQREDS
LEVSSFCSEALVTCAALTHPRVPPLQSVGPTCPAPAPVPPPEAPAPFRAPAFHTPGPLPSAGTMPSAGPMPPAGPLPPTR
PGPPATANHLGLSVPGLVSVPPRLLPGPENHRAGSSEDPVLAPSGSPPPTVPPDETFGGRVPRPAFVHYDKEEASDVEIS
LESDSDDSVVIVPEGLPPPPPPPPSGTTPPPVAPAGPPAASPPVPAKDEPEELPAAPGPLPPPPPPPPVPGPVTLPPPQLVP
EGTPGGGGPPALEEDLTVININSSDEEEEEDEEEEEEDEEDEEEDFEEDEEEEEEYFEEEEEEEEEFEEEFEEEEGELEDE
EDEEEDEELEELEEVEFGPAAGDGEGGGPVPPSLPPPALPPAESPKGPPEPGLEPGLLLEVEEPGTEEAPGPETAPTLAP
EVLPPQGETEREAGSPPAGPPPQELVEAEPSGPPALLEEGAEGGGEKVSPPEEAAAAAEAAALPPEKEQGDTAAMLADFI
DCPPDDEKPPPASEPDS
> Pteropus vampyrus [A0A6P3QGW6]
MFQQHCVSWLRSIQQVLQSQDPPPTMELAVAVLRDLLRYAAQLPTLFRDISMNHLPGLLTSLLGLRPECELSALEGMKAC
MTYFPRACGSLKGKLASFFLSRVDALSPQLQQLACECYSQVPSLGAGFSQGLKHTESWEQELHSLLASLHSLLGALYEGA
EPAPMQYEGPGVEMLLSPSEDGDAHILLRLWQRFSGLARCLGLMLSSEFGAPVSVPVQEILDVICRTLSISAKNISLLGD
GPLRLLLLPSIHLEALDLLSALILACRGRLLRFGALISRLLPQVLNAWSIGRDSLSPGQEKPYSAMRTKVYAILELWVQV
CGASAGVLQGGASGEALLTHLLSDISPSADTLKLRSPRGSPDGGLQMGKPSAPKKLKLDVGEAMAPPSHRKGDSNANSDV
CAAALRGLSQTILMCGPLIKEETHRRLHDLVLPLVMGVQQGEVLGSSPYTSSCCRRELYHLLLALLLAPSPRCPPPLACA
LQAFSLGQREDSLEVSSFCSEALVTCAALTHPRVPPLQSMGPTCPTPAPVPPPEAPSPFRAPPFHPPGPMPSAGPMPSAG
PVPSVGSVPSVGSVPSVGSMPSAGPMPSAGPMPPTRPGPPATANHLGLSVPGLVSLPPRLLPGPENHRAGSNEDPILAPS
GTPPPTIPPDETFGGRVPRPAFVHYDKEEASDVEISLESDSDDSVVIVPEGLLPLPPPPPSGTTPPPVTPAGPPTASPPL
PTQEEPEELPVAPGPLPPPPPPPPVPAPVTLAPPQLVPEGTPGGGGPPALEEDLTVININSSDEEEEEEEEEEDEEEEEEED
FEEEEEDEEEYFEEEEEEEEEEFEEEYEEEEGELEEDEEEDEEEEEEELEEVEELEFGSAGGEIEEGGPPPPNLPPALPPVE
SPKLHPEPEPEPEPEPEPEPGLLLEVEEPGAEEEHGAETAPTLAPEVLPSQEELEREAESPPAGPPQELIEEEPCAPPALLE
EGTEVGSDKVPPPPETSAAEEMQTETEATALQEKEQDDTAAMLADFIDCPPDDEKLPPATEPDS
> Acinonyx jubatus [A0A6J1YDH5]
MELAVAVLRDLLRYAAQLPTLFRDISTNHLPGLLTSLLGLRPECELSAMEGMKACMTYFPRACGSLKGKLASFFLSRVDA
LSPQLQQLACECYARLPSLGAGFSQGLKHTESWEQELHSLLASLHGLLGALYEGADAAPVQCEGPGLDVLLAPSEDGDAH
TLLRLRHRFSGLARCLGLLLSSEFGAPVSVPVQEILDIICRTLSISAKNISLLGDGPLRLLLLPSIHLDALDLLSALILA
CGSRLLRFGALISRLLPQVLNAWNLGRDALPPGQERPYSAVRTKVYAVLDLWVQVCGASAGVLQGGASGEALLSHLLSDI
SPPADALKLRSPRGSPDGGLQSGKPSAPKKLKLDMGEATAPPGHRKGDSNANSDVCAAALRGLSRTVLMCGPLIKEETHR
RLHDLVLPLVMGVQQGEVLGSSPYTSSRCRRELYRLLLALLLAPSPRCPPPLACALQAFSLGQREDSLEVSSFCSEALVT
CAALTHPRVPPLQSMGPACPAPAPAPPPEAPSPFRAPPFHPPGPMPSVGPMPSVGPMPSVGPMPPAGPMPPTRPGPPATA
NHLGLSVPGLVSVPPRLLPGPENHRAGSNDDPVLAPSGTPPPAVPPDETFGGRVPRPAFVHYDKEEASDVEISLESDSDD
SVVIVPEGLPPLPPPPPTGTTPPPAAPAGPPTASPPVPAKEEPEELPAAPGPLPPPPPPPPVPGPVALPPPQLVPEGPPGG
GGPPALEEDLTVININSSDEEEEEEEEEEEEEEEEEEEEEEDFEEEEEEEEEEYFEEEEEEEEEEFEEEFEEEEGELEEEEED
EDEEEEEDELEELEEVEFGPAGGPAEEGGPPPPSPAPALPPAQPPEAPPEPGVEPGLLLEVEEPGPEDEPGAEAAPTLAP
EVLPSQGEGQREAGSPPAGPPPQELVEEEPSAPPPLLEEGTENGGDKVPPPPETPAAEEMEAAAEAETAALQEKEQDDTA
AMLADFIDCPPDDEKPPAAPEPES
> Leptonychotes weddellii [A0A2U3YHJ6]
MFQQHCVSWLRSIQQVLQSQDPPPTMELAVAVLRDLLRYAAQLPTLFRDISMNHLPGLLTSLLGLRPECELSALEGMKAC
MTYFPRACGSLKGKLASFFLSRVDALSPQLQQLACECYSRLPSLGAGFSQGLKHTESWQQELRSLLASLHSLLGALYEGA
ETAPVQYEGPGVEVLLTPAEDGDAHALLRLRQRFCGLARCLGLMLSSEFGAPVSVPVQEILDVICRTLSISAKNISLLGD
GPLRLLLLPSIHLDALDLLSALILACGGRLLRFGGLVSRLLPQVLNAWNMGRDTLSPGQERPYSTVRTKVYAVLELWVQV
CGASAGVLQGGASGEALLSHLLSDIAPPADALKLRSPRGSPDGGLQSGKPSAPKKLKLDLGEAMAPPSHRKGDSNANSDV
CAAALRGLSRTVLMCGPLIKEETHRRLHDLVVPLVMGLQRGETLGSSPYTSSRCRRELYRLLLALLLAPSPRCPPPLACA
LHAFSLGQREDSLEVSSFCSEALVVCAALTHPRVPPLQPAGPPCPTPAPVPPPEAPSPFRAPPFHPPAGPLPPARPGPPA
TANHLGLSVPGLVSVPPRLLPGPENHRAGSNEDPVLAPSGTPPPALPPDETFGGRVPRPAFVHYDKEEASDVEISLESDS
DDSVVIVPEGLPPLPPPPPSGTTPPPVAPAGPPTASPPVPAKEEPEELPAAPGPLPPPPPPPPVPGPVALPPPQLVPEGTP
GGGGPPTLEEDLTVININSSDEEEEEEEEEEEEEEEEEEEEEDFEEEEEDEEEYFEEEEEEEEEFEEEFEEEEGELEEEEE
EEDEEDEEELEELEEAEFGPAGGPAEEGGPAPPSPAPALPPAQSPTVQPEPEGEPGLLLEVEEPGAEEEPGAQAAPTLAP
EVLPSQGEGPRATGSPPAGPPPQELVEEEPLAPPTLLEEGTESGGDRVPPLPETPAAEDGEAEAEAESAALQEKEQDDTA
AMLADFIDCPPDDEKPPPAPEPDS
> Trichechus manatus latirostris [A0A2Y9FWQ0]
MELAVAILKDLLRYAAQLPTLFRDISMNHLPGLLTSLLGLRPECELSALEGMKACMIYFPRACGSLKGKLASFFLSRVEA
LSPHLQQLACECYSRLPSLGAGFSQGLKHTESWEQELHCLLASLHSLLGTLYEGVETAPVQYEGPGVEMLLSSSEDGDAH
ALLRLRQRFSGLARCLGLMLSSEFGAPVSVPVQEILDLICRTLSVSGKNISLLGDGPLRLLLLPSIHLEALDLLSALILA
CGSRLLRFGGLISRLLPQVLNAWSMGRDILSPGQERPYSTVRAKVYAVLELWVKVCGASAGVLQGGASAEALLTHLLSDI
SPPADALKLRSPRGSPDGGLQTGKPSAPKKLKLDVGEAAAPPSHRKGDSNANSDVCAAALRGLSRSILMCGPLIKEETHK
RLHDLVLPLVMGIQQGEVLGSSPYTSSRCRRELYHLLLALLLAPSPRCPPPLACALQAFSLGQREDSLEVSSFCSEALVT
CAALTHPRVPPLQSVGTPCPTPAPVPPLESPSPFRAPPFHPPGPMPSVGPMPSAGPMPSAAPVPSARPGPPATANHLGLS
APGLVSVPPRLLSGPENHRAGSNEDPVLAPSGSPPPSIPPDETFGGRVPRPAFVHYDKEEASDVEISLESDSDDSVVIVP
EGLPPLPPPPPSGSTPPPVAPAGPPAASPPVPAKEEPEELPAAPGPLPPPPPLPVPGPVTLPPPQLVPEGTPGGGGPPAL
EEDLTVININSSDEEEEDEEEEEEEEEEEDEDFEEEEEDEEEYFEEEEEEEEEFEEEFEEEEGELEEEEDEEDEEEEEEELE
EVERKGGSPPAGPPPQELVEEEPPAPPTLLEEETEDGGDKVPPPPEASAEEEMDTETEAAALQEKEQDDTAAMLADFIDC
PPDDEKPPAATEPDS
> Lipotes vexillifer [A0A340XCH5]
MELAVAVLGDLLRYAAQLPTLFRDISMNHLPGLLTSLLGLRPESELSALEGMKACMTYFPRACGSLKGKLASFFLSRVDA
LSPQLQQLACECYSRLPSLGAGFSQGLKHTDSWEQELHSLLASLHSLLGALYAGADTAPMQYEGPGLETLLSPSEDGDAH
VLLRLWQRFSGLARCLGLMLSSEFGAPVSVPVQEILDLICRTLSVSAKNISLLGDGPLRLLLLPSLHLEALDLLSALILA
CRGRLLRFGALISRLLPQVLNAWSIGRDSLSPGQERPYSTMRTKVYAVLELWVQVCGASAGVLQGGASGEALLTHLLSDI
SPPADALKLRSPRGSPDGGLQTGKPSAPKKLKLDMGEAMAPPSHRKGDSNANSDVCAAALRGLSRTVLMCGPLIKEETHR
RLHDLVLPLVMGVQQGEVLGSSPYTSSRCRRELYRLLLALLLAPSPRCPPPLACALQAFSLGQREDSLEVSAFCSEALVT

CAALTHPRVPPLQTMGPACPTPAPVPPPEAPSPFRAPAFHPPGPMPSVGPMPSVGPMPSAGPMPSPGPVPPAGPMPSVGP
MPPARPGPPATANHLGLSVPGLVSVSPRLLPGPENHRAGSNEDAVLAPSGTPPPTIPPDETFGGRVPRPAFVHYDKEEAS
DVEISLESDSDDSVVIVPEGLPPLPPPPPSGTTPPPAAPVGPPTASPPVPAKEEPEELPAAPGPLPPPPPPPVPGPVALP
PPQLVPEGTPSGGGPPALEEDLTVININSSEEDEDEDEDEEDEEDFEEEEEEEEEEEEYFEEEEEEEEEFEEEFEEEEGE
LEEEEEDEDEEEEVEEGEVERERGSPPAVPPPQELVEEEPSVPPTLLEEGAEGGGDKVPPPPEASAAEEMETEAEALPEK
EQDDTAAMLADFIDCPPDDEKPPPATEPDS
> Phascolarctos cinereus [A0A6P5IJE5]
MELAVVILRDLLRYSAQLPELSRDISTNHLPGLLTSLLGLKPECELSALEGMKACMTYFPRACGSLRGKLASFFLSRVEA
LSPQLQQLACECYARLPALGAGFSQGLKHTESWEQELHCLLASLHGLLGALYEGAETAPIQYEGPGVELLLPPPADNDVH
GLLQLRQRFSGLARCLGLMLSSDFGAPVSVPVQDILDIICRTLSVSGKNISWLGDGPLRLLLLPSIHLEALDLLSALILS
CGSRLVRFGGLICRLLPQVLNTWSAGRDPLPPGQERPYSAVRAKVYSVLELWVQTCGAGAGVLQGGAQSEALLTHLLSDI
FPPADALKLRGPRVTQDGGLQSGKPSAPKKLKLDSGETVVPPLHRKGDSNANSDVCTTALRGLSRTILMCGPLIKEETHR
RLHELTLPMVMGTQQGEIPGGSPYTSARCRRELYRLLLALLLAPAPQCPPPLTCALQAFSLGQHEENLEVSSFCSEALVT
CAALAHPRVPPLQSPGPSSPPTAPPTQPEPPSPFRAPPFHPPGPRPPVSATANHVGLPVPGLVSVPPRLLPGPENHRAAL
SEDSAPAPAGTSPPSQPPDEAFGGRPPRPAFVHYEKEETSDVEISLESDSDDSVVIVPEGLLPLPPAPPQGGTPPPAPPV
VATPTSPPLPPKEEPEELPPPPGPLPPPPGPGALPPPQLVAEGTPGGAPAPLEEDLTVININSSDEEEEEEEEEEEEEEE
DFPEEEEEEEEEEEYFEEEEEEEEEFEEEFEEEEEGELEEEEEEEEEEEEEEEELEEVEFEPAEGEVEDRGAPPPDSSPPP
PPSEPPKEEPEPGLLMEVEEPSAEDEKEGGGAPPLALEASPPQGEREEEGRTVVPEAPPAEELAGNEPPALSGEGTDGRG
SQEPPPAKAAEAPATETEMAPMGEKEQDDTAAMLADFIDCPPDEEKPTPPPEPDS
> Geotrypetes seraphini [A0A6P8PE30]
MELAVLVLHDLLKYSSLLPALSREISMNLIPGLLTSLLDLKPECQAPALEGMKACMTFYPRACGSLRGKLASFFMNRLET
ESPQIQQLACECYTLLPSLGSGFAQGVKHTECWEHQLQCLLATLHATVGQLYEGAETELLHYEGAGVELLPPVADDGEAL
HVLRLKHRFSGICKCLCLLLSSSFSVPVTVPVQDVLDAICRVLSISSKNISWLGDGPLKTLLLPSVHAEVLDLLAALILA
CGSRLVRFAGLFCRLFAQVLTLWSGKRDDIIPGQEKPYSAVRTKVYQVLEMWIKVCGASSGVLQGPGHHCDVLLAHLTSD
ISPPSDTIKLRVGRLGAEISLNNGKPGSKKQKMDLGDGSQLQSHRKQDVNANSDTCIMALRVLSRAILLCGTLIKEDTHR
RLHELVMPLLIRLQQSALPTTSPYTSSECRKELYRLLLCLLLTPHPRWPPPLHCAVCIFSQGQKENNMKVSSFCAEALVI
CNCIVHPRVPSLQLPVTSSATNTAATDLLKSSVLGADMPSTFRHSPAPTSLRSQTNHLTIPLQGLASGQPLASRLQSSMV
SNLTLPSVPPCTVGVENHQLDEHMEVESRVHSMPSPSEETFGGKTHRPVFIHYEKEEASDVEISLDSDSDDSVVIVPEGL
LPKQTLSPPTKVVEEAEESSKEHAITVTVATTVNATISVPASPASSNQILAPTAALPPPPPPLPPPPPPPLQLVAETPAS
AVEEDLTVINLNSSEEEEEEEEEEEEDFAEDEYFDDEEDFEEEEYDGEMMDEEEEEDEEGEGQLSLDAIDMDEEEDLDDLE
EDLECEDEVMTEEEEEEEEEDEEDIMPCIEEEPEEELRVGFVEKEALETEGEPAAQADSEAREEKSGAGEQIDESPPRLSP
VREGTQEAKEEEEVATEADTDLLMTVESDSAEREEEEEEEEVKETPLLVQETQTPQLPEETSPPPPPPPPPPPPPPSSPPPP
PPPPPPPLPPPLPPPLPTESEECEKIQMEERSDQEVETSPAQTSTEERIGELVTEKDIKPDKEEETPQSTEKKAEAEIDD
TAAMLADFIDCPPDEDKTSPDISS
> Bison bison bison [A0A6P3I471]
MAAAVLSGPSAGSAAAVPGGPGSLSAVGSGPRLRXXXXESVSGLLQPRTGSAVAPVHPPARSAPHLPGLMCLLRLHGTVG
GAQNLSAVGALVGLSNARLGSIKTRFEGLCLLSLLVGESPTEMFQQHCVSWLRSIQQILQSQDPPPTMELAVTVLKDLLR
YAAQLPAVFRDISMNHLPGLLTSLLGLRPECELSALEGMKACMTHFPRACGSLKGKLASFFLSRVDALSPQLQQLACECY
SRLPSLGAGFSQGLKHTDSWEQELRSLLASLHSLLGGLYEGAEAAPMQYESPGAETLLSSSEDADAHTLLRLRQRFSGLA
RCLGLMLSSEFGAPVSVPVQDILDLICRTLSVSAKNVSLLGDGPLRLLLLPSLHLEALDLLSALILACGARLLRFGALIS
RLLPQVLNAWSIGRENLGPGQERPYSTVRTKVYAVLELWVQVCGASAGVLQGGASGEALLSHLLSDISPPADALRLRSPR
GSPDAGLQTGKPSAPKKLKLDVGEAIAPPSHRKGDSNANSDVCAAALRGLSRTILMCGPLIKEETHRRLHELVLPLVMGV
QQGEALGSSPYTSSHCRRELYHLLLALLLAPSPRCPPPLACALRAFSLGQREDSLEVSSFCSEALVTCAALTHPRVPPLQ
SVGPTCPAPAPVPPPEAPAPFRAPAFHAPSPLPSAGPMPSAGPMPPVGPLPPTRPGPPATANHLGLSVPGLVSVPPRLLP
GPENHRAGSSEDPVLAPSGSPPPTIPPDETFGGRVPRPAFVHYDKEEPSDVEISLESDSDDSVVIVPEGLPPPPPPSSGT
TPPPVAPAGPPAASPPVPAKDEPEELPAAPGPLPPPPPPPVPGPVTLPPPQLVPEGTPGGGGPPALEEDMTVININSSDE
EEEEEEEEEEDEEEDEEEDFEEDEEEEEEYFEEEEEEEEEFEEEFEEEEGELEDEEDEEEDEELEELEEVEFGPAGGEV
EGGGPAPPSLPPALPPAESPKGPPEPGLEPGLLLEVEEPGTEEAPGPETAPMLAPEVLPSQGEVEREGGSPPAGPPPQEL
VEEEPSGPPALLEEGAEGGGDKVSPPPEASAVEETEVEAAALPPEKEQGDTAAMLADFIDCPPDDEKPPPASEPDS
> Tursiops truncates [A0A6J3QMB7]
MELAVAVLGDLLRYAAQLPTLFRDISMNHLPGLLTSLLGLRPESELSALEGMKACMTYFPRACGSLKGKLASFFLSRVDA
LSPQLQQLACECYSRLPSLGAGFSQGLKHTDSWEQELHSLLTSLHSLLGALYAGADTAPMQYEGPGVETLLSPSEDGDAH
VLLRLWQRFSGLARCLGLMLSSEFGAPVSVPVQEILDLICRTLSVSAKNISLLGDGPLRLLLLPSLHLEALDLLSALILA
CRGRLLRFGALISRLLPQVLNAWSIGRDSLSPGQERPYSTMRTKVYTVLELWVQVCGASAGVLQGGASGEALLTHLLSDI
SPPADALKLRSPRGSPDGGLQTGKPSAPKKLKLDMGEALAPPSHRKGDSNANSDVCAAALRGLSRTVLMCGPLIKEETHR
RLHDLVLPLVMGVQQGEVLGSSPYTSSRCRRELYRLLLALLLAPSPRCPPPLACALQAFSLGQREDSLEVSAFCSEALVT
CAALTHPRVPPLQTMGPACPTPAPVPPPEAPSPFRAPAFHPPGPMPSVGPMPSVGPMPSAGPMPSPGPVPPAGPMPSVGP
MPPARPGPPATANHLGLSVPGLVSVSPRLLPGPENHRAGSNEDAVLAPSGTPPPTIPPDETFGGRVPRPAFVHYDKEEAS
DVEISLESDSDDSVVIVPEGLPPLPPPPPSGTTPPPAAPVGPPTASPPVPAKEEPEELPAAPGPLPPPPPPPVPGPVALP
PPQLVPEGTPSGGGPPALEEDLTVININSSEEEEDDDEEDEDEEDFEEEEEEEEEEEEYFEEEEEEEEEFEEEFEEEEGEL
EEEEEDEDEEEEVEEVEFGPAGGEVEEEGPAPPSLPPALPPAASPKVQPQPEPEPGLLLEVEEPGAEEGPGAETAPTLAP
EVLPSQGEVEREGGSPPAVPSPQELVEEEPSVPPTLLEEGAEGGGDKVPTPPEASAAEEMETEAEAAALQEKEQDDTAAM
LADFIDCPPDDEKPPPATEPDS
> Cottoperca gobio [A0A6J2RH89]
MATSAWLRGPSAMRLTEGLVSVLKEQRSEYLPALLANYREHGVFQTQGASAVGGLVGFSNAKLGSSKTRFEGLCLLSMLV
KDSSSDLFQQHCISWLRSLQQVIQSQAPVQTIQLAVNILKDLLQYSSQLAELAREVGLNSILGILTSLLGLKTECELAAM
EGMTACMTYYTRACGSLRDKLGAYFLSKMDSTNKKTQEMACLCYGRLPSLGGLLDRGVSAGRAEGWTNQIHCLLASGNGL
LAQIYQGSETEGTVQYEGPGVELAFSHLDQSDPLLLLQLQHRYTSVCLALKYTLRVDPASAVRLPVRPILNLVCRVLAVS
SKSINLTGDGSVRLLILPIIHTNTLEVLSALMIAVRSGMVQYAAVLQRLFSQTLSAWTPLPEASLGHQRAYSSVRVTLYR
TLELWFQVAGASASVLQGSPGHSELLFSHLLSDITPGAESIKLRAGLSADAVPGGKPGPRRTKPLVIADTVGPSLQRKGD
VLANQDTCISALRALRHIILTSGTLLKDDLHKRLHDVVLPLCVRLQQQQSSSSTSCESAGSVSGQYSSALGRLELYKLLL
ALVLVPSPCWPPPLTCAVSILSNGRNDRNLKVSTFCTEALTICNSLLHPRTPSIALPLPPLTLKPTAAASVLPPSQRTPS
GLTLPTLLGAPAPVPFPTRHSLGLGPASLLGPLDNHLSLVPGQAPTPGDMILSPHAHHQQDPSGLGLPEGQRPVFVRYDR
EEAEDVEISLASDSDDSVVIVPPMMLNMDIQQDETAAAANSLNMSSAAQGGATVTLPGGESVTMVPTTATTTTIDRVSLP
NDLATSSPLLTTSTTPINSFPPSSSSVVSLVPPLNSSTLAAPPGGLGDSLPGRPQLQQMLMQPSTAGQPGPMGLPHQLHQ
LQNQLSQPGRHLHQHQPPPASNEDSAVININSTDEEDEEEEDMEDDEELDEEDEGLEEEEEDEEASDFADEFYDGEEYED
YDEEEGEELEEEEEEEDGDIPPLEGAEDKAGEAGMEGGKVLRAAVDEGGMAGFSVEAEAEGGIEEIQTNRSMFGEDRVKV

QKVESIGVLEEAREGEEDESERMDDPTMPQILCVTGGSLEEREETEEEGGGGRGGDGERLQDEARSWEQGANEIELAAAS
EECTTNQNQQESGAETAQEAGVSDNQPSSHQEQLAAVEEGDSAAAEPETSTGPNTKEQEETVAEKRDGVKAEQQTEGTGE
SDGEEGKGVKRKREEANREEEGQGTEKKKMDDEAMASMLADFVACPPDDEDGASGSNRP
> Acanthochromis polyacanthus [A0A3Q1GMM5]
MSSPAHCRTDAISFSVSSKALSRSLAISRQETATTSSSPSRISPVRRHTVDHGCFQQRIHSNNVTVSHSGDVARRRGKMA
TSTWLHGPSAMRLTEGLVSVLKEQRPEYLPALLANYREHGVFSTQGAGAVGGLVGFSNAKLGSSKTRFEGLCLLALLVKD
SSSDLFQQHCLSWLRSLQQVIQSQAPVQTIQLAVNILKDLLHYSSQLPELTREVGLNSVLGILTTLLSLKTECELAAMEG
MTACMTYYPRACGSLRDKLGAYFLSKMDSSNRKTQEVACQCYGRLPCLGGLLDRGVGAGRAEGWTNHIHCLLASANALLA
QIYQGAETDGAVQYQGPGVELSFPHVDQTDPLLLLQLQHRYTAVSLALKHTLRVDPASAVRVPVRPILNLVCRALAVSSK
SINLTGDGSVRLLVLPIIHTDTLEVLSSLITVVRRGMVQYAAVLQRLFSQTLSAWTPLPEASVGQQRAYSSVRVSVYKTL
ELWVQVAGASANILQGSPSHSELLFGHLLGDITPGAESIKLRAGLSADVVPGGKPGPRRTKQLVMADAVGPSLHRKGDLM
ANQETCLSALRGLRQIIQTSGTLLKDDIHKRLHDVILPLCVRLHQQQCSTSTACESAWGISGQYSSAATRKELYRLLLAL
VLVPSPCWPPPLTCAVSILSNGRTDRNLKVSSFCAEALTICNSILHPRTPSVALPLPPLTLKPTHTAPVLPSSQGPAPGL
TLPTLLGGPTPGPPFPARHSLGLGPSSLLGSLENHLSLVPGLPGQAPAAGDMILSPHTHHQADSAGLGPPEGQRPVFVRY
DKEETEDVEISLESDSDDSVVIVPPGMLNMENQQDDTAANSQNILSAVPGSASVALPGGESVTMVSTTAATTTVEGTSLP
NDLATSSPLLTTSTTPINSFPPSSSSVVSLVPPLTSSTLTGPPGGLGDSMPGRPQLQQMLMQPSTPGQPSPMGLPLQMHQ
LQNQLSQQGRHLHQHPPAPTSNEDSAVININSTDDEEEDEEDMEDDEELEEEEEGLDEEDEEEDVSDLAEDEFYDGEEYE
DYDEEEGEDLEEEEEEEEEDGDIPPLEGAEDKGGEAGIAEGKVLRAAVDEGGMSGFNVEGDAEGGIEEIQTSRTLFGDDRM
KVQEVESIGVLEEAREAGEEDDSEKMNDPTMPQILCVTGGALDEREETEEEGGGTGAGLQEGPSSWEQGAKENEQAAASE
EQPASQSPQESAVEPAQEASVSDNQPSSSPQEEQVVAVEEGDPPADPETSAVLSPKEQEETVDLIKDKTDTEEQEAGGRE
EGSDGEEGKGVKRKREEEQQEAEAEQSTEKKKLDDEAMASMLADFVACPPDDDEDGATGSNCS
>Salmo trutta [A0A673YAA5]
MAATAAWMHGPANMRLTEGLVSVLKEQRPEYLPALLANYREHGVVSTQSSAAVGGLVGVSNAKLGNSKTRFEGLCLLSVL
VKDSSSDVFQQHCLSWLRSLQQVIQSQAPLPSIQLAVGVLQDLLQYSSQLPELAREVGLNSILGILTSLLGLKSEFHLAA
MEGMTACMTFYPRACGSLRDKLGACFLSKMDSVNPEVQDVACECYGRLPCLGGVLERVGVGRTAEGWTNQLHCLLASANG
MLAQLYQGTESEGMVPYEGPGVELPYPPLDDTDPLVLLQLQHRYRGVCLALKHTLSVDPATAVRLPVQQVLNLVCRALAV
SSKSINLTGDGSVRLLVLPSIHNHTVKVLQALITAVGSGLVQYSSMLQRLFSQTLSAWTPLPETSLGQQRAFSAVRVSLY
QTLELWVKVGGASAGVLQGSPTHSEILLAHLLGDITPGADSVRLRAGQSTVADLVSSKPCPKSRKPGLGMGNGGGASLQR
KGDSLANQDTCVSALRALRQIILTSGTLLKEDVHKRLHDVVLPLCVRLQQQHGGDCGTGAVSGQYGSALPRRELYRLLLA
LVLVPPPRWPPPLTCTVSILSHGRRDRSLKVSSFCTEALAICNSLLHPRTPSLCLPLPPLTLKPSPAASLLTPSQASSLT
LPTLLGGPFPGRHSLGLGHTLLGSLDNHLSLVPPGLSGQGSTPGDLLLSPHQGELAGLGLSEGQRPVFIRYDKEEAEDVE
ISLESDSDDSVVIFPRGMLMLENQDGTSTVANLPVSSLVPGGVTLPVPGPGDDPGGDISLPLANDLPSTSLPHPLLPSSS
APNSINSFPPAPLASLVPPLNSTGVTQLGAPSVGLGVGADSLPGAQLQQMLLQGQPPAPGQPTPLGLPIQMLQNQLAQPS
RALQQQQASEEDHSVININSSDEEEEEDEEMEDEDELGEEEDEEGLEDEEEEEEGSDFPDEEEEFYGEEFDDYEEEEGEE
MDEEEEEEEEEEEEEGEIRPLDREGRRGGMGREEGEVLREVPEDGGMGGFCVEGEMEGGIEELGTNRRVYGEEGVKAQEVE
SIGVLEEEREGEEDDADGMNDPTMPQILCVTGGALEEREELGEGHGQEVGSCEQQGADRPEATPSSEGPAPQHKQEVEPA
QEVRVGGSDQPSNQGEEPAKQGGDSAKEEVKPSAAPSETEGEEPEPQEKGGEEGEEDGEEDGRGIKRKREGEEEGTGQGTE
KKKVRTLSQTILNQSHSKPFSLFYNLHIVS
> Sinocyclocheilus anshuiensis [A0A671MU21]
MASAAWLHGPNITRLTEGLVSVLKEDRPEYLPALLANYREHGVVGAQSTGAVGGLVGISNARLGSSKTRFEGLCLLAVLV
KDSSSEVFQQHCLSWLRTLQQVIQSQAPLPTVQLTVSVLQDLLQYSSQLPELAREVGLNSILGILTSLLSLKSECHLVAM
KGMMACMIYYPRACGSLREKLGAYFLSKMDSDNPKVQEVACECYGRVPCLGGVLERGGGGRRAEGWTNQLHCLLASATSM
LGQLYQGAETEGTVQYEGPGVELPFPPLDDVDPLLILQLHHRYKAICLAFKHTLSADPASSVRLPVQHVLNFMCRALAVN
TKSISPTGEGYLRLMVLPSIHNDTLELLAALIKAVGGGLVQYSSVLTRLFSQSLSAWTPLPEASLGQQRAYSAVRVTVYR
TIELWVRVGGASLLQASPSHTELLFTHLMGDITPASEAVKLRSGQSQSMNDLIGSAGKSGPRRTKGLGMGDGISLQRKG
DVLANQDTCVAALRALRQIILTSGTLLKEDLHKRIQDLVVPLCVRLQQQSHCVLEVGAVSGQYGSPSPRRELYRLLLALV
LVPSPRWPPPLSCAVSAFSHGRRHHNIIVSSFCAEALTICNTLIHPRTPSISLPLTPLTLKLTPTAPVLASGQNPSLSIP
TLLGGPATGPPFPARHPMGLGPATLLGSLENHLPLAPPVLPTPAGNTATPGDLLLSPAQPGELAGLGAPEGQRQVFVRYD
KEEPEDVEISLESDSDDSVVIMPAGMMMEMQDGAANAQSLSQSAVPAVGGLQSSAPIVGEVGSVDTSLSNELPTSIPHQI
LPANANNINSFPGSSQTAQLVSLVPPLNSTPASLSASPAGLADSLTGGPQLQQMLMQTSPGGQPPTLGLSLQMQLQNQIA
QTSRQLPQPQPPANEVDQNVININSSDDEEEEEEELEDEDELGEEEEEEEGLEDEEEEEEGSDFIDEEYSVDEFEDYEDEE
GEDEDEEESEEIQPLEGDNGRGMMGEEEAEVMIEAEEQQGMEMFCMEKEREVEPGIEEMEGVRSVYADERIKDKGTMEEI
ENIGAVERNEPVVGKNQIESLVISEDAEGHEEDTRVEAVEPEVKTCEQEVARPDDPAEDAGTSQQSQELTVEDEVQKQEP
ELQPEDTTNQSTPSTSEQEVLQSVAETAEEEVEKESGEQGEDSEARGTKRKMEDREEGESSEHGTEKKKMDDEAMASMLA
DFVDCPPDDDDRGASQSQS
> Bicolor damselfish [A0A3B5BNH5]
MATSTWLHGPSAMRLTEGLVSVLKEQRPEYLPALLANYREHGVFTTQGAGAVGGLVGFSNAKLGSSKTRFEGLCLLAMLV
KDSSSDLFQQHCLSWLRSLQQVIQSQAPVQTIQLAVNILKDLLQYSSQLPELAREVGLNSVLGILTSLLGLKTECELAAM
EGMTACMSYYPRACGSLRDKLGAYFLSKMDSTNRKTQEVACQCYGRLPCLGGLLDRGVGAGRAEGWTNQIHCLLATANGL
LAQIYQGAETDGTVQYQGPGVELTFPHLDQTDPLLLLQLQHRYTAVCLALKHTLRVDPASAVRLPVRPILNLVCRALAVS
SKSINLTGDGSVRLLVLPIIHTNTLEVLSSLITAVRRGMVQYAAVLQRLFSQTLSAWMPLPEASVGQQRAYSSVRVSVYA
TLELWVQVAGASANILQGSPSHSELLFSHLLGDITPGAESIKVGSTCSCLSADVVPGGKPVGPSLQRKGDLLANQDTCLS
ALRGLRQIIQTSGTLLKDDIHKRLHDVVLPLCVRLQQQQCSTSTACESAGGVSGQYSSAITRRELYRLLLALVLVPSPCW
PPPLTCAVSILSNGRTDRNLKVSSFCAEALTICNSILHPRTSSVALPLPPLTLKPTHTAPVLPSSQGPASGLTLPTLLGG
PAPGPPFPTRHSLGLGPTSLLGSLENHLSLVPGLPGQAPAPGDMILSPHAHHQPDPAGLGPPEGQRPVFVRYDKEEAEDV
EISLESDSDDSVVIVPPGMLNMENQQDDVAANSQNILSAAPGGAGVTLSGGESVTMVPTTAATTTVEGTSLSNDLATSSP
LLTTSTTPINSFPPSSASVVSLVPPLNSSTLTGPPGGLGDSMPGRPQLQQMLMQPSTAGQPSPMGLPLQMHQLQNQLSQQ
GRHLHQHPPAPTSNEDSAVININSTDDEEEDEEDMEDDEELEEEEEGMDEEDEEEEVSDLAEDEFYDGEEYEDYDEEEGE
DLEEEEEEEEDGDIPPLEGAEDKVVEAGIEEGKVLRAAVDEGGMSGFSVEGDAEGGIEEIQTNRALFGDDRMKVQEVESI
GVLEDAREGEGEEDDSEKMNDPTMPQILCVTGGALDEREETEEEGGEESAAEPAQEVSVSDNQPSSHQEEQLVAVQEEDT
PADPETSADLSTKEQEETVDLKRDKTDTEQQEAAGTEEESDGEEGKGVKRKREEGQEEEEAGQSTDKKKMDDEAMASMLA
DFVACPPDDEDGASGSNCS
> Sinocyclocheilus anshuiensis [A0A671ML97]
MASAAWLHGPNITRLTEGLVSVLKEDRPEYLPALLANYREHGVVGAQSTGAVGGLVGISNARLGSSKTRFEGLCLLAVLV
KDSSSEVFQQHCLSWLRTLQQVIQSQAPLPTVQLTVSVLQDLLQYSSQLPELAREVGLNSILGILTSLLSLKSECHLVAM
KGMMACMIYYPRACGSLREKLGAYFLSKMDSDNPKVQEVACECYGRVPCLGGVLERGGGGRRAEGWTNQLHCLLASATSM
LGQLYQGAETGMWCVLSVCVVLSVKFTCVKFLLFFCDVTVAEGTVQYEGPGVELPFPPLDDVDPLLILQLHHRYKAICLA

FKHTLSADPASSVRLPVQHVLNFMCRALAVNTKSISPTGEGYLRLMVLPSIHNDTLELLAALIKAVGGGLVQYSSVLTRL
FSQSLSAWTPLPEASLGQQRAYSAVRVTVYRTIELWVRVGGASLLQASPSHTELLFTHLMGDITPASEAVKLRSGQQSQS
MNDLIGSAGKSGPRRTKGLGMGDGISLQRKGDVLANQDTCVAALRALRQIILTSGTLLKEDLHKRIQDLVVPLCVRLQQQ
SHCVLEVGAVSGQYGSPSPRRELYRLLLALVLVPSPRWPPPLSCAVSAFSHGRRHHNIIVSSFCAEALTICNTLIHPRTP
SISLPLTPLTLKLTPTAPVLASGQNPSLSIPTLLGGPATGPPFPARHPMGLGPATLLGSLENHLPLAPPVLPTPAGNTAT
PGDLLLSPAQPGELAGLGAPEGQRQVFVRYDKEEPEDVEISLESDSDDSVVIMPAGMMMEMQDGAANAQSLSQSAVPAVG
GLQSSAPIVGEVGSVDTSLSNELPTSIPHQILPANANNINSFPGSSQTAQLVSLVPPLNSTPASLSASPAGLADSLTGGP
QLQQMLMQTSPGGQPPTLGLSLQMQLQNQIAQTSRQLQPQPPANEVDQNVININSSDDEEEEEEELEDEDELGEEEEEEE
GLEDEEEEEEGSDFIDEEYSVDEFEDYEDEEGEDEDEEESEEIQPLEGDNGRGMMGEEEAEVMIEAEEQQGMEMFCMEKE
REVEPGIEEMEGVRSVYADERIKDKGTMEEIENIGAVERNEPVVGKNQIESLVISEDAEGHEEDTRVEAVEPEVKTCEQE
VARPDDPAEDAGTSQQSQELTVEDEVQKQEPELQPEDTTNQSTPSTSEQEVLQSVAETAEEEVEKESGEQGEDSEARGTK
RKMEDREEGESSEHGTEKKKMDDEAMASMLADFVDCPPDDDDRGASQSQS
> Kryptolebias marmoratus [A0A3Q3F6L3]
MAASSWLHGTGAARLTEGLVSLLKEQRPEFLAEGLANYREHGVFSTQSTSDVAGLVGFSNAKLSSTKTRFEGLCLLSMLV
KDSSSDLFQQHCLSWLRSLQQVIQSQAPVQTIQLAVTILKDLLQYSCQLAELAREVSLNFILGILTSLLALKTECELAAM
EGMKACMIYYPRACGSLKDKLGVYFLSKMDSTNRKTQEVACQGYSHLPCLGGLLDKAVGAGRAEGWTTQIHCLLASANSL
LAQIYQGSETDEAVQYQGPGMELAFPYLDQTDPLLLLQLQHRYTAVCMTIKHTLRADPASAVHLPVRLILNMVCRALAVN
SKSINLAGDGSLRLLILPSIHINSLEVLSELITEVRSGMIQYAGVIQRLFSQTLTAWTPLPDSTVGQQRAYSSVRVSVYT
TLELWVKVVGASASILQGASSHAGLLFTHLLADITPEAESVKLRTGLSVDVVPGGKPGPRRTKQLVLAETVGPSLQRKGD
LLSNQDTCLSALRALRQIVQTSGTLLKDDVHKRLHEVVLPLCVRLQQQQSSSMSACEPAGGISGQYSSALTRRELYRLLL
ALVLVPSPCWPPPLTCAVSILSNGRTERNLKVSTFCAEALTICNSIVHSRSPSIALPLPPLALKPTTPSVLSCQGPGPRL
TLPTLLGGPTSGPPFPTRHSLNLGATSLLGSLENHLSLIPGMPGQAPAPGELMLSPQTHHQSDPSVPPEGQRSVFVRYDK
EEAEDVEISLASDSDDSVVIVPPGMLNTENQQNDSATNSQNVLSSAAGSTGVSLPGGDSITMAPTTAAPTTVDVASLPND
LATPSTAPINSFPPSSASVVSLVPSLNSSSFSASPGGGLGDSGAGKPQLQQMLMQPSAAGQPSSVTLPLQMHQLQNQLTQ
QGRHLHQHPPTPANNEESAVININSTDDEEEDEEDMEDDEELEEEEDGIDEEDEEEEGSDEFYDGEEYEEFEEEEGEELE
EEEEDEDGDIPPLEGSEDKSEEVGMEDDKVLQAAMEEGEVEAYNVEGEAEGGIEELQTSRTLIRGDRMKIQEVESIGVLE
EAREAEREEDETERMDDPTMPQILCVTGGALEERLETAEEGGGGRGGLQEEASLWEQGAKGSEVTLASEEPTASQSQQES
VAENEDGGVRDEQPPAHQEEESAAAEGKASAGAAEETSAGEDVKERQQADADPGDVKEAEQQATEEEETVGEEGKDHGEE
SDGKEEKGIKRKREEENTVEEDEQSAEKKKMEDEAMASMLADFNPCPPED
> Salmo trutta [A0A673YA73]
MAATAAWMHGPANMRLTEGLVSVLKEQRPEYLPALLANYREHGVVSTQVKLAIGGLVGVSNAKLGNSKTRFEGLCLLSVL
VKDSSSDVFQQHCLSWLRSLQQVIQSQAPLPSIQLAVGVLQDLLQYSSQLPELAREVGLNSILGILTSLLGLKSEFHLAA
MEGMTACMTFYPRACGSLRDKLGACFLSKMDSVNPEVQDVACECYGRLPCLGGVLERVGVGRTAEGWTNQLHCLLASANG
MLAQLYQGTESGMVPYEGPGVELPYPPLDDTDPLVLLQLQHRYRGVCLALKHTLSVDPATAVRLPVQQVLNLVCRALAVS
SKSINLTGDGSVRLLVLPSIHNHTVKVLQALITAVGSGLVQYSSMLQRLFSQTLSAWTPLPETSLGQQRAFSAVRVSLYQ
TLELWVKVGGASAGVLQGSPTHSEILLAHLLGDITPGADSVRVIRSDWFIGKAKFCVWYSSINLPHRVCLLCVCVCVCVL
MRSLFPQRLHDVVLPLCVRLQQQHGGDCGTGAVSGQYGSALPRRELYRLLLALVLVPPPRWPPPLTCTVSILSHGRRDRS
LKVSSFCTEALAICNSLLHPRTPSLCLPLPPLTLKPSPAASLLTPSQASSLTLPTLLGGPFPGRHSLGLGHTLLGSLDNH
LSLVPPGLSGQGSTPGDLLLSPHQGELAGLGLSEGQRPVFIRYDKEEAEDVEISLESDSDDSVVIFPRGMLMLENQDGTS
TVANLPVSSLVPGGVTLPVPGPGDDPGGDISLPLANDLPSTSLPHPLLPSSSAPNSINSFPPAPLASLVPPLNSTGQQAS
EEDHSVININSSDEEEEEDEEMEDEDELGEEEDEEGLEDEEEEEEGSDFPDEEEEFYGEEFDDYEEEEGEEMDEEEEEEE
EEEEEGEIRPLDREGRRGGMGREEGEVLREVPEDGGMGGFCVEGEMEGGIEELGTNRRVYGEEGVKAQEVESIGVLEEER
EGEEDDADGMNDPTMPQILCVTGGALEEREELGEGHGQEVGSCEQQGADRPEATPSSEGPAPQHKQEVEPAQEVRVGGSD
QPSNQGEEPAKQGGDSAKEEVKPSAAPSETEGEEPEQEKGGEEGEEDGEEDGRGIKRKREGEEEGTGQGTEKKKVRTLSQ
TILNQSHSKPFSLFYNLHIVS
>Salmo trutta [A0A673YA34]
MAATAAWMHGPANMRLTEGLVSVLKEQRPEYLPALLANYREHGVVSTQSSAAVGGLVGVSNAKLGNSKTRFEGLCLLSVL
VKDSSSDVFQQHCLSWLRSLQQVIQSQAPLPSIQLAVGVLQDLLQYSSQLPELAREVGLNSILGILTSLLGLKSEDKLGA
CFLSKMDSVNPEVQDVACECYGRLPCLGGVLERVGVGRTAEGWTNQLHCLLASANGMLAQLYQGTESEGMVPYEGPGVEL
PYPPLDDTDPLVLLQLQHRYRGVCLALKHTLSVDPATAVRLPVQQVLNLVCRALAVSSKSINLTGDGSVRLLVLPSIHNH
TVKVLQALITAVGSGLVQYSSMLQRLFSQTLSAWTPLPETSLGQQRAFSAVRVSLYQTLELWVKVGGASAGVLQGSPTHS
EILLAHLLGDITPGADSVRLRAGQSTVADLVSSKPCPKSRKPGLGMGNGGGASLQRKGDSLANQDTCVSALRALRQIILT
SGTLLKEDVHKRLHDVVLPLCVRLQQQHGGDCGTGAVSGQYGSALPRRELYRLLLALVLVPPPRWPPPLTCTVSILSHGR
RDRSLKVSSFCTEALAICNSLLHPRTPSLCLPLPPLTLKPSPAASLLTPSQASSLTLPTLLGGPFPGRHSLGLGHTLLGS
LDNHLSLVPPGLSGQGSTPGDLLLSPHQGELAGLGLSEGQRPVFIRYDKEEAEDVEISLESDSDDSVVIFPRGMLMLENQ
DGTSTVANLPVSSLVPGGVTLPVPGPGDDPGGDISLPLANDLPSTSLPHPLLPSSSAPNSINSFPPAPLASLVPPLNSTG
VTQLGAPSVGLGVGADSLPGAQLQQMLLQGQPPAPGQPTPLGLPIQMLQNQLAQPSRALQQQQASEEDHSVININSSDEE
EEEDEEMEDEDELGEEEDEEGLEDEEEEEEGSDFPDEEEEFYGEEFDDYEEEEGEEMDEEEEEEEEEEEEEGEIRPLDREG
RRGGMGREEGEVLREVPEDGGMGGFCVEGEMEGGIEELGTNRRVYGEEGVKAQEVESIGVLEEEREGEEDDADGMNDPTM
PQILCVTGGALEEREELGEGHGQEVGSCEQQGADRPEATPSSEGPAPQHKQEVEPAQEVRVGGSDQPSNQGEEPAKQGGD
SAKEEVKPSAAPSETEGEEPEQEKGGEEGEEDGEEDGRGIKRKREGEEEGTGQGTEKKKVRTLSQTILNQSHSKPFSLFY
NLHIVS
> Bison bison bison [A0A6P3I1T9]
MELAVTVLKDLLRYAAQLPAVFRDISMNHLPGLLTSLLGLRPECELSALEGMKACMTHFP
RACGSLKGKLASFFLSRVDALSPQLQQLACECYSRLPSLGAGFSQGLKHTDSWEQELRSL
LASLHSLLGGLYEGAEAAPMQYESPGAETLLSSSEDADAHTLLRLRQRFSGLARCLGLML
SSEFGAPVSVPVQDILDLICRTLSVSAKNVSLLGDGPLRLLLLPSLHLEALDLLSALILA
CGARLLRFGALISRLLPQVLNAWSIGRENLGPGQERPYSTVRTKVYAVLELWVQVCGASA
GVLQGGASGEALLSHLLSDISPPADALRLRSPRGSPDAGLQTGKPSAPKKLKLDVGEAIA
PPSHRKGDSNANSDVCAAALRGLSRTILMCGPLIKEETHRRLHELVLPLVMGVQQGEALG
SSPYTSSHCRRELYHLLLALLAPSPRCPPPLACALRAFSLGQREDSLEVSSFCSEALVT
CAALTHPRVPPLQSVGPTCPAPAPVPPPEAPAPFRAPAFHAPSPLPSAGPMPSAGPMPPV
GPLPPTRPGPPATANHLGLSVPGLVSVPPRLLPGPENHRAGSSEDPVLAPSGSPPPTIPP
DETFGGRVPRPAFVHYDKEEPSDVEISLESDSDDSVVIVPEGLPPPPPPSSGTTPPPVAP
AGPPAASPPVPAKDEPEELPAAPGPLPPPPPPPVPGPVTLPPPQLVPEGTPGGGGPPALE
EDMTVININSSDEEEEEEEEEEEEEDEEEDEEEDFEEDEEEEEYFEEEEEEEEEFEEEFE
EEEGELEDEEDEEEDEELEELEEVEFGPAGGEVEGGGPAPPSLPPALPPAESPKGPPEPG

LEPGLLLEVEEPGTEEAPGPETAPMLAPEVLPSQGEVEREGGSPPAGPPPQELVEEEPSG
PPALLEEGAEGGGDKVSPPPEASAVEETEVEAAALPPEKEQGDTAAMLADFIDCPPDDEK
PPPASEPDS
> Bison bison bison [A0A6P3I1T6]
MDPLSAYPTPPPIGVRGKRLGEETEAQERSSILPEVTESVNGGIRVQTENLSAVGALVGLSNARLGSIKTRFEGLCLLSL
LVGESPTEMFQQHCVSWLRSIQQILQSQDPPPTMELAVTVLKDLLRYAAQLPAVFRDISMNHLPGLLTSLLGLRPECELS
ALEGMKACMTHFPRACGSLKGKLASFFLSRVDALSPQLQQLACECYSRLPSLGAGFSQGLKHTDSWEQELRSLLASLHSL
LGGLYEGAEAAPMQYESPGAETLLSSSEDADAHTLLRLRQRFSGLARCLGLMLSSEFGAPVSVPVQDILDLICRTLSVSA
KNVSLLGDGPLRLLLLPSLHLEALDLLSALILACGARLLRFGALISRLLPQVLNAWSIGRENLGPGQERPYSTVRTKVYA
VLELWVQVCGASAGVLQGGASGEALLSHLLSDISPPADALRLRSPRGSPDAGLQTGKPSAPKKLKLDVGEAIAPPSHRKG
DSNANSDVCAAALRGLSRTILMCGPLIKEETHRRLHELVLPLVMGVQQGEALGSSPYTSSHCRRELYHLLLALLLAPSPR
CPPPLACALRAFSLGQREDSLEVSSFCSEALVTCAALTHPRVPPLQSVGPTCPAPAPVPPPEAPAPFRAPAFHAPSPLPS
AGPMPSAGPMPPVGPLPPTRPGPPATANHLGLSVPGLVSVPPRLLPGPENHRAGSSEDPVLAPSGSPPPTIPPDETFGGR
VPRPAFVHYDKEEPSDVEISLESDSDDSVVIVPEGLPPPPPPSSGTTPPPVAPAGPPAASPPVPAKDEPEELPAAPGPLP
PPPPPPVPGPVTLPPPQLVPEGTPGGGGPPALEEDMTVININSSDEEEEEEEEEEEEDEEEDEEEDFEEDEEEEEEYFEE
EEEEEEEFEEEFEEEEGELEDEEDEEEDEELEELEEVEFGPAGGEVEGGGPAPPSLPPALPPAESPKGPPEPGLEPGLLL
EVEEPGTEEAPGPETAPMLAPEVLPSQGEVEREGGSPPAGPPPQELVEEEPSGPPALLEEGAEGGGDKVSPPPEASAVEE
TEVEAAALPPEKEQGDTAAMLADFIDCPPDDEKPPPASEPDS
> Bos indicus [A0A6P5DI22]
MLSVAFPFSFHLVNYNSQGLLRMLAARPHRVTTCGKTEYRKFPQSKAIQTCPPSLHCCGLFNFFYKNFLSVCGVLGDDGR
TSGVLQTGQPWTLCLPIPLRHLSXPGENDLNLSAVGALVGLSNARLGSIKTRFEGLCLLSLLVGESPTEMFQQHCVSWLR
SIQQILQSQDPPPTMELAVTVLKDLLRYAAQLPAVFRDISMNHLPGLLTSLLGLRPECELSALEGMKACMTHFPRACGSL
KGKLASFFLSRVDALSPQLQQLACECYSRLPSLGAGFSQGLKHTDSWEQELRSLLASLHSLLGGLYEGAEAAPMQYESPG
AETLLSSSEDADAHTLLRLRQRFSGLARCLGLMLSSEFGAPVSVPVQDILDLICRTLSVSAKNVSLLGDGPLRLLLLPSL
HLEALDLLSALILACGARLLRFGALISRLLPQVLNAWSIGRENLGPGQERPYSTVRTKVYAVLELWVQVCGASAGVLQGG
ASGEALLSHLLSDISPPADALRLRSPRGSPDAGLQTGKPSAPKKLKLDVGEAIAPPSHRKGDSNANSDVCAAALRGLSRT
ILMCGPLIKEETHRRLHELVLPLVMGVQQGEALGSSPYTSSHCRRELYHLLLALLLAPSPRCPPPLACALRAFSLGQRED
SLEVSSFCSEALVTCAALTHPRVPPLQSVGPTCPAPAPVPPPEAPAPFRAPAFHAPSPLPSAGPMPSAXPMPPVGPLPPT
RPGPPATANHLGLSVPGLVSVPPRLLPGPENHRAGSSEDPVLAPSGSPPPTIPPDETFGGRVPRPAFVHYDKEEPSDVEI
SLESDSDDSVVIVPEGLPPPPPPSSGTTPPPVAPAGPPAASPPVPAKDEPEELPAAPGPLPPPPPPPVPGPVTLPPPQLV
PEGTPGGGGPPALEEDMTVININSSDEEEEEEEEEEEEDEEEDEEEDFEEDEEEEEEYFEEEEEEEEEFEEEFEEEEGEL
EDEEDEEEDXELEELEEVEFGPAGGEVEGGGPAPPSLPPALPPAESPKGPPEPGLEPGLLLEVEEPGTEEAPGPETAPML
APEVLPSQGEVEREGGSPPAGPPPQELVEEEPSGPPALLEEGAEGGGDKVSPPPEASAVEETEVEAAALPPEKEQGDTAA
MLADFIDCPPDDEKPPPASEPDS
> Tursiops truncates [A0A6J3QMV0]
MDPVFAYPTPPPIGAWGKPDLNLSAVGALVGLSNARLGSIKTRFEGLCLLSLLVGESPTEMFQQHCVSWLRSIQQVLQSQ
DPPPTMELAVAVLGDLLRYAAQLPTLFRDISMNHLPGLLTSLLGLRPESELSALEGMKACMTYFPRACGSLKGKLASFFL
SRVDALSPQLQQLACECYSRLPSLGAGFSQGLKHTDSWEQELHSLLTSLHSLLGALYAGADTAPMQYEGPGVETLLSPSE
DGDAHVLLRLWQRFSGLARCLGLMLSSEFGAPVSVPVQEILDLICRTLSVSAKNISLLGDGPLRLLLLPSLHLEALDLLS
ALILACRGRLLRFGALISRLLPQVLNAWSIGRDSLSPGQERPYSTMRTKVYTVLELWVQVCGASAGVLQGGASGEALLTH
LLSDISPPADALKLRSPRGSPDGGLQTGKPSAPKKLKLDMGEALAPPSHRKGDSNANSDVCAAALRGLSRTVLMCGPLIK
EETHRRLHDLVLPLVMGVQQGEVLGSSPYTSSRCRRELYRLLLALLLAPSPRCPPPLACALQAFSLGQREDSLEVSAFCS
EALVTCAALTHPRVPPLQTMGPACPTPAPVPPPEAPSPFRAPAFHPPGPMPSVGPMPSVGPMPSAGPMPSPGPVPPAGPM
PSVGPMPPARPGPPATANHLGLSVPGLVSVSPRLLPGPENHRAGSNEDAVLAPSGTPPPTIPPDETFGGRVPRPAFVHYD
KEEASDVEISLESDSDDSVVIVPEGLPPLPPPPPSGTTPPPAAPVGPPTASPPVPAKEEPEELPAAPGPLPPPPPPPVPG
PVALPPPQLVPEGTPSGGGPPALEEDLTVININSSEEEEDDDEEDEDEEDFEEEEEEEEEEEYFEEEEEEEEEEFEEEFEE
EEGELEEEEEDEDEEEEVEEVEFGPAGGEVEEEGPAPPSLPPALPPAASPKVQPQPEPEPGLLLEVEEPGAEEGPGAETA
PTLAPEVLPSQGEVEREGGSPPAVPSPQELVEEEPSVPPTLLEEGAEGGGDKVPTPPEASAAEEMETEAEAAALQEKEQD
DTAAMLADFIDCPPDDEKPPPATEPDS
> Delphinapterus leucas [A0A2Y9Q250]
MFQQHCVSWLRSIQQVLQSQDPPPTMELAVAVLGDLLRYAAQLPTLFRDISMNHLPGLLTSLLGLRPESELSALEGMKAC
MTYFPRACGSLKGKLASFFLSRVDALSPQLQQLACECYSRLPSLGAGFSQGLKHTDSWEQELHSLLASLHSLLGALYAGA
DTAPMQYEGPGVETLLSPSEDGDAHVLLRLWQRFSGLARCLGLMLSSEFGAPVSVPVQEILDLICRTLSVSAKNISLLGD
GPLRLLLLPSLHLEALDLLSALILACRGRLLRFGALISRLLPQVLNAWSIGRDSLSPGQERPYSTMRTKVYAVLELWVQV
CGASAGVLQGGASGEALLTHLLSDISPPADALKLRSPRGSPDGGLQTGKPSAPKKLKLDMGEALAPPSHRKGDSNANSDV
CAAALRGLSRTVLMCGPLIKEETHRRLHDLVLPLVMGVQQGEVLGSSPYTSSRCRRELYRLLLALLLAPSPRCPPPLACA
LQAFSLGQREDSLEVSAFCSEALVTCAALTHPRVPPLQTMGPACPTPAPVPPPEAPSPFRAPAFHPPGPMPSVGPMPSVG
PMPSAGPMPSPGPVPPAGPMPSVGPMPPARPGPPATANHLGLSVPGLVSVSPRLLPGPENHRAGSNEDAVLAPSGTPPPT
LPPDETFGGRVPRPAFVHYDKEEASDVEISLESDSDDSVVIVPEGLPPLPPPPPSGTTPPPAALVGPPTASPPVPAKEEP
EELPAAPGPLPPPPPPPVPGPVALPPPQLVPEGTPSGGGPPALEEDLTVININSSEEEEEDDEEDEDEEDFEEEEEEEEE
EEYFEEEEEEEEEFEEEFEEEEGELEEEEEDEDEEEEVEEVEFGPAGGEVEEGGPAPPSLPPALPPAASPKVQPQPEPEP
GLLLEVEEPGAEEGPGAETAPTLAPEVLPSQGEVEREGGSPPAVPPPQELVEEEPSVPPTLLEEGAEGGGDKVPPPPEAS
AAEEMETEAEAAALQEKEQDDTAAMLADFIDCPPDDEKPPPATEPDS
> Odocoileus virginianus texanus [A0A6J0Z4Q7]
MFQQHCVSWLRSIQQILQSQDPPPTMELAVTVLKDLLRYAAQLPAVSRDISMNHLPGLLTSLLGLRPECELSALEGMKAC
MTHFPRACGSLKGKLASFFLSRVDALSPQLQQLACECYSRLPSLGAGFSQGLKHTDSWEQELRSLLASLHSLLGGLYEGA
EAAPMQYESPGVETLLSPSEDADAHTLLQLRQRFSGLARCLGLMLSSEFGAPVTVPVQDILDLICRTLSVSAKNVSLLGD
GPLRLLLLPSLHLEALDLLSALVLACGARLLRFGALISRLLPQVLNAWSIGRENLGPGQERPYSTVRTKVYAVLELWVQV
CGASAGMLQGGASGEALLSHLLSDISPPADALRLRSPRGSPDGGLQTGKPSAPKKLKLDVGEAMAPPSHRKGDSNANSDV
CAAALRGLSRTILMCGPLIKEETHRRLHELVLPLVMGVQQGEALGSSPYTSSHCRRELYRLLLALLLAPSPRCPPPLACA
LRAFSLGQREDSLEVSSFCSEALVTCAALTHPRVPPLQSVGPTCPAPAPVPPPEAPAPFRAPAFHTPGPLPSAGTMPSAG
PMPPAGPLPPTRPGPPATANHLGLSVPGLVSVPPRLLPGPENHRAGSSEDPVLAPSGSPPPTVPPDETFGGRVPRPAFVH
YDKEEASDVEISLESDSDDSVVIVPEGLPPPPPPPPSGTTPPPVAPAGPPAASPPVPAKDEPEELPAAPGPLPPPPPPPVP
GPVTLPPPQLVPEGTPGGGGPPALEEDLTVININSSDEEEEEDEEEEEEDEEDEEEDFEEDEEEEEEYFEEEEEEEEEFEE
EFEEEEGELEDEEDEEEDEELEELEEVEFGPAAGDGEGGGPVPPSLPPPALPPAESPKGPPEPGLEPGLLLEVEEPGTEE
APGPETAPTLAPEVLPPQGETEREAGSPPAGPPPQELVEAEPSGPPALLEEGAEGGGEKVSPPEEAAAAEAAALPPEKE
QGDTAAMLADFIDCPPDDEKPPPASEPDS

> Dipodomys ordii [A0A1S3EWY4]
MELAVAVLRDLLRYAAQLPTLFRDISTNHLPGLLTSLLGLRPECEHAALEGMKACMTYFPRACGSLKGKLASFFLSRVDA
LSPQLQQLACECYSRLPSLGAGFSQGLKHTENWEQELHSLLTSLHSLLGALYEGSETAPVQSEDSGMETLLSHLDDVDTH
ALFRLQQRFSGLAHCMALMLSSEFGAPVSVPVQEVLDLICRILSVNGKNISLLGDGPLRLLLLPSIHLEALDLLSSLILA
CGGRLLRFGTLINRLLPQVLNVWSIGRDTLSPGQERPYSTIRIKVYAVLELWVKVCGASAGVLQGGAPGEALLTHLLSDI
SPPTDTLKLRSPRGNSDGGLQSGKPSAPKKLKLDMGDAMAPPSHRKGDSNANSDVCAAALRGLSRTILMCGPLIKEETHR
RLHDLVLPLLMSLQQGEVLGSSPYNSSCCRRELYRLLLALLAPSPRCPPPLACALQAFSFGQQEDSLEVSSFCSEALVT
CAALTHPRVPPLQTMGPTCATSAPVPPPEAPSSFRAPPFHPPGPMPSVGPMPTPGPISSAGPLPSAGPMPSAGPMSSTGP
IPSAGPLPSAGPIPSTRPGPPATTNHLGLSVPGLVSVPPRLLPGPENHRAGPSEDPVLASSGTPPPTIPPDETFGGRVPR
PAFVHYDKEEASDVEISLESDSDDSVVIVPEGLPPLPPPPPTSTPPPSVAPTGPPAASPPVPAKEEPEELSATPGSLPPP
PPPPPPPIAGSATLPPPQLVPEGAPGGGGPPALEEDLTVININSSDEEEEEEEEEEEEEEDDEEEEDFEEEDDDEEEYFEE
EEEEEEFEEEFEEEEGELEEEEEEEEEEEEEEELEEVEDLEFGPAGGEVEEGGPPPPTLPPALPPPESPKVQPEPEPEPGL
LLEVEEPGTEEEPGAETAPTLAPEVLPSQGEVEGEGGSPTVGPPPQELVEEPSAHPPLLEEETEGGSDKVPPPPETSEVE
EEMETEAETAALQEKEQDDTAAMLADFIDCPPDDEKPPPTMESDS
> Odocoileus virginianus texanus [A0A6J0Z636]
MAAAVLSGPSAGSAAGVPGGTGGLSAVGSGPRLRLMLLESVSGLLQPRTGSAVAPVHPPARSAPHLPGLMCLLRLHGTVG
GAQNLSAVGALVGLSNARLGSIKTRFEGLCLLSLLVGESPTEMFQQHCVSWLRSIQQILQSQDPPPTMELAVTVLKDLLR
YAAQLPAVSRDISMNHLPGLLTSLLGLRPECELSALEGMKACMTHFPRACGSLKGKLASFFLSRVDALSPQLQQLACECY
SRLPSLGAGFSQGLKHTDSWEQELRSLLASLHSLLGGLYEGAEAAPMQYESPGVETLLSPSEDADAHTLLQLRQRFSGLA
RCLGLMLSSEFGAPVTVPVQDILDLICRTLSVSAKNVSLLGDGPLRLLLLPSLHLEALDLLSALVLACGARLLRFGALIS
RLLPQVLNAWSIGRENLGPGQERPYSTVRTKVYAVLELWVQVCGASAGMLQGGASGEALLSHLLSDISPPADALRLRSPR
GSPDGGLQTGKPSAPKKLKLDVGEAMAPPSHRKGDSNANSDVCAAALRGLSRTILMCGPLIKEETHRRLHELVLPLVMGV
QQGEALGSSPYTSSHCRRELYRLLLALLAPSPRCPPPLACALRAFSLGQREDSLEVSSFCSEALVTCAALTHPRVPPLQ
SVGPTCPAPAPVPPPEAPAPFRAPAFHTPGPLPSAGTMPSAGPMPPAGPLPPTRPGPPATANHLGLSVPGLVSVPPRLLP
GPENHRAGSSEDPVLAPSGSPPPTVPPDETFGGRVPRPAFVHYDKEEASDVEISLESDSDDSVVIVPEGLPPPPPPPSGT
TPPPVAPAGPPAASPPVPAKDEPEELPAAPGPLPPPPPPPVPGPVTLPPPQLVPEGTPGGGGPPALEEDLTVININSSDE
EEEEDEEEEEEDEEDEEEDFEEDEEEEEEYFEEEEEEEEEFEEEFEEEEGELEDEEDEEEDEELEELEEVEFGPAAGDGEG
GGPVPPSLPPPALPPAESPKGPPEPGLEPGLLLEVEEPGTEEAPGPETAPTLAPEVLPPQGETEREAGSPPAGPPPQELV
EAEPSGPPALLEEGAEGGGEKVSPPEEAAAAAEAAALPPEKEQGDTAAMLADFIDCPPDDEKPPPASEPDS
> Bison bison bison [A0A6P3HSV7]
MMAVPPCEDDQGVQIDCYCGENLSAVGALVGLSNARLGSIKTRFEGLCLLSLLVGESPTEMFQQHCVSWLRSIQQILQSQ
DPPPTMELAVTVLKDLLRYAAQLPAVFRDISMNHLPGLLTSLLGLRPECELSALEGMKACMTHFPRACGSLKGKLASFFL
SRVDALSPQLQQLACECYSRLPSLGAGFSQGLKHTDSWEQELRSLLASLHSLLGGLYEGAEAAPMQYESPGAETLLSSSE
DADAHTLLRLRQRFSGLARCLGLMLSSEFGAPVSVPVQDILDLICRTLSVSAKNVSLLGDGPLRLLLLPSLHLEALDLLS
ALILACGARLLRFGALISRLLPQVLNAWSIGRENLGPGQERPYSTVRTKVYAVLELWVQVCGASAGVLQGGASGEALLSH
LLSDISPPADALRLRSPRGSPDAGLQTGKPSAPKKLKLDVGEAIAPPSHRKGDSNANSDVCAAALRGLSRTILMCGPLIK
EETHRRLHELVLPLVMGVQQGEALGSSPYTSSHCRRELYHLLLALLLAPSPRCPPPLACALRAFSLGQREDSLEVSSFCS
EALVTCAALTHPRVPPLQSVGPTCPAPAPVPPPEAPAPFRAPAFHAPSPLPSAGPMPSAGPMPPVGPLPPTRPGPPATAN
HLGLSVPGLVSVPPRLLPGPENHRAGSSEDPVLAPSGSPPPTIPPDETFGGRVPRPAFVHYDKEEPSDVEISLESDSDDS
VVIVPEGLPPPPPPSSGTTPPPVAPAGPPAASPPVPAKDEPEELPAAPGPLPPPPPPPVPGPVTLPPPQLVPEGTPGGGG
PPALEEDMTVININSSDEEEEEEEEEEEEDEEEDEEEDFEEDEEEEEEYFEEEEEEEEEFEEEFEEEEGELEDEEDEEED
EELEELEEVEFGPAGGEVEGGGPAPPSLPPALPPAESPKGPPEPGLEPGLLLEVEEPGTEEAPGPETAPMLAPEVLPSQG
EVEREGGSPPAGPPPQELVEEEPSGPPALLEEGAEGGGDKVSPPPEASAVEETEVEAAALPPEKEQGDTAAMLADFIDCP
PDDEKPPPASEPDS
> Bos indicus [A0A6P5DF41]
MMAVPPCEDDQGVQIDCYCGENLSAVGALVGLSNARLGSIKTRFEGLCLLSLLVGESPTEMFQQHCVSWLRSIQQILQSQ
DPPPTMELAVTVLKDLLRYAAQLPAVFRDISMNHLPGLLTSLLGLRPECELSALEGMKACMTHFPRACGSLKGKLASFFL
SRVDALSPQLQQLACECYSRLPSLGAGFSQGLKHTDSWEQELRSLLASLHSLLGGLYEGAEAAPMQYESPGAETLLSSSE
DADAHTLLRLRQRFSGLARCLGLMLSSEFGAPVSVPVQDILDLICRTLSVSAKNVSLLGDGPLRLLLLPSLHLEALDLLS
ALILACGARLLRFGALISRLLPQVLNAWSIGRENLGPGQERPYSTVRTKVYAVLELWVQVCGASAGVLQGGASGEALLSH
LLSDISPPADALRLRSPRGSPDAGLQTGKPSAPKKLKLDVGEAIAPPSHRKGDSNANSDVCAAALRGLSRTILMCGPLIK
EETHRRLHELVLPLVMGVQQGEALGSSPYTSSHCRRELYHLLLALLLAPSPRCPPPLACALRAFSLGQREDSLEVSSFCS
EALVTCAALTHPRVPPLQSVGPTCPAPAPVPPPEAPAPFRAPAFHAPSPLPSAGPMPSAXPMPPVGPLPPTRPGPPATAN
HLGLSVPGLVSVPPRLLPGPENHRAGSSEDPVLAPSGSPPPTIPPDETFGGRVPRPAFVHYDKEEPSDVEISLESDSDDS
VVIVPEGLPPPPPPSSGTTPPPVAPAGPPAASPPVPAKDEPEELPAAPGPLPPPPPPPVPGPVTLPPPQLVPEGTPGGGG
PPALEEDMTVININSSDEEEEEEEEEEEEDEEEDEEEDFEEDEEEEEEYFEEEEEEEEEFEEEFEEEEGELEDEEDEEED
XELEELEEVEFGPAGGEVEGGGPAPPSLPPALPPAESPKGPPEPGLEPGLLLEVEEPGTEEAPGPETAPMLAPEVLPSQG
EVEREGGSPPAGPPPQELVEEEPSGPPALLEEGAEGGGDKVSPPPEASAVEETEVEAAALPPEKEQGDTAAMLADFIDCP
PDDEKPPPASEPDS
> Delphinapterus leucas [A0A2Y9Q8F1]
MELAVAVLGDLLRYAAQLPTLFRDISMNHLPGLLTSLLGLRPESELSALEGMKACMTYFPRACGSLKGKLASFFLSRVDA
LSPQLQQLACECYSRLPSLGAGFSQGLKHTDSWEQELHSLLASLHSLLGALYAGADTAPMQYEGPGVETLLSPSEDGDAH
VLLRLWQRFSGLARCLGLMLSSEFGAPVSVPVQEILDLICRTLSVSAKNISLLGDGPLRLLLLPSLHLEALDLLSALILA
CRGRLLRFGALISRLLPQVLNAWSIGRDSLSPGQERPYSTMRTKVYAVLELWVQVCGASAGVLQGGASGEALLTHLLSDI
SPPADALKLRSPRGSPDGGLQTGKPSAPKKLKLDMGEALAPPSHRKGDSNANSDVCAAALRGLSRTVLMCGPLIKEETHR
RLHDLVLPLVMGVQQGEVLGSSPYTSSRCRRELYRLLLALLAPSPRCPPPLACALQAFSLGQREDSLEVSAFCSEALVT
CAALTHPRVPPLQTMGPACPTPAPVPPPEAPSPFRAPAFHPPGPMPSVGPMPSVGPMPSAGPMPSPGPVPPAGPMPSVGP
MPPARPGPPATANHLGLSVPGLVSVSPRLLPGPENHRAGSNEDAVLAPSGTPPPTLPPDETFGGRVPRPAFVHYDKEEAS
DVEISLESDSDDSVVIVPEGLPPLPPPPPSGTTPPPAALVGPPTASPPVPAKEEPEELPAAPGPLPPPPPPPVPGPVALP
PPQLVPEGTPSGGGPPALEEDLTVININSSEEEEEDDEEDEDEEDFEEEEEEEEEEEYFEEEEEEEEEFEEEFEEEEGEL
EEEEEDEDEEEEVEEVEFGPAGGEVEEGGPAPPSLPPALPPAASPKVQPQPEPEPGLLLEVEEPGAEEGPGAETAPTLAP
EVLPSQGEVEREGGSPPAVPPPQELVEEEPSVPPTLLEEGAEGGGDKVPPPPEASAAEEMETEAEAAALQEKEQDDTAAM
LADFIDCPPDDEKPPPATEPDS
> Bos indicus [A0A6P5DHI9]
MFQQHCVSWLRSIQQILQSQDPPPTMELAVTVLKDLLRYAAQLPAVFRDISMNHLPGLLTSLLGLRPECELSALEGMKAC
MTHFPRACGSLKGKLASFFLSRVDALSPQLQQLACECYSRLPSLGAGFSQGLKHTDSWEQELRSLLASLHSLLGGLYEGA
EAAPMQYESPGAETLLSSSEDADAHTLLRLRQRFSGLARCLGLMLSSEFGAPVSVPVQDILDLICRTLSVSAKNVSLLGD

GPLRLLLLPSLHLEALDLLSALILACGARLLRFGALISRLLPQVLNAWSIGRENLGPGQERPYSTVRTKVYAVLELWVQV
CGASAGVLQGGASGEALLSHLLSDISPPADALRLRSPRGSPDAGLQTGKPSAPKKLKLDVGEAIAPPSHRKGDSNANSDV
CAAALRGLSRTILMCGPLIKEETHRRLHELVLPLVMGVQQGEALGSSPYTSSHCRRELYHLLLALLLAPSPRCPPPLACA
LRAFSLGQREDSLEVSSFCSEALVTCAALTHPRVPPLQSVGPTCPAPAPVPPPEAPAPFRAPAFHAPSPLPSAGPMPSAX
PMPPVGPLPPTRPGPPATANHLGLSVPGLVSVPPRLLPGPENHRAGSSEDPVLAPSGSPPPTIPPDETFGGRVPRPAFVH
YDKEEPSDVEISLESDSDDSVVIVPEGLPPPPPPSSGTTPPPVAPAGPPAASPPVPAKDEPEELPAAPGPLPPPPPPPVP
GPVTLPPPQLVPEGTPGGGPPALEEDMTVININSSDEEEEEEEEEEEEDEEEDEEEDFEEDEEEEEEYFEEEEEEEEEF
EEEFEEEEGELEDEEDEEEDXELEELEEVEFGPAGGEVEGGGPAPPSLPPALPPAESPKGPPEPGLEPGLLLEVEEPGTE
EAPGPETAPMLAPEVLPSQGEVEREGGSPPAGPPPQELVEEEPSGPPALLEEGAEGGGDKVSPPPEASAVEETEVEAAAL
PPEKEQGDTAAMLADFIDCPPDDEKPPPASEPDS
> Tursiops truncates [A0A6J3QLP8]
MFQQHCVSWLRSIQQVLQSQDPPPTMELAVAVLGDLLRYAAQLPTLFRDISMNHLPGLLTSLLGLRPESELSALEGMKAC
MTYFPRACGSLKGKLASFFLSRVDALSPQLQQLACECYSRLPSLGAGFSQGLKHTDSWEQELHSLLTSLHSLLGALYAGA
DTAPMQYEGPGVETLLSPSEDGDAHVLLRLWQRFSGLARCLGLMLSSEFGAPVSVPVQEILDLICRTLSVSAKNISLLGD
GPLRLLLLPSLHLEALDLLSALILACRGRLLRFGALISRLLPQVLNAWSIGRDSLSPGQERPYSTMRTKVYTVLELWVQV
CGASAGVLQGGASGEALLTHLLSDISPPADALKLRSPRGSPDGGLQTGKPSAPKKLKLDMGEALAPPSHRKGDSNANSDV
CAAALRGLSRTVLMCGPLIKEETHRRLHDLVLPLVMGVQQGEVLGSSPYTSSRCRRELYRLLLALLLAPSPRCPPPLACA
LQAFSLGQREDSLEVSAFCSEALVTCAALTHPRVPPLQTMGPACPTPAPVPPPEAPSPFRAPAFHPPGPMPSVGPMPSVG
PMPSAGPMPSPGPVPPAGPMPSVGPMPPARPGPPATANHLGLSVPGLVSVSPRLLPGPENHRAGSNEDAVLAPSGTPPPT
IPPDETFGGRVPRPAFVHYDKEEASDVEISLESDSDDSVVIVPEGLPPLPPPPPSGTTPPPAAPVGPPTASPPVPAKEEP
EELPAAPGPLPPPPPPPVPGPVALPPPQLVPEGTPSGGGPPALEEDLTVININSSEEEEDDDEEDEDEEDFEEEEEEEEE
EEYFEEEEEEEEEFEEEFEEEEGELEEEEEDEDEEEEVEEVEFGPAGGEVEEEGPAPPSLPPALPPAASPKVQPQPEPEP
GLLLEVEEPGAEEGPGAETAPTLAPEVLPSQGEVEREGGSPPAVPSPQELVEEEPSVPPTLLEEGAEGGGDKVPTPPEAS
AAEEMETEAEAAALQEKEQDDTAAMLADFIDCPPDDEKPPPATEPDS
> Branchiostoma belcheri [A0A6P4Y102]
MAAHMASVLLTSLSEKEGKTLLSWTIEAANEHQLLQSEQSAQDWVSHINTSLGAAKTRLEGLCLLGTVVRQCSADTFIQH
GTTWIRLLAQVLQAYDSPLTLQMASHVLAAVVQQAAQYPEVAREVASTHVPALVQSLLGAQDHQWFPAALEALHSCMKNF
PGPCGPSKGKVESVLCGLMDTNQPRLSLLVQQTCPLLAGCGGGGAGGVKYAEAWAHLCDQVLGSLHQVLDHAYQDMETGL
QTYNTPQASLHLPTVPESDPARTFVLSTRFHNLCGCLQQLVSQEFPSVVRIPTPDILSFLCRALGVNAKMLFGKASMEHV
LLMSALPKMHCSALSILEALIISCRSYLVPHASIISQLLIQTLGWTTSEEGVPGRQRPHSTLRSRAYTVLTVWLNVCGAA
GGVDSHADIILQHVLTDTTPQADTTKLKASRPGSQEPPRRQKKKQRGVDVSDQGLSGHRKVDSQANSDVCSAALGVLKTV
LEVVGPIIKPSFHKEVQEFVIPLLLKIQQNQSDPPIPYSCAKCRKGLYQLLLAAVLVLHPRWPPPTQCAVKIFSVGQQDY
DLQVSSYCREALLTCMSIIHPRAPTLQSPVIISDTPTISKPSTNHQAPSFQVLRSNATQKNDSTNGNLFTAPSFKAASQS
EGMVKDCPSQSESTSPKSIQMERSGTFSEKLTREEDKAGPSVIRLDGSSNSSEGEESSSGDSVELLEDIQSSKQDSSTLE
TRDLNDGVSNTEKKLATDMETVPENEDVVLIQGCTSAGRAVVPADTTDIPTAGGVKRKHESDVEGEEDDSQDTDIQAMLA
SFVPDSTPDEN
> Lingula unguis [A0A1S3HU57]
MHHRFWDSSEFQKASQIQDEIRKINSGLNAAKSRVESLVLLNDLLSQCSTETLSQYTITWTRLLIQIIKGYAPASIHRLA
CYVLGSLTEKSSTCPELARQVALDSLPQLIPALLVMKEESLEAALYCIGKCMQFYSGPCGTFKGKIENWVIDQLTKENPR
VVEAAVSVFPWLSQCGGGGNQGIKHAESWSSNCHKLLGSLHDTLDQLYEGVESGVEENKKKSEKLSLSPVQGTGMERIVG
LRNRLDMLCGCLGGMLSTTFPAVVRIPVPEVIGFIQRALAVTGRALLLRPSTDNLQLLSVLPFLHTSVLRVLSCLISRYV
LPFLHTSVLRVLSCLISRYVLPFLHTSVLRVLSCLILSCQKNLISYTSTVNQLLIQTLSWTITEDTADGRKRPYGNLRKE
VYSTLVVWVQTLGASSNVHNIIDQLLTQLKRDITPDVDVIKLETINKKPDQYTEPPNKRKKGNKAGLEGVSTRKVLDHSA
NADVCLSALAALRHLLVAVGSLLKVQRIRDIQEIALPLALRVQAFTDQGSPGPYSDPECRRELYHVIQCLFLIPASKCPP
PTHCVIRLFTGGQNDPDRKVSSFCQEALTICEAVIHPRVPSLQQTILWTVDGSKIQDVNKTSQVSKSTVDNSLFISGKNR
QCGESIFTTSFQDSQSVVKQTNSLIKDDTIGKSGAPSDLPAAMDKPTAEENSSESEEECSDVAMETEEKEVKKSKRVSTT
ESLTTKPDDIFTEASTSKDDTLSGNGNEFFSVMSTQPTGTKAATVAPPVGGNRNERTATAVTPAGEFLSEDEETKTHKRK
LSEMEESSDQVESMLASFVDAQPDSD
> Actinia tenebrosa [A0A6P8HB07]
MAADTAVVVGSYLNDNNKTDKHIFTLLKCVKENQCLLEYECSKDWTAKANSFLNSKTERWLGISLLATIIPQCDTETFTI
NCITWIKAIVQVIQSSSASSSLIQCSVRVMDDLMKYAIQFPDISRTIANSVMPSLISALLSRKDKDVDSILSGICTCVRY
MPGPVAPFKVQIESYIIPCLDSWNTKIRKNTCQCFALLAGCSSNTTSTDKYKTNWECQWNRVLATINHALNNLYANVEMS
NNPLNDTLLGKTETLAVSDITDAEPERTNVLTARLISLLDCLNIMTREGTHRISMIPIEDTLLLVERIVAVNGNMLKSVS
VDVVLLKTCLSVIHESAIRLLNSLLQRCRSLLLPNNHQISNVLIRELSWTLNDKSQDKRHDGHKPYSNLRCILFKCFINW
CHLKASISTDIIKKVILNILTDIRPQTKQTKLMNPDDHKQPKSSNKAKKRKLQEMEQLSTSQKKENPIHNLCVCHAALSA
LYAILMNGVANLTKDLYQEIQGVLLNLILQCQVSVPGRMPSPYSDADCRKSLYQCLLACVVACPPQAPSSIQCAIRCCTR
GLQDMHPKVSSYCREALTILTAIIHPLVPSLESATQFVMLPTNSKTCVVQDNDKHQETNALSAETSVVNQNGSEVPAHDG
SRLLEGTVLDVPVGVCESMQASSNTYTKTQPRENEELQINTDFNTDIQQINIFRSTLSNQDPMDTRESINTQAIECTKHS
DLYSSSKHQSKSDVLTKDNVDIAASGASKSDVFESDLQEDTNISLPLTDTLDAESIEMSPAKMQKLGAGKHIVVRKDEER
HEGKTSIGNAKTDEDMGDVDEMLATFVNTSPDSDSD
> Pteropus alecto [L5JZU1]
MAAAVLSGPSAGSAAGVPGGTGGLSAVGSVPRLRLLLLESVSGLLQPRAGSAIAPVHPPVRSAPHLPGLMCLLRLHGTVG
GAQNLSAVGALVGLSNARLGSIKTRFEGLCLLSLLVGESSTEMFQQHCVSWLRSIQQVLQSQDPPPTMELAVAVLRDLLR
YAAQLPTLFRDISMNHLPGLLTSLLGLRPECELSALEGMKACMTYFPRACGSLKGKLASFFLSRVDALSPQLQQLACECY
SQVPSLGAGFSQGLKHTESWEQELHSLLASLHSLLGALYEGAEPAPMQYEGPGVEMLLSPSEDGDAHILLRLWQRFSGLA
RCLGLMLSSEFGAPVSVPVQEILDVICRTLSISAKNISLLGDGPLRLLLLPSIHLEALDLLSALILACRGRLLRFGALIS
RLLPQVLNAWSIGRDSLSPGQEKPYSAMRTKVYAILELWVQVCGASAGVLQGGASGEALLTHLLSDISPSADSLKLRSPR
GSPDGGLQMGKPSAPKKLKLDVGEAMAPPSHRKGDSNANSDVCAAALRGLSQTILMCGPLIKEETHRRLHDLVLPLVMGV
QQGEVLGSSPYTSSCCRRELYHLLLALLLAPSPRCPPPLACALQAFSLGQREDSLEVSSFCSEALVTCAALTHPRVPPLQ
SMGPTCPTPAPVPPPEAPSPFRAPPFHPPGPMPSAGPMPSAGPVPSVGSVPSVGSMPSAGPMPSAGPMPPTRPGPPATAN
HLGLSVPGLVSLPPRLLPGPENHRAGSNEDPVLAPSGTPPPTIPPDETFGGRVPRPAFVHYDKEEASDVEISLESDSDDS
VVIVPEGLLPLPPPPPSDTTPPPVTPAGPPTASPPLPTQEEPEELPVAPGPLPPPPPPPVPAPVTLAPPQLVPEGTSGGG
GPPALEEDLTVININSSDEEEEEEEEEDEEEEEEEDFEEEEEDEEEYFEEEEEEEEEFEEEYEEEEGELEEDEEEDEEEE
EELEEVEELEFGSAGGEIEEGGPPPPNLPPALPPVESPKLHPEPEPEPEPEPEPEPGLLLEVEEPGAEEEHGAETAPTLAPE
VLPSQEELEREAESPPAGPPQELVEEEPCAPPALLEEGTEVGSDKVPPPPETSAAEEMQTETEATALQEKEQDDTAAMLA
DFIDCPPDDEKLPPATEPDS
> Fukomys damarensis [A0A091CWA6]

MAAAVLSGPSAGSPAGVPGGAGGLSAMNSGPRLRLLLLESVSGLLQPRTGSGVAPVHPPVRSAPHLPGLMCLLRLHGTVG
GAQNLSAVGALVNLSNACLGSLKTRFEGLCLLSLLVGESPTDMFQQHCVSWLRSIQQVLQSQDPPPTMELAVAVLKDLLR
YAAQLPALSRDISVNHLPGLLTSLLGLRPECEQSALEGMKACMTYFSRACGSLKGKLASFFLSRVHSLSPQLQQLACECY
SKLPSLGAGFSQGLKHTESWEQELHSLLASLHSLLGALYEGAETAPVQNEGPDVETLLSHSEEGDAHVLLRLQQRFSGLA
RCLGLMLSSEFVAPVSVPVQEVLDLICRILSVSGKNISLLGDGPLRLLLLPSIHLEALDLLSALILACGGRLLRFGSLIS
RLLPQVLNAWSIGRDTLSSGQERPYSTIRTKVYAVLELWVQVCGTSAGVLQGGASGEALLTHLLSDISPPADALKLRSPR
GSPDGGLQTGKPSAPKKLKLDMGDAVAPPSQRKGDNNANSDVCAAALRGLSRTILMCGPLIKEETHRRLHDLVLPLAMGV
QQGEVLGSSPYTSSCCRRELYRLLLALLLAPSPRCPPPLSCALQAFSLGQREDSLEVSSFCSEALVTCSALIHPRVPPLQ
TVGPTCPTPTSVPPHEAPSLFRAPPFHPPGPMPSVGPMPSAGPMPSSGPLPSAGPMPSVGSMPSAGPVPPARPGPPATSN
HLGLSVSGLVSVPPRLLPGPENHRAGSSEDPVLAPSGTPPPTIPPDETFAGRVPRPAFVHYDKEEASDVEISLESDSDDS
VVIVPEGLPPLPPPPPSGNSPPPVAPTGPPTASPPVPAKEEPEELPAAPGPLPPPPPPPVPGPVTLPPPQLVPEGTPGGA
GPPALEEDLTVININSSDEEEEEEDEEEEEEEEEEEEEEDFEEEEEEEEEYFEEEEEEEEFEEEFEEEEGELEEEEEEEE
EEEEELEEVEDLEFGSAGGEVEESGPPAPTLPPALPPPESPKVPPEPEPEPGLLLEVEEPGTEEEPGAETAPTLAPEVLP
SQGEVGGESPTGGPAPQEPVEEEPSDPPTLLEEETEGGGDKVLSLPETPAEEMEAEATAVQEKEEDDTAAMLADFIDCPP
DDEKPPPPTEPDS
> Heterocephalus glaber [G5BWB3]
MAAAVLSGTSAGSPAGVPGGPGGLSAMNSGPRLRLLLLESVSGLLQPRTGSSVAPVHPPVRSAPHLPGLMCLLRLHGTVG
GAQNLSAVGALVNLSNACLGSMKTRFEGLCLLSLLVGESPTDMFQQHCVSWLRSIQQVLQDPPPTMELAVAVLKDLLRYA
AQLPTLSRDISVNHLPGLLTSLLGLRPECEQSALEGMKSCMTYFPRACGSLKGKLASFFLSRVHSLSPQLQQLACECYSK
LPSLGAGFSQGLKHTESWEQELHSLLASLHSLLGALYEGAETAPVQNEGPGVETLLSHSEEGDAHVLLRLRQRFSGLARC
LGLMLSSEFEAPVSVPVQEVLDLICRILSVSGKNISLLGDGPLRLLLLPSIHLEALDLLSALILACGGRLLRFGSLISRL
LPQVLNAWSIGRDTLSSGQERPYSTIRTKVYAVLELWVQVCGASAGVLQGGASGEALLTHLLSDISPPADALKLRSPRGT
PDGGLQTGKPSAPKKLKLDMGEAVAPPSQRKGDSNANSDVCVAALRGLSRTILMCGPLIKEETHREVLGSSPYTSSCCRR
ELYRLLLALLLAPSPRCPPPLSCALQAFSLGQREDSLEVSSFCSEALVTCAALTHPRVPPLQTMGPTCPTPASVPPPEAP
SLFRAPPFHPPGPMPSVGPMPSVGPMPAVGPMPSAGPLPSAGPMSSVAPMSSVGPIPSSGPMPSTGPVPPARPGPPATSN
HLGLSVPGLVSVPPRLLPGPENHRAGSSEDPVLAPSGTPPPTIPPDETFGGRVPRPAFVHYDKEEASDVEISLESDSDDS
VVIVPEGLPPLPPPPPSGTSPPPVAPTGPPTASPPMPAKEEPEELPAAPGPLPPPPPPPIPGPVTLPPPQLVPEGTPGGA
GPPALEEDLTVININSSDEEEEEEDEEEEEEEEEEEEEDFEEEEEEEEEEYFEEEEEEEEFEEEFEEEEGELEEEEEEEE
EEEEELEEVEDLEFGSVEESGPPPPTLPPALPPPQSPKVPPVPEPEPGLLLEVEEPGAEEEPGAETAPTLAPEVLPSQGE
VEREGESPTGGPPPQEPVEEEPSDPPTLLEEETEGGGDKVPPLLETPAEEMETEAEATAVQEKEEDDTAAMLADFIDCPP
DDEKPPPTMEPDS
> Myotis brandtii [S7Q3E5]
MDPNLSAVGALVGLSNARLSSIKTRFEGLCLLSLLVGESSTEMFQQHCVSWLRSIQQVLQSQDPPATMDLAVVVLRDLLR
YAAQLPTVFRDISMNHLPGLLTSLLGLRPECELSALEGMKACMTYFPRACGSLKGKLASFFLSRVDSLSPQLQQLACECY
SRLPSLGAGFSQGLKHTESWEQELHTLLASLHSLLGALYEGAEPAPVQYEGPGVEMLFSLEDADAHNLFRLRQRFSGLAC
CLGLMLSSEFVAPVSIPVQEILDFICRTLSISAKNISLLGDGPLRLLLLPSIHLEALDLLSALILACGGRLLRFGALISR
LLPQVLNAWSIGRDTVSLGQEKPYSAMRTKVYAVLELWVQVCGASAGVLQAGASGEALLTHLLSDISPPAEALKLRSPRG
SPDGSLQTGKPSAPKKLKLDMGEAATPPSHRKGDSNANSDVCAAALRGLSRTILMCGPLIKEETHRRLHDLVLPLVMGVQ
QGEVLGSSPYTSSCCRRELYRLLLALLLAPSPRCPPPLACALQAFSLGQREDNLEVSSFCSEALVTCAALTHPRVPPLQS
MGPTCPTPAPVPPPEAPSPFRAPPFHPPGPMPSAGTMPSAGPVPSVGPLPSAGPVPPAGPVPPARPGPPATANHLGLSVP
GLVSVPPRLLPGPENHRAGSNEDPILASSGTPPPVIPPDETFGGRVPRPAFVHYDKEEASDVEISLESDSDDSVVIVPEG
LPPLPLPPPSGSTPPPVAPAGPPAASPPLPAKEEPEELPTVPGPLPPPPPPPVPGPVTLQPPQLVPEGTPSGGGPPALEE
DLTVININSSDEEEEEEEEEEEEEEEEEEEEEDFEEEEEDEEEYFEEEEEEEEFEEEFEEEEGELEEEEEEEEEEEEEELEE
VEELEFGSAGGEVEEGGPPPTSLPPALPPPESPKVQPEPEPEPGLLLEVEEPGAEEERGAETAPTLAPEVLPSQEELEKE
GGSPPAGPPPQELVEEEPCAPPPALLDEGTEGGGDEVPPHQPETSAAEEMETETAPPQEKEQDDTAAMLADFIDCPPDDE
KPPPVTEPDS
> Myotis davidii [L5LDL4]
MSEGPVVGRSRKEGHCAWGSGGGQERGCCGAGENLSAVGALVGLSNARLSSIKTRFEGLCLLSLLVGESSTEMFQQHCVS
WLRSIQQVLQSQDPPATMDLAVVVLRDLLRYAAQLPTVFRDISMNHLPGLLTSLLGLRPECELSALEGMKACMTYFPRAC
GSLKGKLASFFLSRVDSLSPQLQQLACECYSRLPSLGAGFSQGLKHTESWEQELHTLLASLHSLLGALYEGAEPAPVQYE
GPGVEMLFSLEDADAHNLFRLRQRFSGLASCLGLMLSSEFVAPVSVPVQEVLDFICRTLSISAKNISLLGDGPLRLLLLP
SIHLEALDLLSALILACGGRLLRFGALISRLLPQVLNAWSIGRDTVSLGQEKPYSAMRTKVYAVLELWVQVCGASAGVLQ
AGASGEALLTHLLSDISPPAEALKLRSPRGSPDGSLQTGKPSAPKKLKLDMGEAATPPSHRKGDSNANSDVCAAALRGLS
RTVLMCGPLIKEETHRRLHDLVLPLIMGVQQGEVLGSSPYTSSRCRRELYRLLLALLLAPSPRCPPPLACALQAFSLGQR
EDNLEVSSFCSEALVTCAALTHPRVPPLQSMGPTCPTPAPVPPPEAPSPFRAPPFHPPGPMPSAGTMPSVGPVPSVGPLP
SAGPVPPAGPVPPARPGPPATANHLGLSVPGLVSVPPRLLPGPENHRAGSNEDPILASSGTPPPVIPPDETFGGRVPRPA
FVHYDKEEASDVEISLESDSDDSSTPPPVAPAGPPAASPPLPAKEEPEELPTVPGPLPPPPPPPVPGPVTLQPPQLVPEG
TPSGGGPPALEEDLTVININSSDEEEEEEEEEEEEEEEEEEEEEDFEEEEEDEEEYFEEEEEEEEFEEEFEEEEGELEEEE
EEEEEEEELEEVEELEFGSAGGEVEEGGPPPTSLPPALPPPESPKVQPEPEPEPEPGLLLEVEEPGAEEERGAETAPTLA
PEVLPSQEELEKEGGSPPAGPPPQELVEEEPSAPPPALLDEGTEGGGDEVPPPPETSAAEEMETETTPPQEKEQDDTAAM
LADFIDCPPDDEKPPPVTEPDS
> Tupaia chinensis [L9KMD2]
MFQQHCVSWLRSIQQVLQSQDPPPIMELAVAVLKDLLRYAAQLPVLFRDISINHLPGLLTSLLGLRPECEQSALEGMKAC
MTYFPRACGSLKGKLASFFLSRVDALSPQLQQLACECYSRLPSLGAGFSQGLKHTESWEQELHSLLASLHSLLGVLYEGA
ETAPVQYEGPGLETLLSPSEDGDTHVLLRLRQRFSGLARCLGLMLSSEFGAPVSVPVQEILDVICRTLSVSSKNISLLGD
GPLRLLLLPSIHLEALDLLSALILTCGGRLLRFGVLISRLLPQVLTAWSIGRDSLSPGQERPYSTLRTKVYAVLELWVQV
CGASAGVLQGGASGEALLTHLLSDISPPADTLKLRSPRGSPDSNLQTGKPSAPKKIKLDMGETIAPPSHRKGDSNANSDV
CTAALRVDKEDLEKLNDLRVVGSDRADDGLGLRFTLSPAHSHLTPPSPFSGLRQTILMCGPLIKEDTHRLLGAPPPPPPP
PSRRELYRLLLALLLAPSPRCPPPLSCALRAFSRGQQEDSLEVSSFCSEALVTCAALTHPRVPPLQPMGSTCPTPAPVPP
PEAPSPFRAPPFHPPGPVPSVGPLSSTGPMPSAGPVSSAGPMPSTGPVPSGGPMPSAGPMTSARPGPPATANHLGLSVPG
LVSVPSRLLPGPENHRAGSNEDPVLAPSGTPPPSIPPDETFGGRVPRPAFVHYDKEEASDVEISLESDSDDSVVIVPEGL
PPLPPPPPSGTTPPPVPPTGPPTASPPVPAKEEPEELPAAPGPLPPPPPPPVSGPVTLPPPQLVPEGTPGGGGGPALEED
LTVININSSDEEEEEEEEEEEEEEEEEEEEEEEDFEEEEEEEEEEYFEEEEEDEEEFEEEFEEEEGELEEEEEEEEDEEEEEEL
EEVEELEFGSAGGEVEEGGPPPPALPPALPPPESPKVPPEPEPEPEPGLLLEVEEPGAEEEPGAETAPTLAPEVLPSQEE
VEREGESPAAGPPPQELVEEEPSAPPTLLEEGTEGGGEKSPLPPETAAEEETGTEAEAATLEEKVEEFQVDVQEYLGPLL
FVKLRKRHLLQDDAWFCNWISVQGPGASGDEVRFPFYRWVEGKDILSLPEATGSNSSLFTGFPKSLQSLDQLCKFATMCI
FTCTGQHSSNHLGQLDWYAWVPNAPCTMRIPPPTTKDVTMETVMASLPNVHQASLQMSITWQLGRRQPIMVALGQHEEEY

FSDPASKAVLKTFREKLAAMDKDVDARNATLAMPYEYLKPSLVENSVAI
> Alligator mississippiensis [A0A151P2X8]
MKYLFIFVMEFAYFVGDQNAPALGGLVGVTSARLGCGKTRFEGLCLLSLLVQESPSELFQQHCLAWLRSLQHLLQSQDPA
ATVALGVTVLRDLLLYSAQLPELARDISTNHIPGLLTSLLALKPECQLSTLEGTRACMTHYPRACGSLRDKLAAYFLARV
DSKSPELQQLACKCYALLPRLGGGFPQGLPRRECWEQELHCILATLHGLLGTLYEGAETDPVPYEGPGVELLLPALQDGE
PGCIPSLRARFAGLARCLCLMLSNEFGSPVTVPVQDILNLVCRALNISSKNISWFGDGPLKMLLLPSIHLESLDVLAALI
VACGVRLARWGSVLARLFSQVLDVWSGARDAPPGQEKPYSAVRSRLYQVLELWLQVAGAGGGVLQGSGPLSEALLGHMLS
DIMPPTDSIKMRTGHHSLSEGGKPSAPKKPKLMEGAGDAPALHRKVDPAANSDVCQAALQALYRAVLLGGPLIKEEMHRR
LQELVVPLLLRLSQDGGPTGSPYARPACRLALHRLLLALLLAPAPAAPPPPLHCALRIFTQGRHDPSLEVSSFCAEALVI
CSAPEEEVVVAIKEEVEEVDEAATMLADFIDCPPDDDKTPPETGS
> Channa argus [A0A6G1Q512]
MATSVWLHGPAAMRLTEGLVSVLKEHRPEYLPALLANYREHGVFPTQGASTVGGLVGFSSAKLSSSKTRFEGLCLLSMLV
KDSSSDLFQQHCISWLRSLQQVIQSQAPVQTIQLAVHILKDLLQYSSQLAELAREVGLNFILGFLTSLLGLKAECELAAM
EGMMACMTYYPRACGSLRDKLGAYFISKMDSTNKKTQEMACQCYGHLPCLGGLLERAVSAGRAEGWTNQIHCLLASAKSL
LGQIYSGSETDGTAQYEGPGVDLAFPHLDQSDPLLLLQLQHRYTAVCLALKHTLGVDPASAVRLPVRPLLNLVCRALAVN
VKSINLTDGSVRILILPIIHINTLEVLSALITIVQSGMVQYAAVLQRLFSQTLSAWTPSHEASLGQQRAYSSVRVSVYRT
LELWVQVAGASASILEGSPSHSELLFSHLLGDITPGGESVKLRAGLSADVVPGGKPGPRRTKPLVIADAVVPSLQRKGDL
LANQDTCLSALKALRQIILTSGTLLKEDIHKRLHDVVLPLCVRLQQQTSSSTACESAGGVSGQYSSALSRRELYRLLLA
LVLVPSPCWPPPLTCAVSILSNGRTDHNLKVSAFCTEALTICNSLLHPRTPSIALPLPSLTLKPNPTAPVVPSSQGPTPG
LTLPTLLGGPTSGPPFPTRHSLSLGHASLLGSLDNHLSLVPGLQGQAPTPGDMILSPRTHHQPDPTGLGPPEGQRPVFVR
YDKEEAEDVEISLASDSDDSVVIVPPGMLNIENQEDDTAASISQNITSAATGNTVTLPGAEPVTMVSTTAAAATTIDGVS
LSNDLSTSSPLITTSTTPINSFPPSSTSVVSLVPPLNSSTLTAAPGGLGDSLPGRPQLQQMLMQPSTPGQPSSMGLPLQM
HHLQNQISQQGRHLHQPPPPASNEDSAVININSTDEEEEEEEDMEDDEELEEEEEGMDEEDEDEEVSDFADEEFYDGEDY
EDYDEEEGEELEEEEEEEDEDGDIPLLEGADDKAGEVGIEKGKVLQAAINEVGIAGFSMEAEGGIEEVQTNRVRFGEDRI
KVQEVESIGVLEEAREGERDEDESERMDDPTMPQILCVTGGALEERDETEEEGGGAGGEILQKASSWEQGANETEPTGPS
EDVTTNHNRQESRDESTKQTNVSDYQLPGHQEEQPVAVQQEDSATADLEPSSETDVNTEAQAETDIEKKDTMQEEKEETE
AGDGGVSDGDEGKGVKRKREEESGQSTEKKKLDDDAMASMLADFVACPPDEEEGASGSKCS
> Triplophysa tibetana [A0A5A9PQA1]
MASAAWLHGPNISRLTEGLVSVLKEERPEHLPALLANYREHGVVGIQSTGAVGGLVGITNVRLGSSKTRFEGLCLLSVLV
KDSSSEVFHQHCLSWLRSLQQIIQSQAPLPTVQLAVSVLQDLLQYSSQLPELAREVGLNSVLGILTSLLSLKSECHLASM
KGMMACMIYYPRACGSLKEKLGAYFLSKMDSDNTKVQELACVCYGRLPCLGGVLERGGGGHRAEGWTSQIHCLLASANSI
LGQLYQGSEAEGTLQYEGTVVELPFPPQDDLDPLLILQLHHRLKAICLALKHTLSVDPASSVRLPVQNVLNLVCRALAVN
TKSISPTGEGCLKLLVLPSVHIDTLELLSALIKIVGAGVVQYSSVFTRLVSQSLSAWTPLPETSLGQQRAYSAVRVAVYR
TLELWVRVGGASLLQGSPSHLELLFTHLIADITPGAEAVKLRSGPHSAMTDLVGSAGKPGPRRTKGLGMSEGISLQRKGD
VLANQDTCVAALRSLRQIVLASGTLLKEDLHKRLQDLVVPLCVRLQQQSHCTSEAAGVSGQYGSPTPRRELYRLLLALVL
VPSPRWPPPLSCAVSAFSHGRRDHNIMVSSFCAEALTICNTLIHPRTPSISLPLPPLTLKTTPSAPVLASGQNPSLSVPN
LLAGHGTAPHFPARHPMGLGPPTLVGSLENHLPLAPPVLPTSAGLSGTPSDLLLSPVLPNELAGLGTPEGQRQVFVRYDK
EEPDDVEISLESDSDDSVVIMPQGMLMEMQDGATNAQTLSQLPPTGGALPPSAPLVGEVGSGETALPNELATSIPHQILP
ANDNNINSHPGPSQTQLVSLVPPLNSATPPLSASPAGLADSMNSGPQLQQMLMQTSPAGPPPALGLSLQMQLQTQIAQTS
RQLQQQPAANDVDQNVININSTDDEDDEEDEMEEEDEELIDEEDEEEGLDDEEEEEEEEGSDFLEDEEFEDYDEDEEEDE
EENEEIQPMERDDARGMIGQGEAEVIIEPEEQQGMGIFCMEREQEVEGGIEEMEGVRSVYTDDGTKEKGMGEEIENIGAV
ERNEGASDQQQIETLLIGGDVEGPAEDTAVEAVEPEVRTWEQEARPEDAKEQAGPSQRCPELLAEEIQEQAPELQPEDKT
EESTASTSEPPALQNVDETTENENVGGEQEDEADLRGTKRKMEDREEGDSTEQGTEKKKMDDEAMASMLADFVDCPPDDE
DHGASQSQS
>Liparis tanakae [A0A4Z2I8E1]
MATSAWLRETSAMRLTEGLVSVLKEQRPEYLPALLAGYREHGVFQTQSASAVGGLVGLSNAKLGSGKTRSEGLCLLSMMV
KDSSSELFQQHCNSWLRSLQHVIQSQSPEETIKLAVSTLKDLLQYSSQLAQLAREVGLNSILGLLTSLLALKKECELSAM
DGIMACMTFYPRACGSLRMACLCYSRLPCLGGLLDRGVGAGRAEGWTNQIHCLLASADGLLAQIYQNGAVLYEGPGVELA
FPHLDQSDPLLLLQLQDRYTAVCLALKHTLGVDPASAVRLPVRIILNLVCRVLAVSSKSIPEASLGQQRAYSSVRVSVYK
TLELWVQVAGACGGILQGSPGHSELLFTHLLGDITPGAESVKLRAGLSADAVPGGKPGPRRTKHLVMADTAGPSLQRKGD
QLANQDSCLWALRALRQILLTSGTLLKDDMHKRLHDVVLPLCVRLKQQQSSSSILSDAAGCVSGQYSSALPRRELYRLLL
ALVLVPSPCWPPPLTCAVSILSNGRNDRNLKVSTFCSEALTVCNSLLHPRTPSIALPLPPLTLKPVPAASVLPSSQAAGL
ALPTLLGGPAPGPPFPGRHSLCMGPASLLGSLENHLSLAPGLPPAPGDMILSHQQDAAGLGLPEGQRPVFVRYDREEAED
VEISLASDSDDSVVIVPPGMLSLENQQEEAAAAAAAAAGGAQNMSSAPPGGAAAGGETAAIVPAAATATTVDRASLPNDL
AVSSPLLATSTTQINSFPPPSSSVVSLDEEGMAGFGAEGEAEGGIEEIQANRAPFAEDRMKVQKVESIGVLEEAREAEAD
ESERMDDPTMPQILCVTGGTLEEREEPEEEGGAAGEAPQDGAAPWEPAANEIEPTDAAEGGAANRVQEESAAEPPKEAGV
INDKPSPARQEEQLAVQEGDAAVAAGEPETSRGRSPKETEEMAEEAEKRESVTAEPRETEGGAGGESDGEEGKGLKRKRE
EEQEEAGLGTEKKKVDDEVMASMLADFVACPPDDEDGASGSTRS
> Zootermopsis nevadensis [A0A067QXQ9]
MDGMLSLFETVSQYNCNDTILQKFLKTCTENKTFLNKAPVVIQPVVANINSKLNASATRYDGLLLLKTFLPQCSVQIFGE
NVISWMQQCIKSIERGQNNHALASISYQVLKLLLEMSEQMPEMKRVVSGFVVTKIIDNFQQIVPQEASLSMLECLEVLMC
NYGPSCGQQKNVLEKCVLQYVDSADVIVINSAARCVAFLPLLGGGGSQGANHVSRWKQQQQKVCTTLHCILDELFDNVRE
IPNCHSSLASCGTLSLPATSEIVPLLRIYQLMTRFINISKFLQAMLISEFPVEKAVLPDEIFGVVCRGLAVTSHTMEKKV
STDLLMVGAMMPQIHVALLKVLDSLIMCCGRNLLPYAPVICKLVLQTLKWTSAEKWAYGIEKPYGQLRVTAYNTLILWLQ
TSNCGSCVELISEELASVLMQDIWFEKEAVTLNVQGTYSKKKNKLRQHKPEQQQQQEQQKDVWNSIKYIPDQKANSKTCR
ATLQVLQWMLHSAAAFIKPSTHRLLQEATVELLFNIQRTSKVHDIPIPYGESSCRLELYRLLHTLVLEPHATWPPPTQFA
VHMLSAGRSDPDLEVSSFCTAALTALEKLIHPASGTLNFPVSLEEMLESTKRYKQEYPTKAKDTSEEEEVNNYVHKRGGS
GGSKQTDAYRASNSSEEVVLVCENRMQKDDEEISECEVVSLYSEESDSSVKNKATESHGLSEVVSKTHNKQEVSLVDIFD
SDDEYQHKEVQSASFIDISVENGSCSNVGNSKNGLSQNCKSKVDNKIQSATPIRINKESPHIKSKNEIGTSGDLSNEDKL
ISNQQKGTTESYADASNRCENIICALTEKGSKEISGNTKICTAKRTVLVTENEEERENNDFVLHVQEDDHEPNPKKIKLD
SPGSSIKYYKPNTETTKTNSHSNVKNSTALPIAGNEEQVVNGVAVNPKDVTEEEMLQTFVDVLSD
> Eschrichtius robustus [A0A2F0BH19]
MAAEAEDEAQMIKLSPYTDWILRRSTVNGQSQDPPPTMELAVAVLRDLLRYAAQLPTLFRDISMNHLPGLLTSLLGLRPE
SELSALEGMKACMTYFPRACGSLKGKLASFFLSRVDALSPQLQQLACECYSRLPSLGAGFSQGLKHTDSWEHELHSLLAS
LHSLLGALYEGVETAPVQYEGPGVETLLSPSEDGDAHVLLRLWQRFSGLARCLGLMLSSEFGAPVSVPVQEILDLICRTL
SVSAKNISLLGDGPLRLLLLPSLHLEALDLLSALVLACGGRLLRFGALISRLLPQVLNAWSIGRDSLSPGQERPYSTMRT
KVYAVLELWVQVCGASAGVLQGGASGEALLTHLLSDISPPADALKLRSPRGSPDGGLQTGKPSAPKKLKLDMGEAMAPPS

HRKGDSNANSDVCAAALRGLTRTILMCGPLIKEETHRRLHDLVLPLVMGVQQGEVLGNSPYTSSRCRRELYRLLLALLLA
PSPRCPPPLACALQAFSLGQREDSLEKHW
> Scleropages formosus [A0A0P7VLV3]
MATAALLHGSTNMRLTEGLVSALKLERPEYLPSLLANYREHGGVSAQVLVEALGDCAGQSFCILFKKLYCLPCCRFEGLC
LLAVLVRDSSSEVFQQHCLTWLRLLQQVIQSQAPLPSIQLAIVVLQDLLQYSSQLPELAREVGLNSILGILTSLLGLKLE
CHLVAMEGMKACMTFYPRACGSLKDKLGAYFLSKMDSDNPRVQEVACECFGRLPCLGGVLERGGGSRRAEGWASQVHCLL
ATAHSLLGQMYQGAESEGAVQYEGPGMELPLPPLEEADPLLMLQLPQRYRAVCLALTQTLSMDPNSPVSLPVQSVLNLVC
RALAVSCKNINTSGDGCFKLLFLPSVHSDTLEVLSTLITVAGSRLLQYSNVLSRLFSQTLSAWSPPPEGSPGQQRAFSAV
KVCLYRTLELWVRVAGASAGILHGSPTHNELLLTHLLGDITPGPDSVKLRAGQPAVSELVGHGGKAAAAAARRAKGMGDV
TGVSIQRKGDALANQDTCVSALRALRQIILSCGTLLKEDIHKRLQDLVLPLCVRLQQQPCGSDVGTGSSQYGSAPPRREL
YRLLLALVLTPPPRWPPPLPCAVSIFSHGRRDHSLTVASFCTEALAICNSLLHPRVPSIALPLPPLALKHTPAAPNLTPS
QNPSLSLPTLLGGPTQASPFPARHPLGLGPPSLLAPMENHLPLPTPVLPPQAGSTPAPGELLSPPQPGELAALGPNEGRR
PLFVRYEKEEAEDVEISLESDSDDSVVIVPPDMLMQETQEPAGTQPIHPPPGGAVPGLTVSAASETGAVNSPIPNELPTT
MALPSNSNAVTTFPAQSQTQLVSLVPPLSSGATQLAAPSVSLGDSLPGTQLQQMLLQSSPAGQASQLGLPVQIQLQTQLA
QPSRQPQPQQMTNEEDLTVININSSDEEEDEEEEMDDDEIGEEEEEEEDEPLDDEEEEEEGSEFAEEYYDGEEFGDYEEE
EEEEEMIEGEDEEGMIEGEEEEEEEAEELPALETGEGRRLLLSVEEEAVGSGGEGTMEIFCGEEGGESQGTGRETEEQPRS
YGQDSVQEDVKQADERAAGVLEEQGDKLVDVERESEALQDGKTDSQPHGAAGDSSQEADITQGEGTVVDGPEVTSKEQEA
AQEEDAPTVDETAASLQEPMTESPEDVGKVDEKAGERPSVDQPPVELREESKEQLKQGQQMLAGQGGTVGLGEEREDDAA
EENVDDLRSMKRKREINEEGEGEGEVRPSVEKKKLDEEAMASMLADFVDCPPDEEENARSPAQSEG
> Camelus ferus [S9X7Z9]
MLSRETENLSAVGALVGLSNARLGSIKTRFEGLCLLSLLVGESPTEMFQQHCVSWLRSVQQVLQDPPPTMELAVAILRDL
LRYAAQLPTLFRDISMNHLPGLLTSLLGLRPECELSALEGMKACMTYFPRACGSLKGKLASFFLSRVDALSPQLQQLACE
CYSRLPSLGAGFSQGLKHTESWEQELHSLLASLHSLLGALYEGAETAPMQYEGPGVETLLSPSEDGDAHVLLQLRQRFSG
LARCLGLMLSSEFGAPVSVPVQEVLDVICRTLSISAKNISLLGDGPLRLLLLPSLHLEALDLLSALILACGGRLLRFGAL
ISRLLPQVLNAWSIGRDALSPGQERPYSTVRTKVYAVLELWVQVCGASAGVLQGGASGEALLTHLLSDIAPAADALKLRS
PRGSPDGGLQTGKPSAPKKLKLDVGEAMAPPSHRKGDSNANSDVCAAALRGLSRTVLMCGPLIKEETHRRLHDLVLPLAM
GVQQGEVLGSSPYTSARCRLELYRLLLALLLAPSPRCPPPLACALQAFSLGQREDSLEVSSFCSEALVTCAALTHPRVPP
LQSMGPACPTPAPVPPPEAPSPFRAPAFHPPGPMPSVGPMPSASPMPSAGPMPSAGPVSSAGPMPSVGSMPPARPGPPAT
ANHLGVSVPGLVSVPPRLLPGSENHRASSNEDPILAPSGTPPPTVPPDETFGGRVPRPAFVHYDKEEASDVEISLESDSD
DSVVIVPEGLPPLPPPPPSGTTPPPVAPAGPPTASPPVPAKEESEELPATPGPLPPPPPPXXXXXXXXXPEGTPGGGGAP
ALEEDLTVPPEPEPEPGLLLEVEEPGAEEGPGAETAPTLAPEELGEEEPSAPPTLLEEGAEGVGDQVPPQPEASAAEEME
METEAAALQEKEQDDTAAMLADFIDCPPDDENPPPPPPAPEPDS
> Scophthalmus maximus [A0A2U9AVV8]
MLVKDSSSDLFEQHCLSWLRSLQQVIQSQAPVQTIQLAVNILKDLLQYSSQLAELAREVGLNSILGILTSLLGLKTECEL
SAMEGMMACMSYYPRACGSLRDKLGAYFLSKMDSTNKKTQEMACQCYGRLPCLGGLLDRGVGAGRAEGWTNQIHCLLATA
NGLLAQIYQGSETDGTVQYEGPGLELSFSHLDQSDPLLLLQLQHRYTAVCLALKHTLRVDPASAVRLPVRPVLNLVCRAL
AVSSKSINLTGDGSVRMLVLPIIHTNTLEVLSALITAVRSGMVQYTVVLQRLFSQTLSAWSPLQEAGMGQQRAYSTVRVS
VYRTLELWVQVAGASASILQGSPGHSELLFSQLLSDITPGADSVKLRAGLSADVVPGGKPGPRRTKPLVIADAGGTSLQR
KGDLLANQDTCLSALRALRQIILTSGTLLKDDIHKRLHAVVLPLCVRLQQQQSSSSSICESAGGVGGQYSSALSRRELYR
LVLALVLVPSPCWPPPLTCAVSVLSKGRADRNLKVSTFCAEALTMCNSLLHPRRPSIALPMPPLTLKPTPTAPVLPSSQG
PTPGLTLPTLLGGTTPGTPFPTRHSLSLGPTSLLGSLENHLSLGPGLSGQAPTPGDMILSPHTHHLPDQAGLGPPEGQRP
VFVRYEKEEVEDVEISLASDSDDSVVIVPPGMLKMENQHDDTAHQQQLMASRFPTTSPHPPLSSPLPPPPLTPSPLPTPA
PADADAAIHPRPARPHGPTTANAPAAEPADNEDSAVININSTDEEEEEEDMEDDEELEEEEEGMEEEDEDEEVSDFAEEE
FYDGEEYEDYDEEELEEEEEEEEEDGDIPPLEGAEDKAEEVGLEEGKVLQAAVDEGGIAGFSMEGDEEGGIEEIQTNRMLF
SEDRMKESRAELAQEASASDDQPCGHREEQPAAREGDSAAAEPETSAALNTREKEETDEKRDMTETEQQETEGGGGGESD
GEEGKGVKRKRDEVHREEEAGQGSEKKKLDDEAMASMLADFVACPPDDEDGASGSNRS
> Nomascus leucogenys [A0A2I3HDJ9]
MLLFSLSPRVQPAFTQCAGPQPPPHAPSPVGGRRGVFASSRHAITGARGKMAAAVLSGPSAGSAAGVPGGTGGLSAVSSG
PRLRLLLLESVSGLLQPRTGSAVAPVHPPNRSAPHLPGLMCLLRLHGSVGGAQNLSALGALVSLSNARLSSIKTRFEGLC
LLSLLVGESPTELFQQHCVAWLRSIQQVLQTQDPPATMELAVAVLRDLLRYAAQLPALFRDISMNHLPGLLTSLLGLRPE
CEQSALEGMKACMTYFPRACGSLKVSIYALFSPPVFLSFLPQLACECYSRLPSLGAGFSQGLKHTESWEQELHSLLASLH
TLLGALYEGAETAPVQNEGPGVQMLLSSEDGDAHVLLQLRQRFSGLARCLGLMLSSEFGAPVSVPVQEILDFICRTLSVS
SKNISLHGDGPLRLLLLPSIHLEALDLLSALILACGSRLLRFGILISRLLPQVLNSWSIGRDSLSPGQERPYSTVRTKVY
AILELWVQVCGASAGMLQGGASGEALLTHLLSDISPPADALKLRSPRGSPDGSLQTGKPSAPKKLKLDVGEAMAPPSHRK
GDSNANSDVCAAALRGLSRTILMCGPLIKEETHRGPLLCTHWGRLHDLVLPLVMGVQQGEVLGSSPYTSSRCRRELYCLL
LALLLAPSPRCPPPLACALQAFSLGQREDSLEVSSFCSEALVTCAALTHPRVPALQPMGPTCPTPAPVPPPEAPSPFRAP
PFHPPGPMPSVGSMPSAGPMPSAGPMPSAGPVPSARPGPPTTANHLGLSVPGLVSVPPRLLPGPENHRAGSNEDPILAPR
GTPPPTIPPDETFGGRVPRPAFVHYDKEEASDVEISLESDSDDSVVIVPEGLPPLPPPPPSGATPPPIAPTGPPTASPPV
PAKEEPEELPAAPGPLPPPPPPPPPVPGPVTLPPPQLVPEGTPGGGGPSALEEDLTVININSSDEEEEEEEEEEEEEEEE
EEEEEDFEEEEEDEEEYFEEEEEEEEEFEEEFEEEEGELEEEEEEDEEEEEEELEEVEELEFGTAGGEVEEGGPPPPTLPP
ALPPPESPPKVQPEPEPEPGLLLEVEEPGTEEEHGADTAPTLAPEVLPSQGEVEREGGSPAAGPPPQELVEEEPSAPPTL
LEEETEDGSDKVQPPPETPAEEEMETETEAEALQEKEQDDTAAMLADFIDCPPDDEKPPPPTEPDS
> Marmota monax [A0A5E4CZX9]
MAAAVLSGPSAGSPAGVPGGAGGLSAMNSGPRLRLLLLESVSGLLQPRTGSTVAPVHPPVCSVPHLPGLMCLLRLHGTVG
GAQNLSALGALVSLSNARLGSIKTRFEGLCLLSLLVGESPTEMFQQHCVSWLRSIQQVLQSQDPPPTMELAVAVLRDLLR
YAAQLPTLFRDISINHLPGLLTSLLGLRPECEQSALEGMKACMTYFPRACGSLKGKLASFFLSRVDALSPQLQQLACECY
SRLPSLGAGFSQGLKHTESWEQELHSLLASLHSLLGALYEGAETAPVQNEGPGVETLLSPSEDGDAHVLLRLRQRFSGLA
RCLGLMLSSEFGAPVSVPVQEVLDLICRTLSVSGKNISLLGDGPLRLLLLPSIHLEALDLLSALILACGGRLLRFGALIS
RLLPQVLNAWSIGRDALSPGQERPYSTIRTKVYAILELWVQVCGASAGVLQGGASGEALLTHLLSDISPPSDALKLRSPR
GSPDGGLQTGKPSAPKKIKLDVGEAMAPPSHRKGENNANSDVCAAALRGLSRTILMCGPLIKEETHRRLHDLILPLVMGV
QQGEVLGSSPYTSSCCRRELYRLLLALLLAPSPRCPPPLACALQAFSLGQREDSLEVSSFCSEALVTCAALTHPRVPPLQ
AMGPTCPTPAPVPPPEAPSPFRAPSFQPPGPMPSVGPMPSAGPLTSSGSMPSVGPMPSAGPMPSAGPIPSAGPVPSARPG
PPATANHLGLSVPGLVSVPPRLLPGPENHRAGSNEDPVLAPSGTPPPTIPPDETFGGRVPRPAFVHYDKEEASDVEISLE
SDSDDSVVIVPEGLPPLPPPPPSGTTPPPVAPTGPPTASPPVPAKEEPEELPAAPGPLPPPPPPPPPVPGPVTLPPPQLV
PEGTPGGGGPPALEEDLTVININSSDEEEEEEEEEEEEEEEEEEEEEEEFEEEEEEEEEYFEEEEEEEEEFEEEFEEEEG
ELEEEEEEEEEEEEEEELEEVEDLEFGSAGGEVEEGGPPPPTLPPALPPPESPKEQPEPEPEPGLLLEVEEPGSEEQHGTE

TAPTLVPEVLPSQGEEEREGGSPEAGPPPQELVEEEPSAPPTLLEEGTEGGGDNVPPPPETAAEEEMETESEAAVLQEKE
QDDTAAMLADFIDCPPDDEKPPPTTEPDS
>Loxodonta africana [G3UML0]
MAAAVLSGPSAGSAAAVPGGTGGLSAMSSGPRLRLLLLESVSGLLQPRTGSTVPSVHPPVRPGPHLPGLMCLLQLHGTVG
GAQNLSALGALVGLSNARLGSIKTRFEGLCLLSLLVGESPTEMFQQHCVSWLRSIQQVLQSQDPAPTMELAVAILKDLLR
YAAQLPTLFRDISMNHLPGLLTSLLGLRPECELSALEGMKACMIYFPRACGSLKGKLASFFLSRVEALSPHLQQLACECY
SRLPSLGAGFSQGLKHTESWEQELHCLLASLHNLLGTLYEGAETAPVQYEGPGVDMLLSPSEDGDAHGLLRLRQRFSGLA
RCLGLMLSSEFGAPVSVPVQEVLDLICRTLSVSGKNISLLGDGPLRLLLLPSVHLEALDLLSALILACGSRLLRFGGLIS
RLLPQVLGAWSMGRDTLSPGQERPYSTVRAKVYAVLELWVKVCGASAGVLQGGASAEALLTHLLSDISPPADGLKLRSPR
GSPDGSLQTGKPSAPKKLKLDVGEAVAPPTHRKGDSNANSDVCAAALRGLSRTILMCGPLIKEETHKRLHDLVLPLVMGV
QQGEVLGSSPYTSSRCRRELYHLLLALLLAPSPRCPPPLSCALQAFSLGQREDSLEVSSFCSEALVTCAALTHPRVPPLQ
STAPPCPTPAPVPPIEAPSPFRAPPFHPPGTMPSVGPLPSAGPLPSAGPMPTAGPMPSARPGPPATANHLGLSVPGLVSV
PPRLLPGPENHRAGSNEDPVLATSGSPPPPLPADETFGGRVPRPAFVHYDKEEASDVEISLESDSDDSVVIVPEGLPPLP
PPPTSGSTPPPVAPAGPPTASPPVPAKEEPEELPAAPGPLPPPPPLPVPGPVTLPPPQLVPEGTPGGGGPPALEEDLTVI
NINSSDEEEEEEEEDEEEEEEEEEEEEEDFEEEEEDEEEYFEEEEEEEEEFEEEFEEEEGELEEEEEEDEEEEEELEEVE
ELEFGSAGGEAEEGGPPPPTLPPAPPTSESPKAPPEPEPEPGLLLEVEEPGAEEEHGAETAPTLAPEVLPAQGEVERKGG
SPPAGPSPQELVEEEPPAPPTLMEEGTENGGDKVPTPPETSAEEEMETETEAAALEEKEQDDTAAMLADFIDCPPDDEKP
PPATEPDS
>Pig USMARC [A0A4X1UV05]
MAAAVLSGPSAGSAAGVPGGTGGLSAVVSVPRLRLLLLDSVSGLLQPRAGSTVAPVHPPAPSAPHLPGLMCLLRLHGTVG
GAPNLSAVGALVGLSNARLGSIKTRFEGLCLLSLLVGESPTEMFQQHCVSWLRSIQQVLQSQDPPPTMELAVAVLKDLLR
YAAQLPTLFRDISMNHLPGLLTSLLGLRPECELSALEGMKACMTYFPRACGSLKGKLASFFLSRVDALSPQLQQLACECY
SRLPSLGAGFSQGLKHTESWEQELHSLLASLHGLLGALYEGAETAPMQYEGPAVEALLSPSEDGDAHVLLRLRQRFSGLA
RCLGLLLSSEFGAPVSVPVQEVLDVICRTLSVSARNISLLGDGPLRLLLLPSLHLEALDLLSAILACGGRLLRFGALISR
LLPQVLSAWSIGRDTLSPGQERPYSTMRTKVYAVLELWVQVCGASAGVLQGGASGEALLTHLLSDISPPADALKLRSPRG
SPDGGLQTGKPSAPKKLKLDMGEPIAPPSHRKGDSNANSDVCAAALRGLSRTILMCGPLIKEETHRRLHELVLPLVMGVQ
QGEVLGSSPYTSSRCRRELYRLLLALLLAPSPRCPPPLACALQAFSLGQREDSLEVSSFCSEALVTCAALTHPRVPTLQS
MVPTCPTPAAVPPPEAPSPFRAPAFHPPGPMPSAGPMPRGPMPSAGPMPSVGPMPPARPGPPATANHLGLSGSSLVSVPP
RLLPGPENHRAGSNEEPVLAPSGTPPPTLPSDETFGGRVPRPAFVHYDKEEASDVEISLESDSDDSVVIVPEGLPPLPPP
PPSGTTPPPVAPAGPPAASPPVPAKEEPEELPAAPGPLPPPPPTPVPGPATLPPPQLVPEGTPGGGGAPALEEDLTVINI
NSSDEEEEEEEEEEEEEEEEEEEEDFEEEEEDEEEYFEEEEEEEEEFEEEFEEEEGELEEEEEDEEEEEEEELEEVEELE
FGSAGAEVEEGGPPPPSLPPALPPAESPKVQPEPEPEPGLLLEVEEPGAEEAPGPETAPTLVPEALPAQGEAEREAGSPP
TAPPPQELVEEEPSVPPTLLEEGAEGGGDKVPPPPETSAAEEMETETESTALQEKEQDDTAAMLADFIDCPPDDEKPPPA
AEPDS
> Diceros bicornis minor [A0A7J7E832]
MAAAVLSGPSAGSAAGVAGGTGGLSAVGSGPRLRLLLLESVSGLLQPRPGSTVAPVHPQVRSAAHLPGLLCLLRLHGTVG
GAQNLSAVGALVGLSNARLGSMKTRFEGLCLLSLLVGESPTELFQQHCVSWLRSIQQVLQSQDPPPTMELAVAVLRDLLR
YAAQLPTLFRDISMNHLPGLLTSLLGLRPECELSALEGMKACMTYFPRACGSLKGKLASFFLSRVDALSPQLQQLACECY
SRLPSLGAGFSQGLKHTESWEQELHSLLASLHSLLGALYEGAEAAPMQYEGPGVEVLLSPSEDGDAHALLRLRQRFSGLA
RCLGLMLSSEFGAPVSVPVQEILDVICRTLSISAKNISLLGDGPLRLLLLPSIHLEALDLLSALILACGGRLLRFGALIS
RLLPQVLNTWSIGRDTLSPGQERPYSTLRTKVYAVLELWVQVCGASAGMLQGGASGEALLTHLLSDISPPADALKLRSPR
GSPDGGLQSGKPSAPKKLKLDVGEAMAPPSHRKGESNANSDVCAAALRGLSRTILMCGPLIKEETHRRLHDLVLPLVMGV
QQGEVLGSSPYTSSRCRHELYRLLLALLLAPSPRCPPPLACALQAFSLGQREDSLEVSSFCSEALVTCAALTHPRVPPLQ
SVGPTCSTPAPVPPPEAPSPFRAPPFHPPGPMPSAGPMPSAGPMPSAGPIPSVGPLPSVGSMPSAGPMPPARPGPPATAN
HLGLSVPGLVSVPPRLLPGPENHRAGSNEDPVLAPSGTPPPTIPPDETFGGRMPRPAFVHYDKEEASDVEISLESDSDDS
VVIVPEGLPSLPPPPTSGTTPPPVAPAGPPTASPPVPAKEEPEELPAAPGPLPPPPPPPVPGPVTLPPPQLVPEGTAGGG
GPPALEEDLTVININSSDEEEEEEEEEEEEEEEEEEEEEDFEEEEEDEEEYFEEEEEEEEEFEEEFEEEEGELEEEEEEE
EEEEEEELEEVEELEFGSAGGEVEEGGPPPPSLPPALPPAESPKVQPEPEPEPGLLLEVEEPGAEEDHGAETAPTLAPEV
LPAQGEVAREEGSPPAGAPPQELVEEEPSAPPTLLEEGTEGGDEKVPTPPETSAAEEMETETETEGAALQEKEQDDTAAM
LADFIDCPPDDEKPPPLTEPDS
> Pig USMARC [A0A4X1UX07]
MAAAVLSGPSAGSAAGVPGGTGGLSAVVSVPRLRLLLLDSVSGLLQPRAGSTVAPVHPPAPSAPHLPGLMCLLRLHGTVG
GAPNLSAVGALVGLSNARLGSIKTRFEGLCLLSLLVGESPTEMFQQHCVSWLRSIQQVLQSQDPPPTMELAVAVLKDLLR
YAAQLPTLFRDISMNHLPGLLTSLLGLRPECELSALEGMKACMTYFPRACGSLKGKLASFFLSRVDALSPQLQQLACECY
SRLPSLGAGFSQGLKHTESWEQELHSLLASLHGLLGALYEGAETAPMQYEGPAVEALLSPSEDGDAHVLLRLRQRFSGLA
RCLGLLLSSEFGAPVSVPVQEVLDVICRTLSVSARNISLLGDGPLRLLLLPSLHLEALDLLSALSSRCGGRLLRFGALIS
RLLPQVLSAWSIGRDTLSPGQERPYSTMRTKVYAVLELWVQVCGASAGVLQGGASGEALLTHLLSDISPPADALKLRSPR
GSPDGGLQTGKPSAPKKLKLDMGEPIAPPSHRKGDSNANSDVCAAALRGLSRTILMCGPLIKEETHRRLHELVLPLVMGV
QQGEVLGSSPYTSSRCRRELYRLLLALLLAPSPRCPPPLACALQAFSLGQREDSLEVSSFCSEALVTCAALTHPRVPTLQ
SMVPTCPTPAAVPPPEAPSPFRAPAFHPPGPMPSAGPMPAAPCPRRAPSTANHLGLSGSSLVSVPPRLLPGPENHRAGSN
EEPVLAPSGTPPPTLPSDETFGGRVPRPAFVHYDKEEASDVEISLESDSDDSVVIVPEGLPPLPPPPPSGTTPPPVAPAG
PPAASPPVPAKEEPEELPAAPGPLPPPPPTPVPGPATLPPPQLVPEGTPGGGGAPALEEDLTVININSSDEEEEEEEEEEE
EEEEEEEEEEDFEEEEEDEEEYFEEEEEEEEEFEEEFEEEEGELEEEEEDEEEEEEEELEEFGSAGAEVEEGGPPPPSLP
PALPPAESPKVQPEPEPEPGLLLEVEEPGAEEAPGPETAPTLVPEALPAQGEAEREAGSPPTAPPPQELVEEEPSVPPTL
LEEGAEGGGDKVPPPPETSAAEEMETETESTALQEKEQDDTAAMLADFIDCPPDDEKPPPAAEPDS
>Bos indicus x Bos taurus [A0A4W2DZX4]
MSGWKVSRSSVALKVSFWILAPLVLTTCSLSPTARPEPVLYPAHLPGLESWHFHAAHAHSQQARGVQCTGPGPPPHDPSP
RGIRRGVFASSRHATTRAQLKMAAAVLSGPSAGSAAAVPGGPGGLSAVGSGPRLRLMLLESVSGLLQPRTGSAVAPVHPP
ARSAPHLPGLMCLLRLHGTVGGAQNLSAVGALVGLSNARLGSIKTRFEGLCLLSLLVGESPTEMFQQHCVSWLRSIQQIL
QSQDPPPTMELAVTVLKDLLRYAAQLPAVFRDISMNHLPGLLTSLLGLRPECELSALEGMKACMTHFPRACGSLKGKLAS
FFLSRVDALSPQLQQLACECYSRLPSLGAGFSQGLKHTDSWEQELRSLLASLHSLLGGLYEGAEAAPMQYESPGAETLLS
SSEDADAHTLLRLRQRFSGLARCLGLMLSSEFGAPVSVPVQDILDLICRTLSVSAKNVSLLGDGPLRLLLLPSLHLEALD
LLSALILACGARLLRFGALISRLLPQVLNAWSIGRENLGPGQERPYSTVRTKVYAVLELWVQVCGASAGVLQGGASGEAL
LSHLLSDISPPADALRLRSPRGSPDAGLQTGKPSAPKKLKLDVGEAIAPPSHRKGDSNANSDVCAAALRGLSRTILMCGP
LIKEETHRRLHELVLPLVMGVQQGEALGSSPYTSSHCRRELYHLLLALLLAPSPRCPPPLACALRAFSLGQREDSLEVSS
FCSEALVTCAALTHPRVPPLQSVGPTCPAPAPVPPPEAPAPFRAPAFHAPSPLPSAGPMPSAGPMPPVGPLPPTRPGPPA
TANHLGLSVPGLVSVPPRLLPGPENHRAGSSEDPVLAPSGSPPPTIPPDETFGGRVPRPAFVHYDKEEPSDVEISLESDS

DDSVVIVPEGLPPPPPPSSGTTPPPVAPAGPPAASPPVPAKDEPEELPAAPGPLPPPPPPPVPGPVTLPPPQLVPEGTPG
GGGPPALEEDMTVININSSDEEEEEEEEEEEEDEEEDEEEDFEEDEEEEEEYFEEEEEEEEEFEEEFEEEEGELEDEEDE
EEDEELEELEEVEFGPAGGEVEGGGPAPPSLPPALPPAESPKGPPEPGLEPGLLLEVEEPGTEEAPGPETAPMLAPEVLP
SQGEVEREGGSPPAGPPPQELVEEEPSGPPALLEEGAEGGGDKVSPPPEASAVEETEVEAAALPPEKEQGDTAAMLADFI
DCPPDDEKPPPASEPDS
> Muntiacus reevesi [A0A5N3X1R4]
MAAAVLSGPSAGSAAGVPGGTGGLSAVGSGPRLRLMLLESVSGLLQPRTGSAVAPVHPPARSAPHLPGLMCLLRLHGTVG
GAQNLSAVGALVGLSNARLGSIKTRFEGLCLLSLLVGESPTEMFQQHCVSWLRSIQQILQSQDPPPTMELAVTVLKDLLR
YAAQLPAVFRDISMNHLPGLLTSLLGLRPECELSALEGMKACMTHFPRACGSLKGKLASFFLSRVDALSPQLQQLACECY
SRLPSLGAGFSQGLKHTDSWEQELRSLLASLHSLLGGLYEGAEAAPMQYESPGMETLLSPSEDADAHTLLRLRQRFSGLA
RCLGLMLSSEFGAPVTVPVQDILDLICRTLSVSAKNVSLLGDGPLRLLLLPSLHLEALDLLSALILACGARLLRFGALIS
RLLPQVLNAWSIGRENLGPGQERPYSTVRTKVYAVLELWVQVCGASAGVLQGGASGEALLSHLLSDISPPADTLRLRSPR
GSPDGGLQTGKPSAPKKLKLDVGEAMAPPSHRKGDSNANSDVCAAALRGLSRTILMCGPLIKEETHRRLHELVLPLVMGV
QQGEALGSSPYTSSHCRRELYRLLLALLLAPSPRCPPPLACALRAFSLGQREDSLEVSSFCSEALVTCAALTHPRVPPLQ
SVGPTCPAPAPAPPPEAPAPFRAPAFHTPGPLPSAGTMPSAGPMPPAGPLPPARPGPPATANHLGLSVPGLVSVPPRLLP
GPENHRAGSSEDPVLAPSGSPPPTVPPDETFGGRVPRPAFVHYDKEEASDVEISLESDSDDSVVIVPEGLPPPPPPPSGT
TPPPVAPAGPPAASPPVPAKDEPEELPAAPGPLPPPPPPPVPGPVTLPPPQLVPEGTPSGGGPPALEEDLTVININSSDE
EEEEEEEEEEDEEEDEEEDFEEDEEEEEEYFEEEEEEEEEFEEEFEEEEGELEDEEDEEEDEELEELEEVEFGPAGGEV
EGGGPVPPSLPPPALPPAESPKGPPEPGLEPGLLLEVEEPGTEEAPGPETAPTLAPEVLPSQGEMEREAGSPPAGSPLQE
LVEEEPSGPPTLLEEGAEGGAEKVSPPPEASVEAEVEAAALPPEKEQGDTAAMLADFIDCPPDDEKPPPASEPDS
> Sousa chinensis [A0A484GWL5
MAAAVLSGPSAGSAAGVPGGTGGLSAVNSGPRLRLLLLESVSGLLQPRAGSTVSPVHPPTRSVPHLPGLMCLLRLHGTVG
GAQNLSAVGALVGLSNARLGSIKTRFEGLCLLSLLVGESPTEMFQQHCVSWLRSIQQVLQSQDPPPTMELAVAVLGDLLR
YAAQLPTLFRDISMNHLPGLLTSLLGLRPESELSALEGMKACMTYFPRACGSLKGKLASFFLSRVDALSPQLQQLACECY
SRLPSLGAGFSQGLKHTDSWEQELHSLLTSLHSLLGALYAGADTAPMQYEGPGVETLLSPSEDGDAHVLLRLWQRFSGLA
RCLGLMLSSEFGAPVSVPVQEILDLICRTLSVSAKNISLLGDGPLRLLLLPSLHLEALDLLSALILACRGRLLRFGALIS
RLLPQVLNAWSIGRDSLSPGQERPYSTMRTKVYTVLELWVQVCGASAGVLQGGASGEALLTHLLSDISPPADALKLRSPR
GSPDGGLQTGKPSAPKKLKLDMGEALAPPSHRKGDSNANSDVCAAALRGLSRTVLMCGPLIKEETHRRLHDLVLPLVMGV
QQGEVLGSSPYTSSRCRRELYRLLLALLLAPSPRCPPPLACALQAFSLGQREDSLEVSAFCSEALVTCAALTHPRVPPLQ
TMGPACPTPAPVPPPEAPSPFRAPAFHPPGPMPSVGPMPSVGPMPSAGPMPSPGPVPPAGPMPSVGPMPPARPGPPATAN
HLGLSVPGLVSVSPRLLPGPENHRAGSNEDAVLAPSGTPPPTIPPDETFGGRVPRPAFVHYDKEEASDVEISLESDSDDS
VVIVPEGLPPLPPPPPSGTTPPPAAPVGPPTASPPVPAKEEPEELPAAPGPLPPPPPPPVPGPVALPPPQLVPEGTPSGG
GPPALEEDLTVININSSEEEEDDDEEDEDEEDFEEEEEEEEEEEYFEEEEEEEEEFEEEFEEEEGELEEEEEDEDEEEEV
EEVEFGPAGGEVEEGGPAPPSLPPALPPAASPKVQPQPEPEPGLLLEVEEPGAEEGPGAETAPTLAPEVLPSQGEVEREG
GSPPAVPPPQELVEEEPSVPPTLLEEGAEGGGDKVPTPPEASAAEEMETEAEAAALQEKEQDDTAAMLADFIDCPPDDEK
PPPATEPDS
> Muntiacus muntjak [A0A5N3WRN7]
MAAAVLSGPSAGSAAGVPGGTGGLSAVGSGPRLRLMLLESVSGLLQPRTGSAVAPVHPPARSAPHLPGLMCLLRLHGTVG
GAQNLSAVGALVGLSNARLGSIKTRFEGLCLLSLLVGESPTEMFQQHCVSWLRSIQQILQSQDPPPTMELAVTVLKDLLR
YAAQLPAVFRDISMNHLPGLLTSLLGLRPECELSALEGMKACMTHFPRACGSLKGKLASFFLSRVDALSPQLQQLACECY
SRLPSLGAGFSQGLKHTDSWEQELRSLLASLHSLLGGLYEGAEAAPMQYESPGMETLLSPSEDADAHTLLRLRQRFSGLA
RCLGLMLSSEFGAPVTVPVQDILDLICRTLSVSAKNVSLLGDGPLRLLLLPSLHLEALDLLSALILACGARLLRFGALIS
RLLPQVLNAWSIGRENLGPGQERPYSTVRTKVYAVLELWVQVCGASAGVLQGGASGEALLSHLLSDISPPADALRLRSPR
GSPDGGLQTGKPSAPKKLKLDVGEAMAPPSHRKGDSNANSDVCAAALRGLSRTILMCGPLIKEETHRRLHELVLPLVMGV
QQGEALGSSPYTSSHCRRELYRLLLALLLAPSPRCPPPLACALRAFSLGQREDSLEVSSFCSEALVTCAALTHPRVPPLQ
SVGPTCPAPAPVPPPEAPAPFRAPAFHTPGPLPSAGTMPSAGPMPPAGPLPPARPGPPATANHLGLSVPGLVSVPPRLLP
GPENHRAGSSEDPVLAPSGSPPPTVPPDETFGGRVPRPAFVHYDKEEASDVEISLESDSDDSVVIVPEGLPPPPPPGTPP
PPVAPPGPPAASPPVPAKDEPEELPAAPGPLPPPPPPPVPGPVTLPPPQLVPEGTPSGGGPPALEEDLTVININSSDEEE
EEEEEEEEEEEEDEEEDFEEDEEEEEEYFEEEEEEEEEFEEEFEEEEGELEDEEDEEEDEELEELEEVEFGPAGGEVEG
GGPVPPSLPPPALPPAESPKGAPEPGLEPGLLLEVEEPGTEEAPGPETAPTLAPEVLPSQGEMEREAGSPPAGSPPQELV
EEEPSGPPTLLEEGAEGGAEKVSPPPEASAAAEAEVEAAALPPEKEQGDTAAMLADFIDCPPDDEKPPPASEPDS
> Camelus dromedarius [A0A5N4D5G4]
MAAAVLSGPSAGSAAGVPGGTGGLSAVASGPRLRLLLLESVSGLLQPRAGSTVAPVHPPVRSAPHLPGLMCLLRLHGTVG
GAQNLSAVGALVGLSNARLGSIKTRFEGLCLLSLLVGESPTEMFQQHCVSWLRSVQQVLQSQDPPPTMELAVAILRDLLR
YAAQLPTLFRDISMNHLPGLLTSLLGLRPEGKLASFFLSRVDALSPQLQQLACECYSRLPSLGAGFSQGLKHTESWEQEL
HSLLASLHSLLGALYEGAETAPMQYEGPGVETLLSPSEDGDAHVLLQLRQRFSGLARCLGLMLSSEFGAPVSVPVQEVLD
VICRTLSISAKNISLLGDGPLRLLLLPSLHLEALDLLSALILACGGRLLRFGALISRLLPQVLNAWSIGRDALSPGQERP
YSTVRTKVYAVLELWVQVCGASAGVLQGGASGEALLTHLLSDIAPAADALKLRSPRGSPDGGLQTGKPSAPKKLKLDVGE
AMAPPSHRKGDSNANSDVCAAALRGLSRTVLMCGPLIKEETHRRLHDLVLPLAMGVQQGEVLGSSPYTSARCRLELYRLL
LALLLAPSPRCPPPLACALQAFSLGQREDSLEVSSFCSEALVTCAALTHPRVPPLQSMGPACPTPAPVPPPEAPSPFRAP
AFHPPGPMPSVGPMPSASPMPSAGPMPSAGPVSSAGPMPSVGSMPPARPGPPATANHLGVSVPGLVSVPPRLLPGSENHR
ASSNEDPILAPSGTPPPTVPPDETFGGRVPRPAFVHYDKEEASDVEISLESDSDDSVVIVPEGLPPLPPPPPSGTTPPPV
APAGPPTASPPVPAKEESEELPATPGPLPPPPPPPVPGPVTLPPPQLVPEGTPGGGGAPALEEDLTVININSSDEEEEEE
EEEEEEEEEEEEEDFEEEEEDEEEYFEEEEEEEEEFEEEFEEEEGELEEEEEEEEDDEEEEEELEEVEELEFGPAGGEVEEG
GPPPPSLPPALPPAESPKVPPEPEPEPEPGLLLEVEEPGAEEGPGAETAPTLAPEVLPPQGEVQGEEGSPPAVPSPQELGEE
EPSAPPTLLEEGAEGVGDQVPPQPEASAAEEMEMETEAAALQEKEQDDTAAMLADFIDCPPDDENPPPAPEPDS
> Loxodonta africana [G3TBU8]
MAAAVLSGPSAGSAAAVPGGTGGLSAMSSGPRLRLLLLESVSGLLQPRTGSTVPSVHPPVRPGPHLPGLMCLLQLHGTVG
GAQNLSALGALVGLSNARLGSIKTRFEGLCLLSLLVGESPTEMFQQHCVSWLRSIQQVLQSQDPAPTMELAVAILKDLLR
YAAQLPTLFRDISMNHLPGLLTSLLGLRPECELSALEGMKACMIYFPRACGSLKGKLASFFLSRVEALSPHLQQLACECY
SRLPSLGAGFSQGLKHTESWEQELHCLLASLHNLLGTLYEGAETAPVQYEGPGVDMLLSPSEDGDAHGLLRLRQRFSGLA
RCLGLMLSSEFGAPVSVPVQEVLDLICRTLSVSGKNISLLGDGPLRLLLLPSVHLEALDLLSALILACGSRLLRFGGLIS
RLLPQVLGAWSMGRDTLSPGQERPYSTVRAKVYAVLELWVKVCGASAGVLQGGASAEALLTHLLSDISPPADGLKLRSPR
GSPDGSLQTGKPSAPKKLKLDVGEAVAPPTHRKGDSNANSDVCAAALRGLSRTILMCGPLIKEETHKRLHDLVLPLVMGV
QQGEVLGSSPYTSSRCRRELYHLLLALLLAPSPRCPPPLSCALQAFSLGQREDSLEVSSFCSEALVTCAALTHPRVPPLQ
STAPPCPTPAPVPPIEAPSPFRAPPFHPPGTMPSVGPLPSAGPLPSAGPMPTAGPMPSARPGPPATANHLGLSVPGLVSV
PPRLLPGPENHRAGSNEDPVLATSGSPPPPLPADETFGGRVPRPAFVHYDKEEASDVEISLESDSDDSVVIVPEGLPPLP

PPPTSGSTPPPVAPAGPPTASPPVPAKEEPEELPAAPGPLPPPPPLPVPGPVTLPPPQLVPEGTPGGGGPPALEEDLTVI
NINSSDEEELEEVEELEFGSAGGEAEEGGPPPPTLPPAPPTSESPKAPPEPEPEPGLLLEVEEPGAEEEHGAETAPTLAP
EVLPAQGEVERKGGSPPAGPSPQELVEEEPPAPPTLMEEGTENGGDKVPTPPETSAEEEMETETEAAALEEKEQDDTAAM
LADFIDCPPDDEKPPPATEPDS
> Bos indicus x Bos Taurus [A0A4W2EHP8]
MSGWKVSRSSVALKVSFWILAPLVLTTCSLSPTARPEPVLYPAHLPGLESWHFHAAHAHSQQARGVQCTGPGPPPHDPSP
RGIRRGVFASSRHATTRAQLKMAAAVLSGPSAGSAAAVPGGPGGLSAVGSGPRLRLMLLESVSGLLQPRTGSAVAPVHPP
ARSAPHLPGLMCLLRLHGTVGGAQSQDPPPTMELAVTVLKDLLRYAAQLPAVFRDISMNHLPGLLTSLLGLRPECELSAL
EGMKACMTHFPRACGSLKGKLASFFLSRVDALSPQLQQLACECYSRLPSLGAGFSQGLKHTDSWEQELRSLLASLHSLLG
GLYEGAEAAPMQYESPGAETLLSSSEDADAHTLLRLRQRFSGLARCLGLMLSSEFGAPVSVPVQDILDLICRTLSVSAKN
VSLLGDGPLRLLLLPSLHLEALDLLSALILACGARLLRFGALISRLLPQVLNAWSIGRENLGPGQERPYSTVRTKVYAVL
ELWVQVCGASAGVLQGGASGEALLSHLLSDISPPADALRLRSPRGSPDAGLQTGKPSAPKKLKLDVGEAIAPPSHRKGDS
NANSDVCAAALRGLSRTILMCGPLIKEETHRRLHELVLPLVMGVQQGEALGSSPYTSSHCRRELYHLLLALLLAPSPRCP
PPLACALRAFSLGQREDSLEVSSFCSEALVTCAALTHPRVPPLQSVGPTCPAPAPVPPPEAPAPFRAPAFHAPSPLPSAG
PMPSAGPMPPVGPLPPTRPGPPATANHLGLSVPGLVSVPPRLLPGPENHRAGSSEDPVLAPSGSPPPTIPPDETFGGRVP
RPAFVHYDKEEPSDVEISLESDSDDSVVIVPEGLPPPPPPSSGTTPPPVAPAGPPAASPPVPAKDEPEELPAAPGPLPPP
PPPPVPGPVTLPPPQLVPEGTPGGGGPPALEEDMTVININSSDEEEEEEEEEEEEDEEEDEEEDFEEDEEEEEEYFEEEE
EEEEEFEEEFEEEEGELEDEEDEEEDEELEELEEVEFGPAGGEVEGGGPAPPSLPPALPPAESPKGPPEPGLEPGLLLEV
EEPGTEEAPGPETAPMLAPEVLPSQGEVEREGGSPPAGPPPQELVEEEPSGPPALLEEGAEGGGDKVSPPPEASAVEETE
VEAAALPPEKEQGDTAAMLADFIDCPPDDEKPPPASEPDS
> Bos indicus x Bos taurus [A0A4W2CB01]
MLSVAFPFSFHLVNYNSQGLLRMLAARPHRVTTCGKTEYRKFPQSKAIQTCPPSLHCCGLFNFFYKNFLSVCGVLGDDGR
TSGVLQTGQPWTLCLPIPLRHLSEPGENDLNLSAVGALVGLSNARLGSIKTRFEGLCLLSLLVGESPTEMFQQHCVSWLR
SIQQILQSQDPPPTMELAVTVLKDLLRYAAQLPAVFRDISMNHLPGLLTSLLGLRPECELSALEGMKACMTHFPRACGSL
KGKLASFFLSRVDALSPQLQQLACECYSRLPSLGAGFSQGLKHTDSWEQELRSLLASLHSLLGGLYEGAEAAPMQYESPG
AETLLSSSEDADAHTLLRLRQRFSGLARCLGLMLSSEFGAPVSVPVQDILDLICRTLSVSAKNVSLLGDGPLRLLLLPSL
HLEALDLLSALILACGARLLRFGALISRLLPQVLNAWSIGRENLGPGQERPYSTVRTKVYAVLELWVQVCGASAGVLQGG
ASGEALLSHLLSDISPPADALRLRSPRGSPDAGLQTGKPSAPKKLKLDVGEAIAPPSHRKGDSNANSDVCAAALRGLSRT
ILMCGPLIKEETHRRLHELVLPLVMGVQQGEALGSSPYTSSHCRRELYHLLLALLLAPSPRCPPPLACALRAFSLGQRED
SLEVSSFCSEALVTCAALTHPRVPPLQSVGPTCPAPAPVPPPEAPAPFRAPAFHAPSPLPSAGPMPSAGPMPPVGPLPPT
RPGPPATANHLGLSVPGLVSVPPRLLPGPENHRAGSSEDPVLAPSGSPPPTIPPDETFGGRVPRPAFVHYDKEEPSDVEI
SLESDSDDSVVIVPEGLPPPPPPSSGTTPPPVAPAGPPAASPPVPAKDEPEELPAAPGPLPPPPPPPVPGPVTLPPPQLV
PEGTPGGGGPPALEEDMTVININSSDEEEEEEEEEEEEDEEEDEEEDFEEDEEEEEEYFEEEEEEEEEFEEEFEEEEGEL
EDEEDEEEDEELEELEEVEFGPAGGEVEGGGPAPPSLPPALPPAESPKGPPEPGLEPGLLLEVEEPGTEEAPGPETAPML
APEVLPSQGEVEREGGSPPAGPPPQELVEEEPSGPPALLEEGAEGGGDKVSPPPEASAVEETEVEAAALPPEKEQGDTAA
MLADFIDCPPDDEKPPPASEPDS
> Ovis aries [A0A836CZI1]
MSRGLVGEENGRGSSVSVKLPWEVPLVGTEKKGVVLGVRDGEEESGCDENLSAVGALVGLSNARLGSIKTRFEGLCLLSL
LVGESPTEMFQQHCVSWLRSIQQILQSQDPPPTMELAVTVLKDLLRYAAQLPAVFRDISMNHLPGLLTSLLGLRPECELS
ALEGMKACMTHFPRACGSLKGKLASFFLSRVDALSPQLQQLACECYSRLPSLGAGFSQGLKHTDSWEQELRSLLASLHSL
LGGLYEGAEAAPMQYESPGAETLLSPSEDVDAHTLLRLQQRFSGLARCLGLMLSSEFGAPVSVPVQDILDLICRTLSVSA
KNVSLLGDGPLRLLLLPSLHLEALDLLSALILACGARLLRFGALISRLLPQVLSAWSIGRENLGPGQERPYSTVRTKVYA
VLELWVQVCGASAGVLQGGASGEALLSHLLSDISPPADALRLRSPRGSPDAGLQTGKPSAPKKLKLDVGEAIAPPSHRKG
DSNANSDVCAAALRGLSRTILMCGPLIKEETHRRLHELVLPLVMGVQQGEALCSSPYTSSHCRRELYRLLLALLLAPSPR
CPPPLACALRAFSMGQQEDSLEVSSFCSEALVTCAALTHPRVPPLQSVGPTCPAPAPVPPPEAPAPFRAPAFHAPGPLPS
AGPMPSAGPMPPAGPLPTARPGPPATANHLGLSVPGLVSVPPRLLPGPENHRAGSSEDPVLAPSGSPPPTVPPDETFGGR
VPRPAFVHYDKEEASDVEISLESDSDDSVVIVPEGLPPPPPPPSGTTPPPVAPAGPPAASPPVPAKDEPEELPAAPGPLP
PPPPPPVPGPVTLPPPQLVPEGTPGGGGPPALEEDLTVININSSDEEEEEEEEEEEEDEEEDEEEDFEEDEEEEEYFEEE
EEEEEEFEEEFEEEEGELEDEEDEEEDEELEELEEVEFGPAGGEVEGGPAPPSLPPVLPPAESPKGPPEPGLEPGLLLEV
EEPGTEEAPGPEVAPMLAPEVLPSQGEVEREGGSPPAGPPPQELVEEEPSGPPTLLEEGAEGGGDKLSPPPEASAVEEME
VEAAALPPEKEQGDTAAMLADFIDCPPDDEKPPPASEPDS
>Nomascus leucogenys [A0A2I3HFV0]
MELAVAVLRDLLRYAAQLPALFRDISMNHLPGLLTSLLGLRPECEQSALEGMKACMTYFPRACGSLKLACECYSRLPSLG
AGFSQGLKHTESWEQELHSLLASLHTLLGALYEGAETAPVQNEGPGVQMLLSSEDGDAHVLLQLRQRFSGLARCLGLMLS
SEFGAPVSVPVQEILDFICRTLSVSSKNISLHGDGPLRLLLLPSIHLEALDLLSALILACGSRLLRFGILISRLLPQVLN
SWSIGRDSLSPGQERPYSTVRTKVYAILELWVQVCGASAGMLQGGASGEALLTHLLSDISPPADALKLRSPRGSPDGSLQ
TGKPSAPKKLKLDVGEAMAPPSHRKGDSNANSDVCAAALRGLSRTILMCGPLIKEETHRRLHDLVLPLVMGVQQGEVLGS
SPYTSSRCRRELYCLLLALLLAPSPRCPPPLACALQAFSLGQREDSLEVSSFCSEALVTCAALTHPRVPALQPMGPTCPT
PAPVPPPEAPSPFRAPPFHPPGPMPSVGSMPSAGPMPSAGPMPSAGPVPSARPGPPTTANHLGLSVPGLVSVPPRLLPGP
ENHRAGSNEDPILAPRGTPPPTIPPDETFGGRVPRPAFVHYDKEEASDVEISLESDSDDSVVIVPEGLPPLPPPPPSGAT
PPPIAPTGPPTASPPVPAKEEPEELPAAPGPLPPPPPPPPPVPGPVTLPPPQLVPEGTPGGGGPSALEEDLTVININSSD
EEEEEEEEEEEEEEEEEEEEDFEEEEEDEEEYFEEEEEEEEEFEEEFEEEEGELEEEEEEDEEEEEEELEEVEREGGSPA
AGPPPQELVEEEPSAPPTLLEEETEDGSDKVQPPPETPAEEEMETETEAEALQEKEQDDTAAMLADFIDCPPDDEKPPPP
TEPDS
> Rhinopithecus bieti [A0A2K6MR96]
MELAVAVLRDLLRYAAQLPALFRDISMNHLPGLLTSLLGLRPECEQSALEGMKACMTYFPRACGSLKGKLASFFLSRVDA
LSPQLQQLACECYSRLPSLGAGFSQGLKHTESWEQELHSLLASLHTLLGALYEGAETAPVQNEGPGVEMLLSSEDGDAHV
LLRLRQRFSGLARCLGLMLSSEFGAPVSVPVQEILDFICRTLSVSSKNISLHGDGPLRLLLLPSIHLEALDLLSALILAC
GSRLLRFGILISRLLPQVLNSWSIGRDSLSPGQERPYSTVRTKVYALLELWVQVCGASAGVLQGGASGEALLTHLLSDIS
PPADALKLRSPRGSPDGSLQTGKPSAPKKLKLDVGEAMAPPSHRKGDSNANSDVCAAALRGLSRTILMCGPLIKEETHRR
LHDLVLPLVMGVQQGEVLGSSPYTSSRCRRELYCLLLALLLAPSPRCPPPLACALQAFSLGQREDSLEVSSFCSEALVTC
AALTHPRVPPLQPMGPTCPTPAPVPPPEAPSPFRAPPFHPPGPMPSVGPMPSAGPMPSAGPMPSAGPVPSARPGPPTTAN
HLGLSVSGLVSVPPRLLPGPENHRAGSNEDPILAPSGTPPPAIPPDETFGGRVPRPAFVHYDKEEASDVEISLESDSDDS
VVIVPEGLPPLPPPPPSGWGTPVPAKEEPEELPAAPGPVPPPPPPPPPPVPGPVTLPPPQLVPEGTPGGGGPPALEEDLTV
ININSSELEEEEEEEEDEEEEEEELEEVEELEFGTAGGEVEEEPEPGLLLEVEEPGAEEEHGADTAPTLAPEVLPSQGEVER
EEGSPSAGPPPQELVEEEPSAPPTLLEEGTEDGGDRVQPPPETPAEEEMETETEEAEALQEKEQDDTAAMLADFIDCPPD
DEKPPPPTEPDS

> Bos mutus [A0A6B0SA35]
MAAAVLSGPSAGSAAAVPGGPGSLSAVGSGPRLRLMLLESVSGLLQPRTGSAVAPVHPPARSAPHLPGLMCLLRLHGTVG
GAQNLSAVGALVGLSNARLGSIKTRFEGLCLLSLLVGESPTEMFQQHCVSWLRSIQQILQSQDPPPTMELAVTVLKDLLR
YAAQLPAVFRDISMNHLPGLLTSLLGLRPECELSALEGMKACMTHFPRACGSLKGKLASFFLSRVDALSPQLQQLACECY
SRLPSLGAGFSQGLKHTDSWEQELRSLLASLHSLLGGLYEGAEAAPMQYESPGAETLLSSSEDADAHTLLRLRQRFSGLA
RCLGLMLSSEFGAPVSVPVQDILDLICRTLSVSAKNVSLLGDGPLRLLLLPSLHLEALDLLSALILACGARLLRFGALIS
RLLPQVLNAWSIGRENLGPGQERPYSTVRTKVYAVLELWVQVCGASAGVLQGGASGEALLSHLLSDISPPADALRLRSPR
GSPDAGLQTGKPSAPKKLKLDVGEAIAPPSHRKGDSNANSDVCAAALRGLSRTILMCGPLIKEETHRRLHELVLPLVMGV
QQGEALGSSPYTSSHCRRELYHLLLALLLAPSPRCPPPLACALRAFSLGQREDSLEVSSFCSEALVTSTANHLGLSVPGL
VSVPPRLLPGPENHRAGSSEDPVLAPSGSPPPTIPPDETFGGRVPRPAFVHYDKEEPSDVEISLESDSDDSVVIVPEGLP
PPPPPSSGTTPPPVAPAGPPAASPPSPKGPPEPGLEPGLLLEVEEPGTEEAPGPETAPMLAPEVLPSQGEVEREGGSPPA
GPPPQELVEEEPSGPPALLEEGAEGGGDKVSPPPEASAVEETEVEAAALPPEKEQGDTAAMLADFIDCPPDDEKPPPASE
PDS
> Monodelphis domestica [F6U4E3]
MAAAVLSGSPAGSGAGGPGGAGGLPAAGPGSRLRMLLLESVSGLLQPRTGSATPPSPPTAPLVPGLVRLLRLHGTMGGAQ
NLSAVGALVGLSNARLGSIKTRFEGLCLLSLLVAESPTEMFQQHSVSWLRSIQHVLQSQDPAPTMELAVAILRDLLRYSA
QLPELSRDISTNHLPGLLTSLLGLKPECELSALEGMKACMTYFPRACGSLRGKLASFFLSRVEALSPQLQQLACECYARL
PALGAGFSQGLKHTESWEQELHCLLASLHGLLGALYEGAETAPIQYEGPGVELLLPPPADNDVHGLLQLRQRFSGLARCL
GLMLSSEFGAPVSVPVQDILDIICRTLSVSGKNISWLGDGPLRLLLLPSIHLEALDLLSALILSCGSRLVRFGGLICRLL
PQVLNTWSAGRDPLPPGQERPYSAVRAKVYSVLELWVQTCGAGAGVLQGGAQSEALLTHLLSDIFPPADALKLRGPRVTQ
DGGLQSGKPSAPKKLKLDSGEAVVPPMHRKGDSNANSDVCTTALRGLSRTILMCGPLIKEETHRRLHELTLPMVMSTQQG
EIPGGSPYTSARCRRELYRLLLALLLAPAPQCPPPLTCALQAFSLGQREENLEVSSFCSEALVTCAALAHPRVPPLQSPA
PSSPPIAPPTQPEPPPPFRAPSFHPPGPRPPASATANHLGLPVPNLVSAPPRLLPGPENHRAALSEDSAPAPAGTPPPSL
PPDEAFGGRPPRPAFVHYEKEETSDVEISLESDSDDSVVIVPEGLLPLPPAPPQAGTPPPAPPVVATAASPPLPPKEEPE
ELPPAPGPLPPPPGSGPLPPPQLVAEGTPGGAPPPLEEDLTVININSSDEEEEEEEEEEEEEEDFPEDEDEEEDEEEYFE
EEEEEEEEFEEEFEEEEEGELEEEEEEEEEEEEEEEELEEVEFEPVEGEVEIRGAPPPDSSPPPPPSEPSKEESEPGLLME
VEETNAEDEKEGESAPPLAPEASPPQGEREEKERTVVPEAPPAEEPTGNEPPALSGEGTDGKGNQEPLPPAKAAEASATE
METVPKGEKEQDDTAAMLADFIDCPPDEEKPTPPPEPDS
> Monodelphis domestica [A0A5F8GYZ3]
MPRAPHLPYLLLLHSRSVLTSPQCAAAKSAGEDGGSRAEREPRRFWGWGSGRGRRSPGRRSWVASPHAAFGERIWVIAAS
DWVCHASLAPHRPAGAGARASAEAARDDGRGPEPLSCGSTGGSQQCPTWFHQDSSQDPAPTMELAVAILRDLLRYSAQLP
ELSRDISTNHLPGLLTSLLGLKPECELSALEGMKACMTYFPRACGSLRGKLASFFLSRVEALSPQLQQLACECYARLPAL
GAGFSQGLKHTESWEQELHCLLASLHGLLGALYEGAETAPIQYEGPGVELLLPPPADNDVHGLLQLRQRFSGLARCLGLM
LSSEFGAPVSVPVQDILDIICRTLSVSGKNISWLGDGPLRLLLLPSIHLEALDLLSALILSCGSRLVRFGGLICRLLPQV
LNTWSAGRDPLPPGQERPYSAVRAKVYSVLELWVQTCGAGAGVLQGGAQSEALLTHLLSDIFPPADALKLRGPRVTQDGG
LQSGKPSAPKKLKLDSGEAVVPPMHRKGDSNANSDVCTTALRGLSRTILMCGPLIKEETHRRLHELTLPMVMSTQQGEIP
GGSPYTSARCRRELYRLLLALLLAPAPQCPPPLTCALQAFSLGQREENLEVSSFCSEALVTCAALAHPRVPPLQSPAPSS
PPIAPPTQPEPPPPFRAPSFHPPGPRPPASATANHLGLPVPNLVSAPPRLLPGPENHRAALSEDSAPAPAGTPPPSLPPD
EAFGGRPPRPAFVHYEKEETSDVEISLESDSDDSVVIVPEGLLPLPPAPPQAGTPPPAPPVVATAASPPLPPKEEPEELP
PAPGPLPPPPGSGPLPPPQLVAEGTPGGAPPPLEEDLTVININSSDEEEEEEEEEEEEEEDFPEDEDEEEDEEEYFEEEE
EEEEEFEEEFEEEEEGELEEEEEEEEEEEEEEEEELEEVEFEPVEGEVEIRGAPPPDSSPPPPPSEPSKEESEPGLLMEVEE
TNAEDEKEGESAPPLAPEASPPQGEREEKERTVVPEAPPAEEPTGNEPPALSGEGTDGKGNQEPLPPAKAAEASATEMET
VPKGEKEQDDTAAMLADFIDCPPDEEKPTPPPEPDS
> Balaenoptera physalus [A0A643BM64]
MCLLRLHGTVGGAQNLSAVGALVGLSNARLGSIKTRFEGLCLLSLLVGESPTEMFQQHCVSWLRSIQQVLQSQDPPPTME
LAVAVLRDLLRYAAQLPTLFRDISMNHLPGLLTSLLGLRPEGKLASFFLSRVDALSPQLQQLACECYSRLPSLGAGFSQG
LKHTDSWEHELHSLLASLHSLLGALYEGAETAPAQYEGPGVETLLSPSEDGDAHVLLRLWQRFSGLARCLGLMLSSEFGA
PVSVPVQEILDLICRTLSVSAKNILRSPRGSPDGGLQTGKPSAPKKLKLDMGEAMAPPSHRKGDSNANSDVCAAALRGLS
RTILMCGPLIKEETHRRLHDLVLPLVMGVQQGEVLGNSPYTSSRCRRELYRLLLALLLAPSPRCPPPLACALQAFSLGQR
EDSLEEEEEEEEEEEEEEEEEDFEEEEEDEEEYFEEEEEEEEEEFEEEFEEEEGELEEEEEDEDEEEEVEEVEFGPAGGEV
EEGGPAPPSLPPALPPAASPKVQPQPEPEPGLLLEVEEPGAEEGPGAETAPTLAPEVLPSQGEVEREGGSPPAVPPPQEL
VEEEPSVPPTLLEEGAEGGGDKVPPPPEASAAEEMETEVEAAALQEKEQDDTAAMLADFIDCPPDDEKPPPATEPDA
> Chiloscyllium punctatum [A0A401RHN7]
MLFRQVLNSDWDNSSDLSGLISISNSSLRSVKSRFEGVCLLAVLVADSPTDTFQDQCISWLRAVQHIIQSQDPQTTTELA
IWVLHDLLKYSSQLLELAREVAMNHVPAIVTALLALKPECQGAALEGMKACMTFYPRACGSLRGKLAAYFLSKLDTGSPH
MQELACQCYVLLPGLGAGFAQGMKYVESWAQQLHYLLATLHGLVEQLYEGAETDPVRYEGPGVMLPLPELNDGDPFHVLQ
LRQRFSALSKCLSLLLSSDFPVPVKLPVQDVLNLVCRILNISTKNMVLTAWSTPRESAVPGQERAYSSVRVKAYEVLGTW
LKVCGASSGVLQGPFHHCDILLANLIADVTPAVDTTKLRPSKPAGDGSQPFNYGKPSAKRQKLMDLEDAAAMPGHRKRDP
TANGDVCLAALKVCSDVILSCGSLLKEETHKKLHELVVPLLLRLQQNSSMTSPYLRADCRRELYRLLLFLLLVPNSKWPP
PLHCAVRMFSQGQNDSNTEVSSFCLEAMVICNCLIHPRVPSLQMPLSAASLIATAFKPTPNADPQTPARPPQVPTFHLPA
PPPPSHPPPPGAGPRALTDHAPPARFSLPPPARFPSSVESHRGMGQEGIPTAPPGEEAVFGGDANCSRPVFIRYDKEEES
DVEISLDSDSDDSVVIVPEGLLPDRGAPAAMGGTGPSAAGPPSSSSSSPPALPPPPATPADAKRRRKEEEGQGEEAPEEI
EKVVVASPRAQGAKAPTPLPIPTPQPQEPGGPGEARPASQEPDEELTVININSSEDEEGEEDYLEEEEEEEEDYYDEEEE
EEEEEEEEDYEDEEEDFEEEGELEEDEMDEEMEDEDEEEEEEEEGEVEMYRPRGPGDTSEEEPALAPRPVELPPAPPQEGE
GPGPPEPHPEGVPSPRRSEGEGSPGGRGQGAPVQSQPPPQPMGESEERARPEMLQTTPPLPGERADGGDSQPPSAGGGGA
AGPGEEGAPTLQEEEGGDGAADAVEAPPQGEVEEEGVTEAEPMEQQQREEEEEPTEKAAPETEEQEDQAAAMLADFVDCP
PDEEEVKDMAAGATSSSST
> Perca fluviatilis [A0A6A5EMY0]
MATSAWLRGPSATRLTEGLVSVLKEQRTEYLPALLAHYREHGVFQTQGASTVGGLVGFSNAKLGSSKTRFEGLCLLSMLV
KDSSSDLFQQHCVSWLRSLQQVIQSQAPVQTIQLAVNILKDLLQYSSQLAELAREVGLNSILGILTSLLGLKTECELAAM
EGMTACMTYYPRACGSLRDKLGAYFLSKMDSTNKKTQEMACLCYGHLPCLGGLLDRGVGAGRAEGWTNQIHCLLASANGL
LAQIYQGSESDGAVQYEGSGVELAFPHLDQSDPLLLLQLQHRYTAVCLALKHTLRVDPASAVRLPVRPILNLVCRVLAVS
SKSINLTGDGSVRLLVLPVIHTHTLEVLSALITAVRSGMVQYAAVLQRLFSQTLSAWAPPPEASLGQQRAYSSVRVSVYR
TLELWIQVAGASASILQGSPGHSELLFSHLLGDITPGAESVKLRVGLSADAVPGGKPGPRRTKQLVIVDTVGPSLQRKGD
LLANQDTCLSALRALRQIILASGTLLKDDIHKRLHDVVLPLCVRLQQQQSSSSISCESAGGVSGQYSSALARQELYRLLL
ALVLVPSPCWPPPLTCAVSILSNGRNDHNLKVSTFCTEALTICNSLLHPRLPSIALPLPPLTLKPTPAAPVLPSSQGTTP
GLTLPTLLGGPAPGPPFATRHTLGLGPSSLLGSLENHLSLVPGLSGQAPGDMILSPHGHHQPEPAGLGPPEGQRPVFVRY

DREEADDVEISLASDSDDSVVIVPPGMLNMDSQQDETAAAAANSHNMASAAPGTLPGGESVAMVPTTTATGTTIDRLSLA
NDLATSSPLLTTSTAPINSFPPPGSSVVSLVPPLNSSTLAAPPSALGDSLPGRPQLQQMLMQASTPSQPGAMALPLQMHQ
LQNQLSQQGRHLHPHQPPAQSASNEDSGVININSTDEEEEEEDDMEDDEELDEEEEGMDEDDEEEDVSDFADEEELYDGE
EYGEYDEEEGEELEEEEEEEEEDGDIPPLEGAEDKAGEAVTAGVNVLRAAVDDAGMAGFSVEGEAEGGIEEIQNSRSLFG
DDRIKVQKVESIGVLEETREGEEDESERMDDPTMPQILCVTGGALEEREETEEDAAGGGGEGEGEGSWEQGANEIELTAA
SEECTANPSQQEPGAEPAQEAGVSDIQPSSQQEEQLAAVQDGDPPAAADPETSTGPREHEETDAEQRAETKAEQQETEGV
EGGASDGEEGKGVKRKREEPQEEAGRSTEKKKLDDEAMASMLADFVACPPDDEDGASGSNRS

> Astyanax mexicanus [A0A3B1JG98]

MATAAWLHGQSPNSARLTEGLVSALKEERPEYLPALLSNYREHGAVAAQNSGTLGGLVGISNARLGTSKTRFEGLCLLSM
LVKDSSSEVFQQHCLSWLRSLQQIIQSQAPLPSVQLAVSVLQDLLQYSSQLPELAREVGLNSILGILTSLLGLKSECHLA
AMEGMMTCMTYYPQACGSLKEKLGVYFLSKMDSDHPKVQEVASECYGRLPCLGGVLERGGGGRRAEGWTNQVHCLLASAS
SILCQLYQGIETEETIEYDGPGLELPFPPLDDVDPLLILQLRQRYRAVCLALKHTLSVDPATSVRLPIQHVLNLVCRALA
VSTKSINVTGEGCLKLLVLPSIHSDSLELLSTLIKVVGGGLVQYCSVITRLFSQSLSAWTPLPEASLGQQRAYSAVRVAI
YHTLELWVRVGGASASVLQGSTSHSEVLFAHLLGDITPGSEAVKLRAGQTSLSDLVGAAGKAGPRRTKGLTIGDPGGVSL
QRKGDALANQDTCLAALRALRQIILTCGTLLKEDQHKRLQDLVVPLCVRLQQQAQCGSEVGVASGQYGSAAPRRELYRLL
LALVLVPSPRWPPPLSCAVSVFSLGRRDRNVMVSSFCTEALTICNTLLHPRTPSIALPLPPLTLKPIPAAPVLGPTQNPS
LSLPTLLGGPAPGPSFTSRHPLGLGPAALLGSLDNHLPLAPPVLPTTAGATGTQGDLLLSPAQPAELAGLGAPEGQRQVF
VRYDKEEPEDVEISLESDSDDSVVIMPQGMMLEMQEGVANAQNLPPPPGGALTTGAEAGTVDTSLPNELSTPLSHQILPT
DANIINSFPGPGQTDQLVSLVPPLNSTGVSLTPSPGGLGNSLPAGPQLQQMLMQPSPGGQPAQLGLSLHSQLQSQLAQTS
RQLQQPTAAEKDQNVININSSDDEEEEYEEMEDEDELEEDEEDEEDEDDYPDDEEYQGEEDEELEEEEGEEMDEEDEEED
EEDIQALEGENRGGLMGGEEGEVMIEEQEERRINAFQMEQEAQVEAGIEEMEGVRSVYGEEGLKDKVAVEEIENIGAVER
NESEPAEAPLETHVIVEEAEEPEENASAAEPQQEARTWEQEGTGQEPEPAVPEASAEPQENQEVTAEVGVSEQSTEAEQE
VTPNENTASTSEEVPPQQVEEAAEKEKDAGQPVEEEKQQQQQDEKVEEKKEEEEEEKEVRGTKRKIEDREGEEPEQGTE
KKKVDDEAMASMLADFVDCPPDDEDHGASQSRS

> Sphaeramia orbicularis [A0A673B8V5]

MTSPRRTVVTFAWQRGKMAASAWLHGPSAMRLTEGLVSVLKEQRPDYLPALLASYREHGVFPTQGASAVGGLVGFSNAKL
GSSKTRFEGLCLLSMLVKDSSSEVFHQHCLSWLRSLQQVIQSQAPVQTIQLAVNILKDVLQYSTQLAELAREVGLNSILG
ILTSLLGLKTECELTAMEGMMACMTYYPRACGSLRDKLGAYFLSKMDSTNKKTQEMACQCYGRLPCLGGLLDRAAGAGRA
EGWTNQIHCLLASANGLLDQIYQGSETEATVQYEGRGVELAFPHLDQSDPLLLLQLQHRYTAVCLALKHTLRVDPASAVR
LPARLVLNLVCRALAVSSKSINLTGDGNVRLLVLPIIHTNTLEVLSALITVARIGMVQYAAVLQRLFSQTLSAWTPLPET
SLGQQRAYSSVRVAVFRSLELWVQVGGASANILQGSPTHAELLFNHLLGDITPGAESIKLRGGLSADAVPGGKPGPRRTK
PLVMTDTVGPLMQRKGDLMANQDTCLSALSAMRQIILSSGTLLKDDIHKRLHDVVLPLCVRLQQQTSSSSACDSAGGIS
GQYSSALSRRELYRLLLALVLVPSPCYPPPLSCAVSILSKGRTDRNLKVSSFCTEALTICNSLLHPRTPSIALPLPPLTL
KPTPSAAGLTPSQGPTPGLTLPTLLGGPTPGPPFPSRHSLGLGPAASLLGSLENHLSLVPGLPGQAPTPGDMMLSPHTMH
HQPEPTGLGPPEGQRPVFVRYDKEEAEDVEISLESDSDDSVVIVPPGLLGMENQQDETAAAVNSQNMASTTQGSTTLTLP
GGDSVTMVPTTSSTTTIDGLSLPNDLATSSPLLATSTTTPINSFPPSSGSVVSLVPPLNSSALTASPSGLADSLPGRPQL
QQMLMQPSNAGQPGPMGLPLQMHQLQNQLSQGRHLHQQHPPPAPASNEDSAVININSTDEEEEEEEDMEDDEELDEEEEG
MDEEDEEEDVSDYGDEEFYDGEEDFEYDEEEGEELEEEEEEDGDIPPLEGAEDKGAEGGVEEGKVLSAAVDEGGIAGFSV
EGEPEGGIEEIQTRALFGEDRVKVQEVESIGVLEDAREGVGEEDESERMDDPTMPQILCVTGGAMEERDEAEEEDSGGGA
ADGQGDPQEEGPTWEKETNDVEPTDSSEEHMTNQSQQEQDPEAAQESGVSESQPSGPEEEQLEAVQAGPSSSSDPEAPAG
PSTATTTTTTTTTTTEEEEPVSEHRDKEEAEPQAKEAAQKENDEEESKGVKRKREEVIGDEEAGQSTEKKKMDDENMASM
LADFVACPPDDEDGASGSNPN

> Branchiostoma lanceolatum[A0A8J9Z0N3]

MAAHMANVLLTSLSEKEGKSLLPWAIEAANEHQLLQSEQNVQDWVSHINTSLGAAKTRLE
GLCLLGTVVRQCSAGTFIQHGTTWIRMLTQVLQAYDSPLTLQMASHVLACVVQQAAQYPE
VAREVASTNIPALVQCLLGAQDHQWFPSALEALHSCMKNFPGPCGPSKGKIESLVCGLMD
TNQPRLSQLLQQTCPLLAGCGGGGAGGVKYTEAWTHLCDQVLGSLHQVLDDTYQDIETGL
QTYSAPQVSLRLKTVPESDPERTFVLSTRFHNLCGCLQQLVSQDFPSVVRIPVPDILALL
CRALGVNAKMLFGKAAMEHVLLMSALPKIHCSALSLLEALIISCRSHLVPHASVISQLLV
QTLGWTTSDEGVPGRQKPHSTLRSRAYTVLTVWLNVCGAASGVDSHADVILQHVLKDATP
QADTTKLKANRPGSQDQSRRQKKKQRGVDVTDQGLTGHRKVDSQANSDVCSAALEALKTL
LEVVGSIIKPSIHKETQEFVIPLLLKTHQNQSDPPIPYSCARCRKGLYQLLLAAVLVLHP
RWPPPTHCAVKIFSVGQQDYDLQVSSYCREALLTCMSIIHPRAPTLQSPVIISDTPTISK
LPTNHMAPSFQMLRTNASETMLKNSSSNGNLFTVPSFKSASQSEGMLKDNPSQSDSTAEV
PESFSKTLRREEDMAGPSVIHLDGSSSSSEGQSSSEDSVEFLEDVKNSEPDSSTLETGEP
EDAVSNAGRELATDMPDNEDVVLIQGHIEEGSAEAPADTTDLSTTAGVKRKHESDGEGDD
DNDIQNADIQAMLAAFVPDSTPDEN

> Takifugu rubripes [H2SWC4]

MENLCFLCVGGRFEGLCLLSMLVKDSSSDLFQQHCLSWLRSLQQIVQSQAPVQTIQLAVNILKDVLQYSSQIPELAREVG
LNSILGILTSLLGLKTECELAAMEGMTACMTHYPRACGSLKDKLGAYFLSKMDSTNKKTQEMACQCYAHLPCLGGVLDRG
AGAGRAEGWTNQIHCLLASANSHLALIYQGAEMEGTMQYEGPGVELAFPLLDQSDPLFLLQLQHRFTAVCLALRHTLRVD
PASAVRIPVRPILNLVCRALAVSCKSFNLTGDGNVRLLILPIIHLNILEVLAALIIAVRSSMVQYAAVLQRLFSQTLSAW
TPAAEASVGQQRAFSSVRVSVYRTLELWLQVAGASTSILHGSPNHSEILFNHLLSDITPGAESVKLRVGLSAEIVPGGKP
GPRRTKSLVISDTVGPSLQRKGDIMANQDTCLTALRALRQIILVSGTLLKDDIHKRLHDVLLPLCVRLQQQLSSNMSCD
STAGISGQYSSALTRRELYRLLLALVLVPSPCWPPPLTCVVSILSSGRTDRNLKVSTFCCEALTVCNSLLHPRSPSIALP
MPPLSIKPAHAVSVLPTPQASTPGLTLPTLLGEPTPPPPFPSPHTLGMGPSSLLGSLENHLSLVPGLRGQTSGPSEMILS
PHAHHQDLAGLGPPEGQRPVFVRYDREEAEDVEISLASDSDDSVVIVPPGMLNMENQQDDVAAANSQSMTAAAGGAAVTL
AEGEPVTMVPNTATAAPIDGVSLPNDLTTSAPLLTTSAPPINSFPPSSASVVSLVPALNSNPLTAPPGGLVEPIPSRPQL
QQMLMQPSAAVQQGPLSLPLQIHQLQSQLGQQGRPLQQQPPVASNEDSGVININSTDDEEDDEDMEDDEEEEEEEGVEDE
EEEEDEVSSFADEEFFEDYEEFEYDAEELEEEDEEEGDIPPLEGAEEQGEQEEVEQGGVLQAAVEAAEIVDFSVEGEAGG
GIEEIQTKRALFPEDRMKVQEVESIGVMEEARGEAEEDETERVCDPTMPQILCVTGGALEEKEEEGGRVQEDMSSWERDA
KKVEPQGPSGEAAASTAEQEESGGEQQAGSSEERPSSLGEEQEVTVSEEDAALTHPGGSDESTPEPQETVTEKREAAAAA
EQQELHAGGGEEGKGVKRKREELQSENELEGSSKQADEDTMASMLADFVACPPDDDDGPSASNQS

>Sphaeramia orbicularis [A0A673B3T8]

MAASAWLHGPSAMRLTEGLVSVLKEQRPDYLPALLASYREHGVFPTQGASAVGGLVGFSNAKLGSSKTRFEGLCLLSMLV
KDSSSEVFHQHCLSWLRSLQQVIQSQAPVQTIQLAVNILKDVLQYSTQLAELAREVGLNSILGILTSLLGLKTECELTAM
EGMMACMTYYPRACGSLRDKLGAYFLSKMDSTNKKTQEMACQCYGRLPCLGGLLDRAAGAGRAEGWTNQIHCLLASANGL

LDQIYQGSETEATVQYEGRGVELAFPHLDQSDPLLLLQLQHRYTAVCLALKHTLRVDPASAVRLPARLVLNLVCRALAVS
SKSINLTGDGNVRLLVLPIIHTNTLEVLSALITVARIGMVQYAAVLQRLFSQTLSAWTPLPETSLGQQRAYSSVRVAVFR
SLELWVQVGGASANILQGSPTHAELLFNHLLGDITPGAESIKVSPQSTTDAVPGGKPGPRRTKPLVMTDTVGPLMQRKGD
LMANQDTCLSALSAMRQIILSSGTLLKDDIHKRLHDVVLPLCVRLQQQQTSSSSACDSAGGISGQYSSALSRRELYRLLL
ALVLVPSPCYPPPLSCAVSILSKGRTDRNLKVSSFCTEALTICNSLLHPRTPSIALPLPPLTLKPTPSAAGLTPSQGPTP
GLTLPTLLGGPTPGPPFPSRHSLGLGPAASLLGSLENHLSLVPGLPGQAPTPGDMMLSPHTMHHQPEPTGLGPPEGQRPV
FVRYDKEEAEDVEISLESDSDDSVVIVPPGLLGMENQQDETAAAVNSQNMASTTQGSTTLTLPDSVTMVPTTSSTTTIDG
LSLPNDLATSSPLLATSTTTPINSFPPSSGSVVSLVPPLNSSALTASPSGLADSLPGRPQLQQMLMQPSNAGQPGPMGLP
LQMHQLQNQLSQGRHLHQQHPPPAPASNEDSAVININSTDEEEEEEEDMEDDEELDEEEEGMDEEDEEEDVSDYGDEEFY
DGEEDFEYDEEEGEELEEEEEEEDGDIPPLEGAEDKGAEGGVEEGKVLSAAVDEGGIAGFSVEGEPEGGIEEIQTRALFGE
DRVKVQEVESIGVLEDAREGVGEEDESERMDDPTMPQILCVTGGAMEERDEAEEEDSGGGAEQDPEAAQESGVSESQPSG
PEEEQLEAVQAGPSSSSDPEAPAGPSTATTTTTTTTTTTEEEEPVSEHRDKEEAEPQAKEAAQKENDEEESKGVKRKREE
VIGDEEAGQSTEKKKV
> Oryzias javanicus [A0A3S2PFZ3]
MATSVGLHGSSALKLTEGLVSFLKEQRPEYLPEVLVNYREHGLQIQGQNDAAGLVGFSNAKLSSSKTRFEGLCLLSVLVK
DSTSDVFQQHCLFWLRSLQQIIQSQAPVQTIQLAVNILKDLLQYSCQLAELAREVGLNSILGILTSLLGLKTECELAAME
GMVACMVFYPRACGSLKDKLGAYFLSKMDSNNKKTLEMACLCYSHLPCLGGLLDRAISAGKADGWTNQIHCLLATADGLL
TQMYKGSEPETVVQYHGPGVELGFPYLDPTETLVLLRLQQRYTAVCRAVKHTLRVDPASAVRLPVKPILNLVCRALAVNA
KNTSFSADGSLMLLILPSIHCNTLEMLSDLITVVRSSMVPYAAIIQRLFSQTLSAWTPVVEAGVGRQRGYSSVRVSVYRT
LELWVKVVGASVSILQENPMHADLLFSNLLGDITPGPESIKLRAGLGVDVVPGGKPGPRRTKHLVIADPMGPSLQRKGDP
LSNQDTCLAALRALRQIIQTCGTLIKEDIHKRLHEVVLPLCVCLQQQKDSSSVFESTGAISGQYSSALARRELYRLLLAL
VLVPSPSWPPPLTCAVSIFSNGRTDRNLKVSSFCAEALTICNTIVHPRTHSIALPLPPLTLRPSASTSVLSPQGSAPRLT
LPTLLEGHGSGPAFPSRQSLNLGTSSLLSSLENHLSTVPGLPAQAPTPGDLFSPHPHRQHDSAGLGLPESQRPVFVRFDK
EEPDDVEISLASDSDDSVVIVPPGMINLDNQQDEAAANPQTMLSAAPAGSSVTLPATESNSMVPITAATTTIDVSTLPND
LPASSSLLTTSASSINSFPPPSASAATLPPALNSSIVSDPHSELSDSLPCKPQLQQMLMQAPTSSQGSPMGLPLQMHQLQ
NQLTQQGRLHQQLPAPPPSNEDSTIININSTDDEEEEEDLEDDEELDEEEEGLDEEDEEDGSDDFYDGEEYEDYDEEEGE
ELEEEEEEEENGDMPPLEGSEEKLVDVAIEEGQVLPAADEKVLTEHNVEADSERGIQELQPQRTMFGEERVKLQEVESIG
VLEDAQVGVEEEDESERMDDPTMPQIICVSGGAQESVETGDEGEGPAHVIQEDISDQPAKEDEPKAAPEEQASFSAEQEP
EVETAQEDESSSSKTSDQPAELPAAAPAEPTEATNSPADLNAAEQQAESQASEGGEQPKEGEEEEENRSIEPQREESDKE
EAHGTKRKREDELTEEAGPSTEKKKVDDEAMASMLADFVACPPDDEAVPGSTCS
>Perca flavescens [A0A484C8W1]
MLVKDSSSDLFQQHCVSWLRSLQQVIQSQAPVQTIQLAVNILKDLLQYSSQLAELAREVGLNSILGILTSLLGLKTECEL
AAMEGMTACMTYYPRACGSLRDKLGAYFLSKMDSTNKKTQEMACLCYGHLPCLGGLLDRGVGAGRAEGWTNQIHCLLASA
NGLLAQIYQGSESDVAVQYEGSGVELAFPHLDQSDPLLLLQLQHRYTAVCLALKHTLRVDPASAVRLPVRPILNLVCRVL
AVSSKSINLTGDGSVRLLVLPVIHTHTLEVLSALITAVRSGMVQYAAVLQRLFSQTLSAWAPPLEASLGQQRAYSSVRVS
VYRTLELWIQVAGASASILQGSPSHSELLFSHLLGDITPGAESVKLRVGLSADAVPGGKPGPRRTKQLVIVDTVGPSLQR
KGDLLANQDTCLSALRALRQIILASGTLLKDDIHKRLHDVVLPLCVRLQQQQSSSSISCESAGGVSGQYSSALARQELYR
LLLALVLVPSPCWPPPLTCAVSILSNGRNDHNLKVSTFCTEALTICNSLLHPRLPSIALPLPPLTLKPTPAAPVLPSSQG
TTPGLTLPTLLGGPAPGTPFPTRHTLGLGPSSLLGSLENHLSLVPGLSGQAPGDMILSPHGHHQPEPAGLGPPEGQRPVF
VRYDREEADDVEISLASDSDDSVVIVPPGMLDMDSQQDETAAAAAAAANSHNMASAAPGTLPGGESVAMAPTTTATGTTI
DRLSLANDLATSSPLLTTSTAPINSFPPPGSSVVSLVPPLNSSTLAAPPGALGDSLPGRPQLQQMLMQASTPSQPGAMAL
PLQMHQLQNQLSQQGRHLHPHQPPQSASNEDSGVININSTDEEEEEEDDMEDDEELDEEEEGMDEDDEEEDVSDFADEEE
LYDGEEYGEYDEEEGEELEEEEEEEEEEDGDIPPLEGAEDKAGEAGTDGVKVLRAAVDDAGMAGFSVGGEAEGGIEEIQNS
RSLFGDDRIKVQKVESIGVLEETREGEGEGEEDESERMDDPTMPQILCVTGGALEETEEDAAGGGEQGEGGGEGEGEGEG
SWEQGANEIELTAASEECTANQSQQEPGAEPAQDAGVSDLQPSSQQEEQLAAAQDGEPPAAADPETSTGPREHEETDAEQ
RAETKAEQQETEGVEGEASDGEEGKGVKRKREEPQEEAGRSTEKKKLDDEAMASMLADFVACPPDDEDGASGSNRS
> Oryzias latipes [A0A3B3H3F0]
MATSVGLHGSPALRLTEGLVSFLKEQRPEYLPEVLVNYREHGLQIQGANDVAGLVGFSNAKLSSSKTRFEGLCLLSVLVK
DSSSDLFQQHCLFWLRSLQQIIQSQAPVQTVQLAVNILKDLLQYSSHLAELAREVGLNSILGILTSLLGLKTECELAAME
GMVACMLFYPRACGSLKDKLGAYFLSKMDSNNMKTQEMACLCYSHLPCLGGLLDRAISAGKADGWTNQIQCLLATADALL
TQIYKGSEPEMVVQYTGPGVELAFPYLDPTEPLLLLRLQQRYTAVCRAMRHTLRVDPASAVRLPVKPILNLVCRALAVNS
KSTSLSADGSLMLLILPSIHCNTLEMLSDLITVVRSSMVPYAAIIQRLFSQTLSAWTPLVEAGVGRQRGYSSVRVSVYKT
LELWVKVVGASVSILQENPMHADLLFSNLLGDITPGPESIKFRAGLGVDVVPGGKPGPRRTKHLVIADPMGPSLQRKGDP
LSNQDTCLAALRALRQIIQTCGTLIKEDIHKRLHEVVLPLCVCLQQQKGSSSVFESTGAISGQYSSARARRELFRLLLAL
VLVPSPSWPPPLTCAVSIFSNGRTDRNLKVSSFCAEALTICNTMVHPRTPSIALPLPPLTLKPPTSAPVLSPQGSAPRLT
LPTLLEGHGSGPPFPSRQSLNLGPSSMLSSLENHLSTVPGLPAQGPTPGDLFSPHRQHDSGLGLPESQRPVFVRFDKEEP
DDVEISLASDSDDSVVIVPPGMINLDQQQDEAAANVQTMLSAAPAGGGVALPAAESNSMVPITAATSTSDVSTLLNDLPA
SSSLLATSASSVNSFPPSGGSAAPLPPALNSGIVSDPHGDLADALPCKPQLQQMLMQAPAAGQASAMGLPLQMLQTPLSQ
QGRLHPPLPAPPSNEDSAIININSTDDEDEEEDLEDDEELEEEEEDGLDEEEEEEGSEDFYDGEEYEDYDEEEGEELEEE
EEEEEEEIGDMPPLEGSEEKLVDVAIEEGQVLHPAADDKEPSQLHAEADSERGIQELQPQRTVLGEDRVKLQEVESIGVL
EDVQVGVEEDESERMDDPTMPQIICVSGGAQESLESGDEGGGTTSVLQEDRSHQAAKEEELEAAAEEQAGFRAEEPEGET
APEDVSSSDAPDPPAGRPAGAPAEQAEAGRRNPAEEEEKQAENPASEGGEQPKGGEEEEKSEPPREESDKEEAQGTKRKR
EDEVEEEAGPSTEKKKVDEDAMASMLADFVACPPDDEAAAAAAGSTCS
> Oncorhynchus mykiss [A0A060Y1Y5]
MAATAAWMHGPANMRLTEGLLSVLKEQRPEYLPALLANYREHGVVSTQSSAAVGGLVGLS
NAKLGNSKTRFEGLCLLSVLVKDSSSDVFQQHCLSWLRSLQQVIQSQAPLPSIQLAVGVL
QDLLQYSSQLPELAREVGLNSILGILTSLLGLKSEFHLAAMEGMTACMTFYPRACGSLRD
KLGACFLSKMDSVIPEVQEVACECYGRLPCLGGVLERGGGGRRAEGWTNQLHCLLASANG
MLAQLYHSTESEGMVPYEGPGVELPYPPLDDTDPLVLLQLQHRYRGVCLALKHTLGVDPA
SAVRLPVQQVLNLVCRALAVSSKSINVTGDGSVRLLVLPSIHNHTVKVLHALITAVGSGL
VQYSSMLQRLFSQTLSAWTPLPETNLGQQRAFSAVRVSLYRTLELWVKVGGASAGVLQGS
PTHSEILLAHLLGDITPGADSVRLRAGQSTVADLVSSKPCPKSRKPGLGMGNGGGASLQR
KGDSLANQDTCVSALRALRQIILTSGTLLKEDIHKRLHDVVLPLCVRLQQQHGGDCGAGG
VSGQYGSALPRRELYRLLLALVLVPPPRWPPPLTCTVSILSHGRRDRSLKVRGNMHAGTA
QAGTPPPPTHTHTHTQQHTXXGGRESE
> Gambusia affinis [A0A315V774]
MRLTEGLVSVLKERRPEYLSEVLANYREHGVFPTQGASDVAGLVGFSNAKLSSSKTRFEGLCLLSMLVKDSSSDLFQQHC

LSWLRSLQQVIQSQGPCQSIQLAVSILQDLLQYSCQLAELAREVGLNSILGILTSLLGLKTECELAAMEGMRACMVYYPR
ACGSLKMACQGYSLLPCLGGLADRSVGTGRAEGWTNQIQCLLASANGILAQIYQGSETDEAVQYRGPGVELPFPHLDQTD
PLLLLQLQHRYAAVCLVLKHTLRADPASAVHLPVRPVLNLVCRALAVNSRNTSLTADGSLRLLVLPSIHISTLEVLAELI
TVVRSGMVQYAAVLQKLFSQMLCAWTAVPEANVGQQRAYSSVRVSVYRTLDLWLQLVGASANVLQGASSHAELLFTHLLA
DITPGAESIKLRAGLSADVVPGGKPGPRRTKQLVMADPVGASLQRKGDPVSNQDTCLSALKVLRRIIQTSGTLLKNDIHK
RLHEVVLPLCVRLQQQTSSITACESIGGASGQYSSALTRRQLYRLLLALVLVPSPCWPPPLTCAVSIFSNGRLDRNLKV
SSFCSEALTVCNSLLHPRVPSIALPLPPLALKPPPTASVLSGPGPAPRLTLPTLLGGPAPGPPFPGRHPLSLGPNSLLGS
LENHLSLVPGMPGPGSGPGDLLLSPHGQPQPDPAGLGLPDGQRPVFVRYDKEEAEDVEISLASDSDDSVVIVPPGMLGAD
SQLDDPAPGSQNLLSAAAGAGVSLPGGDTVAMVPSTAASASLDVTSLPNDLVPTSSTPVNSFPSSSGSVVSLVTSLTPGS
APTPAGGLADPVVVKPQLQQMLLQPPAPGPQGPPGSVALPLQVQLQNQLAQQGRHLHQHPPPVPPSNQDSAVININSTDD
EDEDEEDMEDDEELEEEEDGLDEEEEEEGSEDFYEGEDYEDFDDEEAEELEEEEEDEDGDMPPLEGSEDKSEEVTMEEE
EEEEKALGAEEEEGEGAGFNPEGGIEELQSSRALFGEERMKVQEVESIGVLEEARXXXXGDRGDGEDDSERMDDPSMPQI
LCVTGGALEERADSSQEAAQAAGELQDQLTLWDQNHQQNPPTGASEEEPTAHRIQAFRLKFRPIPQESVAERQQEAGVRE
EVALEAELQEEDEPVVVQEEAAAGASGGEETPAAPHDPQQDQTDTQQGYEAQAEWTKPQGEKEEESNSEEEKGMKRKREE
EAIEGEAGPSPEKKKMIINYWSLSVLQQDDEAMASMLADFIACPPDDEDVASGSTQS
>Takifugu rubripes [A0A674PP05]
MENLCFLCVGGRFEGLCLLSMLVKDSSSDLFQQHCLSWLRSLQQIVQSQAPVQTIQLAVNILKDVLQYSSQIPELAREVG
LNSILGILTSLLGLKTECELAAMEGMTACMTHYPRACGSLKDKLGAYFLSKMDSTNKKTQEMACQCYAHLPCLGGVLDRG
AGAGRAEGWTNQIHCLLASANRFLLFLTEGTMQYEGPGVELAFPLLDQSDPLFLLQLQHRFTAVCLALRHTLRVDPASAV
RIPVRPILNLVCRALAVSCKSFNLTGDGNVRLLILPIIHLNILEVLAALIIAVRSSMVQYAAVLQRLFSQTLSAWTPAAE
ASVGQQRAFSSVRVSVYRTLELWLQVAGASTSILHGSPNHSEILFNHLLSDITPGAESVKLRVGLSAEIVPGGKPGPRRT
KSLVISDTVGPSLQRKGDIMANQDTCLTALRALRQIILVSGTLLKDDIHKVNICYHPCIGMLLLALVLVPSPCWPPPLTC
VVSILSSGRTDRNLKVSTFCCEALTVCNSLLHPRSPSIALPMPPLSIKPAHAVSVLPTPQASTPGLTLPTLLGEPTPPPP
FPSPHTLGMGPSSLLGSLENHLSLVPGLRGQTSGPSEMILSPHAHHQDLAGLGPPEGQRPVFVRYDREEAEDVEISLASD
SDDSVVIVPPGMLNMENQQDDVAAANSQSMTAAAGGAAVTLAEGEPVTMVPNTATAAPIDGVSLPNDLTTSAPLLTTSAP
PINSFPPSSASVVSLVPALNSNPLTAPPGGLVEPIPSRPQLQQMLMQPSAAVQQGPLSLPLQIHQLQSQLGQQGRPLQQQ
PPVASNEDSGVININSTDDEEDDEDMEDDEEEEEEEGVEDEEEEEDEVSSFADEEFFEDYEEFEYDAEELEEEDEEEGDI
PPLEGAEEQGEQEEVEQGGVLQAAVEAAEIVDFSVEGEAGGGIEEIQTKRALFPEDRMKVQEVESIGVMEEARGEAEEDE
TERVCDPTMPQILCVTGGALEEKEEEGGRVQEDMSSWERDAKKVEPQGPSGEAAASTAEQVGCG
> Danionella translucida [A0A553QMD7]
MASATWQHGPNITRLTEGLVSVLKEDRPEFLPTLLSNYREHGVVGNQVSGTVGGLVGIGNVKLGSSKTRFEGLCLLSVLV
KDSSSEVFQQHCLPWLRMLQQVIQSQAPLPTMQLAVSVLQDLLHYSSQLPELAREVALNSIPGILTSLLSLKYECHLAAM
KGMMACMTYYPRACGSLREKLGVYFLSKMESDNAKVQEVACECYGRLPSLGGVLERGGGGRKADGWTNQLHCLLASANSL
LGLLYQGLETEGTVVYDGPGLELPFPPLDEVDPLLILELHHRYKAVSLAIKHTLSIDPASAVRLPVQNVLNLVCRALAVN
TKSINPTGDGCLKLLVLPSIHIDTLEILSALIKAVGGGLVQYSCVLTRLFSQSLSAWMPPPEASLGQQRAHSGVRVSVYR
IIELWVRVGGASLLQGSPSHTELLFSHLMSDITPGSEAVKLRSEPHTSMSDLIGPAAKTGPRRTKGLVEGVSMQRKGDAV
ANQDTCQQSHCVQELGAIRGQYGSPSPRRELYRVLLALVLVPTPRWPPPLSCAVSTFSHGRRDRNIKVSSFCAEALTICN
TLIHPRTPSISLPLTPLTLKSTPAASGQNPALSVSTLLGAPASGPPFPARHTMGLGPASLLGSLENHLPITPQVLPNSVS
TTATPGELGLSPGHTGELGGLGAPEGQRQVFVRYDKEEPEDVEISLESDSDDSVVIMPAGMMEVQDGVGNAQPTPSTVGG
LQPSTPIISEVGSVDPSLTGELPSSIPHQILPANANNMNSFPGPSQTTQLVSLVPPLNTSATGSSAVLSESLSGGQQLQQ
MLMQTSAGGQSAALGLPFQIQLAQTGRQQPTGNDMDQNVININSSDDEDEEEEELEDEDELGEEEEEEEEGLDEEDDEEE
GSDLMEDEYGEEEFEDEDELEDEDDEESEEILPLEGDDPREIIGEEEADVMIEAEGQHGMELFCIEREGGIEEMEGVRSI
YSEDRNKEKGSLEEIENIGAVERNESILDKEQIETLVIGGDAEDADSTVEEVEPEVKSSEQEPKEPEDPVVEAGPSQQGH
GLTGGNEVQQKYELQREKSDLCPPDTSQQELAQNVDETVQGEKETAEKDVDVRGTKRKMEDREEGVSSEQGTEKKKIDDE
AMASMLADFVDCPPDDEDHGASQAQS
> Danionella translucida [A0A553QN20]
MASATWQHGPNITRLTEGLVSVLKEDRPEFLPTLLSNYREHGVVGNQVSGTVGGLVGIGNVKLGSSKTRFEGLCLLSVLV
KDSSSEVFQQHCLPWLRMLQQVIQSQAPLPTMQLAVSVLQDLLHYSSQLPELAREVALNSIPGILTSLLSLKYECHLAAM
KGMMACMTYYPRACGSLREKLGVYFLSKMESDNAKVQEVACECYGRLPSLGGVLERGGGGRKADGWTNQLHCLLASANSL
LGLLYQEGTVVYDGPGLELPFPPLDEVDPLLILELHHRYKAVSLAIKHTLSIDPASAVRLPVQNVLNLVCRALAVNTKSI
NPTGDGCLKLLVLPSIHIDTLEILSALIKAVGGGLVQYSCVLTRLFSQSLSAWMPPPEASLGQQRAHSGVRVSVYRIIEL
WVRVGGASLLQGSPSHTELLFSHLMSDITPGSEAVKLRSEPHTSMSDLIGPAAKTGPRRTKGLVEGVSMQRKGDAVANQD
TCQQSHCVQELGAIRGQYGSPSPRRELYRVLLALVLVPTPRWPPPLSCAVSTFSHGRRDRNIKVSSFCAEALTICNTLIH
PRTPSISLPLTPLTLKSTPAASGQNPALSVSTLLGAPASGPPFPARHTMGLGPASLLGSLENHLPITPQVLPNSVSTTAT
PGELGLSPGHTGELGGLGAPEGQRQVFVRYDKEEPEDVEISLESDSDDSVVIMPAGMMEVQDGVGNAQPTPSTVGGLQPS
TPIISEVGSVDPSLTGELPSSIPHQILPANANNMNSFPGPSQTTQLVSLVPPLNTSATGSSAVLSESLSGGQQLQQMLMQ
TSAGGQSAALGLPFQIQLAQTGRQQPTGNDMDQNVININSSDDEDEEEEELEDEDELGEEEEEEEEGLDEEDDEEEGSDL
MEDEYGEEEFEDEDELEDEDDEESEEILPLEGDDPREIIGEEEADVMIEAEGQHGMELFCIEREGGIEEMEGVRSIYSED
RNKEKGSLEEIENIGAVERNESILDKEQIETLVIGGDAEDADSTVEEVEPEVKSSEQEPKEPEDPVVEAGPSQQGHGLTG
GNEVQQKYELQREKSDLCPPDTSQQELAQNVDETVQGEKETAEKDVDVRGTKRKMEDREEGVSSEQGTEKKKIDDEAMAS
MLADFVDCPPDDEDHGASQAQS
>Periophthalmus magnuspinnatus [A0A3B3Z7D9]
MDSFVVLLHIWGLGSESLMGFSNAKLGSSKTRFEGLCLLSMLVKDSSSEVFQQHCLSWLRSLQQVIQSQAPVQTIQLAVN
ILKDVLQYSSQIAELSREVGLNSILGILTSLLGLKAECDLAAMEGMTACMNYYPRACGSMRVSKSTSYFLSKMDSINKKT
QEMACQCYGRLPCLGGLLDRGMGGGRAEGWTTQIHCLLASANVLLDQIYQGSEMGTIQYEGPGVELAFPLLDQTDPLLVL
QLQHRYTAICLALKHTLRYVDPASAVRLPVRPILNLVCRALAVSSKSIVRTQLMLYSVRVAVYKALELWLQVAGASANIL
QGSSVHSEHLFNHLLGEITPGAEAIKTLVMSDAVAPMLQRKGDQLANVDTCISALRGEMPSKIILSSGTLLKDDIHKRLH
DVVLPLCVRLQQQQNSSNLCESVGGISGQYNSALSRHELYRLLLALVLVPSPCWPSPLTCTVSILNTAQSQESLLFQEVS
SFCTEALTICNLLLHPRTPSIALPLPPLTLKPTPSATGQAPSAGDLMHPPHPDSAQLGPPDGQRPVFVRYDKEEAEDVEI
SLGSDSDDSVVIVPPGMLDMNNQQEESLPTPGATSVPLPGGDAVSMATTTSAPTTIDGMSLANDLTTSSPHLTSNTTSVN
SFPPSNVSVVSLVPPMGPNTLPGPGTLAEPLSGRPQLQQMLMQPSTAAPMGLLHQLQNQLTQPSRTLQHPPPPPAANNED
SAVININSTDEEEEEEDEEDMDDMEDEDEEGLDEEEEEGSEYAGEEFYDGEDYDEYDEEEPEELEEEEEEEDEDGDIPPL
EGSEDKANESGSEEVKAVQALAEEGPIEEPFSVEAETEGGIEEVQTRAYPELKVQEVESIGVLEEARESEGESTMDPTMP
> Periophthalmus magnuspinnatus [A0A3B3Z815]
SQGASAVGGLMGFSNAKLGSSKTRFEGLCLLSMLVKDSSSEVFQQHCLSWLRSLQQVIQSQAPVQTIQLAVNILKDVLQY
SSQIAELSREVGLNSILGILTSLLGLKAECDLAAMEGMTACMNYYPRACGSMRVSKSTSYFLSKMDSINKKTQEMACQCY
GRLPCLGGLLDRGMGGGRAEGWTTQIHCLLASANVLLDQIYQGSEMGDGSVRLLVLPIIHTNTLEVLSALITASVRVAVY

KALELWLQVAGASANILQGSSVHSEHLFNHLLGEITPGAEAIKLRAGLSADVVPGGKPGPRRTKTLVMSDAVAPMLQRKG
DQLANVDTCISALRGEMPSKIILSSGTLLKDDIHKRLHDVVLPLCVRLQQQQNSSNLCESVGGISGQYNSALSRHELYRL
LLALVLVPSPCWPSPLTCTVSILSRGRTDRNTKVSSFCTEALTICNLLLHPRTPSIALPLPPLTLKPTPSATGIQSAQGP
NPGLTLPTLLVGANSAPSFPSRHSLGLGHGSLLGSLENHLSMVPGLPGQAPSAGDLMHPPHPDSAQLGPPDGQRPVFVRY
DKEEAEDVEISLGSDSDDSVVIVPPGMLDMNNQQEESLPTPGATSVPLPVVSLLQNQLTQPSRTLQHPPPPPAANNEDSA
VININSTDEEEEEEDEEDMDDMEDEDEEGLDEEEEEGSEYAGEEFYDGEDYDEYDEEEPEELEEEEEEEDEDGDIPPLEG
SEDKANESGSEEVKAVQALAEEGPIEEPFSVEAETEGGIEEVQTRAYPELKVQEVESIGVLEEARESEGESTMDPTMPQI
LCVTGGAMEEEETTEGGETSQEEAATWEQGEDNKVEIEDSSDEQVPIGNQQASILHIRKRLK
> Callorhinchus milii [A0A4W3JEA4]
PPPLSGHLSPAENNNKLCAIGLCLLAQLVADSPTEIFQDQCISWLRAVQHIVQAQDPLPTVEMAVWVLRELLRYSSQLPE
LGREVAMNHIPSLVSSLLSLKPEVSHYTSNTICLTLSLSLSLSNCLACHCYALLPGLGTGFAQGMKYSESWSQQLHCLLS
SLHRLTEEIYQGSETEPSRYEGPGVDLPLPTLNDTDPYHVLHLSQRFTALSKCLAQLLRYQLPVTARHCLSVSLSLSLTR
SRYLTQEGFLSAVKVLVLPSLHTDALDILSALITTCGKRLLRFADVICRLFPQVLTAWATPRGSTPPGQERGHSGVRVTV
YTVMETWLKVAGVASGVLQGAAHHPDILLSHLLSDVTPPSHTLQVNTSPVRGHHKQDLTANSDVCLAALRGTAPTWLFVW
QKLQEMVLSLLMGLGRRSGEPSPYLRAQCRLELHRLLLFLLLVPNPHRPPPLHCAINTFSLGQQDSSTEVSAFCWEALVT
CNALIHPRVASLQSPLNLPLKLPTDSHPAPPPSKPHPELPGPARFTLALGAGPGQEEEELEAEPVAGGLEEE
> Japanese medaka HSOK [A0A3P9HWU3]
MEIRIILFSTYFTDMGGGGGVMALYNHPTLYLLFELCCGRDCVNFPSSLCLRSLTASLQPPHTPTLTLAAAASGLSIQND
VAGLVGFSNAKLSSSKTRFEGLCLLSVLVKDSSSDLFQQHCLFWLRSLQQIIQSQAPVQTVQLAVNILKDLLQYSSHLAE
LAREVGLNSILGILTSLLGLKTECELAAMEGMVACMLFYPRACGSLKDKLGAYFLSKMDSNNMKTQEMACLCYSHLPCLG
GLLDRAISAGKADGWTNQIHCLLATADALLTQIYKGSEPGNLYGGFLVHTCIYFTFLCWSVCELRKVFFFLSSSVRVSVY
KTLELWVKVVGASVSILQENPMHADLLFSNLLGDITPGPESIKFRAGLGVDVVPGGKPGPRRTKHLVIADPMGPSLQRKG
DPLSNQDTCLAALRDHQSRLLVVHWGSSIAEVVMTTELSSDKRCRKHVGSVCVTPRLLLALVLVPSPSWPPPLTCAVSIF
SNGRTDRNLKVSSFCAEALTICNTMVHPRTPSIALPLPPPHPQTPHLCPGALPSGVGSQANPADPPGGSRSLENHLSTVP
GLPAQGPTPGDMFSPHRQHDSGLGLPESQRPVFVRFDKEEPDDVEISLASDSDDSVVIVPPGMINLDQQQDEAAANVQTM
LSATPAGGRCGDSAIININSTDDEDEEEDLEDDEELEEEEEEDGLDEEDEEEGSEDFYDGEEYEDYDEEEGEELEEEEEE
EEEIGDMPPLEGSEEKLVDAAIEEGQVLHPAADDKEPSQHHAEADSERGIQELQPQRTSERMDDPTMPQIICVSGGAQES
VESGDEGGGTTSVLQEDRSRQAAKEEELEAAAEEQAGFRAEVRAAAETTACRRDG
> Monterrey platyfish [A0A3B5L4I5]
IQLAVSILQDLLQYSCQLAELAREVGLNSILGILMSLLGLKTECERAAMEGMRACMVYYPRACGSLKDKLGAYFLSKMDS
TSRKTQEMACQGYSLLPCLGGLADRSAGTGRAEGWTNQIQCLLASANGILAQIYQGSETGRFFWPVMCSIFHVSLTADGS
LRLLVLPSVHISTLEVLAELITNVFSISHIFRDCCVANSLVWLSELQVRCELAVSNSWTVFCSSVRVSVYRTLELWLQLV
GASANVLQGASSHAELLFTHLLADITPGAESIKLRAGLSADVVPGGKPGPRRTKQLVMADPVGASLQRKGDPVSNQDTCL
SALKGQLLPLFPEFSLLKPLPVLTCRLLLALVLVPSPCWPPPLTCTVSILSNGRLDRNLKVSSFCSEALTICNSLLHPRV
PSIALPLPPLALKPPPTASVLSGPGPAPRLTLPTLLGGPAPGPPFPGRHPLSLGPNSLLGSLENHLSLVPGMPGPASAPG
DLLLSPHGQPQPDPAGLGLPDGQRPVFVRYDKEEAEDVEISLASDSDDSVVIVPPGMLGAGSQLDDPAPGSQNLLSAGAG
MLLQPPAQGPPGSVALPLQVQLQNQLAQQGRHLHQHPPPAPPSNQDSAVININSTDDEDEDEEDMEDDEELEEEEEEDGL
DEEEEGSEDFYEGEEYEDFDDEEAEELEEEEEDEDGDMPPLEGSEDKSEEVTMEEEEEEEEEEKALGAEEEEGEGAGFNP
EGGIEELQSSRALFGEERMKVQEVESIGVLEEARGGDRGDGEDDSERMDDPSMPQILCVTGGALEERADSSQEAAQAAGE
LQDQLTLWDQNHQQNPPTGASEEEPTAHSQEVGSRPGPSQGHGMLFTFLLRVVALVTALL
> Pan troglodytes [A0A2J8KQT4]
MELAVAVLRDLLRYAAQLPALFRDISMNHLPGLLTSLLGLRPECEQSALEGMKACMTYFPRACGSLKGKLASFFLSRVDA
LSPQLQQLACECYSRLPSLGAGFSQGLKHTESWEQELHSLLASLHTLLGALYEGAETAPVQNEGPGVEMLLSSEDGDAHV
LLQLRQRFSGLARCLGLMLSSEFGAPVSVPVQEILDFICRTLSVSSKNISLHGDGPLRLLLLPSIHLEALDLLSALILAC
GSRLLRFGILIGRLLPQVLNSWSIGRDSLSPGQERPYSTVRTKVYAILELWVQVCGASAGMLQGGASGEALLTHLLSDIS
PPADALKLRSPRGSPDGSLQTGKPSAPKKLKLDVGEAMAPPSHRKGDSNANSDVCAAALRGLSRTILMCGPLIKEETHRR
LHDLVLPLVMGVQQGEVLGSSPYTSSRCRRELYCLLLALLLAPSPRCPPPLACALQAFSLGQREDSLEVSSFCSEALVTC
AALTHPRVPPLQPMGPTCPTPAPVPPPEAPSPFRAPPFHPPGPMPSVGSMPSAGPMPSAGPMPSAGPVSSARPGPPTTAN
HLGLSVPGLVSVPPRLLPGPENHRAGSNEDPILAPSGTPPPTIPPDETFGGRVPRPAFVHYDKEEASDVEISLESDSDDS
VVIVPEGLPPLPPPPPSGATPPPIAPTGPPTASPPVPAKEEPEELPAAPGPLPPPPPPPPPVPGPVTLPPPQLVPEGTPG
GGGPPALEEDLTVININSSDEEEEEEEEEEEEEEEEEEEEDFEEEEEDEEEYFEEEEEEEEEFEEDFEEEEGELEEEEEE
EDDEEEELEDVEDLEFGTAGGEVEEGAPPPPTLPPALPPPESPPKVQPEPEPEPGLLLEVEEPGTEEERGADTAPTLAPE
ALPSQGEVEREGESPAAGPPPQELVEEEPSAPPTLLEEETEDGSDKVQPPPETPAEEEMETETEAEALQEKEQDDTAAML
ADFIDCPPDDEKPPPPTEPDS
> Pongo abelii [A0A2J8SPL2]
MELAVAVLRDLLRYAAQLPALFRDISMNHLPGLLTSLLGLRPECEQSALEGMKACMTYFPRACGSLKGKLASFFLSRVDA
LSPQLQQLACECYSRLPSLGAGFSQGLKHTESWEQELHSLLASLHTLLGALYEGAETAPVQNEGPGVEMLLSSEDGDAHV
LLQLRQRFSGLARCLGLMLSSEFGAPVSVPVQEILDFICRTLSVSSKNISLHGDGPLRLLLLPSIHLEALDLLSALILAC
GSRLLRFGILISRLLPQVLNSWSIGRDSLSPGQERPYSTVRTKVYAILELWVQVCGASAGMLQGGASGEALLTHLLSDIS
PPADALKLRSPRGSPDGSLQTGKPSAPKKLKLDVGEAMAPPSHRKGDSNANSDVCAAALRGLSRTILMCGPLIKEETHRR
LHDLVLPLVMGVQQGEVLGSSPYTSSRCRRELYCLLLALLLAPSPRCPPPLACALQAFSLGQREDSLEVSSFCSEALVTC
AALTHPRVPPLQPMGPTGPTPAPAPPPEAPSPFRAPPFHPPGPMPSAGPMPSAGPMPSAGPVPSARPGPPTTANHLGLSV
PGLVSVPPRLLPGPENHRAGSNEDPILAPSGTPPPTIPPDETFGGRVPRPAFVHYDKEEASDVEISLESDSDDSVVIVPE
GLPPLPPPPPSGATPPPIAPTGPPTASPPVPAKEEPEELPAAPGPLPPPPPPPPPVPGPVTLPPPQLVPEGTPGGGGPPA
LEEDLTVININSSDEEEEEEEEEEEEEEEEEEEEEDFEEEEEDEEEYFEEEEEEEEEFEEEFEEEEGELEEEEEEEDEEE
EEELEEVEDLEFGTAGGEVEEGGPPPPTLPPTLPPPESPPKVQSEPEPEPGLLLEVEEPGTEEEHGADTAPTLAPEVLPS
QGEVEREGESPAAGPPPQELVEEEPSAPPTLLEEETEDGSDKVQPPPETPAEEEMETETEAEAHQEKEQDDTAAMLADFI
DCPPDDEKPLPPTEPDS
> Pan troglodytes [A0A2J8KQV5]
MELAVAVLRDLLRYAAQLPALFRDISMNHLPGLLTSLLGLRPECEQSALEGMKACMTYFPRACGSLKGKLASFFLSRVDA
LSPQLQQLACECYSRLPSLGAGFSQGLKHTESWEQELHSLLASLHTLLGALYEGAETAPVQNEGPGVEMLLSSEDGDAHV
LLQLRQRFSGLARCLGLMLSSEFGAPVSVPVQEILDFICRTLSVSSKNISLHGDGPLRLLLLPSIHLEALDLLSALILAC
GSRLLRFGILIGRLLPQVLNSWSIGRDSLSPGQERPYSTVRTKVYAILELWVQVCGASAGMLQGGASGEALLTHLLSDIS
PPADALKLRSPRGSPDGSLQTGKPSAPKKLKLDVGEAMAPPSHRKGDSNANSDVCAAALRGLSRTILMCGPLIKEETHRR
LHDLVLPLVMGVQQGEVLGSSPYTSSRCRRELYCLLLALLLAPSPRCPPPLACALQAFSLGQREDSLEVSSFCSEALVTC
AALTHPRVPPLQPMGPTCPTPAPVPPPEAPSPFRAPPFHPPGPMPSVGSMPSAGPMPSAGPMPSAGPVSSARPGPPTTAN
HLGLSVPGLVSVPPRLLPGPENHRAGSNEDPILAPSGTPPPTIPPDETFGGRVPRPAFVHYDKEEASDVEISLESDSDDS

VVIVPEGLPPLPPPPPSGATPPPIAPTGPPTASPPVPAKEEPEELPAAPGPLPPPPPPPPPVPGPVTLPPPQLVPEGTPG
GGGPPALEEDLTVININSSDEEEEEEEEEEEEEEEEEEEEDFEEEEEDEEEYFEEEEEEEEEFEEDFEEEEGELEEEEEE
EDDEEEELEDVEREGESPAAGPPPQELVEEEPSAPPTLLEEETEDGSDKVQPPPETPAEEEMETETEAEALQEKEQDDTA
AMLADFIDCPPDDEKPPPPTEPDS
> Pongo abelii [A0A2J8SPN5]
MELAVAVLRDLLRYAAQLPALFRDISMNHLPGLLTSLLGLRPECEQSALEGMKACMTYFPRACGSLKGKLASFFLSRVDA
LSPQLQQLACECYSRLPSLGAGFSQGLKHTESWEQELHSLLASLHTLLGALYEGAETAPVQNEGPGVEMLLSSEDGDAHV
LLQLRQRFSGLARCLGLMLSSEFGAPVSVPVQEILDFICRTLSVSSKNISLHGDGPLRLLLLPSIHLEALDLLSALILAC
GSRLLRFGILISRLLPQVLNSWSIGRDSLSPGQERPYSTVRTKVYAILELWVQVCGASAGMLQGGASGEALLTHLLSDIS
PPADALKLRSPRGSPDGSLQTGKPSAPKKLKLDVGEAMAPPSHRKGDSNANSDVCAAALRGLSRTILMCGPLIKEETHRR
LHDLVLPLVMGVQQGEVLGSSPYTSSRCRRELYCLLLALLLAPSPRCPPPLACALQAFSLGQREDSLEVSSFCSEALVTC
AALTHPRVPPLQPMGPTGPTPAPAPPPEAPSPFRAPPFHPPGPMPSAGPMPSAGPMPSAGPVPSARPGPPTTANHLGLSV
PGLVSVPPRLLPGPENHRAGSNEDPILAPSGTPPPTIPPDETFGGRVPRPAFVHYDKEEASDVEISLESDSDDSVVIVPE
GLPPLPPPPPSGATPPPIAPTGPPTASPPVPAKEEPEELPAAPGPLPPPPPPPPPPVPGPVTLPPPQLVPEGTPGGGGPPA
LEEDLTVININSSDEEEEEEEEEEEEEEEEEEEEEDFEEEEEDEEEYFEEEEEEEEEFEEEFEEEEGELEEEEEEEEDEEE
EEELEEVEREGESPAAGPPPQELVEEEPSAPPTLLEEETEDGSDKVQPPPETPAEEEMETETEAEAHQEKEQDDTAAMLA
DFIDCPPDDEKPLPPTEPDS
> Lynx pardinus [A0A485MPI6]
MAAAVLSGPSAGSAAGVPGGTGGLSAVGSGPRLRLLLLESVSGLLQPRAGSAVAPVHPPVRSAAHLPGLMCLLRLHGTVG
GAQNLSAVGALVGLSNARLGSVKTRFEGLCLLSLLVGESPTELFQQHCVSWLRSIQQVLQSQDPPPTMELAVAVLRDLLR
YAAQLPTLFRDISTNHLPGLLTSLLGLRPECELSAMEGMKACMTYFPRACGSLKGKLASFFLSRVDALSPQLQQLACECY
ARLPSLGAGFSQGLKHTESWEQELHSLLASLHGLLGALYEGADTAPVQCEGPGLDVLLAPSEDGDAHTLLRLRHRFSGLA
RCLGLLLRCLRSPRGSPDGGLQSGKPSAPKKLKLDMGEATAPPGHRKGDSNANSDVCAAALRGLSRTVLMCGPLIKEETH
RRLHDLVLPLVMGVQQGEVLGSSPYTSSRCRRELYRLLLALLLAPSPRCPPPLACALQAFSLGQREDSLEVSSFCSEALV
TCAALTHPRVPPLQSMGPACPAPAPAPPPEAPSPFRAPPFHPPGPMPSVGPMPSVGPMPSVGPMPPAGPMPPTRPGPPAT
ANHLGLSVPGLVSVPPRLLPGPENHRAGSNDDPVLAPSGTPPPAVPPDETFGGRVPRPAFVHYDKEEASDVEISLESDSD
DSVVIVPEGLPPLPPPPAAPAGPPTASPPVPAKEEPEELPAAPGPLPPPPPPPVPGPVALPPPQLVPEGPPGGGGPPALE
EDLTVININSSDEEEEEEEEEEEEEEEEEEEEEDFEEEEEEEEEYFEEEEEEEEEFEEEFEEEEGELEEEEEDEDEEEED
ELEELEEVEFGPAGGPAEEGGPPPPSPAPALPPAQPPEAPPEPGVEPGLLLEVEEPGPEDEPGAEAAPTLAPEVLPSQGE
GQREAGSPPAGPPPRELVEEEPSAPPPLLEEGTENGGDKVPPPPETPAAEEMEAAXXXXXXXXXXXXXXXXXXXXXRSSM
TRLPCWLTSSIVPLMTRSPQRPPSLSPSLPLRPPLLFPIKLCLRLSTAASFAFYTTVSPSRSPAVHWAPQGCP
> Crocuta Crocuta [A0A6G1AA45]
QNLSAVGALVGLSNARLGSIKTRFEGLCLLSLLVGESPTELFQQHCVSWLRSIQQVLQSQDPPPTMELAVAVLTDLLRYA
AQLPALFRDISMNHLPGLLTSLLGLRPECELPAMEGMKACMTYFPRACGSLKGKLASFFLSRVDALSPQLQQLACECYSR
LPSLGAGFSQGLKHTESWEQELRGLLASLHGLLGALYEGADTAPVPYEGPGVDVLLAPSEDGDAHTLLRLRHRFSGLARC
LGLLLSSEFGAPVSVPVQEILDVICRTLSISAKNISLLGDGPLRLLLLPSVHLDALDLLSALILACGGRLLRFGALISRL
LPQVLSAWNLGRDALPPGQERPYSAVRTKVYAVLDLWVQVCGASAGVLQGGASGEALLSHLLSDISPPADTLKLRSPRGS
PDGGLQSGKPSAPKKLKLDMGEAMAPPGHRKGDSNANSDVCAAALRGLSRTILMCGPLIKEETHRRLHDLVLPLVMGLQQ
GEALGSSPYASARCRRELYRLLLALLLAPSPRCPPPLACALQAFSLGQREDSLEVSAAGPGRLHAVGRDRPVSGARTPAF
PMSQPPPPPSAEAPSPSRAPPFHPPGPMPSVGPMPSVGPMPPTRPGPPATANHLGLSVPGLVSVPPRLLPGPENHRAGSN
DEPVLAPSSTPPPAVPADETFGGRVPRPA
> Sus scrofa [A0A480LV57]
MAAAVLSGPSAGSAAGVPGGTGGLSAVVSVPRLRLLLLDSVSGLLQPRAGSTVAPVHPPA
PSAPHLPGLMCLLRLHGTVGGAPNLSAVGALVGLSNARLGSIKTRFEGLCLLSLLVGESP
TEMFQQHCVSWLRSIQQVLQSQDPPPTMELAVAVLKDLLRYAAQLPTLFRDISMNHLPGL
LTSLLGLRPECELSALEGMKACMTYFPRACGSLKGKLASFFLSRVDALSPQLQQLACECY
SRLPSLGAGFSQGLKHTESWEQELHSLLASLHGLLGALYEGAETAPMQYEGPAVEALLSP
SEDGDAHVLLRLRQRFSGLARCLGLLLSSEFGAPVSVPVQEVLDVICRTLSVSARNISLL
GDGPLRLLLLPSLHLEALDLLSALILACGGRLLRFGALISRLLPQVLSAWSIGRDTLSPG
QERPYSTMRTKVYAVLELWVQVCGASAGVLQGGASGEALLTHLLSDISPPADALKVSEAA
APVPWPHPVPLVLTNLVTRCCPDCPAFLWACVWLMFPSPPAAQSPWEP
> Monodon monoceros [A0A4U1F0W7]
LLDSGACSPDHVFFNCHWSGLPPSTQPTDQALTPRELNTHSWVAPALPRRACSLPAGERTAKRRTKAPSSSPSPRGVRRG
VFASSRHATSRARGKMAAAVLSGPSAGSAAGVPGGTGGLSAVNSGPRLRLLLLESVSGLLQPRAGSTVSPVHHPTRSVPH
LPGLMCLLRLHGTVGGAQNLSAVGALVGLSNARLGSIKTRFEGLCLLSLLVGESSTEMFQQHCVSWLRSIQQVLQDPPPT
MELAVAVLGDLLRYAAQLPTLFRDISMNHLPGLLTSLLGLRPESELSALEGMKACMTYFPRACGSLKGKLASFFLSRVDA
LSPQLQQLACECYSRLPSLGAGFSQGLKHTDSWEQELHSLLASLHSLLGALYAGADTAPMQYEGPGVETLLSPSEDGDAH
VLLRLWQRFSGLARCLGLMLSSEFGAPVSVPVQEILDLICRTLSVSAKNISLLGDGPLRLLLLPSLHLEALDLLSALILA
CRGRLLRFGALISRLLPQVLNAWSIGRDSLSPGQERPYSTMRTKVYAVLELWVQVCGASAGVLQGGASGEALLTHLLSDI
SPPADALKLRSPRGSPDGGLQTGKPSAPKKLKLDMGEALAPPSHRKGDSNANSDVCAAALRGLSRTVLMCGPLIKEETHR
RLHDLVLPLVMGVQQGEVLGSSPYTSSRCRRELYRLLLALLLAPSPRCPPPLACALQAFSLGQREDSLEVSAFCSEALVT
CAALTHPRVPPLQTMGPACPTPAPVPPPEAPSPFRAPAFHPPGPMPSVGPMPSVGPMPSAGPMPSPGPVPPAGPMPSVGP
MPPARPGPPATANHLGLSVPGLVSVSPRLLPGPENHRAGSNEDAVLAPSGTPPPTIPPDETFGGRVPRPAFVHYDKEEAS
DVEISLESDSDDSVVIVPEGLPPLPPPPPSGTTPPPAALVGPPTASPPVPAKEEPEELPAAPGPLPPPPPPPVPGPVALP
PPQLVPEGTPSGGGPPALEEDLTVININSSEEEEEDDEEDEDEEDFEEEEEEEEEEEEYFEEEEEEEEEFEEEFEEEEGE
LEEEEEDEDEEEEVEEVEFGPAGGEVEEGGPAPPSLPPALPPAASPKVQPQPEPEPGLLLEVEEPGAEEGPGAETAPTLA
PEVLPSQGEVEREGGSPPAVPPPQELVEEEPSVPPTLLEEGAEGGGDKVPPPPEAAAAEEMEAEAAALQEKEQDDTAAML
ADFIDCPPDDEKPPPATEPDS
> Gallirallus okinawae [A0A6G1RDW7]
PSRRALPELVRGLRDSAGTQSAQAIGGLLGVTSARLGSMKTRFEGLCLLSLLVSESPTEPFQQHCLGWLRCLQHLLQSQD
PAPTMALGVAVLHQLLLFSAQLPELARDIGTNHIPGLLTSLLGLKPECEVSTLEGIKSCMTFYPRACGSMRGKLAAYFLS
RIDSESPQLQQLACECYALLPALGRGFSQGLRHTECWHQELRGLLGTLHRVLGGLLGGSQNEPLPYEGPGVEMLLPPPQD
GDTGLLLTLHTRFSGLARVLRLLLSKDLVAPVTVPVQDILDLVCRALNITSKNLSWFGDGPLKMLLLPSVHMDMLDVLAA
LILACGARLVRWGSVLGRLFPQVLSAWSSARDPPPPGQERPFGAVRTRLYEVLELWVQVAGAASGVLQGAGTPPEVLLTH
LLNDITPPADNVKLKAEPKPSAPKRPKLGDGGDAPPLHRKLEPAANSDVCRAALRALRCAILTGGPLIKEETHRRLQELV
VPLLLRLPQTDARSE
>Xenopus laevis [A0A1L8H380]

MAAVCVGTRGMEVTIAGILERDLSEGELAEAIRGLREHGAFRGEGLPAAMSGLLSSCNSR
LTSASSRIEGLSLLALAVEESPTDVFVQHCVSWLRSLLQIIQSQDPPRVVSLAVFVLRSL
LAHSSALPELSREISTNHIPGLLTSLLGLRRQCLVPALEGIRSCFLSYPRACGSLRGKLT
AFLLSLLDAENQQIQEVACQCYSLLPSLGSGFSQGIKHTENWERQIQSVICSLHSVFLQL
YQGSETDTARYEGSGTELEFPSVEDDGTHGVLQLARRFTALGQCMRLLLREQFPAPVRVP
VSDILSLVCRVVNVSPKNLSWHGEESLKLLLLPRVHSSILEILEATIIACGPRLLPFSAV
ICRLFPQLLLSWAAVKGITGIPSGQERPYSSLRCSVYRVLETWVTTCGISSGVLQGPMHH
SDILLANLLSDITPPTDAIKMSTFVQLGAKKQKVSEVGDDDFQSHRKRDNTANVELCAAA
LKGLCCVILHCGSVIKEDVHRRLQELSIPLLLRLQQGSDQWLGPYISSDCRKELYRLLLC
LTLTPNPKLPAPLHCAIRIFRGGTTEESLQVSRFSTEALAICRILIHPRVPSLQRPLPHL
APRPPVQSDAPTLRPPAALSTFPAMPPANHLPPRPTVPAMSTEPPIPAAVPSPPPEESFG
EKPRRAVFIHFDKEEPSDVEISLESDSDDSVVIVPEGLFAKSDSKPEPSPPAVKPPTEEV
TEQVAPSAVPSSSTAAPPPPPPPPPAPPVCAGPSSAPVPIAEAPPPPQQEVDTVININSSD
DEEDGEEDEEEGLYDDEDEEDYYDEEEDEDLEGLEEDDYEEDEEGITEEEEEDLEEEGED
DEEEVEDEECLMPDEMQIGSEAEEIPDGIETSSLHEGELEEGPPRLSPVQEDEAVDTGLL
MLVESEDREPSGEAPGEGLPESDLTRSPPQPPVLTPPSPPDEPPPMEESDVPPLEEPDLE
VAPVAEEPVEETEEKKPEEVTVEKPEPEPEEEEQIADADAMLADFVDCPPDDDKLPEPCT
> Canis_lupus [P052059]
MAAAVLSGPSAGSAAGVPGGAGGLSAVGSGPRLRLLLLESVSGLLQPRAGSAVAPVHPPVRSAPHLPGLMCLLRLHGTVGGAQNLSAVGALVGL
SNARLGSIKTRFEGLCLLSLLVGESPTEMFQQHCVSWLRSIQQVLQSQDPPPTMELAVAVLRDLLRYAAQLPTLFRDISMNHLPGLLTSLLGLR
PECELSALEGMKACMTYFPRACGSLKGKLASFFLSRVDALSPQLQQLACECYSRLPSLGAGFSQGLKHTESWEQELHSLLASLHSLLGALYEGA
ETAPVQYEGPGVEVLLTPSEDGDTHVLLRLRQRFSGLARCLGLMLSSEFGAPVSVPVQEILDVICRTLSISAKNISLLGDGPLRLLLLPSIHLD
ALDLLSALILACRSRLLRFGALISRLLPQVLNAWNLGRDTLAPGQERPYSTVRTKVYAVLELWVQVCGASAGMLQGGSSGEALLSHLLSDISPP
ADTLKLRSPRGSPDGGLQTGKPSAPKKLKLDVGEAMAPPSHRKGDSNANSDVCTAALRGLSRTILMCGPLIKEETHRRLHDLVLPLVMGVQQGE
VLGSSPYTSSRCRQELYRLLLALLLAPSPRCPPPLACALQAFSLGQREDSLEVSSFCSEALVTCAALTHPRVPSLRSMGPACPTPAPAPPPEAP
SPFRAPPFHPPGPMPSAGPMPSVGPMPSVGPMPPAGPMPPAGPMAPTRPGPPATANHLGLSVPGLVSVPPRLIPGPENHRAGSNEDPVLAPSGT
PPPAIPPDETFGGRVPRPAFVHYDKEEASDVEISLESDSDDSVVIVPEGLPPLPPPPPSGTTPPPVAPAGPPVASPPVPAKEEPEELPVAPGPL
PPPPPPPVPGPVALPPPQLVPEATPGGGGPPALEEDLTVININSS
> Balaena_mysticetus [P014217]
MAAAVLSGPSAGSAAGVPGGTGGLSAVNSGPRLRLLLLESVSGLLQPRAGSTVSPVHPPVRSVPHLPGLMCLLRLHGTVGGAQNLSAVGALVGL
SNARLGSIKTRFEGLCLLSLLVGESPTEMFQQHCVSWLRSIQQVLQSQDPPPTMELAVAVLRDLLRYAAQLPTLFRDISMNHLPGLLTSLLGLR
PESELSALEGMKACMTYFPRACGSLKGKLASFFLSRVDALSPQLQQLACECYARLPSLGAGFSQGLKHTDSWEHELHSLLASLHGLLGALYEGA
ETVPVQYEGPGVETLLSPSEDGDAHVLLRLWQRFSGLARCLGLMLRCVSVGGSEGLEQEGLRWVSSSSRGGRLRVGVESPWGPRAEDLPALTLF
SSEFGAPVSVPVQEILDLICRTLSVSAKNISLLGDGPLRLLLLPSLHLEALDLLSALILACGGRLLRFGALISRLLPQVLNAWSIGRDSLSPGQ
ERPYSTMRTKVYAVLELWVQVCGASAGVLQGGASGEALLTHLLSDISPPADALKLRSPRGSPDGGLQTGKPSAPKKLKLDMGEAMAPPSHRKGD
SNANSDRLHDLVLPLVMGVQQGEVLGSSPYTSSRCRRELYRLLLALLLAPSPRCPPPLACALQAFSLGQREDSLEVSAFCSEALVTCAALTHPR
VPPLQTMGPACPTPAPVPPPEAPSPFRAPAFHPPGPMPSVGPMPSVGPMPSAGPMPSPGPVPPAGPMPSVGPMPPARPGPPATANHLGLSVPGL
VSVSPRLLPGPENHRAGSNEDAVLAPSGTPPPTIPPDETFGGRVPRPAFVHYDKEEASDVEISLESDSDDSVVIVPEGLPPLPPPPPSGTTPPP
AAPVGPPTASPPVPAKEEPEELPAAPGPLPPPPPPPVLGPVALPPPQLVPEGTPSGGGPPALEEDLTVININSS
> Mus_musculus [P022087]
MAAAVLSGASAGSPAGAPGGPGGLSAVGSGPRLRLLLLESISGLLQPRTASPVAPVHPPIQWAPHLPGLMCLLRLHGTAGGAQNLSALGALVNL
SNAHLGSIKTRFEGLCLLSLLIGESPTELFQQHCVSWLRSIQQVLQSQDSPSTMELAVAVLRDLLRHASQLPTLFRDISTNHLPGLLTSLLGLR
PECEQSALEGMKACVTYFPRACGSLKGKLASFFLSRLDSLNPQLQQLACECYSRLPSLGAGFSQGLKHTENWEQELHSLLTSLHSLLGSLFEET
EPAPVQSEGPGIEMLLSHSEDGNTHVLLQLRQRFSGLARCLGLMLSSEFGAPVSVPVQEILDLICRILGISSKNINLLGDGPLRLLLLPSLHLE
ALDLLSALILACGSRLLRFGALISRLLPQVLNAWSTGRDTLAPGQERPYSTIRTKVYAILELWVQVCGASAGMLQGGASGEALLTHLLSDISPP
ADALKLCSTRGSSDGGLQSGKPSAPKKLKLDMGEALAPPSQRKGDRNANSDVCAAALRGLSRTILMCGPLIKEETHRRLHDLVLPLVMSVQQGE
VLGSSPYNSSCCRLGLYRLLLALLLAPSPRCPPPLACALKAFSLGQWEDSLEVSSFCSEALVTCAALTHPRVPPLQSSGPACPTPAPVPPPEAP
SSFRAPAFHPPGPMPSIGAVPSTGPLPSAGPIPTVGSMASTGQVPSRPGPPATANHLGLSVPGLVSVPPRLLPGPENHRAGSGEDPVLAPSGTP
PPSIPPDETFGGRVPRPAFVHYDKEEASDVEISLESDSDDSVVIVPEGLPSLPPAPPSGTPPPAAPAGPPTASPPVPAKEDSEELPATPGPPPP
PPPPPPPASGPVTLPPPQLVPEGTPGGGGPTAMEEDLTVININSS
> Rattus_norvegicus [P003462]
MAAAVLSGPTTGSPAGAPGGPGGLSAAGSGPRLRLLLLESVSGLLQPRTGSHVAPVHPPIQWAPYLPGLMCLLRLHGTAGGAQNLSALGALVNL
SNAHLSSIKTRFEGLCLLSLLVGESPTELFQQHCVSWLRSIQQVLQSQDSPPTMELAVAILRDLLRYASQLPTLFRDISTNHLPGLLTSLLGLR
PECEQSALEGMKACVTYFPRACGFLKGKLASFFLSRLDSLNPQLQQLACECYSRLPSLGAGFSQGLKHTENWEQELHSLLTSLHSLLGSLFEET
ETAPVQSEGPGVEMLLSPSEDDNTHVLLQLWQRFSGLARCLGLMLSSEFGAPVSVPVQEILDLICRILGISSKNINLLGDGPLRLLLLPSLHLE
ALDLLSALILACGGRLLRFGALISRLLPQVLNTWSTGRDALAPGQERPYSTIRTKVYAILELWVQVCGASAGMLQGGASGEALLTHLLSDISPP
ADALKLCSTRGSSDGGLQSGKPSAPKKLKLDMGEALAPPSQRKGDRNANSDVCAAALRGLSRTILMCGPLVKEETHRRLHDLVLPLVMSVQQGE
VLGSSPYNSSCCRLELYRLLLALLLAPSPRCPPPLSCALKAFSLGQWEDSLEVSSFCSEALVTCSALTHPRVPPLQSSGPACPTPAPVPPPEAP
SSFRAPAFHTPGPMPSIGALPSPGPVPSAGPIPTVGSMSSAGSVPSTGPVPSRPGPPATANHLGLAVPGLVSVPPRLLPGSENHRAGSGEDPVL
APSGTPPPSIPPDETFGGRVPRPAFVHYDKEEASDVEISLESDSDDSVVIVPEGLPSLPPPPSGTPPPVAPTGPPTASPPVPAKEDSEELPATP
GPLPPPPPPPPPVSGPVTLPPPQLVPEGTPGGGGPTAMEEDLTVININSSD
>Latimeria_chalumnae [P032313]
VSALLGSLKEHRNLAAQSSSVMGGLVAISNSRLGSVKTRFEGLCLLALLVAESSTEHFQQHCLLWLRNVQHVLQSQDPQPTVDLAVFVLRDLLK
YSSQLPELSREISLNHIPGILTSLLGLKPECQVAALEGMRACMTFYPRPCGSLRSKLAAFFLCRQEAENPELQELACRCYALLPGLGAGFSQGV
KYTELWQQQLHCLLATLHGILGQLYEGAETEAVCYEGPGVELSLPALNETDQLHTLQLKHRFSGLSKSLSLLLSADFSVPVNVPAQEILNFVIR
TLSISSRNISWMGEGPLKVLVLPAVHLDALEVLSSLIIACGSRLARFANVICRLFPQVLSAWTVGGDSAVPGQEKAYSAIRFKVYKVLELWVKV
CGAASGVLQGSYQHSRALLTHLLSDITPATDTLKLKAGRPLAEMVMHHGKAGGRRSKVGDLNEAIQLQGSRKQDANANSDTCSAAIRALSHVVL
QCGSLLPEETHRKLHELVLPLLMRLQQSAGPASTPYLRAECRRELYRLLLFLLLTPSAKRPPPLHCAIRTFSLGQKDNDIEVAAFCAEALVICN
SLIHPRVPSLQIPTAAAGSAMFKSPVLTPEPSSSFRQPQAQSFSLPLQPSRPVTNQLGLPLSGLVAGQQLVTRLPPPLTTSMLLLPMVPCSVKG
ENHLSDQQMDIDPGLQGDLHSPCQDAFDDKGHKPVFVHYDKEEDSDVEISLESDSDDSVVIVPEGLPPKPPASPESKKAEEPEEAAKPAAPATT
ATAAAVAATAPVSMTAPTVAQPLSQAQTLLQNPVPESQGGLPPVAEEDLTVININSS
>Anolis_carolinensis [P025139]
AQASPSLGGLLSVSNARLGSIKTRFEGLCLLSLLVTESSTETFSQNCLPWLRSLQHLIQSQDPPPTMELAVLILGDLLEFSCQLPDLAREIGTN
HIPGLLTSLLALKPQCQLSALEGSKACMTFYPRACGSLRGKLAAFFLSCVDAEVPRLQQLACECYALLPSLGAGFTQGLKYTECWEQQAHCLLA
TLHSLLGTLYEGAETDPLHYEGPGMEMGLPPSEDGETGFLLQLRQRFSGLLRCLCNMLSKEFVAPVTVPVQDILDLVCRALDINTKNISWFGDG
PLRMLFLPSVHLEALDLLSALILACGPRLVRFGGTLRRLFPQVLGTWGAGRELFPAGQERPYSAVRTRLYQVLDLWVQVAGASSGVLQGQRTPS

DEALLGHLLSDISPPTETLKMREGRTPSDGKPSAPKKPKLSSSEVGGLGGSLQQKHDPGANSSVCLAALQVLSRTVLLSGSLIKEQTHKRLQEL
TVVLLVRLGQAESPPGSPLASAACRREMYRLLLALALAPAPSAPAPLHCALRLLSQGRADPNLQVSSFCAEALTVCNALLHPRVPSLQ
> Pelodiscus_sinensis [P017941]
MALGVAVLRDLLHYSCQLPELARDIGTNHIPGLLTSLLALKPECQVSALEGIKACMTFYPRACGSLRGKLAAYFLSHVDSDSPQLQQLACECYA
LLPSLGAGFTQGLKHRECWEQELHALLATLHSLLGNLYEAAETEPLPYEGPGLELLLPPAPPDGETSFVLNLCNRFSGLAKCLQLMLSCEFVAP
VTVPVQDVLDLVCRALNISVKNMSWFGDGPLKMLLLPSVHLEVLDVLAALILACGARLARWGSVLGRLFPQVLGTWSGCREAVPLGQEKPYSAV
RTRLYQVLELWVQVGGAGAGVLQGPSHRSEALLAHLLGDIAPPMDSIKLKVGRPGSEGKPSAPKKPKLSEGGEAPSLHRKQDSMANSDVCRAAF
AEPLVKEETHKKLQELVVPLLLRLAQGDVSPVGPYTSPACRHQLYRLLLALLLAPAPACPPPLQCALRAFALGQRDPSLQVSSFCAEALVTCSA
LARPRVPSLQLPLQGPAPSAGPAPSEVAASPFH
> Callorhinchus_milii [P024990]
VRLEGVCLLAQLVADSPTEIFQDQCISWLRAVQHIVQAQDPLPTVEMAVWVLRELLRYSSQLPELGREVAMNHIPSLVSSLLSLKPEYQLAAME
GMKACMTHYSRACGSLRGKLAAYFLSKMDTSSPQVQELACHCYALLPGLGTGFAQGMKYSESWSQQLHCLLSSLHRLTEEIYQGSETEPSRYEG
PGVDLPLPTLNDTDPYHVLHLSQRFTALSKCLAQLLRKDFSVAVTIPVQDVLGLVCRVLNISSKNMSCLGEGAVKVLVLPSLHTDALDILSALI
TTCGKRLLRFADVICRLFPQVLTAWATPRGSTPPGQERGHSGVRVTVYTVMETWLKVAGVASGVLQGAAHHPDILLSHLLSDVTPPSHTLQVRA
KPAAEVQTGSYGKPYGKRARLGEVTDTSPVRGHHKQDLTANSDVCLAALRVLSRAVLACGSLLKEETHKKLQEMVLSLLMGLGRRSGEPSPYLR
AQCRLELHRLLLFLLLVPNPHRPPPLHCAINTFSLGQQDSSTEVSAFCWEALVTCNALIHPRVASLQ
>Danio_rerio [P015767]
LPALLANYREHAAVGTQSSGAVGGLVGLSNARLGSSKTRFEGLCLLSVLVKDSSSEVFQQHCLSWLRTLQQIIQSQAPLPTVQLAVNVLQDMLQ
YSSQLPELAREVGLNSILGVLTSLLSLKSECHLAAMKGMMACMTYYPRACGSLREKLGAYFLSKMDSDNPKVQEVACECYGRLPCLGGVLERGG
GGRRAEGWTNQLHCLLASANSILGQLYHGAETEGTVQYEGPGVELPFPPLDDVDPLLILQLQHRYKAVTLAMKHTLSVDPASSVRLPVQHVLNL
LCRALAVNTKSISPTGDGCQKLLVLPSIHNDTLELLSALIKAVGGGLVQYSSVLTRLFSQSLSAWTPLPEASLGQQRAYSAVRVAVYRLIELWV
RVGGAALLQGSPSHTELLFTHLMGDVTPGSEAVKLRSVQQSSMTDLIGSAGKSGPRRAKGMGEGLTIQRKGDVLANQDTCVAALRALRQIVLTS
GTLLKEELHKRIQDLVVPLCVRLQQQAHCVQEVGAISGQYGSPSPRRELYRVLLALVLVPSPRWPPPLSCAVSIFSHGRRDRNIKVSSFCAEAL
IICNTLIHPRTPSISLPLTPLTLKSTPSAPVLASGQNSSLSIPTLLGGPTTGPPFPARHPMGLGPANLLGSLENHLPLAPQVLSSSAGTPGDLL
LSPAQPGELTGLGVPEGQRQVFVRYDKEEPDDVEISLESDSDDSVVIMPAGM
> Takifugu_rubripes [P015553]
YIPVLLTGYREHGVFHTQGASAVAGLVGFSNAKLGSSKTRFEGLCLLSMLVKDSSSDLFQQHCLSWLRSLQQIVQSQAPVQTIQLAVNILKDVL
QYSSQIPELAREVGLNSILGILTSLLGLKTECELAAMEGMTACMTHYPRACGSLKDKLGAYFLSKMDSTNKKTQEMACQCYAHLPCLGGVLDRG
AGAGRAEGWTNQIHCLLASANSHLALIYQGAEMEGTMQYEGPGVELAFPLLDQSDPLFLLQLQHRFTAVCLALRHTLRVDPASAVRIPVRPILN
LVCRALAVSCKSFNLTGDGNVRLLILPIIHLNILEVLAALIIAVRSSMVQYAAVLQRLFSQTLSAWTPAAEASVGQQRAFSSVRVSVYRTLELW
LQVAGASTSILHGSPNHSEILFNHLLSDITPGAESVKLRVGLSAEIVPGGKPGPRRTKSLVISDTVGPSLQRKGDIMANQDTCLTALRALRQII
LVSGTLLKDDIHKRLHDVLLPLCVRLQQQQLSSNMSCDSTAGISGQYSSALTRRELYRLLLALVLVPSPCWPPPLTCVVSILSSGRTDRNLKVS
TFCCEALTVCNSLLHPRSPSIALPMPPLSIKPAHAVSVLPTPQASTPGLTLPTLLGEPTPPPPFPSPHTLGMGPSSLLGSLENHLSLVPGLRGQ
TSGPSEMILSPHAHHQDLAGLGPPEGQRPVFVRYDREEAEDVEISLASDSDDSVVIVPPGMLNMENQQDDVAAANSQSMTAAAGGAAVTLAEGE
PVTMVPNTATAAPIDGVSLPNDLTTSAPLLTTSAPPINSFPPSSASVVSLVPALNSNPLTAPPGGLVEPIPSRPQLQQMLMQPSAAVQQGPLSL
PLQIHQLQSQLGQQGRPLQQQPPVASNEDSGVININST
> Salmo_salar [P036958]
YLPALLANYREHGVVSTQSSAAVGGLVGVSNAKLGNSKTRFEGLCLLSVLVKDSSSDVFQQHCLSWLRSLQQVIQSQAPLPSIQLAVGVLQDLL
QYSSQLPELAREVGLNSILGILTSLLGLKSEFHLAAMEGMTACMTFYPRACGSLRDKLGACFLSKMDSVNPEVQEAACECYGRLPCLGGVLERG
GGGRPAEGWTNQLHCLLASANGMLAQLYQGTESGMVPYEGPGVELPYPPLDDTDPLVLLQLQHRYRGVCLALKHTLSVDPATAVRLPVQQVLNL
VCRALAVSSKSINLTGDGSVRLLVLPSIHNHTVKVLQALITAVGSGLVQYSNMLQRLFSQTLSAWTPLPETSLGQQRAFSAVRVSLYQTLELWV
KVGGASAGVLQGSPTHSEILLAHLLGDITPGADSVRLRAGQSTVADLVSSKPCPKSRKPGLGMGNGGGASLQRKGDSLANQDTCVSALRALRQI
ILTSGTLLKEDVHKRLHDVVLPLCVRLQQQHGGDCGTGAVSGQYGSALPRRELYHLLLALVLVPPPRWPPPLTCTVSILSHGRRDRSLKVSSFC
TEALAICNSLLHPRTPSLCLPLPPLTLKPSPAASLLTPSQASSLTLPTLLGGPFPGRHSLGLGHTLLGSLDNHLSLVPPGLSGQGSTPGDLLLS
PHQGELAGLGLSEGQRPVFIRYDKEEAEDVEISLESDSDDSVVIFPRGM
> Salmo_salar [P036957]
YLPALLANYREHGVVSTQSSAAVGGLVGVSNAKLGNSKTRFEGLCLLSVLVKDSSSDVFQQHCLSWLRSLQQVIQSQAPLPSIQLAVGVLQDLL
QYSSQLPELAREVGLNSILGILTSLLGLKSEFHLAAMEGMTACMTFYPRACGSLRDKLGACFLSKMDSVNPEVQEAACECYGRLPCLGGVLERG
GGGRPAEGWTNQLHCLLASANGMLAQLYQGTESEGMVPYEGPGVELPYPPLDDTDPLVLLQLQHRYRGVCLALKHTLSVDPATAVRLPVQQVLN
LVCRALAVSSKSINLTGDGSVRLLVLPSIHNHTVKVLQALITAVGSGLVQYSNMLQRLFSQTLSAWTPLPETSLGQQRAFSAVRVSLYQTLELW
VKVGGASAGVLQGSPTHSEILLAHLLGDITPGADSVRLRAGQSTVADLVSSKPCPKSRKPGLGMGNGGGASLQRKGDSLANQDTCVSALRALRQ
IILTSGTLLKEDVHKRLHDVVLPLCVRLQQQHGGDCGTGAVSGQYGSALPRRELYHLLLALVLVPPPRWPPPLTCTVSILSHGRRDRSLKVSSF
CTEALAICNSLLHPRTPSLCLPLPPLTLKPSPAASLLTPSQASSLTLPTLLGGPFPGRHSLGLGHTLLGSLDNHLSLVPPGLSGQGSTPGDLLL
SPHQGELAGLGLSEGQRPVFIRYDKEEAEDVEISLESDSDDSVVIFPRGM
> Salmo_salar [P036960]
YLPALLANYREHGVVSTQSSAAVGGLVGVSNAKLGNSKTRFEGLCLLSVLVKDSSSDVFQQHCLSWLRSLQQVIQSQAPLPSIQLAVGVLQDLL
QYSSQLPELAREVGLNSILGILTSLLGLKSEFHLAAMEGMTACMTFYPRACGSLRDKLGACFLSKMDSVNPEVQEAACECYGRLPCLGGVLERG
GGGRPAEGWTNQLHCLLASANGMLAQLYQGTESEGMVPYEGPGVELPYPPLDDTDPLVLLQLQHRYRGVCLALKHTLSVDPATAVRLPVQQVLN
LVCRALAVSSKSINLTGDGSVRLLVLPSIHNHTVKVLQALITAVGSGLVQYSNMLQRLFSQTLSAWTPLPETSLGQQRAFSAVRVSLYQTLELW
VKVGGASAGVLQGSPTHSEILLAHLLGDITPGADSLRAGQSTVADLVSSKPCPKSRKPGLGMGNGGGASLQRKGDSLANQDTCVSALRALRQII
LTSGTLLKEDVHKRLHDVVLPLCVRLQQQHGGDCGTGAVSGQYGSALPRRELYHLLLALVLVPPPRWPPPLTCTVSILSHGRRDRSLKVSSFCT
EALAICNSLLHPRTPSLCLPLPPLTLKPSPAASLLTPSQASSLTLPTLLGGPFPGRHSLGLGHTLLGSLDNHLSLVPPGLSGQGSTPGDLLLSP
HQGELAGLGLSEGQRPVFIRYDKEEAEDVEISLESDSDDSVVIFPRGM
> Thunnus_orientalis [P018353]
YLPALLANYREHGVFPTQGASAVGGLVGFSNAKLGSSKTKFEGLCLLSLLVKDSSSDLFQQHCLSWLRSLQQVIQSQAPVQTIQLAVNILKDLL
QYSSQLAELAREVGLNSVLGILTSLLGLKTECELAAMEGMTACMTYYPRACGSLRDKLGAYFLSKMDSTNKKTQEVACQCYARLPCLGGLLDRG
VGAGRAEGWTSQIHCLLASANGLLSQIYQGSETVCEKVLFDVTDGTVQYEGPGVELAFPHLDQSDPLLLLQLQHRYTAVCLALKHTLRVDPASA
VRLPVRPILNLVCRALAVSSKSINLTGDGSVRLLVLPIIHTNTLEVLSALITAVRSGMVQYAAVLQRLFSQTLSAWTPLPEASLGQQRAYSSVR
VSVYRTLELWVQVAGASASILQGSPGHSELLFSHLIGDITPGAESVKLRAGLSADVVPGGKPGPRRTKPLVMADSVGPSLQRKGDLLANQDTCI
SALRGETLPQSDDRMRLHDVVLPLCVRLQQQQSSSSTACESAGGVSGQYGSALTRRELYRLLLALVLVPSPFWPPPLTCAVSILSNCRTDRNLK
VSSFCSEALTICNALLHPRTPSIALPLPPLALKPTPTAPVLSSSQGPTPGLTLPTLLGGPAPGPPFPTRHSLGLGSTSLLGSLENHLSLVPGLP
GQGHGPGDMILSPHTHHQPDLAGLGPPEGQRPVFVRYDKEEAEDVEISLDSDSDDSVVIVPPGM
> Mola mola [P027290]
AQVRSGRRGLCLLSMLVKDSSSDLFQQHCLSWLRSLQQVIQSQAPVQTIQLAVNILKDMLQYSSQLPELAREIGLNSVLGILTSLLGLKTECEL
AAMEGMTACMTYYPRACGSLKDKLGAYFLSRMDCTNKKTREMACQCYGHLPCLGGLLDRGVGARRAEGWTNQIHCLLASANSQLSQIYQGAETD
GTVQYEGPGLELAFPHLDQSDPLLLLQLQHRFTAACLALKHSLRMDPASAVRLPVRPILNLVCRALAVSCKSILVLYGCMHLWLLTFGMVQYAA
LLQRLFSQTLSAWTALPEASLGQQRAYSSVRVSVYRTLELWLQVAGSSACILQGSPCQSELLFSHLLNDITPGAESIKLRVGLSADVVPGGKPG

PRRTKSFVIADTVGVSLQRKGDLMANQETCLSALRALRQIILTSGTLLKGDIHKRLHDVLLPLCVRLQQQQSSSSPLCESAGGISGQYSSALTR
RELYRLLLALVLVPSPCWPPPMACALSILSNGRSDRNLKVSSFCSEALTICNSLLHPRTPSITLPLPPLTLKPTPAPSAPPVSQGSTPGLTLPT
LLGGPPLGPPFPPRQTLNLGPTSLLGSLENHLSLVPGLPGQTPAPGEMILSPHSHHQHDPTVVGPPEGQRPVFVRYDREEAEDVEISLASDSDD
SVVIVPPGMLSMENQQDETAAANSQNMASATSGGALVTLTEGESTATTTTVGVSLPNDLTTSSPLLTTSTTPINSFPPSSASVVSLVPPLNTST
STITVQPDGMGESMPGRPQLQQILMQPSTPGQPGPISLPLQMHQLQNQLSQQGRHLHQHQPPAASNEDSGVININSTD
> Branchiostoma_floridae [P007737]
VSHINTSLGTAKTRLEGLCLLGTVVQQCSAGTFIQHGTTWIRMLTQVLQAYDSPLTLQMASHVLGSVVQQAAQYPEVAREVATTHIPTLVQCLL
GAQDHQWFPSALEALQSCMKNFPGPCGSSKGKVESLICGLMDTSQPRLSQLAQQTCPLLAGCGGGGAGGVKYTEAWAHLCDQVLGSLHQVLDHA
YQDMETGLQTYSVPQASLRLKTVPESDPARTFVLSTRFHNLCGCLEQLVSQEFPAVVRIPVPDILAFLCRALGVNPKMLFGKASMEHVLLMSAL
PKMHCSALSILEALIISCRSHLVPHASVISQLLVQTLGWTTSEEGVPGRQRPYSTLRSRAYTVLTVWLNVCSAASGVDSHADVILQHVLKDATP
QADTTKLKASRPGAQEQSKRQKKKQRGVDMTDQGLSGHMKVDSQANSDVCCAALGALKTVLEVVGSIIKPSFHKETQEFVIPLLLKIHQNQSDP
PIPYSCGRCRKGLYQLLLAAVLVLHPRWPPPTQCAVKIFSVGQQDYDLQVSSFCREALLTCMSIIHPRASTLQ
> Lingula_anatina [P008775]
NSGLNAAKSRVESLVLLNDLLSQCSTETLSQYTITWTRLLIQIIKGYAPASIHRLACYVLGSLTEKSSTCPELARQVALDSLPQLIPALLVMKE
ESLEAALYCIGKCMQFYSGPCGTFKGKIENWVIDQLTKENPRVVEAAVSVFPWLSQCGGGGNQGIKHAESWSSNCHKLLGSLHDTLDQLYEGVE
SGVEENKKKSEKLSLSPVQGTGMERIVGLRNRLDMLCGCLGGMLSTTFPAVVRIPVPEVIGFIQRALAVTGRALLLRPSTDNLQLLSVLPFLHT
SVLRVLSCLISRYVLPFLHTSVLRVLSCLISRYVLPFLHTSVLRVLSCLILSCQKNLISYTSTVNQLLIQTLSWTITEDTADGRKRPYGNLRKE
VYSTLVVWVQTLGASSNVHNIIDQLLTQLKRDITPDVDVIKLETINKKPDQYTEPPNKRKKGNKAGLEGVSTRKVLDHSANADVCLSALAALRH
LLVAVGSLLKVQRIRDIQEIALPLALRVQAFTDQGSPGPYSDPECRRELYHVIQCLFLIPASKCPPPTHCVIRLFTGGQNDPDRKVSSFCQEAL
TICEAVIHPRVPSLQ
>Salmo_salar [P087754]
QSTGAVGGLVGLSNAKLGNSKTKFEGXCLLSVLVKDSSSDVFQQHSLSWLRSLQQVIQSQDPLPSVQLAVGVLQDLLQYSSQLPELAREVGLNS
VLGILTSLLVLRSEVACECYGRLTRLGGVVERGGGGCRAEGWTNQLHCLLASANAILAKLYQGSESGRECFFFHKVFQSMRADLGSSCPTLLWT
TQTPSSCCSYNTDTELPAWLSNIHSVRLPVQQVLNLVCRALAVSSKSINVTGDGCVRLLVLPAIHKDTLQVLHALITALGSGLVQYSSVLQRLF
SQTLSAWTPLPETSLGQQRAFSAVRVSLYRTLELWVRVGGASASVFQGSPTHSELLLAHLLGDITPGADSVRPSNSPQLRDLVSSKPCPKSRKP
GLAVSGGGGASFQRKGDSLANQDTCLSALRALRQVILTSGSLLKEDIHIYILVI
>Petromyzon_marinus[P038269]
RWDGLSLLAALVRDCSTQVFVQHCCAWLRAVTQLLQPYEPEGGLALAVRVLADLLRFSAQLPELAREVSQSHLPALMSALLALRDQRECMAMEG
LLACMRFYPKTSGTTKHWIAIAVLPQWLDNKPRVTKARRASHYRCRRTCWQRPTVYNAILKYKARHCLLSLHSGLNALYRPSVRCRRQTDGDEA
DMALPEPQADSPVYLLLVKQEFSCLARALFAMLREEFAAPVKVPAQPILTLVCRALAISPKRLAAVGEATMQLLVLPAVHTDALSLLEALIRTR
AARCGKRLLRFGDVINRAFPQVLATWSPPAETGGDPGRESPYSEVRVRAYGALAAWVDACGATCRVLQDELQHAHDLLRHLVRDASPASDLIRV
RPLSPAESP
> Hymenochirus boettgeri [A0A8T2J8G0]
MAAACVGTRGMEATMTGLLERDLSEGELAEAVRALREHGVLRGEGSFAAISGLLGCCNSR
LMSAGTRIEGLSLLSLIVEESPTDVFVQNCVSWIRSILQVIQSQDPPRVVSLAVFVLRSL
LAHSSGIPELSREVSTNHIPGLLTSLLGLRCQCLIPALEGIRSCLLSYPRACGSLRGKVT
AFLLSLLDSENQQTQELACQCYSLLPSLGSGFSQGIKHTENWERQMQSVICSLHGVFRQL
YQSSETDTARYEGSATELELPSLEDDGVHSVLQLVRRFTALAHCVCLLLREQFPAPVRVP
ASDLLSLICRVVNVSPKNLSWHGEESLKLLLLPRIHSSVLDILEATIIACGPRLLPFSAV
ICRLFPQLLLSWAAVKGMANIPSGQERPYSSLRGSIYRVLESWVTVCGISSGVLQGPSHH
SDMLLANLLNDITPPTDVIKMSGFVQLGAKKQKVSEVGVNEFQSHRKRDTSVNIELCTAA
LRGLCSIILHCGSIVKEDTHRRLQELSIPLLLRLQQGSDQWLGPYISSECRKELYRYPLF
NTDP
> Melopsittacus undulatus [A0A8V5HB30]
MGGGHCGSQSGAAEGAGAANQGAALALLSQWPCSFEGLCLLSLLVSESPTEPFTQHCLGW
LRALQHLLQSQDPAPTMALGVLVLRELLRFSSQLPELARDIGTDHIPGMLTSLLALRPEC
EVSTLEGIKACMSFYPRACGSMRGKLAAYFLARIDSDSPQVQQLACECYALVPALGRGFA
QGLRHTEAWQQELQGLLATLHALLGELFEGCESEPLPYEGPGVELLLPAPPDGDPSISLT
LHKRCSGLARVLQLLLSKEFVAPVTVPVQDILDLVCRALSITPKTINAAGDGALRLLLLP
AVHLDALDVLEALVLACGARLVRWGSALARLFPQVLSAWSGAREALPGQEKPFGEVRARL
YHVMELWAQVGGASCGLLQQPGAATEALLSHVMGDIAPPCDSLRVSDPKLSDIKYYRNNP
KYHRNSPKYPKYHRNSPNHPLYPVFNPNPPFFTQNPISPPILTKSPLFHPKPPSSRPRPS
PAPPSAPNWGGARMGTPPPCTASRSRQPIVMSAGLPLKGSIGPPIPLIQGDPPLWLQWNS
PKSHREGLGEVPLTPWGGGKRGPPPMGSMGCPHDP
> Chrysochloris asiatica [A0A9B0TDD2]
MKMAAAVLSGPSGGPAAGVPGGTGGLSAMGSGPRLRLLLLESVSGLLQPRTGSTVPSVHP
PVRSVPHLPGLVCLLQLHGTVGGAQNLSALGALVGLCNTRLGSIKTRFEGLCLLSLLVGE
SPTEIFQQHCVSWLRSIQQVLQSQDPAPTMELAVAILKDLLRYAAQLPVLFRDISMNHLP
GLLTSLLGLRPECELSALEGMKACMLYFPRACGSLKGKLTSFFLSRVDSLSPHLQQLACE
CYSRLPSLGAGFSHGLKHTESWEQELQCLMASLHSLLGTLYEGAETAPAQYEGPGVETLL
PPSEDGDAHALLRLRQRFSGLARCLGLMLSSEFGAPVSVPVQEILDLICRTLSINGKNIS
LLGDGPLRLLLLPSIHLEALDLLSALILACGSRLLRFGGLIIRLLPQVLNSWSMGRDILA
AGQERPYSTVRAKVYAVLELWVKVCGPSAGMLQGGASAEALLTHLLTDIFPPADTLKLRS
PRGSPDGGLQTGKPSAPKKLKLDVGEAVAPPCHRKGDNNANSDVCAAALRGLNWTILMCG
PLIKEETHRRLHDLVLPLVMGVQQGEVLGSSPYTSPHCRRELYRLLLALLLAPSPRCPPP
MACALQAFSLGQREDSLEVSSFCSEAMVTCVALTHPRVPPLQSMSPTCPTPAPVPPPEAP
SPFRAPPFHPAGTMPSLGPMPSAGPMPTECPMPSARPGPPATANHLGLSVPGLVPLPPRL
LPGPENHRASSNEDPVLAPSGSPPPIAPDETFGGRVPRPAFVHYDKEEASDVEISLESDS
DDSVVIVPEGLLPLPPLPPSGTTPPPVAPVGPPTASPPVPAKEEPEELPSAPGPLPPPPP
LPVPGPVTLPPPQLVPEGTPGGGGPPTLEEDLTVININSSDEEEEEEEEEEEEEEEEEEE
EEDFEEEEEEEEYFEEEEEEEEEFEEEFEEEEGELEEEEEEEEDEEEEEEELEEVEEVEFGS
AGGEVEEGGPPPPTLPPALPPSESPKAQPEPEPEPGLLLEVEEPGAEEEPGAEMAPTLAP
EVLPSQREVEREEGSPPAAEPPPQELIEEEPSAPTTLLEEGTEDGGDKGPPPPETSAEEE
METEAETAVLQEKEQDDTAAMLADFIDCPPDDEKPPPATQPDS
>Camelus bactrianus [A0A9W3GQK0]
MELAVAILRDLLRYAAQLPTLFRDISMNHLPGLLTSLLGLRPECELSALEGMKACMTYFP
RACGSLKGKLASFFLSRVDALSPQLQQLACECYSRLPSLGAGFSQGLKHTESWEQELHSL

LASLHSLLGALYEGAETAPMQYEGPGVETLLSPSEDGDAHVLLQLRQRFSGLARCLGLML
SSEFGAPVSVPVQEVLDVICRTLSISAKNISLLGDGPLRLLLLPSLHLEALDLLSALILA
CGGRLLRFGALISRLLPQVLNAWSIGRDALSPGQERPYSTVRTKVYAVLELWVQVCGASA
GVLQGGASGEALLTHLLSDIAPAADALKLRSPRGSPDGGLQTGKPSAPKKLKLDVGEAMA
PPSHRKGDSNANSDVCAAALRGLSRTVLMCGPLIKEETHRRLHDLVLPLAMGVQQGEVLG
SSPYTSARCRLELYRLLLALLLAPSPRCPPPLACALQAFSLGQREDSLEVSSFCSEALVT
CAALTHPRVPPLQSMGPACPTPAPVPPPEAPSPFRAPAFHPPGPMPSVGPMPSASPMPSA
GPMPSAGPVSSAGPMPSVGSMPPARPGPPATANHLGVSVPGLVSVPPRLLPGSENHRASS
NEDPILAPSGTPPPTVPPDETFGGRVPRPAFVHYDKEEASDVEISLESDSDDSVVIVPEG
LPPLPPPPPSGTTPPPVAPAGPPRASPPVPAKEESEELPATPGPLPPPPPPPVPGPVTLP
PPQLVPEGTPGGGGAPALEEDLTVININSSDEEEEEEEEEEEEEEEEEEDFEEEEEEDEE
EYFEEEEEEEEEFEEEFEEEEGELEEEEEEEDDEEEEELEEVEELEFGPAGGEVEEGGPP
PPSLPPALPPAESPKVPPEPEPEPGLLLEVEEPGAEEGPGAETAPTLAPEVLPPQGEVQG
EEGSPPAVPSPQELGEEEPSAPPTLLEEGAEGVGDQVPPQPEASAAEEMEMETEAAALQE
KEQDDTAAMLADFIDCPPDDENPPPAPEPDS
> Eublepharis macularius [A0AA97LAL0]
MAAAAASSGAAPAGPGPLVLETLAGLVRGPEPPGGEGGGSAALPRGLPGLVRCARDSGGG
GEAQASPALGGLVSVSHVRLGSLQTRFEGLCLLSLLVTGSSAEAFGQNCLGWLRSLQQVI
QSQDPPATMGLAVLVLRDLLGYSCQLPELARDLGTNHIPGLLTSLLALKPECQISALEGS
KACMMYYPRACGSLRGKLTTYFLARVDAETPHLQQLACDCYALLPSLGAGFAQGLKYTEC
WEQQVHCLLATLHSLLGTLYEGAETESLHYEGRGVEIPLPTPEQGETSFLLHLKLRFTGL
AKCLCRMLSNEFPAPVTVPVQDILDLICRTLNVSTKNFGWFGDGPLRMLLLPAVHLEILD
LLSALILACGPRLVRFGGILCRLFPQVLTSWSAGRDLLLPGQERPYSAVRTRLYQVLDLW
VQVAGAASGILQGHKPRSEALLGHLIADVSPPSETLKLQEGCPGPDGKPSAAKRPRLSDL
GSLGSVHRKYDPQANSDVCLAALQGLSRTVLLSGSLLKDQAHKRLQELVIPLLIRLSQAD
AFPGSPYSSACCRQELYRLLLALVLAPAPSCPPPLHCALRLLSQGRADASLQVSSFCAEA
LVVCSSLLHPRVPSLQLPLAGPSGPHPSSSEISPATASPFRPTPPLPFPAARLPPPASPS
APPSVATSANSLGLPLPSLNLGQLPPRLTPEEPPLPPSPGTAEAAALAAGVRMRRPIFIH
YDKEEEEDVELSLESDSDDSVVIVPKGQLGKVVNNTSAAAVAAAPAPPAPLPLPLAPPPA
APASPPPVSEEGPAEPAAPLPQGPPPPPPAAVVLPPSPAPVANDAVLPALVEEGPPTVIN
INSSEEEDEEEEEDEEEEDFPEDEDYFDEEEEEEFEEEEEEEEEFEGELDEEEEGELDEE
ELEELGEEEEEFDEEEEEEEGLTEEEEEEEEEGLTEEEEEGLPPTLLPPRNNEEPPELLP
PVIEEAPKLVGEEEGAAGLLMEVDEEAFHPPEAAVAEELASSPAEAPPVPPLLLPGTPAL
PLPSTQEAPPPRAASVEREEKPCQEEEEGGPPDGVALAASSPGAAAAEEAAAPEGHAGDA
> Silurus asotus [A0AAD5A5A6]
MATAAWLHGPKNMRLTEGLVSALKEERPEYLPALLADYREHGVVGTQNCGTVGGLVGISN
SRLGSSKTRFEGLCLLSMLVKDSSSEVFQQHCLSWLRSLQQIIQSQAPLPSVQLAVSVLQ
DVLHYSSQLPELAREVGLNSILGILTSLLGLKSECQLAAMEGMMACMTYYPRACGSLKEK
LGVYFLSKMDSDNPKVQDVACECYGRLPCLGGVLERGGGGRRAEGWTSHVHCLLVSANSI
LGQLYQGIETEEAIRYEGPGVELSFPPLDDVDPLLILQLRHRYKAVCLALKHTLSVDPAT
SVRLPIQQVLNVVCRALAVSIKNINVTGEGCLKLLVLPSIHSDSLEVLSALIKAVGAGLV
QYCNVLSRLFSQALCAWSPLPEASLGQQRSYSAVRVALYNTLELWVHVGRASSSVLQGNS
SHSELLFAHLIGDITPGNEAVKLRAGQTPISDLVTAGKPGPRRTKGMGIGDPGGVSLQRK
GDALANQDTCFAALRVLRQIILTSGTLLKEDLHKKLQELVVPLCARLQQQAQCSNWDVDS
VSGQYGSAAPRCELYALLLALVLVPSPRWPAPLSCAVCVFSQGRRDRNITVSSFCAEALT
ICNALLHPRTPSISLPLPPLTLKPTPASSVLAPTQNPSLSLPTLLGGPAPGPSFAPRHPL
SLGTASLLSSLENHLPLGAPVLPTPAGATGTQGELLLSSSAQAAELAGLAPPETQRQVFV
RYDKEEPEDVEISLESDSDDSVVIMPQSMMLEMQDGAANTQSLPPPPGSAMPVPNAGLGN
DAGPVETSLSNDIPTSIAHQLMPPDSNINSFPGSSQTEQLVSLVPPLNSNAVTLPVSSNA
LGNSLPAGAQLQQMLMQPSSGGQPNQLGLSLHMQLQNQLVQSSRQPAANEQDQNVININS
TDDEEEEEEEEMEDEEELGEEEEEEEGLEEDEEEDERRYRDSFCREEYECFDDEDDDEDE
GDEMEEEDEEEEDDEEEIQEAEILEADNRRDVLGVEEGEVMIEGQEERGVGTFPIEGERQ
EEGGIEEMKAVQSIYEEEEIKDKGGVEEIENIGAVERNESVVGEQQIETHVIGAEGEQSE
ENASIEAADQEVQSQEEEASRAQSVVTTEDSGIIPEGQEQEVAIEVRDDEQSAAAQQEEI
PNANTTTTSEDAVPQQAVEAAEKEVAGETQVEPEEARGTKRKIEDREEGEESEQSSEKKK
LDDEAMASMLADFVDCPPDDEDHGASHSHS
> Canis lupus familiaris [A0A8I3S228]
MLGAVSPGGGRGDYKLPGCCAHSPVCAATATATSRPTVNLSAVGALVGLSNARLGSIKTR
FEGLCLLSLLVGESPTEMFQQHCVSWLRSIQQVLQSQDPPPTMELAVAVLRDLLRYAAQL
PTLFRDISMNHLPGLLTSLLGLRPECELSALEGMKACMTYFPRACGSLKGKLASFFLSRV
DALSPQLQQLACECYSRLPSLGAGFSQGLKHTESWEQELHSLLASLHSLLGALYEGAETA
PVQYEGPGVEVLLTPSEDGDTHVLLRLRQRFSGLARCLGLMLSSEFGAPVSVPVQEILDV
ICRTLSISAKNISLLGDGPLRLLLLPSIHLDALDLLSALILACRSRLLRFGALISRLLPQ
VLNAWNLGRDTLAPGQERPYSTVRTKVYAVLELWVQVCGASAGMLQGGSSGEALLSHLLS
DISPPADTLKLRSPRGSPDGGLQTGKPSAPKKLKLDVGEAMAPPSHRKGDSNANSDVCTA
ALRGLSRTILMCGPLIKEETHRRLHDLVLPLVMGVQQGEVLGSSPYTSSRCRQELYRLLL
ALLLAPSPRCPPPLACALQAFSLGQREDSLEVSSFCSEALVTCAALTHPRVPSLRSMGPA
CPTPAPAPPPEAPSPFRAPPFHPPGPMPSAGPMPSVGPMPSVGPMPPAGPMPPAGPMAPT
RPGPPATANHLGLSVPGLVSVPPRLIPGPENHRAGSNEDPVLAPSGTPPPAIPPDETFGG
RVPRPAFVHYDKEEASDVEISLESDSDDSVVIVPEGLPPLPPPPPSGTTPPPVAPAGPPV
ASPPVPAKEEPEELPVAPGPLPPPPPPPVPGPVALPPPQLVPEATPGGGGPPALEEDLTV
ININSSDEEEEEEEEEEEEEEEEEEEEEEEEDFEEEEEEEEYFEEEEEEEEEFEEEFEEE
EGELEEEEEEEDEEEEEELDELEEVAFGPAAGAAEEGGPPPPSPPPALPPAQSPKMQPEP
PGETGLLLEVEEPAAEEEPGAEAAPTLAPEVLPPQGEGPREVGSPPAVPPPQELIEEEPP
APPTLLEEGTESGGDKVPVPQETPAAEDVEAEVEAETAALQEKEQDDTAAMLADFIDCPP
DDDKPPPATEPDS
> Oncorhynchus mykiss [A0A8K9V8H1]

MAATAAWMHGPANMRLTEGLLSVLKEQRPEYLPALLANYREHGVVSTQSSAAVGGLVGLS
NAKLGNSKTRFEGLCLLSVLVKDSSSDVFQQHCLSWLRSLQQVIQSQAPLPSIQLAVGVL
QDLLQYSSQLPELAREVGLNSILGILTSLLGLKSEFHLAAMEGMTACMTFYPRACGSLRD
KLGACFLSKMDSVIPEVQEVACECYGRLPCLGGVLERGGGGRRAEGWTNQLHCLLASANG
MLAQLYQSTESEGMVPYEGPGVELPYPPLDDTDPLVLLQLQHRYRGVCLALKHTLGVDPA
SAVRLPVQQVLNLVCRALAVSSKSINVTGDGSVRLLVLPSIHNHTVKVLHALITAVGSGL
VQYSSMLQRLFSQTLSAWTPLPETNLGQQRAFSAVRVSLYRTLELWVKVGGASAGVLQGS
PTHSEILLAHLLGDITPGADSVRLRAGQSTVADLVSSKPCPKSRKPGLGMGNGGGASLQR
KGDSLANQDTCVSALKALRQIILTSGTLLKEDIHKRLHDVVLPLCVRLQQQHGGDCGAGG
VSGQYGSALPRRELYRLLLALVLVPPPRWPPPLTCTVSILSHGRRDRSLKVSSFCTEALT
ICNSLLHPRTPSLCLPLPPLTLKPSPATSLLTPSQASSLTLPTLLGGPFPGRHSLGLGHT
LLGSLDNHLSLVPPGLSGQGSTPGDLLLSPHQGELAGLGLSEGQRPVFIRYDKEEAEDVE
ISLESDSDDSVVIFPRGMLMLENQDGISTVATLPVSSLVPGGVTLPIPGPGDDPGGDISL
PLANDLPSTSLPHPLLPSSSAPNSINSFPPAPLASLVPPLNSTGVTQLGAPSVGLGVGAD
SIPGAQLQQMLLQGQSPVPGQPTPLGLPIQMLQNQLAQPSRALQQQQASEEDHSVININS
SDEEEEEDEEMEDEDELGEEEDEEGLEDEEEEEEGSDFPDEEEEFYGEEFDDYEEEEGEE
EEEEDEEEEEEGEIRPLDREGRRGGMGREEGEVLREVPEDEGMGGFCVEGEMEGGIEELG
TNRRVYGEEGVKAQEVESIGVLEEEREGEEDDADGMNDPTMPQILCVTGGALEEREELGE
GHGQEVVSCEQQGADRPEAAPSSEGPTPQHKQEAEPAQEVRVGGSDQPSNQGEEPAKQGG
ESAKEEVKPSAAPSETEGEEPEREKGGEEGEEDGEEDGRGMKRKREGEEEGTGQGTEKKK
VRTLSQTIFNHSHSKPFS

> Ursus maritimus [A0A8M1FUL7]

MAAAVLSGPSAGSAAGVPGGTGGLSAVGSGPRLRLLLLESVSGLLQPRAGSAVAPVHPPV
RSAPHLPGLMCLLRLHGTVGGAQNLSAVGALVGLSNARLGSIKTRFEGLCLLSLLVGESP
TEMFQQHCVSWLRSIQQVLQSQDPPPTMELAVAVLRDLLRYAAQLPTLFRDISMNHLPGL
LTSLLGLRPECELSALEGMKACMTYFPRACGSLKGKLASFFLSRVDALSPQLQQLACECY
SRLPSLGAGFSQGLKHTESWEQELHSLLASLHSLLGALYEGAETAPVQYEGPGVEVLLTP
SEDGDAHVLLRLRQRFCGLARCLGLMLSSEFGAPVSVPVQEILDVICRTLSISAKNISLL
GDGPLRLLLLPSIHLDALDLLSALILACGGRLLRFGALISRLLPQVLNAWNFGRDTLSPG
QERPYSTVRTKVYAVLELWVQVCGASAGVLQGGASGEALLSHLLSDISPPADALKLRSPR
GSPDGGLQAGKPSAPKKLKLDMGEAMAPPSHRKGDSNANSDVCAAALRGLSRTVLMCGPL
IKEETHRRLHDLVLPLVMGVQQGEVLGSSPYTSSRCRRELYRLLLALLLAPSPRCPPPLA
CALQAFSLGQREDSLEVSSFCSEALVTCAALTHPRVPPLQSMGPACPTPAPVPPPEAPSP
FRAPPFHTAGPMPSVGPMPSVGPMSSAGPMPSAGPMPPTRPGPPATANHLGLSVPGLVSV
PPRLLPGPENHRAGSNEDPVLAPSGTPPPAIPPDETFGGRVPRPAFVHYDKEEASDVEIS
LESDSDDSVVIVPEGLPPLPXPPPSGTTPPPVAPAGPPTASPPVPAKEEPEELPAAPGPL
PPPPPPPVPGPVALPPPQLVPEGTPGSGGPPALEEDLTVININSSDEEEEEEEEEEEEEE
EEEEEEEDFEEEEEDEEEYFEEEEEEEEEEFEEEFEEEEGELEEEEDEDEEEEEELEELEEV
EFGPAGGPVEEGGPPPASPAPALPPAQSPKVQPEPEGEPGLLLEVEEPGAEEEAGAEAAP
TLAPEVLPSQGEGPRDTGSPPAGPPPQELMEEEPLAPPTLLEEGTESGGDKVPPPAETAV
AEDVETELETATAALQEKEQDDTAAMLADFIDCPPDDEKPPPAPEPES

> Catharus ustulatus [A0A8C3US63]

MALQVRGAVGGAGGGVSPLSQWRCRFEGRGGGACPRSANGVAGSRGCGWGRGRVPAQPMA
LQVRGAVGGAGQGACPGSANGVAGSRGCGRGGGVSRLSQWRCRFDGQGRGGRVPAQPMAL
QVRGAVGGAGQGGVSPLSQWRCRFEGLCLLSLLVSESPTEAFQQHCLGWLRLLQHLLQSQ
DPPPTVALGVSVLRELLRFSAQLPELARDIGTNHIPGILTSLLALRPEVGSKWGWGLQMG
VGASGGKGLGGVIFNVFNPFFAVSQLACECYALLPSLGRGFSQGLRHTECWHQQLQGVLA
TLHGLLGALFEGCETDPLPYEGPGVPLLLPPPQDGDSGDFLTLHSRFSGLCRVLRLLLSK
DFVAPVTVPVQDILDLVCRALNVTTKNLSWFGEGPLRGLVLPQLHLDVLDVLGALVLACG
PRLVRWGSLLCRLFPQVLSSWSCPRDPPAGQERPYGAVRTRLYQVLELWVQVSGAASGVL
QTPGPPSEALLAQLLCDISPPSEGAKLRPEPKPSAPKRPKLGEGPEGPPLHRKGEPTANS
DTCAAALSALRRILLTGGPLIKEETHRELLLPLALRLPLPPLPPEFLGSPFGSGRCRGGL
YGVLQALLLGTPPGAAPPLSCAIRAFTQGQRDPDVSSLCSEFLVVANALARPWVPPLGSP
PPPPAPPPPPDPSLRPLPPPNPEPAPPPPSVCGAAPPPPGGDLGGVPGGVGVPGGGGARP
RRPVFVHYDKEEASDVEISLESDSDDSVVIVPKKPPLPHPAPPPPPPPPAALPPPPPPPV
PLEKTPGGGISAPPPPLPPPPPPPPPPAPPTPPAAPEPPQEGEGGEGQEGGGGGGPAVIN
INSSEEEEEEEEFGEEEEEEEEEEEEEFFEEEEEEEEEEEFEEEEEEEEEEEFEEEEEEEFEEE
EEGEGLSEEEEEEEEEEGEGEGPAEGGGGGALLLMEVEETPPPAPPPPLPEVPPEPLPHPR
EEEEEEGEEEEPPLPHPAPPPQPQPPAAPPPPRGSPRPSPRARGRPQIPSPTDPDPSPTQ
GRAQPRPLPPRPSPTAAGGEQDETTSMLADFIDCPPDDEKTPETPL

> Denticeps clupeoides [A0A8C4FLC8]

CLQTSAAVGGLLGLSHARLGSSKTRFEGLCLLSVLVKDGSTELFQHHCLSWLRSLQQVVQ
SQAPVPTVQLAVNVLQDVLQYSSQIPELAREIGLNSILGILTSLLGLKAECHLVAMEGML
ACMTYYPRACGSLREKLGAYFLSKMDSDNPKAQEVACECYGRLPCLGGVLERGGGGRRAE
SWTNQMHCLLASANSMLGQLYQGTETGTVQYEGPGVELPFPPLDEIDPLLVLQLRHRYRA
VCLALKHTLRSSPVRLPVQHMLNFVCRALAVSSKSINVTGDGSLKLLVLPSVHSDTVEVL
SALIKIVGSGLVQYCGVINQLFSQSLSAWTPLPEASLGQQRAYSAVRVCLYHTLELWVRV
GGASASVLQGSSTHTELLFSHLMGDVTPGSEAVKLRSGQSAAAISDLVGLGGKTGPRRPK
ALGIGETGGVSLQRKGDTIANQDTCLSALRGLFLGTLLKDELHKKLQDMVVPLCVRLQQQ
QAVGEVGGISGQYGSAHPRRELYRLLLALLLVPSPRWPPPLACAVSIFSHGRRDRSLMVS
SFCAEALTICNCLLHPRTPSIALPLPPLTLKPTPTAPVLPSSQNPTMSLPSILGGPAPGT
PFPARHPLTLGTSALMRSLQNHLPLAPSELTTPVGASATPADLLLSPAQQSELTGLPPPE
GQRQVFIRYDKEEAEDVEISLESDSDDSVVIVPQSLPSAPPVPSQGAAAVPGVTLPEPTG
DAVATGNASLPNELPSSSSPPVPPPAPNSINSFTVRAPQLTSALPSLIVPSIGMDLIPSG
AHLLMNPRPAGSVQAQMGRPMLLDMPAQSAPPTRLMPPQQMPSEEDTTVININSSDEEDE
EEEEIEDEDEFGEDEEEEGLDEDDEEEDSEFEEEGTYEDCDYEEEEEGEEIEEEEEEEEE

GAEDLATLEEVTRRGPMGRPETEVMIGSEEEEQQQRGIEMFPLEAQDEGNEEMEGSSAVE
ALEEGGPVEEEEEEEDLEKEKSGEVEVEASEQQKEKDGQDQEVGEELGSQEVRIWEQEGS
QAEFTAVTEEASPPECKEDESKVGGQDQKSVGLDQEEMPTQSRADAPREESEPQVAAEGA
EPEKGGEEKDEEEDSRGMKRKMEDREEETLEQSTEKKKVSSIASSN
> Laticauda laticaudata [A0A8C5SB50]
MPRAARRPRLPPRPARRRSRPRGWRNTRWGGGVAAAAALGARRGSSSCSSPGGGGSSSSA
GAASPSPPLAPSSGLALEALVGLLLPRPGASLPPPPPPPGLPGLVRCARDCGAQAQVSAR
SAGFEGLCLLSLLVTESGSETFAQNCLGWLRSLQHLLQSQDPPATMELAVPVLRDLLRYS
AQLPEVARDIGTNHIPGLLTSLLALKPECQLPILEGCQACMTFYPRACGSLRGKLATYFL
SCMDAETPHLQQLACECYALLPSLGAGFAQGLKYRESWEQQAQSLVATLHRLLGRLYEGA
ETEPLHYDGPGEEVLLPPREEEANPLLLAKRRFAGLAKCLCRMLRNDFGAPVAVPAQAIL
DLVCRALDVSVKSMSWFGDGPLRMLLLPSIHLEALDLLAALILACGPRLVRFGGALCRLF
PQVLNMWRAGQDLPSPGLQRPYRLVVEGLRALAHLTGVAPPLQDSQSGCPAGHLDYDISP
PSDVLKVSTTPPHPVFSPGGIGAEGGPNLALREKRSPACQEAQTVSMWAPWGAPFRKARP
QANSDVCLAACKEFFLPRWLLPEALSLHPQRLQELTIPLLIRLGQADLSLGSPYASASCR
RELYRLLLALSLAPAPSCPPPLHCALRLLSQGRTDPNLLVSSFCTEASVICSALLHPRVP
SLQLPLPAPPAHLPGSSQVSPAVATTLSPFCPAVPVPFPALRPLPPAPGPLPAGSLGLPL
SGLAAPPLPPRLLPEEPLPPPSPGTAEAAALATGAKLRRSVFVHYDKEEEEDVEISLESD
SDDSVVIVPKGQLGKTTSAPNSAPVPAPPEPPPPPPAPPPLPTPPLVASPPRGCGGEGEC
SCAGAPALVLVSAPGWSMDGPLGRAAAPGCLCVSVASLPPAGQEEEFDEEEGDFEEMDEE
EDDFDEEEEEGLTEEEEEEEEGLTEEEEDEETGLPPPLPRRNEDEPPALLPAVKDGSLKLQ
VEEEEEDGGAGLLMEVEEEAFQPPEEEQEEEEGERTAGALLLLKAEEDPAVPLLPGAQPL
PLPPLPESPGPTEQEGPSQQKEAAPAGSLPPEEAAQAGSSGDQAQGGPDSAAAGQGEPKK
LPQPDEEVVEVKEEEVRAPEPGEKGSWRGWSWALCSRAKGHTHTHTLSGGPLACTKAGLM
CGLGGWGGA
> Microcebus murinus [A0A8C5W7U3]
MELAVAVLRDLLRYAAQLPALFRDISMNHLPGLLTSLLGLRPECEQSALEGMKACMTYFP
RACGSLKGKLASFFLSRVDSLVPELQQLACECYSRLPSLGSGFSQGLKHTESWEQELHSL
LASLHSLLGALYEGAETAPVQNEGPGVETLLSSSEEGDTHVLLRLRQRFSGLARCLGLML
SSEFGAPVSVPVQEILDLICRTLSVSGKNISLLGDGPLRLLLLPSIHLEALDLLSALILA
CRGRLLRFGVLISRLLPQVLNAWSIGRDSLSPGQEKPYSTIRTKVYAVLELWVKVCGASA
GVLQGGASGEALLTHLLSDISPPADALKLRSPRGSPDGGLQTGKPSAPKKLKLDVGEAMA
PPSHRKGDSNANSDVCVAALRGLSRTILMCGPLIKEETHRRLHDLVLPLVMGVQQCEALG
TSPYTSSCCRRELYRLLLALLLAPAPRCPPPLACALQAFSLGQQEDSLEVSSFCSEALVT
CAALTHPRVPPLQSMGPTCPTPAPVPPPEAPSPFRAAPFHPPGPMPSVGPMPSAGPMPSA
GPMPSAGPVPPARPGPPATANHLGLPVPGLVSVPPRLLPGPENHRAGSNEDPVLAPSGTP
PPTIPPDETFGGRVPRPAFVHYDKEEASDVEISLESDSDDSVVIVPEGLPPLPPPPPSGT
TPPPVAPSGPPTASPPVPAKEEPEELPAPPGPLPPPPPPPPPPVPGPVTLPPPQLVPEGTP
GGGGPPALEEDLTVININSSDEEEEEEEEEEEEEEEEEEEEEEEDFEEEEEDEEEYFEEEEE
EEEEFEEEFEEEEGELEEEEEEEEEDEEEEELEEVEREGGSPAAGPPPQELVEEEPSAPPT
LLEEGTEGGADKVPPPPETPAEEEMETEAEATALQEKEQDDTAAMLADFIDCPPDDEKPP
PTTEPDS
> Marmota marmota marmota [A0A8C5ZU47]
MAAAVLSGPSAGSPAGVPGGAGGLSAMNSGPRLRLLLLESVSGLLQPRTGSTVAPVHPPV
CSVPHLPGLMCLLRLHGTVGGAQNLSALGALVSLSNARLGSIKTRFEGLCLLSLLVGESP
TEMFQQHCVSWLRSIQQVLQSQDPPPTMELAVAVLRDLLRYAAQLPTLFRDISINHLPGL
LTSLLGLRPECEQSALEGMKACMTYFPRACGSLKGKLASFFLSRVDALSPQLQQLACECY
SRLPSLGAGFSQGLKHTESWEQELHSLLASLHSLLGALYEGAETAPVQNEGPGVETLLSP
SEDGDAHVLLRLRQRFSGLARCLGLMLSSEFGAPVSVPVQEVLDLICRTLSVSGKNISLL
GDGPLRLLLLPSIHLEALDLLSALILACGGRLLRFGALISRLLPQVLNAWSIGRDAVSPG
QERPYSTIRTKVYAILELWVQVCGASAGVLQGGASGEALLTHLLSDISPPSDALKLRSPR
GSPDGGLQTGKPSAPKKIKLDVGEAMAPPSHRKGENNANSDVCAAALRGLSRTILMCGPL
IKEETHRRLHDLILPLVMGVQQGEVLGSSPYTSSCCRRELYRLLLALLLAPSPRCPPPLA
CALQAFSLGQREDSLEVSSFCSEALVTCAALTHPRVPPLQAMGPTCPTPAPVPPPEAPSP
FRAPSFQPPGPMPSVGPMPSAGPLTSSGSMPSVGPMPSAGPMPSRPGPPATANHLGLSVP
GLVSVPPRLLPGPENHRAGSNEDPVLAPSGTPPPTIPPDETFGGRVPRPAFVHYDKEEAS
DVEISLESDSDDSVVIVPEGLPPLPPPPPSGTTPPPVAPTGPPTASPPVPAKEEPEELPA
APGPLPPPPPPPPPPVPGPVTLPPPQLVPEGTPGGGGPPALEEDLTVININSSDEEEEEEE
EEEEEEEEEEEEEEEEFEEEEEEEEEYFEEEEEEEEEFEEEFEEEEGELEEEEEEEEEEE
EEELEEVEDLEFGSAGGEVEEGGPPPPTLPPALPPPESPKEQPEPEPEPGLLLEVEEPGS
EEQHGTETAPTLVPEVLPSQGEEEREGGSPEAGPPPQELVEEEPSAPPTLLEEGTEGGGD
NVPPPPETAAEEEMETESEAAVLQEKEQDDTAAMLADFIDCPPDDEKPPPTTEPDS
> Oncorhynchus kisutch [A0A8C7JA14]
MAATAAWMHGPANMRLTEGLLSVLKEQRPEYLPALLANYREHGVVSTQSSAAVGGLVGLS
NAKLGNSKTRFEGLCLLSVLVKDSSSDVFQQHCLSWLRSLQQVIQSQAPLPSIQLAVGVL
QDLLQYSSQLPELAREVGLNSILGILTSLLGLKSEFHLAAMEGMTACMTFYPRACGSLRD
KLGACFLSKMDSVIPEVQEVACECYGRLPCLGGVLERGGGGRRAEGWTNQLHCLLASANG
MLAQLYQSTESEGMVPYEGPGVELPYPPLDDTDPLVLLQLQHRYRGVCLALKHTLGVDPA
SAVRLPVPQVLNLVCRALAVSSKSINVTGDGSVRLLVLPSIHNHTVKVLHALITAVGSGL
VQYSSMLQRLFSQTLSAWTPLPETNLGQQRAFSAVRVSLYRTLELWVKVGGASAGVLQGS
PTHSEILLAHLLGDITPGADSVRVIRSDWFIGMAKPGLGMGNGGGASLQRKGDSLANQDT
CVSALRGEGAGRLHDVVLPLCVRLQQQHGGDCGAGGVSGQYGSALPRRELYRLLLALVLV
PPPRWPPPLTCTVSILSHGRRDRSLKVSSFCTEALTICNSLLHPRTPSLCLPLPPLTLKP
SPATSLLTPSQASSLTLPTLLGGPFPGRHSLGLGHTLLGSLDNHLSLVPPGLSGQGSTPG
DLLLSPHQGELAGLGLSEGQRPVFIRYDKEEAEDVEISLESDSDDSVVIFPRGMLMLENQ
DGISTVATLPVSSLVPGGVTLPVPGPGDDPGGDISLPLANDLPSTSLPHPLLPSSSAPNS

INSFPPAPLASLVPPLNSTGVTQLGAPSVGLGVGADSLPGAQLQQMLLQGQSPVPGQPTP
LGLPIQMLQNQLAQPSRALQQQQASEEDHSVININSSDEEEEEDEEMEDEDELGEEEDEE
GLEDEEEEEEGSDFPDEEEEFYGEEFDDYEEEEGEEEEEEEEEEEEEGEIRPLDREGRRG
GMGREEGEVLREVPEDGGMGGFCVEGEMEGGIEELGTNRRVYGEEGVKAQEVESIGVLEE
EREGEEDDADGMNDPTMPQILCVTGGALEEREELGEGHGQEVGSCEQQGADRPEATPSSE
GPTPQHKQEAEPAQEVRVGGSDQPSNQGEEPAKQGGDSAKEEVKPSAALIETEGEEPERE
KGGEEGEEDGEEDVRGMKRKREGEEEGTGQGTEKKKVRTLSQTIFNHSHSKPFS
> Phocoena sinus [A0A8C9E7A6]
MAAAVLSGPSAGSAAGVPGGTGGLSAVNSGPRLRLLLLESVSGLLQPRAGSTVSPVHHPT
RSVPHLPGLMCLLRLHGTVGGAQNLSAVGALVGLSNARLGSIKTRFEGLCLLSLLVGESP
TEMFQQHCVSWLRSIQQVLQSQDPPPTMELAVAVLGDLLRYAAQLPTLFRDISMNHLPGL
LTSLLGLRPESELSALEGMKACMTYFPRACGSLKGKLASFFLSRVDALSPQLQQLACECY
SRLPSLGAGFSQGLKHTDSWEQELHSLLASLHSLLGALYAGADTAPMQYEGPGVETLLSP
SEDGDAHVLLRLWQRFSGLARCLGLMLSSEFGAPVSVPVQEILDLICRTLSVSAKNISLL
GDGPLRLLLLPSLHLEALDLLSALILACRGRLLRFGALISRLLPQVLNAWSIGRDSLSPG
QERPYSTMRTKVYAVLELWVQVCGASAGVLQGGASGEALLTHLLSDISPPADALKLRSPR
GSPDGGLQTGKPSAPKKLKLDMGEALAPPSHRKGDSNANSDVCAAALRGLSRTVLMCGPL
IKEETHRRLHDLVLPLVMGVQQGEVLGSSPYTSSRCRRELYRLLLALLLAPSPRCPPPLA
CALQAFSLGQREDSLEVSAFCSEALVTCAALTHPRVPPLQTMGPACPTPAPVPPPEAPSP
FRAPAFHPPGPMPSVGPMPSVGPMPSAGPMPSPGPVPPAGPMPSVGPIPPARPGPPATAN
HLGLSVPGLVSVSPRLLPGPENHRAGSNEDAVLAPSGTPPPTIPPDETFGGRVPRPAFVH
YDKEEASDVEISLESDSDDSVVIVPEGLPPLPPPPPSGTTPPPAAPVGPPTASPPVPAKE
EPEELPAAPGPLPPPPPPPVPGPVALPPPQLVPEGTPSGGGPPALEEDLTVININSSEEE
EEDDEEDEDEEDFEEEEEEEEEEEYFEEEEEEEEEFEEEFEEEEGELEEEEEDEDEEEEV
EEVEFGPAGGEVEEGGPAPPSLPPALPPAASPKVQPQPEPEPGLLLEVEEPGAEEGPGAE
TAPTLAPEVLPSQGEVEREGGSPPAVPPPQELVEEEPSVPPTLLEEGAEGGGDKVPPPPE
ASAAEEMEAEAAALQEKEQDDTAAMLADFIDCPPDDEKPPPATEPDS
> Urocitellus parryii [A0A8D2GLW4]
MAAAVLSGPSAGSPAGVPGGTGGLSAMNSGPRLRLLLLESVSGLLQPRTGSTVAPVHPPV
CSVPHLPGLMCLLRLHGTVGGAQNLSALGALVSLSNARLGSIKTRFEGLCLLSLLVGESP
TEMFQQHCVSWLRSIQQVLQSQDPPPTMELAVAVLRDLLRYAAQLPTLFRDISINHLPGL
LTSLLGLRPECEQSALEGMKACMTYFPRACGSLKGKLASFFLSRVDALSPQLQQLACECY
SRLPSLGAGFSQGLKHTESWEQELHSLLASLHSLLGALYEGAETAPVQNEGPGVETLLSP
SEDGDAHVLLRLRQRFSGLARCLGLMLSSEFGAPVSVPVQEVLDLICRTLSVSGKNISLL
GDGPLRLLLLPSIHLEALDLLSALILACGGRLLRFGALISRLLPQVLNAWSIGRDTLSPG
QERPYSTIRTKVYAILELWVQVCGASAGVLQGGASGEALLTHLLSDISPPSDALKLRSPR
GSPDGGLQTGKPSAPKKIKLDVGEAMAPPSHRKGENNANSDVCAAALRGLSRTILMCGPL
IKEETHRRLHDLILPLVMGVQQGEVLGSSPYTSSCCRRELYRLLLALLLAPSPRCPPPLA
CALQAFSLGQREDSLEVSSFCSEALVTCAALTHPRVPPLQAMGPTCPTPAPVPPPEAPSP
FRAPPFQPPGPMPSVGPMPSAGPMPSAGPLTSSGSMPSVGPMPSAGPMPSAGPIPSAGPV
PSARPGPPATANHLGLSVPGLVSVPPRLLPGPENHRAGSNEDPVLAPSGTPPPTIPPDET
FGGRVPRPAFVHYDKEEASDVEISLESDSDDSVVIVPEGLPPLPPPPPSGTTPPPVAPTG
PPTASPPVPAKEEPEELPAAPGPLPPPPPPPVPGPVTLPPPQLVPEGTPGGGGPPALEED
LTVININSSDEEEEEEEEEEEEEEEEEEEEEFEEEEEEEEEYFEEEEEEEEEFEEEFEEE
EGELEEEEEEEEEEEEEELEEVEDLEFGSAGGEVEEGGPPPPTLPPALPPPESPKEHPEP
EPEPGLLLEVEEPGSEEQHGTETAPTLAPEVLPSQAEEEREGGSPEAGPPPQELVEEEPS
APPTLLEEGTEGGGDNVPPPPETAAEEEMETETEAAVLQEKDDTAAMLADFIDCPPDDEK
PPPTTEPDS
> Molossus molossus [A0A7J8D0P8]
MDLAVAVLRDLLRYAAQLPTLFRDISMNHLPGLLTSLLGLRPECELSALEGMKACMTYFP
RACGSLKGKLASFFLSRVDALSPQLQQLACECYSRLPSLGAGFSQGLKHTESWEQELHSL
LASLHSLLGALYEGAEPAPVQYEGPGVEMLFSPSEDGDAHNLLRLWQRFSGLACCLGLML
SSEFGAPVSIPVQEILDVICRTLSISAKNISLLGDGPLRLLLLPSIHLEALDLLSALILA
CGGRLLRFGALISRLLPQVLNAWSIGRDTLSLGQEKPYSAMRTKVYAVLELWVQVCGASA
GVLQGGASGEALLTHLLSDISPPADALKLRSPRGSPDGSLQTGKPSAPKKLKLDMGEAMA
PPSHRKGDSNANSDVCAAALRGLSRTILMCGPLIKEETHRRLHDLVLPLVMGVQQGEILG
SSPYTSSRCRRELYRLLLALLLAPSPRCPPPLACALQAFSLGQREDSLEVSSFCSEALVT
CAALTHPRVPPLQSMGPTCATPAPVPPPEAPSPFRAPPFHPPGPMPSAGTMPSAGTMPSA
GPVPSAGTMPSAGPLPPARPGPPATANHLGLSVPGLVSVPPRLLPGPENHRAGSNEDPVL
APSGTPPPTIPPDETFGGRVPRPAFVHYDKEEASDVEISLESDSDDSVVIVPEGLPPLPL
PPPSGTTPPPVTPAGPPAVSPPLPAKEEPEELPAVPGPLPPPPPPPVPGPVTLQPPQLVP
EGAPGGGGPPALEEDLTVININSSDEEEEEEEEEEEEEEEEEEEEDFEEEEEDEEEYFEE
EEEEEEFEEEFEEEEGELEEEEEEEDEEEEEELEEELEKEGGSPPAGPPPQELVEEEPCA
PPALLEEGTEGGGDEVPPPPEAPAAEEMDMETETETPALQEKEQDDTAAMLADFIDCPPD
DEKPPPDTEPDS
> Hipposideros armiger [A0A8B7T693]
MATAVLSGPSAGSAAGVPGGTGGLSSVGSVPRLRLLLLESVSGLLQPRPGSAIAPVHHHV
RSASHLPGLMCLLRLHGTVGGAQSLSAVGALVGLSNARLGSIKTRFDGLCLLSLLVAESS
TEMFQQHCVSWLRSIQQVLQSQDPPPTMELAVAVLRDLLRYAAQLPTLFRDISMNHLPGL
LTSLLGLRPECELSALEGMKACMTYFPRACGSLKGKLAAFFLSRVDSLSPQLQQLACECY
SRVPSLGAGFSQGLKHTESWEQELHSLLASLHSLLGALYEGAEPAPMQYEGPGVEMLLSP
SEEGDAHVLLRLWQRFSGLARCLGLMLSSEFAAPVSVPVQEILDIVCRTLSISAKNISLL
GDGPLRLLLLPSIHLEALDLLSALILACGGRLLRFGALISRLLPQVLNAWSIGRDALSPG
QEKPYSAMRTKVYAILELWVQVCGTSAGVLQGGASGEALLTHLLSDICPSADALKLRSPR
GSPDGSLQIGKPSAPKKLKLDMGEAVAPPSHRKGDSNANSDVCAAALRGLSQSILMCGPL
IKEETHRRLHDLVLPLVMGVQQGEVLGSSPYTSSRCRRELYRLLLALLLSPSPRCPPPLA
CALQALSLGQREDNIEVSSFCSEALVICSALIHPRVPPLQSMGPTCPTPAPVPPPEAPSP

FRTPPFHPPGPMPSAGPVPSVGPMPSLGPMPSAGPMPSVGSMPSAGPMPPARPGPPATTN
HLGLSVPGLVSVPPRLLPGPENHQAGSNEDPVLAPSSTPPPTITPDETFGGRVPRPAFVH
YDKEEASDVEISLESDSDDSVVIVPEGLPPLPPPTPSGTTPPPVAPAGPPIASPPLPAKE
EPEELPATPGPLPPPPPPPVPGPVTLPPPQLVPEGTPGGGGPPALEEDLTVININSSDEE
EEEEEEEEEEEEEEEEEDFEEEEEDEEEYFEEEEEEEEEFEEEFEEEEGELEEEEEEEEE
EEEELEEVEELEFGPAAGEVEEGGPPPPSLPPALPPAESPKVQPEPEPEPEPEPQPEPEP
EPAPGLLLEVEEPGAEEEHGAETAPTLAPEVLPSQGELEREVGSPPAGSPPQELVEEEPC
APPTLLEEETEVGGDNLPPPPESSATEEMETETDATDLQEKEQDDTAAMLADFIDCPPDD
EKPPPATEPDS
> Cricetulus griseus [A0A8C2L9X4]
MAAAVLSGPSAGSPAGAPGGTGGLSTVGSGPRLRLLLLESVSGLLQPRTGSPVAPVHPPI
HWAPHLPGLMCLLRLHGTVGGAQNLSALGALVNISNAHLGSIKTRFEGLCLLSLLVGESP
TELFQQHCVSWLRSIQQVLQSQDSPSTMELAVAILRDLLRYASQLPTLFRDISTNHLPGL
LTSLLGLRPECEQSALEGMKACVTYFPRACGSLKVNPWLSYFLSDQLSALFLQLACECYS
RLPSLGAGFSQGLKHTENWEQELHSLLASLHSLLGTLYEGTETDPVQSEGPGVEMLLSQS
EDGNTHVLLQLRQRFSGLARCLGLMLSSEFGAPVSVPVQEILDLICRILSISSKNINLLG
DGPLRLLLLPSIHLEALDLLSALILACGGRLLRFGALIIRLLPQVLNTWSTGRDTLAPGQ
ERPYSTIRTKVYAILELWVQVCGSSAGMLQGGASGEALLTHLLSDISPPTDALKLCATRG
SSDGGLPSGKPSAPKKLKLDMGEALAPASHRKGDRNANSDVCAAALRGLSRTILMCGPLI
KEETHRRLHDLVLPLVMSVQQGEVLGGSPYNSSCCRRELYRLLLALLLSPSPRCPPPLAC
ALKAFSLGQCEDSLEVSSFCSEALVTCSALIHPRVPPLQSSGPACPTPAPVPPPEAPSPF
RAPPFPPPGPMPSIGPIPSSGPLPSAGPIPTVAGPMPSTGLVPSRPGPPATANHLGLSVP
SLVSVPPRLLPGPENHRAGSSEDPVLAPSGTPPPSIPPDETFGGRVPRPAFVHYDKEEAS
DVEISLESDSDDSVVIVPEGLPSLPPPPPSGSPPPVAATGPPTASPPVPAKEDSEELPAT
PGPLPPPPPPPPPVSGPVTLAPPQLVPEGTPGGGGPTAMEEDLTVININSSDEEEEEEEE
EEEEDEDEEEEDFEEEEEDEEEYFEEEEEEEEEFEEEFEEEEEGELEEEEEEEEEEEELEE
VEDVEFGSAGGEVEEGGPPPPTLPPALPPTDSPKIQPEAEPEPGLLLEVEEPGAEEVPGP
ETAPTLAPEVLPSQEEVVQEGGSPPAGPPQELVEEESSAPPNLLEEGTEGGGDKVPPPPE
TPAETEMETEAEAASPQEKEQDDTAAMLADFIDCPPDDEKPPPDPEPDS
> Erpetoichthys calabaricus [A0A8C4X7N7]
MAATLQRSAGMRLTEGLLSVLRTDKREYVPVLLSSYREHGDIATQNASVMAGLVSLSNIR
LGSVKTRFEGLCIMAVMVKDSSTELFQQHCLTWLRSIQQIIQSQDPLQTMELAVHVLHDL
LQYSSQLPELARDIGLNSIPGILTSLLSLKPECHIAAIEGMHACMTFYPRACGSLRGKLG
AYFLSKMDSENTRMQELACSCYSILPTLGSGFSQGIKTSEAWAQQLQCILATAHSLLGQL
YEGAETDPVIYMGPGVELNFSPLDERDPLFILRLSQRYAGLCQSLSKLLSIDVTVPVKLP
VQDIVNLVCRALTVTHKKLNWLGDGPLKMLVLPAVHFRTLDVLIALIETGGSRLIQYSTM
LCHLFAQTLNAWSSTNDTILPGQQRAYSAVRVQVYKTVQIWVSVAGSSSGVLQGSAFSSE
VLLGHILSDITPGTDTIKLRTGKAMPELSNNVGKVGGKRTKGLDLHEATPHLQGYRKQES
NANSDCCKAALKSLSLIIINSGTLLKEETHKKLQELVVPLLVGFQQQGSLAANGTQTSPY
FSPENRKELYHVLLALLLVPSPRWPPPLQCAVRAFSLGLGDINIMVASFCREALAICNVL
IHPRTPSIAHSFSLTAASSTQQTMGKPGMAELLPHPQVPAAFRAGVTPPFIPPNPSSCPQ
PSGPLSLPSLLSSSNSFQARHPLNLPNSATAALPCSSLLGSLDNHLATDSAAQLSTTSDN
LVGTGLMDVLSPHHAGLEDSFSGATDGHKPVFVRYDKEEAEDVEISLESDSDDSVVIVPE
GLLIKQPAPQGITAQNQEGRLPEQDNVEALGSAGGTSPKGQANNITGAVPGIGMPAATDT
VLSTTSASSSVATSALANSFTSSITPLQTVPPTAVVEPQQAQYSSQQMLQQQSEDDSAVI
NINSSDDDEEEEEEEEEEEDDDDEYPEEEGFYEDDEEEEDYEEYEDEECLEDMEEETEEEE
EDGEVIDEDEEDLEDEEDLDHSEVLRTGHGTSFQLEHVEEEMTGLKARPCGRQEMQEDLY
SDPVDLEEEGPVEIDEGDQSLVEIRDGEQEDVMSSEPAIHTVQSDEQEPVKSSADDVAEK
PLIILPVISQEDESVLPQDTSEQEKEEGKSEIMQEVTLVKSVPDKSASPVRESQVQIVLE
TEVQEKVSSTPTTNSEPEHEQDQSVIKDQPEQDFTDSSLSEKHIKEEEVESKTDPEENVL
IQDETGKGSKKETETKQEEVSIKQAEVDLELRELETKDPEEEEQPIDERGLKRKREEEPT
AAENPDKKKLTEEDSTAAMLADFVDSPPDEDEATLLYSKDDCPPHEAEATLLYSKDDS
> Jaculus jaculus [A0A8C5KEL6]
MAAAVLSGPSAGSPAGVPGGAGGLSAAGSGQRLRLLLLESVSGLLQPRTGSPVAPVHPPL
RWAPHLPGLVCLLRLHGSVGGAQNLSALGALVSLSNARLGSVKTRFEGLCLLSLLVGESP
TELFQQHCVSWLRSIQQVLQSQDPPPTMELAVAILRDLLRYASQLPTLFRDISTNHLPGL
LTSLLGLRPECDQSALEGMKACVTYFPRACGSLKGKLASFFLSRVDALSPQLQQLACECY
SRLPSLGAGFSQGLKHTENWEQELHSLLASLHTLLGALYEGAETASVQNEGPGVETLLSH
SEDGDTHILLRLRQKFSGLARCLGLMLSSEFVAPVSMPVQEILDLICRILSVSGKNISLL
GDGPLRLLLLPSIHIEALDLLSALILACGGRLLRFGTLISRLLPQVLNAWSTGRDTLSPG
QERPYSTIRTKVYSILELWVQVCGASAGVLQGGASGEALLTHLLSDISPPADALKLHSPH
GSSDGGLQSGKPSAPKKLKLDMGEAMAPPSHRKGDINANCDVCAAALRGLSRTVLMCGPL
IKEETHRRLHDLVLPLVMSIQQQGEVLGSSPYNSSCCRRELYRLLLALLLAPSPRCPPPL
ACALQAFSLGQREDSLEVSSFCSEALVTCSALTHPRVLPLQTMGTACPTPAPVPPPEAPS
SFRAPPFHPPGPMPSIGPIASSGPVPSAGPMPSASPMPSTGPVPSRPGPPASANHLGLSV
PGLVSVPTRLLPGPENHRASSNEDPVLAPSGTPPPTVPSDETFGGRVPRPAFVHYDKEEV
SDVEISLESDSDDSVVIVPEGLPPLPPPPPSGSGTPPPPPVASTAPPTASPPVPAKEDSE
ELPATPGPLPPPPPPPHPGPGAVTLPPPQLVPEGAPAGGGPPALEEDLTVININSSDEEE
EEEEEEEEEEEEEEEEDFEEEEEDEEEYFEEEEEEEEEFEEEFEDEEGELEEEEEEEEEEE
EEELEEVEDVEFGSAEGEIEEGGPPPPTLPPALPPPDSPKVQPEPEPEPGLLLEVEEPGA
EKGSGAEMAPNLAPEVVPSQEEVEKEGGSPTAGSPPQELVEESSVPPTLVEEGTEGGGDE
VPPPPETPADEEMETEAETAALPTKEQDDTAAMLADFIDCPPDDEKPPPTTEPDS
> Naja naja [A0A8C6Y677]
MPGPGPVTWPPKQPIASAAPPSPPPPKPRSPASLLSASQPSRGWAGRGFGGGGGVPARRG
GARRGAGSGRGGLSSLWAGGVRVPLPGMPRAARRPPPRPPPAGAQTQSASGLAKHTMAAA
RRGSSSCSSPGGCGSSSSAGAASPSPPLAPSSGLALEALAGLLLPRRGASPPPPGLAGLV
RCARDCGGQAQVSGRLVGREAPARVFEGLCLLSLLVTESGSETFAQNCLAWLRSLQHLLQ

SQDPPATMELAVLVLRDLLQYSAQLPEVARDIGTNHIPGLLTSLLALKPECQLPVLEGCK
ACMTFYPRACGSLRGKLSTYFLSCMDAETPHLQQLACECYALLPSLGAGFAQGLKYRESW
EQQAHSLVATLHCLLGRLYEGAETEPLHYDGPGEEVLLPPREEEANSLLLAKCQFAGLAK
CLCQMLRNDFGAPVAVPAQAILDLVCRALDVSVKSMSWFGDGPLRMLLLPSIHLEALDLL
AALILACGPRLVRFGGALCRLFPQVLNMWRAGQDLPSPGLQRPYSAVRARLYQVLDLWVQ
VAGAASGVLLGHSSQSDALLGHLINDISPPSDALKVRPPPSPLGVSPTSEGKPSAAKKPK
LSPVGSLGCPFRKHDPQANSDVCLAALQEALSFCPQRLQELAIPLLIRLGQADLPLGSPY
ASASCRRELYRLLLALSLAPAPTCPPPLHCTVQLLSQGRTDPNLLVISRLTVAATLSPFC
PAVPVPFPALRPLPPGPGALPANSLGLPLSGLATPLPPPRLLPEEPLPPPSPGTAEAAAL
ASGAKLRRSVFVHYDKEEEEDVEISLESDSDDSVVIVPKGQLGKTTSAPNSAPVPVAAAA
PAPPPPPAPPAPPPPLPPSPLVACEEAPSEPPAPLALGIPPPPSAAQVLPSSPVAVATEA
PLPALLEDDPTVININSSEEEEEEEEEEEEEDFPEDEEFLDEEEEVRTGWGSSAGLGSKAR
PPSWCVRGVGVQLWWSPSAHPGLSLSKDGPWENSCSCVCVLCACFSSFSPPAGQEEEFDE
EEGDFEEMDEEDDDFDEEEEEGLTEEEEEEEEEEEEEEEGLPPSLPRRNEDELPALLPAVK
DGPLKLAEEEEEEEEDGGAAGLLMEVEEEAFQAPQEEQEEEEEEEEEEEESERQGPGRC
> Oncorhynchus kisutch [A0A8C7J7I5]
MAATAAWMHGPANMRLTEGLLSVLKEQRPEYLPALLANYREHGVVSTQSSAAVGGLVGLS
NAKLGNSKTRFEGLCLLSVLVKDSSSDVFQQHCLSWLRSLQQVIQSQAPLPSIQLAVGVL
QDLLQYSSQLPELAREVGLNSILGILTSLLGLKSEFHLAAMEGMTACMTFYPRACGSLRD
KLGACFLSKMDSVIPEVQEVACECYGRLPCLGGVLERGGGGRRAEGWTNQLHCLLASANG
MLAQLYQSTESEGMVPYEGPGVELPYPPLDDTDPLVLLQLQHRYRGVCLALKHTLGVDPA
SAVRLPVPQVLNLVCRALAVSSKSINVTGDGSVRLLVLPSIHNHTVKVLHALITAVGSGL
VQYSSMLQRLFSQTLSAWTPLPETNLGQQRAFSAVRVSLYRTLELWVKVGGASAGVLQGS
PTHSEILLAHLLGDITPGADSVRLRAGQSTVADLVSSKPCPKSRKPGLGMGNGGGASLQR
KGDSLANQDTCVSALRALRQIILTSGTLLKEDIHKRLHDVVLPLCVRLQQQHGGDCGAGG
VSGQYGSALPRRELYRLLLALVLVPPPRWPPPLTCTVSILSHGRRDRSLKVSSFCTEALT
ICNSLLHPRTPSLCLPLPPLTLKPSPATSLLTPSQASSLTLPTLLGGPFPGRHSLGLGHT
LLGSLDNHLSLVPPGLSGQGSTPGDLLLSPHQGELAGLGLSEGQRPVFIRYDKEEAEDVE
ISLESDSDDSVVIFPRGMLMLENQDGISTVATLPVSSLVPGGVTLPVPGPGDDPGGDISL
PLANDLPSTSLPHPLLPSSSAPNSINSFPPAPLASLVPPLNSTGVTQLGAPSVGLGVGAD
SLPGAQLQQMLLQGQSPVPGQPTPLGLPIQMLQNQLAQPSRALQQQQASEEDHSVININS
SDEEEEEDEEMEDEDELGEEEDEEGLEDEEEEEEGSDFPDEEEEFYGEEFDDYEEEEGEE
EEEEEEEEEEEGEIRPLDREGRRGGMGREEGEVLREVPEDGGMGGFCVEGEMEGGIEELG
TNRRVYGEEGVKAQEVESIGVLEEEREGEEDDADGMNDPTMPQILCVTGGALEEREELGE
GHGQEVGSCEQQGADRPEATPSSEGPTPQHKQEAEPAQEVRVGGSDQPSNQGEEPAKQGG
DSAKEEVKPSAALIETEGEEPEREKGGEEGEEDGEEDVRGMKRKREGEEEGTGQGTEKKK
VRTLSQTIFNHSHSKPFS
> Piliocolobus tephrosceles [A0A8C9LZW5]
MELAVAVLRDLLRYAAQLPALFRDISMNHLPGLLTSLLGLRPECEQSALEGMKACMTYFP
RACGSLKGKLASFFLSRVDALSPQLQQLACECYSRLPSLGAGFSQGLKHTESWEQELHSL
LASLHTLLGALYEGAETAPVQNEGPGVEMLLSSEDGDAHVLLRLRQRFSGLARCLGLMLS
SEFGAPVSVPVQEILDFICRTLSVSSKNISLHGDGPLRLLLLPSIHLEALDLLSALILAC
GSRLLRFGILISRLLPQVLNSWSIGRDSLSPGQERPYSTVRTKVYAVLELWVQVCGASAG
MLQGGASGEALLTHLLSDISPPADALKLRSPRGSPDGSLQTGKPSAPKKLKLDVGEAMAP
PSHRKGDSNANSDVCAAALRGLSRTILMCGPLIKEETHRRLHDLVLPLVMGVQQGEVLGS
SPYTSSRCRRELYCLLLALLLAPSPRCPPPLACALQAFSLGQQEDSLEVSSFCSEALVTC
AALTHPRVPPLQPMGPTCPTPAPVPPPEAPSPFRAPPFHPPGPMPSVGPMPSAGPMPSVG
PMPSAGPVPSARPGPPTTANHLGLSVSGLVSVPPRLLPGPENHRAGSNEDPILAPSGTPP
PAIPPDETFGGRVPRPAFVHYDKEEASDVEISLESDSDDSVVIVPEGLPPLPPPPPSGAT
PPPIAPTGPPTASPPVPAKEEPEELPAAPGPLPPPPPPPPPVPGPVTLPPPQLVPEGTPG
GGGPPALEEDLTVININSSDEEEEEEEEEEEEEEDEEEEEEDFEEEEEDEEEYFEEEEEEE
EEFEEEFEEEEGELEEEEEEEDEEEEEELEEVEREEGSPSAGPPPQELVEEEPSAPPTLL
EEGTEDGGDRVQPPPETPAEEEMETETEAEALQEKEQDDTAAMLADFIDCPPDDEKPPPP
TEPDS
> Theropithecus gelada [A0A8D2FMT2]
MLLPSLSPRVQPAFIQCAGPQPPPHAHSPVGGRRGVFASSRHATTGARGKMAAAVLSGPS
AGSAAGVPGGTGGLSAVNSGPRLRLLLLESVSGLLQPRTGSAVAPVHPPNRSAPHLPGLM
CLLRLHGSVGGAQNLSALGALVSLSNARLSSIKTRFEGLCLLSLLVGESPTELFQQHCVS
WLRSIQQVLQGKLASFFLSRVDALSPQLQQLACECYSRLPSLGAGFSQGLKHTESWEQEL
HSLLASLHTLLGALYEGAETAPVQNEGPGVEMLLSSEDGDAHVLLRLRQRFSGLARCLGL
MLSSEFGAPVSVPVQEILDFICRTLSVSSKNISLHGDGPLRLLLLPSIHLEALDLLSALI
LACGSRLLRFGILISRLLPQVLNSWSIGRDSLSPGQERPYSTVRTKVYAVLELWVQVCGA
SAGMLQGGASGEALLTHLLSDISPPADALKLRSPRGSPDGSLQTGKPSAPKKLKLDVGEA
MAPPSHRKGDSNANSDVCAAALKGLSRTILMCGPLIKEETHRRLHDLVLPLVMGVQQGEV
LGSSPYTSSRCRRELYCLLLALLLAPSPRCPPPLACALQAFSLGQREDSLEVSSFCSEAL
VTCAALTHPRVPPLQPMGPTCPTPAPVPPPEAPSPFRAPPFHPPGPMPSVGPMPSAGPMP
SAGPMPSAGPVPSARPGPPTTANHLGLSVSGLVSVPPRLLPGPENHRAGSNEDPILAPSG
TPPPAIPPDETFGGRVPRPAFVHYDKEEASDVEISLESDSDDSVVIVPEGLPPLPPPPPS
GATPPPIAPTGPPTASPPVPAKEEPEELPAAPGPLPPPPPPPPPVPGPVTLPPPQLVPEG
TPGGGGPPALEEDLTVININSSDEEEEEEEEEEEEEEEEEEEEEEEDFEEEEEDEEEYFEEE
EEEEEEFEEEFEEEEGELEEEEEEEDEEEEEELEEVEELEFGTAGGEVEEGGPPPPTLPP
ALPPPESPPKVPPEPEPEPGLLLEVEEPGAEEEHGADTAPTLAPEVLPSQGEVEREEGSP
SAGPPPQELVEEEPSAPPTLLEEGTEDGGDRVQPPPETPAEEEMETETEAEALQEKEQDD
TAAMLADFIDCPPDDEKPPPPTEPDS
> Theropithecus gelada [A0A8D2FP48]
MELAVAVLRDLLRYAAQLPALFRDISMNHLPGLLTSLLGLRPECEQSALEGMKACMTYFP
RACGSLKGKLASFFLSRVDALSPQLQQLACECYSRLPSLGAGFSQGLKHTESWEQELHSL

LASLHTLLGALYEGAETAPVQNEGPGVEMLLSSEDGDAHVLLRLRQRFSGLARCLGLMLS
SEFGAPVSVPVQEILDFICRTLSVSSKNISLHGDGPLRLLLLPSIHLEALDLLSALILAC
GSRLLRFGILISRLLPQVLNSWSIGRDSLSPGQERPYSTVRTKVYAVLELWVQVCGASAG
MLQGGASGEALLTHLLSDISPPADALKLRSPRGSPDGSLQTGKPSAPKKLKLDVGEAMAP
PSHRKGDSNANSDVCAAALKGLSRTILMCGPLIKEETHRRLHDLVLPLVMGVQQGEVLGS
SPYTSSRCRRELYCLLLALLLAPSPRCPPPLACALQAFSLGQREDSLEVSSFCSEALVTC
AALTHPRVPPLQPMGPTCPTPAPVPPPEAPSPFRAPPFHPPGPMPSVGPMPSAGPMPSAG
PMPSAGPVPSARPGPPTTANHLGLSVSGLVSVPPRLLPGPENHRAGSNEDPILAPSGTPP
PAIPPDETFGGRVPRPAFVHYDKEEASDVEISLESDSDDSVVIVPEGLPPLPPPPPSGAT
PPPIAPTGPPTASPPVPAKEEPEELPAAPGPLPPPPPPPPPVPGPVTLPPPQLVPEGTPG
GGGPPALEEDLTVININSSDEEEEEEEEEEEEEEEEEEEEEEEDFEEEEEDEEEYFEEEEEE
EEEFEEEFEEEEGELEEEEEEEDEEEEEELEEVEREEGSPSAGPPPQELVEEEPSAPPTL
LEEGTEDGGDRVQPPPETPAEEEMETETEAEALQEKEQDDTAAMLADFIDCPPDDEKPPP
PTEPDS
> Varanus komodoensis[A0A8D2LH15]
MELAVLVLRDLLECSCQLPELARDISTNHVPGLLTSLLALRPECQLSALEGSKACMMFYP
RACGSLRGKLAAYFLSRVDAETPQVQQLACECYALLPSLGAGFTQGLKYTECWEQQAHCL
LATLHSLMGTLYEGAETDPLHYEGPGMEIPLPAPEEGETNFVLHLKQRFSGLAKCLCRML
SNEFVAPVTVPVQDILDFICRALDISMKNISWFGDGPLRMLFLHSVHLESLDLLFALILA
CGPRLVRFGGTLCRLFPQVLTAWSAGRDLVPAGQERPYSAVRTRLYQVLDLWAQVAGAAS
GLLQGHGTQSEALLGHLLSDISPPTDTLKLRESAPGPDRRPSAPKKPKLAGLGPLHHKHD
PRANSDVCLAALQGQEPLGAAGRRGWASREQGGAPSRPPPRRPALASSSGGGWPPLLQVS
SFCSEALVVCNALLHPRVPALQVPLPGPPAPPPGSSSEISPATASPFRSAPPLPFPPPRL
PPPASPPAPPSAAPPAGSLGLPLPPLALAQLPPRLAPEEPAVLPSPGGAAEAAALAAGAK
LRRSVFIHYDKEEEEDVEISLESDSDDSVVIMPKGQLGRGPGGAGAVAAVAAAPAPLPPP
PPTACPRGSWGWGWAWAGGSRCLRLSDQEEEFEEDEGEFDELEEEIEEMDEEEEEEDEFD
EEECMTEEEEEEEEEEEEEEEEEGLTEEEEEGELPKLVGEEEGGEGEEEEEGAGLLMEVEE
EAFHAPEDGSGGQEGEEEAAAGTREVGRTAGDALLRKAEEPPVPTSAVEELLPPPLPLPP
SPAQEPPTLPATAFTDPEELAEEQERPMAGGPPFAAAPQDPEGERGPAGHAGGPAEAGPE
ARAPQGEAEEQLPALGGHVPEEEPEVRAWGGQAGGRAGSPATPGCPMASAAAWRHWKPQL
SCPVLGLPAPASGAPAALAEGESDPQPGPLGLGAGRRWAGGPREGVS
> Canis lupus familiaris [A0A8I3P2E1]
MAAAVLSGPSAGSAAGVPGGAGGLSAVGSGPRLRLLLLESVSGLLQPRAGSAVAPVHPPV
RSAPHLPGLMCLLRLHGTVGGAQNLSAVGALVGLSNARLGSIKTRFEGLCLLSLLVGESP
TEMFQQHCVSWLRSIQQVLQSQDPPPTMELAVAVLRDLLRYAAQLPTLFRDISMNHLPGL
LTSLLGLRPECELSALEGMKACMTYFPRACGSLKGKLASFFLSRVDALSPQLQQLACECY
SRLPSLGAGFSQGLKHTESWEQELHSLLASLHSLLGALYEGAETAPVQYEGPGVEVLLTP
SEDGDTHVLLRLRQRFSGLARCLGLMLSSEFGAPVSVPVQEILDVICRTLSISAKNISLL
GDGPLRLLLLPSIHLDALDLLSALILACRSRLLRFGALISRLLPQVLNAWNLGRDTLAPG
QERPYSTVRTKVYAVLELWVQVCGASAGMLQGGSSGEALLSHLLSDISPPADTLKLRSPR
GSPDGGLQTGKPSAPKKLKLDVGEAMAPPSHRKGDSNANSDVCTAALRGLSRTILMCGPL
IKEETHRRLHDLVLPLVMGVQQGEVLGSSPYTSSRCRQELYRLLLALLLAPSPRCPPPLA
CALQAFSLGQREDSLEVSSFCSEALVTCAALTHPRVPSLRSMGPACPTPAPAPPPEAPSP
FRAPPFHPPGPMPSAGPMPSVGPMPSVGPMPPAGPMPPAGPMAPTRPGPPATANHLGLSV
PGLVSVPPRLIPGPENHRAGSNEDPVLAPSGTPPPAIPPDETFGGRVPRPAFVHYDKEEA
SDVEISLESDSDDSVVIVPEGLPPLPPPPPSGTTPPPVAPAGPPVASPPVPAKEEPEELP
VAPGPLPPPPPPPVPGPVALPPPQLVPEATPGGGGPPALEEDLTVININSSDEEEEEEEE
EEEEEEEEEEEEEEEEDFEEEEEEEEYFEEEEEEEEEFEEEFEEEEGELEEEEEEEEDEEE
EEELDELEEVAFGPAAGAAEEGGPPPPSPPPALPPAQSPKMQPEPPGETGLLLEVEEPAA
EEEPGAEAAPTLAPEVLPPQGEGPREVGSPPAVPPPQELIEEEPPAPPTLLEEGTESGGD
KVPVPQETPAAEDVEAEVEAETAALQEKEQDDTAAMLADFIDCPPDDDKPPPATEPDS
> Sus scrofa [A0A8W4F864]
MCLLRLHGTVGGAPNLSAVGALVGLSNARLGSIKTRFEGLCLLSLLVGESPTEMFQQHCV
SWLRSIQQVLQSQDPPPTMELAVAVLKDLLRYAAQLPTLFRDISMNHLPGLLTSLLGLRP
ECELSALEGMKACMTYFPRACGSLKGKLASFFLSRVDALSPQLQQLACECYSRLPSLGAG
FSQGLKHTESWEQELHSLLASLHGLLGALYEGAETAPMQYEGPAVEALLSPSEDGDAHVL
LRLRQRFSGLARCLGLLLSSEFGAPVSVPVQEVLDVICRTLSVSARNISLLGDGPLRLLL
LPSLHLEALDLLSALILACGGRLLRFGALISRLLPQVLSAWSIGRDTLSPGQERPYSTMR
TKVYAVLELWVQVCGASAGVLQGGASGEALLTHLLSDISPPADALKLRSPRGSPDGGLQT
GKPSAPKKLKLDMGEPIAPPSHRKGDSNANSDVCAAALRGLSRTILMCGPLIKEETHRRL
HELVLPLVMGVQQGEVLGSSPYTSSRCRRELYRLLLALLLAPSPRCPPPLACALQAFSLG
QREDSLEVSSFCSEALVTCAALTHPRVPTLQSMVPTCPTPAAVPPPEAPSPFRAPAFHPP
GPMPSAGPMPSGPMPSAGPMPSVGPMPPARPGPPATANHLGLSGSSLVSVPPRLLPGPEN
HRAGSNEEPVLAPSGTPPPTLPSDETFGGRVPRPAFVHYDKEEASDVEISLESDSDDSVV
IVPEGLPPLPPPPPSGTTPPPVAPAGPPAASPPVPAKEEPEELPAAPGPLPPPPPTPVPG
PATLPPPQLVPEGTPGGGGAPALEEDLTVININSSDEEEEEEEEEEEEEEEEEEEEEEDFE
EEEEDEEEYFEEEEEEEEEEFEEEFEEEEGELEEEEEDEEEEEEEEEELEEAEREAGSPPTAP
PPQELVEEEPSVPPTLLEEGAEGGGDKVPPPPETSAAEEMETETESTALQEKEQDDTAAM
LADFIDCPPDDEKPPPAAEPDS
> Cyprinus carpio carpio [A0A9J8AKL6]
MASAAWLHGPNITRLTEGLVSVLKEDRPEYLPALLANYREHGVVGTQSTGAVGGLVGISN
ARLGSSKTRFEGLCLLSVLVKDSSSEVFQQHCLSWLRTLQQVIQSQAPLPTVQLAVSVLQ
DLLQYSSQLPELAREVGLNSILGILTSLLSLKSECHLAAMKGMMTCMIYYPRACGSLREK
LGAYFLSKMDSDNPKVQEVACESFGRLPCLGGVLERGGGGRRAEGWTNQLHCLLASANSM
LGQLYQGAETEGTVQYEGPGVELPFPPLDDVDPLLILQLHHRYKAICLAIKHTLSADPAS
SVCLPVQHVLKLVCQALAVNTKSISPTGEGCLKLLVLPSIHIDTLELLSALIKAVGGGLV
QYSSVLTRLLSQSLSAWTPLPEASLGQQRAYSAVRVTVYRTIELWVRVGGASLLQGSPTH

TELLFTHLMGDITPASEAVKLRSGQQSQSMSDLIGSAGKSGPRRTKGLVMGDGISLQRKG
DVLANQDTCVAALRALRQIILTSGTLLKEDLHKRIQDLVVPLCVRLQQQSHCVLDVGAVS
GQYGSPTPRRELYRLLLALVLVPSPRWPPPLSCAVSVFSHGRRDHNIMVSSFCAEALTIC
NTLIHPRTPSISLPLTPLTLKSTPSAPVLASGQNPSLSIPTLLGGPPFPSRHPMGLGPAT
LLGSLENHLPLAPPGLPTPAGTTATPGDLLLSPAQPGELAGLGAPEGQRQVFVRYEKEEP
EDVEISLESDSDDSVVIMPAGMMMEMQDGAANAQSLSQPAVPAPIVGEVGLVDTLLPNEL
PTSIPHQILPANANNINSFPGPSQTAQLVSLVPPLNSTTASLSASPAGLADSLTGGPQLQ
QMLMQTSPGGQPPTLGLSLQIQLQNQIAQTSRQLQQQHPANEVDQNVININSSDDEEEEL
EEDELGEEEEEEEGLEEEEEEEEGSDLMDEEYYEGEEFDEYEDEEEEEESEEIQPLEGDN
SRGMMDEEEAEVMIEAEDQQGMEMFCMEREREVEPGIEEMEGVRSVYADDRIKDKGTVEE
IENIGAVERNEPVVGKEQIESLVISGETEGHEEDTSVEAVEPEVKTCEQEVAKPEDPTEN
AGPSQQGQELTVEVEVQKQEPELQPEDTTNQSAPSTSKQEALQSVAVTAEEEEEVEKESG
EQGEDSDARGTKRKMEDREEGESSEQGTEKKKVDEAMASMLADFVDCPPDDDDHGASQSQ
S
> Polypterus senegalus [A0A8X7XMS0]
MAGLVSLSNIRLGSVKTRFEGLCIMAVMVKDSSTELFQQHCLTWLRSIQQIIQSQDPLQT
MELAVHVLHDLLQYSSQLPELARNIGLNSIPGILTSLLSVKPECHIAAMEGMHACMTFYP
RACGSLRGKLGAYFLSKMDSENTRMQELACSCYSILPTLGSGFSQGIKTSEAWAQQLQCI
LATAHSLLGQLYEGAETDPVIYMGPGVELNFSPLDERDPLFILRLSQRYAGLCQSLSKLL
SIDVTVPVKLPVQDIINLVCRALAVTRKKLNWLGDGPLKMLVLPTIHFRTLDVLIALIET
GGSRLIRYSTVLCHLFAQTLNAWSFTNDTILPGQQRAYSTVRVQVYKTVQIWVSVAGSSS
GVLQGSANSSEVLLGHILSDITPGTDTIKLRTEKAMPELSNNIGKVGGKRTKGLDVHEAT
SHMQGYRKQESNANSDCCKAALKSLNLIIINSGTLLKEETHKKLQELVVPLLVGFQQHGS
LAANGTQTSPYFSPENRKELYHVLLALLLVPSPRWPPPLQCAVRAFSLGLGDINIMVASF
CREALAICNVLIHPRTPSIAHSFSLTAAASTQQTMGKPGMAELLPHPQGPTAFRAGVTPP
FIPPNPSSCPQPSGPLSLPSLLSSSNSFQARHPMNLPNSATATLPCSSLLGSLENHLATD
SAAQLSTTSDNLVGTGLMDVLSPHHAGLEESFSGAADGHKPVFVRYDKEEAEDVEISLES
DSDDSVVIVPEGLLIKQPAPQSIAAQNQEGRLLEQDNVEGVGSAGGTSPKGQANNITGAV
PGIGMPSATDTVLSTTSASSSVVASALANSFTSSITPLQTVPPTAVVEPQQAQYSSQQML
QQQSEEDSAVININSSDDDEEEEEEEEEEDDEEYPEEEGFYEDDEEEEDYEEYEDEECLE
DLEEESEEEEEDGEMIDDDEEDLEDEEDLDHSEVMRTGHGTSFKLEHVEEEMTGLKARPC
GRQEMQEDLYSDPVDLEEEGPVEIDEGDHSLVEIRDGEQEEVMSSEPAIHTVQSDEQEPM
KSSVDDLVEKPLITLPVVSQEEESVLSQEDTSEQEKEEGKSEIMQEVTFIKNVPDKSASP
VRESQVQIVLETEVQEKVSSTSTANSEPEHEQDQSVIKDQPEQDFADSSLSEKHIKEDVE
SKTDAEENVLIQDETGKGSKKDTETKQEEVSIKQAEVDIELRELEAKDPEEEEQPVDERG
LKRKREEEPTADENPDKKKLTEEDSTAAMLADFVDCPPDEEEATPSFSKDDS
> Camelus bactrianus [A0A9W3EL22]
MELAVAILRDLLRYAAQLPTLFRDISMNHLPGLLTSLLGLRPECELSALEGMKACMTYFP
RACGSLKGKLASFFLSRVDALSPQLQQLACECYSRLPSLGAGFSQGLKHTESWEQELHSL
LASLHSLLGALYEGAETAPMQYEGPGVETLLSPSEDGDAHVLLQLRQRFSGLARCLGLML
SSEFGAPVSVPVQEVLDVICRTLSISAKNIFSPQSLLGDGPLRLLLLPSLHLEALDLLSA
LILACGGRLLRFGALISRLLPQVLNAWSIGRDALSPGQERPYSTVRTKVYAVLELWVQVC
GASAGVLQGGASGEALLTHLLSDIAPAADALKLRSPRGSPDGGLQTGKPSAPKKLKLDVG
EAMAPPSHRKGDSNANSDVCAAALRGLSRTVLMCGPLIKEETHRRLHDLVLPLAMGVQQG
EVLGSSPYTSARCRLELYRLLLALLLAPSPRCPPPLACALQAFSLGQREDSLEVSSFCSE
ALVTCAALTHPRVPPLQSMGPACPTPAPVPPPEAPSPFRAPAFHPPGPMPSVGPMPSASP
MPSAGPMPSAGPVSSAGPMPSVGSMPPARPGPPATANHLGVSVPGLVSVPPRLLPGSENH
RASSNEDPILAPSGTPPPTVPPDETFGGRVPRPAFVHYDKEEASDVEISLESDSDDSVVI
VPEGLPPLPPPPPSGTTPPPVAPAGPPRASPPVPAKEESEELPATPGPLPPPPPPPVPGP
VTLPPPQLVPEGTPGGGGAPALEEDLTVININSSDEEEEEEEEEEEEEEEEEEEEDFEEEE
EDEEEYFEEEEEEEEEEFEEEFEEEEGELEEEEEEEEDDEEEEEELEEVEELEFGPAGGEVEE
GGPPPPSLPPALPPAESPKVPPEPEPEPEPGLLLEVEEPGAEEGPGAETAPTLAPEVLPPQG
EVQGEEGSPPAVPSPQELGEEEPSAPPTLLEEGAEGVGDQVPPQPEASAAEEMEMETEAA
ALQEKEQDDTAAMLADFIDCPPDDENPPPAPEPDS
> Stegastes partitus [A0A9Y4K793]
MATSTWLHGPSAMRLTEGLVSVLKEQRPEYLPALLANYREHGVFTTQGAGAVGGLVGFSN
AKLGSSKTRFEGLCLLAMLVKDSSSDLFQQHCLSWLRSLQQVIQSQAPVQTIQLAVNILK
DLLQYSSQLPELAREVGLNSVLGILTSLLGLKTECELAAMEGMTACMSYYPRACGSLRDK
LGAYFLSKMDSTNRKTQEVACQCYGRLPCLGGLLDRGVGAGRAEGWTNQIHCLLATANGL
LAQIYQDGTVQYQGPGVELTFPHLDQTDPLLLLQLQHRYTAVCLALKHTLRVDPASAVRL
PVRPILNLVCRALAVSSKSINLTGDGSVRLLVLPIIHTNTLEVLSSLITAVRRGMVQYAA
VLQRLFSQTLSAWMPLPEASVGQQRAYSSVRVSVYATLELWVQVAGASANILQGSPSHSE
LLFSHLLGDITPGAESIKLRAGLSADVVPGGKPGPRRTKQLVMADAVGPSLQRKGDLLAN
QDTCLSALRGLRQIIQTSGTLLKDDIHKRLHDVVLPLCVRLQQQQCSTSTACESAGGVSG
QYSSAITRRELYRLLLALVLVPSPCWPPPLTCAVSILSNGRTDRNLKVSSFCAEALTICN
SILHPRTSSVALPLPPLTLKPTHTAPVLPSSQGPASGLTLPTLLGGPAPGPPFPTRHSLG
LGPTSLLGSLENHLSLVPGLPGQAPAPGDMILSPHAHHQPDPAGLGPPEGQRPVFVRYDK
EEAEDVEISLESDSDDSVVIVPPGMLNMENQQDDVAANSQNILSAAPGGAGVTLSGGESV
TMVPTTAATTTVEGTSLSNDLATSSPLLTTSTTPINSFPPSSASVVSLVPPLNSSTLTGP
PGGLGDSMPGRPQLQQMLMQPSTAGQPSPMGLPLQMHQLQNQLSQQGRHLHQHPPAPTSN
EDSAVININSTDDEEEDEEDMEDDEELEEEEEGMDEEDEEEEVSDLAEDEFYDGEEYEDY
DEEEGEDLEEEEEEEEDGDIPPLEGAEDKVVEAGIEEGKVLRAAVDEGGMSGFSVEGDAE
GGIEEIQTNRALFGDDRMKVQEVESIGVLEDAREGEGEEDDSEKMNDPTMPQILCVTGGA
LDEREETEEEGGEAGGGLEEGESSWEQGGKEEEELTAAPEEQSTNQTQQESAAEPAQEVS
VSDNQPSSHQEEQLVAVQEEDTPADPETSADLSTKEQEETVDLKRDKTDTEQQEAAGTEE
ESDGEEGKGVKRKREEGQEEEEAGQSTDKKKMDDEAMASMLADFVACPPDDEDGASGSNC
S

> Sciurus carolinensis [A0AA41T5K0]
MAAAVLSGPSAGSPAGAPGGTGGLSAMSSGPRLRLLLLESVSGLLQPRTGSTVAPVHPPV
RSVPHLPGLMCLLRLHGTVGGAQNLSALGALVSLSNARLGSIKTRFEGLCLLSLLVGESP
TEMFQQHCVSWLRSIQQVLQSQDPPPTMELAVAVLRDLLRYAAQLPTLFRDISINHLPGL
LTSLLGLRPECEQSALEGMKACMTYFPRACGSLKGKLASFFLSRVDALSPQLQQLACECY
SRLPSLGAGFSQGLKHTESWEQELHSLLASLHSLLGALYEGAETAPVQNEGPGVETLLSH
SEDGDAHVLLRLRQRFSGLARCLGLMLSSEFGAPVSVPVQEVLDLICRTLSVSGKNISLL
GDGPLRLLLLPSIHLEALDLLSALILACGGRLLRFGALVSRLLPQVLNAWSIGRDTLSPG
QERPYSTIRTKVYAVLELWVQVCGASAGMLQGGASGEALLTHLLSDISPPADALKLRSPR
GSPDGGLQTGKPSAPKKIKLDVGEAMAPPSHRKGENNANSDVCAAALRGLSRTILMCGPL
IKEETHRRLHDLVLPLVMGVQQGEVLGSSPYTSSCCRRELYRLLLALLLAPSPRCPPPLA
CALQAFSLGQREDSLEVSSFCSEALVTCAALTHPRVPPLQAMGPTCPTPAPVPPPETPSP
FRAPPFHPPGPMPSVGPMPSVGPLPSAGPLPSSGSMPSVGPMPSTGPMPSAGPIPSAGPV
PSARPGPPATANHLGLSVPGLVSVPPRLLPGPENHRAGSNEDPVLAPSGTPPPTIPPDET
FGGRVPRPAFVHYDKEEASDVEISLESDSDDSVVIVPEGLPPLPPPPPSGTTPPPVAPTG
PPIASPPVPAKEEPEELPAAPGPLPPPPPPPVPGPVTLPPPQLVPEGTPGGGGPPALEED
LTVININSSDEEEEEEEEEEEEEEEEEEEEEDFEEEEEDEEEYFEEEEEEEEEFEEEFEE
EEGELEEEEEEEEEEEEEEELEEVEDLEFGSAGGEVEEGGPPPPTLPPALPPPESPKEQP
EPEPEPGLLLEVEEPGAEEQHGTETAPTLAPEVLPSQGEEEREGGSPDAGPPPQELVEEE
PSAPPTLLEEGTEGGDDRVPPPPETSAEEEMETEAEAAVLQEKEQDDTAAMLADFIDCPP
DDEKPPPTTEPDS
> Ovis ammon polii [A0AAD4Y3Q2]
MAAAVLSGPSAGSAAAVPGGAGGLSAVGSGPRLRLMLLESVSGLLQPRTGSAVAPVHPPA
RSAPHLPGLMCLLRLHGTVGGAQNLSAVGALVGLSNARLGSIKTRFEGLCLLSLLVGESP
TEMFQQHCVSWLRSIQQILQSQDPPPTMELAVTVLKDLLRYAAQLPAVFRDISMNHLPGL
LTSLLGLRPECELSALEGMKACMTHFPRACGSLKGKLASFFLSRVDALSPQLQQLACECY
SRLPSLGAGFSQGLKHTDSWEQELRSLLASLHSLLGGLYEGAEAAPMQYESPGAETLLSP
SEDVDAHTLLRLRQRFSGLARCLGLMLSSEFGAPVSVPVQDILDLICRTLSVSAKNVSLL
GDGPLRLLLLPSLHLEALDLLSALILACGARLLRFGALISRLLPQVLSAWSIGRENLGPG
QERPYSTVRTKVYAVLELWVQVCGASAGVLQGGASGEALLSHLLSDISPPADALRLRSPR
GSPDVGLQTGKPSAPKKLKLDVGEAIAPPSHRKGDSNANSDVCAAALRGLSRTILMCGPL
IKEETHRRLHELVLPLVMGVQQGEALCSSPYTSSHCRRELYRLLLALLLAPSPRCPPPLA
CALRAFSMGQQEDSLEVSSFCSEALVTCAALTHPRVPPLQSVGPTCPAPAPVPPPEAPAP
FRAPAFHAPGPLPSAGPMPSAGPMPPAGPLPTARPGPPATANHLGLSVPGLVSVPPRLLP
GPENHRAGSSEDPVLAPSGSPPPTVPPDETFGGRVPRPAFVHYDKEEASDVEISLESDSD
DSVVIVPEGLPPPPPPPPSGTTPPPVAPAGPPAASPPVPAKDEPEELPAAPGPLPPPPPP
VPGPVTLPPPQLVPEGTPGGGGPPALEEDLTVININSSDEEEEEEEEEEEEDEEEDEEED
FEEDEEEEEYFEEEEEEEEEFEEEFEEEEGELEDEEDEEEDEELEELEEVEFGPAGGEVE
GGPAPPSLPPVLPPAESPKGPPEPGLEPGLLLEVEEPGTEEAPGPEVAPMLAPEVLPSQG
EVEREGGSPPAGPPPQELVEEEPSGPPTLLEEGAEGGGDKLSPPPEASAVEEMEVEAAAL
PPEKEQGDTAAMLADFIDCPPDDEKPPPASEPDS
> Nothobranchius kuhntae [A0A1A8IS81]
MAASAWLHGSAAMRLTEGLVLLMKEQRPEFLPEVLANYREHGVLSSQSASDVAGLVGLCN
GKLNSSKTRLEGLCLLSMLVKDSSGDLFQQHCLSWLRSLQQVVQSQAPVQTIQLAVNVLK
DLLQYSCQLPELAREVGLNSILGILTSLLGLKTECELSAMEGMKACMVYYPRACGSLKDK
LGAYFLSKMDSTSRKTQEAACQGYSHLPCLGGLMDRAVGASRAEGWTNQIHCLLASANSL
LAQIYQGSETDEAVRYQGPGVELAFPHIDQTEPLLLLQHRYAAVCMTIKHTLRTDPASAV
SVPVRPILNLVCRALAVNSKNMILTADGSLRFLVLPSVHINALEVLSELITVVRSGMVQY
AAVIQRLFSQTLSAWTPLPETSVGQQRAYSSVRVSVFRSLELWVRVAGASASILHGTSSH
VDLLLNHLLGDITPGAESVKLRAGLSADVVPVGKPGPRRTKQLVVADAVGPSLQRKGDLL
SNQDTCLAALKALRQVLQSTGMLLKDDIHKRLHEVVLPLCVRLQQQQSSSISAWEPAGGV
SGQYSSALTRRELYRVLLALVLVPSPSWPPPLTCAVSILSNGRIDRHLKVSSFCAEALTI
CNSILHPRRPSLALPLPPLTLKPAATPSVLSCQGPGPRLTLPTLLGGPSSAPPFPARHTL
NLGPSSLLGSLDNHFSLVPGLPGQAPGPGDLMASSQTHHQPDPSGLAPPEGQRPVFVRYD
KEETEDVEISLASDSDDSVVIVPPGMLNVEKQQNDLAANSQSLLSSAPGGAELVTMVPTS
STPTTLDVTSLPNDLATSSALLTSSVTPINSFPPSSTSVVSLVPSVNSSSASAPPGGLGD
PLAGKPQLQQMLMQPSTTGQPSSMALPLQMHQLTQQGRHLHPHPPTPAANEESAVININS
TDDEEDDEEDMEDDEELEDEEDGIDEEDDEEEGSDEFYEGEDYDDFDEEEGEELGEEEEE
EDGDMPPLEGSEDRSEEVGMEDDKVLLPMEEGEVGEFCVEGDGEGGIEELQTGRALFGED
RMKVQEVESIGVLEESREEGEGEEDESEQMDDPTMPQILCVTGGALEERLESGEEGVVAG
DGLQEEMGLWDQGAKETELIIAAEEQEASQSQLDMVAENELQAAEGENKPTSQEEEEAAG
GGEPAGADEQPLVAEDIKEQQQTDLDQGDSKEPETSQTEGELGGGGNGCGGERGREEEKG
MKRKRENENTEEEESTQSPEKKKLDEEAVASMLADFVACPPDDEDVATGSNG
> Nothobranchius rachovii [A0A1A8NW86]
MAASAWLHGSAAMRLTEGLVLLMKEQRPEFLPEVLANYREHGVLSLQSASDVAGLVGLCN
GKLSSSKTRLEGLCLLSMLVKDSSSDLFQQHCLSWLRSLQQVVQSQAPVQTIQLAVNVLK
DLLQYSCQLPELAREVGLNSILGILTSLLGLKTECELSAMEGMKACMVYYPRACGSLKDK
LGAYFLSKMDSTSRKTQEAACQGYSHLPCLGGLLDRAVGASRAEGWTNQIHCLLASANSL
LAQIYQGSETDEAVRYQGPGVELAFPHIDHTEPLLLLQHRYAAVCMTIKHTLRTDPASAV
SVPVRPILNLVCRALAVNSKNMILTADGSLRFLVLPSVHINALEVLSELITVVRSGMVQY
AAVIQRLFSQTLSAWTPLPETSVGQQRAYSSVRVSVFRSLELWVKVAGASACILHGTSSH
VDLLLNHLLGDITPGAESVKLRAGLSADVVPVGKPGPRRTKQLVVADAVGPSLQRKGDLL
SNQDTCLAALKALRQVLQSTGMLLKDDIHKRLHEVVLPLCVRLQQQQSSSISAWEPAGGV
SGQYSSALTRRELYRVLLALVLVPSPSWPPPLTCAVSILSNGRIDRHLKVSSFCAEALTI
CNSILHPRRPSLALPLPPLTLKPTATPSVLSCQGPGPRLTLPTLLGGPSSAPPFPARHTL
NLGPSSLLGSLDNHFSLVPGLPGQAPGPGDLMASSQTHHQPDPSGLAPPEGQRPVFVRYD
KEETEDVEISLASDSDDSVVIVPPGMLNVEKQQNDLAANSQSLLSSAPGGAELVTMVPTS

STPTTLDVTSLPNDLATSSALLTSSVTPVNSFPPSSTSVVSLVPSVNSSSTSAPPGGLGD
PLAGKPQLQQMLMQPSTTGQPSSMALPLQMHQLTQQGRHLHQHPPTPAANEESAVININS
TDDEEDDEEDMEEDEELEDEEDGIDEEDEEEEGSDEFYEGEDYDDFDEEEGEELGEEEEE
EEDGDMPPLEGSEDRSEEVGMEDDKVLLPMEEGEVGEFCVEGDGEGGIEELQTGRALFGE
DRMKVQEVESIGVLEESREEGEGEEDESEQMDDPTMPQILCVTGGALEERLESGEEGVVA
GDGLQEEMGLWDQGAKETELIIAAEEQEASQSQLDMVAENELQAAEGENQPPSQEEEEAA
AGGGEPAGADEQPLVAEDIKEQPQTDLDQGDLKEPETSQTEGELGGGGNGCGGERGREEE
KGMKRKRENENTEEEESTQSPEKKKLDEEAVASMLADFVACPPDDEDVATGSNG
> Salmo salar [A0A1S3SE19]
MSLLALIPQSTGAVGGLVGLSNAKLGNSKTKFEGXCLLSVLVKDSSSDVFQQHSLSWLRS
LQQVIQSQDPLPSVQLAVGVLQDLLQYSSQLPELAREVGLNSVLGILTSLLVLRSEVACE
CYGRLTRLGGVVERGGGGCRAEGWTNQLHCLLASANAILAKLYQGSESGRECFFFHKVFQ
SMRADLGSSCPTLLWTTQTPSSCCSYNTDTELPAWLSNIHSVRLPVQQVLNLVCRALAVS
SKSINVTGDGCVRLLVLPAIHKDTLQVLHALITALGSGLVQYSSVLQRLFSQTLSAWTPL
PETSLGQQRAFSAVRVSLYRTLELWVRVGGASASVFQGSPTHSELLLAHLLGDITPGADS
VRPSNSPQLRDLVSSKPCPKSRKPGLAVSGGGGASFQRKGDSLANQDTCLSALRALRQVI
LTSGSLLKEDIHIYILVI
> Microcaecilia unicolor [A0A6P7WPL0]
MAAVATHGVWEQLVESVSGLFRRGDRPGAPTPAPLLLLPGLVRGLWEHGGLRVQSSTSIG
GLTGISNARLSSIKTRFEGLCLLSLLAAESPTDHFQQHCVSWLRSIQHIIQSQDLPPTME
LAVLVLHDLLKYSSLLPALSREISMNLIPGLLTSLLDLKPECQAPALEGMKACMTFYPXA
CGSLRGKLASFFLSQLEAESPQIQQLACECYTLLPSLGSGFAQGVKHTECWEHQLQCLLA
TLHATVGQLYEEAETEPLHYEGPGVELLLPVADDGESLHVLRLKHRFSGISKCLCLLLSS
SFSVPVTVPVQDVLDAICRVLSINSKNISWLGDGPLKTLLLPSIHAEVLDLLAALIMACG
SRLVRFADLFCRLFAQVLTLWSGKRDTVIPGQEKPYSAVRTKVYQVLELWIKVCGASSGV
LQGAGHQCDVLLAHLTSDISPPTDTIKLRVGRLGAEILSNGKPGSKKQKMDVGDGPQLQN
HRKQDVNANSDTCTMALRVLSRAILLCGTLIKEDTHRRLHELVVPLLIHLQQSTLPMTSP
YASSECRKELYRLLLCLLLTPHPRWPPPLHCAVCIFSQGQKENNMKVSSFCAEALVICNC
ILHPRVPSLQLPVLSATGTAATDLLKSSVLGAEVPSTFRHSPLATSVRPQTNHLAIPLQG
LPSGQPLASRLQPSLVSNLTLPSVPPCTIGAENHQLDEQMEVEPRVPSIPSPSEETFGGK
AHRPVFIHYEKEETSDVEISLDSDSDDSVVIVPEGLLPKQPISPPAKAVEEAEELSKEHA
VTVTGATTANATVSAPAPPSNQPPAPPAAPPPPPPPLPPPPPPLQLVAETPALAVEEDLT
VINLNSSEEEEEEEEEEDFAEDEYFDEEDYEEEEYDGEMMEDDDDEEEEEEDEDEEGEEG
RLDLEEIDDIDDEELEEDLEEDLEGEDEVMTEEEEEEEEEEELLQDVMPRIEEEPEEELRI
GFVEKEAPEAEGEPAVQVDAEVREEKAGAEERMDESPPRLSPVREDAQEVKPEEEEDMAA
DADTDLLMTVESDSAERLEDEDEEEEEVRETPILVQETRTPQLPEETSPPPPPPPPPPPP
PSSPPPPPPPPPPPPLSPPLPPPLPTESEECEEIQTEEKSDQNVEAPPLPATTEEGIGELV
TEGDIKPEKEEETPESSEKKAEADIDDSAALLADFIDCAPDGNKISHDTSS
> Bos mutus grunniens [A0A8B9YZ70]
MAAAVLSGPSAGSAAAVPGGPGSLSAVGSGPRLRLMLLESVSGLLQPRTGSAVAPVHPPA
RSAPHLPGLMCLLRLHGTVGGAQVIRSHSGHRLSCVVTQLCLTRFEGLCLLSLLVGESPT
EMFQQHCVSWLRSIQQILQSQDPPPTMELAVTVLKDLLRYAAQLPAVFRDISMNHLPGLL
TSLLGLRPECELSALEGMKACMTHFPRACGSLKGKLASFFLSRVDALSPQLQQLACECYS
RLPSLGAGFSQGLKHTDSWEQELRSLLASLHSLLGGLYEGAEAAPMQYESPGAETLLSSS
EDADAHTLLRLRQRFSGLARCLGLMLSSEFGAPVSVPVQDILDLICRTLSVSAKNVSLLG
DGPLRLLLLPSLHLEALDLLSALILACGARLLRFGALISRLLPQVLNAWSIGRENLGPGQ
ERPYSTVRTKVYAVLELWVQVCGASAGVLQGGASGEALLSHLLSDISPPADALRLRSPRG
SPDAGLQTGKPSAPKKLKLDVGEAIAPPSHRKGDSNANSDVCAANGRVVWRHLQVCTLSF
QRLHELVLPLVMGVQQGEALGSSPYTSSHCRRELYHLLLALLLAPSPRCPPPLACALRAF
SLGQREDSLEVSSFCSEALVTCAALTHPRVPPLQSVGPTCPAPAPVPPPEAPAPFRAPAF
HAPSPLPSAGPMPSAGPMPPVGPLPPTRPGPPATANHLGLSVPGLVSVPPRLLPGPENHR
AGSSEDPVLAPSGSPPPTIPPDETFGGRVPRPAFVHYDKEEPSDVEISLESDSDDSVVIV
PEGLPPPPPPSSGTTPPPVAPAGPPAASPPVPAKDEPEELPAAPGPLPPPPPPPVPGPVT
LPPPQLVPEGTPGGGGPPALEEDMTVININSSDEEEEEEEEEEEEEDEEEDEEEDFEEEEE
EEEEYFEEEEEEEEEFEEEFEEEEGELEDEEDEEEDEELEELEEVEFGPAGGEVEGGGPA
PPSLPPALPPAESPKGPPEPGLEPGLLLEVEEPGTEEAPGPETAPMLAPEVLPSQGEVER
EGGSPPAGPPPQELVEEEPSGPPALLEEGAEGGGDKVSPPPEASAVEETEVEAAALPPEK
QGDTAAMLADFIDCPPDDEKPPPASEPDS
> Catagonus wagneri [A0A8C3VJ53]
MLPGSPSAVQPPFTQCARPQPPPHAPSPRGVRRGVFASSRHATSGARVKMAAAVLSGPSA
GSAAGVPGGTGGLSAVGSGPRLRLLLLESVSGLLQPRAGSTVAPVHPPARSAPHLPGLMC
LLRLHGTVGGAQNLSAVGALIGLSNTRLGSIKTRFEGLCLLSLLVGESPTEMFQQHCVSW
LRSIQQVLQSQDPPPTMELAVAVLKDLLRYAAQLPTLFRDISMNHLPGLLTSLLGLRPEC
ELSALEGMKACMTYFPRACGSLKGKLASFFLSRVDALSPQLQQLACECYSRLPSLGAGFS
QGLKHTESWEQELHSLLASLHGLLGALYEGAETAPVQYEGPGVEALLSPSEDGDAHVLLR
LRQRFSGLARCLGLLLSSEFGAPVSVPVQEVLDVICRTLSVSARNISLLGDGPLRLLLLP
SLHLEALDLLSALILACGGRLLRFGALISRLLPQVLNAWSIGRDTLSPGQERPYSTVRTK
VYAVLELWVQVCGASAGVLQGGASGEALLTHLLSDISPPADALKLRSPRGSPDGGLQTGK
PSAPKKLKLDMGETVAPPSHRKGDSNANSDVCAAALRGLSRTILMCGPLIKEETHRRLHE
LVLPLVMGVQQGEVLGSSPYTSSRCRCELYRLLLALLLSPSPHCPPPLACALQAFSLGQR
EDSLEVSSFCSEALVTCAALTHPRVPPLQSMVPTCPTPAPVPPPEALSPFRAPAFHPPGP
MPSVGPMPPARPGPPATANHLGLSGPSLVSVPPRLLPGPENHRAGSNEEPVLAPSGTPPP
TLPSDETFGGRVPRPAFVHYDKEEASDVEISLESDSDDSVVIVPEGLPPLPPPPTSGTTP
PPVAPAGPPTASPPVPAKEEPEELPAAPGPLPPPPPTPVPGPVTLPPPQLVPEGTPGGGG
APALEEDLTVININSSDEEEEEEEEEEEEEEEEEEEEDFEEEEEDEEEYFEEEEEEEEEFE
EEFEEEEGELEEEEEDEEEEEEEEELEEVEELEFGSTGAEVEEGGPPPPSLPPALPPAESP
KVQPEPEPEPEPGLLLEVEEPGPEEGPGPETAPTLVPEALPSQGEAEREAGSPPSVPPPQEL

VEEEPPVPPTLLEEGAEGGGDKVPPLAETSAAEEMEMETESAALQEKEQDDTAAMLADFI
DCPPDDEKPQPATEPDS
> Denticeps clupeoides [A0A8C3ZDW3]
MAAAACLHGSASTRLTEGLVSALKEERPEHLPTLLASYREHGAVSAQTSAAVGGLLGLSH
ARLGSSKTRFEGLCLLSVLVKDGSTELFQHHCLSWLRSLQQVVQSQAPVPTVQLAVNVLQ
DVLQYSSQIPELAREIGLNSILGILTSLLGLKAECHLVAMEGMLACMTYYPRACGSLREK
LGAYFLSKMDSDNPKAQEVACECYGRLPCLGGVLERGGGGRRAESWTNQMHCLLASANSM
LGQLYQGTETEGTVQYEGPGVELPFPPLDEIDPLLVLQLRHRYRAVCLALKHTLSVDPAS
PVRLPVQHMLNFVCRALAVSSKSINVTGDGSLKLLVLPSVHSDTVEVLSALIKIVGSGLV
QYCGVINQLFSQSLSAWTPLPEASLGQQRAYSAVRVCLYHTLELWVRVGGASASVLQGSS
THTELLFSHLMGDVTPGSEAVKLRSGQSAAAISDLVGLGGKTGPRRPKALGIGETGGVSL
QRKGDTIANQDTCLSALRALRQIVLTSGTLLKDELHKKLQDMVVPLCVRLQQQQAVGEVG
GISGQYGSAHPRRELYRLLLALLLVPSPRWPPPLACAVSIFSHGRRDRSLMVSSFCAEAL
TICNCLLHPRTPSIALPLPPLTLKPTPTAPVLPSSQNPTMSLPSILGGPAPGTPFPARHP
LTLGTSALMRSLQNHLPLAPSELTTPVGASATPADLLLSPAQQSELTGLPPPEGQRQVFI
RYDKEEAEDVEISLESDSDDSVVIVPPGTLMPEGADVTNTSQSLPSAPPVPSQGAAAVPG
VTLPEPTGDAVATGNASLPNELPSSSSPPVPPPAPNSINSFTVRAPQLTSALPSLIVPSI
GMDLIPSGAHLLMNPRPAGSVQAQMGRPMLLDMPAQSAPPTRLMPPQQMPSEEDTTVINI
NSSDEEDEEEEEIEDEDEFGEDEEEEGLDEDDEEEDSEFEEEGTYEDCDYEEEEEGEEIE
EEEEEEEEGAEDLATLEEVTRRGPMGRPETEVMIGSEEEEQQQRGIEMFPLEAQDEGNEE
MEGSSAVEALEEGGPVEEEEEEEDLEKEKSGEVEVEASEQQKEKDGQDQEVGEELGSQEV
RIWEQEGSQAEFTAVTEEASPPECKEDESKVGGQDQKSVGLDQEEMPTQSRADAPREESE
PQVAAEGAEPEKGGEEKDEEEDSRGMKRKMEDREEETLEQSTEKKKFHDEAMESMLAAFV
ACSPDEEENASSDPSCS
> Moschus moschiferus [A0A8C6DF71]
MAAAVLSGPSAGSAAAVPGGTGGLSAVGSGPRLRLMLLESVSGLLQPRTGSAVAPVHPPA
RSAPHLPGLMCLLRLHGTVGGAQNLSAVGALVGLSNARLGSIKTRFEGLCLLSLLVGESP
TEMFQQHCVSWLRSIQQILQSQDPPPTMELAVTVLKDLLRYAAQLPAVFRDISMNHLPGL
LTSLLGLRPECELSALEGMKACMTHFPRACGSLKGKLASFFLSRVDALSPQLQQLACECY
SRLPSLGAGFSQGLKHTDSWEQELRSLLASLHSLLGGLYEGAEAVPMQYESPGAETLLSP
SEDADAHTLLRLRQRFSGLARCLGLMLSSEFGAPVSVPVQDILDLVCRTLSVSAKNVSLL
GDGPLRLLLLPSLHLEALDLLSALILACGARLLRFGALISRLLPQVLNAWSIGRENLGPG
QERPYSTMRTKVYAVLELWVQVCGASAGVLQGGASGEALLSHLLSDISPPADALRLRSPR
GSPDGGLQTGKPSAPKKLKLDVGEAIAPPGHRKGDSNANSDVCAAALRGLSRTILMCGPL
IKEETHRRLHELVLPLVMGVQQGEALGSSPYTSSHCRRELYRLLLALLLAPSPHCPPPLA
CALRAFSLGQREDSLEVSSFCSEALVTCAALTHPRVPPLQSVGPACPAPAPVPPPEAPAP
FRAPAFHTPGPLPSAGPMPSAGPMPPAGPLPPARPGPPATANHLGLSVPGLVSVPPRLLP
GPENHRAGSSEDPVLAPSGSPPPTVPPDETFGGRVPRPAFVHYDKEEASDVEISLESDSD
DSVVIVPEGLPPPPPPPPSGSTPPPVAPSGPPAASPPVPAKDEPEELPAAPGPLPPPPPPP
VPGPVTLPPPQLVPEGTPGGGGPPALEEDLTVININSSDEEEEEEEEEEEEEDEEDEEEEF
EEDEEEEEEYFEEEDEEEEEFEEEFEEEEGELEEEEDEEEDEELEELEEVEFGPAGGEVE
GGGPAPPSLPPALPPAESPKGPPEPGLEPGLLLEVEEPGTEEAPGPETAPTLAPEVLPSQ
GEVEREAGSPPAGPPPQELVEEEPSGPPTLLEEGAEGGGDKVSPPPEVSAAEETEVEAAA
LPTEKEQGDAAAMLADFIDCPPDDEKPPPASEPDS
> Pelusios castaneus [A0A8C8S6G9]
MAAGPGCPGRLVLEALGGGGPALPGLVRGLRDSGAGGQNMPALGSLIGVTNARLGSVKTR
FEGLCLLSLLVSESPTEPFQQHCLAWLRSLQHLLQSQDPAPTMALGASVLQDLLQYSCQL
PELARDIGTNHIPGLLTSLLALKAECQLSALEGIKACMTFYPRACGSLRGKLAAYFLSRV
DADSPQLQQLACECYALLPSLGAGFTQGLKHRECWEQQLHGLLATLHGLLGNLFEGAETD
PLPYEGPGVELLLPAPPDGESGFVLNLHNRFSGLAKCLQLMLSNEFVAPVMVPVQDVLDL
ICRALNISSKNFSWFGDGPLRMLLLPSVHLQILDVLSALILACGARLARWGSLLARLFPQ
VLATWSGSRESVLPGQEKPYSAVRTRLYQVLELWVQVGGAGAGVLQGPPHRSEALLGHLL
SDITPPTDSVKLKVGRSASEGKPSAPKKPKLSEGGDAPFLYRKQDPMANSDVCKAALQAL
SQAILVGGSLIKEETHRRLQELVVPLLLRLAQGEVAAGSPYAHPACRLHLYRLLLALLLA
PAPTCPPPLHCALRAFTLGQQDPSLQVSSFCTEALVICNALARPRVPSLQLPLPSPAPST
GPAPPELAAASPFRQALPPVRPPPANHLGLAPPRLPPAAAMPGLAPAEECMEGSESRLPA
SPSPGGAEEGGPGPGGKARRPVYVHYEKEEESDVEISLESDSDDSVVIVPKGLLPRPPPP
PPPPPPPAPPSPPPTEPPEEAPPPPTSEVAPGLGEEEPAVININSSEEEEEEEEEEDFAEE
EEEEDEEEEEEEEEEEFFDEEEDFEEEEFEEEEEEEEEYEEEEGLSEEEGLSEEEGALSEEER
PASGPEEVPPMLGEPEDLIMEVEESQAPEPPAPGEEEEEEEEVVVMKTSPPPVPQLPREEE
EPPPAPPTPPRRVSPLQAPSPGQEEMGVEPPLLEATPPAPEEPPPEEKEEGASPAGEQGA
GTGEEPHAGALSEEQGEAVKGEVEADETATMLADFIDCPPDDDKGPPEPSS
> Salvator merianae [A0A8D0AZQ1]
MPGRGGGTWCFVRTRSVQESGRRACSDGGGGGASVVLLVVPGGGVLLAAGAREPGRAAAR
RTCRGAPASSREPCGHPPGPGRSGAMRLGGGRGGRRRGRQGEGALASLSALRLGSMKTRF
EGLCLLSLLVTESNAETFSQHCLSWLRSLQHLIQSQDPPPTMELAMLILRDLLEYSCQFP
ELSRDISTNHIPGLLTSLLALKPECQLSALEGIKTCMILYPRACGSLRGKLTAHFLAQVD
AEMPRLQQLACECYALVPSLGAGFTKGLKYTECWEQQAHGLLATLHSLLGTLYEGAETDP
LLYEGPGXEVPLPAPEDEDTNLILHLQHRFTGLMKCLCRMLSNEFVAPVTVPVQDVLDLV
CRVLATSMKNFTWFGDGPLRMLLLPTLHLEALDLLAALILACGPRLVRFGGSLGRLFPQV
LTAWSLGRELFLPGQKRPYSVVRTRXYEVLELWIKVAGAASGVLQGPEAQSEALLGHLIS
DISPPSDTLKLHESHPGPDSKPSAPKRPKLSDPGSLGSLHRKHDPQANSNVCLAALRGLS
RAVLLAGSLMKEQAHKRLQELVIPVLIRLGQAEPLPGSPYASASCRQELYRLVLSLVLAP
PPSCPPPLQCALRLLSHGRRDPNLQVSAFCAEALVVCNALLHPRVPSWQLPLAGSSVPLP
GTSEISPAVNSPFRSAPPLSFSAPRLLPPSVSPSAPPSVTPSANSLGLPLPGLASAQLPP
RLTPEEPTLPPSPGTAEAAALAAGAKLRRSVFIHYDKEEEEDVEISLESDSDDSVVIVPK
GQLGKAPSASSPAVAAMAPAVPAPLPPPPPPPLPPLMGRKGGGHGHSWPQFRCFPLSVVQ

DEEFDEEEEEFEEELDEEEEEELEEEEMEEEEEEDEFDEEGMTEEEEGMTEEEEEEEEEG
AGLLMEVDEEAFHPPEEVGEEEEEEEEEEEESRAEPDVLLLKPRPPTLPSPEVLPLPAQLP
LPAVASLPAAPCAGRPLSPAVAGTHPEEKPVSQEREGAGEQGMEAGALGPPSAVSPQREQ
AAEEVPEGGRRDAGEAVAEGKEEKSPGKDVVEVKEEKEVRAWGGHAVDETEAMLADFVDC
PPDEEKVPPAVEPSA
> Salvator merianae [A0A8D0B679]
MPGRGGGTWCFVRTRSVQESGRRACSDGGGGGASVVLLVVPGGGVLLAAGAREPGRAAAR
RTCRGAPASSREPCGHPPGPGRSGAMRLGGGRGGRRRGRQGEGAPRRGGARRAVETPPAF
TLSLPPQVSSLLGSLASLSALRLGSMKTRFEGLCLLSLLVTESNAETFSQHCLSWLRSLQ
HLIQSQDPPPTMELAMLILRDLLEYSCQFPELSRDISTNHIPGLLTSLLALKPECQLSAL
EGIKTCMILYPRACGSLRGKLTAHFLAQVDAEMPRLQQLACECYALVPSLGAGFTKGLKY
TECWEQQAHGLLATLHSLLGTLYEGAETDPLLYEGPGXEVPLPAPEDEDTNLILHLQHRF
TGLMKCLCRMLSNEFVAPVTVPVQDVLDLVCRVLATSMKNFTWFGDGPLRMLLLPTLHLE
ALDLLAALILACGPRLVRFGGSLGRLFPQVLTAWSLGRELFLPGQKRPYSVVRTRXYEVL
ELWIKVAGAASGVLQGPEAQSEALLGHLISDISPPSDTLKLHESHPGPDSKPSAPKRPKL
SDPGSLGSLHRKHDPQANSNVCLAALRALPFSSLPFQRLQELVIPVLIRLGQAEPLPGSP
YASASCRQELYRLVLSLVLAPPPSCPPPLQCALRLLSHGRRDPNLQVSAFCAEALVVCNA
LLHPRVPSWQLPLAGSSVPLPGTSEISPAVNSPFRSAPPLSFSAPRLLPPSVSPSAPPSV
TPSANSLGLPLPGLASAQLPPRLTPEEPTLPPSPGTAEAAALAAGAKLRRSVFIHYDKEE
EEDVEISLESDSDDSVVIVPKGQLGKAPSASSPAVAAMAPAVPAPLPPPPPPPLPASPPP
PSEETPLEPVVPVALGGAPPPAPPALVLPPSPAPLASDVPFPALVEEDPAVININSSEEE
EEEEEEEEEEEEDFPEDEDYFEEEEEVGLCVARAGHPLPRLMGLRICQGSISSRPGPGGR
SARRLGHIWQHLVLPLWPLLGAEAGRHEGLPAGGCSGRWAALCPSPFRAGGHVGLFLFSP
RQAPAVDETEAMLADFVDCPPDEEKVPPAVEPSA
> Sciurus vulgaris [A0A8D2BEE4]
MAAAVLSGPSAGSPAGAPGGTGGLSAMSSGPRLRLLLLESVSGLLQPRTGSTVAPVHPPV
RSVPHLPGLMCLLRLHGTVGGAQNLSALGALVSLSNARLGSIKTRFEGLCLLSLLVGESP
TEMFQQHCVSWLRSIQQVLQSQDPPPTMELAVAVLRDLLRYASQLPTLFRDISINHLPGL
LTSLLGLRPECEQSALEGMKACMTYFPRACGSLKGKLASFFLSRVDALSPQLQQLACECY
SRLPSLGAGFSQGLKHTESWEQELHSLLASLHSLLGALYEGAETAPVQNEGPGVETLLSH
SEDGDAHVLLRLRQRFSGLARCLGLMLSSEFGAPVSVPVQEVLDLICRTLSVSGKNISLL
GDGPLRLLLLPSIHLEALDLLSALILACGGRLLRFGALVSRLLPQVLNAWSIGRDTLSPG
QERPYSTIRTKVYAVLELWVQVCGASAGMLQGGASGEALLTHLLSDISPPADALKLRSPR
GSPDGGLQTGKPSAPKKIKLDVGEAMAPPSHRKGENNANSDVCAAALRGLSRTILMCGPL
IKEETHRRLHDLVLPLVMGVQQGEVLGSSPYTSSCCRRELYRLLLALLLAPSPRCPPPLA
CALQAFSLGQREDSLEVSSFCSEALVTCAALTHPRVPPLQAMGPTCPTPAPVPPPEAPSP
FRAPPFHPPGPMPSVGPMPSVGPLPSAGPLPSSGSMPSVGPMPSTGPMPSAGPIPSAGPV
PSARPGPPATANHLGLSVPGLVSVPPRLLPGPENHRAGSNEDPVLAPSGTPPPTIPPDET
FGGRVPRPAFVHYDKEEASDVEISLESDSDDSVVIVPEGLPPLPPPPPSGTTPPPVAPTG
PPIASPPVPAKEEPEELPAAPGPLPPPPPPPVPGPVTLPPPQLVPEGTPGGGGPPALEED
LTVININSSDEEEEEEEEEEEEEEEEEEEEEEDFEEEEEDEEEYFEEEEEEEEEFEEEFEE
EEGELEEEEEEEEEEEEEEEELEEVEDLEFGSAGGEVEEGGPPPPTLPPALPPPESPKEQP
EPEPEPGLLLEVEEPGAEEQHGAETAPTLAPEGLPSQGEEEREGGSPDAGPPPQELVEEE
PSAPPTLLEEGTEGGDDRVPPPPETSVEEEMETEAEAAVLQEKEQDDTAAMLADFIDCPP
DDEKPPPTTEPDS
> Urocitellus parryii [A0A8D2KC90]
MAAAVLSGPSAGSPAGVPGGTGGLSAMNSGPRLRLLLLESVSGLLQPRTGSTVAPVHPPV
CSVPHLPGLMCLLRLHGTVGGAQNLSALGALVSLSNARLGSIKTRFEGLCLLSLLVGESP
TEMFQQHCVSWLRSIQQVLQSQDPPPTMELAVAVLRDLLRYAAQLPTLFRDISINHLPGL
LTSLLGLRPECEQSALEGMKACMTYFPRACGSLKGKLASFFLSRVDALSPQLQQLACECY
SRLPSLGAGFSQGLKHTESWEQELHSLLASLHSLLGALYEGAETAPVQNEGPGVETLLSP
SEDGDAHVLLRLRQRFSGLARCLGLMLSSEFGAPVSVPVQEVLDLICRTLSVSGKNISLL
GDGPLRLLLLPSIHLEALDLLSALILACGGRLLRFGALISRLLPQVLNAWSIGRDTLSPG
QERPYSTIRTKVYAILELWVQVCGASAGVLQGGASGEALLTHLLSDISPPSDALKLRSPR
GSPDGGLQTGKPSAPKKIKLDVGEAMAPPSHRKGENNANSDVCAAALRGLSRTILMCGPL
IKEETHRRLHDLILPLVMGVQQGEVLGSSPYTSSCCRRELYRLLLALLLAPSPRCPPPLA
CALQAFSLGQREDSLEVSSFCSEALVTCAALTHPRVPPLQAMGPTCPTPAPVPPPEAPSP
FRAPPFQPPGPMPSVGPMPSAGPMPSAGPLTSSGSMPSVGPMPSAGPMPSAGPIPSAGPV
PSARPGPPATANHLGLSVPGLVSVPPRLLPGPENHRAGSNEDPVLAPSGTPPPTIPPDET
FGGRVPRPAFVHYDKEEASDVEISLESDSDDSVVIVPEGLPPLPPPPPSGTTPPPVAPTG
PPTASPPVPAKEEPEELPAAPGPLPPPPPPPVPGPVTLPPPQLVPEGTPGGGGPPALEED
LTVININSSDEEEEEEEEEEEEEEEEEEEEEEEFEEEEEEEEEYFEEEEEEEEEFEEEFEEE
EGELEEEEEEEEEEEEEEEELEEVEDLEFGSAGGEVEEGGPPPPTLPPALPPPESPKEHPEP
EPEPGLLLEVEEPGSEEQHGTETAPTLAPEVLPSQAEEEREGGSPEAGPPPQELVEEEPS
APPTLLEEGTEGGGDNVPPPPETAAEEEMETETEAAVLQEKVRARWRSTLARKVGLVGKW
GAAAFIREEVCHFSMYTYVYVSNLMSLFLKGAG
> Varanus komodoensis [A0A8D2LGX3]
GGCSAPPRPAAGAPRGAAPAAGGGAVPSGPAAVGRHLPSAFHGGPGGGPWSILWHCAGPW
PPSWRPFLSGAGTWGGLLWAHVGWLGGARRGAGLVPRRRPDPVLRSYPFCRPPCRFEGLC
LLSLLVTESTTETFSQNCLSWLRSLQHLIQSQDPAPTMELAVLVLRDLLECSCQLPELAR
DISTNHVPGLLTSLLALRPECQLSALEGSKACMMFYPRACGSLRGKLAAYFLSRVDAETP
QVQQLACECYALLPSLGAGFTQGLKYTECWEQQAHCLLATLHSLMGTLYEGAETDPLHYE
GPGMEIPLPAPEEGETNFVLHLKQRFSGLAKCLCRMLSNEFVAPVTVPVQDILDFICRAL
DISMKNISWFGDGPLRMLFLHSVHLESLDLLFALILACGPRLVRFGGTLCRLFPQVLTAW
SAGRDLVPAGQERPYSAVRTRLYQVLDLWAQVAGAASGLLQGHGTQSEALLGHLLSDISP
PTDTLKLRESAPGPDRRPSAPKKPKLAGLGPLHHKHDPRANSDVCLAALGLVRRSPRAPC
LNPTQRLQELVIPLLIRLGQAEPLPGSPYASAACRRELFRLLLALVLAPAPACPPPLHCA

LWLLKQGCADPSLQVSSFCSEALVVCNALLHPRVPALQVPLPGPPAPPPGSSSEISPATA
SPFRSAPPLPFPPPRLPPPASPPAPPSAAPPAGSLGLPLPPLALAQLPPRLAPEEPAVLP
SPGGAAEAAALAAGAKLRRSVFIHYDKEEEEDVEISLESDSDDSVVIMPKGQLGRGPGGA
GAVAAVAAAPAPLPPPPPAPPRGSRCLRLSDQEEEFEEDEGEFDELEEEIEEMDEEEEEE
DEFDEEECMTEEEEEEEEEEEEEEEEGLTEEEEEGAPPLPTLPHGNGGEPPALMPMKEEL
PKLVGEEEGGEGEEEEEGAGLLMEVEEEAFHAPEDGSGGQEGEEEAAAGTREVGRTAGDA
LLRKAEEPPVPTSAVEELLPPPLPLPPSPAQEPPTLPATAFTDPEELAEEQERPMAGGPP
FAAAPQDPEGERGPAGHAGGPAEAGPEARAPQGEAEEQLPALGGHVPEEEPEVRAWGGQA
GGRAGSPATPGCPMASAAAWRHWKPQLSCPVLGLPAPASGAPAALAEGESDPQPGPLGLG
AGRRWAGGPREGVS
> Canis lupus familiaris [A0A8P0PLL7]
MAAAVLSGPSAGSAAGVPGGAGGLSAVGSGPRLRLLLLESVSGLLQPRAGSAVAPVHPPV
RSAPHLPGLMCLLRLHGTVGGAQNLSAVGALVGLSNARLGSIKTRFEGLCLLSLLVGESP
TEMFQQHCVSWLRSIQQVLQSQDPPPTMELAVAVLRDLLRYAAQLPTLFRDISMNHLPGL
LTSLLGLRPECELSALEGMKACMTYFPRACGSLKGKLASFFLSRVDALSPQLQQLACECY
SRLPSLGAGFSQGLKHTESWEQELHSLLASLHSLLGALYEGAETAPVQYEGPGVEVLLTP
SEDGDTHVLLRLRQRFSGLARCLGLMLSSEFGAPVSVPVQEILDVICRTLSISAKNISLL
GDGPLRLLLLPSIHLDALDLLSALILACRSRLLRFGALISRLLPQVLNAWNLGRDTLAPG
QERPYSTVRTKVYAVLELWVQVCGASAGMLQGGSSGEALLSHLLSDISPPADTLKLRSPR
GSPDGGLQTGKPSAPKKLKLDVGEAMAPPSHRKGDSNANSDVCTAALRGLSRTILMCGPL
IKEETHRRLHDLVLPLVMGVQQGEVLGSSPYTSSRCRQELYRLLLALLLAPSPRCPPPLA
CALQAFSLGQREDSLEVSSFCSEALVTCAALTHPRVPSLRSMGPACPTPAPAPPPEAPSP
FRAPPFHPPGPMPSAGPMPSVGPMPSVGPMPPAGPMPPAGPMAPTRPGPPATANHLGLSV
PGLVSVPPRLIPGPENHRAGSNEDPVLAPSGTPPPAIPPDETFGGRVPRPAFVHYDKEEA
SDVEISLESDSDDSVVIVPEGLPPLPPPPPSGTTPPPVAPAGPPVASPPVPAKEEPEELP
VAPGPLPPPPPPPVPGPVALPPPQLVPEATPGGGGPPALEEDLTVININSSDEEEEEEEE
EEEEEEEEEEEEEEEDFEEEEEEEEYFEEEEEEEEEFEEEFEEEEGELEEEEEEEDEEEE
EELDELEEVAFGPAAGAAEEGGPPPPSPPPALPPAQSPKMQPEPPGETGLLLEVEEPAAE
EEPGAEAAPTLAPEVLPPQGEGPREVGSPPAVPPPQELIEEEPPAPPTLLEEGTESGGDK
VPVPQETPAAEDVEAEVEAETAALQEKVGDRGRSLGGKVGWAAAAKLSPACLSGACACFQ
PYVFVSERSRMTRLPCWLTSSIVPLMTTSHHRPQSLIPSPLWRPPLPVSNKVMSWDNTCC
FCLLHKCVPGQKPCQLPGFTRYPWRSPCVCMASWGLRHPQGDPLT
> Canis lupus familiaris [A0A8P0SK05]
MLGAVSPGGGRGDYKLPGCCAHSPVCAATATATSRPTVNLSAVGALVGLSNARLGSIKTR
FEGLCLLSLLVGESPTEMFQQHCVSWLRSIQQVLQSQDPPPTMELAVAVLRDLLRYAAQL
PTLFRDISMNHLPGLLTSLLGLRPECELSALEGMKACMTYFPRACGSLKGKLASFFLSRV
DALSPQLQQLACECYSRLPSLGAGFSQGLKHTESWEQELHSLLASLHSLLGALYEGAETA
PVQYEGPGVEVLLTPSEDGDTHVLLRLRQRFSGLARCLGLMLSSEFGAPVSVPVQEILDV
ICRTLSISAKNISLLGDGPLRLLLLPSIHLDALDLLSALILACRSRLLRFGALISRLLPQ
VLNAWNLGRDTLAPGQERPYSTVRTKVYAVLELWVQVCGASAGMLQGGSSGEALLSHLLS
DISPPADTLKLRSPRGSPDGGLQTGKPSAPKKLKLDVGEAMAPPSHRKGDSNANSDVCTA
ALRGLSRTILMCGPLIKEETHRRLHDLVLPLVMGVQQGEVLGSSPYTSSRCRQELYRLLL
ALLLAPSPRCPPPLACALQAFSLGQREDSLEVSSFCSEALVTCAALTHPRVPSLRSMGPA
CPTPAPAPPPEAPSPFRAPPFHPPGPMPSAGPMPSVGPMPSVGPMPPAGPMPPAGPMAPT
RPGPPATANHLGLSVPGLVSVPPRLIPGPENHRAGSNEDPVLAPSGTPPPAIPPDETFGG
RVPRPAFVHYDKEEASDVEISLESDSDDSVVIVPEGLPPLPPPPPSGTTPPPVAPAGPPV
ASPPVPAKEEPEELPVAPGPLPPPPPPPVPGPVALPPPQLVPEATPGGGGPPALEEDLTV
ININSSDEEEEEEEEEEEEEEEEEEEEEEEDFEEEEEEEEYFEEEEEEEEEFEEEFEEEE
GELEEEEEEEDEEEEEELDELEEVAFGPAAGAAEEGGPPPPSPPPALPPAQSPKMQPEPP
GETGLLLEVEEPAAEEEPGAEAAPTLAPEVLPPQGEGPREVGSPPAVPPPQELIEEEPPA
PPTLLEEGTESGGDKVPVPQETPAAEDVEAEVEAETAALQEKEQDDTAAMLADFIDCPPD
DDKPPPATEPDS
> Nothobranchius furzeri [A0A9D3BH42]
MAASAWLHGSAAMRLTEGLVLLMKEQRPEFLPEVLANYREHGVLSSQSASDAAGLVGLCN
GKLSSSKTRLEGLCLLSMLVKDSSSDLFQQHCLSWLRSLQQVVQSQAPVQTIQLAVNVLK
DLLQYSCQLPELAREVGLNSILGILTSLLGLKTECELSAMEGMKACMVYYPRACGSLKDK
LGAYFLSKMDSTSRKTQEAACQGYSHLPCLGGLMDRAVGASRAEGWTNQIHCLLASANSL
LAQIYQGSETDEAVRYQGPGVELAFPHIDQTEPLLLLQHRYAAVCMTIKHTLRTDPASAV
SVPVRPILNLVCRALAVNSKNMILTADGSLRFLVLPSVHINALEVLSELITVVRSGMVQY
AAVIQRLFSQTLSAWTPPPETSVGQQRAYSSVRVSVFRSLELWVRVAGASASILHGTSSH
VDLLLNHLLGDISPGAESVKLRAGLSADVVPVGKPGPRRTKQLVVADAVGPSLQRKGDLL
SNQDTCLAALKALRQVLQSTGMLLKDDIHKRLHEVVLPLCVRLQQQQSSSISAWEPAGGV
SGQYSSALTRRELYRVLLALVLVPSPSWPPPLTCAVSILSNGRIDRHLKVSSFCAEALTI
CNSILHPRRPSLALPLPPLTLKPTATPSVLSCQGPGPRLTLPTLLGGPSSAPPFPARHTL
NLGPSSLLGSLDNHFSLVPGLPGQAPGPGDLMASSQTHHQPDPSGLGPPEGQRPVFVRYD
KEETEDVEISLASDSDDSVVIVPPGLLNVEKQQNDVAANSQSLLSSAPGGAELVTMVPTS
STPTTLDVTSLPNDLATSSALLTSSVTPINSFPPSSTSVVSLVPSVNSSSASAPPGGLGD
PLAGKPQLQQMLMQPSTTGQPSSMALPLQMHQLTQQGRHLHPHPPTPAANEESAVININS
TDDEEDDEEDMEDDEELEDEEDGIDEEDDEEEGSDEFYEGEDYDDFDEEEGEELGEEEEE
EEDGDMPPLEGSEDRSEEVGMEDDKVLLPMEEGEVGEFCVEGDGEGGIEELQTGRALFGE
DRMKVQEVESIGVLEESREEGEGEEDESEQMDDPTMPQILCVTGGALEERLESGEEGVVA
GDGLQEEMGLWDQGAKETELIIAAEEQEASQSQLDMVAENELQAAEGEKPPSQEEEEAAA
GGGEPAGADEQPFVAEDIKEQQQTDLDQGDLKEPETSQTEGELGGDGCGGERGREEEKGM
KRKRENENTEEEESTQSPEKKKLDEEAVASMLADFVACPPDDEDVATGSNG
> Branchiostoma floridae [A0A9J7M1L4]
MAAHMANVLLTSLSEKDGKTSLPWAIEVANEHQLLQSEQNAQDWVSHINTSLGTAKTRLE
GLCLLGTVVQQCSAGTFIQHGTTWIRMLTQVLQAYDSPLTLQMASHVLGSVVQQAAQYPE

VAREVATTHIPTLVQCLLGAQDHQWFPSALEALQSCMKNFPGPCGSSKGKVESLICGLMD
TSQPRLSQLAQQTCPLLAGCGGGGAGGVKYTEAWAHLCDQVLGSLHQVLDHAYQDMETGL
QTYSVPQASLRLKTVPESDPARTFVLSTRFHNLCGCLEQLVSQEFPAVVRIPVPDILAFL
CRALGVNPKMLFGKASMEHVLLMSALPKMHCSALSILEALIISCRSHLVPHASVISQLLV
QTLGWTTSEEGVPGRQRPYSTLRSRAYTVLTVWLNVCSAASGVDSHADVILQHVLKDATP
QADTTKVCQLE
> Equus caballus [A0A9L0RVG2]
MDCLSNHRSGGNEHSRSPCGHVCWPQQNLSAVGALVGLSNARLGSIKTRFEGLCLLSLLV
GESPTEMFQQHCVSWLRSIQQVLQSQDSPPTMELAVAVLRDLLRYAAQLPTLFRDISMNH
LPGLLTSLLGLRPECELSALEGMKACMTYFPRACGSLKGKLASFFLSRVDALSPQLQQLA
CECYSRLPSLGAGFSQGLKHTESWEQELHSLLASLHSLLGALYEGAETAPMQYEGPGVEV
LLSPSEDGDAHALLRLRQRFSGLARCLGLMLSSEFGAPVSVPVQEILDVICRTLSISAKN
ISLLGDGPLRLLLLPSIHLEALDLLSALILACGGRLLRFGALISRLLPQVLNAWSIGRDT
LSPGQERPYSTMRTKVYAVLELWVQVCGASAGVLQGGASGEALLTHLLSDISPPADALKL
RSPRGSPDGGLQSGKPSAPKKLKLDVGEAMAPPSHRKGESNANSDVCAAALRGLSRTILM
CGPLIKEETHRRLHDLVLPLVMGVQQGEVLGSSPYTSSRCRRELYRLLLALLLAPSPRCP
PPLACALQAFSLGQREDSLEVSSFCSEALVTCAALTHPRVPPLQSMGPTCPTSAPVPPPE
APSPFRAAPFHPPGPMPSAGPMPSAGPVPSAGPMPSVGPLPSVGPMPSVGPMPPARPGPP
ATANHLGLSVPGLVSVPPRLLPGPENHRAASNEDPVLAPSGTPPPAIPPDETFGGRMPRP
AFVHYDKEEASDVEISLESDSDDSVVIVPEGLPSLPPPPPSGTTPPPVAPAGPPTASPPV
PAKEEPEELPAAPGPLPPPPPPPPVPGPVTLPPPQLVPEGTPGGGGPPALEEDLTVININS
SDEEEEEEEEEEEEEEEEEEEEEEDFEEEEEDEEEYFEEEEEEEEEEFEEEFEEEEGELEEE
EEEEDEEEEEEELEEVEELEFGSAGGEVDEAGPPPPSLPPALPPAESPKVQPEPEPEPGLL
LEVEEPGVEEERGAETAPTLAPEVLPSQGEVEREEGSPPAGPPPQELVEEEPSAPPTLLE
EGTEGGDDKVPPLPEPPAAEEMETETEVAALQEKEQDDTAAMLADFIDCPPDEEKPPSAT
EPDS
> Equus caballus [A0A9L0RZG4]
MNLSAVGALVGLSNARLGSIKTRFEGLCLLSLLVGESPTEMFQQHCVSWLRSIQQVLQSQ
DSPPTMELAVAVLRDLLRYAAQLPTLFRDISMNHLPGLLTSLLGLRPECELSALEGMKAC
MTYFPRACGSLKGKLASFFLSRVDALSPQLQQLACECYSRLPSLGAGFSQGLKHTESWEQ
ELHSLLASLHSLLGALYEGAETAPMQYEGPGVEVLLSPSEDGDAHALLRLRQRFSGLARC
LGLMLSSEFGAPVSVPVQEILDVICRTLSISAKNISLLGDGPLRLLLLPSIHLEALDLLS
ALILACGGRLLRFGALISRLLPQVLNAWSIGRDTLSPGQERPYSTMRTKVYAVLELWVQV
CGASAGVLQGGASGEALLTHLLSDISPPADALKLRSPRGSPDGGLQSGKPSAPKKLKLDV
GEAMAPPSHRKGESNANSDVCAAALRGLSRTILMCGPLIKEETHRRLHDLVLPLVMGVQQ
GEVLGSSPYTSSRCRRELYRLLLALLLAPSPRCPPPLACALQAFSLGQREDSLEVSSFCS
EALVTCAALTHPRVPPLQSMGPTCPTSAPVPPPEAPSPFRAAPFHPPGPMPSAGPMPSAG
PVPSAGPMPSVGPLPSVGPMPSVGPMPPARPGPPATANHLGLSVPGLVSVPPRLLPGPEN
HRAASNEDPVLAPSGTPPPAIPPDETFGGRMPRPAFVHYDKEEASDVEISLESDSDDSVV
IVPEGLPSLPPPPPSGTTPPPVAPAGPPTASPPVPAKEEPEELPAAPGPLPPPPPPPPVPG
PVTLPPPQLVPEGTPGGGGPPALEEDLTVININSSDEEEEEEEEEEEEEEEEEEEEEEDFE
EEEDEEEYFEEEEEEEEEEFEEEFEEEEGELEEEEEEEEDEEEEEEELEEVEELEFGSAGGE
VDEAGPPPPSLPPALPPAESPKVQPEPEPEPGLLLEVEEPGVEEERGAETAPTLAPEVLP
SQGEVEREEGSPPAGPPPQELVEEEPSAPPTLLEEGTEGGDDKVPPLPEPPAAEEMETET
EVAALQEKEQDDTAAMLADFIDCPPDEEKPPSATEPDS
> Panthera pardus [A0A9V1DWM2]
MAAAVLSGPSAGSAAGVPGGTGGLSAVGSGPRLRLLLLESVSGLLQPRAGSAVAPVHPPV
RSAAHLPGLMCLLRLHGTVGGAQNLSAVGALVGLSNARLGSVKTRFEGLCLLSLLVGESP
TELFQQHCVSWLRSIQQVLQSQDPPPTMELAVAVLRDLLRYAAQLPTLFRDISTNHLPGL
LTSLLGLRPECELSAMEGMKACMTYFPRACGSLKGKLASFFLSRVDALSPQLQQLACECY
ARLPSLGAGFSQGLKHTESWEQELHSLLASLHGLLGALYEGADTAPVQCEGPGLDVLLAP
SEDGDAHTLLRLRHRFSGLARCLGLLLSSEFGAPVSVPVQEILDIICRTLSISAKNISLL
GDGPLRLLLLPSIHLDALDLLSTLILACGSRLLRFGALISRLLPQVLNAWNLGRDALPPG
QERPYSAVRTKVYAVLDLWVQVCGASAGVLQGGASGEALLSHLLSDISPPADALKLRSPR
GSPDGGLQSGKPSAPKKLKLDVGEATAPPGHRKGDSNANSDVCAAALRGLSRTVLMCGPL
IKEETHRRLHDLVLPLVMGVQQGEVLGSSPYTSSRCRRELYRLLLALLLAPSPRCPPPLA
CALQAFSLGQREDSLEVSSFCSEALVTCAALTHPRVPPLQSMGPACPAPAPAPPPEAPSP
FRAPPFHPPGPMPSVGPMPSVGPMPSVGPMPPAGPMPPTRPGPPATANHLGLSVPGLVSV
PPRLLPGPENHRAGSNDDPVLAPSGTPPPAVPPDETFGGRVPRPAFVHYDKEEASDVEIS
LESDSDDSVVIVPEGLPPLPPPPPTGTTPPPAAPAGPPTASPPAPAKEEPEELPAAPGPL
PPPPPXPPVPGPVALPPPQLVPEGPPGGGGPPALEEDLTVININSSDEEEEEEEEEEEEE
EEEEEEEEDFEEEEEEEEEEYFEEEEEEEEEEFEEEFEEEEGELEEEEEEDEDEDEEDELEEL
EEVEFGPAGGPAEEGGPPPPSPAPALPPAQPPEAPPEPGVEPGLLLEVEEPGPEDEPGAE
AAPTLAPEVLPSQGEGQREAGSPPAGPPPQELVEEEPSAPPPLLEEGTENGGDKVPPPPE
TPAAEEMEAAAEAETAALQEKEQDDTAAMLADFIDCPPDDEKPPAAPEPES
> Eublepharis macularius [A0AA97LBD5]
MAAAAASSGAAPAGPGPLVLETLAGLVRGPEPPGGEGGGSAALPRGLPGLVRCARDSGGG
GEAQASPALGGLVSVSHVRLGSLQTRFEGLCLLSLLVTGSSAEAFGQNCLGWLRSLQQVI
QSQDPPATMGLAVLVLRDLLGYSCQLPELARDLGTNHIPGLLTSLLALKPECQISALEGS
KACMMYYPRACGSLRGKLTTYFLARVDAETPHLQQLACDCYALLPSLGAGFAQGLKYTEC
WEQQVHCLLATLHSLLGTLYEGAETESLHYEGRGVEIPLPTPEQGETSFLLHLKLRFTGL
AKCLCRMLSNEFPAPVTVPVQDILDLICRTLNVSTKNFGWFGDGPLRMLLLPAVHLEILD
LLSALILACGPRLVRFGGILCRLFPQVLTSWSAGRDLLLPGQERPYSAVRTRLYQVLDLW
VQVAGAASGILQGHKPRSEALLGHLIADVSPPSETLKLQEGCPGPDGKPSAAKRPRLSDL
GSLGSVHRKYDPQANSDVCLAALQGLSRTVLLSGSLLKDQAHKRLQELVIPLLIRLSQAD
AFPGSPYSSACCRQELYRLLLALVLAPAPSCPPPLHCALRLLSQGRADASLQVSSFCAEA
LVVCSSLLHPRVPSLQLPLAGPSGPHPSSSEISPATASPFRPTPPLPFPAARLPPPASPS

APPSVATSANSLGLPLPSLNLGQLPPRLTPEEPPLPPSPGTAEAAALAAGVRMRRPIFIH
YDKEEEEDVELSLESDSDDSVVIVPKGQLGKVVNNTSAAAVAAAPAPPAPLPLPLAPPPA
APASPPPVSEEGPAEPAAPLPQGPPPPPPAAVVLPPSPAPVANDAVLPALVEEGPPTVIN
INSSEEEDEEEEEDEEEEDFPEDEDYFDEEEEEEFEEEEEEEEEEFEGELDEEEEGELDEE
ELEELGEEEEEEFDEEEEEEGLTEEEEEEEEEGLTEEEEEGLPPTLLPPRNNEEPPELLP
PVIEEAPKLVGEEEGAAGLLMEVDEEAFHPPEAAVAEELASSPAEAPPVPPLLLPGTPAL
PLPSTQEAPPPRAASVEREEKPCQEEEEGGPPDGVALAASSPGAAAAEEAAAPEGHAGDA
GEGSSEAGPLQGESEKLSLAEQEVVEATTTDETEAMLADFVDCPPDEEKMLLEPGS
> Aldrovandia affinis [A0AAD7X0R0]
MATAVWLHGSNNMRLTEGLVSALKEERPEYLPALLTNYREHGGISTQSSVAAGGLIGLSN
ARLSSSKTRFEGLCLLSILVKDSSSEVFQQHCLSWLRSLQQVIQSQAPLPSVQLAVSVLQ
DLLQYSSQLPELAREVGLNSILGILTSLLGLKSECHLVAMEGMTACMTFYPRACGSLRDK
LGAYFLSKMDSDNPKVQEVACKCYGHLPCLGGVLERGGGGRRAEGWANQLHCLLASAHSI
LGQLYQGAESERTMQYEGPGIELPFPLLDEADPLLVVQLQHRYRAVCLALKHTLSVDPVS
PVRLPVQNVLNLVCRALAVSCKSINVSGDGCLKLLVLPSVHSDTLELLSALINTVGSGLV
QYCSVLIRLFSQTLSAWSPLPEAGLGQQRAYSAVRVSLYHTLELWVRVAGATSAVLQGSP
THTELLMAHLLGDITPGADSIKLRVGQAVVSELMGHAGKAGPRRAKGLGIGDGSTVSLQR
KGDALANQDTCLSALRAMRQIILTSGTLLKEDIHKRLQDLVLPLCVRLQQHLKCGSEVGG
VSGQYGNAPPRRELYRLLLAMVLAPPPRWPPPLNCAVSIFSHGRKDRSLSVSSFCTEALT
VCNSLLHPRTPSLALPLPPLTLKHTPAAPNLTPSQNPSLSLPALLGGPTQGAPFPARHPL
SLGPAGLLGPLENHLPLPPSVLPPQAGPAPTPGDLLMSPSQPGELTALGAPEGHRPVFVR
YDKEEPEDVEISLESDSDDSVVIVPQGMLMLESQDSAGTQPLPPPPVGAVPAVGVGTGSD
TGVVDSPLPNELPTSLPHQILPSNSNAINTFPSQNQAQLVSLVPQLNSTGAQLTAPPVGL
GDSLPGAQLQQMLLQPSTASQSSQLGLPMQIQLQTQLAQSSRQLQQQQQQQQIASEEDL
TVININSSDEEDEEDEEMEDEDDLGEEEEEEEEEEEEEGLEDEEEEEEGSDFPEDDDYYGE
EEFEDFEDEEEGMIDAEEMEEEEAEGLPPLEGENRRALLGRQEGEVLMGSASERGLGMFR
IERGGEGDAEVEEGSSELEGGHSIYRQPLDPTEGNRELGLKEESEGEVGVAEEGKDKNGE
GEQEDAAVQESQGDPQPQGPVGEVMEKASELEKEAGVELGGQEGISPEPGPIQEGEDATA
VETALSRQDGDVDSLQGLRIAEEKGVEQTSEQPRPVQQEKPKQEVTIPEGEVRSAMPDEE
GGAIIEKEDEEEEDEEDLRGMKRKREEHEEEVAEQSIEKKKLDEEAMASMLADFVDCPPD
EEDNAHSPTHT
> Nothobranchius pienaari [A0A1A8P9H7]
MAASAWLHGSAAMRLTEGLVLLMKEQRPEFLPEVLANYREHGVLSLQSASDVAGLVGLCN
GKLSSSKTRLEGLCLLSMLVKDSSSDLFQQHCLSWLRSLQQVVQSQAPVQTIQLAVNVLK
DLLQYSCQLPELAREVGLNSILGILTSLLGLKTECELSAMEGMKACMVYYPRACGSLKDK
LGAYFLSKMDSTSRKTQEAACQGYSHLPCLGGLLDRAVGASRAEGWTNQIHCLLASANSL
LAQIYQGSETDEAVRYQGPGVELAFPHIDHAEPLLLLQHRYAAVCMTIKHTLRTDPASAV
SVPVRPILNLVCRALAVNSKNMILTADGSLRFLVLPSVHINALEVLSELITVVRSGMVQY
AAVIQRLFSQTLSAWTPLPETSVGQQRAYSSVRVSVFRSLELWVKVAGASACILHGTSSH
VDLLLNHLLGDITPGAESVKLRAGLSADVVPVGKPGPRRTKQLVVADAVGPSLQRKGDLL
SNQDTCLAALKALRQVLQSTGMLLKDDIHKRLHEVVLPLCVRLQQQQSSSISAWEPAGGV
SGQYSSALTRRELYRVLLALVLVPSPSWPPPLTCAVSILSNGRIDRHLKVSSFCAEALTI
CNSILHPRRPSLALPLPPLTLKPTATPSVLSCQGPGPRLTLPTLLGGPSSAPPFPARHTL
NLGPSSLLGSLDNHFSLVPGLPGQAPGPGDLMASSQTHHQPDPSGLAPPEGQRPVFVRYD
KEETEDVEISLASDSDDSVVIVPPGMLNVEKQQNDLAANSQSLLSSAPGGAELVTMVPTS
STPTTLDVTSLPNDLATSSALLTSSVTPVNSFPPSSTSVVSLVPSVNSSSASAPPGGLGD
PLAGKPQLQQMLMQPSTTGQPSSMALPLQMHQLTQQGRHLHQHPPTPAANEESAVININS
TDDEEDDEEDMEEDEELEDEEDGIDEEDEEEEGSDEFYEGEDYDDFDEEEGEELGEEEEE
EE
> Cyprinus carpio carpio [A0A9J7XQV1]
MVFQSTGAVGGLVGISNSRLGSSKTRFEGLCLLSVLVKDSSSEVFQQNCLSWLRTLQQVI
QSQAPLPTVQLAVSVLQDLLQYSSQLPELAREVGLNSILGILTSLLSLKSECHLVAMNGM
MACMIYYPRACGSLREKLGAYFLSKMDSDNPKVQEVACECYGRLPCLGGVLERGGGGRRA
EGWTNQLHCLLASANGMLGQLYQGAESEGTVQYEGPGVELPFPPLDDVDPLLVLQLHHRY
KAICLAIKHTLSADPASSVRLPVQHVLNFVCRALAVNTKSISPTGEGCLRLMILPSIHND
TLELLSALIKAVGGGLVQYSSVLTRLFSQSLSAWTPLPESSLGQQRAYSAVRVTVYRTIE
LWVRVGGASLLQASPSHTELLFTHLMGDITPASEAVKLRSGQQSQSMNDLIGSAGKSGPR
RTKGLGLGDGISLQRKGDVLANQDTCVAALRALRQIILTSGTLLKEDLHKRIQDLVVPLC
VRLQQQSHCVLEVGAISGQYGSPSPRRELYRLLLALVLVPSPHWPPPLSCAVSVFSHGRR
DRNIMVSSFCAEALTICNTLIHPRTPSICLPLTPLTLKSTPTAPVLSSGQNPSLSIPTLL
GGPATGPFPARHPMGLGPASLLGSLENHLPLAPPVLPTPAGTTAIPGDLLLSPAQPGELA
GLGAPEGQRQVFVRYDKEEPEDVEISLESDSDDSVVIMPAGMIMEMQDGAANAQSLSQSA
VPAIGGLQPSAPIVGEVGSVDTLLPNELPTSIPHQILPANANNINSFPGTSQTAQLVSLV
PPLNSSVSPAGLADSMTGGPQLQQMLMQTSPGGQPPTLGLSLQMQLQNQIAQTSRQLQTQ
PPANEVDQNVININSSDDEEEEEEEELEEEDELGEEEEEEEEGLEDEEEEEEGSDLIDGEYC
EDELEDYDDEEDEDDEESEEIQPLEGDSDRGMIGEEDAEVMIEAEQQGMEMFCMEREREV
EPGIEEMEGVRSVYADERIKDKGTMEEIENIGAVERNEPVVDKDQIESLVISGDAEGHEE
DSRVEVVEPEVKTCGQEVARPEDPVEDAGLTQQGQELTVEDEVQKQEPELKPEGTTNQSA
PSTSEQEVLQSVAETAEEEVGKESGEQGEDSETRGTKRKMEDREEGESSEQGTEKKKMDD
EAMASMLADFVDCPPDDDDGGASQSQT
> Microcebus murinus [A0A8B7HDA4]
MAAAVLSGPSAGSAAGVPGGTGGLSAVSSGPRLRLLLLESVSGLLQPRTGSTVAPVHPPI
RSTPHLPGLMCLLRLHGTVGGAQNLSAVGALVSLSNARLGSIKTRFEGLCLLSLLVGESP
TEIFQQHCVSWLRSIQQVLQSQDPPPTMELAVAVLRDLLRYAAQLPALFRDISMNHLPGL
LTSLLGLRPECEQSALEGMKACMTYFPRACGSLKGKLASFFLSRVDSLVPELQQLACECY
SRLPSLGSGFSQGLKHTESWEQELHSLLASLHSLLGALYEGAETAPVQNEGPGVETLLSS
SEEGDTHVLLRLRQRFSGLARCLGLMLSSEFGAPVSVPVQEILDLICRTLSVSGKNISLL

GDGPLRLLLLPSIHLEALDLLSALILACRGRLLRFGVLISRLLPQVLNAWSIGRDSLSPG
QEKPYSTIRTKVYAVLELWVKVCGASAGVLQGGASGEALLTHLLSDISPPADALKLRSPR
GSPDGGLQTGKPSAPKKLKLDVGEAMAPPSHRKGDSNANSDVCVAALRGLSRTILMCGPL
IKEETHRRLHDLVLPLVMGVQQCEALGTSPYTSSCCRRELYRLLLALLLAPAPRCPPPLA
CALQAFSLGQQEDSLEVSSFCSEALVTCAALTHPRVPPLQSMGPTCPTPAPVPPPEAPSP
FRAAPFHPPGPMPSVGPMPSAGPMPSAGPMPSTGPMPSAGPVPPARPGPPATANHLGLPV
PGLVSVPPRLLPGPENHRAGSNEDPVLAPSGTPPPTIPPDETFGGRVPRPAFVHYDKEEA
SDVEISLESDSDDSVVIVPEGLPPLPPPPPSGTTPPPVAPSGPPTASPPVPAKEEPEELP
APPGPLPPPPPPPPPVPGPVTLPPPQLVPEGTPGGGGPPALEEDLTVININSSDEEEEEE
EEEEEEEEEEEEEEEDFEEEEEDEEEYFEEEEEEEEEFEEEFEEEEGELEEEEEEEEDEE
EEELEEVEELEFGSAGGEVEEGGPPPPTLPPALPPPESPKAQPEPQPEPEPGLLLEVEEP
GAEEEHEAETAPTLALEVLPSQGEVEREGGSPAAGPPPQELVEEEPSAPPTLLEEGTEGG
ADKVPPPPETPAEEEMETEAEATALQEKEQDDTAAMLADFIDCPPDDEKPPPTTEPDS

> Capra hircus [A0A8C2SCF6]
KMAAAVLSGPSAGSAAAVPGGAGGLSAVGSGPRLRLMLLESVSGLLQPRTGSAVAPVHPP
ARSAPHLPGLMCLLRLHGTVGGAQNLSAVGALVGLSNARLSSIKTRFEGLCLLSLLVGES
PTEMFQQHCVSWLRSIQQILQVSLLPATPHLRPIFRDISMNHLPGLLTSLLALRPECELS
ALEGMKACMTHFPRACGSLKGKLASFFLSRVDALSPQLQQLACECYSRLPSLGAGFSQGL
KHTDSWEQELRSLLASLHSLLGGLYEGAEAAPMQYESPGAETLLSPSEDADAHTLLRLRQ
RFSGLARCLGLMLSSEFGAPVSVPVQDILDLICRTLSVSAKNVSLLGDGPLRLLLLPSLH
LEALDLLSALILACGARLLRFGALISRLLPQVLSAWSIGRENLGPGQERPYSTVRTKVYA
VLELWVQVCGASAGVLQGGASGEALLSHLLSDISPPADALRLRSPRGSPDAGLQTGKPSA
PKKLKLDVGEAIAPPSHRKGDSNANSDVCAAALDDGRVVWRYLQVCALSFQRLHELVLPL
VMGVQQGEALCSSPYTSSHCRRELYRLLLALLLAPSPRCPPPLACALRREDSLEVSSFCS
EALVTCAALTHPRVPPLQSVGPTCPAPAPVPPPEAPAPFRAPAFHAPGPLPSAGPMPSAG
PMPPAGPLPTTRPGPPATANHLGLSVPGLVSVPPRLLPGPENHRAGSSEDPVLAPSGSPP
PTVPPDETFGGRVPRPAFVHYDKEEASDVEISLESDSDDSVVIGLPPPPPPPSGTTPPPV
APAGPPAASPPVPAKDEPEELPAAPGPLPPPPPPPVPGPVTLPPPQLVPEGTPGGGGPPA
LEEDLTVININSSDEEEEEEEEEEEEDEEEDEEEDFEEDEEEEEYFEEEEEEEEEFEEEF
EEEEGELEDEEDEEEDEELEELEEVEFGPAGGEVEGGGPAPPSLPPVLPPTESPKGPPEP
GLEPGLLLEVEEPGTEEAPGPEMAPMLAPEVLPSQGEVEREGGSPPAGPPPQELVEEEPS
GPPTLLEEGAEGGGDNDMHPDLVCVCDCSELFVCPWKEQGDTAAMLADFIDCPPDDEKPP
PVSEPDS
> Equus asinus [A0A8C4MIG0]
MAAAVLSGPSAGSAAGAAGGTGGLSAVASGPRLRLLLLESVSGLLQPRAGSTVAPVHPHV
RSAAHLPGLMCLLRLHGTVGGAQNLSAVGALVGLSNARLGSIKTRFEGLCLLSLLVGESP
TEMFQQHCVSWLRSIQQVLQSQDSPPTMELAVAVLRDLLRYAAQLPTLFRDISMNHLPGL
LTSLLGLRPECELSALEGMKACMTYFPRACGSLKGKLASFFLSRVDALSPQLQQLACECY
SRLPSLGAGFSQGLKHTESWEQELHSLLASLHSLLGALYEGAETAPMQYEGPGVEVLLSP
SEDGDAHALLRLRQRFSGLARCLGLMLSSEFGAPVSVPVQEILDVICRTLSISAKNISLL
GDGPLRLLLLPSIHLEALDLLSALILACGGRLLRFGALISRLLPQVLNAWSIGRDTLSPG
QERPYSTMRTKVYAVLELWVQVCGASAGVLQGGASGEALLTHLLSDISPPADALKLRSPR
GSPDGGLQSGKPSAPKKLKLDVGEAMAPPSHRKGESNANSDVCAAALRGTIPAWGKMSPV
ELTFCPGLSRTILMCGPLIKEETHRRLHDLVLPLVMGVQQGEVLGSSPYTSSRCRHELYR
LLLALLLAPSPRCPPPLACALQAFSLGQREDSLEVSSFCSEALVTCAALTHPRVPPLQSM
GPTCPTSAPVPPPEAPSPFRAAPFHPPGPMPSAGPMPSAGPVPSAGPMPSVGPLPSVGPM
PSVGPMPPARPGPPATANHLGLSVPGLVSVPPRLLPGPENHRAASNEDPVLAPSGTPPPA
IPPDETFGGRMPRPAFVHYDKEEASDVEISLESDSDDSVVIVPEGLPSLPPPPPSGTTPP
PVAPAGPPTASPPVPAKEEPEELPAAPGPLPPPPPPPVPGPVTLPPPQLVPEGTPGGGGP
PALEEDLTVININSSDEEEEEEEEEEEEEEEEEEEEEDFEEEEEDEEEYFEEEEEEEEEF
EEEFEEEEGELEEEEEEEDEEEEEELEEVEELEFGSAGGEVDEAGPPPPSLPPALPPAES
PKVQPEPEPEPEPGLLLEVEEPGVEEERGAETAPTLAPEVLPSQGEVEQEGGSPPAGPPPQE
LVEEEPSAPPTLLEEGTEGGDDKVPPLPEPPAAEEMETETEVAALQEKEQDDTAAMLADF
IDCPPDEEKPPSATEPDS
> Leptobrachium leishanense [A0A8C5ML02]
MTRPAGGKMAASACVGQRMMETVVTGLLERDLSEGELGEAIRGLREHGALRVEGSVAALS
GLLSSCNSRLVSPSTRVEGLSLLSLVVEESPTDVFHQHCVSWIRSVLQVIQSQDPPRVVS
FAVFVLRSLLAHSSALPELSREISTNLIPGLLTSLLGLRNQCLIPALEGIRSCLISYPRA
CGSLRGKLTTFLLSLLDVENLEIQELACQCFSLLPSLGSGFSQGVKHTENWERQIQSVLC
SLHSVFRQLYQSAETDSTRYEGPGTELELPRLEDDGTHSVLQLVRRLTALGQCVGLLLRQ
PFPAPVRVPVSDLLSFVCRVINVSPKNLSWQGEESVKLLLLPRVHGIVLDILEATITACG
PRLLPYSSVICRLFPQLLSAWGAMRGAAGTIAGQERPFSSLRVSVYRVLEKWLSVCGVSS
GVLQGPVHHSDILLAHLFSDITPPSDTVKMSGFVQLGAKKQKVSEVGGTDFQGHRKKQLS
ANIDLCAAALRVLCSIVLHCGSVIKEDTHRRLQEFSIPLLIRLQQGTESSLGPYVSSDCR
KELYHLLLCLTLTPSPRLPAPLHCAIRVFSGGMVEESVQVSLFCAEAIAICRALIHPRGP
SLQHPLPLQGPRPPAVTGDAPIQRTPAPATTFPVLPPSNHLPPRPSALPIEPAVATAEPS
PPQEETFGGRPRHAVFIHFEKEEPSDVEISLESDSDDSVVIVPEGLLSKSAPKPEPSPPA
IKPPQEETAEVPPPPVLPSVPSVSTAPPPAPPPACTGPPPAPLPLPVPEVPVQPMPEGSM
TVININSSDDEGEDEEEDEEEEEMYDDEEEEFYDDEEEEDLEGLDEEDYEEDEEGLTEEE
EEEDLEEGEDEEEEGEDEEDEEGDMAEEMQIGPAPVEIQEDTEPVEPEEPVLQEPELEEG
PPRLSPVQEDDGGDTGLLMLVESEEKEIPQEEEPQEKVSLPEPEVNRSPPPPPVLTPPPP
PVLPPETDENEGLHLQSEPEEEPVVTPVTEDTVQDKEVVMEEEEAEEEDEKKLEAEKIEE
IEEKIADAETMLADFVDCPPDDDKPMESAT
> Leptobrachium leishanense [A0A8C5PDH4]
MTRPAGGKMAASACVGQRMMETVVTGLLERDLSEGELGEAIRGLREHGALRVEGSVAALS

GLLSSCNSRLVSPSTRVEGLSLLSLVVEESPTDVFHQHCVSWIRSVLQVIQGKLTTFLLS
LLDVENLEIQELACQCFSLLPSLGSGFSQGVKHTENWERQIQSVLCSLHSVFRQLYQSAE
TDSTRYEGPGTELELPRLEDDGTHSVLQLVRRLTALGQCVGLLLRQPFPAPVRVPVSDLL
SFVCRVINVSPKNLSWQGEESVKLLLLPRVHGIVLDILEATITACGPRLLPYSSVICRLF
PQLLSAWGAMRGAAGTIAGQERPFSSLRVSVYRVLEKWLSVCGVSSGVLQGPVHHSDILL
AHLFSDITPPSDTVKMSGFVQLGAKKQKVSEVGGTDFQGHRKKQLSANIDLCAAALRVLC
SIVLHCGSVIKEDTHRRLQEFSIPLLIRLQQGTESSLGPYVSSDCRKELYHLLLCLTLTP
SPRLPAPLHCAIRVFSGGMVEESVQVSLFCAEAIAICRALIHPRGPSLQHPLPLQGPRPP
AVTGDAPIQRTPAPATTFPVLPPSNHLPPRPSALPIEPAVATAEPSPPQEETFGGRPRHA
VFIHFEKEEPSDVEISLESDSDDSVVIVPEGLLSKSAPKPEPSPPAIKPPQEETAEVPPP
PVLPSVPSVSTAPPPAPPPACTGPPPAPLPLPVPEVPVQPMPEGSMTVININSSDDEGED
EEEDEEEEMYDDEEEEFYDDEEEEDLEGLDEEDYEEDEEGLTEEEEEEEDLEEGEDEEEE
GEDEEDEEGDMAEEMQIGPAPVEIQEDTEPVEPEEPVLQEPELEEGPPRLSPVQEDDGGD
TGLLMLVESEEKEIPQEEEPQEKVSLPEPEVNRSPPPPPVLTPPPPPVLPPETDENEGLH
LQSEPEEEPVVTPVTEDTVQDKEVVMEEEEAEEEDEKKLEAEKIEEIEEKIADAETMLAD
FVDCPPDDDKPMESAT

> Microcebus murinus [A0A8C5XYE9]

MLLPDLSRRVQPPFPHVRRGVFASSRHAATRARGKMAAAVLSGPSAGSAAGVPGGTGGLS
AVSSGPRLRLLLLESVSGLLQPRTGSTVAPVHPPIRSTPHLPGLMCLLRLHGTVGGAQNL
SAVGALVSLSNARLGSIKTRFEGLCLLSLLVGESPTEIFQQHCVSWLRSIQQVLQSQDPP
PTMELAVAVLRDLLRYAAQLPALFRDISMNHLPGLLTSLLGLRPECEQSALEGMKACMTY
FPRACGSLKGKLASFFLSRVDSLVPELQQLACECYSRLPSLGSGFSQGLKHTESWEQELH
SLLASLHSLLGALYEGAETGRGGQWLESWAGGNRRGPPALILFSSEFGAPVSVPVQEILD
LICRTLSVSGKNISLLGDGPLRLLLLPSIHLEALDLLSALILACRGRLLRFGVLISRLLP
QVLNAWSIGRDSLSPGQEKPYSTIRTKVYAVLELWVKVCGASAGVLQGGASGEALLTHLL
SDISPPADALKLRSPRGSPDGGLQTGKPSAPKKLKLDVGEAMAPPSHRKGDSNANSDVCV
AALRGLSRTILMCGPLIKEETHRRLHDLVLPLVMGVQQCEALGTSPYTSSCCRRELYRLL
LALLLAPAPRCPPPLACALQAFSLGQQEDSLEVSSFCSEALVTCAALTHPRVPPLQSMGP
TCPTPAPVPPPEAPSPFRAAPFHPPGPMPSVGPMPSAGPMPSAGPMPSTGPMPSAGPVPP
APTANHLGLPVPGLVSVPPRLLPGPENHRAGSNEDPVLAPSGTPPPTIPPDETFGGRVPR
PAFVHYDKEEASDVEISLESDSDDSVVIVPEGLPPLPPPPPSGTTPPPVAPSGPPTASPP
VPAKEEPEELPAPPGPLPPPPPPPPPVPGPVTLPPPQLVPEGTPGGGGPPALEEDLTVIN
INSSDEEEEEEEEEEEEEEEEEEEEEDFEEEEEDEEEYFEEEEEEEEEFEEEFEEEEAAM
LADFIDCPPDDEKPPPTTEPDS

>Scleropages formosus [A0A8C9TNY7]

MATAALLHGSTNMRLTEGLVSALKLERPEYLPSLLANYREHGGVSAQSSAAVSGLIGLSN
GQLSSSKTRFEGLCLLAVLVRDSSSEVFQQHCLTWLRLLQQVIQSQAPLPSIQLAIVVLQ
DLLQYSSQLPELAREVGLNSILGILTSLLGLKLECHLVAMEGMKACMTFYPRACGSLKDK
LGAYFLSKMDSDNPRVQEVACECFGRLPCLGGVLERGGGSRRAEGWASQVHCLLATAHSL
LGQMYQGAESEGAVQYEGPGMELPLPPLEEADPLLMLQLPQRYRAVCLALTQTLSMDPNS
PVSLPVQSVLNLVCRALAVSCKNIVSPNTSGDGCFKLLFLPSVHSDTLEVLSTLITVAGS
RLLQYSNVLSRLFSQTLSAWSPPPEGSPGQQRAFSAVKVCLYRTLELWVRVAGASAGILH
GSPTHNELLLTHLLGDITPGPDSVKLRAGQPAVSELVGHGGKAAAAAARRAKGMGDVTGV
SIQRKGDALANQDTCVSALRGEGTSHCVCARARALSRLQDLVLPLCVRLQQQPCGSDVGT
GSSQYGSAPPRRELYRLLLALVLTPPPRWPPPLPCAVSIFSHGRRDHSLTVASFCTEALA
ICNSLLHPRVPSIALPLPPLALKHTPAAPNLTPSQNPSLSLPTLLGGPTQASPFPARHPL
GLGPPSLLAPMENHLPLPTPVLPPQAGSTPAPGELLSPPQPGELAALGPNEGRRPLFVRY
EKEEAEDVEISLESDSDDSVVIVPPDMLMQETQEPAGTQPIHPPPGGAVPGLTVSAASET
GAVNSPIPNELPTTMALPSNSNAVTTFPAQSQTQLVSLVPPLSSGATQLAAPSVSLGDSL
PGTQLQQMLLQSSPAGQASQLGLPVQIQLQTQLAQPSRQPQPQQMTNEEDLTVININRSE
FAEEYYDGEEFGDYEEEEEEEEEEMIEGEDEEGMIEGEEEEAEELPALETGEGRRLLLSV
EEEAVGSGGEGTMEIFCGEEGGESQGTGRETEEQPRSYGQDSVQEDVKQADERAAGVLEE
QGDKLVDVERESEALQDGKTDSQPHGAAGDSSQEADITQGEGTVVDGPEVTSKEQEAAQE
EDAPTVDETAASLQEPMTESPEDVGKVDEKAGERPSVDQPPVELREESKEQSMKRKREIN
EEGEGEGEVRPSVEKKKEAMASMLADFVDCPPDEEENARSPAQSEG

> Sus scrofa [A0A8D0QP19]

MAAAVLSGPSAGSAAGVPGGTGGLSAVVSVPRLRLLLLDSVSGLLQPRAGSTVAPVHPPA
PSAPHLPGLMCLLRLHGTVGGAPNLSAVGALVGLSNARLGSIKTRFEGLCLLSLLVGESP
TEMFQQHCVSWLRSIQQVLQSQDPPPTMELAVAVLKDLLRYAAQLPTLFRDISMNHLPGL
LTSLLGLRPECELSALEGMKACMTYFPRACGSLKGKLASFFLSRVDALSPQLQQLACECY
SRLPSLGAGFSQGLKHTESWEQELHSLLASLHGLLGALYEGAETAPMQYEGPAVEALLSP
SEDGDAHVLLRLRQRFSGLARCLGLLLSSEFGAPVSVPVQEVLDVICRTLSVSARNISLL
GDGPLRLLLLPSLHLEALDLLSALILACGGRLLRFGALISRLLPQVLSAWSIGRDTLSPG
QERPYSTMRTKVYAVLELWVQVCGASAGVLQGGASGEALLTHLLSDISPPADALKLRSPR
GSPDGGLQTGKPSAPKKLKLDMGEPIAPPSHRKGDSNANSDVCAAALRGLSRTILMCGPL
IKEETHRRLHELVLPLVMGVQQGEVLGSSPYTSSRCRRELYRLLLALLLAPSPRCPPPLA
CALQAFSLGQREDSLEVSSFCSEALVTCAALTHPRVPTLQSMVPTCPTPAAVPPPEAPSP
FRAPAFHPPGPMPSAGPMPSAGPMPSAGPMPSVGPMPPARPGPPATANHLGLSGSSLVSV
PPRLLPGPENHRAGSNEEPVLAPSGTPPPTLPSDETFGGRVPRPAFVHYDKEEASDVEIS
LESDSDDSVVIVPEGLPPLPPPPPSGTTPPPVAPAGPPAASPPVPAKEEPEELPAAPGPL
PPPPPTPVPGPATLPPPQLVPEGTPGGGAPALEEDLTVININSSDEEEEEEEEEEEEEEE
EEEEEEEDFEEEEEDEEEYFEEEEEEEEEFEEEFEEEEGELEEEEEDEEEEEEEELEEVE
ELEFGSAGAEVEEGGPPPPSLPPALPPAESPKVQPEPEPEPGLLLEVEEPGAEEAPGPET
APTLVPEALPAQGEAEREAGSPPTAPPPQELVEEEPSVPPTLLEEGAEGGGDKVPPPPET
SAAEEMETETESTALQEKEQDDTAAMLADFIDCPPDDEKPPPAAEPDS

> Canis lupus familiaris [A0A8I3P804]
MAAAVLSGPSAGSAAGVPGGAGGLSAVGSGPRLRLLLLESVSGLLQPRAGSAVAPVHPPV
RSAPHLPGLMCLLRLHGTVGGAQNLSAVGALVGLSNARLGSIKTRFEGLCLLSLLVGESP
TEMFQQHCVSWLRSIQQVLQSQDPPPTMELAVAVLRDLLRYAAQLPTLFRDISMNHLPGL
LTSLLGLRPECELSALEGMKACMTYFPRACGSLKGKLASFFLSRVDALSPQLQQLACECY
SRLPSLGAGFSQGLKHTESWEQELHSLLASLHSLLGALYEGAETAPVQYEGPGVEVLLTP
SEDGDTHVLLRLRQRFSGLARCLGLMLSSEFGAPVSVPVQEILDVICRTLSISAKNISLL
GDGPLRLLLLPSIHLDALDLLSALILACRSRLLRFGALISRLLPQVLNAWNLGRDTLAPG
QERPYSTVRTKVYAVLELWVQVCGASAGMLQGGSSGEALLSHLLSDISPPADTLKLRSPR
GSPDGGLQTGKPSAPKKLKLDVGEAMAPPSHRKGDSNANSDVCTAALRGLSRTILMCGPL
IKEETHRRLHDLVLPLVMGVQQGEVLGSSPYTSSRCRQELYRLLLALLLAPSPRCPPPLA
CALQAFSLGQREDSLEVSSFCSEALVTCAALTHPRVPSLRSMGPACPTPAPAPPPEAPSP
FRAPPFHPPGPMPSAGPMPSVGPMPSVGPMPPAGPMPPAGPMAPTRPGPPATANHLGLSV
PGLVSVPPRLIPGPENHRAGSNEDPVLAPSGTPPPAIPPDETFGGRVPRPAFVHYDKEEA
SDVEISLESDSDDSVVIVPEGLPPLPPPPPSGTTPPPVAPAGPPVASPPVPAKEEPEELP
VAPGPLPPPPPPVPGPVALPPPQLVPEATPGGGGPPALEEDLTVININSSDEEEEEEEEE
EEEEEEEEEEEEEEEEDFEEEEEEEEYFEEEEEEEEEFEEEFEEEGELEEEEEEEEDEEE
EEELDELEEVAFGPAAGAAEEGGPPPPSPPPALPPAQSPKMQPEPPGETGLLLEVEEPAA
EEEPGAEAAPTLAPEVLPPQGEGPREVGSPPAVPPPQELIEEEPPAPPTLLEEGTESGGD
KVPVPQETPAAEDVEAEVEAETAALQEKVGDRGRSLGGKVGWAAAAKLSPACLSGACACF
QPYVFVSERSRMTRLPCWLTSSIVPLMTTSHHRPQSLIPSPLWRPPLPVSNKVMSWDNTC
CFCLLHKCVPGQKPCQLPGFTRYPWRSPCVCMASWGLRHPQGDPLT
>Callithrix jacchus [A0A8I3WBT1]
MCYARGGRGSHRSPLQICSFIIHLWHLTLISENLSAVGALVSLSNARLSSIKTRFEGLCL
LSLLVGESPTELFQQHCVSWLRSIQQVLQSQDPPPTMELAVAVLRDLLRYAAQLPALFRD
ISMNHLPGLLTSLLGLRPECEQSALEGMKACMTYFPRACGSLKGKLASFFLSRVDALSPQ
LQQLACECYSKLPSLGAGFSQGLKHTESWEQELHSLLASLHTLLGALYEGAETAPVQHEG
PGVEMLLSSEDGDAHVLLRLWQRFSGLAHCLGLMLSSEFGAPVSVPVQEILDFICRTLSV
SSKNISLHGDGPLRLLLLPSIHLEALDLLSALIVACGSRLLRFGIPISRLLPQVLNSWSI
GRDSLSPGQERPYSTVRTKVYAVLELWVQVCGASAGVLQGGASGEALLTHLLSDISPPAD
ALKLRSPRGSPDGGLQTGKPSAPKKLKLDVGEAMTPPSHRKGDSNANSDVCAAALRGLSR
TILMCGPLIKEETHRRLHDLVLPLVMGVQQGEVLGSSPYTSSRCRRELYCLLLALLLSPS
PRCPPPLACALQAFSLGQREDSLEVSSFCSEALVICAALTHPRVPPLQSMGPTCPTPAPV
PPPEAPSPFRAPSFHPPGPMPSVGPMPSAGPMPSAGPMPSAGPVPSAGPVPSARPGPPTT
ANHLGLSVPGLVSVPPRLLPGPENHRASSNEDPILAPSGTPPPTIPPDETFGGRVPRPAF
VHYDKEEASDVEISLESDSDDSVVIVPEGLPPLPPPPPSGATPPPVAPSGPPTASPPLPA
KEEPEELPAAPGPLPPPPPPPPPVPGPVTLPPPQLVPEGTPGGGGPPALEEDLTVININS
SDEEEEEEEEEEEEEEEEEEEEEDFEEEEDEEEYFEEEEEEEEEEFEEEFEEEEGELEEE
EEEEDEDEEEEELEEVEELEFGTAGGEVEEGGPPPPTLPPALPPPESPPKVQPEPEPEPG
LLLEVEEPGAEEEHEADTAPTLAPEVLPSQGEVERDEGSPEAGLPPQELVEEEPSAPPTL
LEEGTEDGGDKVQLPPETSAEEEMETETEAEALQEKEQDDTAAMLADFIDCPPDDEKPPP
ATEPDS
> Xenopus laevis [A0A8J0URX3]
MAAVCVGTRGMEVTIAGILERDLSEGELAEAIRGLREHGAFRGEGLPAAMSGLLSSCNSR
LTSASSRIEGLSLLALAVEESPTDVFVQHCVSWLRSLLQIIQSQDPPRVVSLAVFVLRSL
LAHSSALPELSREISTNHIPGLLTSLLGLRRQCLVPALEGIRSCFLSYPRACGSLRGKLT
AFLLSLLDAENQQIQEVACQCYSLLPSLGSGFSQGIKHTENWERQIQSVICSLHSVFLQL
YQGSETDTARYEGSGTELEFPSVEDDGTHGVLQLARRFTALGQCMRLLLREQFPAPVRVP
VSDILSLVCRVVNVSPKNLSWHGEESLKLLLLPRVHSSILEILEATIIACGPRLLPFSAV
ICRLFPQLLLSWAAVKGITGIPSGQERPYSSLRCSVYRVLETWVTTCGISSGVLQGPMHH
SDILLANLLSDITPPTDAIKMSTFVQLGAKKQKVSEVGDDDFQSHRKRDNTANVELCAAA
LKGLCCVILHCGSVIKEDVHRRLQELSIPLLLRLQQGSDQWLGPYISSDCRKELYRLLLC
LTLTPNPKLPAPLHCAIRIFRGGTTEESLQVSRFSTEALAICRILIHPRVPSLQRPLPHL
APRPPVQSDAPTLRPPAALSTFPAMPPANHLPPRPTVPAMSTEPPIPAAVPSPPPEESFG
EKPRRAVFIHFDKEEPSDVEISLESDSDDSVVIVPEGLFAKSDSKPEPSPPAVKPPTEEV
TEQVAPSAVPSSSTAAPPPPPPPPPAPPVCAGPSSAPVPIAEAPPPPQQEVDTVININSSD
DEEDGEEDEEEGLYDDEDEEDYYDEEEDEDLEGLEEDDYEEDEEGITEEEEEDLEEEGED
DEEEVEDEECLMPDEMQIGSEAEEIPDGIETSSLHEGELEEGPPRLSPVQEDEAVDTGLL
MLVESEDREPSGEAPGEGLPESDLTRSPPQPPVLTPPSPPDEPPPMEESDVPPLEEPDLE
VAPVAEEPVEETEEKKPEEVTVEKPEPEPEEEVSAQVTLRNTPGATQTNRCLSSDLSQEQ
IADADAMLADFVDCPPDDDKLPEPCT
> Silurus meridionalis [A0A8T0BHE3]
MATAAWLHGPKNMRLTEGLVSALKEERPEYLPALLADYREHGVVGTQNCGTVGGLVGISN
SRLGSSKTRFEGLCLLSMLVKDSSSEVFQQHCLSWLRSLQQIIQSQAPLPSVQLAVSVLQ
DVLHYSSQLPELAREVGLNSILGILTSLLGLKSECQLAAMEGMMACMTYYPRACGSLKEK
LGVYFLSKMDSDNPKVQDVACECYGRLPCLGSVLERGGGGRRAEGWTSHVHCLLVSANSI
LRQLYQGIETEEAIQYEGPGVELSFSPLDDVDPLLILQLRHRYKAVCLALKHTLSVDPAT
SVRLPIQQVLNVVCRALAVSIKNINVTGEGCLKLLVLPSIHSDSLEVLSALIKAVGAGLV
QYCNVLSRLFSQALCAWSPLPEASLGQQRSYSAVRVALYNTLELWVHVGRASSSVLQGNS
SHSELLFAHLIGDITPGNEAVKLRAGQTPISDLVTAGKPGPRRTKGMGIGDPGGVSLQRK
GDALANQDTCFAALRVLRQIILTSGTLLKEDLHKKLQELVVPLCARLQQQAQSSNWDVDS
VNGQYGSAAPRCELYALLLALVLVPSPRWPAPLSCAVCVFSQGRRDRNITVSSFCAEALT
ICNALLHPRTPSISLPLPPLTLKPTPASSVLAPTQNPSLSLPTLLGGPAPGPSFAPRHPL
SLGTASLLGSLENHLPLGAPVLPTPAGATGTQGELLLSSSAQAAELAGLAPPETQRQVFV
RYDKEEPEDVEISLESDSDDSVVIMPQSMMLEMQDGAANTQSLPPPPGSAMPVPNAGLGN
EAGPVETSLSNDIPTSIAHQLMPPDSNINSFPGSSQTEQLVSLVPPLNSNAVTLPVSSSA

LGNSLPAGAQLQQMLMQPSSGGQPNQLGLSLHMQLQNQLVQSSRQPAANEQDQNVININS
TDDEEEEEEEEMEDEEELGEEEEEEEGLEEDEEEDERRYRDSFCREEYECFDDEEEDDDE
GDEMEEEDEEEEDDEEEIQEAEILEAENRRNVLGAEEGEVMIEGQEERGVGPFPIEGERQ
EEGGIEEMKAVQSIYEEEEIKDKGGVEEIENIGAVERNESVVGEQQIETHVIGAEAEQSE
ENASIEAADQEVQSQEEEASGPQSVVTAEDSGIIPEGQEQEVAIEVRDDEQSAAAQQEEI
PNANTATTSEDAVPQQAVEAAEKEVAGETQVEQEEARGTKRKIEDREEGEESEQSSEKKK
LDDEAMASMLADFVDCPPDDEDHGASHSHA

>Hemibagrus wyckioides[A0A9D3SST9]

MATAAWLHGPKNMRLTEGLVSALKEERPEYLPALLANYREHGVVGTQNCGTVGGLVGISN
ARLGSSKTRFEGLCLLSMLVKDSSSEVFQQHCLSWLRSLQQIIQSQAPLPSVQLAVSVLQ
DVLQYSSQLPELAREVGLNSILGILTSLLGLKSECHLAAMEGMMACMTYYPRACGSLKEK
LGVYFLSKMDSDNPKVQDVACKCYGRLPCLGGVLERGGGGRRAEGWTSQVHCLLASANSF
LGLLYQGIETEETMQYEGPGVELPFPPLDDVDPLLILQLRHRYKAVCLALKHTLSIDPAT
SVRLPIQHVLNLVCRALAVGIKNINVTSEGCLKLLVLPSIHSDTLEVLSALIKAVGAGLV
QYCNVLSRLFSQALCAWTPLPEASLGQQRAYSAVRVALYHTLELWVRAGKASSSVLQGSS
SHSELLFAHLIGDITPGSEAVKLRAGQTAMSDLVAAGKTGPRRTKGMGIVDPGGVSLQRK
GDALANQDTCFAALRVLRQIILTSGTLLKEDLHKKLQELVVPLCVRLQQQAQCSNWDVGG
VTGQYGSAAPRCELYALLLALVLVPSPRWPAPLSCAVCVFSQGRKDRNITVSSFCAEALT
ICNALLHPRTPSISLPLPPLALKPTSASSVLAPTQNPSLSLPTLLGAPAPGPSFASRHPL
SLDSASLLGSLENHVPLGPPVLSTPGGVTGAQGELLLSSPAQAAELAGLAAPPETQRQVF
VRYDKEEPEDVEISLESDSDDSVVIMPQGMMQEMQEGAANTQSLPPPPGSAMPVPNAGLG
NEAGPVETSLSSDLPTTIGHQMLPADGNNINSFPGSSQTEQLVSLVPPLNSNAVPLAASS
SALGNSLPAGAQLQQMLMQPSQGGQPSQLGLSLHMQLQNQLAQSSRQPAANDQDQNVINI
NSTDDEEEDEDEEMDDEDELGEEEEEEEGLEEDDEEEDEFRSGYYRHGEFEGFDDEEDEG
EEMDEEEEEEDDEEEIQALEADNRRDVIDAEEGEVMMEGQEERGVGTFPMEGERPVEAGI
EEMKAVQSIYDEEEEIKDKAGIEEIENIGAVERNESVVGEQQIETHIIGAEGEQSEENTS
TEAADQEVQPQEQEESRPESVVTPEDSGIPSECQEQEVAIEVRDNEPSAEAGQEEIPNPS
TATTSEDAASQQVVETAEKEMVEGVQAEEKEARGTKRKMEDIEEGEVSEQNSEKKKLDDE
AMASMLADFVDCPPDDEDHGASHSHS

> Mauremys mutica [A0A9D3XX58]

MAAAASGCPGRLVLEALGGARPLPGLVRGLRDSGQNLQALGGLIGATNARLGTVKTRFEG
LCLLSLLVSESPTELFQPHCVGWLRALQHLLQSQDPAPTMALGVAVLRDLLLYSCQLPEL
GRDIATNHIPGLLTSLLALKPECQLSALEGIRACMTCYPRACGSLRGKLAAYFLSRVDAE
SPQLQQLACECYALVPSLGAGFTHGLKHTECWEQELHALLATLHGLLGNLYEGAETDPLP
YEGPGVELLLPPAPPDGETNFVLNLCNRFSGLAKCLELLLSSEFVAPVTVPVQDVLDLVC
RALNISSQNISWFGDGPLKMLLLPSVHLEILEVLAALILACGARLARWGSVLGRLFPQVL
GAWSSSRDSVPLGQEKPYSAVRTRLYQVLELWVQVGGAGAGVLQGPPQHSEALLAHLLSD
IAPPTDSVKLKVGRPGSEGKPSAPKKPKLSEGGDAPSLHRKQDPMANSDVCKAALQALSR
AILLGGSLVKEETHRRLQELVVPLLLRLAQGDVPPGGPYASPACRHQLYRLLLALLLAPA
PACPPPLHCALRAFALGQRDPSLQVSSFCAEALVTCSALARPRVPSLQLPLPGPAPSAGP
APADLAASPFRQAPPFPAPPPARPPPTNHLGLAPPRLPPSAAPGLALAEEPGEAGDPRLL
APPSPGGAEDGGVGLGGKPRRPVYVHYEKEEESDVEISLESDSDDSVVIVPKGLLPKPPP
PPPPPPAPPSPPPAQPPEEPPAAPLPLPLPPAAGEALPGLGEENPAVININSSEEEEEEE
EFPEEDEEDEEEEEEDEEEEEEEYFDEEDEDFEEEEEEEEEFEEEEELGEEEEEEEYEEEE
GLSEEEEEEEEEEGALSEDERAPPSPQEPPAAPGEPDDLIMEVEESQGPGPLPPEEVEEE
VVVMKASPPPAPQPPPPREEEELPPAPPTPPQQASPLQAPPPAPEEMGEEPPLLEASPPI
LEEERPPKEEGAPPAGEQGDGAGEGAQPSPEREQPAVKAEAEADETETMLADFIDCPPDD
EKGPPEPSP

>Cyprinus carpio carpio [A0A9J8BB07]

MASAAWLHGPNITRLTEGLVSVLKEDRPEYLPALLANYREHGVVGTQSTGAVGGLVGISN
ARLGSSKTRFEGLCLLSVLVKDSSSEVFQQHCLSWLRTLQQVIQSQAPLPTVQLAVSVLQ
DLLQYSSQLPELAREVGLNSILGILTSLLSLKSECHLAAMKGMMTCMIYYPRACGSLREK
LGAYFLSKMDSDNPKVQEVACESFGRLPCLGGVLERGGGGRRAEGWTNQLHCLLASANSM
LGQLYQGAETEGTVQYEGPGVELPFPPLDDVDPLLILQLHHRYKAICLAIKHTLSADPAS
SVCLPVQHVLKLVCQALAVNTKSISPTGEGCLKLLVLPSIHIDTLELLSALIKAVGGGLV
QYSSVLTRLLSQSLSAWTPLPEASLGQQRAYSAVRVTVYRTIELWVRVGGASLLQGSPTH
TELLFTHLMGDITPASEAVKLRSGQQSQSMSDLIGSAGKSGPRRTKGLVMGDGISLQRKG
DVLANQDTCVAALRALRQIILTSGTLLKEDLHKRIQDLVVPLCVRLQQQSHCVLDVGAVS
GQYGSPTPRRELYRLLLALVLVPSPRWPPPLSCAVSVFSHGRRDHNIMVSSFCAEALTIC
NTLIHPRTPSISLPLTPLTLKSTPSAPVLASGQNPSLSIPTLLGGPPFPSRHPMGLGPAT
LLGSLENHLPLAPPGLPTPAGTTATPGDLLLSPAQPGELAGLGAPEGQRQVFVRYEKEEP
EDVEISLESDSDDSVVIMPAGMMMEMQDGAANAQSLSQPAVPAPIVGEVGLVDTLLPNEL
PTSIPHQILPANANNINSFPGPSQTAQLVSLVPPLNSTTASLSASPAGLADSLTGGPQLQ
QMLMQTSPGGQPPTLGLSLQIQLQNQIAQTSRQLQQQHPANEEIQPLEGDNSRGMMDEEE
AEVMIEAEDQQGMEMFCMEREREVEPGIEEMEGVRSVYADDRIKDKGTVEEIENIGAVER
NEPVVGKEQIESLVISGETEGHEEDTSVEAVEPEVKTCEQEVAKPEDPTENAGPSQQGQE
LTVEVEVQKQEPELQPEDTTNQSAPSTSKQEALQSVAVTAEEEEEVEKESGEQGEDSDAR
GTKRKMEDREEGESSEQGTEKKKVDEAMASMLADFVDCPPDDDDHGASQSQS

> Mus musculus [A0A158SIT8]

MAAAVLSGASAGSPAGAPGGPGGLSAVGSGPRLRLLLLESISGLLQPRTASPVAPVHPPI
QWAPHLPGLMCLLRLHGTAGGAQNLSALGALVNLSNAHLGSIKTRFEGLCLLSLLIGESP
TELFQQHCVSWLRSIQQVLQSQDSPSTMELAVAVLRDLLRHASQLPTLFRDISTNHLPGL
LTSLLGLRPECEQSALEGMKACVTYFPRACGSLKGKLASFFLSRLDSLNPQLQQLACECY
SRLPSLGAGFSQGLKHTENWEQELHSLLTSLHSLLGSLFEETEPAPVQSEGPGIEMLLSH
SEDGNTHVLLQLRQRFSGLARCLGLMLSSEFGAPVSVPVQEILDLICRILGISSKNINLL
GDGPLRLLLLPSLHLEALDLLSALILACGSRLLRFGALISRLLPQVLNAWSTGRDTLAPG

QERPYSTIRTKVYAILELWVQVCGASAGMLQGGASGEALLTHLLSDISPPADALKLCSTR
GSSDGGLQSGKPSAPKKLKLDMGEALAPPSQRKGDRNANSDVCAAALRGLSRTILMCGPL
IKEETHRRLHDLVLPLVMSVQQGEVLGSSPYNSSCCRLGLYRLLLALLLAPSPRCPPPLA
CALKAFSLGQWEDSLEVSSFCSEALVTCAALTHPRVPPLQSSGPACPTPAPVPPPEAPSS
FRAPAFHPPGPMPSIGAVPSTGPLPSAGPIPTVGSMASTGQVPSRPGPPATANHLGLSVP
GLVSVPPRLLPGPENHRAGSGEDPVLAPSGTPPPSIPPDETFGGRVPRPAFVHYDKEEAS
DVEISLESDSDDSVVIVPEGLPSLPPAPPSGTPPPAAPAGPPTASPPVPAKEDSEELPAT
PGPPPPPPPPPPPASGPVTLPPPQLVPEGTPGGGGPTAMEEDLTVININSSDEEEEEEEE
EEEEDEDEEEEDFEEEEEDEEEYFEEEEEEEEFEEEFEEEEGELEEEEEEEEEELDEVED
VEFGSAGEVEEGGPPPPTLPPALPPSDSPKVQPEAEPEPGLLLEVEEPGPEEVPGPETAP
TLAPEVLPSQEEGEQEVGSPAAGPPQELVEESSAPPALLEEGTEGGGDKVPPPPETPAEE
METEAEVPAPQEKEQDDTAAMLADFIDCPPDDEKPPPATEPDS

> Accipiter nisus [A0A8B9NHV7]

MALGVAVLHDLLLFSSQLPELARDIGTNHIPGLLTSLLALKPECEVSTLEGIKSCMTFYP
RACGSMRGKLAAYFLSRIDSESPQLQQLACECYALLPALGRGFSQGLRHTECWNQEVQGL
LATLHGLLGALFEGSETDPLPYEGPGVEMLLPAPQDGDTGFVLTLHNRFSGLARVLQLLL
SKEFVAPVTVPVQDVLDLICRALNITSKNINWFGDGPLKMLLLPSVHLDMLDVLSSLILA
CGARLVRWGSVLGRLFPQVLSAWSGAREAVLPGQEKPFSAVRTRLYQVLELWVEVAGAAS
GVLQGPGAPGEVLLAHLLSDITPPAEGIKLKADPKPSAPKRPKLGDGVDAPALHRKLEPA
ANSDVCQAALRALRRAILTGGPLIKEETHRRLQELVVPLLLRLPQTEVPPGSPAAAAGTP
YASPPCRAALYQLLLALVLAPAPAGAPPLHCALRAFGQGQRDPALQVSSVCTEALVVANA
LARPWVPPLHPTVVPGPPPEPPAPPFRPLPPAAFPGAPRATPANPLGPPRLPPLAEEPPG
EAPPGQPPAAPGGEEESGPAGGRPRRPVFVHYDKEEPSDVEISLESDSDDSVVIVPKRGA
PPSPPAPKSGGPPPPPPSPPLAPPEPPEEPGPPPPPPPPPPPPLPPVVPPAAPAEPPGIPG
EPDPAVININSSEEEEEEEEEEEEEEEEEFGEEEFFEEEEEEEEEEEEEEFDEEEELGEEEY
EEDEEEYEEDEGLTEEEEEEEEEEEEGGAEEGQPLPVPEEPPPPPGDTGALVMEVDEGHPP
PPAGGGGEEEEEEEDEEEEEGEGTKAPQDGGEEEGEPPPGIPEPPPPLLEPPEVPPPTLD
AAHPPPHPEPPGGAGSPPPAPPQLEEEPLPPADKEEELDETATMLADFIDCPPDDDKAPE
AAL

>Balaenoptera musculus [A0A8C0DZD1]

MAAAVLSGPSAGSAAGVPGGTGGLSAVNSGPRLRLLLLESVSGLLQPRAGSTVSPVHPPV
RSVPHLPGLMCLLRLHGTVGGAQNLSAVGALVGLSNARLGSIKTRFEGLCLLSLLVGESP
TEMFQQHCVSWLRSIQQVLQSQDPPPTMELAVAVLRDLLRYAAQLPTLFRDISMNHLPGL
LTSLLGLRPESELSALEGMKACMTYFPRACGSLKGKLASFFLSRVDALSPQLQQLACECY
SRLPSLGAGFSQGLKHTDSWEHELHSLLASLHSLLGALYEGAETAPVQYEGPGVETLLSP
SEDGDAHVLLRLWQRFSGLARCLGLMLSSEFGAPVSVPVQEILDLICRTLSVSAKNISLL
GDGPLRLLLLPSLHLEALDLLSALVLACGGRLLRFGALINRLLPQVLNAWSIGRDSLSPG
QERPYSTMRTKVYAVLELWVQVCGASAGVLQGGASGEALLTHLLSDISPPADALKLRSPR
GSPDGGLQTGKPSAPKKLKLDMGEAMAPPSHRKGDSNANSDVCAAALRGLSRTILMCGPL
IKEETHRRLHDLVLPLVMGVQQGEALGSSPYTSSRCRRELYRLLLALLLAPSPRRPPPLA
CALQAFSLGQREGSLEVSTFCSEALVTCAALTHPRVPPLQTMGPACPTPAPVPPPEAPSP
FRAPAFHPPGPMPSVGPMPSVGPMPSAGPMPSPGPVPPAGPMPSVGPMPPARPGPPATAN
HLGLSVPGLVSVSPRLLPGPENHRAGSNEDAVLAPSGTPPPTIPPDETFGGRVPRPAFVH
YDKEEASDVEISLESDSDDSVVIVPEGLPPLPPPPPSGTTPPPAAPVGPPTASPPVPAKE
EPEELPAAPGPLPPPPPPPVPGPVALPPPQLVPEGTPSGGGPPALEEDLTVININSSDEE
EEEEEEEEEEEEEEEEDFEEEEEDEEEYFEEEEEEEEEFEEEFEEEEGELEEEEEDEDEE
EEVEEVEFGPAGREVEEGGPAPPSLPPALPPAASPKVQPQPEPEPEPGLLLEVEEPGAEEGP
GAETAPTLAPEVLPSQGEVEREGGSPPAVPPPQELVEEEPSVPPTLLEEGAEGGGDKVPP
PPEASAAEEMETEVEAAALQEKEQDDTAAMLADFIDCPPDDEKPPPATEPDA

> Canis lupus familiaris [A0A8C0MNV6]

MAAAVLSGPSAGSAAGVPGGAGGLSAVGSGPRLRLLLLESVSGLLQPRAGSAVAPVHPPV
RSAPHLPGLMCLLRLHGTVGGAQNLSAVGALVGLSNARLGSIKTRFEGLCLLSLLVGESP
TEMFQQHCVSWLRSIQQVLQSQDPPPTMELAVAVLRDLLRYAAQLPTLFRDISMNHLPGL
LTSLLGLRPECELSALEGMKACMTYFPRACGSLKGKLASFFLSRVDALSPQLQQLACECY
SRLPSLGAGFSQGLKHTESWEQELHSLLASLHSLLGALYEGAETAPVQYEGPGVEVLLTP
SEDGDTHVLLRLRQRFSGLARCLGLMLSSEFGAPVSVPVQEILDVICRTLSISAKNISLL
GDGPLRLLLLPSIHLDALDLLSALILACRSRLLRFGALISRLLPQVLNAWNLGRDTLAPG
QERPYSTVRTKVYAVLELWVQVCGASAGMLQGGSSGEALLSHLLSDISPPADTLKLRSPR
GSPDGGLQTGKPSAPKKLKLDVGEAMAPPSHRKGDSNANSDVCTAALRGLSRTILMCGPL
IKEETHRRLHDLVLPLVMGVQQGEVLGSSPYTSSRCRQELYRLLLALLLAPSPRCPPPLA
CALQAFSLGQREDSLEVSSFCSEALVTCVALTHPRVPSLRSMGPACPTPAPAPPPEAPSP
FRAPPFHPPGPMPSAGPMPSVGPMPSVGPMPPAGPMPPAGPMAPTRPGPPATANHLGLSV
PGLVSVPPRLIPGPENHRAGSNEDPVLAPSGTPPPAIPPDETFGGRVPRPAFVHYDKEEA
SDVEISLESDSDDSVVIVPEGLPPLPPPPPSGTTPPPVAPAGPPVASPPVPAKEEPEELP
VAPGPLPPPPPPPVPGPVALPPPQLVPEATPGGGGPPALEEDLTVININSSDEEEEEEEE
EEEEEEEEEEEEEEEDFEEEEEEEEEYFEEEEEEEEEEFEEEFEEEEGELEEEEEEEEDEEEE
EELDELEEVAFGPAAGAAEEGGPPPPSPPPALPPAQSPKMQPEPPGETGLLLEVEEPAAE
EEPGAEAAPTLAPEVLPPQGEGPREVGSPPAVPPPQELIEEEPPAPPTLLEEGTESGGDK
VPVPQETPAAEDVEAEVEAETAALQEKEQDDTAAMLADFIDCPPDDDKPPPATEPDS

>Cyprinus carpio [A0A8C1QXV7]

MASAAWLHGPNITRLTEGLVSVLKEDRPEYLPALLANYREHGVVGTQSTGAVGGLVGISN
ARLGSSKTRFEGLCLLSVLVKDSSSEVFQQHCLSWLRTLQQVIQSQAPLPTVQLAVSVLQ
DLLQYSSQLPELAREVGLNSILGILTSLLSLKSECHLAAMKGMMTCMIYYPRACGSLREK
LGAYFLSKMDSDNPKVQEVACESFGRLPCLGGVLERGGGGRRAEGWTNQLHCLLASANSM
LGQLYQGAETEGTVQYEGPGVELPFPPLDDVDPLLILQLHHRYKAVCLAIKHTLSADPAS
SVRLPVQHVLKLVCQALAVNTKSISPTGEGCLKLLVLPSIHIDTLELLSALIKAFSSQFC

LAVRVTVYRTIELWVRVGGASLLQGSPTHTELLFTHLMGDITPASEAVKLRSGQQSQSMS
DLIGSAGKSGPRRTKGLVMGDGISLQRKGDVLANQDTCVAALRALRQIILTSGTLLKEDL
QKRIQDLVVPLCVRLQQQSHCVLDVGAVSGQYGSPTPRRELYRLLLALVLVPSPRWPPPL
SCAVSVFSHGRRDHNIMVSSFCAEALTICNTLIHPRTPSISLPLTPLTLKSTPSAPVLAS
GQNPSLSIPTLLGGPPFPSRHPMGLGPATLLGSLENHLPLAPPGLPTPAGTTATPGDLLL
SPAQPGELAGLGAPEGQRQVFVRYEKEEPEDVEISLESDSDDSVVIMPAGMMMEMQDGAA
NAQSLSQPAVPAPIVGEVGLVDTLLPNELPTSIPHQILPANANNINSFPGPSQTAQLVSL
VPPLNSTTASLSASPAGLADSLTGGPQLQQMLMQTSPGGQPPTLGLSLQIQLQNQIAQTS
RQLQQQHPANEVDQNVININSSDDEEEELEEDELGEEEEEEEGLEEEEEEEEGSDLMDEE
YYEGEEFDEYEDDEEEEESEEIQPLEGDNSRGMMDEEEAEVMIEAEDQQGMEMFCMERER
EVEPGIEEMEGVRSVYADDRIKDKGTVEEIENIGAVERNEPVVGKEQTESLVISGETEGH
EEDTSVEAVEPEVKTCEQEVAKPEDPTENAGPSQQGQELTVEVEVQKQEPELQPEDTTNQ
SAPSTSKQEALQSVAVTAEAEEEEEVEKDWGEQGEDSDARGTKRKMEDREEGESSEQGTE
KKKVDEAMASMLADFVDCPPDDDDHGASQSQS
> Cyprinus carpio [A0A8C2E6S8]
MASAAWLHGPNITRLTEGLVSVLKEDRPEYLPALLANYREHGVVGTQSTGAVGGLVGISN
ARLGSSKTRFEGLCLLSVLVKDSSSEVFQQHCLSWLRTLQQVIQSQAPLPTVQLAVSVLQ
DLLQYSSQLPELAREVGLNSILGILTSLLSLKSECHLAAMKGMMACMIYYPRACGSLREK
LGAYFLSKMDSDNPKVQEVACESFGRLPCLGGVLERGGGGRRAEGWTNQLHCLLASANSM
LGQLYQGAETEGTVQYEGPGVELPFPPLDDVDPLLILQLHHRYKAVCLAIKHTLSADPAS
SVRLPVQHVLKLVCQALAVNTKSISPTGEGCLKLLVLPSIHIDTLELLSALIKAVGGGLV
QYSSVLTRLLSQSLSAWTPLPEASLGQQRAYSAVRVTVYHTIELWVRVGGASLLQGSPTH
TELLFTHLMGDITPASEAVKLRSGQQSQSMSDLIGSAGKSGPRRTKGLVMGDGISLQRKG
DVLANQDTCVAALRALRQIILTSGTLLKEDLHKRIQDLVVPLCVRLQQQSHCVLDVGAVS
GQYGSPTPRRELYRLLLALVLVPSPRWPPPLSCAVSVFSHGRRDHNIMVSSFCAEALTIC
NTLIHPRTPSISLPLTPLTLKSTPSAPVLASGQNPSLSIPTLLGGPPFPSRHPMGLGPAT
LLGSLENHLPLAPPGLPTPAGTTATPGDLLLSPAQPGELAGLGAPEGQRQVFVRYEKEEP
EDVEISLESDSDDSVVIMPAGMMMEMQDGAANAQSLSQPAVPAPIVGEVGSVDTLLPNEL
PTSIPHQILPANANNINSFPGPSQTAQLVSLVPPLNSTTASLSASPAGPADSLTGGPQLQ
QMLMQTSPGGQPPTLGLSLHIQLQNQIAQTSRQLQQQHPANEVDQNDININSSDDEEEEL
EEDELGEEEEEEEGLEEEEEEEEGSDLMDEEYYEGEEFDEYEDEEEEEESEEIQPLEGDN
SRGMMDEEEAEVMIEAEDQQGMEMFCMEREREVEPGIEEMEGVRSVYADDRIKDKGTVEE
IENIGAVERNEPVVGKEQIESLVISGETEGHEEDTSVEAVEPEVKTCEQEVAKPEDPTEN
AGPSQQGQELTVEVEVQKQEPELQPEDTTNQSAPSTSKQEALQSVAVTAEAEEEEEEEVE
KESGEQGEDSDARGTKRKMEDREEGESSEQGTEKKKVDEAMASMLADFVDCPPDDDDHGA
SQSQS
> Cyprinus carpio [A0A8C2HU31]
MASGTWLHGPNITRLTEALVSVLKEDRPEYLPALLANYREHGVVGAQSTGAVGGLVGISN
SRLGSSKTRFEGLCLLSVLVKDSSSEVFQQNCLSWLRTLQQVIQSQAPLPTVQLAVSVLQ
DLLQYSSQLPELAREVGLNSILGILTSLLSLKSECHLVAMNGMMACMIYYPRACGSLREK
LGAYFLSKMDSDNPKVQEVACECYGRLPCLGGVLERGGGGRRAEGWTNQLHCLLASANGM
LGQLYQGAETEGTVQYEGPGVELPFPPLDDVDPLLVLQLHHRYKAICLAIKHTLSADPAS
SVRLPVQHVLNFVCRALAVNTKSISPTGEGCLRLMILPSIHNDTLELLSALIKAVGGGLV
QYSSVLTRLFSQSLSAWTPLPESSLGQQRAYSAVRVTVYRTIELWVRVGGASLLQASPSH
TELLFTHLMGDITPASEAVKLRSGQQSQSMNDLIGSAGKSGPRRTKGLGLGDGISLQRKG
DVLANQDTCVAALRALRQIILTSGTLLKEDLHKRIQDLVVPLCVRLQQQSHCVLEVGAVS
GQYGSPSPRRELYRLLLALVLVPSPRWPPPLSCAVSVFSHGRRDRNIMVSSFCAEALTIC
NTLIHPRTPSICLPLTPLTLKSTPTAPVLSSGQNPSLSIPTLLGAPEGQRQVFVRYDKEE
PEDVEISLESDSDDSVVIMPAGMIMEMQDGAANAQSLSQSAVPAIGGLQPSAPIVGEVGS
VDTLLPNELPTSIPHQILPANANNINSFPGPSQTALVSLVPPLNSSVSPAGLADSMTGGP
QLQQMLMQTSPGGQPPTLGLSLQMQLQNQIAQTSRQLQTQPPANEVDQNVININSSDDEE
EEEEELEEEDELGEEEEEEEGLEDEEEEEEGSDLIDGEYCEDELEDYDDEEDEDDEESEE
IQPLEGDSDRGMIGEEDAEVMIEAEQQGMEMFCMEREREVEPGIEEMEGVRSVYADERIK
DKGTMEEIENIGAVERNEPVVDKDQIESLVISGDAEGHEEDSRVEVVEPEVKTCGQEVAR
PEDPAEDAGLTQQGQELTVEDEVQKQEPELKPEGTTNQSAPSTSEQEVLQSVAETAEEEV
GKESSEQGEDSETRGTKRKMEDREEGESSEQGTEKKKMDDEAMASMLADFVDCPPDDDDG
GASQSQT
> Chinchilla lanigera [A0A8C2VDZ1]
MAAAVLSGPSAGSPAGVPGGAGGLSAMSSGPRLRLLLLESVSGLLQPRTGSSVAPVHPPV
RSAPHLPGLVCLLRLHGSVGGAQNLSAVGALVNLSNACLGSMKTRFEGLCLLSLLVGESP
TDMFQQHCVSWLRSIQQVLQSQDPPPTMELAVAVLKELLRYAAQLPALSRDISVNHLPGL
LTSLLGLRPECEQSALEGMKACMTHFPRACGSLKGKLASFFLSRVHSLSPQLQQLACECY
SRLPSLGAGFSQGLKHTESWEQELHSLLTSLHSLLGALYEEAETAPVQSEGPGVETLLSR
TEEGDAHDLLRLRQSFSGLARCLGLMLSSEFEAPVSVPVQEVLDLICRILSVSGKNISLL
GDGPLRLLLLPSIHLEALDLLSALILACGSRLLRFGALINRLLPQVLNAWSIGRDSLSSS
QERPYSTIRTKVYAVLELWVQVCGASAGVLQGGASGEALLTHLLSDISPPADALKLRSPR
GSPDGGLQTGKPSAPKKLKLDMGEAMAPPSQRKGDSNANSDVCAAALRGLSRTILMCGPL
IKEETHRRLHDLVLPLAMSIQQGEVLGSSPYSSSCCRRELYRLLLALLLAPSPRCPPPLS
CALQAFSLGQREDSLEVSSFCSEALVTCAALTHPRVPPLQTMGPACPTPASVPPPEAPSP
FRAPPFHPPGPMPSVGPMPSVGPMSSTGPLPAAGPMSSVGPMPSVGPVPPARPGPPATAN
HLGLSVPGLVSVPPRLLSGPENHRGGSSEDPVLAPSCTPPPTIPSDETFGGRVPRPAFVH
YDKEEASDVEISLESDSDDSVVIVPEGLPPLPPPPPSGTSPPPVAPAGPPTASPPVPAKE
EPEELPATPGPLPPPPPPPVPGPVTLPPPQLVPEGTPGGAGPPALEEDLTVININSSDEE
EEEEDEEEEEEEEEEEEEEDFEEEEEEEEEEYFEEEEEEEEEFEEEFEEEEGELEEEEEEEEE
EEEEELEEVEDLEFGSAGAEVEEESGPPPPTLPPALPPPESPKVPPEPEPEPEPGLLLEVEE
PGTGEEPGAETAPTLAPEVLPSQGEVERERESPTGGPPAQEPAEEEPSGPPALLEEETEG
GSDKVPPLLETPTEEEMETEAEATAVQEKEEDDTAAMLADFIDCPPDDEKPPPTTEPDS

>Microcebus murinus[A0A8C5XB53]
MAAAVLSGPSAGSAAGVPGGTGGLSAVSSGPRLRLLLLESVSGLLQPRTGSTVAPVHPPI
RSTPHLPGLMCLLRLHGTVGGAQNLSAVGALVSLSNARLGSIKTRFEGLCLLSLLVGESP
TEIFQQHCVSWLRSIQQVLQSQDPPPTMELAVAVLRDLLRYAAQLPALFRDISMNHLPGL
LTSLLGLRPECEQSALEGMKACMTYFPRACGSLKGKLASFFLSRVDSLVPELQQLACECY
SRLPSLGSGFSQGLKHTESWEQELHSLLASLHSLLGALYEGAETAPVQNEGPGVETLLSS
SEEGDTHVLLRLRQRFSGLARCLGLMLSSEFGAPVSVPVQEILDLICRTLSVSGKNISLL
GDGPLRLLLLPSIHLEALDLLSALILACRGRLLRFGVLISRLLPQVLNAWSIGRDSLSPG
QEKPYSTIRTKVYAVLELWVKVCGASAGVLQGGASGEALLTHLLSDISPPADALKLRSPR
GSPDGGLQTGKPSAPKKLKLDVGEAMAPPSHRKGDSNANSDVCVAALRGLSRTILMCGPL
IKEETHRRLHDLVLPLVMGVQQCEALGTSPYTSSCCRRELYRLLLALLLAPAPRCPPPLA
CALQAFSLGQQEDSLEVSSFCSEALVTCAALTHPRVPPLQSMGPTCPTPAPVPPPEAPSP
FRAAPFHPPGPMPSVGPMPSAGPMPSAGPMPSAGPVPPARPGPPATANHLGLPVPGLVSV
PPRLLPGPENHRAGSNEDPVLAPSGTPPPTIPPDETFGGRVPRPAFVHYDKEEASDVEIS
LESDSDDSVVIVPEGLPPLPPPPPSGTTPPPVAPSGPPTASPPVPAKEEPEELPAPPGPL
PPPPPPPPPPVPGPVTLPPPQLVPEGTPGGGGPPALEEDLTVININSSDEEEEEEEEEEE
EEEEEEEEEDFEEEEEDEEEYFEEEEEEEEEFEEEFEEEEGELEEEEEEEDEEEEEELEE
VEELEFGSAGGEVEEGGPPPPTLPPALPPPESPKAQPEPQPEPEPEPGLLLEVEEPGAEEEH
EAETAPTLALEVLPSQGEVEREGGSPAAGPPPQELVEEEPSAPPTLLEEGTEGGADKVPP
PPETPAEEEMETEAEATALQEKEQDDTAAMLADFIDCPPDDEKPPPTTEPDS
> Piliocolobus tephrosceles [A0A8C9IT74]
MEIIRTLAPFVRTTCTLNITGPPRTRSPSITPGTQHPGASAPSLLSPRMLLPSLSPRVQS
AFTQCAGPQPPPHAHSPVGGRRGVFASSRHATTGAREKMAAAVLSGPSAGSAAGVPGGTG
GLSAVNSGPRLRLLLLESVSGLLQPRTGSAVAPVHPPNRSAPHLPGLMCLLRLHGSVGGA
QNLSALGALVSLSNARLSSIKTRFEGLCLLSLLVGESPTELFQQHCVSWLRSIQQVLQTQ
DPPATMELAVAVLRDLLRYAAQLPALFRDISMNHLPGLLTSLLGLRPECEQSALEGMKAC
MTYFPRACGSLKGKLASFFLSRVDALSPQLQQLACECYSRLPSLGAGFSQGLKHTESWEQ
ELHSLLASLHTLLGALYEGAETAPVQNEGPGVEMLLSSEDGDAHVLLRLRQRFSGLARCL
GLMLSSEFGAPVSVPVQEILDFICRTLSVSSKNISLHGDGPLRLLLLPSIHLEALDLLSA
LILACGSRLLRFGILISRLLPQVLNSWSIGRDSLSPGQERPYSTVRTKVYAVLELWVQVC
GASAGMLQGGASGEALLTHLLSDISPPADALKLRSPRGSPDGSLQTGKPSAPKKLKLDVG
EAMAPPSHRKGDSNANSDVCAAALRGLSRTILMCGPLIKEETHRRLHDLVLPLVMGVQQG
EVLGSSPYTSSRCRRELYCLLLALLLAPSPRCPPPLACALQAFSLGQQEDSLEVSSFCSE
ALVTCAALTHPRVPPLQPMGPTCPTPAPVPPPEAPSPFRAPPFHPPGPMPSVGPMPSAGP
MPSVGPMPSAGPVPSARPGPPTTANHLGLSVSGLVSVPPRLLPGPENHRAGSNEDPILAP
SGTPPPAIPPDETFGGRVPRPAFVHYDKEEASDVEISLESDSDDSVVIVPEGLPPLPPPP
PSGATPPPIAPTGPPTASPPVPAKEEPEELPAAPGPLPPPPPPPPPVPGPVTLPPPQLVP
EGTPGGGGPPALEEDLTVININSSDEEEEEEEEEEEEEDEEEEEEDFEEEEEDEEEYFEE
EEEEEEEFEEEFEEEEGELEEEEEEEDEEEEEELEEVEELEFGTAGGEVEEGGPPPPTLP
PALPPPESPPKVQPEPEPEPGLLLEVEEPGAEEEHGADTAPTLAPEVLPSQGEVEREEGS
PSAGPPPQELVEEEPSAPPTLLEEGTEDGGDRVQPPPETPAEEEMETETEAEALQEKEQD
DTAAMLADFIDCPPDDEKPPPPTEPDS
>Piliocolobus tephrosceles [A0A8C9J1S7]
MEIIRTLAPFVRTTCTLNITGPPRTRSPSITPGTQHPGASAPSLLSPRMLLPSLSPRVQS
AFTQCAGPQPPPHAHSPVGGRRGVFASSRHATTGAREKMAAAVLSGPSAGSAAGVPGGTG
GLSAVNSGPRLRLLLLESVSGLLQPRTGSAVAPVHPPNRSAPHLPGLMCLLRLHGSVGGA
QNLSALGALVSLSNARLSSIKTRFEGLCLLSLLVGESPTELFQQHCVSWLRSIQQVLQTQ
DPPATMELAVAVLRDLLRYAAQLPALFRDISMNHLPGLLTSLLGLRPECEQSALEGMKAC
MTYFPRACGSLKGKLASFFLSRVDALSPQLQQLACECYSRLPSLGAGFSQGLKHTESWEQ
ELHSLLASLHTLLGALYEGAETAPVQNEGPGVEMLLSSEDGDAHVLLRLRQRFSGLARCL
GLMLSSEFGAPVSVPVQEILDFICRTLSVSSKNISLHGDGPLRLLLLPSIHLEALDLLSA
LILACGSRLLRFGILISRLLPQVLNSWSIGRDSLSPGQERPYSTVRTKVYAVLELWVQVC
GASAGMLQGGASGEALLTHLLSDISPPADALKLRSPRGSPDGSLQTGKPSAPKKLKLDVG
EAMAPPSHRKGDSNANSDVCAAALRGLSRTILMCGPLIKEETHRRLHDLVLPLVMGVQQG
EVLGSSPYTSSRCRRELYCLLLALLLAPSPRCPPPLACALQAFSLGQQEDSLEVSSFCSE
ALVTCAALTHPRVPPLQPMGPTCPTPAPVPPPEAPSPFRAPPFHPPGPMPSVGPMPSAGP
MPSVGPMPSAGPVPSARPGPPTTANHLGLSVSGLVSVPPRLLPGPENHRAGSNEDPILAP
SGTPPPAIPPDETFGGRVPRPAFVHYDKEEASDVEISLESDSDDSVVIVPEGLPPLPPPP
PSGATPPPIAPTGPPTASPPVPAKEEPEELPAAPGPLPPPPPPPPPVPGPVTLPPPQLVP
EGTPGGGGPPALEEDLTVININSSDEEEEEEEEEEEEEDEEEEEEDFEEEEEDEEEYFEE
EEEEEEEFEEEFEEEEGELEEEEEEEDEEEEEELEEVEELEFGTAGGEVEEGGPPPPTLP
PALPPPESPPKVQPEPEPEPGLLLEVEEPGAEEEHGADTAPTLAPEVLPSQGEVEREEGS
PSAGPPPQELVEEEPSAPPTLLEEGTEDGGDRVQPPPETPAEEEMETETEAEALQEKVRG
PGREGSLGEKVGLPGEWVAAAYLRRCLPSLNHSSVYVCKTILNLVSLCLKGAG
> Sus scrofa [A0A8D0Z9M4]
MAAAVLSGPSAGSAAGVPGGTGGLSAVVSVPRLRLLLLDSVSGLLQPRAGSTVAPVHPPA
PSAPHLPGLMCLLRLHGTVGGAPNLSAVGALVGLSNARLGSIKTRFEGLCLLSLLVGESP
TEMFQQHCVSWLRSIQQVLQSQDPPPTMELAVAVLKDLLRYAAQLPTLFRDISMNHLPGL
LTSLLGLRPECELSALEGMKACMTYFPRACGSLKGKLASFFLSRVDALSPQLQQLACECY
SRLPSLGAGFSQGLKHTESWEQELHSLLASLHGLLGALYEGAETAPMQYEGPAVEALLSP
SEDGDAHVLLRLRQRFSGLARCLGLLLSSEFGAPVSVPVQEVLDVICRTLSVSARNIVSG
VRPPPRIPRRLPCAASAWMCSCVVETRVADASPGRASWGSVESLPRGLASPRLVLPPCSA
LEDGSLVWWEAVWTRASVCTALSRLVLPAPHLGPFVPQSLLGDGPLRLLLLPSLHLEALD
LLSALILACGGRLLRFGALISRLLPQVLSAWSIGRDTLSPGQERPYSTMRTKVYAVLELW
VQVCGASAGVLQGGASGEALLTHLLSDISPPADALKLRSPRGSPDGGLQTGKPSAPKKLK
LDMGEPIAPPSHRKGDSNANSDVCAAALRGLSRTILMCGPLIKEETHRRLHELVLPLVMG
VQQGEVLGSSPYTSSRCRRELYRLLLALLLAPSPRCPPPLACALQAFSLGQREDSLEVSS

FCSEALVTCAALTHPRVPTLQSMVPTCPTPAAVPPPEAPSPFRAPAFHPPGPMPSAGPMP
SAGPMPSVGPMPPARPGPPATANHLGLSGSSLVSVPPRLLPGPENHRAGSNEEPVLAPSG
TPPPTLPSDETFGGRVPRPAFVHYDKEEASDVEISLESDSDDSVVIVPEGLPPLPPPPPS
GTTPPPVAPAGPPAASPPVPAKEEPEELPAAPGPLPPPPPTPVPGPATLPPPQLVPEGTP
GGGGAPALEEDLTVININSSDEEEEEEEEEEEEEEEEEEEEEDFEEEEEDEEEYFEEEEEEE
EEFEEEFEEEEGELEEEEEDEEEEEEEELEEVEELEFGSAGAEVEEGGPPPPSLPPALPP
AESPKVQPEPEPEPGLLLEVEEPGAEEAPGPETAPTLVPEALPAQGEAEREAGSPPTAPP
PQELVEEEPSVPPTLLEEGAEGGGDKVPPPPETSAAEEMETETESTALQEKEQDDTAAML
ADFIDCPPDDEKPPPAAEPDS

>Dicentrarchus labrax [A0A8P4FYJ9]
MATSAWLRGPSAMRLTEGLVSVLKEQRPEYLPALLANYREHGVFPTQGASAVGGLVGFSN
AKLASSKTRFEGLCLLSMLVKDSSSDLFQQHCLSWLRSLQQVIQSQAPIQTIQLAVNILK
DVLQYSSQLAELAREVGLNSILGILTSLLGLKTECELAAMEGMTACMTYYPRACGSLRDK
LGAYFLSKMDSTNKKTQEMACQCYGRLPCLGGLLDRGVGAGRAEGWTNQIHCLLASANDL
LAQIYQGSEADGTVQYEGPGVELAFPHLDQSDPLLLLQLQHRYTAVCLALKHTLRVDPAS
AVRLPVRPILNLVCRALAVSSKSINLAGDGSVRLLVLPIIHTNTLEVLSALITAVRTGMV
QYAAVLQRLFSQTLSTWMPLPEASLGQQRAYSALRVSVYRTLELWGQVAGASASILQGSP
GHSELLFSHLLGDITPGAESVKLRVGLSADVVPGGKPGPRRTKPLVIADSVGPSLQRKGD
LLANQDTCLSALRALRQIILSSGTLLKDDIHKRLHDVVLPLCVRLQQQQSSSSTSCESAG
GTSGQYSSALTRRELYRLLLALVLVPSPCWPPPMTCAVSILSNGRTDRNLKVSTFCSEAL
TICNSLLHPRTPSIALPLPPLTLKTTPTAPVLPSSQGTTPGLTLPTLLGPAPGPPFPTRH
SLGLGPTSLLGSLENHLSLVPGLQGPAPTPGDMILSPHAHHQQDPTGLGPPEGQRPVFVR
YDREEAEDVEISLASDSDDSVVIVPPGMLNFENQQDESAVANSQTMASAAPGSAPVSLPG
GAESVTMVPTTAATTTMDAVSLPNDLATSSPLLTTSTAPINSFPPSGPSVVSLVPPLNSN
ALTAPPGLGDSLSGRPQLQQMLMQPATPGQPGPMGLPLQMHQLQNQLSQQGRHLHQHQPP
PGSNEDSGVININSTDDEEEEEEDMEDDEELEEDEEGMDEEDEEEEEVSDFAEEEFYDGE
EYEDYDEEEGEELEEEEEEEDDGDIPPLEGAEDKAGEVGIEEGKMLRAVVDEGGMGGFSV
EGEAEGGIEEIQTNRTLFGEDRMKVQEVESIGVLEEAREGAVEGVGEEDESERMDDPTMP
QILCVTGGALEEREEAEEEEEEEEEGAEAGRGDGGGLEEESSLWEQGANETEPIAASEEC
ITNKNQKESGAEPTQEASVSETQPPTHQEEQLAAVQEGEIAVADPETCTGQNTKQQEETD
AEKGEKIQPKQQQTEGGGESDGEEGKGVKRKREEVQRQEEEEAGQSTEKKKRKVRLHKGL
TERSVREKVSRAVKEELGGECLKVLLKRRKLALEEQRHSGMSHRPGHC

>Salvelinus namaycush [A0A8U0PUX8]
MAATAAWMHGPANMRLAEGLVSVLKEQRPEYLPALLANYREHGVVSTQSSAAVGGLVGLS
NAKLGNSKTRFEGLCLLSVLVKDSSSDVFQQHCLSWLRSLQQVIQSQAPLPSIQLAVGVL
QDLLQYSSQLPELAREVGLNSILGILTSLLGLKSEFHLAAMEGMTACMTFYPRACGSLRD
KLGACFLSKMDSVNPEVQEVACECYGRLPCLGGVLERGGGGRRAEGWTNQLHCLLASANC
MLAQLYQGTESEGMVPYEGPGVELPYPPLDDTDPLVLLQLQHRYRGVCLALKHTLSVDPA
SAVRLPVQQVLNLVCRALAVSSKSINLTGDGSVRLLVLPSIHNHSVKVLHALITAVGSGL
VQYSSMLQRLFSQTLSAWTPLPETNLGQQRAFSAVRVSLYRTLELWVKVGGASAGVLQGS
PTHSEILLAHLLGDITPGADSVRLRAGQSTVADLVSSKPCPKSRKPGLGMGNGGGASLQR
KGDSLANQDTCMSALRALRQIILTSGTLLKEDVHKRLHDVVLPLCVRLQQQHGGDCGTGA
GGVSGQYGSALPRRELYRLLLALVLVPPPRWPPPLTCTVSILSHGRRDRSLEVSSFCTEA
LTVCNSLLHPRTPSLCLPLPPLTLKPSPAASLLTPSQASNLTLPTLLGGPFPGRHSLGLG
HTLLGSLDNHLSLVPPGLSGQGSTPGDLLLSPHQGELAGLGLSEGQRPVFIRYDKEEAED
VEISLESDSDDSVVIFPRGMLMLENQDGTSTVATLPVSSLVPGGVTLPVPGPGDDPGGDI
SLPLANDLPSTSLPHPLLPSSSAPNSINSFPPAPLASLVPPLNSTGVTQLGAPSVGLGVG
ADSLPGAQLQQMLLQGQPPVPGQPTPLGLPIQMLQNQLAQPSRALQQQQASEEDHSVINI
NSSDEEEEEDEEMEDEDELGEEEDEEGLEDEEEEEEGSDFPDEEEEFYGEEFDDYEEEEG
EEMEEEEEEEEEEEEEGEIRSLDREGRRGGMGREEGEVLREVPEDGGMGGFCVEGEMEGG
IEELGTNRRVYGEEGVKAQEVESIGVLEEEREGEEDDADGMNDPTMPQILCVTGGALEER
EELGEGHGQEVGSCEQQGADRPEAMPSSEGPAPQHKQEAEPAQEVRVGGSDQPSNQGEEP
AKQGGDSAKEEVKPSAAPSETEGEEPEREKGGEEGEEDGRGMKRKREGEEEGTGQGTEKK
KLDEEVMASMLADFVACPPDDEEKGPSASNLPS

>Salvelinus namaycush [A0A8U0QXA6]
MAVTAAWMHGSSNMRLTEGLVSVLKEQRPEYLPALLANYREHGVLSTQSTGAVGGLVGLS
NAKLGNSKTKFEGLCLLSVLVKDSSSDVFQQHCLSWLRSLQQVIQSQDPLPSVQLAVGVL
QDLLQYSSQLPELAREVGLNSVLGILTSLLGLKSECHLAAMEGMTACMTFYPRACGSLRD
KLGAYFLSKMDSVNPKVQEVACECYGRLTCLGGVVERGGGGRRAEGWTNQLHCLLASANG
ILAQLYQGSESEGTVQYEGPGVELPYHPLDDSDPLLLLQLQHRYRAACLALKHTLSVDPA
SAVRLPVQQVLNLVCRALAVSSKSINVTGDGCVRLLVLPAVHKDTLQVLHALITALGSGL
VQYSGVLQRLFSQTLSAWTPLPETSLGQQRAFSAVRVSLYRTLELWVRVGGASASVLQGS
PTHSELLLAHLLGDITPGADSVRPSSSPQLRDLVSSKPCPKSRKPGLAVSDGGGASFQRK
GDSLANQDTCLSALRALRQVILTSGSLLKEDIHKRLHDVVLPLCVCLQQQHGANWNDSGA
GGVSGQYGSAPPRRELYRLLLALVLVPAPRWPPPLTCTVSILSHGRRDRSLKVSSFCTEA
LTICNSLLHPRTPSLSFPLTLKPTPSQASNLTLPTLLGGPTPGPPFPSSLDNHLPLVLPG
LSGGQRPVFIRYNKEEAEDVEISLESDSDDSVVIVPQRMLMLESQDGTSAAATLPGSSLA
PGGVTLPVPCPGGDPGGDISLPLSNDLPSTSLPHPLLPSSTPNSTINSFPPAPLVSLVPP
LNSTGVAQLGADSLPGAQLQQMLLQGQPPAPDLPIQVHQNQLVQSSRALQQQQQSTDEDH
TVININSSDEEEEDELEEEEEEEESDFPDEEEYDDEELDDYEAEEGEEMEDEEEGEIRLLD
GEGRRGGMEGEVLRGLAEEGGIGGFRVENEGEGRIKAQEVESEGEGGIKAQEVESEGEGG
IKAQEVESEGEGGIKAQEVESIGVMEEEREGEEDANEGMNDPTMPQILCVTGGALREREE
LDEEGAEHGQKVRSWEQKGSNRPEGTSSFEDGPAAQQQQEAEPAPEVRVDGDDQPSNQEG
EISKQGGDNTGQDMRSSTAPRETKGEGPEREKEGKEGEDGRGMKRKREGEEEGTGQRTEK
KKLDEEAMASMLADFVACPPDNVENVPSASNRPS

>Phrynocephalus forsythii [A0A9Q0XFH6]

MAAAAAAAATALAAGPGVVSSSSSSSSSSSSSSSSSSSSSSGAAAAAGGPGSCGLLLSPP
PCPGGGAAAFLGGAGDRRLEALAGLLRSPPGAAPWRLLPGLVRCARQSGTREGQASPALG
GLLSISNLRLGSSKTRFEGLCLLSLLVTESSAETFSQNCLSWLLSLQHLLQSQDPPPTVE
LAVLILRDLLQYSCQLPEVAREIGTNHIPGLLTSLLALKPECQLSALEGCKACMTFYPRA
CGSLRGRLTAYFLACLDAETPQLQLLACECYTLLPSLGAGFTQGLKHKECWEHQAHSLLA
TLHSLLGTLYEGAETDPLHYEGPGMEILLPAPEDGESSFLLQLKHRFSALTKCLCKMLSD
GFVAPVTVPVQDILDLICRSLDISAKNISWFGDGPLRMLLLPSVHLDCLDLLAALLLACG
PRLVRFGGTLCRLLPQVLTTWSSSRDLFPPGQERPYSALRTRVYQVLDLWVQVAGAASGI
LQGHGTQAEALLGHLVSDISPPSDTLKMQEGRLSSEGKPSAPKKPKLSALGGLPPLHQKH
DPRANSSVCLAALQGLSRAVLLTGSLIKEKTHKRLQELVVLLLIRLGQAEPHPGSPYASA
DCRRELYRLLLALVLAPSPSCPPPLHCALRLLDLGRTDPNLQVSSFCAEALVVCNALLHP
RAPSLQIPLATPSAAPLGSSEISPAAGVGVAAATAASPFRPVPPLPFPAVRLPPPASPSA
PPSMATSLGLPLPGLASAQLPPRLTPEEPALPASPGTAEAAALAAGAKLRRSIFIHYDKE
EEEDVEISLESDSDDSVVIVPKGQLGKGVVGPVAATAGPAVAAAPTPPAPPPPPLLAPAP
ASPSPPPLPASEEPPLEAVVPLPLGGQGPGPSSSAAVALPPSPAAEAGETTLPALMEEDP
TVININSSEEEEEEEEEEEEEDFPEDEEYFDEDEEEEEEEFDEEEGEFEEDEDEEMDEEDLEE
LEEEEEEEEDFDDDEEGLTEEEEEEEEEALTEEEEEEEEGGLPPTLPHRNEEEEPPELLPGK
DEPPKLVEEEEEEEEEEEGAGLHMEVDEEAFHPAQEGQEEEEGEEEEKRTEGARPAGEALL
LKAEGPALPLLAQELQLPSGLALLPPPVQDAPSPPPASLQEEEGLPPEQLGPPDHEEETR
RRATATTTANPPREGEDAEEATVRAGDSREARETGPEEAGGHVPGVAEKQQPPADEVVEV
KEEEEATAVDETEAMLADFVDCPPDDEKCPPDPCS
>Cyprinus carpio [A0A8C1GCK7]
MASATWLHGPNITRLTEALVSVLKEDRPEYLPALLANYREHGVVGAQSTGAVGGLVGISN
SRLGSSKTRFEGLCLLSVLVKDSSSEVFQQNCLSWLRTLQQVIQSQAPLPTVQLAVSVLQ
DLLQYSSQLPELAREVGLNSILGILTSLLSLKSECHLVAMNGMMACMIYYPRACGSLREK
LGAYFLSKMDSDNPKVQEVACECYGRLPCLGGVLERGGGGRRAEGWTNQLHCLLASANGM
LGQLYQGAESEGTVQYEGPGVELPFPPLDDVDPLLVLQLHHRYKAICLAIKHTLSADPAS
SVRLPVQHVLNFVCRALAVNTKSISPTGEGCLRLMILPSIHNDTLELLSALIKAVGGGLV
QYSSVLTRLFSQSLSAWTPLPESSLGQQRAYSAVRVTVYRTIELWVRVGGASLLQASPSH
TELLFTHLMGDITPASEAVKLRSGQQSQSMNDLIGSAGKSGPRRTKGLGLGDGISLQRKG
DVLANQDTCVAALRALRQIILTSGTLLKEDLHKRIQDLVVPLCVRLQQQSHCVLEVGAIS
GQYGSPSPRRELYRLLLALVLVPSPHWPPPLSCAVSVFSHGRRDRNIMVSSFCAEALTIC
NTLIHPRTPSICLPLTPLTLKSTPTAPVLSSGQNPSLSIPTLLGGPATGPFPARHPMGLG
PASLLGSLENHLPLAPPVLPTPAGTTAIPGDLLLSPAQPGELAGLGAPEGQRQVFVRYDK
EEPEDVEISLESDSDDSVVIMPAGMIMEMQDGAANAQSLSQSAVPAIGGLQPSAPIVGEV
GSVDTLLPNELPTSIPHQILPANANNINSFPGTSQTAQLVSLVPPLNSSVSPAGLADSMT
GGPQLQQMLMQTSPGGQPPTLGLSLQMQLQNQIAQTSRQLQTQPPANEVDQNVININSSD
DEEEEEEELEEEDELGEEEEEEEEGLEDEEEEEEEGSDLIDGEYCEDELEDYDDEEDEDDEE
SEEIQPLEGDSDRGMIGEEDAEVMIEAEQQGMEMFCMEREREVEPGIEEMEGVRSVYADE
RIKDKGTMEEIENIGAVERNEPVVDKDQIESLVISGDAEGHEEDSRVEVVEPEVKTCGQE
VARPEDPVEDAGLTQQGQELTVEDEVQKQEPELKPEGTTNQSAPSTSEQEVLQSVAETAE
EEVGKESSEQGEDSETRGTKRKMEDREEGESSEQGTEKKKMDDEAMASMLADFVDCPPDD
DDGGASQSQT
>Dicentrarchus labrax [A0A8C4ELL6]
MATSAWLRGPSAMRLTEGLVSVLKEQRPEYLPALLANYREHGVFPTQGASAVGGLVGFSN
AKLASSKTRFEGLCLLSMLVKDSSSDLFQQHCLSWLRSLQQVIQSQAPIQTIQLAVNILK
DVLQYSSQLAELAREVGLNSILGILTSLLGLKTECELAAMEGMTACMTYYPRACGSLRDK
LGAYFLSKMDSTNKKTQEMACQCYGRLPCLGGLLDRGVGAGRAEGWTNQIHCLLASANDL
LAQIYQGSEADGTVQYEGPGVELAFPHLDQSDPLLLLQLQHRYTAVCLALKHTLRVDPAS
AVRLPVRPILNLVCRALAVSSKSIVTGDGSVRLLVLPIIHTNTLEVLSALITAVRTGMVQ
YAAVLQRLFSQTLSTWMPLPEASLGQQRAYSALRVSVYRTLELWGQVAGASASILQGSPG
HSELLFSHLLGDITPGAESVKLRVGLSADVVPGGKPGPRRTKPLVIADSVGPSLQRKGDL
LANQDTCLSALRALRQIILSSGTLLKDDIHKRLHDVVLPLCVRLQQQQSSSSTSCESAGG
TSGQYSSALTRRELYRLLLALVLVPSPCWPPPMTCAVSILSNGRTDRNLKVSTFCSEALT
ICNSLLHPRTPSIALPLPPLTLKTTPTAPVLPSSQGTTPGLTLPTLLGPAPGPPFPTRHS
LGLGPTSLLGSLENHLSLVPGLQGPAPTPGDMILSPHAHHQQDPTGLGPPEGQRPVFVRY
DREEAEDVEISLASDSDDSVVIVPPGMLNFENQQDESAVANSQTMASAAPGSAPVSLPGG
AESVTMVPTTAATTTMDAVSLPNDLATSSPLLTTSTAPINSFPPSGPSVVSLVPPLNSNA
LTAPPGLGDSLSGRPQLQQMLMQPATPGQPGPMGLPLQMHQLQNQLSQQGRHLHQHQPPP
GSNEDSGVININSTDDEEEEEEEDMEDDEELEEDEEGMDEEDEEEEEVSDFAEEEFYDGEE
YEDYDEEEGEELEEEEEEEEDDGDIPPLEGAEDKAGEVGIEEGKMLRAVVDEGGMGGFSVE
GEAEGGIEEIQTNRTLFGEDRMKVQEVESIGVLEEAREGAVEGVGEEDESERMDDPTMPQ
ILCVTGGALEEREEAEEEEEEEEEGAEAGRGDGGGLEEESSLWEQGANETEPILDDDAMA
SMLADFVACPPDDEDGASGSNRV
>Prolemur simus [A0A8C8YI40]
RPLPTKARGKMAAAVLSGPSAGSAAGVPGGTGGLSAVSSGPRLRLLLLESVSGLLQPRTG
PTVAPVHPPARSAPHLPGLMCLLRLHGTVGGAQNLSAVGALVSLSNARLGSIKTRFEGLC
LLSLLVGESPTEIFQQHCVSWLRSIQQVLQSQDPPPTMELAVAVLRDLLRYAAQLPALFR
DISINHLPGLLTSLLGLRPECEQSALEGMKACMTYFPRACGSLKGKLASFFLSRVDSLSP
QLQQLACECYSRLPSLGAGFSQGLKHTESWEQELHSLLASLHSLLGALYEGAETAPVQNE
GPGVETLLSSSEDDDAHVLLRLRQRFSGLARCLGLMLSSEFGAPVSVPVQEILDLICRTL
SVSGKNISLLGDGPLRLLLLPSIHFEALDLLSALILACGGRLLRFGVLISRLLPQVLNAW
SIGRDSLSAGQERPYSTIRTKVYAVLELWVKVCGASAGVLQGGASGEALLTHLLSDISPP
ADALKLRSPRGSPDGGLQTGKPSAPKKLKLDVGEAMAPPSHRKGDSNANSDVCAAALRGL
SRTILMCGPLIKEETHRRLHDLVLPLVMGVQQGEVLGSSPYTSSCCRRELYRLLLALLLA
PSPRCPPPLACALQAFCLGQQEDSLEVSSFCSEALVTCAALTHPRVPPLQSMGPTCPTPA

PVPPPEAPSPFRAPPFHPPGPMPSAGPMPSAGPMPSAGPMPSAGPVPPARPGPPATANHL
GLPVPGLVSVPPRLLPGPENHRGGSNEDPVLAPSGTPPPTIPPDETFGGRVPRPAFVHYD
KEEASDVEISLESDSDDSVVIVPEGLPPLPPPPPSGATPPPVAPAGPPTASPPLPAKEEP
EELPATPGPLPPPPPPPPPVPGPVTLPPPQLVPEGTPGGGGPPALEEDLTVININSSDEE
EEEEEEEEEEEEEEEEEEEDFEEEEEDEEEYFEEEEEEEEEEFEEEFEEEEGELEEEEEEE
DEEEEEELEESPKVQPEPEPEPGLLLEVEEPGTEEDHGAETAPTLAPEVLPSQGEVEREG
GSPAAGPPPQELIEEEASAPPTLLEQGTEGGDDKVLPPPETPTEEEMETEAEAAALQEKQ
QDDTAAMLADFIDCPPDDEKPPPTTEPDS

>Sus scrofa [A0A8D0QP29]
MAAAVLSGPSAGSAAGVPGGTGGLSAVVSVPRLRLLLLDSVSGLLQPRAGSTVAPVHPPA
PSAPHLPGLMCLLRLHGTVGGAPLSLQSSHPPLIITHLFLVRFEGLCLLSLLVGESPTEM
FQQHCVSWLRSIQQVLQSQDPPPTMELAVAVLKDLLRYAAQLPTLFRDISMNHLPGLLTS
LLGLRPECELSALEGMKACMTYFPRACGSLKGKLASFFLSRVDALSPQLQQLACECYSRL
PSLGAGFSQGLKHTESWEQELHSLLASLHGLLGALYEGAETAPMQYEGPAVEALLSPSED
GDAHVLLRLRQRFSGLARCLGLLLSSEFGAPVSVPVQEVLDVICRTLSVSARNISLLGDG
PLRLLLLPSLHLEALDLLSALILACGGRLLRFGALISRLLPQVLSAWSIGRDTLSPGQER
PYSTMRTKVYAVLELWVQVCGASAGVLQGGASGEALLTHLLSDISPPADALKLRSPRGSP
DGGLQTGKPSAPKKLKLDMGEPIAPPSHRKGDSNANSDVCAAALRGLSRTILMCGPLIKE
ETHRRLHELVLPLVMGVQQGEVLGSSPYTSSRCRRELYRLLLALLLAPSPRCPPPLACAL
QAFSLGQREDSLEVSSFCSEALVTCAALTHPRVPTLQSMVPTCPTPAAVPPPEAPSPFRA
PAFHPPGPMPSAGPMPSAGPMPSAATANHLGLSGSSLVSVPPRLLPGPENHRAGSNEEPV
LAPSGTPPPTLPSDETFGGRVPRPAFVHYDKEEASDVEISLESDSDDSVVIVPEGLPPLP
PPPPSGTTPPPVAPAGPPAASPPVPAKEEPEELPAAPGPLPPPPPTPVPGPATLPPPQLV
PEGTPGGGGAPALEEDLTVININSSDEEEEEEEEEEEEEEEEEEEEEEDFEEEEEDEEEYF
EEEEEEEEEFEEEFEEEEGELEEEEEDEEEEEEEEELEEVEELEFGSAGAEVEEGGPPPPS
LPPALPPAESPKVQPEPEPEPGLLLEVEEPGAEEAPGPETAPTLVPEALPAQGEAEREAG
SPPTAPPPQELVEEEPSVPPTLLEEGAEGGGDKVPPPPETSAAEEMETETESTALQEKEQ
DDTAAMLADFIDCPPDDEKPPPAAEPDS

>Eleutherodactylus coqui [A0A8J6B1T2]
ASSAAVSVLLSCCTSRLSSASTRTEGLSLLSLVLEESSTDVFQQHCVSWIRSVLQVIQSQ
DPPRVVSLAVFVLRSLLAHSSALPELSREISTNHIPGLLTSLLGLRRQCLMPALEGIRSC
LISYPRACGSLRGKLTAFVLSLLDEENLQIQELACQCFSLLPSLGSGFSQGVKHTENWER
QIQSVLCSLHATFQQLYQAAETDTTRYEGPGTELDLPVLEDDGPSSILQLVRRFTALGQC
VRMLLRENFPAPVRVPASDILNLVCRVVNVSAKNLSWQGEEALKMLLLPRVHKGALDILE
AAILACGPRLLAFSSVICRLFPQLLSSWASVRSAGGFPPGQEKPYSSLRGSVYLVVERWV
TMCGVSSGILQGMSHHSDILLAHLLSDITPPADSVKMSGFVQLGAKKQKVSEVGNGEFQS
HRKRENSANTELCTAALRALCCIILYGGSLIKEDTHRRLQELSIPLLIRLQQGPDQWIGP
YTNSECRKQLYRLLLSLTLSPNPRLPPPLHCAIRIFRMGLTEENMQVSMFCTEALSICRV
IIHPRVPSLQRPLPQHGPRPPMQADVPVQRTSAPPLTFPAALPLNHLPIRTPILPQPTEP
PSMPLVHSPQETFGGKTPRPIFIHYEKEEMSDVEISLESDSDDSVVIVPEGLLQKPVPNS
EPSPPSAKPPTEETPATEVPAQPVVPSPTSVAAAPPPPPPCPGPSPAQMPVAAELPPPPP
PPQQPPALAPSEGDMVININSTDEEEDDDEDEEGMYDEDEEEYYDEEEDLEGLDEDEFDD
DEGLTDEDEEEEEEEELDAEVEEEEDEGEEEEEDGLIAGDMQMAPSVESVPEAVQPIMHN
PEAEEGPPRLSPVQEDDGGDTDLLMLVESEERETPEDGAQDGLSDAEVMSSPSPPPVLTP
PPAVAREEEEDDTAVPNVIESEPAPEPEPPATEEEPMKEEEEEKMQKPQVIVEEEEDDVE
EEEDPEEEHRDAESMMADFIDCPPDEVDDKDPERLVT

>Atractosteus spatula [A0A8J7T7L0]
MAASAWVDGPTSARLAEGLLSALQEERPGHLPGLLASYREHGGVSAQSAGVVGNLIGFSN
ARLSSTKTRFQGLCLLSVLVRDSSSDVFQQHCLSWLRAVQQVIQSQAPLPSVQLAVTILQ
DLLQYSSQLPELSREIGLNSIPGILTSLLGLKQECHLGAMEGMTACMTYYPRACGSLREK
LGAYFLSKMDSDNPKIQEMACACYGRLSSLGGVFERGSRQAEGWAQHLHCLLATAHGVLG
QLYEGVETEGGVQYEGPGIELLLPPLDDTDPLLVLQLRHRYRAVTLALTHTLSVDLSSPV
RLPVQSVLNLVCRALAVTAKGISVSGDGCLKLLVLPSLHRDTLELLSTLITVAGSRLVQY
SSVLTRLFSQTLSAWTPLPEASPGQQRAYSAVRVALYRSLDLWVRVGGAAASVLQQSPTH
SELLLAHLLGDITPGADSIKLRPGRTGMADLAGHPGKAGGKRGKGLDLGEAVGAGIALQR
KSDVLANQDTCLSALRALRQIVLTSGTLLKEDTHKRLQELALPLCVRLQQCGGAAEAGGL
YASPLARRALYQLVLAMVLVPSPRWPPPLHCAVRIFSQGLNDPSVQVSSFCNEALTVCNS
LLHPRTPSIALPLPSLALKSSPAPPAVPTTATASAPQAALSLPSLLGTPAQGPPSFTARH
PIGLAQGLLGGPLDNHLPLQPPALPQSASQQQHPPAPQPHLPPPAGDLLSTPQLGDPGTL
GGPEGHRPVFVRYDKEEPEDVEISLESDSDDSVVIVPEGMLQNKQELTQTVPGSAMGPAA
VGSGTRRLGEEGGAEAGVNSPLANELPAHQILPPNSAVMNAFPSQNQTQVSGLVTPPALQ
APAGSLGESLPPAQLQQMLMQPPQPAALSLSMQMQLVQTPRLQQQQQQQQQQQGEEDLT
VININSSDEEEEEDELEDEEEEEEGLEDLEEEDEEGSEFAEEEEEEEEYYGEEEEFEEEGE
FEEEEEEGVMEGEEIEEEEEEEEEGEVLPSEEGAVMMDPRGEREGLTGVFELEREQDGEEG
DERERVDVEAYKQPLDLEVMKEGEGLEGEGRVLEEVNGDGGGREEEREAFPPQEKAESSP
SQGTVGQETQGKEAQPKVEGVEVPGQEVTQELGTAQEILPTQAEEKATSSQDQDLEPVQE
VKSSLELKGEELSQGELATKLQEDVGERQERAGQEEEALGQEVRSSAPEEIGCARERDIE
EDAEERQGKRKREEEEEDLTEQSAEKKKPAEDSMASMLADFVDCPPDDEEEPPKLDT

>Astyanax mexicanus [A0A8T2LWU7]
MATAAWLHGQSPNSARLTEGLVSALKEERPEYLPALLSNYREHGAVAAQNSGTVGGLVGI
SNARLGTSKTRFEGLCLLSMLVKDSSSEVFQQHCLSWLRSLQQIIQSQAPLPSVQLAVSV
LQDLLQYSSQLPELAREVGLNSILGILTSLLGLKSECHLAAMEGMMTCMTYYPQACGSLK
EKLGVYFLSKMDSDNPKVQEVASECYGRLPCLGGVLERGGGGRRAEGWTNQVHCLLASAN
SILCQLYQGIETEETIEYDGPGLELPFPPLDDVDPLLILQLRQRYRAVCLALKHTLSVDP
ATSVRLPIQHVLNLVCRALAVSTKSINVTGEGCLKLLVLPSIHSDSLELLSTLIKVVGGG
LVQYCSVITRLFSQSLSAWTPLPEASLGQQRAYSAVRVAIYHTLELWVRVGGASASVLQG

STSHSEVLFAHLLGDITPGSEAVKLRAGQTSLSDLVGAAGKAGPRRTKGLTIGDPGGVSL
QRKGDALANQDTCLAALRALRQIILTCGTLLKEDQHKRLQDLVVPLCVRLQQQAQCGSEV
GVASGQYGSAAPRRELYRLLLALVLVPSPRWPPPLSCAVSVFSLGRRDRNVMVSSFCTEA
LTICNTLLHPRTPSIALPLPPLTLKPIPAAPVLGPTQNPSLSLPTLLGGPAPGPSFTSRH
PLGLGPAALLGSLDNHLPLAPPVLPTTAGATGTQGDLLLSPAQPAELAGLGAPEGQRQVF
VRYDKEEPEDVEISLESDSDDSVVIMPQGMMLEMQEGVANAQNLPPPPGGALTTGAEAGT
VDTSLPNELSTPLSHQILPTDANIINSFPGPGQTDQLVSLAPPLNSTGVSLTPSPGGLGN
SLPAGPQLQQMLMQPSPGGQPAQLGLSLHSQLQSQLAQTSRQLQQPTAAEKDQNVININS
SDDEEEEYEEMEDEDELEEDEEDEEDEDDYPDDEEYQGEEDEELEEEEGEEIDEEDEEED
EEDIQALEGENRGGLMGGEEGEVMIEEQEERRINAFQMEQEAQVEAGIEEMEGVRSVYGE
EGLKDKVAVEEIENIGAVERNESEPAEAPLETHVIVEEAEEPEENASAAEPQQEARTWEQ
EGTGQEPEPAVPEASAEPQENQEVTAEVGVSEQSTEAEQEVTPNENTASTSEEVPPQQVE
EAAEKEKDAGQPVEEEKQQQQDEKVEEKKEEEEEEEKEARGTKRKIEDREGEEPEQGTEKK
KVDDEAMASMLADFVDCPPDDEDHGASQSRS

>Albula goreensis [A0A8T3DX49]
MATAVWLHGSNNMRLTEGLVSALKEERSEYLPALLANYREHGGISTQSSGAAGGLIGLSN
ARLSSSKTRFEGLCLLSILVKDSSSEVFQQHCLSWLRSLQQVIQSQAPLPSVQLAVSVLQ
DLLQYSSQLPELAREVGLNSILGILTSLLGLKSECHLAAMEGMTACMTFYPRACGSLRDK
LGAYFLSKMDSDNPKVQEVACQCYGRLPCLGGVLERAGGGRRAEGWASQLHCLLASAHSI
LDQLYQSAECVERTMQYEGPGIELPFSPLDETDPLLVLQLRHRYRAVCLALKYTLSVDPV
SPVRLPVQNVLNLVCRALAVSCKSINVSGDGSLKLLVLPSVHTDSLELLSALITAVRSGL
VQYCSVLTRLFSQTLSAWSPMPEASLGQQRAYSAVRVSLYHTMELWVRVGGASSGVLQGN
STHTELLMAHLLGDITPGADSVKLRVGQGAVSELMGHAGKAGPRRAKGLGIGDGSAISLQ
RKGDVLANQDTCLSALRALRQIILTSGTLLKEDIHKRLQDLVLPLCVRLQQQLQCGAEVG
GGSGQYGNAPPRRELYRLLLAMVLAPPPRWPPPLTCAVSIFSHGRKDRSLMVSSFCTEAL
TVCNSLLHPRTASLALPLPPLSLKHTPAAPSLTPSQNPSLSLPALLGGPTQGAPFPARHP
LSLGPAGLLGPLENHLPLPPSVLPPQAGPAPTPGDLLLSPPQPGELAALGAPEGHRPVFV
RYDKEEAEDVEISLESDSDDSVVIVPQGMLMLESQDSAGTQPIPPPPPGGAVPTVGVGTG
GDTGVVNSPLPNELPTSLPHQILPSNSNAINTFTGQSQAQLVSLVPQLNSTAAQLTAPPV
GLGDSLPGAQLQQMLMQTSPAGQPSQLGLPIQMQLQTQLAQTSRQLQQQQQQQQMASEE
DLTVININSSDEEEEEDEDMEDEDDLGEEEEEEEEEGLDDEEEEEEEEGSDFAEEDEYYGEE
DFEDYEDEEEGMIEAEEMEEEAEGLPMLEGESRRAMLGREEGEVMMGSAEDRGLGMYPVE
REGEEDTEVQGGSSEPEGGHGVYRQPVDSTEGNREEGAKEEAEGEVGVTEEGKDSNRDGE
QDDGAIQESRGDSQPQAPAGEIIEDPSELEKDSGVELGGQQVTSQEQGPSLEGEDTIAGE
IAISCQEGDAESSKGVRITEEEKGTDLEPEQPRLVQQEKAKQETDISEEVKLAGPSVESQ
EVVENKDEDDDEEDLRGTKRKREEQEEEMVEQNVEKKKMDEEAMASMLADFVDCPPDEED
NAHSPTHS

>Megalops atlanticus [A0A9D3T0H3]
MATAVWLHGSTSMRLTEGLVSVLKEERPEYLPALLANYREHGGIATQNSGAAGGLIGLTN
ARLGSSKTRFEGLCLLSVLVKDSSSEVFQQHCLSWLRSLQQVIQSQAPLPSVQLAVSVLQ
DLLQYSSQLPELAREVGLNSILGILTSLLGLKSECHLAAMEGMTACMTFYPRACGSLRDK
LGAYFVSKMDSDNPKVQEVACECYGRLPCLGGVLERGGGGRRAEGWANQLHCLLASAHSI
LGQLYQGAESEGTVQYEGPGVELPFSPLDETDPLLVVQLRHRYRAVCLALKHTLSVDPVS
PVRLPVQNVLNLVCRALAVSCKSINVSGDGCLKLLVLPSVHSDTLELLSALITAVGSGLV
QYCSVLTRLFSQTLSAWSPLPEASLGQQRTYSAVRVSLYHTLELWARVAGASAGVLQGSS
THSELLLAHLLGDITPGADSVKLRAGQTAVSDLIGHAGKAGPRRTKGLGMGEGGGVSLQR
KGDALANQDTCLSALRALRQVILTSGTLLKEDIHKRLQDLVLPLCVRLHQQQQCGTEVGG
VSGQYGSAPPRRELYRLLLAMVLAPPPRWPPPLTCAVSIFSHGRKDHSLSVSSFCAEALT
VCNSLLHPRTPSLSLPLPPLTLKHTPTAPTLTPSQNPALSLPALLGGPTQGAPFPARHPL
SLGPAGLLGPLENHLPLPPSVLPPQTGPAPTPGDLLLSPPQPGELATLGAPEGHRPVFVR
YDKEEAEDVEISLESDSDDSVVIVPQGMLMLEPQDSAGTQPIPPPPGGAVPAGGDTGAVN
SPLANELPTSLPHQILPSNTNAINTFPGQSQAQLVSLVPPLNSTAAQLTAPPVGLGDSLP
GTQLQQMLMQPSPAGQPSQLGLPMQMQLQTQLAQSGRQQQQQQQQQQQQQMASEEDLAV
ININSSDEEEEEDEEMEDDDELGEEEEEEEEEGMEDEEEEEEEGSDFLEEDEYYGEEEFEDY
EDEEQGMIEAEEMEEEEAEGLPPLEGESRRALLGREEGEVLMGSEEERGMGMFRVERERE
TDTEVERGSGELEGGHGVYRQPMESSEGTQEEGVKEEERREVGIVEEEKDKSREGEQEGA
DVQESQGDQQPHGPAGEVTGEAGELVKEAGPELGGQEVTSQEQGATQEGEVATVEETAVT
PQEGETESSPGARVMEEKNGAEQMSEQPGPMQEEKPKQEGETPEEEARSTEQCEEGGPRG
TKEDEEEGDDEDLRGMKRKREEREEEGSEQSVEKRKLDEEAMASMLADFVDCPPDEEDNA
RSPAHS

>Mauremys mutica [A0A9D4B4H1]
MAAAAAAAASGCPGRLVLEALGGARPLPGLVRGLRDSGQNLQALGGLIGATNARLGTVKT
RFEGLCLLSLLVSESPTELFQPHCVGWLRALQHLLQSQDPAPTMALGVAVLRDLLLYSCQ
LPELGRDIATNHIPGLLTSLLALKPECQLSALEGIRACMTCYPRACGSLRGKLAAYFLSR
VDAESPQLQQLACECYALVPSLGAGFTHGLKHTECWEQELHALLATLHGLLGNLYEGAET
DPLPYEGPGVELLLPPAPPDGETNFVLNLCNRFSGLAKCLELLLSSEFVAPVTVPVQDVL
DLVCRALNISSQNISWFGDGPLKMLLLPSVHLEILEVLAALILACGARLARWGSVLGRLF
PQVLGAWSSSRDSVPLGQEKPYSAVRTRLYQVLELWVQVGGAGAGVLQGPPQHSEALLAH
LLSDIAPPTDSVKLKVGRPGSEGKPSAPKKPKLSEGGDAPSLHRKQDPMANSDVCKAALQ
ALSRAILLGGSLVKEETHRRLQELVVPLLLRLAQGDVPPGGPYASPACRHQLYRLLLALL
LAPAPACPPPLHCALRAFALGQRDPSLQVSSFCAEALVTCSALARPRVPSLQLPLPGPAP
SAGPAPADLAASPVPAPPPARPPPANHLGLAPPRLPPSAAAPGLALAEEPGEAGDPRLLA
PPSPGGAEEGGRGWGANPGAPSTCTTRRMRSRTWRSPWRATPTTASSSSPRGCCPSPPRR
PPPPPRPPPRPPPTPRRSRPPPRSRSRCRPRPARRCPAWARRTRP

>Pleuronectes platessa [A0A9N7Z881]
MATSTWQHGSSAMRLTEGLVSVLKEHRPEYLPALLASYREHGVFPTQGASAVGGLVGFSN

AKLGSSKTRFEGLCLLSMLVKDSSSSLFEQHCLSWLRSLQQVIQSQAPVQTIQLAVNILK
DLLHYSSQLAELAREVGLNSILGILTSLLGLKTECEISAMEGMTACMTYYPRACGSLRDK
LGAYFLSKMDSTNKKTQVMGCQCYGRLPCLGGLLDRGMGAGRADGWTNQIHCLLASANGL
LAQIYQGSEMDGTAQYEGSGVELAFPHLDQSDPLLLLQLQNRYTALCMALKHTLRVDPAS
AVRLPVRPILNLVCRALAVSSKSINLTGDGSVRMLVLPIIHTNTLEVLSALITVVRSGMV
QYTVVLQRLFSQTLSSWAPLHEANLGQQKAYSSMRVSVYRTLELWIQVAGASASILQGSP
GHSELLFSHLLSDITPGVDSVKLRAGLSADVVPGGKPGPRRTKPLVIADAGGSSLQRKGD
LLANQDTCLSALRALRQIILTSGTLLKDDIHKRLHDVVLPLCVRLQQQQSCSNTACDSAV
GVSGQYSSALTRRELYRLLLALVLVPSPCWPPPLTCAVSILSKGLTDRNLKISTFCTEAL
TICNSLLHPRNPSIALPLPPLTLRPTAPVLSSSQGSTPGLTLPTLLGGTAPGPPFATRQT
LSLGLGSLENHLSLVPGLSGQAPTPGDMILSPHAHHLSDQPGLGPLEGQRPVFVRYEKEE
ADDVEISLASDSDDSVVIVPPGMLNMENQQEDTAVSANSQNSASAAPGGSTVTLPGGECV
TMVPTTAAPTTVDGISLSNHLPNSSPLLTTSTAPINSFPPSSTSVVSLVPPLNSSTLTVP
PGSLGDSMLGRPQLQQMLMQPTTTGQPSAMGLQLHQHQNQLGRHLHQHQPPNALSEDSAV
ININSTDEEEEEEEDIEDDEEEEEEGMEEEDEEEEVSDFAEDEFYDGEEFEEDEEYDAQE
GEELEEDEEGDGDIPPLEGAGEMGMEEGKVLQAAVDEGGIAGFSVEGDDEGGIEEIQTNR
VLFGEDRIKVQEVESIGVLEEARVGDDEESERMDDPTMPQILCVTGGPLEKREETEEKEG
GAGGVQEASLWEKGASEIEPTIPSEECTTNQNQQESWAETAQEASVSDNQPCCHQEEQPA
SAEEGETAAAKPETAIALNTKEQEETNENRDNKVETELQETKGGGGENDGEEVKGLKRKR
EEVHRAEEAGHGTEKKKLDDDAMASMLADFVACPPDDEDGASGSNCS
>Triplophysa rosa [A0A9W7X6B8]
MASAAMLHGPNITRLTEGLVSVLKEERPEHLPVLLASYREHGVVGIQSTGAVGGLVGITN
VRLGSSKTRFEGLCLLSVLVKDSSSEVFHQHCLSWLRSLQQIIQSQAPLPTVQLAVSVLQ
DLLQYSSQLPELAREVGLNSVLGILTSLLSLKYECHLAAMKGMIACMIYYPRACGSLKEK
LGAYFLSKMDSDNTKVQEVACECYGRLPCLGGVLERGGGGHRAEGWTSQMHCLLTSANGI
FRQLYQGSEAEGTVQYEGLGVELPFPPLDDLDPLLILQLHHRLKAICLALKHTLSVDPAS
SVRLPVQNVLNLVCRALAVNTKSISPTGEGCLKLLVLPSIHIDTLELLSALIKTVGAGLV
QYSSVLTRLFSQSLSAWTPLPETSLGQQRAYSAVRVAVYRTLELWVRVGGASLLQGSPSH
LELLFTHLIGDITPGAEAVKLRSGPHSAMTDLVGSAGKPGPRRTKGLGMSDGILLQKKGD
VLANQDTCVAALHSLRQIVLTSGTLLKEDLHKRLQDLVVPLCVRLQQQSHCASEAAAVSG
QYGSPTPRRELYRLLLALVLVPSPRWPPPLSCAVSAFSHGRRDHNIMVSSFCAESLTICN
TLIHPRTPSISLPIPPLTLKTTPSAPVLASGQNPSLSIPNLLAGPATAPPFPARHPMGLG
PATLLGSLENHLPLAPPVLPTSAGPSATPGDLLLSPVQPNELAGLGAPEGQRQVFVRYDK
EEPDDVEISLESDSDDSVVIMPQGMLMEMQDGATNAQTLSQPPPPGGALPPSTPSVGEVG
SGETALPNELATSIPHQILPANANNINSLPGPSQTQLVSLVPPLNSATAPLSASPAGLAD
SMTGGPQLQQMLMQTSPAGQPPALGLSLQMQLQTQMAQTSRQLQQQPAANDVDQNVININ
STDDEDEEEEEMEEEDEELGDEEDEEEGLDDEEEEEEEEEGSDFPEDEEYYEGEEFEDYED
EEEEDEEESEEIQPMERDDARGMIGQEEAEVIIEPEEQQGMGMFCMEREQEVEGGIEEME
GVRSVYADDGIKEKGTVEEIENIGAVERNESVSDQQQIETLLIGGDEEVPEEDTAVEAVE
PEVRTWEQEEARPEDETEQAGSSQQCQELPAEEIQVQAPELQPEDKTEQSTASTPEQPVL
QNVDETTENENVGGEQEDEADPRGTKRKMEDREEGDSTEQGTEKKKMDDEAMASMLADFV
DCPPDDEDHGASQSQSSHD
>Eptesicus nilssonii [A0AA40LHC2]
MAAAVLSGPSAGSAAGVPGGAGGLSAVGSGPRLRMLLLESVSGLLQPRAGSTIPPVHPPA
RSAPHLPGLMCLLRLHGTVGGAQNLSAVGALVGLSNARLSSIKTRFEGLCLLSLLVGESS
TEMFQQHCVSWLRSIQQVLQDPPATMDLAVVVLRDLLRYAAQLPTLFRDISMNHLPGLLT
SLLGLRPECEFSALEGMKACMTYFPRACGSLKGKLASFFLSRVDALSPQLQQLACECYSR
LPSLGAGFSQGLKHTESWEQELHSLLASLHSLLGALYEGAEPEDADAHNLFRLRQRFSGL
ASCLGLMLSSEFGAPVSIPVQEVLDFICRTLSISAKNISLLGDGPLRLLLLPSIHLEALD
LLSALILACGGRLLRFGALISRLLPQVLNAWSIGRDSVSLGQEKPYSAVRTKVYAVLELW
VQVCGASAGVLQAGASGEALLTHLLSDISPPADALKLRSPRGSPDGGLQTGKPSAPKKLK
LDVGEAAAPPSHRKGDSNANSDVCAAALRGLSRTILMCGPLIKEETHRRLHDLVLPLVMG
VQQGDVLGSSPYTSARCRRELYRLLLALLLAPSPRCPPPLACALQAFSLGQREDNLEVSS
FCSKHW
>Balaenoptera musculus [A0A8B8W8X4]
MDPVFAYPTPPPIGAWGKLDLNLSAVGALVGLSNARLGSIKTRFEGLCLLSLLVGESPTE
MFQQHCVSWLRSIQQVLQSQDPPPTMELAVAVLRDLLRYAAQLPTLFRDISMNHLPGLLT
SLLGLRPESELSALEGMKACMTYFPRACGSLKGKLASFFLSRVDALSPQLQQLACECYSR
LPSLGAGFSQGLKHTDSWEHELHSLLASLHSLLGALYEGAETAPVQYEGPGVETLLSPSE
DGDAHVLLRLWQRFSGLARCLGLMLSSEFGAPVSVPVQEILDLICRTLSVSAKNISLLGD
GPLRLLLLPSLHLEALDLLSALVLACGGRLLRFGALINRLLPQVLNAWSIGRDSLSPGQE
RPYSTMRTKVYAVLELWVQVCGASAGVLQGGASGEALLTHLLSDISPPADALKLRSPRGS
PDGGLQTGKPSAPKKLKLDMGEAMAPPSHRKGDSNANSDVCAAALRGLSRTILMCGPLIK
EETHRRLHDLVLPLVMGVQQGEALGSSPYTSSRCRRELYRLLLALLLAPSPRRPPPLACA
LQAFSLGQREGSLEVSTFCSEALVTCAALTHPRVPPLQTMGPACPTPAPVPPPEAPSPFR
APAFHPPGPMPSVGPMPSVGPMPSAGPMPSPGPVPPAGPMPSVGPMPPARPGPPATANHL
GLSVPGLVSVSPRLLPGPENHRAGSNEDAVLAPSGTPPPTIPPDETFGGRVPRPAFVHYD
KEEASDVEISLESDSDDSVVIVPEGLPPLPPPPPSGTTPPPAAPVGPPTASPPVPAKEEP
EELPAAPGPLPPPPPPPPVPGPVALPPPQLVPEGTPSGGGPPALEEDLTVININSSDEEEE
EEEEEEEEEEEEEDFEEEEEDEEEYFEEEEEEEEEEFEEEFEEEEGELEEEEEDEDEEEE
VEEVEFGPAGREVEEGGPAPPSLPPALPPAASPKVQPQPEPEPGLLLEVEEPGAEEGPGA
ETAPTLAPEVLPSQGEVEREGGSPPAVPPPQELVEEEPSVPPTLLEEGAEGGGDKVPPPP
EASAAEEMETEVEAAALQEKEQDDTAAMLADFIDCPPDDEKPPPATEPDA
>Astyanax mexicanus [A0A8B9KPJ1]
MATAAWLHGQSPNSARLTEGLVSALKEERPEYLPALLSNYREHGAVAAQNSGTVGGLVGI
SNARLGTSKTRFEGLCLLSMLVKDSSSEVFQQHCLSWLRSLQQIIQSQAPLPSVQLAVSV

LQDLLQYSSQLPELAREVGLNSILGILTSLLGLKSECHLAAMEGMMTCMTYYPQACGSLK
EKLGVYFLSKMDSDNPKVQEVASECYGRLPCLGGVLERGGGGRRAEGWTNQVHCLLASAN
SILCQLYQGIETGECHTPRQARRREMAMVTYFYLKILVGGGLVQYCSVITRLFSQSLSAW
TPLPEASLGQQRAYSAVRVAIYHTLELWVRVGGASASVLQGSTSHSEVLFAHLLGDITPG
SEAVKLRAGQTSLSDLVGAAGKAGPRRTKGLTIGDPGGVSLQRKGDALANQDTCLAALRA
LRQIILTCGTLLKEDQHKRLQDLVVPLCVRLQQQAQCGSEVGVASGQYGSAAPRRELYRL
LLALVLVPSPRWPPPLSCAVSVFSLGRRDRNVMVSSFCTEALTICNTLLHPRTPSIALPL
PPLTLKPIPAAPVLGPTQNPSLSLPTLLGGPAPGPSFTSRHPLGLGPAALLGSLDNHLPL
APPVLPTTAGATGTQGDLLLSPAQPAELAGLGAPEGQRQVFVRYDKEEPEDVEISLESDS
DDSVVIMPQGMMLEMQEGVANAQNLPPPPGGALTTGAEAGTVDTSLPNELSTPLSHQILP
TDANIINSFPGPGQTDQLVSLAPPLNSTGVSLTPSPGGLGNSLPAGPQLQQMLMQPSPGG
QPAQLGLSLHSQLQSQLAQTSRQLQQPTAAEKDQNVININSSDDEEEEYEEMEDEDELEE
DEEDEEDEDDYPDDEEYQGEEDEELEEEEGEEIDEEDEEEDEEDIQALEGENRGGLMGGE
EGEVMIEEQEERRINAFQMEQEAQVEAGIEEMEGVRSVYGEEGLKDKVAVEEIENIGAVE
RNESEPAEAPLETHVIVEEAEEPEENASAAEPQQEARTWEQEGTGQEPEPAVPEASAEPQ
ENQEVTAEVGVSEQSTEAEQEVTPNENTASTSEEVPPQQVEEKVEEKKEEEEEEKEARGT
KRKIEDREGEEPEQGTEKKKVDDEAMASMLADFVDCPPDDEDHGASQSRS
>Canis lupus familiaris [A0A8C0MNK9]
MAAAVLSGPSAGSAAGVPGGAGGLSAVGSGPRLRLLLLESVSGLLQPRAGSAVAPVHPPV
RSAPHLPGLMCLLRLHGTVGGAQNLSAVGALVGLSNARLGSIKTRFEGLCLLSLLVGESP
TEMFQQHCVSWLRSIQQVLQSQDPPPTMELAVAVLRDLLRYAAQLPTLFRDISMNHLPGL
LTSLLGLRPECELSALEGMKACMTYFPRACGSLKGKLASFFLSRVDALSPQLQQLACECY
SRLPSLGAGFSQGLKHTESWEQELHSLLASLHSLLGALYEGAETAPVQYEGPGVEVLLTP
SEDGDTHVLLRLRQRFSGLARCLGLMLSSEFGAPVSVPVQEILDVICRTLSISAKNISLL
GDGPLRLLLLPSIHLDALDLLSALILACRSRLLRFGALISRLLPQVLNAWNLGRDTLAPG
QERPYSTVRTKVYAVLELWVQVCGASAGMLQGGSSGEALLSHLLSDISPPADTLKLRSPR
GSPDGGLQTGKPSAPKKLKLDVGEAMAPPSHRKGDSNANSDVCTAALRGLSRTILMCGPL
IKEETHRRLHDLVLPLVMGVQQGEVLGSSPYTSSRCRQELYRLLLALLLAPSPRCPPPLA
CALQAFSLGQREDSLEVSSFCSEALVTCVALTHPRVPSLRSMGPACPTPAPAPPPEAPSP
FRAPPFHPPGPMPSAGPMPSVGPMPSVGPMPPAGPMPPAGPMAPTRPGPPATANHLGLSV
PGLVSVPPRLIPGPENHRAGSNEDPVLAPSGTPPPAIPPDETFGGRVPRPAFVHYDKEEA
SDVEISLESDSDDSVVIVPEGLPPLPPPPPSGTTPPPVAPAGPPVASPPVPAKEEPEELP
VAPGPLPPPPPPPVPGPVALPPPQLVPEATPGGGGPPALEEDLTVININSSDEEEEEEEE
EEEEEEEEEEEEEEEDFEEEEEEEEYFEEEEEEEEEFEEEFEEEEGELEEEEEEEDEEEE
EELDELEEVAFGPAAGAAEEGGPPPPSPPPALPPAQSPKMQPEPPGETGLLLEVEEPAAE
EEPGAEAAPTLAPEVLPPQGEGPREVGSPPAVPPPQELIEEEPPAPPTLLEEGTESGGDK
VPVPQETPAAEDVEAEVEAETAALQEKVGDRGRSLGGKVGWAAAAKLSPACLSGACACFQ
PYVFVSERSRMTRLPCWLTSSIVPLMTTSHHQPQSLIPSPLWRPPLPVSNKVMSWDNTCC
FCLLHKCVPGQKPCQLPGFTRYPWRSPCVCMASWGLRHPQGDPAHLGVADCAPSLCRLRP
GFPPVGCLLLLVLHVPGCLGGEDSKEGLLVPLVRVWSMCGNV
>Canis lupus familiaris [A0A8C0RWF4]
MELAVAVLRDLLRYAAQLPTLFRDISMNHLPGLLTSLLGLRPECELSALEGMKACMTYFP
RACGSLKGKLASFFLSRVDALSPQLQQLACECYSRLPSLGAGFSQGLKHTESWEQELHSL
LASLHSLLGALYEGAETAPVQYEGPGVEVLLTPSEDGDTHVLLRLRQRFSGLARCLGLML
SSEFGAPVSVPVQEILDVICRTLSISAKNISLLGDGPLRLLLLPSIHLDALDLLSALILA
CRSRLLRFGALISRLLPQVLNAWNLGRDTLAPGQERPYSTVRTKVYAVLELWVQVCGASA
GMLQGGSSGEALLSHLLSDISPPADTLKLRSPRGSPDGGLQTGKPSAPKKLKLDVGEAMA
PPSHRKGDSNANSDVCTAALRGLSRTILMCGPLIKEETHRRLHDLVLPLVMGVQQGEVLG
SSPYTSSRCRQELYRLLLALLLAPSPRCPPPLACALQAFSLGQREDSLEVSSFCSEALVT
CAALTHPRVPSLRSMGPACPTPAPAPPPEAPSPFRAPPFHPPGPMPSAGPMPSVGPMPSA
GPMPPAGPMAPTRPGPPATANHLGLSVPGLVSVPPRLIPGPENHRAGSNEDPVLAPSGTP
PPAIPPDETFGGRVPRPAFVHYDKEEASDVEISLESDSDDSVVIVPEGLPPLPPPPPSGT
TPPPVAPAGPPVASPPVPAKEEPEELPVAPGPLPPPPPPPVPGPVALPPPQLVPEATPGGG
GPPALEEDLTVININSSDEEEEEEEEEEEEEEEEEEEEEEEDFEEEEEEEEYFEEEEEEE
EEFEEEFEEEEGELEEEEEEEDEEEEEELDEGPREVGSPPAVPPPQELIEEEPPAPPTLL
EEGTESGGDKVPVPQETPAAEDVEAEVEAETAALQEKEQDDTAAMLADFIDCPPDDDKPP
PATEPDS
>Castor canadensis [A0A8C0ZPJ1] MLPASPIPRVLLHPLRAQDRSPLPPALPERRSSRCVCVIGLRHHAKPGKMAAAVLSGPSA
GSSAGVPGGTGGLSGMGSGPRLRLLLLESVSGLLQPRTGSPVAPVHPPVHWAPHLPGLMC
LLRLHGTVGGAQNLSALGTLVSLSNAHLGSIKTRFEGLCLLSLLVGESPTELFQQHCVSW
LRSIQQVLQSQDPPPTMELAVAVLKDLLRYAAQLPMLFRDISTNHLPGLLTSLLGLRPEC
EHSALEGMKACMIYFPRACGSLKGKLASFFLSRVDALSPQLQQLACECYSRLPSLGAGFS
QGLKHTENWEQELHSLLTSLHSLLGALYEGAETAPLQSEGPGVETLLSHSEDGDTHALLR
LRQRFSGLAHCLGLLLSSEFGAPVSVPVQDVLDLICRTLSVSGKNISLLGDGPLRLLLLP
SIHLEALDLLSSLILACGGRLLRFGALISRLLPQVLNAWSIGRDTLSPGQERPYSTIRTK
VYAILELWVKVCGASSGMLQGGASGEALLTHLLSDISPPADALKLRSPRVSSDAGLQTGK
PSAPKKLKLDMGEAMAPPSHRKGDSNANSDVCAAALRGLSRTILMCGGRLHDLVLPLVMG
VQQGEVLGSSPYNSSCCRRELYRLLLALLLAPSPRFPPPLACALQAFSFGQREDSLEVTA
NCTGDLCCSDPPRVPPLQTMGPTCPTPAPVPPPEAPSPFRAPSFHPPGPMPSLGPMPSPG
PIPSAGPLPSAGPMPSVGPMSATGPMPSTGPMPSVGPMPSTRPGPPATANHLGLSVPGLV
SVPPRLLPGPENHRAASNEDPVLAPNGTPPPTITPDETFGGRVPRPAFVHYDKEEASDVE
ISLESDSDDSVVIVPEGLPPLPPPPPTGTPPPPVAPTGPPTASPPMPAKEEPEELPATPG
PLPPPPPPPPVPGPVTLPPPQLVPEGNPGGGGPPALEEDLTVININSSDEEEEEEEEEEE
EEEEEEEEEEEEDFEEEEEDEEEYFEEEEEEEEEFEEEFEEEEGELEEEEEEEDEEEEEE
LEEVEDLEFGSAGGEVEEGGPPPPTLPPAVPPPESPKVQPEPEPEPGLLLEVEEPGAEDE
PTAETAPTLAPEVLPSQGEVEREGGSPTAGSPPQELVEEPSAPPTLLEEGTEGGGDKVPP

LPETSAEEMETEAQEQDDTAAMLADFIDCPPDDEKPPPTTEPDS
>Cyprinus carpio carpio [A0A8C1AS58]
MASATWLHGPNITRLTEALVSVLKEDRPEYLPALLANYREHGVVGAQSTGAVGGLVGISN
SRLGSSKTRFEGLCLLSVLVKDSSSEVFQQNCLSWLRTLQQVIQSQAPLPTVQLAVSVLQ
DLLQYSSQLPELAREVGLNSILGILTSLLSLKSECHLVAMNGMMACMIYYPRACGSLREK
LGAYFLSKMDSDNPKVQEVACECYGRLPCLGGVLERGGGGRRAEGWTNQLHCLLASANGM
LGQLYQGAESEGTVQYEGPGVELPFPPLDDVDPLLVLQLHHRYKAICLAIKHTLSADPAS
SVRLPVQHVLNFVCRALAVNTKSISPTGEGCLRLMILPSIHNDTLELLSALIKAVGGGLV
QYSSVLTRLFSQSLSAWTPLPESSLGQQRAYSAVRVTVYRTIELWVRVGGASLLQASPSH
TELLFTHLMGDITPASEAVKLRSGQQSQSMNDLIGSAGKSGPRRTKGLGLGDGISLQRKG
DVLANQDTCVAALRALRQIILTSGTLLKEDLHKRIQDLVVPLCVRLQQQSHCVLEVGAIS
GQYGSPSPRRELYRLLLALVLVPSPHWPPPLSCAVSVFSHGRRDRNIMVSSFCAEALTIC
NTLIHPRTPSICLPLTPLTLKSTPTAPVLSSGQNPSLSIPTLLGGPATGPFPARHPMGLG
PASLLGSLENHLPLAPPVLPTPAGTTAIPGDLLLSPAQPGELAGLGAPEGQRQVFVRYDK
EEPEDVEISLESDSDDSVVIMPAGMIMEMQDGAANAQSLSQSAVPAIGGLQPSAPIVGEV
GSVDTLLPNELPTSIPHQILPANANNINSFPGTSQTAQLVSLVPPLNSSVSPAGLADSMT
GGPQLQQMLMQTSPGGQPPTLGLSLQMQLQNQIAQTSRQLQTQPPANEDDEESEEIQPLE
GDSDRGMIGEEDAEVMIEAEQQGMEMFCMEREREVEPGIEEMEGVRSVYADERIKDKGTM
EEIENIGAVERNEPVVDKDQIESLVISGDAEGHEEDSRVEVVEPEVKTCGQEVARPEDPV
EDAGLTQQGQELTVEDEVQKQEPELKPEGTTNQSAPSTSEQEVLQSVAETAEEEVGKESG
EQGEDSETRGTKRKMEDREEGESSEQGTEKKKMDDEAMASMLADFVDCPPDDDDGGASQS
QT
>Cyprinus carpio [A0A8C1UXX1]
MASAAWLHGPNITRLTEGLVSVLKEDRPEYLPALLANYREHGVVGTQSTGAVGGLVGISN
ARLGCSKTRFEGLCLLSVLVKDSSSEVFQQHCLSWLRTLQQVIQSQAPLPTVQLAVSVLQ
DLLQYSSQLPELAREVGLNSILGILTSLLSLKSECHLAAMKGMMTCMIYYPRACGSLREK
LGAYFLSKMDSDNPKVQEVACESFGRLPCLGGVLERGGGGRRAEGWTNQLHCLLASANSM
LGQLYQGAETEGTVQYEGPGVELPFPPLDDVDPLLILQLHHRYKAVCLAIKHTLSADPAS
SVRLPVQHVLKLVCQALAVNTKSISPTGEGCLKLLVLPSIHIDTLELLSALIKAVGGGLV
QYSSVLTRLLSQSLSAWTPLPEASLGQQRAYSAVRVTVYRTIELWVRVGGASLLQGSPTH
TELLFTHLMGDITPASEAVKLRSGQQSQSMSDLIGSAGKSGPRRTKGLVMGDGISLQRKG
DVLANQDTCVAALRALRQIILTSGTLLKEDLHKRIQDLVVPLCVRLQQQSHCVLDVGAVS
GQYGSPTPRRELCRLLLALVLVPSPRWPPPLSCAVSVFSHGRRDHNIMVSSFCAEALTIC
NTLIHPRTPSISLPLTPLTLKSTPSAPVLASGQNPSLSIPTLLGGPPFPSRHPMGLGPAT
LLGSLENHLPLAPPGLPTPAGTTATPGDLLLSPAQPGELAGLGAPEGQRQVFVRYEKEEP
EDVEISLESDSDDSVVIMPAGMMMEMQDGAANAQSLSQPAVPAPIVGEVGSVDTLLPNEL
PTSIPHQILPANANNINSFPGPSQTAQLVSLVPPLNSTTASLSASPAGLADSLTGGPQLQ
QMLMQTSPGGQPPTLGLSLQIQLQNQIAQTSRQLQQQHPANEVDQNVININSSDDEEEEL
EEDELGEEEEEEEGLEEEEEEEEGSDLMDEEYYEGEEFDEYEDEEEEEEESEEIQPLEGDN
SRGMMDEEEAEVMIEAEDQQGMEMFCMEREREVEPGIEEMEGVRSVYADDRIKDKGTVEE
IENIGAVERNEPVVGKEQTESLVISGETEGHEEDTSVEAVEPEVKTYEQEVAKPEDPTEN
AGPSQQGQELTVEVEVQKQEPELQPEDTTNQSAPSTSKQEALQSVAVTAEAEEEEEVEKE
SGEQGEDSDARGTKRKMEDREEGESSEQGTEKKKVDEAMASMLADFVDCPPDDDDHGASQ
SQS
> Gouania willdenowi [A0A8C5EHW8]
MATSAWLHGPAAARLTEGFVSALKEQRAEYLPALLASYREHGVISTQGAGVVGGLVGFCH
TKLGSSKTRFEGLCLLAMLVKDSSSDLFQQHCISWLRSLQQVIQSQAPLQTIQLAVLILK
DLLQYSAQLAELSREVGLNSILGILTSLLGLKTECELAAMEGMAACMSHYPRACGSLRDK
LGAYFLSKMDSTNRKTQEMACQCYARLPCLGGLLDRGLGASRAEGWTNQIHGLLLTANGL
LAQIYQGAETDGAIQYQGPGIELSFPPLDQSDPLLLLQLQHRYAAVCMTFTHTLRVDPTS
AVRLPVRPILNLVCRALAVSPRSINLTGDGSMRLLVLPGIHSSTLEVLSSLITAVRSSMV
QYAAVLQRLFSQTLSAWMPLPEATVGQQRSYSAVRVAVYRTLDLWVQVAGPSACMLQGSP
SHSELLFTHLLGEMMPGADSIKLRAGLSTEVVLGGKPGPRRSKQIILVDTAGPSVQRKGD
VFANQDTCLSALRAMRHIIQTSGTLLKDDIHKRLHDLALPLCVRLQQQSSSEGAGGISGQ
YSSALARRELYRLLLSLVLVPSPCWPPPLSCVVSILSNGRRDRSIKVSSYCAEALTVCNA
ILHPRTPSIALPLPPLTLKPSTTVPVLPPTQGTAPGLTLPTLLGSPAAVSPFPSRHSLGL
GSASLLGSLENHLSLIPGIPGQAPSTADMILSPHTHQQPELGPPEGQRPVFVRYDKEEAE
DVEISLASDSDDSVVIVPPGMLNMENQTDEAAANTQNVLGAAPGDASVPLPTGESITMVQ
TTSASRADGDPLSNNLSTSSPLLTSSTNPTNSFPTMTSVVSLVPSLNSSTLTSPPVGLGD
SLPGRPQLQQMLMQASTPGEPGPMGLPLQMLQNQLSQSGRHLHSHPPPPANNEDSGVINI
NSTDDEEEDEEDLEDDEDLDEEGEGMDEEDEEDEVSDLAEDEFYDGEDYEDYDEEEGEEL
DEEEDGDIPPLEGVEDKAEEERGEEEQVLEEAENEGDLSGFGVEGDTEGGIEEIGTSRAV
FTEDRMKVQEVESIGVLEETREREEDDGEKMDDPTMPQILCVTGGALEEREEAEEEEGRR
REEELQEEASSSEQGASEEDHTVASEEQTTSPSLQESANEPALGTSTSDTQVSCHQEEEP
AAAPESGANPETPPVSTSSDQQTSSEERSKAEGEEQQEQGEEDGNEEEGKGVKRKREEEE
GGQGQEKKRVAFYFNILVLCFQERERN
> Panthera leo [A0A8C8XKY9]
MLPRSPRRAQPPPAQCAGPRPPPHAPSPPGARRGAFASSRHATGARVKMAAAVLSGPSAG
SAAGVPGGTGGLSAVGSGPRLRLLLLESVSGLLQPRAGSAVAPVHPPVRSAAHLPGLMCL
LRLHGTVGGAQNLSAVGALVGLSNARLGSVKTRFEGLCLLSLLVGESPTELFQQHCVSWL
RSIQQVLQSQDPPPTMELAVAVLRDLLRYAAQLPTLFRDISTNHLPGLLTSLLGLRPECE
LSAMEGMKACMTYFPRACGSLKGKLASFFLSRVDALSPQLQQLACECYARLPSLGAGFSQ
GLKHTESWEQELHSLLASLHGLLGALYEGADTAPVQCEGPGLDVLLAPSEDGDAHTLLRL
RHRFSGLARCLGLLLSSEFGAPVSVPVQEILDIICRTLSISAKNISLLGDGPLRLLLLPS
IHLDALDLLSALILACGSRLLRFGALISRLLPQVLNAWNLGRDALPPGQERPYSAVRTKV
YAVLDLWVQVCGASAGVLQGGASGEALLSHLLSDISPPADALKLRSPRGSPDGGLQSGKP

```
SAPKKLKLDVGEATAPPGHRKGDSNANSDVCAAALRGLSRTVLMCGPLIKEETHRRLHDL
VLPLVMGVQQGEVLGSSPYTSSRCRRELYRLLLALLLAPSPRCPPPLACALQAFSLGQRE
DSLEVSSFCSEALVTCAALTHPRVPPLQSMGPACPAPAPAPPPEAPSPFRAPPFHPPGPM
PSVGPMPSVGPMPSVGPMPPTRPGPPATANHLGLSVPGLVSVPPRLLPGPENHRAGSNDD
PVLAPSGTPPPAVPPDETFGGRVPRPAFVHYDKEEASDVEISLESDSDDSVVIVPEGLPP
LPPPPPTGTTPPPAAPAGPPTASPPAPAKEEPEELPAAPGPLPPPPPPPVPGPVALPPPQ
LVPEGPPGGGGPPALEEDLTVININSSDEEEEEEEEEEEEEEEEEEEEEDFEEEEEEEEE
YFEEEEEEEEEFEEEFEEEEGELEEEEEDEDEDEEDELEELEEVEFGPAGGPAEEGGPPP
PSPAPALPPAQPPEAPPEPGVEPGLLLEVEEPGPEDEPGAEAAPTLAPEVLPSQGEGQRE
AGSPPAGPPPQELVEEEPSAPPPLLEEGTENGGDKVPPPPETPAAEEMEAAAEAEEQDDT
AAMLADFIDCPPDDEKPPAAPEPES
> Prolemur simus [A0A8C8YLK1]
MLLPDLSKIQPPFPQCAGPQPPPHAPCPPSVRRGVFASSLHATTRARGKMAAAVLSGPSA
GSAAGVPGGTGGLSAVSSGPRLRLLLLESVSGLLQPRTGPTVAPVHPPARSAPHLPGLMC
LLRLHGTVGGAQNLSAVGALVSLSNARLGSIKTRFEGLCLLSLLVGESPTEIFQQHCVSW
LRSIQQVLQSQDPPPTMELAVAVLRDLLRYAAQLPALFRDISINHLPGLLTSLLGLRPEC
EQSALEGMKACMTYFPRACGSLKGKLASFFLSRVDSLSPQLQQLACECYSRLPSLGAGFS
QGLKHTESWEQELHSLLASLHSLLGALYEGAETAPVQNEGPGVETLLSSSEDDDAHVLLR
LRQRFSGLARCLGLMLSSEFGAPVSVPVQEILDLICRTLSVSGKNISLLGDGPLRLLLLP
SIHFEALDLLSALILACGGRLLRFGVLISRLLPQVLNAWSIGRDSLSAGQERPYSTIRTK
VYAVLELWVKVCGASAGVLQGGASGEALLTHLLSDISPPADALKLRSPRGSPDGGLQTGK
PSAPKKLKLDVGEAMAPPSHRKGDSNANSDVCAAALRGLSRTILMCGPLIKEETHRRLHD
LVLPLVMGVQQGEVLGSSPYTSSCCRRELYRLLLALLLAPSPRCPPPLACALQAFCLGQQ
EDSLEVSSFCSEALVTCAALTHPRVPPLQSMGPTCPTPAPVPPPEAPSPFRAPPFHPPGP
MPSAGPMPSAGPMPSAGPMPSAGPVPPARPGPPATANHLGLPVPGLVSVPPRLLPGPENH
RGGSNEDPVLAPSGTPPPTIPPDETFGGRVPRPAFVHYDKEEASDVEISLESDSDDSVVI
VPEGLPPLPPPPPSGATPPPVAPAGPPTASPPLPAKEEPEELPATPGPLPPPPPPPPPVP
GPVTLPPPQLVPEGTPGGGGPPALEEDLTVININSSDEEEEEEEEEEEEEEEEEEEEEDF
EEEEEDEEEYFEEEEEEEEEFEEEFEEEEGELEEEEEEEDEEEEEELEEVEELEFGSAGG
EEEEGGPPPPTLPPALPPPESPKVQPEPEPEPGLLLEVEEPGTEEDHGAETAPTLAPEVL
PSQGEVEREGGSPAAGPPPQELIEEEASAPPTLLEQGTEGGDDKVLPPPETPTEEEMETE
AEAAALQEKQQDDTAAMLADFIDCPPDDEKPPPTTEPDS
>Sus scrofa [A0A8D0Y119]
MAAAVLSGPSAGSAAGVPGGTGGLSAVVSVPRLRLLLLDSVSGLLQPRAGSTVAPVHPPA
PSAPHLPGLMCLLRLHGTVGGAPNLSAVGALVGLSNARLGSIKTRFEGLCLLSLLVGESP
TEMFQQHCVSWLRSIQQVLQSQDPPPTMELAVAVLKDLLRYAAQLPTLFRDISMNHLPGL
LTSLLGLRPECELSALEGMKACMTYFPRACGSLKGKLASFFLSRVDALSPQLQQLACECY
SRLPSLGAGFSQGLKHTESWEQELHSLLASLHGLLGALYEGAETAPMQYEGPAVEALLSP
SEDGDAHVLLRLRQRFSGLARCLGLLLSSEFGAPVSVPVQEVLDVICRTLSVSARNIVSG
VRPPPRIPRRLPCAASAWMCSCVVETRVADASPGRASWGSVESLPRGLASPRLVLPPCSA
LEDGSLVWWEAVWTRASVCTALSRLVLPAPHLGPFVPQSLLGDGPLRLLLLPSLHLEALD
LLSALILACGGRLLRFGALISRLLPQVLSAWSIGRDTLSPGQERPYSTMRTKVYAVLELW
VQVCGASAGVLQGGASGEALLTHLLSDISPPADALKLRSPRGSPDGGLQTGKPSAPKKLK
LDMGEPIAPPSHRKGDSNANSDVCAAALRGLSRTILMCGPLIKEETHRRLHELVLPLVMG
VQQGEVLGSSPYTSSRCRRELYRLLLALLLAPSPRCPPPLACALQAFSLGQREDSLEVSS
FCSEALVTCAALTHPRVPTLQSMVPTCPTPAAVPPPEAPSPFRAPAFHPPGPMPSAGPMP
SAGPMPSVGPMPPARPGPPATANHLGLSGSSLVSVPPRLLPGPENHRAGSNEEPVLAPSG
TPPPTLPSDETFGGRVPRPAFVHYDKEEASDVEISLESDSDDSVVIVPEGLPPLPPPPPS
GTTPPPVAPAGPPAASPPVPAKEEPEELPAAPGPLPPPPPTPVPGPATLPPPQLVPEGTP
GGGGAPALEEDLTVININSSDEEEEEEEEEEEEEEEEEEEEEDFEEEEEDEEEYFEEEEE
EEEEFEEEFEEEEGELEEEEEEEDEEEEEELEEVEELEFGSAGAEVEEGGPPPPSLPPAL
PPAESPKVQPEPEPEPGLLLEVEEPGAEEAPGPETAPTLVPEALPAQGEAEREAGSPPTA
PPPQELVEEEPSVPPTLLEEGAEGGGDKVPPPPETSAAEEMETETESTALQEKEQDDTAA
MLADFIDCPPDDEKPPPAAEPDS
>Theropithecus gelada [A0A8D2FLE5]
MLLPSLSPRVQPAFIQCAGPQPPPHAHSPVGGRRGVFASSRHATTGARGKMAAAVLSGPS
AGSAAGVPGGTGGLSAVNSGPRLRLLLLESVSGLLQPRTGSAVAPVHPPNRSAPHLPGLM
CLLRLHGSVGGAQNLSALGALVSLSNARLSSIKTRFEGLCLLSLLVGESPTELFQQHCVS
WLRSIQQVLQTQDPPATMELAVAVLRDLLRYAAQLPALFRDISMNHLPGLLTSLLGLRPE
CEQSALEGMKACMTYFPRACGSLKGKLASFFLSRVDALSPQLQQLACECYSRLPSLGAGF
SQGLKHTESWEQELHSLLASLHTLLGALYEGAETAPVQNEGPGVEMLLSSEDGDAHVLLR
LRQRFSGLARCLGLMLSSEFGAPVSVPVQEILDFICRTLSVSSKNISLHGDGPLRLLLLP
SIHLEALDLLSALILACGSRLLRFGILISRLLPQVLNSWSIGRDSLSPGQERPYSTVRTK
VYAVLELWVQVCGASAGMLQGGASGEALLTHLLSDISPPADALKLRSPRGSPDGSLQTGK
PSAPKKLKLDVGEAMAPPSHRKGDSNANSDVCAAALKGLSRTILMCGPLIKEETHRRLHD
LVLPLVMGVQQGEVLGSSPYTSSRCRRELYCLLLALLLAPSPRCPPPLACALQAFSLGQR
EDSLEVSSFCSEALVTCAALTHPRVPPLQPMGPTCPTPAPVPPPEAPSPFRAPPFHPPGP
MPSVGPMPSAGPMPSAGPMPSAGPVPSARPGPPTTANHLGLSVSGLVSVPPRLLPGPENH
RAGSNEDPILAPSGTPPPAIPPDETFGGRVPRPAFVHYDKEEASDVEISLESDSDDSVVI
VPEGLPPLPPPPPSGATPPPIAPTGPPTASPPVPAKEEPEELPAAPGPLPPPPPPPPPVP
GPVTLPPPQLVPEGTPGGGGPPALEEDLTVININSSDEEEEEEEEEEEEEEEEEEEEEDF
EEEEEDEEEYFEEEEEEEEEFEEEFEEEEGELEEEEEEEDEEEEEELEEVEELEFGTAGG
EVEEGGPPPPTLPPALPPPESPPKVPPEPEPEPGLLLEVEEPGAEEEHGADTAPTLAPEV
LPSQGEVEREEGSPSAGPPPQELVEEEPSAPPTLLEEGTEDGGDRVQPPPETPAEEEMET
ETEAEALQEKEQDDTAAMLADFIDCPPDDEKPPPPTEPDS
>Microtus ochrogaster [A0A8J6KVF2]
```

MAAAVLSGPSAGSPAGAPGGTGGLSTVGSGPRLRLLLLESVSGLLQPRTGSPVAPVHPPI
QWAPYLPGLMCLLRLHGTVGGAQNPSALGTLVNLSNAHLGSIKTRFEGLCLLSLLVGESP
TEFFQQHCVSWLRSIQQVLQSQDSPPTIELAVAILRDLLRYASQLPTLFRDISTNHLPGL
LTSLLGLRPECEQSALEGMKACVTYFPRACGSLKGKLASFFLSRLDALNPQLQQLACECY
SRLPSLGAGFSQGLKHTENWEQELHSLMTSLHSLLGTLYEGAETAPVQSEGPEVETLLSQ
SEDGNTHVLLQLRQRFSGLARCLGLMLSSEFGAPVSVPVQEILDLICRILSISNKHINLH
GDGPLRLLLLPSIHLEALDLLSALILACGGRLLRFGALISRLLPQVLNTWSTGRDTLVPG
QERPYSTIRTKVYAILELWVQVCGASAGMLQGGASGEALLTHLLSDISPPADALKLCNAR
GNSDGNLQSGKPSAPKKLKLDMGEALAPSSHRKGDRNANSDVCAAALRGLSRTILMCGPL
IKEETHRRLHDLVLPLVLSVQQGEVLGGPPYSSSCCRRELYRLLLALLLAPSPRCPPPLA
CALKAFSLGQLEDSLEVSSFCSEALVTCAALTHPRVPPLQSSGLTCPTPAPVPPPEAPSP
FRAPAFHPPGPMPSIGAMPSPGTISSAGPLPSAGPIPTVGSMSSAGPMPSTGPVPSRPGP
PATANHLGLSVPGLVSVPPRLLPGPENHRAGSSEDPVLAPTGTPPPSIPPDETFGGRVPR
PAFVHYDKEEASDVEISLESDSDDSVVIVPEGLPSLPPPPPSGSPPPVAPTGPPTTSPPV
PAKEDSEELPATPGPLPPPPPPPPPVSGPVTLPPPQLVPEGTPGGGPTAMEEDLTVININ
SSDEEEEEEEEEEEEDEDEEEEDFEEEEEDEEEYFEEEEEEEEEFEEEFEEEEEGELEEE
EEEEEEELEEVEDVEFGSAGGEVEEGGPPPPTLPPALPPTDSPKVQPEVEPEPGLLLEVE
EPGAEEVPEPETAPTLAPEVLPSQEEVEQEVGSPTTGPPQELVEDESSAPPTLVEEGNEG
GGDKVPTPPKTPAEEEIEADGEAAAPQEKEQDDTAAMLADFIDCPPDDEKPPPATEPDS

>Xenopus laevis [A0A974HUK2]

MAAVCVGTRGMEVTIAGILERDLSEGELAEAIRGLREHGAFRGEGLPAAMSGLLSSCNSR
LTSASSRIEGLSLLALAVEESPTDVFVQHCVSWLRSLLQIIQSQDPPRVVSLAVFVLRSL
LAHSSALPELSREISTNHIPGLLTSLLGLRRQCLVPALEGIRSCFLSYPRACGSLRGKLT
AFLLSLLDAENQQIQEVACQCYSLLPSLGSGFSQGIKHTENWERQIQSVICSLHSVFLQL
YQGSETDTARYEGSGTELEFPSVEDDGTHGVLQLARRFTALGQCMRLLLREQFPAPVRVP
VSDILSLVCRVVNVSPKNLSWHGEESLKLLLLPRVHSSILEILEATIIACGPRLLPFSAV
ICRLFPQLLLSWAAVKGITGIPSGQERPYSSLRCSVYRVLETWVTTCGISSGVLQGPMHH
SDILLANLLSDITPPTDAIKMSTFVQLGAKKQKVSEVGDDDFQSHRKRDNTANVELCAAA
LKGLCCVILHCGSVIKEDVHRRLQELSIPLLLRLQQGSDQWLGPYISSDCRKELYRLLLC
LTLTPNPKLPAPLHCAIRIFRGGTTEESLQVSRFSTEALAICRILIHPRVPSLQRPLPHL
APRPPVQSDAPTLRPPAALSTFPAMPPANHLPPRPTVPAMSTEPPIPAAVPSPPPEESFG
EKPRRAVFIHFDKEEPSDVEISLESDSDDSVVIPEGLFAKSDSKPEPSPPAVKPPTEEV
TEQVAPSAVPSSSTAAPPPPPPPPAPPVCAGPSSAPVPIAEAPPPPQQEVDTVININSSD
DEEDGEEDEEEGLYDDEDEEDYYDEEEDEDLEGLEEDDYEEDEEGITEEEEEDLEEEGED
DEEEVEDEECLMPDEMQIGSEAEEIPDGIETSSLHEGELEEGPPRLSPVQEDEAVDTGLL
MLVESEDREPSGEAPGEGLPESDLTRSPPQPPVLTPPSPPDEPPPMEESDVPPLEEPDLE
VAPVAEEPVEETEEKKPEEVTVEKPEPEPEEEEQIADADAMLADFVDCPPDDDKLPEPCT

>Anguilla anguilla [A0A9D3MNG6]

MATAVWLHGSSNMRLTEGLVSALKEERPEYLPSLLANYREHGGISTQSSGAAGGLIGLSN
ARLSSSKTRFEGLCLLSMLVKDSSSEVFQQHCLSWLRSLQQVIQSQAPLPSVQLAVSVLQ
DLLQYSSQLPELAREVGLNSILGILTSLLGLKSECHLAAMEGMTACMTFYPRACGSLRDK
LGAYFLSKMESDNPKVQEVASECYGRLPCLGGVLERGGGGRRAEGWANQLHCLLASAHNI
LSQLYQGAECERTVQYEGPGIELPFPPLDEADPLLVVQLRHRYRAVCLALKYTLSVDPVS
PVRLPVQNVLNLVCRALAVSCKSINLSGDGCLKLLVLPSVHSDTLELLSAVITTVGSGLV
PYCSVLTRLFSQTLSAWSPLPEASLGQQRAYSAVRVSLYQTLELWVRVAGASSGVLQGSP
THTELLMAHLLGDITPGADAVKLRVGQAALSELMGHAGKTGPRRGKGLGMGDGSTVSLQR
KGDALANQDTCLSALRALRQIILTSGTLLKEDIHKRLQDLVLPLCVRLQQQLQCGGEVGG
VSGQYGNAPPRRELYRLLLAMVLAPPPRWPPPLTCAVSIFSHGRKDRNLSVSSFCAEALT
VCNSLLHPRTPSLAPPLPPLTLKHTATAPTLTPSQNPSLSLPSLLGGPAQGAPFPARHPL
SLGPTGLLGPLENHLPLPPSVLPPQAGSAPTPGDLLLSTPQPGELAALGAPEGHRPVFVR
YDKEEAEDVEISLESDSDDSVVIVPQGMLMVESHDAASTQPLPPPGGAVATVGTGAAGDT
GVVSSPLPNELPASMPHQILPANSNAINTFPGQSQAQLVSVVPQLNSTASQLAPPPVGLG
DSLPSAQLQQMLMQPSPAGQPSQLGLPMQMQLQNQMAQSSRQLQQQQQQQQQQQMPSEED
LTVININSSDEEEEEDEEMEDEDDLGEEDEEEEEGLEDEEEEEEGSDFQEEDEYYGEEFD
DYEDEEEGMIEAEEMEEEPEGLPPLEGESRRALMNREEGGVLMSSADERGMGIFRLEREG
EGDAELEGVGRGLEGDHGVYRQPLDSTEGDGHEGLKEEGEREVRVTEEGKDKIEEGEHKD
VAVHESKGDSQPQGPAEEVIEEASEFEKETGVELGRLDQTSQEQGPTQEGDGATVDETAV
SHQEGEVESLQEVRMAEEKEGVELTSEQPRLEEEEKAKQDEDVPVEEVRSPRQGEEGRAS
VAKDDVEEEGEDDLRGMKRKREEHEVEGAEQSVEKKKLDEEAMASMLADFVDCPPDEDDN
AHSTTHS

>Equus asinus [A0A9L0J079]

MDCLSNHRSGGNEHSRSSCSHVCWPQQNLSAVGALVGLSNARLGSIKTRFEGLCLLSLLV
GESPTEMFQQHCVSWLRSIQQVLQSQDSPPTMELAVAVLRDLLRYAAQLPTLFRDISMNH
LPGLLTSLLGLRPECELSALEGMKACMTYFPRACGSLKGKLASFFLSRVDALSPQLQQLA
CECYSRLPSLGAGFSQGLKHTESWEQELHSLLASLHSLLGALYEGAETAPMQYEGPGVEV
LLSPSEDGDAHALLRLRQRFSGLARCLGLMLSSEFGAPVSVPVQEILDVICRTLSISAKN
ISLLGDGPLRLLLLPSIHLEALDLLSALILACGGRLLRFGALISRLLPQVLNAWSIGRDT
LSPGQERPYSTMRTKVYAVLELWVQVCGASAGVLQGGASGEALLTHLLSDISPPADALKL
RSPRGSPDGGLQSGKPSAPKKLKLDVGEAMAPPSHRKGESNANSDVCAAALRGLSRTILM
CGPLIKEETHRRLHDLVLPLVMGVQQGEVLGSSPYTSSRCRHELYRLLLALLLAPSPRCP
PPLACALQAFSLGQREDSLEVSSFCSEALVTCAALTHPRVPPLQSMGPTCPTSAPVPPPE
APSPFRAAPFHPPGPMPSAGPMPSAGPVPSAGPMPSVGPLPSVGPMPSVGPMPPARPGPP
ATANHLGLSVPGLVSVPPRLLPGPENHRAASNEDPVLAPSGTPPPAIPPDETFGGRMPRP
AFVHYDKEEASDVEISLESDSDDSVVIVPEGLPSLPPPPPSGTTPPPVAPAGPPTASPPV
PAKEEPEELPAAPGPLPPPPPPPVPGPVTLPPPQLVPEGTPGGGGPPALEEDLTVININS
SDEEEEEEEEEEEEEEEEEEEEDFEEEEEDEEEYFEEEEEEEEEFEEEFEEEEEGELEEE

EEEEDEEEEEEELEEVEELEFGSAGGEVDEAGPPPPSLPPALPPAESPKVQPEPEPEPGLL
LEVEEPGVEEERGAETAPTLAPEVLPSQGEVEQEGGSPPAGPPPQELVEEEPSAPPTLLE
EGTEGGDDKVPPLPEPPAAEEMETETEVAALQEKEQDDTAAMLADFIDCPPDEEKPPSAT
EPDS
>Synaphobranchus kaupii [A0A9Q1EGE0
MATVAWLHGSNNMRLTEGLVSALKEERPEYLPALLANYREHGGISTQSSGAAGGVIGLSN
ARLGSSKTRFEGLCLLSILVKDSSSEVFQQHCLSWLRSLQQVIQSQAPLPSVQLAVSVLQ
DLLQYSSQLPELAREVGLNSVLGILTSLLGLKSECHLAAMDGMTACMTYYPRACGSLRDK
LGAYFLSKMDSDNPKVQEVASECYGCLPCLGGGAGEGKGVGRRAEGWANQLHCLLASAHG
ILSQLYEGAECERTVQYEGPGIELPFPPLDEADPMLVVQLRHRYRGCVPGSQTHTKCGPS
VPSSCKSINLSGDGCLKLLVLPSVHSDTLELLSAAITAVGSGLVQYCSVLTRLFSQTLSA
WSPLPEGSLGQQRAYSAVRVSLYQTLELWVRVAGASSGVLQGSPTHTELLLAHLLGDITP
GADSVKLRVGQTAISELMGHAGKAGPRRAKGLGMGDGSTVSLQRKGDALANQDTCLSALR
ALRQIILTSGTLLKEDIHKRLQDLVLPLCVRLQQQLQCGSEAGGVSGQYGNAPPRRELFR
LLLAMVLAPPPRWPPPLTCAVSIFSHGRKDRSLSVSSFCAEALTVCNSLLHPRTPCLALP
LPPLALKHTATAPTLAPAQNPSLSLPSLLGGPTQGAPFFPARHSLSLGATGLLGPLENHLP
LPPSVLPPQAGPAPTPGDLLLSPPQPGELAALGAPEGHRPVFVRYDKEEAEDVEISLESD
SDDSVVIVPQGMLMLESHDAASTQPLPPPPGGAVSVVGAGTGGDTGVVSSPLPNELPASM
PHQILPSNSNAINTFPSQSQAQLVSVVPQLNSTPAQLAAPPVGLGDSLPSAQLQQMLMQP
SPAGQPSQLGLPMQMQLQSQMAQSSRQMQQQQQQQQQQMASEDDLTVININSSDEEEEED
EEMEDEDDLGEEEEEEEEEEEGLEDEEEEEEGSDFPEEDDYYGEEEFDDYEDDEEGMMEAE
EMEEEAEGPPPLEGESRRALLSRGEGEVLVSSADERGMGMFRLERVEEEDAEVEGGGSEV
EGDHDVYRQALDSTDENLEEGVKEEEVGVTEEGKDKIREGEQEDVAVQESKGDPQPQGPA
GEVMEEASELEKETGVELPRLEGTSQEQGPAQEGDGATMDEMAVSHQEGEVEPLQEVKMA
EEKKGVEQTSEQPRPGQEEKAKQEEDVPVEEVRSPRQSEEGRASVVKDDEEEESEDDLRG
MKRKREEREVEGAEHSVEKKKLDEEAMASMLADFVDCPPDEEDNAHSTTHS
>Canis lupus familiaris [A0A8C0MT20]
MMVGALGCSACPARSAVRRGLGRPDPNLSAVGALVGLSNARLGSIKTRFEGLCLLSLLVG
ESPTEMFQQHCVSWLRSIQQVLQSQDPPPTMELAVAVLRDLLRYAAQLPTLFRDISMNHL
PGLLTSLLGLRPECELSALEGMKACMTYFPRACGSLKGKLASFFLSRVDALSPQLQQLAC
ECYSRLPSLGAGFSQGLKHTESWEQELHSLLASLHSLLGALYEGAETAPVQYEGPGVEVL
LTPSEDGDTHVLLRLRQRFSGLARCLGLMLSSEFGAPVSVPVQEILDVICRTLSISAKNI
SLLGDGPLRLLLLPSIHLDALDLLSALILACRSRLLRFGALISRLLPQVLNAWNLGRDTL
APGQERPYSTVRTKVYAVLELWVQVCGASAGMLQGGSSGEALLSHLLSDISPPADTLKLR
SPRGSPDGGLQTGKPSAPKKLKLDVGEAMAPPSHRKGDSNANSDVCTAALRGLSRTILMC
GPLIKEETHRRLHDLVLPLVMGVQQGEVLGSSPYTSSRCRQELYRLLLALLLAPSPRCPP
PLACALQAFSLGQREDSLEVSSFCSEALVTCVALTHPRVPSLRSMGPACPTPAPAPPPEA
PSPFRAPPFHPPGPMPSAGPMPSVGPMPSVGPMPPAGPMPPAGPMAPTRPGPPATANHLG
LSVPGLVSVPPRLIPGPENHRAGSNEDPVLAPSGTPPPAIPPDETFGGRVPRPAFVHYDK
EEASDVEISLESDSDDSVVIVPEGLPPLPPPPPSGTTPPPVAPAGPPVASPPVPAKEEPE
ELPVAPGPLPPPPPPPVPGPVALPPPQLVPEATPGGGGPPALEEDLTVININSSDEEEEE
EEEEEEEEEEEEEEEEEDFEEEEEEEEYFEEEEEEEEEFEEEFEEEEGELEEEEEEEDE
EEEEELDELEEVAFGPAAGAAEEGGPPPPSPPPALPPAQSPKMQPEPPGETGLLLEVEEP
AAEEEPGAEAAPTLAPEVLPPQGEGPREVGSPPAVPPPQELIEEEPPAPPTLLEEGTESG
GDKVPVPQETPAAEDVEAEVEAETAALQEKVGDRGRSLGGKVGWAAAAKLSPACLSGACA
CFQPYVFVSERSRMTRLPCWLTSSIVPLMTTSHHQPQSLIPSPLWRPPLPVSNKVMSWDN
TCCFCLLHKCVPGQKPCQLPGFTRYPWRSPCVCMASWGLRHPQGDPAHLGVADCAPSLCR
LRPGFPPVGCLLLLVLHVPGCLGGEDSKEGLLVPLVRVWSMCGNV
>Cyprinus carpio [A0A8C2GFQ9]
MASGTWLHGPNITRLTEALVSVLKEDRPEYLPALLANYREHGVVGAQSTGAVGGLVGISN
SRLGSSKTRFEGLCLLSVLVKDSSSEVFQQNCLSWLRTLQQVIQSQAPLPTVQLAVSVLQ
DLLQYSSQLPELAREVGLNSILGILTSLLSLKSECHLVAMNGMMACMIYYPRACGSLREK
LGAYFLSKMDSDNPKVQEVACECYGRLPCLGGVLERGGGGRRAEGWTNQLHCLLASANGM
LGQLYQGAETEGTVQYEGPGVELPFPPLDDVDPLLVLQLHHRYKAICLAIKHTLSADPAS
SVRLPVQHVLNFVCRALAVNTKSISPTGEGCLRLMILPSIHNDTLELLSALIKAVGGGLV
QYSSVLTRLFSQSLSAWTPLPESSLGQQRAYSAVRVTVYRTIELWVRVGGASLLQASPSH
TELLFTHLMGDITPASEAVKLRSGQQSQSMNDLIGSAGKSGPRRTKGLGLGDGISLQRKG
DVLANQDTCVAALRALRQIILTSGTLLKEDLHKRIQDLVVPLCVRLQQQSHCVLEVGAVS
GQYGSPSPRRELYRLLLALVLVPSPRWPPPLSCAVSVFSHGRRDRNIMVSSFCAEALTIC
NTLIHPRTPSICLPLTPLTLKSTPTAPVLSSGQNPSLSIPTLLGGPATGPFFPARHPMGLG
PASLLGSLENHLPLAPPVLPTPAGTTAIPGDLLLSPAQPGELAGLGAPEGQRQVFVRYDK
EEPEDVEISLESDSDDSVVIMPAGMIMEMQDGAANAQSLSQSAVPAIGGLQPSAPIVGEV
GSVDTLLPNELPTSIPHQILPANANNINSFPGPSQTALVSLVPPLNSSVSPAGLADSMTG
GPQLQQMLMQTSPGGQPPTLGLSLQMQLQNQIAQTSRQLQTQPPANEVDQNVININSSDD
EEEEEEELEEEDELGEEEEEEEEGLEDEEEEEEGSDLIDGEYCEDELEDYDDEEDEDDEES
EEIQPLEGDSDRGMIGEEDAEVMIEAEQQGMEMFCMEREREVEPGIEEMEGVRSVYADER
IKDKGTMEEIENIGAVERNEPVVDKDQIESLVISGDAEGHEEDSRVEVVEPEVKTCGQEV
ARPEDPAEDAGLTQQGQELTVEDEVQKQEPELKPEGTTNQSAPSTSEQEVLQSVAETAEE
EVGKESSEQGEDSETRGTKRKMEDREEGESSEQGTEKKKMDDEAMASMLADFVDCPPDDD
DGGASQSQT
>Cricetulus griseus [A0A8C2LCM9]
MAAAVLSGPSAGSPAGAPGGTGGLSTVGSGPRLRLLLLESVSGLLQPRTGSPVAPVHPPI
HWAPHLPGLMCLLRLHGTVGGAQNLSALGALVNISNAHLGSIKTRFEGLCLLSLLVGESP
TELFQQHCVSWLRSIQQVLQSQDSPSTMELAVAILRDLLRYASQLPTLFRDISTNHLPGL
LTSLLGLRPECEQSALEGMKACVTYFPRACGSLKGKLASFFLSRLDALNPQLQQLACECY
SRLPSLGAGFSQGLKHTENWEQELHSLLASLHSLLGTLYEGTETDPVQSEGPGVEMLLSQ

SEDGNTHVLLQLRQRFSGLARCLGLMLSSEFGAPVSVPVQEILDLICRILSISSKNINLL
GDGPLRLLLLPSIHLEALDLLSALILACGGRLLRFGALIIRLLPQVLNTWSTGRDTLAPG
QERPYSTIRTKVYAILELWVQVCGSSAGMLQGGASGEALLTHLLSDISPPTDALKLCATR
GSSDGGLPSGKPSAPKKLKLDMGEALAPASHRKGDRNANSDVCAAALRGLSRTILMCGPL
IKEETHRRLHDLVLPLVMSVQQGEVLGGSPYNSSCCRRELYRLLLALLLSPSPRCPPPLA
CALKAFSLGQCEDSLEVSSFCSEALVTCSALIHPRVPPLQSSGPACPTPAPVPPPEAPSP
FRAPPFPPPGPMPSIGAMPSPGPIPSSGPLPSAGPIPTVGAISSAGPMPSTGLVPSRPGP
PATANHLGLSVPSLVSVPPRLLPGPENHRAGSSEDPVLAPSGTPPPSIPPDETFGGRVPR
PAFVHYDKEEASDVEISLESDSDDSVVIVPEGLPSLPPPPPSGSPPPVAATGPPTASPPV
PAKEDSEELPATPGPLPPPPPPPPPVSGPVTLAPPQLVPEGTPGGGGPTAMEEDLTVINI
NSSDEEEEEEEEEEEEDEDEEEEDFEEEEEDEEEYFEEEEEEEEFEEEFEEEEEGELEEE
EEEEEEEEELEEVEDVEFGSAGGEVEEGGPPPPTLPPALPPTDSPKIQPEAEPEPGLLLE
VEEPGAEEVPGPETAPTLAPEVLPSQEEVVQEGGSPPAGPPQELVEEESSAPPNLLEEGT
EGGGDKVPPPPETPAETEMETEAEAASPQEKEQDDTAAMLADFIDCPPDDEKPPPDPEPD
S
>Capra hircus [A0A8C2SD54]
MAAAVLSGPSAGSAAAVPGGAGGLSAVGSGPRLRLMLLESVSGLLQPRTGSAVAPVHPPA
RSAPHLPGLMCLLRLHGTVGGAQNLSAVGALVGLSNARLSSIKTRFEGLCLLSLLVGESP
TEMFQQHCVSWLRSIQQILQVSLLPATPHLRPIFRDISMNHLPGLLTSLLALRPECELSA
LEGMKACMTHFPRACGSLKGKLASFFLSRVDALSPQLQQLACECYSRLPSLGAGFSQGLK
HTDSWEQELRSLLASLHSLLGGLYEGAEAAPMQYESPGAETLLSPSEDADAHTLLRLRQR
FSGLARCLGLMLSSEFGAPVSVPVQDILDLICRTLSVSAKNVSLLGDGPLRLLLLPSLHL
EALDLLSALILACGARLLRFGALISRLLPQVLSAWSIGRENLGPGQERPYSTVRTKVYAV
LELWVQVCGASAGVLQGGASGEALLSHLLSDISPPADALRLRSPRGSPDAGLQTGKPSAP
KKLKLDVGEAIAPPSHRKGDSNANSDVCAAALRGLSRTILMCGPLIKEETHRRLHELVLP
LVMGVQQGEALCSSPYTSSHCRRELYRLLLALLLAPSPRCPPPLACALRREDSLEVSSFC
SEALVTCAALTHPRVPPLQSVGPTCPAPAPVPPPEAPAPFRAPAFHAPGPLPSAGPMPSA
GPMPPAGPLPTTRPGPPATANHLGLSVPGLVSVPPRLLPGPENHRAGSSEDPVLAPSGSP
PPTVPPDETFGGRVPRPAFVHYDKEEASDVEISLESDSDDSVVIGLPPPPPPPSGTTPPP
VAPAGPPAASPPVPAKDEPEELPAAPGPLPPPPPPPVPGPVTLPPPQLVPEGTPGGGGPP
ALEEDLTVININSSDEEEEEEEEEEEEEDEEEDEEEDFEEDEEEEEYFEEEEEEEEEFEEE
FEEEEGELEDEEDEEEDEELEELEEVEFGPAGGEVEGGGPAPPSLPPVLPPTESPKGPPE
PGLEPGLLLEVEEPGTEEAPGPEMAPMLAPEVLPSQGEVEREGGSPPAGPPPQELVEEEP
SGPPTLLEEGAEGGGDNDMHPDLVCVCDCSELFVCPWKEQGDTAAMLADFIDCPPDDEKP
PPVSEPDS
>Dicentrarchus labrax [A0A8C4EJI1]
MATSAWLRGPSAMRLTEGLVSVLKEQRPEYLPALLANYREHGVFPTQGASAVGGLVGFSN
AKLASSKTRFEGLCLLSMLVKDSSSDLFQQHCLSWLRSLQQVIQSQAPIQTIQLAVNILK
DVLQYSSQLAELAREVGLNSILGILTSLLGLKTECELAAMEGMTACMTYYPRACGSLRDK
LGAYFLSKMDSTNKKTQEMACQCYGRLPCLGGLLDRGVGAGRAEGWTNQIHCLLASANDL
LAQIYQGSEADGTVQYEGPGVELAFPHLDQSDPLLLLQLQHRYTAVCLALKHTLRVDPAS
AVRLPVRPILNLVCRALAVSSKSINLAGDGSVRLLVLPIIHTNTLEVLSALITAVRTGMV
QYAAVLQRLFSQTLSTWMPLPEASLGQQRAYSALRVSVYRTLELWGQVAGASASILQGSP
GHSELLFSHLLGDITPGAESVKLRVGLSADVVPGGKPGPRRTKPLVIADSVGPSLQRKGD
LLANQDTCLSALRALRQIILSSGTLLKDDIHKRLHDVVLPLCVRLQQQQSSSSTSCESAG
GTSGQYSSALTRRELYRLLLALVLVPSPCWPPPMTCAVSILSNGRTDRNLKVSTFCSEAL
TICNSLLHPRTPSIALPLPPLTLKTTPTAPVLPSSQGTTPGLTLPTLLGPAPGPPFPTRH
SLGLGPTSLLGSLENHLSLVPGLQGPAPTPGDMILSPHAHHQQDPTGLGPPEGQRPVFVR
YDREEAEDVEISLASDSDDSVVIVPPGMLNFENQQDESAVANSQTMASAAPGSAPVSLPG
GAESVTMVPTTAATTTMDAVSLPNDLATSSPLLTTSTAPINSFPPSGPSVVSLVPPLNSN
ALTAPPGLGDSLSGRPQLQQMLMQPATPGQPGPMGLPLQMHQLQNQLSQQGRHLHQHQPP
PGSNEDSGVININSTDDEEEEEEDMEDDEELEEDEEGMDEEDEEEEEVSDFAEEEFYDGE
EYEDYDEEEGEELEEEEEEEEDDGDIPPLEGAEDKAGEVGIEEGKMLRAVVDEGGMGGFSV
EGEAEGGIEEIQTNRTLFGEDRMKVQEVESIGVLEEAREGAVEGVGEEDESERMDDPTMP
QILCVTGGALEEREEAEEEEEEEEEGAEAGRGDGGGLEEESSLWEQGANETEPIAASEEC
ITNKNQKESGAEPTQEASVSETQPPTHQEEQLAAVQEGEIAVADPETCTGQNTKQQEETD
AEKGEKIQPKQQQTEGGGESDGEEGKGVKRKREEVQRQEEEEAGQSTEKKKLDDDAMASM
LADFVACPPDDEDGASGSNRV
>Mus spicilegus [A0A8C6HPB6]
MAAAVLSGASAGSPAGAPGGPGGLSAVGSGPRLRLLLLESISGLLQPRTASPVAPVHPPI
QWAPHLPGLMCLLRLHGTAGGAQNLSALGALVNLSNAHLGSIKTRFEGLCLLSLLVGESP
TELFQQHCVSWLRSIQQVLQSQDSPSTMELAVAVLRDLLRYASQLPTLFRDISTNHLPGL
LTSLLGLRPECEQSALEGMKACVTYFPRACGSLKGKLASFFLSRLDSLNPQLQQLACECY
SRLPSLGAGFSQGLKHTENWEQELHSLLTSLHSLLGSLFEETEPAPVQSEGPGIEMLLSH
SEDGNTHVLLQLRQRFSGLARCLGLMLSSEFGAPVSVPVQEILDLICRILGISSKNINLL
GDGPLRLLLLPSLHLEALDLLSALILACGSRLLRFGALISRLLPQVLNAWSTGRDTLAPG
QERPYSTIRTKVYAILELWVQVCGASAGMLQGGASGEALLTHLLSDISPPADALKLCSTR
GSSDGGLQSGKPSAPKKLKLDMGEALAPPSQRKGDRNANSDVCAAALRGLSRTILMCGPL
IKEETHRRLHDLVLPLVMSVQQGEVPGSSPYNSSCCRLGLYRLLLALLLAPSPRCPPPLA
CALKAFSLGQWEDSLEVSSFCSEALVTCAALTHPRVPPLQSSGPACPTPAPVPPPEAPSS
FRAPAFHPPGPMPSIGAVPSTGPLPSAGPIPTVGSMASTGQVPSRPGPPATANHLGLSVP
GLVSVPPRLLPGPENHRAGSGEDPVLAPSGTPPPSIPPDETFGGRVPRPAFVHYDKEEAS
DVEISLESDSDDSVVIVPEGLPSLPPAPPSGTPPPAAPAGPPTASPPMPAKEDSEELPAT
PGPPPPPPPPPPASGPVTLPPPQLVPEGTPGGGGPTAMEEDLTVININSSDEEEEEEEEEE
EEDEDEEEEDFEEEEEDEEEYFEEEEEEEEEFEEEFEEEEGELEEEEEEEEEEELEEVEDVE
FGSAGEVEEGGPPPPTLPPALPPTDSPKVQPEAEPEPGLLLEVEEPGPEEVPGPETAPTL
APEVLPSQEEGEQEVGSPAAGPPQELVEESSAPPALLEEGNEGGGDKVPPPPETPAEEME

TEAEVSAPQEKEQDDTAAMLADFIDCPPDDEKPPPATEPDS

>Scleropages formosus [A0A8C9V200]

MATAALLHGSTNMRLTEGLVSALKLERPEYLPSLLANYREHGGVSAQSSAAVSGLIGLSN
GQLSSSKTRFEGLCLLAVLVRDSSSEVFQQHCLTWLRLLQQVIQSQAPLPSIQLAIVVLQ
DLLQYSSQLPELAREVGLNSILGILTSLLGLKLECHLVAMEGMKACMTFYPRACGSLKDK
LGAYFLSKMDSDNPRVQEVACECFGRLPCLGGVLERGGGSRRAEGWASQVHCLLATAHSL
LGQMYQGAESEGAVQYEGPGMELPLPPLEEADPLLMLQLPQRYRAVCLALTQTLSMDPNS
PVSLPVQSVLNLVCRALAVSCKNINTSGDGCFKLLFLPSVHSDTLEVLSTLITVAGSRLL
QYSNVLSRLFSQTLSAWSPPPEGSPGQQRAFSAVKVCLYRTLELWVRVAGASAGILHGSP
THNELLLTHLLGDITPGPDSVKLRAGQPAVSELVGHGGKAAAAAARRAKGMGDVTGVSIQ
RKGDALANQDTCVSALRALRQIILSCGTLLKEDIHKRLQDLVLPLCVRLQQQPCGSDVGT
GSSQYGSAPPRRELYRLLLALVLTPPPRWPPPLPCAVSIFSHGRRDHSLTVASFCTEALA
ICNSLLHPRVPSIALPLPPLALKHTPAAPNLTPSQNPSLSLPTLLGGPTQASPFPARHPL
GLGPPSLLAPMENHLPLPTPVLPPQAGSTPAPGELLSPPQPGELAALGPNEGRRPLFVRY
EKEEAEDVEISLESDSDDSVVIVPPDMLMQETQEPAGTQPIHPPPGGAVPGLTVSAASET
GAVNSPIPNELPTTMALPSNSNAVTTFPAQSQTQLVSLVPPLSSGATQLAAPSVSLGDSL
PGTQLQQMLLQSSPAGQASQLGLPVQIQLQTQLAQPSRQPQPQQMTNEEDLTVININSSD
EEEDEEEEMDDDEIGEEEEEEEEDEPLDDEEEEEEEGSEFAEEYYDGEEFGDYEEEEEEEEM
IEGEDEEGMIEGEEEEEEEAEELPALETGEGRRLLLSVEEEAVGSGGEGTMEIFCGEEGGE
SQGTGRETEEQPRSYGQDSVQEDVKQADERAAGVLEEQGDKLVDVERESEALQDGKTDSQ
PHGAAGDSSQEADITQGEGTVVDGPEVTSKEQEAAQEEDAPTVDETAASLQEPMTESPED
VGKVDEKAGERPSVDQPPVELREESKEQLKQGQQMLAGQGGTVGLGEEREDDAAEENVDD
LRSMKRKREINEEGEGEGEVRPSVEKKKLDEEAMASMLADFVDCPPDEEENARSPAQSEG

>Sus scrofa [A0A8D0LSZ3]

LCSTWRCPNLSAVGALVGLSNARLGSIKTRFEGLCLLSLLVGESPTEMFQQHCVSWLRSI
QQVLQSQDPPPTMELAVAVLKDLLRYAAQLPTLFRDISMNHLPGLLTSLLGLRPECELSA
LEGMKACMTYFPRACGSLKGKLASFFLSRVDALSPQLQQLACECYSRLPSLGAGFSQGLK
HTESWEQELHSLLASLHGLLGALYEGAETAPMQYEGPAVEALLSPSEDGDAHVLLRLRQR
FSGLARCLGLLLSSEFGAPVSVPVQEVLDVICRTLSVSARNISLLGDGPLRLLLLPSLHL
EALDLLSALILACGGRLLRFGALISRLLPQVLSAWSIGRDTLSPGQERPYSTMRTKVYAV
LELWVQVCGASAGVLQGGASGEALLTHLLSDISPPADALKLRSPRGSPDGGLQTGKPSAP
KKLKLDMGEPIAPPSHRKGDSNANSDVCAAALRGLSRTILMCGPLIKEETHRRLHELVLP
LVMGVQQGEVLGSSPYTSSRCRRELYRLLLALLLAPSPRCPPPLACALQAFSLGQREDSL
EVSSFCSEALVTCAALTHPRVPTLQSMVPTCPTPAAVPPPEAPSPFRAPAFHPPGPMPSA
GPMPSAGPMPSVATANHLGLSGSSLVSVPPRLLPGPENHRAGSNEEPVLAPSGTPPPTLP
SDETFGGRVPRPAFVHYDKEEASDGLPPLPPPPPSGTTPPPVAPAGPPAASPPVPAKEEP
EELPAAPGPLPPPPPTPVPGPATLPPPQLVPEGTPGGGGAPALEEDLTVININSSDEEEE
EEEEEEEEEEEEEEEEDFEEEEEDEEEYFEEEEEEEEEFEEEFEEEEGELEEEEEDEEE
EEEEELEEVEELEFGSAGAEVEEGGPPPPSLPPALPPAESPKVQPEPEPEPEPGLLLEVEEP
GAEEAPGPETAPTLVPEALPAQGEAEREAGSPPTAPPPQELVEEEPSVPPTLLEEGAEGG
GDKVPPPPETSAAEEMETETESTALQEKEQDDTAAMLADFIDCPPDDEKPPPAAEPDS

>Sus scrofa [A0A8D0Z6G8]

MAAAVLSGPSAGSAAGVPGGTGGLSAVVSVPRLRLLLLDSVSGLLQPRAGSTVAPVHPPA
PSAPHLPGLMCLLRLHGTVGGAPLSLQSSHPPLIITHLFLVRFEGLCLLSLLVGESPTEM
FQQHCVSWLRSIQQVLQSQDPPPTMELAVAVLKDLLRYAAQLPTLFRDISMNHLPGLLTS
LLGLRPECELSALEGMKACMTYFPRACGSLKGKLASFFLSRVDALSPQLQQLACECYSRL
PSLGAGFSQGLKHTESWEQELHSLLASLHGLLGALYEGAETAPMQYEGPAVEALLSPSED
GDAHVLLRLRQRFSGLARCLGLLLSSEFGAPVSVPVQEVLDVICRTLSVSARNISLLGDG
PLRLLLLPSLHLEALDLLSALILACGGRLLRFGALISRLLPQVLSAWSIGRDTLSPGQER
PYSTMRTKVYAVLELWVQVCGASAGVLQGGASGEALLTHLLSDISPPADALKLRSPRGSP
DGGLQTGKPSAPKKLKLDMGEPIAPPSHRKGDSNANSDVCAAALRGLSRTILMCGPLIKE
ETHRRLHELVLPLVMGVQQGEVLGSSPYTSSRCRRELYRLLLALLLAPSPRCPPPLACAL
QAFSLGQREDSLEVSSFCSEALVTCAALTHPRVPTLQSMVPTCPTPAAVPPPEAPSPFRA
PAFHPPGPMPSAGPMPSAGPMPSVGPMPPARPGPPATANHLGLSGSSLVSVPPRLLPGPE
NHRAGSNEEPVLAPSGTPPPTLPSDETFGGRVPRPAFVHYDKEEASDVEISLESDSDDSV
VIVPEGLPPLPPPPPSGTTPPPVAPAGPPAASPPVPAKEEPEELPAAPGPLPPPPPTPVP
GPATLPPPQLVPEGTPGGGGAPALEEDLTVININSSDEEEEEEEEEEEEEEEEEEEEDFEE
EEEDEEEYFEEEEEEEEEFEEEFEEEEGELEEEEEDEEEEEEEEELEEVEELEFGSAGAEV
EEGGPPPPSLPPALPPAESPKVQPEPEPEPEPGLLLEVEEPGAEEAPGPETAPTLVPEALPA
QGEAEREAGSPPTAPPPQELVEEEPSVPPTLLEEGAEGGGDKVPPPPETSAAEEMETETE
STALQEKEQDDTAAMLADFIDCPPDDEKPPPAAEPDS

>Clarias magur [A0A8J4XFP7]

MATAAWLHGPKNMRLTEGLVSALKEERPEYLPALLANYREHGVVGTQNSGTVGGLVGISN
SRLNSSKTRFEGLCLLSMLVKDSSSEVFQQHCLSWLRSLQQIIQSQAPLPSVQLAVSVLQ
DVLQYSSQLPELAREVGLNSILGILTSLLGLKSECHLAAMEGMMACMTYYPRACGSLKEK
LGVYFLSKMDSDNPKVQDVACECYGRLPCLGGMLERGGGGRRAEGWTSQVHCLLASANSI
LGHLYQGMETEETIQYEGPGVELPFPPLDDVDPLLILHLRHRYRAVCLALKHTLSIDPAT
SVRLPIQHLLNIVCRALAVNIKNINVTSQECLKLLVLPSIHTDTLEVLSALIKAVGAGLV
QYCNVLSRLFSQALCAWTPLPEASLGQQRAYSAVRVALYRTLELWVRVGRPSSSVLQGSC
SHSELLFANLIGDITPGTEAVKLRAGQTGMSDLGAAAGKTGPRRTKAMGIGDPGGVSLQR
KGDALANQDTCYAALRVLRQIILISGTLLKEDLHKKLQELVVPLCLKLQQQAQCSNWDVN
SVSGQYGSGAPRCELYALLLALVLVPSPRWPAPLSCAVCVFSQGRRDQNITVSSFCAEAL
TICNALLHPRTPSISLPLPPLTLKPTPAASVLGPTQNPSLSLPTLLGTPAPGHSFAPRHP
LSLGPASLLGSLENHIPLDPPVLPTPTGVTGAQGELLLSSPAQAAELAGLAAPPETQRQV
FVRYDKEEPEDVEISLESDSDDSVVIMPQGMMLEMQEGAATTQSLPPPSGSAIPVPNAGM
GSEAGAVETSLSSDLPTSIGHQMLPSDANNINSFPGSSQTDQLVSLVPTLNSNSVPLATS

TGALGNSLPAGAQLQQMLMQPSPGVQPSQLGLSLHMQLQNQLAQSSRQPVANDQDQNVIN
ITDDEEEEEDEEMEDEDELGEEEEDEEGLDDEEEEVCEFAEEGFIQDEEGFEEFDEEEED
DDDVDDIEEEEEEEDEEEIQALEADNQGNVIGAEEGEAMMEGQEERAVGTFPMEGERPVE
GGIEEMKAVQSIYEEEGIKDKGGIEEIENIGAVERNESMVGEQPIETHVIGAEEVQSEEN
ALVQADHHEVQPQEQKGSRPESVVNPEDSGIIPEGQEQEVAIEVKDNESSAEAEHEEMPI
PSSVTTSENTAPQQGVETAEKDVVEETEMVGEEARGTKRKIEDREEGEVSEQRSEKRK
> Equus asinus [A0A8C4MIG0]
MAAAVLSGPSAGSAAGAAGGTGGLSAVASGPRLRLLLLESVSGLLQPRAGSTVAPVHPHV
RSAAHLPGLMCLLRLHGTVGGAQNLSAVGALVGLSNARLGSIKTRFEGLCLLSLLVGESP
TEMFQQHCVSWLRSIQQVLQSQDSPPTMELAVAVLRDLLRYAAQLPTLFRDISMNHLPGL
LTSLLGLRPECELSALEGMKACMTYFPRACGSLKGKLASFFLSRVDALSPQLQQLACECY
SRLPSLGAGFSQGLKHTESWEQELHSLLASLHSLLGALYEGAETAPMQYEGPGVEVLLSP
SEDGDAHALLRLRQRFSGLARCLGLMLSSEFGAPVSVPVQEILDVICRTLSISAKNISLL
GDGPLRLLLLPSIHLEALDLLSALILACGGRLLRFGALISRLLPQVLNAWSIGRDTLSPG
QERPYSTMRTKVYAVLELWVQVCGASAGVLQGGASGEALLTHLLSDISPPADALKLRSPR
GSPDGGLQSGKPSAPKKLKLDVGEAMAPPSHRKGESNANSDVCAAALRGTIPAWGKMSPV
ELTFCPGLSRTILMCGPLIKEETHRRLHDLVLPLVMGVQQGEVLGSSPYTSSRCRHELYR
LLLALLLAPSPRCPPPLACALQAFSLGQREDSLEVSSFCSEALVTCAALTHPRVPPLQSM
GPTCPTSAPVPPPEAPSPFRAAPFHPPGPMPSAGPMPSAGPVPSAGPMPSVGPLPSVGPM
PSVGPMPPARPGPPATANHLGLSVPGLVSVPPRLLPGPENHRAASNEDPVLAPSGTPPPA
IPPDETFGGRMPRPAFVHYDKEEASDVEISLESDSDDSVVIVPEGLPSLPPPPPSGTTPP
PVAPAGPPTASPPVPAKEEPEELPAAPGPLPPPPPPPVPGPVTLPPPQLVPEGTPGGGGP
PALEEDLTVININSSDEEEEEEEEEEEEEEEEEEEEEDFEEEEEDEEEYFEEEEEEEEEF
EEEFEEEEGELEEEEEEEDEEEEEELEEVEELEFGSAGGEVDEAGPPPPSLPPALPPAES
PKVQPEPEPEPGLLLEVEEPGVEEERGAETAPTLAPEVLPSQGEVEQEGGSPPAGPPPQE
LVEEEPSAPPTLLEEGTEGGDDKVPPLPEPPAAEEMETETEVAALQEKEQDDTAAMLADF
IDCPPDEEKPPSATEPDS
>Equus asinus[A0A9L0ILJ5]
MAAAVLSGPSAGSAAGAAGGTGGLSAVASGPRLRLLLLESVSGLLQPRAGSTVAPVHPHV
RSAAHLPGLMCLLRLHGTVGGAQNLSAVGALVGLSNARLGSIKTRFEGLCLLSLLVGESP
TEMFQQHCVSWLRSIQQVLQSQDSPPTMELAVAVLRDLLRYAAQLPTLFRDISMNHLPGL
LTSLLGLRPECELSALEGMKACMTYFPRACGSLKGKLASFFLSRVDALSPQLQQLACECY
SRLPSLGAGFSQGLKHTESWEQELHSLLASLHSLLGALYEGAETAPMQYEGPGVEVLLSP
SEDGDAHALLRLRQRFSGLARCLGLMLSSEFGAPVSVPVQEILDVICRTLSISAKNISLL
GDGPLRLLLLPSIHLEALDLLSALILACGGRLLRFGALISRLLPQVLNAWSIGRDTLSPG
QERPYSTMRTKVYAVLELWVQVCGASAGVLQGGASGEALLTHLLSDISPPADALKLRSPR
GSPDGGLQSGKPSAPKKLKLDVGEAMAPPSHRKGESNANSDVCAAALRGLSRTILMCGPL
IKEETHRVSSFCSEALVTCAALTHPRVPPLQSMGPTCPTSAPVPPPEAPSPFRAAPFHPP
GPMPSAGPMPSAGPVPSAGPMPSVGPLPSVGPMPSVGPMPPARPGPPATANHLGLSVPGL
VSVPPRLLPGPENHRAASNEDPVLAPSGTPPPAIPPDETFGGRMPRPAFVHYDKEEASDV
EISLESDSDDSVVIVPEGLPSLPPPPPSGTTPPPVAPAGPPTASPPVPAKEEPEELPAAP
GPLPPPPPPPVPGPVTLPPPQLVPEGTPGGGGPPALEEDLTVININSSDEEEEEEEEEEE
EEEEEEEEEEDFEEEEEDEEEYFEEEEEEEEEFEEEFEEEEGELEEEEEEEDEEEEEELE
EVEELEFGSAGGEVDEAGPPPPSLPPALPPAESPKVQPEPEPEPGLLLEVEEPGVEEERG
AETAPTLAPEVLPSQGEVEQEGGSPPAGPPPQELVEEEPSAPPTLLEEGTEGGDDKVPPL
PEPPAAEEMETETEVAALQEKEQDDTAAMLADFIDCPPDEEKPPSATEPDS
>Tachysurus vachellii [A0AA88NIX2]
MATAAWLHVPKNMRLTEGLVSALKEERPEYVPALLSNYREHGVVGTQNCGTVGGLVGISN
SRLGSSKTRFEGLCMLSMLVKDSSSEVFQQHCLSWLRSLQQIIQSQAPLPSVQLAVNVLQ
DVLQYSSQLPELAREVGLNSILGILTSLLGLKSECHLAAMEGMMACMTYYPRACGSLKEK
LGVYFLSKMDSDNPKVQDVACKCYGRLPCLGGVLERGGGGRRAEGWTSQVHCLLASANSI
LGQLYQGIETEETMQYEGPGVELPFLPLDDVDPLLILQLRHRYRSVCLALKHTLSVDPAI
SVRLPVQHILNLVCRALAVGIKNINVTSEGCLKLLVLPSIHSDTLEVLSALIKVVGAGLV
QYCNVLSRLFSQALCAWTPLPEASLGQQRAYSAVRVALYHTLELWVCVSRASSVLQGNSS
HSELLFAHLIGDITPGTEAVKLRAGQAAMSDLVSAPGKTGPRRTKGIGIVEPGGVSLQRK
GDALANQDTCFAALRVLRQIILTSGTLLKEDLHKKLQDLVVPLCVRLQQQAQCSNWDMGG
VSGQYGSAAPRCELYALLLALVLVPSPRWPAPLSCAVCVFSQGRKDRNITVSSYCAEALT
ICNALLHPRTLSISLPLPPLALKPTPAASVLAPTQNPSLSLPTLLGAPAPGPSFAPRHPL
SLDSASLLGSLENHLPLGPPVLSTPAGVTGVQGELLLSSPAQAAELAGLTAPPETQRQVF
VRYDKEEPEDVEISLESDSDDSVVIMPQGMMLEMQEGAGNTQSLPPPTGSAMPVPNAGLG
SEAGPVETSLSSDHPTTIGHQILPADGNNINSFPGTSQTEQLVSLVPPLNSNAVPLAASS
GALGNSLPAGAQLQQMLMLPSSGGQQSQIGLSLHIQLQNQLVQSSRQPAANEQDQNVINI
NSTDDEEEEEEEEELEDEDELGEEEEEEEEGLEEDEEEEEGSEYPEDGFLPGDDFEGFDDE
EDEGEEIEEEEDEEEDDEEEIQALEAESQRDVIGAEEGEVMIEEQEERRVGTFPMEGERP
VEGGIEEMKAVQSIFEEEGVKEKVGIEEIENIGAVERNESVVGGQQIETHVIGAEGEQSE
LNASTEAADQEVLPQEQDGSRPESVVTPEDSGIPSEGQEQEVAIGVRENEPSSEVEQEEI
PNPCTATTSEDTETKQDNRESETTEKEVVEGVQVEEEETRGTKRKIEDIEEGEVSEQSSE
KKKLDDEAMASMLADFVDCPPDDEDHGSSHWNLWNLATACLGPALRVNHLSGWVGSLLRN
QTQATVMEYKVTVATGTLEYSGTNNYVYVTLVGENGESERTLLDKPGLDLCRGAVDDYIV
RSSAPLGRVLLVRLEKQRYFLEDNWFCNYVKVTPPGGENLQTFPCYRWLVGDIKIEVREG
TAKKLSDEILPQELAHRKAQLKERQNTFRWQAWAPGIPKCIDAKSEADLPQDVRFANEKR
SDFERSLQFALLELSLKKLVIWFGRSWEDLDDFKQIFWKLRSPIAEYTMEHWKEDWFFGH
QFLNGSNPRMIQRCRQLPSNFPVSGDMVQAFLSPNTTLNKELKAGNIYLVDYAIMDGIPE
NVIRGKKQYIAAPLCLLYEHPDDGLIPIAIQLEQNPNKETPIFLPNDPPLAWLLAKMWVR
HAEFQIFQVLSHLLRTHLIVEVFCVATLRHLPSVHPINKLLNPHLKYTLEINCRGRTQLI
SRNGIFKRVVSTGGDGLLVLAQREYKVLTYRSLQPRYDFIDRGVTKLNKYFYREHSLMLW
DAIEKFVSSIVSLYYGSDNDVVQDSELQAWIKDIVEEGFVNVPHFGLPNELKNKQELITV

LSVVIFCSTAQHAATNNGQFDFCSWVPNTPCTMRQPAPTDKDGVTMELIMNTLPDISQSC
VEMAITWHLGRAQPDAIPLAQYEEQYFTEPAAQKMIDNFRQDLKDIEEEILEQNKGLEPP
YLYLCPSRIENSITI
>Cirrhinus molitorella [A0AA88Q305]
MASAAWLHGPNVTRLTEGLVSVLKENRPEYLPALLANYREHGVVGTQSTGAVGGLVGISN
ARLGSSKTRFEGLCLLSILVMDSSSEVFQHHCLSWLRTLQQVIQSQAPLPTVQLAVTVLQ
DLLQYSSQLPELAREVGLNSILGILTSLLSLKSECHLAAMKGMMACMIYYPRACGSLREK
LGAYFLSKMDSDNPKVQEMACECYGRLPCLGGVLERGGGGRRAEGWTNHLHCLLASANSM
LGQLYHGSETEGTVQYEGPGVELPFPPVDDVDPLLILQLHHRYKAVSLAIKHTLSADPAS
SVRLPVQHVLNLVCRALAVNTKSISPTGEGCLKLLVLPSIHNDTLELLSALIKAVGGGLV
QYSSVLTRLFSQSLSAWTPLPEASLGQQRAYSAVRVTVYRAIELWVKVGGASLLQGSPSH
TELLFTHLMGDITPASEAVKLRSGQQSQSMNELIGSAGKSGPRRTKGLGMGDGISLQRKG
DVLANQDTCVAALRALRQIIVTSGTLLKDDLHKRIQVLVVPLCVRLQQQSHCVLEVGAVS
GQYGSPAPRRELYRLLLALVLVPSPRWPPPLSCAVSAFSHGRRDRNIMVSSFCAEALTIC
NTLIHPRTPSISLPLNPLTLKSTPSAPVLASGQNPSLSIPTLLGGPATGPPFPARHPMGL
GPASLLGSLENHLPLAPSVLPTPAGTTATPGDLLLSPAQPGELPGLGAPEGQRQVFVRYD
KEEPEDVEISLESDSDDSVVIMPAGMMMEMQDGAANAQLLSQPAVPAVGGLQSSAPIVGE
VGSVDSSLPNELPTSIPQQILPANANNINSFPGASQTAQLVSLVPPLNSTTASLSASPAG
LADSLTGGQQLQQMLMQTSPGGQPVSFQIQLQSQIAQTSRQLQQQPPANEVDQNVININS
SDDEEEEELEEEDELGEEEEEEEGLEDEEEEEEGSDFMEEEEEYLEEEDFEDYDDEEGED
EDEEESEEIQPLEGDNGRGMMGEEEAEVMIEAEEQQQRIEMFCMEREQEVEPGIEEMEGV
RSVYADDRIKDKATVEEIENIGAVERNEPVIDKEEIESLVIGGDAEGHEEDIGVEAVEPE
AKTSEQEVARPEDLAEEAGPSQQGQELTVEEEVQKQETESQPEDTTNQSAPSTSEQEPLQ
SVAEAAEEEAEKEAEKESSERGEDSDARGTKRKMEDREEGESSEQGTEKKKMDEAMASML
ADFVDCPPDDDDHGASQSQS
>Conger conger [A0A9Q1DUH1]
MATAVWLHGSNNMRLTEGLVSALKEERPEYLPALLANYREHGGISTQSSGAAGGLIGLSN
ARLGSSKTRFEGLCLLSILVKDSSSEVFQQHCLSWLRTLQQVIQSQAPLPSVQLAVSVLQ
DLLQYSSQLPELAREVGLNSILGILTSLLGLKSECHLAAMEGMTACMTFYPRACGSLRDK
LGAHFLSKMDSDYPKVQEVASECYGRLPCLGGVLERGGGGRRAEGWTNQLHCLLASAHAT
LSQLYQGVECERTVQYEGPGIELPFPPLDEADPLLIVQLRHRYRAVCLALKHTLSVDPVS
PVRLPVQSVLNLVCRALAVSCKSINVSGDGCLKLLVLPSVHSDTLELLSAVITAVGSRLV
PYCSVLTRLFSQTLSAWSPLAEVSLGQQRAYSAVRVSLYQTLELWVRVAGASSGVLQGSP
THTELLLAHLLGDITPGADSVKLRVGQAAISELMGHTGKAGPRRPKGLGMGDGSAVSLQR
KGDSLANQDTCLSALRALRQIILTSGTLLKEDIHKRLQDLVLPLCVRLQQQLQCGSEVGG
VSGQYGNAPPRRALYRLLLAMVLAPPPRWPPPLTCAVSIFSHGRKDRSLLVSSFCAEALT
VCNSLLHPRTPSLALPLPSLTLKHTAAAPTLTPSQNPSLSLPSLLGGPAQGAPFPARHPL
SLGPAGLLGPLENHLPLPPSVLPPQAGPATTAGDLLLSPPQPGELAALGAPEGHRPVFVR
YDKEEAEDVEISLESDSDDSVVIVPQGMLLLESHDAASTQPLPPPPPGGAVPSVGAGTGG
DTGVVSSPLPNELPASMPHQILPSNSNAISTFPGQSQAQLVSVVPQLNSTAAQLAAPPVG
LGDSLPGAQLQQMLMQPSSAGQPSQLGLPMQMQLQNQMAQTSRQLQQQPQPSEEDLTVIN
INSSDEEEEEDEEMEDEDDLCEEEEEEEEGLEDEEEEEEGSDFADDDDYYGEEEFEDYED
EEEGMIEAEEMEEEAEGLPPLEGESRRALMSREEGGVLMTSADERGMGMFRLEREEEEDA
EVEGGGSELEGDHSVYRQPLHSKEGNREERVKEEGEGEVVVTEEGMDKIREGEQDDVAVQ
ESKGDPQSQGPAEEVMEEASEFEKETGVELGSLEQASQEPGPTQECDSATVDDMAISHEE
GEVESLQEVAVPEEKEPVESTSEQPRPEEGEKAKLHEDIPVEEARSPRQSEEGRASVAQE
DEEEEGEDDLRGMKRKREEREVEGAELSVEKKKLDEEAMASMLADFVDCPPDEEDNAHST
THS
>Electrophorus voltai [A0AAD9DXX0]
MSTFQAFPPLPEERKPLSRLQERLLKKLGQHAHPFNFTIPQNLPCSVTLQPGPEDTGKAC
GVDFEIRAFCAKSVEEKIHKRNSVRLVIRKVQYAPEKPGPQPMVETTRSFLMSDRSLHLE
ASLDKELYYHGEPISVNVHVTNNSTKTVKRVKISGEERWKPQAMLFSSPGNPAVCSLLSL
PTVRQYADICLFSTAQYKCPVAQAEADDQVSPSSTFCKVYTLTPTLNNNREKRGLALDGK
LKHEDTNLASSTIVKDISNKEVLGILVSYRVKVKLVVSRGGDVSVELPFVLMHPKPSEQP
NSRPQSAVPETDAPVDTNLIEFETNNFSQDDDFGGKMAAAAWLHGPNGVRLTEGLASALR
EDRAEHLPALLADYREHGVVATHHSGAVGGLVGISNARLGSSKTRFEGLCLLSMLVKDSS
SEVFQQHCLSWLRTLQQIIQSQAPLPTVQLAVSVLSDLLQYSSQLPELAREVGLNSVLGI
LTSLLGLKSECHLAAMEGMIACMTYYPRACGSLKEKLGVYFLSKMDSDNPKVQEVACECY
GRLPCLGGVLERGGGGRRAEGWTNQVHCLLASANNILGQLYQGIETEETVQYEGPGVELP
FPPIDDIDPLLILQLRHRYRAVCLALRHTLGVDPATSVRLSVQHVLNLVCRALAVSIKSI
NATGEGCLKLLVLPSIHSDSLELLSALIKAWVNTNQLLKMAVLPPASISSLKSCVRSGLV
RYCSVLNRLFSQSLSTWASLPEASLGQQRSYSAVRVTLYHTLELWVRVGGASASVLQGST
SHSELLFTHLLGDITPGTEAVKLRAGQPAMPDLVGAAAKAGPRRTKGLGIGEPGCISLQR
KGDALANQDTCFAALQALRQIVLTSGTLLKEDLHKRLQDLVVPLCVRLQQQAQCGAEKGS
VSGQYGGALPRRELYRLLLALVLVPSPRWPPPLSCAVSVFSQGRRDRNIVVSSFCAEALT
ICNSLLHPRTPSISLPLPPLTLKPTPSAPVHAPTQNPSLSVPTLLGAPAPGPSFPARHPL
GLGPAALLGSLENHLPMVPPVLPTPAGASGPPGDLLLSPAQPAELAGLGAPEGQRQVFVR
YDKEEPEDVEISLESDSDDSVVIMPEGMMLEMQEGAVNPQTLAPPPGGAMPAPAAGVVGE
AGTVETALPNELPTSISHQILPSDANNINSFPGPSQGEQLASLVPSLNSNAVPLTAPPGG
LGNSLPAAPQLQQMLLQPSPGGQAAQLTLSLHMQLQNQLAQTSRQLQQQQQPAGAEEDQN
VININSSDDDEEEDDEGIEDEEELGEEEEEEEALEEDEEEEGSDFHDDDIYTEYHDFDMQ
EEEGEEIEDEEEEEEEEEEEEEEDEEEEIQPLDGDNRRGVMAEEQGVVMIEEQEEGGMGAF
HVERERQVEGGIEEMEAVRSVCGEEGMKDKGAVEEIENIGAVEGNESLAPEQQIETHVIG
EDAEQPEESAPAEAVEQEERPWAQEESAPEPSAAPEDTGMPREDQEVTVEGGVNEESAGG
EQDVAPDQSAVSTSGEAAPQQGDEPGEKAKGEEEQAGALEEEEEEEARGTKRKIEDVEEA
EASEQGSENKMEYKVTVATGTSEYSGTNNYVYVTLVGERGASERTLLDNPGLDLCRGAVD
EYTVRSSAPLGRILLVRLEKQRYWVEDNWFCRYVTVTSPGGHSTSTFPCYCWLVGDVQVE

VREGTAKKQSGDTLPQERAHRKAELQERQKIYRWQHWAPGLPKCIDAKSEADLPQDARFA
NEKRSDFERSLQFALLELSLKKLVIKFGKSWDDLDDFKRIFWQLKSSIAEYTMQHWREDW
FFGYQFLNGSNPRMIQRCRKLPSNFPVSGDMVQAFLGPNTTLDKEIKAGNVFLVDHGVMD
GIPANSIRNQKQYIAAPLCLLYEHPDKGLIPIAIQLEQNPSRDTPIFLPNDPPLAWLLAK
MWVRHAEFQVFQVLSHLLSTHLVVEVICVATLRQLPAVHPIYKVGVRSGQGMEIKSKQTS
KTYTSVLVSDKIHFNASINVEPFMAHGIFTVESKQPGSCISYFSFSLTCYLTLPLSLRPI
QLLSPHLKYSLEINCRARTQLISADGIFKRVVSTGGEGLLVLAQRGYKVFTYRSLHPHFD
FVDRGVNKLKNYFYRDHVLMLWEAIHKFVSSMVSQYYSSDSEVEEDTELQAWISDVTEEG
FVDAPQFGLANDLKTREELITLLSVVIFTSTAQHAATNNGQFDWCAWVPNTPCTMRQPPP
TDKDAVTMGFIMDTLPDISQSCVQMAITWHLGRVQPDAIPLAQYAEQYFTEPEALRVIDS
FREDLKEVEEEILRQNKGLELPYLYLCPSRIENSITI
>Homo sapiens [B4DR36]
MLLPSLSPRVQPAFTQCARPQPPPHAPSPVGGRRGVSASSRHATTGTRGKMAAAVLSGPS
AGSAAGVPGGTGGLSAVSSGPRLRLLLLESVSGLLQPRTGSAVAPVHPPNRSAPHLPGLM
CLLRLHGSVGGAQNLSALGALVSLSNARLSSIKTRFEGLCLLSLLVGESPTELFQQHCVS
WLRSIQQVLQTQDPPATMELAVAVLRDLLRYAAQLPALFRDISMNHLPGLLTSLLGLRPE
CEQSALEGMKACMTYFPRACGSLKGKLASFFLSRVDALSPQLQQLACECYSRLPSLGAGF
SQGLKHTESWEQELHSLLASLHTLLGALYEGAETAPVQNEGPGVEMLLSSEDGDAHVLLQ
LRQRFSGLARCLGLMLSSEFGAPVSVPVQEILDFICRTLSVSSKNISLHGDGPLRLLLLP
SIHLEALDLPSALILACGSRLLRFGILIGRLLPQVLNSWSIGRDSLSPGQERPYSTVRTK
VYAILELWVQVCGASAGMLQGGASGEALLTHLLSDISPPADALKLRSPRGSPDGSLQTGK
PSAPKKLKLDVGEAMAPPSHRKGDSNANSDVCAAALRGLSRTILMCGPLIKEETHRRLHD
LVLPLVMGVQQGEVLGSSPYTSSRCRRELYCLLLALLLAPSPRCPPPLACALQAFSLGQR
EDSLEVSSFCSEALVTCAALTHPRVPPLQPMGPTCPTPAPVPPPEAPSPFRAPPFHPPGP
MPSVGSMPSAGPMPSAGPMPSAGPVPSARPGPPTTANHLGLSVPGLVSVPPRLLPGPENH
RAGSNEDPILAPSGTPPPTIPPDETFGGRVPRPAFVHYDKEEASDVEISLESDSDDSVVI
VPEGLPPLPPPPPSGATPPPIAPTGPPTASPPVPAKEEPEELPAAPGPLPPPPPPPPPVP
GPVTLPPPQLVPEGTPGGGGPPALEEDLTVININSSDEEEEEEEEEEEEEEEEEEEEEDF
EEEEEDEEEYFEEEEEEEEEFEEEFEEEEGELEEEEEEEDEEEEEELEEVEDLEFGTAGG
EVEEGAPPPPTLPPALPPPESPPKVQPEPEPEPGLLLEVEEPGTEEERGADTAPTLAPEA
LPSQGEVEREGESPAAGPPPQELVEEEPSAPPTLLEEETEDGSDKVQPPPETPAEEEMET
ETEAEALQEKEQDDTAAMLADFIDCPPDDEKPPPPTEPDS
>Homo sapiens [B4DEX7]
MELAVAVLRDLLRYAAQLPALFRDISMNHLPGLLTSLLGLRPECEQSALEGMKACMTYFP
RACGSLKGKLASFFLSRVDALSPQLQQLACECYSRLPSLGAGFSQGLKHTESWEQELHSL
LASLHTLLGALYEGAETAPVQNEGPGVEMLLSSEDGDAHVLLQLRQRFSGLARCLGLMLS
SEFGAPVSVPVQEILDFICRTLSVSSKNISLHGDGPLRLLLLPSIHLEALDLLSALILAC
GSRLLRFGILIGRLLPQVLNSWSIGRDSLSPGQERPYSTVRTKVYAILELWVQVCGASAG
MLQGGASGEALLTHLLSDISPPADALKPRSPRGSPDGSLQTGKPSAPKKLKLDVGEAMAP
PSHRKGDSNANSDVCAAALRGLSRTILMCGPLIKEETHRRLHDLVLPLVMGVQQGEVLGS
SPYTSSRCRRELYCLLLALLLAPSPRCPPPLACALQAFSLGQREDSLEVSSFCSEALVTC
AALTHPRVPPLQPMGPTCPTPAPVPPPEAPSPFRAPPFHPPGPMPSVGSMPSAGPMPSAG
PMPSAGPVPSARPGPPTTANHLGLSVPGLVSVPPRLLPGPENHRAGSNEDPILAPSGTPP
PTIPPDETFGGRVPRPAFVHYDKEEASDVEISLESDSDDSVVIVPEGLPPLPPPPPSGAT
PPPIAPTGPPTASPPVPAKEEPEELPAAPGPLPPPPPPPPPVPGPVTLPPPQLVPEGTPG
GGGPPALEEDLTVININSSDEEEEEEEEEEEEEEEEEEEEEDFEEEEEDEEEYFEEEEEE
EEEFEEEFEEEEGELEEEEEEEDEEEEEELEEVEREGESPAAGPPPQELVEEEPSAPPTL
LEEETEDGSDKVQPPPETPAEEEMETETEAEALQEKEQDDTAAMLADFIDCPPDDEKPPP
PTEPDS
>Bos Taurus [A0AAA9RZS9]
MAAAVLSGPSAGSAAAVPGGPGGLSAVGSGPRLRLMLLESVSGLLQPRTGSAVAPVHPPA
RSAPHLPGLMCLLRLHGTVGGAQSQDPPPTMELAVTVLKDLLRYAAQLPAVFRDISMNHL
PGLLTSLLGLRPECELSALEGMKACMTHFPRACGSLKGKLASFFLSRVDALSPQLQQLAC
ECYSRLPSLGAGFSQGLKHTDSWEQELRSLLASLHSLLGGLYEGAEAAPMQYESPGAETL
LSSSEDADAHTLLRLRQRFSGLARCLGLMLSSEFGAPVSVPVQDILDLICRTLSVSAKNV
SLLGDGPLRLLLLPSLHLEALDLLSALILACGARLLRFGALISRLLPQVLNAWSIGRENL
GPGQERPYSTVRTKVYAVLELWVQVCGASAGVLQGGASGEALLSHLLSDISPPADALRLR
SPRGSPDAGLQTGKPSAPKKLKLDVGEAIAPPSHRKGDSNANSDVCAAALRGLSRTILMC
GPLIKEETHRRLHELVLPLVMGVQQGEALGSSPYTSSHCRRELYHLLLALLLAPSPRCPP
PLACALRAFSLGQREDSLEVSSFCSEALVTCAALTHPRVPPLQSVGPTCPAPAPVPPPEA
PAPFRAPAFHAPSPLPSAGPMPSAGPMPPVGPLPPTRPGPPATANHLGLSVPGLVSVPPR
LLPGPENHRAGSSEDPVLAPSGSPPPTIPPDETFGGRVPRPAFVHYDKEEPSDVEISLES
DSDDSVVIVPEGLPPPPPPSSGTTPPPVAPAGPPAASPPVPAKDEPEELPAAPGPLPPPP
PPPVPGPVTLPPPQLVPEGTPGGGGPPALEEDMTVININSSDEEEEEEEEEEEEDEEEDE
EEDFEEDEEEEEEYFEEEEEEEEEFEEEFEEEEGELEDEEDEEEDEELEELEEVEFGPAG
GEVEGGGPAPPSLPPALPPAESPKGPPEPGLEPGLLLEVEEPGTEEAPGPETAPMLAPEV
LPSQGEVEREGGSPPAGPPPQELVEEEPSGPPALLEEGAEGGGDKVSPPPEASAVEETEV
EAAALPPEKEQGDTAAMLADFIDCPPDDEKPPPASEPDS
>Bos taurus [A0AAA9TEC2]
MENLSAVGALVGLSNARLGSIKTRFEGLCLLSLLVGESPTEMFQQHCVSWLRSIQQILQS
QDPPPTMELAVTVLKDLLRYAAQLPAVFRDISMNHLPGLLTSLLGLRPECELSALEGMKA
CMTHFPRACGSLKGKLASFFLSRVDALSPQLQQLACECYSRLPSLGAGFSQGLKHTDSWE
QELRSLLASLHSLLGGLYEGAEAAPMQYESPGAETLLSSSEDADAHTLLRLRQRFSGLAR
CLGLMLSSEFGAPVSVPVQDILDLICRTLSVSAKNVSLLGDGPLRLLLLPSLHLEALDLL
SALILACGARLLRFGALISRLLPQVLNAWSIGRENLGPGQERPYSTVRTKVYAVLELWVQ
VCGASAGVLQGGASGEALLSHLLSDISPPADALRLRSPRGSPDAGLQTGKPSAPKKLKLD

VGEAIAPPSHRKGDSNANSDVCAAALRGLSRTILMCGPLIKEETHRRLHELVLPLVMGVQ
QGEALGSSPYTSSHCRRELYHLLLALLLAPSPRCPPPLACALRAFSLGQREDSLEVSSFC
SEALVTCAALTHPRVPPLQSVGPTCPAPAPVPPPEAPAPFRAPAFHAPSPLPSAGPMPSA
GPMPPVGPLPPTRPGPPATANHLGLSVPGLVSVPPRLLPGPENHRAGSSEDPVLAPSGSP
PPTIPPDETFGGRVPRPAFVHYDKEEPSDVEISLESDSDDSVVIVPEGLPPPPPPSSGTT
PPPVAPAGPPAASPPVPAKDEPEELPAAPGPLPPPPPPPPVPGPVTLPPPQLVPEGTPGGG
GPPALEEDMTVININSSDEEEEEEEEEEEEDEEEDEEEDFEEDEEEEEEYFEEEEEEEEE
FEEEFEEEEGELEDEEDEEEDEELEELEEVEFGPAGGEVEGGGPAPPSLPPALPPAESPK
GPPEPGLEPGLLLEVEEPGTEEAPGPETAPMLAPEVLPSQGEVEREGGSPPAGPPPQELV
EEEPSGPPALLEEGAEGGGDKVSPPPEASAVEETEVEAAALPPEKEQGDTAAMLADFIDC
PPDDEKPPPASEPDS
>Nothobranchius rachovii [A0A1A8Q1P4]
MAASAWLHGSAAMRLTEGLVLLMKEQRPEFLPEVLANYREHGVLSLQSASDVAGLVGLCN
GKLSSSKTRLEGLCLLSMLVKDSSSDLFQQHCLSWLRSLQQVVQSQAPVQTIQLAVNVLK
DLLQYSCQLPELAREVGLNSILGILTSLLGLKMECELSAMEGMKACMVYYPRACGSLKDK
LGAYFLSKMDSTSRKTQEAACQGYSHLPCLGGLLDRAVGASRAEGWTNQIHCLLASANSL
LAQIYQGSETDEAVRYQGPGVELAFPHIDHTEPLLLLQHRYAAVCMTIKHTLRTDPASAV
SVPVRPILNLVCRALAVNSKNMILTADGSLRFLVLPSVHINALEVLSELITVVRSGMVQY
AAVIQRLFSQTLSAWTPLPETSVGQQRAYSSVRVSVFRSLELWVKVAGASACILHGTSSH
VDLLLNHLLGDITPGAESVKLRAGLSADVVPVGKPGPRRTKQLVVADAVGPSLQRKGDLL
SNQDTCLAALKALRQVLQSTGMLLKDDIHKRLHEVVLPLCVRLQQQQSSSISAWEPAGGV
SGQYSSALTRRELYRVLLALVLVPSPSWPPPLTCAVSILSNGRIDRHLKVSSFCAEALTI
CNSILHPRRPSLALPLPPLTLKPTATPSVLSCQGPGPRLTLPTLLGGPSSAPPFPARHTL
NLGPSSLLGSLDNHFSLVPGLPGQAPGPGDLMASSQTHHQPDPSGLAPPEGQRPVFVRYD
KEETEDVEISLASDSDDSVVIVPPGMLNVEKQQNDLAANSQSLLSSAPGGAELVTMVPTS
STPTTLDVTSLPNDLATSSALLTSSVTPVNSFPPSSTSVVSLVPSVNSSSTSAPPGGLGD
PLAGKPQLQQMLMQPSTTGQPSSMALPLQMHQLTQQGRHLHQHPPTPAANEESAVININS
TDDEEDDEEDMEEDEELEDEEDGIDEEDEEEEGSDEFYEGEDYDDFDEEEGEELGEEEEE
EEDGDMPPLEGSEDRSEEVGMEDDKVLLPMEEGEVGEFCVEGDGEGGIEELQTGRALFGE
DRMKVQEVESIGVLEESREEGEGEEDESEQMDDPTMPQILCVTGGALEERLESGEEGVVA
GDGLQEEMGLWDQGAKETELIIAAEEQEASQSQLDMVAENELQAAEGENQPPSQEEEEAA
AGGGEPAGADEQPLVAEDIKEQPQTDLDQGDLKEPETSQTEGELGGGGNGCGGERGREEE
KGMKRKRENENTEEEESTQSPEKKKLDEEAVASMLADFVACPPDDEDVATGSNG
>Vulpes vulpes [A0A3Q7SVY9]
MAAAVLSGPSAGSAAGVPGGAGGLSAVGSGPRLRLLLLESVSGLLQPRAGSAVAPVHPPV
RSAPHLPGLMCLLRLHGTVGGAQNLSAVGALVGLSNARLGSIKTRFEGLCLLSLLVGESP
TEMFQQHCVSWLRSIQQVLQSQDPPPTMELAVAVLRDLLRYAAQLPTLFRDISMNHLPGL
LTSLLGLRPECELSALEGMKACMTYFPRACGSLKGKLASFFLSRVDALSPQLQQLACECY
SRLPSLGAGFSQGLKHTESWEQELHSLLASLHSLLGALYEGAETAPVQYEGPGVEVLLTP
SEDGDTHVLLRLRQRFSGLARCLGLMLSSEFGAPVSVPVQEILDVICRTLSISAKNISLL
GDGPLRLLLLPSIHLDALDLLSALILACRGRLLRFGALISRLLPQVLNAWNLGRDTLAPG
QERPYSTVRTKVYAVLELWVQVCGASAGMLQGGSSGEALLSHLLSDISPPADTLKLRSPR
GSPDGGLQTGKPSAPKKLKLDVGEAMAPPSHRKGDSNANSDVCTAALRGLSRTILMCGPL
IKEETHRRLHDLVLPLVMAVQQGEVLGSSPYTSSRCRQELYRLLLALLLAPSPRCPPPLA
CALQAFSLGQREDSLEVSSFCSEALVTCAALTHPRVPSLRSMGPACPTPAPAPPPEAPSP
FRAPPFHPPGPMPSAGPMPSAGPMPSVGPMPSVGPMPPAGPMPPAGPMAPTRPGPPATAN
HLGLSVPGLVSVPPRLIPGPENHRAGSNEDPVLAPSGTPPPAIPPDETFGGRVPRPAFVH
YDKEEASDVEISLESDSDDSVVIVPEGLPPLPPPPPSGTTPPPVAPSGPPAASPPVPAKE
EPEELYYDKEEASDVEISLESDSDDSVVIVPEGLPPLPPPPPSGTTPPPVAPSGPPAASP
PVPAKEEPEELPLEEEEEEEYFEEEEEEEEEFEEEFEEEEGELEEEDEEEDEEEEEEELDE
LEEVAFGSAAGAAEEGGPPPPXXXXXXXPALPPAQSPKMQPEPQGETGLLLEVEEPAAEE
EPRAEAAPTLAPEVLPPQGEGPREVGSPPAVPPPQELIEEEPPAPPTLLEEGTESGGDKV
PVPQETPVSEDVEAEVEAETAALQEKEQDDTAAMLADFIDCPPDDDKPPPATEPDS
>Camelus ferus [A0A8B6YBY4]
MAAAVLSGPSAGSAAGVPGGTGGLSAVASGPRLRLLLLESVSGLLQPRAGSTVAPVHPPV
RSAPHLPGLMCLLRLHGTVGGAQNLSAVGALVGLSNARLGSIKTRFEGLCLLSLLVGESP
TEMFQQHCVSWLRSVQQVLQSQDPPPTMELAVAILRDLLRYAAQLPTLFRDISMNHLPGL
LTSLLGLRPECELSALEGMKACMTYFPRACGSLKGKLASFFLSRVDALSPQLQQLACECY
SRLPSLGAGFSQGLKHTESWEQELHSLLASLHSLLGALYEGAETAPMQYEGPGVETLLSP
SEDGDAHVLLQLRQRFSGLARCLGLMLSSEFGAPVSVPVQEVLDVICRTLSISAKNISLL
GDGPLRLLLLPSLHLEALDLLSALILACGGRLLRFGALISRLLPQVLNAWSIGRDALSPG
QERPYSTVRTKVYAVLELWVQVCGASAGVLQGGASGEALLTHLLSDIAPAADALKLRSPR
GSPDGGLQTGKPSAPKKLKLDVGEAMAPPSHRKGDSNANSDVCAAALRGLSRTVLMCGPL
IKEETHRRLHDLVLPLAMGVQQGEVLGSSPYTSARCRLELYRLLLALLLAPSPRCPPPLA
CALQAFSLGQREDSLEVSSFCSEALVTCAALTHPRVPPLQSMGPACPTPAPVPPPEAPSP
FRAPAFHPPGPMPSVGPMPSASPMPSAGPMPSAGPVSSAGPMPSVGSMPPARPGPPATAN
HLGVSVPGLVSVPPRLLPGSENHRASSNEDPILAPSGTPPPTVPPDETFGGRVPRPAFVH
YDKEEASDVEISLESDSDDSVVIVPEGLPPLPPPPPSGTTPPPVAPAGPPTASPPVPAKE
ESEELPATPGPLPPPPPPPVPGPVTLPPPQLVPEGTPGGGGAPALEEDLTVININSSDEE
EEEEEEEEEEEEEEEEEDFEEEEEDEEEYFEEEEEEEEEFEEEFEEEEGELEEEEEEEDD
EEEEELEEVEELEFGPAGGEVEEGGPPPPSLPPALPPAESPKVPPEPEPEPEPGLLLEVEEP
GAEEGRGPETAPTLAPEVLPPQGEVQGEEGSPPAVPSPQELGEEEPSAPPTLLEEGAEGV
GDQVPPQPEASAAEEMEMETEAAALQEKEQDDTAAMLADFIDCPPDDENPPPAPEPDS
>Canis lupus dingo [A0A8C0KDS9]
MAAAVLSGPSAGSAAGVPGGAGGLSAVGSGPRLRLLLLESVSGLLQPRAGSAVAPVHPPV
RSAPHLPGLMCLLRLHGTVGGAQNLSAVGALVGLSNARLGSIKTRFEGLCLLSLLVGESP

TEMFQQHCVSWLRSIQQVLQSQDPPPTMELAVAVLRDLLRYAAQLPTLFRDISMNHLPGL
LTSLLGLRPECELSALEGMKACMTYFPRACGSLKGKLASFFLSRVDALSPQLQQLACECY
SRLPSLGAGFSQGLKHTESWEQELHSLLASLHSLLGALYEGAETAPVQYEGPGVEVLLTP
SEDGDTHVLLRLRQRFSGLARCLGLMLSSEFGAPVSVPVQEILDVICRTLSISAKNISLL
GDGPLRLLLLPSIHLDALDLLSALILACRSRLLRFGALISRLLPQVLNAWNLGRDTLAPG
QERPYSTVRTKVYAVLELWVQVCGASAGMLQGGSSGEALLSHLLSDISPPADTLKLRSPR
GSPDGGLQTGKPSAPKKLKLDVGEAMAPPSHRKGDSNANSDVCTAALRGLSRTILMCGPL
IKEETHRVSSFCSEALVTCAALTHPRVPSLRSMGPACPTPAPAPPPEAPSPFRAPPFHPP
GPMPSAGPMPSVGPMPSVGPMPPAGPMPPAGPMAPTRPGPPATANHLGLSVPGLVSVPPR
LIPGPENHRAGSNEDPVLAPSGTPPPAIPPDETFGGRVPRPAFVHYDKEEASDVEISLES
DSDDSVVIVPEGLPPLPPPPPSGTTPPPVAPAGPPVASPPVPAKEEPEELPVAPGPLPPP
PPPPVPGPVALPPPQLVPEATPGGGGPPALEEDLTVININSSDEEEEEEEEEEEEEEEEE
EEEEEEDFEEEEEEEEEYFEEEEEEEEEFEEEFEEEEGELEEEEEEEDEEEEEELDELEEV
AFGPAAGAAEEGGPPPPSPPPALPPAQSPKMQPEPPGETGLLLEVEEPAAEEEPGAEAAP
TLAPEVLPPQGEGPREVGSPPAVPPPQELIEEEPPAPPTLLEEGTESGGDKVPVPQETPA
AEDVEAEVEAETAALQEKEQDDTAAMLADFIDCPPDDDKPPPATEPDS
>Cyprinus carpio [A0A8C1G6C6]
SISFLQPTASSHSDQFKFSSSSIIPFTPKYIKYEKNRTVATSFILTLYGCDDVCFRFEGL
CLLSVLVKDSSSEVFQQNCLSWLRTLQQVIQSQAPLPTVQLAVSVLQDLLQYSSQLPELA
REVGLNSILGILTSLLSLKSECHLVAMNGMMACMIYYPRACGSLREKLGAYFLSKMDSDN
PKVQEVACECYGRLPCLGGVLERGGGGRRAEGWTNQLHCLLASANGMLGQLYQGAESGTV
QYEGPGVELPFPPLDDVDPLLVLQLHHRYKAICLAIKHTLSADPASSVRLPVQHVLNFVC
RALAVNTKSISPTGEGCLRLMILPSIHNDTLELLSALIKAVGGGLVQYSSVLTRLFSQSL
SAWTPLPESSLGQQRAYSAVRVTVYRTIELWVRVGGASLLQASPSHTELLFTHLMGDITP
ASEAVKLRSGQQSQSMNDLIGSAGKSGPRRTKGLGLGDGISLQRKGDVLANQDTCVAALR
GERSIILTSGTLLKEDLHKQSHCVLEVGAISGQYGSPSPRRELYRLLLALVLVPSPHWPP
PLSCAVSVFSHGRRDRNIMVSSFCAEALTICNTLIHPRTPSICLPLTPLTLKSTPTAPVL
SSGQNPSLSIPTLLGGPATGPFPARHPMGLGPASLLGSLENHLPLAPPVLPTPAGTTAIP
GDLLLSPAQPGELAGLGAPEGQRQVFVRYDKEEPEDVEISLESDSDDSVVIMPAGMIMEM
QDGAANAQSLSQSAVPAIGGLQPSAPIVGEVGSVDTLLPNELPTSIPHQILPANANNINS
FPGTSQTAQLVSLVPPLNSSVSPAGLADSMTGGPQLQQMLMQTSPGGQPPTLGLSLQMQL
QNQIAQTSRQLQTQPPANEVDQNVININSSDDEEEEEEELEEEDELGEEEEEEEGLEDEE
EEEEGSDLIDGEYCEDELEDYDDEEDEDDEESEEIQPLEGDSDRGMIGEEDAEVMIEAEQ
QGMEMFCMEREREVEPGIEEMEGVRSVYADERIKDKGTMEEIENIGAVERNEPVVDKDQI
ESLVISGDAEGHEEDSRVEVVEPEVKTCGQEVARPEDPVEDAGLTQQGQELTVEDEVQKQ
EPELKPEGTTNQSAPSTSEQEVLQSVAETAEEEVGKESSEQGEDSETRGTKRKMEDREEG
ESSEQGTEKKKVTE
>Monodon monoceros [A0A8C6C7P5]
MAAAVLSGPSAGSAAGVPGGTGGLSAVNSGPRLRLLLLESVSGLLQPRAGSTVSPVHHPT
RSVPHLPGLMCLLRLHGTVGGAQNLSAVGALVGLSNARLGSIKTRFEGLCLLSLLVGESS
TEMFQQHCVSWLRSIQQVLQSQDPPPTMELAVAVLGDLLRYAAQLPTLFRDISMNHLPGL
LTSLLGLRPESELSALEGMKACMTYFPRACGSLKGKLASFFLSRVDALSPQLQQLACECY
SRLPSLGAGFSQGLKHTDSWEQELHSLLASLHSLLGALYAGADTAPMQYEGPGVETLLSP
SEDGDAHVLLRLWQRFSGLARCLGLMLSSEFGAPVSVPVQEILDLICRTLSVSAKNISLL
GDGPLRLLLLPSLHLEALDLLSALILACRGRLLRFGALISRLLPQVLNAWSIGRDSLSPG
QERPYSTMRTKVYAVLELWVQVCGASAGVLQGGASGEALLTHLLSDISPPADALKLRSPR
GSPDGGLQTGKPSAPKKLKLDMGEALAPPSHRKGDSNANSDVCAAALRGLSRTVLMCGPL
IKEETHRRLHDLVLPLVMGVQQGEVLGSSPYTSSRCRRELYRLLLALLLAPSPRCPPPLA
CALQAFSLGQREDSLEVSAFCSEALVTCAALTHPRVPPLQTMGPACPTPAPVPPPEAPSP
FRAPAFHPPGPMPSVGPMPSVGPMPSAGPMPSPGPVPPAGPMPSVGPMPPARPGPPATAN
HLGLSVPGLVSVSPRLLPGPENHRAGSNEDAVLAPSGTPPPTIPPDETFGGRVPRPAFVH
YDKEEASDVEISLESDSDDSVVIVPEGLPPLPPPPPSGTTPPPAALVGPPTASPPVPAKE
EPEELPAAPGPLPPPPPPPPVPGPVALPPPQLVPEGTPSGGGPPALEEDLTVININSSEEE
EEDDEEDEDEEDFEEEEEEEEEEEEEYFEEEEEEEEEEFEEEFEEEEGELEEEEEEDEDEEEE
VEEVEFGPAGGEVEEGGPAPPSLPPALPPAASPKVQPQPEPEPGLLLEVEEPGAEEGPGA
ETAPTLAPEVLPSQGEVEREGGSPPAVPPPQELVEEEPSVPPTLLEEGAEGGGDKVPPPP
EAAAAEEMEAEAAALQEKEQDDTAAMLADFIDCPPDDEKPPPATEPDS
>Nothobranchius furzeri [A0A8C6KW32]
MAASAWLHGSAAMRLTEGLVLLMKEQRPEFLPEVLANYREHGVLSSQSASDAAGLVGLCN
GKLSSSKTRLEGLCLLSMLVKDSSSDLFQQHCLSWLRSLQQVVQSQAPVQTIQLAVNVLK
DLLQYSCQLPELAREVGLNSILGILTSLLGLKTECELSAMEGMKACMVYYPRACGSLKDK
LGAYFLSKMDSTSRKTQEAACQGYSHLPCLGGLMDRAVGASRAEGWTNQIHCLLASANSL
LAQIYQGSETDEAVRYQGPGVELAFPHIDQTEPLLLLQHRYAAVCMTIKHTLRTDPASAV
SVPVRPILNLVCRALAVNSKNMILTADGSLRFLVLPSVHINALEVLSELITVVRSGMVQY
AAVIQRLFSQTLSAWTPPPETSVGQQRAYSSVRVSVFRSLELWVRVAGASASILHGTSSH
VDLLLNHLLGDISPGAESVKLRAGLSADVVPVGKPGPRRTKQLVVADAVGPSLQRKGDLL
SNQDTCLAALKALRQVLQSTGMLLKDDIHKRLHEVVLPLCVRLQQQQSSSISAWEPAGGV
SGQYSSALTRRELYRVLLALVLVPSPSWPPPLTCAVSILSNGRIDRHLKVSSFCAEALTI
CNSILHPRRPSLALPLPPLTLKPTATPSVLSCQGPGPRLTLPTLLGGPSSAPPFPARHTL
NLGPSSLLGSLDNHFSLVPGLPGQAPGPGDLMASSQTHHQPDPSGLGPPEGQRPVFVRYD
KEETEDVEISLASDSDDSVVIVPPGLLNVEKQQNDVAANSQSLLSSAPGGAELVTMVPTS
STPTTLDVTSLPNDLATSSALLTSSVTPINSFPPSSTSVVSLVPSVNSSSASAPPGGLGD
PLAGKPQLQQMLMQPSTTGQPSSMALPLQMHQLTQQGRHLHPHPPTPAANEESAVININS
TDDEEDDEEDMEDDEELEDEEDGIDEEDDEEEGSDEFYEGEDYDDFDEEEGEELGEEEEE
EEDGDMPPLEGSEDRSEEVGMEDDKVLLPMEEGEVGEFCVEGDGEGGIEELQTGRALFGE
DRMKVQEVESIGVLEESREEGEGEEDESEQMDDPTMPQILCVTGGALEERLESGEEGVVA

GDGLQEEMGLWDQGAKETELIIAAEEQEASQSQLDMVAENELQAAEGEKPPSQEEEEAAA
GGGEPAGADEQPFVAEDIKEQQQTDLDQGDLKEPETSQTEGELGGDGCGGERGREEEKGM
KRKRENENTEEEESTQSPEKKKVKV
>Nannospalax galili [A0A8C6QUJ5]
MAAAVLSGPSAGSPAGAAGGAGGLSAAGSGPRLRLLLLESVSGLLQPRTGSSVAPVHPPV
HWAPHLPGLMCLLRLHGTVGGAQNLSALGALVNLSNAHLGSIKTRFEGLCLLSLLVGESP
TELFQQHCISWLRSIQQVLQSQDPPPTMELAVAILKDLLRYASQLPTLFRDISTNHLPGL
LTSLLGLRPECEQSALEGMKACVTYFPRACGSLKGKLASFFLSRVDALSPQLQQLACECY
SRLPSLGAGFSQGLKHTENWEQELHSLLASLHSLLGALYEGAEMAPVQSECPGVETLLAH
SEDGNTHVLLRLQQRFSGLAHCLGLMLSSELGAPVSMPVQEILDLICRILSISGKNISLL
GDGPLRLLLLPSIHLEALDLLSALILVCGGRLMRFGTLISRLLPQVLNTWSTGRDTFSPG
QERPYSSIRTKVYAILELWVQVCGASAGMLQGGASGEALLTHLLSDISPPADALKLRSPQ
GSSDGGLQSGKPSAPKKLKLDVGEAMAPSSHRKGDSNANSDVCAAALRGLSQTILMCGPL
IKEETHRRLHDLVLPLVMGLQQGDVLGSSPYNSSCCRRELYRLLLALLLAPSPRCPPPLA
CALQAFSLGQREGSLEVSSFCSEALVTCAALTHPRVPPLQAMGPTCSTPAPVPPSEAPSP
FRAPPFHPPGPMPSIGAMPSPGPVPSAGPMSSTGPMTSAGPMPSTGPVPSRPGPPATTNH
LGLSVPGLVSVPPRLLPGPENHRAGSNEDSVLAPSGTPPPSIPPDETFGGRVPRPAFVHY
DKEEASDVEISLESDSDDSVVIVPEGLPPLPPPPPSGSPPPPVAPTGPPTASPPMPAKEE
SEELPATPGPLPPPPPPPPPASGPVALPPPQLVPEGTAGGGGPPALEEDLTVININSSDE
EEEEEEEEEEEDEDEEEEDFEEDEEEEEEYFEEEEEEEEFEEEFEEEEGELEEEEEEEE
EEEELEDVEDMEFGSAGGEIEEGGPPPPTLPPALPSADSPKIQPEPDAEPEPGLLLEVEE
PGTEEVPGPETAPTLAPEVLSSQEGVEQEGGSPVAGPPLPETVEEESSAPPTLLEEGTES
GGDKVPPPAETSAEEEMETEAEATTVKEKEQDDTAAMLADFIDCPPDDEKPPPATEPDS
>Spermophilus dauricus [A0A8C9PD36]
MNSGPRLRLLLLESVSGLLQPRTGSTVAPVHPPVCSVPHLPGLMCLLRLHGTVGGAQNLS
ALGALVSLSNARLGSIKTRFEGLCLLSLLVGESPTEMFQQHCVSWLRSIQQVLQSQDPPP
TMELAVAVLRDLLRYAAQLPTLFRDISINHLPGLLTSLLGLRPEVRHFPTALPLGVFALI
YTKWDGGGFHLTSFLPFLLQLACECYSRLPSLGAGFSQGLKHTESWEQELHSLLASLHSL
LGALYEGAETAPVQNEGPGVETLLSPSEDGDAHVLLRLRQRFSGLARCLGLMLSSEFGAP
VSVPVQEVLDLICRTLSVSGKNISLLGDGPLRLLLLPSIHLEALDLLSALILACGGRLLR
FGALISRLLPQVLNAWSIGRDTLSPGQERPYSTIRTKVYAILELWVQVCGASAGVLQGGA
SGEALLTHLLSDISPPSDALKLRSPRGSPDGGLQTGKPSAPKKIKLDVGEAMAPPSHRKG
ENNANSDVCAAALRGLSRTILMCGPLIKEETHRRLHDLILPLVMGVQQGEVLGSSPYTSS
CCRRELYRLLLALLLAPSPRCPPPLACALQAFSLGQREDSLEVSSFCSEALVTCAALTHP
RVPPLQAMGPTCPTPAPVPPPEAPSPFRAPPFQPPGPMPSVGSMPSVGPMPSAGPMPSAG
PVPSARPGPPATANHLGLSVPGLVSVPPRLLPGPENHRAGSNEDPVLAPSGTPPPTIPPD
ETFGGRVPRPAFVHYDKEEASDVEISLESDSDDSVVIVPEGLPPLPPPPPSGTTPPPVAP
TGPPTASPPVPAKEEPEELPAAPGPLPPPPPPPPVPGPVTLPPPQLVPEGTPGGGGPPALE
EDLTVININSSDEEEEEEEEEEEEEEEEEEEEEEEEEFEEEEEEEEEYFEEEEEEEEEEFEEE
FEEEEGELEEEEEEEEEEEEELEELEEVEDLEFGSAGGEVEEGGPPPPTLPPALPPPESPK
EHPEPEPEPGLLLEVEEPGSEEQHGTETAPTLAPEVLPSQGEEEREGGSPEAGPPPQELV
EEEPSAPPTLLEEGTEGGGDNEPPPPETAAEEEMETETEAAVLQEKEQDDTAAMLADFID
CPPDDEKPPPTTEPDS
>Varanus komodoensis [A0A8D2LHP3]
MTPSCAGPWPPSWRPFLSGAGTWGGLLWAHVGWLGGARRGAGLVPRRRPDPVLRSYPFCR
PPCRFEGLCLLSLLVTESTTETFSQNCLSWLRSLQHLIQSQDPAPTMELAVLVLRDLLEC
SCQLPELARDISTNHVPGLLTSLLALRPECQLSALEGSKACMMFYPRACGSLRGKLAAYF
LSRVDAETPQVQQLACECYALLPSLGAGFTQGLKYTECWEQQAHCLLATLHSLMGTLYEG
AETDPLHYEGPGMEIPLPAPEEGETNFVLHLKQRFSGLAKCLCRMLSNEFVAPVTVPVQD
ILDFICRALDISMKNISWFGDGPLRMLFLHSVHLESLDLLFALILACGPRLVRFGGTLCR
LFPQVLTAWSAGRDLVPAGQERPYSAVRTRLYQVLDLWAQVAGAASGLLQGHGTQSEALL
GHLLSDISPPTDTLKLRESAPGPDRRPSAPKKPKLAGLGPLHHKHDPRANSDVCLAALGL
VRRSPRAPCLNPTQRLQELVIPLLIRLGQAEPLPGSPYASAACRRELFRLLLALVLAPAP
ACPPPLHCALWLLKQGCADPSLQVSSFCSEALVVCNALLHPRVPALQVPLPGPPAPPPGS
SSEISPATASPFRSAPPLPFPPPRLPPPASPPAPPSAAPPAGSLGLPLPPLALAQLPPRL
APEEPAVLPSPGGAAEAAALAAGAKLRRSVFIHYDKEEEEDVEISLESDSDDSVVIMPKG
QLGRGPGGAGAVAAVAAAPAPLPPPWVLAGWASLSTPGPVAALEHPPPGPRLRPPASPPL
CAPLLVHLGSWGWGGSRCLRLSDQEEEFEEDEGEFDELEEEIEEMDEEEEEEDEFDEEEC
MTEEEEEEEEEEEEEEEEEGLTEEEEEGAPPLPTLPHGNGGEPPALMPMKEELPKLVGEEE
GGEGEEEEEGAGLLMEVEEEAFHAPEDGSGGQEGEEEAAAGTREVGRTAGDALLRKAEEP
PVPTSAVEELLPPPLPLPPSPAQEPPTLPATAFTDPEELAEEQERPMAGGPPFAAAPQDP
EGERGPAGHAGGPAEAGPEARAPQGEAEEQLPALGGHVPEEEPEVRAWGGQAGGRAGSPA
TPGCPMASAAAWRHWKPQLSCPVLGLPAPASGAPAALAEGESDPQPGPLGLGAGRRWAGG
PREGVS
>Nothobranchius furzeri [A0A1A8VH82]
MAASAWLHGSAAMRLTEGLVLLMKEQCPEFLPEVLANYREHGVLSSQSASDAAGLVGLCN
GKLSSSKTRLEGLCLLSMLVKDSSSDLFQQHCLSWLRSLQQVVQSQAPVQTIQLAVNVLK
DLLQYSCQLPELAREVGLNSILGILTSLLGLKTECELSAMEGMKACMVYYPRACGSLKDK
LGAYFLSKMDSTSRKTQEAACQGYSHLPCLGGLMDRAVGASRAEGWTNQIHCLLASANSL
LAQIYQGSETDEAVRYQGPGVELAFPHIDQTEPLLLLQHRYAAVCMTIKHTLRTDPASAV
SVPVRPILNLVCRALAVNSKNMILTADGSLRFLVLPSVHINALEVLSELITVVRSGMVQY
AAVIQRLFSQTLSAWTPPPETSVGQQRAYSSVRVSVFRSLELWVRVAGASASILHGTSSH
VDLLLNHLLGDISPGAESVKLRAGLSADVVPVGKPGPRRTKQLVVADAVGPSLQRKGDLL
SNQDTCLAALKALRQVLQSTGMLLKDDIHKRLHEVVLPLCVRLQQQQSSSISAWEPAGGV
SGQYSSALTRRELYRVLLALVLVPSPSWPPPLTCAVSILSNGRIDRHLKVSSFCAEALTI
CNSILHPRRPSLALPLPPLTLKPTATPSVLSCQGPGPRLTLPTLLGGPSSAPPFPARHTL

NLGPSSLLGSLDNHFSLVPGLPGQAPGPGDLMASSQTHHQPDPSGLGPPEGQRPVFVRYD
KEETEDVEISLASDSDDSVVIVPPGLLNVEKQQNDVAANSQSLLSSAPGGAELVTMVPTS
STPTTLDVTSLPNDLATSSALLTSSVTPINSFPPSSTSVVSLVPSVNSSSASAPPGGLGD
PLAGKPQLQQMLMQPSTTGQPSSMALPLQMHQLTQQGRHLHPHPPTPAANEESAVININS
TDDEEDDEEDMEDDEELEDEEDGIDEEDDEEEGSDEFYEGEDYDDFDEEEGEELGEEEEE
EE
>Platysternon megacephalum [A0A4D9DQ02]
MAAAAAAAGSGCPGRLVLEALGGARPLPGLVRGLRDSGQNLQALGGLIGVTNARLGAVKT
RFEGLCLLSLLLSESPTEPFQQHCLAWLRALQHLLQSQDPAPTMALGAAVLRDLLLYSSQ
LPELARDIATNHIPGLLTSLLALKPECQLSALEGIKACMTCYPRACGSLRGKLAAYFLSR
VDADSPQLQQLACECYALLPSLGAGFTQGLKHTECWEQELHGLLATLHGLLGNLYDGAET
DPLPYEGPGVELLLPPAPPDGETSFVLNLCNRFSGLAKCLQLMLSSEFVAPVTVPVQDVL
DLVCRALNISIKNISWFGDGPLKMLLLPSVHLETLDVLAALILACGARLARWGSVLGRLF
PQVLSAWSSSRDSVPPGQEKPYSAVRTRLYQVLELWVQVGGAGAGVLQGPPHRSEALLAH
LLSDITPPTDSLKAGRPGSEGKPSAPKKPKLSEGGEAPSLHRKQDPMANSDVCKAALQAL
SRAILLGGSLVKEDTHRRLQELVLPLLLRLAQGDVPPGGPYASPACRHQLYRLLLALLLA
PAPACPPPLHCALRAFALGQRDPSLQVSSFCAEALVTCSALARPRVPSLQLPLPGPAPSA
GPAPSDLAASPFRQAPPFPAPPPARPPPANHLGLAPPRLPPSAAPGLALAEEPGEAGDPR
LLAPPSPGGAEEGGAGLGGKPRRPVYVHYEKEEESDVEISLESDSDDSVVIVPKGLLPKP
PPPPPPPPAPPSPPPAQPPEEPPPAPLLPAAGEALPGLGEENPAVININSSEEEEEEFPE
EEEDEEEEEEEEEEEEEYFDEEDEDFDEDEEEEEFEEEEELGEEEEEEEEEEGALSEEER
APPSPQEPLAAPGEPDDLIMEVEESQAPEPPPPEELEEEEEEEEVVVMKASPPPAPQPPR
EQEEEEEELPPAPPTPPQQASPLQAPPPDQEEMGEEPPLLEAPPPVLEEEQPPKEEGAPP
AGEQGDGAGEGAQPSPEREQPAVKAEAEADETETMLADFIDCPPDDEKGPPEPGP
>Orycteropus afer afer[A0A8B7AS70]
MAAAVLSGPSAGSAAGVPGGTGGFSAMGSGPRLRLLLLESVSGLLQPRTGSAVPSVHPPV
RPVPHLPGLMCLLQLHGTVGGAQNLSALGALVGLSNARLGSIKTRFEGLCLLSLLVGESP
TEMFQQHCVSWLRSIQQVLQSQDPAPTMELAVAILKDLLRYAAQLPTLFRDISMNHLPGL
LTSLLGLRPECELSALEGMKACMIYFPRACGSLKGKLAAFFLSRVEALSPHLQQLACQCY
SRLPSLGAGFSQGLKHTESWEQELHCLLASLHSLLGTLYEGAETAPVQYEGPGVEMLLPP
SEDGDASALLQLRQRFSGLAQCLGLMLSSEFGAPVSVPVQEILDLICRTLSVSGKNISLL
GDGPLRLLLLPSIHLEALDLLSALILACGSRLVRFGGLISRLLPQVLNAWSMGRDTLSPG
QERPYSTVRAKAYAVLELWVKMCGASAGVLQGGASAEALLTHLLSDISPPADALKLRSPR
GSPDGGLQTGKPSAPKKLKLDVGEAVAPPSHRKGDSNANSDVCAAALRGLSRTILMCGPL
IKEETHRRLHDLVLPLVMSVQQGEVLGSSPYTSSHCRRELYRLLLALLLAPSPRCPPPLS
CALQAFSLGQREDSLEVSSFCSEALVTCAALTHPRVPPLQSMGSTCPTSTPVPPPEAPSP
FRAPPFHPPGSMPSAGSMPSAGPMPSVGPMPSARPGPPATANHLGLSVPGLVSVPPRLLP
GSENHRAGSSEDSVLAPSGSPPPAIPSDETFGGRVPRPAFVHYDKEEASDVEISLESDSD
DSVVIVPEGLPPLAPPPPSGTTPPPVAPAGPPTASPPVPAKEEPEELPAAPGPLPPPPPL
PVPGPVTLPPPQLVPEGTPGGGGPTALEEDLTVININSSDEEEEEEEEEEEEEEEEEEEE
EEDFEEEEEDEEEYFEEEEDEEEEFEEEFEEEEGELEEEEDEEEEEEEEEELEEVEELEFG
SAGGEVEEGGPPPPTLPPALPASESPKAQPEPEPEPGLLLEVEEPGPEEEPGAETAPTLA
PEVLPAQGEVEREGGSPPAGSPPQALVEEEPSAPPTLLEEGIEDGGDKVPPPPETSAEEE
METETEAAALQEKEQDDTAAMLADFIDCPPDDEKPPPVTEPDS
>Castor canadensis [A0A8B7VYG4]
MAAAVLSGPSAGSSAGVPGGTGGLSGMGSGPRLRLLLLESVSGLLQPRTGSPVAPVHPPV
HWAPHLPGLMCLLRLHGTVGGAQNLSALGTLVSLSNAHLGSIKTRFEGLCLLSLLVGESP
TELFQQHCVSWLRSIQQVLQSQDPPPTMELAVAVLKDLLRYAAQLPMLFRDISTNHLPGL
LTSLLGLRPECEHSALEGMKACMIYFPRACGSLKGKLASFFLSRVDALSPQLQQLACECY
SRLPSLGAGFSQGLKHTENWEQELHSLLTSLHSLLGALYEGAETAPLQSEGPGVETLLSH
SEDGDTHALLRLRQRFSGLAHCLGLLLSSEFGAPVSVPVQDVLDLICRTLSVSGKNISLL
GDGPLRLLLLPSIHLEALDLLSSLILACGGRLLRFGALISRLLPQVLNAWSIGRDTLSPG
QERPYSTIRTKVYAILELWVKVCGASSGMLQGGASGEALLTHLLSDISPPADALKLRSPR
VSSDAGLQTGKPSAPKKLKLDMGEAMAPPSHRKGDSNANSDVCAAALRGLSRTILMCGPL
IKEETHRRLHDLVLPLVMGVQQGEVLGSSPYNSSCCRRELYRLLLALLLAPSPRFPPPLA
CALQAFSFGQREDSLEVSSFCSEALVTCAALTHPXVPPLQTMGPTCPTPAPVPPPEAPSP
FRAPSFHPPGPMPSLGPMPSPGPIPSAGPLPSAGPMPSVGPMSATGPMPSTGPMPSVGPM
PSTRPGPPATANHLGLSVPGLVSVPPRLLPGPENHRAASNEDPVLAPNGTPPPTITPDET
FGGRVPRPAFVHYDKEEASDVEISLESDSDDSVVIVPEGLPPLPPPPPTGTPPPPVAPTG
PPTASPPMPAKEEPEELPATPGPLPPPPPPPPPVPGPVTLPPPQLVPEGNPGGGGPPALE
EDLTVININSSDEEEEEEEEEEEEEEEEEEEEEEEDFEEEEEDEEEYFEEEEEEEEEFEEE
FEEEEGELEEEEEEEEDEEEEEEELEEVEDLEFGSAGGEVEEGGPPPPTLPPAVPPPESPKV
QPEPEPEPGLLLEVEEPGAEDEPTAETAPTLAPEVLPSQGEVEREGGSPTAGSPPQELVE
EPSAPPTLLEEGTEGGGDKVPPLPETSAEEMETEAQAEVEAVAVQEKEQDDTAAMLADFI
DCPPDDEKPPPTTEPDS
>Balaenoptera musculus [A0A8B8W8X6]
MFQQHCVSWLRSIQQVLQSQDPPPTMELAVAVLRDLLRYAAQLPTLFRDISMNHLPGLLT
SLLGLRPESELSALEGMKACMTYFPRACGSLKGKLASFFLSRVDALSPQLQQLACECYSR
LPSLGAGFSQGLKHTDSWEHELHSLLASLHSLLGALYEGAETAPVQYEGPGVETLLSPSE
DGDAHVLLRLWQRFSGLARCLGLMLSSEFGAPVSVPVQEILDLICRTLSVSAKNISLLGD
GPLRLLLLPSLHLEALDLLSALVLACGGRLLRFGALINRLLPQVLNAWSIGRDSLSPGQE
RPYSTMRTKVYAVLELWVQVCGASAGVLQGGASGEALLTHLLSDISPPADALKLRSPRGS
PDGGLQTGKPSAPKKLKLDMGEAMAPPSHRKGDSNANSDVCAAALRGLSRTILMCGPLIK
EETHRRLHDLVLPLVMGVQQGEALGSSPYTSSRCRRELYRLLLALLLAPSPRRPPPLACA
LQAFSLGQREGSLEVSTFCSEALVTCAALTHPRVPPLQTMGPACPTPAPVPPPEAPSPFR
APAFHPPGPMPSVGPMPSVGPMPSAGPMPSPGPVPPAGPMPSVGPMPPARPGPPATANHL

GLSVPGLVSVSPRLLPGPENHRAGSNEDAVLAPSGTPPPTIPPDETFGGRVPRPAFVHYD
KEEASDVEISLESDSDDSVVIVPEGLPPLPPPPPSGTTPPPAAPVGPPTASPPVPAKEEP
EELPAAPGPLPPPPPPPPVPGPVALPPPQLVPEGTPSGGGPPALEEDLTVININSSDEEEE
EEEEEEEEEEEEEEDFEEEEEDEEEYFEEEEEEEEEFEEEFEEEEGELEEEEEDEDEEEE
VEEVEFGPAGREVEEGGPAPPSLPPALPPAASPKVQPQPEPEPGLLLEVEEPGAEEGPGA
ETAPTLAPEVLPSQGEVEREGGSPPAVPPPQELVEEEPSVPPTLLEEGAEGGGDKVPPPP
EASAAEEMETEVEAAALQEKEQDDTAAMLADFIDCPPDDEKPPPATEPDA
>Bos mutus grunniens [A0A8B9YSD6]
MAAAVLSGPSAGSAAAVPGGPGSLSAVGSGPRLRLMLLESVSGLLQPRTGSAVAPVHPPA
RSAPHLPGLMCLLRLHGTVGGAQNLSAVGALVGLSNARLGSIKTRFEGLCLLSLLVGESP
TEMFQQHCVSWLRSIQQILQSQDPPPTMELAVTVLKDLLRYAAQLPAVFRDISMNHLPGL
LTSLLGLRPECELSALEGMKACMTHFPRACGSLKVSLACECYSRLPSLGAGFSQGLKHTD
SWEQELRSLLASLHSLLGGLYEGAEAAPMQYESPGAETLLSSSEDADAHTLLRLRQRFSG
LARCLGLMLSSEFGAPVSVPVQDILDLICRTLSVSAKNVSLLGDGPLRLLLLPSLHLEAL
DLLSALILACGARLLRFGALISRLLPQVLNAWSIGRENLGPGQERPYSTVRTKVYAVLEL
WVQVCGASAGVLQGGASGEALLSHLLSDISPPADALRLRSPRGSPDAGLQTGKPSAPKKL
KLDVGEAIAPPSHRKGDSNANSDVCAAALRGLSRTILMCGPLIKEETHRRLHELVLPLVM
GVQQGEALGSSPYTSSHCRRELYHLLLALLLAPSPRCPPPLACALRAFSLGQREDSLEVS
SFCSEALVTCAALTHPRVPPLQSVGPTCPAPAPVPPPEAPAPFRAPAFHAPSPLPSAGPM
PSAGPMPPVGPLPPTRPGPPATANHLGLSVPGLVSVPPRLLPGPENHRAGSSEDPVLAPS
GSPPPTIPPDETFGGRVPRPAFVHYDKEEPSDVEISLESDSDDSVVIVPEGLPPPPPPSS
GTTPPPVAPAGPPAASPPVPAKDEPEELPAAPGPLPPPPPPPVPGPVTLPPPQLVPEGTP
GGGGPPALEEDMTVININSSDEEEEEEEEEEEDEEEDEEEDFEEEEEEEEEEYFEEEEEE
EEEFEEEFEEEEGELEDEEDEEEDEELEELEEVEFGPAGGEVEGGGPAPPSLPPALPPAE
SPKGPPEPGLEPGLLLEVEEPGTEEAPGPETAPMLAPEVLPSQGEVEREGGSPPAGPPPQ
ELVEEEPSGPPALLEEGAEGGGDKVSPPPEASAVEETEVEAAALPPEKEQGDTAAMLADF
IDCPPDDEKPPPASEPDS
>Cyclopterus lumpus [A0A8C3A3V2]
MATSAWLRETSAMRLTEGLVSVLKEQRPEYLPALLASYREHGVFQTQSASAVGGLVGFSN
VKLGSGKTRSEGLCLLSMMVKDSSSELFQQHCNSWLRSLQHVIQSQSPDETIQLAVNTLK
DLLQYSSQLAQLAREVGLNSILGLLTSLLGLKKECELAAMDGIMACMTFYPRACGSLRGK
LGAYFLSKMDSTNKKTQEMACLCYSRLPCLGGLLDRGVGAGRAEGWTNQIHCLLASANGL
LAQIYQSAETDGTVQYEGPGVELAFPHLDQSDPLLLLQLQDRYTAVCLALKHTLRVDPAS
AVRLPVRTILNLVCRVLAVSSKSINLTGDGSVRLMVLPVIHTNTLEVLSALIAAVRGGMV
QYAAVLQRLFSQTLSAWTPQPEASLGQQRAYSSVRVSVYRTLELWVQVAGACASILQGSP
GHSELLFTHLLGDITPGAESVKLRAGLSADAVPGGKPGPRRTKHLVMADTAGPSLQRKGD
QLANQDSCLSALRALRQIILTSGTLLKDDIHKRLHDVVLPLCVRLKQQQSSSSILCDSAG
CVSGQYSSALPRRELYRLLLALVLVPSPCWPPPLTCAVSILSNGRNDRNLKVSTFCSEAL
TICNTLLHPRTPSIALPLPPLTLKPIPTASVLPSSQGPTTGLTLPTLLGGPAPGPPFPTR
HSLGLGPASLLGSLENHLSLVPGLPGQAPTPGDMILSHQQDPAGLGLPEGQRPVFVRYDR
EEAEDVEISLASDSDDSVVIVPPGMLNLENQQEETAAAGAQNMASVAPGGATVTVAGGDT
VAIVPTTATTTTIDRVSLPNDLATSSPLLTTSTTQINSFPPLSSSVVSLVPPLNSNTLAA
PPGGLGDSLPGRPQLQQMLMQPSAAGQSGPMGMPLQMHQLQNQLGQQGRHQHQHQPPP
ASNEDSAVININSTDEEEEEEEEEEDLEEDEDLDEEEEEEEEEEEEDVSDFPEDDLYDGE
YDNYDEEEGEELEEEEEEEEEEEEDGDIPPLEGVEDNDGEAGIEGGKVLRAVVDEEGLAG
FSVEGEAEGGIEEIQTNRSLFAEDRMKVQKVESIGVLEEAREGEEDESERMDDPTMPQIL
CVTGGTLEEREETDEEGGAAGGLQDGAKPWEQGANEIEPPAGAEDGTTNQVQQEPGAEPA
QEASVSDKQSPGHQEEQLADQEGDVAAGDPQTSRGPSPKETEDTTDAEKKESVTAEPQET
EGSAAGGGGGGGGGGGGGGGGGGGGGGGGGESDGEEGKGLKRKREDTQEEAGLGTEKKKV
DDEAMASMLADFVACPPDDEDGASGSNCS
>Chrysemys picta bellii [A0A8C3HGD7]
QALGGLIGVTNARLGAVKTRFEGLCLLSLLVSESPTEPFQQHCLGWLRALQHLLQSQDPA
PTMALGAAVLRDLLLYSCQLPELARDIATNHIPGLLTSLLALKPEVMPATPAEVPAPPCS
SPPPTFPVSDRTGLSGLAKCLQLLLSSEFVAPVTVPVQDILDLVCRALNISIKNISWFGD
GPLKMLLLPSVHLEILDVLAALILACGARLARWGSVLGRLFPQVLGAWSSSSDSVPPGQE
KPYSAVRTRLYQVLELWVQVGGAGAGVLQGPPHHSEALLAHLLNDIAPPTDSVKVSPPPQ
SPRDTLCTGKPSAPKKPKLSEGGEAPSLHRKQDPMANSDVCKAALQALSRAILLGGSLVK
EETHRRLQELVVPLLLRLAQGDVPPGGSYASPACRHQLYRLLLALLLAPAPTCPPPLHCA
LRAFALGQRDPSLQVSSFCAEALVTCSALARPRVPSLQLPLPAPTPSAGPAPSDLAASPF
RQAPPFPAPPPARPPSANHLGLAPPRLPPSTGPGLALAEEPGEAGDPRLLAPPSPGGAEE
GGAGLGGKSRRPVYVHYEKEEESDVEISLESDSDDSVVIVPKGLLPKPPPPPPPPPAPPS
PPPAQPPEEPPAAPLLPAAGEALPGLGEENPAVININSSEEEEEEFPEEEEDEEEEEEEE
EEEYFDEEDEDFEEEEEEEEEFEEEEELGEEEEEEEYEEEEEGLSEEEEEEEGALSEEER
VAPRPPGAAGRAQGAGRSHHGGRGEPGTRAAAPRGGGGGGGGDEG
>Gadus morhua [A0A8C5BVT1]
MATSAWLHVPTNMRLTECLESVLKEQRPHCLPALLANYREHGVVPTQGSSGVAGLVGCSN
AKLSTSKTKFDGLCMLSVLVKDSSTEVFQQHCLSWLRSLQQVIQAPAPGPTARLAVGVLA
DLLQYSSMLPELAREVGLTWLLGLLTSLLGLQAECELTALEGIAACMTHYPRACGSLRDK
LAAFFLARITSSDPATQEMACRCYGRLPCLGGALERGVGTSRAEAWANQLHCLLASANST
LAQIYQGAEAEGTVSYEGPGVELSFPALDEADPLLLLELQHRYRAACLALQHTLSVDPQC
AVVVPVRQLLNLVCRALSVGPKSLGVRGDDSMRLLVLPAVHSNTLTVLSALITAVRGGMV
QYAGVLQKVFSQTISAWTPASETSGGQQKAFSSVRVALYSTLGLWVQVGGASASLLQGSP
THSELLFSHLLGDVSPGAEGVKLRAGAGSLGELASLGGRPGPRRSKAQPAAPEGTPSLQR
KSDPLANQDACLMALKVLRRIIMTSGSLLKEEIHKRLHDLVIPLCVRLQQQLSSRCTGAS
EAAGGSSGQYASAQTRRELYRLLLALVLVPSARWPAPLSCTVSILSHGRRDRSLKVASFC
GEALTVCNGLLHPRAPTLSRPLRAPPGPAPLQPQGPASGLPLPAMLSPAFPPRHPLGPSA

LLGSLENHLSLGLPDHGPPGPEGPPEVLRPVFVRYDREEAEDVEISLASDSDDSVVIVPP
GMLGAESPPGLLAPPPPLDGAAPAPAAPEGGPPPADLASSLLAASDGAALNSFPPSAVAL
MAPAPPPGPGAAGEGLPGGAQLQQMLMLPPGPPGAAGHQLAPQNRHLHGPAHPLGPGPKD
SAVININSSDEEEEEEEEEEEDELGEEEEEDEDGMEEEEEGSDFQDEEPYDEEYDDYEDEE
AEELEEEQDGDMPPLEGKVEEEEGAETSGVEGEMGGIEEVQTNRAAFGAERGERLQEVES
IGVMEEAREALEAREALEEEGGDPTMPQILCVTGGSLEEQEVGPPEEEPARAAPEEEEQW
RPAPEEVSPPPGEEPAGGEAGPPGPPSMEGPGVCAEPAPGQPEAPPPGSPPSSTLAPSGP
PQEGDGAANLQTTGAEDESPEDVKGLKRKREEEDLQGADKRKVEEGGVASMLADFIACPP
DEEEGGSSSSLPCLD
>Gadus morhua [A0A8C5CHW6]
MATSAWLHVPTNMRLTECLESVLKEQRPHCLPALLANYREHGVVPTQGSSGVAGLVGCSN
AKLSTSKTKFDGLCMLSVLVKDSSTEVFQQHCLSWLRSLQQVIQAPAPGPTARLAVGVLA
DLLQYSSMLPELAREVGLTWLLGLLTSLLGLQAECELTALEGIAACMTHYPRACGSLRDK
LAAFFLARITSSDPATQEMACRCYGRLPCLGGALERGVGTSRAEAWANQLHCLLASANST
LAQIYQGAEAEGTVSYEGPGVELSFPALDEADPLLLLELQHRYRAACLALQHTLSVDPQC
AVVVPVRQLLNLVCRALSVGPKSLVSNTPGGGGGVTGLHSFITLASLPHTSHSCSRVRGG
MVQYAGVLQKVFSQTISAWTPASETSGGQQKAFSSVRVALYSTLGLWVQVGGASASLLQG
SPTHSELLFSHLLGDVSPGAEGVKLRAGAGSLGELASLGGRPGPRRSKAQPAAPEGTPSL
QRKSDPLANQDACLMALKGLASYVNMLLPVFQRLHDLVIPLCVRLQQQLSSRCTGASEAA
GGSSGQYASAQTRRELYRLLLALVLVPSARWPAPLSCTVSILSHGRRDRSLKVASFCGEA
LTVCNGLLHPRAPTLSRPLRAPPGPAPLQPQGPASGLPLPAMLSPAFPPRHPLGPSALLG
SLENHLSLGLPDHGPPGPEGPPEVLRPVFVRYDREEAEDVEISLASDSDDSVVIVPPGML
GAESPPGLLAPPPPLDGAAPAPAAPEGGPPPADLASSLLAASDGAALNSFPPSAVALMAP
APPPGPGAAGEGLPGGAQLQQMLMLPPGPPGAAGHQLAPQNRHLHGPAHPLGPGPKDSAV
ININSSDEEEEEEEEEEEDELGEEEEEDEDGMEEEEEGSDFQDEEPYDEEYDDYEDEEAEE
LEEEQDGDMPPLEGKVEEEEGAETSGVEGEMGGIEEVQTNRAAFGAERGERLQEVESIGV
MEEAREALEAREALEEEGGDPTMPQILCVTGGSLEEQEVGPPEEEPARAAPEEEEQWRPA
PEEVSPPPGEEPAGGEAGPPVSHAP
>Jaculus jaculus [A0A8C5KHA8]
MAAAVLSGPSAGSPAGVPGGAGGLSAAGSGQRLRLLLLESVSGLLQPRTGSPVAPVHPPL
RWAPHLPGLVCLLRLHGSVGGAQNLSALGALVSLSNARLGSVKTRFEGLCLLSLLVGESP
TELFQQHCVSWLRSIQQVLQSQDPPPTMELAVAILRDLLRYASQLPTLFRDISTNHLPGL
LTSLLGLRPECDQSALEGMKACVTYFPRACGSLKGKLASFFLSRVDALSPQLQQLACECY
SRLPSLGAGFSQGLKHTENWEQELHSLLASLHTLLGALYEGAETASVQNEGPGVETLLSH
SEDGDTHILLRLRQKFSGLARCLGLMLSSEFVAPVSMPVQEILDLICRILSVSGKNISLL
GDGPLRLLLLPSIHIEALDLLSALILACGGRLLRFGTLISRLLPQVLNAWSTGRDTLSPG
QERPYSTIRTKVYSILELWVQVCGASAGVLQGGASGEALLTHLLSDISPPADALKLHSPH
GSSDGGLQSGKPSAPKKLKLDMGEAMAPPSHRKGDINANCDVCAAALRGLSRTVLMCGPL
IKEETHRRLHDLVLPLVMSIQQQGEVLGSSPYNSSCCRRELYRLLLALLLAPSPRCPPPL
ACALQAFSLGQREDSLEVSSFCSEALVTCSALTHPRVLPLQTMGTACPTPAPVPPPEAPS
SFRAPPFHPPGPMPSIGPIASSGPVPSAGPMPSASPMPSTANHLGLSVPGLVSVPTRLLP
GPENHRASSNEDPVLAPSGTPPPTVPSDETFGGRVPRPAFVHYDKEEVSDVEISLESDSD
DSVVIVPEGLPPLPPPPPSGSGTPPPPPVASTAPPTASPPVPAKEDSEELPATPGPLPPP
PPPPHPGPGAVTLPPPQLVPEGAPAGGGPPALEEDLTVININSSDEEEEEEEEEEEEEE
EEEEDFEEEEEDEEEYFEEEEEEEEFEEEFEDEEGELEEEEEEEEEEEEEEELEEVEDVEF
GSAEGEIEEGGPPPPTLPPALPPPDSPKVQPEPEPEPGLLLEVEEPGAEKGSGAEMAPNL
APEVVPSQEEVEKEGGSPTAGSPPQELVEESSVPPTLVEEGTEGGGDEVPPPPETPADEE
METEAETAALPTKEQDDTAAMLADFIDCPPDDEKPPPTTEPDS
>Neovison vison [A0A8C7BLY7]
MAAAVLSGPSAGSAAGVPGGTGGLSAVGSGPRLRLLLLESVSGLLQPRAGGAVAPVHPPV
RSAPHLPGLMCLLRLHGTVGGAQNLSAVGALVGLSNARLGSIKTRFEGLCLLSLLVGESP
TEMFQQHCVSWLRSIQQVLQSQDPPPTMELAVAVLRDLLRYAAQLPTLFRDISMNHLPGL
LTSLLGLRPECELSALEGMKACMTYFPRACGSLKGKLASFFLSRVDALSPQLQQLACECY
SKLPSLGAGFSQGLKHTESWEQELRSLLASLHSLLGALYEGAETAPMQYEGPGVETLLTP
SEDGDAHVLLRLRQRFCGLARCLGLMLSSEFGAPVSVPVQEILDVICRALSISAKSISLL
GDGPLRLLLLPSIHLDALDLLSALILACGGRLLRFGALISRLLPQVLNAWNLGRDTLSPG
QERPYSTVRAKVYAVLELWVQVCGASAGVLQGGASGEALLSHLLSDISPPADTLKLRSPR
GSLDGGLQSGKPSAPKKLKLDMGEATAPPSHRKGDTNANSDVCAAALRGLSRTVLMCGPL
IKEETHRRLHELVLPLVMGVQQGEVLGSSPYTSSRCRRELYRLLLALLLAPAPRCPPPLS
CAVQAFSLGQREDSLEVASFCSEALVTCAALTHPRVPPLQSMGPACPTPAPVPTPEAPSP
FRAPPFHPPGPMPSVGPMPSVGPMPSAGAMPPAGPMPPTRPGPPATANHLGLSVPGLVSV
PPRLLPGPENHRAGSNEDPVLAPSGTPPPIPPDETFGGRVPRPAFVHYDKEEASDVEIS
LESDSDDSVVIVPEGLPPLPPLPPSGSTPPPVAPAGPPTASPPGLAKEEPEELPAAPGPL
PPPPPPPVPGPVALPPPQLVPEGTPGSGGPPGLEEDLTVININSSDEEEEEEEEEEEEEE
EEEEDFEEEEEDYFEEEEEEEEEFEEEFEEEGELEEEEEEEDEEEEEELEELEEVEFG
PAGAPAEESGPPPPSPPPALPPAQSPKVQPEPEGEPGLLLEVEEPGAEEGPGAEAAPTLV
PEVLPSEGEGPRDTESPPAGPPPQELVEEEPSAPPTLLEEGTESGGDEVPVPPETPAVAD
VEAEAEQDDTAAMLADFIDCPPDDEKPPPAPEPDS
>Oncorhynchus mykiss [A0A8C7LS98]
MAATAAWMHGPANMRLTEGLLSVLKEQRPEYLPALLANYREHGVVSTQSSAAVGGLVGLS
NAKLGNSKTRFEGLCLLSVLVKDSSSDVFQQHCLSWLRSLQQVIQSQAPLPSIQLAVGVL
QDLLQYSSQLPELAREVGLNSILGILTSLLGLKSEFHLAAMEGMTACMTFYPRACGSLRD
KLGACFLSKMDSVIPEVQEVACECYGRLPCLGGVLERGGGGRRAEGWTNQLHCLLASANG
MLAQLYQSTESEGMVPYEGPGVELPYPPLDDTDPLVLLQLQHRYRGVCLALKHTLGVDPA
SAVRLPVQQVLNLVCRALAVSSKSINVTGDGSVRLLVLPSIHNHTVKVLHALITAVGSGL
VQYSSMLQRLFSQTLSAWTPLPETNLGQQRAFSAVRVSLYRTLELWVKVGGASAGVLQGS
PTHSEILLAHLLGDITPGADSVRVIRSDWFIGMAKPGLGMGNGGGASLQRKGDSLANQDT

CVSALKGEGAGRLHDVVLPLCVRLQQQHGGDCGAGGVSGQYGSALPRRELYRLLLALVLV
PPPRWPPPLTCTVSILSHGRRDRSLKVSSFCTEALTICNSLLHPRTPSLCLPLPPLTLKP
SPATSLLTPSQASSLTLPTLLGGPFPGRHSLGLGHTLLGSLDNHLSLVPPGLSGQGSTPG
DLLLSPHQGELAGLGLSEGQRPVFIRYDKEEAEDVEISLESDSDDSVVIFPRGMLMLENQ
DGISTVATLPVSSLVPGGVTLPIPGPGDDPGGDISLPLANDLPSTSLPHPLLPSSSAPNS
INSFPPAPLASLVPPLNSTGVTQLGAPSVGLGVGADSIPGAQLQQMLLQGQSPVPGQPTP
LGLPIQMLQNQLAQPSRALQQQQASEEDHSVININSSDEEEEEDEEMEDEDELGEEEDEE
GLEDEEEEEGSDFPDEEEEEEFYGEEFDDYEEEEGEEEEEEDEEEEEGEIRPLDREGRRG
GMGREEGEVLREVPEDEGMGGFCVEGEMEGGIEELGTNRRVYGEEGVKAQEVESIGVLEE
EREGEEDDADGMNDPTMPQILCVTGGALEEREELGEGHGQEVVSCEQQGADRPEAAPSSE
GPTPQHKQEAEPAQEVRVGGSDQPSNQGEEPAKQGGESAKEEVKPSAAPSETEGEEPERE
KGGEEGEEDGEEDGRGMKRKREGEEEGTGQGTEKKKVRTLSQTIFNHSHSKPFS
>Pelusios castaneus [A0A8C8S3X1]
PGAAGAGGAGRGGPGPARPGSGPAPKSRGPAFSILSGSGLAPPARGSTAPPLGPGGPLFS
APWAPGLTPPTRVSAALTPGLAPPTRSSTAPPFEGLCLLSLLVSESPTEPFQQHCLAWLR
SLQHLLQSQDPAPTMALGASVLQDLLQYSCQLPELARDIGTNHIPGLLTSLLALKAECQL
SALEGIKACMTFYPRACGSLRGKLAAYFLSRVDADSPQLQQLACECYALLPSLGAGFTQG
LKHRECWEQQLHGLLATLHGLLGNLFEGAETDPLPYEGPGVELLLPAPPDGESGFVLNLH
NRFSGLAKCLQLMLSNEFVAPVMVPVQDVLDLICRALNISSKNFSWFGDGPLRMLLLPSV
HLQILDVLSALILACGARLARWGSLLARLFPQVLATWSGSRESVLPGQEKPYSAVRTRLY
QVLELWVQVGGAGAGVLQGPPHRSEALLGHLLSDITPPTDSVKLKVGRSASEGKPSAPKK
PKLSEGGDAPFLYRKQDPMANSDVCKAALQGLGPPALTASRLQELVVPLLLRLAQGEVAA
GSPYAHPACRLHLYRLLLALLLAPAPTCPPPLHCALRAFTLGQQDPSLQVSSFCTEALVI
CNALARPRVPSLQLPLPSPAPSTGPAPPELAAASPFRQALPPVRPPPANHLGLAPPRLPP
AAAMPGLAPAEECMEGSESRLPASPSPGGAEEGGPGPGGKARRPVYVHYEKEEESDVEIS
LESDSDDSVVIVPKGLLPRPPPPPPPPPPPAPPSPPPTEPPEEAPPPPTSEVAPGLGEEE
PAVININSSEEEEEEEEEDFAEEEEEEDEEEEEEEEEFFDEEEDFEEEEFEEEEEEEEYE
EEEGLSEEEGLSEEEGALSEEERPASGPEEVPPMLGEPEDLIMEVEESQAPEPPAPGEEE
EEEEVVVMKTSPPPVPQLPREEEEEPPPAPPTPPRRVSPLQAPSPGQEEMGVEPPLLEATP
PAPEEPPPEEKEEGASPAGEQGAGTGEEPHAGALSEEQGEAVKGEVRGGEPAGGAREALG
VAVGRDLGDTAPHRGAGWMWAWGRCGAGGTGSFVGIWPLR
>Panthera leo [A0A8C8XFV5]
MAAAVLSGPSAGSAAGVPGGTGGLSAVGSGPRLRLLLLESVSGLLQPRAGSAVAPVHPPV
RSAAHLPGLMCLLRLHGTVGGAQNLSAVGALVGLSNARLGSVKTRFEGLCLLSLLVGESP
TELFQQHCVSWLRSIQQVLQSQDPPPTMELAVAVLRDLLRYAAQLPTLFRDISTNHLPGL
LTSLLGLRPECELSAMEGMKACMTYFPRACGSLKGKLASFFLSRVDALSPQLQQLACECY
ARLPSLGAGFSQGLKHTESWEQELHSLLASLHGLLGALYEGADTAPVQCEGPGLDVLLAP
SEDGDAHTLLRLRHRFSGLARCLGLLLSSEFGAPVSVPVQEILDIICRTLSISAKNISLL
GDGPLRLLLLPSIHLDALDLLSALILACGSRLLRFGALISRLLPQVLNAWNLGRDALPPG
QERPYSAVRTKVYAVLDLWVQVCGASAGVLQGGASGEALLSHLLSDISPPADALKLRSPR
GSPDGGLQSGKPSAPKKLKLDVGEATAPPGHRKGDSNANSDVCAAALRGLSRTVLMCGPL
IKEETHRRLHDLVLPLVMGVQQGEVLGSSPYTSSRCRRELYRLLLALLLAPSPRCPPPLA
CALQAFSLGQREDSLEVSSFCSEALVTCAALTHPRVPPLQSMGPACPAPAPAPPPEAPSP
FRAPPFHPPGPMPSVGPMPSVGPMPSVGPMPPTRPGPPATANHLGLSVPGLVSVPPRLLP
GPENHRAGSNDDPVLAPSGTPPPAVPPDETFGGRVPRPAFVHYDKEEASDVEISLESDSD
DSVVIVPEGLPPLPPPPPPTGTTPPPAAPAGPPTASPPAPAKEEPEELPAAPGPLPPPPP
PVPGPVALPPPQLVPEGPPGGGGPPALEEDLTVININSSDEEEEEEEEEEEEEEEEEEEE
EDFEEEEEEEEYFEEEEEEEEEEFEEEFEEEEGELEEEEEDEDEDEEDELEELEEVEFGP
AGGPAEEGGPPPPSPAPALPPAQPPEAPPEPGVEPGLLLEVEEPGPEDEPGAEAAPTLAP
EVLPSQGEGQREAGSPPAGPPPQELVEEEPSAPPPLLEEGTENGGDKVPPPPETPAAEEM
EAAAEAEEQDDTAAMLADFIDCPPDDEKPPAAPEPES
>Salvator merianae [A0A8D0AZP9]
MPGRGGGTWCFVRTRSVQESGRRACSDGGGGGASVVLLVVPGGGVLLAAGAREPGRAAAR
RTCRGAPASSREPCGHPPGPGRSGAMRLGGGRGGRRRGRQGEGAPRRGGARRAVETPPAF
TLSLPPQVSSLLGSLASLSALRLGSMKTRFEGLCLLSLLVTESNAETFSQHCLSWLRSLQ
HLIQSQDPPPTMELAMLILRDLLEYSCQFPELSRDISTNHIPGLLTSLLALKPECQLSAL
EGIKTCMILYPRACGSLRVTGPSSFCQLACECYALVPSLGAGFTKGLKYTECWEQQAHGL
LATLHSLLGTLYEGAETDPLLYEGPGXEVPLPAPEDEDTNLILHLQHRFTGLMKCLCRML
SNEFVAPVTVPVQDVLDLVCRVLATSMKNFTWFGDGPLRMLLLPTLHLEALDLLAALILA
CGPRLVRFGGSLGRLFPQVLTAWSLGRELFLPGQKRPYSVVRTRXYEVLELWIKVAGAAS
GVLQGPEAQSEALLGHLISDISPPSDTLKLHESHPGPDSKPSAPKRPKLSDPGSLGSLHR
KHDPQANSNVCLAALRGLSRAVLLAGSLMKEQAHKRLQELVIPVLIRLGQAEPLPGSPYA
SASCRQELYRLVLSLVLAPPPSCPPPLQCALRLLSHGRRDPNLQVSAFCAEALVVCNALL
HPRVPSWQLPLAGSSVPLPGTSEISPAVNSPFRSAPPLSFSAPRLLPPSVSPSAPPSVTP
SANSLGLPLPGLASAQLPPRLTPEEPTLPPSPGTAEAAALAAGAKLRRSVFIHYDKEEEE
DVEISLESDSDDSVVIVPKGQLGKAPSASSPAVAAMAPAVPAPLPPPPPPPLPASPPPPS
EETPLEPVVPVALGGAPPPAPPALVLPPSPAPLASDVPFPALVEEDPAVININSSEEEEE
EEEEEEEEEEDFPEDEDYFEEEEEVGLCVARAGHPLPRLMGLRICQGRXLAGGRQGAQGL
QGTSSPGGRSARRLGHIWQHLVLPLWPLLGAEAGRHEGLPAGGCSGRWAALCPSPFRAGG
HVGLFLFSPRQAPAVDETEAMLADFVDCPPDEEKVPPAVEPSA
>Theropithecus gelada [A0A8D2FMY6]
MLLPSLSPRVQPAFIQCAGPQPPPHAHSPVGGRRGVFASSRHATTGARGKMAAAVLSGPS
AGSAAGVPGGTGGLSAVNSGPRLRLLLLESVSGLLQPRTGSAVAPVHPPNRSAPHLPGLM
CLLRLHGSVGGAQTQDPPATMELAVAVLRDLLRYAAQLPALFRDISMNHLPGLLTSLLGL
RPECEQSALEGMKACMTYFPRACGSLKGKLASFFLSRVDALSPQLQQLACECYSRLPSLG
AGFSQGLKHTESWEQELHSLLASLHTLLGALYEGAETAPVQNEGPGVEMLLSSEDGDAHV

LLRLRQRFSGLARCLGLMLSSEFGAPVSVPVQEILDFICRTLSVSSKNISLHGDGPLRLL
LLPSIHLEALDLLSALILACGSRLLRFGILISRLLPQVLNSWSIGRDSLSPGQERPYSTV
RTKVYAVLELWVQVCGASAGMLQGGASGEALLTHLLSDISPPADALKLRSPRGSPDGSLQ
TGKPSAPKKLKLDVGEAMAPPSHRKGDSNANSDVCAAALKGLSRTILMCGPLIKEETHRR
LHDLVLPLVMGVQQGEVLGSSPYTSSRCRRELYCLLLALLLAPSPRCPPPLACALQAFSL
GQREDSLEVSSFCSEALVTCAALTHPRVPPLQPMGPTCPTPAPVPPPEAPSPFRAPPFHP
PGPMPSVGPMPSAGPMPSAGPMPSAGPVPSARPGPPTTANHLGLSVSGLVSVPPRLLPGP
ENHRAGSNEDPILAPSGTPPPAIPPDETFGGRVPRPAFVHYDKEEASDVEISLESDSDDS
VVIVPEGLPPLPPPPPSGATPPPIAPTGPPTASPPVPAKEEPEELPAAPGPLPPPPPPPP
PVPGPVTLPPPQLVPEGTPGGGGPPALEEDLTVININSSDEEEEEEEEEEEEEEEEEEEE
EDFEEEEEDEEEYFEEEEEEEEEEFEEEFEEEEGELEEEEEEEEDEEEEEEELEEVEELEFGT
AGGEVEEGGPPPPTLPPALPPPESPPKVPPEPEPEPGLLLEVEEPGAEEEHGADTAPTLA
PEVLPSQGEVEREEGSPSAGPPPQELVEEEPSAPPTLLEEGTEDGGDRVQPPPETPAEEE
METETEAEALQEKEQDDTAAMLADFIDCPPDDEKPPPPTEPDS
>Iconisemion striatum [A0A1A7YBW4]
MAASAWLHGSAAMRLTEGLVLLLKEQRPEFLPEVLANFREHGVFSTQSASDGAGLVGFCN
AKLSSSKTRFEGLCLLSMLVKDSSSDLFQQHCLSWLRSLQQVIQSQAPVQTIQLAVNVLK
DLLQYSCQLPELAREVGLNSILGILTSLLGLKTECELSAMEGMKACMVYYPRACGSLKDK
LGAYFLSKMDSTNRKTQEIACQGYSHLPCLGGLLDRAVGASRAEGWTNQIHCLLASANSL
LAQIYQGSETDEAVHYQGSGVELAFPHVDQTEPLLLLQHRYTAVCMTIKHTLRTDPSSAV
SVPVRPILNLVCRALAVNSKNMNLTADGSLRFLLLPSVHINALEVLSELITVVRSGMVQY
AAVIQRLFSQTLSAWTPLPETSVGQQRAYSSVRVSVYRSLELWVRVAGASASILHGTSGH
VDLLFNHLLADITPGAESVKLRAGLSADVVPVGKPGPRRTKQLVVADVVGPSLQRKGDLL
SNQDTCLFALRALRQVLQSTGTLLKDDIHKRLHEVVLPLCVRLQQQQSSSISAWEPAGGV
SGQYSSALTRRELYRVLLTLVLVPSPFWPPPLTCAVSILSNGRIDRNLKVSTFCAEALTI
CNSILHPRRPCLALPLPPLTLKPTPTPSVLSCHAPGPRLTLPTLLGGPSSGPPFPARHTL
NLGPSSLLGSLENHFSLVPGLPGQAPGPGDLMVSSQTHHQPDPSGLAPPEGQRPVFVRYD
KEETEDVEISLASDSDDSVVIVPPGMLNVDKQQNDLAANSQSLLSSAPGGVSLAGGESVT
MVPTSSTPTTLDVTSLPNDLATSSTLLTSSVTPINSFPPSSTSVVSLVPSVNSSSASAPP
SGLGDPLTGKPQLQQMLMQPSTTGQPSSMALPLQMHQLTQQGRHLHQHPPTPAANEESAV
ININSTDDEEEDEEDMEEDEELEDEEDGLDEEDEEEEGSDEFYEGEDYDDFEEDEGEELG
EEEEEEDGDMPPLEGSEDRSEEVGMEDDKVLIPMEEGEMGEFGVEGDAEGGIEELQTGRA
LFGEDRMKVQEVESIGVLEESREGEGEEDESEQMDDPTMPQILCVTGGALEERLESGEEG
IVSGDGLQEEIGLWDQRAKETELITTAEEQESSQSQLDMITENEPQAAERENEPLSQQEE
EEEPATGEGEPAGADEPPVASEDIKEQEQTDLDPGDPKEPETSQTGELEEGNGGGDENGG
EEEKGMKRKRENENIEEEESRPIPDKKKLDEDIMASMLADFVECPPDDEDVASGSNG
>Nothobranchius kuhntae [A0A1A8JK99]
MAASAWLHGSAAMRLTEGLVLLMKEQRPEFLPEVLANYREHGVLSSQSASDVAGLVGLCN
GKLNSSKTRLEGLCLLSMLVKDSSGDLFQQHCLSWLRSLQQVVQSQAPVQTIQLAVNVLK
DLLQYSCQLPELAREVGLNSILGILTSLLGLKTECELSAMEGMKACMVYYPRACGSLKDK
LGAYFLSKMDSTCRKTQEAACQGYSHLPCLGGLMDRAVGASRAEGWTNQIHCLLASANSL
LAQIYQGSETDEAVRYQGPGVELAFPHIDQTEPLLLLQHRYAAVCMTIKHTLRTDPASAV
SVPVRPILNLVCRALAVNSKNMILTADGSLRFLVLPSVHINALEVLSELITVVRSGMVQY
AAVIQRLFSQTLSAWTPLPETSVGQQRAYSSVRVSVFRSLELWVRVAGASASILHGTSSH
VDLLLNHLLGDITPGAESVKLRAGLSADVVPVGKPGPRRTKQLVVADAVGPSLQRKGDLL
SNQDTCLAALKALRQVLQSTGMLLKDDIHKRLHEVVLPLCVRLQQQQSSSISAWEPAGGV
SGQYSSALTRRELYRVLLALVLVPSPSWPPPLTCAVSILSNGRIDRHLKVSSFCAEALTI
CNSILHPRRPSLALPLPPLTLKPAATPSVLSCQGPGPRLTLPTLLGGPSSAPPFPARHTL
NLGPSSLLGSLDNHFSLVPGLPGQAPGPGDLMASSQTHHQPDPSGLAPPEGQRPVFVRYD
KEETEDVEISLASDSDDSVVIVPPGMLNVEKQQNDLAANSQSLLSSAPGGAELVTMVPTS
STPTTLDVTSLPNDLATSSALLTSSVTPINSFPPSSTSVVSLVPSVNSSSASAPPGGLGD
PLAGKPQLQQMLMQPSTTGQPSSMALPLQMHQLTQQGRHLHPHPPTPAANEESAVININS
TDDEEDDEEDMEDDEELEDEEDGIDEEDDEEEGSDEFYEGEDYDDFDEEEGEELGEEEEE
EDGDMPPLEGSEDRSEEVGMEDDKVLLPMEEGEVGEFCVEGDGEGGIEELQTGRALFGED
RMKVQEVESIGVLEESREEGEGEEDESEQMDDPTMPQILCVTGGALEERLESGEEGVVAG
DGLQEEMGLWDQGAKETELIIAAEEQEASQSQLDMVAENELQAAEGENKPTSQEEEEAAG
GGEPAGADEQPLVAEDIKEQQQTDLDQGDSKEPETSQTEGELGGGGNGCGGERGREEEKG
MKRKRENENTEEEESTQSPEKKKLDEEAVASMLADFVACPPDDEDVATGSNG
>Acanthaster planci [A0A8B7YKJ6]
MAQPTEAMCSMMLEVLRQKSNTSSPDTSINLLSSFIEDSTELQIFSQERNCASWIGHING
CLNSSKTRLEGLIVLRALVNQCPAEVFQQYCASWVRLLTQILQSYDPPPVIALASVALRN
ILKEAAQVSELSREISNTFITPIITAALELKTEWQDSAIQILAACIQSFPGPCGTFRTRT
ENFLIQVMGSSGSVDIAVSASQCLALLPRVGGGGQGGTKHAEAWSYYCHRVLATIKDLTD
KIYHDSQPEKQSDSSHPPFTMPDAPLKEPTRMYTLVRQFKVMSACLQHMLRAEFPEMVKI
PVKSILSLCVRILVPSMLFKGLKSALQTGNFSSVHSEAFNILAALLISCRGNLSLHKDII
NELFVKSHAFRPSVQSDDAKYRVLSGLKLSVYGALEIWLETSGSCSGIETGADKLLEAIL
NDARPQAQMTKLRVTTEKPTSMNAKKSGKSKQKGETAGGETTTTSLASQQKTSPPSSSRV
CLAALKVLRCLLTSIGTRVKPNFHKDVHEFVIPLLLHLQQLPPILIPAPYSNADCRRALY
HVLLSSVTAPHPRWPAPLQCTAGLFSRGRQDASINVSSFCAEASIICQSIIHPRVPCLQQ
PFRIAQLDPKRVPSQVLSKPQASSEVDRMEISDPNFGPASMDQNTGGIVWRSRDTTAGSV
EITQPAVLAATTEVEPSNKEKSEDSSDEDSDDQGIEEDDSRMEDNCQKNGDDQEQQEVIP
ESQEQSTVSQEALDTEDAQENASPALPVETNSVSDVILTSMGSTDVADPSKASEDRTTSP
SRRSKRQRTRAQKEDRSETEDEVFRSGSAVLGSVKRRKGEDKDEERSDDGSKDKDNSGGS
ESKGKVDVMLASFVDSLPDQD
>Exaiptasia diaphana [A0A913WZI7]
MAADIAFLVSSYLDGNKPTNLSIStLLEAVSETQSLQGSGCTKEWIGKLNSLLNSNNDRW

LGIALLGPTLQQCNTDVFTANCISWMKVLQHILELPTSSLSLIQTSVTILDDLMRYTTQF
PELMRNTSNSTIPAILSALLSLQGKDTVCSLVGICACLKYFPGPSSQFKTQIEDYLISCL
DRNSPNIRKYCAQCFALVAGLSNNQNDKQKKNWDLQWYRVVGTLNVTLDILHAYESTTSE
IKIIETLPVKTIETDEPKKTNTCTSRLRSLLKCLDAMIRQGSRRVSMVPLDITIALLERV
LSLNGKMLRGSISVDNVLLQGCLPLIHLDAVHLLCSLVQRCGSLLLPNANRIAKLMVNEL
SWMSDKHLNASQNGSIKPYSNLSCAVYDCILHWGQLNAGFHEDVIKDITRYLLKEIKVGK
NIETKLRAASAEGNKQKGLSKKGKKRKMQAEIEQLSTNPEKSDVYCNEKLCHAALTTLFS
VMMNFGDNMKKGTFQEIQLTLLEVVSLFQPSSQGVFPQPYNSPDCRKEIYHCLLGCVLSR
PSGAPNAISCVIKYLNDGLQDMSFQVSNYCREALTITTALIHPVVPSIEVXTKSLIFTNH
VVQEISAASHHEQIIVEASERLEEEKRQMLSDSIQDVQISQKTLDSNHNQRLQEDNSPFS
KKPQSVAQKTINEQTLQTNIRDSSIIEQNIENSSYQSSTESNKNSVIHVDCSNSMSSKQT
ETNEQKLHRTSQVSLGSELPGTSSLNIDLNCRDGMVDDNSGTIDEIIPVKRAKINPDVYH
SVGDKAKTASDTQMDKQQQEIQDTDDVSEMIASFVNSSPDSD

>Patiria miniata [A0A913ZFE7]

MARPMEDMCSVMLEIFKQESSPLSPDSSISLLTSFIEDTKELQIFGQERNCASWIGYING
CLNSSKTRLEGLSCLGALVSQCPAEIFQQHCVSWVRLLTQTLQSYDSPPIIALASGALRS
ILKEASQVSELSRELSNTHIAPIITAALELKTEWQESAVEILTACIQYFPGPSGTFRTRV
ENFLVQLMGSSNDIDIAVSAGQCLALLPRVGGGGQGGVKHTEAWSYYCQRALASIDGVVD
KIYGNSRPGKQTEPSQPPFPLPDAPLKEPTRTYTLIRQYKVLCACLQHMLRTDFPEIVKI
PVNRILSLCVSILVPNMLSKGLKSSLQSGNFSSVHIEAFNILAALISSCRGNLSLHRNVV
NELFVKSHAFRQGVQSEDTKHRVLSGLKLSAYAALEIWLETVGMNSGIETAAEKLLESIL
TDAQPQAQRTTLKVTSDKPSLTVKSSGKAKKKGETAGGEASTSLAGQQAAVPSPSGRVCL
AALKVLRCLQMSVGTRVKPDFHKDVHEFVVPLLLRLQQSPQGLAPAPYSSADCRKALYHV
LLSSVIAPHPRWSAPLQCAVGLFSRGRQDTSIKVSSFCAEASIICQSIIHPRVPCLQQPL
SLAQLDPKRAPSQSVSEARTRSDPDYMETTDLSVGLATPSQTAEGNSVGLDSGGGIAPSV
DKHIPIRSVGIAQPAVATAKMEVPSNGTEESEDSSDEDIEDQEITEDDPGIKEDNQIKGD
YQEIIQDRPEVSASKETTSRSDASVASTSSQLQDDSVLASTVSISGGKKVASSSASSDDS
KISSPTRRSKRQLARVQEEERRERENAGAREESGDADSGSKKRRREDEDEDETKEEEKNL
EGDVLDETDVMLASFVDSLPDKE

>Patiria miniata [A0A913ZGG4]

MARPMEDMCSVMLEIFKQESSPLSPDSSISLLTSFIEDTKELQIFGQERNCASWIGYING
CLNSSKTRLEGLSCLGALVSQCPAEIFQQHCVSWVRLLTQTLQSYDSPPIIALASGALRS
ILKEASQVSELSRELSNTHIAPIITAALELKTEWQESAVEILTACIQYFPGPSGTFRTRV
ENFLVQLMGSSNDIDIAVSAGQCLALLPRVGGGGQGGVKHTEAWSYYCQRALASIDGVVD
KIYGNSRPGKQTEPSQPPFPLPDAPLKEPTRTYTLIRQYKVLCACLQHMLRTDFPEIVKI
PVNRILSLCVSILVPNMLSKGLKSSLQSGNFSSVHIEAFNILAALISSCRGNLSLHRNVV
NELFVKSHAFRQGVQSEDTKHRVLSGLKLSAYAALEIWLETVGMNSGIETAAEKLLESIL
TDAQPQAQRTTLKVTSDKPSLTVKSSGKAKKKGETAGGEASTSLAGQQAAVPSPSGRVCL
AALKVLRCLQMSVGTRVKPDFHKDVHEFVVPLLLRLQQSPQGLAPAPYSSADCRKALYHV
LLSSVIAPHPRWSAPLQCAVGLFSRGRQDTSIKVSSFCAEASIICQSIIHPRVPCLQQPL
SLAQLDPKRAPSQSVSEARTRSDPDYMETTDLSVGLATPSQTAEGNSVGLDSGGGIAPSV
DKHIPIRSVGIAQPAVATAKMEVPSNGTEESEDSSDEDIEDQEITEDDPGIKEDNQIKGD
YQEIIQDRPEVSASKETTSRSDASVASTSSQLQDDSVLASTVSISGGKKVASSSASSDDS
KISSPTRRSKRQLARVQEEERRERENAGAREESGDADSGSKKRRREDEDEDETKEEKNLE
GDVLDETDVMLASFVDSLPDKE

>Rattus norvegicus [A6HG46]

MAAAVLSGPTTGSPAGAPGGPGGLSAAGSGPRLRLLLLESVSGLLQPRTGSHVAPVHPPI
QWAPYLPGLMCLLRLHGTAGGAQNLSALGALVNLSNAHLSSIKTRFEGLCLLSLLVGESP
TELFQQHCVSWLRSIQQVLQSQDSPPTMELAVAILRDLLRYASQLPTLFRDISTNHLPGL
LTSLLGLRPECEQSALEGMKACVTYFPRACGFLKGKLASFFLSRLDSLNPQLQQLACECY
SRLPSLGAGFSQGLKHTENWEQELHSLLTSLHSLLGSLFEETETAPVQSEGPGVEMLLSP
SEDDNTHVLLQLWQRFSGLARCLGLMLSSEFGAPVSVPVQEILDLICRILGISSKNINLL
GDGPLRLLLLPSLHLEALDLLSALILACGGRLLRFGALISRLLPQVLNTWSTGRDALAPG
QERPYSTIRTKVYAILELWVQVCGASAGMLQGGASGEALLTHLLSDISPPADALKLCSTR
GSSDGGLQSGKPSAPKKLKLDMGEALAPPSQRKGDRNANSDVCAAALRGLSRTILMCGPL
VKEETHRRLHDLVLPLVMSVQQGEVLGSSPYNSSCCRLELYRLLLALLLAPSPRCPPPLS
CALKAFSLGQWEDSLEDLQQQPTT

>Cyprinus carpio [A0A8C1XEP6]

MASATWLHGPNITRLTEALVSVLKEDRPEYLPALLANYREHGVVGAQSTGAVGGLVGISN
SRLGSSKTRFEGLCLLSVLVKDSSSEVFQQNCLSWLRTLQQVIQSQAPLPTVQLAVSVLQ
DLLQYSSQLPELAREVGLNSILGILTSLLSLKSECHLVAMNGMMACMIYYPRACGSLREK
LGAYFLSKMDSDNPKVQEVACECYGRLPCLGGVLERGGGGRRAEGWTNQLHCLLASANGM
LGQLYQGAESEGTVQYEGPGVELPFPPLDDVDPLLVLQLHHRYKAICLAIKHTLSADPAS
SVRLPVQHVLNFVCRALAVNTKSISPTGEGCLRLMILPSIHNDTLELLSALIKAVGGGLV
QYSSVLTRLFSQSLSAWTPLPESSLGQQRAYSAVRVTVYRTIELWVRVGGASLLQASPSH
TELLFTHXYLRSGQQSQSMNDLIGSAGKSGPRRTKGLGLGDGISLQRKGDVLANQDTCVA
ALRALRQIILTSGTLLKEDLHKRIQDLVVPLCVRLQQQSHCVLEVGAVSGQYGSPSPRRE
LYRLLLALVLVPSPRWPPPLSCAVSVFSHGRRDRNIMVSSFCAEALTICNTLIHPRTPSI
CLPLTPLTLKSTPTAPVLSSGQNPSLSIPTLLGGPATGPFFARHPMGLGPASLLGSLENH
LPLAPPVLPTPAGTTAIPGDLLLSPAQPGELAGLGAPEGQRQVFVRYDKEEPEDVEISLE
SDSDDSVVIMPAGMIMEMQDGAANAQSLSQSAVPAIGGLQPSAPIVGEVGSVDTLLPNEL
PTSIPHQILPANANNINSFPGTSQTAQLVSLVPPLNSSVSPAGLADSMTGGPQLQQMLMQ
TSPGGQPPTLGLSLQMQLQNQIAQTSRQLQTQPPANEVDQNVININSSDDEEEEEEELEE
EDELGEEEEEEEGLEDEEEEEEGSDLIDGEYCEDELEDYDDEEDEDDEESEEIQPLEGDS
DRGMIGEEDAEVMIEAEQQGMEMFCMEREREVEPGIEEMEGVRSVYADERIKDKGTMEEI
ENIGAVERNEPVVDKDQIESLVISGDAEGHEEDSRVEVVEPEVKTCGQEVARPEDPVEDA

GLTQQGQELTVEDEVQKQEPELKPEGTTNQSAPSTSEQEVLQSVAETAEEEVGKESGEQG
EDSETRGTKRKMEDREEGESSEQGTEKKKMDDEAMASMLADFVDCPPDDDDGGASQSQT
>Patiria miniata [A0A913ZFE8]
MARPMEDMCSVMLEIFKQESSPLSPDSSISLLTSFIEDTKELQIFGQERNCASWIGYING
CLNSSKTRLEGLSCLGALVSQCPAEIFQQHCVSWVRLLTQTLQSYDSPPIIALASGALRS
ILKEASQVSELSRELSNTHIAPIITAALELKTEWQESAVEILTACIQYFPGPSGTFRTRV
ENFLVQLMGSSNDIDIAVSAGQCLALLPRVGGGGQGGVKHTEAWSYYCQRALASIDGVVD
KIYGNSRPGKQTEPSQPPFPLPDAPLKEPTRTYTLIRQYKVLCACLQHMLRTDFPEIVKI
PVNRILSLCVSILVPNMLSKGLKSSLQSGNFSSVHIEAFNILAALISSCRGNLSLHRNVV
NELFVKSHAFRQGVQSEDTKHRVLSGLKLSAYAALEIWLETVGMNSGIETAAEKLLESIL
TDAQPQAQRTTLKVTSDKPSLTVKSSGKAKKKGETAGGEASTSLAGQQAAVPSPSGRVCL
AALKVLRCLQMSVGTRVKPDFHKDVHEFVVPLLLRLQQSPQGLAPAPYSSADCRKALYHV
LLSSVIAPHPRWSAPLQCAVGLFSRGRQDTSIKVSSFCAEASIICQSIIHPRVPCLQQPL
SLAQLDPKRAPSQSVSEARTRSDPDYMETTDLSVGLATPSQTAEGNSVGLDSGGGIAPSV
DKHIPIRSVGIAQPAVATAKMEVPSNGTEESEDSSDEDIEDQEITEDDPGIKEDNQIKGD
YQEIIQDRPEVSASKETTSRSDASVASTSSQLQDDSVLASTVSISGGKKVASSSASSDDS
KISSPTRRSKRQLARVQEEERRERENAGAREESGDADSGSKKRRREDEDEDETKEEVPEK
NLEGDVLDETDVMLASFVDSLPDKE
>Hemibagrus guttatus [A0AAE0VBS4]
RVSLRLALLCSHFSFYYLSLDGVLLVDPEYLKDRKVFVTLTCAFRYGREDLDVLGLSFRK
DLYISTFQAFPPLPEERKPLSRLQERLLKKLGQHAHPFNFTIPQNLPCSVTLQPGPEDTG
KACGVDFEIRAFCAKSVEEKIHKRNSVRLVIRKVQYAPEKPGPQPMVETTRSFLMSDRSL
HLEASLDKELYYHGEPISVNVHVTNNSTKTVKRIKISVRQYADICLFSTAQYKCPVAQVE
ADDQVSSSSTFCKVYTLTPTLSNNREKRGLALDGKLKHEDTNLASSTIVKDASNKEVLGI
LVSYRVKVKLVVSRGGDVAVELPFVLMHPKPSEQPSSRPQSTVPETDAPVDTNLIEFETK
GGKMATAAWLHGPKNMRLTEGLVSALKEERPEYLPALLANYREHGVVGTQNCGTVGGLVG
ISNSRLGSSKTRFEGLCLLSMLVKDSSSEVFQQHCLSWLRSLQQIIQSQAPLPSVQLAVS
VLQDVLQYSSQLPELAREVGLNSILGILTSLLGLKSECHLAAMEGMMACMTYYPRACGSL
KEKLGVYFISKMDSDNPKVQDVACKCYGRLPCLGGVLERGGGGRRAEGWTSQVHCLLASA
NSILGQLYQGIETEETMQYEGPGVELPFPPLDDVDPLLILQLRHRYRAVCLALKHTLSVD
PATSVRLPIQHVLNLVCRALAVGIKNINVTSEGCLKMLVLPSIHSDSLEVLSALIKAVGA
GLVQYCNVLSRLFSQALCAWTPLPEASLGQQRAYSAVRVALYHTLELWVRAGRASSSVLQ
GSSSHSELLFAHLIGDITPGTDAVKLRAGQTAMSDLVAAGKTGPRRTKGMGVVDPGGVSL
QRKGDALANQDTCFAALRVLRQIILTSGTLLKEDLHKKLQELLVPLCVRLQQQARCSNWE
VGGVSGQYGSAAPRSELYALLLALVLVPSPRWPAPLSCAVCVFSQGRKDHNTTATELAGL
AAPPETQRQVFVHYDKEEPEDVEISLESDPDDSVVIIPQGMMLEMQEGAANTQSLPPPPG
STMSVPNTGLGNEAGPVETSLSSDLPTTIGHQMLPADSNNINSFPGSGQMEQLVSLVHSY
NKC
>Sander lucioperca [A0A8D0A9T3]
MATSAWLRGPSATRLTEGLVSVLKEQRTEYLPALLANYREHGVFQTQGASTVGGLVGFSN
AKLGSSKTRFEGLCLLSMLVKDSSSDLFQQHCVSWLRSLQQVIQSQAPVQTIQLAVNILK
DLLQYSSQLAELAREVGLNSILGILTSLLGLKTECELAAMEGMTACMTYYPRACGSLRDK
LGAYFLSKMDSTNKKTQEMACLCYGHLPCLGGLLDRGVGAGRAEGWTNQIHCLLASANGL
LAQLYQGSESGTLFSVQYEGSGVELAFPHLDQSDPLLLLQLQHRYTAVCLALRHTLRVDP
ASAVRLPVRPILNLVCRVLAVSSKSIVSILHKTTILCVVVCCSARSGMVQYAAVLQRLFS
QTLSAWAPPPEASLGQQRAYSSVRVSVYRTLDILQGSPGHSELLFSHLLGDITPGAESVK
LRVGLSADAVPGGKPGPRRTKQLVIVDTVGPSLQRKGDLLANQDTCLSALRALRQIILAS
GTLLKDDIHKRLHDVVLPLCVRLQQQQSSSSISCESAGGVSGQYSSALARQELYRLLLAL
VLVPSPCWPPPLTCAVSILSNGRNDHNLKVSTFCTEALTICNSLLHPRLPSIALPLPPLS
LKPSPAAPVLSSSQGTTPGLTLPTLLGGPAPGPPFPTRHTLGLGPSSLLGSLENHLSLVP
GLSGQAPGDMILSPHGHHQPEPAGLGPPEGQRPVFVRYDREEADDVEISLASDSDDSVVI
VPPGMLNMDSQQDETAAVVAAAAAANSHNMASAAPGTLPGGESVAMVPTTTATGTTIDRL
SLANDLATSSPLLTTSTAPINSFPPPGSSVVSLVPPLNSSTLAAPPGALGDSLPGRPQLQ
QMLMQASTPSQPGAMALPLQMHQLQNQLSQQGRHLHPHQPPSASNEDSAVININSTDEEE
EEEDDLEDDEELDEEEEGMDEDDEEEDVSDFADDEELYDGEEYGEYDEEEGEELEEEEEE
EEEEDGDIPPLEGAEDKAGEAGADGVKVLRAAVDDGGMAGFSVEGEAEGGIEEIQNSRSL
FGDDRIKVQKVCSGNLPRKKSPYSLVCSPPKADSSIHQESGAEPAQEAGGSDTQPPSQQE
EPLAAVEDGDPPETDAEQREEVKAEQQETEGVEMGESDGEEGKGVKRKREEPQEEAGRST
EKKKLDDEAMASMLADFVACPPDDEDGASGSNRS
>Varanus komodoensis [A0A8D2LI79]
MTPSCAGPWPPSWRPFLSGAGTWGGLLWAHVGWLGGARRGAGLVPRRRPDPVLRSYPFCR
PPCRFEGLCLLSLLVTESTTETFSQNCLSWLRSLQHLIQSQDPAPTMELAVLVLRDLLEC
SCQLPELARDISTNHVPGLLTSLLALRPECQLSALEGSKACMMFYPRACGSLRGKLAAYF
LSRVDAETPQVQQLACECYALLPSLGAGFTQGLKYTECWEQQAHCLLATLHSLMGTLYEG
AETDPLHYEGPGMEIPLPAPEEGETNFVLHLKQRFSGLAKCLCRMLSNEFVAPVTVPVQD
ILDFICRALDISMKNISWFGDGPLRMLFLHSVHLESLDLLFALILACGPRLVRFGGTLCR
LFPQVLTAWSAGRDLVPAGQERPYSAVRTRLYQVLDLWAQVAGAASGLLQGHGTQSEALL
GHLLSDISPPTDTLKLRESAPGPDRRPSAPKKPKLAGLGPLHHKHDPRANSDVCLAALQG
QEPLGAAGRRGWASREQGGAPSRPPPRRPALASSSGGGWPPLLQVSSFCSEALVVCNALL
HPRVPALQVPLPGPPAPPPGSSSEISPATASPFRSAPPLPFPPPRLPPPASPPAPPSAAP
PAGSLGLPLPPLALAQLPPRLAPEEPAVLPSPGGAAEAAALAAGAKLRRSVFIHYDKEEE
EDVEISLESDSDDSVVIMPKGQLGRGPGGAGAVAAVAAAPAPLPPPPPAPPRGSRCLRLS
DQEEEFEEDEGEFDELEEEIEEMDEEEEEEDEFDEEECMTEEEEEEEEEEEEEEEEGLTE
EEEEGAPPLPTLPHGNGGEPPALMPMKEELPKLVGEEEGGEGEEEEEGAGLLMEVEEEAF
HAPEDGSGGQEGEEEAAAGTREVGRTAGDALLRKAEEPPVPTSAVEELLPPPLPLPPSPA
QEPPTLPATAFTDPEELAEEQERPMAGGPPFAAAPQDPEGERGPAGHAGGPAEAGPEARA
PQGEAEEQLPALGGHVPEEEPEVRAWGGQAGGRAGSPATPGCPMASAAAWRHWKPQLSCP

VLGLPAPASGAPAALAEGESDPQPGPLGLGAGRRWAGGPREGVS
>Dissostichus eleginoides [A0AAD9BUF8]
MATSAWMRGPSAMRLTEGLVSVLKEQRPEYLPALLANYREHGVFQTQGASAVGGLVGFSN
AKLGSSKTRFEGLCLLSMLVKDSSSDLFQQHCVSWLRSLQQVIQSQAPVQTIQLAVNILK
DLLQYSSQLPEVAREVGLNSILGILTSLLGLKTECELAAMEGMTACMTYYTRACGSLRDK
LGAYFLSKMDSTNKKTQEMACQCYGRLPSLGGLLDRGVSAGRAEGWTNQIHCLLASANGL
LAQIYHGSETDGTVQYEGPGVELAFPHLDQSDPVLLLQLQHRFTAVCMALKHTLRVDPSS
AVRLPVRPILNLVCRVLAVSSKSINLTGDGSVRLLVLPIIHTNTLEVLSALITAVRCGMV
QYAAVLQRLFSQTLSAWTPLPEASLGQQRAYSSVRVSVYRTLELWVQVAGASASILQGSP
GHSELLFSHLLGDITPGAESIKLRAGLSSDAVPGGKPGPRRTKTLVMSDSVGPSLQRKGD
VLANQDTCLSALRALRHIILTSGTLLKDDIHKRLHDVVLPLCSAGSVSGQYSSAPSRKEL
HRLLLALVLVPSPCWPPPLTCAVSILSKGRNDRNLKALTICNSLLHPRIPSIALPLPPLT
LKPTTNAPNHLSLVPGQTDMILSPHGHHQQDAAGLGLPEGQRPVFVRYDREEAEDVEISL
ASDSDDSVVIVPPGMLHLETQQDEAAAAANSLNMVNAPGGATLPLPDSLSAAPLGDSLPG
RPQLQQMLMQPPAAGQPGPMGLPLQMHQLQNQLSQSGRHLHQHQAPPASNEDSAVININS
TDEEDEEEEDMEDEEELDEEEEGLDEEDEDEEASDFVEEEFYDGEEYDDYDEEEGEELEE
DEEEDGEIPPLEGAEDNAGEAGIEGGKVLRAVVDEGGMAGFSVEAEAEGGIEEIQTNRAM
FGEDRVKVQKVESIGVLEEAREGEGEEDENERMDDPTMPQILCVTGGALEDREEAEEEGG
AARGGEQEEARTWEQGANEIELKAASEECTANQNQESADEPAQEASVSDSLPSNQQEAQP
EGERDPAAAEPMDDDSMASMLADFVACPPDDEDGASGSNRS
>Potamilus streckersoni [A0AAE0WC21]
MEDYREHVVSTYKTTWLEDDRGQQNILSALDIAAQHQLFALQKKGSCQEILSYIHDCLNS
YKNSGPGLLLLSHLIKECSTEVFMENAMTWLRLCTQIIQSYNKAAVHSLACDVCANILSI
ATSFTDLSREITSSVIPQLLPPLLIANKEWRQFAFICINSCIRNFSGSCGPFKNKIEACV
LEDIRTCKPCKAAVVCFSLLARCGGGGNQGVKYTEGWKQQCDQLTDSMSHTLSLLYDGME
TDKNLREQCAKLIFTSTVPDSLSERLTNLVGRWKVLCDCLSSLLSESFPAVVNLPIERIL
GLICRTLSVHGKMLLSRPTTERVILASLIPAVHESALEMLKVLFTCCKGLLIPFTRVISD
MISQELSWTKSVDQYGQEKPYGQLRRLVYECVITWCQNLDSNTGITGDDEAQLIHQILHD
LTPQMDILKIDSSKSSQKPGADTVLSGKKKKGGYSEISKGISTQRKVDLTANAALVSSGL
AALNWLLTTSGSSLGRKALQGIQEFVITTLLTIHQSIMSPPIPYTDSECRQSLYRVLLAL
SLLTHHTVPPPLQCALGLFRTGLLDTSLKVSSFCIEASRVCEALIHPRVPCLNGPIVCDS
LVHIGNQKIRELSNGNTYQSEEDSLVQHEETLPMATFTVLPSSSNISVTDTAKHTVEDEP
KSDSWRGRKRSFEETGGHKGNGNSNKQMRVDEVISHNVHNPSTSGSTKPQNFVNNQEHRA
SDLHDSQRTEAPDDGQHAVIDKDKSSDVSSEEKRDNKMEQDQEASEEDLDIEEEKDSEAD
VDFVQKTSRENIASLDSIGIVEEDVDIVREGMSPEKEEPRNDSHHSTSFAEQTHLKEKEE
IDDMLSSFVDVGPDAD
>Geospiza parvula [A0A8C3Q550]
SLAATLGWAGQTALIRKVGGVCHPPQQPIRAVGRVCVGVSRLSQWRCSFEGLCLLSLLVS
ESPSEAFQQHCLGWLRLLQHLLQSQDPPPTVALGVAVLRELLRFSAQLPELARDIGTNHI
PGILTSLLALRPEVCPPPQFRGNLGRGLGPGTPRNLPEFRAQGKLAAHFLSRIDSESPRL
QQLACECYALLPALGRGFSQGLRHTECWQQQLQGVLATLHGLLGALFEGCETDPLPYEGP
GVELLLPPPQDDAGGILTLHSRFSGLCRVLKLLLRYQNPRNSPQKSPKSWFGEGPLRGLL
LPQVHLDALDVLGALLLACGARLVRWGSLLGRLFPQVLSSWSGPRDPPPGQERPFGAVRS
RLYQVLELWVQVAGAASGVLQSPGTPSEALLAHLVSDISPPSDGAKVRPNRPESPQTPKP
SAPKRPKLGEGLEGPPLHRKGEPAANSDTCAAALAALRRILLTGGPLIKEETHRRLQELV
VPLALRLPQLCPPELFGPSGSPYGSARCRGGLYGVLQALLLGAPPGAAPPLQCALRAFGQ
GQRDPDVSSQCRESLLLCTALCRPLLAPPPAPPPPAPPPPPTPFRAPPPNPDPSGAAPPP
PPPPSVPKPELPPLPPPAQGEAGPAGAGPAGAGPAGGGARPRRPVFVHYEREESSDVEIS
LESDSDDSVVIVPKKGPELPDRMGGRVAAPPPPPPPPPPPPPPPPPTPPAAPEPAQEEEE
EEEGEEGMGEGVMVGVGDGLGDGGGAGSPVGGGGGRGRGGQEMVGEGPAVININSSEEEE
EEEFGEEEEEGGGGGGGGLSEEEEEEEEEGKQRGRGRKPRPPLPPPPRLPPSPSPTPKQE
GEEEEAAEPPLPHPEPPPPPPPTSPPPLPHPEAPPAPPPALEAAPKSPPPAPPPAGVPAPA
PPPPALPHPWVSIFAPPLRQEQDETTTMLADFIDCPPDDEKTPEAPL
>Pogonophryne albipinna [A0AAD6A9Y5]
MATSAWMRGPSAMRLTEGLVSVLKEQRPEYLPTLLANYREHGVFQTQGASAVGGLVGFSN
AKLGSSKTRFEGLCLLSMLVKDSSSDLFQQHCVSWLRSLQQVIQSQAPVQTIQLAVNILK
DLLQYSSQLPEVAREVGLNSILGILTSLLGLKTECELAAMEGMTACMTYYTRACGSLRDK
LGAYFLSKMDSTNKKTQEMACQCYGRLPSLGGLLDRGVSAGRAEGWTNQIHCLLASANGL
LAQIYHGSETDGTVQYEGPGVEVDPSSAVRLPVRPLLNLVCRVLAVSSKSINLTGDGSVR
LLVLPIIHTNTLEVLSALITAVRCGMVQYAAVLQRLFSQTLSAWTPLPEASLGQQRAYSS
VRVSVYRTLELWVQVAGASASILQGSPGHSELLFSHLLGDITPGAESIKPGPRRTKTLVM
SDSVGPSLQRKGDVLANQDTCLSALRALRHIILTSGTLLKDDIHKRLHDVSAGSVSGQYS
SAPSRKELHRLLLALVLVPSPCWPPPLTCAVSILSKGRNDRNLKVSTFCIEALTICNSLL
HPRIPSIALSLPPLTLKPTPNVPVLPTSQNHLSLAPGLPGQSDMILSPHAHHQQDAAEGQ
RPVFVRYDREEAEDVEISLASDSDDSVVIVPPGMLHLETQQDEVAAAAAANSLNMVNAPG
GATLPLPESLSMVPTTAATTIDRVSLPNDLATSSPLLTTSTTPINSFPPSNSSVVSLVPP
LNSSSLSAAPLGDSLPGRPQLQQMLMQPPAAGQPGPMGLPLQMHQLQNQLSQSGRHLHQH
QAPPTSNEDSAVININSTDEEDEEEEDDEELDEEEEGLDEEDEDEEASDFVEEEFYDGEE
YDDYDEEEGEELEEDEEEDGEIPPLEGAEDKAGEAGIEGGKVLRAVVDEGGMAGFSVEAE
AEGGIEEIQTNRAMFGEDRVKVQKVESIGVLEEAREGEEDENERMDDPTMPQILCVTGGA
LEDREEAKEEGGAARGGEGGEQEEARTWEQGANEMELKAASEECTAHQNQESADEPAQEA
SVSDSLPSNQQEAQPEGERDPAAAEPATSTGPKTKQQQQEEEEAEQMEAGRGESEGEEGK
GVKRKREETHREEEPGSEKKKMDDDSMASMLADFVACPPDDEDGASGSNRS
>Cyprinus carpio carpio [A0A9J7YXH3]
SSISFLQPTASSHSDQFKFSSSSIIPFTPKYIKYEKNRTVATSFILTLYGCDDVCFRFEG
LCLLSVLVKDSSSEVFQQNCLSWLRTLQQVIQSQAPLPTVQLAVSVLQDLLQYSSQLPEL
AREVGLNSILGILTSLLSLKSECHLVAMNGMMACMIYYPRACGSLREKLGAYFLSKMDSD

NPKVQEVACECYGRLPCLGGVLERGGGGRRAEGWTNQLHCLLASANGMLGQLYQGAESGT
VQYEGPGVELPFPPLDDVDPLLVLQLHHRYKAICLAIKHTLSADPASSVRLPVQHVLNFV
CRALAVNTKSISPTGEGCLRLMILPSIHNDTLELLSALIKAVGGGLVQYSSVLTRLFSQS
LSAWTPLPESSLGQQRAYSAVRVTVYRTIELWVRVGGASLLQASPSHTELLFTHLMGDIT
PASEAVKLRSGQQSQSMNDLIGSAGKSGPRRTKGLGLGDGISLQRKGDVLANQDTCVAAL
RGERSIILTSGTLLKEDLHKQSHCVLEVGAISGQYGSPSPRRELYRLLLALVLVPSPHWP
PPLSCAVSVFSHGRRDRNIMVSSFCAEALTICNTLIHPRTPSICLPLTPLTLKSTPTAPV
LSSGQNPSLSIPTLLGGPATGPFPARHPMGLGPASLLGSLENHLPLAPPVLPTPAGTTAI
PGDLLLSPAQPGELAGLGAPEGQRQVFVRYDKEEPEDVEISLESDSDDSVVIMPAGMIME
MQDGAANAQSLSQSAVPAIGGLQPSAPIVGEVGSVDTLLPNELPTSIPHQILPANANNIN
SFPGTSQTAQLVSLVPPLNSSVSPAGLADSMTGGPQLQQMLMQTSPGGQPPTLGLSLQMQ
LQNQIAQTSRQLQTQPPANEVDQNVININSSDDEEEEEEELEEEDELGEEEEEEEGLEDE
EEEEEGSDLIDGEYCEDELEDYDDEEDEDDEESEEIQPLEGDSDRGMIGEEDAEVMIEAE
QQGMEMFCMEREREVEPGIEEMEGVRSVYADERIKDKGTMEEIENIGAVERNEPVVDKDQ
IESLVISGDAEGHEEDSRVEVVEPEVKTCGQEVARPEDPVEDAGLTQQGQELTVEDEVQK
QEPELKPEGTTNQSAPSTSEQEVLQSVAETAEEEVGKESGEQGEDSETRGTKRKMEDREE
GESSEQGTEKKKVTE
>Podarcis lilfordi [A0AA35L9A9]
MAASGLSAGSCSSSSVLGGPGGAAPSGRLVLETLAGLLRGGAQAGGGPETTPGLAGLLRC
AREAGGGGGAQASPTLGGLVSLSNTRLGSIKTRFEGLCLLSLLVTESSTEAFSQNCLGWL
RSLQHLIQSQDPPPTMELAVLILRDLLEYSCQIPELARDIGTNHIPGLLTSLLALKPECQ
ISTLEGSKACMMFYPRACGSLRGKLAAYFLARVDAETPHLQQLACDCYALLPSLGAGFTQ
GLKYTECWGQQAHCLLATLHSLLGTLYEGAETDPLHYEGPGVEILLPTPEDGEVNFILRL
KHRFSGLAKCLCQMLSNDFVAPVSVPVQDILDLVCRALNISTKNISWFGDGPLRMLLLPS
VHLETLDLLSALILACGPRLVRFGATLGRLFPQVLTTWSSGRDLFPPGQERPYSAVRTRL
YEVLDLWVQSAGAACGILQGPRTQSEALLGHLISDISPPADTLKMRDSRPASDLKPSAPK
KPKLSDLGTLGSLHPKQDSQANSSTCLAALRGLSRAVLLTGSLMKEQVHKRLQELVVPLL
IRLGQAETPLGSPYASAPCRRELHRLLLALVLAPPPSWPPPFHCALRLFSQGRADPNLQV
SSFCTEALAVCNALLHPRVPSLQLPLAAPPGGPQAPPPGPSELSPALASPFRPAPPAPFS
APRLLLPPPASSAANPLGLPPPGLASPAQLPPRLAPEEPSLPTSPGAAEAALGAKLRRSV
FVHYDKEEEEDVEISLESDSDDSVVIVPKGQLGKGPGSAMVVAAIAAQAPTPPAPPPAPP
APPSPPPASEETPPEPPAPPLSLGVPLPPAPAAVLALPASPSPAAMDPPFPALVEEDPTV
ININSSEEEEDDEDDYPEDEEYFEEDEEEEEEYDEEDEEFGEDLEEEEDEEFDEEEGLTE
EEEDEEEGLTEEEEEEEEEGEEEEQRVLPSLPPPLPPHRSDEEPPEVLPIKEEPPKLEEE
EEEENEDEEAAAGLLMEVDEEAFHPPEEMAEEEEEGGRLERDPGKAEGQASPLRTAEGQS
PLPSAAPLPSVKLEEEQGEKQPPQEEQEGPAAAAAPRTTTPQGQEEEEEEEVVVHEGGRR
EPGGAAPEAAALQGEAEKQEPPPGEEGTEVDETEAMLADFVDCPPDEEKVPSEPSS
>Mustela putorius furo [A0A8U0SA23]
MAAAVLSGPSAGSAAGVPGGTGGLSAVGSGPRLRLLLLESVSGLLQPRAGGAVAPVHPPV
RSAPHLPGLMCLLRLHGTVGGAQNLSAVGALVGLSNARLGSIKTRFEGLCLLSLLVGESP
TEMFQQHCVSWLRSIQQVLQSQDPPPTMELAVAVLRDLLRYAAQLPTLFRDISMNHLPGL
LTSLLGLRPECELSALEGMKACMTYFPRACGSLKGKLASFFLSRVDALSPQLQQLACECY
SKLPSLGAGFSQGLKHTESWEQELRSLLASLHSLLGALYEGAETAPMQYEGPGVEMLLTP
SEDGDAHVLLRLRQRFCGLARCLGLMLSSEFGAPVSVPVQEILDVICRALSISAKSISLL
GDGPLRLLLLPSIHLDALDLLSALILACGGRLLRFGALISRLLPQVLNAWNLGRDTLSPG
QERPYSTVRAKVYAVLELWVQVCGASAGVLQGGASGEALLSHLLSDISPPADTLKLRSPR
GSPDAGLQSGKPSAPKKLKLDMGDAAAPPSHRKGDANANSDVCAAALRGLSRTVLMCGPL
IKEETHRRLHDLVLPLVMGVQQGEVLGSSPYTSSRCRRELYRLLLALLLAPAPRCPPPLS
CAVQAFSLGQREDSLEVASFCSEALVTCAALTHPRVPPLQSMGPACPTPAPVPPPEAPSP
FRAPPFHPPGPMPSVGPMPSVGPMPSASAMPPTGPLPPAGPLPPTRPGPPATANHLGLSV
PGLVSVPPRLHPGPENHRAGSNEDPVLAPSGTPPPPIPPDETFGGRVPRPAFVHYDKEEA
SDVEISLESDSDDSVVIVPEGLPPLPPLPPSGSTPPPVAPAGPPTASPPGPAKEEPEELP
TAPGPLPPPPPPPVPGPVALPPPQLVPEGTPGGGGPPGLEEDLTVININSSDEEEEEEEE
EEEEEEEEEEEEEEDFEEEEEDEEEYFEEEEEEEEEFEEEFEEEEGELEEEEEEEEDEEE
EELEELEEVEFGPTGAPAEESGPPPPSPPPALPPAQSPKAQPEPEGEPGLLLEVEEPGAE
EGPGAEAAPTLVPEVLPSEGEGPPDPESVPAGPPPQELEEEEPSAPPTLLEEGTESGGDE
VPVPPETPAVADVEAEAEAETAALPEKEQDDTAAMLADFIDCPPDDEKPPPAPEPDS
>Callithrix jacchus [A0A8I4A6F2]
MAAAVLSGPTAGSAAGVPGGTGGLSAVSSGPRLRLLLLESVSGLLQPRTGSAVAPVHPPN
RSAPHLPGLMCLLRLHGSVGGAQNLSAVGALVSLSNARLSSIKTRFEGLCLLSLLVGESP
TELFQQHCVSWLRSIQQVLQGKLASFFLSRVDALSPQLQQLACECYSKLPSLGAGFSQGL
KHTESWEQELHSLLASLHTLLGALYEGAETAPVQHEGPGVEMLLSSEDGDAHVLLRLWQR
FSGLAHCLGLMLSSEFGAPVSVPVQEILDFICRTLSVSSKNISLHGDGPLRLLLLPSIHL
EALDLLSALIVACGSRLLRFGIPISRLLPQVLNSWSIGRDSLSPGQERPYSTVRTKVYAV
LELWVQVCGASAGVLQGGASGEALLTHLLSDISPPADALKLRSPRGSPDGGLQTGKPSAP
KKLKLDVGEAMTPPSHRKGDSNANSDVCAAALRGLSRTILMCGPLIKEETHRRLHDLVLP
LVMGVQQGEVLGSSPYTSSRCRRELYCLLLALLLSPSPRCPPPLACALQAFSLGQREDSL
EVSSFCSEALVICAALTHPRVPPLQSMGPTCPTPAPVPPPEAPSPFRAPSFHPPGPMPSV
GPMPSAGPMPSAGPMPSAGPVPSAGPVPSARPGPPTTANHLGLSVPGLVSVPPRLLPGPE
NHRASSNEDPILAPSGTPPPTIPPDETFGGRVPRPAFVHYDKEEASDVEISLESDSDDSV
VIVPEGLPPLPPPPPSGATPPPVAPSGPPTASPPLPAKEEPEELPAAPGPLPPPPPPPPP
VPGPVTLPPPQLVPEGTPGGGGPPALEEDLTVININSSDEEEEEEEEEEEEEEEEEEEEE
DFEEEEEDEEEYFEEEEEEEEEFEEEFEEEEGELEEEEEEEEDEDEEEELEEVEELEFGT
AGGEVEEGGPPPPTLPPALPPPESPPKVQPEPEPEPGLLLEVEEPGAEEEHEADTAPTLA
PEVLPSQGEVERDEGSPEAGLPPQELVEEEPSAPPTLLEEGTEDGGDKVQLPPETSAEEE
METETEAEALQEKEQDDTAAMLADFIDCPPDDEKPPPATEPDS
>Galemys pyrenaicus [A0A8J6A8K1]

MCACSYKVGPQLRPRYPHRAGPKPSTHAPSPGGLCRVVFASSRHATSRARVKMAAAVLSG
PSAGSAAGVPGGTGGLSAASSGPRLRLLLLESVSGLLQPRAGSTVAPVPPPARSAPHLPG
LMCLLRLHGTVGGAQNLSAVGALVALSNARLGSIKTRFEGLCLLSLLVGESPTEMFQQHC
VSWLRSIQQVLQSQDPPPTMELAVAVLKDLLRYAAQLPTLFRDISMNHLPGLLTSLLGLR
PECELSALEGMKACMTYFPRACGSLKGKLASFFLSRIDALSPQLQQLACECYSRLPSLGA
GFSQGLKHTESWEQELHSLLASLHSLLGALYEGAENAPTQHEGPGVETLLSPSEDGDAHV
LLRLRQRFSGLARCLGLMLSSEFGAPVSVPVQEILDVICRTLSISAKNISLLGDGPLRLL
LLPSIHLEALDLLSALILACGSRLLRFGALISRLLPQVLNAWSIGRDTLSPGQERPYSTM
RTKVYAVLELWVQVCGASAGVLQGGASGEALLTHLLSDISPPTDALKLRSPRGSPDGVLQ
SGKPSAPKKLKLDMGEAVAPPSHRKGDSNANSDVCAAALRGLSRTILMCGPLIKEETHRR
LHDLVLPLVMGVQQGEVLGSSPYTSSRCRRELYRLLLALLLAPSPRCPPPLSCALQAFSL
GQREDNLEVSSFCSEALVTCAALTHPRVPPLQSMGPSCPPAAPVPPPETPSPFRAPPFHP
PGPMPSAGPVPSVGPMPSVGPMPSAGPMPSAGSMPSVGPLPPTRPGPPVTANHLGLSVPG
LVSVPPRLLPGPENHRAGSNEDPVLAPSGTPPPIPPDETFGGRVPRPAFVHYDKEEASDV
EISLESDSDDSVVIVPEGLPPLPPPLPSTTTSPPVTPAGPPTASPPVPAKEEPEELPAAP
GPLPPPPPTPVPGPVTLPPPQLVPEGTPGGGGPPALEEDLTVININSSDEEEEEEEEEEE
EEEEEEEEEEDFEEEEDEEEYFEEEEEEEEFEEEFEEEEGELEEEEEEEEEEEEEELEEV
EELEFASTGGEVEEGGPPPPSLPPALPPPESPKVQPEPEPETGLLLEVEEPGAEEDQGAE
TAPTLAPEVLPSQEEVEREAGSPSAGPSPPQELVEEEPSAPPTLLEEGTEGESDKLPTLP
ETSEVGEMESETEVAALQEKEQDDTAAMLADFIDCPPDDEKPPSATESDS
>Equus caballus [A0A9L0SGF2]
MDPLSAYPAPPSVGAWGKLDLNLSAVGALVGLSNARLGSIKTRFEGLCLLSLLVGESPTE
MFQQHCVSWLRSIQQVLQSQDSPPTMELAVAVLRDLLRYAAQLPTLFRDISMNHLPGLLT
SLLGLRPECELSALEGMKACMTYFPRACGSLKGKLASFFLSRVDALSPQLQQLACECYSR
LPSLGAGFSQGLKHTESWEQELHSLLASLHSLLGALYEGAETAPMQYEGPGVEVLLSPSE
DGDAHALLRLRQRFSGLARCLGLMLSSEFGAPVSVPVQEILDVICRTLSISAKNISLLGD
GPLRLLLLPSIHLEALDLLSALILACGGRLLRFGALISRLLPQVLNAWSIGRDTLSPGQE
RPYSTMRTKVYAVLELWVQVCGASAGVLQGGASGEALLTHLLSDISPPADALKLRSPRGS
PDGGLQSGKPSAPKKLKLDVGEAMAPPSHRKGESNANSDVCAAALRGLSRTILMCGPLIK
EETHRRLHDLVLPLVMGVQQGEVLGSSPYTSSRCRRELYRLLLALLLAPSPRCPPPLACA
LQAFSLGQREDSLEVSSFCSEALVTCAALTHPRVPPLQSMGPTCPTSAPVPPPEAPSPFR
AAPFHPPGPMPSAGPMPSAGPVPSAGPMPSVGPLPSVGPMPSVGPMPPARPGPPATANHL
GLSVPGLVSVPPRLLPGPENHRAASNEDPVLAPSGTPPPAIPPDETFGGRMPRPAFVHYD
KEEASDVEISLESDSDDSVVIVPEGLPSLPPPPPSGTTPPPVAPAGPPTASPPVPAKEEP
EELPAAPGPLPPPPPPPVPGPVTLPPPQLVPEGTPGGGGPPALEEDLTVININSSDEEEE
EEEEEEEEEEEEEEEEEDFEEEEEDEEEYFEEEEEEEEEFEEEFEEEEGELEEEEEEEDE
EEEEELEEVEELEFGSAGGEVDEAGPPPPSLPPALPPAESPKVQPEPEPEPGLLLEVEEP
GVEEERGAETAPTLAPEVLPSQGEVEREEGSPPAGPPPQELVEEEPSAPPTLLEEGTEGG
DDKVPPLPEPPAAEEMETETEVAALQEKEQDDTAAMLADFIDCPPDEEKPPSATEPDS
>Menidia menidia [A0A8S4BDW5]
MATSGWLHGTSAMRLTEGLVLVLKEQRPEYLPDVLAKYREHGVFPIQGASDVAGLVGYTN
AKLSSSKTRFEGLCTLSLLVKDSSSDLFQVHCLSWLRFLQQVIQSQAPLQTIQLAVNILK
DVLQYSHQQPQLAREVGLNSILGILTSLLGLKMECELAAMEGMIACMVYYPRACGSLRDK
LGAYFLSKMDNTNKKIQEMACQCYSHLPCMGGMLDRPMSVGRANGWNNQIHCLLASANAL
LAQIYQGSEAAGALQYQGPGVELAFPHLDEADPLYVLQLQHRYSAVCKTIKHTLRVDPFS
AVHLPVRPILNLVCRALAVSRKSLNLSTDGSLRLLVLPSVHTSTFEVLSDVITVVRSGMV
QYAAVIHKLFSQTLSAWTSPSETNVGKQKAYSSVRESVYRALELWVRVVGASANILQGSH
GHAQLLFSHLLGDITPEAESARLRAGLSVDVVPGGKPGPRRTKQLVIADGGEQSLQRKGD
FFPNQDTCLSALRALRQIIQTSGTLLKEDIHKRLHEVVLPLCVRLQQQQSVSSPAHESAR
GISGQYSSAITRRELYRLLLALVLVPSPFCPPPSSCAVSIFSNGRVDRNIKVSSFCAKAL
TICNSVLHSRIPSIGLPLPPLTPKSSGPASVLSSQGPASRLTLSTLLEGSLPGPAFPARH
SLSLGPSSLLDSLENHLSLVPGLPGQAPPPGDLILSQHSHHQPDVSGLGPPEGQRPVFVR
YDKEEAEDVEISLASDSDDSVVIVPPGMLNVENQQVDPSTNPQNMLPTLPDGTGITLTND
SVTMVPTTAATTAIDVSSLPNTMTTSSPLITTSTAPNNSLPPSSTSVVSLVPPLNSGTIS
APPGGLGDQLPCKAQLQQMLMQSPTAGPPNPIGLPLQMHQLTQQGRHLNQHAPAPASEES
AVININSSDEEDDEEDLEDDEELEEEGIDEDEEEEGSDEFYDGEEYEDFDEEDAEELEEE
EEDGDMPPLEGTEDKLVEEDIEEDKVLRPAVDEGGMAGYIVEGETEGGIEELQTSRAVLE
DRKKVQEVESIGVLEDERDAEGEEDESEKMDDPTMPQILCVTGGALEEGAGAGSGLQEEM
SMWDQDAKESERTAASGEHVSSQSLQEPDNAMEDAVKDDKPSKNQEEEMVAQVCDSGDAD
LESSSGQNIKGQEQTGADQAEIKEAEQSTREEKCAGQVEQEGRSVGVDGKGTKRKREDGY
TEEDEQSTEKIKPDDDAMASMLADFVACPPDDEDIATGSNSS
>Stegastes partitus [A0A9Y4KC94]
MATSTWLHGPSAMRLTEGLVSVLKEQRPEYLPALLANYREHGVFTTQGAGAVGGLVGFSN
AKLGSSKTRFEGLCLLAMLVKDSSSDLFQQHCLSWLRSLQQVIQSQAPVQTIQLAVNILK
DLLQYSSQLPELAREVGLNSVLGILTSLLGLKTECELAAMEGMTACMSYYPRACGSLRDK
LGAYFLSKMDSTNRKTQEVACQCYGRLPCLGGLLDRGVGAGRAEGWTNQIHCLLATANGL
LAQIYQGAETDGTVQYQGPGVELTFPHLDQTDPLLLLQLQHRYTAVCLALKHTLRVDPAS
AVRLPVRPILNLVCRALAVSSKSINLTGDGSVRLLVLPIIHTNTLEVLSSLITAVRRGMV
QYAAVLQRLFSQTLSAWMPLPEASVGQQRAYSSVRVSVYATLELWVQVAGASANILQGSP
SHSELLFSHLLGDITPGAESIKLRAGLSADVVPGGKPGPRRTKQLVMADAVGPSLQRKGD
LLANQDTCLSALRGLRQIIQTSGTLLKDDIHKRLHDVVLPLCVRLQQQQCSTSTACESAG
GVSGQYSSAITRRELYRLLLALVLVPSPCWPPPLTCAVSILSNGRTDRNLKVSSFCAEAL
TICNSILHPRTSSVALPLPPLTLKPTHTAPVLPSSQGPASGLTLPTLLGGPAPGPPFPTR
HSLGLGPTSLLGSLENHLSLVPGLPGQAPAPGDMILSPHAHHQPDPAGLGPPEGQRPVFV
RYDKEEAEDVEISLESDSDDSVVIVPPGMLNMENQQDDVAANSQNILSAAPGGAGVTLSG
GESVTMVPTTAATTTVEGTSLSNDLATSSPLLTTSTTPINSFPPSSASVVSLVPPLNSST
LTGPPGGLGDSMPGRPQLQQMLMQPSTAGQPSPMGLPLQMHQLQNQLSQQGRHLHQHPPA

PTSNEDSAVININSTDDEEEDEEDMEDDEELEEEEEGMDEEDEEEEVSDLAEDEFYDGEE
YEDYDEEEGEDLEEEEEEEEEDGDIPPLEGAEDKVVEAGIEEGKVLRAAVDEGGMSGFSVE
GDAEGGIEEIQTNRALFGDDRMKVQEVESIGVLEDAREGEGEEDDSEKMNDPTMPQILCV
TGGALDEREETEEEGGEAGGGLEEGESSWEQGGKEEEELTAAPEEQSTNQTQQESAAEPA
QEVSVSDNQPSSHQEEQLVAVQEEDTPADPETSADLSTKEQEETVDLKRDKTDTEQQEAA
GTEEESDGEEGKGVKRKREEGQEEEEAGQSTDKKKMDDEAMASMLADFVACPPDDEDGAS
GSNCS
>Podarcis lilfordi [A0AA35L8U9]
MAASGLSAGSCSSSSVLGGPGGAAPSGRLVLETLAGLLRGGAQAGGGPETTPGLAGLLRC
AREAGGGGGAQASPTLGGLVSLSNTRLGSIKTRFEGLCLLSLLVTESSTEAFSQNCLGWL
RSLQHLIQSQDPPPTMELAVLILRDLLEYSCQIPELARDIGTNHIPGLLTSLLALKPECQ
ISTLEGSKACMMFYPRACGSLRGKLAAYFLARVDAETPHLQQLACDCYALLPSLGAGFTQ
GLKYTECWGQQAHCLLATLHSLLGTLYEGAETDPLHYEGPGVEILLPTPEDGEVNFILRL
KHRFSGLAKCLCQMLSNDFVAPVSVPVQDILDLVCRALNISTKNISWFGDGPLRMLLLPS
VHLETLDLLSALILACGPRLVRFGATLGRLFPQVLTTWSSGRDLFPPGQERPYSAVRTRL
YEVLDLWVQSAGAACGILQGPRTQSEALLGHLISDISPPADTLKMRDSRPASDLKPSAPK
KPKLSDLGTLGSLHPKQDSQANSSTCLAALRGLSRAVLLTGSLMKEQVHKRLQELVVPLL
IRLGQAETPLGSPYASAPCRRELHRLLLALVLAPPPSWPPPFHCALRLFSQGRADPNLQV
SSFCTEALAVCNALLHPRVPSLQLPLAAPPGGPQAPPPGPSELSPALASPFRPAPPAPFS
APRLLLPPPASSAANPLGLPPPGLASPAQLPPRLAPEEPSLPTSPGAAEAAALGAKLRRSV
FVHYDKEEEEDVEISLESDSDDSVVIVPKGQLGKGPGSAMVVAAIAAQAPTPPAPPPAPP
APPSPPPASEETPPEPPAPPLSLGVPLPPAPAAVLALPASPSPAAMDPPFPALVEEDPTV
IN問INSSEEEEDDEDDYPEDEEYFEEDEEEEEEYDEEDEEFGEDLEEEEDEEFDEEEGLTE
EEEDEEEGLTEEEEEEEEEEGEEEEQRVLPSLPPPLPPHRSDEEPPEVLPIKEEPPKLEEE
EEEENEDEEAAAGLLMEVDEEAFHPPEEMAEEEEEGGRLERDPGKAEGQASPLRTAEGQS
PLPSAAPLPSVKLEEEQGEKQPPQEEQEGPAAAAAPRTTTPQGQEEEEEEEVVVHEGGRR
EPGGAAPEAAALQGEAEKQEPPPGEEVGMRGRAGREVTAHVGKNIPSLTELCTVHLPLWG
DSGAQGVTLTFQGERNLGRLSRQSRRPFISESWGQAPHRAKESCTAGGGTRCPPTSQFCD
SIILSMKALQAESFVLFGKFVDP
>Canis lupus familiaris [A0A8P0PB36]
6MAAAVLSGPSAGSAAGVPGGAGGLSAVGSGPRLRLLLLESVSGLLQPRAGSAVAPVHPPV
RSAPHLPGLMCLLRLHGTVGGAQNLSAVGALVGLSNARLGSIKTRFEGLCLLSLLVGESP
TEMFQQHCVSWLRSIQQVLQSQDPPPTMELAVAVLRDLLRYAAQLPTLFRDISMNHLPGL
LTSLLGLRPECELSALEGMKACMTYFPRACGSLKGKLASFFLSRVDALSPQLQQLACECY
SRLPSLGAGFSQGLKHTESWEQELHSLLASLHSLLGALYEGAETAPVQYEGPGVEVLLTP
SEDGDTHVLLRLRQRFSGLARCLGLMLSSEFGAPVSVPVQEILDVICRTLSISAKNISLL
GDGPLRLLLLPSIHLDALDLLSALILACRSRLLRFGALISRLLPQVLNAWNLGRDTLAPG
QERPYSTVRTKVYAVLELWVQVCGASAGMLQGGSSGEALLSHLLSDISPPADTLKLRSPR
GSPDGGLQTGKPSAPKKLKLDVGEAMAPPSHRKGDSNANSDVCTAALRGLSRTILMCGPL
IKEETHRRLHDLVLPLVMGVQQGEVLGSSPYTSSRCRQELYRLLLALLLAPSPRCPPPLA
CALQAFSLGQREDSLEVSSFCSEALVTCAALTHPRVPSLRSMGPACPTPAPAPPPEAPSP
FRAPPFHPPGPMPSAGPMPSVGPMPSVGPMPPAGPMPPAGPMAPTRPGPPATANHLGLSV
PGLVSVPPRLIPGPENHRAGSNEDPVLAPSGTPPPAIPPDETFGGRVPRPAFVHYDKEEA
SDVEISLESDSDDSVVIVPEGLPPLPPPPPSGTTPPPVAPAGPPVASPPVPAKEEPEELP
VAPGPLPPPPPPPVPGPVALPPPQLVPEATPGGGGPPALEEDLTVININSSDEEEEEEEE
EEEEEEEEEEEEEEEDFEEEEEEEEYFEEEEEEEEEFEEEFEEEEGELEEEEEEEEDEEEE
EELDELEEVAFGPAAGAAEEGGPPPPSPPPALPPAQSPKMQPEPPGETGLLLEVEEPAAE
EEPGAEAAPTLAPEVLPPQGEGPREVGSPPAVPPPQELIEEEPPAPPTLLEEGTESGGDK
VPVPQETPAAEDVEAEVEAETAALQEKEQDDTAAMLADFIDCPPDDDKPPPATEPDS
>Cyprinus carpio [A0A9Q9WHB3]
MASATWLHGPNITRLTEALVSVLKEDRPEYLPALLANYREHGVVGAQSTGAVGGLVGISN
SRLGSSKTRFEGLCLLSVLVKDSSSEVFQQNCLSWLRTLQQVIQSQAPLPTVQLAVSVLQ
DLLQYSSQLPELAREVGLNSILGILTSLLSLKSECHLVAMNGMMACMIYYPRACGSLREK
LGAYFLSKMDSDNPKVQEVACECYGRLPCLGGVLERGGGGRRAEGWTNQLHCLLASANGM
LGQLYQGAESEGTVQYEGPGVELPFPPLDDVDPLLVLQLHHRYKAICLAIKHTLSADPAS
SVRLPVQHVLNFVCRALAVNTKSISPTGEGCLRLMILPSIHNDTLELLSALIKAVGGGLV
QYSSVLTRLFSQSLSAWTPLPESSLGQQRAYSAVRVTVYRTIELWVRVGGASLLQASPSH
TELLFTHLMGDITPASEAVKLRSGQQSQSMNDLIGSAGKSGPRRTKGLGLGDGISLQRKG
DVLANQDTCVAALRALRQIILTSGTLLKEDLHKRIQDLVVPLCVRLQQQSHCVLEVGAIS
GQYGSPSPRRELYRLLLALVLVPSPHWPPPLSCAVSVFSHGRRDRNIMVSSFCAEALTIC
NTLIHPRTPSICLPLTPLTLKSTPTAPVLSSGQNPSLSIPTLLGGPATGPFPARHPMGLG
PASLLGSLENHLPLAPPVLPTPAGTTAIPGDLLLSPAQPGELAGLGAPEGQRQVFVRYDK
EEPEDVEISLESDSDDSVVIMPAGMIMEMQDGAANAQSLSQSAVPAIGGLQPSAPIVGEV
GSVDTLLPNELPTSIPHQILPANANNINSFPGTSQTAQLVSLVPPLNSSVSPAGLADSMT
GGPQLQQMLMQTSPGGQPPTLGLSLQMQLQNQIAQTSRQLQTQPPANEVDQNVININSSD
DEEEEEEELEEEDELGEEEEEEEGLEDEEEEEEGSDLIDGEYCEDELEDYDDEEDEDDEE
SEEIQPLEGDSDRGMIGEEDAEVMIEAEQQGMEMFCMEREREVEPGIEEMEGVRSVYADE
RIKDKGTMEEIENIGAVERNEPVVDKDQIESLVISGDAEGHEEDSRVEVVEPEVKTCGQE
VARPEDPVEDAGLTQQGQELTVEDEVQKQEPELKPEGTTNQSAPSTSEQEVLQSVAETAE
EEVGKESGEQGEDSETRGTKRKMEDREEGESSEQGTEKKKMDDEAMASMLADFVDCPPDD
DDGGASQSQT
>Mustela putorius furo [A0A8U0SA07]
MAAAVLSGPSAGSAAGVPGGTGGLSAVGSGPRLRLLLLESVSGLLQPRAGGAVAPVHPPV
RSAPHLPGLMCLLRLHGTVGGAQNLSAVGALVGLSNARLGSIKTRFEGLCLLSLLVGESP
TEMFQQHCVSWLRSIQQVLQSQDPPPTMELAVAVLRDLLRYAAQLPTLFRDISMNHLPGL
LTSLLGLRPECELSALEGMKACMTYFPRACGSLKPLPLFPPTQGKLASFFLSRVDALSPQ
LQQLACECYSKLPSLGAGFSQGLKHTESWEQELRSLLASLHSLLGALYEGAETAPMQYEG

PGVEMLLTPSEDGDAHVLLRLRQRFCGLARCLGLMLSSEFGAPVSVPVQEILDVICRALS
ISAKSISLLGDGPLRLLLLPSIHLDALDLLSALILACGGRLLRFGALISRLLPQVLNAWN
LGRDTLSPGQERPYSTVRAKVYAVLELWVQVCGASAGVLQGGASGEALLSHLLSDISPPA
DTLKLRSPRGSPDAGLQSGKPSAPKKLKLDMGDAAAPPSHRKGDANANSDVCAAALRGLS
RTVLMCGPLIKEETHRRLHDLVLPLVMGVQQGEVLGSSPYTSSRCRRELYRLLLALLLAP
APRCPPPLSCAVQAFSLGQREDSLEVASFCSEALVTCAALTHPRVPPLQSMGPACPTPAP
VPPPEAPSPFRAPPFHPPGPMPSVGPMPSVGPMPSASAMPPTGPLPPAGPLPPTRPGPPA
TANHLGLSVPGLVSVPPRLHPGPENHRAGSNEDPVLAPSGTPPPPIPPDETFGGRVPRPA
FVHYDKEEASDVEISLESDSDDSVVIVPEGLPPLPPLPPSGSTPPPVAPAGPPTASPPGP
AKEEPEELPTAPGPLPPPPPPPVPGPVALPPPQLVPEGTPGGGGPPGLEEDLTVININSS
DEEEEEEEEEEEEEEEEEEEEEEDFEEEEEDEEEYFEEEEEEEEEEFEEEFEEEEGELEEE
EEEEDEEEEEELEELEEVEFGPTGAPAEESGPPPPSPPPALPPAQSPKAQPEPEGEPGLL
LEVEEPGAEEGPGAEAAPTLVPEVLPSEGEGPPDPESVPAGPPPQELEEEEPSAPPTLLE
EGTESGGDEVPVPPETPAVADVEAEAEAETAALPEKEQDDTAAMLADFIDCPPDDEKPPP
APEPDS
>Cyprinus carpio [A0A9R0B0Y]
MASAAWLHGPNITRLTEGLVSVLKEDRPEYLPALLANYREHGVVGTQSTGAVGGLVGISN
ARLGSSKTRFEGLCLLSVLVKDSSSEVFQQHCLSWLRTLQQVIQSQAPLPTVQLAVSVLQ
DLLQYSSQLPELAREVGLNSILGILTSLLSLKSECHLAAMKGMMTCMIYYPRACGSLREK
LGAYFLSKMDSDNPKVQEVACESFGRLPCLGGVLERGGGGRRAEGWTNQLHCLLASANSM
LGQLYQGAETEGTVQYEGPGVELPFPPLDDVDPLLILQLHHRYKAICLAIKHTLSADPAS
SVCLPVQHVLKLVCQALAVNTKSISPTGEGCLKLLVLPSIHIDTLELLSALIKAVGGGLV
QYSSVLTRLLSQSLSAWTPLPEASLGQQRAYSAVRVTVYRTIELWVRVGGASLLQGSPTH
TELLFTHLMGDITPASEAVKLRSGQQSQSMSDLIGSAGKSGPRRTKGLVMGDGISLQRKG
DVLANQDTCVAALRALRQIILTSGTLLKEDLHKRIQDLVVPLCVRLQQQSHCVLDVGAVS
GQYGSPTPRRELYRLLLALVLVPSPRWPPPLSCAVSVFSHGRRDHNIMVSSFCAEALTIC
NTLIHPRTPSISLPLTPLTLKSTPSAPVLASGQNPSLSIPTLLGGPPFPSRHPMGLGPAT
LLGSLENHLPLAPPGLPTPAGTTATPGDLLLSPAQPGELAGLGAPEGQRQVFVRYEKEEP
EDVEISLESDSDDSVVIMPAGMMMEMQDGAANAQSLSQPAVPAPIVGEVGLVDTLLPNEL
PTSIPHQILPANANNINSFPGPSQTAQLVSLVPPLNSTTASLSASPAGLADSLTGGPQLQ
QMLMQTSPGGQPPTLGLSLQIQLQNQIAQTSRQLQQQHPANEVDQNVININSSDDEEEEL
EEDELGEEEEEEEGLEEEEEEEEGSDLMDEEYYEGEEFDEYEDEEEEEESEEIQPLEGDN
SRGMMDEEEAEVMIEAEDQQGMEMFCMEREREVEPGIEEMEGVRSVYADDRIKDKGTVEE
IENIGAVERNEPVVGKEQIESLVISGETEGHEEDTSVEAVEPEVKTCEQEVAKPEDPTEN
AGPSQQGQELTVEVEVQKQEPELQPEDTTNQSAPSTSKQEALQSVAVTAEEEEEVEKESG
EQGEDSDARGTKRKMEDREEGESSEQGTEKKKVDEAMASMLADFVDCPPDDDDHGASQSQ
S
>Channa striata [A0AA88N8H3]
MATSAWLHGPAAMRLTEGLVSVLKEQRPEYLPALLANYREHGVFSTQGASTVGGLVGFSN
AKLSSSKTRFEGLCLLSMLVKDSSSDLFQQHCISWLRSLQQVIQSQAPVQTIQLAVHILK
DLLQYSSQLAELAREVGLNSILGFLTSLLALKAECEMVAMEGMMACMTYYPRACGSLRDK
LGAYFLSKMDSTNKKTQEMACQCYGRLPCLGGLLERGVSAGRTEGWTNHIHCLVASANSL
LAQIYNSSETDGTVQYEGPGVDLAFPHLDQSDPLLLLQLQHRYTAVCLALKHTLRVDPAS
AVRLPVRPVINLVCRALAVNLKSINLTGDGSVRILILPIIHINTLEVLSALITVVQSGMV
QYAAVLQRLFSQTLSAWTPPHETSLGQQRAYSSVRVSVYRTLELWVQVAGASASILEGSP
GHSELLFSHLLGDITPGAESVKLRAGLSADVVPGGKPGPRRTKPLVIADAVAPSLQRKGD
LLANQDTCLSALRALRQIILTSGTLLKDDIHKRLHDVVLPLCVRLQQQQTSSNTACESGG
ISGQYSSALSRRELYRLLLALVLVPSPFWPPPLTCAVSILSNGRTDHTLKVSAFCTEALT
ICNSLLHPRTPSIALPLPSLTLKPNPTAPGVPSSQGPAPGLTLPTLLGGPSSGSSFPTRH
SLGLGHASLLGSLENHLSLVPGLQGQAPTPGDMILSPRTHHQPDPAGLGPPEGQRPVFVR
L
>Cyprinus carpio [A0A9R0B0Y1]
MASAAWLHGPNITRLTEGLVSVLKEDRPEYLPALLANYREHGVVGTQSTGAVGGLVGISN
ARLGSSKTRFEGLCLLSVLVKDSSSEVFQQHCLSWLRTLQQVIQSQAPLPTVQLAVSVLQ
DLLQYSSQLPELAREVGLNSILGILTSLLSLKSECHLAAMKGMMTCMIYYPRACGSLREK
LGAYFLSKMDSDNPKVQEVACESFGRLPCLGGVLERGGGGRRAEGWTNQLHCLLASANSM
LGQLYQGAETEGTVQYEGPGVELPFPPLDDVDPLLILQLHHRYKAICLAIKHTLSADPAS
SVCLPVQHVLKLVCQALAVNTKSISPTGEGCLKLLVLPSIHIDTLELLSALIKAVGGGLV
QYSSVLTRLLSQSLSAWTPLPEASLGQQRAYSAVRVTVYRTIELWVRVGGASLLQGSPTH
TELLFTHLMGDITPASEAVKLRSGQQSQSMSDLIGSAGKSGPRRTKGLVMGDGISLQRKG
DVLANQDTCVAALRALRQIILTSGTLLKEDLHKRIQDLVVPLCVRLQQQSHCVLDVGAVS
GQYGSPTPRRELYRLLLALVLVPSPRWPPPLSCAVSVFSHGRRDHNIMVSSFCAEALTIC
NTLIHPRTPSISLPLTPLTLKSTPSAPVLASGQNPSLSIPTLLGGPPFPSRHPMGLGPAT
LLGSLENHLPLAPPGLPTPAGTTATPGDLLLSPAQPGELAGLGAPEGQRQVFVRYEKEEP
EDVEISLESDSDDSVVIMPAGMMMEMQDGAANAQSLSQPAVPAPIVGEVGLVDTLLPNEL
PTSIPHQILPANANNINSFPGPSQTAQLVSLVPPLNSTTASLSASPAGLADSLTGGPQLQ
QMLMQTSPGGQPPTLGLSLQIQLQNQIAQTSRQLQQQHPANEVDQNVININSSDDEEEEL
EEDELGEEEEEEEGLEEEEEEEEGSDLMDEEYYEGEEFDEYEDEEEEEESEEIQPLEGDN
SRGMMDEEEAEVMIEAEDQQGMEMFCMEREREVEPGIEEMEGVRSVYADDRIKDKGTVEE
IENIGAVERNEPVVGKEQIESLVISGETEGHEEDTSVEAVEPEVKTCEQEVAKPEDPTEN
AGPSQQGQELTVEVEVQKQEPELQPEDTTNQSAPSTSKQEALQSVAVTAEEEEEVEKESG
EQGEDSDARGTKRKMEDREEGESSEQGTEKKKVDEAMASMLADFVDCPPDDDDHGASQSQ
S
>Bos taurus [A0AAA9RT44]
MLSVAFPFSFHLVNYNSQGLLRMLAARPHRVTTCGKTEYRKFPQSKAIQTCPPSLHCCGL
FNFFYKNFLSVCGVLGDDGRTSGVLQTGQPWTLCLPIPLRHLSEPGENDLAKSSEVKEQV
SGVRGMMAVPPCEDDQGVQIDCYCGENLSAVGALVGLSNARLGSIKTRFEGLCLLSLLVG

ESPTEMFQQHCVSWLRSIQQILQSQDPPPTMELAVTVLKDLLRYAAQLPAVFRDISMNHL
PGLLTSLLGLRPECELSALEGMKACMTHFPRACGSLKGKLASFFLSRVDALSPQLQQLAC
ECYSRLPSLGAGFSQGLKHTDSWEQELRSLLASLHSLLGGLYEGAEAAPMQYESPGAETL
LSSSEDADAHTLLRLRQRFSGLARCLGLMLSSEFGAPVSVPVQDILDLICRTLSVSAKNV
SLLGDGPLRLLLLPSLHLEALDLLSALILACGARLLRFGALISRLLPQVLNAWSIGRENL
GPGQERPYSTVRTKVYAVLELWVQVCGASAGVLQGGASGEALLSHLLSDISPPADALRLR
SPRGSPDAGLQTGKPSAPKKLKLDVGEAIAPPSHRKGDSNANSDVCAAALRGLSRTILMC
GPLIKEETHRRLHELVLPLVMGVQQGEALGSSPYTSSHCRRELYHLLLALLLAPSPRCPP
PLACALRAFSLGQREDSLEVSSFCSEALVTCAALTHPRVPPLQSVGPTCPAPAPVPPPEA
PAPFRAPAFHAPSPLPSAGPMPSAGPMPPVGPLPPTRPGPPATANHLGLSVPGLVSVPPR
LLPGPENHRAGSSEDPVLAPSGSPPPTIPPDETFGGRVPRPAFVHYDKEEPSDVEISLES
DSDDSVVIVPEGLPPPPPPSSGTTPPPVAPAGPPAASPPVPAKDEPEELPAAPGPLPPPP
PPPVPGPVTLPPPQLVPEGTPGGGGPPALEEDMTVININSSDEEEEEEEEEEEEDEEEDE
EEDFEEDEEEEEEYFEEEEEEEEEFEEEFEEEEGELEDEEEDEEEDEELEELEEVEFGPAG
GEVEGGGPAPPSLPPALPPAESPKGPPEPGLEPGLLLEVEEPGTEEAPGPETAPMLAPEV
LPSQGEVEREGGSPPAGPPPQELVEEEPSGPPALLEEGAEGGGDKVSPPPEASAVEETEV
EAAALPPEKEQGDTAAMLADFIDCPPDDEKPPPASEPDS
>Sus scrofa [A0A8D0JWQ7]
XXXXXXXXXXXXXXXXXXXXXXXXXXXXXXXXXXXXXXXXXXXXXXXXXXXXXXXXXXXX
XXXXXXXXXXXXXXXXXXXXXXXXXXXXXXXXXXXXXXXXXXXXXXXXXXXXXXXXXXXX
XXXXXXXXXXXXXNLSAVGALVGLSNARLGSIKTRFEGLCLLSLLVGESPTEMFQQHCVS
WLRSIQQVLQSQDPPPTMELAVAVLKDLLRYAAQLPTLFRDISMNHLPGLLTSLLGLRPE
CELSALEGMKACMTYFPRACGSLKGKLASFFLSRVDALSPQLQQLACECYSRLPSLGAGF
SQGLKHTESWEQELHSLLASLHGLLGALYEGAETAPMQYEGPAVEALLSPSEDGDAHVLL
RLRQRFSGLARCLGLLLSSEFGAPVSVPVQEVLDVICRTLSVSARNISLLGDGPLRLLLL
PSLHLEALDLLSALILACGGRLLRFGALISRLLPQVLSAWSIGRDTLSPGQERPYSTMRT
KVYAVLELWVQVCGASAGVLQGGASGEALLTHLLSDISPPADALKLRSPRGSPDGGLQTG
KPSAPKKLKLDMGEPIAPPSHRKGDSNANSDVCAAALRGLSRTILMCGPLIKEETHRRLH
ELVLPLVMGVQQGEVLGSSPYTSSRCRRELYRLLLALLLAPSPRCPPPLACALQAFSLGQ
REDSLEVSSFCSEALVTCAALTHPRVPTLQSMVPTCPTPAAVPPPEAPSPFRAPAFHPPG
PMPSAGPMPSAGPMPSVGPMPSVGPMPPARPGPPATANHLGLSGSSLVSVPPRLLPGPEN
HRAGSNEEPVLAPSGTPPPTLPSDETFGGRVPRPAFVHYDKEEASDVEISLESDYSVPEG
LPPLPPPPPSGTTPPPVAPAGPPAASPPVPAKEEPEELPAAPGPLPPPPPTPVPGPATLP
PPQLVPEGTPGGGGAPALEEDLTVININSSDEEEEEEEEEEEEEEEEEEEEEDFEEEEED
EEEYFEEEEEEEEEFEEEFEEEEGELEEEEEDEEEEEEEELEEVEELEFGSAGAEVEEGG
PPPPSLPPALPPAESPKVQPEPEPEPGLLLEVEEPGAEEAPGPETAPTLVPEALPAQGEA
EREAGSPPTAPPPQELVEEEPSVPPTLLEEGAEGGGDKVPPPPETSAAEEMETETESTAL
QEKEQDDTAAMLADFIDCPPDDEKPPPAAEPDS
>Ciona_intestinalis_P008744
ISWLHICCETATSPHEDDSSRQVAAENFHEILKYSKNLAELSRKLSTETISQIMSKLISLKPTDHSNKHFLICLEAVLKFFPGPCGPFKGKIAK
CLASSMLSLESELRKFSVRCWSYIPVLGGGGTGRRNHVMQWQQQITDILDCLDHICSCLFDDHHNEEILDPSLLMLSIQFPPLSKEEPLLSNQL
IQRLFTFCECLSSLLEVNLPAQVEIPFERIFYFLLSCFNVNPKQLGFTSEKILRSSYLCDIHMSCLNVFDVLTRQCRQHISRHSSALNQIFLNL
LTLWRDRTPMAGRTNLFSEIRLQIYNSVSCWIKCCGSKSNFLCGESHVVEPMLELLIKDILPTPDNIKLSGQGENLYEKNRNKNKLNNLTGLSG
SRIFDNSSNWEVCTKALEVGRLCVLYLGPQLSVNVHKMLQESVVAICMKLIQKDPNLQPYNSCESVRLQLYHLLLSLTISSSPSWPSPLQYSSR
IFSHGSNHDQSPDVRFFCTEACITLTHVTHPRSAPMHRTSST
>Nomascus leucogenys [G1S6D6]
MLLFSLSPRVQPAFTQCAGPQPPPHAPSPVGGRRGVFASSRHAITGARGKMAAAVLSGPSAGSAAGVPGGTGGLSAVSSG
PRLRLLLLESVSGLLQPRTGSAVAPVHPPNRSAPHLPGLMCLLRLHGSVGGAQNLSALGALVSLSNARLSSIKTRFEGLC
LLSLLVGESPTELFQQHCVAWLRSIQQVLQTQDPPATMELAVAVLRDLLRYAAQLPALFRDISMNHLPGLLTSLLGLRPE
CEQSALEGMKACMTYFPRACGSLKLACECYSRLPSLGAGFSQGLKHTESWEQELHSLLASLHTLLGALYEGAETAPVQNE
GPGVQMLLSSEDGDAHVLLQLRQRFSGLARCLGLMLSSEFGAPVSVPVQEILDFICRTLSVSSKNISLHGDGPLRLLLLP
SIHLEALDLLSALILACGSRLLRFGILISRLLPQVLNSWSIGRDSLSPGQERPYSTVRTKVYAILELWVQVCGASAGMLQ
GGASGEALLTHLLSDISPPADALKLRSPRGSPDGSLQTGKPSAPKKLKLDVGEAMAPPSHRKGDSNANSDVCAAALRGLS
RTILMCGPLIKEETHRRLHDLVLPLVMGVQQGEVLGSSPYTSSRCRRELYCLLLALLLAPSPRCPPPLACALQAFSLGQR
EDSLEVSSFCSEALVTCAALTHPRVPALQPMGPTCPTPAPVPPPEAPSPFRAPPFHPPGPMPSVGSMPSAGPMPSAGPMP
SAGPVPSARPGPPTTANHLGLSVPGLVSVPPRLLPGPENHRAGSNEDPILAPRGTPPPTIPPDETFGGRVPRPAFVHYDK
EEASDVEISLESDSDDSVVIVPEGLPPLPPPPPSGATPPPIAPTGPPTASPPVPAKEEPEELPAAPGPLPPPPPPPPPVP
GPVTLPPPQLVPEGTPGGGGPSALEEDLTVININSSDEEEEEEEEEEEEEEEEEEEDFEEEEEDEEEYFEEEEEEEEEEE
FEEEFEEEEGELEEEEEEDEEEEEELEEVEELEFGTAGGEVEEGGPPPPTLPPALPPPESPPKVQPEPEPEPGLLLEVEE
PGTEEEHGADTAPTLAPEVLPSQGEVEREGGSPAAGPPPQELVEEEPSAPPTLLEEETEDGSDKVQPPPETPAEEEMETE
TEAEALQEKEQDDTAAMLADFIDCPPDDEKPPPPTEPDS
>Homo sapiens [E7EV54]
MELAVAVLRDLLRYAAQLPALFRDISMNHLPGLLTSLLGLRPECEQSALEGMKACMTYFPRACGSLKGKLASFFLSRVDA
LSPQLQQLACECYSRLPSLGAGFSQGLKHTESWEQELHSLLASLHTLLGALYEGAETAPVQNEGPGVEMLLSSEDGDAHV
LLQLRQRFSGLARCLGLMLSSEFGAPVSVPVQEILDFICRTLSVSSKNISLHGDGPLRLLLLPSIHLEALDLLSALILAC
GSRLLRFGILIGRLLPQVLNSWSIGRDSLSPGQERPYSTVRTKVYAILELWVQVCGASAGMLQGGASGEALLTHLLSDIS
PPADALKLRSPRGSPDGSLQTGKPSAPKKLKLDVGEAMAPPSHRKGDSNANSDVCAAALRGLSRTILMCGPLIKEETHRR
LHDLVLPLVMGVQQGEVLGSSPYTSSRCRRELYCLLLALLLAPSPRCPPPLACALQAFSLGQREDSLEVSSFCSEALVTC
AALTHPRVPPLQPMGPTCPTPAPVPPPEAPSPFRAPPFHPPGPMPSVGSMPSAGPMPSAGPMPSAGPVPSARPGPPTTAN
HLGLSVPGLVSVPPRLLPGPENHRAGSNEDPILAPSGTPPPTIPPDETFGGRVPRPAFVHYDKEEASDVEISLESDSDDS
VVIVPEGLPPLPPPPPSGATPPPIAPTGPPTASPPVPAKEEPEELPAAPGPLPPPPPPPPPVPGPVTLPPPQLVPEGTPG
GGGPPALEEDLTVININSSDEEEEEEEEEEEEEEEEEEEDFEEEEEDEEEYFEEEEEEEEEEEFEEEFEEEEGELEEEEE
EEDEEEEEELEEVEREGESPAAGPPPQELVEEEPSAPPTLLEEETEDGSDKVQPPPETPAEEEMETETEAEALQEKEQDD
TAAMLADFIDCPPDDEKPPPPTEPDS
>Nematostella vectensis [A7S5S7]
MAADVAVVVNALLNEDSTPEKYISTVLDCLEEKKSLQDSRTTSEWIGKVNGYLNSKKERSLGLCMLEATIEQCDTDAFNANCCTWVRALLQIIQ
SSPESRTTIHCAIQLLENILQYAVQFSELSRTLATSILPSILGALLSLPDQLDSLSGLSACMRHMPGPAGAVKVQIEDLVVSCFEHSNENIRKA

SSECFALLASLSSGGGGQNKQQINWELQWRRLLLSLKQSLEMLYEDTSEHSSSESRVQLGELLSLKDVPDEEPSRTMTLATRCAAMLDALTAMT
RTGISRFAVIPLEQTLQLVERVSSITEPVLKQSGSLTVVLLRTVLPLVHIPAIDLLSALIQRCGSLLLPFTSEIGNLLTRALGWTGNATNNNHE
QAIPYSKLRCAVYECIINWSHLNCSLSQQMFSKLVASILSDITPQVSQTKLIAPVDQGRSKKAKKRKLQEDVEELATRQRKMNPLCNKSVCNKA
LQGLSSLLMDGVKMPANIFQTAPPSLVPGVLIGFPVVGFLVDGLSSLLMDGVKMPANNFQAGLKILARLCQAGLKILARLCQAGLKILARLCQA
GLKILARLCQAGLKRTKTGLTTRAVIG
>Crassostrea virginica [A0A8B8ET28]
MAASMENVMSQLLCDKNLPNGELVNLLRKINDTHFFQTKKSNLSEIVGPVHTFLNTSRDRVRGLLILSCLVSNCESKDLVQYIETWLRLIIQIL
QTRERVVVFKLTCHVCCQIIRVSTPLQTLRKPMLSFVPQLIPLLVSASPEWRKHSLGVLNACIIHYGGVCSPFKSKIEEVVNHALQQAPITKEA
VLSFCLLSCCGSSGNQKIKYTEAWGSRLSHLLDDLHGILGHFDSEDGFSIDVQMSETDLAPTKEDHQVTFFRTLMVCLQGMLRKSFGATIKLPV
EKILTFVDQVLALNVYSESLLHGYLPLLHTDAVNIINVLFESCRKLMLPYCKRIVQVLNRELLWSHANPGDRGSIHFRELREAVYRALQTMLMA
TGCFRETLSEEDSVLQELLADCKQRSQTMKSSLKPGNGMDPTPAKRMKMQDNPADVNKQPEEDYSTVTHSALQTLYWWIVSSVVKMKGKNFQKI
CDFVVTTALCVYRNRCDPIMPYSDWRCRLHLLKVLQACIMTPHTEGPSPLQCAIAIFKMAEQDNNLQVSSFSMEAQRLVEVLVHSRAVCLTTPR
YQSQEINTSAVVMTQRKTAPDIGASETSPPGPNDVTEEQITQTAKNGKDSVVISDDEDEMDDSDSIMITEQSREEDSEVGGEGQWVIMDTPPSV
LHAASSTQGLTSPLLGPTETQTDRQTEDADHKPSDQVGDKTPDCHKETEESAEDRAMSETEDYDPQSVNVECSDHCGEVLVIQSSTLEASSSNT
DKFTDDSGNIKENPIQSSNLLPTEADHPQGKELEDILLTFVDVDPE
>Crassostrea gigas [A0A8W8IH25]
MAAPMKNMISKHLFDNKLSLDDLIAYLKRIDDTQSLQITKLNLSELTGQTHTFLHTSSNRLRGLFILSSLLRNYDVQELSPHLDTWMKIIIQIL
QSRDPAPVHKLTCHVCCQLIKMSNEIQTARKAMQSYLPQLIPLVIAASPEWRRQSLAVLNACITHYGGTCGPFKSKIEEVVTQELQCVPVTKEA
VLSYCLLSCCGTSGNQKCKYTEGWGARLSHLLSDLHGIIDLITTDDVSNTAVSLSDLDAEPLHADHSVTYFRTLMQCLQKMLRKAFSAPIKLPL
EKILDFVDKVLSMDVSSVKSSSSKDSLLLGYLPIIHTDAISVINVLYEGCRKLMLPYNKKIVRVLSRELMWSHSNSSVTQTRPYRELRVAVYRT
LQTMMMTTGCFLETLREEDSVLQVLLTDCRPGEHSLKTSLKPSHTMVPTPAKKMKTPDSSGDLRRPIGHSPGGDSVVTKGALQALYWWIVAGGV
KMKGKNFQKVCDFVITTLLSVYRNRFDPSVPYNDWSCRLHLLRLLQACLMTPHTEGTSPLQCAIAIFKMAEQDNNLQVSSFCMEAQRLCDLLIH
SRAVCLTTPRYVTQERTSSSPMLTTDFSKTSEDEPMEHLPKSREPGAETQLPSNDVEEVGNEDAVVTGENEADSDVEMEQRREEEEDGEGDPIT
DLPTSLQEPPCQEATDPQTDLQRQAESIVEDLPETRLSVAEGPVADSEATDHNLDPQTDSDCLSVRGENEVNQSPATSGSSDIDKAMEDSSQPK
DNLAQIASSSFVKEINEPQDDDLKDMLMTFVDADPE
>Mytilus coruscus [A0A6J8DVR9]
MDAPMENVIFAFKSEIIKNSKEISDFTCLLQLTDENKFFSLQKTYGLQETISYIHGCLNTSKLRTEGLILLEKLIEQCVTEIFTQNAVTWIRFI
VQCLESHDPVVVKRLSCHVGCKIIPLTVNFTALSKDIISVLPQLLATLISASSEWLTASLKLLGVCIQSYPGPCGTFKTKLEAMVMELFKEQSV
PKEAIALYVLLARCGGGGNKGIRFTEAWSYKFQQIITSLTEIVNLLYNGLNIPELASTFSVPVLPMRELPEMYTNRFNILSSYHHTYSLCLQEL
LSQPFPAPVKIPVSDVIKLVTKVLSVNIRDLTVRPSTERLLLSGYISTLHSDATVRPSTERLLLSGYISTLHSDAVLSLHSLISRCKTLLLPYS
KSIITVMVKELMLFRTKVVYGQNRAHSSLRQIVYRTLVLWLKTTKSFTITTEEEDWLVQEILHDVRPHTDNMKTVSIKSKKSESFEPPPSKKKK
KGGYSEISHGISTQKKVDLEANSELCMVALEVLYWFLTTVGCQLKQKIVEELQDFLISVAITFRCKGNYGIPYADDRCRHSLLRVMQAFNTMPH
PTASSLLHYSLSVLRCTSQDSSLLVSSYSIESLQACEALIHARAPSYNVLKKVSREVIEDKIDIIQQDKGLERSNIFLSGQKTSLFESVGEKHN
KDNGESKKSFTEKRCVEKNVIESNVEDIYERKQKQWGHSDSDSQQSRIIDHTVTMSTNNHGQMKPNLSGSNERQSPMEEQDYTIMDTDVDNTIG
DFMESSENNSEGVKKEEEKHTLSTEDQENIVDMLSTFVDIDEDQ
>Mytilus coruscus [A0A6J8DXE7]
MDAPMENVIFAFKSEIIKNSKEISDFTCLLQLTDENKFFSLQKTYGLQETISYIHGCLNTSKLRTEGLILLEKLIEQCVTEIFTQNAVTWIRFI
VQCLESHDPVVVKRLSCHVGCKIIPLTVNFTALSKDIISVLPQLLATLISASSEWLTASLKLLGVCIQSYPGPCGTFKTKLEAMVMELFKEQSV
PKEAIALYVLLARCGGGGNKGIRFTEAWSYKFQQIITSLTEIVNLLYNGLNIPELASTFSVPVLPMRELPEMYTNRFNILSSYHHTYSLCLQEL
LSQPFPAPVKIPVSDVIKLVTKVLSVNIRDLTVRPSTERLLLSGYISTLHSDAVLSLHSLISRCKTLLLPYSKSIITVMVKELMLFRTKVVYGQ
NRAHSSLRQIVYRTLVLWLKTTKSFTITTEEEDWLVQEILHDVRPHTDNMKTVSIKSKKSESFEPPPSKKKKKGGYSEISHGISTQKKVDLEAN
SELCMVALEVLYWFLTTVGCQLKQKIVEELQDFLISVAITFRCKGNYGIPYADDRCRHSLLRVMQAFNTMPHPTASSLLHYSLSVLRCTSQDSS
LLVSSYSIESLQACEALIHARAPSYNVLKKVSREVIEDKIDIIQQDKGLERSNIFLSGQKTSLFESVGEKHNKDNGESKKSFTEKRCVEKNVIE
SNVEDIYERKQKQWGHSDSDSQQSRIIDHTVTMSTNNHGQMKPNLSGSNERQSPMEEQDYTIMDTDVDNTIGDFMESSENNSEGVKKEEEKHTL
STEDQENIVDMLSTFVDIDEDQ
>Mytilus coruscus [A0A6J8DW64]
MDAPMENVIFAFKSEIIKNSKEISDFTCLLQLTDENKFFSLQKTYGLQETISYIHGCLNTSKLRTEGLILLEKLIEQCVTEIFTQNAVTWIRFI
VQCLESHDPVVVKRLSCHVGCKIIPLTVNFTALSKDIISVLPQLLATLISASSEWLTASLKLLGVCIQSYPGPCGTFKTKLEAMVMELFKEQSV
PKEAIALYVLLARCGGGGNKGIRFTEAWSYKFQQIITSLTEIVNLLYNGLNIPELASTFSVPVLPMRELPEMYTNRFNILSSYHHTYSLCLQEL
LSQPFPAPVKIPVSDVIKLVTKVLSVNIRDLTVRPSTERCKTLLLPYSKSIITVMVKELMLFRTKVVYGQNRAHSSLRQIVYRTLVLWLKTTKS
FTITTEEEDWLVQEILHDVRPHTDNMKTVSIKSKKSESFEPPPSKKKKKGGYSEISHGISTQKKVDLEANSELCMVALEVLYWFLTTVGCQLKQ
KIVEELQDFLISVAITFRCKGNYGIPYADDRCRHSLLRVMQAFNTMPHPTASSLLHYSLSVLRCTSQDSSLLVSSYSIESLQACEALIHARAPS
YNVLKKVSREVIEDKIDIIQQDKGLERSNIFLSGQKTSLFESVGEKHNKDNGESKKSFTEKRCVEKNVIESNVEDIYERKQKQWGHSDSDSQQS
RIIDHTVTMSTNNHGQMKPNLSGSNERQSPMEEQDYTIMDTDVDNTIGDFMESSENNSEGVKKEEEKHTLSTEDQENIVDMLSTFVDIDEDQ
>Dreissena polymorpha [A0A9D4G4M3]
MAAPMESMIEKLLNNSFQKYAVQRSGDLQLQSFVKVIETNKLLLKRDDEGSNDIVGYIHQCLNNSKQRYEGLILLGVLVKQSSTAIFQKNAVTW
LKLLLNILQSHCPPPVHQACCKLLSQVIDHSDSFSDVSREVTSTFIPQLLPLLLTCEPGCLSAALTCVSMCIRKYPGPVGPFKAKLQTLVEQKL
ANGKNYEELYECFALLASCGSGGNLGIKHTEAWSELAEGVLASLHETLSCMYSDVDMGSGSSCTGKPLAGLPPIDFSHNDRVVALFKRWCSLTK
CMQALLREDFNAVARVPADDILNIIFHCLSVNTNMLMSRPTSERSLLASFSQDVHIHALYLLQQLITLCGRNLIPQGVMIVSVLTQELSWSHSE
GDGHDQHYGCLREVVFETLTVWIKALDACSSVKSGETTLVKEIIHDCLPYTYTLKMTSGLSTGSKTDKMTSGQGSRKGHKGKGYHEISQGISTQ
RKSQPLANHSLVKAALEALYWMLTTSGWEFSQKSLQSVQEFVIRSLLALHQAPLGSLPAPYADPSCRRGLYRVLSACVTSTHANVPPPVQVALG
LFSAGLRDKHADVSSYCMEAMRLCEVLIHHRVPCLKGPVVCENLIQISGQNDQEPIVSHVINKSGNQAHISLETKNGYSDASSEFTRNEEAIAG
RTAGNITYNGGFSEREIRKEKGKE
>Mytilus coruscus [A0A6J8DV91]
MDAPMENVIFAFKSEIIKNSKEISDFTCLLQLTDENKFFSLQKTYGLQETISYIHGCLNTSKLRTEGLILLEKLIEQCVTEIFTQNAVTWIRFI
VQCLESHDPVVVKRLSCHVGCKIIPLTVNFTALSKDIISVLPQLLATLISASSELLATLISASSEWLTASLKLLGVCIQSYPGPCGTFKTKLEA
MVMELFKEQSVPKEAIALYVLLARCGGGGNKGIRFTEAWSYKFQQIITSLTEIVNLLYNGLNIPELASTFSVPVLPMRELPEMYTNRFNILSSY
HHTYSLCLQELLSQPFPAPVKIPVSDVIKLVTKVLSVNIRDLTVRPSTERLLLSGYISTLHSDAVLSLHSLISRCKTLLLPYSKSIITVMVKEL
MLFRTKVVYGQNRAHSSLRQIVYRTLVLWLKTTKSFTITTEEEDWLVQEILHDVRPHTDNMKTVSIKSKKSESFEPPPSKKKKKGGYSEISHGI
STQKKVDLEANSELCMVALEVLYWFLTTVGCQLKQKIVEELQDFLISVAITFRCKGNYGIPYADDRCRHSLLRVMQAFNTMPHPTASSLLHYSL
SVLRCTSQDSSLLVSSYSIESLQACEALIHARAPSYNVLKKVSREVIEDKIDIIQQDKGLERSNIFLSGQKTSLFESVGEKHNKDNGESKKSFT
EKRCVEKNVIESNVEDIYERKQKQWGHSDSDSQQSRIIDHTVTMSTNNHGQMKPNLSGSNERQSPMEEQDYTIMDTDVDNTIGDFMESSENNSE
GVKKEEEKHTLSTEDQENIVDMLSTFVDIDEDQ
>Daphnia magna [A0A0P5ZPZ6]
MDGLLNLLPLYTESVEGEHNLWQFLETCKSHQSFGELVANETDLNHAVGVINGKLNNPNTRAEGALLLEVVITQCGTDMFTSNCVLWTQQVMRL
LHGPTKRTVGQNVFKVLGKLLAFSSQFPELSRQLSTTVISQLVTLLCEPEFSQKASIAVHVLECLKSCMKYYGNPCGPVKGAIERHLLSLIDWD
EMATDSSLRAVWAACMAYLPSVGSSGSQGAQHRSNWIEFCLQLIDSIHFTINGMFRNIEELKIQEVSSNPLKLPELQHKNPMVLLHDQQRRFVN

LCSALVVLLNEPFPTTKNVPIDRFLAMISRLLALNSKSLSKTSRANFEQLTLASIIPDLQSSMLDVLKSLVAVCRTQLLTRATTVMNFFVQVLQ
WTFTPVDRRRVGIERPYGKLRSQVYRNLCLWVVASRSACGWGKCVDVLFSQLLSDILVHRDTVQLLTVNTPSNAKMTRKGKKKMDTTGVILQKD
DLTANADVCQMALQCLSTILLCCGPRIKPAIHKEMQEIVLSILVDIMNGAELNLLVPYNDPRCRAAMYRVLEKLVLCPSPQWPAPLNYASAIFS
SGMNDPNIEVSSVCIEALASIQSILRPRGPTINFALEEKQLRSIQQEVPLLNGSVKLVEPSSAASAMVASQPILAVKSVGQATIPTDAALINHM
ATPASKIHENSLDLMIEIRQPESSPLPSNRLSISNGSHDDDFSTPPMGVTTISSESPVKSTRMMTRKEALKVIAAVALPVQSVKEKSPIPSEIK
QVKNETSPARKRPYEDSKPSTFSPPEKMQTEELENGEDELDDNVDAMLASFHDIPA
>Daphnia galeata [A0A8J2S4H9]
MDGLLNLLPLYTDSVEGEHNLGQFLETCRAHQSFGELVANEIDLNHAVGVINSKLNNPDSRIEGALLLDVVITQCGTDTFTSNCVLWTQQVLRL
LQGPSKISVGRTACKVLGKLLAFSSKFPELSRQLSTTVISQLVALLCEQEFLQKASITVHVLECLKSCMKYYGNPCGTVKGTIERHLLSLIDWD
VMATDPSLRSTWAACMAQLPSVGSSGSQGAQHRSNWIEFCLQLVDSIHCTINGMFRNIEELKTQEASSNPLKLPELKHKNPMVLLHDQQRRFIN
LCSALVFLLNEPFPTIKNVPIDRLLAMISRLLALNSKSLSKTARANFEQLTLASIIPDLQSSVLDVLKSLVAVCRSQLLTRATTVMNLFVQVLQ
WTFTPLDRRRVGAERPYGKLRSQVYRILCLWVAASRSACGMGKCVDILFSQLLSDILVHRDTVQLLTVNPLSNGKTTTKKGKKKMDTPGVILQK
DDLKANADVCQMALQCLSTIFLCCGPRIKPTIHKEVQEIVLSILIEIMNGAELNSLLPYNDPRCRTGLYQVLEKLVLCPSPQWPAPLNYASAVL
NSGLNDPNLEVSTRCIEVLASIQSILRPRGPTLNFAMEMKQLRSVQQEAPLLNGWSSKFVESSLPTSAPSQPAIAAGSIENPPVVVVSIDQPST
PPRRQIPPNSIDSSIEIRQPETSPPPSKRLSLTKSNEEQDEDFLTPPMGVTTISSESPVKSTRVMTRKEALQVLVSQSATKQSANHPKPKQIKN
EASPTRKRPHENSKRESAEEKMETENDENGVDELDDNLEDMLASFHDVPA
>Daphnia sinensis [A0AAD5LFD2]
MDGLLNLLPLYTESVEGEHNLWQFLETCKSHQSFGELAANETDLNHTVGVINGKLNNTSTRAEGALLLEVVITQCGTDTFTSNCVLWTQQVMRL
LHGPTKMTVSPTVFKVLGKLLAFSSQFPELSRQLSTTVISQLVTLLCEPEFSQKASITVHVLECLKSCMKYYGNPCGPVKGAIERHLLSLIDWD
EMATDPNLRTVWASCMAYLPSVGSSGSQGAQHRSNWIEFCLQLIDSIHFTINGMFRNIEELKIQEVSSNPLNLPELQHKSPMVLLHDQQRRFVN
LCSALVVLLNEPFPTTKNIPIDRLLAMISRLLALNSKSLSKTARANFEQLTLASIIPDLQSSMLDVLRSLVAVCRTQLLTRATTVMNLFVQVLQ
WTFTPVDRRRVGIERPYGKLRIQVYRTLCLWVAASRSSCGWGKCVDVLFSQLLSDIIVHRDTVQLLTVNPPSNGKMTRKGKKKMDTTGVILQKE
DLTANADVCQMALQCLSTILLCCGPRIKPAIHKETQEIVLSILVDIMNGAELNSLVPYNDPRCRAGMYRVLEKLVVCPSPQWPAPLNYASAIFS
NGMNDPNIEVSSVCIEALASIQSILRPRGPTINFALEEKQLRSIQQEVPLLNGSVKLVEPSSTSSAMAAAISVGQPTIPTDAALINQVTTPAKT
IHENSLDFTVEIRQPESSPLPSNRLSISNGSHDDDFSTPPMGVTTISSESPVRSTRMMTRIEALKVIAAVALPTQSIIENSPIPSEMQQVKNET
SPARKRPHEDSKPTVFSPQETMGEMENGEDELDDNVDAMLASFHDVPA
>Artemia franciscana [A0AA88IAQ7]
MPSMESILPASTVSEINRLLNSSGTRLDGLNTLELSLNYFSLDVLNDNASQWIVQLLRIMRAGDYGFQKAAKALGNLLEISASSSELSRDLSSA
IPQVINVLGNPKQKEENLPASFDCLATVLKNFRGPVFPFKNAMEDLLLSYFDLVDLIAVSSLAKCYAQLPQIGGSGIKGLSHKQAWSEQSDKVI
LSLKSTLKSFLGETVSEEGLNLPKMTAVDPITDMHRMAVRYRNLCMVLSRMLSDPFPAPKCIPVASILSLSNEVFCLSPLALSKRIGGNTSEGK
ERQKLFPDLQVVMLQLLNNLIKSCRKQLLPHGRSICSFALQVLQWSHTPLELREVGRDRPHRKLRSAAYNTLAHWVNASNTGSRIEKFAIDLSK
EIFSDIVTDRDVVALQKVEISAKKKRGSLSYDTVKPIDHHIDRLANAEVCNAALTLLGRILRSLSSIITLEFYQMAQKIILPIVIELEAGIEES
PIPYSDSAVCRKSIYKVLEALVVCPHSSGYPPYSYAISAFSCGQNDPDLEVSSTCFDALKSCEAIIHPVTVPVGFAMKPPVNENITSESMVESS
ISKLWKTVEEDITRKSTVEPSKITDVNGRASASIYVQSHSEERVTSIVDEKMEDDDQDRMDSVSNISTSPEHNNFDSDVDIADTPLMKEVVDCQ
TLLHLHYPQQLRMYGQYYGRAQENR
>Artemia franciscana [A0AA88I454]
MPSMESILPASTVSEINRLLNSSGTRLDGLNTLELSLNYFSLDVLNDNASQWIVQLLRIMRAGDYGFQKAAKALGNLLEISASSSELSRDLSSA
IPQVINVLGNPKQKEENLPASFDCLATVLKNFRGPVFPFKNAMEDLLLSYFDLVDLIAVSSLAKCYAQLPQIGGSGIKGLSHKQAWSEQSDKVI
LSLKSTLKSFLGETVSEEGLNLPKMTAVDPITDMHRMAVRYRNLCMVLSRMLSDPFPAPKCIPVASILSLSNEVFCLSPLALSKRIGGNTSEGK
ERQKLFPDLQVVMLQLLNNLIKSCRKQLLPHGRSICSFALQVLQWSHTPLELREVGRDRPHRKLRSAAYNTLAHWVNASNTGSRIEKFAIDLSK
EIFSDIVTDRDVVALQKVEISAKKKRGSLSYDTVKPIDHHIDRLANAEVCNAALTLLGRILRSLSSIITLEFYQMAQKIILPIVIELEAGIEES
PIPYSDSAVCRKSIYKVLEALVVCPHSSGYPPYSYAISAFSCGQNDPDLEVSSTCFDALKSCEAIIHPVTVPVGFAMKPPVNENITSESMVESS
ISKLWKTVEEDITRKSTVEPSKITDVNGRASASIYVQSHSEERVTSIVDEKMEDDDQDRMDSVSNISTSPEHNNFDSDVDIADTPLMKEVVDCQ
VSQNELPALVPVTLSIKESQAVHLEKEAKEADNESSVASDVEDMLTDFVDIGP
>Strigamia maritima [T1J4S9]
MDITSLLALFTDANVPKSCTHLWIEMMRTHGNLSKDGPEIKSVIKYLDNALKKYKSDVTGPLMLLNYLVEDCSDSVFVENCMSWLDKLSKIIQV
NCNVKIVSLACDVVINIISFVVQNSDLNRQFCNSVLSNLVTSLLDINEHKLCCLRKVMKTFPGPCRQFQIKIENLVMEIFLESTNQQLQREAAQ
CFLILPNLNLESDVGWNECFKKILATIHNILTELFETVERGDPIWKSDYTESLSFVAIPNAQPMLKFQSLYQRFNLLCYCISEMLSGVYSGIKQ
IPVRDVIGFVTRVLIITPFSIKSATTENLMLAGVLPLLHEYAFRVLHSLIIVCRKYLIPESSTISKLIIKGLKWTSRVQSETTSKKPFSDLRCQ
IYSTLTLENSSVENNRKKRKRGNTFSGVGSVGPIKEDPLVNNSICLNCLKALKAVHLAIGSTISSAVYLEITKFTIHLMCDVQKTISNRPTPYD
DFKCRHALYDVILAFVLVAPQETGSCIQLCMKIFSCGRQDAYQKISSLCLQAQSTCLEIIHPRLPPLQFDMKCRKQVIHKSDEAEEQNGIDEEE
NIEMTEEFAQNGVEIRVEGNTEKTEGLEQNEQTTKGDIEMTEELKQNGLEKEETITHENKRSKIMSDSTESTAPIIANDDFQVVVTLESEVILP
KDESEENSIADSDIEIVSSSLNSSPSKKPNPLENLTITPKALPIGVNESPVSPTKDEANLTVEEMLQDFVNDPIE
>Capsaspora owczarzaki (strain ATCC 30864)[A0A0D2WKE6]
MLTLLGGATSSASDASASAAASSSSGHPSSAGAAATPANSMMLKMLAETTAAAPSGSGAGSQSSHDWCLKLLQAISHVMKSKSAAASLDAALTC
LARVLESHLPAELRRDILSTHLARLVQQTTALASSAGLTTQVAAFGALSAMIRSLSSAMRPHVDKVEAVCSEALDSVHIDVVRAAASCLALCPT
CGGSSSTYTQANPSDAWLVLANRALLTATKLLDVVRIDETAYLAPAVAGTPSSSASSSAKATKTNGGSKGNKSAKNGQSGALPMDESTTEPVVA
NNVAAGVLLPLFPYLDQQQQLAQPAAGTNTSISSLSQAALARDANVNSPTLSALIARRIQATFIVLAAMLQQPVSFVVSMPTHRILSLRHVLYL
SEQQLLQEASKRPNKLAWIAWLPFLQHCTLDLLNVAITRCGAGILGDADDMLQTLVHVLEQHRRLQPVHRQRKAETLVAALTTLETLLSTLGGM
LDATQLGSVGELLCEILCGGALFQAVSPATVPQSHLSGKKQKQQQQQKNIEESLGAASGALAVSRPLQFANTDICKLSLKALTRLLYTAGSRLP
DAVRARIDATASRLVLETQLGAEAISLLSMQRPACSPMVEPTCRRALYRLLLACVVASASPVHSPVLMQVLRLLTTGTRDPALKVSTFCREAVA
VCDNIIRPKFPTLRNGVAVANAIIAPYTPSVASTNDSTLTSILAPVRVDKPPMSTFGFGVPTTLPAPRPATVQAQPAPAPAPEPVAPASSVSAP
AAPVDAAPVKSTASTPALVPAAVSKSESVTVSADATATPTRTKRGREEDRESVAEATASAPSKRIAAESLDEPSRPEVLFAACQPSIPPTVAPV
VPPAAPIVNKHASSTSSVPAKSAVDDDDDDSEFMIVDEDPDSD
>Capsaspora owczarzaki (strain ATCC 30864)[A0A0D2VJA3]
MDASDLPQEAALQLLLANWMTDEIALHHVPHILASLSSLHLTHEAYHRLFKRLTALARSEVQVARCSSLLILAAIIPDGMLTLLGGATSSASDA
SASAAASSSSGHPSSAGAAATPANSMMLKMLAETTAAAPSGSGAGSQSSHDWCLKLLQAISHVMKSKSAAASLDAALTCLARVLESHLPAELRR
DILSTHLARLVQQTTALASSAGLTTQVAAFGALSAMIRSLSSAMRPHVDKVEAVCSEALDSVHIDVVRAAASCLALCPTCGGSSSTYTQANPSD
AWLVLANRALLTATKLLDVVRIDETAYLAPAVAGTPSSSASSSAKATKTNGGSKGNKSAKNGQSGALPMDESTTEPVVANNVAAGVLLPLFPYL
DQQQQLAQPAAGTNTSISSLSQAALARDANVNSPTLSALIARRIQATFIVLAAMLQQPVSFVVSMPTHRILSLRHVLYLSEQQLLQEASKRPNK
LAWIAWLPFLQHCTLDLLNVAITRCGAGILGDADDMLQTLVHVLEQHRRLQPVHRQRKAETLVAALTTLETLLSTLGGMLDATQLGSVGELLCE
ILCGGALFQAVSPATVPQSHLSGKKQKQQQQQKNIEESLGAASGALAVSRPLQFANTDICKLSLKALTRLLYTAGSRLPDAVRARIDATASRLV
LETQLGAEAISLLSMQRPACSPMVEPTCRRALYRLLLACVVASASPVHSPVLMQVLRLLTTGTRDPALKVSTFCREAVAVCDNIIRPKFPTLRN
GVAVANAIIAPYTPSVASTNDSTLTSILAPVRVDKPPMSTFGFGVPTTLPAPRPATVQAQPAPAPAPEPVAPASSVSAPAAPVDAAPVKSTAST
PALVPAAVSKSESVTVSADATATPTRTKRGREEDRESVAEATASAPSKRIAAESLDEPSRPEVLFAACQPSIPPTVAPVVPPAAPIVNKHASST
SSVPAKSAVDDDDDDSEFMIVDEDPDSD
>Ambispora leptoticha [A0A9N9C5W4]

MAQQRAPELILHSLLVNYLSKEDNIESNVPFLLETITFHQLLSSISSDGSGSGKTDSNNDTAEKIKTPLHKWCTRVNSLLLSKVPSARWAGVCF
VKVSIEQSWLLFTENLSQWSSALLSLLARPEPVTTLRVTISTLSEMFSKTVDRPELQREVTSQLLPRFNSCLINLSGNKDLLQFPSMFRPILQN
AQGLCLSIFDNNSDDSYDSDSEVVKKAAKCYANICNAGGKISASDQWQLSLTWLVGSAHVILDRLFDTMDEENKFTKKQQPHPPGQHQFEFLKV
TDDYIIGFPILFKRFRILCQFIVSMLSSTNLASVQVPVNLLMGLICRVCNVCDGGSDNKDKNEFSILLIGLPTLQLAINRVLGSLILCLVRAST
YNLFNLCIQKYRNGFLLSSSSSSSLIFNIVEDLTINDLQIAALEVLKTSLSTTGSSSIPPASRTLLDNLLLSRILGNNNTKTNPKIKLRLYECL
LASVIAPSEYQANILPYALRIYTSGINDDSLEIQSFCSYALTICDLIYHSRLPPIQKSIPSTSTSNSSSIEPSLERQNITNEVSSSASILSSTT
TTTTKSEKKNCRLQPIQKSIPSTSTSNSSSIEPSFIEPSLERQNITNEVSSSASILTSTTTTTKSEKKRLFPSEFENASQPRFKMYRDNNHDGP
DSEYDINNDEKEDNDYSMLE
>Rhizophagus clarus [A0A8H3QT74]
MNSTESPKFLTTLIANYTFNDNKIEAHVPFILETITQHQLLSSKNEPTREQQVALHKWCTRINSLLQSKVTNARWAGVCFIKISLKQSEELFVQ
NLQSWTTSLMVLLTKSEPTFILKEIITALTEIFNKTQNKPELQREITTQQLPRFNTFLIKLSGLNKELLPTILNALSHSVKNFPTIFRPVNDQT
QKLCLNILLDDTCYYESELVKMAIECFPRIINFGGKLNMDYWKLTLLKLVGSLNNILDRLFDTIDEEKGISKKLTGFEMPQVSEDYVVAFPILF
SRFKCLSECLISLISLPTSSPVQIPVNQILDLLYRVYNIYDVSFVTDSKDKNEYYTLMLGIPSLLINCNKVLSAVTLSVGEHLTRHLRMVSSIL
LKLLNNSKTRWLLRVSTYNLISLCMQKYGIGLTNFISSSILLSFVIDDIEIIQKSQYDVSINTTVSTNNKKSGKKKESTQITNSDALVSSSGII
YLQTNSDVQCSALEVLKILLKTCGSSISSNKRLSLDNILLSRILLRNISSTYNSKYQPTILPHALRIFSAGQNSQSIQLRSFCSYALSICDLIF
HNRLPPLERTSANISITQQLEHTTIENISQLSYIENNDSGNMAVLDQQPQKTFDIVSEETNSLITEQNTINKLDVNLDKAITVEDSNNALGDDN
KSKPNIIEDDNIPTDDKINMQKDKLPQTTSLEMRIENQKKRIRSPSPVGVKEVLSREDKEEDYDHANKSRRMLDTNDSDDDMEIPEIIMESPDS
EFEDDE
>Rhizophagus clarus [A0A2Z6SEU8]
MNSTESPKFLTTLIANYTFNDNKIEAHVPFILETITQHQLLSSKNEPTREQQVALHKWCTRINSLLQSKVTNARWAGVCFIKISLKQSEELFVQ
NLQSWTTSLMVLLTKSEPTFILKEIITALTEIFNKTQNKPELQREITTQQLPRFNTFLIKLSGLNKELLPTILNALSHSVKNFPTIFRPVNDQT
QKLCLNILLDDTCYYESELVKMAIECFPRIINFGGKLNMDYWKLTLLKLVGSLNNILDRLFDTIDEEKGISKKLTGFEMPQVSEDYVVAFPILF
SRFKCLSECLISLISLPTSSPVQIPVNQILDLLYRVYNIYDVSFVTDSKDKNEYYTLMLGIPSLLINCNKVLSAVTLSVGEHLTRHLRMVSSIL
LKLLNNSKTRWLLRVSTYNLISLCMQKYGIGLTNFISSSILLSFVIDDIEIIQKSQYDVSINTTVSTNNKKSGKKKESTQITNSDALVSSSGII
YLQTNSDVQCSALEVLKILLKTCGSSISSNKRLSLDNILLSRILLRNISSTYNSSNILHNEENVNIKLYECLLYSIISPSEYQPTILPHALRIF
SAGQNSQSIQLRSFCSYALSICDLIFHNRLPPLERTSANISITQQLEHTTIENISQLSYIENNDSGNMAVLDQQPQKTFDIVSEETNSLITEQN
TINKLDVNLDKAITVEDSNNALGDDNKSKPNIIEDDNIPTDDKINMQKDKLPQTTSLEMRIENQKKRIRSPSPVGVKEVLSREDKEEDYDHANK
SRRMLDTNDSDDDMEIPEIIMESPDSEFEDDE
>Glomus cerebriforme [A0A397S9F8]
MRDNAESSQFLTTLLANYTFNDNKIETYVPFILETITQHQLLSNKNELSGREQQVVALHKWCTRINSLLQSKVTNARWAGICFIKITLKQSEES
FIQNLQSWTTTLMVLLTKPEPILILKEIITTLTELFNKTQNKPELQREITTQQLPRFNTFLIKLSGLNKELLPTILNALSHSVKNFPTIFRPVN
EQTQKLCLNILLDGTYEFESELVKMAIECFTHIINFGGKLNMDYWKITLLKLVGSLNNILDRLFDAIDEDKDASKKLTGFEMPRVSEDYVVAFP
ILFSRFKCLSECLISLISLPTSLPVQIPVNQILDLLCRVYNIHDGSLIANNKDKTEYYTLMLGIPSLFMRCNKILSAVILSVDGHLTRHLRMIS
SILLKLLNNSKSRWLLRVSTYNLINLCIQKYGIGLTNFTSSSILLSFIIDDIEIIQKSQYDISINTTASTSNKKSGKKKELSSQITNSDALVNP
SGIIYSQTNSDVQCSALEVLKNYLKKCGASISSNTRSSLDNVLLSRILSQNISSTYNSSNILLNEENVNMKLYECLLYSIISPSVYQPTILPHA
LRIFSAGQNSQSIQLQSFCSYALSICDLIFHNRLPPLERSSANTLITKQLEHTTVENISQQFYTENNQSENEKGVDNINMIGLDQSSQKTFDII
SAQTSSSTMANIDEDISIDVFDGDSNTSDSKNDILTDKIDIDIVEQPQSISSEMSIENQKKRIRCPSPVGVEKELAKKKDHDRVYKSQRIYNTD
EDSDDMEIPEIIMEGPDSEFEDNE
>Ladona fulva [A0A8K0P465]
MEGLTHLFHTVLQSPHRNSCLGDLLLDCAEHQAFSTIDSSELQSVVAQINSNLNEHNNRFCGILILKTFLSQCPDEIFSSNAFSWVSQLMRAIN
DKTELAVDSENELSFLVLRDIIVASSQFPDVSKQLLPLIPKLLTILTSGITKDCEESLLCLQSCMKYYSSQCFSFKAAICKLLLDELTSECESL
VEVAAECFALLPLLGGSSGIKRVSIINGINANYSGEWNQLLSKVIRTLHIILNSFFAGVNEVKGYSEMEDVLPLPSESDDFSVVMKQNVVRKFT
NLSRYLCLMLQNPLPAAKIIPVEEVMGLLCRALAVTCNSLKDKVSSEFLALGARLPEIHSASFSILSVLMAWMHLLPYGSLIVKLVMQSLKWTS
TSKDWPSSIRKPYSTLRCSAYHCISMWLHVSAAASCAEDVAEELINCALIDMVPTREKLKLVSSNIGNKHLGKQKRRKMGVFDLHTDENTPSSF
LSGMRCAVGDGSANSEICCAALEMLSSLLQSVGLFLKPNLHKILQDTTILILIDIQRSTGTISTKQSNNRDILASMIVPYQSDAKCYLALLKLM
LALVLEPHSHKWPSPLGLAVQIFSTGQNDSNIEVATFCATAKRVVERLIHPVAPTLNFPVSLPESQDMSISEENSEFISTEVSNQTKVSVCSAV
GTQTDEVLQKGTEARESTDFRILGNGNHESTETSDLIVLSDDDSKLSRHSVATSPNDFSKSRIPSPDVLFIRTGLADGKLKGEKNVMLPFTYLK
SKGVQCTGSPLLDDRGDTSANSPPLLVVKVPIDKCSTDKMVDFSQQFPYKPMEDVVPIEVNSDDEDEPVEMEMNHVDDCSELEVTAKLGTAAPG
GENGDSGEVMVVDVEPPKAENLHSNSSSVKSPTGKKIIETCKENAAEVEIVELDDTSNTPVTKNKVVELDEEIAELEDKLNSFVDVSSEDD
>Bemisia tabaci [A0A9P0AKA7]
MEQVIDLLQTCLESSQEDFQQILSSFTEHKVFLRKGFKVDSVVSQINALLNSDDNRAKGVLLLHAFLPQCSSSVLATHGFFWMQQCLKSAEQDA
AILILELQTLGHLFTLSAESIDLRKNVVNTLLPKFFDKLDGKGSKEIRAAKMKCLINVMSHYAGACGLRRGIIDKFIQKSLKVMSEDDKEWLAD
ACKCYLLLAQCGGSGNQGSIHKGNWSQQVQVLLNTAHSLLDSLFDSVTEIQVSKTLSADVTEKKLGAMESVPTFENSLARYNFYSSMFYKTMKA
LQIMLLGAFPAPKSIMPIAVFNLICRGLAVNCKTVSKSSLTLDRIALVSVIPFIHVGLLDTLDALIKCCGRNLIPYGSLICKLTCQSLKWTNEE
WEYALEKPYKSVRISTYKALQSWLNCSRLASCIEPFLDEIIELLEKDFKYQEPTIKLQDKNQSGDAKGKRKKITYAQDEEVGRKDLSKTINFMC
NKDVCRESLVTLQLILQAAPQNLTASQHKELQVTIVKLLLDIHQSSSGTVPLPYLDVSCRLQLYRTLHFLVMESHSDSCPPTTYSAFIFMHGLR
DSSHQVSSFCAEALSSIEKIIHPICNSLYFPNEVSDRDDQHRSRIIQAPSSETPHEEESLNEVSHVSESILSDTNNLTRSSEGDTTKFSINTSR
LELDNTKTHPSPLLQRFRNSLGSAGADSPKSSDGSFSSGSAVTVVENPAFINKDKLATSDTIIHSSEESKSNSLQGGKSSIELNKRSAEAEEIL
IDSEENSREAEVSSTEPPQKRPKTKESDRTEAIEAPAVIRLDQAETGGSDDEVQEMLGDFVDLVNEE
>Cimex lectularius [A0A8I6S0I9]
MSDPAESLRLIVQNDDRLELLNYLNCCKESKIFSQEDDVWPTLISSMNKFINDRSKRIYGLIIILEKLLDQCPKSFISPNLIFWIDQCIKGNFQV
DDESIMTLSISLLSQLLTMSVGFSDVKKEITLTLLPKILDSFFKHGNLKQGNKFEKLKCLEVILKLYAKSCAKWKENIHQYIFEHLESGDAEVH
HAGMCYLYLALAGPPSTQNVKYTEAWKQQTYYIISALHSRLNILFENINEIQSPYNDYLVEVDLKDLADINLEKSLFTKMQRVTSQFKNLAEFL
YILCRAKIPCQTPIRVEAILSIICRGAAVNGQTLKANVSADSINLLTQMPVIHQACIRLAEALILSLNFNVFRYTVIPKVLLQTLSWTQSSDWP
IGHQKPYSKLRISAYRCLSLWMSTTHSIYSRSDVEQYIEDIIHNIITDISYKRETVTLNMEMGGKSKRKSKKFKASLTTKEKSSPFIENHLANS
DICHAALEIIQPFMEGAKYDINEDHFSKLLEVIVGLLSSTSCSAKGDKIYPMPYSDDNCRLELYRSLLVMTLYCKPQFPSPVLYASKLFQEGYH
DTCPEISKFCAFAILSVNNVLYPNVPLEGFVKNADKLHNYLQRFKIEKKRETRYDSEELERDNNLDISNKDISIINESASLTCEDDSVLNDLPN
QSLSRSPVFKETDKVREASPGESTSKQAEENNSTPCKESTTADIETTDLSDLHTGKRTPDRSNSRNQNAKRIKFQIVEERIDEESESGNDAVTS
ENEIISTEVKTSENEIVSTEVPTSENESVSDPLESEMLESFVDVV
>Triatoma dimidiata [A0A0V0G6Y3]
MNSTVDLLKLALGESDKYALKNFLQVCTDSTLFQRKDCNIENVVSLVNSLLNVSADYEKGVLILKVLLPQCKSDLVERNLLQWLQQCLKGLQSH
SYLVTPYALLRELLIYSKDYPDIRKQTALNIISKILEHYFKNTRQLATELLLPFLQCLEQILLHYGKACGHWKNKVEQFLLQHLECRSAVVNAC
ARCFLKLSGIGPITPDMEVIKEQWYGKQKFLISALHTLLDRLYNHINEIQGTYEKYVVRSDSTCLPEISETFSLKNAQRLTVQFTSLAEFLRVM
LLGMFPVTKRIYPDAILSVICRGLSVTCTTLEANVSNDSIILATQIPAIHCACLNILDSLIQSCRRSLLPQAELICKLPSQTLKWTKVNFCEFG
QCKPYSFLRVRAYNCLSTWLTVSRVGSYVQHYINDIVPSVLSDIHSQKPAIILNVSAKRLKGKVGKRERKKQRELQNLENKLTTTVNPLANKEV
CSAALNFLSSLLGSSFFLISPNIHKQLQEQIVSELLMVQSALNLSLDLPIPYTDESCRLALYTTLLALCNDHHPKFPAPCNYAVQIFTNGLNDN
SLLVSRFCGQAVSNIDKMLHPIGPTLSCALELSNEVCTSRNVLPTTTIGLNTNIDNLNGSITETDNNRTYQDHVPMNDSLVLESIPKLFENRML

SESVSNDKSKNENVNNIVKPTEEQHIQQTEINESCVKMQETQIKENEITSQIGENFYVQEPITEENTINSPDEIGKPKRKLNNEIISETPREKK
RKEDDHEHFETEKDDLEQEMLSSFVDIIDGILI
>Timema douglasi [A0A7R8VSP2]
MYMFVCSFRLQGLLILEAFLPQCSDQVFVEHAVSWMQQCLKGVETRNDARTTALSYKLLKHVLEMSSNFSELTKQVASSVIPKFLERFSKDMEG
MLAGLRCLEVVLTNYGGACGPGRVSLERFLMKLVDVPTAPRLLRAVGRCVALMPLVGGGGTQRTNHRQQWIKAHLTLCHTLHHLLNQLYQPAED
MRTLLPDAETLSLRKVRDKDPVTRVQRLTTQLGNVAKFLQAMLNGVFPVPKNVSAQAVLDVVCRGLSVQCASLLSRNSSSEAVILACHLPDIHL
QLLDVLKSLILCAGRNLLPHASIICKLVLQELKWTSTDSPQYGVERPYRVLRTKAYETLVTWLRAAKVGSSVEKVSDELVIALIQDIHTNKADV
TLTVLGAKNKNLSKRQKRKLAQDDRSQTTSSQRVKLLNHCANSSLCSAALSLLQWILRTAGCLVKPSLHKVLQETTLGLILDIQRSSGPAQFPA
PYTVANCRRELYELLLVLVLEPHPKWPPPTHLAMRAFSLGQIDSHQEVSAVCISALSSIEKLIHPPFASLQLPAMVQEEVDSLSPSTETRITEV
LTSVSLNNSLGLPSETVLKKRVIEPKLPTGPALVKKTNKPDQLDIKASSSKPQPTKAQPDVIEEIYLTSSDDSNMEIDVNGLEKEHTDLGLNSN
FSEQISSDDDLQEISTQNTTSSQLENEIFDKVKDNQSENKGLHLETNVINLESSVAPEARRSNLEEEKYETLVKNHPIFKWLRIPGTEPLEQQP
QNNSSQTDSEKLREYLKRPNSPSSPATQPLDSYLQNISSTTDIGKIRENYLRSESPFTPVTHSPEHQPEKMSSPADTENLRENYWRTSSPSPEK
QSLDQQSQKSLIPTEFDKLYEYNWKPKSPSSLETQTLGQQNTKVHKEQTEETEKKSVTRQGTEYQKKSPPKTNSYSTDKQVPNSEKESETNSEI
DVEKSPLAESSSMAKEDNLINSPEKIDKTTVNSLPFLTVKNSILVSVGDMDTLDVNYPLVTVEDSITETVDKTDKPKVDKSPLLTKRKIHQTML
SCEKFEKTKIQKTNKNLEGKGENDASLDKEKAKEKIDCVEEKENDNQTKENGKSDDSFIAVNNI
>Timema shepardi [A0A7R9G1Z7]
MSQIRFPWLLNGLVALSGSALSSFAVDHDPLATGQDLARRNNCPESPVLDMVRCLQELPVETLVQADSGLQELRLAAQGFVAGLTSLLGASPAV
DGSDDQRSLPGFIEKSPLEALKLGQFPDIPLLTGVTAAETASALSGPYLKEVLEKSRTVPDFWNSVMHDFTKQSGVPLFANATNQWMNTWTSGY
QNLLQHGHNNQSNGTNIVSQLVEATDDALFNVQAFETANLWSQKSSSFLYSFDHQSARHGGSHFLAGLPIVASANSTSQEKTRPSHGDDLPFLF
DIFSLEGHSEGPHLKLTDPEDVKVQDIFTELVAQFITSRIPNLPNKPAWPTFSSDTSSYLSISSIPKLSDNFRYCEMALWNGLVSHLQSSTCSA
IKGIEQLQAQLFGTVNSVANTIIDPIKNLSSVVNPLQNISTLVLPGSVTNQSLVPTNPVTTHNSSVGSIQRNLTSVITSPVGVLLPKGNTSKPV
LPIVPPRLPWGSPRKPSGGFLGGYRQDFQMILFGAIESQFYVVLAVPHGVAREPLLQGLLILEAFLPQCSDQVFVEHAVSWMQQCLKGVETRND
ARTTALSYKLLKHVLEMSSNFSELTKQVASSVIPKFLERFSKDMEGMLAGLRCLEVALTNYGGACGPGRVSLERFLMKLVDVPTAPRLLRAVGR
CVALMPLVGGGGTQRTNHRQQWIKAHLTLCHTLHHLLNQLYQPAEDMRTLLPDAETLSLRKVRDKDPVTRVQRLTTQLGNVAKFLQAMLNGVFP
VPKNVSAQAVLDVVCRGLSVQCSSLLSRNSSSEAVILACHLPDIHLQLLDVLKSLILCAGRNLLPHASIICKLVLQELKWTSTDNPLYGVERPY
RALRTKAYETLVTWLRAAKVGSSVEKVSDELVIALIQDIHTNKADVTLTAKAIVEHYSRSPSTRKRLHTVMEEMVLPVLELIQFVDTRWSSEYN
MLSRLHAVRKAVGAELANSENNIEILTEVEWKQAAGIVEVLGPLADATKEINKALKSRFSFYDSDPIFCPSMLCDPRFRGVLIDDMVAVNTLAI
EVKKLSDKSSLEPNVKDEHPSCSSSSSGLWSSFDSIPNTTQPAIDNNSEVKDYLNEPRVLGAKNKNLSKRQKRKLAQDDRSQTTSSQRVKLLDH
CANSSLCSAALSLLQWILRAAGCLVKPSLHKVLQETTLGLILDIQRSSGPAQFPAPYTVANCRRELYELLLVLVLEPHPKWPPPTHLAMRAFSL
GQIDSHQEVSAVCVSALSSIEKLIHPPFASLQLPAMVQEEVESSSPSTETRSTEVLTSVSLNSSLDLPSETVLKKRVIEPKLPTGPALVKNANK
PDQLDIKASSSKPQPTKAQPDVIEEIYLTSSDDSNMEIDVNGLEKEHTDLGLNSNFSEQISSDDDLQEISTQNTTSSELGNEVFDKAKDNQSEN
KGLHLETNVINLESSVANEVRSSNLEEEKYETLVKNHPIFKWLRIPGTEPLEQQPQNNSSQTDSEKLREYLKRPNSPSSPATQPLDSHLQNISS
TTDIGKIRENYLISESPFTPVTHSPEHQTQKMSSPADTEKLRENYWRTASPSPEKQSLEQQSQKMSSPADTEKLRENYWRTSSPSPEKQSLEQQ
SQKSLIPTEFDKLYEYNWKPKSPSSIETQTLGQQNTKVHKEQTEETEKKSVTRQGTEYQKKSPPKKDSYSTDKQVPNSEKDSETNSEIDVEKSP
LAESSSMAEEDNLINSERRSAPDIVKPNLPTPSPCYPFNAASGVTLGDVGAECWRSGGLLLVGLAGAAGVSHPVRSPSEGTTRLEGERTESRQH
GVAVGGKGTVPTTGAREQVPTRTDQLVTPCGSQKLRKGCRVSRSSETKNQILKIISNIGGRGLWDVDDVVPKPLDKRIGGTGKSSEEKLRQSLE
VVLDGRHIQVVV
>Neodiprion lecontei [A0A6J0BN46]
MADILNLISTSNQNNDERYKFIQDLLVYKSDVPFDKDEVNAIQNSLLSLINNHLNHAATRHEGLKLLLKTLPRCSRDVLLKHAMLWMTKALQSL
EAVQNEQLQVTTAAKVVATLAISCKTIPELQKQISMQNVKQIITHANNLPDARRFGAIFYLVAVLLYHYPEPCERLQVTIRKLILPLVDSGQDN
LVNAGARCFALLTAATERSFQPIAGKANLTAWTHSQILICNSLHETMDDLFSGVAEIEHIDIGDKLTLPIIPQTDMMLHYLGLERRFNNLCIYL
GITLRGCGSKNSVSPSNIFKILSRGLFITPAGLGNENAFRKQILHLILPNLHSSLLHVLDAFVLGFGKELVPYAQTILQLLHQVLTWTKKTTEN
TRTLGGTRPFKNIRIRVYKSLSTWLMHAGVLSGVENVCEELLSHILKDIVPEKDRVVLSVQKTHNMSKRALKRHRESQFDNNMSQTNILHDSNP
LVNADLVDEALVALQNIFLNTGCRMKASFYESSESMLVALLHDCYLETHEETFYKRSATCRLNLVKALKMLQLNPHPLVPPPTQYCLEIFQMAQ
NDADTNVSHEAKIALAEIEKIAHPAAPTLRLPIVVDLGDRENEPIELDFAMDSNMGPKTAHTSSPNNEDRAGHSPSPILEDPEDESLDTRVNLD
ETNLDTNNENQENVSEPESTKEKRMSDSSAVEIILISITEQVDDSRSGDDSRENTLDDSTNNKGDPSHDTAEAADADEPQVKRQNIEITESVSG
KTADKPQGEIDSEDDAAMLDSFCDAVQGEK
>Coemansia sp. RSA [A0A9W7ZJU6]
MDAKVERAERTLSLLTTTFLANSDSVKANIDLVLDAVLSQDLFAFVGAPDVSAEISKRYSSAIHKWLARINSLATGRTSDARMAGILLIKHTAQ
QSPQLFSENVAKWNTALLSVLSKAEITPVIATTLQTLLVFIDAVRDIPMLHREIVSAHVPRMNQAILAMVDKNVDLTSPVLEMLEYSAAWFPTL
FRPSIDKAEALCLRLLDGSKIRRSPEQCQQAARCLAAFSLVGGKISPEERWFQCVQKAIGTMRLCVDHIMCVDTNATEQPQQHFALAGLSDDFA
KSLPQATDRITVMAEVIIALLTQPTSMDISVPADRIVDIASRVAMISMRAANSKSKRTEYDLIPLLTPQLQSASIRVMAALAISLGSHMQPFLS
AVARTATAIHTRQIVSPTISVALHNLLRLFIERYGYGFVTHLPYDMVVSVVNDICVHRKSNVPTTTTAKADSTASKKRSGNGKTRKANALVADE
SVQASRVYWNDTGLAALATVLALLQHTPTALTTALRTRIDSQILTLLMLFSIGGIELPFASRQTPTSFKVLLYECLEASLLSPDPWQRAILPHA
ISAFNCGLEDPSSQVQKVCSTALLAIDPIIHSRLPAQLREPDNSAEEKQVAQMMSACDGSTTVETVLAGTNASTSGSLNEPMDVTDDQPMSSIK
RFKPSVSNSSHQEPQLAVAAVDSQPGHPPLKQSQPSMSDPTPSKQAMPTPSINTSTSLKSDVSDKPRLAQATAPPVFSKPSNPVHAAEVADEDD
GDDDIPDIVMEGSDSEDE
>Coemansia brasiliensis [A0A9W8I9Z8]
MSTDVAASILFGDLRESYTKQQMRVDILRKAAERLEAQFLASVCERATKRSSCVSKEERQMHEEIRLALGRTRLQLADEIKLLQWIRRRHNMAS
MDARVERAERTLSLLTTTFLANSEAVKANIDLVLDAVLSQNLFAFVGASDISAEITKRYSSAIHKWLARINSLATGRTSDARMAGVLLIKHTAQ
QSPQLFSENVAKWNTVLLSVLSKAEITPVIEVTLQTLLLFIDAVRDIPMLYREIVSAHVPRMNQAILAMVDKNTDLTVPVLEMLEHSATWFPTL
FRPSIDKAEALCLRLLDGSNSRMLLEQCQQAARCLAAFSLAGGKNTPEERWFQCMQKAAGTMRQCVDHIMCVDANSNDQPQQQFALAGLSDDFT
KSIPQAADRIAAMAEVIVALLTQPTPMEISVPADRIVDAASRVAMISMRAANSKSKRAEYDLIPLLTPQLQRASIRIMAALAISLGSHMQPFLS
AVARTATAIHTQQIVSPTISVALHSLLRLFIERYGYGFVTHLPYDIIVSVVNDICVHKKSHAPSTNSTKADHTASKKRSGNGKSRNPSALNTDE
SVHTTRIHWNDTGLAALSTVLVLLQRTPTALATALRTRIDSQILTLLMLFSIGGIEIPFASRQTLTSFKVLLYRCLEASLLSPDPWQRAIIPHA
ISAFNCGLEDPSPQVQKVCSEALLTIDPIVHSRLPAQLREPDNAADEKQVPQIMSACDGSTTVETVLAGIDASANGNHSESMEIINDQPLASAK
RFKPSVSNSSYQEPQFAAADSQPNHQPLKQDQPSASMPTPLSQQTVSTSLASTSAPLKLNATDKPRLVQSTTPSLFNKPNDPVQAVVVTDEDDD
NIPDIVMEGSDSEDE
>Micrurus lemniscatus lemniscatus [A0A2D4I2H5]
SLLALKAECQLPVLEGCQACMTFYPRACGSLRGKLATYFLSCMDAETPHLQQLACECYALLPSLGAGFAQGLKYRESWEQQAHSLVATLHRLLG
RLYEGAETEPLHYDGPGEEVLLPPPRQEEQTASLLLAKHRFAGLAKCLCRMLRNDFGTPVTVPAQAILDLVCRALDVSVKSMSWFGDGPLRMLL
LPSIHLEALDLLAALILACGPRLVRFGGALCRLFPQVLNMWRAGQDLLSPGLQRPYSAVRARLYQVLDLWVQVAGASSGLLLGHSSQSDALLGH
LIDDISPPSDALKMQPNPTSEGKPSAPKKAKLS
>Polar bear [A0A452UG38]
GLSNARLGSIKTRFEGLCLLSLLVGESPTEMFQQHCVSWLRSIQQVLTRPPRWSXXXXXXXXXXXHYISMNHLPGLLTSLLGLRPECELSALEG
MKACMTYFPRACGSLKGKLASFFLSRVDALSPQLQQLACECYSRLPSLGAGFSQGLKHTESWEQELHSLLASLHPLLGALYEGAETAPVQYEGP
GVEVLLTPSEDGDAHVLLRLRQRFCGLARCLGLMLSSEFGAPVSVPVQEILDVICRTLSISAKNISLLGDGPLRLLLLPSIHLDALDLLSALIL

ACGGRLLRFGALISRLLPQVLNAWNFGRDTLSPGQERRGWVRGPEEDPACGLRSPRGSPDGGLQAGKPSAPKKLKLDMGEAMAPPSHRKGDSNA
NSDVCAAALRGLSRTVLPLIKEETHRRLHDLVLPLVMGVQQGEVLGSSPYTSSRCRRELYRLLLALLLAPSPRCPPPLACALQAFSHGQREDSL
EVSSFCSEALVTCAALTHPRVPPLQCRGPACXXXXXXXXXXXXXXXXXXXXXXXXXXXXXXXXXXXXXXXXXXXXXXXXXXXXXXXXXXXXXXXXXXXXXXXXXXXXXXX
XXXXXXXXXXXXXXXXXXXXXXXXXXXXXXXXXXXXXXXXXXXXXXXXXXXXXXXXXXXXXXXXXXXXXXXXXXXXXXXXXXXXXXXXXXXXXXXXXXXXXXXXXXXXXXXXXXXXXXXXXXXXXXXXXXXXX
XXXXXXXXXXXXXXXXXXXXXXXXXXXXXXXXXLTVININSSDEEEEEEEEEEEEEEEEEEEEEDFEEEEEDEEEYFEEEEEEEEEFEEEFEEE
EGELEEEEDEDEEEEELEELEEVEFGPAGGPVEEGGPPPASPAPALPPAQSPKVQPEPEGEPGLLLEVEEPGAEEEAGAEAAPTLAPEVLPSQG
EGPRDTGSPPAGPPPQELMEEEPLAPPTLLEEGTESGGDKVPPPAETAVAEDVETELETATAALQEKEQDDTAAMLADFIDCPPDDEKPPPAPE
PES
>Lynx pardinus [A0A485MNG5]
MAAAVLSGPSAGSAAGVPGGTGGLSAVGSGPRLRLLLLESVSGLLQPRAGSAVAPVHPPVRSAAHLPGLMCLLRLHGTVGGAQNLSAVGALVGL
SNARLGSVKTRFEGLCLLSLLVGESPTELFQQHCVSWLRSIQQVLQGKLASFFLSRVDALSPQLQQLACECYARLPSLGAGFSQGLKHTESWEQ
ELHSLLASLHGLLGALYEGADTAPVQCEGPGLDVLLAPSEDGDAHTLLRLRHRFSGLARCLGLLLRCLRSPRGSPDGGLQSGKPSAPKKLKLDM
GEATAPPGHRKGDSNANSDVCAAALRGLSRTVLMCGPLIKEETHRRLHDLVLPLVMGVQQGEVLGSSPYTSSRCRRELYRLLLALLLAPSPRCP
PPLACALQAFSLGQREDSLEVSSFCSEALVTCAALTHPRVPPLQSMGPACPAPAPAPPPEAPSPFRAPPFHPPGPMPSVGPMPSVGPMPSVGPM
PPAGPMPPTRPGPPATANHLGLSVPGLVSVPPRLLPGPENHRAGSNDDPVLAPSGTPPPAVPPDETFGGRVPRPAFVHYDKEEASDVEISLESD
SDDSVVIVPEGLPPLPPPPAAPAGPPTASPPVPAKEEPEELPAAPGPLPPPPPPPPVPGPVALPPPQLVPEGPPGGGGPPALEEDLTVININSSD
EEEEEEEEEEEEEEEEEEEEEDFEEEEEEEEEEYFEEEEEEEEEEFEEEFEEEEGELEEEEEDEDEEEEDELEELEEVEFGPAGGPAEEGGPPPPS
PAPALPPAQPPEAPPEPGVEPGLLLEVEEPGPEDEPGAEAAPTLAPEVLPSQGEGQREAGSPPAGPPPRELVEEEPSAPPPLLEEGTENGGDKV
PPPPETPAAEEMEEQYDTAAMLADFIDCPPDDEKPPAPKSDAPPTEPPGRPQTVTPLSTRKLFK
>Owenia fusiformis [A0A8J1UZI8]
SNSSMRELVSHVNTWLNSSQFRVQGLGILYHILQDCSAQVITDYGLTWMALLTQALQSYDPAPTHKAACVATRHLLKHTINHPELARQISTTHL
PSLLNTLLAAKPHWLEGALLCTRACIQFYHGACGPFKSKIETFAVSHLEHSDTAVSKAAILCYSVIPMCGGGGNQGIKHTESWAKHLDKLLGTM
NDILDNLYDGLESGDFTYFVPKDRLNLLPLKTDHVEKVHQLSDQFGIVAKCIAQLLRTDYPSLVNLPLEELLGIVCRTLAITGTFLLSRPSSER
VTLVTVLPNVHQEALKLLEQLIPTIGGHLVPHTYTVNQLLKQSLAWTKTGDQQTRLYSSLRSQIYQTLAMWEKTLRASSNSQKIAESIVENVIS
DARIPKDILKLNETTETDYSEPLNKKQRRKGKGSSGIAQGNPSKLMGISKGSSKLCSDSLAALKWLLTTVGHELKPATQKAVQEFLVPLILEIQ
QNPQALPHPYSDSQCRHLVYDCLLATVVVPCPKWPPPTQCAMQIFKQGLLDKNTLVSSFCQTALTQCTSIIHPRAPTLLSPLSLAVLPTELRIS
QKTTNQDSETISHDFTLGKNPAEKAAENMNVEMPSTSNEDDSNEINPPSDISDVHPVESNDSNTEDQKESDDDSDMVNEDSDMDNDDSDGNNVN
SDKNNDDTDRYNEDKDDTIDEQPENLNENKIRSEDPEVVTETNVSKGEFVKEGASQIHKDEEVDKLTVEDVSSVKKADSDLADEMLMDFVDASP
DSD
>Owenia fusiformis [A0A8J1U294]
MSAPMKSVVDQILNNISENNGNPLELVNLISCSREFSIFQDASNSSMRELVSHVNTWLNSSQFRVQGLGILYHILQDCSAQVITDYGLTWMALL
TQALQSYDPAPTHKAACVAMRHLLKHTINHPELARQISTTHLPSLLNTLLAAKPHWLEGALLCTRACIQFYHGACGPFKSKIETFAVSHLEHSD
AAVSKAAILCYSVVPMCGGGGNQGIKHTESWAKHLDKLLGTMNDILDNLYDGLESGDFTYFVPKDRLNLLPLKTDHVEKVHQLSDQFGIVAKCI
AQLLRTDYPSLVNLPLEELLGIVCRTLAITGTFLLSRPSSERVTLVTVLPNVHQEALKLLEQLIPTIEGHLVPHTYTVNQLLKQSLAWTKTGDQ
QTRLYSSLRSQIYQTLSMWEKTLRASSNSQKIAESIVENVISDARIPKDILKLNETTETDYSEPLNKKQRRKGKGSSGMAQGNPSKLMGISKGS
SKLCSDSLAALKWLLTTVGHELKPATQKAVQEFLIPLILEIQQNPQALPHPYSDSQCRHLVYDCLLATVVVPCPKWPPPTQCAMQIFKQGLLDK
NTLVSSFCQTALTQCTSIIHPRAPTLLSPLSLADLPTELRISQKTTNQDSETISHDLTLGKNPAEKAAENMNVEMPSTSNEDDSNEINPPSDIS
DVHPVESNDSNTEDQKESDDDSDMVNEESDMDNDDSDGNNVKSDKNNDDTDRYNDDKDDTIDDQPENLNENKIRGEDPEVITETDISKGEFVKE
GASQIHKDEEVDKLTVEDVSSVKKADSDLADEMLMDFVDASPDSD
>Paralvinella palmiformis [A0AAD9NFV2]
MEPQLFCMQHRMRSLDRLGVNQCSYDLAWLERSRDHRTTSETISYAHSCINASKSRLKGLLLLHQILDQCDSPIFQEHALSWSQMLLSVLQSYN
SDAVHQMAAEVLSELLHHAVTFPAISRDLATSLVPTIIPTLLDVGTECELSAFRCLKTCLHFFTGACSTLKNKIEAKLREGLLKAKASSEALSI
ITECYTLLALCGGGGDKKDIDSYRRPASRLFTVDCPDAEPTRTQMLVHHYRVLAQTMATMLRPSVSVTLHVPVEDILTIICRATSISPKFLMTR
PTHQRVLLCSVLPTIYKMSLHMLKHLITCVETGLILHCSVINKLLIQILDSVAGCTDGGDGNHDNPFSELKQSVYEVASTWLIVAGTTSAMVIV
AGDFIDHILTDARPRVETIKSISGSKILEAAEQILGKRKKKKGKGSKEYLQSSWQHKSKPFANWELCSKALEALRQLMIHCGTELKPFDHKKIQ
DFIIPPLLSISQCSETVAPPYGHPDCRKRLYSVLEASVLVPSALWPPPIHVALSVFSKAAVCESDLETRSYCHQALVICQNLVHPRIPPLVAPQ
SVADLVERYRCLSSAAETDQFQQPGSVNPDLQYTVSHTNNVQDLDGHILDTSVMDHDGVQTDDIQKEATQNEEGVAVEMSSKEEGERCIGEEEQ
QMSEKEGDPFEEVIELSSSDRDDGDNDGDDDDDDEEGDSNSGDKGNITEDDKNIKQSNQEDQPQQIIISDPGSDPECNQTSELTASQQSTYNAP
TEFQNKSMDNSHSTSGKRKHEILEDNDIAEIGDGEPSTKKQIEDVDLMLQSFVDADPDVD
>Tursiops truncates [A0A6J3QLU3]
MAAAVLSGPSAGSAAGVPGGTGGLSAVNSGPRLRLLLLESVSGLLQPRAGSTVSPVHPPT
RSVPHLPGLMCLLRLHGTVGGAQNLSAVGALVGLSNARLGSIKTRFEGLCLLSLLVGESP
TEMFQQHCVSWLRSIQQVLQGKLASFFLSRVDALSPQLQQLASPMQYEGPGVETLLSPSE
DGDAHVLLRLWQRFSGLARCLGLMLSSEFGAPVSVPVQEILDLICRTLSVSAKNISLLGD
GPLRLLLLPSLHLEALDLLSALILACRGRLLRFGALISRLLPQVLNAWSIGRDSLSPGQE
RPYSTMRTKVYTVLELWVQVCGASAGVLQGGASGEALLTHLLSDISPPADALKLRSPRGS
PDGGLQTGKPSAPKKLKLDMGEALAPPSHRKGDSNANSDVCAAALRGLSRTVLMCGPLIK
EETHRRLHDLVLPLVMGVQQGEVLGSSPYTSSRCRRELYRLLLALLLAPSPRCPPPLACA
LQAFSLGQREDSLEVSAFCSEALVTCAALTHPRVPPLQTMGPACPTPAPVPPPEAPSPFR
APAFHPPGPMPSVGPMPSVGPMPSAGPMPSPGPVPPAGPMPSVGPMPPARPGPPATANHL
GLSVPGLVSVSPRLLPGPENHRAGSNEDAVLAPSGTPPPTIPPDETFGGRVPRPAFVHYD
KEEASDVEISLESDSDDSVVIVPEGLPPLPPPPPSGTTPPPAAPVGPPTASPPVPAKEEP
EELPAAPGPLPPPPPPPPVPGPVALPPPQLVPEGTPSGGGPPALEEDLTVININSSEEEED
DDEEDEDEEDFEEEEEEEEEEEEYFEEEEEEEEEEFEEEFEEEEGELEEEEEDEDEEEEVEE
VEFGPAGGEVEEEGPAPPSLPPALPPAASPKVQPQPEPEPGLLLEVEEPGAEEGPGAETA
PTLAPEVLPSQGEVEREGGSPPAVPSPQELVEEEPSVPPTLLEEGAEGGGDKVPTPPEAS
AAEEMETEAEAAALQEKEQDDTAAMLADFIDCPPDDEKPPPATEPDS
> Tursiops truncates [A0A6J3QME7]
MAAAVLSGPSAGSAAGVPGGTGGLSAVNSGPRLRLLLLESVSGLLQPRAGSTVSPVHPPT
RSVPHLPGLMCLLRLHGTVGGAQNLSAVGALVGLSNARLGSIKTRFEGLCLLSLLVGESP
TEMFQQHCVSWLRSIQQVLQGKLASFFLSRVDALSPQLQQLACECYSRLPSLGAGFSQGL
KHTDSWEQELHSLLTSLHSLLGALYAGADTAPMQYEGPGVETLLSPSEDGDAHVLLRLWQ
RFSGLARCLGLMLSSEFGAPVSVPVQEILDLICRTLSVSAKNISLLGDGPLRLLLLPSLH
LEALDLLSALILACRGRLLRFGALISRLLPQVLNAWSIGRDSLSPGQERPYSTMRTKVYT
VLELWVQVCGASAGVLQGGASGEALLTHLLSDISPPADALKLRSPRGSPDGGLQTGKPSA
PKKLKLDMGEALAPPSHRKGDSNANSDVCAAALRGLSRTVLMCGPLIKEETHRRLHDLVL
PLVMGVQQGEVLGSSPYTSSRCRRELYRLLLALLLAPSPRCPPPLACALQAFSLGQREDS

LEVSAFCSEALVTCAALTHPRVPPLQTMGPACPTPAPVPPPEAPSPFRAPAFHPPGPMPS
VGPMPSVGPMPSAGPMPSPGPVPPAGPMPSVGPMPPARPGPPATANHLGLSVPGLVSVSP
RLLPGPENHRAGSNEDAVLAPSGTPPPTIPPDETFGGRVPRPAFVHYDKEEASDVEISLE
SDSDDSVVIVPEGLPPLPPPPPSGTTPPPAAPVGPPTASPPVPAKEEPEELPAAPGPLPP
PPPPPVPGPVALPPPQLVPEGTPSGGGPPALEEDLTVININSSEEEEDDDEEDEDEEDFE
EEEEEEEEEYFEEEEEEEEEFEEEFEEEEGELEEEEEDEDEEEEVEEVEFGPAGGEVEE
EGPAPPSLPPALPPAASPKVQPQPEPEPGLLLEVEEPGAEEGPGAETAPTLAPEVLPSQG
EVEREGGSPPAVPSPQELVEEEPSVPPTLLEEGAEGGGDKVPTPPEASAAEEMETEAEAA
ALQEKEQDDTAAMLADFIDCPPDDEKPPPATEPDS
>Mustela putorius furo [G9KFZ9]
RDISMNHLPGLLTSLLGLRPECELSALEGMKACMTYFPRACGSLKGKLASFFLSRVDALS
PQLQQLACECYSKLPSLGAGFSQGLKHTESWEQELRSLLASLHSLLGALYEGAETAPMQY
EGPGVEMLLTPSEDGDAHVLLRLRQRFCGLARCLGLMLSSEFGAPVSVPVQEILDVICRA
LSISAKSISLLGDGPLRLLLLPSIHLDALDLLSALILACGGRLLRFGALISRLLPQVLNA
WNLGRDTLSPGQERPYSTVRAKVYAVLELWVQVCGASAGVLQGGASGEALLSHLLSDISP
PADTLKLRSPRGSPDAGLQSGKPSAPKKLKLDMGDAAAPPSHRKGDANANSDVCAAALRG
LSRTVLMCGPLIKEETHRRLHDLVLPLVMGVQQGEVLGSSPYTSSRCRRELYRLLLALLL
APAPRCPPPLSCAVQAFSLGQREDSLEVASFCSEALVTCAALTHPRVPPLQSMGPACPTP
APVPPPEAPSPFRAPPFHPPGPMPSVGPMPSVGPMPSASAMPPTGPLPPAGPLPPTRPGP
PATANHLGLSVPGLVSVPPRLHPGPENHRAGSNEDPVLAPSGTPPPPIPPDETFGGRVPR
PAFVHYDKEEASDVEISLESDSDDSVVIVPEGLPPLPPLPPPPPPAPPPPPGAPAGPPTA
SPPGPAKEEPEELPTAPGPLPPPPPP
> Canada lynx [A0A667HQW4]
MAAAVLSGPSAGSAAGVPGGTGGLSAVGSGPRLRLLLLESVSGLLQPRAGSAVAPVHPPV
RSAAHLPGLMCLLRLHGTVGGAQNLSAVGALVGLSNARLGSVKTRFEGLCLLSLLVGESP
TELFQQHCVSWLRSIQQVLQGKLASFFLSRVDALSPQLQQLACECYARLPSLGAGFSQGL
KHTESWEQELHSLLASLHGLLGALYEGADTAPVQCEGPGLDVLLAPSEDGDAHTLLRLRH
RFSGLARCLGLLLSSEFGAPVSVPVQEILDIICRTLSISAKNISLLGDGPLRLLLLPSIH
LDALDLLSALILACGSRLLRFGALISRLLPQVLNAWNLGRDALPPGQERPYSAVRTKVYA
VLDLWVQVCGASAGVLQGGASGEALLSHLLSDISPPADALKLRSPRGSPDGGLQSGKPSA
PKKLKLDMGEATAPPGHRKGDSNANSDVCAAALRGLSRTVLMCGPLIKEETHRRLHDLVL
PLVMGVQQGEVLGSSPYTSSRCRRELYRLLLALLLAPSPRCPPPLACALQAFSLGQREDS
LEVSSFCSEALVTCAALTHPRVPPLQSMGPACPAPAPAPPPEAPSPFRAPPFHPPGPMPS
VGPMPSVGPMPSVGPMPPAGPMPPTRPGPPATANHLGLSVPGLVSVPPRLLPGPENHRAG
SNDDPVLAPSGTPPPAVPPDETFGGRVPRPAFVHYDKEEASDVEISLESDSDDSVVIVPE
GLPPLPPPPPTGTTPPPAAPAGPPTASPPMPAKEEPEELPAAPGPLPPPPPPPPVPGPVAL
PPPQLVPEGPPGGGGPPALEEDLTVININSSDEEEEEEEEEEEEEEEEEEEEEEEDFEEEEE
EEEEYFEEEEEEEEEFEEEFEEEEGELEEEEEDEDEEEEDELEELEEVEFGPAGGPAEEG
GPPPPSPAPALPPAQPPEAPPEPGVEPGLLLEVEEPGPEDEPGAEAAPTLAPEVLPSQGE
GQREAGSPPAGPPPRELVEEEPSAPPPLLEEGTENGGDKVPPPPETPAAEEMEAAAEAET
AALQEKEQDDTAAMLADFIDCPPDDEKPPAAPEPES
>Spermophilus dauricus [A0A8C9PAF3]
MELAVAVLRDLLRYAAQLPTLFRDISINHLPGLLTSLLGLRPECEQSALEGMKACMTYFP
RACGSLKGKLASFFLSRVDALSPQLQQLACECYSRLPSLGAGFSQGLKHTESWEQELHSL
LASLHSLLGALYEGAETAPVQNEGPGVETLLSPSEDGDAHVLLRLRQRFSGLARCLGLML
SSEFGAPVSVPVQEVLDLICRTLSVSGKNISLLGDGPLRLLLLPSIHLEALDLLSALILA
CGGRLLRFGALISRLLPQVLNAWSIGRDTLSPGQERPYSTIRTKVYAILELWVQVCGASA
GVLQGGASGEALLTHLLSDISPPSDALKLRSPRGSPDGGLQTGKPSAPKKIKLDVGEAMA
PPSHRKGENNANSDVCAAALRGLSRTILMCGPLIKEETHRRLHDLILPLVMGVQQGEVLG
SSPYTSSCCRRELYRLLLALLLAPSPRCPPPLACALQAFSLGQREDSLEVSSFCSEALVT
CAALTHPRVPPLQAMGPTCPTPAPVPPPEAPSPFRAPPFQPPGPMPSVGSMPSVGPMPSA
GPMPSAGPVPSARPGPPATANHLGLSVPGLVSVPPRLLPGPENHRAGSNEDPVLAPSGTP
PPTIPPDETFGGRVPRPAFVHYDKEEASDVEISLESDSDDSVVIVPEGLPPLPPPPPSGT
TPPPVAPTGPPTASPPVPAKEEPEELPAAPGPLPPPPPPPPVPGPVTLPPPQLVPEGTPGG
GGPPALEEDLTVININSSDEEEEEEEEEEEEEEEEEEEEEEEEEFEEEEEEEEEYFEEEEEE
EEEFEEEFEEEEGELEEEEEEEEEEEELEEEEREGGSPEAGPPPQELVEEEPSAPPTLLE
EGTEGGGDNEPPPPETAAEEEMETETEAAVLQEKEQDDTAAMLADFIDCPPDDEKPPPTT
EPDS
>Pocillopora damicornis [A0A3M6V4K3]
MELAVAVLRDLLRYAAQLPTLFRDISINHLPGLLTSLLGLRPECEQSALEGMKACMTYFP
RACGSLKGKLASFFLSRVDALSPQLQQLACECYSRLPSLGAGFSQGLKHTESWEQELHSL
LASLHSLLGALYEGAETAPVQNEGPGVETLLSPSEDGDAHVLLRLRQRFSGLARCLGLML
SSEFGAPVSVPVQEVLDLICRTLSVSGKNISLLGDGPLRLLLLPSIHLEALDLLSALILA
CGGRLLRFGALISRLLPQVLNAWSIGRDTLSPGQERPYSTIRTKVYAILELWVQVCGASA
GVLQGGASGEALLTHLLSDISPPSDALKLRSPRGSPDGGLQTGKPSAPKKIKLDVGEAMA
PPSHRKGENNANSDVCAAALRGLSRTILMCGPLIKEETHRRLHDLILPLVMGVQQGEVLG
SSPYTSSCCRRELYRLLLALLLAPSPRCPPPLACALQAFSLGQREDSLEVSSFCSEALVT
CAALTHPRVPPLQAMGPTCPTPAPVPPPEAPSPFRAPPFQPPGPMPSVGSMPSVGPMPSA
GPMPSAGPVPSARPGPPATANHLGLSVPGLVSVPPRLLPGPENHRAGSNEDPVLAPSGTP
PPTIPPDETFGGRVPRPAFVHYDKEEASDVEISLESDSDDSVVIVPEGLPPLPPPPPSGT
TPPPVAPTGPPTASPPVPAKEEPEELPAAPGPLPPPPPPPPVPGPVTLPPPQLVPEGTPGG
GGPPALEEDLTVININSSDEEEEEEEEEEEEEEEEEEEEEEEEEFEEEEEEEEEYFEEEEEE
EEEFEEEFEEEEGELEEEEEEEEEEEELEEEEREGGSPEAGPPPQELVEEEPSAPPTLLE
EGTEGGGDNEPPPPETAAEEEMETETEAAVLQEKEQDDTAAMLADFIDCPPDDEKPPPTT
EPDS
>Phallusia mammillata [A0A6F9DPF1]
MQEVDKELFSLLHKKSWSKNLDISNINEWIRLSAERWKGFYTMELVLSRCTEDVILKNGI

SWIEIAATTVVKNHIEESVRTIAASNFAKLLEISTQIPSVSKELSSNHVINILQLLLSSD
NCNPVPCLACMEAIMKHFPAPCNPHKGKVSKYIFPHTLSLKTNAQDAAIRCWSMVALLGG
GGQGRKNHALQWEQQFDETLKAWKFISTNLWDPKLDEGEVDPSIGSLSFHFDPLPSSEPD
LSITLVQRFLMVCKCLCGLLNLNFPHLLNVSVDKIFNLLIANFDVTPKSLKHIIESHLSV
DCLQTVHIHCLYVLRACIESGHTALCRRATQVNRMFLNLLTMWRDTNPTFGMSKNCIKIR
LNIYGVMLYWVESCGSKSRFLMGQKHFTQPLLEYMVENFSLIPDYERGNTDIQAKKPIVS
GRKKSKRLINSDVSFSRIVDSSANSQVCIASLKVLRICIYSNGSMLPSFVHKSLQKHVIL
LLMDIMRAKNIIPYTHCAGCRKELYHLLLALTVVPSPEYPAPLQLALTIIRHGSARDSDM
DVRSFCMEASATLSNIVRPRIPALNRICPSMDVIRSVQTSAQYTSETHLVHVEESEQPIS
QTSNEVEMNTETDVRSSETSTSPLLPVNYEEQESVAKTISETESNDDPVPEANQPVMKLP
VTSDASFLSPFRNTHEVAIESDCDHDVNKLGENSSNHENQLNEIYLDNNSTRGQKRKLEN
TQSPTADMNTEENEQKVELTDDITAMLSDFVDASPDDDTSGGDQ
>Pinctada imbricate [A0AA89BYM2]
MWLRSILQILQSHDPPNVLKMTCHVCRDLLKTVSVFTSLGREITAIIPQILTAVIAASQQ
WRDDSFIVINSCIRYYSGTCGTFRETIYCWALLARCGTGGNQGIKYTEGWSTQCSKVIST
LEHVAFLLYRDINDGNLTPSLPSISVISTQCSKVISTLEHVAFLLYRDINDDFISSRHLQ
SSIPLGQIPSTVSDKLPILTTRFETLTRCLVKLIRFKTLLLPYSKQIISMYVTELIWSHS
SSDYGQLKLYSSLRGSVYTGLTRWFQHCKCLIETVNEEDNLLSEILHDCRPHVDNVKVRY
YFAVGLIYTMSRPHIDECQGEILHDCRPHIDERQGEILHDCRPHIDECQGEILYDCRPHI
DECQGEILHDCRQHIDNVKINSKISNVKSGSDPPPAKKKKKGGYQELSMGISSQRKVDPV
ADHKLTTLSLEALYQWIVSAGTQMKEKTYVTIMDFIINTSIKVSQNKQDPGIPYGESHCR
LQLLHCLKALTMVPHPKVVCPVQCVISILKTASCDTCTQVSTFCSETLRYLDVIIHPRAP
YFTKPKTINEEQSDKVHAQTSGPYHIFSQQSVPMSSLFVGQNWNQPQSSKEDQEMQVDSP
IPLSSKANTISNSDSDLDDSSDSDSDNTEVSNTVQYSGENSTENAASSSVTAVSDTSKVV
ASDNMGDIGVSNTGKAIVQDSIDQDIIVRRGSIVLGVGEQLVDSEEISPKESGDAGSSSC
ITTKTTGEKTPVQHEDSCTTQSDRITSTEEGLGGDADCSDVLEERTDNGAPYSGSVDITE
GESVKSSNVDKSGAGQGCMEKTEVDSMLSAFMDVDAEGTGD
