# Supplementary figures and images for: Proline-, Glutamic Acid-, Leucine-Rich Protein 1 (PELP1): Diversity, Structural Conservation, and Evolutionary Origins Across the Species
Source: Int J Mol Sci. 2025 Dec 12;26(24):11989. doi: 10.3390/ijms262411989 (PMC12733202; doi:10.3390/ijms262411989)

Tree scale: 0.1

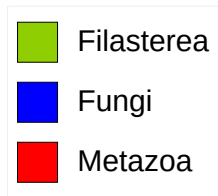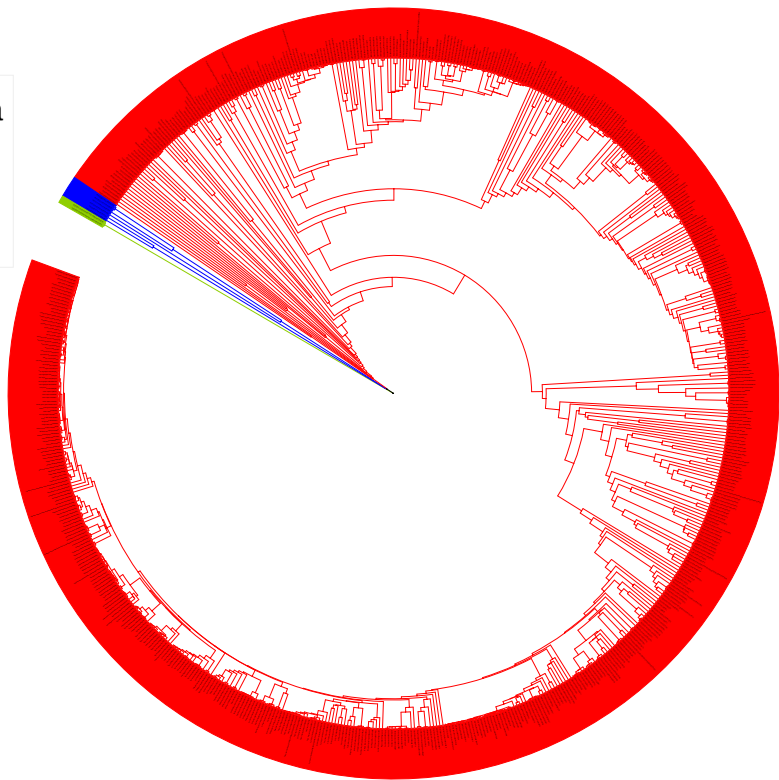

Supplement: Supplementary file 1 [file ijms-26-11989-s001.zip › Figure S1.pdf]
